# Stings on wings: Proteotranscriptomic and biochemical profiling of the lesser banded hornet (*Vespa affinis*) venom

**DOI:** 10.3389/fmolb.2022.1066793

**Published:** 2022-12-19

**Authors:** Kartik Sunagar, Suyog Khochare, Anurag Jaglan, Samyuktha Senthil, Vivek Suranse

**Affiliations:** Evolutionary Venomics Lab, Centre for Ecological Sciences, Indian Institute of Science, Bangalore, India

**Keywords:** *V. affinis*, venom proteome, venom gland transcriptome, wasp venom, arthropod venom

## Abstract

Distinct animal lineages have convergently recruited venoms as weaponry for prey capture, anti-predator defence, conspecific competition, or a combination thereof. Most studies, however, have been primarily confined to a narrow taxonomic breadth. The venoms of cone snails, snakes, spiders and scorpions remain particularly well-investigated. Much less explored are the venoms of wasps (Order: Hymenoptera) that are infamous for causing excruciating and throbbing pain, justifying their apex position on Schmidt’s pain index, including some that are rated four on four. For example, the lesser banded wasp (*V. affinis*) is clinically important yet has only been the subject of a few studies, despite being commonly found across tropical and subtropical Asia. Stings from these wasps, especially from multiple individuals of a nest, often lead to clinically severe manifestations, including mastocytosis, myasthenia gravis, optic neuropathy, and life-threatening pathologies such as myocardial infarction and organ failure. However, their venom composition and activity remain unexplored in the Indian subcontinent. Here, we report the proteomic composition, transcriptomic profile, and biochemical and pharmacological activities of *V. affinis* venom from southern India. Our findings suggest that wasp venoms are rich in diverse toxins that facilitate antipredator defence. Biochemical and pharmacological assessments reveal that these toxins can exhibit significantly higher activities than their homologues in medically important snakes. Their ability to exert potent effects on diverse molecular targets makes them a treasure trove for discovering life-saving therapeutics. Fascinatingly, wasp venoms, being evolutionarily ancient, exhibit a greater degree of compositional and sequence conservation across very distant populations/species, which contrasts with the patterns of venom evolution observed in evolutionarily younger lineages, such as advanced snakes and cone snails.

## 1 Introduction

The innovation of venom systems in animals has served as an effective tool for the incapacitation of prey and predators (Sunagar et al., 2016; Casewell et al., 2020). Wasps (order Hymenoptera), with origins dating over 250 million years ago [MYA; (Peters et al., 2017)], primarily owe their evolutionary success to an ability to produce and effectively deliver venoms. Considering their widespread occurence and deep evolutionary origins, hymenopterans are an ideal system to investigate the role of natural selection in shaping animal venoms. Previous studies characterising wasp venoms have recorded an abundance of haemolysins, vasodilators, vasospastic amines and various enzymes (Piek, 2000; Kularatne et al., 2014; Zhao et al., 2015). Being rich in such pharmacologically active components, wasp stings can be clinically severe to humans. The clinical manifestations of wasp envenoming range from mild allergic reactions to severe effects, such as mastocytosis, reversible optic neuropathy, intravascular hemolysis, myasthenia gravis, renal failure, fatal acute pulmonary oedema and multiple organ failure (Maltzman et al., 2000; Das and Mukherjee, 2008; Kularatne et al., 2011; Kularatne et al., 2014; Rungsa et al., 2016). Furthermore, numerous cases of wasp sting-associated mortality have been recorded in many countries (Mosbech, 1983; McGaln et al., 2000; Vikrant et al., 2019; Feás, 2021), highlighting the medical importance of these hymenopterans.

The lesser banded hornet (*V. affinis*; family Vespidae), with a widespread distribution across tropical and subtropical Asia, is one of the most commonly encountered hornets in the Indian subcontinent (Bequaert, 1936). These wasps are eusocial and often build nests in proximity to human settlements. They have been known to cause accidental stings with many recorded fatalities and other grave medical conditions from Europe in the west, all the way up to Southeast Asia and China in the east (Scragg and Szent-Ivany, 1965; Barss, 1989; Lee et al., 2005; Kularatne et al., 2014; Feás, 2021; Liu et al., 2022). There have also been several reports of deaths resulting from *Vespa* stings in India (Pramanik and Banerjee, 2007; Nandi and Sarkar, 2012; Dhanapriya et al., 2016). Despite the evident medical relevance, our understanding of their venom composition and activity is limited. Investigation of the vespid venom arsenal could augment the current treatment and provide insights into the venom evolution of this fascinating hymenopteran lineage.

We implemented a multifaceted approach to address this knowledge gap and performed proteomic, transcriptomic, biochemical and pharmacological characterisation of *V. affinis* venom. Sequencing of the venom gland transcriptome of this species, for the first time, revealed the complexity of *Vespa* venoms. While venom proteomics and comparative transcriptomics revealed the highly defensive nature of *Vespa* venoms, biochemical and pharmacological assays provided insights into their biodiscovery potential. We further leveraged the bioinformatic and phylogenetic analyses to assess the role of natural selection in shaping the venom arsenal of these clinically relevant wasps.

## 2 Materials and methods

### 2.1 Venom extraction

Adult *V. affinis* individuals (n = 94) from the same nest were collected from the Indian Institute of Science (IISc) campus in Bangalore, India. Prior to venom extraction, wasps were immobilised by exposing them to 4°C on ice for 5 minutes. Venom was then extracted from these individuals using mild electrical stimulation (9 V–12 V DC) for 30 s. The venom was collected in an RNase-free microcentrifuge, flash-frozen and stored at −80°C until further use. For the comparative evaluation, the venoms of the “big four” Indian snakes, namely Russell’s viper (*Daboia russelii*), common cobra (*Naja naja*), saw-scaled viper (*Echis carinatus*) and common krait (*Bungarus caeruleus*) were sourced from the Irula Snake Catchers’ Industrial Cooperative Society.

### 2.2 Venom proteomics

#### 2.2.1 Sodium dodecyl sulfate-polyacrylamide gel electrophoresis (SDS-PAGE)

Crude venom (12 μg) mixed with molecular grade water (10 μl) and loading dye (5 μl) was boiled at 100°C for 8 min, loaded onto a 15% polyacrylamide gel along with a protein molecular weight ladder (Bio-Rad Laboratories, United States of America) and ran at a constant voltage of 80 V (Smith, 1984). Following electrophoresis, the gel was stained with Coomassie Brilliant Blue R-250 (Sisco Research Laboratories Pvt. Ltd., India), and the protein bands were visualised using an iBright CL1000 (Thermo Fisher Scientific, United States).

#### 2.2.2 Tandem mass spectrometry

The proteomic composition of *V. affinis* venom was determined using tandem mass spectrometry, wherein, following SDS-PAGE separation, the excised protein bands were treated with dithiothreitol (DTT 10 mM; Sigma-Aldrich, United States), alkylated with iodoacetamide (IAA 40 mM; Sigma-Aldrich, United States), and trypsin digested (25 ng/μl; Promega Corporation, United States) overnight at 37°C. Subsequently, the analytes were desalted with spin columns and subjected to liquid chromatography on a Thermo EASY nLC system (Ultimate 3,000 series Thermo Fisher Scientific, MA, United States) with a PepMap C18 nano-LC column (50 cm × 75 μm, 2 µm particle size and 100 Å pore size). A sample volume of 6 µl was injected into the column and run with buffer A (0.1% formic acid in MS grade water) and buffer B (0.1% formic acid in 80% Acetonitrile) solutions at a constant flow rate of 250 nL/min for 90 min. A gradient of buffer B was used for the elution of venom toxins: 8–35% over the first 70 min, followed by 35–95% over the next 5 minutes and 95% over the last 15 min. Tandem mass spectrometry was carried out in the Orbitrap Fusion Mass Spectrometer (Thermo Fisher Scientific, MA, United States). MS scan was performed using a scan range (m/z) of 300–2000, a resolution of 120,000, and a maximum injection time of 100 ms. Fragment scans (MS/MS) were performed using an ion trap detector with high collision dissociation (HCD) fragmentation (30%), a scan range (m/z) of 110–2000, and a maximum injection time of 50 ms. PEAKS Studio X (Bioinformatics Solutions Inc.) was used to identify protein families by searching the raw MS/MS spectra against the Uniprot protein database (www.uniprot.org/; 8^th^ September 2022), as well as *V. affinis* tissue transcriptomes generated in this study. A monoisotopic mass search was performed with “semispecific” trypsin digestion and a maximum of three missed cleavages, and the parent and fragment mass error tolerance of 10 ppm and 0.06 Da, respectively. Carbamidomethylation was set as a fixed modification, while oxidation (M) was set as a variable modification. Quality filtering parameters were set to a False Discovery Rate (FDR) of 0.1%, detection of ≥1 unique peptide and a −10 log P protein score of ≥50. The Common Repository of Adventitious Proteins (CRAP; www.thegpm.org/crap/) database was included during the spectral searches to eliminate common contaminants effectively. The raw mass spectrometry data have been made available at the ProteomeXchange Consortium *via* the PRIDE partner repository (Perez-Riverol et al., 2019), with the data identifier PXD037171. The relative abundance of each toxin hit present in a gel band was estimated by quantifying the area under the spectral intensity curve (AUC) relative to the total AUC of all toxin hits in all bands. The mean spectral intensities retrieved from PEAKS were normalised across bands by densitometric estimation of the proportion of the area of the respective band (Tan et al., 2017). Thus, the relative abundance of a toxin hit (X) was calculated as follows (here, N indicates the number of bands excised from the gel)

$$Relative\;abundance\;of\;X\;\left(\%\right)=\overset{N}{\underset{n=1}{\sum }}\frac{AUC\;of\;X\;in\;band\;B_{n\;}\times Proportion\;of\;the\;band\;B_{n}\;\left(\%\right)}{Total\;AUC\;of\;all\;toxin\;hits\;in\;band\;B_{n}\;}$$

### 2.3 Comparative tissue transcriptomics

#### 2.3.1 RNA isolation, library preparation and sequencing

Venom gland and thorax tissue samples were harvested from 94 individuals belonging to a single colony of *V. affinis* and snap frozen. Both of these tissue types were then homogenised separately, and the total RNA was isolated using the TRIzol™ Reagent (Invitrogen, Thermo Fisher Scientific, Waltham, MA, United States) following the manufacturer’s protocol. DNA contamination from the extract was removed using Turbo DNase (Thermo Fisher Scientific, MA, United States), followed by the second round of extraction with the TRIzol™ reagent. The purity and concentration of the isolated RNA samples were determined using an EPOCH 2 spectrophotometer (BioTek Instruments, Inc., United States). The integrity of the isolated RNA samples was assessed on a TapeStation system using RNA HS ScreenTape (Cat# 5067–5579; Agilent Technologies, Santa Clara, CA, United States), and samples that passed quality checks were selected for sequencing. cDNA libraries were generated using the NEBNext^®^ Ultra™ RNA Library Prep Kit (New England Biolabs, Ipswich, MA, United States), and sequenced on an Illumina HiSeq X platform (2 × 150 bp paired-end with a sequencing depth of 20 million reads). The raw data has been submitted to NCBI’s Sequence Read Archive (SRA) (Bioproject: PRJNA886082).

#### 2.3.2 Transcriptome assembly and annotation

Transcriptome data were curated to retain only high-quality reads using Trimmomatic (Bolger et al., 2014). The quality filtering steps involved the removal of adapters, leading and trailing low-quality bases (<3), short reads (<20 bases) and low-quality reads determined using a sliding window (quality score: <25; window size: 4). The quality of data pre- and post-trimming was assessed using FastQC (Andrews, 2010). Trimmed data from both thorax and venom gland tissues were then *de novo* assembled as a superassembly using Trinity (Grabherr et al., 2011) with default settings: not strand-specific; minimum contig length: 200. An assembly was also built using a minimum contig length of 150 to account for mastoparans. The completeness of the transcriptome assembly was tested using BUSCO (Simão et al., 2015), and the reads were mapped back onto the assembly using BowTie2 (Langmead and Salzberg, 2012) to evaluate the quality of the assembly. TransDecoder (Haas et al., 2013) was used to predict the coding regions from contigs, followed by annotation using BLAST searches against the NCBI-NR database [May 2022; (Altschul et al., 1990)].

#### 2.3.3 Transcriptome quantification and differential expression analysis

Transcript abundances were calculated using the RSEM package (Li and Dewey, 2011) and expressed in transcripts per million (TPM) units. Pairwise differential expression analysis was performed using a novel non-parametric approach implemented in NOISeq (Tarazona et al., 2015).

### 2.4 Biochemical characterisations of *V. affinis* venom

#### 2.4.1 Hyaluronidase assay

A previously described method was used to determine the hyaluronidase activity (Di Ferrante, 1956; Laxme et al., 2019). Briefly, the reaction mixture containing acetate buffer (0.2 M sodium acetate-acetic acid, 0.15 M NaCl, pH 6.0), 1 mg/ml of hyaluronic acid (Sigma-Aldrich, United States) and 2.5 μg of crude venom in a final volume of 100 μl was incubated at 37°C for 20 min. The reaction was subsequently stopped with 0.2 ml of 2.5% (w/v) cetyltrimethylammonium bromide (CTAB) dissolved in 2% NaOH (w/v). An EPOCH 2 (BioTek) microplate reader was used to record the absorbance values at 400 nm. The activity was quantified in a turbidity reduction unit (TRU), which is defined as the amount of enzyme required to reduce 50% of turbidity in the reaction and expressed as TRU mg^−1^ min^−1^.

#### 2.4.2 Colorimetric phospholipase assay

A chromogenic lipid substrate, 4-nitro-3-(octanoyloxy) benzoic acid (NOB; Enzo Life Sciences, New York, NY, United States), was used to assess the phospholipase activity of *V. affinis* venom using a previously described protocol (Holzer and Mackessy, 1996; Freitas-de-Sousa et al., 2020). Briefly, a 5 μg venom sample was incubated with 500 mM NOB substrate dissolved in a 200 µl reaction buffer (10 mM Tris-HCl, 10 mM CaCl2, 100 mM NaCl, pH 7.8) at 37°C for 40 min. During this, the kinetics of the assay was monitored by measuring absorbance at 425 nm every 10 min *via* an EPOCH 2 microplate spectrophotometer (BioTek Instruments, Inc. United States). For plotting a standard curve, a similar assay was performed using varying NOB substrate concentrations and 4 N NaOH. The amount of NOB substrate cleaved in nmol per minute per mg of the venom was calculated by extrapolation of the standard curve.

### 2.5 Pharmacological assays

#### 2.5.1 Blood coagulation

Dose-dependent (1–30 μg) effect of the crude *V. affinis* venom on activated partial prothrombin time (aPTT) and prothrombin time (PT) was assayed against human platelet-poor plasma (PPP). Five milliliters of blood was drawn from healthy volunteers with informed consent and collected in 3.2% sodium citrate coated vacutainers. These tubes were centrifuged at 3,000 revolutions per minute for 15 min at 4°C. In the aPTT assay, the reaction mixture containing cephaloplastin reagent (a phospholipid) and 0.02 M calcium chloride was mixed with 50 µl PPP and varying amounts of the crude venom (1, 5, 10, 15, and 30 μg). The PT assay was performed by mixing 50 µl PPP with varying concentrations of venom and prewarmed thromboplastin reagent (a tissue factor; Uniplastin; Tulip diagnostics, Mumbai). The time to form the first clot was recorded using a Hemostar XF 2.0 coagulometer (Tulip Diagnostics).

#### 2.5.2 Haemolytic assay

To evaluate the haemolytic effects of *V. affinis* venom, an assay was conducted using previously described methods (Maisano et al., 2013; Laxme et al., 2019). Varying amounts of venom (1, 5, 10, 15, and 30 μg) were treated with a fixed concentration of RBCs (2% v/v solution) from healthy volunteers. Briefly, blood was centrifuged to separate RBCs from the plasma, followed by resuspension of the separated RBCs in phosphate buffer saline (PBS; pH 7.4). This was followed by an overnight incubation, after which the absorbance was measured at 540 nm in an EPOCH 2 microplate spectrophotometer (BioTek Instruments, Inc., United States). The relative haemolytic activities were calculated using 0.5% Triton X as a positive control (treated as 100% activity).

#### 2.5.3 Insect-specific toxicity

*V. affinis* venom in varying doses was injected into crickets (*Acheta domesticus*) to assess its insect-specific potency. Five dose groups (3, 6, 9, 12 and 15 μg) with two crickets in each group were used in these assays. Two microliters of the venom, reconstituted in insect saline buffer (pH 7.4), was injected with a Hamilton Gastight syringe into the abdomen of crickets. These animals were then observed and the number of paralysed and non-paralysed crickets was counted 2 hours post-injection (Rungsa et al., 2016). Crickets, which were unable to upright themselves upon being turned upside down, were considered paralysed. In addition, 24 hours-post injections, we also counted the number of dead and live individuals to estimate the insect-specific toxicity.

#### 2.5.4 Phylogenetic reconstructions

To infer the molecular evolution of vespid wasp venom toxin encoding genes, including cysteine-rich secretory protein of the CAP (antigen five and pathogenesis-related proteins) superfamily, dipeptidyl peptidase (DPP), hyaluronidase (HYL), phospholipase A1 (PLA_1_) and serine protease, nucleotide sequences were retrieved from the transcriptome assembly generated in this study, as well as from the NCBI NR database using BLAST searches against ants, wasps and bees (taxid: 7399), as well as the family Vespidae (taxid: 7438) (Altschul et al., 1990). The retrieved accessions were then pruned to retain only Vespidae members. Sequences were aligned using MUSCLE (Edgar, 2004), manually inspected for gaps and curated (Supplementary Data File S1). Subsequently, model selection was performed for each dataset in IQTree using Model Finder (Nguyen et al., 2015; Kalyaanamoorthy et al., 2017) and a Bayesian phylogeny was constructed using MrBayes (Ronquist et al., 2012). The simulation was executed on four Markov Chain Monte Carlo (MCMC) runs, each running nine chains simultaneously. A standard deviation of the split frequency (sdsf) of 0.01 was predefined as a convergence diagnostic. From the posterior probability distribution, trees and the corresponding model parameters were sampled every 100^th^ generation. The effective sample size (ESS) for sampled parameters was checked using Tracer (Rambaut et al., 2018). If the ESS for parameters post-convergence at sdsf of 0.01 was <250, the analysis was run for at least 20 million generations to improve ESS. Post-convergence, the initial 25% of the sampled trees and model parameters were discarded as “burn-in” and used the rest for generating the final tree topology using a majority-rule consensus. Support values for branches were evaluated using Bayesian Posterior Probabilities (BPP), and the FigTree package was used to visualise trees (Rambaut, 2012).

#### 2.5.5 Rate of evolution of vespid venom toxins

Site-specific maximum likelihood models from CodeML of the PAML (Phylogenetic Analysis by Maximum Likelihood) package were employed to identify the regimes of natural selection influencing the evolution of vespid venom toxins (Yang, 2007). An omega value (*ω*) that corresponds to the ratio of non-synonymous (nucleotide changes that alter the coded protein) to synonymous (nucleotide changes that do not alter the coded protein) substitutions was estimated. An *ω* value of less than, greater than or equal to one signifies negative selection, positive selection, and neutral evolution, respectively. Nested model [M7 (null) and M8 (alternate)] comparisons were implemented to detect the signatures of positive selection. The statistical significance of the outcomes was determined by performing a likelihood ratio test (LRT). Further, to identify the amino acid sites under positive selection, a Bayesian approach was employed using the Bayes Empirical Bayes (BEB) approach (Yang et al., 2005). Additionally, to uncover episodic and pervasive effects of selection, the Mixed Effect Model of Evolution [MEME; (Murrell et al., 2012)] and the Fast Unconstrained Bayesian AppRoximation [FUBAR; (Murrell et al., 2013)] analyses were performed on the Datamonkey web server (Weaver et al., 2018).

#### 2.5.6 Structural analysis

Phyre2 web server was used to generate three-dimensional (3D) homology models for the genes of interest (Kelley et al., 2015). The evolutionary variability of vespid toxins was determined using the ConSurf web server (Ashkenazy et al., 2016). Additionally, positively selected sites were mapped on the homology models and visualised using PyMOL (The PyMOL Molecular Graphics System, Version 2.0, Schrödinger, LLC.).

## 3 Results

### 3.1 The venom profile

**FIGURE 1:**
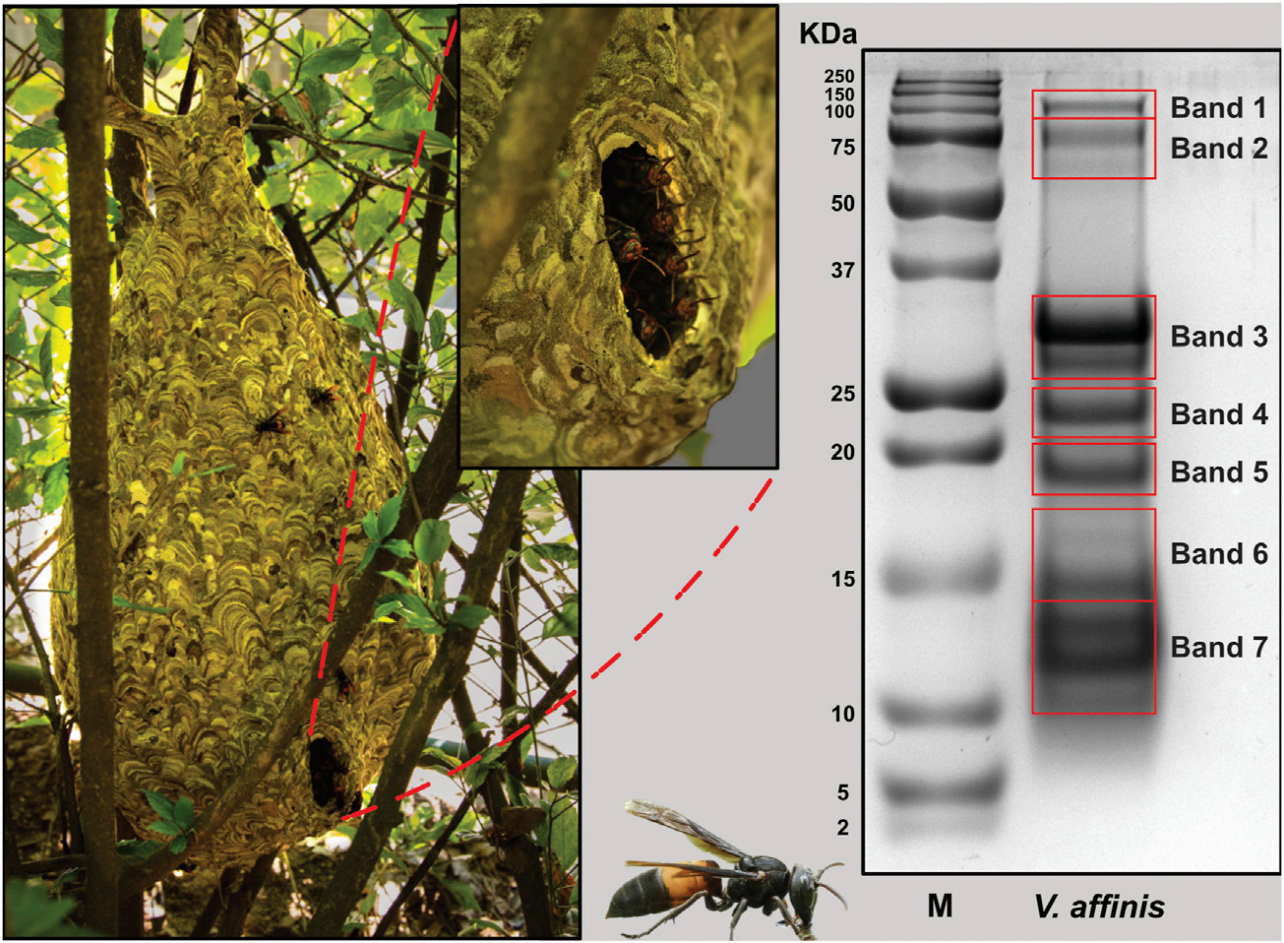
The lesser banded hornet and its venom composition. Images of *V. affinis* and its nest are shown (panel A) alongside the SDS-PAGE profile of the venom (panel B). Red boxes in panel B are indicative of the bands that were excised for mass spectrometric analysis.

The overall proteomic profile of *V. affinis* venom was evaluated using reducing SDS-PAGE, which revealed that the venom is composed of diverse components with molecular weights ranging from 10 to 100 kDa (Figure 1). Low-molecular-weight components within the 10–30 kDa weight range dominated the venom profile, while minor bands were also documented between 75 and 100 kDa. To establish the identity of these toxins, we further excised the gel and subjected individual bands to mass spectrometry.

### 3.2 Comparative tissue transcriptomics

Sequencing of *V. affinis* tissues on Illumina’s HiSeq X platform resulted in 23,163,263 and 22,630,858 sequences from the venom gland and thorax, respectively (Supplementary Table S1). The *de novo* transcriptome assembly generated with these sequences produced N50 statistics of 2,274 and 73,947 transcripts. Completeness analyses of the assembly with BUSCO revealed that 88.75% of the BUSCO groups have complete gene representation (single-copy or duplicated), while 1.97% are only partially recovered, and 9.28% are missing. Furthermore, aligning quality-filtered reads onto the *de novo* assembly revealed the superior nature of the generated transcriptome, as it identified an overall alignment rate of 95.69%. Annotation of transcript sequences, followed by differential expression analyses, revealed the overexpression of numerous toxin-coding transcripts in the venom gland in comparison to the thorax tissue (Supplementary Table S2). Over half of the *V. affinis* venom gland transcriptome was dominated by arginine kinase (30.38%) and phospholipase A_1_ (PLA_1_: 25.67%) toxins. This was followed by hyaluronidase (7.63%), aminopeptidase (5.84%), neprilysin (4.84%), chitinase (4.10%) and acid phosphatase (4.03%). Additionally, transcripts for many other toxins, such as carboxypeptidase, dipeptidyl peptidase (DPP), peroxiredoxin, phospholipase B (PLB), serine protease, 5′-nucleotidases (5′-NTD), metalloproteinase inhibitor (MPi) and antigen 5, and pathogenesis-related one proteins (CAP), including cysteine-rich secretory proteins (CRISP), were also recovered.

### 3.3 Mass spectrometry

**FIGURE 2:**
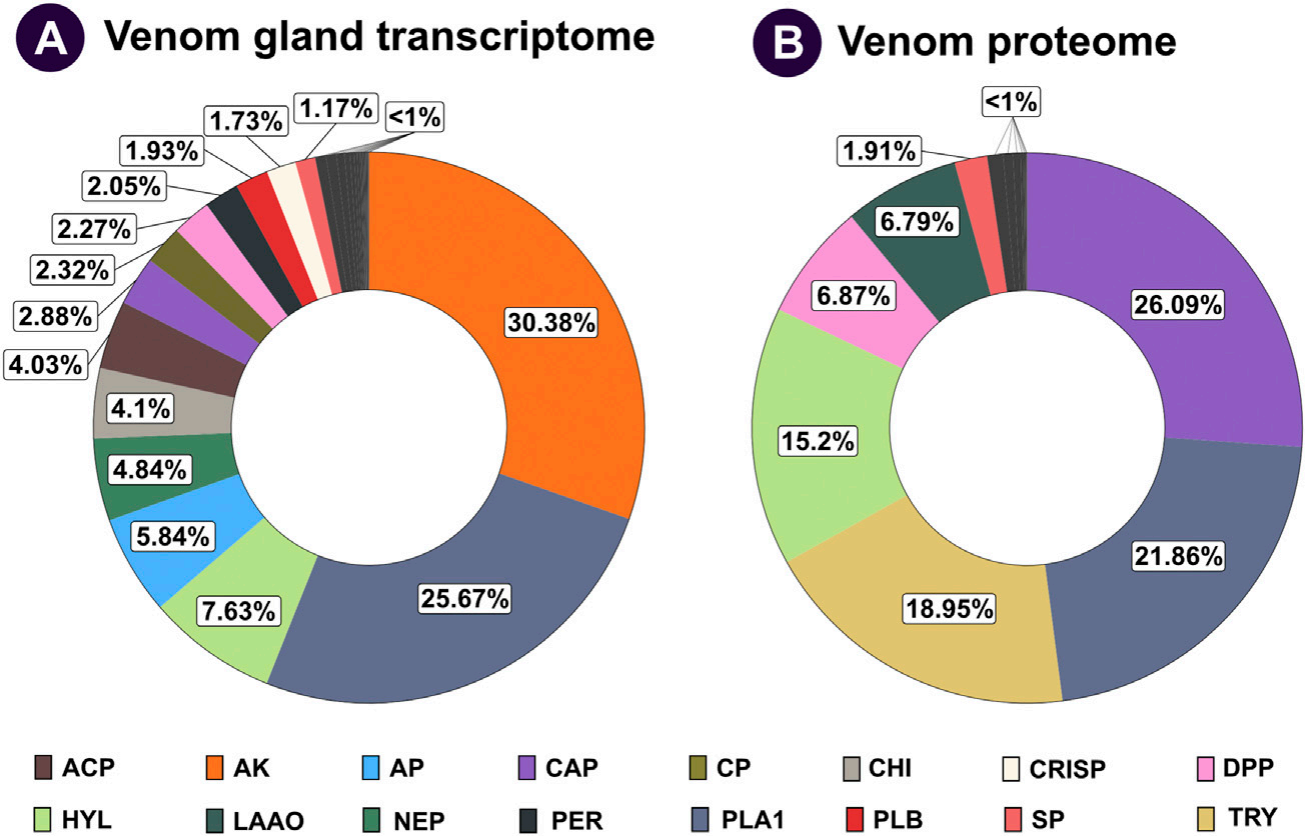
Comparative venom gland transcriptome and venom proteome of the lesser banded hornet. The venom gland transcriptome and venom proteome of *V. affinis* are shown as doughnut charts in panels **(A)** and **(B)**, respectively. Each toxin is uniquely colour coded, and its relative abundance is mentioned in percentages: ACP: acid phosphatase; AP: aminopeptidase; AK: arginine kinase; CAP: antigen 5, and pathogenesis-related one proteins; CP: carboxypeptidase; CHI: chitinase; CRISP: cysteine-rich secretory proteins; DPP: dipeptidyl peptidase; HYL: hyaluronidase; LAAO: L-amino acid oxidases; NEP: neprilysin; PER: peroxiredoxin; PLA_1_: phospholipase A_1_; PLB: phospholipase B; SP: serine protease; TRY: trypsin.

Individual protein bands excised from SDS-PAGE of *V. affinis* venom were subjected to tandem mass spectrometry. The resultant spectra were searched against the UniProtKB database, as well as the tissue transcriptomes assembled in this study. This strategy identified 114 non-redundant protein groups, among which were 28 toxin proteins belonging to 14 groups (Figures 2A,B; Supplementary Table S3; Supplementary Data File S2). In contrast to their limited abundance in the venom gland transcriptome (2.88%), the venom proteome was dominated by the CAP superfamily of toxins (26.09%; Figure 2), revealing a nearly ten-fold increase in translation. This was followed by trypsin (18.95%), for which we only recovered a minor fraction of transcripts. Similarly, hyaluronidase (15.20%) and DPP (6.87%) were other such components that had a very high translation in comparison to their transcriptomic abundances (7.63% and 2.27%, respectively). In comparison, the abundance of PLA_1_ toxins matched nearly perfectly between the venom gland transcriptome and the venom proteome (25.67% and 21.86%, respectively). Surprisingly, proteomic characterisation did not detect arginine kinase, for which we recovered the highest number of transcripts from the venom gland (30.38%), suggesting that these are, perhaps, physiological proteins and not toxins. Similarly, we did not find neprilysin, acid phosphatase, PLB, CRISP, 5′-NTD, mastoparan, MPi and apyrase in the venom, although transcripts were detected for each of these genes. On the other hand, we also found L-amino acid oxidase (LAAO: 6.79%) in the venom. Other components, including serine protease (1.91%), aminopeptidase (0.93%), carboxypeptidase (0.58%), PLA_2_-inhibitor (0.45%), PLA_2_ (0.17%), chitinase (0.11%), chymotrypsin (0.08%) and peroxiredoxin (0.02%) were found in the venom proteome, albeit in limited amounts (Figures 2A,B; Supplementary Table S3; Supplementary Data File S2).

To understand the functional profile of *V. affinis* venom, we subjected the crude venom to various *in vitro* biochemical and pharmacological assays. We evaluated *V. affinis* venom against the venoms of the “big four” snakes to assess the relative efficiencies.

#### 3.3.1 Hyaluronidase activity

**FIGURE 3:**
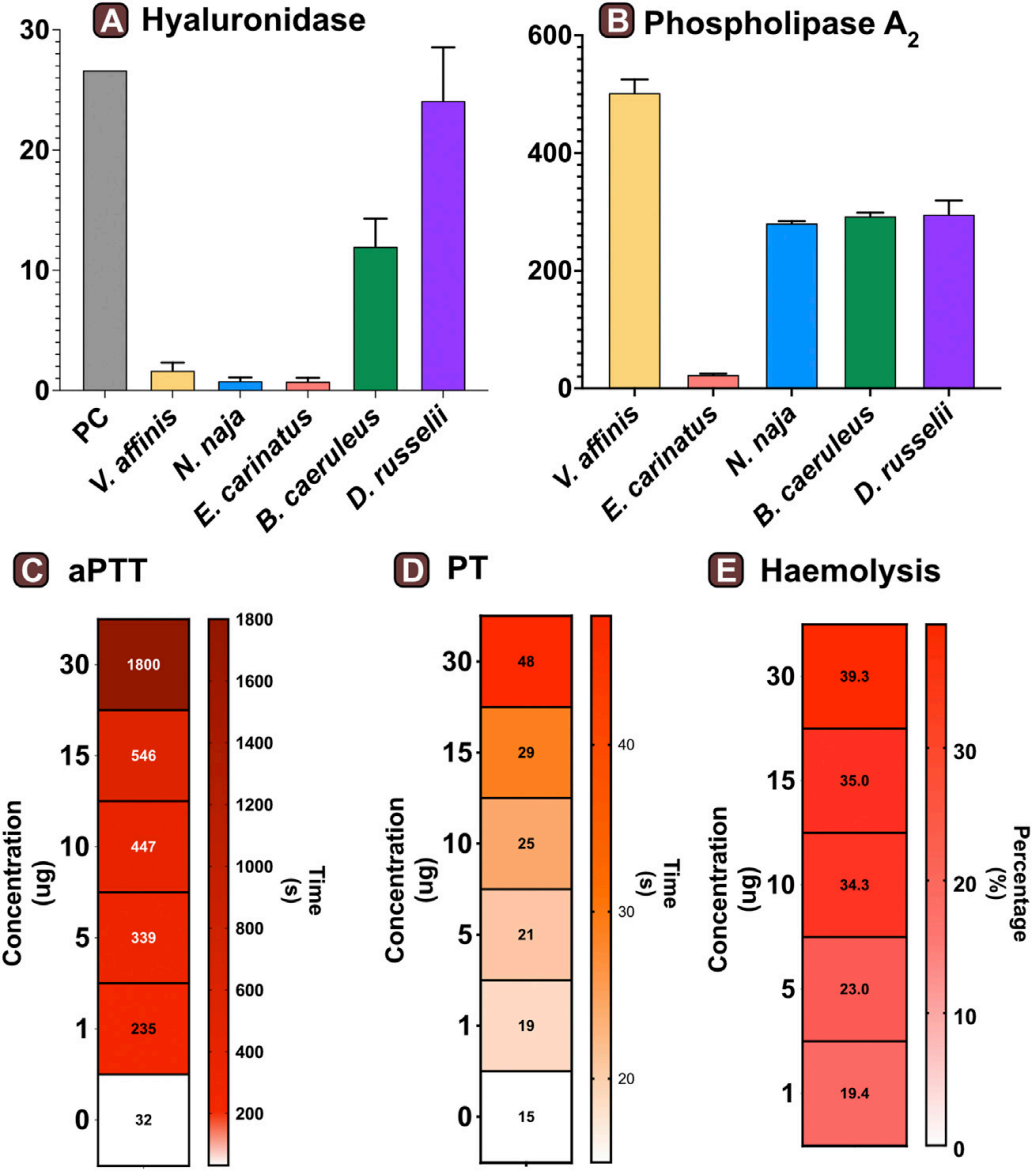
Functional profile of *Vespa* venom. Graphs in panels **(A)** and **(B)** represent the hyaluronidase (TRU·mg^−1^·min^−1^) and phospholipase (nmol·ng^−1^·min^−1^) activity of *V. affinis* venom in comparison to the venoms of the “big four” Indian snakes. Here, the error bars represent the standard deviation, and PC denotes the positive control. Heatmaps depict the anomalies caused by wasp venom on the blood coagulation cascade *via* **(C)** intrinsic and **(D)** extrinsic pathways. The colour scales in these panels represent the time in seconds, while the time required to form the first fibrin clot is denoted within each cell. Panel **(E)** depicts the haemolytic potential of *V. affinis* venom as a percentage relative activity to the positive control (0.5% Triton X). The numbers within cells show the percentage of haemolysis.

We assessed the hyaluronidase activity of *V. affinis* venom in comparison to the venoms of “big four” Indian snakes by incubating a fixed amount of the venom (2.5 μg) with hyaluronic acid. The venom of the lesser banded wasp showed limited hyaluronidase activity (1.68 TRU mg^−1^ min^−1^), which was relatively higher than that of *N. naja* (0.81 TRU mg^−1^ min^−1^) and *E. carinatus* (0.78 TRU mg^−1^ min^−1^). However, the highest activities were exhibited by *D. russelii* (24.13 TRU mg^−1^ min^−1^) and *B. caeruleus* (12 TRU mg^−1^ min^−1^) venoms, which were nearly 14 and 7 times higher than that of the *V. affinis* venom*,* respectively (Figure 3A).

#### 3.3.2 Phospholipase activity

Consistent with the results of venom proteomics (electrophoretic patterns and mass spectrometry) that identified PLA_1_ as amongst the major venom components, a significant phospholipase activity was documented in the *V. affinis* venom (502.76 nmol·ng^−1^·min^−1^; Figure 3B). The phospholipase activity of *Vespa* venom was significantly higher than that of the ‘big four’ Indian snakes (*p* < 0.05), being nearly twice as much in comparison to *N. naja* (211.88 nmol·ng^−1^·min^−1^), *B. caeruleus* (293.26 nmol·ng^−1^·min^−1^) and *D. russelii* (217.09 nmol·ng^−1^·min^−1^), and as much as 21 times greater than that of *E. carinatus* (23.60 nmol·ng^−1^·min^−1^).

#### 3.3.3 Venom-inflicted coagulopathy

The effects of the lesser banded wasp venom on the intrinsic and extrinsic blood coagulation cascades were assessed by performing aPTT and PT assays, respectively. The coagulation time of the control plasma without the venom was comparatively evaluated with the test plasma that was mixed with various concentrations of the wasp venom. The results of these coagulation assays revealed that *V. affinis* affects both the intrinsic and extrinsic coagulation cascades and acts as a highly potent anticoagulant in a dose-dependent manner (Figures 3C,D). *V. affinis* venom was found to significantly target the intrinsic coagulation cascade, more so than the extrinsic pathway, as the lowest concentration of the venom (1 μg) significantly increased the aPTT clotting time to 235 s in comparison to 32 s of control plasma. This clotting time increased with the increase in venom concentrations to a point where clot formation was completely prevented even after 1800 s (or 30 min) against the highest venom concentration (30 μg). In contrast, the effects on the external coagulation pathway were not as pronounced. However, an increase in time taken to form the first fibrin clot was noticed with an increase in venom concentrations, with the highest venom concentration (30 μg) delaying the clot formation by 40 s compared to the control plasma (15 s).

The haemolytic potential of *V. affinis* venom was comparatively tested against Triton X (positive control) and the venoms of the “big four” Indian snakes. Varying concentrations of the venom were added to a 2% solution of RBC from healthy volunteers. In these tests, the *V. affinis* venom exhibited significantly higher haemolytic activity than all of the ‘big four’ snakes at any given concentration (*p* < .05; Figure 3E and Supplentary Figure S1). The haemolytic activity was found to vary in a dose-dependent manner. Even the lowest concentration (1 μg) of *V. affinis* was found to exhibit a haemolytic activity nearly ten times higher than that of *N. naja*. At the highest concentration tested (30 μg), *V. affinis* exhibited nearly 40% relative haemolytic activity, which was twice as much as that of *N. naja*. *E. carinatus* exhibited the lowest haemolytic activity amongst all the venoms tested, which was 7.7% lower than that of *V. affinis* at the highest concentration.

### 3.4 Insect-specific toxicity

**FIGURE 4:**
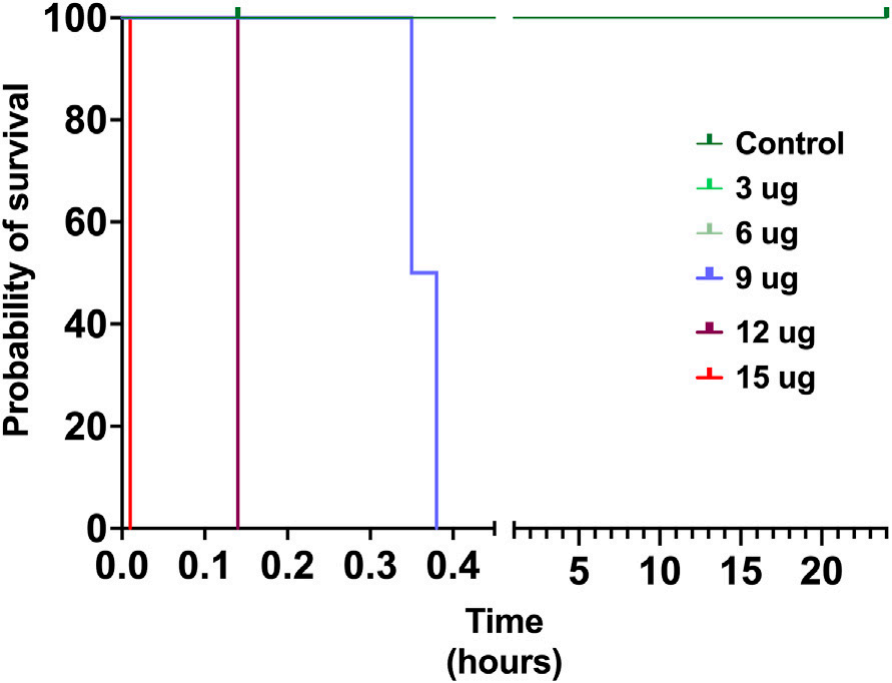
Insect specific toxicity. This Kaplan-Meier survival plot depicts the survival probability of crickets injected with the *V. affinis* venom (2 per dose group), where the x-axis depicts the time in hours, and the y-axis the probability of survival. Curves represent various doses of venoms administered into crickets. Experimental animals were monitored over a period of 24 h.

The toxicity of *V. affinis* venom on insects was tested using the domestic cricket, *A. domesticus*. When we injected the wasp venom into the abdomen of crickets, it did not exhibit any deleterious effects (paralytic or fatal) at lower concentrations of up to 6 μg, even after 24 h (Figure 4; Supplementary Table S4). However, venom concentrations of over 9 μg proved fatal to these insects just an hour after injection. As the concentration was increased to 12 μg, mortality was recorded in no more than 14 min of injection, with one of the subjects being paralysed within 9 min. The highest concentration of *V. affinis* venom (15 μg) proved to be potently lethal to crickets as both subjects displayed immediate mortality post-injection.

### 3.5 Molecular evolution of the vespid toxin arsenal

To determine the regime of natural selection shaping the evolution of wasp venom encoding genes (Figure S2-S6), we estimated the ratio of non-synonymous (nucleotide changes that alter the coded protein) to synonymous (nucleotide changes that do not alter the coded protein) substitutions, also known as omega (*ω*). Our analyses revealed that all of the investigated toxin-encoding genes were evolving under the strong influence of negative selection, with *ω* ranging from 0.15 to 0.44 (Table 1). The only exception was the DPP clade 1, which was characterised by an *ω* of 3, suggesting that it may have experienced positive selection. The BEB method that detects sites evolving under positive selection identified a large number of positively selected (PS) sites in the serine protease dataset (PS: 15) and a limited number of sites in CRISP (PS: 4) and phospholipase A_1_ (PS: 2) datasets. No such sites were detected in DPP clades 1 to 4 and hyaluronidase. MEME identified a significant effect of episodic selection (*p* < .05) on PLA1 (PS: 35), serine protease (PS: 34), CRISP (PS: 10) and DPP clade 3 (PS: 2), with no signatures detected for DPP clades 1, 2 and 4. FUBAR did not detect any pervasively diversifying sites except in DPP clade 3 (PS: 1; BPP: 0.95). However, numerous sites evolving under the pervasive influence of negative selection (NS) were detected in PLA_1_ (NS: 154), serine protease (NS: 143), hyaluronidase (NS: 75), CRISP (NS: 49), DPP clade 3 (NS: 38), DPP clade 4 (NS: 27) and DPP clade 1 (NS: 10). FUBAR failed to detect the signatures of pervasive positive or negative selection on the DPP clade 2.

**TABLE 1:** The rate of molecular evolution of various vespid toxins.

Family	FUBAR^a^	MEME sites^b^	PAML^c^ (M8)
CRISP	ω > 1^d^: 0	10	4
	ω > 1^e^: 49		ω: 0.42
DPP clade 1	ω > 1^d^: 0	0	0
	ω > 1^e^: 10		ω: 3
DPP clade 2	ω > 1^d^: 0	0	0
	ω > 1^e^: 0		ω: 0.4
DPP clade 3	ω > 1^d^: 1	2	0
	ω > 1^e^: 38		ω: 0.33
DPP clade 4	ω > 1^d^: 0	0	0
	ω > 1^e^: 27		ω: 0.15
Hyaluronidase	ω > 1^d^: 0	20	0
	ω > 1^e^: 75		ω: 0.41
PLA_1_	ω > 1^d^: 0	35	2
	ω > 1^e^: 154		ω: 0.35
Serine protease	ω > 1^d^: 0	34	15
	ω > 1^e^: 143		ω: 0.44

^a^ : Fast Unconstrained Bayesian Approximation.

^b^ : Sites identified under the influence of episodic diversifying selection (0.05 significance) by the Mixed Effects Model Evolution (MEME).

^c^ : Positively selected sites detected by the Bayes Empirical Bayes approach implemented in M8. Sites detected at *p* ≥ .95.

^d^ : Sites experiencing pervasive diversifying selection at the posterior probability ≥0.95 (FUBAR).

^e^ : Sites experiencing pervasive purifying selection at the posterior probability ≥0.95 (FUBAR).

ω: mean dN/dS.

## 4 Discussion

### 4.1 The venom compositions and activities of clinically important wasps

Various enzymatic and non-enzymatic toxin proteins have been characterised from wasp venoms to date, including phospholipases, mastoparan, CAP, kinins and DPP (Yang et al., 2008; Lee et al., 2016; Rungsa et al., 2016; Abd El-Wahed et al., 2021). These toxins have been theorised to aid social wasps in defending against predators and subduing prey (Lee et al., 2016; Abd El-Wahed et al., 2021). Previously, *V. affinis* from Thailand has been shown to abundantly express phospholipases (39.99%), DPP (13.33%), CAP (12.06%) and hyaluronidases [10.41%; (Rungsa et al., 2016)]. Despite being separated by over 2,900 km, the venom composition of *V. affinis* from southern India was highly similar to its conspecifics in Thailand. In our proteomic analyses, we found CAP superfamily of toxins (26.09%) and phospholipases (21.86%) dominating the venom composition and constituting over half of the venom proteome. This was followed by trypsin (18.95%), hyaluronidase (15.2%), DPP (6.87%) and LAAO (6.79%). We also recovered other components, such as serine protease, aminopeptidase, carboxypeptidase, PLA_2_-inhibitor, PLA_2_, chitinase, chymotrypsin and peroxiredoxin, albeit in minor amounts. Consistent with *Vespa* venom transcriptomes and proteomes reported to date (HIRAI et al., 1981; Sookrung et al., 2014; Liu et al., 2015; Patnaik et al., 2016; Rungsa et al., 2016; Zhou et al., 2019), we did not detect melittin. However, when the crude venom was subjected to in-solution digestion, followed by mass spectrometry, we were able to identify mastoparans in the *Vespa* venom, albeit in very limited amounts, supporting their previous identification in the venom (HIRAI et al., 1981; Zhou et al., 2019). Interestingly, expression of the aforementioned venom toxins appears to be conserved across the genus *Vespa*, as these components were also reported in the venom of the greater banded hornet, *V. tropica* (Rungsa et al., 2016)*.* The incredibly similar venom profile of wasps spread over such a large geographical area is astonishing. Such a pattern of venom variation, or a lack thereof, is in stark contrast to the venoms of other animals, such as the medically important snakes, where venom variation has been documented across much smaller geographical scales (Gren et al., 2017; Senji Laxme et al., 2021a; Senji Laxme et al., 2021b; Rashmi et al., 2021).

Wasp envenoming is associated with diverse systemic effects, including rhabdomyolysis, acute kidney injury and anaphylaxis (Pramanik and Banerjee, 2007). Phospholipases are the major venom allergens in wasp venoms responsible for the lysis of lipid membranes, leading to tissue damage and inflammatory responses (Sookrung et al., 2014). The lysis of these biological membranes is accompanied by the release of arachidonic acid, which serves as a mediator for nociception and the throbbing pain in envenomed victims (Zambelli et al., 2017). In our phospholipase assays, the activity of *V. affnis* venom was found to be significantly higher than that of the clinically important “big four” Indian snakes, being nearly twice as potent as *N. naja*, *B. caeruleus* and *D. russelii* venoms*,* and over 21 times as potent as the *E. carinatus* venom (*p* < .05; Figure 3B). The significant haemolytic potential of *Vespa* venoms has also been attributed to phospholipases (Tuĭchibaev et al., 1988). Consistently, a very high haemolytic activity was observed in the venom of *V. affinis* from southern India. This activity was found to be significantly greater than the venoms of the “big four” Indian snakes under investigation (*p* < .05; Figure 3E). Moreover, vespid stings are characterised by pronounced anticoagulant effects that result from the action of venom phospholipases. Previous studies conducted on *V. orientalis* showed that the venom of this species targets and impairs the clotting time in both the intrinsic (PT) and extrinsic (aPTT) coagulation cascades, with a more prominent effect on the latter (Kornberg et al., 1988). A similar trend was seen in this study, where the time taken to form the first fibrin clot *via* the extrinsic cascade significantly increased (235 s), even at the lowest concentration of *V. affinis* venom (1 μg). Moreover, no clots were seen in the plasma even after 1800 s at the highest tested venom concentration [30 μg; Figure 3D; (Kornberg et al., 1988; Tan and Ponnudurai, 1992)].

Hyaluronidase is yet another major component previously reported from the venom of *V. affinis*. This enzymatic toxin is known to degrade hyaluronic acid, a vital component of the extracellular matrix (Necas et al., 2008). The presence of hyaluronidases in venom aids the diffusion of other toxic components and may also induce inflammation (Kemparaju and Girish, 2006). Although a previous study has reported high hyaluronidase activity of *V. affinis* venom (Kemparaju and Girish, 2006), being greater than the venoms of snakes and scorpions, we found relatively low activity when compared to *B. caeruleus* and *D. russelii*, and only marginally higher than that of *N. naja* and *E. carinatus* (Figure 3A).

### 4.2 Defensive venoms of vespid wasps

Most vespid wasps are known to live in large colonies and use stinging as a defensive behaviour against predators (Evans and Schmidt, 1990). Thus, to facilitate predator deterrence, it has been theorised that their venoms are evolutionarily optimised to induce severe pain and elicit an aggravated allergenic response in the target (Evans and Schmidt, 1990). The wasp stinger—a modified ovipositor-coupled with a complex biochemical arsenal—serves as an effective defence against predators. An in-depth characterisation of the wasp venom proteome and transcriptome revealed that it is rich in various types of hyperallergic components, including arginine kinase, CAP, hyaluronidase, maltase, and phospholipase (Figure 2; Supplentary Table S3), which have been previously reported from several hymenopterans (Bilo et al., 2005; Hoffman et al., 2005; Hemmer and Wantke, 2020). The CAP superfamily of proteins that constituted nearly one-fourth of the wasp venom proteome (∼26%), are well-known for their role in inhibiting various ion-channels, modulation of vascular permeability and eliciting IgE-mediated immune reactions (Tadokoro et al., 2020; Wangorsch et al., 2022). Similarly, hymenopteran PLA_1_s, which constitute 21.86% of the *V. affinis* venom, are shown to be hyperallergic (Sookrung et al., 2014). Hyaluronidase, yet another highly abundant toxin in *V. affini*s (15.20%), is also known to result in degradation of hyaluronic acid found in cell matrices, consequently acting as a “spreading factor” for other toxic components in the venom. Hyaluronidases have also been reported as a major allergen in hymenopteran venoms with potential to induce severe immunogenic reactions in hyperallergic individuals (Marković-Housley et al., 2000; Padavattan et al., 2007). Furthermore, the transcripto-proteomic approach in this study facilitated the identification of several toxin families that were not reported previously from the *V. affinis* venom (Table 2). For example, trypsin, which was recovered as the third major component (18.95%) in the venom, has been shown to interfere with the blood coagulation cascade and induce fibrinolysis (De Graaf et al., 2010). We also detected low levels of LAAO (6.79%), a toxin component responsible for the release of reactive oxygen species, resulting in apoptosis and cytotoxicity (Yu and Qiao, 2012). Arthropod venom serine proteases, which were detected in minor amounts, are capable of inducing the phenoloxidase cascade complement response in invertebrates (Zhang et al., 2004). They are also known to cause fibrinogenolysis and fibrinolysis in mammals (Choo et al., 2010). Furthermore, we also deleted lower amounts of components such as maltase, glucosidase, galactosidase and trehalase. While the allergenic activity of maltase has been previously described (Hemmer and Wantke, 2020), the allergenic/toxic potential of the other components is yet to be investigated. These findings clearly demonstrate that wasps of the genus *Vespa* are equipped with highly defensive venom. While certain venomous snakes are capable of yielding very large amounts of venom [up to 200–300 mg in a single bite: (Senji Laxme et al., 2021a; Senji Laxme et al., 2021b)], wasps rely on numbers to defend themselves and their kin. Human envenoming from wasps often involves stings from multiple individuals of the colony. Moreover, the pharmacologically rich venom can induce severe allergic reactions, facilitating their antipredator defence.

**TABLE 2:** Comparative proteomes of *Vespa* venoms.

Venom component	*V. tropica*	*V. affinis* (Thailand)	*V. affinis* (southern India)
CAP	10.3%	12.1%	26.09
Phospholipases	33.3%	37.9%	22.03
Trypsin	—	—	18.95
Hyaluronidase	25.1%	10.4%	15.2
DPP	9%	13.3%	6.87
LAAO	—	—	6.79
Serine protease	—	—	1.91

This table summarises the relative proportions of highly expressed toxins identified in the venoms of *V. tropica* and *V. affinis* from Thailand (Rungsa et al., 2016), in comparison to *V. affinis* from Southern India.

### 4.3 The largely conserved venoms of “ancient” clades

Venomous animals and their prey and predators are in an ever-escalating arms race, with both organisms devising novel strategies to counteract each other (Bdolah et al., 1997; Biardi et al., 2006; Rowe and Rowe, 2008; Drabeck et al., 2022). It is theorised that venom components involved in predator deterrence have lower rates of diversification than those employed for predation (Sunagar and Moran, 2015; Herzig et al., 2020). Moreover, animal venom proteins have been theorised to follow a “two-speed” mode of evolution, wherein the venom components of ancient evolutionary lineages exhibit a higher degree of sequence conservation. In contrast, those in relatively younger lineages are marked with an elevated rate of diversification (Sunagar and Moran, 2015). Several studies have uncovered this evolutionary trend for various venom toxins in both ancient (Sunagar and Moran, 2015; Baumann et al., 2018) and young venomous clades (Brust et al., 2013; Sunagar et al., 2013). The insects of the order Hymenoptera are amongst the relatively ancient venomous lineages, with their evolutionary origin dating back to over 230 MYA (Peters et al., 2017). Moreover, most hymenopteran animals are believed to chiefly employ venom for antipredator defence. In support of the “two-speed” and “defensive venom evolution” hypothese s, we uncovered a strong effect of negative selection on the evolution of the majority of wasp venom encoding genes (PS: 2 to 15; *ω*: 0.15 to 0.44; Table 1), which was also consistent with previous reports (Sunagar and Moran, 2015; Baumann et al., 2018). The venom arsenal of these ancient lineages can experience bouts of rapid diversification in the event of stark ecological and/or environmental shifts (Sunagar and Moran, 2015). Our findings suggest a significant effect of such episodic changes on toxin-encoding genes in *V. affinis*, as we found numerous sites undergoing episodic diversification (n = 2 to 35; Table 1). Moreover, the overall venom composition of *V. tropica* and *V. affinis*, including the two distant populations of *V. affinis* in India and Thailand (separated by a geographical distance of ∼2900 KM), appears to be largely conserved. This contrasts with the significant venom variation documented in evolutionarily younger venomous lineages that also employ venom for predation. A significant geographical variation is observed in these animals even across smaller geographical regions (Gren et al., 2017; Senji Laxme et al., 2021a; Senji Laxme et al., 2021b; Rashmi et al., 2021). Thus, our findings not only provide additional evidence supporting the defensive role of venom in wasps but also indicate that they follow a dual tempo of molecular evolution.

### 4.4 The biodiscovery potentials of wasp venoms

In addition to being capable of inducing clinically severe and life-threatening toxicities, wasp venoms have tremendous potential to augment drug design and venom-derived therapeutics. As phospholipases are known to activate platelet aggregation, the venom of *V. affinis*, being chiefly composed of PLA_1_, could be potentially harnessed to modulate haemodynamics (Yang et al., 2008). Moreover, *V. affinis* venom was found to significantly affect the intrinsic coagulation cascade and prevent blood coagulation for up to 1800 s or 30 min, which was the upper time limit of measurement for the coagulometer. Therefore, *V. affinis* venom is an excellent research candidate for discovering potent anticoagulant drugs. We also found transcripts encoding mastoparan in the venom glands of *V. affinis*. These proteins are known for their antitumor (da Silva et al., 2018; Henriksen et al., 2014; de Azevedo et al., 2015) and antimicrobial (Park et al., 1995; Yang et al., 2013) activities, as well as the ability to prevent biofilm formation in various bacterial species (Chen et al., 2018). With the help of comparative tissue transcriptomics, we were also able to detect traces of kinins and chemotactic peptides, which are reported to exhibit antitumor potential at lower concentrations (Kaushik et al., 2014; Yoon et al., 2016). Ironically, allergens found in wasp venoms, which trigger the release of histamine and result in life-threatening envenoming, could be leveraged in allergen-directed immunotherapy to prevent anaphylactic reactions (Gattinger et al., 2018). Similarly, venom proteins recently identified from wasps are promising in treating epilepsy—a complex neurological disorder (Silva JdC et al., 2020). Considering their specificity and dosage efficacy, resulting from millions of years of evolution, *Vespa* venoms are an ideal candidate for drug discovery research. Wasps also play a crucial ecological role in various multi-trophic interactions and pollination. They have evolved a venom that is extremely toxic to insects (Yasuhara et al., 1987; Konno et al., 2016), making them an excellent candidate as a biocontrol agent against pests of economically important crops.

## 5 Conclusion

In summary, our findings reveal that despite the large geographic distance separating the Indian *V. affinis* from its conspecifics in Thailand, the overall venom arsenal appears to be very well-conserved. Moreover, the conservation of venom profile was not just documented within the conspecifics. The venom composition of *V. affinis* was also found to be highly similar to that of *V. tropica*. In addition to being characterised by highly conserved venom profiles, wasp venoms were also found to lack sequence diversity, as the majority of venom-coding genes were found to be evolving under the significant influence of purifying selection. The overall constitution and activity of vespid wasp venoms clearly suggested their defensive nature. Finally, our findings reveal that *Vespa* venoms are rich in toxins with profound biodiscovery potential, making them an ideal candidate for biodiscovery research. Thus, we highlight the importance of research into the venoms of the neglected venomous lineages of the Indian subcontinent.

## Data Availability

The raw proteomics data generated for this study can be found at PRIDE Database (Accession No: PXD037171). The transcriptomics data presented in this study can be openly accessed via Sequence Read Archive (SRA) at NCBI (Bioproject: PRJNA886082).
