# Supplementary material for: Stings on wings: Proteotranscriptomic and biochemical profiling of the lesser banded hornet (Vespa affinis) venom
Source: Front Mol Biosci. 2022 Dec 19;9:1066793. doi: 10.3389/fmolb.2022.1066793 (PMC9806352; doi:10.3389/fmolb.2022.1066793)
Supplement: Supplementary file 1 [file DataSheet3.DOCX]

***Supplementary Material***

**Figure S1**. Haemolysis potential comparison between the venoms of *V. affinis* and the 'big four' medically relevant snakes of India.


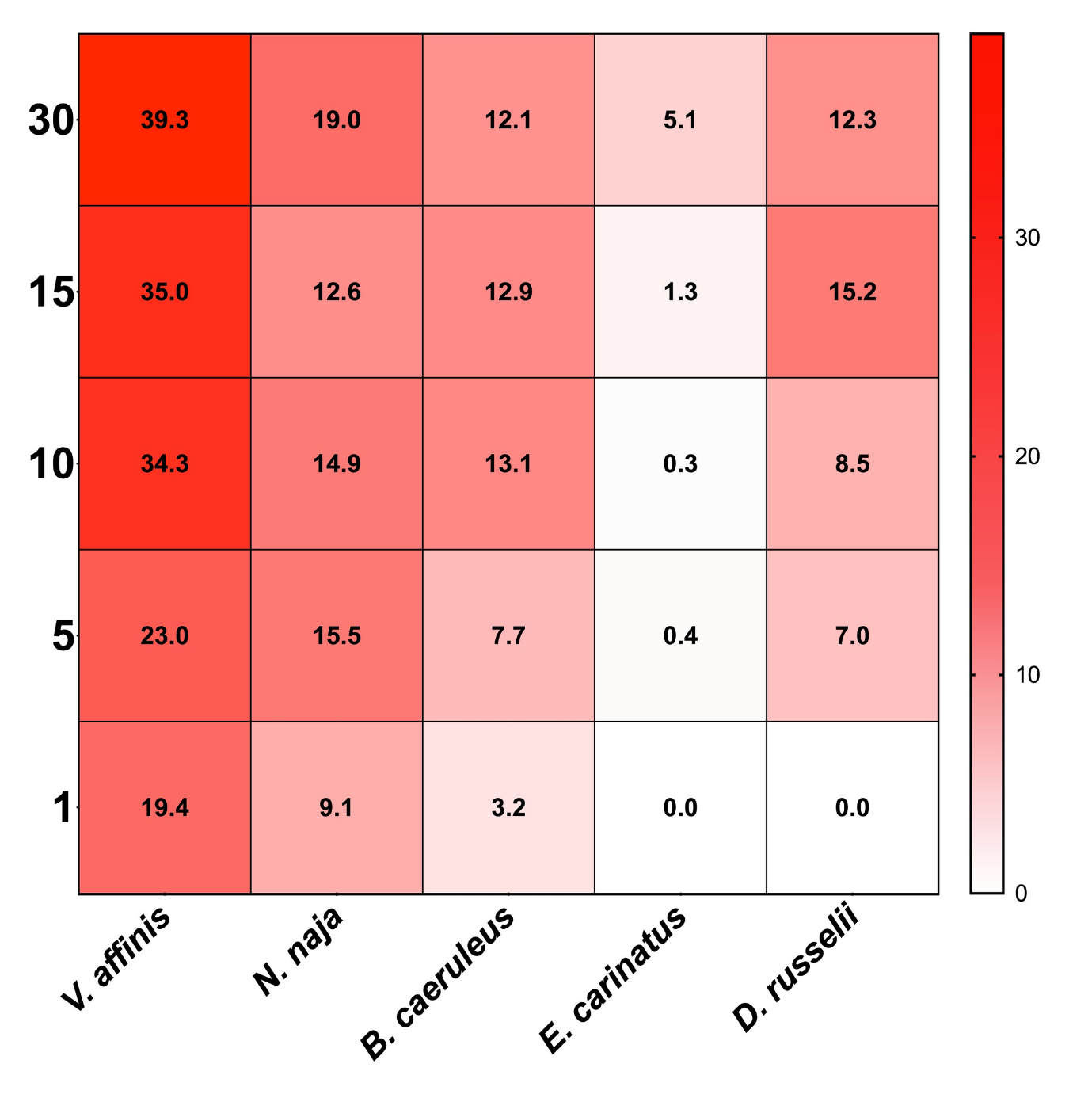


This figure depicts the haemolytic potential of *V. affinis* venom in comparison to the ‘big four’ medically important Indian snakes. The venom concentrations are indicated on the y-axis, while the names of the species are shown on the x-axis. The colour scale on the right indicates the percentage of relative activity with respect to the positive control (0.5% Triton X) and values within each cell represent the mean relative activity. The assay was performed in triplicates.

**Figure S2.** Bayesian phylogeny for vespid CAP family.


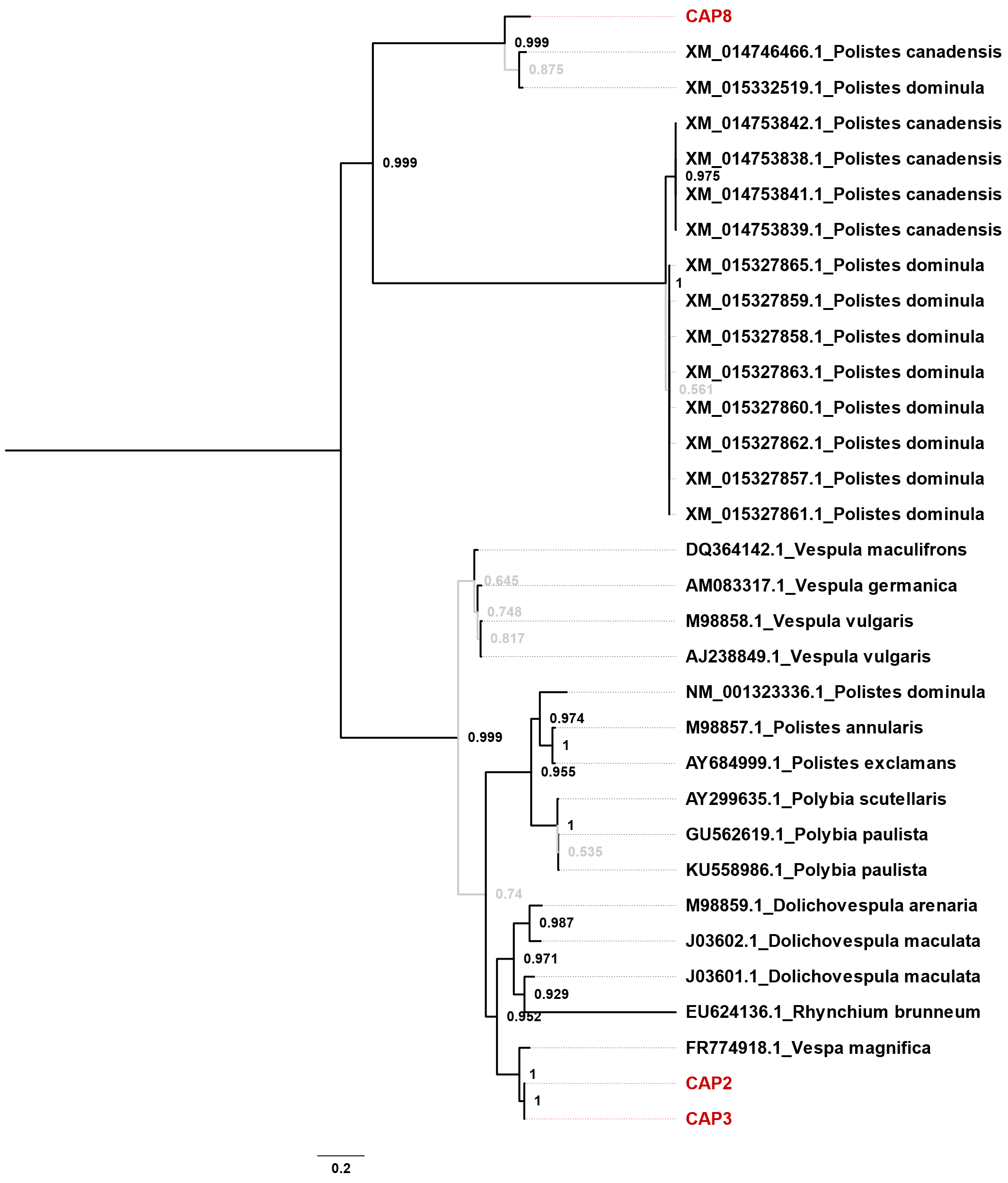


Phylogenetic relationships of the vespid CAP family are depicted here. The sequences generated in the current study are indicated in red colour. Thick black lines indicate well-supported branches (BPP ≥ 0.9).

**Figure S3.** Bayesian phylogeny for vespid DPP family.

**
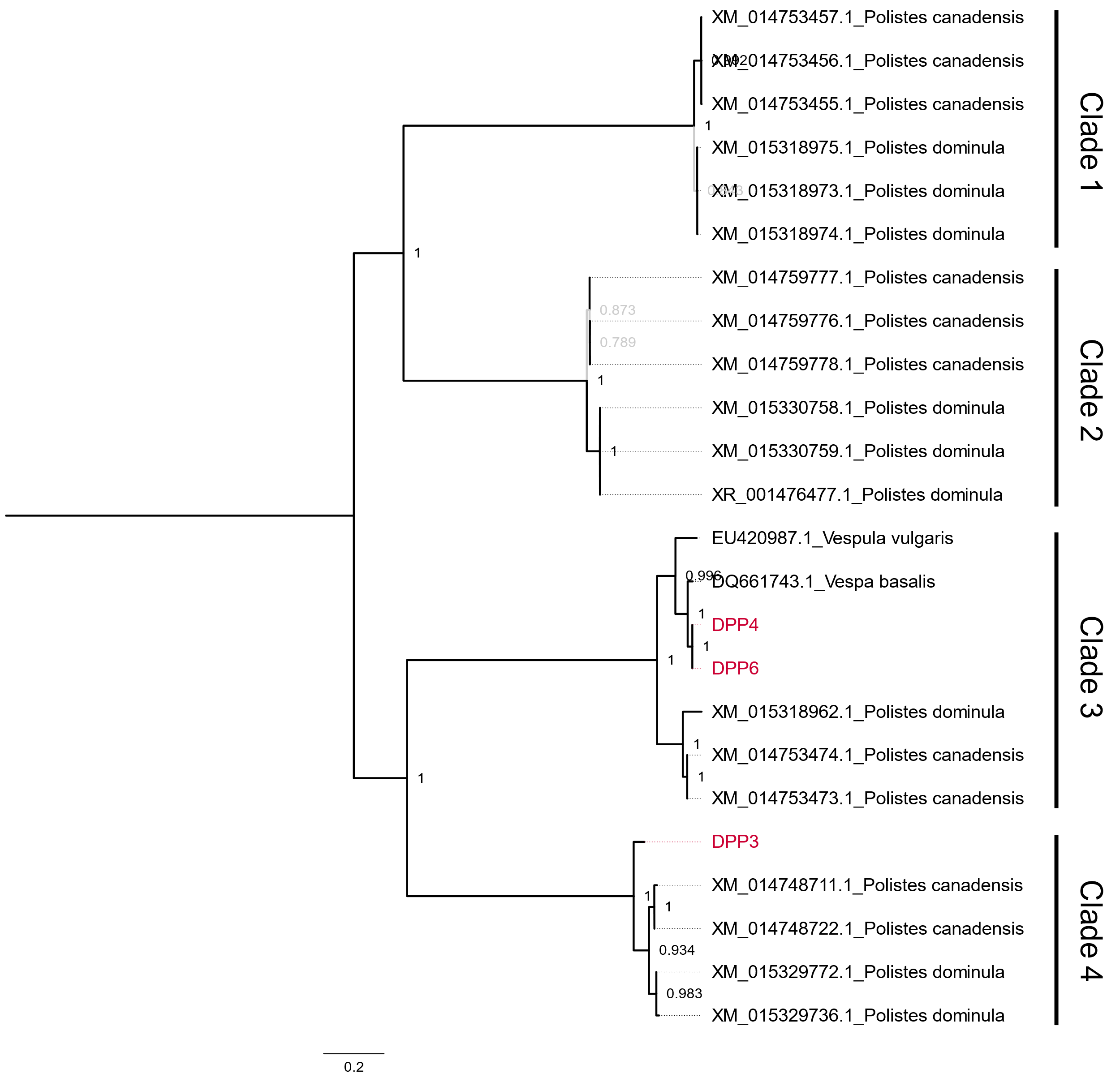
**

Phylogenetic relationships of the vespid DPP family are depicted here. The sequences generated in the current study are indicated in red colour. Thick black lines indicate well-supported branches (BPP ≥ 0.9). The individual clades have also been indicated in the figure.

**Figure S4.** Bayesian phylogeny for vespid HYL family.

**
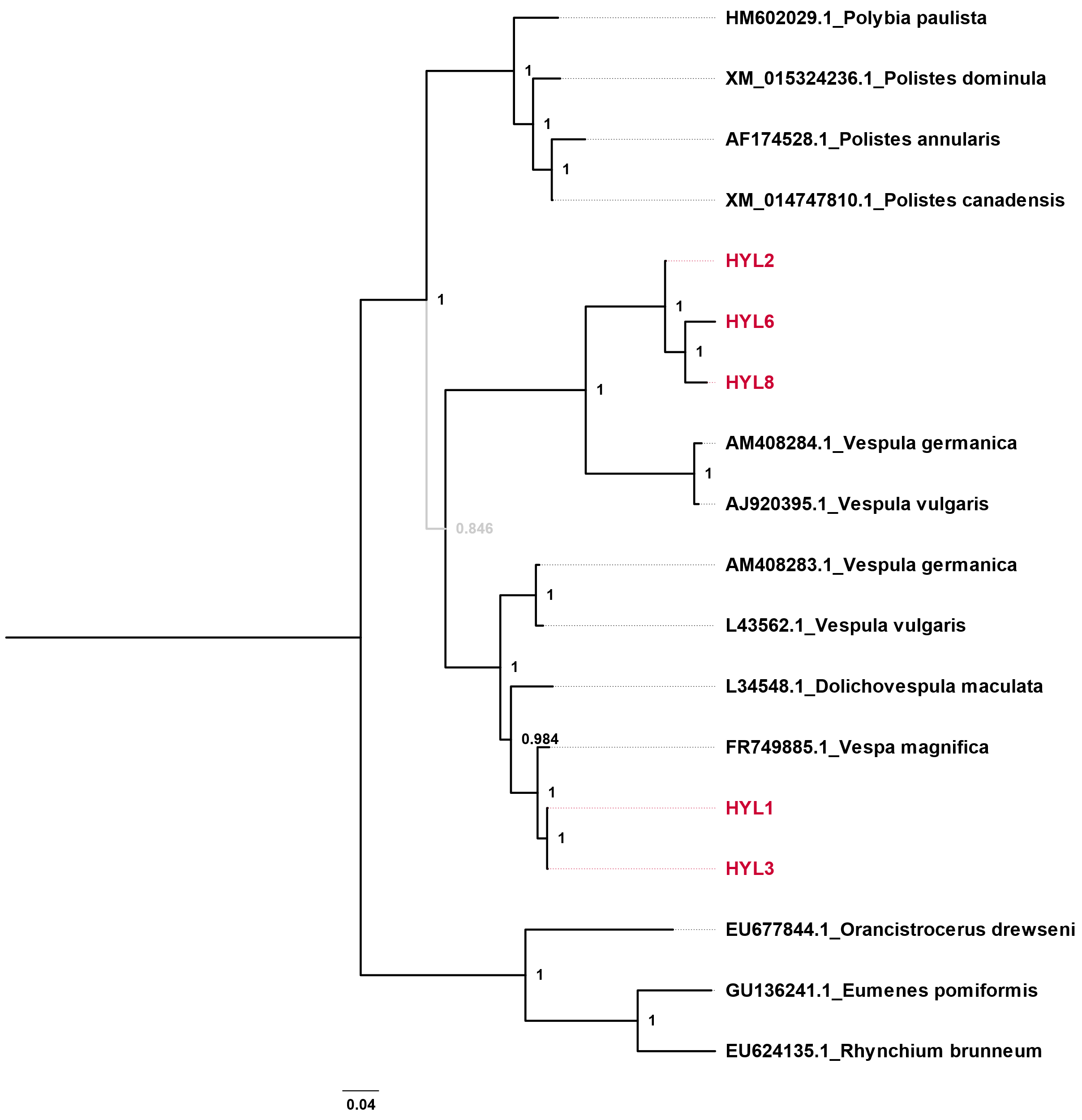
**

Phylogenetic relationships of the vespid HYL family are depicted here. The sequences generated in the current study are indicated in red colour. Thick black lines indicate well-supported branches (BPP ≥ 0.9).

**Figure S5.** Bayesian phylogeny for vespid PLA_1_ family.

**
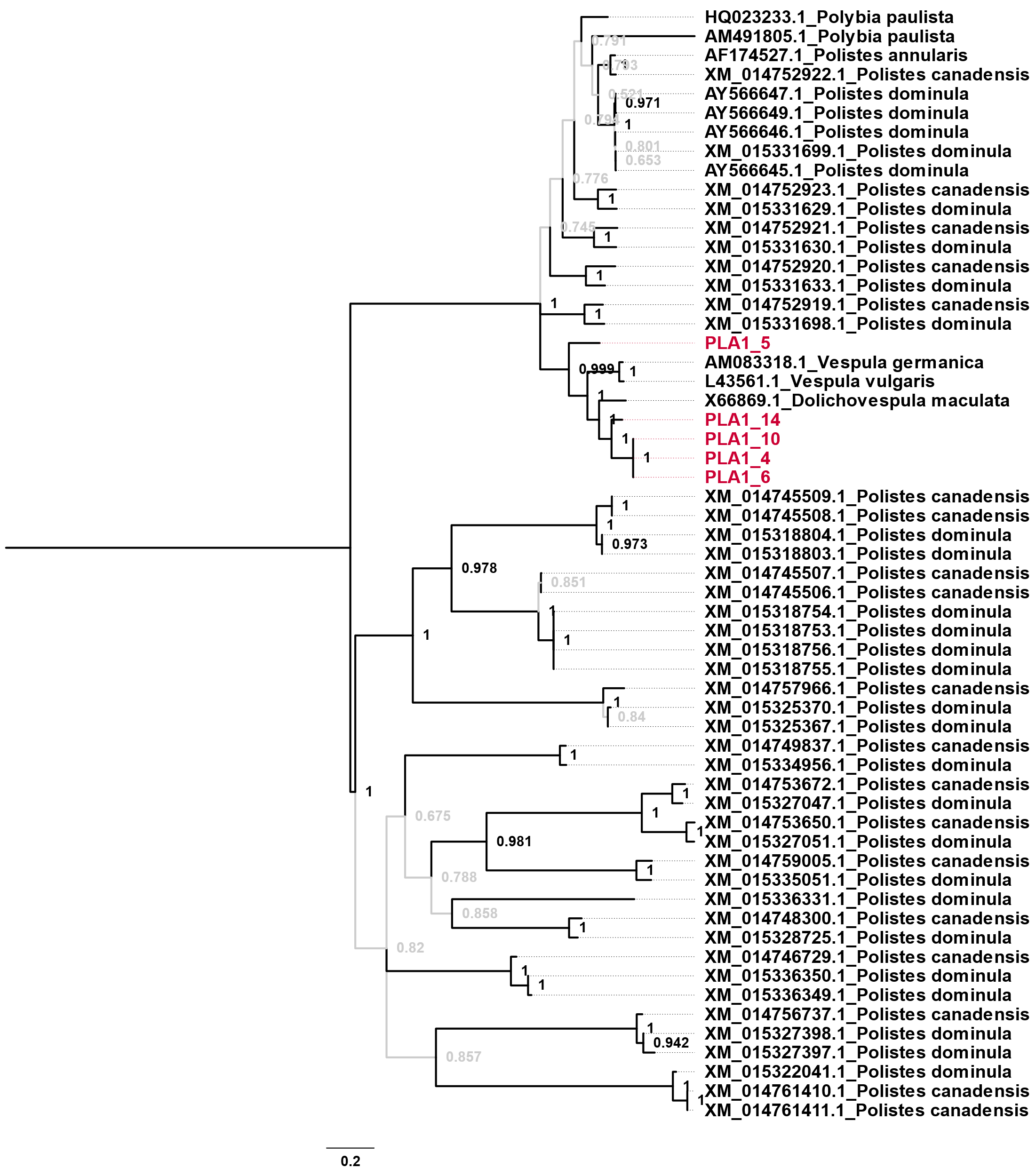
**

Phylogenetic relationships of the vespid PLA_1_ family are depicted here. The sequences generated in the current study are indicated in red colour. Thick black lines indicate well-supported branches (BPP ≥ 0.9).

**Figure S6.** Bayesian phylogeny for vespid serine protease family.

**
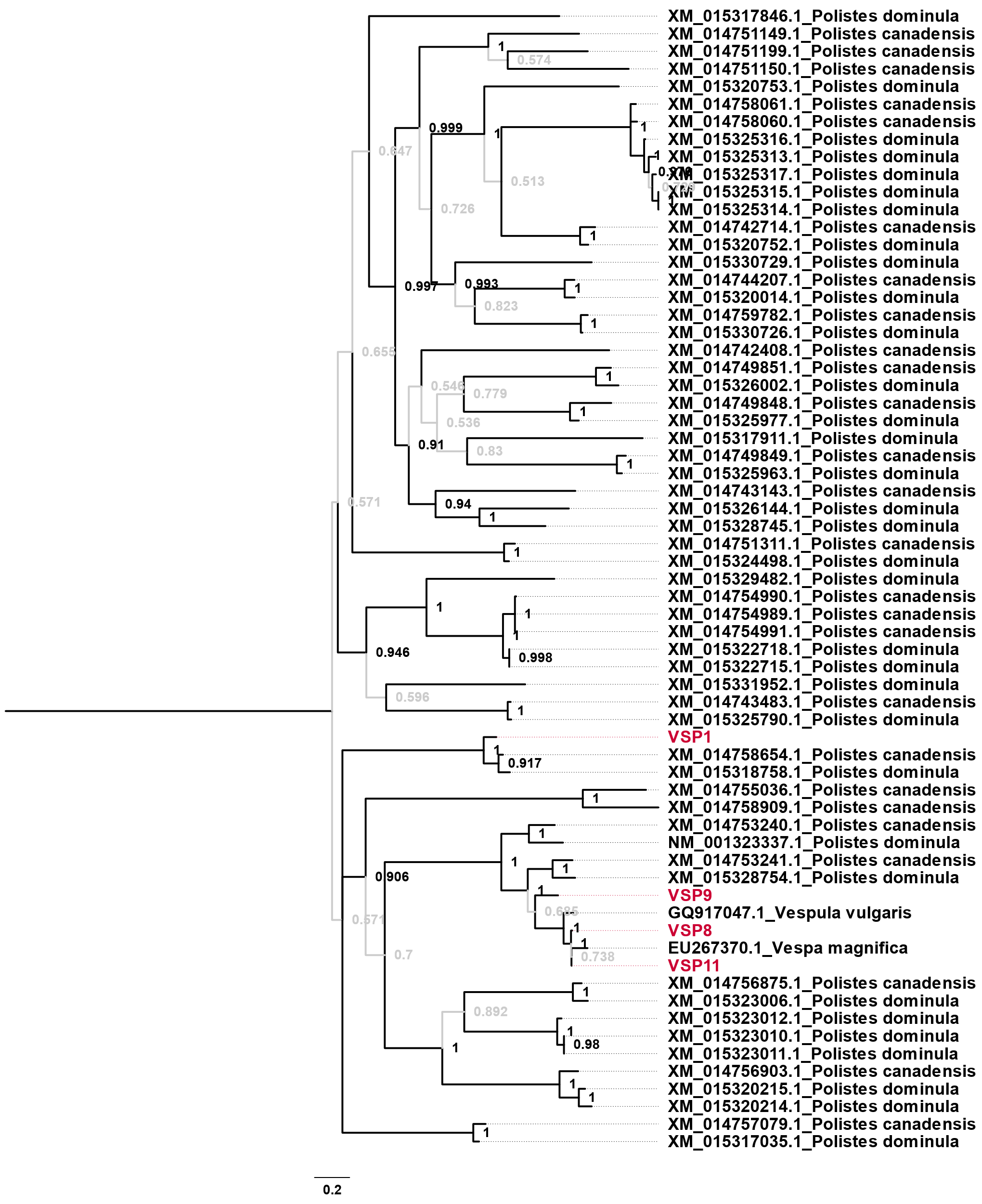
**

Phylogenetic relationships of the vespid serine protease family are depicted here. The sequences generated in the current study are indicated in red colour. Thick black lines indicate well-supported branches (BPP ≥ 0.9).

**Tables S1.** *V. affinis* venom gland and thorax tissue transcriptome sequencing and assembly statistics.

| **Reads** | | | | **Assembly** | |
| --- | --- | --- | --- | --- | --- |
| **Tissue** | **Raw** | **Filtered** | **Annotated** | **Transcripts** | **N50** |
| Venom gland | 22,630,858 | 21,478,981 | 91,188 | 73,947 | 2,274 |
| Thorax tissue | 23,163,263 | 22,144,524 |  |  |  |

**Tables S2.** Venom gland toxin transcripts from *V. affinis*.

| **Sr.no.** | **Isoform** | **Venom gland** | **Thorax tissue** | **M** | **Family** | **Abundance** | **Accession** | **Species** |
| --- | --- | --- | --- | --- | --- | --- | --- | --- |
| **5'-nucleotidase (5’-NTD): 0.63%** | | | | | | | | |
| 1 | TRINITY_DN4335_c0_g1_i14 | 2469.356 | 42.22 | 5.87 | 5'-NTD | 0.585704104 | XP_047349466.1 | *Vespa velutina* |
| 2 | TRINITY_DN5072_c0_g1_i1 | 206.686 | 59.605 | 1.794 | 5'-NTD | 0.049023648 | XP_046812737.1 | *Vespa crabro* |
| **Acetylcholesterase (AChE): 0.08%** | | | | | | | | |
| 3 | TRINITY_DN8037_c0_g5_i5 | 327.555 | 39.736 | 3.043 | AChE | 0.077692446 | XP_046819221.1 | *Vespa crabro* |
| **Acid phosphatase (ACP): 4.03%** | | | | | | | | |
| 4 | TRINITY_DN4822_c0_g3_i2 | 3180.066 | 610.948 | 2.38 | ACP | 0.754276705 | XP_046814492.1 | *Vespa crabro* |
| 5 | TRINITY_DN890_c0_g1_i4 | 2456.06 | 7.451 | 8.365 | ACP | 0.582550439 | XP_046837740.1 | *Vespa crabro* |
| 6 | TRINITY_DN2507_c0_g1_i28 | 2210.696 | 21.524 | 6.682 | ACP | 0.524352795 | XP_046837730.1 | *Vespa crabro* |
| 7 | TRINITY_DN890_c0_g1_i7 | 1665.576 | 0.5 | 11.702 | ACP | 0.395056322 | XP_046837740.1 | *Vespa crabro* |
| 8 | TRINITY_DN2507_c0_g1_i27 | 1582.176 | 34.769 | 5.508 | ACP | 0.375274758 | XP_046837730.1 | *Vespa crabro* |
| 9 | TRINITY_DN890_c0_g1_i8 | 1466.142 | 0.5 | 11.518 | ACP | 0.347752769 | XP_046837740.1 | *Vespa crabro* |
| 10 | TRINITY_DN4717_c0_g1_i1 | 832.788 | 76.989 | 3.435 | ACP | 0.197528161 | XP_035729511.1 | *Vespa mandarinia* |
| 11 | TRINITY_DN4822_c0_g3_i5 | 725.215 | 119.209 | 2.605 | ACP | 0.172013028 | XP_046814492.1 | *Vespa crabro* |
| 12 | TRINITY_DN890_c0_g1_i3 | 601.928 | 0.5 | 10.233 | ACP | 0.142770706 | XP_046837740.1 | *Vespa crabro* |
| 13 | TRINITY_DN2507_c0_g1_i14 | 589.841 | 0.5 | 10.204 | ACP | 0.139903803 | XP_046837730.1 | *Vespa crabro* |
| 14 | TRINITY_DN2507_c0_g1_i11 | 518.528 | 21.524 | 4.59 | ACP | 0.122989143 | XP_046837730.1 | *Vespa crabro* |
| 15 | TRINITY_DN890_c0_g1_i1 | 286.46 | 0.5 | 9.162 | ACP | 0.067945164 | XP_046837740.1 | *Vespa crabro* |
| 16 | TRINITY_DN4822_c0_g3_i1 | 262.286 | 26.491 | 3.308 | ACP | 0.062211357 | XP_046814492.1 | *Vespa crabro* |
| 17 | TRINITY_DN2507_c0_g1_i33 | 259.869 | 0.5 | 9.022 | ACP | 0.061638071 | XP_046837738.1 | *Vespa crabro* |
| 18 | TRINITY_DN2507_c0_g1_i20 | 252.616 | 23.18 | 3.446 | ACP | 0.059917739 | XP_046837730.1 | *Vespa crabro* |
| 19 | TRINITY_DN7993_c0_g1_i1 | 112.408 | 4.139 | 4.763 | ACP | 0.026661942 | XP_046837682.1 | *Vespa crabro* |
| **Aminopeptidase (AP): 5.84%** | | | | | | | | |
| 20 | TRINITY_DN5919_c0_g1_i4 | 6994.694 | 144.045 | 5.602 | AP | 1.659064541 | XP_035728915.1 | *Vespa mandarinia* |
| 21 | TRINITY_DN1113_c0_g1_i53 | 2660.329 | 115.898 | 4.521 | AP | 0.6310008 | XP_046828774.1 | *Vespa crabro* |
| 22 | TRINITY_DN3884_c0_g1_i1 | 1988.297 | 351.833 | 2.499 | AP | 0.471602196 | XP_046837634.1 | *Vespa crabro* |
| 23 | TRINITY_DN1894_c0_g4_i2 | 1715.132 | 287.262 | 2.578 | AP | 0.40681046 | XP_035728678.1 | *Vespa mandarinia* |
| 24 | TRINITY_DN1113_c0_g1_i73 | 1441.968 | 0.5 | 11.494 | AP | 0.342018962 | XP_046828774.1 | *Vespa crabro* |
| 25 | TRINITY_DN1113_c0_g1_i59 | 1374.282 | 84.44 | 4.025 | AP | 0.325964586 | XP_046828774.1 | *Vespa crabro* |
| 26 | TRINITY_DN1113_c0_g1_i77 | 1365.821 | 1.656 | 9.688 | AP | 0.32395773 | XP_047344597.1 | *Vespa velutina* |
| 27 | TRINITY_DN5919_c0_g1_i1 | 1185.726 | 164.741 | 2.848 | AP | 0.281241175 | XP_035728915.1 | *Vespa mandarinia* |
| 28 | TRINITY_DN1308_c0_g1_i15 | 972.996 | 233.452 | 2.059 | AP | 0.230783957 | XP_047361480.1 | *Vespa velutina* |
| 29 | TRINITY_DN2787_c0_g2_i22 | 701.041 | 19.04 | 5.202 | AP | 0.16627922 | XP_047356431.1 | *Vespa velutina* |
| 30 | TRINITY_DN12376_c0_g1_i1 | 647.858 | 0.5 | 10.34 | AP | 0.153664797 | XP_046828831.1 | *Vespa crabro* |
| 31 | TRINITY_DN11796_c0_g1_i1 | 622.476 | 115.07 | 2.436 | AP | 0.147644466 | XP_047357597.1 | *Vespa velutina* |
| 32 | TRINITY_DN5919_c0_g1_i3 | 606.763 | 0.5 | 10.245 | AP | 0.143917515 | XP_035728915.1 | *Vespa mandarinia* |
| 33 | TRINITY_DN18663_c0_g1_i18 | 433.92 | 66.227 | 2.712 | AP | 0.102921055 | XP_035722418.1 | *Vespa mandarinia* |
| 34 | TRINITY_DN18663_c0_g1_i24 | 398.868 | 0.5 | 9.64 | AP | 0.094607106 | XP_035722418.1 | *Vespa mandarinia* |
| 35 | TRINITY_DN1113_c0_g1_i6 | 385.572 | 0.5 | 9.591 | AP | 0.091453441 | XP_046828774.1 | *Vespa crabro* |
| 36 | TRINITY_DN8800_c0_g1_i1 | 346.894 | 42.22 | 3.038 | AP | 0.082279444 | XP_047347344.1 | *Vespa velutina* |
| 37 | TRINITY_DN1113_c0_g1_i47 | 285.251 | 0.5 | 9.156 | AP | 0.067658402 | XP_046828774.1 | *Vespa crabro* |
| 38 | TRINITY_DN1113_c0_g1_i95 | 190.973 | 56.293 | 1.762 | AP | 0.045296697 | XP_046828774.1 | *Vespa crabro* |
| 39 | TRINITY_DN1113_c0_g1_i12 | 174.051 | 0.5 | 8.443 | AP | 0.041282984 | XP_046828774.1 | *Vespa crabro* |
| 40 | TRINITY_DN1113_c0_g1_i67 | 140.208 | 0.5 | 8.131 | AP | 0.033255797 | XP_047344600.1 | *Vespa velutina* |
| **Apyrase (APY): 0.32%** | | | | | | | | |
| 41 | TRINITY_DN4335_c0_g1_i18 | 1232.865 | 96.858 | 3.67 | APY | 0.292422028 | XP_046814685.1 | *Vespa crabro* |
| 42 | TRINITY_DN4335_c0_g1_i24 | 118.452 | 10.762 | 3.46 | APY | 0.028095513 | XP_047349504.1 | *Vespa velutina* |
| **Arginine kinase (AK): 30.38%** | | | | | | | | |
| 43 | TRINITY_DN1950_c0_g1_i1 | 127477.008 | 1120.899 | 6.829 | ArgK | 30.23614526 | XP_035728187.1 | *Vespa mandarinia* |
| 44 | TRINITY_DN1950_c0_g1_i2 | 605.554 | 25.663 | 4.56 | ArgK | 0.143630753 | XP_035728187.1 | *Vespa mandarinia* |
| **Cysteine-rich secretory proteins, antigen 5, and pathogenesis-related 1 proteins (CAP): 2.88%** | | | | | | | | |
| 45 | TRINITY_DN6370_c0_g1_i1 | 10823.827 | 24.007 | 8.817 | CAP | 2.567292805 | XP_047350048.1 | *Vespa velutina* |
| 46 | TRINITY_DN7674_c0_g4_i1 | 1137.378 | 0.5 | 11.151 | CAP | 0.269773561 | XP_046819082.1 | *Vespa crabro* |
| 47 | TRINITY_DN7674_c0_g4_i2 | 192.182 | 1.656 | 6.859 | CAP | 0.045583458 | XP_046819082.1 | *Vespa crabro* |
| **Carboxylesterase (CES): 0.17%** | | | | | | | | |
| 48 | TRINITY_DN1983_c0_g1_i4 | 727.632 | 61.26 | 3.57 | CES | 0.172586313 | XP_047361470.1 | *Vespa velutina* |
| **Carboxypeptidase (CP): 2.32%** | | | | | | | | |
| 49 | TRINITY_DN36984_c0_g1_i1 | 3470.152 | 918.906 | 1.917 | CBP | 0.823081916 | XP_035733667.1 | *Vespa mandarinia* |
| 50 | TRINITY_DN2940_c0_g1_i2 | 1788.863 | 117.554 | 3.928 | CBP | 0.424298643 | XP_035743134.1 | *Vespa mandarinia* |
| 51 | TRINITY_DN2940_c0_g1_i5 | 1742.932 | 451.174 | 1.95 | CBP | 0.413404315 | XP_035743134.1 | *Vespa mandarinia* |
| 52 | TRINITY_DN2940_c0_g1_i5 | 1742.932 | 451.174 | 1.95 | CBP | 0.413404315 | XP_035743134.1 | *Vespa mandarinia* |
| 53 | TRINITY_DN3209_c0_g2_i5 | 482.268 | 131.627 | 1.873 | CBP | 0.114388669 | XP_035727941.1 | *Vespa mandarinia* |
| 54 | TRINITY_DN2369_c0_g1_i1 | 311.842 | 86.096 | 1.857 | CBP | 0.073965495 | XP_047354686.1 | *Vespa velutina* |
| 55 | TRINITY_DN1787_c0_g1_i12 | 128.121 | 36.425 | 1.815 | CBP | 0.030388893 | XP_047350840.1 | *Vespa velutina* |
| 56 | TRINITY_DN14820_c0_g2_i1 | 120.869 | 38.909 | 1.635 | CBP | 0.028668798 | XP_046827936.1 | *Vespa crabro* |
| **Chitinase (CHI): 4.10%** | | | | | | | | |
| 57 | TRINITY_DN167_c0_g1_i1 | 16621.918 | 5034.941 | 1.723 | CHI | 3.942536266 | XP_046817819.1 | *Vespa crabro* |
| 58 | TRINITY_DN11942_c0_g1_i5 | 379.529 | 92.718 | 2.033 | CHI | 0.090020108 | XP_046825380.1 | *Vespa crabro* |
| 59 | TRINITY_DN6350_c0_g1_i2 | 277.999 | 70.367 | 1.982 | CHI | 0.065938307 | XP_046838481.1 | *Vespa crabro* |
| **Chymotrypsin (CHY): 0.09%** | | | | | | | | |
| 60 | TRINITY_DN1042_c3_g1_i1 | 397.659 | 78.645 | 2.338 | CHY | 0.094320344 | XP_046826107.1 | *Vespa crabro* |
| **Cysteine-rich secretory proteins (CRISP): 1.73%** | | | | | | | | |
| 61 | TRINITY_DN3760_c1_g4_i1 | 7154.242 | 221.034 | 5.016 | CRISP | 1.696907574 | XP_035727042.1 | *Vespa mandarinia* |
| 62 | TRINITY_DN5841_c0_g1_i3 | 136.582 | 34.769 | 1.974 | CRISP | 0.032395749 | XP_035724773.1 | *Vespa mandarinia* |
| **C-type lectin (CTL): 0.24%** | | | | | | | | |
| 63 | TRINITY_DN1366_c0_g2_i2 | 1008.048 | 260.771 | 1.951 | CTL | 0.239097907 | XP_035731748.1 | *Vespa mandarinia* |
| **DNase: 0.06%** | | | | | | | | |
| 64 | TRINITY_DN2837_c1_g1_i2 | 146.252 | 24.007 | 2.607 | DNase | 0.034689367 | XP_047351595.1 | *Vespa velutina* |
| 65 | TRINITY_DN2837_c1_g1_i5 | 106.365 | 11.59 | 3.198 | DNase | 0.025228609 | XP_047351595.1 | *Vespa velutina* |
| **Dipeptidyl peptidases (DPP): 2.27%** | | | | | | | | |
| 66 | TRINITY_DN3450_c0_g1_i24 | 5265.058 | 10.762 | 8.934 | DPP | 1.248813892 | XP_047346320.1 | *Vespa velutina* |
| 67 | TRINITY_DN7582_c0_g1_i2 | 2002.801 | 287.262 | 2.802 | DPP | 0.475042385 | XP_047355273.1 | *Vespa velutina* |
| 68 | TRINITY_DN3450_c0_g1_i51 | 852.127 | 0.5 | 10.735 | DPP | 0.202115159 | XP_047346320.1 | *Vespa velutina* |
| 69 | TRINITY_DN3450_c0_g1_i26 | 362.607 | 116.726 | 1.635 | DPP | 0.086006395 | XP_047346320.1 | *Vespa velutina* |
| 70 | TRINITY_DN3450_c0_g1_i47 | 258.66 | 54.638 | 2.243 | DPP | 0.061351309 | XP_047346320.1 | *Vespa velutina* |
| 71 | TRINITY_DN3450_c0_g1_i44 | 247.782 | 10.762 | 4.525 | DPP | 0.058771167 | XP_047346320.1 | *Vespa velutina* |
| 72 | TRINITY_DN7582_c0_g1_i1 | 221.19 | 29.802 | 2.892 | DPP | 0.052463837 | XP_046826415.1 | *Vespa crabro* |
| 73 | TRINITY_DN1128_c0_g1_i5 | 184.93 | 0.5 | 8.531 | DPP | 0.043863364 | XP_047367711.1 | *Vespa velutina* |
| 74 | TRINITY_DN0_c7_g1_i1 | 160.756 | 12.418 | 3.694 | DPP | 0.038129556 | XP_047356031.1 | *Vespa velutina* |
| **Hyaluronidse (HYL): 7.63%** | | | | | | | | |
| 75 | TRINITY_DN2228_c0_g4_i1 | 27665.727 | 118.382 | 7.869 | HYL | 6.562006384 | XP_047355416.1 | *Vespa velutina* |
| 76 | TRINITY_DN2228_c0_g2_i1 | 2951.623 | 4.967 | 9.215 | HYL | 0.700092536 | XP_046814512.1 | *Vespa crabro* |
| 77 | TRINITY_DN2228_c0_g4_i2 | 743.345 | 0.5 | 10.538 | HYL | 0.176313264 | XP_035735901.1 | *Vespa mandarinia* |
| 78 | TRINITY_DN2228_c0_g2_i2 | 510.068 | 5.795 | 6.46 | HYL | 0.120982524 | XP_046814512.1 | *Vespa crabro* |
| 79 | TRINITY_DN2228_c0_g4_i3 | 314.26 | 9.106 | 5.109 | HYL | 0.074539018 | XP_035735901.1 | *Vespa mandarinia* |
| **Mastoparans (MPN): 0.61%** | | | | | | | | |
| 80 | TRINITY_DN9405_c0_g1_i2 | 2570.886 | 0.828 | 11.601 | MPN | 0.609785904 | ADN92459.1 | *Vespa affinis* |
| **Metallopreotease (MP): 0.04%** | | | | | | | | |
| 81 | TRINITY_DN7909_c0_g1_i6 | 154.712 | 45.531 | 1.765 | MP | 0.036695986 | XP_047351390.1 | *Vespa velutina* |
| **Metalloprotease inhibitors (MPi): 0.47%** | | | | | | | | |
| 82 | TRINITY_DN1596_c0_g3_i9 | 1759.854 | 172.191 | 3.353 | MPi | 0.417418027 | XP_035726401.1 | *Vespa mandarinia* |
| 83 | TRINITY_DN1596_c0_g3_i5 | 234.486 | 36.425 | 2.686 | MPi | 0.055617502 | XP_035726401.1 | *Vespa mandarinia* |
| **Neprilysin (NEP): 4.84%** | | | | | | | | |
| 84 | TRINITY_DN12426_c0_g2_i4 | 5927.42 | 23.18 | 7.998 | NEP | 1.405918879 | XP_035741306.1 | *Vespa mandarinia* |
| 85 | TRINITY_DN12426_c0_g4_i3 | 5927.42 | 641.578 | 3.208 | NEP | 1.405918879 | XP_047368889.1 | *Vespa velutina* |
| 86 | TRINITY_DN7196_c2_g1_i4 | 4962.885 | 1020.73 | 2.282 | NEP | 1.177141777 | XP_035721592.1 | *Vespa mandarinia* |
| 87 | TRINITY_DN12426_c0_g2_i3 | 1977.418 | 85.268 | 4.535 | NEP | 0.469021817 | XP_035741307.1 | *Vespa mandarinia* |
| 88 | TRINITY_DN12426_c0_g2_i2 | 1612.394 | 9.934 | 7.343 | NEP | 0.382442136 | XP_046836510.1 | *Vespa crabro* |
| **Peroxiredoxin (PER): 2.05%** | | | | | | | | |
| 89 | TRINITY_DN234_c0_g1_i2 | 8657.853 | 1274.05 | 2.765 | PRDX | 2.053547577 | XP_046814161.1 | *Vespa crabro* |
| **Phospholipase A_1_ (PLA_1_): 25.67%** | | | | | | | | |
| 90 | TRINITY_DN2326_c0_g1_i5 | 93812.548 | 8.278 | 13.468 | PLA_1_ | 22.25130534 | P0DMB5.1 | *Vespa affinis* |
| 91 | TRINITY_DN2326_c0_g1_i13 | 6686.478 | 0.5 | 13.707 | PLA_1_ | 1.585959094 | XP_035732040.1 | *Vespa mandarinia* |
| 92 | TRINITY_DN2326_c0_g1_i4 | 2553.964 | 0.5 | 12.319 | PLA_1_ | 0.605772191 | XP_047357816.1 | *Vespa velutina* |
| 93 | TRINITY_DN2326_c0_g1_i15 | 2153.887 | 0.5 | 12.073 | PLA_1_ | 0.510878324 | XP_047357816.1 | *Vespa velutina* |
| 94 | TRINITY_DN2326_c0_g1_i6 | 1535.037 | 0.5 | 11.584 | PLA_1_ | 0.364093906 | P0DMB4.1 | *Vespa affinis* |
| 95 | TRINITY_DN1436_c0_g1_i9 | 264.703 | 0.5 | 9.048 | PLA_1_ | 0.062784642 | XP_035723994.1 | *Vespa mandarinia* |
| 96 | TRINITY_DN1436_c0_g1_i17 | 178.886 | 24.835 | 2.849 | PLA_1_ | 0.042429793 | XP_035723994.1 | *Vespa mandarinia* |
| 97 | TRINITY_DN1436_c0_g1_i18 | 403.703 | 68.711 | 2.555 | PLA_1_ | 0.095753915 | XP_035723994.1 | *Vespa mandarinia* |
| 98 | TRINITY_DN2326_c0_g1_i14 | 262.286 | 0.5 | 9.035 | PLA_1_ | 0.062211357 | P0DMB4.1 | *Vespa affinis* |
| 99 | TRINITY_DN594_c1_g4_i1 | 239.321 | 0.5 | 8.903 | PLA_1_ | 0.056764311 | XP_046816619.1 | *Vespa crabro* |
| 100 | TRINITY_DN2326_c0_g1_i2 | 137.791 | 0.5 | 8.106 | PLA_1_ | 0.032682511 | XP_035732040.1 | *Vespa mandarinia* |
| **Phospholipase A_2_ (PLA_2_): 0.08%** | | | | | | | | |
| 101 | TRINITY_DN838_c0_g3_i1 | 346.894 | 86.096 | 2.01 | PLA_2_ | 0.082279444 | XP_035728062.1 | Vespa mandarinia |
| **Phospholipase A_2_ inhibitor (PLA_2_i): 0.18%** | | | | | | | | |
| 102 | TRINITY_DN1444_c0_g4_i4 | 662.363 | 5.795 | 6.837 | PLA_2_i | 0.157105224 | XP_046816074.1 | Vespa crabro |
| 103 | TRINITY_DN1444_c0_g4_i5 | 102.739 | 15.729 | 2.707 | PLA_2_i | 0.024368562 | XP_046816072.1 | Vespa crabro |
| **Phospholipase B (PLB): 1.93%** | | | | | | | | |
| 104 | TRINITY_DN303_c0_g3_i16 | 1553.168 | 212.756 | 2.868 | PLB | 0.368394379 | XP_046834493.1 | *Vespa crabro* |
| 105 | TRINITY_DN303_c0_g2_i1 | 436.337 | 65.4 | 2.738 | PLB | 0.103494341 | XP_046834493.1 | *Vespa crabro* |
| 106 | TRINITY_DN303_c0_g3_i6 | 403.703 | 24.007 | 4.072 | PLB | 0.095753915 | XP_012275336.1 | *Orussus abietinus* |
| 107 | TRINITY_DN303_c0_g2_i10 | 5735.239 | 1814.632 | 1.66 | PLB | 1.360335658 | XP_046834493.1 | *Vespa crabro* |
| **Scoloptoxin (SLPTX): 0.08%** | | | | | | | | |
| 108 | TRINITY_DN4607_c0_g1_i2 | 207.895 | 21.524 | 3.272 | SLPTX | 0.049310409 | XP_035737389.1 | *Vespa mandarinia* |
| 109 | TRINITY_DN4607_c0_g1_i13 | 117.243 | 2.484 | 5.561 | SLPTX | 0.027808751 | XP_035737389.1 | *Vespa mandarinia* |
| **Serine protease (SP): 1.17%** | | | | | | | | |
| 110 | TRINITY_DN1494_c0_g1_i6 | 343.268 | 0.5 | 9.423 | SP | 0.081419397 | XP_046827166.1 | *Vespa crabro* |
| 111 | TRINITY_DN605_c3_g1_i3 | 2451.225 | 206.133 | 3.572 | SP | 0.58140363 | XP_035729961.1 | *Vespa mandarinia* |
| 112 | TRINITY_DN36581_c0_g1_i1 | 460.511 | 9.934 | 5.535 | SP | 0.109228148 | XP_046828959.1 | *Vespa crabro* |
| 113 | TRINITY_DN1494_c0_g1_i8 | 380.738 | 26.491 | 3.845 | SP | 0.090306869 | XP_047367862.1 | *Vespa velutina* |
| 114 | TRINITY_DN15759_c0_g1_i14 | 337.225 | 91.063 | 1.889 | SP | 0.079986064 | XP_046835710.1 | *Vespa crabro* |
| 115 | TRINITY_DN15759_c0_g1_i8 | 285.251 | 62.088 | 2.2 | SP | 0.067658402 | XP_046835710.1 | *Vespa crabro* |
| 116 | TRINITY_DN453_c2_g1_i10 | 154.712 | 48.015 | 1.688 | SP | 0.036695986 | XP_035727445.1 | *Vespa mandarinia* |
| 117 | TRINITY_DN605_c3_g1_i2 | 152.295 | 14.901 | 3.353 | SP | 0.0361227 | XP_035729961.1 | *Vespa mandarinia* |
| 118 | TRINITY_DN1382_c2_g7_i2 | 128.121 | 30.63 | 2.064 | SP | 0.030388893 | XP_047371433.1 | *Vespa velutina* |
| 119 | TRINITY_DN5879_c0_g1_i2 | 113.617 | 31.458 | 1.853 | SP | 0.026948704 | XP_047343675.1 | *Vespa velutina* |
| 120 | TRINITY_DN5879_c0_g1_i2 | 113.617 | 31.458 | 1.853 | SP | 0.026948704 | XP_047343675.1 | *Vespa velutina* |
| **Trypsin (TRY): 0.03%** | | | | | | | | |
| 121 | TRINITY_DN33420_c0_g1_i1 | 151.086 | 1.656 | 6.512 | Trypsin | 0.035823101 | XP_047346991.1 | *Vespa velutina* |
| 122 | TRINITY_DN7688_c0_g2_i1 | 13.296 | 6.623 | 1.005 | Trypsin | 0.000030834 | XP_046828083.1 | *Vespa crabro* |
| **Vascular endothelial growth factor (VEGF): 0.09%** | | | | | | | | |
| 123 | TRINITY_DN710_c1_g1_i9 | 392.825 | 70.367 | 2.481 | VEGF | 0.093173773 | XP_035733748.1 | *Vespa mandarinia* |
| **Other interesting hits** | | | | | | | | |
| TRINITY_DN1342_c0_g1_i4 | | 788.066 | 201.994 | 1.964 | Maltase | | XP_046826177.1 | *Vespa crabro* |
| TRINITY_DN1342_c0_g1_i34 | | 236.903 | 30.63 | 2.951 | Maltase | | XP_046826177.1 | *Vespa crabro* |
| TRINITY_DN10047_c0_g1_i1 | | 142.626 | 0.5 | 8.156 | Maltase | | XP_047343302.1 | *Vespa velutina* |
| TRINITY_DN11986_c0_g1_i3 | | 391.616 | 52.154 | 2.909 | Galactosidase | | XP_047360063.1 | *Vespa velutina* |
| TRINITY_DN1709_c0_g1_i5 | | 1353.734 | 221.034 | 2.615 | Glucosidase | | XP_046827524.1 | *Vespa crabro* |
| TRINITY_DN10256_c0_g5_i4 | | 460.511 | 0.5 | 9.847 | Trehalase | | XP_046821105.1 | *Vespa crabro* |

The table summarises BLAST annotation for the *Vespa affinis* venom gland and thorax assembly, generated by searching against the NCBI-NR database. The key results of these searches, including the accession number, species name and family of the matching entry from the database are shown here. The expression values of each transcript in venom gland and thorax tissue have also been presented. The percentage indicated adjacent to the toxin family corresponds to its proportion in the *V. affinis* venom gland transcriptome. The log2fold change in expression value (M) has also been reported.

**Tables S3.** Proteomic composition of the *V. affinis* venom.

| **Sr.no.** | **Accession/Transcript ID** | **Species** | **-10lgP** | **#Peptides** | | **#Unique** | **Relative abundance**  **of toxin hit (%)** | | **Avg. mass (KDa)** | | **Toxin**  **family** | | **Band number** | |
| --- | --- | --- | --- | --- | --- | --- | --- | --- | --- | --- | --- | --- | --- | --- |
| **Cysteine-rich secretory proteins, antigen 5, and pathogenesis-related 1 proteins (CAP): 26.09%** | | | | | | | | | | | | | | |
| 1 | TRINITY_DN6370_c0_g1_i1.p1 | *Vespa affinis* | 337.99 | 17 | | 15 | 22.74754482 | | 25.005 | | CAP | | 3-7 | |
| 2 | P35782 | *Vespa crabro* | 165.93 | 5 | | 3 | 3.187453387 | | 22.781 | | CAP | | 4, 6, 7 | |
| 3 | P0DMB9 | *Vespa velutina* | 120.77 | 3 | | 1 | 0.002050016 | | 22.718 | | CAP | | 7 | |
| **Phospholipase A_1_ (PLA_1_): 21.86%** | | | | | | | | | | | | | | |
| 4 | TRINITY_DN2326_c0_g1_i13.p1 | *Vespa affinis* | 350.79 | 16 | | 14 | 18.89213935 | | 32.507 | | PLA_1_ | | 1-7 | |
| 5 | TRINITY_DN2326_c0_g1_i12.p1 | *Vespa affinis* | 365.86 | 24 | | 5 | 1.702905022 | | 37.536 | | PLA_1_ | | 2-4, 6, 7 | |
| 6 | TRINITY_DN594_c1_g4_i5.p1 | *Vespa affinis* | 170.39 | 3 | | 3 | 0.150551385 | | 23.117 | | PLA_1_ | | 4, 5 | |
| 7 | C0HLL3 | *Vespa velutina* | 301.21 | 11 | | 1 | 0.058626448 | | 33.957 | | PLA_1_ | | 5, 7 | |
| 8 | A0A0M3KKW3 | *Vespa basalis* | 348.32 | 20 | | 1 | 0.007130593 | | 33.185 | | PLA_1_ | | 7 | |
| **Trypsin (TRY): 18.95%** | | | | | | | | | | | | | | |
| 9 | TRINITY_DN33420_c0_g1_i1.p1 | *Vespa affinis* | 326.68 | 14 | | 14 | 17.7294894 | | 29.157 | | Trypsin | | 1-7 | |
| 10 | TRINITY_DN7688_c0_g2_i1.p1 | *Vespa affinis* | 122.82 | 2 | | 2 | 0.033378975 | | 27.76 | | Trypsin | | 5-7 | |
| **Hyaluronidase (HYL): 15.2%** | | | | | | | | | | | | | | |
| 11 | TRINITY_DN2228_c0_g4_i1.p1 | *Vespa affinis* | 246.57 | 15 | | 14 | 12.75385804 | | 42.077 | | Hyaluronidase | | 1, 3, 4, 6, 7 | |
| 12 | TRINITY_DN2228_c0_g2_i2.p1 | *Vespa affinis* | 165.68 | 6 | | 6 | 1.510655451 | | 36.957 | | Hyaluronidase | | 3-7 | |
| 13 | Q5D7H4 | *Vespula vulgaris* | 114.81 | 3 | | 2 | 0.445387877 | | 40.072 | | Hyaluronidase | | 3- 7 | |
| **Dipeptidyl peptidase (DPP): 6.87%** | | | | | | | | | | | | | | |
| 14 | TRINITY_DN3450_c0_g1_i51.p1 | *Vespa affinis* | 397.97 | 33 | | 33 | 6.556303376 | | 79.62 | | DPP | | 2-7 | |
| **L-amino acid oxidase (LAAO): 6.79%** | | | | | | | | | | | | | | |
| 15 | G8XQX1 | *Daboia russelii* | 93.11 | 1 | 1 | | | 0.939163069 | | 56.888 | | LAAO | | 1- 7 |
| 16 | B5U6Y8 | *Echis ocellatus* | 104.2 | 1 | 1 | | | 0.014380895 | | 56.523 | | LAAO | | 1, 3 |
| **Serine protease (SP): 1.91%** | | | | | | | | | | | | | | |
| 17 | TRINITY_DN1494_c0_g1_i8.p1 | *Vespa affinis* | 220.12 | 5 | | 5 | 1.840607185 | | 46.901 | | Protease | | 3-5, 7 | |
| **Aminopeptidase (AP): 0.93%** | | | | | | | | | | | | | | |
| 18 | TRINITY_DN1113_c0_g1_i95.p1 | *Vespa affinis* | 250.96 | 11 | | 11 | 0.899356413 | | 107.782 | | Aminopeptidase | | 3-7 | |
| 19 | TRINITY_DN11796_c0_g1_i1.p1 | *Vespa affinis* | 92.4 | 1 | | 1 | 0.004814972 | | 113.195 | | Aminopeptidase | | 6 | |
| **Carboxypeptidase (CP): 0.58%** | | | | | | | | | | | | | | |
| 20 | TRINITY_DN33395_c0_g1_i1.p1 | *Vespa affinis* | 162.14 | 5 | 5 | | | 0.269108188 | | 14.62 | | Carboxypeptidase | | 5-7 |
| 21 | TRINITY_DN3209_c0_g2_i3.p1 | *Vespa affinis* | 63.54 | 1 | 1 | | | 0.003035846 | | 53.297 | | Carboxypeptidase | | 2 |
| **Phospholipase A_2_ inhibitor (PLA_2_i): 0.45%** | | | | | | | | | | | | | | |
| 22 | TRINITY_DN2938_c0_g1_i1.p1 | *Vespa affinis* | 159.6 | 4 | | 4 | 0.436645426 | | 31.741 | | PLA_2_-inhibitor | | 3-7 | |
| **Phospholipase A_2_ (PLA_2_): 0.17%** | | | | | | | | | | | | | | |
| 23 | TRINITY_DN838_c0_g3_i1.p1 | *Vespa affinis* | 128.43 | 2 | | 2 | 0.162462477 | | 25.927 | | PLA_2_ | | 5-7 | |
| **Chitinase (CHI): 0.11%** | | | | | | | | | | | | | | |
| 24 | TRINITY_DN36831_c0_g1_i1.p1 | *Vespa affinis* | 180.75 | 6 | | 6 | 0.101610522 | | 21.424 | | Chitinase | | 3-6 | |
| **Chymotrypsin (CHY): 0.08%** | | | | | | | | | | | | | | |
| 25 | P00768 | *Vespa orientalis* | 112.16 | 3 | | 3 | 0.083184236 | | 23.471 | | Chymotrypsin | | 6, 7 | |
| **Peroxiredoxin (PER): 0.02%** | | | | | | | | | | | | | | |
| 26 | TRINITY_DN1450_c3_g2_i1.p1 | *Vespa affinis* | 74.23 | 2 | | 2 | 0.003484796 | | 21.512 | | Peroxiredoxin | | 1, 6 | |
| **Other interesting hits** | | | | | | | | | | | | | | |
| TRINITY_DN10047_c0_g1_i1.p1 | | *Vespa velutina* | 329.91 | 16 | | 16 |  | | 64.848 | | Maltase | | 1-6 | |
| TRINITY_DN1342_c0_g1_i23.p1 | | *Vespa crabro* | 246.03 | 13 | | 5 |  | | 68.697 | | Maltase | | 1, 2, 4, 5 | |
| TRINITY_DN1342_c0_g1_i29.p1 | | *Vespa velutina* | 248.4 | 12 | | 3 |  | | 68.886 | | Maltase | | 1-5 | |
| TRINITY_DN23073_c0_g1_i1.p1 | | *Vespa crabro* | 98.32 | 1 | | 1 |  | | 24.34 | | Maltase | | 2, 4, 5 | |
| TRINITY_DN1342_c0_g1_i9.p1 | | *Vespa crabro* | 234.4 | 10 | | 1 |  | | 52.685 | | Maltase | | 1, 2 | |
| TRINITY_DN9761_c0_g1_i1.p1 | | *Vespa crabro* | 171.34 | 4 | | 1 |  | | 18.606 | | Maltase | | 2 | |
| TRINITY_DN1342_c0_g1_i34.p1 | | *Vespa crabro* | 200.62 | 9 | | 1 |  | | 67.118 | | Maltase | | 6 | |
| TRINITY_DN11986_c0_g1_i3.p1 | | *Vespa velutina* | 309.84 | 13 | | 13 |  | | 72.596 | | Galactosidase | | 1-4, 6, 7 | |
| TRINITY_DN1709_c0_g1_i4.p1 | | *Vespa crabro* | 228.01 | 9 | | 9 |  | | 107.097 | | Glucosidase | | 1, 2 | |
| TRINITY_DN10256_c0_g10_i1.p1 | | *Vespa crabro* | 254.07 | 11 | | 11 |  | | 67.221 | | Trehalase | | 1-6 | |

Peaks Studio X was used to search raw MS/MS spectra against the NCBI-NR database combined with the tissue transcriptome data for the identification of toxins. The key statistics of these searches, including the accession number, species name, -10lgP values, number of high confidence peptides, unique peptides, percent abundance of each toxin hit, average molecular mass (KDa), the toxin family of the matching entry and the number of SDS-PAGE gel bands in which the toxin was identified, have been shown. The percentage indicated adjacent to the toxin family corresponds to its proportion in the venom of *V. affinis* venom as determined by tandem mass spectrometry.

**Table S4. Insect-specific toxicity of *V. affinis* venom**

| **Venom**  **(μg)** | **Observations** |  | **Venom**  **(μg)** | **Observations** |  |
| --- | --- | --- | --- | --- | --- |
|  |  |  |  |  |  |
| **Control 0 μg** | Both individuals were alive for 24 hours |  | **9 μg** | Both were alive for one hour post-injection |  |
| **3 μg** | Both individuals were alive for 24 hours |  | **12 μg** | Both died within 14 minutes of injection |  |
| **6 μg** | Both individuals were alive for 24 hours |  | **15 μg** | Both died immediately after injection |  |

The table depicts lethal toxicity range of *V. affinis* venom on *A. domesticus* (2/dose group).
