# Supplementary material for: Stings on wings: Proteotranscriptomic and biochemical profiling of the lesser banded hornet (Vespa affinis) venom
Source: Front Mol Biosci. 2022 Dec 19;9:1066793. doi: 10.3389/fmolb.2022.1066793 (PMC9806352; doi:10.3389/fmolb.2022.1066793)
Supplement: Supplementary file 3 [file DataSheet2.ZIP › HTML/peptides.html]

peptide list


|  |
| --- |
| Peptide Sequence Contains: |
| Peptide Sample Area >= |
| Peptide PTM Contains: |
| Show Peptide From: |
| With Protein Accession: False |

Peptide List

  

| Peptide | -10lgP | Mass | Length | ppm | m/z | RT | Fraction | Scan | Source File | Area F1 | Area F2 | Area F3 | Area F4 | Area F5 | Area F6 | Area F7 | #Feature | #Feature F1 | #Feature F2 | #Feature F3 | #Feature F4 | #Feature F5 | #Feature F6 | #Feature F7 | Accession | PTM | AScore | Found By |
| --- | --- | --- | --- | --- | --- | --- | --- | --- | --- | --- | --- | --- | --- | --- | --- | --- | --- | --- | --- | --- | --- | --- | --- | --- | --- | --- | --- | --- |
| SIVHESYPGGVAPYDIALVELQTPLSLSK | 133.98 | 3082.6226 | 29 | 1.2 | 1028.5493 | 82.39 | 5 | F5:26950 | Fraction\_5\_23092022.raw |  |  | 9.6481E7 | 4.2493E6 | 5.2887E6 | 1.4567E7 |  | 10 | 0 | 0 | 7 | 1 | 1 | 1 | 0 | TRINITY\_DN33420\_c0\_g1\_i1.p1 |  |  | PEAKS DB |
| NVLHTQSSDILRGPTLIDVNC(+57.02)GTDNVC(+57.02)LKNEDVIHTVIR | 132.10 | 4415.2324 | 39 | 4.1 | 884.0574 | 64.10 | 1 | F1:17835 | Fraction\_1\_23092022r.raw | 7.1965E7 | 3.7026E6 |  |  |  |  |  | 5 | 3 | 2 | 0 | 0 | 0 | 0 | 0 | TRINITY\_DN4276\_c0\_g1\_i1.p1 | Carbamidomethylation | C21:Carbamidomethylation:1000.00;C27:Carbamidomethylation:1000.00 | PEAKS DB |
| YSEIIGLDPAGPSFKSQEC(+57.02)SQR | 129.67 | 2468.1641 | 22 | 1.1 | 823.7296 | 54.77 | 5 | F5:14505 | Fraction\_5\_23092022.raw | 3.6189E5 | 4.2722E5 | 1.3853E7 | 1.6435E7 | 1.2898E7 | 8.3058E6 | 6.9584E6 | 11 | 1 | 1 | 2 | 2 | 2 | 2 | 1 |  | Carbamidomethylation | C19:Carbamidomethylation:1000.00 | PEAKS DB |
| AIDHSHGIIYYLATGPGKPSQR | 129.48 | 2380.2288 | 22 | 0.6 | 596.0648 | 45.92 | 7 | F7:13419 | Fraction\_7\_23092022.raw |  | 1.9371E5 | 1.3801E5 | 2.4405E6 | 3.3191E6 | 5.5043E6 | 2.4626E7 | 15 | 0 | 1 | 1 | 3 | 3 | 3 | 4 | TRINITY\_DN3450\_c0\_g1\_i51.p1 |  |  | PEAKS DB |
| SVVKASGLTNAEKLEILKQHNEFR | 128.24 | 2710.4766 | 24 | -0.5 | 678.6261 | 41.71 | 7 | F7:11488 | Fraction\_7\_23092022.raw |  |  |  | 1.8012E6 | 1.4014E5 | 9.5343E5 | 1.2374E8 | 7 | 0 | 0 | 0 | 1 | 1 | 1 | 4 | TRINITY\_DN6370\_c0\_g1\_i1.p1 |  |  | PEAKS DB |
| GGGGSFGYSYGGGSGGGFSASSLGGGFGGGSR | 127.32 | 2704.1538 | 32 | 0.8 | 1353.0852 | 64.22 | 2 | F2:18090 | Fraction\_2\_23092022.raw | 1.0449E7 | 1.2773E7 | 4.5417E6 | 1.5477E6 | 1.7544E6 | 6.6E6 | 3.9126E6 | 14 | 2 | 2 | 2 | 2 | 2 | 2 | 2 | P35527|K1C9\_HUMAN |  |  | PEAKS DB |
| GSSGGGC(+57.02)FGGSSGGYGGLGGFGGGSFR | 126.87 | 2341.9771 | 27 | -1.2 | 1171.9944 | 61.28 | 5 | F5:17467 | Fraction\_5\_23092022.raw | 1.5792E7 | 3.8379E6 | 5.2059E6 | 1.1871E6 | 1.5145E6 | 4.031E6 | 1.6271E6 | 11 | 2 | 2 | 2 | 1 | 1 | 2 | 1 | P13645|K1C10\_HUMAN | Carbamidomethylation | C7:Carbamidomethylation:1000.00 | PEAKS DB |
| GPTLIDVNC(+57.02)GTDNVC(+57.02)LKNEDVIHTVIR | 123.58 | 3051.5117 | 27 | 0.2 | 1018.1780 | 62.49 | 1 | F1:17111 | Fraction\_1\_23092022r.raw | 1.6056E8 | 2.1669E6 |  |  |  |  |  | 4 | 2 | 2 | 0 | 0 | 0 | 0 | 0 | TRINITY\_DN4276\_c0\_g1\_i1.p1 | Carbamidomethylation | C9:Carbamidomethylation:1000.00;C15:Carbamidomethylation:1000.00 | PEAKS DB |
| LGKYSEIIGLDPAGPSFK | 121.87 | 1891.0090 | 18 | -0.1 | 946.5117 | 61.28 | 5 | F5:17430 | Fraction\_5\_23092022.raw | 9.4496E6 | 1.2017E7 | 3.3429E8 | 6.4717E8 | 4.0696E8 | 1.9234E8 | 2.1516E8 | 21 | 2 | 2 | 4 | 5 | 3 | 2 | 3 |  |  |  | PEAKS DB |
| TLGTVDFYM(+15.99)NNGYNQPGC(+57.02)SLIDGDSC(+57.02)SHAR | 121.69 | 3364.4182 | 30 | 1.9 | 1122.4822 | 59.54 | 7 | F7:21274 | Fraction\_7\_23092022.raw |  |  |  |  |  |  | 1.4735E6 | 1 | 0 | 0 | 0 | 0 | 0 | 0 | 1 | TRINITY\_DN2326\_c0\_g1\_i13.p1 | Oxidation (M); Carbamidomethylation | M9:Oxidation (M):1000.00;C18:Carbamidomethylation:1000.00;C26:Carbamidomethylation:1000.00 | PEAKS DB |
| LGEDNINVVEGNEQFISASK | 121.50 | 2162.0491 | 20 | 1.0 | 721.6910 | 59.80 | 5 | F5:16776 | Fraction\_5\_23092022.raw | 2.2434E10 | 1.6266E10 | 6.5593E9 | 1.4506E10 | 1.5645E10 | 1.6786E10 | 1.0288E10 | 124 | 15 | 13 | 10 | 21 | 21 | 14 | 30 | P00760|TRY1\_BOVIN |  |  | PEAKS DB |
| LGKYSEIIGLDPAGPSFKSQEC(+57.02)SQR | 120.46 | 2766.3645 | 25 | -0.1 | 923.1287 | 52.73 | 5 | F5:13586 | Fraction\_5\_23092022.raw | 1.27E6 | 2.4057E6 | 5.4356E7 | 9.3879E7 | 7.8325E7 | 3.292E7 | 5.5631E7 | 16 | 2 | 2 | 2 | 3 | 3 | 2 | 2 |  | Carbamidomethylation | C22:Carbamidomethylation:1000.00 | PEAKS DB |
| LIGHSLGAHTSGFAGK | 119.60 | 1551.8157 | 16 | 0.5 | 776.9155 | 28.03 | 7 | F7:5409 | Fraction\_7\_23092022.raw |  |  | 2.1628E6 | 1.6313E7 | 5.047E6 | 3.3174E7 | 1.2635E8 | 13 | 0 | 0 | 2 | 3 | 3 | 2 | 3 | A0A0M3KKW3|PA1\_VESBA |  |  | PEAKS DB |
| GGGFGGGSSFGGGSGFSGGGFGGGGFGGGR | 118.87 | 2398.0110 | 30 | -0.1 | 1200.0127 | 63.88 | 7 | F7:23843 | Fraction\_7\_23092022.raw | 5.284E6 | 2.7455E6 | 5.8629E6 | 1.3454E6 | 1.7026E6 | 5.2095E6 | 3.742E6 | 13 | 1 | 2 | 2 | 2 | 2 | 2 | 2 | P35908|K22E\_HUMAN |  |  | PEAKS DB |
| SGGGGGRFSSC(+57.02)GGGGGSFGAGGGFGSR | 118.80 | 2292.9678 | 27 | 0.6 | 765.3303 | 36.42 | 7 | F7:9109 | Fraction\_7\_23092022.raw | 5.0934E5 | 2.8449E6 |  | 1.2764E5 | 1.0065E5 | 1.5029E5 | 5.6802E5 | 6 | 1 | 1 | 0 | 1 | 1 | 1 | 1 | P04264|K2C1\_HUMAN | Carbamidomethylation | C11:Carbamidomethylation:1000.00 | PEAKS DB |
| YSEIIGLDPAGPLFKSQEC(+57.02)SQR | 118.28 | 2494.2161 | 22 | 1.3 | 832.4137 | 66.74 | 5 | F5:19894 | Fraction\_5\_23092022.raw |  |  | 6.7672E5 | 1.4969E6 | 1.1853E6 | 4.4708E6 | 5.0007E6 | 5 | 0 | 0 | 1 | 1 | 1 | 1 | 1 | TRINITY\_DN2326\_c0\_g1\_i13.p1 | Carbamidomethylation | C19:Carbamidomethylation:1000.00 | PEAKS DB |
| SIVHPSYNSNTLNNDIMLIK | 117.24 | 2272.1521 | 20 | 0.2 | 1137.0835 | 60.06 | 5 | F5:16905 | Fraction\_5\_23092022.raw | 7.7815E9 | 8.7199E7 | 2.1298E7 | 2.2202E8 | 6.0417E7 | 9.6987E7 | 2.0008E7 | 25 | 12 | 2 | 2 | 4 | 2 | 2 | 1 | P00760|TRY1\_BOVIN |  |  | PEAKS DB |
| VALQC(+57.02)PPGLHFNPNLQVC(+57.02)DLPEQANC(+57.02)K | 116.65 | 3118.4788 | 27 | 1.7 | 1040.5020 | 65.21 | 4 | F4:19436 | Fraction\_4\_23092022.raw |  |  | 3.4165E6 | 2.4828E7 | 1.9413E6 | 3.772E5 |  | 6 | 0 | 0 | 1 | 3 | 1 | 1 | 0 | TRINITY\_DN2925\_c0\_g1\_i5.p1 | Carbamidomethylation | C5:Carbamidomethylation:1000.00;C18:Carbamidomethylation:1000.00;C26:Carbamidomethylation:1000.00 | PEAKS DB |
| GGSGGSHGGGSGFGGESGGSYGGGEEASGSGGGYGGGSGK | 116.25 | 3222.2742 | 40 | 0.4 | 1075.0991 | 26.95 | 7 | F7:4975 | Fraction\_7\_23092022.raw |  |  |  |  | 2.2816E5 |  | 5.2951E5 | 2 | 0 | 0 | 0 | 0 | 1 | 0 | 1 | P35527|K1C9\_HUMAN |  |  | PEAKS DB |
| YFTEC(+57.02)IRHEC(+57.02)C(+57.02)LIGVPQSK | 115.76 | 2396.1074 | 19 | -0.2 | 799.7096 | 48.14 | 5 | F5:11534 | Fraction\_5\_23092022.raw | 4.8811E5 | 2.2609E5 | 1.6566E7 | 3.2384E7 | 1.6922E7 | 1.3707E7 | 4.2621E6 | 12 | 1 | 1 | 2 | 2 | 2 | 2 | 2 |  | Carbamidomethylation | C5:Carbamidomethylation:1000.00;C10:Carbamidomethylation:1000.00;C11:Carbamidomethylation:1000.00 | PEAKS DB |
| LASYLDKVQALEEANNDLENK | 114.91 | 2376.1809 | 21 | 0.4 | 793.0679 | 62.34 | 1 | F1:17066 | Fraction\_1\_23092022r.raw | 8.3051E6 | 8.0962E5 | 6.241E5 |  |  | 6.6574E5 | 5.0998E5 | 6 | 2 | 1 | 1 | 0 | 0 | 1 | 1 | P35527|K1C9\_HUMAN |  |  | PEAKS DB |
| IC(+57.02)ETDAHYVQIIHTSNNLGTER | 114.42 | 2570.2183 | 22 | 0.2 | 857.7468 | 44.41 | 5 | F5:9876 | Fraction\_5\_23092022.raw |  | 5.1745E5 | 2.2932E7 | 1.5069E7 | 4.1552E7 | 1.5463E7 | 4.742E6 | 12 | 0 | 1 | 3 | 2 | 2 | 2 | 2 | TRINITY\_DN2326\_c0\_g1\_i12.p1:C0HLL3|PA1\_VESVE | Carbamidomethylation | C2:Carbamidomethylation:1000.00 | PEAKS DB |
| VASISLPTSC(+57.02)ASAGTQC(+57.02)LISGWGNTK | 114.17 | 2665.2839 | 26 | 0.6 | 1333.6500 | 71.44 | 5 | F5:22034 | Fraction\_5\_23092022.raw | 8.4621E8 | 9.7042E5 | 7.1976E4 | 1.5115E5 | 3.8809E7 | 2.4884E5 |  | 17 | 9 | 2 | 1 | 1 | 3 | 1 | 0 | P00760|TRY1\_BOVIN | Carbamidomethylation | C10:Carbamidomethylation:1000.00;C17:Carbamidomethylation:1000.00 | PEAKS DB |
| SNRVFDLGIISYGSTC(+57.02)ADNSPGVNTK | 112.90 | 2771.3184 | 26 | -0.4 | 924.7797 | 63.64 | 3 | F3:18213 | Fraction\_3\_23092022.raw |  |  | 1.7606E6 | 4.8131E4 |  |  |  | 2 | 0 | 0 | 1 | 1 | 0 | 0 | 0 | TRINITY\_DN1494\_c0\_g1\_i8.p1 | Carbamidomethylation | C16:Carbamidomethylation:1000.00 | PEAKS DB |
| IVKDSDAKLIDC(+57.02)YELGDDTEWFGGPQLR | 112.84 | 3239.5444 | 28 | 0.3 | 1080.8557 | 70.20 | 1 | F1:20607 | Fraction\_1\_23092022r.raw | 8.1223E7 | 1.4069E6 |  |  |  |  |  | 4 | 3 | 1 | 0 | 0 | 0 | 0 | 0 | TRINITY\_DN4276\_c0\_g1\_i1.p1 | Carbamidomethylation | C12:Carbamidomethylation:1000.00 | PEAKS DB |
| YFTEC(+57.02)IRHEC(+57.02)C(+57.02)LIGVPQSKNPQPVSK | 112.05 | 3146.5100 | 26 | 0.1 | 787.6349 | 43.78 | 7 | F7:12462 | Fraction\_7\_23092022.raw |  | 4.4307E5 | 9.1365E6 | 2.4674E7 | 1.0734E7 | 9.0602E6 | 1.1587E7 | 11 | 0 | 1 | 2 | 3 | 1 | 2 | 2 |  | Carbamidomethylation | C5:Carbamidomethylation:1000.00;C10:Carbamidomethylation:1000.00;C11:Carbamidomethylation:1000.00 | PEAKS DB |
| LIGHSLGAQIAGFAGKEYQK | 111.41 | 2087.1162 | 20 | 0.0 | 696.7126 | 49.84 | 5 | F5:12302 | Fraction\_5\_23092022.raw |  |  | 3.7265E6 | 5.2349E6 | 7.6906E6 | 9.6072E6 | 2.4268E6 | 10 | 0 | 0 | 2 | 2 | 2 | 2 | 2 | TRINITY\_DN2326\_c0\_g1\_i12.p1:C0HLL3|PA1\_VESVE |  |  | PEAKS DB |
| ASGLTNAEKLEILKQHNEFR | 111.21 | 2297.2126 | 20 | -0.5 | 766.7444 | 41.28 | 7 | F7:11300 | Fraction\_7\_23092022.raw |  |  | 1.0005E6 | 5.4413E7 | 4.0692E6 | 3.7503E7 | 2.527E8 | 11 | 0 | 0 | 1 | 2 | 2 | 2 | 4 | TRINITY\_DN6370\_c0\_g1\_i1.p1 |  |  | PEAKS DB |
| LQGIVSWGSGC(+57.02)AQK | 110.64 | 1489.7347 | 14 | 1.0 | 745.8754 | 50.99 | 5 | F5:12871 | Fraction\_5\_23092022.raw | 2.78E9 | 1.5163E8 | 1.6078E7 | 1.6759E8 | 3.4116E8 | 2.0995E8 | 6.2589E7 | 22 | 7 | 1 | 2 | 4 | 2 | 5 | 1 | P00760|TRY1\_BOVIN | Carbamidomethylation | C11:Carbamidomethylation:1000.00 | PEAKS DB |
| YSEIIGLDPAGPSFK | 110.20 | 1592.8086 | 15 | 1.4 | 797.4127 | 66.23 | 5 | F5:19733 | Fraction\_5\_23092022.raw | 3.4371E6 | 3.3474E6 | 1.0312E8 | 1.5296E8 | 1.0352E8 | 5.9801E7 |  | 12 | 1 | 1 | 3 | 3 | 2 | 2 | 0 | A0A0M3KKW3|PA1\_VESBA |  |  | PEAKS DB |
| AIGGGLSSVGGGSSTIKYTTTSSSSR | 109.90 | 2417.2034 | 26 | 0.7 | 806.7423 | 41.60 | 5 | F5:8647 | Fraction\_5\_23092022.raw | 4.9517E4 | 1.4777E5 |  |  | 4.6006E5 | 6.9356E4 |  | 4 | 1 | 1 | 0 | 0 | 1 | 1 | 0 | P48668|K2C6C\_HUMAN |  |  | PEAKS DB |
| NVQEILYLHYGEPGNLVDQYPTEVK | 109.89 | 2917.4497 | 25 | 0.3 | 973.4908 | 70.28 | 4 | F4:21723 | Fraction\_4\_23092022.raw |  |  | 2.3239E5 | 2.3856E6 | 4.719E5 | 2.1779E5 | 1.5527E6 | 5 | 0 | 0 | 1 | 1 | 1 | 1 | 1 | TRINITY\_DN3450\_c0\_g1\_i51.p1 |  |  | PEAKS DB |
| SKAEAESLYQSKYEELQITAGR | 108.66 | 2500.2444 | 22 | 0.2 | 834.4222 | 47.34 | 1 | F1:10360 | Fraction\_1\_23092022r.raw | 2.1498E7 | 9.2416E6 | 8.8604E6 | 1.3779E6 | 1.8892E6 | 8.9535E6 | 2.5166E6 | 13 | 2 | 2 | 2 | 2 | 2 | 2 | 1 | P04264|K2C1\_HUMAN |  |  | PEAKS DB |
| SYNPQLTSYDYDAPLTEAGDPTEKYFLIRDVISK | 108.25 | 3908.8994 | 34 | 1.2 | 978.2333 | 76.80 | 2 | F2:23726 | Fraction\_2\_23092022.raw | 1.0871E6 | 5.7549E6 |  |  |  |  |  | 2 | 1 | 1 | 0 | 0 | 0 | 0 | 0 | TRINITY\_DN11986\_c0\_g1\_i3.p1 |  |  | PEAKS DB |
| GQYYLHQFVAGQPDLNYR | 108.15 | 2168.0439 | 18 | 0.0 | 1085.0293 | 56.80 | 2 | F2:14732 | Fraction\_2\_23092022.raw | 1.552E7 | 1.7261E8 | 3.7628E6 | 2.3123E6 | 1.1948E6 | 1.6543E6 |  | 8 | 1 | 2 | 2 | 1 | 1 | 1 | 0 | TRINITY\_DN10047\_c0\_g1\_i1.p1 |  |  | PEAKS DB |
| GPTLIDVNC(+57.02)GTDNVC(+57.02)LK | 107.75 | 1874.8866 | 17 | 1.0 | 938.4515 | 57.48 | 1 | F1:14848 | Fraction\_1\_23092022r.raw | 3.3406E7 |  |  |  |  |  |  | 1 | 1 | 0 | 0 | 0 | 0 | 0 | 0 | TRINITY\_DN4276\_c0\_g1\_i1.p1 | Carbamidomethylation | C9:Carbamidomethylation:1000.00;C15:Carbamidomethylation:1000.00 | PEAKS DB |
| IC(+57.02)ETDANYVQIIHTSSFK | 107.70 | 2125.0149 | 18 | -1.5 | 709.3445 | 55.25 | 5 | F5:14755 | Fraction\_5\_23092022.raw |  |  | 4.4581E5 | 1.9754E6 | 8.6142E5 | 2.2597E6 |  | 4 | 0 | 0 | 1 | 1 | 1 | 1 | 0 | TRINITY\_DN2326\_c0\_g1\_i13.p1 | Carbamidomethylation | C2:Carbamidomethylation:1000.00 | PEAKS DB |
| YTDLHADITPLILDRMNEC(+57.02)IK | 107.49 | 2530.2559 | 21 | 0.1 | 844.4260 | 68.72 | 1 | F1:19973 | Fraction\_1\_23092022r.raw | 1.5863E8 |  |  |  |  |  |  | 7 | 7 | 0 | 0 | 0 | 0 | 0 | 0 | TRINITY\_DN4276\_c0\_g1\_i1.p1 | Carbamidomethylation | C19:Carbamidomethylation:1000.00 | PEAKS DB |
| SLVGLGGTKSISISVAGGGGGFGAAGGFGGR | 107.15 | 2650.3826 | 31 | 1.2 | 884.4692 | 66.92 | 5 | F5:19957 | Fraction\_5\_23092022.raw | 2.5347E5 | 3.1515E5 | 6.9208E4 |  | 3.8346E5 | 2.0756E5 |  | 5 | 1 | 1 | 1 | 0 | 1 | 1 | 0 | P35908|K22E\_HUMAN |  |  | PEAKS DB |
| SAYPGQITSNMFC(+57.02)AGYLEGGK | 106.76 | 2250.0085 | 21 | 1.0 | 1126.0127 | 69.01 | 5 | F5:20902 | Fraction\_5\_23092022.raw | 5.3917E8 | 5.8696E5 | 5.5681E5 | 3.2969E5 | 1.0035E6 | 4.6883E5 |  | 9 | 3 | 1 | 1 | 1 | 2 | 1 | 0 | P00760|TRY1\_BOVIN | Carbamidomethylation | C13:Carbamidomethylation:1000.00 | PEAKS DB |
| APIELVGTDNVLSNVQWK | 106.26 | 1982.0472 | 18 | 0.6 | 992.0315 | 75.90 | 6 | F6:24672 | Fraction\_6\_23092022.raw |  |  |  |  |  | 2.1479E5 |  | 1 | 0 | 0 | 0 | 0 | 0 | 1 | 0 | TRINITY\_DN3450\_c0\_g1\_i51.p1 |  |  | PEAKS DB |
| FSSC(+57.02)GGGGGSFGAGGGFGSR | 105.96 | 1764.7274 | 20 | 1.0 | 883.3718 | 42.93 | 7 | F7:12066 | Fraction\_7\_23092022.raw | 1.8294E7 | 9.2408E6 | 4.9664E6 | 1.3448E6 | 9.6022E5 | 3.0926E6 | 1.4195E6 | 10 | 3 | 1 | 2 | 1 | 1 | 1 | 1 | P04264|K2C1\_HUMAN | Carbamidomethylation | C4:Carbamidomethylation:1000.00 | PEAKS DB |
| IC(+57.02)ETDANYVQIIHTSSFKGTLR | 105.86 | 2552.2693 | 22 | 0.6 | 639.0750 | 54.07 | 5 | F5:14209 | Fraction\_5\_23092022.raw |  |  | 1.3949E6 | 4.2902E6 | 2.5963E6 | 6.3456E6 | 1.7424E6 | 9 | 0 | 0 | 2 | 2 | 2 | 2 | 1 | TRINITY\_DN2326\_c0\_g1\_i13.p1 | Carbamidomethylation | C2:Carbamidomethylation:1000.00 | PEAKS DB |
| IVGGENANRGEFPYQVSLQWGLTSSNTK | 105.80 | 3051.5049 | 28 | 0.9 | 1018.1765 | 68.30 | 7 | F7:26144 | Fraction\_7\_23092022.raw |  |  | 7.4009E5 |  | 1.8087E6 | 1.5957E6 | 3.6201E6 | 4 | 0 | 0 | 1 | 0 | 1 | 1 | 1 | TRINITY\_DN33420\_c0\_g1\_i1.p1 |  |  | PEAKS DB |
| GPTLIDVNC(+57.02)GTDNVC(+57.02)LKNEDVIHTVIRLSTNSDVINVSR | 105.73 | 4337.1743 | 39 | 1.1 | 868.4431 | 76.73 | 1 | F1:23503 | Fraction\_1\_23092022r.raw | 3.6443E6 |  |  |  |  |  |  | 2 | 2 | 0 | 0 | 0 | 0 | 0 | 0 | TRINITY\_DN4276\_c0\_g1\_i1.p1 | Carbamidomethylation | C9:Carbamidomethylation:1000.00;C15:Carbamidomethylation:1000.00 | PEAKS DB |
| LIGHSLGAQIAGFAGK | 105.57 | 1538.8568 | 16 | -0.6 | 513.9592 | 52.10 | 5 | F5:13308 | Fraction\_5\_23092022.raw |  |  | 9.856E6 | 7.8605E6 | 1.4847E7 | 1.2515E7 | 2.5951E6 | 9 | 0 | 0 | 2 | 2 | 2 | 2 | 1 | TRINITY\_DN2326\_c0\_g1\_i12.p1:C0HLL3|PA1\_VESVE |  |  | PEAKS DB |
| DIENQYETQITQIEHEVSSSGQEVQSSAK | 105.54 | 3263.5066 | 29 | 0.6 | 1088.8435 | 74.64 | 3 | F3:23085 | Fraction\_3\_23092022.raw | 1.3801E7 | 5.2053E6 | 3.1999E6 | 5.8106E5 | 6.3169E5 | 2.837E6 | 2.7315E6 | 8 | 2 | 1 | 1 | 1 | 1 | 1 | 1 | P35527|K1C9\_HUMAN |  |  | PEAKS DB |
| NVSTGDVNVEMNAAPGVDLTQLLNNM(+15.99)R | 105.53 | 2887.3804 | 27 | 1.4 | 963.4688 | 79.48 | 1 | F1:24717 | Fraction\_1\_23092022r.raw | 3.7272E6 |  |  |  |  |  |  | 2 | 2 | 0 | 0 | 0 | 0 | 0 | 0 | P13645|K1C10\_HUMAN | Oxidation (M) | M26:Oxidation (M):133.36 | PEAKS DB |
| AGTSNPVVSLTLVDLHDPTLNKIDLK | 105.10 | 2759.5068 | 26 | 1.0 | 690.8846 | 72.00 | 5 | F5:22224 | Fraction\_5\_23092022.raw |  |  | 2.0917E6 | 7.4661E6 | 2.1356E6 | 9.6734E5 |  | 7 | 0 | 0 | 2 | 2 | 2 | 1 | 0 | TRINITY\_DN3450\_c0\_g1\_i51.p1 |  |  | PEAKS DB |
| YSLGRVPIGGSDFSTR | 104.69 | 1710.8689 | 16 | 1.9 | 571.2980 | 51.63 | 2 | F2:12390 | Fraction\_2\_23092022.raw |  | 1.0063E7 |  |  |  |  |  | 1 | 0 | 1 | 0 | 0 | 0 | 0 | 0 | TRINITY\_DN122\_c0\_g1\_i5.p1 |  |  | PEAKS DB |
| LVGNYIATVTKM(+15.99)LVQQYNVPM(+15.99)ANIR | 104.63 | 2867.5037 | 25 | 0.6 | 956.8424 | 80.21 | 4 | F4:26218 | Fraction\_4\_23092022.raw |  |  |  | 1.3055E7 | 1.436E7 |  | 1.0563E6 | 9 | 0 | 0 | 0 | 4 | 4 | 0 | 1 |  | Oxidation (M) | M12:Oxidation (M):1000.00;M21:Oxidation (M):1000.00 | PEAKS DB |
| IIHQC(+57.02)PPGLHFNKELQIC(+57.02)DLPEHAK | 104.54 | 2993.5002 | 25 | 1.1 | 599.7080 | 46.12 | 7 | F7:13546 | Fraction\_7\_23092022.raw |  |  |  | 1.4228E5 | 2.7779E6 |  | 6.1179E6 | 6 | 0 | 0 | 0 | 1 | 3 | 0 | 2 | TRINITY\_DN2925\_c0\_g1\_i5.p1 | Carbamidomethylation | C5:Carbamidomethylation:1000.00;C18:Carbamidomethylation:1000.00 | PEAKS DB |
| SIVHPSYNSNTLNNDIM(+15.99)LIK | 104.45 | 2288.1470 | 20 | 0.5 | 763.7233 | 56.09 | 7 | F7:19157 | Fraction\_7\_23092022.raw | 5.7658E9 | 1.038E9 | 2.2611E8 | 8.2567E8 | 4.5647E8 | 3.7932E8 | 2.3519E8 | 51 | 8 | 8 | 10 | 4 | 6 | 8 | 7 | P00760|TRY1\_BOVIN | Oxidation (M) | M17:Oxidation (M):1000.00 | PEAKS DB |
| GTYIYVDMKDPLFLDQNNYM(+15.99)DKYIC(+57.02)LVAK | 104.36 | 3545.6921 | 29 | 0.5 | 887.4308 | 77.18 | 1 | F1:23700 | Fraction\_1\_23092022r.raw | 1.9986E6 |  |  |  |  |  |  | 1 | 1 | 0 | 0 | 0 | 0 | 0 | 0 | TRINITY\_DN4276\_c0\_g1\_i1.p1 | Oxidation (M); Carbamidomethylation | M20:Oxidation (M):160.66;C25:Carbamidomethylation:1000.00 | PEAKS DB |
| NQC(+57.02)VC(+57.02)VGLNAKTYPKTGSFYVPVESK | 104.28 | 2945.4414 | 26 | 0.2 | 737.3678 | 50.98 | 4 | F4:13049 | Fraction\_4\_23092022.raw |  |  | 7.2394E5 | 5.3522E6 | 9.4966E4 | 3.5396E5 |  | 5 | 0 | 0 | 1 | 2 | 1 | 1 | 0 |  | Carbamidomethylation | C3:Carbamidomethylation:1000.00;C5:Carbamidomethylation:1000.00 | PEAKS DB |
| DIVVVGAGMSGLSAAYVLAGAGHK | 104.20 | 2242.1780 | 24 | 0.0 | 748.3999 | 79.31 | 1 | F1:24687 | Fraction\_1\_23092022r.raw | 2.516E5 |  |  |  |  |  |  | 1 | 1 | 0 | 0 | 0 | 0 | 0 | 0 | B5U6Y8|OXLA\_ECHOC |  |  | PEAKS DB |
| NQILNLTTDNANILLQIDNAR | 104.06 | 2366.2554 | 21 | 0.1 | 1184.1351 | 82.41 | 1 | F1:26051 | Fraction\_1\_23092022r.raw | 2.675E6 | 8.2335E5 | 1.1205E6 | 9.2039E4 |  | 1.0172E5 |  | 7 | 2 | 1 | 2 | 1 | 0 | 1 | 0 | P13645|K1C10\_HUMAN |  |  | PEAKS DB |
| SYVYFSESIENPHGFR | 103.40 | 1930.8849 | 16 | 0.6 | 644.6360 | 55.92 | 5 | F5:15053 | Fraction\_5\_23092022.raw |  |  |  | 1.2044E6 | 8.6364E5 |  |  | 3 | 0 | 0 | 0 | 1 | 2 | 0 | 0 | TRINITY\_DN594\_c1\_g4\_i5.p1:TRINITY\_DN594\_c1\_g4\_i1.p1 |  |  | PEAKS DB |
| DIENQYETQITQIEHEVSSSGQEVQSSAKEVTQLR | 103.11 | 3989.9089 | 35 | 0.5 | 998.4850 | 79.81 | 2 | F2:25063 | Fraction\_2\_23092022.raw | 2.8028E6 | 3.4203E6 | 6.2879E5 | 1.5665E5 | 2.5602E5 | 1.3422E6 |  | 6 | 1 | 1 | 1 | 1 | 1 | 1 | 0 | P35527|K1C9\_HUMAN |  |  | PEAKS DB |
| LIGHSLGAHVSGFAGK | 102.96 | 1549.8364 | 16 | 0.5 | 517.6197 | 34.32 | 7 | F7:8159 | Fraction\_7\_23092022.raw |  |  | 8.726E6 | 2.4662E7 | 1.3571E7 | 6.2907E7 | 6.214E7 | 14 | 0 | 0 | 2 | 3 | 3 | 3 | 3 | TRINITY\_DN2326\_c0\_g1\_i13.p1 |  |  | PEAKS DB |
| FC(+57.02)GLNSFVVESISSK | 102.85 | 1672.8130 | 15 | 1.0 | 837.4146 | 71.17 | 5 | F5:21870 | Fraction\_5\_23092022.raw |  |  |  | 7.1285E5 | 1.0534E6 |  |  | 2 | 0 | 0 | 0 | 1 | 1 | 0 | 0 | TRINITY\_DN1494\_c0\_g1\_i8.p1 | Carbamidomethylation | C2:Carbamidomethylation:1000.00 | PEAKS DB |
| TPIIESGSLESILVTEK | 102.40 | 1814.9877 | 17 | -0.6 | 908.5006 | 74.02 | 2 | F2:22451 | Fraction\_2\_23092022.raw | 5.1461E6 | 1.1492E7 | 8.2889E5 | 6.9336E4 | 1.196E5 | 1.6955E5 |  | 8 | 2 | 2 | 1 | 1 | 1 | 1 | 0 | TRINITY\_DN10047\_c0\_g1\_i1.p1 |  |  | PEAKS DB |
| AEAESLYQSKYEELQITAGR | 102.37 | 2285.1174 | 20 | 0.9 | 762.7138 | 52.63 | 3 | F3:13276 | Fraction\_3\_23092022.raw | 8.3304E6 | 2.3011E6 | 2.5869E6 |  |  | 1.8828E6 |  | 5 | 2 | 1 | 1 | 0 | 0 | 1 | 0 | P04264|K2C1\_HUMAN |  |  | PEAKS DB |
| GSLGGGFSSGGFSGGSFSR | 102.25 | 1706.7648 | 19 | 0.3 | 854.3899 | 55.16 | 5 | F5:14691 | Fraction\_5\_23092022.raw | 3.7889E7 | 1.3171E7 | 1.8799E7 | 3.0863E6 | 5.0574E6 | 1.378E7 | 1.0368E7 | 10 | 1 | 1 | 4 | 1 | 1 | 1 | 1 | P13645|K1C10\_HUMAN |  |  | PEAKS DB |
| GSIGGGFSSGGFSGGSFSR | 102.25 | 1706.7648 | 19 | 0.3 | 854.3899 | 55.16 | 5 | F5:14691 | Fraction\_5\_23092022.raw | 3.7889E7 | 1.3171E7 | 1.8799E7 | 3.0863E6 | 5.0574E6 | 1.378E7 | 1.0368E7 | 10 | 1 | 1 | 4 | 1 | 1 | 1 | 1 |  |  |  | PEAKS DB |
| YTDLHADITPLILDR | 102.15 | 1754.9203 | 15 | 1.6 | 585.9816 | 65.87 | 1 | F1:18704 | Fraction\_1\_23092022r.raw | 4.1574E8 | 2.3241E6 |  |  |  |  |  | 9 | 7 | 2 | 0 | 0 | 0 | 0 | 0 | TRINITY\_DN4276\_c0\_g1\_i1.p1 |  |  | PEAKS DB |
| AVKYFTEC(+57.02)IRHEC(+57.02)C(+57.02)LIGVPQSK | 101.99 | 2694.3081 | 22 | -0.6 | 674.5839 | 47.27 | 7 | F7:14097 | Fraction\_7\_23092022.raw |  | 7.2065E5 | 8.0384E5 | 1.8626E7 | 1.4568E7 | 8.4117E6 | 1.2209E7 | 14 | 0 | 1 | 1 | 3 | 3 | 3 | 3 |  | Carbamidomethylation | C8:Carbamidomethylation:1000.00;C13:Carbamidomethylation:1000.00;C14:Carbamidomethylation:1000.00 | PEAKS DB |
| FLEQQNQVLQTKWELLQQVDTSTR | 101.90 | 2931.5090 | 24 | 1.4 | 978.1783 | 70.89 | 1 | F1:20862 | Fraction\_1\_23092022r.raw | 1.545E7 | 6.6621E5 |  |  | 1.0796E5 | 5.2756E5 |  | 6 | 3 | 1 | 0 | 0 | 1 | 1 | 0 | P04264|K2C1\_HUMAN |  |  | PEAKS DB |
| NVLHTQSSDILRGPTLIDVNC(+57.02)GTDNVC(+57.02)LK | 101.83 | 3238.6074 | 29 | 0.0 | 810.6591 | 61.01 | 1 | F1:16464 | Fraction\_1\_23092022r.raw | 1.2859E7 |  |  |  |  |  |  | 1 | 1 | 0 | 0 | 0 | 0 | 0 | 0 | TRINITY\_DN4276\_c0\_g1\_i1.p1 | Carbamidomethylation | C21:Carbamidomethylation:1000.00;C27:Carbamidomethylation:1000.00 | PEAKS DB |
| YSEIIGLDPAGPLFK | 101.75 | 1618.8606 | 15 | -0.2 | 810.4374 | 78.54 | 7 | F7:31178 | Fraction\_7\_23092022.raw |  | 1.0474E5 | 5.7087E6 | 1.3039E7 | 1.1011E7 | 4.0201E7 | 5.0361E7 | 6 | 0 | 1 | 1 | 1 | 1 | 1 | 1 | TRINITY\_DN2326\_c0\_g1\_i13.p1 |  |  | PEAKS DB |
| RLPGSTVVLLINFSNSDIQVDAR | 101.68 | 2513.3601 | 23 | 0.3 | 838.7943 | 78.23 | 2 | F2:24381 | Fraction\_2\_23092022.raw |  | 9.4099E5 |  |  |  |  |  | 1 | 0 | 1 | 0 | 0 | 0 | 0 | 0 | TRINITY\_DN10047\_c0\_g1\_i1.p1 |  |  | PEAKS DB |
| VPVHNVSVVDLTVR | 101.62 | 1532.8674 | 14 | -0.1 | 511.9630 | 48.43 | 7 | F7:14689 | Fraction\_7\_23092022.raw |  |  | 8.7264E5 | 7.3854E5 | 4.5131E5 | 8.643E6 | 1.271E6 | 6 | 0 | 0 | 1 | 1 | 1 | 2 | 1 | TRINITY\_DN5849\_c0\_g1\_i1.p1:TRINITY\_DN5849\_c0\_g1\_i2.p1:TRINITY\_DN5849\_c0\_g1\_i10.p1 |  |  | PEAKS DB |
| ATKELLFHAIDLPGLGYKR | 100.59 | 2141.1997 | 19 | 2.1 | 536.3083 | 59.56 | 7 | F7:21253 | Fraction\_7\_23092022.raw |  |  |  |  |  |  | 1.1769E6 | 1 | 0 | 0 | 0 | 0 | 0 | 0 | 1 | TRINITY\_DN2373\_c0\_g1\_i19.p1 |  |  | PEAKS DB |
| DPNYFIHKDATNLGYYVK | 100.58 | 2157.0530 | 18 | -0.2 | 720.0248 | 54.78 | 1 | F1:13669 | Fraction\_1\_23092022r.raw | 3.1758E6 | 3.3896E5 |  |  |  |  |  | 3 | 2 | 1 | 0 | 0 | 0 | 0 | 0 | TRINITY\_DN1709\_c0\_g1\_i4.p1:TRINITY\_DN1709\_c0\_g1\_i5.p1:TRINITY\_DN1709\_c0\_g1\_i6.p1:TRINITY\_DN1709\_c0\_g1\_i8.p1 |  |  | PEAKS DB |
| THNLEPYFESFINNLR | 100.49 | 1992.9694 | 16 | 0.2 | 665.3305 | 78.54 | 4 | F4:25449 | Fraction\_4\_23092022.raw | 7.8036E6 | 3.6542E6 | 9.2992E5 | 4.1137E5 |  | 1.6729E6 | 2.437E6 | 9 | 2 | 2 | 1 | 1 | 0 | 2 | 1 | P04264|K2C1\_HUMAN |  |  | PEAKS DB |
| VQELRLGKYSEIIGLDPAGPSFK | 99.90 | 2516.3638 | 23 | 0.6 | 839.7957 | 64.95 | 4 | F4:19342 | Fraction\_4\_23092022.raw |  |  | 1.5389E6 | 7.6412E6 | 5.5702E6 | 1.882E6 | 3.5477E6 | 10 | 0 | 0 | 2 | 2 | 2 | 2 | 2 |  |  |  | PEAKS DB |
| GGGFGGGSGFGGGSGFGGGSGFSGGGFGGGGFGGGR | 99.61 | 2830.1868 | 36 | 1.2 | 1416.1023 | 70.34 | 1 | F1:20652 | Fraction\_1\_23092022r.raw | 2.4621E6 | 1.4833E5 | 2.4652E6 |  | 5.2791E5 | 1.55E6 | 8.2632E5 | 10 | 2 | 1 | 2 | 0 | 2 | 2 | 1 |  |  |  | PEAKS DB |
| ASGLTNAEKLEILKQHNEFRQK | 99.09 | 2553.3662 | 22 | 3.0 | 511.6820 | 36.03 | 7 | F7:8978 | Fraction\_7\_23092022.raw |  |  |  | 4.3408E6 | 8.2582E5 | 3.2321E6 | 5.8582E8 | 8 | 0 | 0 | 0 | 1 | 1 | 1 | 5 | TRINITY\_DN6370\_c0\_g1\_i1.p1 |  |  | PEAKS DB |
| VQELRLGKYSEIIGLDPAGPSFKSQEC(+57.02)SQR | 98.88 | 3391.7192 | 30 | -0.4 | 679.3508 | 58.20 | 4 | F4:16337 | Fraction\_4\_23092022.raw |  |  |  | 4.7224E5 | 3.7864E5 |  |  | 2 | 0 | 0 | 0 | 1 | 1 | 0 | 0 |  | Carbamidomethylation | C27:Carbamidomethylation:1000.00 | PEAKS DB |
| SAYPGQITSNM(+15.99)FC(+57.02)AGYLEGGK | 98.79 | 2266.0034 | 21 | 0.2 | 1134.0092 | 59.38 | 5 | F5:16637 | Fraction\_5\_23092022.raw | 4.4784E8 | 2.8128E7 | 8.2349E6 | 1.2119E7 | 2.0754E7 | 1.3701E7 | 1.0659E7 | 38 | 14 | 4 | 4 | 4 | 4 | 4 | 4 | P00760|TRY1\_BOVIN | Oxidation (M); Carbamidomethylation | M11:Oxidation (M):1000.00;C13:Carbamidomethylation:1000.00 | PEAKS DB |
| ELTTEIDNNIEQISSYK | 98.62 | 1995.9636 | 17 | 0.4 | 998.9895 | 66.77 | 1 | F1:19027 | Fraction\_1\_23092022r.raw | 3.7204E6 | 1.491E6 | 4.4544E6 | 7.7249E5 | 5.7468E5 | 7.3368E5 |  | 6 | 1 | 1 | 1 | 1 | 1 | 1 | 0 | P13645|K1C10\_HUMAN |  |  | PEAKS DB |
| SYFHITDQPLDTYLDTTGWGK | 98.61 | 2457.1489 | 21 | 0.3 | 820.0571 | 77.78 | 1 | F1:23980 | Fraction\_1\_23092022r.raw | 3.0562E5 |  |  |  |  |  |  | 1 | 1 | 0 | 0 | 0 | 0 | 0 | 0 | TRINITY\_DN11986\_c0\_g1\_i3.p1 |  |  | PEAKS DB |
| EIETYHNLLEGGQEDFESSGAGK | 98.51 | 2509.1245 | 23 | 0.1 | 837.3822 | 55.97 | 1 | F1:14223 | Fraction\_1\_23092022r.raw | 6.3311E6 | 8.0004E5 | 5.5376E5 |  |  | 6.5908E5 |  | 5 | 2 | 1 | 1 | 0 | 0 | 1 | 0 | P35527|K1C9\_HUMAN |  |  | PEAKS DB |
| DYVTALANNFDDNIEVR | 97.99 | 1967.9225 | 17 | 0.4 | 984.9689 | 75.48 | 1 | F1:22928 | Fraction\_1\_23092022r.raw | 1.2398E9 | 1.5506E7 | 5.3382E5 | 2.1294E5 | 1.3799E5 |  |  | 13 | 8 | 2 | 1 | 1 | 1 | 0 | 0 | TRINITY\_DN4276\_c0\_g1\_i1.p1 |  |  | PEAKS DB |
| TGSFYVPVESKAPYC(+57.02)NNK | 97.81 | 2059.9673 | 18 | 0.0 | 1030.9910 | 45.28 | 3 | F3:9956 | Fraction\_3\_23092022.raw |  |  | 3.5089E8 | 1.0426E8 |  | 1.0646E6 |  | 12 | 0 | 0 | 7 | 4 | 0 | 1 | 0 | A0A0M3KKW3|PA1\_VESBA:C0HLL3|PA1\_VESVE | Carbamidomethylation | C15:Carbamidomethylation:1000.00 | PEAKS DB |
| TYPKTGSFYVPVER | 97.78 | 1642.8354 | 14 | 0.3 | 548.6193 | 45.36 | 7 | F7:13154 | Fraction\_7\_23092022.raw |  |  | 1.257E5 | 3.071E6 |  | 1.0585E6 | 4.1939E6 | 6 | 0 | 0 | 1 | 2 | 0 | 1 | 2 | TRINITY\_DN2326\_c0\_g1\_i12.p1 |  |  | PEAKS DB |
| HGVQELEIELQSQLSK | 97.55 | 1836.9581 | 16 | 0.8 | 919.4871 | 63.63 | 2 | F2:17855 | Fraction\_2\_23092022.raw | 1.322E7 | 5.9817E6 | 3.2595E6 | 4.595E5 | 2.431E5 | 1.8834E6 | 2.5033E6 | 10 | 2 | 2 | 2 | 1 | 1 | 1 | 1 | P35527|K1C9\_HUMAN |  |  | PEAKS DB |
| SYNPQLTSYDYDAPLTEAGDPTEKYFLIR | 97.53 | 3366.5930 | 29 | 1.1 | 1123.2062 | 75.19 | 2 | F2:23001 | Fraction\_2\_23092022.raw |  | 1.2172E6 |  |  |  |  |  | 1 | 0 | 1 | 0 | 0 | 0 | 0 | 0 | TRINITY\_DN11986\_c0\_g1\_i3.p1 |  |  | PEAKS DB |
| NVSTGDVNVEM(+15.99)NAAPGVDLTQLLNNM(+15.99)R | 97.09 | 2903.3752 | 27 | 1.3 | 968.8003 | 74.06 | 1 | F1:22288 | Fraction\_1\_23092022r.raw | 1.0332E7 | 1.0315E6 | 7.2533E5 | 3.3252E5 | 2.0239E5 | 3.6883E5 |  | 8 | 2 | 2 | 1 | 1 | 1 | 1 | 0 | P13645|K1C10\_HUMAN | Oxidation (M) | M11:Oxidation (M):1000.00;M26:Oxidation (M):1000.00 | PEAKS DB |
| TGAPLNPPIWWVDPTDKTAHK | 96.88 | 2343.2012 | 21 | 0.0 | 782.0743 | 65.11 | 1 | F1:18306 | Fraction\_1\_23092022r.raw | 2.0499E8 | 2.8895E5 |  |  |  |  |  | 5 | 4 | 1 | 0 | 0 | 0 | 0 | 0 | TRINITY\_DN4276\_c0\_g1\_i1.p1 |  |  | PEAKS DB |
| GTYIYVDMKDPLFLDQNNYM(+15.99)DK | 96.44 | 2698.2295 | 22 | 0.9 | 900.4179 | 72.90 | 1 | F1:21806 | Fraction\_1\_23092022r.raw | 1.7664E6 |  |  |  |  |  |  | 1 | 1 | 0 | 0 | 0 | 0 | 0 | 0 | TRINITY\_DN4276\_c0\_g1\_i1.p1 | Oxidation (M) | M20:Oxidation (M):181.38 | PEAKS DB |
| SGYRSGGGFSSGSAGIINYQR | 96.44 | 2120.0034 | 21 | -0.2 | 707.6749 | 41.81 | 7 | F7:11558 | Fraction\_7\_23092022.raw |  | 2.7805E5 |  |  |  |  | 3.3385E5 | 2 | 0 | 1 | 0 | 0 | 0 | 0 | 1 | P04264|K2C1\_HUMAN |  |  | PEAKS DB |
| DFTYEETVDPAGC(+57.02)QAGPDR | 96.40 | 2126.8850 | 19 | 0.8 | 1064.4507 | 52.44 | 2 | F2:12778 | Fraction\_2\_23092022.raw | 3.8165E5 | 5.7528E5 |  |  |  |  |  | 2 | 1 | 1 | 0 | 0 | 0 | 0 | 0 | TRINITY\_DN10047\_c0\_g1\_i1.p1 | Carbamidomethylation | C13:Carbamidomethylation:1000.00 | PEAKS DB |
| SKEEAEALYHSKYEELQVTVGR | 96.23 | 2565.2710 | 22 | 0.8 | 856.0983 | 44.97 | 1 | F1:9339 | Fraction\_1\_23092022r.raw | 7.0702E6 | 8.8942E5 | 1.2092E6 |  |  |  |  | 4 | 2 | 1 | 1 | 0 | 0 | 0 | 0 | P35908|K22E\_HUMAN |  |  | PEAKS DB |
| AVEYLTEC(+57.02)IRHEC(+57.02)C(+57.02)LIGVPQSK | 96.18 | 2661.2712 | 22 | 0.1 | 888.0978 | 57.79 | 3 | F3:15577 | Fraction\_3\_23092022.raw |  |  | 3.5563E6 | 1.083E6 | 7.3455E5 | 2.3381E6 | 5.9298E6 | 10 | 0 | 0 | 2 | 2 | 2 | 2 | 2 | TRINITY\_DN2326\_c0\_g1\_i12.p1 | Carbamidomethylation | C8:Carbamidomethylation:1000.00;C13:Carbamidomethylation:1000.00;C14:Carbamidomethylation:1000.00 | PEAKS DB |
| HGVQELEIELQSQLSKK | 96.03 | 1965.0531 | 17 | 0.4 | 656.0253 | 56.76 | 7 | F7:19525 | Fraction\_7\_23092022.raw | 1.2248E7 |  |  |  |  | 2.7415E6 | 2.1209E6 | 3 | 1 | 0 | 0 | 0 | 0 | 1 | 1 | P35527|K1C9\_HUMAN |  |  | PEAKS DB |
| LLEGEDAHLSSSQFSSGSQSSR | 95.80 | 2308.0566 | 22 | 0.7 | 770.3600 | 40.83 | 1 | F1:7538 | Fraction\_1\_23092022r.raw | 6.2238E5 |  |  |  |  |  |  | 1 | 1 | 0 | 0 | 0 | 0 | 0 | 0 | P02533|K1C14\_HUMAN |  |  | PEAKS DB |
| SLYNNLKAGAESGWDFSGR | 95.54 | 2070.9758 | 19 | 0.7 | 691.3330 | 59.17 | 2 | F2:15841 | Fraction\_2\_23092022.raw |  | 9.8861E5 |  |  |  |  |  | 1 | 0 | 1 | 0 | 0 | 0 | 0 | 0 | TRINITY\_DN10256\_c0\_g10\_i1.p1:TRINITY\_DN10256\_c0\_g5\_i4.p1 |  |  | PEAKS DB |
| ALIYVDSELVGTLSR | 95.49 | 1634.8879 | 15 | 0.9 | 818.4520 | 74.52 | 4 | F4:23613 | Fraction\_4\_23092022.raw | 1.336E6 | 1.7629E6 | 7.0291E5 | 3.4769E5 |  | 3.8846E5 |  | 6 | 1 | 2 | 1 | 1 | 0 | 1 | 0 | TRINITY\_DN11986\_c0\_g1\_i3.p1 |  |  | PEAKS DB |
| ELGLQYPGGYKVEDLYEDVNYGVLLPHTK | 95.28 | 3308.6604 | 29 | 0.9 | 828.1732 | 78.63 | 2 | F2:24536 | Fraction\_2\_23092022.raw |  | 5.2189E6 |  |  |  |  |  | 2 | 0 | 2 | 0 | 0 | 0 | 0 | 0 | TRINITY\_DN2611\_c0\_g1\_i12.p1:TRINITY\_DN2611\_c0\_g1\_i23.p1:TRINITY\_DN2611\_c0\_g1\_i19.p1:TRINITY\_DN2611\_c0\_g1\_i9.p1 |  |  | PEAKS DB |
| TGQNLLEILELQYVPVTRK | 95.15 | 2213.2419 | 19 | 0.3 | 738.7548 | 82.32 | 7 | F7:32804 | Fraction\_7\_23092022.raw | 3.5673E5 | 1.8611E6 |  | 1.6914E5 |  |  | 6.3833E5 | 4 | 1 | 1 | 0 | 1 | 0 | 0 | 1 | TRINITY\_DN11986\_c0\_g1\_i3.p1 |  |  | PEAKS DB |
| IIAATIENAQPILQIDNAR | 95.10 | 2063.1375 | 19 | 0.2 | 1032.5762 | 69.02 | 1 | F1:20068 | Fraction\_1\_23092022r.raw | 5.8134E5 |  | 2.1668E5 |  |  |  |  | 4 | 2 | 0 | 2 | 0 | 0 | 0 | 0 | P08779|K1C16\_HUMAN |  |  | PEAKS DB |
| TLLEGEESRM(+15.99)SGEC(+57.02)APNVSVSVSTSHTTISGGGSR | 94.59 | 3594.6526 | 35 | 0.2 | 899.6706 | 44.22 | 1 | F1:8997 | Fraction\_1\_23092022r.raw | 1.8861E6 | 4.5726E5 |  |  |  |  |  | 2 | 1 | 1 | 0 | 0 | 0 | 0 | 0 | P04264|K2C1\_HUMAN | Oxidation (M); Carbamidomethylation | M10:Oxidation (M):1000.00;C14:Carbamidomethylation:1000.00 | PEAKS DB |
| LLKDDAFGVDEALNEM(+15.99)AYDEGLVVR | 94.27 | 2797.3479 | 25 | 1.5 | 933.4580 | 73.18 | 3 | F3:22468 | Fraction\_3\_23092022.raw |  |  | 3.7553E5 |  |  |  |  | 1 | 0 | 0 | 1 | 0 | 0 | 0 | 0 | TRINITY\_DN2373\_c0\_g1\_i12.p1:TRINITY\_DN2373\_c0\_g1\_i1.p1:TRINITY\_DN2373\_c0\_g1\_i22.p1 | Oxidation (M) | M16:Oxidation (M):1000.00 | PEAKS DB |
| ISSSKGSLGGGFSSGGFSGGSFSR | 94.19 | 2209.0398 | 24 | 0.3 | 737.3541 | 47.03 | 7 | F7:13977 | Fraction\_7\_23092022.raw | 6.1881E5 | 2.6543E6 |  |  | 5.7646E5 | 6.1611E5 | 3.3283E6 | 5 | 1 | 1 | 0 | 0 | 1 | 1 | 1 | P13645|K1C10\_HUMAN |  |  | PEAKS DB |
| ISSSKGSIGGGFSSGGFSGGSFSR | 94.19 | 2209.0398 | 24 | 0.3 | 737.3541 | 47.03 | 7 | F7:13977 | Fraction\_7\_23092022.raw | 6.1881E5 | 2.6543E6 |  |  | 5.7646E5 | 6.1611E5 | 3.3283E6 | 5 | 1 | 1 | 0 | 0 | 1 | 1 | 1 |  |  |  | PEAKS DB |
| TYPKTGSFYVPVESKAPYC(+57.02)NNK | 94.16 | 2549.2261 | 22 | 0.5 | 638.3141 | 43.76 | 3 | F3:9275 | Fraction\_3\_23092022.raw |  |  | 5.2116E6 | 4.0395E6 |  |  |  | 4 | 0 | 0 | 2 | 2 | 0 | 0 | 0 | A0A0M3KKW3|PA1\_VESBA | Carbamidomethylation | C19:Carbamidomethylation:1000.00 | PEAKS DB |
| GTYIYVDM(+15.99)KDPLFLDQNNYM(+15.99)DKYIC(+57.02)LVAK | 94.12 | 3561.6870 | 29 | 0.9 | 891.4298 | 71.47 | 1 | F1:21196 | Fraction\_1\_23092022r.raw | 1.0129E7 |  |  |  |  |  |  | 1 | 1 | 0 | 0 | 0 | 0 | 0 | 0 | TRINITY\_DN4276\_c0\_g1\_i1.p1 | Oxidation (M); Carbamidomethylation | M8:Oxidation (M):1000.00;M20:Oxidation (M):1000.00;C25:Carbamidomethylation:1000.00 | PEAKS DB |
| MIAPILDEIADEYQGKLTVAK | 94.00 | 2317.2239 | 21 | -0.3 | 773.4150 | 82.46 | 1 | F1:26078 | Fraction\_1\_23092022r.raw | 7.4787E5 |  |  |  |  |  |  | 1 | 1 | 0 | 0 | 0 | 0 | 0 | 0 | P0AA29|THIO\_SALTI:P0AA26|THIO\_ECOL6:P0AA27|THIO\_ECO57:P0AA30|THIO\_SHIFL:P0AA25|THIO\_ECOLI:P0AA28|THIO\_SALTY |  |  | PEAKS DB |
| ESITLNYEIGIFSNPR | 93.89 | 1851.9366 | 16 | 0.1 | 926.9757 | 82.16 | 3 | F3:26521 | Fraction\_3\_23092022.raw | 1.4576E8 | 2.064E7 | 4.7708E6 | 1.2075E6 |  |  |  | 12 | 5 | 2 | 3 | 2 | 0 | 0 | 0 | TRINITY\_DN4276\_c0\_g1\_i1.p1 |  |  | PEAKS DB |
| TYPKTGSFYVPVESK | 93.80 | 1701.8613 | 15 | 0.4 | 851.9383 | 45.22 | 3 | F3:9935 | Fraction\_3\_23092022.raw | 1.9E5 | 2.1319E6 | 2.3998E7 | 7.7395E7 | 2.1649E6 | 1.0072E7 | 4.9841E6 | 14 | 1 | 2 | 3 | 2 | 2 | 2 | 2 | A0A0M3KKW3|PA1\_VESBA |  |  | PEAKS DB |
| TGAPLNPPIWWVDPTDK | 93.64 | 1905.9624 | 17 | 0.7 | 953.9891 | 81.96 | 1 | F1:25869 | Fraction\_1\_23092022r.raw | 6.395E7 |  |  |  |  |  |  | 1 | 1 | 0 | 0 | 0 | 0 | 0 | 0 | TRINITY\_DN4276\_c0\_g1\_i1.p1 |  |  | PEAKS DB |
| TGSFYVPVESKAPYC(+57.02)NNKGKII | 93.49 | 2471.2517 | 22 | 0.6 | 618.8206 | 48.89 | 4 | F4:12117 | Fraction\_4\_23092022.raw |  | 2.8013E6 | 5.6198E6 | 8.8359E6 | 3.2966E5 | 1.2371E6 | 1.0754E7 | 11 | 0 | 2 | 2 | 2 | 1 | 2 | 2 | A0A0M3KKW3|PA1\_VESBA | Carbamidomethylation | C15:Carbamidomethylation:1000.00 | PEAKS DB |
| TSQNSELNNMQDLVEDYKK | 93.44 | 2255.0376 | 19 | 0.9 | 752.6871 | 56.35 | 1 | F1:14382 | Fraction\_1\_23092022r.raw | 4.5807E6 |  |  |  |  |  |  | 2 | 2 | 0 | 0 | 0 | 0 | 0 | 0 | P35908|K22E\_HUMAN |  |  | PEAKS DB |
| LVPEEISFVLSTR | 93.13 | 1488.8187 | 13 | 0.7 | 497.2805 | 76.42 | 7 | F7:30218 | Fraction\_7\_23092022.raw |  |  | 2.5384E7 | 5.5521E6 |  | 3.1395E5 | 7.2902E7 | 8 | 0 | 0 | 3 | 2 | 0 | 1 | 2 | TRINITY\_DN2326\_c0\_g1\_i12.p1 |  |  | PEAKS DB |
| IFFAGEYTANAHGWIDSTIK | 93.11 | 2240.0901 | 20 | 1.1 | 747.7048 | 69.01 | 7 | F7:26501 | Fraction\_7\_23092022.raw | 6.8963E6 | 5.4364E6 | 3.7554E6 | 3.4857E6 | 3.1336E6 | 2.7058E6 | 3.37E6 | 15 | 4 | 3 | 2 | 3 | 1 | 1 | 1 | G8XQX1|OXLA\_DABRR:Q4F867|OXLA\_DABSI:P0C2D7|OXLA\_VIPBB |  |  | PEAKS DB |
| LIDC(+57.02)YELGDDTEWFGGPQLR | 92.96 | 2383.0791 | 20 | 1.7 | 1192.5488 | 81.00 | 1 | F1:25406 | Fraction\_1\_23092022r.raw | 4.5875E8 | 7.0609E6 | 8.0799E5 | 5.4813E5 | 3.9879E5 | 3.8792E5 |  | 11 | 5 | 2 | 1 | 1 | 1 | 1 | 0 | TRINITY\_DN4276\_c0\_g1\_i1.p1 | Carbamidomethylation | C4:Carbamidomethylation:1000.00 | PEAKS DB |
| DKPTEDVVISNSGSETVTEPFGVSRDDATE | 92.69 | 3180.4583 | 30 | 0.6 | 1061.1606 | 56.92 | 5 | F5:15526 | Fraction\_5\_23092022.raw |  |  |  |  | 1.1402E6 | 1.4355E6 |  | 2 | 0 | 0 | 0 | 0 | 1 | 1 | 0 | TRINITY\_DN6873\_c0\_g1\_i3.p1:TRINITY\_DN6873\_c0\_g1\_i2.p1 |  |  | PEAKS DB |
| AETEC(+57.02)QNTEYQQLLDIK | 92.68 | 2081.9575 | 17 | 1.2 | 1041.9873 | 60.86 | 5 | F5:17273 | Fraction\_5\_23092022.raw | 6.127E6 | 8.616E5 | 9.6727E5 | 4.4686E5 | 5.0529E5 | 5.831E5 |  | 7 | 2 | 1 | 1 | 1 | 1 | 1 | 0 | P13645|K1C10\_HUMAN | Carbamidomethylation | C5:Carbamidomethylation:1000.00 | PEAKS DB |
| LYEPISGNYYPVTTK | 92.67 | 1743.8719 | 15 | -0.2 | 872.9431 | 57.03 | 3 | F3:15220 | Fraction\_3\_23092022.raw |  |  | 7.0406E6 |  | 1.724E5 | 5.2852E5 |  | 3 | 0 | 0 | 1 | 0 | 1 | 1 | 0 | TRINITY\_DN2373\_c0\_g1\_i19.p1 |  |  | PEAKS DB |
| DPNRTPFQWSYVR | 92.58 | 1664.8059 | 13 | -0.6 | 555.9423 | 57.76 | 1 | F1:14997 | Fraction\_1\_23092022r.raw | 2.4455E5 | 9.7444E5 |  |  |  |  |  | 2 | 1 | 1 | 0 | 0 | 0 | 0 | 0 | TRINITY\_DN1342\_c0\_g1\_i29.p1:TRINITY\_DN1342\_c0\_g1\_i9.p1 |  |  | PEAKS DB |
| NIKNAVVTIEINQNVATLR | 92.57 | 2109.1907 | 19 | 0.4 | 1055.6030 | 58.38 | 1 | F1:15269 | Fraction\_1\_23092022r.raw | 2.6064E8 | 6.0471E6 | 2.1288E5 | 4.4515E5 | 2.0673E5 | 9.6067E5 |  | 13 | 7 | 2 | 1 | 1 | 1 | 1 | 0 | TRINITY\_DN4276\_c0\_g1\_i1.p1 |  |  | PEAKS DB |
| DIVVVGAGM(+15.99)SGLSAAYVLAGAGHK | 92.41 | 2258.1729 | 24 | 1.3 | 753.7325 | 70.57 | 1 | F1:20752 | Fraction\_1\_23092022r.raw | 2.5963E5 |  | 3.9238E4 |  |  |  |  | 2 | 1 | 0 | 1 | 0 | 0 | 0 | 0 | B5U6Y8|OXLA\_ECHOC | Oxidation (M) | M9:Oxidation (M):1000.00 | PEAKS DB |
| NAVVTIEINQNVATLR | 92.35 | 1753.9686 | 16 | 0.5 | 877.9921 | 62.19 | 5 | F5:17872 | Fraction\_5\_23092022.raw | 3.2435E8 | 7.5827E6 | 8.6342E5 | 4.8287E5 | 2.4253E5 | 1.0314E6 |  | 12 | 7 | 1 | 1 | 1 | 1 | 1 | 0 | TRINITY\_DN4276\_c0\_g1\_i1.p1 |  |  | PEAKS DB |
| MSGEC(+57.02)APNVSVSVSTSHTTISGGGSR | 92.25 | 2564.1594 | 26 | 0.2 | 855.7273 | 40.30 | 1 | F1:7289 | Fraction\_1\_23092022r.raw | 3.5731E6 |  |  |  |  |  |  | 1 | 1 | 0 | 0 | 0 | 0 | 0 | 0 | P04264|K2C1\_HUMAN | Carbamidomethylation | C5:Carbamidomethylation:1000.00 | PEAKS DB |
| DFTYEETVDPAGC(+57.02)QAGPDRYYLK | 92.19 | 2694.1907 | 23 | 0.6 | 899.0714 | 58.58 | 3 | F3:15949 | Fraction\_3\_23092022.raw | 7.9891E6 | 4.2569E7 | 1.3343E6 | 3.9536E5 |  |  |  | 6 | 1 | 3 | 1 | 1 | 0 | 0 | 0 | TRINITY\_DN10047\_c0\_g1\_i1.p1 | Carbamidomethylation | C13:Carbamidomethylation:1000.00 | PEAKS DB |
| VLYDAEISQIHQSVTDTNVILSM(+15.99)DNSR | 91.68 | 3063.4819 | 27 | 1.4 | 1022.1693 | 68.11 | 1 | F1:19646 | Fraction\_1\_23092022r.raw | 9.5473E5 |  |  |  |  |  |  | 1 | 1 | 0 | 0 | 0 | 0 | 0 | 0 | P35908|K22E\_HUMAN | Oxidation (M) | M23:Oxidation (M):1000.00 | PEAKS DB |
| YTDLHADITPLILDRM(+15.99)NEC(+57.02)IK | 91.55 | 2546.2510 | 21 | 3.0 | 637.5719 | 59.53 | 1 | F1:15860 | Fraction\_1\_23092022r.raw | 2.057E8 | 1.3968E6 |  |  |  |  |  | 14 | 12 | 2 | 0 | 0 | 0 | 0 | 0 | TRINITY\_DN4276\_c0\_g1\_i1.p1 | Oxidation (M); Carbamidomethylation | M16:Oxidation (M):1000.00;C19:Carbamidomethylation:1000.00 | PEAKS DB |
| QFTVLTGTAPLPQMFTLGYHQSR | 91.54 | 2592.3159 | 23 | 1.7 | 865.1141 | 80.04 | 1 | F1:24992 | Fraction\_1\_23092022r.raw | 4.1699E5 |  |  |  |  |  |  | 1 | 1 | 0 | 0 | 0 | 0 | 0 | 0 | TRINITY\_DN1709\_c0\_g1\_i4.p1:TRINITY\_DN1709\_c0\_g1\_i5.p1:TRINITY\_DN1709\_c0\_g1\_i6.p1:TRINITY\_DN1709\_c0\_g1\_i8.p1 |  |  | PEAKS DB |
| DISTINRAQLIDDALNLAR | 91.27 | 2111.1335 | 19 | -0.3 | 704.7183 | 79.44 | 5 | F5:25581 | Fraction\_5\_23092022.raw |  |  |  | 2.0669E5 | 4.0594E5 | 2.772E6 |  | 3 | 0 | 0 | 0 | 1 | 1 | 1 | 0 | TRINITY\_DN1113\_c0\_g1\_i95.p1:TRINITY\_DN1113\_c0\_g1\_i47.p1:TRINITY\_DN1113\_c0\_g1\_i59.p1:TRINITY\_DN1113\_c0\_g1\_i53.p1:TRINITY\_DN1113\_c0\_g1\_i73.p1:TRINITY\_DN1113\_c0\_g1\_i6.p1:TRINITY\_DN1113\_c0\_g1\_i12.p1 |  |  | PEAKS DB |
| ALEESNYELEGK | 91.02 | 1380.6409 | 12 | 0.1 | 691.3278 | 37.46 | 5 | F5:6790 | Fraction\_5\_23092022.raw | 7.8781E5 | 1.0987E5 | 3.4777E6 | 1.0546E6 | 7.6134E5 | 1.1081E6 | 8.9163E5 | 8 | 1 | 1 | 2 | 1 | 1 | 1 | 1 | P13645|K1C10\_HUMAN |  |  | PEAKS DB |
| KTPIIESGSLESILVTEK | 91.00 | 1943.0826 | 18 | 0.6 | 648.7019 | 63.38 | 2 | F2:17714 | Fraction\_2\_23092022.raw | 2.5309E6 | 2.6725E6 | 2.1154E5 |  |  |  |  | 5 | 2 | 2 | 1 | 0 | 0 | 0 | 0 | TRINITY\_DN10047\_c0\_g1\_i1.p1 |  |  | PEAKS DB |
| IAPEESPVLLTEAPLNPK | 90.85 | 1917.0458 | 18 | 1.2 | 959.5313 | 66.09 | 3 | F3:19284 | Fraction\_3\_23092022.raw |  |  | 6.6192E5 |  |  |  |  | 1 | 0 | 0 | 1 | 0 | 0 | 0 | 0 | P86700|ACT\_CHIOP |  |  | PEAKS DB |
| SGIQVRLGEDNINVVEGNEQFISASK | 90.47 | 2802.4148 | 26 | 1.5 | 935.1470 | 63.71 | 5 | F5:18538 | Fraction\_5\_23092022.raw | 1.2244E5 | 2.4081E5 | 4.3185E4 | 1.5418E5 | 2.0903E5 | 1.9088E5 |  | 6 | 1 | 1 | 1 | 1 | 1 | 1 | 0 | P00760|TRY1\_BOVIN |  |  | PEAKS DB |
| YDISYPEKGEQVIKVKR | 90.36 | 2051.1050 | 17 | 0.0 | 513.7835 | 37.04 | 7 | F7:9380 | Fraction\_7\_23092022.raw |  |  | 4.002E5 |  |  | 2.7909E5 | 3.0374E6 | 4 | 0 | 0 | 1 | 0 | 0 | 1 | 2 | TRINITY\_DN33420\_c0\_g1\_i1.p1 |  |  | PEAKS DB |
| NVNNLKINKEVSIDLVR | 90.06 | 1967.1163 | 17 | 0.6 | 656.7131 | 48.02 | 5 | F5:11499 | Fraction\_5\_23092022.raw |  |  | 4.9647E5 | 3.6441E6 | 1.812E7 | 1.0791E8 | 8.881E6 | 13 | 0 | 0 | 2 | 2 | 2 | 5 | 2 | TRINITY\_DN2228\_c0\_g4\_i1.p1 |  |  | PEAKS DB |
| AGKYDISYPEKGEQVIK | 89.98 | 1923.9941 | 17 | 2.3 | 482.0069 | 36.48 | 7 | F7:9116 | Fraction\_7\_23092022.raw | 3.5912E5 | 5.0562E5 | 3.0452E7 | 2.5865E6 | 7.4445E6 | 1.5414E7 | 8.8141E7 | 15 | 2 | 1 | 2 | 2 | 2 | 3 | 3 | TRINITY\_DN33420\_c0\_g1\_i1.p1 |  |  | PEAKS DB |
| SFKDSNGDGIGDLNGITSK | 89.83 | 1923.9174 | 19 | 1.9 | 962.9678 | 46.02 | 2 | F2:9900 | Fraction\_2\_23092022.raw | 1.569E6 | 2.7587E6 |  |  |  |  |  | 4 | 2 | 2 | 0 | 0 | 0 | 0 | 0 | TRINITY\_DN10047\_c0\_g1\_i1.p1 |  |  | PEAKS DB |
| VFDEFKPLVEEPQNLIK | 89.69 | 2044.0880 | 17 | -0.1 | 682.3699 | 71.98 | 1 | F1:21393 | Fraction\_1\_23092022r.raw | 1.006E6 |  |  |  |  |  |  | 1 | 1 | 0 | 0 | 0 | 0 | 0 | 0 | P02768|ALBU\_HUMAN |  |  | PEAKS DB |
| ILTATVDNANVLLQIDNAR | 89.65 | 2053.1167 | 19 | 0.5 | 1027.5662 | 72.98 | 1 | F1:21821 | Fraction\_1\_23092022r.raw | 1.0482E6 |  | 8.4193E4 |  |  |  |  | 3 | 2 | 0 | 1 | 0 | 0 | 0 | 0 | P02533|K1C14\_HUMAN |  |  | PEAKS DB |
| NEDVIHTVIRLSTNSDVINVSR | 89.48 | 2480.2983 | 22 | 0.1 | 621.0819 | 69.63 | 1 | F1:20333 | Fraction\_1\_23092022r.raw | 2.9184E6 |  |  |  |  |  |  | 2 | 2 | 0 | 0 | 0 | 0 | 0 | 0 | TRINITY\_DN4276\_c0\_g1\_i1.p1 |  |  | PEAKS DB |
| SFESIGPILPNLEK | 89.39 | 1542.8293 | 14 | 0.7 | 772.4225 | 72.57 | 3 | F3:22184 | Fraction\_3\_23092022.raw |  |  | 3.1013E6 | 9.1105E4 | 1.6627E5 | 1.134E6 | 6.6382E5 | 5 | 0 | 0 | 1 | 1 | 1 | 1 | 1 | TRINITY\_DN2938\_c0\_g1\_i1.p1 |  |  | PEAKS DB |
| ISIGGGSC(+57.02)AISGGYGSR | 89.23 | 1597.7518 | 17 | 0.1 | 799.8833 | 45.05 | 2 | F2:9467 | Fraction\_2\_23092022.raw | 3.4544E5 | 3.97E5 | 2.1039E5 |  |  | 3.439E5 | 1.3764E5 | 5 | 1 | 1 | 1 | 0 | 0 | 1 | 1 | P48668|K2C6C\_HUMAN | Carbamidomethylation | C8:Carbamidomethylation:1000.00 | PEAKS DB |
| GTLYFEQTNESQPVQVTGEVSGLKK | 89.15 | 2738.3762 | 25 | 0.5 | 913.7998 | 54.51 | 6 | F6:15080 | Fraction\_6\_23092022.raw |  |  |  |  |  | 6.2755E5 |  | 1 | 0 | 0 | 0 | 0 | 0 | 1 | 0 | TRINITY\_DN1931\_c0\_g1\_i1.p1:TRINITY\_DN1931\_c0\_g1\_i2.p1:TRINITY\_DN1931\_c0\_g1\_i4.p1 |  |  | PEAKS DB |
| LGKDAVEDLESVGK | 89.03 | 1458.7566 | 14 | 0.6 | 730.3860 | 46.58 | 4 | F4:11107 | Fraction\_4\_23092022.raw | 2.7311E6 | 9.7168E5 | 8.7419E5 | 1.3943E6 | 1.133E6 | 8.6708E5 | 1.1205E6 | 13 | 2 | 2 | 2 | 2 | 2 | 2 | 1 | P81605|DCD\_HUMAN |  |  | PEAKS DB |
| NVAAGSPAVITGWGYVSNSSPAFSQTLR | 88.99 | 2836.4143 | 28 | 0.7 | 946.4794 | 76.41 | 5 | F5:24238 | Fraction\_5\_23092022.raw |  |  |  |  | 8.9582E4 | 1.5861E5 |  | 2 | 0 | 0 | 0 | 0 | 1 | 1 | 0 | TRINITY\_DN7688\_c0\_g2\_i1.p1:TRINITY\_DN7688\_c0\_g2\_i2.p1 |  |  | PEAKS DB |
| AAFGGSGGRGSSSGGGYSSGSSSYGSGGR | 88.86 | 2500.0598 | 29 | 0.8 | 834.3612 | 24.36 | 7 | F7:3967 | Fraction\_7\_23092022.raw |  |  |  |  |  |  | 2.3221E5 | 1 | 0 | 0 | 0 | 0 | 0 | 0 | 1 | P35908|K22E\_HUMAN |  |  | PEAKS DB |
| AWADEVKDYQYGSPNKGR | 88.72 | 2082.9758 | 18 | 0.6 | 695.3329 | 40.36 | 7 | F7:10894 | Fraction\_7\_23092022.raw |  |  |  | 7.9142E5 |  | 2.9073E6 | 3.9955E6 | 6 | 0 | 0 | 0 | 2 | 0 | 2 | 2 | TRINITY\_DN6370\_c0\_g1\_i1.p1 |  |  | PEAKS DB |
| IIAVDQDPLGIQGR | 88.70 | 1493.8202 | 14 | 1.2 | 747.9183 | 59.22 | 2 | F2:15828 | Fraction\_2\_23092022.raw | 1.0165E6 | 6.4892E7 | 2.4161E5 | 2.8049E5 | 8.3802E5 | 1.3147E7 |  | 6 | 1 | 1 | 1 | 1 | 1 | 1 | 0 | TRINITY\_DN2611\_c0\_g1\_i12.p1:TRINITY\_DN2611\_c0\_g1\_i23.p1:TRINITY\_DN2611\_c0\_g1\_i19.p1:TRINITY\_DN2611\_c0\_g1\_i9.p1 |  |  | PEAKS DB |
| SSDSTATDRDYTYLDHIYTKDDPRTYELVK | 88.46 | 3567.6641 | 30 | 0.6 | 892.9238 | 53.26 | 2 | F2:13133 | Fraction\_2\_23092022.raw | 4.6338E6 | 1.4147E8 |  | 1.1258E6 | 3.1845E6 |  |  | 7 | 1 | 4 | 0 | 1 | 1 | 0 | 0 | TRINITY\_DN1342\_c0\_g1\_i23.p1:TRINITY\_DN1342\_c0\_g1\_i54.p1 |  |  | PEAKS DB |
| TGSFYVPVESKAPYC(+57.02)NNKGK | 88.43 | 2245.0837 | 20 | -0.5 | 749.3682 | 38.45 | 4 | F4:7505 | Fraction\_4\_23092022.raw |  | 2.2402E6 | 9.7062E6 | 4.9178E7 | 2.0417E6 | 5.5689E6 | 1.0137E7 | 15 | 0 | 1 | 3 | 4 | 2 | 2 | 3 | A0A0M3KKW3|PA1\_VESBA:C0HLL3|PA1\_VESVE | Carbamidomethylation | C15:Carbamidomethylation:1000.00 | PEAKS DB |
| HEC(+57.02)C(+57.02)LIGVPQSKNPQPVSK | 88.10 | 2177.0720 | 19 | -0.3 | 726.6978 | 31.57 | 7 | F7:6948 | Fraction\_7\_23092022.raw |  |  | 2.8577E5 | 1.4782E6 | 1.272E6 | 5.2592E5 | 6.9825E5 | 7 | 0 | 0 | 1 | 1 | 2 | 1 | 2 | C0HLL3|PA1\_VESVE | Carbamidomethylation | C3:Carbamidomethylation:1000.00;C4:Carbamidomethylation:1000.00 | PEAKS DB |
| NQC(+57.02)VC(+57.02)VGLNAKTYPK | 88.08 | 1750.8495 | 15 | 0.1 | 876.4321 | 34.46 | 5 | F5:5732 | Fraction\_5\_23092022.raw |  |  | 1.6286E6 | 1.1018E7 | 2.5915E7 | 2.0589E6 | 1.0177E7 | 8 | 0 | 0 | 1 | 2 | 2 | 1 | 2 |  | Carbamidomethylation | C3:Carbamidomethylation:1000.00;C5:Carbamidomethylation:1000.00 | PEAKS DB |
| AQWDFTVTADTAILKPQVK | 88.03 | 2131.1313 | 19 | 0.7 | 711.3849 | 67.55 | 6 | F6:20959 | Fraction\_6\_23092022.raw |  |  |  |  |  | 2.5047E5 |  | 1 | 0 | 0 | 0 | 0 | 0 | 1 | 0 | TRINITY\_DN1697\_c0\_g1\_i11.p1:TRINITY\_DN1697\_c0\_g1\_i18.p1 |  |  | PEAKS DB |
| LAYYSYAASNTR | 87.89 | 1378.6517 | 12 | -0.2 | 690.3330 | 40.38 | 7 | F7:10898 | Fraction\_7\_23092022.raw | 4.8637E5 | 1.8829E5 | 3.893E6 | 2.5854E7 | 1.4027E6 | 8.5362E5 | 6.9724E5 | 8 | 1 | 1 | 1 | 2 | 1 | 1 | 1 |  |  |  | PEAKS DB |
| YVLYLQESGFDVIKR | 87.66 | 1828.9723 | 15 | 0.8 | 610.6652 | 65.52 | 6 | F6:20037 | Fraction\_6\_23092022.raw |  |  |  |  |  | 1.3637E6 |  | 2 | 0 | 0 | 0 | 0 | 0 | 2 | 0 | TRINITY\_DN13586\_c0\_g2\_i1.p1 |  |  | PEAKS DB |
| GQVGGDVNVEM(+15.99)DAAPGVDLSR | 87.65 | 2100.9746 | 21 | 0.5 | 1051.4951 | 50.17 | 1 | F1:11644 | Fraction\_1\_23092022r.raw | 4.1512E5 |  |  |  |  |  |  | 1 | 1 | 0 | 0 | 0 | 0 | 0 | 0 | P02533|K1C14\_HUMAN | Oxidation (M) | M11:Oxidation (M):1000.00 | PEAKS DB |
| LGEYGFQNALIVR | 87.65 | 1478.7881 | 13 | -0.4 | 740.4010 | 66.86 | 1 | F1:19104 | Fraction\_1\_23092022r.raw | 4.7265E5 | 1.4217E5 |  |  |  |  |  | 2 | 1 | 1 | 0 | 0 | 0 | 0 | 0 | P02769|ALBU\_BOVIN |  |  | PEAKS DB |
| LGEYGFQNALLVR | 87.65 | 1478.7881 | 13 | -0.4 | 740.4010 | 66.86 | 1 | F1:19104 | Fraction\_1\_23092022r.raw | 4.7265E5 | 1.4217E5 |  |  |  |  |  | 2 | 1 | 1 | 0 | 0 | 0 | 0 | 0 |  |  |  | PEAKS DB |
| LGEYGFQNAILVR | 87.65 | 1478.7881 | 13 | -0.4 | 740.4010 | 66.86 | 1 | F1:19104 | Fraction\_1\_23092022r.raw | 4.7265E5 | 1.4217E5 |  |  |  |  |  | 2 | 1 | 1 | 0 | 0 | 0 | 0 | 0 |  |  |  | PEAKS DB |
| LAYYSYAASNTRLVGNYIATVTK | 87.61 | 2538.3118 | 23 | 2.0 | 847.1129 | 69.74 | 4 | F4:21451 | Fraction\_4\_23092022.raw |  |  | 3.181E5 | 3.2117E7 | 2.8293E6 |  |  | 4 | 0 | 0 | 1 | 2 | 1 | 0 | 0 |  |  |  | PEAKS DB |
| TTETSGILLNGPVFFR | 87.55 | 1750.9253 | 16 | -0.1 | 876.4698 | 78.54 | 2 | F2:24484 | Fraction\_2\_23092022.raw | 6.1885E6 | 5.256E6 | 7.7536E5 | 6.3138E5 |  | 6.6E5 |  | 6 | 1 | 2 | 1 | 1 | 0 | 1 | 0 | TRINITY\_DN11986\_c0\_g1\_i3.p1 |  |  | PEAKS DB |
| SSPIQM(+15.99)DLTPGVTEVQDIR | 87.41 | 2101.0361 | 19 | 0.4 | 1051.5258 | 60.06 | 5 | F5:16927 | Fraction\_5\_23092022.raw |  |  |  |  | 6.4546E5 |  | 8.7982E5 | 2 | 0 | 0 | 0 | 0 | 1 | 0 | 1 | TRINITY\_DN3450\_c0\_g1\_i51.p1 | Oxidation (M) | M6:Oxidation (M):1000.00 | PEAKS DB |
| LTALGVLGNINDPEYSAR | 87.23 | 1901.9846 | 18 | 0.4 | 952.0000 | 71.30 | 2 | F2:21275 | Fraction\_2\_23092022.raw |  | 1.9919E5 | 2.7094E5 | 6.0627E4 | 1.1387E5 |  |  | 4 | 0 | 1 | 1 | 1 | 1 | 0 | 0 | TRINITY\_DN1455\_c0\_g1\_i1.p1 |  |  | PEAKS DB |
| LGIFNSEGNINEEVLR | 87.15 | 1802.9163 | 16 | 0.2 | 902.4656 | 67.92 | 7 | F7:25933 | Fraction\_7\_23092022.raw |  |  |  |  |  |  | 8.5033E5 | 1 | 0 | 0 | 0 | 0 | 0 | 0 | 1 | TRINITY\_DN36822\_c0\_g1\_i1.p1 |  |  | PEAKS DB |
| KYTDLHADITPLILDR | 87.11 | 1883.0153 | 16 | 0.5 | 628.6793 | 59.02 | 1 | F1:15556 | Fraction\_1\_23092022r.raw | 3.9042E7 |  |  |  |  |  |  | 3 | 3 | 0 | 0 | 0 | 0 | 0 | 0 | TRINITY\_DN4276\_c0\_g1\_i1.p1 |  |  | PEAKS DB |
| YDISYPEKGEQVIK | 87.06 | 1667.8406 | 14 | 0.4 | 556.9543 | 42.93 | 7 | F7:12070 | Fraction\_7\_23092022.raw |  |  | 7.2549E6 |  | 5.9338E5 | 1.2977E6 | 3.2744E6 | 8 | 0 | 0 | 2 | 0 | 2 | 2 | 2 | TRINITY\_DN33420\_c0\_g1\_i1.p1 |  |  | PEAKS DB |
| GQYYLHQFVAGQPDLNYRNK | 87.05 | 2410.1819 | 20 | 0.8 | 603.5532 | 49.47 | 2 | F2:11448 | Fraction\_2\_23092022.raw |  | 1.488E6 |  |  |  |  |  | 2 | 0 | 2 | 0 | 0 | 0 | 0 | 0 | TRINITY\_DN10047\_c0\_g1\_i1.p1 |  |  | PEAKS DB |
| KYTDLHADITPLILDRMNEC(+57.02)IK | 87.01 | 2658.3508 | 22 | -0.1 | 532.6774 | 63.40 | 1 | F1:17525 | Fraction\_1\_23092022r.raw | 1.251E7 |  |  |  |  |  |  | 3 | 3 | 0 | 0 | 0 | 0 | 0 | 0 | TRINITY\_DN4276\_c0\_g1\_i1.p1 | Carbamidomethylation | C20:Carbamidomethylation:1000.00 | PEAKS DB |
| SGGGFSSGSAGIINYQRR | 86.83 | 1812.8867 | 18 | -0.1 | 605.3028 | 39.49 | 7 | F7:10504 | Fraction\_7\_23092022.raw | 6.4703E5 | 3.1744E6 | 1.0298E5 | 6.2905E4 | 2.1742E5 | 5.9953E5 | 6.5816E5 | 8 | 1 | 2 | 1 | 1 | 1 | 1 | 1 | P04264|K2C1\_HUMAN |  |  | PEAKS DB |
| TLVVHADPDDLGKGGHELSKTTGNAGGR | 86.66 | 2801.4055 | 28 | -0.4 | 561.2881 | 33.04 | 6 | F6:5615 | Fraction\_6\_23092022.raw |  |  |  |  |  | 1.1091E6 |  | 3 | 0 | 0 | 0 | 0 | 0 | 3 | 0 | TRINITY\_DN1931\_c0\_g1\_i1.p1:TRINITY\_DN1931\_c0\_g1\_i2.p1:TRINITY\_DN1931\_c0\_g1\_i4.p1 |  |  | PEAKS DB |
| YSTYINSEDEFYTDSNGR | 86.54 | 2159.8918 | 18 | 0.9 | 1080.9542 | 53.51 | 3 | F3:13680 | Fraction\_3\_23092022.raw |  |  | 1.1769E5 |  |  |  |  | 1 | 0 | 0 | 1 | 0 | 0 | 0 | 0 | TRINITY\_DN2373\_c0\_g1\_i19.p1 |  |  | PEAKS DB |
| M(+15.99)VELNAGEPIC(+57.02)SIYNDRGIIQK | 86.45 | 2535.2461 | 22 | 0.8 | 846.0900 | 60.03 | 5 | F5:16923 | Fraction\_5\_23092022.raw |  |  |  |  | 8.2902E5 | 1.4595E6 |  | 2 | 0 | 0 | 0 | 0 | 1 | 1 | 0 | TRINITY\_DN838\_c0\_g3\_i1.p1:TRINITY\_DN838\_c0\_g3\_i3.p1:TRINITY\_DN838\_c0\_g3\_i2.p1 | Oxidation (M); Carbamidomethylation | M1:Oxidation (M):1000.00;C11:Carbamidomethylation:1000.00 | PEAKS DB |
| TFVDLSQKDDPEITLSK | 86.40 | 1934.9836 | 17 | 0.2 | 646.0020 | 55.15 | 2 | F2:14017 | Fraction\_2\_23092022.raw |  | 7.291E5 |  |  |  |  |  | 1 | 0 | 1 | 0 | 0 | 0 | 0 | 0 | TRINITY\_DN10256\_c0\_g10\_i1.p1:TRINITY\_DN10256\_c0\_g5\_i4.p1 |  |  | PEAKS DB |
| QISYITGVATSIEGIAR | 86.40 | 1777.9574 | 17 | 0.6 | 889.9865 | 75.00 | 4 | F4:23825 | Fraction\_4\_23092022.raw |  |  | 2.0272E5 | 1.5358E6 | 3.4602E5 |  |  | 3 | 0 | 0 | 1 | 1 | 1 | 0 | 0 | TRINITY\_DN28260\_c0\_g1\_i1.p1:TRINITY\_DN28260\_c0\_g1\_i2.p1:TRINITY\_DN28260\_c0\_g1\_i3.p1 |  |  | PEAKS DB |
| SIVHPSYNSNTLNNDIMLIKLK | 86.37 | 2513.3311 | 22 | -0.7 | 838.7837 | 61.82 | 1 | F1:16814 | Fraction\_1\_23092022r.raw | 6.9245E5 |  |  |  |  |  |  | 1 | 1 | 0 | 0 | 0 | 0 | 0 | 0 | P00760|TRY1\_BOVIN |  |  | PEAKS DB |
| AVKYFTEC(+57.02)IRHEC(+57.02)C(+57.02)LIGVPQSKNPQPVSK | 86.35 | 3444.7104 | 29 | 0.2 | 862.1851 | 44.03 | 7 | F7:12579 | Fraction\_7\_23092022.raw |  |  |  | 3.7863E5 |  |  | 4.7494E6 | 3 | 0 | 0 | 0 | 1 | 0 | 0 | 2 |  | Carbamidomethylation | C8:Carbamidomethylation:1000.00;C13:Carbamidomethylation:1000.00;C14:Carbamidomethylation:1000.00 | PEAKS DB |
| DC(+57.02)SQRIC(+57.02)ETDAHYVQIIHTSNNLGTER | 86.26 | 3216.4675 | 27 | 0.3 | 805.1244 | 45.52 | 5 | F5:10410 | Fraction\_5\_23092022.raw |  |  | 7.48E5 |  | 9.5662E5 |  |  | 2 | 0 | 0 | 1 | 0 | 1 | 0 | 0 | TRINITY\_DN2326\_c0\_g1\_i12.p1 | Carbamidomethylation | C2:Carbamidomethylation:1000.00;C7:Carbamidomethylation:1000.00 | PEAKS DB |
| VQALEEANNDLENK | 86.18 | 1585.7583 | 14 | 0.4 | 793.8868 | 38.18 | 7 | F7:9888 | Fraction\_7\_23092022.raw | 1.0998E6 | 1.2058E6 | 1.9072E6 | 2.8175E5 | 2.6402E5 | 3.2816E5 | 2.8373E5 | 10 | 2 | 2 | 2 | 1 | 1 | 1 | 1 | P35527|K1C9\_HUMAN |  |  | PEAKS DB |
| YGHDDC(+57.02)RNTAKYLVGQNIAK | 86.11 | 2322.1174 | 20 | 0.6 | 581.5370 | 32.01 | 7 | F7:7134 | Fraction\_7\_23092022.raw |  |  |  |  |  |  | 3.5541E6 | 3 | 0 | 0 | 0 | 0 | 0 | 0 | 3 | TRINITY\_DN6370\_c0\_g1\_i1.p1 | Carbamidomethylation | C6:Carbamidomethylation:1000.00 | PEAKS DB |
| TGSFYVPVESK | 86.10 | 1212.6027 | 11 | 1.0 | 607.3092 | 47.23 | 4 | F4:11441 | Fraction\_4\_23092022.raw | 1.7405E6 | 3.3208E6 | 1.4279E8 | 1.6292E8 | 3.0697E6 | 1.7065E7 | 5.4182E6 | 10 | 1 | 1 | 2 | 2 | 1 | 2 | 1 | A0A0M3KKW3|PA1\_VESBA:C0HLL3|PA1\_VESVE |  |  | PEAKS DB |
| DSDAKLIDC(+57.02)YELGDDTEWFGGPQLR | 85.96 | 2899.2969 | 25 | 0.7 | 967.4402 | 78.81 | 1 | F1:24487 | Fraction\_1\_23092022r.raw | 1.2279E7 | 2.2383E5 |  |  |  |  |  | 2 | 1 | 1 | 0 | 0 | 0 | 0 | 0 | TRINITY\_DN4276\_c0\_g1\_i1.p1 | Carbamidomethylation | C9:Carbamidomethylation:1000.00 | PEAKS DB |
| DPLFLDQNNYM(+15.99)DKYIC(+57.02)LVAK | 85.90 | 2475.1814 | 20 | 0.6 | 826.0682 | 72.05 | 1 | F1:21402 | Fraction\_1\_23092022r.raw | 2.7959E6 |  |  |  |  |  |  | 1 | 1 | 0 | 0 | 0 | 0 | 0 | 0 | TRINITY\_DN4276\_c0\_g1\_i1.p1 | Oxidation (M); Carbamidomethylation | M11:Oxidation (M):1000.00;C16:Carbamidomethylation:1000.00 | PEAKS DB |
| AVEYLTEC(+57.02)IRHEC(+57.02)C(+57.02)LIGVPQSKNPQPVSK | 85.83 | 3411.6736 | 29 | -0.2 | 853.9255 | 53.01 | 3 | F3:13434 | Fraction\_3\_23092022.raw |  |  | 3.3583E6 | 1.7314E5 | 3.6709E5 | 2.2147E6 | 9.0429E6 | 8 | 0 | 0 | 2 | 1 | 1 | 2 | 2 |  | Carbamidomethylation | C8:Carbamidomethylation:1000.00;C13:Carbamidomethylation:1000.00;C14:Carbamidomethylation:1000.00 | PEAKS DB |
| SGGGFSSGSAGIINYQR | 85.81 | 1656.7855 | 17 | -0.1 | 829.4000 | 47.84 | 1 | F1:10583 | Fraction\_1\_23092022r.raw | 2.6904E6 | 1.9667E6 | 5.2027E5 |  | 2.7457E5 | 1.0091E6 | 3.7751E5 | 6 | 1 | 1 | 1 | 0 | 1 | 1 | 1 | P04264|K2C1\_HUMAN |  |  | PEAKS DB |
| ENNNSPDLC(+57.02)TSQIYC(+57.02)QGNLLK | 85.52 | 2467.1108 | 21 | 1.5 | 823.3788 | 58.53 | 2 | F2:15508 | Fraction\_2\_23092022.raw |  | 3.2098E7 | 4.4193E5 | 7.2858E5 | 5.167E5 |  |  | 7 | 0 | 2 | 1 | 2 | 2 | 0 | 0 | TRINITY\_DN10256\_c0\_g10\_i1.p1:TRINITY\_DN10256\_c0\_g5\_i4.p1 | Carbamidomethylation | C9:Carbamidomethylation:1000.00;C15:Carbamidomethylation:1000.00 | PEAKS DB |
| SIVHPSYNSNTLNNDIM(+15.99)LIKLK | 85.48 | 2529.3262 | 22 | 0.8 | 633.3394 | 58.89 | 1 | F1:15517 | Fraction\_1\_23092022r.raw | 6.2078E5 |  |  |  |  | 9.6013E4 |  | 2 | 1 | 0 | 0 | 0 | 0 | 1 | 0 | P00760|TRY1\_BOVIN | Oxidation (M) | M17:Oxidation (M):1000.00 | PEAKS DB |
| C(+57.02)NTDC(+57.02)KNDPDNC(+57.02)ISDQLFR | 85.42 | 2370.9626 | 19 | -1.1 | 791.3273 | 49.58 | 2 | F2:11492 | Fraction\_2\_23092022.raw |  | 4.6319E6 |  |  |  |  |  | 1 | 0 | 1 | 0 | 0 | 0 | 0 | 0 | TRINITY\_DN2611\_c0\_g1\_i12.p1:TRINITY\_DN2611\_c0\_g1\_i23.p1:TRINITY\_DN2611\_c0\_g1\_i19.p1:TRINITY\_DN2611\_c0\_g1\_i9.p1 | Carbamidomethylation | C1:Carbamidomethylation:1000.00;C5:Carbamidomethylation:1000.00;C12:Carbamidomethylation:1000.00 | PEAKS DB |
| ENKKHDFYTLNTIKNHNEFK | 85.36 | 2519.2556 | 20 | 0.8 | 504.8588 | 30.46 | 7 | F7:6449 | Fraction\_7\_23092022.raw |  |  |  |  |  |  | 1.5211E7 | 3 | 0 | 0 | 0 | 0 | 0 | 0 | 3 |  |  |  | PEAKS DB |
| IAEHYPYFTGGVIALNK | 85.35 | 1891.9832 | 17 | -0.5 | 631.6680 | 59.95 | 6 | F6:17535 | Fraction\_6\_23092022.raw |  |  |  |  |  | 1.7409E6 |  | 1 | 0 | 0 | 0 | 0 | 0 | 1 | 0 | TRINITY\_DN5723\_c0\_g1\_i5.p1:TRINITY\_DN5723\_c0\_g1\_i7.p1 |  |  | PEAKS DB |
| M(+15.99)SGEC(+57.02)APNVSVSVSTSHTTISGGGSR | 85.15 | 2580.1545 | 26 | 0.8 | 861.0594 | 38.02 | 1 | F1:6371 | Fraction\_1\_23092022r.raw | 1.0032E7 |  | 1.87E5 |  |  | 3.3246E5 | 7.0092E4 | 4 | 1 | 0 | 1 | 0 | 0 | 1 | 1 | P04264|K2C1\_HUMAN | Oxidation (M); Carbamidomethylation | M1:Oxidation (M):1000.00;C5:Carbamidomethylation:1000.00 | PEAKS DB |
| FGGGSGFGGGGFGGGSFGGGR | 85.13 | 1734.7498 | 21 | -0.6 | 868.3817 | 56.68 | 7 | F7:19468 | Fraction\_7\_23092022.raw |  |  |  | 9.4701E4 |  |  | 5.2227E5 | 2 | 0 | 0 | 0 | 1 | 0 | 0 | 1 | Q3TTY5|K22E\_MOUSE |  |  | PEAKS DB |
| ELLFHAIDLPGLGYKR | 84.99 | 1841.0199 | 16 | 0.6 | 614.6810 | 69.01 | 2 | F2:20234 | Fraction\_2\_23092022.raw |  | 2.6422E6 |  |  |  |  | 1.3445E7 | 4 | 0 | 2 | 0 | 0 | 0 | 0 | 2 | TRINITY\_DN2373\_c0\_g1\_i19.p1 |  |  | PEAKS DB |
| DSC(+57.02)QGDSGGPVVC(+57.02)SGK | 84.90 | 1608.6508 | 16 | 0.5 | 805.3331 | 22.93 | 5 | F5:3388 | Fraction\_5\_23092022.raw | 1.9478E6 |  |  |  | 2.0258E5 |  |  | 2 | 1 | 0 | 0 | 0 | 1 | 0 | 0 | P00760|TRY1\_BOVIN | Carbamidomethylation | C3:Carbamidomethylation:1000.00;C13:Carbamidomethylation:1000.00 | PEAKS DB |
| ASGLTKQENLEILKQHNEFRQK | 84.56 | 2610.3877 | 22 | 3.0 | 523.0864 | 35.85 | 7 | F7:8886 | Fraction\_7\_23092022.raw |  |  |  |  |  | 4.5984E5 | 1.4646E8 | 3 | 0 | 0 | 0 | 0 | 0 | 1 | 2 | P35782|VA52\_VESCR:P35781|VA51\_VESCR |  |  | PEAKS DB |
| LIGHSLGAHTSGFAGKKVQELR | 84.47 | 2305.2654 | 22 | 0.8 | 577.3241 | 29.66 | 7 | F7:6120 | Fraction\_7\_23092022.raw |  |  |  |  |  |  | 1.125E7 | 4 | 0 | 0 | 0 | 0 | 0 | 0 | 4 |  |  |  | PEAKS DB |
| YYQYGAHIPFNFK | 84.11 | 1646.7881 | 13 | 0.1 | 824.4014 | 58.06 | 2 | F2:15314 | Fraction\_2\_23092022.raw | 1.0775E6 | 9.8453E6 | 4.1487E5 | 1.2391E5 | 1.2335E5 |  |  | 6 | 1 | 2 | 1 | 1 | 1 | 0 | 0 | TRINITY\_DN1342\_c0\_g1\_i29.p1 |  |  | PEAKS DB |
| C(+57.02)IHSIC(+57.02)NYSPIIR | 83.94 | 1631.7913 | 13 | 1.0 | 544.9382 | 45.07 | 5 | F5:10182 | Fraction\_5\_23092022.raw |  |  |  |  | 7.2723E5 | 7.0208E5 | 1.8558E6 | 3 | 0 | 0 | 0 | 0 | 1 | 1 | 1 | TRINITY\_DN838\_c0\_g3\_i1.p1:TRINITY\_DN838\_c0\_g3\_i3.p1:TRINITY\_DN838\_c0\_g3\_i2.p1 | Carbamidomethylation | C1:Carbamidomethylation:1000.00;C6:Carbamidomethylation:1000.00 | PEAKS DB |
| QEIEC(+57.02)QNQEYSLLLSIK | 83.87 | 2094.0303 | 17 | 0.5 | 1048.0229 | 71.72 | 1 | F1:21237 | Fraction\_1\_23092022r.raw | 2.5842E6 |  |  |  |  |  |  | 2 | 2 | 0 | 0 | 0 | 0 | 0 | 0 | P35527|K1C9\_HUMAN | Carbamidomethylation | C5:Carbamidomethylation:1000.00 | PEAKS DB |
| YVM(+15.99)GNNPADLLAVDSR | 83.76 | 1749.8356 | 16 | -0.2 | 875.9249 | 59.21 | 1 | F1:15652 | Fraction\_1\_23092022r.raw | 4.7542E5 |  |  |  |  |  |  | 1 | 1 | 0 | 0 | 0 | 0 | 0 | 0 | Q02413|DSG1\_HUMAN | Oxidation (M) | M3:Oxidation (M):1000.00 | PEAKS DB |
| NC(+57.02)EPAYSTALNNSYFVK | 83.72 | 1976.8938 | 17 | -0.4 | 989.4537 | 58.66 | 1 | F1:15393 | Fraction\_1\_23092022r.raw | 8.6203E6 |  |  |  |  |  |  | 2 | 2 | 0 | 0 | 0 | 0 | 0 | 0 | TRINITY\_DN4276\_c0\_g1\_i1.p1 | Carbamidomethylation | C2:Carbamidomethylation:1000.00 | PEAKS DB |
| WFIGPDGKESNNLIDINTK | 83.69 | 2160.0850 | 19 | 2.0 | 721.0370 | 65.13 | 2 | F2:18560 | Fraction\_2\_23092022.raw |  | 4.2328E6 |  |  |  | 3.8644E5 |  | 2 | 0 | 1 | 0 | 0 | 0 | 1 | 0 | TRINITY\_DN10256\_c0\_g10\_i1.p1:TRINITY\_DN10256\_c0\_g5\_i4.p1 |  |  | PEAKS DB |
| DLYANTVLSGGTTMYPGIADR | 83.63 | 2214.0627 | 21 | 0.8 | 1108.0396 | 73.94 | 1 | F1:22276 | Fraction\_1\_23092022r.raw | 1.3622E5 |  |  |  |  |  |  | 1 | 1 | 0 | 0 | 0 | 0 | 0 | 0 | TRINITY\_DN273\_c0\_g4\_i11.p1:P83750|ACTB\_CYPCA:P02572|ACT2\_DROME:P60713|ACTB\_SHEEP:Q4R561|ACTB\_MACFA:Q76N69|ACTB\_CHLAE:Q4L0Y2|ACTB\_SPECI:TRINITY\_DN273\_c0\_g4\_i2.p1:TRINITY\_DN273\_c0\_g4\_i4.p1:TRINITY\_DN273\_c0\_g4\_i9.p1:P84856|ACTB\_CHLPG:P63258|ACTG\_BOVIN:P63260|ACTG\_MOUSE:P63259|ACTG\_RAT:A2BDB0|ACTG\_XENLA:Q8JJB8|ACTG\_TRISC:Q5JAK2|ACTG\_PELLE:P15475|ACTB\_XENBO:TRINITY\_DN836\_c0\_g1\_i1.p1:P10987|ACT1\_DROME:TRINITY\_DN836\_c0\_g1\_i3.p1:P68556|ACT1\_DIBDE:TRINITY\_DN836\_c0\_g1\_i2.p1:P04829|ACT3\_BOMMO |  |  | PEAKS DB |
| TVQQAVEQAEANIK | 83.62 | 1527.7893 | 14 | 0.7 | 764.9025 | 53.92 | 5 | F5:14133 | Fraction\_5\_23092022.raw |  |  |  | 1.2796E6 | 7.1371E5 |  |  | 4 | 0 | 0 | 0 | 2 | 2 | 0 | 0 | TRINITY\_DN1113\_c0\_g1\_i95.p1:TRINITY\_DN1113\_c0\_g1\_i47.p1:TRINITY\_DN1113\_c0\_g1\_i59.p1:TRINITY\_DN1113\_c0\_g1\_i53.p1:TRINITY\_DN1113\_c0\_g1\_i73.p1:TRINITY\_DN1113\_c0\_g1\_i6.p1:TRINITY\_DN1113\_c0\_g1\_i12.p1 |  |  | PEAKS DB |
| GTYIYVDM(+15.99)KDPLFLDQNNYMDKYIC(+57.02)LVAK | 83.44 | 3545.6921 | 29 | -0.4 | 887.4299 | 79.81 | 1 | F1:24902 | Fraction\_1\_23092022r.raw | 4.2438E6 |  |  |  |  |  |  | 1 | 1 | 0 | 0 | 0 | 0 | 0 | 0 | TRINITY\_DN4276\_c0\_g1\_i1.p1 | Oxidation (M); Carbamidomethylation | M8:Oxidation (M):89.82;C25:Carbamidomethylation:1000.00 | PEAKS DB |
| VEDLYEDVNYGVLLPHTK | 83.40 | 2103.0525 | 18 | 0.3 | 702.0250 | 68.15 | 2 | F2:19876 | Fraction\_2\_23092022.raw |  | 3.0315E5 |  |  |  | 1.5647E5 |  | 2 | 0 | 1 | 0 | 0 | 0 | 1 | 0 | TRINITY\_DN2611\_c0\_g1\_i12.p1:TRINITY\_DN2611\_c0\_g1\_i23.p1:TRINITY\_DN2611\_c0\_g1\_i19.p1:TRINITY\_DN2611\_c0\_g1\_i9.p1 |  |  | PEAKS DB |
| NPQPVSTC(+57.02)TRNEC(+57.02)VC(+57.02)VGLNAK | 83.32 | 2403.1094 | 21 | -0.5 | 802.0433 | 36.11 | 7 | F7:8969 | Fraction\_7\_23092022.raw |  |  | 4.6146E5 |  |  | 6.0973E5 | 3.3034E6 | 3 | 0 | 0 | 1 | 0 | 0 | 1 | 1 | TRINITY\_DN2326\_c0\_g1\_i12.p1 | Carbamidomethylation | C8:Carbamidomethylation:1000.00;C13:Carbamidomethylation:1000.00;C15:Carbamidomethylation:1000.00 | PEAKS DB |
| LGTVEVEDQISVTR | 83.26 | 1544.8046 | 14 | 0.8 | 773.4102 | 50.53 | 7 | F7:15742 | Fraction\_7\_23092022.raw |  |  | 1.7203E6 |  |  |  | 4.6328E6 | 2 | 0 | 0 | 1 | 0 | 0 | 0 | 1 | TRINITY\_DN3450\_c0\_g1\_i51.p1 |  |  | PEAKS DB |
| HSLIYVDNAFVVPGGR | 83.21 | 1742.9104 | 16 | -0.1 | 581.9774 | 61.13 | 5 | F5:17406 | Fraction\_5\_23092022.raw |  | 1.8244E6 | 1.5223E5 | 4.4864E5 | 3.1981E5 |  |  | 5 | 0 | 2 | 1 | 1 | 1 | 0 | 0 | TRINITY\_DN10256\_c0\_g10\_i1.p1:TRINITY\_DN10256\_c0\_g5\_i4.p1 |  |  | PEAKS DB |
| IIGNKAVLYTTDGINDR | 83.12 | 1861.9897 | 17 | 0.2 | 621.6707 | 44.86 | 2 | F2:9383 | Fraction\_2\_23092022.raw | 2.7184E5 | 6.3343E5 |  |  |  |  |  | 2 | 1 | 1 | 0 | 0 | 0 | 0 | 0 | TRINITY\_DN11986\_c0\_g1\_i3.p1 |  |  | PEAKS DB |
| VFYTVNEPAILC(+57.02)GEGYGSTTK | 83.07 | 2305.0935 | 21 | 0.5 | 1153.5546 | 66.75 | 2 | F2:19248 | Fraction\_2\_23092022.raw |  | 2.614E5 |  |  |  |  |  | 1 | 0 | 1 | 0 | 0 | 0 | 0 | 0 | TRINITY\_DN36500\_c0\_g1\_i2.p1 | Carbamidomethylation | C12:Carbamidomethylation:1000.00 | PEAKS DB |
| YKIPGRIGDSPIPGAGAYADQQVGAAAGTGDGDIM(+15.99)M(+15.99)R | 83.04 | 3707.7671 | 37 | -1.3 | 927.9479 | 53.42 | 6 | F6:14583 | Fraction\_6\_23092022.raw |  |  |  |  |  | 3.0039E5 |  | 1 | 0 | 0 | 0 | 0 | 0 | 1 | 0 | TRINITY\_DN5723\_c0\_g1\_i5.p1:TRINITY\_DN5723\_c0\_g1\_i7.p1 | Oxidation (M) | M35:Oxidation (M):1000.00;M36:Oxidation (M):1000.00 | PEAKS DB |
| IIHQC(+57.02)PPGLHFNK | 82.92 | 1559.8031 | 13 | 0.1 | 520.9417 | 32.25 | 4 | F4:5141 | Fraction\_4\_23092022.raw |  |  |  | 2.5474E6 |  |  |  | 2 | 0 | 0 | 0 | 2 | 0 | 0 | 0 | TRINITY\_DN2925\_c0\_g1\_i5.p1 | Carbamidomethylation | C5:Carbamidomethylation:1000.00 | PEAKS DB |
| AGTSNPVVSLTLVDLHDPTLNK | 82.85 | 2290.2168 | 22 | 0.4 | 764.4131 | 69.52 | 3 | F3:20851 | Fraction\_3\_23092022.raw |  |  | 6.1456E5 | 1.8069E6 | 8.228E5 | 3.4027E5 |  | 4 | 0 | 0 | 1 | 1 | 1 | 1 | 0 | TRINITY\_DN3450\_c0\_g1\_i51.p1 |  |  | PEAKS DB |
| YAYYPIAVQNIR | 82.84 | 1469.7666 | 12 | 2.4 | 735.8923 | 59.67 | 7 | F7:21300 | Fraction\_7\_23092022.raw |  |  | 5.2532E6 | 2.4981E7 | 2.2894E7 | 5.2224E7 | 1.0633E8 | 5 | 0 | 0 | 1 | 1 | 1 | 1 | 1 | TRINITY\_DN2326\_c0\_g1\_i13.p1 |  |  | PEAKS DB |
| NVQALEIELQSQLALK | 82.81 | 1796.0043 | 16 | 0.7 | 899.0101 | 78.36 | 3 | F3:24764 | Fraction\_3\_23092022.raw | 1.5024E6 | 1.2348E6 | 1.5662E6 | 5.1703E5 | 3.6324E5 | 5.6729E5 |  | 14 | 3 | 2 | 3 | 2 | 2 | 2 | 0 | P13645|K1C10\_HUMAN |  |  | PEAKS DB |
| KHDFYTLNTIKNHNEFKK | 82.66 | 2276.1702 | 18 | 1.5 | 570.0507 | 28.55 | 7 | F7:5614 | Fraction\_7\_23092022.raw |  |  |  |  |  |  | 4.3888E7 | 4 | 0 | 0 | 0 | 0 | 0 | 0 | 4 |  |  |  | PEAKS DB |
| IIHLTDDSFDTDVLK | 82.55 | 1730.8727 | 15 | -0.3 | 866.4434 | 61.84 | 1 | F1:16822 | Fraction\_1\_23092022r.raw | 4.6358E6 | 5.6366E5 |  |  |  |  |  | 3 | 2 | 1 | 0 | 0 | 0 | 0 | 0 | P0AA29|THIO\_SALTI:P0AA26|THIO\_ECOL6:P0AA27|THIO\_ECO57:P0AA30|THIO\_SHIFL:P0AA25|THIO\_ECOLI:P0AA28|THIO\_SALTY |  |  | PEAKS DB |
| QISNLQQSISDAEQR | 82.43 | 1715.8438 | 15 | 0.0 | 858.9291 | 47.35 | 1 | F1:10357 | Fraction\_1\_23092022r.raw | 1.3527E7 | 1.8835E6 | 1.3085E6 | 5.8787E5 | 6.9414E5 | 4.8023E5 | 2.3967E5 | 14 | 2 | 3 | 3 | 2 | 2 | 1 | 1 | P04264|K2C1\_HUMAN |  |  | PEAKS DB |
| FM(+15.99)LIHGSGDDNVHYQQSLALAK | 82.40 | 2459.1902 | 22 | 0.0 | 615.8048 | 49.19 | 6 | F6:12713 | Fraction\_6\_23092022.raw |  |  |  |  |  | 3.4937E6 |  | 1 | 0 | 0 | 0 | 0 | 0 | 1 | 0 | TRINITY\_DN3450\_c0\_g1\_i51.p1 | Oxidation (M) | M2:Oxidation (M):1000.00 | PEAKS DB |
| YVLYLQESGFDVIK | 82.33 | 1672.8712 | 14 | -0.2 | 837.4427 | 75.65 | 6 | F6:24543 | Fraction\_6\_23092022.raw |  |  |  |  |  | 2.0976E6 |  | 1 | 0 | 0 | 0 | 0 | 0 | 1 | 0 | TRINITY\_DN13586\_c0\_g2\_i1.p1 |  |  | PEAKS DB |
| ISGGIGVINFNK | 82.29 | 1217.6768 | 12 | -0.1 | 609.8456 | 56.88 | 5 | F5:15472 | Fraction\_5\_23092022.raw |  |  | 8.4174E5 | 2.1313E6 | 9.6524E6 | 7.1056E6 |  | 4 | 0 | 0 | 1 | 1 | 1 | 1 | 0 | TRINITY\_DN2228\_c0\_g4\_i1.p1 |  |  | PEAKS DB |
| YSNVVANPTLR | 82.28 | 1232.6514 | 11 | 0.5 | 617.3333 | 38.72 | 3 | F3:7055 | Fraction\_3\_23092022.raw |  |  | 2.1727E5 | 3.9106E5 | 8.9155E4 | 1.376E5 |  | 4 | 0 | 0 | 1 | 1 | 1 | 1 | 0 | TRINITY\_DN36831\_c0\_g1\_i1.p1 |  |  | PEAKS DB |
| NPQPVSTC(+57.02)TRNEC(+57.02)VC(+57.02)VGLNAKTYPK | 82.25 | 2892.3682 | 25 | -0.1 | 724.0992 | 36.33 | 7 | F7:9075 | Fraction\_7\_23092022.raw |  |  |  |  |  |  | 8.6003E5 | 1 | 0 | 0 | 0 | 0 | 0 | 0 | 1 | TRINITY\_DN2326\_c0\_g1\_i12.p1 | Carbamidomethylation | C8:Carbamidomethylation:1000.00;C13:Carbamidomethylation:1000.00;C15:Carbamidomethylation:1000.00 | PEAKS DB |
| SLVNLGGSKSISISVAR | 82.21 | 1686.9628 | 17 | -0.5 | 563.3279 | 52.86 | 5 | F5:13654 | Fraction\_5\_23092022.raw | 7.302E6 | 9.2273E6 | 1.6252E6 | 1.2995E6 | 4.1436E6 | 3.6138E6 | 1.8729E6 | 12 | 2 | 2 | 2 | 1 | 2 | 2 | 1 | P04264|K2C1\_HUMAN |  |  | PEAKS DB |
| IVLNADRWTVTDSDSIPTGEIR | 82.13 | 2457.2500 | 22 | 0.8 | 820.0912 | 62.08 | 6 | F6:18516 | Fraction\_6\_23092022.raw |  |  |  |  |  | 3.2699E5 |  | 1 | 0 | 0 | 0 | 0 | 0 | 1 | 0 | TRINITY\_DN3922\_c0\_g1\_i1.p1:TRINITY\_DN3922\_c0\_g1\_i2.p1:TRINITY\_DN3922\_c0\_g1\_i3.p1 |  |  | PEAKS DB |
| C(+57.02)KLVPEEISFVLSTR | 82.05 | 1776.9443 | 15 | -0.3 | 593.3219 | 68.54 | 7 | F7:26245 | Fraction\_7\_23092022.raw |  |  | 8.9535E5 |  |  |  | 5.1253E6 | 2 | 0 | 0 | 1 | 0 | 0 | 0 | 1 | TRINITY\_DN2326\_c0\_g1\_i12.p1 | Carbamidomethylation | C1:Carbamidomethylation:1000.00 | PEAKS DB |
| SKELTTEIDNNIEQISSYK | 82.04 | 2211.0906 | 19 | 0.5 | 738.0378 | 58.96 | 1 | F1:15535 | Fraction\_1\_23092022r.raw | 1.0388E6 |  |  |  |  |  |  | 1 | 1 | 0 | 0 | 0 | 0 | 0 | 0 | P13645|K1C10\_HUMAN |  |  | PEAKS DB |
| LYDSLPEDKVEEIFQK | 81.90 | 1951.9778 | 16 | 0.5 | 651.6669 | 64.66 | 7 | F7:24256 | Fraction\_7\_23092022.raw |  |  |  |  |  |  | 1.0379E6 | 1 | 0 | 0 | 0 | 0 | 0 | 0 | 1 | TRINITY\_DN36822\_c0\_g1\_i1.p1 |  |  | PEAKS DB |
| YYQYGSHIPFNFK | 81.90 | 1662.7831 | 13 | 0.1 | 832.3989 | 55.57 | 2 | F2:14195 | Fraction\_2\_23092022.raw | 1.2315E6 | 8.1127E5 |  |  |  |  |  | 3 | 2 | 1 | 0 | 0 | 0 | 0 | 0 | TRINITY\_DN1342\_c0\_g1\_i9.p1 |  |  | PEAKS DB |
| FISYLSDQKFSDIRK | 81.88 | 1845.9624 | 15 | 0.2 | 923.9886 | 47.40 | 7 | F7:14163 | Fraction\_7\_23092022.raw |  |  |  |  |  |  | 2.0263E8 | 4 | 0 | 0 | 0 | 0 | 0 | 0 | 4 | TRINITY\_DN13586\_c0\_g2\_i1.p1 |  |  | PEAKS DB |
| KLKESETFSKFISYLSDQK | 81.73 | 2277.1892 | 19 | 0.6 | 570.3049 | 59.83 | 7 | F7:21409 | Fraction\_7\_23092022.raw |  |  |  |  |  |  | 2.1113E6 | 1 | 0 | 0 | 0 | 0 | 0 | 0 | 1 | TRINITY\_DN13586\_c0\_g2\_i1.p1 |  |  | PEAKS DB |
| GTYIYVDM(+15.99)KDPLFLDQNNYMDK | 81.49 | 2698.2295 | 22 | 1.0 | 900.4180 | 68.03 | 1 | F1:19611 | Fraction\_1\_23092022r.raw | 1.5295E6 |  |  |  |  |  |  | 1 | 1 | 0 | 0 | 0 | 0 | 0 | 0 | TRINITY\_DN4276\_c0\_g1\_i1.p1 | Oxidation (M) | M8:Oxidation (M):98.81 | PEAKS DB |
| NKLNDLEDALQQAKEDLAR | 81.44 | 2183.1182 | 19 | 0.3 | 728.7136 | 70.64 | 1 | F1:20835 | Fraction\_1\_23092022r.raw | 4.449E6 | 2.8934E5 |  |  |  | 1.7807E5 |  | 3 | 1 | 1 | 0 | 0 | 0 | 1 | 0 | P04264|K2C1\_HUMAN |  |  | PEAKS DB |
| KIIAVDQDPLGIQGR | 81.39 | 1621.9150 | 15 | 0.5 | 541.6459 | 49.67 | 5 | F5:12253 | Fraction\_5\_23092022.raw | 2.7777E5 | 1.4111E7 |  |  | 1.3766E5 | 2.7292E6 |  | 6 | 1 | 2 | 0 | 0 | 1 | 2 | 0 | TRINITY\_DN2611\_c0\_g1\_i12.p1:TRINITY\_DN2611\_c0\_g1\_i23.p1:TRINITY\_DN2611\_c0\_g1\_i19.p1:TRINITY\_DN2611\_c0\_g1\_i9.p1 |  |  | PEAKS DB |
| RSIVHESYPGGVAPYDIALVELQTPLSLSK | 81.34 | 3238.7236 | 30 | 1.3 | 810.6892 | 77.53 | 3 | F3:24426 | Fraction\_3\_23092022.raw |  |  | 3.4215E6 |  | 1.069E5 |  |  | 3 | 0 | 0 | 2 | 0 | 1 | 0 | 0 | TRINITY\_DN33420\_c0\_g1\_i1.p1 |  |  | PEAKS DB |
| FVGQLNDYLQGFYR | 81.13 | 1718.8416 | 14 | -0.4 | 860.4277 | 77.39 | 4 | F4:24889 | Fraction\_4\_23092022.raw |  |  | 8.706E5 | 1.509E6 | 1.2277E5 | 4.5552E5 |  | 4 | 0 | 0 | 1 | 1 | 1 | 1 | 0 | TRINITY\_DN1113\_c0\_g1\_i95.p1:TRINITY\_DN1113\_c0\_g1\_i47.p1:TRINITY\_DN1113\_c0\_g1\_i59.p1:TRINITY\_DN1113\_c0\_g1\_i53.p1:TRINITY\_DN1113\_c0\_g1\_i73.p1:TRINITY\_DN1113\_c0\_g1\_i6.p1:TRINITY\_DN1113\_c0\_g1\_i12.p1 |  |  | PEAKS DB |
| GSYGSGGSSYGSGGGSYGSGGGGGGHGSYGSGSSSGGYR | 81.12 | 3311.3008 | 39 | 0.5 | 1104.7748 | 27.54 | 7 | F7:5208 | Fraction\_7\_23092022.raw | 1.0342E6 |  |  |  |  |  | 8.5781E5 | 2 | 1 | 0 | 0 | 0 | 0 | 0 | 1 | P04264|K2C1\_HUMAN |  |  | PEAKS DB |
| KFM(+15.99)LIHGSGDDNVHYQQSLALAK | 80.97 | 2587.2852 | 23 | 0.1 | 647.8286 | 43.63 | 6 | F6:10156 | Fraction\_6\_23092022.raw |  |  |  |  |  | 2.4747E6 |  | 1 | 0 | 0 | 0 | 0 | 0 | 1 | 0 | TRINITY\_DN3450\_c0\_g1\_i51.p1 | Oxidation (M) | M3:Oxidation (M):1000.00 | PEAKS DB |
| TTSGYAGGLSSAYGGLTSPGLSYSLGSSFGSGAGSSSFSR | 80.81 | 3724.7129 | 40 | 3.3 | 1242.5823 | 78.22 | 1 | F1:24186 | Fraction\_1\_23092022r.raw | 3.4882E5 | 1.2177E5 |  |  |  |  |  | 2 | 1 | 1 | 0 | 0 | 0 | 0 | 0 | P05787|K2C8\_HUMAN |  |  | PEAKS DB |
| ELTTEIDNNIEQISSYKSEITELRR | 80.74 | 2980.4988 | 25 | 0.9 | 746.1326 | 68.55 | 1 | F1:19854 | Fraction\_1\_23092022r.raw | 1.5526E6 | 2.0539E5 | 3.0809E5 |  |  |  |  | 3 | 1 | 1 | 1 | 0 | 0 | 0 | 0 | P13645|K1C10\_HUMAN |  |  | PEAKS DB |
| TSQNSELNNMQDLVEDYK | 80.54 | 2126.9426 | 18 | 0.7 | 1064.4794 | 64.81 | 1 | F1:18162 | Fraction\_1\_23092022r.raw | 5.3704E5 |  |  |  |  |  |  | 1 | 1 | 0 | 0 | 0 | 0 | 0 | 0 | P35908|K22E\_HUMAN |  |  | PEAKS DB |
| IILDVHQFKPEEISVR | 80.51 | 1922.0625 | 16 | 0.0 | 481.5229 | 57.05 | 7 | F7:19706 | Fraction\_7\_23092022.raw |  |  |  |  |  |  | 1.3336E6 | 1 | 0 | 0 | 0 | 0 | 0 | 0 | 1 | TRINITY\_DN5303\_c0\_g1\_i4.p1 |  |  | PEAKS DB |
| SAIVHLINYQDDAELATR | 80.50 | 2028.0276 | 18 | 0.0 | 677.0165 | 56.51 | 3 | F3:15031 | Fraction\_3\_23092022.raw |  |  | 9.9975E4 |  |  | 4.1769E4 |  | 2 | 0 | 0 | 1 | 0 | 0 | 1 | 0 | Q8SPJ1|PLAK\_BOVIN:P14923|PLAK\_HUMAN:Q8WNW3|PLAK\_PIG:Q02257|PLAK\_MOUSE |  |  | PEAKS DB |
| TLGTVDFYVNNGHNQPGC(+57.02)LPSFLGETC(+57.02)SHTR | 80.43 | 3477.5830 | 31 | 1.0 | 870.4039 | 65.29 | 4 | F4:19503 | Fraction\_4\_23092022.raw |  |  | 4.1532E6 | 9.7174E5 | 4.7255E5 | 1.6737E6 |  | 5 | 0 | 0 | 2 | 1 | 1 | 1 | 0 | TRINITY\_DN2326\_c0\_g1\_i12.p1 | Carbamidomethylation | C18:Carbamidomethylation:1000.00;C27:Carbamidomethylation:1000.00 | PEAKS DB |
| FIGYPTAVENTR | 80.37 | 1366.6881 | 12 | -0.5 | 684.3510 | 48.74 | 5 | F5:11816 | Fraction\_5\_23092022.raw | 3.3353E5 | 2.1858E5 | 1.0951E7 | 9.4032E6 | 1.3563E7 | 8.6082E6 | 3.584E6 | 8 | 1 | 1 | 2 | 1 | 1 | 1 | 1 | TRINITY\_DN2326\_c0\_g1\_i12.p1 |  |  | PEAKS DB |
| GSLGGGYSSGGFSGGSFSR | 80.29 | 1722.7598 | 19 | 0.3 | 862.3875 | 48.33 | 1 | F1:10823 | Fraction\_1\_23092022r.raw | 1.8958E5 |  | 9.4999E4 | 1.2666E5 |  |  |  | 3 | 1 | 0 | 1 | 1 | 0 | 0 | 0 |  |  |  | PEAKS DB |
| SYELPDGQVITIGNER | 80.24 | 1789.8846 | 16 | 0.5 | 895.9501 | 65.25 | 3 | F3:18910 | Fraction\_3\_23092022.raw | 3.1717E5 | 6.818E4 | 5.522E5 |  |  | 1.4708E5 |  | 4 | 1 | 1 | 1 | 0 | 0 | 1 | 0 | TRINITY\_DN273\_c0\_g4\_i11.p1:P83750|ACTB\_CYPCA:P02572|ACT2\_DROME:P60713|ACTB\_SHEEP:Q4R561|ACTB\_MACFA:Q76N69|ACTB\_CHLAE:Q4L0Y2|ACTB\_SPECI:TRINITY\_DN273\_c0\_g4\_i2.p1:TRINITY\_DN273\_c0\_g4\_i4.p1:TRINITY\_DN273\_c0\_g4\_i9.p1:P84856|ACTB\_CHLPG:P63258|ACTG\_BOVIN:P63260|ACTG\_MOUSE:P63259|ACTG\_RAT:A2BDB0|ACTG\_XENLA:Q8JJB8|ACTG\_TRISC:Q5JAK2|ACTG\_PELLE:P15475|ACTB\_XENBO:TRINITY\_DN836\_c0\_g1\_i1.p1:P10987|ACT1\_DROME:TRINITY\_DN836\_c0\_g1\_i3.p1:P68556|ACT1\_DIBDE:TRINITY\_DN836\_c0\_g1\_i2.p1:P04829|ACT3\_BOMMO:P86700|ACT\_CHIOP |  |  | PEAKS DB |
| SYELPDGQVLTIGNER | 80.24 | 1789.8846 | 16 | 0.5 | 895.9501 | 65.25 | 3 | F3:18910 | Fraction\_3\_23092022.raw | 3.1717E5 | 6.818E4 | 5.522E5 |  |  | 1.4708E5 |  | 4 | 1 | 1 | 1 | 0 | 0 | 1 | 0 |  |  |  | PEAKS DB |
| GIDGFRIDAVPHLFEREDLHDEPR | 80.16 | 2832.3943 | 24 | -0.4 | 473.0728 | 62.45 | 2 | F2:17317 | Fraction\_2\_23092022.raw |  | 1.0771E7 |  |  |  |  |  | 3 | 0 | 3 | 0 | 0 | 0 | 0 | 0 | TRINITY\_DN1342\_c0\_g1\_i23.p1:TRINITY\_DN1342\_c0\_g1\_i54.p1 |  |  | PEAKS DB |
| TLVVHADPDDLGKGGHELSK | 80.08 | 2087.0647 | 20 | -0.4 | 522.7733 | 34.45 | 6 | F6:6156 | Fraction\_6\_23092022.raw |  |  |  |  |  | 2.104E6 |  | 3 | 0 | 0 | 0 | 0 | 0 | 3 | 0 | TRINITY\_DN1931\_c0\_g1\_i1.p1:TRINITY\_DN1931\_c0\_g1\_i2.p1:TRINITY\_DN1931\_c0\_g1\_i4.p1 |  |  | PEAKS DB |
| TIVVHADPDDLGKGGHELSK | 80.08 | 2087.0647 | 20 | -0.4 | 522.7733 | 34.45 | 6 | F6:6156 | Fraction\_6\_23092022.raw |  |  |  |  |  | 2.104E6 |  | 3 | 0 | 0 | 0 | 0 | 0 | 3 | 0 |  |  |  | PEAKS DB |
| NKLNDLEDALQQAK | 80.05 | 1598.8264 | 14 | 0.1 | 800.4205 | 54.49 | 1 | F1:13565 | Fraction\_1\_23092022r.raw | 9.973E5 | 1.0003E5 | 1.1391E5 |  |  |  |  | 4 | 2 | 1 | 1 | 0 | 0 | 0 | 0 | P04264|K2C1\_HUMAN |  |  | PEAKS DB |
| TLNLDIQKNSLISHYK | 79.80 | 1886.0261 | 16 | 0.5 | 629.6830 | 50.12 | 1 | F1:11607 | Fraction\_1\_23092022r.raw | 9.9281E5 | 2.0578E6 |  |  |  |  |  | 3 | 1 | 2 | 0 | 0 | 0 | 0 | 0 | TRINITY\_DN10047\_c0\_g1\_i1.p1 |  |  | PEAKS DB |
| VVNRQISYITGVATSIEGIAR | 79.68 | 2246.2383 | 21 | 0.8 | 749.7540 | 71.90 | 4 | F4:22477 | Fraction\_4\_23092022.raw |  |  |  | 4.2439E4 |  |  |  | 1 | 0 | 0 | 0 | 1 | 0 | 0 | 0 | TRINITY\_DN28260\_c0\_g1\_i1.p1:TRINITY\_DN28260\_c0\_g1\_i2.p1:TRINITY\_DN28260\_c0\_g1\_i3.p1 |  |  | PEAKS DB |
| FDQHGYILSAAVSAAATSAR | 79.65 | 2035.0122 | 20 | -0.1 | 679.3446 | 61.76 | 4 | F4:17937 | Fraction\_4\_23092022.raw |  |  |  | 4.3575E5 | 7.232E4 |  |  | 2 | 0 | 0 | 0 | 1 | 1 | 0 | 0 | TRINITY\_DN36831\_c0\_g1\_i1.p1 |  |  | PEAKS DB |
| KISGGIGVINFNK | 79.64 | 1345.7717 | 13 | 0.2 | 449.5980 | 47.17 | 5 | F5:11168 | Fraction\_5\_23092022.raw |  |  | 3.1909E6 | 6.8845E6 | 2.3022E7 | 1.119E7 | 5.4209E5 | 9 | 0 | 0 | 2 | 2 | 2 | 2 | 1 | TRINITY\_DN2228\_c0\_g4\_i1.p1 |  |  | PEAKS DB |
| TYVVTGNM(+15.99)GSNDKVGDFVATDLDTGRPSTTVR | 79.56 | 3388.6204 | 32 | 0.6 | 848.1628 | 52.22 | 1 | F1:12546 | Fraction\_1\_23092022r.raw | 4.3315E5 |  |  |  |  |  |  | 1 | 1 | 0 | 0 | 0 | 0 | 0 | 0 | Q02413|DSG1\_HUMAN | Oxidation (M) | M8:Oxidation (M):1000.00 | PEAKS DB |
| KTPIIESGSLESILVTEKVLGVVR | 79.54 | 2566.4944 | 24 | 0.2 | 642.6310 | 79.40 | 1 | F1:24709 | Fraction\_1\_23092022r.raw | 4.1597E5 | 1.0039E6 |  |  |  |  |  | 4 | 2 | 2 | 0 | 0 | 0 | 0 | 0 | TRINITY\_DN10047\_c0\_g1\_i1.p1 |  |  | PEAKS DB |
| HDFYTLNTIKNHNEFK | 79.50 | 2019.9802 | 16 | -0.4 | 506.0021 | 38.96 | 7 | F7:10255 | Fraction\_7\_23092022.raw |  |  |  |  |  |  | 9.2153E5 | 2 | 0 | 0 | 0 | 0 | 0 | 0 | 2 |  |  |  | PEAKS DB |
| LNDLEDALQQAK | 79.46 | 1356.6885 | 12 | 0.3 | 679.3517 | 53.62 | 4 | F4:14237 | Fraction\_4\_23092022.raw | 6.0051E6 | 8.0494E6 | 1.159E7 | 2.6536E6 | 2.3375E6 | 3.0877E6 | 3.4363E6 | 7 | 1 | 1 | 1 | 1 | 1 | 1 | 1 | P04264|K2C1\_HUMAN |  |  | PEAKS DB |
| LEKEIETYHNLLEGGQEDFESSGAGK | 79.43 | 2879.3459 | 26 | 1.0 | 720.8445 | 55.38 | 1 | F1:13973 | Fraction\_1\_23092022r.raw | 5.4438E5 |  |  |  |  |  |  | 1 | 1 | 0 | 0 | 0 | 0 | 0 | 0 | P35527|K1C9\_HUMAN |  |  | PEAKS DB |
| QNC(+57.02)ELFEQLGEYKFQNALLVR | 79.27 | 2598.2900 | 21 | 1.0 | 867.1049 | 77.52 | 1 | F1:23878 | Fraction\_1\_23092022r.raw | 2.7648E5 |  |  |  |  |  |  | 1 | 1 | 0 | 0 | 0 | 0 | 0 | 0 | P02768|ALBU\_HUMAN | Carbamidomethylation | C3:Carbamidomethylation:1000.00 | PEAKS DB |
| SISISVAGGGGGFGAAGGFGGR | 79.24 | 1837.9070 | 22 | -0.1 | 919.9607 | 63.10 | 4 | F4:18566 | Fraction\_4\_23092022.raw | 4.0255E6 | 2.4211E6 | 4.0175E6 | 4.9726E5 | 1.3035E6 | 2.4713E6 | 4.288E5 | 7 | 1 | 1 | 1 | 1 | 1 | 1 | 1 | P35908|K22E\_HUMAN |  |  | PEAKS DB |
| YSEIIGLDPAGPM(+15.99)FK | 79.02 | 1652.8119 | 15 | 0.6 | 827.4138 | 66.27 | 5 | F5:19734 | Fraction\_5\_23092022.raw |  |  | 1.2949E7 | 7.2826E6 | 1.3421E7 | 1.3527E7 | 3.307E6 | 10 | 0 | 0 | 2 | 2 | 2 | 2 | 2 | TRINITY\_DN2326\_c0\_g1\_i12.p1 | Oxidation (M) | M13:Oxidation (M):1000.00 | PEAKS DB |
| LARKELPLVKDLYVR | 78.96 | 1812.0985 | 15 | -0.8 | 454.0315 | 44.03 | 7 | F7:12606 | Fraction\_7\_23092022.raw |  |  |  |  | 1.7484E5 |  | 1.2509E7 | 4 | 0 | 0 | 0 | 0 | 1 | 0 | 3 | TRINITY\_DN3450\_c0\_g1\_i51.p1 |  |  | PEAKS DB |
| RENNNSPDLC(+57.02)TSQIYC(+57.02)QGNLLK | 78.95 | 2623.2119 | 22 | -0.3 | 875.4110 | 51.10 | 2 | F2:12167 | Fraction\_2\_23092022.raw |  | 4.6187E5 |  |  |  |  |  | 1 | 0 | 1 | 0 | 0 | 0 | 0 | 0 | TRINITY\_DN10256\_c0\_g10\_i1.p1:TRINITY\_DN10256\_c0\_g5\_i4.p1 | Carbamidomethylation | C10:Carbamidomethylation:1000.00;C16:Carbamidomethylation:1000.00 | PEAKS DB |
| VDLLNQEIEFLK | 78.90 | 1459.7922 | 12 | 0.2 | 730.9036 | 78.62 | 5 | F5:25216 | Fraction\_5\_23092022.raw | 3.9725E6 | 5.0395E6 | 9.7411E6 | 1.2817E6 | 1.2932E6 | 1.4844E6 | 2.0665E6 | 7 | 1 | 1 | 1 | 1 | 1 | 1 | 1 | P35908|K22E\_HUMAN |  |  | PEAKS DB |
| ENKKHDFYTLNTIK | 78.80 | 1749.9049 | 14 | 0.2 | 584.3090 | 30.46 | 7 | F7:6450 | Fraction\_7\_23092022.raw |  |  |  |  |  |  | 2.17E7 | 2 | 0 | 0 | 0 | 0 | 0 | 0 | 2 |  |  |  | PEAKS DB |
| QSLEASLAETEGR | 78.74 | 1389.6736 | 13 | -0.5 | 695.8438 | 44.67 | 7 | F7:12899 | Fraction\_7\_23092022.raw | 4.4287E6 | 2.5816E6 | 5.3296E5 | 8.1428E5 | 7.0666E5 | 5.9797E5 | 3.6144E5 | 8 | 1 | 2 | 1 | 1 | 1 | 1 | 1 | P13645|K1C10\_HUMAN |  |  | PEAKS DB |
| ESETFSKFISYLSDQK | 78.66 | 1907.9152 | 16 | 0.6 | 636.9794 | 71.47 | 7 | F7:27704 | Fraction\_7\_23092022.raw |  |  |  |  |  |  | 1.4093E6 | 1 | 0 | 0 | 0 | 0 | 0 | 0 | 1 | TRINITY\_DN13586\_c0\_g2\_i1.p1 |  |  | PEAKS DB |
| SVVKASGLTNAEKLEILK | 78.58 | 1899.1040 | 18 | -0.1 | 634.0419 | 45.97 | 7 | F7:13452 | Fraction\_7\_23092022.raw |  |  |  | 5.4849E6 |  | 3.3763E6 | 2.4312E7 | 7 | 0 | 0 | 0 | 2 | 0 | 2 | 3 | TRINITY\_DN6370\_c0\_g1\_i1.p1 |  |  | PEAKS DB |
| LTVAKLNIDQNPGTAPK | 78.51 | 1778.9890 | 17 | -0.1 | 594.0035 | 43.40 | 2 | F2:8717 | Fraction\_2\_23092022.raw | 3.6352E5 | 1.7834E6 |  |  |  |  |  | 3 | 1 | 2 | 0 | 0 | 0 | 0 | 0 | P0AA29|THIO\_SALTI:P0AA26|THIO\_ECOL6:P0AA27|THIO\_ECO57:P0AA30|THIO\_SHIFL:P0AA25|THIO\_ECOLI:P0AA28|THIO\_SALTY |  |  | PEAKS DB |
| KVPQVSTPTLVEVSR | 78.42 | 1638.9304 | 15 | 0.1 | 547.3174 | 46.27 | 1 | F1:9857 | Fraction\_1\_23092022r.raw | 2.9405E6 | 6.5876E5 | 2.0378E5 |  | 1.8424E5 |  |  | 4 | 1 | 1 | 1 | 0 | 1 | 0 | 0 | P02768|ALBU\_HUMAN:P02769|ALBU\_BOVIN |  |  | PEAKS DB |
| QFTVLTGTAPLPQM(+15.99)FTLGYHQSR | 78.28 | 2608.3108 | 23 | 1.1 | 870.4452 | 71.23 | 1 | F1:21031 | Fraction\_1\_23092022r.raw | 1.0421E6 |  |  |  |  |  |  | 1 | 1 | 0 | 0 | 0 | 0 | 0 | 0 | TRINITY\_DN1709\_c0\_g1\_i4.p1:TRINITY\_DN1709\_c0\_g1\_i5.p1:TRINITY\_DN1709\_c0\_g1\_i6.p1:TRINITY\_DN1709\_c0\_g1\_i8.p1 | Oxidation (M) | M14:Oxidation (M):1000.00 | PEAKS DB |
| KHDFYTLNTIKNHNEFK | 78.02 | 2148.0752 | 17 | 1.6 | 430.6230 | 33.27 | 7 | F7:7699 | Fraction\_7\_23092022.raw |  |  |  |  |  |  | 6.2802E6 | 1 | 0 | 0 | 0 | 0 | 0 | 0 | 1 |  |  |  | PEAKS DB |
| NSLIYQIYPR | 77.99 | 1265.6768 | 10 | 0.3 | 633.8458 | 60.11 | 2 | F2:16228 | Fraction\_2\_23092022.raw | 1.2519E7 | 4.3107E7 |  |  |  | 3.7005E6 |  | 3 | 1 | 1 | 0 | 0 | 0 | 1 | 0 | TRINITY\_DN10047\_c0\_g1\_i1.p1 |  |  | PEAKS DB |
| HRESITLNYEIGIFSNPR | 77.95 | 2145.0967 | 18 | -0.8 | 716.0389 | 59.81 | 1 | F1:15959 | Fraction\_1\_23092022r.raw | 4.4706E7 | 2.4355E6 |  |  |  |  |  | 9 | 7 | 2 | 0 | 0 | 0 | 0 | 0 | TRINITY\_DN4276\_c0\_g1\_i1.p1 |  |  | PEAKS DB |
| ADLIAYIEQASK | 77.90 | 1320.6925 | 12 | 0.1 | 661.3536 | 74.89 | 7 | F7:29501 | Fraction\_7\_23092022.raw |  |  |  |  |  | 6.3116E6 | 1.0339E7 | 2 | 0 | 0 | 0 | 0 | 0 | 1 | 1 | P00038|CYC\_APIME |  |  | PEAKS DB |
| VSLAGAC(+57.02)GVGGYGSR | 77.82 | 1409.6721 | 15 | 0.0 | 705.8433 | 41.93 | 2 | F2:8051 | Fraction\_2\_23092022.raw | 4.6468E5 | 3.9894E5 | 2.1731E5 | 2.6554E4 | 3.3682E4 | 7.8008E4 |  | 6 | 1 | 1 | 1 | 1 | 1 | 1 | 0 | P13647|K2C5\_HUMAN | Carbamidomethylation | C7:Carbamidomethylation:1000.00 | PEAKS DB |
| SYNPQLTSYDYDAPLTEAGDPTEK | 77.76 | 2674.1921 | 24 | 0.6 | 1338.1041 | 61.19 | 2 | F2:16747 | Fraction\_2\_23092022.raw |  | 4.1997E5 |  |  |  |  |  | 1 | 0 | 1 | 0 | 0 | 0 | 0 | 0 | TRINITY\_DN11986\_c0\_g1\_i3.p1 |  |  | PEAKS DB |
| VALQC(+57.02)PPGLHFNPNLQVC(+57.02)DLPEQANC(+57.02)KSKPGDKEDEK | 77.71 | 4232.0088 | 37 | 1.2 | 706.3429 | 53.88 | 4 | F4:14405 | Fraction\_4\_23092022.raw |  |  |  | 4.2556E5 |  |  |  | 1 | 0 | 0 | 0 | 1 | 0 | 0 | 0 | TRINITY\_DN2925\_c0\_g1\_i5.p1 | Carbamidomethylation | C5:Carbamidomethylation:1000.00;C18:Carbamidomethylation:1000.00;C26:Carbamidomethylation:1000.00 | PEAKS DB |
| LALDLEIATYR | 77.60 | 1276.7026 | 11 | 0.4 | 639.3588 | 69.92 | 5 | F5:21320 | Fraction\_5\_23092022.raw | 1.0555E7 | 3.286E6 | 4.4833E6 |  | 9.0556E5 | 1.6127E6 | 1.5171E6 | 6 | 1 | 1 | 1 | 0 | 1 | 1 | 1 | P04264|K2C1\_HUMAN |  |  | PEAKS DB |
| LALDIEIATYR | 77.60 | 1276.7026 | 11 | 0.4 | 639.3588 | 69.92 | 5 | F5:21320 | Fraction\_5\_23092022.raw | 1.0555E7 | 3.286E6 | 4.4833E6 |  | 9.0556E5 | 1.6127E6 | 1.5171E6 | 6 | 1 | 1 | 1 | 0 | 1 | 1 | 1 | P05787|K2C8\_HUMAN:Q6KB66|K2C80\_HUMAN |  |  | PEAKS DB |
| GFRVTLWIHPFINK | 77.54 | 1726.9670 | 14 | 0.3 | 432.7492 | 67.94 | 1 | F1:19560 | Fraction\_1\_23092022r.raw | 2.2518E6 |  |  |  |  |  |  | 2 | 2 | 0 | 0 | 0 | 0 | 0 | 0 | TRINITY\_DN4276\_c0\_g1\_i1.p1 |  |  | PEAKS DB |
| TSQNSELNNM(+15.99)QDLVEDYKK | 77.51 | 2271.0325 | 19 | 0.3 | 758.0183 | 38.16 | 1 | F1:6461 | Fraction\_1\_23092022r.raw | 1.0913E6 |  |  |  |  |  |  | 2 | 2 | 0 | 0 | 0 | 0 | 0 | 0 | P35908|K22E\_HUMAN | Oxidation (M) | M10:Oxidation (M):1000.00 | PEAKS DB |
| ELQIC(+57.02)DLPEHAK | 77.42 | 1451.7079 | 12 | -0.2 | 726.8611 | 42.40 | 5 | F5:8963 | Fraction\_5\_23092022.raw |  |  | 7.1777E5 | 1.0075E7 | 2.2977E7 | 3.7552E6 | 2.3377E6 | 10 | 0 | 0 | 2 | 2 | 2 | 2 | 2 | TRINITY\_DN2925\_c0\_g1\_i5.p1 | Carbamidomethylation | C5:Carbamidomethylation:1000.00 | PEAKS DB |
| SNGKTELVGIVSWGIIPC(+57.02)GTR | 77.36 | 2243.1731 | 21 | 0.6 | 748.7321 | 71.80 | 6 | F6:22841 | Fraction\_6\_23092022.raw |  |  | 2.9E5 | 9.9456E4 |  | 2.6381E6 | 3.5337E6 | 4 | 0 | 0 | 1 | 1 | 0 | 1 | 1 | TRINITY\_DN33420\_c0\_g1\_i1.p1 | Carbamidomethylation | C18:Carbamidomethylation:1000.00 | PEAKS DB |
| ISNYLDFIVKSTPGETYC(+57.02)QVY | 77.33 | 2496.1882 | 21 | 0.9 | 1249.1025 | 82.39 | 3 | F3:26625 | Fraction\_3\_23092022.raw |  |  | 1.6798E6 |  |  |  |  | 1 | 0 | 0 | 1 | 0 | 0 | 0 | 0 | TRINITY\_DN1494\_c0\_g1\_i8.p1 | Carbamidomethylation | C18:Carbamidomethylation:1000.00 | PEAKS DB |
| AQLIDDALNLAR | 77.33 | 1311.7146 | 12 | 0.3 | 656.8647 | 68.07 | 5 | F5:20470 | Fraction\_5\_23092022.raw |  |  |  | 9.1366E5 | 2.2643E6 | 1.4183E7 | 2.1683E6 | 4 | 0 | 0 | 0 | 1 | 1 | 1 | 1 | TRINITY\_DN1113\_c0\_g1\_i95.p1:TRINITY\_DN1113\_c0\_g1\_i47.p1:TRINITY\_DN1113\_c0\_g1\_i59.p1:TRINITY\_DN1113\_c0\_g1\_i53.p1:TRINITY\_DN1113\_c0\_g1\_i73.p1:TRINITY\_DN1113\_c0\_g1\_i6.p1:TRINITY\_DN1113\_c0\_g1\_i12.p1 |  |  | PEAKS DB |
| DLIDFITENKDELGSATR | 77.32 | 2036.0061 | 18 | -0.5 | 679.6757 | 77.19 | 5 | F5:24575 | Fraction\_5\_23092022.raw |  |  |  | 1.1819E6 | 4.3715E5 |  |  | 2 | 0 | 0 | 0 | 1 | 1 | 0 | 0 | TRINITY\_DN1113\_c0\_g1\_i95.p1:TRINITY\_DN1113\_c0\_g1\_i47.p1:TRINITY\_DN1113\_c0\_g1\_i59.p1:TRINITY\_DN1113\_c0\_g1\_i53.p1:TRINITY\_DN1113\_c0\_g1\_i73.p1:TRINITY\_DN1113\_c0\_g1\_i6.p1:TRINITY\_DN1113\_c0\_g1\_i12.p1 |  |  | PEAKS DB |
| LIGHSLGAHTSGFAGKK | 77.30 | 1679.9106 | 17 | -0.2 | 420.9848 | 23.12 | 7 | F7:3582 | Fraction\_7\_23092022.raw |  |  |  |  |  |  | 5.9502E6 | 1 | 0 | 0 | 0 | 0 | 0 | 0 | 1 | A0A0M3KKW3|PA1\_VESBA |  |  | PEAKS DB |
| M(+15.99)SGDLSSNVTVSVTSSTISSNVASK | 77.27 | 2473.1853 | 25 | 0.1 | 1237.6001 | 54.51 | 1 | F1:13564 | Fraction\_1\_23092022r.raw | 7.315E5 |  |  |  |  |  |  | 2 | 2 | 0 | 0 | 0 | 0 | 0 | 0 | P35908|K22E\_HUMAN | Oxidation (M) | M1:Oxidation (M):1000.00 | PEAKS DB |
| DYTYLDHIYTKDDPR | 77.26 | 1913.8795 | 15 | 0.3 | 638.9673 | 47.98 | 5 | F5:11468 | Fraction\_5\_23092022.raw | 6.7442E5 | 6.2862E6 |  | 2.496E5 | 7.4735E5 |  |  | 5 | 1 | 2 | 0 | 1 | 1 | 0 | 0 | TRINITY\_DN1342\_c0\_g1\_i29.p1:TRINITY\_DN1342\_c0\_g1\_i23.p1:TRINITY\_DN1342\_c0\_g1\_i54.p1:TRINITY\_DN1342\_c0\_g1\_i9.p1:TRINITY\_DN1342\_c0\_g1\_i34.p1:TRINITY\_DN9761\_c0\_g1\_i1.p1 |  |  | PEAKS DB |
| THNLEPYFESFINNLRR | 77.20 | 2149.0703 | 17 | 0.1 | 538.2749 | 73.02 | 1 | F1:21884 | Fraction\_1\_23092022r.raw | 5.1649E6 | 4.3233E6 |  |  |  | 1.3216E6 | 2.4858E6 | 8 | 2 | 2 | 0 | 0 | 0 | 2 | 2 | P04264|K2C1\_HUMAN |  |  | PEAKS DB |
| KDIENQYETQITQIEHEVSSSGQEVQSSAK | 77.13 | 3391.6016 | 30 | -0.7 | 848.9071 | 64.61 | 1 | F1:18107 | Fraction\_1\_23092022r.raw | 5.1202E5 |  |  |  |  |  |  | 1 | 1 | 0 | 0 | 0 | 0 | 0 | 0 | P35527|K1C9\_HUMAN |  |  | PEAKS DB |
| FISYLSDQKFSDIR | 77.11 | 1717.8674 | 14 | 0.5 | 573.6301 | 55.96 | 7 | F7:18941 | Fraction\_7\_23092022.raw |  |  |  |  |  | 2.1992E5 | 5.8297E7 | 3 | 0 | 0 | 0 | 0 | 0 | 1 | 2 | TRINITY\_DN13586\_c0\_g2\_i1.p1 |  |  | PEAKS DB |
| LLLPNYADGIQAPTR | 76.98 | 1640.8885 | 15 | 0.6 | 821.4520 | 63.74 | 6 | F6:19218 | Fraction\_6\_23092022.raw |  |  | 8.2942E4 |  |  | 3.1285E6 | 2.1533E6 | 4 | 0 | 0 | 1 | 0 | 0 | 2 | 1 | TRINITY\_DN7919\_c0\_g1\_i8.p1:TRINITY\_DN7919\_c0\_g1\_i2.p1:TRINITY\_DN7919\_c0\_g1\_i4.p1 |  |  | PEAKS DB |
| LLNLESQKNIAGASVYK | 76.98 | 1847.0153 | 17 | 0.7 | 924.5156 | 48.00 | 2 | F2:10802 | Fraction\_2\_23092022.raw |  | 1.9327E6 |  | 1.5115E5 | 2.1031E5 |  |  | 4 | 0 | 2 | 0 | 1 | 1 | 0 | 0 | TRINITY\_DN23073\_c0\_g1\_i1.p1 |  |  | PEAKS DB |
| LLDEAQAGDNIGALLR | 76.92 | 1667.8842 | 16 | 0.6 | 834.9498 | 69.72 | 3 | F3:20923 | Fraction\_3\_23092022.raw |  |  | 1.6189E5 |  |  |  |  | 1 | 0 | 0 | 1 | 0 | 0 | 0 | 0 | Q0TMN0|EFTU\_CLOP1:Q8XFP8|EFTU\_CLOPE:Q0SQC8|EFTU\_CLOPS:Q877L9|EFTU\_CLOTE:A6LPP6|EFTU\_CLOB8:A9KRZ4|EFTU\_LACP7 |  |  | PEAKS DB |
| APILSDSSC(+57.02)K | 76.62 | 1076.5172 | 10 | -0.4 | 539.2657 | 27.37 | 7 | F7:5117 | Fraction\_7\_23092022.raw | 9.1494E6 | 8.0298E5 | 1.7801E6 | 1.8968E6 | 1.2655E6 | 1.7225E6 | 1.7471E6 | 8 | 1 | 1 | 1 | 1 | 1 | 1 | 2 | P00760|TRY1\_BOVIN | Carbamidomethylation | C9:Carbamidomethylation:1000.00 | PEAKS DB |
| DYTYLDHIYTKDDPRTYELVK | 76.51 | 2647.2805 | 21 | 1.1 | 662.8281 | 57.24 | 2 | F2:14940 | Fraction\_2\_23092022.raw |  | 5.1388E6 | 1.891E5 |  | 1.9821E5 |  |  | 4 | 0 | 2 | 1 | 0 | 1 | 0 | 0 | TRINITY\_DN1342\_c0\_g1\_i29.p1:TRINITY\_DN1342\_c0\_g1\_i23.p1:TRINITY\_DN1342\_c0\_g1\_i54.p1:TRINITY\_DN1342\_c0\_g1\_i9.p1:TRINITY\_DN1342\_c0\_g1\_i34.p1:TRINITY\_DN9761\_c0\_g1\_i1.p1 |  |  | PEAKS DB |
| YEELQVTVGR | 76.39 | 1192.6088 | 10 | -0.2 | 597.3115 | 44.41 | 5 | F5:9899 | Fraction\_5\_23092022.raw | 1.4421E6 | 1.1711E6 | 1.7404E6 | 2.5053E5 | 2.3909E5 |  |  | 5 | 1 | 1 | 1 | 1 | 1 | 0 | 0 | P35908|K22E\_HUMAN |  |  | PEAKS DB |
| FHHVIRYEYTFR | 76.33 | 1666.8368 | 12 | 0.3 | 417.7166 | 34.33 | 7 | F7:8215 | Fraction\_7\_23092022.raw |  |  |  |  |  |  | 3.6534E6 | 1 | 0 | 0 | 0 | 0 | 0 | 0 | 1 | TRINITY\_DN3450\_c0\_g1\_i51.p1 |  |  | PEAKS DB |
| LLETEC(+57.02)PQYIR | 76.32 | 1420.7020 | 11 | -0.4 | 711.3580 | 46.20 | 5 | F5:10710 | Fraction\_5\_23092022.raw | 5.59E5 | 2.3619E5 | 1.3896E5 | 7.7215E4 | 4.4886E4 | 7.7496E4 |  | 6 | 1 | 1 | 1 | 1 | 1 | 1 | 0 | P05109|S10A8\_HUMAN | Carbamidomethylation | C6:Carbamidomethylation:1000.00 | PEAKS DB |
| LNDLEEALQQAK | 76.28 | 1370.7041 | 12 | 0.8 | 686.3599 | 55.92 | 5 | F5:15050 | Fraction\_5\_23092022.raw | 2.4146E6 | 2.8944E6 | 3.8055E6 | 9.5826E5 | 1.1029E6 | 1.3559E6 |  | 6 | 1 | 1 | 1 | 1 | 1 | 1 | 0 | P35908|K22E\_HUMAN |  |  | PEAKS DB |
| TDGTPSDVSVTLKELGLQYPGGYK | 76.14 | 2524.2695 | 24 | 0.6 | 842.4310 | 73.36 | 2 | F2:22192 | Fraction\_2\_23092022.raw |  | 2.8914E5 |  |  |  |  |  | 1 | 0 | 1 | 0 | 0 | 0 | 0 | 0 | TRINITY\_DN2611\_c0\_g1\_i12.p1:TRINITY\_DN2611\_c0\_g1\_i23.p1:TRINITY\_DN2611\_c0\_g1\_i19.p1:TRINITY\_DN2611\_c0\_g1\_i9.p1 |  |  | PEAKS DB |
| IVC(+57.02)YFGSWAVYR | 75.90 | 1519.7281 | 12 | 0.9 | 760.8721 | 72.48 | 4 | F4:22715 | Fraction\_4\_23092022.raw |  |  |  | 1.477E5 |  |  |  | 1 | 0 | 0 | 0 | 1 | 0 | 0 | 0 | TRINITY\_DN36831\_c0\_g1\_i1.p1 | Carbamidomethylation | C3:Carbamidomethylation:1000.00 | PEAKS DB |
| IKPYDDYYIWR | 75.89 | 1530.7506 | 11 | 0.1 | 511.2575 | 58.17 | 1 | F1:15221 | Fraction\_1\_23092022r.raw | 1.3678E6 | 1.208E6 |  |  |  |  |  | 2 | 1 | 1 | 0 | 0 | 0 | 0 | 0 | TRINITY\_DN10047\_c0\_g1\_i1.p1 |  |  | PEAKS DB |
| DGAC(+57.02)TNEFQILKFIGYPTAVENTR | 75.80 | 2743.3274 | 24 | -4.5 | 915.4456 | 82.31 | 6 | F6:27538 | Fraction\_6\_23092022.raw |  |  | 3.939E5 |  |  | 2.673E6 |  | 2 | 0 | 0 | 1 | 0 | 0 | 1 | 0 | TRINITY\_DN2326\_c0\_g1\_i12.p1 | Carbamidomethylation | C4:Carbamidomethylation:1000.00 | PEAKS DB |
| SLVFDNIIEGYSK | 75.71 | 1483.7559 | 13 | 0.7 | 742.8857 | 72.99 | 6 | F6:23358 | Fraction\_6\_23092022.raw |  |  |  |  |  | 3.1009E6 | 3.8819E5 | 2 | 0 | 0 | 0 | 0 | 0 | 1 | 1 | TRINITY\_DN33395\_c0\_g1\_i1.p1 |  |  | PEAKS DB |
| TAAENDFVTLK | 75.68 | 1207.6084 | 11 | 0.1 | 604.8115 | 46.18 | 3 | F3:10375 | Fraction\_3\_23092022.raw | 1.0099E6 | 7.7457E5 | 1.1497E6 |  |  |  |  | 3 | 1 | 1 | 1 | 0 | 0 | 0 | 0 | P35908|K22E\_HUMAN |  |  | PEAKS DB |
| SQYEQLAEQNR | 75.63 | 1364.6321 | 11 | 0.1 | 683.3234 | 29.39 | 7 | F7:6031 | Fraction\_7\_23092022.raw |  |  |  |  |  |  | 6.198E4 | 1 | 0 | 0 | 0 | 0 | 0 | 0 | 1 | P13645|K1C10\_HUMAN |  |  | PEAKS DB |
| TGSFYVPVER | 75.62 | 1153.5768 | 10 | -0.2 | 577.7955 | 48.04 | 7 | F7:14466 | Fraction\_7\_23092022.raw |  | 2.0625E5 | 9.108E6 | 1.0098E7 |  | 3.7144E6 | 7.9345E6 | 5 | 0 | 1 | 1 | 1 | 0 | 1 | 1 | TRINITY\_DN2326\_c0\_g1\_i12.p1 |  |  | PEAKS DB |
| ASGLTKQENLEILKQHNEFR | 75.55 | 2354.2341 | 20 | -0.2 | 589.5657 | 41.58 | 4 | F4:8850 | Fraction\_4\_23092022.raw |  |  |  | 3.5359E6 |  | 1.9178E6 | 2.9167E7 | 5 | 0 | 0 | 0 | 1 | 0 | 2 | 2 | P35782|VA52\_VESCR:P35781|VA51\_VESCR |  |  | PEAKS DB |
| WELLQQVDTSTR | 75.54 | 1474.7416 | 12 | 0.4 | 738.3784 | 62.27 | 3 | F3:17590 | Fraction\_3\_23092022.raw | 4.3932E6 | 1.0276E6 | 7.5303E5 | 3.9044E5 | 1.2815E5 | 1.1274E6 | 2.2708E5 | 7 | 1 | 1 | 1 | 1 | 1 | 1 | 1 | P04264|K2C1\_HUMAN |  |  | PEAKS DB |
| LVGNYIATVTK | 75.39 | 1177.6707 | 11 | 0.1 | 589.8427 | 48.23 | 5 | F5:11564 | Fraction\_5\_23092022.raw | 4.9288E6 | 2.5048E6 | 4.5191E7 | 4.3416E8 | 3.1788E8 | 3.9701E7 | 1.0579E8 | 10 | 1 | 1 | 1 | 2 | 2 | 1 | 2 | A0A0M3KKW3|PA1\_VESBA |  |  | PEAKS DB |
| SVIYAYIDGRGSSNK | 75.39 | 1628.8158 | 15 | -0.1 | 815.4151 | 42.17 | 6 | F6:9530 | Fraction\_6\_23092022.raw |  |  | 1.6986E5 |  |  | 9.841E5 | 5.7092E5 | 4 | 0 | 0 | 1 | 0 | 0 | 2 | 1 | TRINITY\_DN3450\_c0\_g1\_i51.p1 |  |  | PEAKS DB |
| NVLHTQSSDILR | 75.39 | 1381.7313 | 12 | -0.2 | 691.8728 | 36.08 | 1 | F1:5791 | Fraction\_1\_23092022r.raw | 1.1052E7 |  |  |  |  | 3.198E5 |  | 3 | 2 | 0 | 0 | 0 | 0 | 1 | 0 | TRINITY\_DN4276\_c0\_g1\_i1.p1 |  |  | PEAKS DB |
| LIGHSLGAHVSGFAGKK | 75.09 | 1677.9314 | 17 | 1.4 | 420.4907 | 28.17 | 7 | F7:5463 | Fraction\_7\_23092022.raw |  |  |  | 6.2248E5 |  | 2.5269E6 | 6.6358E6 | 4 | 0 | 0 | 0 | 1 | 0 | 1 | 2 | TRINITY\_DN2326\_c0\_g1\_i13.p1 |  |  | PEAKS DB |
| SLNNQFASFIDKVR | 75.00 | 1637.8525 | 14 | 0.1 | 819.9336 | 64.93 | 1 | F1:18201 | Fraction\_1\_23092022r.raw | 6.8927E7 | 1.0717E7 | 1.3632E7 | 2.2062E6 | 1.9222E6 | 1.0131E7 | 7.4412E6 | 15 | 3 | 2 | 2 | 2 | 2 | 2 | 2 | P04264|K2C1\_HUMAN |  |  | PEAKS DB |
| AIGGGLSSVGGGSSTIK | 74.89 | 1446.7678 | 17 | 0.2 | 724.3914 | 40.87 | 6 | F6:8999 | Fraction\_6\_23092022.raw |  | 8.1429E4 |  |  | 1.5433E5 | 1.3125E5 |  | 3 | 0 | 1 | 0 | 0 | 1 | 1 | 0 | P48668|K2C6C\_HUMAN |  |  | PEAKS DB |
| AITEFNAQPIISEIYGSGSTK | 74.84 | 2225.1216 | 21 | 0.7 | 1113.5688 | 78.12 | 2 | F2:24320 | Fraction\_2\_23092022.raw |  | 6.2463E5 |  |  |  |  |  | 1 | 0 | 1 | 0 | 0 | 0 | 0 | 0 | TRINITY\_DN1455\_c0\_g1\_i1.p1 |  |  | PEAKS DB |
| DGAC(+57.02)TNEFQILK | 74.83 | 1394.6500 | 12 | 0.0 | 698.3323 | 57.21 | 5 | F5:15614 | Fraction\_5\_23092022.raw |  |  | 1.1719E6 | 1.4003E6 | 2.1747E6 | 1.4246E6 |  | 4 | 0 | 0 | 1 | 1 | 1 | 1 | 0 | TRINITY\_DN2326\_c0\_g1\_i12.p1 | Carbamidomethylation | C4:Carbamidomethylation:1000.00 | PEAKS DB |
| ALEEANADLEVK | 74.72 | 1300.6510 | 12 | 0.5 | 651.3331 | 40.27 | 3 | F3:7722 | Fraction\_3\_23092022.raw | 4.9827E5 | 2.0105E5 | 6.9699E5 | 6.4208E4 | 5.161E4 | 1.2986E5 |  | 6 | 1 | 1 | 1 | 1 | 1 | 1 | 0 | P02533|K1C14\_HUMAN:P08779|K1C16\_HUMAN |  |  | PEAKS DB |
| QIAAYDYNPLSGTISNKR | 74.55 | 2010.0170 | 18 | -0.7 | 671.0125 | 52.72 | 4 | F4:13863 | Fraction\_4\_23092022.raw |  |  | 1.3568E5 | 1.6835E5 |  |  |  | 2 | 0 | 0 | 1 | 1 | 0 | 0 | 0 | TRINITY\_DN1736\_c0\_g1\_i5.p1:TRINITY\_DN1736\_c0\_g1\_i25.p1:TRINITY\_DN1736\_c0\_g1\_i30.p1:TRINITY\_DN1736\_c0\_g1\_i24.p1:TRINITY\_DN1736\_c0\_g1\_i4.p1 |  |  | PEAKS DB |
| TITLEVEPSDTIENVK | 74.53 | 1786.9200 | 16 | 0.3 | 894.4675 | 59.25 | 5 | F5:16571 | Fraction\_5\_23092022.raw | 2.0921E5 | 2.2744E5 | 4.5763E5 | 8.1486E4 | 8.2535E4 |  |  | 5 | 1 | 1 | 1 | 1 | 1 | 0 | 0 | P62972|UBIQP\_XENLA:P0CG65|UBB\_PANTR:P0CG62|UBB\_CHICK:P0CG51|UBB\_RAT:P0CG68|UBC\_PIG:P62976|UBIQP\_CRIGR:P0CG48|UBC\_HUMAN:P29504|RS27A\_MANSE:P15357|RS27A\_DROME:P62979|RS27A\_HUMAN |  |  | PEAKS DB |
| NVQDAIADAEQR | 74.52 | 1328.6321 | 12 | 0.1 | 665.3234 | 41.68 | 5 | F5:8658 | Fraction\_5\_23092022.raw | 1.8991E6 | 3.032E6 | 4.2427E6 | 1.1358E6 | 5.9496E5 | 9.5408E5 | 9.4723E5 | 7 | 1 | 1 | 1 | 1 | 1 | 1 | 1 | P35908|K22E\_HUMAN |  |  | PEAKS DB |
| GAVHDVKDVLDSVL | 74.52 | 1465.7776 | 14 | 1.1 | 733.8969 | 71.67 | 1 | F1:21251 | Fraction\_1\_23092022r.raw | 8.8165E5 | 6.9589E5 | 2.28E5 | 9.1474E5 | 3.0072E5 |  | 2.2789E6 | 8 | 2 | 1 | 1 | 2 | 1 | 0 | 1 | P81605|DCD\_HUMAN |  |  | PEAKS DB |
| YLVGQNIAKQSTTAASFAPVSNM(+15.99)VK | 74.46 | 2640.3582 | 25 | 0.3 | 881.1270 | 52.74 | 4 | F4:13835 | Fraction\_4\_23092022.raw |  |  |  | 3.0247E5 |  | 7.2766E5 |  | 2 | 0 | 0 | 0 | 1 | 0 | 1 | 0 | TRINITY\_DN6370\_c0\_g1\_i1.p1 | Oxidation (M) | M23:Oxidation (M):1000.00 | PEAKS DB |
| YNNFGVYWVNR | 74.41 | 1430.6731 | 11 | 0.5 | 716.3442 | 62.45 | 6 | F6:18677 | Fraction\_6\_23092022.raw |  |  |  |  |  | 7.636E5 |  | 1 | 0 | 0 | 0 | 0 | 0 | 1 | 0 | TRINITY\_DN33395\_c0\_g1\_i1.p1 |  |  | PEAKS DB |
| YKDDPQIINGYIYLHHK | 74.37 | 2116.0742 | 17 | -0.4 | 530.0256 | 44.92 | 6 | F6:10765 | Fraction\_6\_23092022.raw |  |  |  |  |  | 4.3453E5 |  | 1 | 0 | 0 | 0 | 0 | 0 | 1 | 0 | TRINITY\_DN13586\_c0\_g2\_i1.p1 |  |  | PEAKS DB |
| IGDSPIPGAGAYADQQVGAAAGTGDGDIM(+15.99)M(+15.99)R | 74.35 | 2993.3494 | 31 | 0.6 | 998.7910 | 55.97 | 6 | F6:15730 | Fraction\_6\_23092022.raw |  |  |  |  |  | 1.0585E6 |  | 1 | 0 | 0 | 0 | 0 | 0 | 1 | 0 | TRINITY\_DN5723\_c0\_g1\_i5.p1:TRINITY\_DN5723\_c0\_g1\_i7.p1 | Oxidation (M) | M29:Oxidation (M):1000.00;M30:Oxidation (M):1000.00 | PEAKS DB |
| DYTYLDHIYTK | 74.29 | 1430.6718 | 11 | 0.2 | 716.3433 | 52.27 | 5 | F5:13434 | Fraction\_5\_23092022.raw |  | 1.8665E5 |  |  | 1.9599E5 |  |  | 3 | 0 | 1 | 0 | 0 | 2 | 0 | 0 | TRINITY\_DN1342\_c0\_g1\_i29.p1:TRINITY\_DN1342\_c0\_g1\_i23.p1:TRINITY\_DN1342\_c0\_g1\_i54.p1:TRINITY\_DN1342\_c0\_g1\_i9.p1:TRINITY\_DN1342\_c0\_g1\_i34.p1:TRINITY\_DN9761\_c0\_g1\_i1.p1 |  |  | PEAKS DB |
| KLGTVEVEDQISVTR | 74.25 | 1672.8995 | 15 | -0.6 | 558.6401 | 43.29 | 7 | F7:12220 | Fraction\_7\_23092022.raw |  |  | 2.0616E6 | 3.3199E5 | 1.7947E5 | 5.2975E6 | 3.9832E6 | 8 | 0 | 0 | 2 | 1 | 1 | 2 | 2 | TRINITY\_DN3450\_c0\_g1\_i51.p1 |  |  | PEAKS DB |
| APSTYGGGLSVSSSR | 74.23 | 1424.6896 | 15 | 0.7 | 713.3525 | 34.68 | 6 | F6:6260 | Fraction\_6\_23092022.raw |  |  |  |  |  | 1.0711E5 |  | 1 | 0 | 0 | 0 | 0 | 0 | 1 | 0 | P02533|K1C14\_HUMAN |  |  | PEAKS DB |
| NTAKYLVGQNIAK | 74.15 | 1418.7881 | 13 | -0.3 | 710.4011 | 31.85 | 7 | F7:7110 | Fraction\_7\_23092022.raw |  |  |  |  |  | 3.7878E5 | 1.2002E6 | 4 | 0 | 0 | 0 | 0 | 0 | 2 | 2 | TRINITY\_DN6370\_c0\_g1\_i1.p1 |  |  | PEAKS DB |
| VRLFLPHNFDESK | 74.09 | 1600.8362 | 13 | -0.1 | 534.6193 | 46.90 | 7 | F7:13921 | Fraction\_7\_23092022.raw |  |  |  |  |  |  | 1.5592E6 | 2 | 0 | 0 | 0 | 0 | 0 | 0 | 2 | TRINITY\_DN3450\_c0\_g1\_i51.p1 |  |  | PEAKS DB |
| LNDLEEALQQAKEDLAR | 74.07 | 1954.9960 | 17 | 0.4 | 652.6729 | 74.42 | 1 | F1:22474 | Fraction\_1\_23092022r.raw | 3.0756E5 |  |  |  |  |  |  | 1 | 1 | 0 | 0 | 0 | 0 | 0 | 0 | P35908|K22E\_HUMAN |  |  | PEAKS DB |
| LGTVEVEDQISVTRK | 74.00 | 1672.8995 | 15 | -0.1 | 558.6404 | 42.28 | 7 | F7:11780 | Fraction\_7\_23092022.raw |  |  | 1.9664E5 |  |  | 6.5498E5 | 3.3517E5 | 3 | 0 | 0 | 1 | 0 | 0 | 1 | 1 | TRINITY\_DN3450\_c0\_g1\_i51.p1 |  |  | PEAKS DB |
| YQHWPIQHM(+15.99)YYEEEPYVPTHPVNM(+15.99)AVAER | 73.92 | 3645.6445 | 29 | 0.2 | 730.1364 | 49.11 | 1 | F1:11234 | Fraction\_1\_23092022r.raw | 7.8258E6 |  |  |  |  |  |  | 2 | 2 | 0 | 0 | 0 | 0 | 0 | 0 | TRINITY\_DN4276\_c0\_g1\_i1.p1 | Oxidation (M) | M9:Oxidation (M):1000.00;M24:Oxidation (M):1000.00 | PEAKS DB |
| HTFSGVASVESSSGEAFHVGK | 73.75 | 2118.9971 | 21 | -1.1 | 707.3389 | 43.07 | 1 | F1:8489 | Fraction\_1\_23092022r.raw | 4.2769E5 |  |  |  |  |  |  | 1 | 1 | 0 | 0 | 0 | 0 | 0 | 0 | P12763|FETUA\_BOVIN |  |  | PEAKS DB |
| EIIAILPVEELKELHKR | 73.74 | 2029.1935 | 17 | -0.8 | 508.3052 | 61.19 | 7 | F7:22240 | Fraction\_7\_23092022.raw |  |  |  |  |  |  | 8.7018E6 | 2 | 0 | 0 | 0 | 0 | 0 | 0 | 2 | TRINITY\_DN13586\_c0\_g2\_i1.p1 |  |  | PEAKS DB |
| HDFYTLNTIK | 73.71 | 1250.6295 | 10 | -0.2 | 626.3219 | 43.98 | 7 | F7:12548 | Fraction\_7\_23092022.raw |  |  | 9.247E5 |  |  |  | 2.073E6 | 2 | 0 | 0 | 1 | 0 | 0 | 0 | 1 |  |  |  | PEAKS DB |
| YVIYDIELGGYDK | 73.54 | 1546.7555 | 13 | 0.6 | 774.3855 | 69.66 | 6 | F6:21892 | Fraction\_6\_23092022.raw |  |  |  | 1.0394E5 |  | 4.332E5 | 9.399E5 | 3 | 0 | 0 | 0 | 1 | 0 | 1 | 1 | TRINITY\_DN3450\_c0\_g1\_i51.p1 |  |  | PEAKS DB |
| EREQIKSLNNQFASFIDKVR | 73.50 | 2421.2764 | 20 | -0.5 | 606.3260 | 58.06 | 2 | F2:15327 | Fraction\_2\_23092022.raw |  | 8.092E5 |  |  |  |  |  | 1 | 0 | 1 | 0 | 0 | 0 | 0 | 0 | P04264|K2C1\_HUMAN |  |  | PEAKS DB |
| SGGGFSSGSAGIINYQRRTTSSSTR | 73.33 | 2533.2268 | 25 | 0.6 | 634.3143 | 34.02 | 7 | F7:8077 | Fraction\_7\_23092022.raw |  |  |  |  |  |  | 7.9048E4 | 1 | 0 | 0 | 0 | 0 | 0 | 0 | 1 | P04264|K2C1\_HUMAN |  |  | PEAKS DB |
| NKILTATVDNANVLLQIDNAR | 73.26 | 2295.2546 | 21 | 0.6 | 766.0927 | 67.17 | 1 | F1:19248 | Fraction\_1\_23092022r.raw | 2.5238E5 |  |  |  |  |  |  | 1 | 1 | 0 | 0 | 0 | 0 | 0 | 0 | P02533|K1C14\_HUMAN |  |  | PEAKS DB |
| SKDC(+57.02)SQRIC(+57.02)ETDAHYVQIIHTSNNLGTER | 73.26 | 3431.5947 | 29 | -0.1 | 687.3262 | 39.88 | 5 | F5:7848 | Fraction\_5\_23092022.raw |  |  |  |  | 2.5666E6 | 7.5961E5 | 1.327E5 | 5 | 0 | 0 | 0 | 0 | 3 | 1 | 1 | TRINITY\_DN2326\_c0\_g1\_i12.p1 | Carbamidomethylation | C4:Carbamidomethylation:1000.00;C9:Carbamidomethylation:1000.00 | PEAKS DB |
| ELTPDQRIGLVQGRVKEAVR | 73.23 | 2263.2759 | 20 | -0.1 | 453.6624 | 42.68 | 7 | F7:11954 | Fraction\_7\_23092022.raw |  |  |  |  |  | 2.5389E5 | 7.4035E5 | 2 | 0 | 0 | 0 | 0 | 0 | 1 | 1 | TRINITY\_DN2228\_c0\_g2\_i2.p1:TRINITY\_DN2228\_c0\_g2\_i1.p1 |  |  | PEAKS DB |
| NAVFYQIYPR | 73.17 | 1269.6505 | 10 | 0.3 | 635.8327 | 57.87 | 7 | F7:20277 | Fraction\_7\_23092022.raw | 1.1663E7 | 9.8135E7 | 2.3489E6 | 2.1542E5 | 2.417E5 | 9.3508E7 | 6.8859E7 | 8 | 1 | 1 | 1 | 1 | 1 | 2 | 1 | TRINITY\_DN1342\_c0\_g1\_i29.p1:TRINITY\_DN1342\_c0\_g1\_i23.p1:TRINITY\_DN1342\_c0\_g1\_i54.p1:TRINITY\_DN1342\_c0\_g1\_i9.p1:TRINITY\_DN1342\_c0\_g1\_i34.p1 |  |  | PEAKS DB |
| FINYM(+15.99)QAVADLNPK | 73.10 | 1638.8075 | 14 | 0.9 | 820.4117 | 64.73 | 4 | F4:19264 | Fraction\_4\_23092022.raw |  |  |  | 3.6461E5 | 2.691E5 |  |  | 2 | 0 | 0 | 0 | 1 | 1 | 0 | 0 | TRINITY\_DN594\_c1\_g4\_i5.p1:TRINITY\_DN594\_c1\_g4\_i1.p1 | Oxidation (M) | M5:Oxidation (M):1000.00 | PEAKS DB |
| M(+15.99)IAPILDEIADEYQGK | 73.06 | 1820.8866 | 16 | -0.3 | 911.4503 | 78.99 | 2 | F2:24679 | Fraction\_2\_23092022.raw | 5.1846E6 | 2.0664E6 | 4.9225E5 | 4.3955E5 |  |  |  | 4 | 1 | 1 | 1 | 1 | 0 | 0 | 0 | P0AA29|THIO\_SALTI:P0AA26|THIO\_ECOL6:P0AA27|THIO\_ECO57:P0AA30|THIO\_SHIFL:P0AA25|THIO\_ECOLI:P0AA28|THIO\_SALTY | Oxidation (M) | M1:Oxidation (M):1000.00 | PEAKS DB |
| KIYSWENNLSLR | 72.97 | 1521.7939 | 12 | 0.9 | 508.2724 | 50.52 | 7 | F7:15713 | Fraction\_7\_23092022.raw |  |  |  |  |  | 3.2213E5 | 1.097E6 | 2 | 0 | 0 | 0 | 0 | 0 | 1 | 1 | TRINITY\_DN3450\_c0\_g1\_i51.p1 |  |  | PEAKS DB |
| M(+15.99)LVQQYNVPM(+15.99)ANIR | 72.97 | 1707.8436 | 14 | 0.0 | 854.9291 | 47.44 | 4 | F4:11455 | Fraction\_4\_23092022.raw | 4.2312E5 |  | 2.7821E6 | 1.1326E7 | 7.4596E6 | 1.5611E6 | 7.3433E6 | 13 | 2 | 0 | 2 | 2 | 3 | 2 | 2 |  | Oxidation (M) | M1:Oxidation (M):1000.00;M10:Oxidation (M):1000.00 | PEAKS DB |
| KLGIFNSEGNINEEVLR | 72.93 | 1931.0112 | 17 | -0.1 | 644.6776 | 56.72 | 7 | F7:19481 | Fraction\_7\_23092022.raw |  |  |  |  |  |  | 8.0931E5 | 1 | 0 | 0 | 0 | 0 | 0 | 0 | 1 | TRINITY\_DN36822\_c0\_g1\_i1.p1 |  |  | PEAKS DB |
| YSEIIGLDPAGPM(+15.99)FKSK | 72.91 | 1867.9390 | 17 | 0.1 | 623.6537 | 55.88 | 3 | F3:14752 | Fraction\_3\_23092022.raw |  |  | 1.296E5 |  |  |  |  | 1 | 0 | 0 | 1 | 0 | 0 | 0 | 0 | TRINITY\_DN2326\_c0\_g1\_i12.p1 | Oxidation (M) | M13:Oxidation (M):1000.00 | PEAKS DB |
| LAADDFRLKYENEVALR | 72.85 | 2022.0533 | 17 | 0.1 | 675.0251 | 53.39 | 2 | F2:13215 | Fraction\_2\_23092022.raw | 8.8907E6 | 4.2685E6 | 2.9208E6 | 6.3854E5 | 6.7978E5 | 1.4936E6 | 1.4353E6 | 13 | 2 | 2 | 2 | 2 | 2 | 1 | 2 | P13645|K1C10\_HUMAN |  |  | PEAKS DB |
| LTGM(+15.99)AFRVPVHNVSVVDLTVR | 72.84 | 2325.2627 | 21 | 0.6 | 582.3233 | 58.07 | 6 | F6:16689 | Fraction\_6\_23092022.raw |  |  |  |  |  | 2.8368E6 |  | 2 | 0 | 0 | 0 | 0 | 0 | 2 | 0 | TRINITY\_DN5849\_c0\_g1\_i1.p1:TRINITY\_DN5849\_c0\_g1\_i2.p1:TRINITY\_DN5849\_c0\_g1\_i10.p1 | Oxidation (M) | M4:Oxidation (M):1000.00 | PEAKS DB |
| MLVQQYNVPM(+15.99)ANIR | 72.76 | 1691.8488 | 14 | 1.4 | 846.9329 | 52.52 | 1 | F1:12675 | Fraction\_1\_23092022r.raw | 3.8832E5 |  |  |  |  |  |  | 2 | 2 | 0 | 0 | 0 | 0 | 0 | 0 |  | Oxidation (M) | M10:Oxidation (M):162.16 | PEAKS DB |
| IYSWENNLSLR | 72.69 | 1393.6990 | 11 | -0.1 | 697.8567 | 61.06 | 5 | F5:17355 | Fraction\_5\_23092022.raw |  |  |  | 1.3573E5 | 8.828E5 | 1.4541E6 | 4.0913E6 | 4 | 0 | 0 | 0 | 1 | 1 | 1 | 1 | TRINITY\_DN3450\_c0\_g1\_i51.p1 |  |  | PEAKS DB |
| TGDILQFDVIKQR | 72.51 | 1531.8358 | 13 | 0.6 | 511.6195 | 58.23 | 4 | F4:16325 | Fraction\_4\_23092022.raw |  |  | 2.8091E5 | 1.9997E6 |  | 2.6038E6 | 1.1819E7 | 7 | 0 | 0 | 1 | 2 | 0 | 2 | 2 | TRINITY\_DN3450\_c0\_g1\_i51.p1 |  |  | PEAKS DB |
| GTYIYVDM(+15.99)KDPLFLDQNNYM(+15.99)DK | 72.44 | 2714.2244 | 22 | 1.1 | 905.7498 | 64.10 | 1 | F1:17868 | Fraction\_1\_23092022r.raw | 4.6244E6 |  |  |  |  |  |  | 1 | 1 | 0 | 0 | 0 | 0 | 0 | 0 | TRINITY\_DN4276\_c0\_g1\_i1.p1 | Oxidation (M) | M8:Oxidation (M):1000.00;M20:Oxidation (M):1000.00 | PEAKS DB |
| AQYEDIAQKSKAEAESLYQSKYEELQITAGR | 72.32 | 3546.7478 | 31 | 0.0 | 710.3568 | 62.42 | 1 | F1:17105 | Fraction\_1\_23092022r.raw | 6.9791E5 | 7.8389E5 |  |  |  |  |  | 3 | 1 | 2 | 0 | 0 | 0 | 0 | 0 | P04264|K2C1\_HUMAN |  |  | PEAKS DB |
| VLDTKWTLLQEQGTK | 72.29 | 1758.9515 | 15 | 0.5 | 587.3248 | 54.32 | 1 | F1:13486 | Fraction\_1\_23092022r.raw | 2.7823E5 |  |  |  |  |  |  | 1 | 1 | 0 | 0 | 0 | 0 | 0 | 0 | P48668|K2C6C\_HUMAN:P13647|K2C5\_HUMAN |  |  | PEAKS DB |
| GFSSGSAVVSGGSR | 72.08 | 1253.6000 | 14 | -0.5 | 627.8069 | 29.48 | 7 | F7:6074 | Fraction\_7\_23092022.raw |  |  |  |  |  |  | 6.6622E4 | 1 | 0 | 0 | 0 | 0 | 0 | 0 | 1 | P35908|K22E\_HUMAN:Q3TTY5|K22E\_MOUSE |  |  | PEAKS DB |
| SGGGGGGGFGRVSLAGAC(+57.02)GVGGYGSR | 72.07 | 2256.0454 | 26 | 0.7 | 753.0229 | 47.29 | 2 | F2:10490 | Fraction\_2\_23092022.raw |  | 1.6936E5 |  |  |  |  |  | 1 | 0 | 1 | 0 | 0 | 0 | 0 | 0 | P13647|K2C5\_HUMAN | Carbamidomethylation | C18:Carbamidomethylation:1000.00 | PEAKS DB |
| LSFNPTQLEEQC(+57.02)HI | 72.06 | 1714.7985 | 14 | 0.0 | 858.4065 | 65.07 | 5 | F5:19163 | Fraction\_5\_23092022.raw | 2.8946E5 | 1.1975E5 | 2.0216E5 | 1.4495E5 | 2.1549E5 | 4.217E5 |  | 6 | 1 | 1 | 1 | 1 | 1 | 1 | 0 | P02754|LACB\_BOVIN | Carbamidomethylation | C12:Carbamidomethylation:1000.00 | PEAKS DB |
| LTALGVLGNINDPEYSARENELR | 71.83 | 2543.2979 | 23 | -1.0 | 848.7723 | 66.42 | 3 | F3:19465 | Fraction\_3\_23092022.raw |  | 1.1883E5 | 3.1282E5 |  | 1.2544E5 |  |  | 3 | 0 | 1 | 1 | 0 | 1 | 0 | 0 | TRINITY\_DN1455\_c0\_g1\_i1.p1 |  |  | PEAKS DB |
| HVGDLGNIEAGDNGVAK | 71.75 | 1664.8118 | 17 | 0.4 | 555.9448 | 36.17 | 6 | F6:6849 | Fraction\_6\_23092022.raw |  |  |  |  |  | 2.6132E5 |  | 1 | 0 | 0 | 0 | 0 | 0 | 1 | 0 | TRINITY\_DN1931\_c0\_g1\_i1.p1:TRINITY\_DN1931\_c0\_g1\_i2.p1:TRINITY\_DN1931\_c0\_g1\_i4.p1 |  |  | PEAKS DB |
| LALDLEIATYRTLLEGEESR | 71.74 | 2291.2009 | 20 | 0.4 | 764.7412 | 82.43 | 1 | F1:26086 | Fraction\_1\_23092022r.raw | 2.5215E5 |  |  |  |  |  |  | 1 | 1 | 0 | 0 | 0 | 0 | 0 | 0 | P04264|K2C1\_HUMAN |  |  | PEAKS DB |
| TNAENEFVTIK | 71.61 | 1264.6299 | 11 | 0.8 | 633.3228 | 44.73 | 3 | F3:9705 | Fraction\_3\_23092022.raw | 4.5718E6 | 3.9495E6 | 5.4204E6 | 1.1702E6 | 8.1968E5 | 1.4631E6 | 6.4716E5 | 7 | 1 | 1 | 1 | 1 | 1 | 1 | 1 | P04264|K2C1\_HUMAN |  |  | PEAKS DB |
| QISNLQQSISDAEQRGENALKDAK | 71.59 | 2642.3259 | 24 | 0.3 | 661.5889 | 61.65 | 1 | F1:16733 | Fraction\_1\_23092022r.raw | 1.5775E6 |  |  |  |  |  |  | 1 | 1 | 0 | 0 | 0 | 0 | 0 | 0 | P04264|K2C1\_HUMAN |  |  | PEAKS DB |
| M(+15.99)IAPILDEIADEYQGKLTVAK | 71.58 | 2333.2188 | 21 | 0.3 | 778.7471 | 78.29 | 2 | F2:24355 | Fraction\_2\_23092022.raw | 2.3234E6 | 2.6165E6 |  | 3.8839E5 |  |  |  | 3 | 1 | 1 | 0 | 1 | 0 | 0 | 0 | P0AA29|THIO\_SALTI:P0AA26|THIO\_ECOL6:P0AA27|THIO\_ECO57:P0AA30|THIO\_SHIFL:P0AA25|THIO\_ECOLI:P0AA28|THIO\_SALTY | Oxidation (M) | M1:Oxidation (M):1000.00 | PEAKS DB |
| WIATTQFQATDAR | 71.57 | 1507.7419 | 13 | 0.4 | 754.8785 | 50.93 | 4 | F4:13047 | Fraction\_4\_23092022.raw |  |  |  | 9.3696E5 |  |  |  | 1 | 0 | 0 | 0 | 1 | 0 | 0 | 0 | TRINITY\_DN1113\_c0\_g1\_i95.p1:TRINITY\_DN1113\_c0\_g1\_i47.p1:TRINITY\_DN1113\_c0\_g1\_i59.p1:TRINITY\_DN1113\_c0\_g1\_i53.p1:TRINITY\_DN1113\_c0\_g1\_i73.p1:TRINITY\_DN1113\_c0\_g1\_i6.p1:TRINITY\_DN1113\_c0\_g1\_i12.p1 |  |  | PEAKS DB |
| GGSGGSHGGGSGFGGESGGSYGGGEEASGSGGGYGGGSGKSSHS | 71.53 | 3620.4292 | 44 | 0.3 | 906.1149 | 23.51 | 7 | F7:3707 | Fraction\_7\_23092022.raw |  |  |  |  |  |  | 2.8278E5 | 1 | 0 | 0 | 0 | 0 | 0 | 0 | 1 | P35527|K1C9\_HUMAN |  |  | PEAKS DB |
| TLNNKFASFIDKVR | 71.43 | 1651.9045 | 14 | -0.2 | 551.6420 | 51.36 | 2 | F2:12305 | Fraction\_2\_23092022.raw | 2.671E5 | 1.8567E6 | 2.1296E5 |  |  | 8.2338E5 | 3.789E5 | 6 | 1 | 2 | 1 | 0 | 0 | 1 | 1 | P35908|K22E\_HUMAN:P48668|K2C6C\_HUMAN:P13647|K2C5\_HUMAN:Q3TTY5|K22E\_MOUSE:P05787|K2C8\_HUMAN |  |  | PEAKS DB |
| SLNNQFASFIDK | 71.31 | 1382.6830 | 12 | -0.3 | 692.3486 | 63.00 | 1 | F1:17345 | Fraction\_1\_23092022r.raw | 5.9946E6 | 5.1364E5 | 8.2546E5 | 3.0075E5 |  | 3.8456E5 | 2.7708E5 | 6 | 1 | 1 | 1 | 1 | 0 | 1 | 1 | P04264|K2C1\_HUMAN |  |  | PEAKS DB |
| SGYRSGGGFSSGSAGIINYQRR | 71.30 | 2276.1045 | 22 | 1.2 | 570.0341 | 36.14 | 7 | F7:9004 | Fraction\_7\_23092022.raw |  |  |  |  |  |  | 6.0926E5 | 1 | 0 | 0 | 0 | 0 | 0 | 0 | 1 | P04264|K2C1\_HUMAN |  |  | PEAKS DB |
| VGPNLYGVYGRK | 71.26 | 1321.7142 | 12 | -0.2 | 441.5786 | 39.26 | 7 | F7:10402 | Fraction\_7\_23092022.raw |  |  |  |  |  | 1.6484E5 | 1.5958E5 | 2 | 0 | 0 | 0 | 0 | 0 | 1 | 1 | P00038|CYC\_APIME |  |  | PEAKS DB |
| NVQDAIADAEQRGEHALKDAR | 71.22 | 2306.1362 | 21 | 0.4 | 577.5416 | 53.98 | 6 | F6:14877 | Fraction\_6\_23092022.raw | 1.9881E6 | 1.2033E6 | 5.2445E5 |  |  | 8.7336E5 |  | 4 | 1 | 1 | 1 | 0 | 0 | 1 | 0 | P35908|K22E\_HUMAN |  |  | PEAKS DB |
| KELPLVKDLYVR | 71.14 | 1471.8762 | 12 | 0.4 | 368.9765 | 47.77 | 7 | F7:14316 | Fraction\_7\_23092022.raw |  |  | 9.6471E5 | 5.0971E5 | 3.2805E6 |  | 4.2881E7 | 5 | 0 | 0 | 1 | 1 | 1 | 0 | 2 | TRINITY\_DN3450\_c0\_g1\_i51.p1 |  |  | PEAKS DB |
| LSFNPTQLEEQC(+57.02)HV | 71.06 | 1700.7828 | 14 | 0.1 | 851.3987 | 61.62 | 5 | F5:17618 | Fraction\_5\_23092022.raw |  |  |  |  | 5.3867E5 | 3.6218E5 |  | 2 | 0 | 0 | 0 | 0 | 1 | 1 | 0 | P02755|LACB\_BUBBU | Carbamidomethylation | C12:Carbamidomethylation:1000.00 | PEAKS DB |
| SLVFDNIIEGYSKR | 70.98 | 1639.8569 | 14 | 0.5 | 820.9362 | 64.81 | 6 | F6:19710 | Fraction\_6\_23092022.raw |  |  |  |  | 6.1162E4 | 7.9442E6 | 2.1201E6 | 5 | 0 | 0 | 0 | 0 | 1 | 2 | 2 | TRINITY\_DN33395\_c0\_g1\_i1.p1 |  |  | PEAKS DB |
| VTLWIHPFINK | 70.95 | 1366.7761 | 11 | 0.2 | 684.3955 | 64.95 | 1 | F1:18212 | Fraction\_1\_23092022r.raw | 8.5965E7 |  |  |  |  |  |  | 2 | 2 | 0 | 0 | 0 | 0 | 0 | 0 | TRINITY\_DN4276\_c0\_g1\_i1.p1 |  |  | PEAKS DB |
| SPLVTDISFK | 70.92 | 1105.6019 | 10 | 0.6 | 553.8086 | 57.58 | 4 | F4:16046 | Fraction\_4\_23092022.raw | 1.2152E5 |  | 2.0747E5 | 7.675E5 |  | 5.2151E4 |  | 4 | 1 | 0 | 1 | 1 | 0 | 1 | 0 | TRINITY\_DN4312\_c0\_g1\_i15.p1:TRINITY\_DN4312\_c0\_g1\_i7.p1:TRINITY\_DN4312\_c0\_g1\_i9.p1 |  |  | PEAKS DB |
| LSTNSDVINVSR | 70.90 | 1303.6732 | 12 | 1.3 | 652.8447 | 39.42 | 1 | F1:6965 | Fraction\_1\_23092022r.raw | 7.8193E7 |  |  |  |  | 2.1369E5 |  | 2 | 1 | 0 | 0 | 0 | 0 | 1 | 0 | TRINITY\_DN4276\_c0\_g1\_i1.p1 |  |  | PEAKS DB |
| VFDLGIISYGSTC(+57.02)ADNSPGVNTK | 70.84 | 2414.1423 | 23 | -0.9 | 1208.0774 | 72.86 | 3 | F3:22346 | Fraction\_3\_23092022.raw |  |  | 1.6253E6 |  |  |  |  | 3 | 0 | 0 | 3 | 0 | 0 | 0 | 0 | TRINITY\_DN1494\_c0\_g1\_i8.p1 | Carbamidomethylation | C13:Carbamidomethylation:1000.00 | PEAKS DB |
| LNDLEDALQQAKEDLAR | 70.67 | 1940.9802 | 17 | 1.9 | 971.4992 | 72.49 | 1 | F1:21615 | Fraction\_1\_23092022r.raw | 1.7355E6 | 1.5073E5 | 1.2303E5 |  |  | 1.4633E5 |  | 5 | 2 | 1 | 1 | 0 | 0 | 1 | 0 | P04264|K2C1\_HUMAN |  |  | PEAKS DB |
| IRLENEIQTYR | 70.57 | 1433.7627 | 11 | 0.0 | 478.9282 | 40.46 | 3 | F3:7815 | Fraction\_3\_23092022.raw | 4.5903E6 | 8.1016E5 | 1.002E6 | 2.8274E5 | 2.5433E5 | 3.8169E5 | 1.8818E5 | 7 | 1 | 1 | 1 | 1 | 1 | 1 | 1 | P13645|K1C10\_HUMAN |  |  | PEAKS DB |
| ALVIHAKPDDFGR | 70.56 | 1437.7728 | 13 | -0.4 | 360.4503 | 34.70 | 6 | F6:6275 | Fraction\_6\_23092022.raw |  |  |  |  |  | 6.6635E4 |  | 1 | 0 | 0 | 0 | 0 | 0 | 1 | 0 | TRINITY\_DN16204\_c0\_g1\_i2.p1 |  |  | PEAKS DB |
| NVDNLKINKDISIDLVR | 70.50 | 1968.1003 | 17 | 0.3 | 657.0409 | 50.83 | 6 | F6:13384 | Fraction\_6\_23092022.raw |  |  | 9.5454E4 | 2.26E5 | 1.2458E6 | 1.9023E7 | 3.2093E5 | 12 | 0 | 0 | 1 | 1 | 2 | 7 | 1 | Q5D7H4|HUGAB\_VESVU |  |  | PEAKS DB |
| TDGTGAFPSLLKGGPGSR | 70.42 | 1716.8794 | 18 | -1.3 | 573.2997 | 51.53 | 5 | F5:13065 | Fraction\_5\_23092022.raw |  |  |  |  | 1.8262E5 |  |  | 1 | 0 | 0 | 0 | 0 | 1 | 0 | 0 | TRINITY\_DN254\_c0\_g1\_i1.p1 |  |  | PEAKS DB |
| RDPNYFIHKDATNLGYYVK | 70.24 | 2313.1541 | 19 | 0.5 | 579.2961 | 46.37 | 1 | F1:9935 | Fraction\_1\_23092022r.raw | 9.6459E5 |  |  |  |  |  |  | 1 | 1 | 0 | 0 | 0 | 0 | 0 | 0 | TRINITY\_DN1709\_c0\_g1\_i4.p1:TRINITY\_DN1709\_c0\_g1\_i5.p1:TRINITY\_DN1709\_c0\_g1\_i6.p1:TRINITY\_DN1709\_c0\_g1\_i8.p1 |  |  | PEAKS DB |
| LKLLEEIGIDSFK | 70.20 | 1503.8547 | 13 | 2.7 | 502.2935 | 71.45 | 1 | F1:21117 | Fraction\_1\_23092022r.raw | 1.1777E7 |  |  |  |  |  |  | 2 | 2 | 0 | 0 | 0 | 0 | 0 | 0 | TRINITY\_DN4276\_c0\_g1\_i1.p1 |  |  | PEAKS DB |
| SVGNYIATVTK | 70.20 | 1151.6187 | 11 | 0.5 | 576.8169 | 44.60 | 7 | F7:12832 | Fraction\_7\_23092022.raw | 3.8953E5 | 2.632E5 | 9.612E6 | 2.0408E7 | 1.0122E7 | 3.1536E7 | 4.1914E7 | 7 | 1 | 1 | 1 | 1 | 1 | 1 | 1 | TRINITY\_DN2326\_c0\_g1\_i13.p1 |  |  | PEAKS DB |
| NSAGEDSC(+57.02)TRNVLHTQSSDILR | 70.20 | 2459.1460 | 22 | 0.4 | 820.7229 | 37.53 | 1 | F1:6187 | Fraction\_1\_23092022r.raw | 1.204E6 |  |  |  |  |  |  | 1 | 1 | 0 | 0 | 0 | 0 | 0 | 0 | TRINITY\_DN4276\_c0\_g1\_i1.p1 | Carbamidomethylation | C8:Carbamidomethylation:1000.00 | PEAKS DB |
| AWADEVKDYQYGSPNK | 70.19 | 1869.8533 | 16 | -0.1 | 935.9338 | 45.49 | 6 | F6:11027 | Fraction\_6\_23092022.raw |  |  |  | 2.1122E5 |  | 7.5074E5 |  | 3 | 0 | 0 | 0 | 1 | 0 | 2 | 0 | TRINITY\_DN6370\_c0\_g1\_i1.p1 |  |  | PEAKS DB |
| SGGGGGGGLGSGGSIRSSYSR | 70.07 | 1811.8510 | 21 | 1.1 | 604.9583 | 25.90 | 7 | F7:4537 | Fraction\_7\_23092022.raw |  |  |  |  |  |  | 3.6723E4 | 1 | 0 | 0 | 0 | 0 | 0 | 0 | 1 | P35527|K1C9\_HUMAN |  |  | PEAKS DB |
| LSTNSDVINVSRIVKDSDAK | 70.04 | 2160.1387 | 20 | 4.0 | 541.0441 | 47.98 | 1 | F1:10647 | Fraction\_1\_23092022r.raw | 2.2844E7 | 6.6757E5 |  |  |  |  |  | 4 | 2 | 2 | 0 | 0 | 0 | 0 | 0 | TRINITY\_DN4276\_c0\_g1\_i1.p1 |  |  | PEAKS DB |
| YVSGSFHYFR | 69.80 | 1261.5880 | 10 | 1.3 | 421.5371 | 42.55 | 1 | F1:8279 | Fraction\_1\_23092022r.raw | 5.3828E5 | 1.3946E6 |  |  |  |  |  | 3 | 1 | 2 | 0 | 0 | 0 | 0 | 0 | TRINITY\_DN11986\_c0\_g1\_i3.p1 |  |  | PEAKS DB |
| YQHWPIQHM(+15.99)YYEEEPYVPTHPVNMAVAER | 69.71 | 3629.6497 | 29 | -0.4 | 726.9369 | 54.00 | 1 | F1:13328 | Fraction\_1\_23092022r.raw | 7.4695E6 |  |  |  |  |  |  | 2 | 2 | 0 | 0 | 0 | 0 | 0 | 0 | TRINITY\_DN4276\_c0\_g1\_i1.p1 | Oxidation (M) | M9:Oxidation (M):120.73 | PEAKS DB |
| YIATSKFEPTYAR | 69.62 | 1545.7827 | 13 | 0.1 | 516.2682 | 38.21 | 6 | F6:7752 | Fraction\_6\_23092022.raw |  |  |  |  |  | 2.7447E5 |  | 1 | 0 | 0 | 0 | 0 | 0 | 1 | 0 | TRINITY\_DN11796\_c0\_g1\_i1.p1 |  |  | PEAKS DB |
| IERNIKNAVVTIEINQNVATLR | 69.61 | 2507.4182 | 22 | -0.2 | 627.8617 | 54.56 | 1 | F1:13593 | Fraction\_1\_23092022r.raw | 7.5001E5 |  |  |  |  |  |  | 1 | 1 | 0 | 0 | 0 | 0 | 0 | 0 | TRINITY\_DN4276\_c0\_g1\_i1.p1 |  |  | PEAKS DB |
| TGDILQFDVIK | 69.51 | 1247.6761 | 11 | 0.3 | 624.8455 | 69.45 | 4 | F4:21363 | Fraction\_4\_23092022.raw |  |  |  | 1.9069E5 |  | 2.2556E5 | 6.1063E5 | 3 | 0 | 0 | 0 | 1 | 0 | 1 | 1 | TRINITY\_DN3450\_c0\_g1\_i51.p1 |  |  | PEAKS DB |
| AEAESLYQSKYEELQITAGRHGDSVR | 69.50 | 2936.4263 | 26 | 0.6 | 588.2929 | 44.92 | 1 | F1:9313 | Fraction\_1\_23092022r.raw | 1.1507E5 | 3.7507E5 |  |  |  |  |  | 3 | 1 | 2 | 0 | 0 | 0 | 0 | 0 | P04264|K2C1\_HUMAN |  |  | PEAKS DB |
| MLVQQYNVPMANIR | 69.46 | 1675.8538 | 14 | 1.2 | 838.9352 | 60.90 | 1 | F1:16429 | Fraction\_1\_23092022r.raw | 4.6145E5 |  |  |  |  |  |  | 1 | 1 | 0 | 0 | 0 | 0 | 0 | 0 |  |  |  | PEAKS DB |
| ASGLTNAEKLEILK | 69.29 | 1485.8402 | 14 | 0.9 | 496.2878 | 47.09 | 7 | F7:13989 | Fraction\_7\_23092022.raw |  |  | 2.9631E6 | 2.2338E7 | 1.8351E6 | 9.7441E6 | 3.2738E7 | 10 | 0 | 0 | 2 | 2 | 2 | 2 | 2 | TRINITY\_DN6370\_c0\_g1\_i1.p1 |  |  | PEAKS DB |
| AGAESGWDFSGR | 69.21 | 1238.5316 | 12 | 1.1 | 620.2737 | 47.46 | 1 | F1:10445 | Fraction\_1\_23092022r.raw | 6.326E4 |  |  |  |  |  |  | 1 | 1 | 0 | 0 | 0 | 0 | 0 | 0 | TRINITY\_DN10256\_c0\_g10\_i1.p1:TRINITY\_DN10256\_c0\_g5\_i4.p1 |  |  | PEAKS DB |
| VSAFNNWILQK | 69.07 | 1318.7034 | 11 | 0.8 | 660.3595 | 64.61 | 6 | F6:19607 | Fraction\_6\_23092022.raw | 3.9554E5 | 7.1007E4 | 1.7413E6 | 3.7565E5 | 2.0316E5 | 2.2757E7 | 5.5819E6 | 7 | 1 | 1 | 1 | 1 | 1 | 1 | 1 | TRINITY\_DN33420\_c0\_g1\_i1.p1 |  |  | PEAKS DB |
| FLEQQNQVLQTK | 69.06 | 1474.7780 | 12 | 0.5 | 738.3966 | 42.12 | 3 | F3:8546 | Fraction\_3\_23092022.raw | 8.0973E6 | 7.8535E5 | 1.0847E6 | 3.5575E5 | 2.39E5 | 3.7556E5 | 9.8393E4 | 7 | 1 | 1 | 1 | 1 | 1 | 1 | 1 | P04264|K2C1\_HUMAN:P35908|K22E\_HUMAN |  |  | PEAKS DB |
| SVGNYIATVTKTLVQK | 68.94 | 1720.9723 | 16 | 0.4 | 574.6649 | 65.51 | 5 | F5:19322 | Fraction\_5\_23092022.raw |  |  | 9.1546E4 | 1.5229E6 | 1.0203E6 | 1.6382E6 | 8.7521E6 | 8 | 0 | 0 | 1 | 2 | 1 | 2 | 2 | TRINITY\_DN2326\_c0\_g1\_i13.p1 |  |  | PEAKS DB |
| GTNYLADVFEK | 68.92 | 1255.6084 | 11 | 1.0 | 628.8121 | 64.32 | 4 | F4:19080 | Fraction\_4\_23092022.raw | 2.8908E5 | 9.93E4 | 1.1071E5 | 1.1403E5 | 4.9608E4 | 6.224E4 |  | 6 | 1 | 1 | 1 | 1 | 1 | 1 | 0 | P31151|S10A7\_HUMAN |  |  | PEAKS DB |
| AVGGLGKLGKDAVEDLESVGK | 68.91 | 2041.1055 | 21 | 0.5 | 681.3761 | 60.51 | 2 | F2:16466 | Fraction\_2\_23092022.raw | 7.4799E4 | 2.5245E5 |  |  |  |  |  | 2 | 1 | 1 | 0 | 0 | 0 | 0 | 0 | P81605|DCD\_HUMAN |  |  | PEAKS DB |
| SVGNYIATVTKTLVQKYNVPM(+15.99)ANIR | 68.91 | 2795.5002 | 25 | 0.2 | 699.8824 | 73.56 | 4 | F4:23202 | Fraction\_4\_23092022.raw |  |  |  | 7.4542E5 | 7.1489E5 | 7.7341E5 |  | 3 | 0 | 0 | 0 | 1 | 1 | 1 | 0 | TRINITY\_DN2326\_c0\_g1\_i13.p1 | Oxidation (M) | M21:Oxidation (M):1000.00 | PEAKS DB |
| AVEYFTEC(+57.02)IR | 68.79 | 1286.5964 | 10 | 0.5 | 644.3058 | 52.34 | 4 | F4:13677 | Fraction\_4\_23092022.raw |  |  |  | 7.516E5 | 5.0889E5 | 3.7392E5 | 2.1853E6 | 4 | 0 | 0 | 0 | 1 | 1 | 1 | 1 | TRINITY\_DN2326\_c0\_g1\_i13.p1 | Carbamidomethylation | C8:Carbamidomethylation:1000.00 | PEAKS DB |
| IDAVPHLFEREDLHDEPR | 68.72 | 2187.0708 | 18 | 0.7 | 438.4218 | 47.06 | 2 | F2:10334 | Fraction\_2\_23092022.raw | 7.6093E5 | 3.8278E6 |  |  | 4.9871E5 |  |  | 6 | 2 | 2 | 0 | 0 | 2 | 0 | 0 | TRINITY\_DN1342\_c0\_g1\_i23.p1:TRINITY\_DN1342\_c0\_g1\_i54.p1 |  |  | PEAKS DB |
| LNIDQNPGTAPKYGIR | 68.53 | 1755.9268 | 16 | 0.2 | 586.3163 | 40.14 | 4 | F4:8222 | Fraction\_4\_23092022.raw | 9.9323E5 | 4.0151E6 | 2.1003E5 | 4.5004E5 |  |  |  | 6 | 2 | 2 | 1 | 1 | 0 | 0 | 0 | P0AA29|THIO\_SALTI:P0AA26|THIO\_ECOL6:P0AA27|THIO\_ECO57:P0AA30|THIO\_SHIFL:P0AA25|THIO\_ECOLI:P0AA28|THIO\_SALTY |  |  | PEAKS DB |
| SQYEQLAEQNRKDAEAWFNEK | 68.52 | 2583.1990 | 21 | 0.5 | 646.8074 | 53.68 | 1 | F1:13201 | Fraction\_1\_23092022r.raw | 1.3001E6 |  |  |  |  |  |  | 2 | 2 | 0 | 0 | 0 | 0 | 0 | 0 | P13645|K1C10\_HUMAN |  |  | PEAKS DB |
| DQIVDLTVGNNK | 68.43 | 1314.6780 | 12 | 0.4 | 658.3466 | 50.10 | 3 | F3:12123 | Fraction\_3\_23092022.raw | 5.3699E5 | 2.0511E5 | 7.081E5 |  |  |  |  | 3 | 1 | 1 | 1 | 0 | 0 | 0 | 0 | P35527|K1C9\_HUMAN |  |  | PEAKS DB |
| GIPTLLLFKNGEVAATK | 68.38 | 1771.0243 | 17 | -0.1 | 886.5193 | 66.23 | 1 | F1:18826 | Fraction\_1\_23092022r.raw | 1.3031E5 | 4.627E6 | 2.6873E5 |  |  |  |  | 4 | 1 | 2 | 1 | 0 | 0 | 0 | 0 | P0AA29|THIO\_SALTI:P0AA26|THIO\_ECOL6:P0AA27|THIO\_ECO57:P0AA30|THIO\_SHIFL:P0AA25|THIO\_ECOLI:P0AA28|THIO\_SALTY |  |  | PEAKS DB |
| VLLNLGDSVTTDHISPAGSIAR | 68.25 | 2235.1858 | 22 | 1.6 | 746.0704 | 61.18 | 1 | F1:16561 | Fraction\_1\_23092022r.raw | 2.0736E5 |  |  |  |  |  |  | 1 | 1 | 0 | 0 | 0 | 0 | 0 | 0 | TRINITY\_DN747\_c0\_g1\_i8.p1:TRINITY\_DN747\_c0\_g1\_i4.p1:TRINITY\_DN747\_c0\_g1\_i7.p1:TRINITY\_DN747\_c0\_g1\_i1.p1 |  |  | PEAKS DB |
| VSAFNNWILQKISK | 68.22 | 1646.9144 | 14 | -0.2 | 549.9786 | 65.45 | 6 | F6:20009 | Fraction\_6\_23092022.raw |  |  |  |  |  | 1.0245E6 | 2.1406E6 | 3 | 0 | 0 | 0 | 0 | 0 | 2 | 1 | TRINITY\_DN33420\_c0\_g1\_i1.p1 |  |  | PEAKS DB |
| LLEEIGIDSFK | 68.09 | 1262.6758 | 11 | 1.8 | 632.3463 | 68.08 | 1 | F1:19620 | Fraction\_1\_23092022r.raw | 3.418E7 | 1.6687E5 |  |  |  |  |  | 2 | 1 | 1 | 0 | 0 | 0 | 0 | 0 | TRINITY\_DN4276\_c0\_g1\_i1.p1 |  |  | PEAKS DB |
| ISSVLAGGSC(+57.02)RAPSTYGGGLSVSSSR | 68.08 | 2512.2339 | 26 | 0.2 | 838.4187 | 45.64 | 7 | F7:13350 | Fraction\_7\_23092022.raw | 1.0175E5 |  |  |  |  |  | 3.6841E5 | 2 | 1 | 0 | 0 | 0 | 0 | 0 | 1 | P02533|K1C14\_HUMAN | Carbamidomethylation | C10:Carbamidomethylation:1000.00 | PEAKS DB |
| TIVEEVQDGKVISSREQVHQTTR | 68.04 | 2638.3674 | 23 | -0.7 | 660.5987 | 34.97 | 7 | F7:8489 | Fraction\_7\_23092022.raw |  |  |  |  |  |  | 1.3888E5 | 1 | 0 | 0 | 0 | 0 | 0 | 0 | 1 | Q6IFU8|K1C17\_RAT:Q9QWL7|K1C17\_MOUSE |  |  | PEAKS DB |
| FTSVNLHSLSR | 68.01 | 1259.6622 | 11 | -0.3 | 420.8946 | 40.69 | 4 | F4:8484 | Fraction\_4\_23092022.raw |  |  |  | 1.1207E5 |  |  |  | 2 | 0 | 0 | 0 | 2 | 0 | 0 | 0 | TRINITY\_DN27415\_c0\_g1\_i1.p1 |  |  | PEAKS DB |
| TNAENEFVTIKK | 68.00 | 1392.7249 | 12 | -0.5 | 465.2487 | 33.01 | 7 | F7:7601 | Fraction\_7\_23092022.raw |  |  |  |  |  | 9.7181E5 | 4.8108E5 | 4 | 0 | 0 | 0 | 0 | 0 | 2 | 2 | P04264|K2C1\_HUMAN |  |  | PEAKS DB |
| TVGKYIAEFSK | 67.90 | 1241.6655 | 11 | -0.4 | 621.8398 | 40.96 | 5 | F5:8354 | Fraction\_5\_23092022.raw |  |  |  |  | 9.2439E5 | 7.046E5 |  | 4 | 0 | 0 | 0 | 0 | 3 | 1 | 0 | TRINITY\_DN2326\_c0\_g1\_i12.p1 |  |  | PEAKS DB |
| FNPC(+57.02)PYSDDTVKM(+15.99)IILTR | 67.84 | 2185.0547 | 18 | 0.7 | 729.3594 | 61.78 | 3 | F3:17358 | Fraction\_3\_23092022.raw |  |  | 2.5328E7 |  |  | 9.1102E5 | 2.8195E5 | 4 | 0 | 0 | 2 | 0 | 0 | 1 | 1 | TRINITY\_DN2326\_c0\_g1\_i13.p1:A0A0M3KKW3|PA1\_VESBA | Carbamidomethylation; Oxidation (M) | C4:Carbamidomethylation:1000.00;M13:Oxidation (M):1000.00 | PEAKS DB |
| AVKYFTEC(+57.02)IR | 67.79 | 1285.6488 | 10 | 1.2 | 429.5574 | 38.78 | 4 | F4:7597 | Fraction\_4\_23092022.raw |  |  | 2.6071E6 | 6.2175E6 | 4.4092E6 | 1.8308E6 | 1.2961E6 | 10 | 0 | 0 | 2 | 2 | 2 | 2 | 2 |  | Carbamidomethylation | C8:Carbamidomethylation:1000.00 | PEAKS DB |
| GIAYVNGYNLGR | 67.77 | 1295.6622 | 12 | 0.2 | 648.8385 | 50.83 | 1 | F1:11932 | Fraction\_1\_23092022r.raw | 2.9059E5 |  |  |  |  |  |  | 1 | 1 | 0 | 0 | 0 | 0 | 0 | 0 | TRINITY\_DN11986\_c0\_g1\_i3.p1 |  |  | PEAKS DB |
| NLDLDSIIAEVK | 67.73 | 1328.7188 | 12 | -0.3 | 665.3665 | 80.82 | 4 | F4:26420 | Fraction\_4\_23092022.raw | 1.3444E7 | 1.1783E7 | 2.3503E7 | 6.3504E6 | 3.9084E6 | 1.1367E7 | 9.0701E6 | 8 | 1 | 1 | 2 | 1 | 1 | 1 | 1 | P35908|K22E\_HUMAN:P48668|K2C6C\_HUMAN:P13647|K2C5\_HUMAN |  |  | PEAKS DB |
| GGGGGGFRGFSSGSAVVSGGSR | 67.66 | 1898.8983 | 22 | -0.6 | 633.9730 | 38.16 | 6 | F6:7760 | Fraction\_6\_23092022.raw | 7.754E4 | 2.0655E5 |  |  | 8.7993E4 | 1.2583E5 | 1.1908E5 | 5 | 1 | 1 | 0 | 0 | 1 | 1 | 1 | P35908|K22E\_HUMAN |  |  | PEAKS DB |
| TVRPISIIQANK | 67.65 | 1338.7983 | 12 | -0.8 | 447.2730 | 38.73 | 6 | F6:8023 | Fraction\_6\_23092022.raw |  |  |  |  |  | 1.2631E6 | 1.822E5 | 3 | 0 | 0 | 0 | 0 | 0 | 2 | 1 | TRINITY\_DN7688\_c0\_g2\_i1.p1:TRINITY\_DN7688\_c0\_g2\_i2.p1 |  |  | PEAKS DB |
| FLDEPSANRPDLPPDDYDTLDHIYTK | 67.57 | 3046.4194 | 26 | 1.2 | 1016.4817 | 61.15 | 1 | F1:16482 | Fraction\_1\_23092022r.raw | 4.5112E5 | 3.2929E7 |  |  |  |  |  | 3 | 1 | 2 | 0 | 0 | 0 | 0 | 0 | TRINITY\_DN10047\_c0\_g1\_i1.p1 |  |  | PEAKS DB |
| SLDLDSIIAEVK | 67.53 | 1301.7078 | 12 | -0.2 | 651.8610 | 80.92 | 4 | F4:26543 | Fraction\_4\_23092022.raw | 8.2236E7 | 5.1234E7 | 7.5638E7 | 2.6564E7 | 1.4738E7 | 3.5929E7 | 2.2646E7 | 15 | 2 | 3 | 4 | 1 | 1 | 3 | 1 | P04264|K2C1\_HUMAN |  |  | PEAKS DB |
| SSDSTATDRDYTYLDHIYTK | 67.49 | 2351.0554 | 20 | 0.1 | 588.7712 | 46.79 | 2 | F2:10235 | Fraction\_2\_23092022.raw | 5.662E5 | 3.4895E6 |  |  | 3.6296E5 |  |  | 4 | 2 | 1 | 0 | 0 | 1 | 0 | 0 | TRINITY\_DN1342\_c0\_g1\_i23.p1:TRINITY\_DN1342\_c0\_g1\_i54.p1 |  |  | PEAKS DB |
| KVQELRLGKYSEIIGLDPAGPSFK | 67.40 | 2644.4587 | 24 | -0.6 | 529.8987 | 58.78 | 7 | F7:20738 | Fraction\_7\_23092022.raw |  |  |  | 2.1944E5 |  |  | 2.406E6 | 2 | 0 | 0 | 0 | 1 | 0 | 0 | 1 |  |  |  | PEAKS DB |
| TFVDLSQKDDPEITLSKFK | 67.38 | 2210.1470 | 19 | 0.4 | 553.5443 | 58.45 | 2 | F2:15512 | Fraction\_2\_23092022.raw |  | 2.2053E5 |  |  |  |  |  | 1 | 0 | 1 | 0 | 0 | 0 | 0 | 0 | TRINITY\_DN10256\_c0\_g10\_i1.p1:TRINITY\_DN10256\_c0\_g5\_i4.p1 |  |  | PEAKS DB |
| ALEESNYELEGKIKEWYEK | 67.36 | 2357.1426 | 19 | 1.8 | 590.2940 | 56.44 | 1 | F1:14459 | Fraction\_1\_23092022r.raw | 1.1943E6 |  |  |  |  |  |  | 2 | 2 | 0 | 0 | 0 | 0 | 0 | 0 | P13645|K1C10\_HUMAN |  |  | PEAKS DB |
| HDFYTLNTIKNHNEFKK | 67.24 | 2148.0752 | 17 | 0.2 | 538.0262 | 33.43 | 7 | F7:7789 | Fraction\_7\_23092022.raw |  |  |  |  |  |  | 8.4028E6 | 2 | 0 | 0 | 0 | 0 | 0 | 0 | 2 |  |  |  | PEAKS DB |
| TLVQKYNVPM(+15.99)ANIR | 67.18 | 1661.8923 | 14 | 0.1 | 554.9714 | 40.24 | 7 | F7:10847 | Fraction\_7\_23092022.raw |  |  |  | 1.6047E6 | 1.5365E6 | 3.9489E6 | 1.2025E7 | 11 | 0 | 0 | 0 | 2 | 2 | 3 | 4 | TRINITY\_DN2326\_c0\_g1\_i13.p1 | Oxidation (M) | M10:Oxidation (M):1000.00 | PEAKS DB |
| LVNEVTEFAK | 67.15 | 1148.6077 | 10 | -0.6 | 575.3108 | 47.29 | 1 | F1:10351 | Fraction\_1\_23092022r.raw | 6.3091E5 |  |  |  |  |  |  | 1 | 1 | 0 | 0 | 0 | 0 | 0 | 0 | P02768|ALBU\_HUMAN |  |  | PEAKS DB |
| GLGTDEDSLIEIIC(+57.02)SR | 67.14 | 1776.8563 | 16 | -0.6 | 889.4349 | 80.94 | 1 | F1:25390 | Fraction\_1\_23092022r.raw | 1.4108E5 |  |  |  |  |  |  | 1 | 1 | 0 | 0 | 0 | 0 | 0 | 0 | P19620|ANXA2\_PIG:A2SW69|ANXA2\_SHEEP:P04272|ANXA2\_BOVIN:P07356|ANXA2\_MOUSE:Q6TEQ7|ANXA2\_CANLF:Q2Q1M6|ANXA2\_CEREL:C0HJG9|ANXA2\_MESAU:Q5R5A0|ANXA2\_PONAB:P07355|ANXA2\_HUMAN:Q07936|ANXA2\_RAT | Carbamidomethylation | C14:Carbamidomethylation:1000.00 | PEAKS DB |
| FGGFGGPGGVGGLGGPGGFGPGGYPGGIHEVSVNQSLLQPLNVKVDPEIQNVK | 67.12 | 5113.6050 | 53 | 0.3 | 1279.4089 | 80.58 | 1 | F1:25233 | Fraction\_1\_23092022r.raw | 6.5682E5 | 1.1101E6 |  |  |  |  |  | 2 | 1 | 1 | 0 | 0 | 0 | 0 | 0 | P35908|K22E\_HUMAN |  |  | PEAKS DB |
| VAGILC(+57.02)FSR | 67.10 | 1021.5378 | 9 | 0.8 | 511.7766 | 56.47 | 4 | F4:15555 | Fraction\_4\_23092022.raw |  |  |  | 1.0966E5 | 1.0968E5 |  |  | 2 | 0 | 0 | 0 | 1 | 1 | 0 | 0 | TRINITY\_DN4312\_c0\_g1\_i15.p1:TRINITY\_DN4312\_c0\_g1\_i7.p1:TRINITY\_DN4312\_c0\_g1\_i9.p1 | Carbamidomethylation | C6:Carbamidomethylation:1000.00 | PEAKS DB |
| SLDKLETLDLSNNR | 66.87 | 1616.8369 | 14 | 0.8 | 539.9534 | 48.83 | 6 | F6:12520 | Fraction\_6\_23092022.raw |  |  | 8.0244E4 |  |  | 2.153E5 |  | 2 | 0 | 0 | 1 | 0 | 0 | 1 | 0 | TRINITY\_DN2938\_c0\_g1\_i1.p1 |  |  | PEAKS DB |
| M(+15.99)IAPILDEIADEYQGKLTVAKLNIDQNPGTAPK | 66.85 | 3581.8650 | 33 | -0.6 | 896.4730 | 78.74 | 2 | F2:24625 | Fraction\_2\_23092022.raw |  | 7.7061E5 |  |  |  |  |  | 1 | 0 | 1 | 0 | 0 | 0 | 0 | 0 | P0AA29|THIO\_SALTI:P0AA26|THIO\_ECOL6:P0AA27|THIO\_ECO57:P0AA30|THIO\_SHIFL:P0AA25|THIO\_ECOLI:P0AA28|THIO\_SALTY | Oxidation (M) | M1:Oxidation (M):1000.00 | PEAKS DB |
| AVEYLTEC(+57.02)IR | 66.81 | 1252.6122 | 10 | -0.2 | 627.3132 | 49.19 | 7 | F7:15094 | Fraction\_7\_23092022.raw |  |  | 1.079E6 | 6.0394E5 |  | 6.2548E5 | 1.1357E6 | 4 | 0 | 0 | 1 | 1 | 0 | 1 | 1 | TRINITY\_DN2326\_c0\_g1\_i12.p1 | Carbamidomethylation | C8:Carbamidomethylation:1000.00 | PEAKS DB |
| EIALSAISYLK | 66.78 | 1206.6859 | 11 | 0.7 | 604.3506 | 68.54 | 5 | F5:20709 | Fraction\_5\_23092022.raw | 6.3127E5 | 1.8624E6 | 7.6118E5 | 9.3599E5 | 1.4628E6 | 6.7308E4 |  | 6 | 1 | 1 | 1 | 1 | 1 | 1 | 0 | TRINITY\_DN10256\_c0\_g10\_i1.p1:TRINITY\_DN10256\_c0\_g5\_i4.p1 |  |  | PEAKS DB |
| AEFAEVSKLVTDLTK | 66.69 | 1649.8876 | 15 | 0.6 | 550.9702 | 73.71 | 1 | F1:22169 | Fraction\_1\_23092022r.raw | 1.1675E5 |  |  |  |  |  |  | 1 | 1 | 0 | 0 | 0 | 0 | 0 | 0 | P02768|ALBU\_HUMAN |  |  | PEAKS DB |
| DATNLGYYVK | 66.64 | 1142.5608 | 10 | 0.5 | 572.2880 | 48.50 | 1 | F1:10872 | Fraction\_1\_23092022r.raw | 3.3709E6 | 5.0668E5 |  |  |  |  |  | 2 | 1 | 1 | 0 | 0 | 0 | 0 | 0 | TRINITY\_DN1709\_c0\_g1\_i4.p1:TRINITY\_DN1709\_c0\_g1\_i5.p1:TRINITY\_DN1709\_c0\_g1\_i6.p1:TRINITY\_DN1709\_c0\_g1\_i8.p1 |  |  | PEAKS DB |
| SFMDSNDDGVGDLK | 66.57 | 1498.6245 | 14 | 0.6 | 750.3200 | 52.27 | 1 | F1:12582 | Fraction\_1\_23092022r.raw | 1.6384E5 |  |  |  |  |  |  | 1 | 1 | 0 | 0 | 0 | 0 | 0 | 0 | TRINITY\_DN1342\_c0\_g1\_i29.p1:TRINITY\_DN1342\_c0\_g1\_i23.p1:TRINITY\_DN1342\_c0\_g1\_i54.p1:TRINITY\_DN1342\_c0\_g1\_i9.p1:TRINITY\_DN1342\_c0\_g1\_i34.p1 |  |  | PEAKS DB |
| FDIKYNSKGNYR | 66.49 | 1503.7469 | 12 | 0.7 | 502.2566 | 29.10 | 7 | F7:5897 | Fraction\_7\_23092022.raw |  |  |  |  |  |  | 8.6251E5 | 2 | 0 | 0 | 0 | 0 | 0 | 0 | 2 | TRINITY\_DN2228\_c0\_g4\_i1.p1 |  |  | PEAKS DB |
| KQISNLQQSISDAEQR | 66.43 | 1843.9387 | 16 | -0.5 | 615.6532 | 37.88 | 1 | F1:6339 | Fraction\_1\_23092022r.raw | 5.9913E5 |  |  |  |  |  |  | 1 | 1 | 0 | 0 | 0 | 0 | 0 | 0 | P04264|K2C1\_HUMAN |  |  | PEAKS DB |
| KYTDLHADITPLILDRM(+15.99)NEC(+57.02)IK | 66.42 | 2674.3457 | 22 | -0.3 | 892.4556 | 54.88 | 1 | F1:13724 | Fraction\_1\_23092022r.raw | 2.6479E6 |  |  |  |  |  |  | 2 | 2 | 0 | 0 | 0 | 0 | 0 | 0 | TRINITY\_DN4276\_c0\_g1\_i1.p1 | Oxidation (M); Carbamidomethylation | M17:Oxidation (M):1000.00;C20:Carbamidomethylation:1000.00 | PEAKS DB |
| HEC(+57.02)C(+57.02)LIGVPQSK | 66.41 | 1426.6697 | 12 | 0.1 | 714.3422 | 33.72 | 7 | F7:7919 | Fraction\_7\_23092022.raw |  |  |  |  |  |  | 1.5167E5 | 1 | 0 | 0 | 0 | 0 | 0 | 0 | 1 | TRINITY\_DN2326\_c0\_g1\_i12.p1:A0A0M3KKW3|PA1\_VESBA:C0HLL3|PA1\_VESVE | Carbamidomethylation | C3:Carbamidomethylation:1000.00;C4:Carbamidomethylation:1000.00 | PEAKS DB |
| FFEITPEKNPQLR | 66.41 | 1617.8514 | 13 | 0.8 | 540.2915 | 50.17 | 1 | F1:11651 | Fraction\_1\_23092022r.raw | 2.4976E5 |  |  |  |  |  |  | 1 | 1 | 0 | 0 | 0 | 0 | 0 | 0 | P0DO15|GLCA2\_SOYBN:P0DO16|GLCA1\_SOYBN:F7J077|GLCB2\_SOYBN:P25974|GLCB1\_SOYBN |  |  | PEAKS DB |
| DTVANWVVGNHDNHR | 66.40 | 1732.8030 | 15 | 0.6 | 578.6086 | 40.87 | 1 | F1:7531 | Fraction\_1\_23092022r.raw | 3.2277E5 |  |  |  |  |  |  | 1 | 1 | 0 | 0 | 0 | 0 | 0 | 0 | TRINITY\_DN10047\_c0\_g1\_i1.p1 |  |  | PEAKS DB |
| LLYENIFNNEK | 66.36 | 1395.7034 | 11 | 0.5 | 698.8593 | 63.08 | 5 | F5:18278 | Fraction\_5\_23092022.raw | 1.3169E5 | 7.8026E5 | 2.4406E5 | 3.7341E5 | 1.0101E5 |  |  | 5 | 1 | 1 | 1 | 1 | 1 | 0 | 0 | TRINITY\_DN2373\_c0\_g1\_i19.p1 |  |  | PEAKS DB |
| YEELQITAGR | 66.25 | 1178.5931 | 10 | 0.0 | 590.3038 | 43.49 | 1 | F1:8687 | Fraction\_1\_23092022r.raw | 4.6349E6 |  | 2.848E6 |  |  |  |  | 3 | 2 | 0 | 1 | 0 | 0 | 0 | 0 | P04264|K2C1\_HUMAN |  |  | PEAKS DB |
| KM(+15.99)PDPDTNNPISPNLK | 66.18 | 1795.8774 | 16 | 1.4 | 599.6339 | 34.19 | 4 | F4:5844 | Fraction\_4\_23092022.raw |  |  |  | 2.4364E5 |  |  |  | 2 | 0 | 0 | 0 | 2 | 0 | 0 | 0 | TRINITY\_DN1113\_c0\_g1\_i95.p1:TRINITY\_DN1113\_c0\_g1\_i47.p1:TRINITY\_DN1113\_c0\_g1\_i59.p1:TRINITY\_DN1113\_c0\_g1\_i53.p1:TRINITY\_DN1113\_c0\_g1\_i73.p1:TRINITY\_DN1113\_c0\_g1\_i6.p1:TRINITY\_DN1113\_c0\_g1\_i12.p1 | Oxidation (M) | M2:Oxidation (M):1000.00 | PEAKS DB |
| ALFTSFTITSIK | 66.05 | 1327.7388 | 12 | 0.4 | 664.8770 | 74.03 | 6 | F6:23845 | Fraction\_6\_23092022.raw |  |  |  |  |  | 1.6124E5 |  | 1 | 0 | 0 | 0 | 0 | 0 | 1 | 0 | TRINITY\_DN2373\_c0\_g1\_i12.p1:TRINITY\_DN2373\_c0\_g1\_i1.p1:TRINITY\_DN2373\_c0\_g1\_i22.p1 |  |  | PEAKS DB |
| NYVTITEDEEDKLKK | 66.02 | 1823.9153 | 15 | 0.9 | 608.9796 | 38.45 | 5 | F5:7243 | Fraction\_5\_23092022.raw |  |  | 7.7648E5 | 7.3361E5 | 6.3942E5 | 8.9076E5 |  | 4 | 0 | 0 | 1 | 1 | 1 | 1 | 0 | TRINITY\_DN4312\_c0\_g1\_i15.p1:TRINITY\_DN4312\_c0\_g1\_i7.p1:TRINITY\_DN4312\_c0\_g1\_i9.p1 |  |  | PEAKS DB |
| GVDEVTIVNILTNR | 65.97 | 1541.8413 | 14 | 1.5 | 771.9291 | 80.14 | 1 | F1:25039 | Fraction\_1\_23092022r.raw | 2.0326E5 |  | 5.1373E4 |  |  |  |  | 2 | 1 | 0 | 1 | 0 | 0 | 0 | 0 | P19620|ANXA2\_PIG:A2SW69|ANXA2\_SHEEP:P04272|ANXA2\_BOVIN:P07356|ANXA2\_MOUSE:Q6TEQ7|ANXA2\_CANLF:Q2Q1M6|ANXA2\_CEREL:C0HJG9|ANXA2\_MESAU:Q5R5A0|ANXA2\_PONAB:P07355|ANXA2\_HUMAN:Q07936|ANXA2\_RAT |  |  | PEAKS DB |
| GFPVC(+57.02)GNFKPSVYI | 65.97 | 1583.7806 | 14 | 0.4 | 792.8979 | 71.37 | 2 | F2:21356 | Fraction\_2\_23092022.raw | 3.7868E5 | 3.3082E6 |  |  |  |  |  | 2 | 1 | 1 | 0 | 0 | 0 | 0 | 0 | TRINITY\_DN6486\_c0\_g1\_i4.p1 | Carbamidomethylation | C5:Carbamidomethylation:1000.00 | PEAKS DB |
| SSGTSYPDVLK | 65.96 | 1152.5663 | 11 | 1.5 | 577.2913 | 40.70 | 7 | F7:11046 | Fraction\_7\_23092022.raw | 7.4472E8 | 7.9108E7 | 1.1101E9 | 1.4888E9 | 1.082E9 | 1.5073E9 | 1.1447E9 | 17 | 3 | 1 | 2 | 3 | 1 | 2 | 5 | P00760|TRY1\_BOVIN |  |  | PEAKS DB |
| FNPC(+57.02)PYSDDTVKMIILTR | 65.72 | 2169.0598 | 18 | 0.6 | 724.0276 | 67.77 | 3 | F3:20037 | Fraction\_3\_23092022.raw | 4.9936E5 |  | 9.8917E5 |  |  |  |  | 2 | 1 | 0 | 1 | 0 | 0 | 0 | 0 | TRINITY\_DN2326\_c0\_g1\_i13.p1:A0A0M3KKW3|PA1\_VESBA | Carbamidomethylation | C4:Carbamidomethylation:1000.00 | PEAKS DB |
| AGKYDISYPEKGEQVIKVK | 65.70 | 2151.1575 | 19 | 0.2 | 718.0599 | 36.97 | 7 | F7:9360 | Fraction\_7\_23092022.raw |  |  | 2.4969E6 | 1.3951E5 | 3.7482E5 | 7.19E5 | 1.0484E7 | 8 | 0 | 0 | 2 | 1 | 1 | 1 | 3 | TRINITY\_DN33420\_c0\_g1\_i1.p1 |  |  | PEAKS DB |
| ADTLTDEINFLR | 65.61 | 1406.7041 | 12 | 0.4 | 704.3596 | 72.52 | 2 | F2:21808 | Fraction\_2\_23092022.raw | 7.689E5 | 6.8039E5 | 1.0794E6 | 1.5412E5 |  | 2.6106E5 |  | 5 | 1 | 1 | 1 | 1 | 0 | 1 | 0 | P48668|K2C6C\_HUMAN |  |  | PEAKS DB |
| SLDLDSIISEVK | 65.60 | 1317.7028 | 12 | 0.2 | 659.8588 | 78.96 | 3 | F3:25064 | Fraction\_3\_23092022.raw |  |  | 3.4587E5 |  |  |  |  | 1 | 0 | 0 | 1 | 0 | 0 | 0 | 0 |  |  |  | PEAKS DB |
| NIKNAVVTIEINQNVATLRIC(+57.02)RNSAGEDSC(+57.02)TR | 65.56 | 3615.8210 | 32 | 0.2 | 724.1716 | 54.03 | 1 | F1:13452 | Fraction\_1\_23092022r.raw | 3.8551E6 |  |  |  |  |  |  | 2 | 2 | 0 | 0 | 0 | 0 | 0 | 0 | TRINITY\_DN4276\_c0\_g1\_i1.p1 | Carbamidomethylation | C21:Carbamidomethylation:1000.00;C30:Carbamidomethylation:1000.00 | PEAKS DB |
| SSDSTATDRDYTYLDHIYTKDDPR | 65.52 | 2834.2629 | 24 | -0.5 | 945.7611 | 44.63 | 2 | F2:9256 | Fraction\_2\_23092022.raw |  | 1.5598E8 |  |  | 2.8075E6 |  |  | 5 | 0 | 4 | 0 | 0 | 1 | 0 | 0 | TRINITY\_DN1342\_c0\_g1\_i23.p1:TRINITY\_DN1342\_c0\_g1\_i54.p1 |  |  | PEAKS DB |
| SSLLEKGLDGAK | 65.33 | 1216.6663 | 12 | 0.2 | 406.5628 | 36.69 | 7 | F7:9243 | Fraction\_7\_23092022.raw |  |  |  | 1.3485E5 | 5.1245E4 | 9.6435E4 | 1.128E5 | 4 | 0 | 0 | 0 | 1 | 1 | 1 | 1 | P81605|DCD\_HUMAN |  |  | PEAKS DB |
| ALM(+15.99)GSPQLVAAVVR | 65.31 | 1426.7966 | 14 | 0.4 | 714.4059 | 57.87 | 1 | F1:15081 | Fraction\_1\_23092022r.raw | 1.8804E5 |  |  |  |  |  |  | 1 | 1 | 0 | 0 | 0 | 0 | 0 | 0 | Q8SPJ1|PLAK\_BOVIN:P14923|PLAK\_HUMAN:Q8WNW3|PLAK\_PIG:Q02257|PLAK\_MOUSE | Oxidation (M) | M3:Oxidation (M):1000.00 | PEAKS DB |
| TPFQWSYVR | 65.12 | 1182.5822 | 9 | 1.5 | 592.2993 | 59.87 | 1 | F1:15973 | Fraction\_1\_23092022r.raw | 4.492E5 | 6.4111E5 |  |  |  |  |  | 2 | 1 | 1 | 0 | 0 | 0 | 0 | 0 | TRINITY\_DN1342\_c0\_g1\_i29.p1:TRINITY\_DN1342\_c0\_g1\_i9.p1 |  |  | PEAKS DB |
| SGFSSISVSR | 64.90 | 1025.5142 | 10 | -0.5 | 513.7641 | 39.10 | 6 | F6:8160 | Fraction\_6\_23092022.raw |  |  |  |  | 4.542E4 | 1.3482E5 |  | 2 | 0 | 0 | 0 | 0 | 1 | 1 | 0 | P48668|K2C6C\_HUMAN |  |  | PEAKS DB |
| GAAQNIIPAATGAAK | 64.85 | 1352.7412 | 15 | 0.3 | 677.3781 | 40.32 | 6 | F6:8717 | Fraction\_6\_23092022.raw |  |  |  |  |  | 1.3614E5 | 1.1358E5 | 2 | 0 | 0 | 0 | 0 | 0 | 1 | 1 | TRINITY\_DN5849\_c0\_g1\_i1.p1:TRINITY\_DN5849\_c0\_g1\_i2.p1:TRINITY\_DN5849\_c0\_g1\_i10.p1 |  |  | PEAKS DB |
| ESC(+57.02)ITESGVDPAIVENAK | 64.78 | 1917.8989 | 18 | 3.1 | 959.9597 | 50.19 | 7 | F7:15562 | Fraction\_7\_23092022.raw |  |  |  |  |  |  | 1.6313E5 | 1 | 0 | 0 | 0 | 0 | 0 | 0 | 1 | TRINITY\_DN36822\_c0\_g1\_i1.p1 | Carbamidomethylation | C3:Carbamidomethylation:1000.00 | PEAKS DB |
| TSVAEM(+15.99)PDILQK | 64.76 | 1346.6752 | 12 | 0.3 | 674.3450 | 41.19 | 6 | F6:9079 | Fraction\_6\_23092022.raw |  |  | 5.1822E6 |  | 1.7533E5 | 2.5349E6 |  | 5 | 0 | 0 | 2 | 0 | 1 | 2 | 0 | TRINITY\_DN33420\_c0\_g1\_i1.p1 | Oxidation (M) | M6:Oxidation (M):1000.00 | PEAKS DB |
| SGGGFSSGSAGIINYQRRTTSSSTRR | 64.71 | 2689.3281 | 26 | -0.2 | 673.3392 | 29.61 | 7 | F7:6110 | Fraction\_7\_23092022.raw |  |  |  |  |  |  | 2.026E5 | 2 | 0 | 0 | 0 | 0 | 0 | 0 | 2 | P04264|K2C1\_HUMAN |  |  | PEAKS DB |
| SDLEM(+15.99)QYETLQEELM(+15.99)ALKK | 64.71 | 2330.1021 | 19 | 1.1 | 777.7088 | 76.03 | 1 | F1:23219 | Fraction\_1\_23092022r.raw | 1.1277E6 |  |  |  |  |  |  | 2 | 2 | 0 | 0 | 0 | 0 | 0 | 0 | P35527|K1C9\_HUMAN | Oxidation (M) | M5:Oxidation (M):1000.00;M15:Oxidation (M):1000.00 | PEAKS DB |
| LFLPHNFDESK | 64.70 | 1345.6666 | 11 | 0.1 | 449.5629 | 48.29 | 7 | F7:14587 | Fraction\_7\_23092022.raw |  |  | 3.5424E5 | 3.9359E5 | 1.7704E6 | 1.6064E6 | 1.1746E7 | 6 | 0 | 0 | 1 | 1 | 1 | 1 | 2 | TRINITY\_DN3450\_c0\_g1\_i51.p1 |  |  | PEAKS DB |
| M(+15.99)IKHPIWSTWAR | 64.64 | 1540.7972 | 12 | -0.2 | 514.6063 | 43.60 | 1 | F1:8765 | Fraction\_1\_23092022r.raw | 3.1999E7 |  |  |  |  |  |  | 3 | 3 | 0 | 0 | 0 | 0 | 0 | 0 | TRINITY\_DN4276\_c0\_g1\_i1.p1 | Oxidation (M) | M1:Oxidation (M):1000.00 | PEAKS DB |
| ITGC(+57.02)SLEKIEDDAFK | 64.62 | 1724.8291 | 15 | 1.5 | 575.9512 | 52.85 | 6 | F6:14345 | Fraction\_6\_23092022.raw |  |  | 1.9808E5 |  |  | 1.8826E5 |  | 2 | 0 | 0 | 1 | 0 | 0 | 1 | 0 | TRINITY\_DN2938\_c0\_g1\_i1.p1 | Carbamidomethylation | C4:Carbamidomethylation:1000.00 | PEAKS DB |
| MIKHPIWSTWAR | 64.56 | 1524.8024 | 12 | 0.0 | 509.2747 | 45.95 | 1 | F1:9719 | Fraction\_1\_23092022r.raw | 6.7723E6 |  |  |  |  |  |  | 1 | 1 | 0 | 0 | 0 | 0 | 0 | 0 | TRINITY\_DN4276\_c0\_g1\_i1.p1 |  |  | PEAKS DB |
| NEC(+57.02)VC(+57.02)VGLNAKTYPK | 64.53 | 1751.8335 | 15 | 0.2 | 876.9242 | 35.82 | 7 | F7:8817 | Fraction\_7\_23092022.raw |  |  | 4.6572E5 | 5.8753E5 | 1.8031E6 |  | 5.1284E6 | 9 | 0 | 0 | 1 | 1 | 3 | 0 | 4 | TRINITY\_DN2326\_c0\_g1\_i12.p1:A0A0M3KKW3|PA1\_VESBA | Carbamidomethylation | C3:Carbamidomethylation:1000.00;C5:Carbamidomethylation:1000.00 | PEAKS DB |
| NQC(+57.02)VC(+57.02)VGLNAKTYPKTGSFYVPVESKAPYC(+57.02)NNK | 64.51 | 3792.8062 | 33 | 1.0 | 759.5693 | 48.94 | 4 | F4:12156 | Fraction\_4\_23092022.raw |  |  | 1.7089E5 | 7.9576E5 |  |  |  | 2 | 0 | 0 | 1 | 1 | 0 | 0 | 0 |  | Carbamidomethylation | C3:Carbamidomethylation:1000.00;C5:Carbamidomethylation:1000.00;C30:Carbamidomethylation:1000.00 | PEAKS DB |
| NVNNLKINKEVSIDLVRK | 64.50 | 2095.2112 | 18 | 0.7 | 524.8104 | 41.35 | 6 | F6:9142 | Fraction\_6\_23092022.raw |  |  |  |  | 1.1412E6 | 3.6447E6 | 6.1332E5 | 5 | 0 | 0 | 0 | 0 | 2 | 2 | 1 | TRINITY\_DN2228\_c0\_g4\_i1.p1 |  |  | PEAKS DB |
| DLYVRANGYESKVR | 64.41 | 1668.8583 | 14 | -0.2 | 557.2933 | 32.49 | 7 | F7:7364 | Fraction\_7\_23092022.raw |  |  |  |  |  |  | 4.5572E5 | 1 | 0 | 0 | 0 | 0 | 0 | 0 | 1 | TRINITY\_DN3450\_c0\_g1\_i51.p1 |  |  | PEAKS DB |
| FASFIDKVR | 64.36 | 1081.5920 | 9 | 1.5 | 361.5385 | 44.57 | 4 | F4:10216 | Fraction\_4\_23092022.raw | 6.0874E6 | 1.3079E6 | 2.6129E6 | 3.499E5 | 3.5976E5 | 1.1286E6 |  | 11 | 1 | 2 | 2 | 2 | 2 | 2 | 0 | P35908|K22E\_HUMAN:P48668|K2C6C\_HUMAN:P13647|K2C5\_HUMAN:Q3TTY5|K22E\_MOUSE:P05787|K2C8\_HUMAN:Q6IME9|K2C72\_MOUSE:Q86Y46|K2C73\_HUMAN:Q3SY84|K2C71\_HUMAN:Q148H5|K2C71\_BOVIN:Q14CN4|K2C72\_HUMAN:E1AB55|K2C71\_FELCA:Q6IG04|K2C72\_RAT:Q7RTS7|K2C74\_HUMAN |  |  | PEAKS DB |
| YPYQVSLRAPK | 64.31 | 1320.7190 | 11 | -1.1 | 441.2465 | 40.17 | 7 | F7:10797 | Fraction\_7\_23092022.raw |  |  |  |  |  |  | 8.3929E5 | 1 | 0 | 0 | 0 | 0 | 0 | 0 | 1 | P00768|CTR2\_VESOR:P00769|CTR2\_VESCR |  |  | PEAKS DB |
| KHDFYTLNTIK | 64.31 | 1378.7245 | 11 | 1.8 | 460.5829 | 35.21 | 7 | F7:8547 | Fraction\_7\_23092022.raw |  |  | 1.2452E6 |  |  | 8.7958E5 | 2.2158E7 | 4 | 0 | 0 | 1 | 0 | 0 | 1 | 2 |  |  |  | PEAKS DB |
| DAEAWFNEK | 64.27 | 1108.4825 | 9 | 0.0 | 555.2485 | 53.27 | 1 | F1:13013 | Fraction\_1\_23092022r.raw | 3.1891E6 |  |  |  |  |  |  | 1 | 1 | 0 | 0 | 0 | 0 | 0 | 0 | P13645|K1C10\_HUMAN |  |  | PEAKS DB |
| FSNNGFNDVQR | 64.20 | 1296.5847 | 11 | -0.1 | 649.2996 | 33.82 | 7 | F7:7958 | Fraction\_7\_23092022.raw |  |  |  |  |  | 4.942E4 | 3.8972E5 | 2 | 0 | 0 | 0 | 0 | 0 | 1 | 1 | TRINITY\_DN3450\_c0\_g1\_i51.p1 |  |  | PEAKS DB |
| ELTPDQRIGLVQGRVK | 64.13 | 1808.0267 | 16 | 0.4 | 453.0142 | 38.13 | 7 | F7:9871 | Fraction\_7\_23092022.raw |  |  |  |  |  | 5.8158E5 | 1.5611E6 | 3 | 0 | 0 | 0 | 0 | 0 | 1 | 2 | TRINITY\_DN2228\_c0\_g2\_i2.p1:TRINITY\_DN2228\_c0\_g2\_i1.p1 |  |  | PEAKS DB |
| MNEC(+57.02)IKTGAPLNPPIWWVDPTDKTAHK | 64.06 | 3118.5369 | 27 | -1.9 | 624.7134 | 62.27 | 1 | F1:17073 | Fraction\_1\_23092022r.raw | 1.5369E6 |  |  |  |  |  |  | 2 | 2 | 0 | 0 | 0 | 0 | 0 | 0 | TRINITY\_DN4276\_c0\_g1\_i1.p1 | Carbamidomethylation | C4:Carbamidomethylation:1000.00 | PEAKS DB |
| SKELTTEIDNNIEQISSYKSEITELRR | 63.98 | 3195.6257 | 27 | 0.9 | 640.1330 | 63.95 | 1 | F1:17827 | Fraction\_1\_23092022r.raw | 5.5905E5 |  |  |  |  |  |  | 1 | 1 | 0 | 0 | 0 | 0 | 0 | 0 | P13645|K1C10\_HUMAN |  |  | PEAKS DB |
| IKVKVNPSGVVILR | 63.94 | 1520.9766 | 14 | 0.5 | 507.9997 | 47.25 | 6 | F6:11791 | Fraction\_6\_23092022.raw |  | 3.9089E6 |  |  |  | 1.0616E6 |  | 2 | 0 | 1 | 0 | 0 | 0 | 1 | 0 | TRINITY\_DN2611\_c0\_g1\_i12.p1:TRINITY\_DN2611\_c0\_g1\_i23.p1:TRINITY\_DN2611\_c0\_g1\_i19.p1:TRINITY\_DN2611\_c0\_g1\_i9.p1 |  |  | PEAKS DB |
| LKESETFSKFISYLSDQK | 63.88 | 2149.0942 | 18 | 0.8 | 717.3726 | 66.81 | 7 | F7:25373 | Fraction\_7\_23092022.raw |  |  |  |  |  |  | 1.4916E6 | 1 | 0 | 0 | 0 | 0 | 0 | 0 | 1 | TRINITY\_DN13586\_c0\_g2\_i1.p1 |  |  | PEAKS DB |
| SAYPGQITSNMFC(+57.02)AGYLEGGKDSC(+57.02)QGDSGGPVVC(+57.02)SGK | 63.79 | 3840.6487 | 37 | 1.2 | 1281.2251 | 63.33 | 1 | F1:17514 | Fraction\_1\_23092022r.raw | 5.536E5 |  |  |  |  |  |  | 1 | 1 | 0 | 0 | 0 | 0 | 0 | 0 | P00760|TRY1\_BOVIN | Carbamidomethylation | C13:Carbamidomethylation:1000.00;C24:Carbamidomethylation:1000.00;C34:Carbamidomethylation:1000.00 | PEAKS DB |
| LAC(+57.02)GVIGITKSG | 63.71 | 1174.6379 | 12 | 0.6 | 588.3266 | 43.93 | 6 | F6:10320 | Fraction\_6\_23092022.raw |  |  |  |  |  | 2.6798E5 |  | 1 | 0 | 0 | 0 | 0 | 0 | 1 | 0 | TRINITY\_DN1931\_c0\_g1\_i1.p1:TRINITY\_DN1931\_c0\_g1\_i2.p1:TRINITY\_DN1931\_c0\_g1\_i4.p1 | Carbamidomethylation | C3:Carbamidomethylation:1000.00 | PEAKS DB |
| AAEFGAVAALIR | 63.54 | 1187.6663 | 12 | 1.2 | 594.8411 | 69.63 | 2 | F2:20503 | Fraction\_2\_23092022.raw |  | 3.8513E5 |  |  |  |  |  | 1 | 0 | 1 | 0 | 0 | 0 | 0 | 0 | TRINITY\_DN3209\_c0\_g2\_i3.p1:TRINITY\_DN3209\_c0\_g2\_i5.p1 |  |  | PEAKS DB |
| ELTTEIDNNIEQISSYKSEITELR | 63.45 | 2824.3977 | 24 | 1.3 | 942.4744 | 74.67 | 1 | F1:22588 | Fraction\_1\_23092022r.raw | 3.3114E5 |  |  |  |  |  |  | 1 | 1 | 0 | 0 | 0 | 0 | 0 | 0 | P13645|K1C10\_HUMAN |  |  | PEAKS DB |
| SVIYAYIDGRGSSNKGSK | 63.42 | 1900.9642 | 18 | 0.5 | 634.6624 | 34.56 | 7 | F7:8264 | Fraction\_7\_23092022.raw |  |  |  |  |  | 8.7678E5 | 3.8227E6 | 4 | 0 | 0 | 0 | 0 | 0 | 1 | 3 | TRINITY\_DN3450\_c0\_g1\_i51.p1 |  |  | PEAKS DB |
| AILQNKKIIAVDQDPLGIQGR | 63.34 | 2289.3169 | 21 | -0.3 | 573.3363 | 51.34 | 2 | F2:12260 | Fraction\_2\_23092022.raw |  | 2.8591E6 |  |  |  |  |  | 1 | 0 | 1 | 0 | 0 | 0 | 0 | 0 | TRINITY\_DN2611\_c0\_g1\_i12.p1:TRINITY\_DN2611\_c0\_g1\_i23.p1:TRINITY\_DN2611\_c0\_g1\_i19.p1:TRINITY\_DN2611\_c0\_g1\_i9.p1 |  |  | PEAKS DB |
| VVVDALSGLKGDLAGR | 63.33 | 1568.8885 | 16 | 0.5 | 523.9704 | 57.58 | 3 | F3:15518 | Fraction\_3\_23092022.raw |  |  | 7.2941E4 |  |  |  |  | 1 | 0 | 0 | 1 | 0 | 0 | 0 | 0 | Q29577|KCRU\_PIG:Q9TTK8|KCRU\_BOVIN:P12532|KCRU\_HUMAN:P30275|KCRU\_MOUSE:P25809|KCRU\_RAT |  |  | PEAKS DB |
| DAIPENLPPLTADFAEDK | 63.32 | 1954.9523 | 18 | 0.9 | 978.4843 | 75.12 | 3 | F3:23333 | Fraction\_3\_23092022.raw |  | 8.381E5 | 9.9163E5 |  |  |  |  | 2 | 0 | 1 | 1 | 0 | 0 | 0 | 0 | P02769|ALBU\_BOVIN |  |  | PEAKS DB |
| SFSTASAITPSVSR | 63.29 | 1409.7151 | 14 | 0.8 | 705.8654 | 44.69 | 1 | F1:9208 | Fraction\_1\_23092022r.raw | 2.2271E5 | 3.7261E5 |  |  |  | 1.8547E5 |  | 3 | 1 | 1 | 0 | 0 | 0 | 1 | 0 | P13647|K2C5\_HUMAN |  |  | PEAKS DB |
| DPLFLDQNNYMDK | 63.27 | 1611.7239 | 13 | 0.5 | 806.8696 | 65.71 | 1 | F1:18588 | Fraction\_1\_23092022r.raw | 3.4432E5 |  |  |  |  |  |  | 1 | 1 | 0 | 0 | 0 | 0 | 0 | 0 | TRINITY\_DN4276\_c0\_g1\_i1.p1 |  |  | PEAKS DB |
| EVATNSELVQSGKSEISELR | 63.14 | 2175.1018 | 20 | 0.4 | 726.0415 | 49.48 | 1 | F1:11348 | Fraction\_1\_23092022r.raw | 1.8745E5 |  |  |  |  |  |  | 1 | 1 | 0 | 0 | 0 | 0 | 0 | 0 | P02533|K1C14\_HUMAN:Q6IFU8|K1C17\_RAT:Q9QWL7|K1C17\_MOUSE |  |  | PEAKS DB |
| TLNDMRQEYEQLIAK | 63.07 | 1850.9197 | 15 | -0.1 | 617.9805 | 61.23 | 1 | F1:16523 | Fraction\_1\_23092022r.raw | 6.9008E6 |  |  |  |  |  |  | 1 | 1 | 0 | 0 | 0 | 0 | 0 | 0 | P35527|K1C9\_HUMAN |  |  | PEAKS DB |
| FNPC(+57.02)PYSDDTVKM(+15.99)IILTRENK | 62.94 | 2556.2351 | 21 | -0.4 | 640.0658 | 54.01 | 7 | F7:17800 | Fraction\_7\_23092022.raw |  |  |  |  |  |  | 1.9691E6 | 1 | 0 | 0 | 0 | 0 | 0 | 0 | 1 | A0A0M3KKW3|PA1\_VESBA | Carbamidomethylation; Oxidation (M) | C4:Carbamidomethylation:1000.00;M13:Oxidation (M):1000.00 | PEAKS DB |
| VGDFVATDLDTGRPSTTVR | 62.78 | 2006.0068 | 19 | -0.1 | 669.6761 | 48.18 | 1 | F1:10779 | Fraction\_1\_23092022r.raw | 2.3162E5 |  |  |  |  |  |  | 1 | 1 | 0 | 0 | 0 | 0 | 0 | 0 | Q02413|DSG1\_HUMAN |  |  | PEAKS DB |
| NQC(+57.02)VC(+57.02)VGLNAK | 62.78 | 1261.5907 | 11 | 0.4 | 631.8029 | 32.57 | 7 | F7:7382 | Fraction\_7\_23092022.raw |  |  | 4.2995E6 | 2.497E6 | 1.9005E6 | 1.1193E6 | 8.1138E6 | 5 | 0 | 0 | 1 | 1 | 1 | 1 | 1 |  | Carbamidomethylation | C3:Carbamidomethylation:1000.00;C5:Carbamidomethylation:1000.00 | PEAKS DB |
| RHPEYAVSVLLR | 62.76 | 1438.8044 | 12 | 0.7 | 480.6091 | 44.13 | 1 | F1:8993 | Fraction\_1\_23092022r.raw | 1.7682E5 |  |  |  |  |  |  | 1 | 1 | 0 | 0 | 0 | 0 | 0 | 0 | P02769|ALBU\_BOVIN |  |  | PEAKS DB |
| LAC(+57.02)GVIGITK | 62.68 | 1030.5845 | 10 | 0.3 | 516.2997 | 44.08 | 6 | F6:10377 | Fraction\_6\_23092022.raw |  |  |  |  |  | 7.6327E5 |  | 1 | 0 | 0 | 0 | 0 | 0 | 1 | 0 | TRINITY\_DN1931\_c0\_g1\_i1.p1:TRINITY\_DN1931\_c0\_g1\_i2.p1:TRINITY\_DN1931\_c0\_g1\_i4.p1 | Carbamidomethylation | C3:Carbamidomethylation:1000.00 | PEAKS DB |
| TVMENFVAFVDK | 62.66 | 1398.6853 | 12 | -0.1 | 700.3499 | 76.25 | 1 | F1:23312 | Fraction\_1\_23092022r.raw | 3.8211E5 |  |  |  |  |  |  | 1 | 1 | 0 | 0 | 0 | 0 | 0 | 0 | P02769|ALBU\_BOVIN |  |  | PEAKS DB |
| DSNGDGIGDLNGITSK | 62.55 | 1561.7219 | 16 | 0.5 | 781.8687 | 50.72 | 1 | F1:11905 | Fraction\_1\_23092022r.raw | 1.6171E5 |  |  |  |  |  |  | 1 | 1 | 0 | 0 | 0 | 0 | 0 | 0 | TRINITY\_DN10047\_c0\_g1\_i1.p1 |  |  | PEAKS DB |
| YPYQVSLR | 62.54 | 1024.5342 | 8 | -0.1 | 513.2743 | 45.13 | 7 | F7:13104 | Fraction\_7\_23092022.raw |  |  |  |  |  |  | 3.4821E5 | 1 | 0 | 0 | 0 | 0 | 0 | 0 | 1 | P00768|CTR2\_VESOR:P00769|CTR2\_VESCR |  |  | PEAKS DB |
| YLGYLEQLLR | 62.46 | 1266.6971 | 10 | 0.9 | 634.3564 | 79.83 | 3 | F3:25448 | Fraction\_3\_23092022.raw | 9.0559E5 | 2.6621E5 | 5.3122E5 | 1.7287E5 | 3.4714E5 | 3.8821E5 | 2.0483E6 | 7 | 1 | 1 | 1 | 1 | 1 | 1 | 1 | O62823|CASA1\_BUBBU:P02662|CASA1\_BOVIN:P18626|CASA1\_CAPHI:P04653|CASA1\_SHEEP |  |  | PEAKS DB |
| AEKLEILKQHNEFR | 62.43 | 1753.9475 | 14 | -0.3 | 439.4940 | 32.15 | 7 | F7:7226 | Fraction\_7\_23092022.raw |  |  |  |  |  |  | 1.2172E5 | 1 | 0 | 0 | 0 | 0 | 0 | 0 | 1 | P0DMB9|VA5\_VESVE |  |  | PEAKS DB |
| LALDVEIATYR | 62.33 | 1262.6870 | 11 | 0.5 | 632.3511 | 64.39 | 3 | F3:18523 | Fraction\_3\_23092022.raw | 1.5189E6 | 6.2742E5 | 1.3952E6 | 2.3872E5 | 2.1606E5 | 3.32E5 |  | 6 | 1 | 1 | 1 | 1 | 1 | 1 | 0 | P35908|K22E\_HUMAN:P48668|K2C6C\_HUMAN:P13647|K2C5\_HUMAN |  |  | PEAKS DB |
| AVLYTTDGINDR | 62.30 | 1336.6622 | 12 | -0.3 | 669.3382 | 43.07 | 1 | F1:8507 | Fraction\_1\_23092022r.raw | 6.9744E5 | 5.3173E5 |  |  |  |  |  | 2 | 1 | 1 | 0 | 0 | 0 | 0 | 0 | TRINITY\_DN11986\_c0\_g1\_i3.p1 |  |  | PEAKS DB |
| ELTPDQRIGLVQGR | 62.17 | 1580.8634 | 14 | 1.0 | 527.9623 | 41.61 | 7 | F7:11520 | Fraction\_7\_23092022.raw |  |  | 1.2245E6 |  | 5.7715E5 | 5.7725E7 | 1.315E7 | 4 | 0 | 0 | 1 | 0 | 1 | 1 | 1 | TRINITY\_DN2228\_c0\_g2\_i2.p1:TRINITY\_DN2228\_c0\_g2\_i1.p1 |  |  | PEAKS DB |
| C(+57.02)TRNEC(+57.02)VC(+57.02)VGLNAK | 62.11 | 1679.7542 | 14 | 0.2 | 560.9254 | 30.48 | 7 | F7:6463 | Fraction\_7\_23092022.raw |  |  |  |  |  |  | 1.1536E6 | 2 | 0 | 0 | 0 | 0 | 0 | 0 | 2 | A0A0M3KKW3|PA1\_VESBA:C0HLL3|PA1\_VESVE | Carbamidomethylation | C1:Carbamidomethylation:1000.00;C6:Carbamidomethylation:1000.00;C8:Carbamidomethylation:1000.00 | PEAKS DB |
| LLEGEDAHLSSQQASGQSYSSR | 61.77 | 2349.0833 | 22 | 0.6 | 784.0355 | 35.11 | 1 | F1:5497 | Fraction\_1\_23092022r.raw | 7.0078E4 |  |  |  |  |  |  | 1 | 1 | 0 | 0 | 0 | 0 | 0 | 0 | P08779|K1C16\_HUMAN |  |  | PEAKS DB |
| VLYDAEISQIHQSVTDTNVILSMDNSR | 61.77 | 3047.4871 | 27 | 0.4 | 1016.8367 | 73.57 | 1 | F1:22123 | Fraction\_1\_23092022r.raw | 1.7326E5 |  |  |  |  |  |  | 1 | 1 | 0 | 0 | 0 | 0 | 0 | 0 | P35908|K22E\_HUMAN |  |  | PEAKS DB |
| ENKKHDFYTLDTIK | 61.76 | 1750.8889 | 14 | 0.3 | 584.6371 | 33.91 | 7 | F7:7990 | Fraction\_7\_23092022.raw |  |  |  |  |  |  | 4.2338E5 | 1 | 0 | 0 | 0 | 0 | 0 | 0 | 1 | A0A0M3KKW3|PA1\_VESBA |  |  | PEAKS DB |
| ISGVGIDQPPYGIFVINQK | 61.67 | 2044.0992 | 19 | 0.5 | 1023.0574 | 77.32 | 1 | F1:23761 | Fraction\_1\_23092022r.raw | 5.3696E5 |  |  |  |  |  |  | 1 | 1 | 0 | 0 | 0 | 0 | 0 | 0 | Q02413|DSG1\_HUMAN |  |  | PEAKS DB |
| FRC(+57.02)NTDC(+57.02)KNDPDNC(+57.02)ISDQLFR | 61.62 | 2674.1323 | 21 | 0.8 | 669.5409 | 46.02 | 2 | F2:9891 | Fraction\_2\_23092022.raw |  | 1.0062E6 |  |  |  |  |  | 1 | 0 | 1 | 0 | 0 | 0 | 0 | 0 | TRINITY\_DN2611\_c0\_g1\_i12.p1:TRINITY\_DN2611\_c0\_g1\_i23.p1:TRINITY\_DN2611\_c0\_g1\_i19.p1:TRINITY\_DN2611\_c0\_g1\_i9.p1 | Carbamidomethylation | C3:Carbamidomethylation:1000.00;C7:Carbamidomethylation:1000.00;C14:Carbamidomethylation:1000.00 | PEAKS DB |
| RSLVFDNIIEGYSKR | 61.61 | 1795.9580 | 15 | -0.4 | 599.6597 | 54.32 | 6 | F6:15000 | Fraction\_6\_23092022.raw |  |  |  |  |  | 7.7447E5 |  | 1 | 0 | 0 | 0 | 0 | 0 | 1 | 0 | TRINITY\_DN33395\_c0\_g1\_i1.p1 |  |  | PEAKS DB |
| ISNYLDFIVK | 61.49 | 1210.6598 | 10 | -0.2 | 606.3370 | 72.84 | 3 | F3:22313 | Fraction\_3\_23092022.raw |  |  | 1.4421E7 |  |  |  | 6.8673E5 | 2 | 0 | 0 | 1 | 0 | 0 | 0 | 1 | TRINITY\_DN1494\_c0\_g1\_i8.p1 |  |  | PEAKS DB |
| KIIAVDQDPLGIQGRR | 61.34 | 1778.0162 | 16 | -0.2 | 445.5113 | 43.25 | 2 | F2:8662 | Fraction\_2\_23092022.raw |  | 2.7076E6 |  |  |  |  |  | 1 | 0 | 1 | 0 | 0 | 0 | 0 | 0 | TRINITY\_DN2611\_c0\_g1\_i12.p1:TRINITY\_DN2611\_c0\_g1\_i23.p1:TRINITY\_DN2611\_c0\_g1\_i19.p1:TRINITY\_DN2611\_c0\_g1\_i9.p1 |  |  | PEAKS DB |
| AVMDDFAAFVEK | 61.31 | 1341.6274 | 12 | 0.4 | 671.8213 | 74.55 | 1 | F1:22531 | Fraction\_1\_23092022r.raw | 2.3926E5 |  |  |  |  |  |  | 1 | 1 | 0 | 0 | 0 | 0 | 0 | 0 | P02768|ALBU\_HUMAN |  |  | PEAKS DB |
| VNYDRTNWQLIIK | 61.26 | 1661.8889 | 13 | -0.4 | 554.9700 | 56.29 | 6 | F6:15899 | Fraction\_6\_23092022.raw |  |  |  |  |  | 4.1501E5 |  | 1 | 0 | 0 | 0 | 0 | 0 | 1 | 0 | TRINITY\_DN1113\_c0\_g1\_i95.p1:TRINITY\_DN1113\_c0\_g1\_i47.p1:TRINITY\_DN1113\_c0\_g1\_i59.p1:TRINITY\_DN1113\_c0\_g1\_i53.p1:TRINITY\_DN1113\_c0\_g1\_i73.p1:TRINITY\_DN1113\_c0\_g1\_i6.p1:TRINITY\_DN1113\_c0\_g1\_i12.p1 |  |  | PEAKS DB |
| NEC(+57.02)VC(+57.02)VGLNAK | 61.26 | 1262.5747 | 11 | 0.2 | 632.2947 | 34.83 | 7 | F7:8388 | Fraction\_7\_23092022.raw |  |  | 3.1121E6 | 3.1889E6 | 7.1825E5 |  | 3.0319E6 | 10 | 0 | 0 | 2 | 4 | 2 | 0 | 2 | TRINITY\_DN2326\_c0\_g1\_i12.p1:A0A0M3KKW3|PA1\_VESBA:C0HLL3|PA1\_VESVE | Carbamidomethylation | C3:Carbamidomethylation:1000.00;C5:Carbamidomethylation:1000.00 | PEAKS DB |
| AVFPSIVGRPR | 61.14 | 1197.6981 | 11 | -0.2 | 400.2399 | 45.42 | 3 | F3:10004 | Fraction\_3\_23092022.raw |  |  | 5.4273E5 |  |  |  |  | 1 | 0 | 0 | 1 | 0 | 0 | 0 | 0 | TRINITY\_DN273\_c0\_g4\_i11.p1:P83750|ACTB\_CYPCA:P02572|ACT2\_DROME:P60713|ACTB\_SHEEP:Q4R561|ACTB\_MACFA:Q76N69|ACTB\_CHLAE:Q4L0Y2|ACTB\_SPECI:TRINITY\_DN273\_c0\_g4\_i2.p1:TRINITY\_DN273\_c0\_g4\_i4.p1:TRINITY\_DN273\_c0\_g4\_i9.p1:P84856|ACTB\_CHLPG:P63258|ACTG\_BOVIN:P63260|ACTG\_MOUSE:P63259|ACTG\_RAT:A2BDB0|ACTG\_XENLA:Q8JJB8|ACTG\_TRISC:Q5JAK2|ACTG\_PELLE:P15475|ACTB\_XENBO:TRINITY\_DN836\_c0\_g1\_i1.p1:P10987|ACT1\_DROME:TRINITY\_DN836\_c0\_g1\_i3.p1:P68556|ACT1\_DIBDE:TRINITY\_DN836\_c0\_g1\_i2.p1:P04829|ACT3\_BOMMO:P86700|ACT\_CHIOP |  |  | PEAKS DB |
| LLEAQIATGGIIDPK | 61.14 | 1537.8715 | 15 | 0.5 | 769.9434 | 62.22 | 3 | F3:17586 | Fraction\_3\_23092022.raw |  |  | 1.0495E5 |  |  |  |  | 1 | 0 | 0 | 1 | 0 | 0 | 0 | 0 | E9Q557|DESP\_MOUSE:P15924|DESP\_HUMAN |  |  | PEAKS DB |
| ELPLVKDLYVR | 60.92 | 1343.7812 | 11 | -0.6 | 448.9341 | 60.72 | 7 | F7:21963 | Fraction\_7\_23092022.raw |  | 5.5529E4 | 7.5096E5 | 3.6436E5 | 3.1945E6 | 4.1851E6 | 5.7128E7 | 9 | 0 | 1 | 1 | 1 | 2 | 2 | 2 | TRINITY\_DN3450\_c0\_g1\_i51.p1 |  |  | PEAKS DB |
| FAGSLIELR | 60.91 | 1004.5654 | 9 | 0.3 | 503.2901 | 58.22 | 6 | F6:16757 | Fraction\_6\_23092022.raw |  |  | 8.1851E5 |  |  | 6.0674E5 |  | 2 | 0 | 0 | 1 | 0 | 0 | 1 | 0 | TRINITY\_DN2938\_c0\_g1\_i1.p1 |  |  | PEAKS DB |
| AC(+57.02)IPNVALYVAK | 60.85 | 1317.7114 | 12 | 0.2 | 659.8631 | 60.72 | 5 | F5:17213 | Fraction\_5\_23092022.raw |  |  |  | 3.4399E5 | 2.5891E5 |  |  | 2 | 0 | 0 | 0 | 1 | 1 | 0 | 0 | TRINITY\_DN594\_c1\_g4\_i5.p1:TRINITY\_DN594\_c1\_g4\_i1.p1 | Carbamidomethylation | C2:Carbamidomethylation:1000.00 | PEAKS DB |
| ENFPNFLSAC(+57.02)DKK | 60.71 | 1568.7292 | 13 | -0.6 | 523.9167 | 53.25 | 1 | F1:13031 | Fraction\_1\_23092022r.raw | 3.9291E5 |  |  |  |  |  |  | 1 | 1 | 0 | 0 | 0 | 0 | 0 | 0 | P31151|S10A7\_HUMAN | Carbamidomethylation | C10:Carbamidomethylation:1000.00 | PEAKS DB |
| AIGGGLSSVGGGSSTIKYTTTSSSSRK | 60.69 | 2545.2983 | 27 | 0.1 | 637.3319 | 35.96 | 5 | F5:6237 | Fraction\_5\_23092022.raw |  |  |  |  | 1.226E5 |  |  | 1 | 0 | 0 | 0 | 0 | 1 | 0 | 0 | P48668|K2C6C\_HUMAN |  |  | PEAKS DB |
| NIETIINTFHQYSVK | 60.66 | 1805.9312 | 15 | 0.9 | 602.9849 | 70.04 | 1 | F1:20537 | Fraction\_1\_23092022r.raw | 1.7372E5 |  |  |  |  |  |  | 1 | 1 | 0 | 0 | 0 | 0 | 0 | 0 | P06702|S10A9\_HUMAN |  |  | PEAKS DB |
| LREYFGTSLVLINNIVK | 60.64 | 1978.1251 | 17 | 0.8 | 660.3828 | 75.86 | 4 | F4:24204 | Fraction\_4\_23092022.raw |  |  |  | 3.3605E5 |  |  |  | 1 | 0 | 0 | 0 | 1 | 0 | 0 | 0 | TRINITY\_DN1113\_c0\_g1\_i95.p1:TRINITY\_DN1113\_c0\_g1\_i47.p1:TRINITY\_DN1113\_c0\_g1\_i59.p1:TRINITY\_DN1113\_c0\_g1\_i53.p1:TRINITY\_DN1113\_c0\_g1\_i73.p1:TRINITY\_DN1113\_c0\_g1\_i6.p1:TRINITY\_DN1113\_c0\_g1\_i12.p1 |  |  | PEAKS DB |
| FVNSVVDFLK | 60.59 | 1166.6335 | 10 | 1.1 | 584.3247 | 71.87 | 3 | F3:21892 | Fraction\_3\_23092022.raw |  |  | 1.8204E5 | 7.3454E4 |  |  |  | 2 | 0 | 0 | 1 | 1 | 0 | 0 | 0 | TRINITY\_DN36831\_c0\_g1\_i1.p1 |  |  | PEAKS DB |
| SFM(+15.99)DSNDDGVGDLK | 60.49 | 1514.6195 | 14 | -0.3 | 758.3168 | 39.80 | 6 | F6:8482 | Fraction\_6\_23092022.raw | 4.5737E4 | 1.5252E5 |  |  |  | 3.4969E5 |  | 5 | 1 | 2 | 0 | 0 | 0 | 2 | 0 | TRINITY\_DN1342\_c0\_g1\_i29.p1:TRINITY\_DN1342\_c0\_g1\_i23.p1:TRINITY\_DN1342\_c0\_g1\_i54.p1:TRINITY\_DN1342\_c0\_g1\_i9.p1:TRINITY\_DN1342\_c0\_g1\_i34.p1 | Oxidation (M) | M3:Oxidation (M):1000.00 | PEAKS DB |
| TAAENEFVTLK | 60.47 | 1221.6240 | 11 | 0.3 | 611.8195 | 46.65 | 3 | F3:10589 | Fraction\_3\_23092022.raw |  |  | 1.8006E5 |  |  |  |  | 1 | 0 | 0 | 1 | 0 | 0 | 0 | 0 | P48668|K2C6C\_HUMAN |  |  | PEAKS DB |
| GEFPYQVSLQWGLTSSNTK | 60.42 | 2141.0430 | 19 | 0.5 | 1071.5293 | 76.68 | 5 | F5:24399 | Fraction\_5\_23092022.raw |  |  |  |  | 1.5659E5 |  |  | 1 | 0 | 0 | 0 | 0 | 1 | 0 | 0 | TRINITY\_DN33420\_c0\_g1\_i1.p1 |  |  | PEAKS DB |
| ILVENQGHLNYGNEIHDFK | 60.38 | 2239.1021 | 19 | 0.3 | 560.7830 | 46.20 | 1 | F1:9880 | Fraction\_1\_23092022r.raw | 2.4826E5 |  |  |  |  |  |  | 1 | 1 | 0 | 0 | 0 | 0 | 0 | 0 | TRINITY\_DN11986\_c0\_g1\_i3.p1 |  |  | PEAKS DB |
| ISSVLAGGSC(+57.02)RAPSTYGGGLSVSSR | 60.37 | 2425.2019 | 25 | -0.6 | 809.4074 | 45.90 | 7 | F7:13457 | Fraction\_7\_23092022.raw |  | 1.265E5 |  |  |  |  | 1.6022E5 | 2 | 0 | 1 | 0 | 0 | 0 | 0 | 1 | P08779|K1C16\_HUMAN | Carbamidomethylation | C10:Carbamidomethylation:1000.00 | PEAKS DB |
| EIIAILPVEELKELHK | 60.28 | 1873.0924 | 16 | -0.3 | 625.3712 | 68.82 | 7 | F7:26410 | Fraction\_7\_23092022.raw |  |  |  |  |  |  | 1.9268E6 | 1 | 0 | 0 | 0 | 0 | 0 | 0 | 1 | TRINITY\_DN13586\_c0\_g2\_i1.p1 |  |  | PEAKS DB |
| YQDVLLPSVYVK | 60.27 | 1422.7759 | 12 | 0.5 | 712.3956 | 66.01 | 5 | F5:19561 | Fraction\_5\_23092022.raw | 1.8277E5 |  | 5.1958E6 | 3.3139E5 | 4.1258E5 | 3.5187E6 |  | 5 | 1 | 0 | 1 | 1 | 1 | 1 | 0 | TRINITY\_DN2228\_c0\_g4\_i1.p1 |  |  | PEAKS DB |
| YLVGQNIAK | 60.09 | 1004.5654 | 9 | 0.2 | 503.2901 | 36.33 | 7 | F7:9050 | Fraction\_7\_23092022.raw |  |  |  | 8.847E5 |  | 1.0698E7 | 1.2472E7 | 3 | 0 | 0 | 0 | 1 | 0 | 1 | 1 | TRINITY\_DN6370\_c0\_g1\_i1.p1 |  |  | PEAKS DB |
| LDSELKNM(+15.99)QDM(+15.99)VEDYR | 60.09 | 2016.8768 | 16 | 0.4 | 673.2998 | 36.31 | 1 | F1:5806 | Fraction\_1\_23092022r.raw | 6.035E5 |  |  |  |  |  |  | 1 | 1 | 0 | 0 | 0 | 0 | 0 | 0 | P04264|K2C1\_HUMAN | Oxidation (M) | M8:Oxidation (M):1000.00;M11:Oxidation (M):1000.00 | PEAKS DB |
| AVQYFTEC(+57.02)IRHEC(+57.02)C(+57.02)LIGVPQSKNPQPVSK | 59.81 | 3444.6741 | 29 | 9.4 | 689.9485 | 44.51 | 5 | F5:9955 | Fraction\_5\_23092022.raw |  |  |  |  | 7.6272E5 |  | 7.6637E5 | 2 | 0 | 0 | 0 | 0 | 1 | 0 | 1 | C0HLL3|PA1\_VESVE | Carbamidomethylation | C8:Carbamidomethylation:1000.00;C13:Carbamidomethylation:1000.00;C14:Carbamidomethylation:1000.00 | PEAKS DB |
| EIALSAISYLKK | 59.67 | 1334.7809 | 12 | -0.2 | 668.3976 | 57.01 | 2 | F2:14857 | Fraction\_2\_23092022.raw |  | 1.2737E6 | 2.6943E5 |  | 4.4535E5 |  |  | 3 | 0 | 1 | 1 | 0 | 1 | 0 | 0 | TRINITY\_DN10256\_c0\_g10\_i1.p1:TRINITY\_DN10256\_c0\_g5\_i4.p1 |  |  | PEAKS DB |
| RELTPDQRIGLVQGR | 59.62 | 1736.9645 | 15 | -1.5 | 435.2477 | 34.51 | 6 | F6:6210 | Fraction\_6\_23092022.raw |  |  |  |  |  | 6.6849E4 |  | 1 | 0 | 0 | 0 | 0 | 0 | 1 | 0 | TRINITY\_DN2228\_c0\_g2\_i2.p1:TRINITY\_DN2228\_c0\_g2\_i1.p1 |  |  | PEAKS DB |
| QSTTAASFAPVSNM(+15.99)VK | 59.32 | 1653.8032 | 16 | 0.6 | 827.9094 | 40.92 | 6 | F6:8967 | Fraction\_6\_23092022.raw |  |  |  | 5.0088E5 |  | 7.6404E5 | 1.1568E5 | 3 | 0 | 0 | 0 | 1 | 0 | 1 | 1 | TRINITY\_DN6370\_c0\_g1\_i1.p1 | Oxidation (M) | M14:Oxidation (M):1000.00 | PEAKS DB |
| WTLLQEQGTK | 59.30 | 1202.6295 | 10 | -0.2 | 602.3219 | 51.37 | 2 | F2:12309 | Fraction\_2\_23092022.raw |  | 1.3976E5 |  |  |  |  |  | 1 | 0 | 1 | 0 | 0 | 0 | 0 | 0 | P48668|K2C6C\_HUMAN:P13647|K2C5\_HUMAN |  |  | PEAKS DB |
| ELGIC(+57.02)PDDAAVIPIK | 59.23 | 1609.8385 | 15 | -0.3 | 805.9263 | 68.14 | 1 | F1:19670 | Fraction\_1\_23092022r.raw | 2.3594E5 |  |  |  |  |  |  | 1 | 1 | 0 | 0 | 0 | 0 | 0 | 0 | P12273|PIP\_HUMAN | Carbamidomethylation | C5:Carbamidomethylation:1000.00 | PEAKS DB |
| VAPEEHPVLLTEAPLNPK | 59.19 | 1953.0570 | 18 | -0.4 | 652.0260 | 52.34 | 1 | F1:12620 | Fraction\_1\_23092022r.raw | 2.3939E5 |  |  |  |  |  |  | 1 | 1 | 0 | 0 | 0 | 0 | 0 | 0 | TRINITY\_DN273\_c0\_g4\_i11.p1:P83750|ACTB\_CYPCA:P02572|ACT2\_DROME:P60713|ACTB\_SHEEP:Q4R561|ACTB\_MACFA:Q76N69|ACTB\_CHLAE:Q4L0Y2|ACTB\_SPECI:TRINITY\_DN273\_c0\_g4\_i2.p1:TRINITY\_DN273\_c0\_g4\_i4.p1:TRINITY\_DN273\_c0\_g4\_i9.p1:P84856|ACTB\_CHLPG:P63258|ACTG\_BOVIN:P63260|ACTG\_MOUSE:P63259|ACTG\_RAT:A2BDB0|ACTG\_XENLA:Q8JJB8|ACTG\_TRISC:Q5JAK2|ACTG\_PELLE:P15475|ACTB\_XENBO:TRINITY\_DN836\_c0\_g1\_i1.p1:P10987|ACT1\_DROME:TRINITY\_DN836\_c0\_g1\_i3.p1:P68556|ACT1\_DIBDE:TRINITY\_DN836\_c0\_g1\_i2.p1:P04829|ACT3\_BOMMO |  |  | PEAKS DB |
| VPQVSTPTLVEVSR | 59.16 | 1510.8354 | 14 | 0.5 | 756.4254 | 53.78 | 3 | F3:13777 | Fraction\_3\_23092022.raw | 1.692E5 |  | 1.8626E5 |  |  |  |  | 2 | 1 | 0 | 1 | 0 | 0 | 0 | 0 | P02768|ALBU\_HUMAN:P02769|ALBU\_BOVIN |  |  | PEAKS DB |
| TLLDIDNTR | 59.12 | 1059.5560 | 9 | 0.6 | 530.7856 | 48.06 | 7 | F7:14511 | Fraction\_7\_23092022.raw | 6.3217E6 | 4.7772E6 | 6.1536E6 | 6.3721E5 |  | 1.0117E6 | 4.789E5 | 6 | 1 | 1 | 1 | 1 | 0 | 1 | 1 | P35527|K1C9\_HUMAN |  |  | PEAKS DB |
| TLLDLDNTR | 59.12 | 1059.5560 | 9 | 0.6 | 530.7856 | 48.06 | 7 | F7:14511 | Fraction\_7\_23092022.raw | 6.3217E6 | 4.7772E6 | 6.1536E6 | 6.3721E5 |  | 1.0117E6 | 4.789E5 | 6 | 1 | 1 | 1 | 1 | 0 | 1 | 1 |  |  |  | PEAKS DB |
| SRDPNRTPFQWSYVR | 59.04 | 1907.9391 | 15 | 3.4 | 477.9937 | 46.49 | 2 | F2:10101 | Fraction\_2\_23092022.raw |  | 6.5811E6 |  |  |  |  |  | 1 | 0 | 1 | 0 | 0 | 0 | 0 | 0 | TRINITY\_DN1342\_c0\_g1\_i29.p1 |  |  | PEAKS DB |
| LIPLDKLGAVLER | 59.03 | 1435.8762 | 13 | 0.0 | 479.6327 | 68.92 | 5 | F5:20876 | Fraction\_5\_23092022.raw |  |  |  | 9.9343E4 | 2.2425E5 | 2.1566E7 | 4.1166E6 | 5 | 0 | 0 | 0 | 1 | 1 | 2 | 1 | TRINITY\_DN13586\_c0\_g2\_i1.p1 |  |  | PEAKS DB |
| AWADEVKDYQYGSPNKGRK | 58.93 | 2211.0708 | 19 | -0.4 | 553.7748 | 35.02 | 7 | F7:8490 | Fraction\_7\_23092022.raw |  |  |  |  |  |  | 5.578E5 | 1 | 0 | 0 | 0 | 0 | 0 | 0 | 1 | TRINITY\_DN6370\_c0\_g1\_i1.p1 |  |  | PEAKS DB |
| EIIAILPVEELK | 58.89 | 1365.8119 | 12 | 0.3 | 683.9135 | 78.20 | 7 | F7:31056 | Fraction\_7\_23092022.raw |  |  |  |  |  |  | 1.3553E7 | 1 | 0 | 0 | 0 | 0 | 0 | 0 | 1 | TRINITY\_DN13586\_c0\_g2\_i1.p1 |  |  | PEAKS DB |
| TSFSPC(+57.02)VPQC(+57.02)QTQGSYGSFTEQHR | 58.89 | 2788.1970 | 24 | 0.9 | 930.4071 | 47.53 | 1 | F1:10450 | Fraction\_1\_23092022r.raw | 2.8275E5 |  |  |  |  |  |  | 1 | 1 | 0 | 0 | 0 | 0 | 0 | 0 | Q5T749|KPRP\_HUMAN | Carbamidomethylation | C6:Carbamidomethylation:1000.00;C10:Carbamidomethylation:1000.00 | PEAKS DB |
| EFLNLGVLTYAK | 58.80 | 1366.7496 | 12 | 0.0 | 684.3821 | 77.49 | 4 | F4:24958 | Fraction\_4\_23092022.raw |  |  | 3.6571E5 | 1.0821E5 |  | 3.7818E5 |  | 3 | 0 | 0 | 1 | 1 | 0 | 1 | 0 | TRINITY\_DN4312\_c0\_g1\_i15.p1:TRINITY\_DN4312\_c0\_g1\_i7.p1:TRINITY\_DN4312\_c0\_g1\_i9.p1 |  |  | PEAKS DB |
| FISYLSDQK | 58.72 | 1099.5549 | 9 | 0.1 | 550.7848 | 45.40 | 7 | F7:13206 | Fraction\_7\_23092022.raw |  |  |  |  |  |  | 8.7298E6 | 1 | 0 | 0 | 0 | 0 | 0 | 0 | 1 | TRINITY\_DN13586\_c0\_g2\_i1.p1 |  |  | PEAKS DB |
| GVDGFRIDAVPHLFESEDLRDEPR | 58.67 | 2768.3518 | 24 | 0.0 | 693.0952 | 66.25 | 2 | F2:19048 | Fraction\_2\_23092022.raw |  | 1.7185E5 |  |  |  |  |  | 1 | 0 | 1 | 0 | 0 | 0 | 0 | 0 | TRINITY\_DN9761\_c0\_g1\_i1.p1 |  |  | PEAKS DB |
| FYTIEILKVE | 58.67 | 1253.6907 | 10 | 0.0 | 627.8526 | 74.00 | 6 | F6:23803 | Fraction\_6\_23092022.raw | 2.1299E6 | 1.2382E6 | 3.5017E5 | 5.2023E5 | 6.6449E5 | 1.8421E6 | 1.2852E6 | 7 | 1 | 1 | 1 | 1 | 1 | 1 | 1 | P12273|PIP\_HUMAN |  |  | PEAKS DB |
| INKEVSIDLVR | 58.65 | 1284.7401 | 11 | -0.6 | 429.2537 | 41.36 | 7 | F7:11343 | Fraction\_7\_23092022.raw |  |  | 1.0761E7 | 7.7769E6 | 3.5163E7 | 1.1978E8 | 5.5704E6 | 9 | 0 | 0 | 2 | 1 | 2 | 2 | 2 | TRINITY\_DN2228\_c0\_g4\_i1.p1 |  |  | PEAKS DB |
| GRFLLIGYDFQK | 58.63 | 1455.7874 | 12 | 1.0 | 486.2702 | 63.64 | 7 | F7:23697 | Fraction\_7\_23092022.raw |  |  |  |  |  | 2.0583E5 | 2.8844E6 | 2 | 0 | 0 | 0 | 0 | 0 | 1 | 1 | TRINITY\_DN3450\_c0\_g1\_i51.p1 |  |  | PEAKS DB |
| KELPLVKDLYVRANGYESK | 58.56 | 2221.2107 | 19 | -0.1 | 556.3099 | 48.72 | 7 | F7:14912 | Fraction\_7\_23092022.raw |  |  |  |  |  |  | 2.6876E6 | 1 | 0 | 0 | 0 | 0 | 0 | 0 | 1 | TRINITY\_DN3450\_c0\_g1\_i51.p1 |  |  | PEAKS DB |
| VTM(+15.99)QNLNDRLASYLDKVR | 58.54 | 2151.1106 | 18 | 0.9 | 538.7854 | 56.01 | 1 | F1:14301 | Fraction\_1\_23092022r.raw | 7.88E6 | 8.0553E5 |  |  |  |  |  | 3 | 2 | 1 | 0 | 0 | 0 | 0 | 0 | P13645|K1C10\_HUMAN:P02533|K1C14\_HUMAN:P08779|K1C16\_HUMAN | Oxidation (M) | M3:Oxidation (M):1000.00 | PEAKS DB |
| KLVVIIDPHIK | 58.49 | 1273.8121 | 11 | -0.7 | 425.6110 | 45.20 | 1 | F1:9404 | Fraction\_1\_23092022r.raw | 3.2703E6 | 1.6077E5 |  |  |  |  |  | 2 | 1 | 1 | 0 | 0 | 0 | 0 | 0 | TRINITY\_DN1709\_c0\_g1\_i4.p1:TRINITY\_DN1709\_c0\_g1\_i5.p1:TRINITY\_DN1709\_c0\_g1\_i6.p1:TRINITY\_DN1709\_c0\_g1\_i8.p1 |  |  | PEAKS DB |
| FNPC(+57.02)PYSDDTVK | 58.37 | 1441.6184 | 12 | 0.6 | 721.8169 | 39.39 | 7 | F7:10521 | Fraction\_7\_23092022.raw |  |  | 2.4537E7 |  |  |  | 5.0642E7 | 2 | 0 | 0 | 1 | 0 | 0 | 0 | 1 | TRINITY\_DN2326\_c0\_g1\_i13.p1:A0A0M3KKW3|PA1\_VESBA | Carbamidomethylation | C4:Carbamidomethylation:1000.00 | PEAKS DB |
| NEDVIHTVIR | 58.26 | 1194.6356 | 10 | 0.2 | 598.3252 | 41.03 | 1 | F1:7672 | Fraction\_1\_23092022r.raw | 8.5911E6 |  |  |  |  |  |  | 2 | 2 | 0 | 0 | 0 | 0 | 0 | 0 | TRINITY\_DN4276\_c0\_g1\_i1.p1 |  |  | PEAKS DB |
| SRTEAESWYQTKYEELQQTAGR | 58.16 | 2660.2466 | 22 | 0.0 | 666.0689 | 50.00 | 1 | F1:11570 | Fraction\_1\_23092022r.raw | 3.189E5 |  |  |  |  |  |  | 1 | 1 | 0 | 0 | 0 | 0 | 0 | 0 | P13647|K2C5\_HUMAN |  |  | PEAKS DB |
| WIITAGHC(+57.02)INAVPSIGAFLIK | 58.10 | 2280.2451 | 21 | 0.5 | 761.0894 | 81.61 | 6 | F6:27215 | Fraction\_6\_23092022.raw |  |  |  |  |  | 9.1369E5 | 4.6881E6 | 2 | 0 | 0 | 0 | 0 | 0 | 1 | 1 | TRINITY\_DN33420\_c0\_g1\_i1.p1 | Carbamidomethylation | C8:Carbamidomethylation:1000.00 | PEAKS DB |
| VTMQNLNDRLASYLDKVR | 58.08 | 2135.1157 | 18 | 0.3 | 712.7127 | 63.69 | 1 | F1:17659 | Fraction\_1\_23092022r.raw | 2.701E6 |  |  |  |  |  |  | 2 | 2 | 0 | 0 | 0 | 0 | 0 | 0 | P13645|K1C10\_HUMAN:P02533|K1C14\_HUMAN:P08779|K1C16\_HUMAN |  |  | PEAKS DB |
| M(+15.99)LVQQYNVPMANIR | 57.94 | 1691.8488 | 14 | -0.7 | 846.9311 | 56.44 | 1 | F1:14434 | Fraction\_1\_23092022r.raw | 1.9266E5 |  |  |  |  |  |  | 1 | 1 | 0 | 0 | 0 | 0 | 0 | 0 |  | Oxidation (M) | M1:Oxidation (M):140.79 | PEAKS DB |
| GLETRGNPGPQPPAK | 57.93 | 1517.7949 | 15 | -0.4 | 506.9387 | 22.00 | 7 | F7:3354 | Fraction\_7\_23092022.raw |  |  |  |  |  |  | 7.2952E4 | 1 | 0 | 0 | 0 | 0 | 0 | 0 | 1 | TRINITY\_DN6370\_c0\_g1\_i1.p1:P35782|VA52\_VESCR:P35781|VA51\_VESCR:P0DMB9|VA5\_VESVE |  |  | PEAKS DB |
| LLRDYQELMNTK | 57.86 | 1522.7814 | 12 | -0.8 | 508.6006 | 47.69 | 1 | F1:10527 | Fraction\_1\_23092022r.raw | 5.6309E5 |  |  |  |  |  |  | 1 | 1 | 0 | 0 | 0 | 0 | 0 | 0 | P04264|K2C1\_HUMAN:Q3TTY5|K22E\_MOUSE |  |  | PEAKS DB |
| TTGNAGGRLAC(+57.02)GVIGITKSG | 57.81 | 1888.9789 | 20 | 0.1 | 630.6670 | 43.16 | 6 | F6:9980 | Fraction\_6\_23092022.raw |  |  |  |  |  | 7.2697E4 |  | 1 | 0 | 0 | 0 | 0 | 0 | 1 | 0 | TRINITY\_DN1931\_c0\_g1\_i1.p1:TRINITY\_DN1931\_c0\_g1\_i2.p1:TRINITY\_DN1931\_c0\_g1\_i4.p1 | Carbamidomethylation | C11:Carbamidomethylation:1000.00 | PEAKS DB |
| SVVTVIDVFYK | 57.75 | 1268.7017 | 11 | -0.2 | 635.3580 | 76.64 | 3 | F3:24038 | Fraction\_3\_23092022.raw | 3.9196E5 |  | 1.3817E5 |  |  |  |  | 2 | 1 | 0 | 1 | 0 | 0 | 0 | 0 | Q5D862|FILA2\_HUMAN |  |  | PEAKS DB |
| TLNDM(+15.99)RQEYEQLIAK | 57.71 | 1866.9146 | 15 | 0.4 | 623.3124 | 47.08 | 2 | F2:10362 | Fraction\_2\_23092022.raw |  | 2.9847E6 |  |  | 2.0646E5 | 1.1581E6 |  | 5 | 0 | 2 | 0 | 0 | 1 | 2 | 0 | P35527|K1C9\_HUMAN | Oxidation (M) | M5:Oxidation (M):1000.00 | PEAKS DB |
| IAQVWASQC(+57.02)K | 57.63 | 1189.5913 | 10 | -0.5 | 595.8026 | 36.74 | 6 | F6:7122 | Fraction\_6\_23092022.raw |  |  |  |  |  | 5.8961E4 |  | 1 | 0 | 0 | 0 | 0 | 0 | 1 | 0 | TRINITY\_DN6370\_c0\_g1\_i1.p1 | Carbamidomethylation | C9:Carbamidomethylation:1000.00 | PEAKS DB |
| YLDGLTAER | 57.62 | 1036.5189 | 9 | 0.2 | 519.2668 | 43.30 | 1 | F1:8634 | Fraction\_1\_23092022r.raw | 2.7187E6 |  |  |  |  |  |  | 1 | 1 | 0 | 0 | 0 | 0 | 0 | 0 | P35908|K22E\_HUMAN |  |  | PEAKS DB |
| VNPSGVVILR | 57.58 | 1052.6342 | 10 | -0.5 | 527.3241 | 48.05 | 2 | F2:10800 | Fraction\_2\_23092022.raw | 1.3489E5 | 2.0299E6 |  |  |  | 7.2177E5 |  | 3 | 1 | 1 | 0 | 0 | 0 | 1 | 0 | TRINITY\_DN2611\_c0\_g1\_i12.p1:TRINITY\_DN2611\_c0\_g1\_i23.p1:TRINITY\_DN2611\_c0\_g1\_i19.p1:TRINITY\_DN2611\_c0\_g1\_i9.p1 |  |  | PEAKS DB |
| DATNLGYYVKTR | 57.56 | 1399.7096 | 12 | -0.8 | 467.5768 | 42.28 | 1 | F1:8171 | Fraction\_1\_23092022r.raw | 1.1559E5 |  |  |  |  |  |  | 2 | 2 | 0 | 0 | 0 | 0 | 0 | 0 | TRINITY\_DN1709\_c0\_g1\_i4.p1:TRINITY\_DN1709\_c0\_g1\_i5.p1:TRINITY\_DN1709\_c0\_g1\_i6.p1:TRINITY\_DN1709\_c0\_g1\_i8.p1 |  |  | PEAKS DB |
| VKVNPSGVVILR | 57.54 | 1279.7976 | 12 | 0.8 | 427.6068 | 43.60 | 2 | F2:8807 | Fraction\_2\_23092022.raw |  | 1.1973E6 |  |  |  | 4.1132E5 |  | 2 | 0 | 1 | 0 | 0 | 0 | 1 | 0 | TRINITY\_DN2611\_c0\_g1\_i12.p1:TRINITY\_DN2611\_c0\_g1\_i23.p1:TRINITY\_DN2611\_c0\_g1\_i19.p1:TRINITY\_DN2611\_c0\_g1\_i9.p1 |  |  | PEAKS DB |
| TLNNDIMLIK | 57.43 | 1173.6427 | 10 | 0.4 | 587.8289 | 57.88 | 4 | F4:16144 | Fraction\_4\_23092022.raw | 1.0805E9 |  | 8.4451E5 | 4.535E6 | 1.3832E6 |  |  | 4 | 1 | 0 | 1 | 1 | 1 | 0 | 0 | P07477|TRY1\_HUMAN:P00763|TRY2\_RAT |  |  | PEAKS DB |
| NYSPYYNTIDDLK | 57.41 | 1604.7358 | 13 | -0.2 | 803.3751 | 56.42 | 1 | F1:14439 | Fraction\_1\_23092022r.raw | 3.4112E5 |  |  |  |  |  |  | 1 | 1 | 0 | 0 | 0 | 0 | 0 | 0 | P35527|K1C9\_HUMAN |  |  | PEAKS DB |
| LIFAGKQLEDGR | 57.40 | 1345.7354 | 12 | 0.8 | 449.5861 | 41.76 | 7 | F7:11524 | Fraction\_7\_23092022.raw |  |  | 7.085E4 |  |  | 1.6527E5 | 3.3803E5 | 3 | 0 | 0 | 1 | 0 | 0 | 1 | 1 | P62972|UBIQP\_XENLA:P0CG65|UBB\_PANTR:P0CG62|UBB\_CHICK:P0CG51|UBB\_RAT:P0CG68|UBC\_PIG:P62976|UBIQP\_CRIGR:P0CG48|UBC\_HUMAN:P29504|RS27A\_MANSE:P15357|RS27A\_DROME:P62979|RS27A\_HUMAN |  |  | PEAKS DB |
| HFC(+57.02)GGSILNEK | 57.39 | 1260.5920 | 11 | -0.2 | 421.2045 | 32.09 | 7 | F7:7219 | Fraction\_7\_23092022.raw |  |  |  |  |  |  | 8.5097E5 | 2 | 0 | 0 | 0 | 0 | 0 | 0 | 2 | TRINITY\_DN33420\_c0\_g1\_i1.p1 | Carbamidomethylation | C3:Carbamidomethylation:1000.00 | PEAKS DB |
| QSVEADINGLR | 57.26 | 1200.6099 | 11 | 0.7 | 601.3126 | 44.12 | 4 | F4:9998 | Fraction\_4\_23092022.raw | 2.8401E6 | 5.2058E5 | 4.4994E5 | 1.64E5 | 1.0317E5 | 1.7377E5 |  | 6 | 1 | 1 | 1 | 1 | 1 | 1 | 0 | P13645|K1C10\_HUMAN |  |  | PEAKS DB |
| YFTEC(+57.02)IR | 57.25 | 987.4484 | 7 | -0.1 | 494.7314 | 38.90 | 6 | F6:8073 | Fraction\_6\_23092022.raw |  |  | 1.0487E6 | 1.9714E6 | 7.3924E5 | 7.9648E5 |  | 4 | 0 | 0 | 1 | 1 | 1 | 1 | 0 |  | Carbamidomethylation | C5:Carbamidomethylation:1000.00 | PEAKS DB |
| LVVIIDPHIK | 57.23 | 1145.7172 | 10 | -0.9 | 382.9127 | 53.58 | 1 | F1:13154 | Fraction\_1\_23092022r.raw | 9.6738E5 |  |  |  |  |  |  | 2 | 2 | 0 | 0 | 0 | 0 | 0 | 0 | TRINITY\_DN1709\_c0\_g1\_i4.p1:TRINITY\_DN1709\_c0\_g1\_i5.p1:TRINITY\_DN1709\_c0\_g1\_i6.p1:TRINITY\_DN1709\_c0\_g1\_i8.p1 |  |  | PEAKS DB |
| ALVIHAKPDDFGRGGTEESIKTGSSGAR | 57.20 | 2855.4524 | 28 | -0.1 | 572.0977 | 32.95 | 6 | F6:5572 | Fraction\_6\_23092022.raw |  |  |  |  |  | 3.894E5 |  | 1 | 0 | 0 | 0 | 0 | 0 | 1 | 0 | TRINITY\_DN16204\_c0\_g1\_i2.p1 |  |  | PEAKS DB |
| GDVNTQIALGPALK | 57.17 | 1395.7721 | 14 | -0.1 | 698.8932 | 53.06 | 6 | F6:14440 | Fraction\_6\_23092022.raw |  |  |  |  |  | 1.1528E5 |  | 1 | 0 | 0 | 0 | 0 | 0 | 1 | 0 | TRINITY\_DN3717\_c3\_g1\_i1.p1 |  |  | PEAKS DB |
| VGWRSQDLPIFVR | 57.16 | 1571.8572 | 13 | 0.8 | 524.9601 | 63.62 | 1 | F1:17600 | Fraction\_1\_23092022r.raw | 9.1183E6 |  |  |  |  |  |  | 1 | 1 | 0 | 0 | 0 | 0 | 0 | 0 | TRINITY\_DN4276\_c0\_g1\_i1.p1 |  |  | PEAKS DB |
| LVAASQAALGL | 57.07 | 1012.5917 | 11 | 0.6 | 507.3034 | 65.53 | 1 | F1:18514 | Fraction\_1\_23092022r.raw | 2.3598E5 | 1.6952E5 |  |  |  |  |  | 2 | 1 | 1 | 0 | 0 | 0 | 0 | 0 | P02768|ALBU\_HUMAN |  |  | PEAKS DB |
| NIKNAVVTIEINQNVATLRIC(+57.02)R | 57.03 | 2538.4065 | 22 | 0.4 | 635.6091 | 60.02 | 1 | F1:16039 | Fraction\_1\_23092022r.raw | 9.9085E5 |  |  |  |  |  |  | 1 | 1 | 0 | 0 | 0 | 0 | 0 | 0 | TRINITY\_DN4276\_c0\_g1\_i1.p1 | Carbamidomethylation | C21:Carbamidomethylation:1000.00 | PEAKS DB |
| VTMQNLNDRLASYLDK | 56.99 | 1879.9462 | 16 | -0.8 | 627.6555 | 61.55 | 1 | F1:16687 | Fraction\_1\_23092022r.raw | 5.9999E5 |  |  |  |  |  |  | 1 | 1 | 0 | 0 | 0 | 0 | 0 | 0 | P13645|K1C10\_HUMAN:P02533|K1C14\_HUMAN:P08779|K1C16\_HUMAN |  |  | PEAKS DB |
| MTLDDFR | 56.97 | 896.4062 | 7 | 0.4 | 449.2106 | 51.02 | 1 | F1:12050 | Fraction\_1\_23092022r.raw | 1.0123E6 |  |  |  |  |  |  | 1 | 1 | 0 | 0 | 0 | 0 | 0 | 0 | P35527|K1C9\_HUMAN |  |  | PEAKS DB |
| NLADYIHSKGLK | 56.96 | 1357.7354 | 12 | 0.0 | 453.5857 | 42.00 | 2 | F2:8063 | Fraction\_2\_23092022.raw |  | 2.7559E6 |  |  |  |  |  | 3 | 0 | 3 | 0 | 0 | 0 | 0 | 0 | TRINITY\_DN2611\_c0\_g1\_i12.p1:TRINITY\_DN2611\_c0\_g1\_i23.p1:TRINITY\_DN2611\_c0\_g1\_i19.p1:TRINITY\_DN2611\_c0\_g1\_i9.p1 |  |  | PEAKS DB |
| FLLIGYDFQK | 56.95 | 1242.6648 | 10 | 0.5 | 622.3400 | 74.96 | 6 | F6:24216 | Fraction\_6\_23092022.raw |  |  | 2.8801E5 | 6.4509E5 | 3.5338E5 | 1.7797E6 |  | 4 | 0 | 0 | 1 | 1 | 1 | 1 | 0 | TRINITY\_DN3450\_c0\_g1\_i51.p1 |  |  | PEAKS DB |
| ALEESNYELEGKIK | 56.95 | 1621.8198 | 14 | 0.5 | 811.9176 | 39.50 | 1 | F1:6978 | Fraction\_1\_23092022r.raw | 4.8202E5 |  |  |  |  |  |  | 2 | 2 | 0 | 0 | 0 | 0 | 0 | 0 | P13645|K1C10\_HUMAN |  |  | PEAKS DB |
| TLNNDIM(+15.99)LIK | 56.92 | 1189.6377 | 10 | -0.5 | 595.8258 | 50.96 | 5 | F5:12811 | Fraction\_5\_23092022.raw | 1.6613E9 | 1.8884E8 | 5.1004E7 | 7.0393E7 | 7.6514E7 | 6.0169E7 | 4.1853E7 | 8 | 2 | 1 | 1 | 1 | 1 | 1 | 1 | P07477|TRY1\_HUMAN:P00763|TRY2\_RAT | Oxidation (M) | M7:Oxidation (M):1000.00 | PEAKS DB |
| SLVNLGGSKSISISVARGGGR | 56.80 | 2014.1283 | 21 | 0.4 | 504.5396 | 46.34 | 2 | F2:10048 | Fraction\_2\_23092022.raw | 2.8919E5 | 2.0375E5 |  |  |  |  |  | 2 | 1 | 1 | 0 | 0 | 0 | 0 | 0 | P04264|K2C1\_HUMAN |  |  | PEAKS DB |
| LAELEEALQK | 56.77 | 1142.6183 | 10 | 0.5 | 572.3167 | 48.80 | 3 | F3:11578 | Fraction\_3\_23092022.raw | 3.8165E5 | 8.1985E5 | 9.5108E5 | 1.8174E5 |  |  |  | 4 | 1 | 1 | 1 | 1 | 0 | 0 | 0 | P13647|K2C5\_HUMAN |  |  | PEAKS DB |
| FLPSFLAVEEM(+15.99)R | 56.76 | 1453.7275 | 12 | 0.6 | 727.8715 | 71.98 | 6 | F6:22974 | Fraction\_6\_23092022.raw |  |  |  |  |  | 1.1595E6 |  | 1 | 0 | 0 | 0 | 0 | 0 | 1 | 0 | TRINITY\_DN5723\_c0\_g1\_i5.p1:TRINITY\_DN5723\_c0\_g1\_i7.p1 | Oxidation (M) | M11:Oxidation (M):1000.00 | PEAKS DB |
| FLEQQNQVLETK | 56.69 | 1475.7620 | 12 | 0.1 | 738.8883 | 43.35 | 3 | F3:9095 | Fraction\_3\_23092022.raw |  | 7.6243E4 | 1.7453E5 |  |  |  |  | 2 | 0 | 1 | 1 | 0 | 0 | 0 | 0 | Q6IME9|K2C72\_MOUSE:Q86Y46|K2C73\_HUMAN:Q3SY84|K2C71\_HUMAN:Q148H5|K2C71\_BOVIN:Q14CN4|K2C72\_HUMAN:E1AB55|K2C71\_FELCA:Q6IG04|K2C72\_RAT:Q7RTS7|K2C74\_HUMAN |  |  | PEAKS DB |
| TVDQYYFGSQSK | 56.57 | 1421.6462 | 12 | 0.6 | 711.8308 | 42.69 | 5 | F5:9150 | Fraction\_5\_23092022.raw |  | 7.6837E4 |  |  | 8.0396E4 |  |  | 2 | 0 | 1 | 0 | 0 | 1 | 0 | 0 | TRINITY\_DN2373\_c0\_g1\_i19.p1 |  |  | PEAKS DB |
| DSC(+57.02)QGDSGGPVVC(+57.02)NGQLQGIVSWGYGC(+57.02)AQKR | 56.51 | 3339.4819 | 31 | 5.7 | 1114.1743 | 55.52 | 1 | F1:14028 | Fraction\_1\_23092022r.raw | 1.6515E6 |  |  |  |  |  |  | 2 | 2 | 0 | 0 | 0 | 0 | 0 | 0 | Q8NHM4|TRY6\_HUMAN | Carbamidomethylation | C3:Carbamidomethylation:1000.00;C13:Carbamidomethylation:1000.00;C27:Carbamidomethylation:1000.00 | PEAKS DB |
| HGVQELEIELQSQLSKKAALEK | 56.31 | 2477.3489 | 22 | 0.4 | 620.3447 | 57.32 | 6 | F6:16369 | Fraction\_6\_23092022.raw |  | 9.7025E5 |  |  |  | 2.3819E5 |  | 2 | 0 | 1 | 0 | 0 | 0 | 1 | 0 | P35527|K1C9\_HUMAN |  |  | PEAKS DB |
| KLGTVEVEDQISVTRK | 56.26 | 1800.9945 | 16 | -0.3 | 601.3386 | 36.67 | 6 | F6:7079 | Fraction\_6\_23092022.raw |  |  | 1.6683E5 |  |  | 5.7131E5 | 2.9486E5 | 3 | 0 | 0 | 1 | 0 | 0 | 1 | 1 | TRINITY\_DN3450\_c0\_g1\_i51.p1 |  |  | PEAKS DB |
| VLSYWWYAYEDK | 56.16 | 1621.7452 | 12 | 0.4 | 811.8802 | 75.16 | 6 | F6:24324 | Fraction\_6\_23092022.raw |  |  |  |  |  | 2.0996E5 |  | 1 | 0 | 0 | 0 | 0 | 0 | 1 | 0 | TRINITY\_DN2228\_c0\_g4\_i1.p1 |  |  | PEAKS DB |
| SKIERNIKNAVVTIEINQNVATLR | 56.14 | 2722.5454 | 24 | 0.4 | 681.6439 | 53.05 | 1 | F1:12925 | Fraction\_1\_23092022r.raw | 2.3763E5 |  |  |  |  |  |  | 1 | 1 | 0 | 0 | 0 | 0 | 0 | 0 | TRINITY\_DN4276\_c0\_g1\_i1.p1 |  |  | PEAKS DB |
| YIPIQYVLSR | 56.05 | 1250.7023 | 10 | 0.2 | 626.3585 | 68.89 | 5 | F5:20866 | Fraction\_5\_23092022.raw | 2.3545E5 | 1.2772E5 |  | 7.2711E4 | 2.7997E5 | 2.3126E5 |  | 5 | 1 | 1 | 0 | 1 | 1 | 1 | 0 | Q28417|CASK\_GIRCA:P11840|CASK\_BUBBU:P02668|CASK\_BOVIN:P50420|CASK\_CAPSU:P50423|CASK\_OREAM:P50422|CASK\_NEMGO:P50421|CASK\_CAPSW:P02670|CASK\_CAPHI:P50424|CASK\_RUPRU:P42156|CASK\_CAPCR:P02669|CASK\_SHEEP:P50425|CASK\_SAITA |  |  | PEAKS DB |
| VAVLDFVTPSPR | 56.04 | 1299.7186 | 12 | 0.5 | 650.8669 | 66.89 | 3 | F3:19685 | Fraction\_3\_23092022.raw |  |  | 1.0731E5 |  |  |  |  | 1 | 0 | 0 | 1 | 0 | 0 | 0 | 0 | TRINITY\_DN1101\_c0\_g1\_i12.p1:TRINITY\_DN1101\_c0\_g1\_i9.p1:TRINITY\_DN1101\_c0\_g1\_i15.p1:TRINITY\_DN1101\_c0\_g1\_i17.p1:TRINITY\_DN1101\_c0\_g1\_i11.p1 |  |  | PEAKS DB |
| LEEQLGVKVFNK | 56.04 | 1402.7820 | 12 | 7.9 | 468.6050 | 47.93 | 5 | F5:11466 | Fraction\_5\_23092022.raw |  |  |  | 4.1763E5 | 1.3375E6 |  |  | 4 | 0 | 0 | 0 | 2 | 2 | 0 | 0 | Q06833|NVJ2\_YEAST |  |  | PEAKS DB |
| LEGLEDALQK | 56.03 | 1114.5869 | 10 | 0.4 | 558.3010 | 49.41 | 3 | F3:11827 | Fraction\_3\_23092022.raw |  |  | 4.0269E5 |  |  |  |  | 1 | 0 | 0 | 1 | 0 | 0 | 0 | 0 | P48668|K2C6C\_HUMAN |  |  | PEAKS DB |
| IDAVPHLFER | 55.84 | 1195.6349 | 10 | 1.2 | 399.5527 | 51.46 | 5 | F5:13030 | Fraction\_5\_23092022.raw |  |  |  |  | 1.2393E5 |  |  | 1 | 0 | 0 | 0 | 0 | 1 | 0 | 0 | TRINITY\_DN1342\_c0\_g1\_i29.p1:TRINITY\_DN1342\_c0\_g1\_i23.p1:TRINITY\_DN1342\_c0\_g1\_i54.p1:TRINITY\_DN1342\_c0\_g1\_i9.p1:TRINITY\_DN1342\_c0\_g1\_i34.p1 |  |  | PEAKS DB |
| NSLISHYKVFKDM(+15.99)IALK | 55.83 | 2022.0972 | 17 | -0.7 | 506.5312 | 47.88 | 2 | F2:10723 | Fraction\_2\_23092022.raw |  | 1.7238E6 |  |  |  |  |  | 2 | 0 | 2 | 0 | 0 | 0 | 0 | 0 | TRINITY\_DN10047\_c0\_g1\_i1.p1 | Oxidation (M) | M13:Oxidation (M):1000.00 | PEAKS DB |
| WDKSM(+15.99)IETEASNRFEK | 55.75 | 1985.9153 | 16 | -0.4 | 497.4859 | 39.04 | 6 | F6:8127 | Fraction\_6\_23092022.raw |  |  |  |  |  | 1.0503E6 |  | 1 | 0 | 0 | 0 | 0 | 0 | 1 | 0 | TRINITY\_DN2228\_c0\_g4\_i1.p1 | Oxidation (M) | M5:Oxidation (M):1000.00 | PEAKS DB |
| VLDELTLTK | 55.72 | 1030.5911 | 9 | 0.1 | 516.3029 | 51.28 | 3 | F3:12634 | Fraction\_3\_23092022.raw | 3.2189E6 | 2.8075E6 | 3.531E6 | 1.112E6 | 1.4529E6 | 8.622E5 | 1.0181E6 | 7 | 1 | 1 | 1 | 1 | 1 | 1 | 1 | P13645|K1C10\_HUMAN |  |  | PEAKS DB |
| NIYSLPLLNPYGR | 55.69 | 1518.8195 | 13 | 0.7 | 760.4175 | 77.54 | 6 | F6:25381 | Fraction\_6\_23092022.raw | 1.4989E6 | 2.3009E6 |  | 4.9542E5 |  | 6.1894E5 |  | 4 | 1 | 1 | 0 | 1 | 0 | 1 | 0 | TRINITY\_DN11986\_c0\_g1\_i3.p1 |  |  | PEAKS DB |
| TLNNQFASFIDKVR | 55.33 | 1651.8682 | 14 | -0.7 | 551.6296 | 64.32 | 1 | F1:17972 | Fraction\_1\_23092022r.raw | 1.7056E5 |  |  |  |  |  |  | 1 | 1 | 0 | 0 | 0 | 0 | 0 | 0 | Q8N1N4|K2C78\_HUMAN |  |  | PEAKS DB |
| HPIWSTWAR | 55.31 | 1152.5829 | 9 | 1.2 | 577.2994 | 47.86 | 1 | F1:10667 | Fraction\_1\_23092022r.raw | 5.1056E7 |  |  |  |  |  |  | 1 | 1 | 0 | 0 | 0 | 0 | 0 | 0 | TRINITY\_DN4276\_c0\_g1\_i1.p1 |  |  | PEAKS DB |
| FVLDGKVYQVSR | 55.17 | 1409.7666 | 12 | 0.6 | 470.9297 | 47.30 | 6 | F6:11874 | Fraction\_6\_23092022.raw |  |  |  |  |  | 3.274E5 |  | 1 | 0 | 0 | 0 | 0 | 0 | 1 | 0 | TRINITY\_DN3922\_c0\_g1\_i1.p1:TRINITY\_DN3922\_c0\_g1\_i2.p1:TRINITY\_DN3922\_c0\_g1\_i3.p1 |  |  | PEAKS DB |
| EIKIEISELNR | 55.12 | 1342.7456 | 11 | -0.3 | 448.5890 | 50.98 | 1 | F1:11996 | Fraction\_1\_23092022r.raw | 7.4809E5 |  |  |  |  |  |  | 1 | 1 | 0 | 0 | 0 | 0 | 0 | 0 | P35908|K22E\_HUMAN |  |  | PEAKS DB |
| NVDNLKINKDISIDLVRK | 54.72 | 2096.1953 | 18 | 0.3 | 525.0563 | 44.25 | 6 | F6:10451 | Fraction\_6\_23092022.raw |  |  |  |  |  | 8.6278E5 |  | 3 | 0 | 0 | 0 | 0 | 0 | 3 | 0 | Q5D7H4|HUGAB\_VESVU |  |  | PEAKS DB |
| ASGLTKQENLEILK | 54.71 | 1542.8617 | 14 | -0.5 | 515.2943 | 46.87 | 7 | F7:13890 | Fraction\_7\_23092022.raw |  |  |  | 6.5316E5 |  |  | 2.4172E6 | 3 | 0 | 0 | 0 | 1 | 0 | 0 | 2 | P35782|VA52\_VESCR:P35781|VA51\_VESCR |  |  | PEAKS DB |
| DVDGAYMTKVDLQAK | 54.58 | 1652.8080 | 15 | -0.1 | 827.4112 | 47.08 | 1 | F1:10267 | Fraction\_1\_23092022r.raw | 4.5839E4 |  |  |  |  |  |  | 1 | 1 | 0 | 0 | 0 | 0 | 0 | 0 | P04264|K2C1\_HUMAN |  |  | PEAKS DB |
| KHDFYTLDTIK | 54.33 | 1379.7085 | 11 | 0.2 | 460.9102 | 38.68 | 7 | F7:10126 | Fraction\_7\_23092022.raw |  |  |  |  |  |  | 6.3068E5 | 1 | 0 | 0 | 0 | 0 | 0 | 0 | 1 | A0A0M3KKW3|PA1\_VESBA |  |  | PEAKS DB |
| IGINGFGR | 54.17 | 832.4555 | 8 | 0.6 | 417.2353 | 44.71 | 6 | F6:10653 | Fraction\_6\_23092022.raw |  |  |  |  |  | 6.6921E5 |  | 1 | 0 | 0 | 0 | 0 | 0 | 1 | 0 | TRINITY\_DN5849\_c0\_g1\_i1.p1:TRINITY\_DN5849\_c0\_g1\_i2.p1:TRINITY\_DN5849\_c0\_g1\_i10.p1 |  |  | PEAKS DB |
| LGINGFGR | 54.17 | 832.4555 | 8 | 0.6 | 417.2353 | 44.71 | 6 | F6:10653 | Fraction\_6\_23092022.raw |  |  |  |  |  | 6.6921E5 |  | 1 | 0 | 0 | 0 | 0 | 0 | 1 | 0 |  |  |  | PEAKS DB |
| SQDLPIFVR | 54.07 | 1073.5869 | 9 | 1.2 | 537.8014 | 60.07 | 1 | F1:16014 | Fraction\_1\_23092022r.raw | 1.3682E9 | 3.9237E7 |  |  |  |  |  | 2 | 1 | 1 | 0 | 0 | 0 | 0 | 0 | TRINITY\_DN4276\_c0\_g1\_i1.p1 |  |  | PEAKS DB |
| VVSQIIPIHER | 53.95 | 1289.7455 | 11 | -0.3 | 430.9223 | 40.75 | 7 | F7:11098 | Fraction\_7\_23092022.raw |  |  |  |  |  |  | 1.4061E6 | 1 | 0 | 0 | 0 | 0 | 0 | 0 | 1 | TRINITY\_DN2373\_c0\_g1\_i19.p1 |  |  | PEAKS DB |
| LLEGEDAHLSSSQFSSGSQSSRDVTSSSR | 53.93 | 3040.3970 | 29 | 0.1 | 761.1066 | 38.62 | 2 | F2:6607 | Fraction\_2\_23092022.raw |  | 8.5264E4 |  |  |  |  |  | 1 | 0 | 1 | 0 | 0 | 0 | 0 | 0 | P02533|K1C14\_HUMAN |  |  | PEAKS DB |
| APSTYGGGLSVSSR | 53.90 | 1337.6575 | 14 | 0.8 | 669.8365 | 34.95 | 6 | F6:6355 | Fraction\_6\_23092022.raw |  |  |  |  |  | 1.0062E5 |  | 1 | 0 | 0 | 0 | 0 | 0 | 1 | 0 | P08779|K1C16\_HUMAN |  |  | PEAKS DB |
| ALGKDGLPIEGPK | 53.89 | 1293.7292 | 13 | -0.5 | 432.2501 | 38.14 | 6 | F6:7740 | Fraction\_6\_23092022.raw |  |  |  |  |  | 1.0075E5 |  | 1 | 0 | 0 | 0 | 0 | 0 | 1 | 0 | TRINITY\_DN16204\_c0\_g1\_i2.p1 |  |  | PEAKS DB |
| GTNYLADVFEKK | 53.73 | 1383.7034 | 12 | 0.2 | 692.8591 | 55.55 | 1 | F1:14033 | Fraction\_1\_23092022r.raw | 1.4307E5 |  |  |  |  |  |  | 1 | 1 | 0 | 0 | 0 | 0 | 0 | 0 | P31151|S10A7\_HUMAN |  |  | PEAKS DB |
| DYQELMNTK | 53.32 | 1140.5121 | 9 | 0.8 | 571.2638 | 43.26 | 1 | F1:8611 | Fraction\_1\_23092022r.raw | 3.6715E4 |  |  |  |  |  |  | 1 | 1 | 0 | 0 | 0 | 0 | 0 | 0 | P04264|K2C1\_HUMAN:Q3TTY5|K22E\_MOUSE |  |  | PEAKS DB |
| GTYIYVDMKDPLFLDQNNYMDK | 53.17 | 2682.2346 | 22 | -0.3 | 895.0852 | 76.13 | 1 | F1:23253 | Fraction\_1\_23092022r.raw | 7.3672E5 |  |  |  |  |  |  | 1 | 1 | 0 | 0 | 0 | 0 | 0 | 0 | TRINITY\_DN4276\_c0\_g1\_i1.p1 |  |  | PEAKS DB |
| FSSSGGGGGGGRFSSSSGYGGGSSR | 53.05 | 2197.9373 | 25 | 0.1 | 733.6531 | 24.57 | 7 | F7:4029 | Fraction\_7\_23092022.raw |  |  |  |  |  |  | 2.6807E5 | 1 | 0 | 0 | 0 | 0 | 0 | 0 | 1 | P35527|K1C9\_HUMAN |  |  | PEAKS DB |
| ELPLVKDLYVRANGYESK | 53.03 | 2093.1157 | 18 | -0.5 | 698.7122 | 58.52 | 7 | F7:20710 | Fraction\_7\_23092022.raw |  |  |  |  |  |  | 1.0339E6 | 2 | 0 | 0 | 0 | 0 | 0 | 0 | 2 | TRINITY\_DN3450\_c0\_g1\_i51.p1 |  |  | PEAKS DB |
| GNPGPQPPAK | 52.98 | 961.4981 | 10 | 0.3 | 481.7565 | 17.36 | 7 | F7:2608 | Fraction\_7\_23092022.raw |  |  |  |  |  |  | 3.8663E4 | 1 | 0 | 0 | 0 | 0 | 0 | 0 | 1 | TRINITY\_DN6370\_c0\_g1\_i1.p1:P35782|VA52\_VESCR:P35781|VA51\_VESCR:P0DMB9|VA5\_VESVE |  |  | PEAKS DB |
| EYVAILPVKEIK | 52.95 | 1400.8279 | 12 | 0.6 | 467.9502 | 53.64 | 7 | F7:17578 | Fraction\_7\_23092022.raw |  |  |  |  |  |  | 3.3602E5 | 1 | 0 | 0 | 0 | 0 | 0 | 0 | 1 | TRINITY\_DN13586\_c0\_g1\_i1.p1 |  |  | PEAKS DB |
| HVLKPEEKIGLVR | 52.93 | 1516.9089 | 13 | -0.5 | 380.2343 | 28.27 | 7 | F7:5567 | Fraction\_7\_23092022.raw |  |  |  |  |  |  | 8.6259E6 | 2 | 0 | 0 | 0 | 0 | 0 | 0 | 2 | TRINITY\_DN2228\_c0\_g4\_i1.p1 |  |  | PEAKS DB |
| QC(+57.02)ANLQNAIADAEQR | 52.83 | 1700.7900 | 15 | 0.2 | 851.4025 | 50.02 | 1 | F1:11583 | Fraction\_1\_23092022r.raw | 1.223E5 |  |  |  |  |  |  | 1 | 1 | 0 | 0 | 0 | 0 | 0 | 0 | P13647|K2C5\_HUMAN | Carbamidomethylation | C2:Carbamidomethylation:1000.00 | PEAKS DB |
| WDKSM(+15.99)IETEASNRFEKSAR | 52.77 | 2300.0854 | 19 | 0.6 | 576.0290 | 37.06 | 6 | F6:7252 | Fraction\_6\_23092022.raw |  |  |  |  |  | 6.0704E5 |  | 1 | 0 | 0 | 0 | 0 | 0 | 1 | 0 | TRINITY\_DN2228\_c0\_g4\_i1.p1 | Oxidation (M) | M5:Oxidation (M):1000.00 | PEAKS DB |
| YLNTNVGSEKDLLLQALGC(+57.02)SR | 52.74 | 2350.1951 | 21 | -0.1 | 784.4055 | 70.62 | 4 | F4:21896 | Fraction\_4\_23092022.raw |  |  |  | 8.0462E4 |  |  |  | 1 | 0 | 0 | 0 | 1 | 0 | 0 | 0 | TRINITY\_DN1113\_c0\_g1\_i95.p1:TRINITY\_DN1113\_c0\_g1\_i47.p1:TRINITY\_DN1113\_c0\_g1\_i59.p1:TRINITY\_DN1113\_c0\_g1\_i53.p1:TRINITY\_DN1113\_c0\_g1\_i73.p1:TRINITY\_DN1113\_c0\_g1\_i6.p1:TRINITY\_DN1113\_c0\_g1\_i12.p1 | Carbamidomethylation | C19:Carbamidomethylation:1000.00 | PEAKS DB |
| NYLNGDATLTQER | 52.50 | 1493.7111 | 13 | 0.5 | 747.8632 | 41.93 | 6 | F6:9406 | Fraction\_6\_23092022.raw |  |  |  |  |  | 7.4213E4 |  | 1 | 0 | 0 | 0 | 0 | 0 | 1 | 0 | TRINITY\_DN1113\_c0\_g1\_i95.p1:TRINITY\_DN1113\_c0\_g1\_i47.p1:TRINITY\_DN1113\_c0\_g1\_i59.p1:TRINITY\_DN1113\_c0\_g1\_i53.p1:TRINITY\_DN1113\_c0\_g1\_i73.p1:TRINITY\_DN1113\_c0\_g1\_i6.p1:TRINITY\_DN1113\_c0\_g1\_i12.p1 |  |  | PEAKS DB |
| YYQYGSDIPFNFK | 52.43 | 1640.7511 | 13 | 0.3 | 821.3831 | 68.00 | 2 | F2:19833 | Fraction\_2\_23092022.raw |  | 1.0668E5 |  |  |  |  |  | 1 | 0 | 1 | 0 | 0 | 0 | 0 | 0 | TRINITY\_DN1342\_c0\_g1\_i23.p1:TRINITY\_DN1342\_c0\_g1\_i54.p1:TRINITY\_DN1342\_c0\_g1\_i34.p1 |  |  | PEAKS DB |
| VC(+57.02)NYVSWIK | 52.34 | 1167.5747 | 9 | 0.5 | 584.7949 | 56.71 | 5 | F5:15392 | Fraction\_5\_23092022.raw | 1.8675E9 |  | 7.4896E6 | 8.5439E7 | 2.1758E8 | 1.3813E8 |  | 7 | 3 | 0 | 1 | 1 | 1 | 1 | 0 | P00760|TRY1\_BOVIN | Carbamidomethylation | C2:Carbamidomethylation:1000.00 | PEAKS DB |
| DAFLGSFLYEYSR | 52.22 | 1566.7354 | 13 | -0.1 | 784.3749 | 82.62 | 1 | F1:26203 | Fraction\_1\_23092022r.raw | 1.5205E6 |  |  |  |  |  |  | 1 | 1 | 0 | 0 | 0 | 0 | 0 | 0 | P02769|ALBU\_BOVIN |  |  | PEAKS DB |
| RHPDYSVVLLLR | 52.15 | 1466.8357 | 12 | 0.4 | 489.9527 | 54.73 | 1 | F1:13663 | Fraction\_1\_23092022r.raw | 2.1143E5 |  |  |  |  |  |  | 1 | 1 | 0 | 0 | 0 | 0 | 0 | 0 | P02768|ALBU\_HUMAN |  |  | PEAKS DB |
| LVVIIDPHIKR | 52.12 | 1301.8184 | 11 | 0.1 | 434.9468 | 44.44 | 1 | F1:9092 | Fraction\_1\_23092022r.raw | 1.3859E6 | 8.6579E4 |  |  |  |  |  | 2 | 1 | 1 | 0 | 0 | 0 | 0 | 0 | TRINITY\_DN1709\_c0\_g1\_i4.p1:TRINITY\_DN1709\_c0\_g1\_i5.p1:TRINITY\_DN1709\_c0\_g1\_i6.p1:TRINITY\_DN1709\_c0\_g1\_i8.p1 |  |  | PEAKS DB |
| GGGGGGYGSGGSSYGSGGGSYGSGGGGGGGR | 51.76 | 2382.9446 | 31 | 0.1 | 1192.4797 | 25.04 | 7 | F7:4209 | Fraction\_7\_23092022.raw |  |  |  |  |  |  | 4.0219E5 | 1 | 0 | 0 | 0 | 0 | 0 | 0 | 1 | P04264|K2C1\_HUMAN |  |  | PEAKS DB |
| APILSDSSC(+57.02)KSAYPGQITSNM(+15.99)FC(+57.02)AGYLEGGK | 51.76 | 3324.5100 | 31 | 0.2 | 832.1349 | 48.71 | 7 | F7:14875 | Fraction\_7\_23092022.raw |  |  |  |  | 1.5856E6 |  | 2.7881E7 | 2 | 0 | 0 | 0 | 0 | 1 | 0 | 1 | P00760|TRY1\_BOVIN | Carbamidomethylation; Oxidation (M) | C9:Carbamidomethylation:1000.00;M21:Oxidation (M):1000.00;C23:Carbamidomethylation:1000.00 | PEAKS DB |
| C(+57.02)PPGLHFNPVM(+15.99)QIC(+57.02)DLPENAK | 51.62 | 2452.1338 | 21 | 0.4 | 818.3855 | 60.72 | 6 | F6:17890 | Fraction\_6\_23092022.raw |  |  |  |  |  | 2.7398E5 |  | 1 | 0 | 0 | 0 | 0 | 0 | 1 | 0 | TRINITY\_DN2925\_c0\_g1\_i5.p1 | Carbamidomethylation; Oxidation (M) | C1:Carbamidomethylation:1000.00;M11:Oxidation (M):1000.00;C14:Carbamidomethylation:1000.00 | PEAKS DB |
| NFSGIGIIDFER | 51.61 | 1366.6881 | 12 | 0.7 | 684.3518 | 74.24 | 4 | F4:23508 | Fraction\_4\_23092022.raw |  |  |  | 1.4573E5 |  | 1.8187E5 |  | 2 | 0 | 0 | 0 | 1 | 0 | 1 | 0 | TRINITY\_DN2228\_c0\_g2\_i2.p1:TRINITY\_DN2228\_c0\_g2\_i1.p1 |  |  | PEAKS DB |
| NSKIEISELNRVIQR | 51.54 | 1798.0061 | 15 | -0.3 | 600.3425 | 46.84 | 2 | F2:10259 | Fraction\_2\_23092022.raw | 1.7933E5 | 8.9786E5 |  |  |  |  |  | 2 | 1 | 1 | 0 | 0 | 0 | 0 | 0 | P04264|K2C1\_HUMAN |  |  | PEAKS DB |
| ESETFSKFISYLSDQKFSDIRK | 51.53 | 2654.3228 | 22 | 0.1 | 664.5880 | 68.73 | 7 | F7:26341 | Fraction\_7\_23092022.raw |  |  |  |  |  |  | 8.9428E6 | 1 | 0 | 0 | 0 | 0 | 0 | 0 | 1 | TRINITY\_DN13586\_c0\_g2\_i1.p1 |  |  | PEAKS DB |
| TYLISSIPLQGAFNYK | 51.46 | 1813.9614 | 16 | 0.5 | 907.9885 | 78.54 | 1 | F1:24331 | Fraction\_1\_23092022r.raw | 1.0821E5 |  |  |  |  |  |  | 1 | 1 | 0 | 0 | 0 | 0 | 0 | 0 | P12273|PIP\_HUMAN |  |  | PEAKS DB |
| LALDVEIATYRK | 51.28 | 1390.7820 | 12 | 0.0 | 464.6013 | 53.42 | 1 | F1:13083 | Fraction\_1\_23092022r.raw | 4.7232E5 |  |  |  |  |  |  | 1 | 1 | 0 | 0 | 0 | 0 | 0 | 0 | P35908|K22E\_HUMAN:P48668|K2C6C\_HUMAN:P13647|K2C5\_HUMAN |  |  | PEAKS DB |
| LLYENIFNNEKM(+15.99)AAK | 51.21 | 1812.9080 | 15 | 0.3 | 605.3101 | 53.71 | 2 | F2:13370 | Fraction\_2\_23092022.raw |  | 4.5897E5 |  |  |  |  |  | 1 | 0 | 1 | 0 | 0 | 0 | 0 | 0 | TRINITY\_DN2373\_c0\_g1\_i19.p1 | Oxidation (M) | M12:Oxidation (M):1000.00 | PEAKS DB |
| ISISTSGGSFR | 51.21 | 1110.5669 | 11 | -0.5 | 556.2905 | 42.03 | 1 | F1:8027 | Fraction\_1\_23092022r.raw | 7.6343E5 |  |  |  |  |  |  | 1 | 1 | 0 | 0 | 0 | 0 | 0 | 0 | P13647|K2C5\_HUMAN |  |  | PEAKS DB |
| KC(+57.02)EGGLPEPNELR | 51.02 | 1497.7245 | 13 | 0.1 | 500.2488 | 34.64 | 6 | F6:6211 | Fraction\_6\_23092022.raw |  |  |  |  |  | 3.5119E5 |  | 1 | 0 | 0 | 0 | 0 | 0 | 1 | 0 | TRINITY\_DN1697\_c0\_g1\_i11.p1:TRINITY\_DN1697\_c0\_g1\_i18.p1 | Carbamidomethylation | C2:Carbamidomethylation:1000.00 | PEAKS DB |
| QSVEADINGLRR | 50.96 | 1356.7109 | 12 | -0.1 | 453.2442 | 37.62 | 1 | F1:6288 | Fraction\_1\_23092022r.raw | 4.0964E6 |  |  |  |  |  |  | 1 | 1 | 0 | 0 | 0 | 0 | 0 | 0 | P13645|K1C10\_HUMAN |  |  | PEAKS DB |
| EC(+57.02)C(+57.02)LIGVPQSKNPQPVSK | 50.93 | 2040.0132 | 18 | -0.1 | 681.0116 | 38.56 | 3 | F3:6984 | Fraction\_3\_23092022.raw |  |  | 5.6714E4 |  |  |  |  | 1 | 0 | 0 | 1 | 0 | 0 | 0 | 0 |  | Carbamidomethylation | C2:Carbamidomethylation:1000.00;C3:Carbamidomethylation:1000.00 | PEAKS DB |
| M(+15.99)SGEC(+57.02)APNVSVSVSTSHTTISGGGSRGGGGGGYGSGGSSYGSGGGSYGSGGGGGGGR | 50.92 | 4945.0884 | 57 | 0.4 | 1237.2799 | 38.47 | 1 | F1:6582 | Fraction\_1\_23092022r.raw | 6.1732E5 |  |  |  |  |  |  | 1 | 1 | 0 | 0 | 0 | 0 | 0 | 0 | P04264|K2C1\_HUMAN | Oxidation (M); Carbamidomethylation | M1:Oxidation (M):1000.00;C5:Carbamidomethylation:1000.00 | PEAKS DB |
| IKFEMEQNLR | 50.84 | 1306.6703 | 10 | -0.2 | 436.5639 | 43.39 | 1 | F1:8657 | Fraction\_1\_23092022r.raw | 6.8241E5 |  |  |  |  |  |  | 1 | 1 | 0 | 0 | 0 | 0 | 0 | 0 | P35527|K1C9\_HUMAN |  |  | PEAKS DB |
| ELDEIYPK | 50.82 | 1005.5018 | 8 | 0.3 | 503.7584 | 41.97 | 7 | F7:11626 | Fraction\_7\_23092022.raw |  |  |  | 1.6356E6 | 5.2505E6 | 7.199E5 | 4.2396E5 | 4 | 0 | 0 | 0 | 1 | 1 | 1 | 1 | TRINITY\_DN2228\_c0\_g4\_i1.p1:Q5D7H4|HUGAB\_VESVU |  |  | PEAKS DB |
| RSLVFDNIIEGYSK | 50.79 | 1639.8569 | 14 | 1.1 | 547.6268 | 60.43 | 6 | F6:17768 | Fraction\_6\_23092022.raw |  |  |  |  |  | 1.4032E5 |  | 1 | 0 | 0 | 0 | 0 | 0 | 1 | 0 | TRINITY\_DN33395\_c0\_g1\_i1.p1 |  |  | PEAKS DB |
| VVAGVANALAHKYH | 50.77 | 1448.7888 | 14 | -0.9 | 483.9365 | 34.60 | 5 | F5:5741 | Fraction\_5\_23092022.raw |  |  |  |  | 1.068E5 |  |  | 1 | 0 | 0 | 0 | 0 | 1 | 0 | 0 | P02075|HBB\_SHEEP:P18982|HBB\_AILFU:B3EWD0|HBB\_OTOBE:P68044|HBB\_MUSPF:P20855|HBB\_CTEGU:P02087|HBB\_DASNO:P19886|HBD\_COLPO:P61773|HBD\_GORGO:P68232|HBB\_ATEGE:P60524|HBB\_CANLF:P60525|HBB\_CANLA:P02052|HBB\_TUPGL |  |  | PEAKS DB |
| SQEC(+57.02)SQRIC(+57.02)ETDANYVQIIHTSSFKGTLR | 50.71 | 3427.6248 | 29 | -1.2 | 686.5314 | 50.38 | 6 | F6:13200 | Fraction\_6\_23092022.raw |  |  |  |  |  | 2.6279E5 |  | 1 | 0 | 0 | 0 | 0 | 0 | 1 | 0 | TRINITY\_DN2326\_c0\_g1\_i13.p1 | Carbamidomethylation | C4:Carbamidomethylation:1000.00;C9:Carbamidomethylation:1000.00 | PEAKS DB |
| SM(+15.99)IETEASNRFEKSAR | 50.70 | 1870.8843 | 16 | -0.2 | 468.7283 | 26.49 | 6 | F6:3955 | Fraction\_6\_23092022.raw |  |  |  |  |  | 4.2525E5 |  | 1 | 0 | 0 | 0 | 0 | 0 | 1 | 0 | TRINITY\_DN2228\_c0\_g4\_i1.p1 | Oxidation (M) | M2:Oxidation (M):1000.00 | PEAKS DB |
| NYVTITEDEEDKLK | 50.70 | 1695.8203 | 14 | 0.8 | 566.2812 | 44.11 | 6 | F6:10402 | Fraction\_6\_23092022.raw |  |  |  |  |  | 9.581E4 |  | 1 | 0 | 0 | 0 | 0 | 0 | 1 | 0 | TRINITY\_DN4312\_c0\_g1\_i15.p1:TRINITY\_DN4312\_c0\_g1\_i7.p1:TRINITY\_DN4312\_c0\_g1\_i9.p1 |  |  | PEAKS DB |
| GIDGFRIDAVPHLFER | 50.68 | 1840.9584 | 16 | 0.0 | 614.6601 | 68.71 | 2 | F2:20128 | Fraction\_2\_23092022.raw |  | 2.1436E5 |  |  |  |  |  | 1 | 0 | 1 | 0 | 0 | 0 | 0 | 0 | TRINITY\_DN1342\_c0\_g1\_i29.p1:TRINITY\_DN1342\_c0\_g1\_i23.p1:TRINITY\_DN1342\_c0\_g1\_i54.p1:TRINITY\_DN1342\_c0\_g1\_i9.p1:TRINITY\_DN1342\_c0\_g1\_i34.p1 |  |  | PEAKS DB |
| TYPKTGSFYVPVESKAPYC(+57.02)NNKGK | 50.63 | 2734.3425 | 24 | 0.1 | 547.8759 | 38.42 | 4 | F4:7511 | Fraction\_4\_23092022.raw |  |  |  | 5.1464E5 |  |  |  | 1 | 0 | 0 | 0 | 1 | 0 | 0 | 0 | A0A0M3KKW3|PA1\_VESBA | Carbamidomethylation | C19:Carbamidomethylation:1000.00 | PEAKS DB |
| SDLEM(+15.99)QYETLQEELMALKK | 50.62 | 2314.1072 | 19 | 0.8 | 772.3770 | 78.52 | 1 | F1:24324 | Fraction\_1\_23092022r.raw | 1.156E5 |  |  |  |  |  |  | 1 | 1 | 0 | 0 | 0 | 0 | 0 | 0 | P35527|K1C9\_HUMAN | Oxidation (M) | M5:Oxidation (M):43.70 | PEAKS DB |
| GGKPADKQNYISLLK | 50.60 | 1630.9042 | 15 | 0.6 | 544.6423 | 38.16 | 6 | F6:7749 | Fraction\_6\_23092022.raw |  |  |  | 2.3055E5 |  | 1.5484E5 |  | 2 | 0 | 0 | 0 | 1 | 0 | 1 | 0 | TRINITY\_DN36831\_c0\_g1\_i1.p1 |  |  | PEAKS DB |
| MSVEADINGLRR | 50.41 | 1359.6929 | 12 | -0.4 | 454.2381 | 43.39 | 1 | F1:8670 | Fraction\_1\_23092022r.raw | 7.1864E4 |  |  |  |  |  |  | 1 | 1 | 0 | 0 | 0 | 0 | 0 | 0 | P02533|K1C14\_HUMAN:Q6IFU8|K1C17\_RAT:Q9QWL7|K1C17\_MOUSE |  |  | PEAKS DB |
| KLVVIIDPHIKR | 50.41 | 1429.9132 | 12 | 0.5 | 477.6452 | 37.88 | 1 | F1:6401 | Fraction\_1\_23092022r.raw | 6.2527E5 |  |  |  |  |  |  | 1 | 1 | 0 | 0 | 0 | 0 | 0 | 0 | TRINITY\_DN1709\_c0\_g1\_i4.p1:TRINITY\_DN1709\_c0\_g1\_i5.p1:TRINITY\_DN1709\_c0\_g1\_i6.p1:TRINITY\_DN1709\_c0\_g1\_i8.p1 |  |  | PEAKS DB |
| VILDLVPNHTSDK | 50.32 | 1449.7827 | 13 | -0.1 | 484.2681 | 48.20 | 6 | F6:12267 | Fraction\_6\_23092022.raw |  |  |  |  |  | 7.1972E4 |  | 1 | 0 | 0 | 0 | 0 | 0 | 1 | 0 | TRINITY\_DN1342\_c0\_g1\_i34.p1 |  |  | PEAKS DB |
| GLFIIDDKQNLR | 50.20 | 1430.7881 | 12 | 0.3 | 477.9368 | 53.71 | 6 | F6:14729 | Fraction\_6\_23092022.raw |  |  |  |  |  | 7.7847E4 |  | 1 | 0 | 0 | 0 | 0 | 0 | 1 | 0 | TRINITY\_DN1450\_c3\_g2\_i1.p1:Q9V3P0|PRDX1\_DROME |  |  | PEAKS DB |
| YENAEEIDPEGEISYERPPSYLPGSQVNALNK | 50.20 | 3607.6953 | 32 | -0.8 | 1203.5714 | 63.16 | 1 | F1:17499 | Fraction\_1\_23092022r.raw | 5.21E5 |  |  |  |  |  |  | 1 | 1 | 0 | 0 | 0 | 0 | 0 | 0 | TRINITY\_DN126\_c0\_g1\_i17.p1:TRINITY\_DN126\_c0\_g1\_i12.p1:TRINITY\_DN126\_c0\_g1\_i25.p1 |  |  | PEAKS DB |
| LFM(+15.99)EETLKLAK | 49.81 | 1337.7264 | 11 | -0.1 | 446.9160 | 45.99 | 4 | F4:10849 | Fraction\_4\_23092022.raw |  |  |  | 1.9873E6 |  |  |  | 1 | 0 | 0 | 0 | 1 | 0 | 0 | 0 | TRINITY\_DN2228\_c0\_g2\_i2.p1:TRINITY\_DN2228\_c0\_g2\_i1.p1 | Oxidation (M) | M3:Oxidation (M):1000.00 | PEAKS DB |
| LINDIGLIR | 49.71 | 1025.6233 | 9 | -0.5 | 513.8187 | 58.17 | 6 | F6:16716 | Fraction\_6\_23092022.raw |  |  |  |  |  | 1.1555E6 | 2.548E6 | 2 | 0 | 0 | 0 | 0 | 0 | 1 | 1 | P00768|CTR2\_VESOR:P00769|CTR2\_VESCR |  |  | PEAKS DB |
| SGYRSGGGFSSGSAGIINYQRRTTSSSTR | 49.67 | 2996.4448 | 29 | 1.7 | 600.2972 | 32.11 | 7 | F7:7215 | Fraction\_7\_23092022.raw |  |  |  |  |  |  | 2.8886E4 | 1 | 0 | 0 | 0 | 0 | 0 | 0 | 1 | P04264|K2C1\_HUMAN |  |  | PEAKS DB |
| LLNDEDPVVVTK | 49.66 | 1340.7188 | 12 | -0.1 | 671.3666 | 47.10 | 1 | F1:10288 | Fraction\_1\_23092022r.raw | 4.9891E4 |  |  |  |  |  |  | 1 | 1 | 0 | 0 | 0 | 0 | 0 | 0 | Q8SPJ1|PLAK\_BOVIN:P14923|PLAK\_HUMAN:Q8WNW3|PLAK\_PIG:Q02257|PLAK\_MOUSE |  |  | PEAKS DB |
| LRSEIDNVKKQISNLQQSISDAEQR | 49.60 | 2898.5159 | 25 | 0.1 | 725.6363 | 62.20 | 1 | F1:17035 | Fraction\_1\_23092022r.raw | 4.1147E5 |  |  |  |  |  |  | 1 | 1 | 0 | 0 | 0 | 0 | 0 | 0 | P04264|K2C1\_HUMAN |  |  | PEAKS DB |
| SLEEAEAYSRSQLEEQAAR | 49.35 | 2166.0188 | 19 | 0.1 | 723.0136 | 46.13 | 3 | F3:10368 | Fraction\_3\_23092022.raw |  |  | 6.8861E4 |  |  |  |  | 1 | 0 | 0 | 1 | 0 | 0 | 0 | 0 | Q6KB66|K2C80\_HUMAN |  |  | PEAKS DB |
| TLLDIDNTRM(+15.99)TLDDFR | 49.27 | 1953.9465 | 16 | 0.6 | 652.3232 | 64.62 | 1 | F1:18095 | Fraction\_1\_23092022r.raw | 1.7001E5 |  |  |  |  |  |  | 1 | 1 | 0 | 0 | 0 | 0 | 0 | 0 | P35527|K1C9\_HUMAN | Oxidation (M) | M10:Oxidation (M):1000.00 | PEAKS DB |
| VVNRYLVVEGKHEER | 49.25 | 1825.9799 | 15 | -0.4 | 457.5021 | 23.62 | 7 | F7:3749 | Fraction\_7\_23092022.raw |  |  |  |  |  |  | 6.1283E4 | 1 | 0 | 0 | 0 | 0 | 0 | 0 | 1 | TRINITY\_DN5303\_c0\_g1\_i4.p1 |  |  | PEAKS DB |
| GIPTLLLFK | 48.88 | 1000.6321 | 9 | -0.3 | 501.3232 | 78.13 | 4 | F4:25248 | Fraction\_4\_23092022.raw |  | 5.0567E6 |  | 1.2317E6 |  |  |  | 2 | 0 | 1 | 0 | 1 | 0 | 0 | 0 | P0AA29|THIO\_SALTI:P0AA26|THIO\_ECOL6:P0AA27|THIO\_ECO57:P0AA30|THIO\_SHIFL:P0AA25|THIO\_ECOLI:P0AA28|THIO\_SALTY |  |  | PEAKS DB |
| AVM(+15.99)DDFAAFVEK | 48.87 | 1357.6223 | 12 | 0.3 | 679.8186 | 62.27 | 1 | F1:17060 | Fraction\_1\_23092022r.raw | 9.6033E4 |  |  |  |  |  |  | 1 | 1 | 0 | 0 | 0 | 0 | 0 | 0 | P02768|ALBU\_HUMAN | Oxidation (M) | M3:Oxidation (M):1000.00 | PEAKS DB |
| QISNLQQSISDAEQRGENALK | 48.85 | 2328.1670 | 21 | -0.4 | 777.0626 | 60.56 | 1 | F1:16275 | Fraction\_1\_23092022r.raw | 2.1124E6 |  |  |  |  |  |  | 1 | 1 | 0 | 0 | 0 | 0 | 0 | 0 | P04264|K2C1\_HUMAN |  |  | PEAKS DB |
| LAADDFRTKYETELNLR | 48.77 | 2054.0432 | 17 | 0.2 | 685.6885 | 52.79 | 1 | F1:12803 | Fraction\_1\_23092022r.raw | 3.1521E5 |  |  |  |  |  |  | 1 | 1 | 0 | 0 | 0 | 0 | 0 | 0 | P02533|K1C14\_HUMAN |  |  | PEAKS DB |
| DGLYDFYQVR | 48.72 | 1274.5931 | 10 | 0.8 | 638.3044 | 67.94 | 1 | F1:19571 | Fraction\_1\_23092022r.raw | 1.8168E5 |  |  |  |  |  |  | 1 | 1 | 0 | 0 | 0 | 0 | 0 | 0 | TRINITY\_DN126\_c0\_g1\_i17.p1:TRINITY\_DN126\_c0\_g1\_i12.p1:TRINITY\_DN126\_c0\_g1\_i25.p1 |  |  | PEAKS DB |
| FLLIGYDFQKGFR | 48.70 | 1602.8558 | 13 | 0.0 | 535.2925 | 73.15 | 7 | F7:28562 | Fraction\_7\_23092022.raw |  |  |  |  |  |  | 3.0007E6 | 1 | 0 | 0 | 0 | 0 | 0 | 0 | 1 | TRINITY\_DN3450\_c0\_g1\_i51.p1 |  |  | PEAKS DB |
| IIEAWIAKTPK | 48.69 | 1268.7493 | 11 | 1.0 | 423.9241 | 46.93 | 2 | F2:10307 | Fraction\_2\_23092022.raw |  | 3.7018E5 |  |  |  |  |  | 1 | 0 | 1 | 0 | 0 | 0 | 0 | 0 | TRINITY\_DN1342\_c0\_g1\_i29.p1 |  |  | PEAKS DB |
| QNLEPLFEQYINNLRR | 48.69 | 2046.0646 | 16 | 0.4 | 683.0291 | 77.65 | 1 | F1:23976 | Fraction\_1\_23092022r.raw | 2.1213E6 |  |  |  |  |  |  | 1 | 1 | 0 | 0 | 0 | 0 | 0 | 0 | P48668|K2C6C\_HUMAN:P13647|K2C5\_HUMAN |  |  | PEAKS DB |
| EGC(+57.02)DSAGPHFNPYM(+15.99)VNHGAPNDPLR | 48.66 | 2767.1868 | 25 | 0.0 | 692.8040 | 43.53 | 6 | F6:10139 | Fraction\_6\_23092022.raw |  |  |  |  |  | 2.0581E5 |  | 1 | 0 | 0 | 0 | 0 | 0 | 1 | 0 | TRINITY\_DN16204\_c0\_g1\_i2.p1 | Carbamidomethylation; Oxidation (M) | C3:Carbamidomethylation:1000.00;M14:Oxidation (M):1000.00 | PEAKS DB |
| LSTNSDVINVSRIVK | 48.64 | 1643.9207 | 15 | -0.7 | 548.9805 | 49.99 | 1 | F1:11545 | Fraction\_1\_23092022r.raw | 6.156E5 |  |  |  |  |  |  | 1 | 1 | 0 | 0 | 0 | 0 | 0 | 0 | TRINITY\_DN4276\_c0\_g1\_i1.p1 |  |  | PEAKS DB |
| NEDVIHTVIRLSTNSDVINVSRIVKDSDAK | 48.51 | 3336.7637 | 30 | 0.5 | 668.3604 | 70.89 | 1 | F1:20918 | Fraction\_1\_23092022r.raw | 1.4819E5 |  |  |  |  |  |  | 1 | 1 | 0 | 0 | 0 | 0 | 0 | 0 | TRINITY\_DN4276\_c0\_g1\_i1.p1 |  |  | PEAKS DB |
| IAQVWASQC(+57.02)KYGHDDC(+57.02)R | 48.48 | 2092.9207 | 17 | -0.1 | 524.2374 | 31.26 | 6 | F6:5037 | Fraction\_6\_23092022.raw |  |  |  |  |  | 1.5963E5 |  | 1 | 0 | 0 | 0 | 0 | 0 | 1 | 0 | TRINITY\_DN6370\_c0\_g1\_i1.p1 | Carbamidomethylation | C9:Carbamidomethylation:1000.00;C16:Carbamidomethylation:1000.00 | PEAKS DB |
| EKFDQHGYILSAAVSAAATSAR | 48.44 | 2292.1499 | 22 | -0.3 | 574.0446 | 55.54 | 4 | F4:15128 | Fraction\_4\_23092022.raw |  |  |  | 1.7934E5 |  |  |  | 1 | 0 | 0 | 0 | 1 | 0 | 0 | 0 | TRINITY\_DN36831\_c0\_g1\_i1.p1 |  |  | PEAKS DB |
| WFIGPDGK | 48.31 | 918.4599 | 8 | -0.5 | 460.2370 | 55.08 | 2 | F2:14002 | Fraction\_2\_23092022.raw |  | 1.8407E5 |  |  |  |  |  | 1 | 0 | 1 | 0 | 0 | 0 | 0 | 0 | TRINITY\_DN10256\_c0\_g10\_i1.p1:TRINITY\_DN10256\_c0\_g5\_i4.p1 |  |  | PEAKS DB |
| C(+57.02)PPGVSFK | 48.20 | 890.4320 | 8 | -1.6 | 891.4379 | 66.11 | 7 | F7:25071 | Fraction\_7\_23092022.raw |  |  |  |  |  |  | 5.436E5 | 1 | 0 | 0 | 0 | 0 | 0 | 0 | 1 | Q25C93|FEZF1\_DANRE | Carbamidomethylation | C1:Carbamidomethylation:1000.00 | PEAKS DB |
| FKVEESYDLKDTLR | 48.14 | 1741.8885 | 14 | 0.1 | 581.6368 | 46.83 | 1 | F1:10159 | Fraction\_1\_23092022r.raw | 2.3427E5 |  |  |  |  |  |  | 1 | 1 | 0 | 0 | 0 | 0 | 0 | 0 | P29508|SPB3\_HUMAN |  |  | PEAKS DB |
| QITVNDLPVGR | 48.07 | 1210.6670 | 11 | -0.1 | 606.3407 | 50.63 | 1 | F1:11862 | Fraction\_1\_23092022r.raw | 9.9582E4 |  |  |  |  |  |  | 1 | 1 | 0 | 0 | 0 | 0 | 0 | 0 | TRINITY\_DN1450\_c3\_g2\_i1.p1:Q9V3P0|PRDX1\_DROME |  |  | PEAKS DB |
| TGSFYVPVERK | 47.95 | 1281.6716 | 11 | 0.3 | 641.8433 | 37.45 | 4 | F4:7026 | Fraction\_4\_23092022.raw |  |  |  | 1.4327E5 |  |  |  | 1 | 0 | 0 | 0 | 1 | 0 | 0 | 0 | TRINITY\_DN2326\_c0\_g1\_i12.p1 |  |  | PEAKS DB |
| TVDQYYFGSQSKIQK | 47.90 | 1790.8839 | 15 | 0.3 | 597.9688 | 39.55 | 2 | F2:7028 | Fraction\_2\_23092022.raw |  | 8.7729E4 |  |  |  |  |  | 1 | 0 | 1 | 0 | 0 | 0 | 0 | 0 | TRINITY\_DN2373\_c0\_g1\_i19.p1 |  |  | PEAKS DB |
| FSVFTDR | 47.84 | 870.4235 | 7 | 0.0 | 436.2191 | 48.92 | 3 | F3:11591 | Fraction\_3\_23092022.raw |  |  | 6.1699E5 |  |  |  |  | 1 | 0 | 0 | 1 | 0 | 0 | 0 | 0 | TRINITY\_DN2373\_c0\_g1\_i19.p1 |  |  | PEAKS DB |
| C(+57.02)LKAPILSDSSC(+57.02)K | 47.72 | 1477.7268 | 13 | 0.6 | 493.5832 | 36.92 | 1 | F1:5993 | Fraction\_1\_23092022r.raw | 2.7394E4 |  |  |  |  |  |  | 1 | 1 | 0 | 0 | 0 | 0 | 0 | 0 | P00760|TRY1\_BOVIN | Carbamidomethylation | C1:Carbamidomethylation:1000.00;C12:Carbamidomethylation:1000.00 | PEAKS DB |
| FVSTTYSGVTR | 47.71 | 1216.6088 | 11 | 0.3 | 609.3118 | 35.32 | 7 | F7:8656 | Fraction\_7\_23092022.raw |  |  |  |  |  |  | 5.0361E4 | 1 | 0 | 0 | 0 | 0 | 0 | 0 | 1 | P04264|K2C1\_HUMAN |  |  | PEAKS DB |
| STSSFSC(+57.02)LSR | 47.66 | 1130.5026 | 10 | 0.0 | 566.2585 | 33.34 | 7 | F7:7761 | Fraction\_7\_23092022.raw |  |  |  |  |  |  | 1.2519E5 | 1 | 0 | 0 | 0 | 0 | 0 | 0 | 1 | P35908|K22E\_HUMAN | Carbamidomethylation | C7:Carbamidomethylation:1000.00 | PEAKS DB |
| KLQEM(+15.99)FPWIDSKR | 47.47 | 1692.8657 | 13 | 0.4 | 565.2961 | 47.90 | 6 | F6:12164 | Fraction\_6\_23092022.raw |  |  |  |  |  | 4.657E5 |  | 1 | 0 | 0 | 0 | 0 | 0 | 1 | 0 | TRINITY\_DN3450\_c0\_g1\_i51.p1 | Oxidation (M) | M5:Oxidation (M):1000.00 | PEAKS DB |
| AILQNKKIIAVDQDPLGIQGRR | 47.47 | 2445.4180 | 22 | 0.4 | 612.3620 | 46.39 | 2 | F2:10087 | Fraction\_2\_23092022.raw |  | 1.0483E5 |  |  |  |  |  | 1 | 0 | 1 | 0 | 0 | 0 | 0 | 0 | TRINITY\_DN2611\_c0\_g1\_i12.p1:TRINITY\_DN2611\_c0\_g1\_i23.p1:TRINITY\_DN2611\_c0\_g1\_i19.p1:TRINITY\_DN2611\_c0\_g1\_i9.p1 |  |  | PEAKS DB |
| INKEVSIDLVRK | 47.43 | 1412.8351 | 12 | 0.2 | 471.9524 | 32.98 | 7 | F7:7590 | Fraction\_7\_23092022.raw |  |  |  |  |  |  | 5.519E5 | 1 | 0 | 0 | 0 | 0 | 0 | 0 | 1 | TRINITY\_DN2228\_c0\_g4\_i1.p1 |  |  | PEAKS DB |
| SISISPTNVQPR | 47.43 | 1297.6990 | 12 | 0.5 | 649.8571 | 42.23 | 2 | F2:8246 | Fraction\_2\_23092022.raw |  | 4.7244E6 |  |  |  |  |  | 1 | 0 | 1 | 0 | 0 | 0 | 0 | 0 | TRINITY\_DN4582\_c0\_g1\_i1.p1:TRINITY\_DN4582\_c0\_g1\_i2.p1 |  |  | PEAKS DB |
| FDIKYNSK | 47.40 | 1013.5182 | 8 | -0.5 | 507.7661 | 30.18 | 7 | F7:6337 | Fraction\_7\_23092022.raw |  |  |  |  |  |  | 2.0292E6 | 1 | 0 | 0 | 0 | 0 | 0 | 0 | 1 | TRINITY\_DN2228\_c0\_g4\_i1.p1 |  |  | PEAKS DB |
| TLAVASSPIAPEQHLK | 47.35 | 1660.9148 | 16 | -0.3 | 554.6454 | 42.91 | 2 | F2:8489 | Fraction\_2\_23092022.raw |  | 8.6069E4 |  |  |  |  |  | 1 | 0 | 1 | 0 | 0 | 0 | 0 | 0 | TRINITY\_DN1455\_c0\_g1\_i1.p1 |  |  | PEAKS DB |
| LKYENEVALR | 47.21 | 1233.6716 | 10 | -1.1 | 412.2307 | 33.64 | 7 | F7:7885 | Fraction\_7\_23092022.raw |  |  |  |  |  |  | 1.2434E5 | 1 | 0 | 0 | 0 | 0 | 0 | 0 | 1 | P13645|K1C10\_HUMAN |  |  | PEAKS DB |
| C(+57.02)TRNQC(+57.02)VC(+57.02)VGLNAK | 47.14 | 1678.7701 | 14 | -0.5 | 560.5970 | 26.19 | 7 | F7:4646 | Fraction\_7\_23092022.raw |  |  |  |  |  |  | 2.1545E5 | 1 | 0 | 0 | 0 | 0 | 0 | 0 | 1 |  | Carbamidomethylation | C1:Carbamidomethylation:1000.00;C6:Carbamidomethylation:1000.00;C8:Carbamidomethylation:1000.00 | PEAKS DB |
| total 735 peptides |
| --- |
