# Supplementary figures and images for: Stings on wings: Proteotranscriptomic and biochemical profiling of the lesser banded hornet (Vespa affinis) venom (part 2 of 2)
Source: Front Mol Biosci. 2022 Dec 19;9:1066793. doi: 10.3389/fmolb.2022.1066793 (PMC9806352; doi:10.3389/fmolb.2022.1066793)

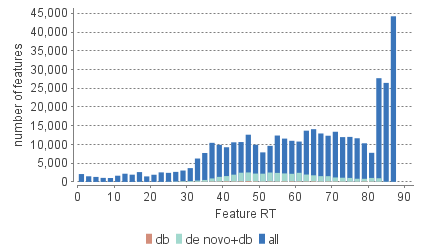

Supplement: Supplementary file 3 [file DataSheet2.ZIP › HTML/img/FeatureRtHistogram8784400061360296103.png]

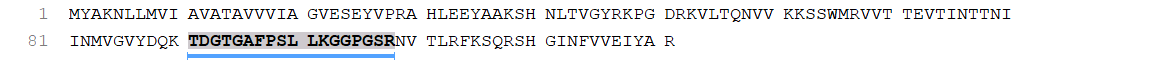

Supplement: Supplementary file 3 [file DataSheet2.ZIP › HTML/img/cov_1385.png]

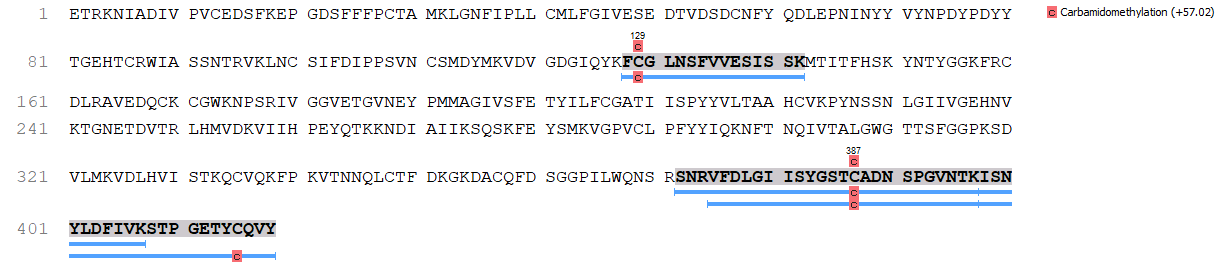

Supplement: Supplementary file 3 [file DataSheet2.ZIP › HTML/img/cov_213.png]

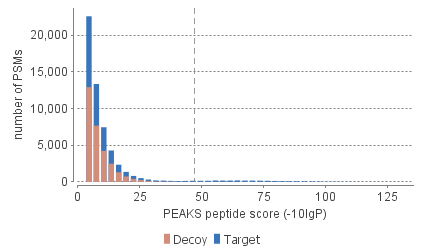

Supplement: Supplementary file 3 [file DataSheet2.ZIP › HTML/img/ScoreHistogram4281511531758126763.png]

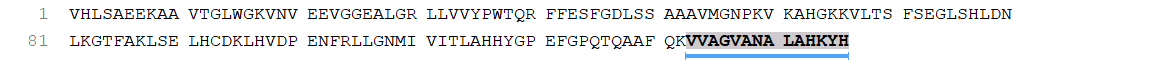

Supplement: Supplementary file 3 [file DataSheet2.ZIP › HTML/img/cov_763.png]

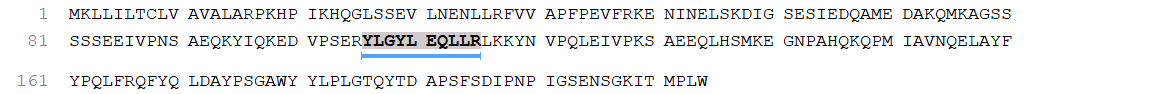

Supplement: Supplementary file 3 [file DataSheet2.ZIP › HTML/img/cov_2289.png]

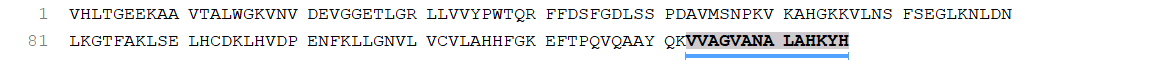

Supplement: Supplementary file 3 [file DataSheet2.ZIP › HTML/img/cov_762.png]

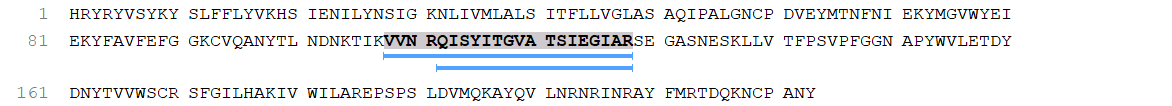

Supplement: Supplementary file 3 [file DataSheet2.ZIP › HTML/img/cov_2262.png]

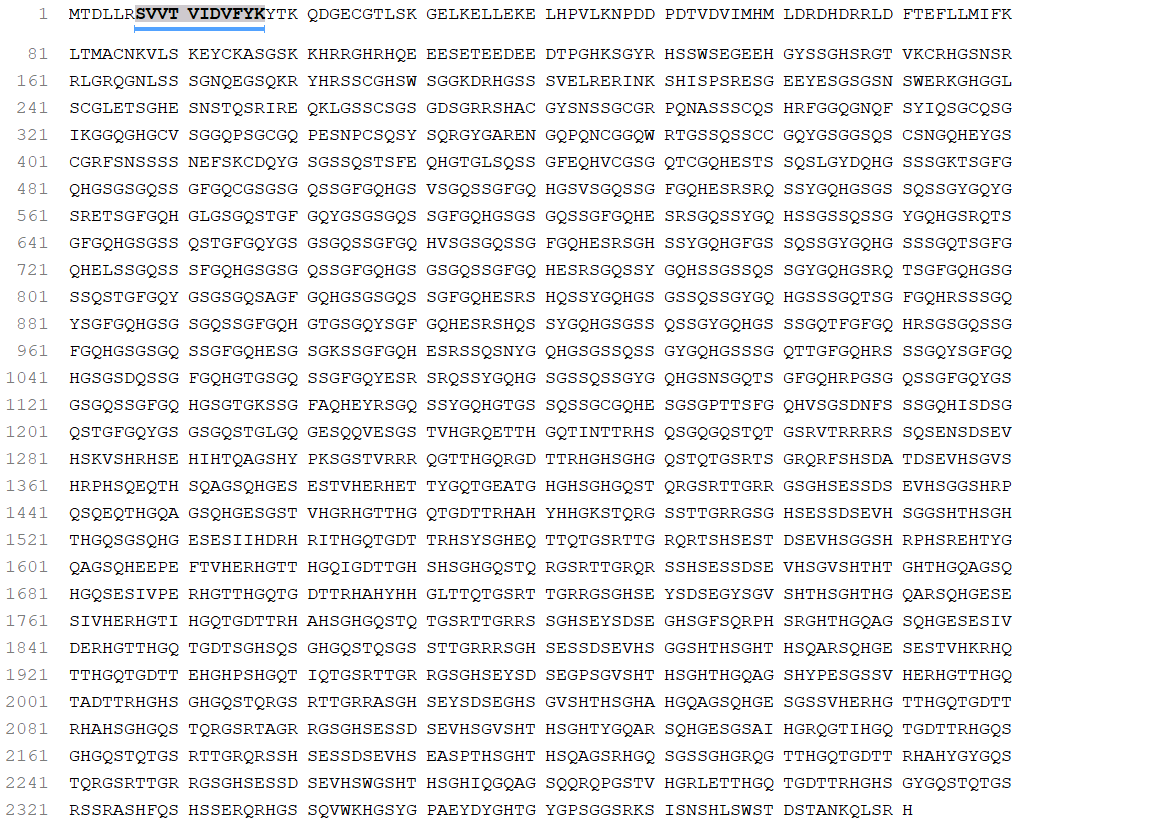

Supplement: Supplementary file 3 [file DataSheet2.ZIP › HTML/img/cov_428.png]

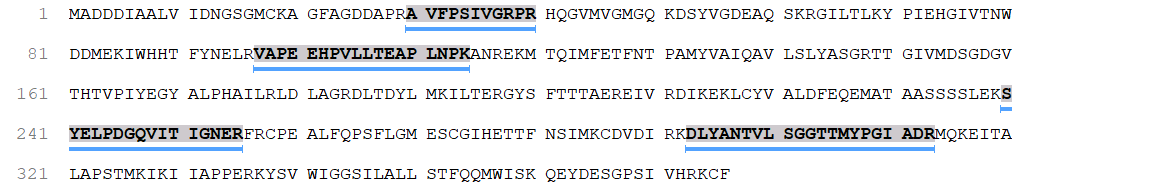

Supplement: Supplementary file 3 [file DataSheet2.ZIP › HTML/img/cov_158.png]

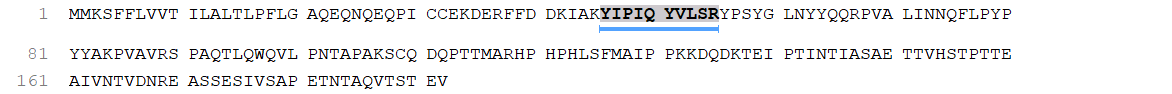

Supplement: Supplementary file 3 [file DataSheet2.ZIP › HTML/img/cov_2316.png]

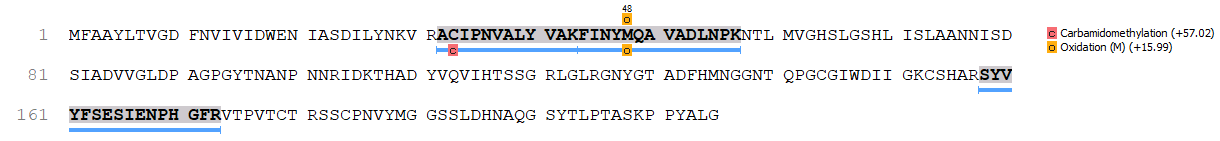

Supplement: Supplementary file 3 [file DataSheet2.ZIP › HTML/img/cov_182.png]

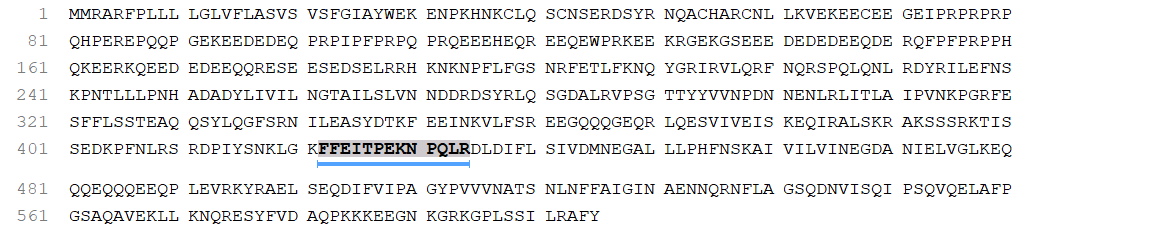

Supplement: Supplementary file 3 [file DataSheet2.ZIP › HTML/img/cov_814.png]

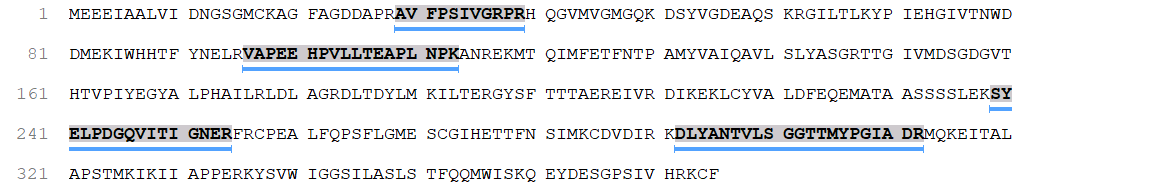

Supplement: Supplementary file 3 [file DataSheet2.ZIP › HTML/img/cov_155.png]

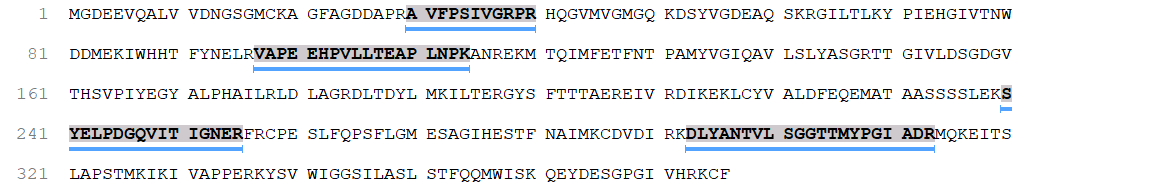

Supplement: Supplementary file 3 [file DataSheet2.ZIP › HTML/img/cov_169.png]

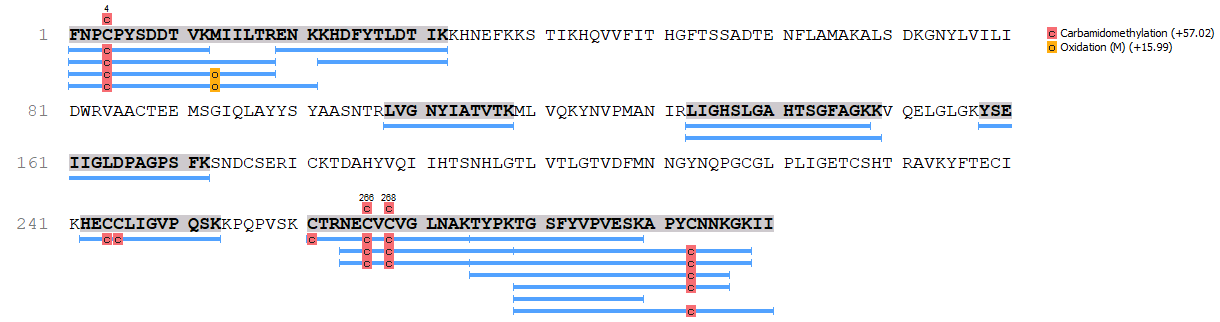

Supplement: Supplementary file 3 [file DataSheet2.ZIP › HTML/img/cov_39.png]

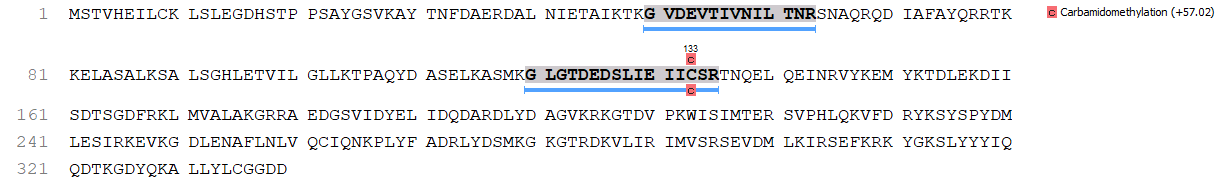

Supplement: Supplementary file 3 [file DataSheet2.ZIP › HTML/img/cov_357.png]

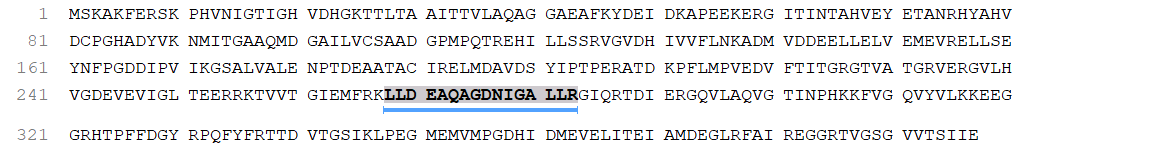

Supplement: Supplementary file 3 [file DataSheet2.ZIP › HTML/img/cov_431.png]

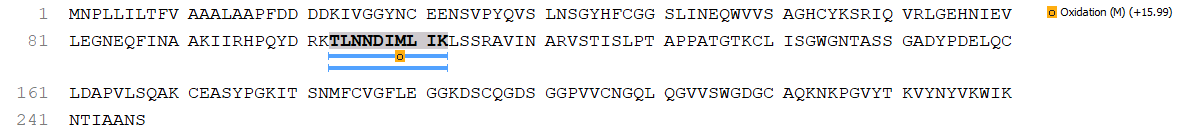

Supplement: Supplementary file 3 [file DataSheet2.ZIP › HTML/img/cov_343.png]

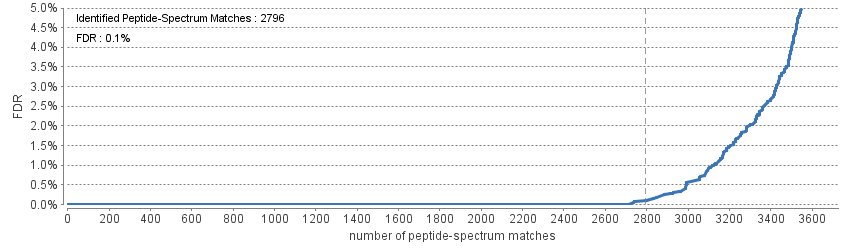

Supplement: Supplementary file 3 [file DataSheet2.ZIP › HTML/img/FDRFigure243534934204043203.png]

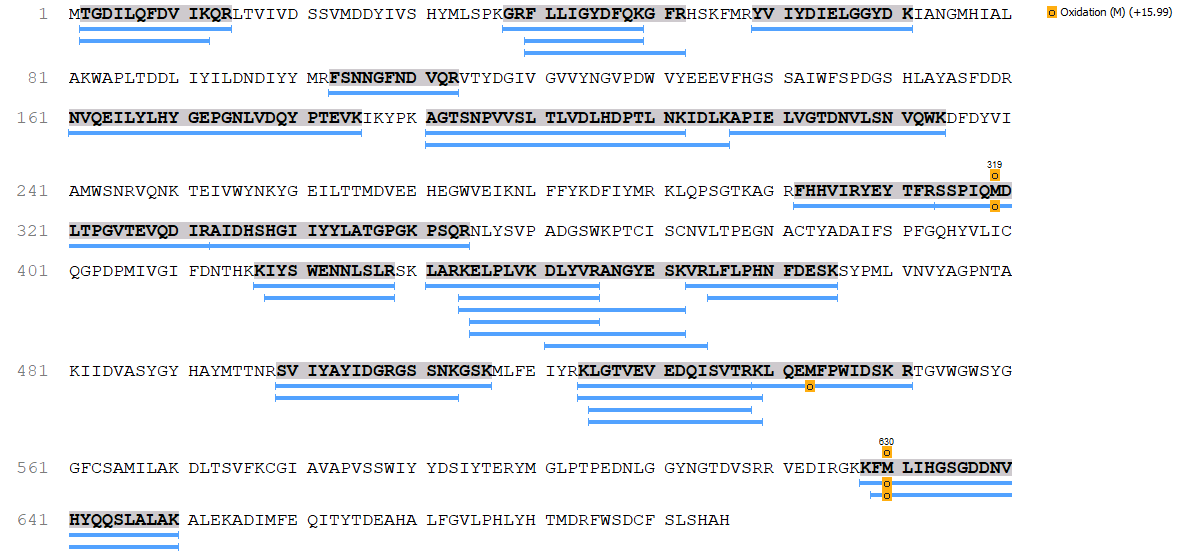

Supplement: Supplementary file 3 [file DataSheet2.ZIP › HTML/img/cov_6.png]

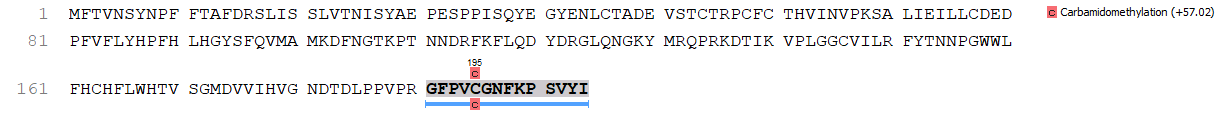

Supplement: Supplementary file 3 [file DataSheet2.ZIP › HTML/img/cov_2284.png]

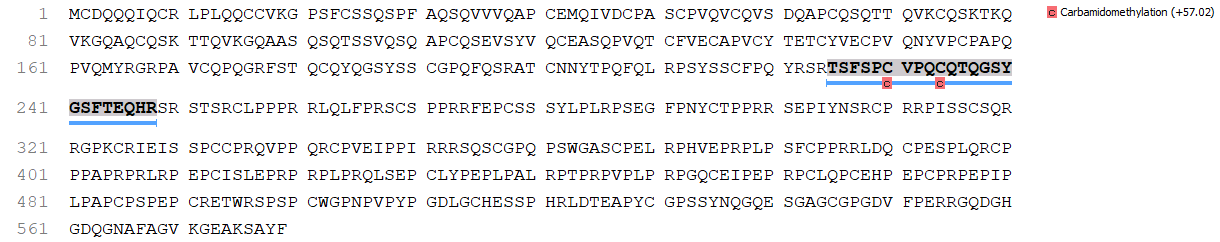

Supplement: Supplementary file 3 [file DataSheet2.ZIP › HTML/img/cov_2291.png]

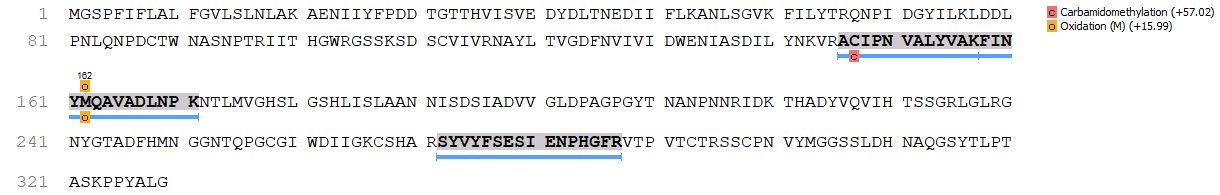

Supplement: Supplementary file 3 [file DataSheet2.ZIP › HTML/img/cov_183.png]

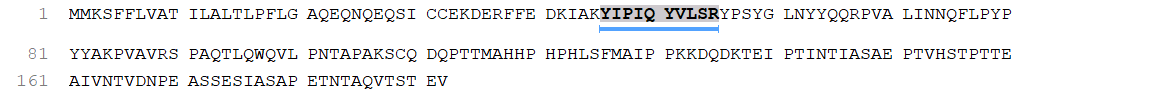

Supplement: Supplementary file 3 [file DataSheet2.ZIP › HTML/img/cov_2318.png]

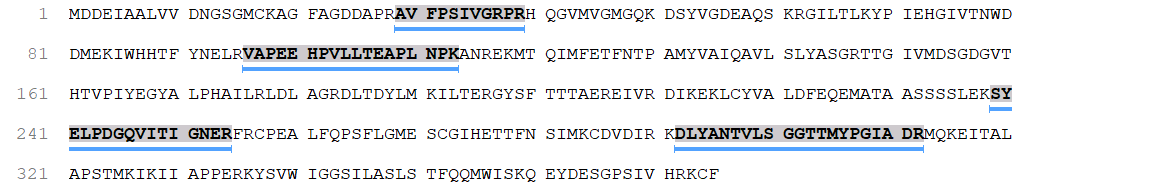

Supplement: Supplementary file 3 [file DataSheet2.ZIP › HTML/img/cov_156.png]

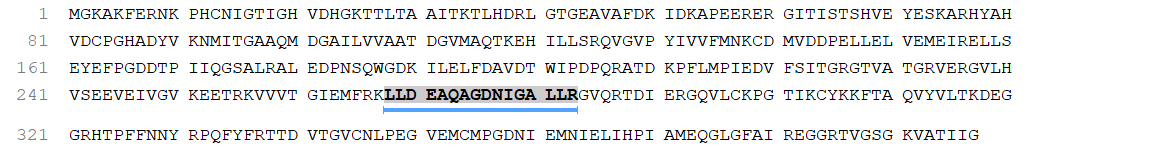

Supplement: Supplementary file 3 [file DataSheet2.ZIP › HTML/img/cov_630.png]

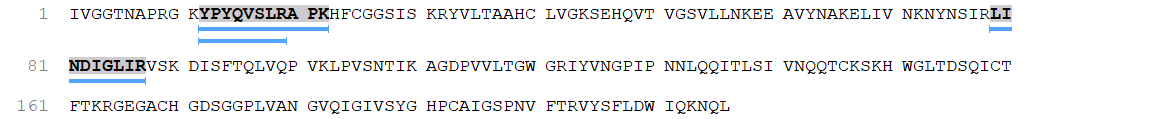

Supplement: Supplementary file 3 [file DataSheet2.ZIP › HTML/img/cov_383.png]

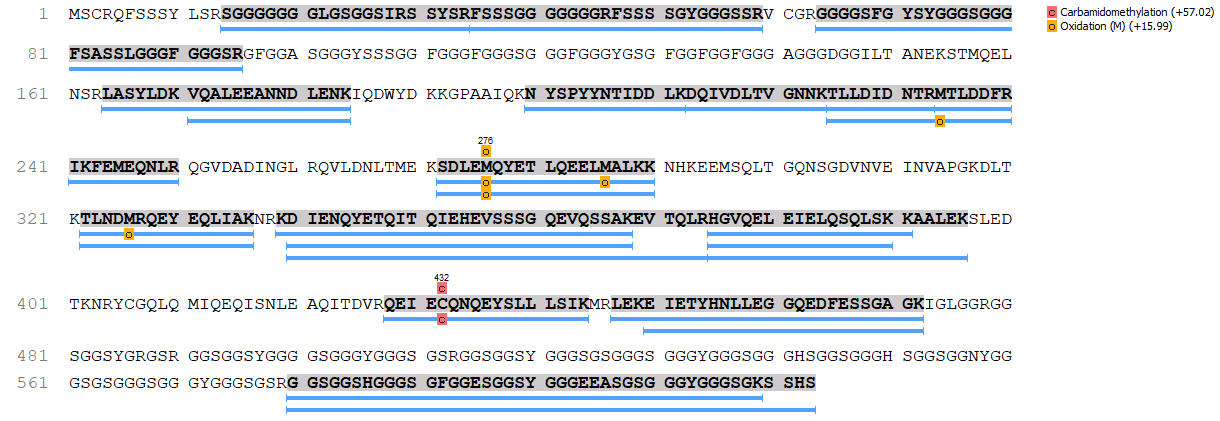

Supplement: Supplementary file 3 [file DataSheet2.ZIP › HTML/img/cov_12.png]

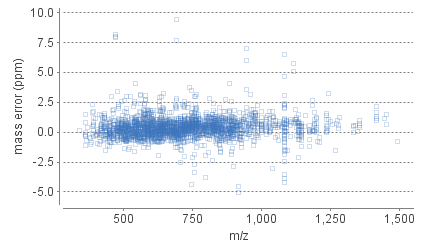

Supplement: Supplementary file 3 [file DataSheet2.ZIP › HTML/img/ErrorPlotFigure1654546958493341374.png]

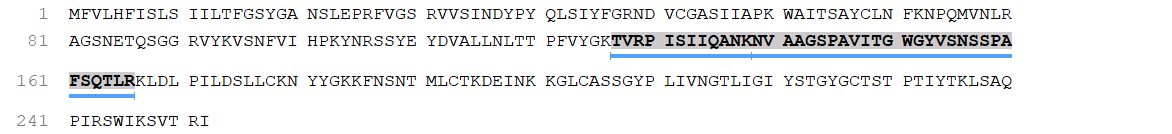

Supplement: Supplementary file 3 [file DataSheet2.ZIP › HTML/img/cov_368.png]

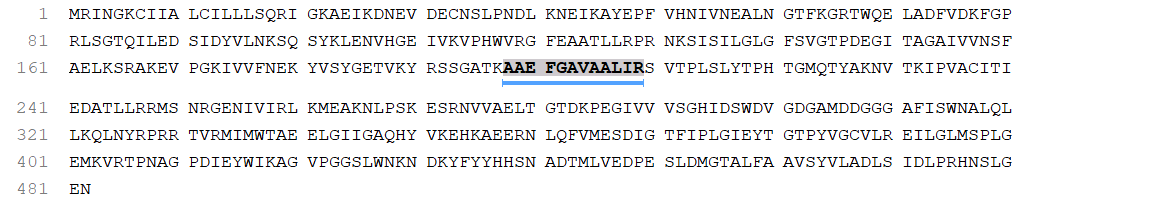

Supplement: Supplementary file 3 [file DataSheet2.ZIP › HTML/img/cov_546.png]

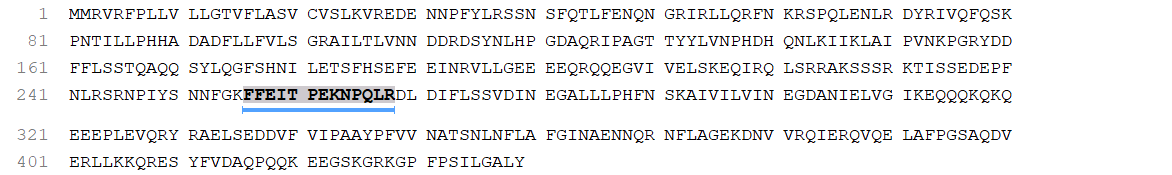

Supplement: Supplementary file 3 [file DataSheet2.ZIP › HTML/img/cov_2278.png]

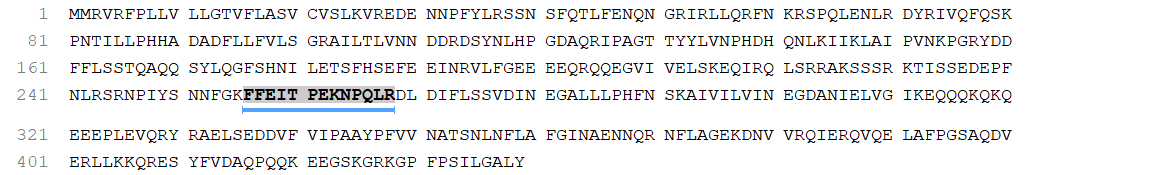

Supplement: Supplementary file 3 [file DataSheet2.ZIP › HTML/img/cov_2279.png]

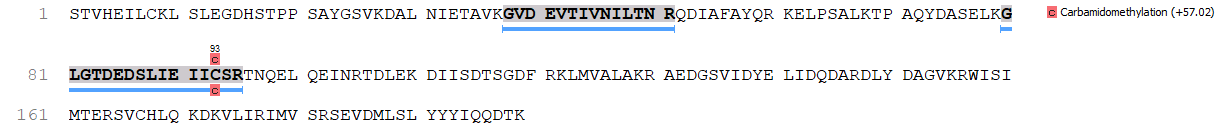

Supplement: Supplementary file 3 [file DataSheet2.ZIP › HTML/img/cov_355.png]

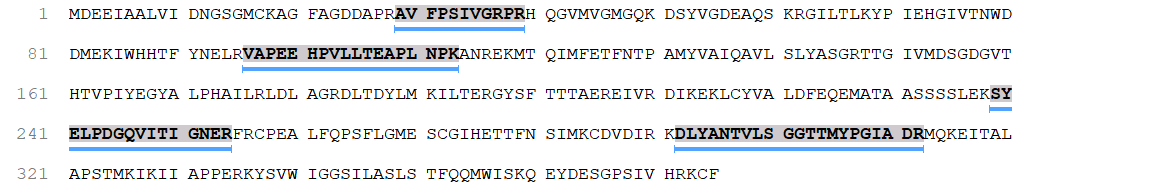

Supplement: Supplementary file 3 [file DataSheet2.ZIP › HTML/img/cov_157.png]

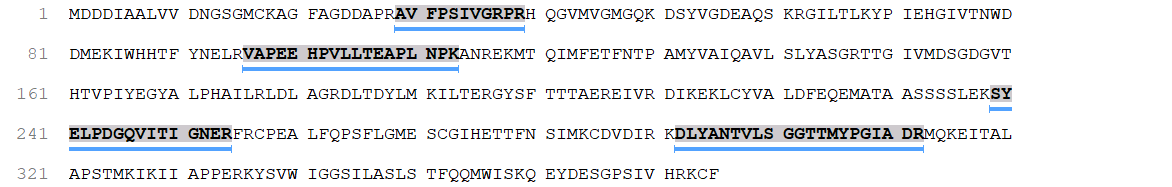

Supplement: Supplementary file 3 [file DataSheet2.ZIP › HTML/img/cov_143.png]

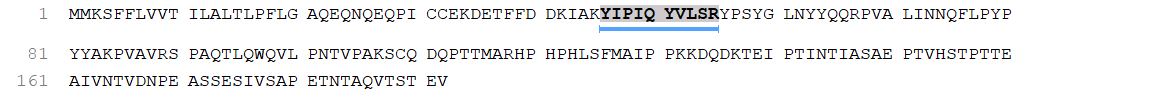

Supplement: Supplementary file 3 [file DataSheet2.ZIP › HTML/img/cov_2319.png]

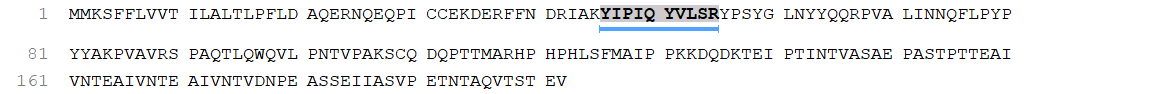

Supplement: Supplementary file 3 [file DataSheet2.ZIP › HTML/img/cov_2321.png]

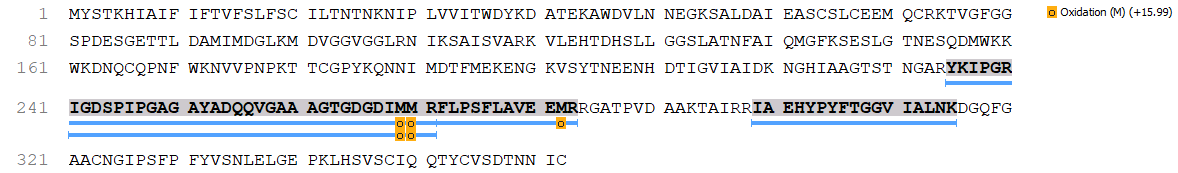

Supplement: Supplementary file 3 [file DataSheet2.ZIP › HTML/img/cov_184.png]

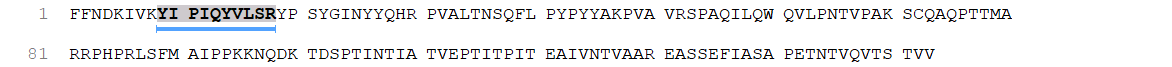

Supplement: Supplementary file 3 [file DataSheet2.ZIP › HTML/img/cov_2309.png]

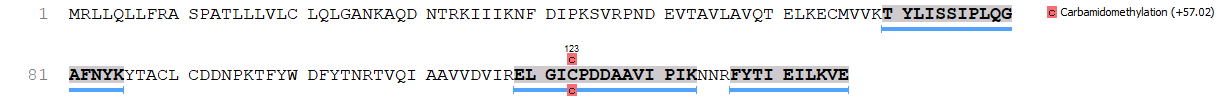

Supplement: Supplementary file 3 [file DataSheet2.ZIP › HTML/img/cov_190.png]

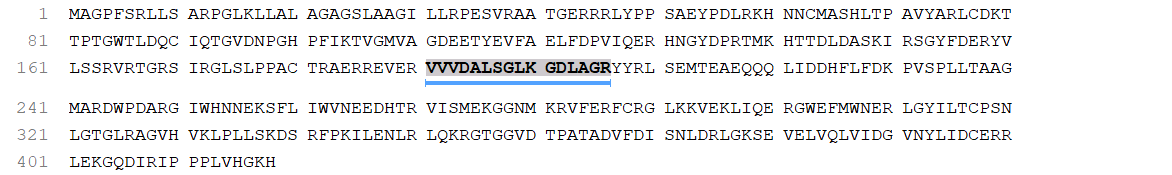

Supplement: Supplementary file 3 [file DataSheet2.ZIP › HTML/img/cov_1370.png]

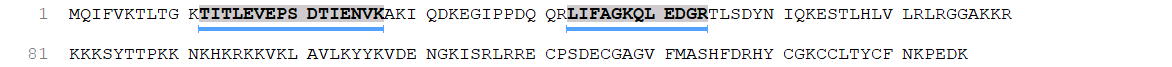

Supplement: Supplementary file 3 [file DataSheet2.ZIP › HTML/img/cov_379.png]

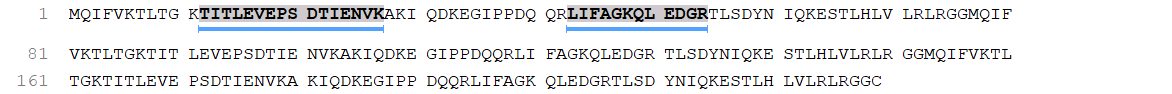

Supplement: Supplementary file 3 [file DataSheet2.ZIP › HTML/img/cov_345.png]

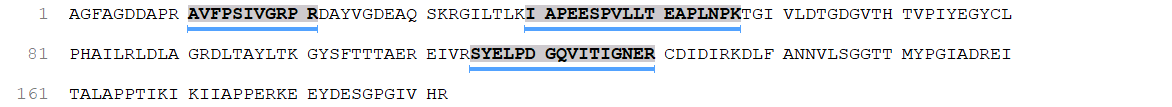

Supplement: Supplementary file 3 [file DataSheet2.ZIP › HTML/img/cov_231.png]

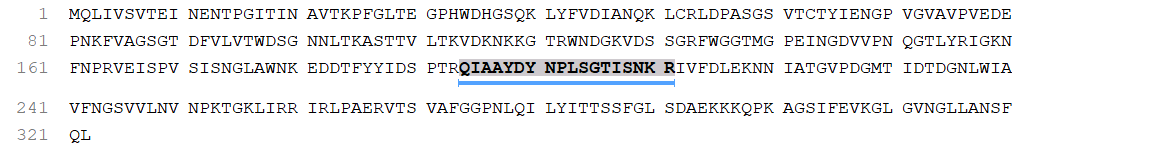

Supplement: Supplementary file 3 [file DataSheet2.ZIP › HTML/img/cov_2269.png]

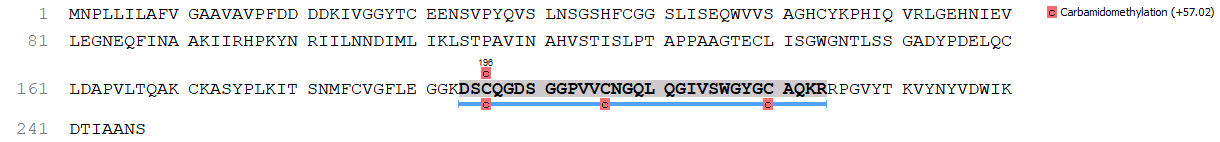

Supplement: Supplementary file 3 [file DataSheet2.ZIP › HTML/img/cov_2296.png]

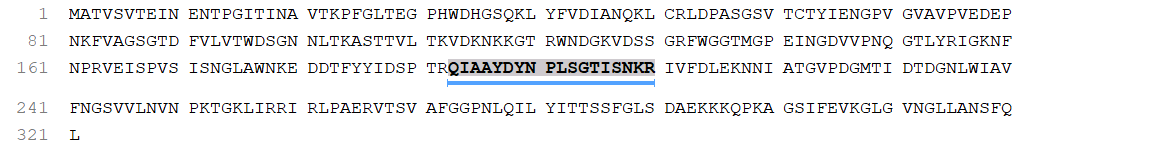

Supplement: Supplementary file 3 [file DataSheet2.ZIP › HTML/img/cov_2268.png]

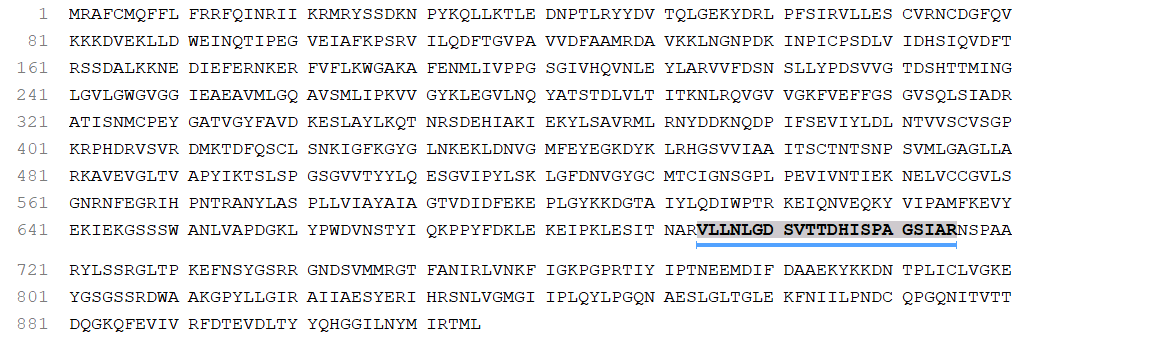

Supplement: Supplementary file 3 [file DataSheet2.ZIP › HTML/img/cov_1588.png]

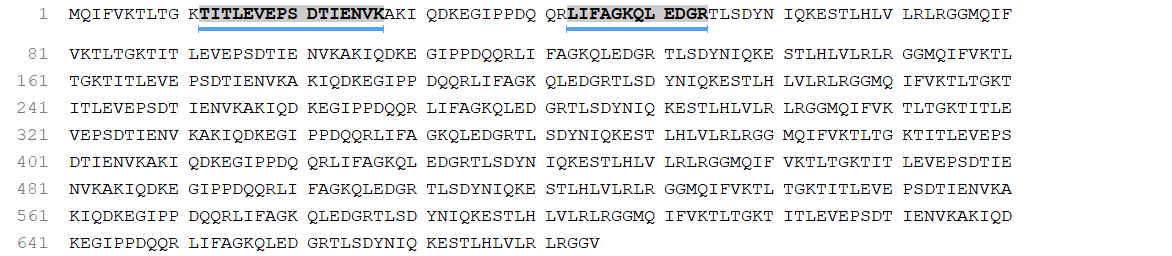

Supplement: Supplementary file 3 [file DataSheet2.ZIP › HTML/img/cov_350.png]

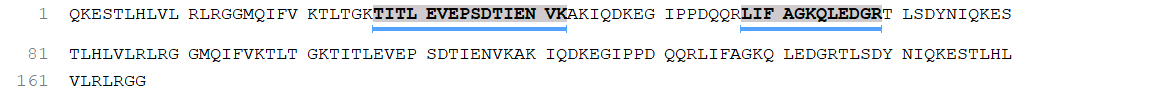

Supplement: Supplementary file 3 [file DataSheet2.ZIP › HTML/img/cov_344.png]

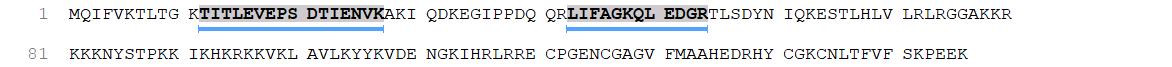

Supplement: Supplementary file 3 [file DataSheet2.ZIP › HTML/img/cov_378.png]

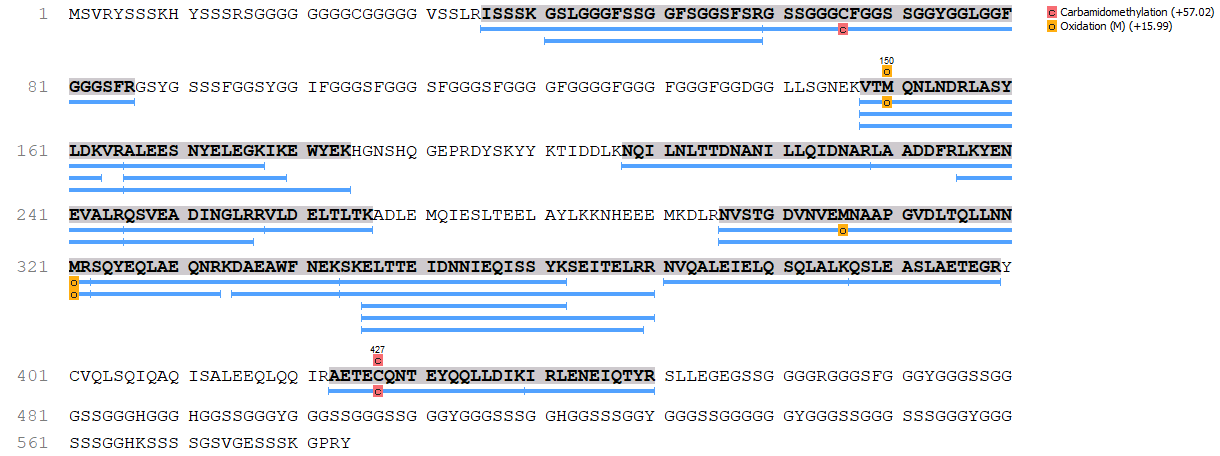

Supplement: Supplementary file 3 [file DataSheet2.ZIP › HTML/img/cov_16.png]

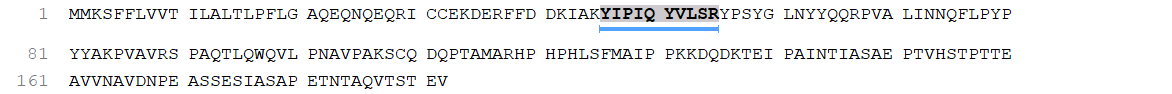

Supplement: Supplementary file 3 [file DataSheet2.ZIP › HTML/img/cov_2320.png]

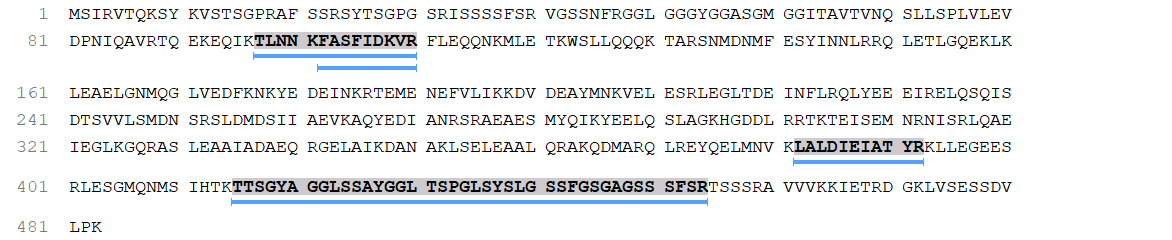

Supplement: Supplementary file 3 [file DataSheet2.ZIP › HTML/img/cov_187.png]

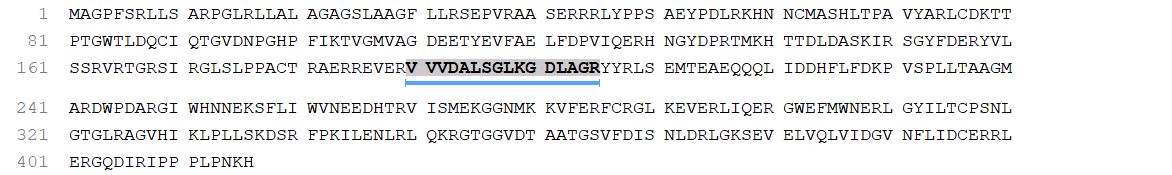

Supplement: Supplementary file 3 [file DataSheet2.ZIP › HTML/img/cov_1367.png]

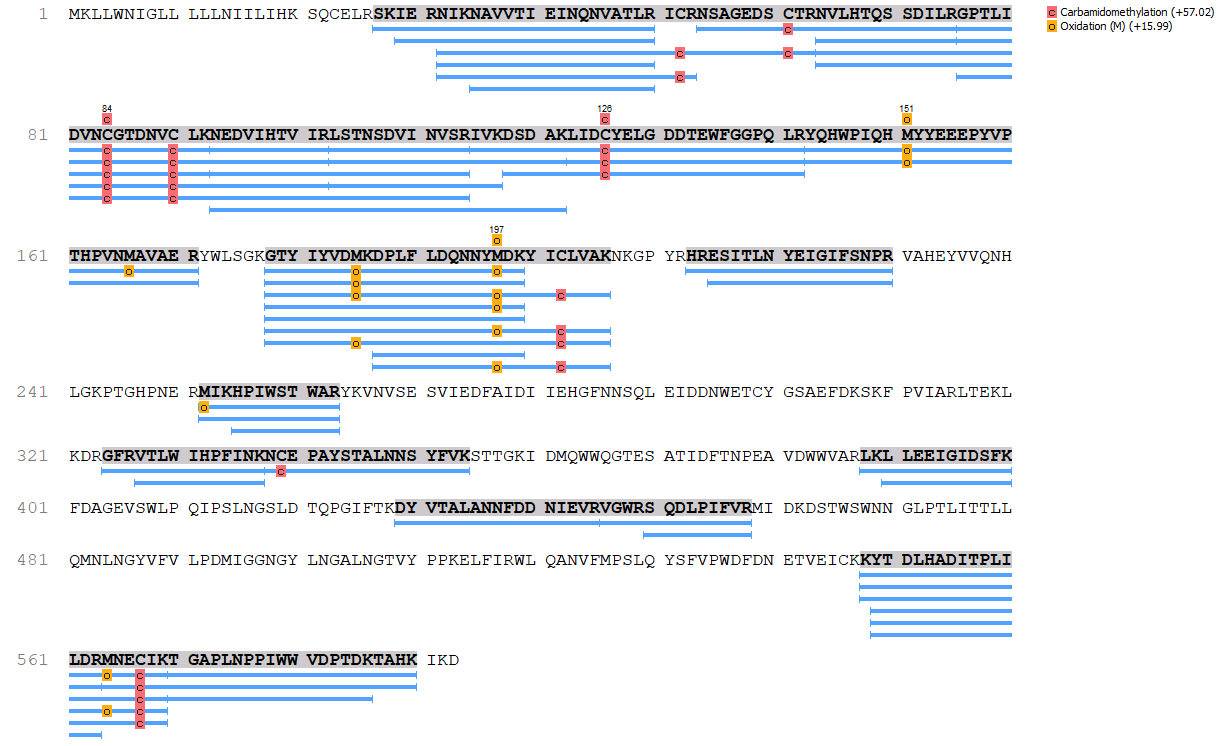

Supplement: Supplementary file 3 [file DataSheet2.ZIP › HTML/img/cov_14.png]

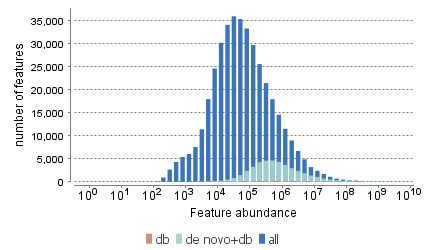

Supplement: Supplementary file 3 [file DataSheet2.ZIP › HTML/img/FeatureIntensityDistributionHistogram329112937019771352.png]

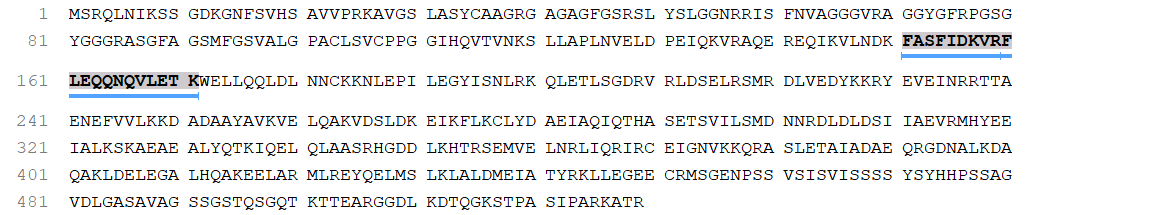

Supplement: Supplementary file 3 [file DataSheet2.ZIP › HTML/img/cov_352.png]

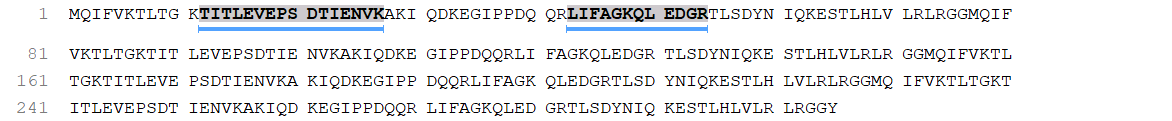

Supplement: Supplementary file 3 [file DataSheet2.ZIP › HTML/img/cov_346.png]
