# Supplementary figures and images for: Stings on wings: Proteotranscriptomic and biochemical profiling of the lesser banded hornet (Vespa affinis) venom (part 1 of 2)
Source: Front Mol Biosci. 2022 Dec 19;9:1066793. doi: 10.3389/fmolb.2022.1066793 (PMC9806352; doi:10.3389/fmolb.2022.1066793)

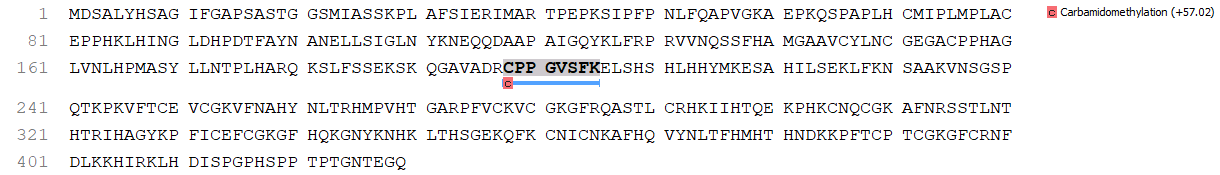

Supplement: Supplementary file 3 [file DataSheet2.ZIP › HTML/img/cov_2403.png]

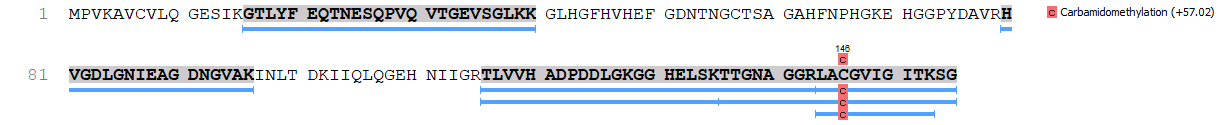

Supplement: Supplementary file 3 [file DataSheet2.ZIP › HTML/img/cov_117.png]

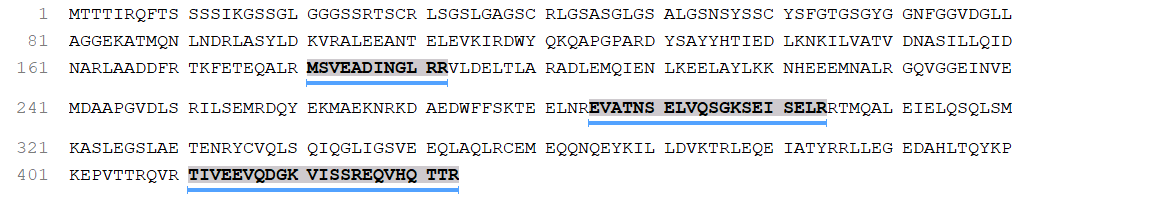

Supplement: Supplementary file 3 [file DataSheet2.ZIP › HTML/img/cov_103.png]

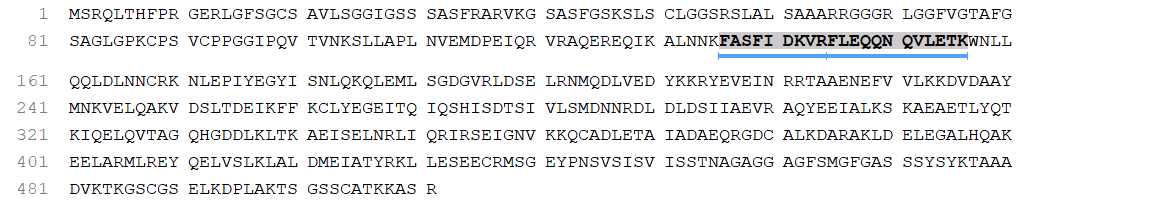

Supplement: Supplementary file 3 [file DataSheet2.ZIP › HTML/img/cov_301.png]

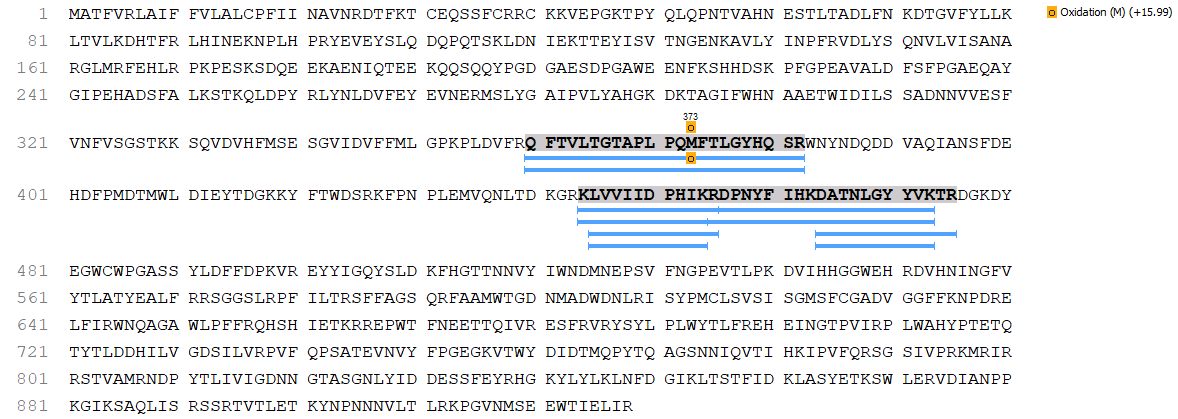

Supplement: Supplementary file 3 [file DataSheet2.ZIP › HTML/img/cov_90.png]

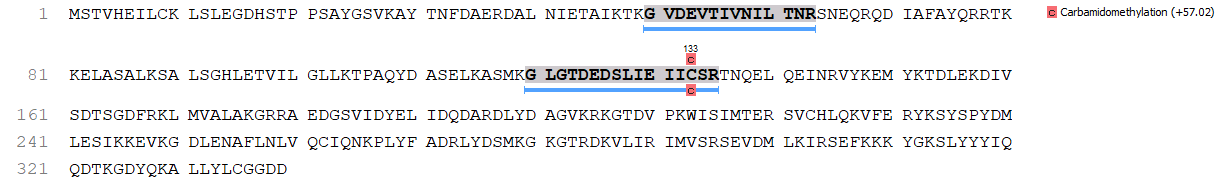

Supplement: Supplementary file 3 [file DataSheet2.ZIP › HTML/img/cov_329.png]

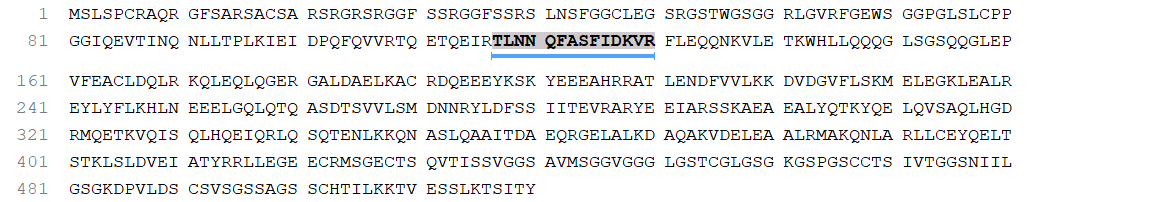

Supplement: Supplementary file 3 [file DataSheet2.ZIP › HTML/img/cov_261.png]

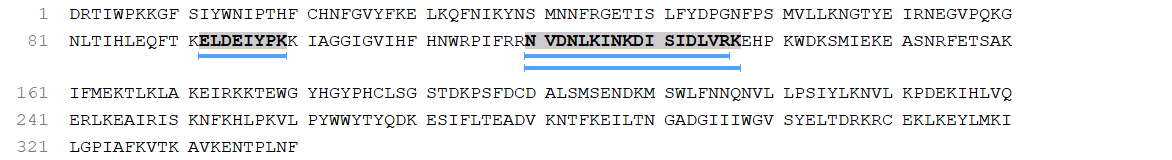

Supplement: Supplementary file 3 [file DataSheet2.ZIP › HTML/img/cov_249.png]

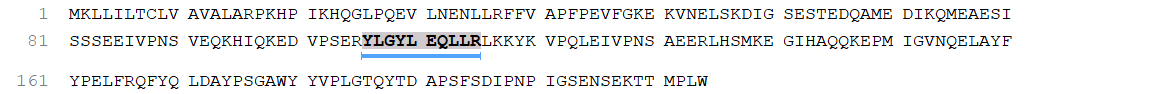

Supplement: Supplementary file 3 [file DataSheet2.ZIP › HTML/img/cov_738.png]

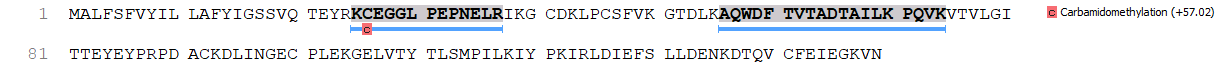

Supplement: Supplementary file 3 [file DataSheet2.ZIP › HTML/img/cov_248.png]

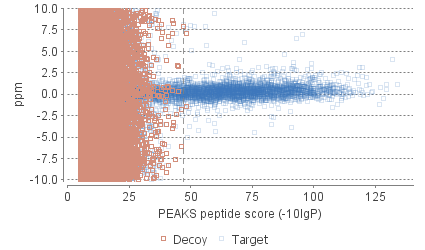

Supplement: Supplementary file 3 [file DataSheet2.ZIP › HTML/img/ScorePlotFigure1601598634069930957.png]

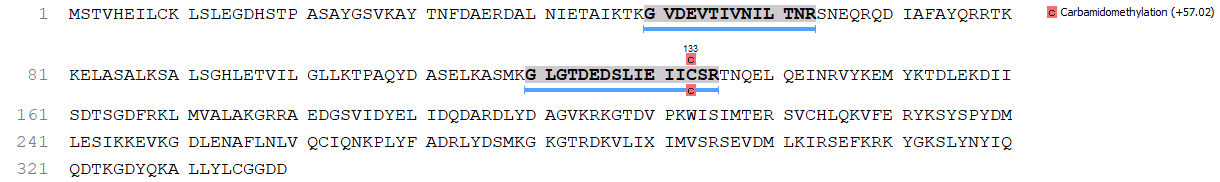

Supplement: Supplementary file 3 [file DataSheet2.ZIP › HTML/img/cov_328.png]

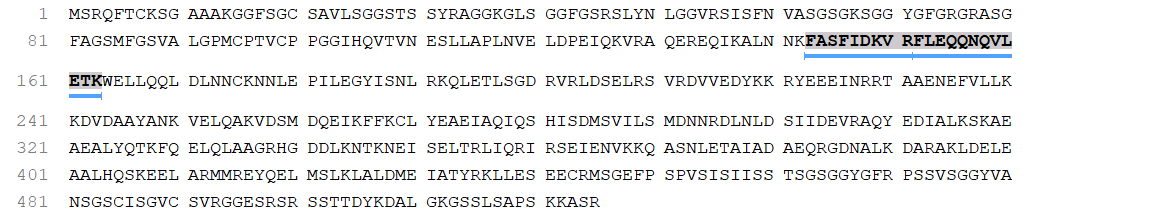

Supplement: Supplementary file 3 [file DataSheet2.ZIP › HTML/img/cov_300.png]

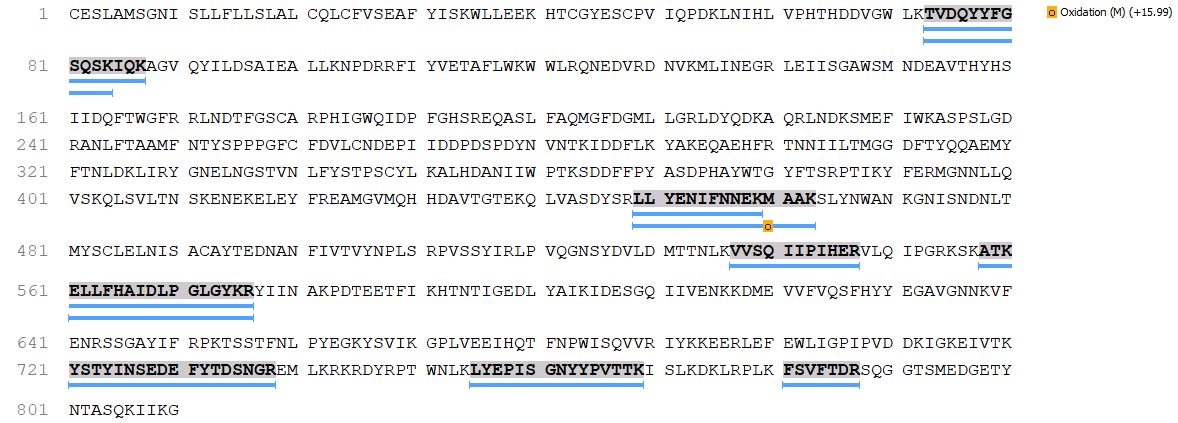

Supplement: Supplementary file 3 [file DataSheet2.ZIP › HTML/img/cov_52.png]

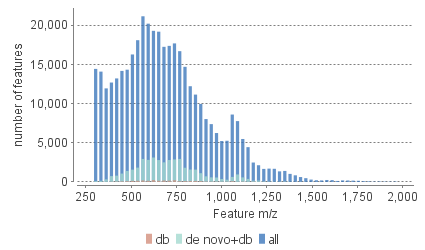

Supplement: Supplementary file 3 [file DataSheet2.ZIP › HTML/img/FeatureMzHistogram2380740725532930224.png]

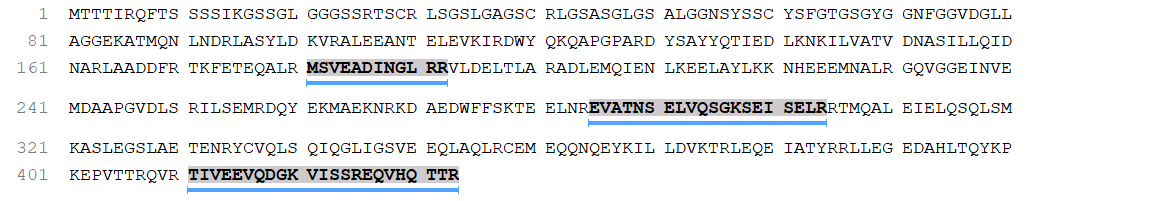

Supplement: Supplementary file 3 [file DataSheet2.ZIP › HTML/img/cov_100.png]

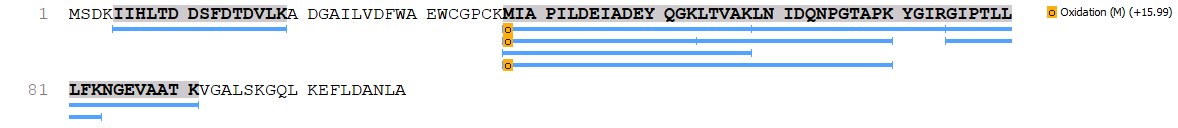

Supplement: Supplementary file 3 [file DataSheet2.ZIP › HTML/img/cov_128.png]

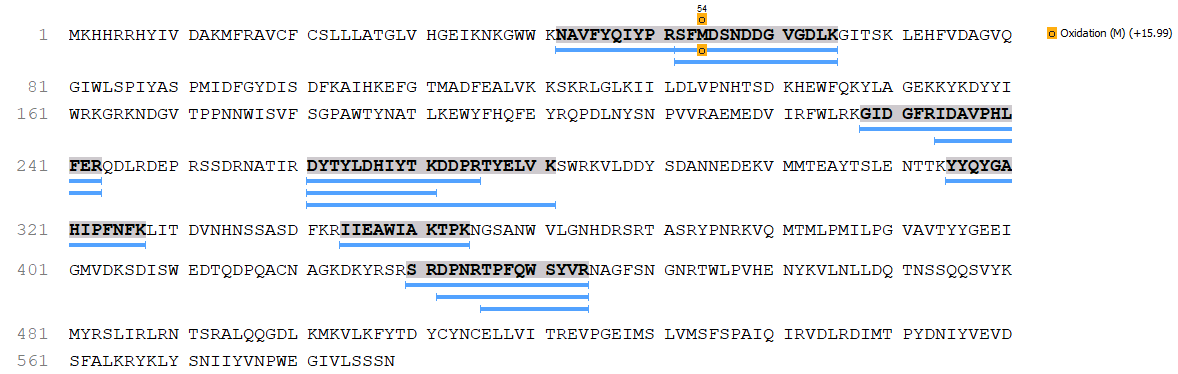

Supplement: Supplementary file 3 [file DataSheet2.ZIP › HTML/img/cov_50.png]

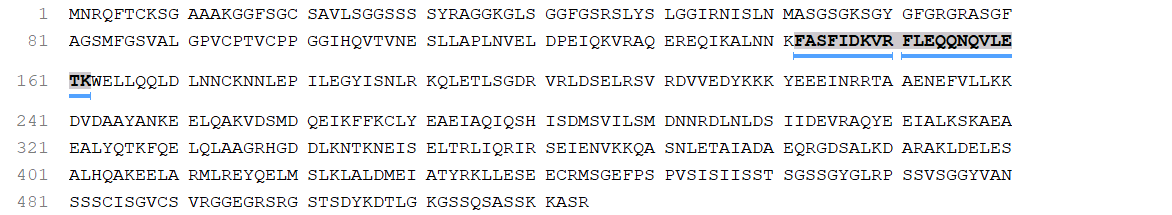

Supplement: Supplementary file 3 [file DataSheet2.ZIP › HTML/img/cov_302.png]

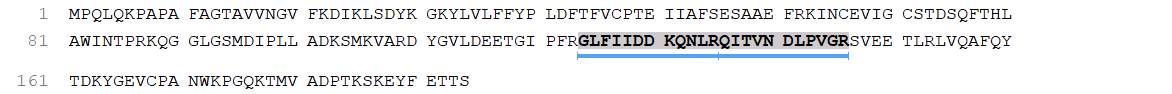

Supplement: Supplementary file 3 [file DataSheet2.ZIP › HTML/img/cov_712.png]

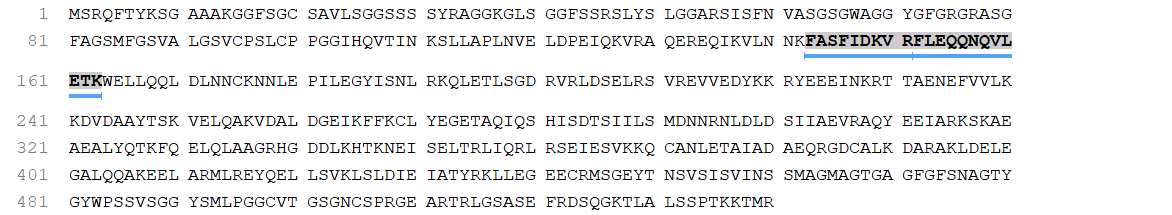

Supplement: Supplementary file 3 [file DataSheet2.ZIP › HTML/img/cov_288.png]

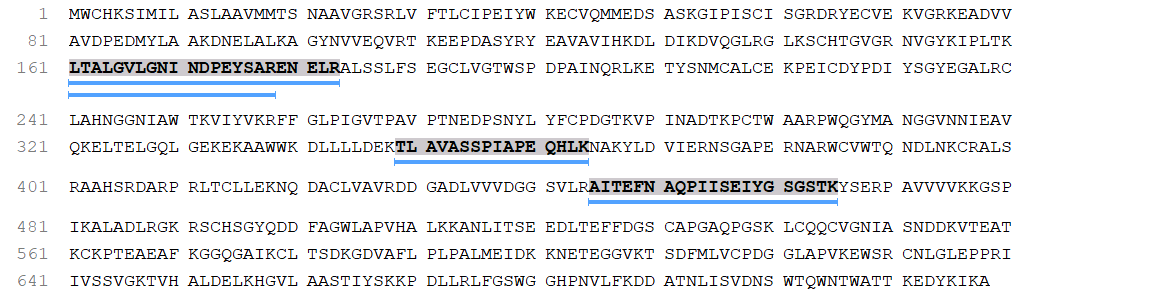

Supplement: Supplementary file 3 [file DataSheet2.ZIP › HTML/img/cov_86.png]

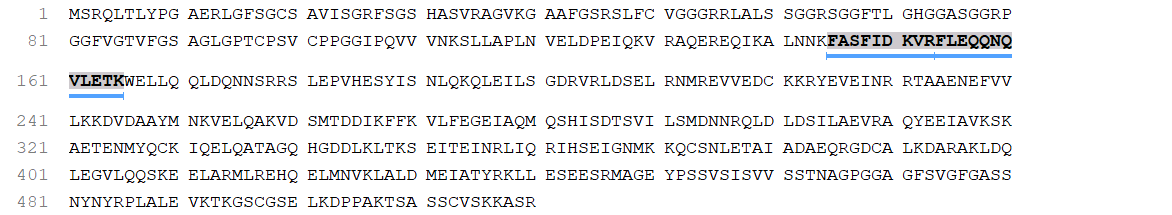

Supplement: Supplementary file 3 [file DataSheet2.ZIP › HTML/img/cov_303.png]

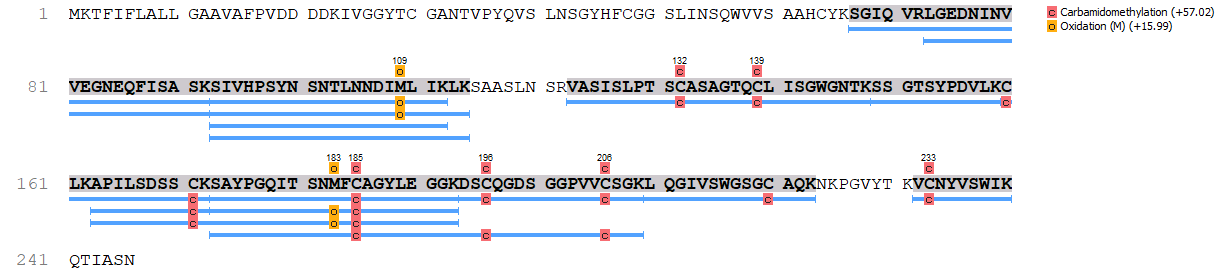

Supplement: Supplementary file 3 [file DataSheet2.ZIP › HTML/img/cov_45.png]

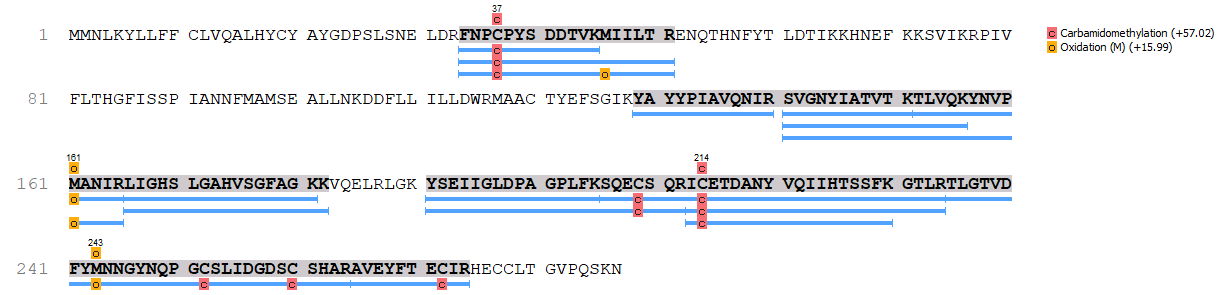

Supplement: Supplementary file 3 [file DataSheet2.ZIP › HTML/img/cov_51.png]

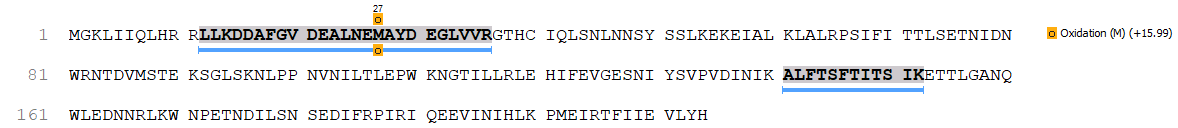

Supplement: Supplementary file 3 [file DataSheet2.ZIP › HTML/img/cov_698.png]

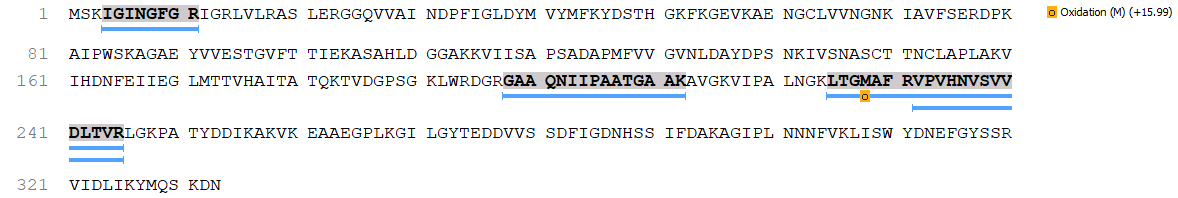

Supplement: Supplementary file 3 [file DataSheet2.ZIP › HTML/img/cov_139.png]

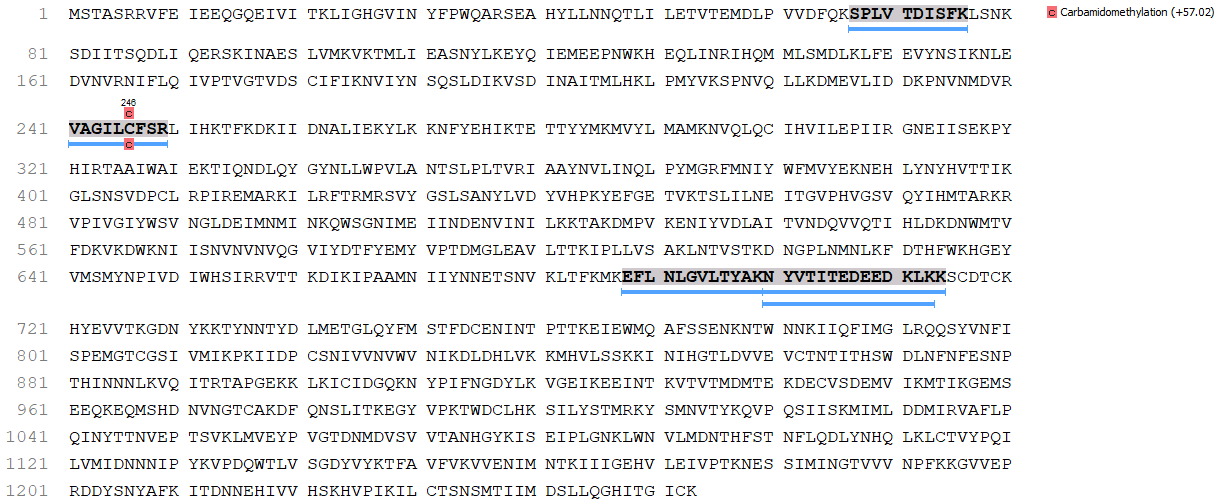

Supplement: Supplementary file 3 [file DataSheet2.ZIP › HTML/img/cov_105.png]

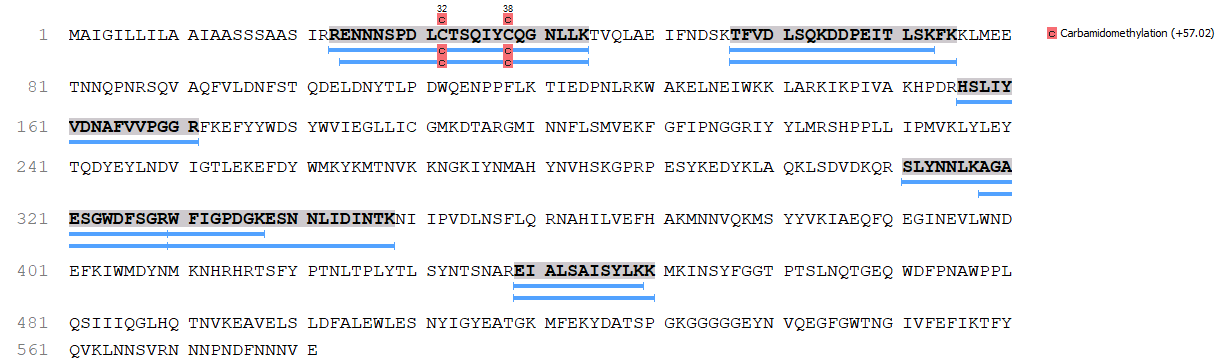

Supplement: Supplementary file 3 [file DataSheet2.ZIP › HTML/img/cov_69.png]

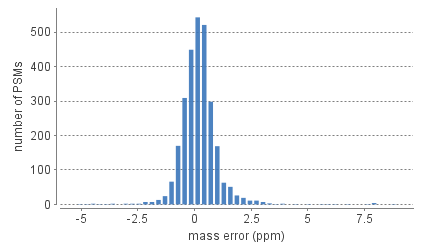

Supplement: Supplementary file 3 [file DataSheet2.ZIP › HTML/img/ErrorCalibratedHistogram1712327621383594172.png]

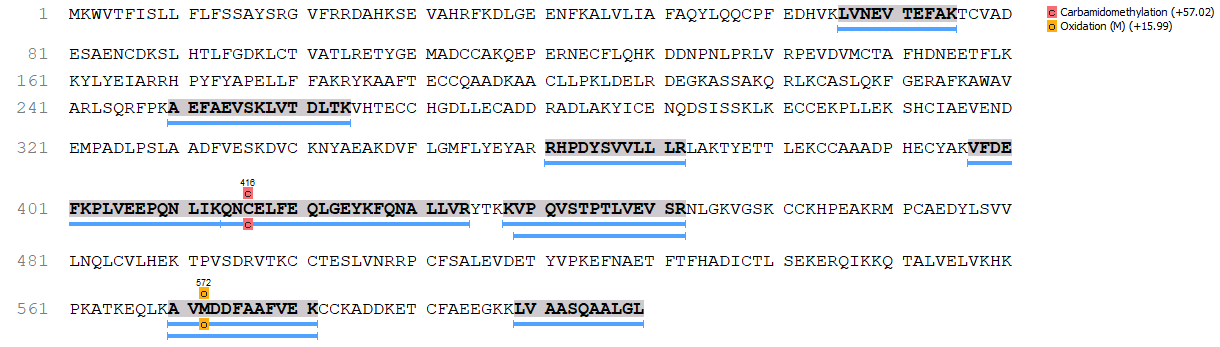

Supplement: Supplementary file 3 [file DataSheet2.ZIP › HTML/img/cov_41.png]

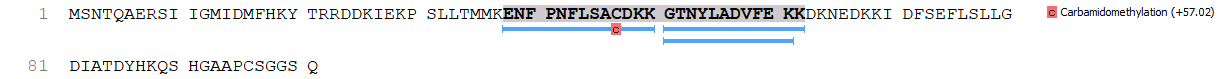

Supplement: Supplementary file 3 [file DataSheet2.ZIP › HTML/img/cov_313.png]

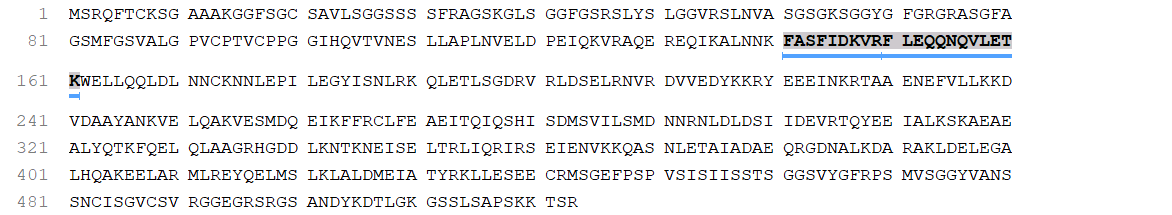

Supplement: Supplementary file 3 [file DataSheet2.ZIP › HTML/img/cov_298.png]

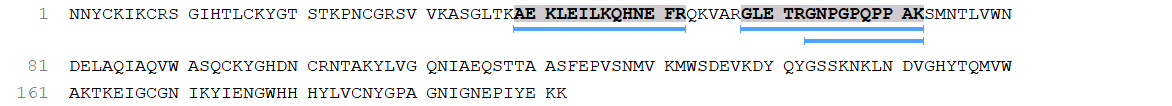

Supplement: Supplementary file 3 [file DataSheet2.ZIP › HTML/img/cov_702.png]

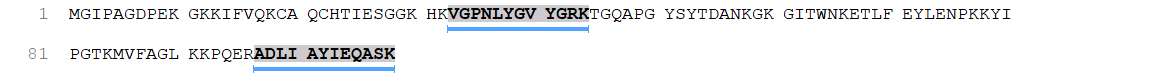

Supplement: Supplementary file 3 [file DataSheet2.ZIP › HTML/img/cov_312.png]

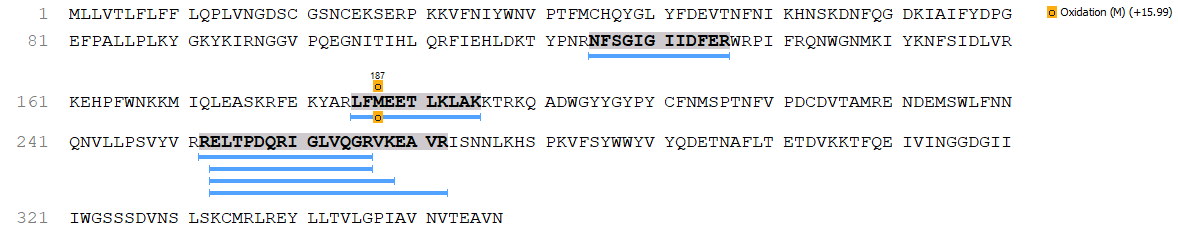

Supplement: Supplementary file 3 [file DataSheet2.ZIP › HTML/img/cov_97.png]

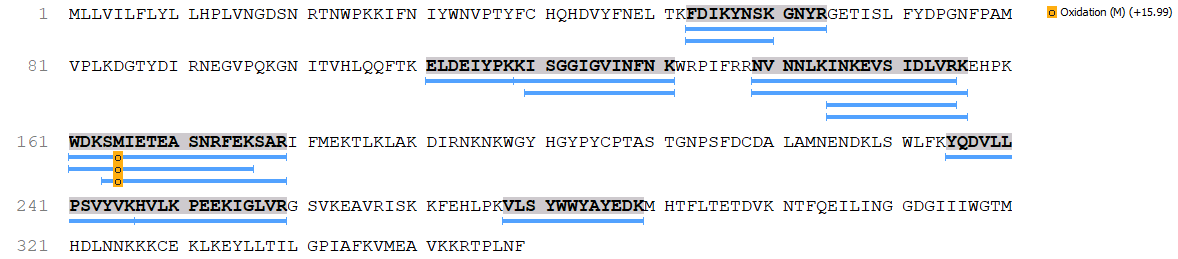

Supplement: Supplementary file 3 [file DataSheet2.ZIP › HTML/img/cov_40.png]

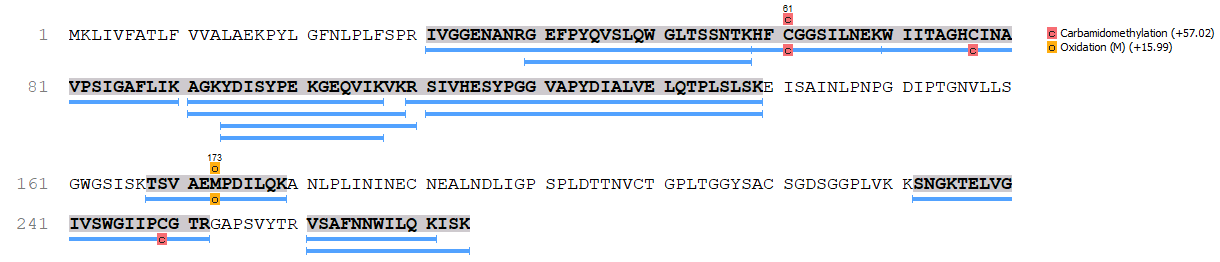

Supplement: Supplementary file 3 [file DataSheet2.ZIP › HTML/img/cov_54.png]

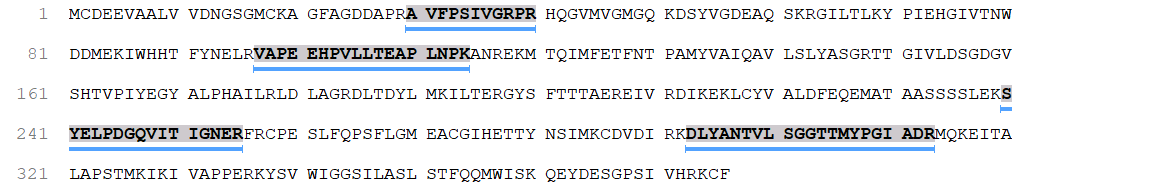

Supplement: Supplementary file 3 [file DataSheet2.ZIP › HTML/img/cov_138.png]

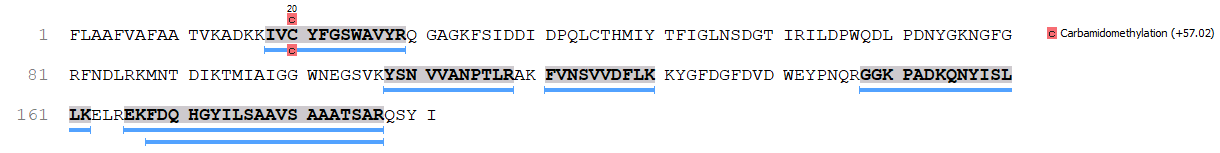

Supplement: Supplementary file 3 [file DataSheet2.ZIP › HTML/img/cov_112.png]

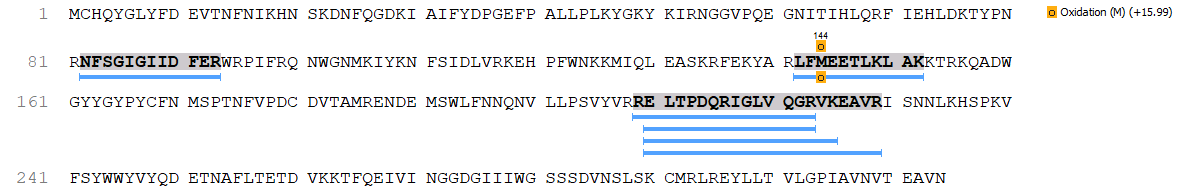

Supplement: Supplementary file 3 [file DataSheet2.ZIP › HTML/img/cov_95.png]

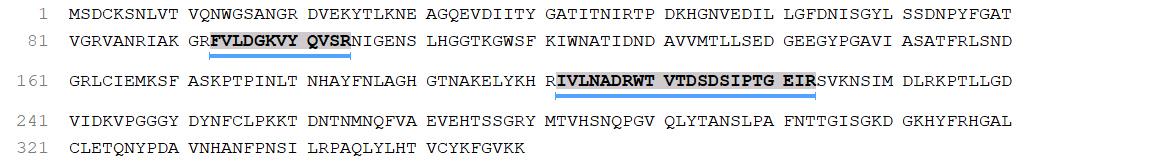

Supplement: Supplementary file 3 [file DataSheet2.ZIP › HTML/img/cov_700.png]

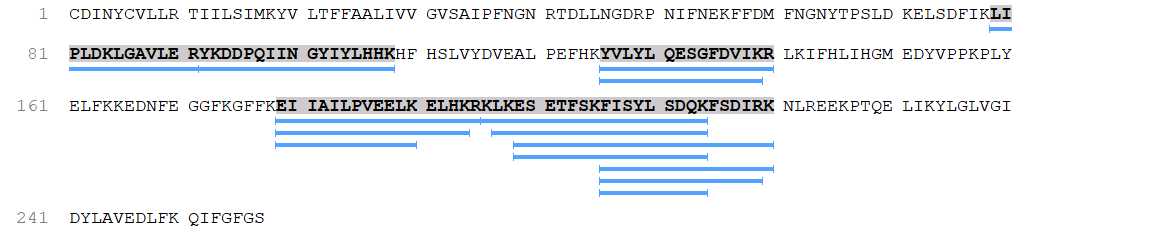

Supplement: Supplementary file 3 [file DataSheet2.ZIP › HTML/img/cov_57.png]

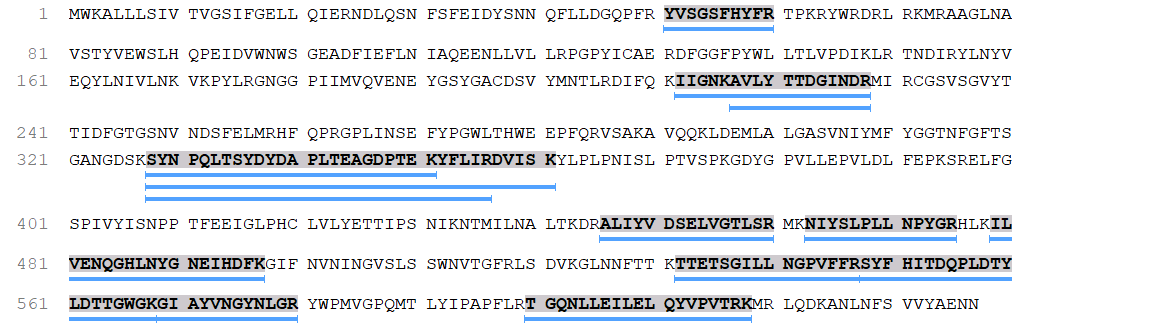

Supplement: Supplementary file 3 [file DataSheet2.ZIP › HTML/img/cov_43.png]

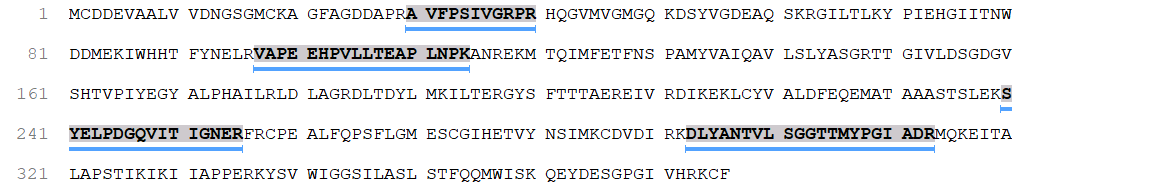

Supplement: Supplementary file 3 [file DataSheet2.ZIP › HTML/img/cov_148.png]

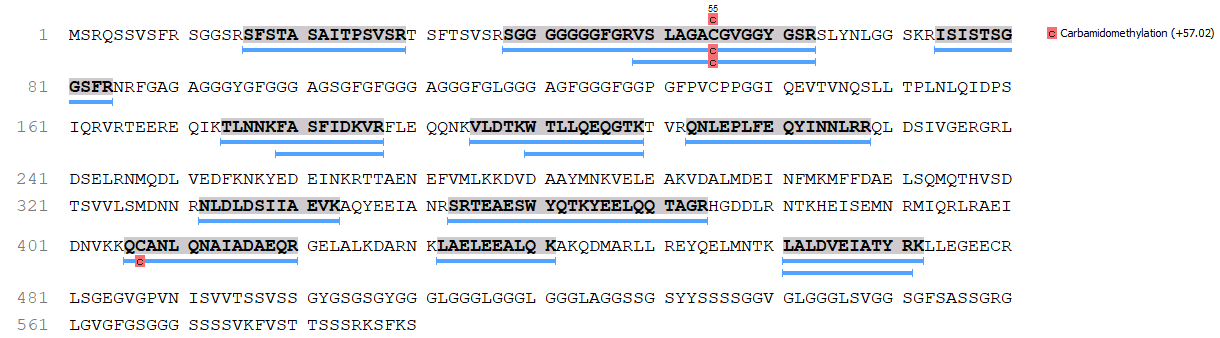

Supplement: Supplementary file 3 [file DataSheet2.ZIP › HTML/img/cov_24.png]

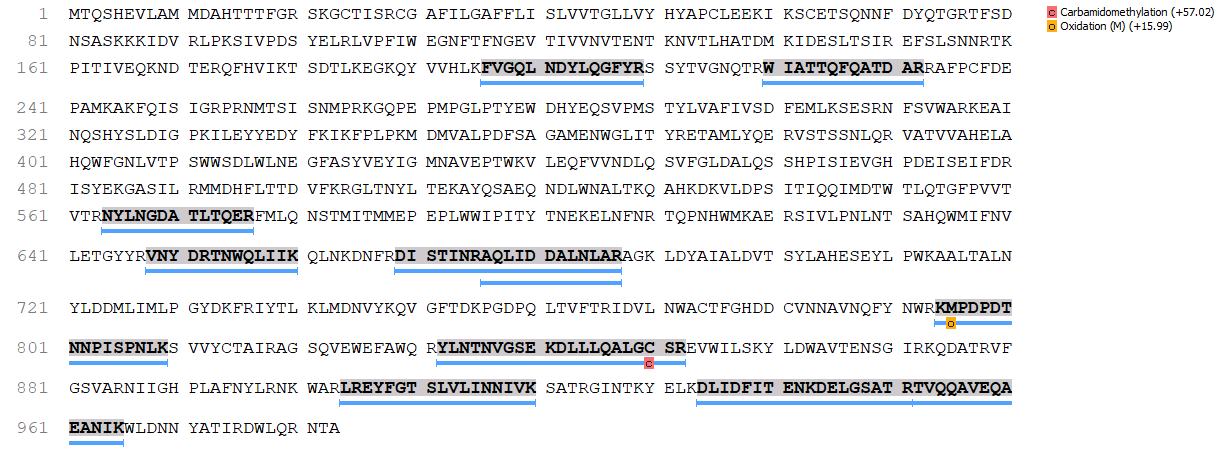

Supplement: Supplementary file 3 [file DataSheet2.ZIP › HTML/img/cov_30.png]

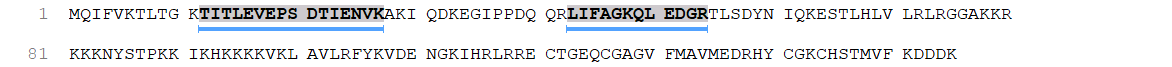

Supplement: Supplementary file 3 [file DataSheet2.ZIP › HTML/img/cov_376.png]

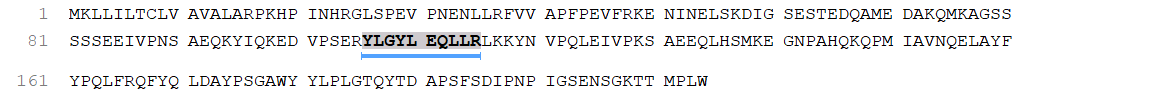

Supplement: Supplementary file 3 [file DataSheet2.ZIP › HTML/img/cov_1545.png]

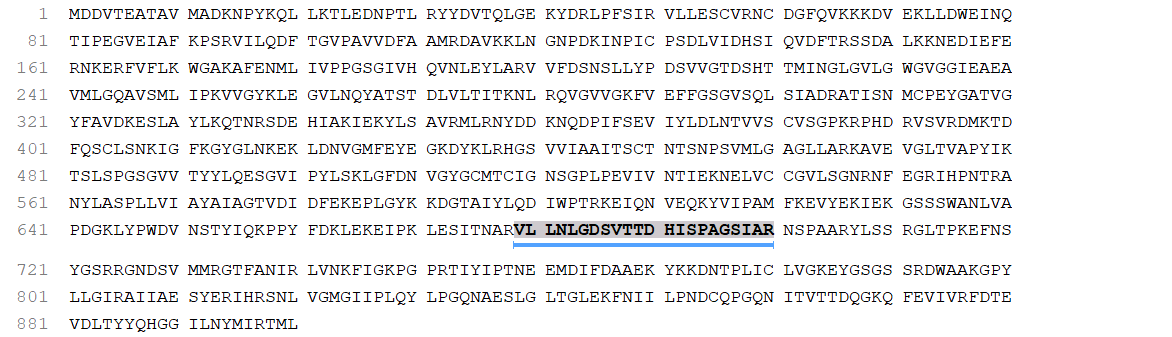

Supplement: Supplementary file 3 [file DataSheet2.ZIP › HTML/img/cov_1586.png]

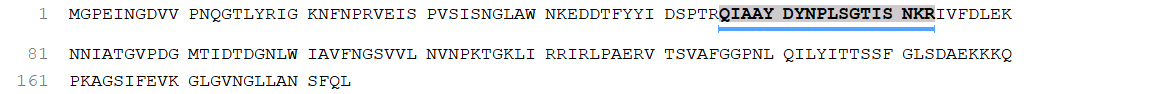

Supplement: Supplementary file 3 [file DataSheet2.ZIP › HTML/img/cov_2266.png]

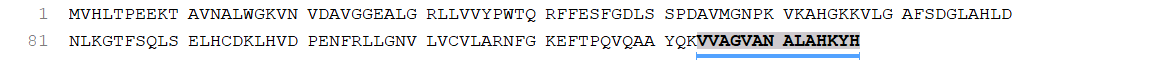

Supplement: Supplementary file 3 [file DataSheet2.ZIP › HTML/img/cov_766.png]

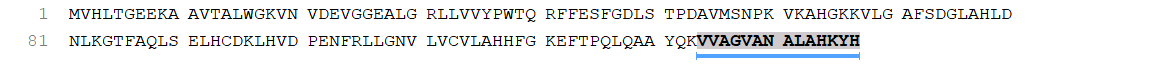

Supplement: Supplementary file 3 [file DataSheet2.ZIP › HTML/img/cov_767.png]

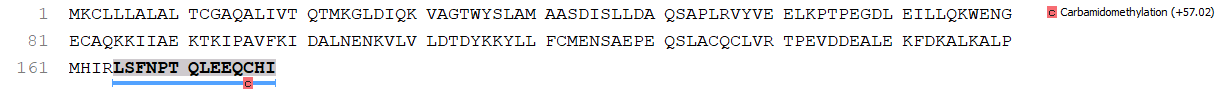

Supplement: Supplementary file 3 [file DataSheet2.ZIP › HTML/img/cov_2273.png]

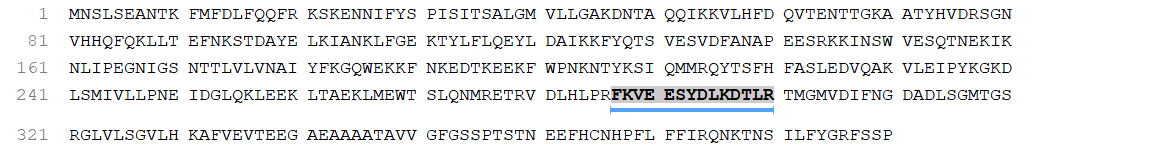

Supplement: Supplementary file 3 [file DataSheet2.ZIP › HTML/img/cov_968.png]

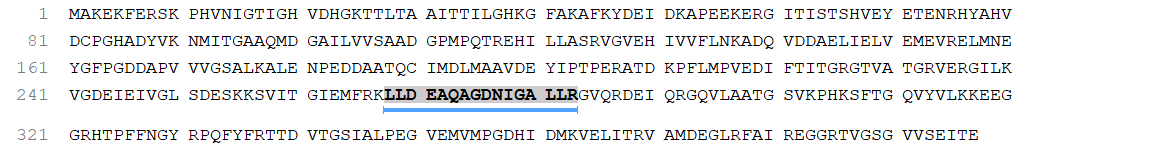

Supplement: Supplementary file 3 [file DataSheet2.ZIP › HTML/img/cov_439.png]

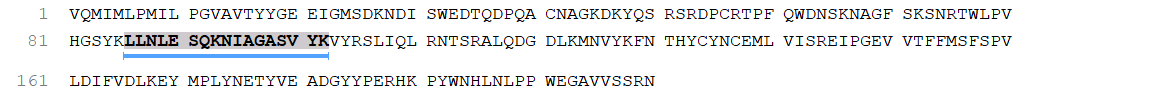

Supplement: Supplementary file 3 [file DataSheet2.ZIP › HTML/img/cov_363.png]

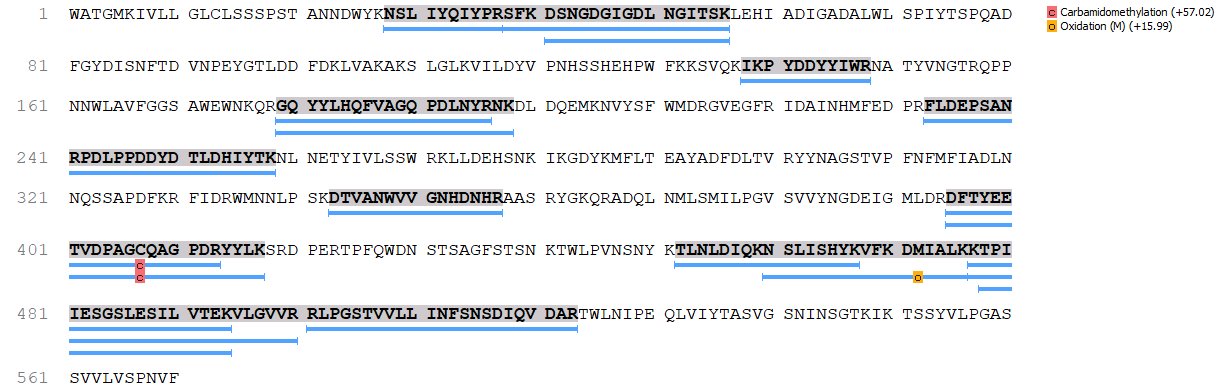

Supplement: Supplementary file 3 [file DataSheet2.ZIP › HTML/img/cov_19.png]

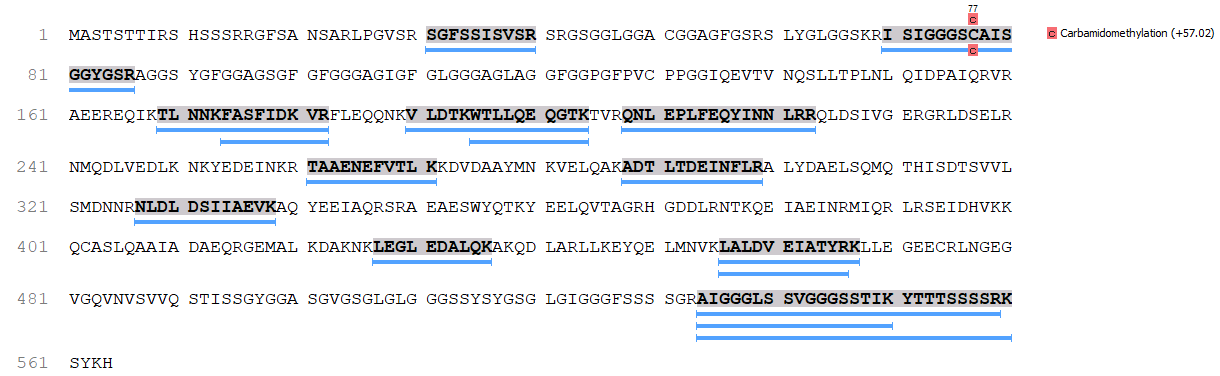

Supplement: Supplementary file 3 [file DataSheet2.ZIP › HTML/img/cov_25.png]

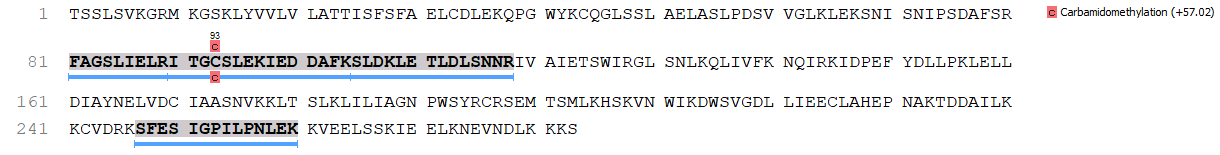

Supplement: Supplementary file 3 [file DataSheet2.ZIP › HTML/img/cov_175.png]

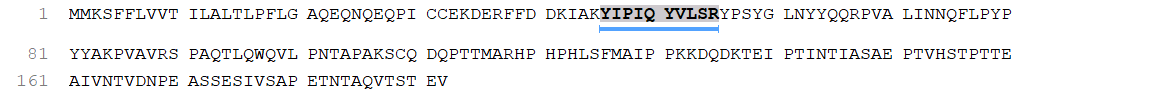

Supplement: Supplementary file 3 [file DataSheet2.ZIP › HTML/img/cov_2313.png]

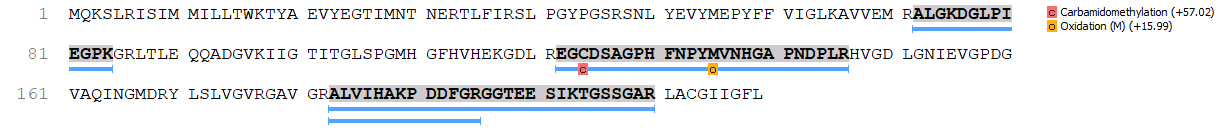

Supplement: Supplementary file 3 [file DataSheet2.ZIP › HTML/img/cov_188.png]

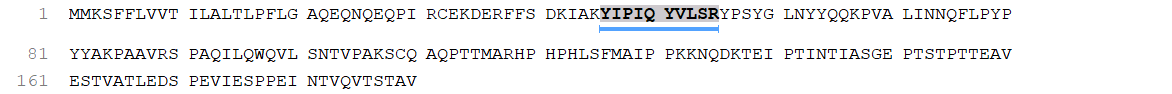

Supplement: Supplementary file 3 [file DataSheet2.ZIP › HTML/img/cov_2311.png]

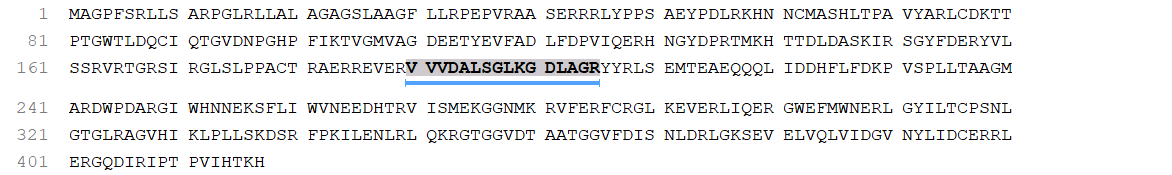

Supplement: Supplementary file 3 [file DataSheet2.ZIP › HTML/img/cov_1368.png]

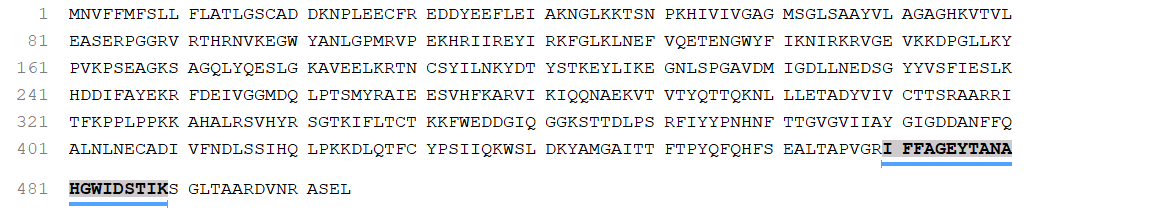

Supplement: Supplementary file 3 [file DataSheet2.ZIP › HTML/img/cov_413.png]

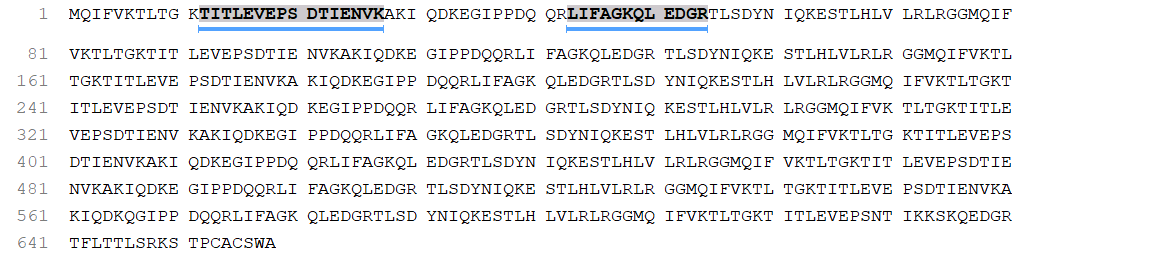

Supplement: Supplementary file 3 [file DataSheet2.ZIP › HTML/img/cov_349.png]

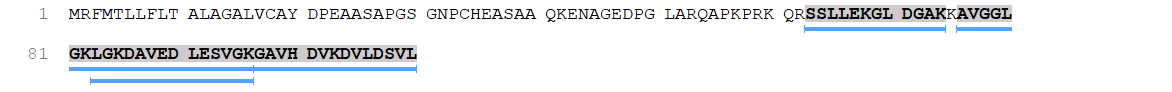

Supplement: Supplementary file 3 [file DataSheet2.ZIP › HTML/img/cov_229.png]

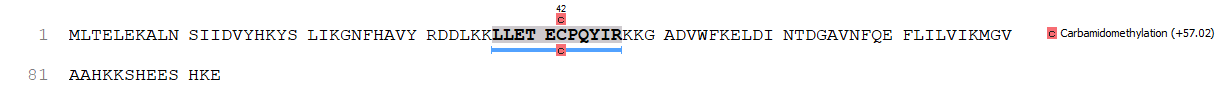

Supplement: Supplementary file 3 [file DataSheet2.ZIP › HTML/img/cov_2271.png]

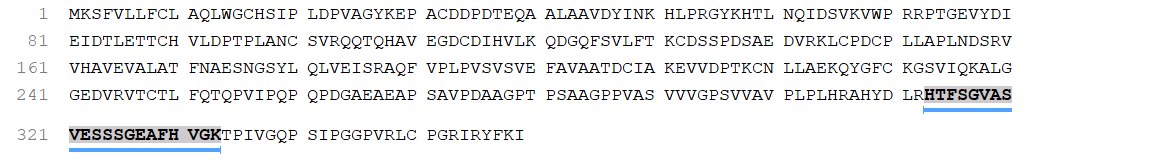

Supplement: Supplementary file 3 [file DataSheet2.ZIP › HTML/img/cov_2265.png]

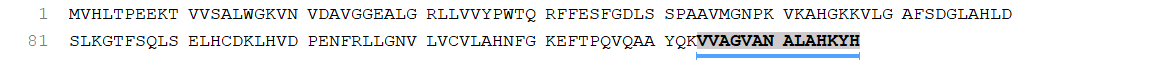

Supplement: Supplementary file 3 [file DataSheet2.ZIP › HTML/img/cov_765.png]

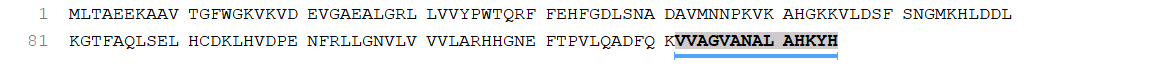

Supplement: Supplementary file 3 [file DataSheet2.ZIP › HTML/img/cov_759.png]

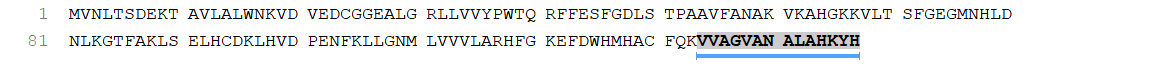

Supplement: Supplementary file 3 [file DataSheet2.ZIP › HTML/img/cov_764.png]

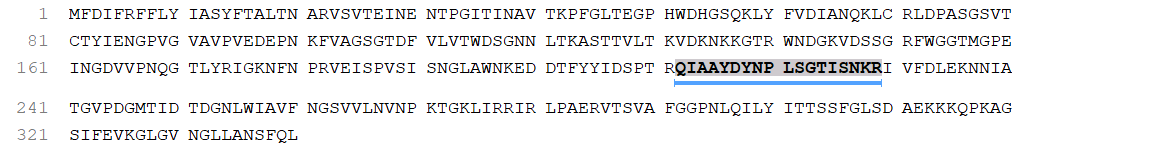

Supplement: Supplementary file 3 [file DataSheet2.ZIP › HTML/img/cov_2270.png]

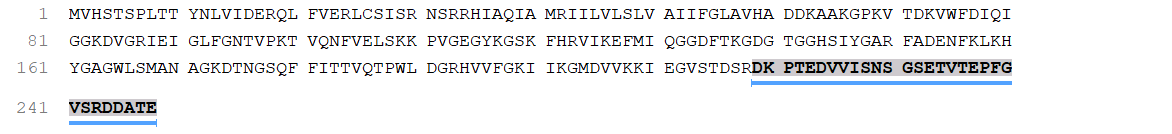

Supplement: Supplementary file 3 [file DataSheet2.ZIP › HTML/img/cov_2258.png]

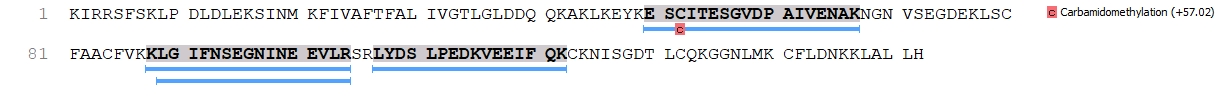

Supplement: Supplementary file 3 [file DataSheet2.ZIP › HTML/img/cov_228.png]

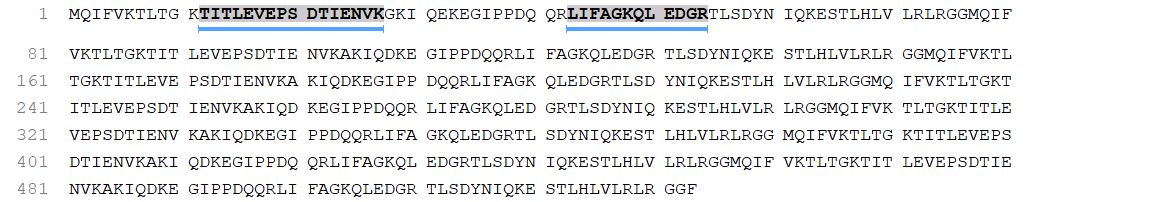

Supplement: Supplementary file 3 [file DataSheet2.ZIP › HTML/img/cov_348.png]

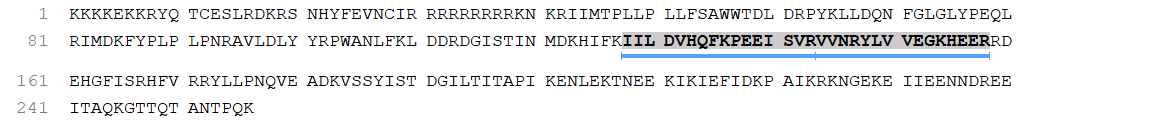

Supplement: Supplementary file 3 [file DataSheet2.ZIP › HTML/img/cov_412.png]

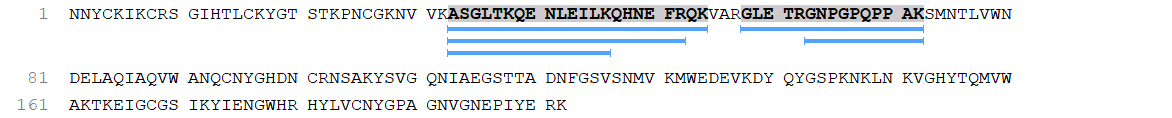

Supplement: Supplementary file 3 [file DataSheet2.ZIP › HTML/img/cov_360.png]

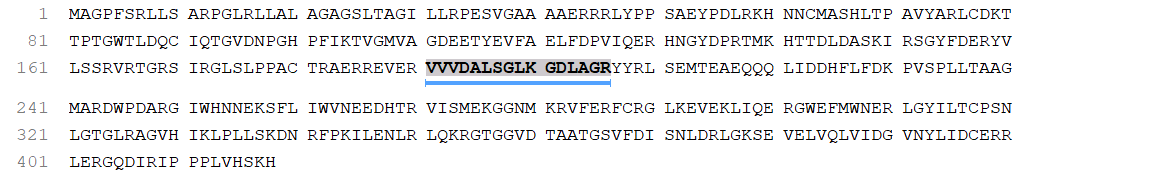

Supplement: Supplementary file 3 [file DataSheet2.ZIP › HTML/img/cov_1369.png]

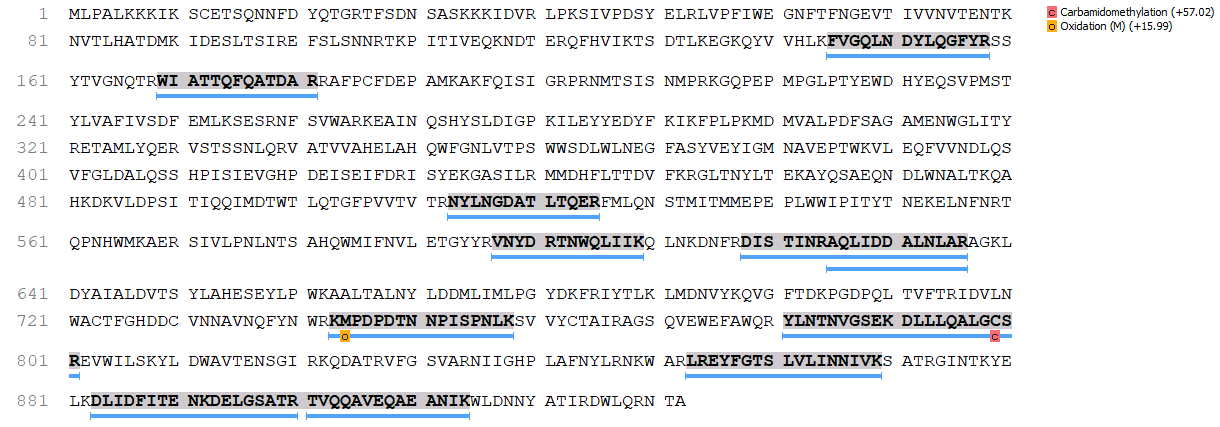

Supplement: Supplementary file 3 [file DataSheet2.ZIP › HTML/img/cov_26.png]

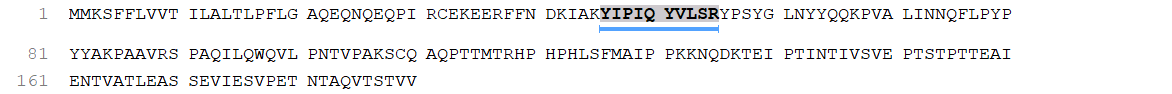

Supplement: Supplementary file 3 [file DataSheet2.ZIP › HTML/img/cov_2310.png]

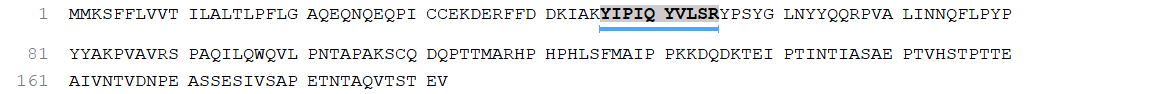

Supplement: Supplementary file 3 [file DataSheet2.ZIP › HTML/img/cov_2314.png]

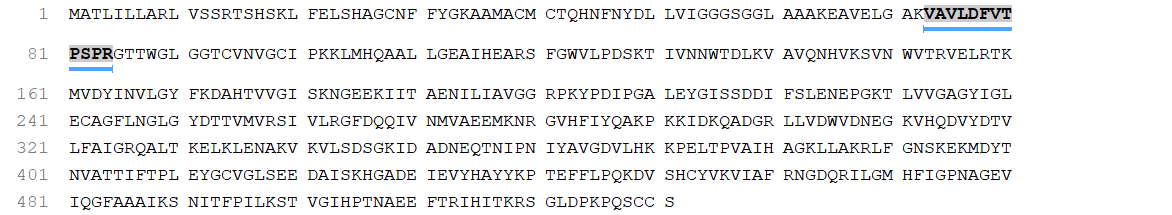

Supplement: Supplementary file 3 [file DataSheet2.ZIP › HTML/img/cov_2328.png]

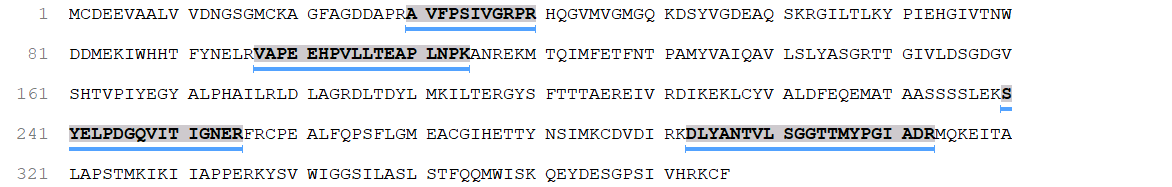

Supplement: Supplementary file 3 [file DataSheet2.ZIP › HTML/img/cov_166.png]

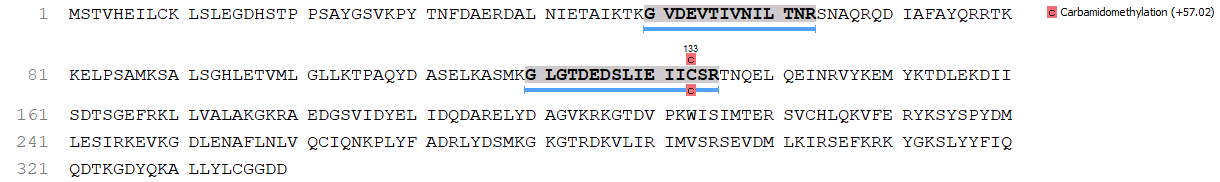

Supplement: Supplementary file 3 [file DataSheet2.ZIP › HTML/img/cov_358.png]

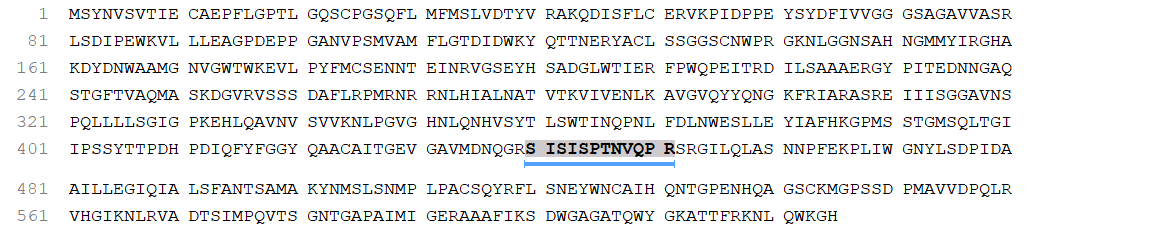

Supplement: Supplementary file 3 [file DataSheet2.ZIP › HTML/img/cov_364.png]

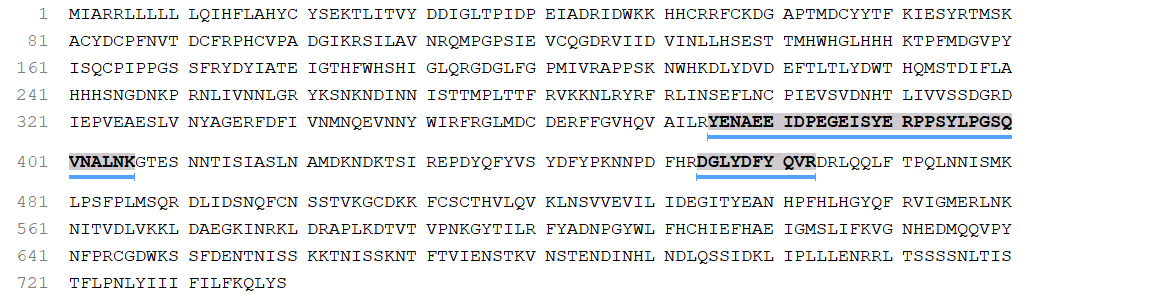

Supplement: Supplementary file 3 [file DataSheet2.ZIP › HTML/img/cov_238.png]

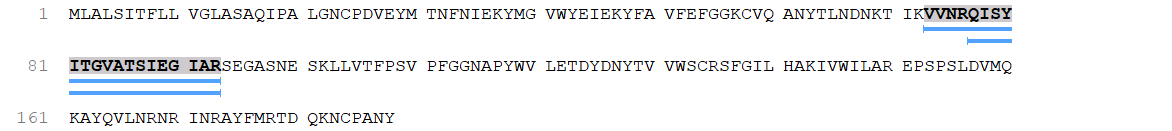

Supplement: Supplementary file 3 [file DataSheet2.ZIP › HTML/img/cov_2260.png]

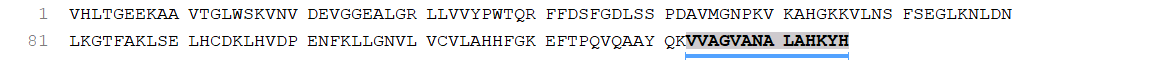

Supplement: Supplementary file 3 [file DataSheet2.ZIP › HTML/img/cov_760.png]

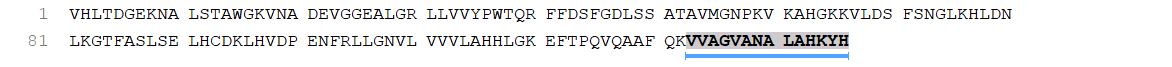

Supplement: Supplementary file 3 [file DataSheet2.ZIP › HTML/img/cov_761.png]

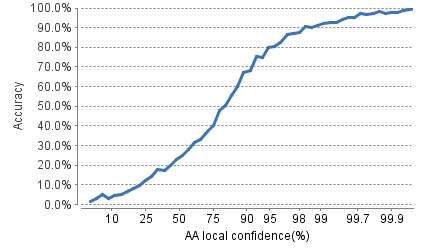

Supplement: Supplementary file 3 [file DataSheet2.ZIP › HTML/img/DenovoFDRCurveFigure4716994871203850499.png]

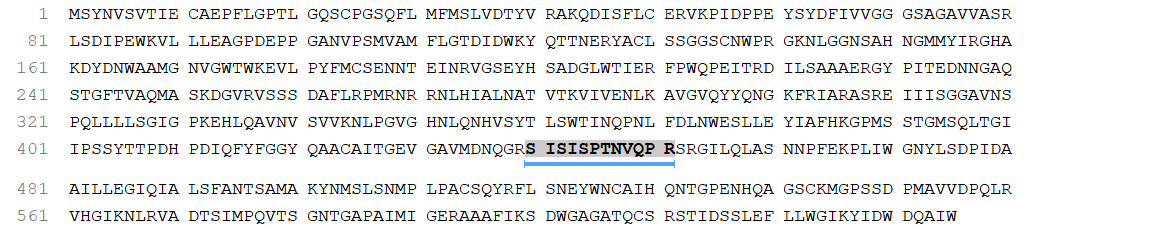

Supplement: Supplementary file 3 [file DataSheet2.ZIP › HTML/img/cov_365.png]

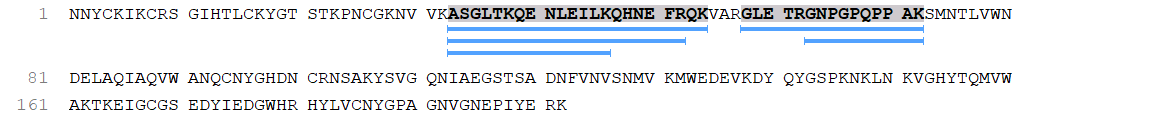

Supplement: Supplementary file 3 [file DataSheet2.ZIP › HTML/img/cov_359.png]

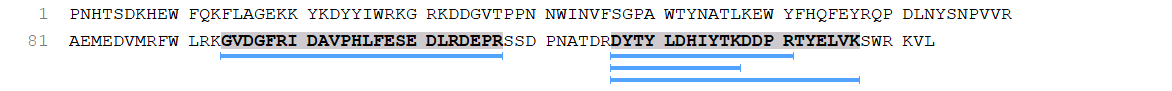

Supplement: Supplementary file 3 [file DataSheet2.ZIP › HTML/img/cov_173.png]

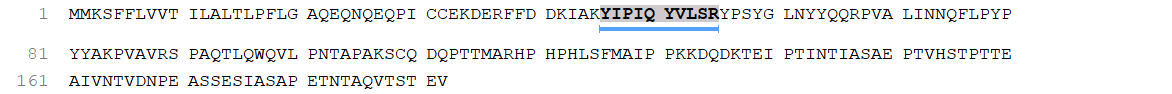

Supplement: Supplementary file 3 [file DataSheet2.ZIP › HTML/img/cov_2315.png]

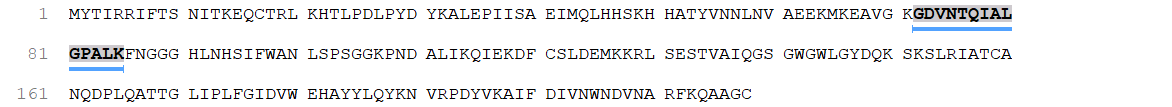

Supplement: Supplementary file 3 [file DataSheet2.ZIP › HTML/img/cov_2301.png]

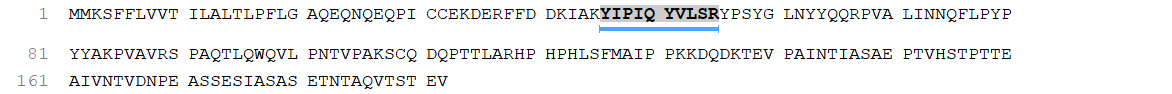

Supplement: Supplementary file 3 [file DataSheet2.ZIP › HTML/img/cov_2317.png]

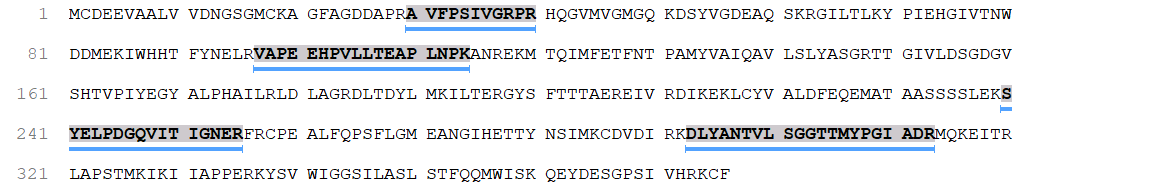

Supplement: Supplementary file 3 [file DataSheet2.ZIP › HTML/img/cov_171.png]

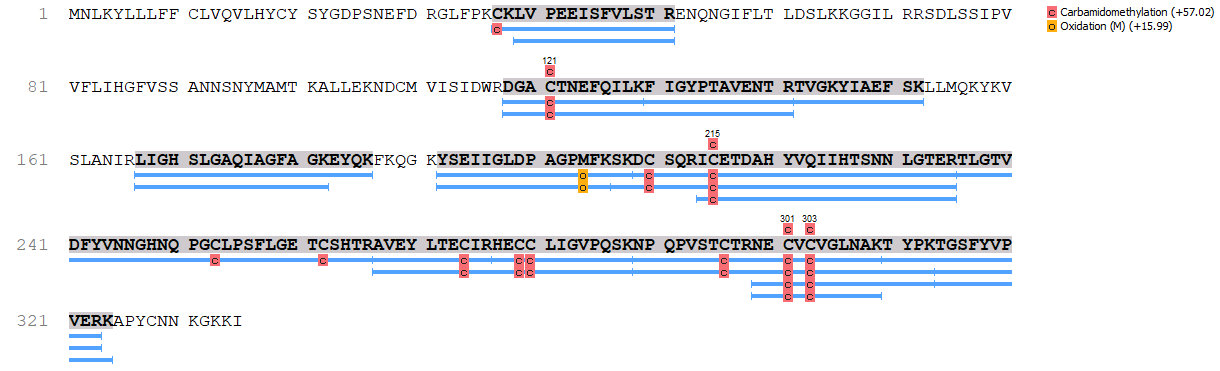

Supplement: Supplementary file 3 [file DataSheet2.ZIP › HTML/img/cov_21.png]

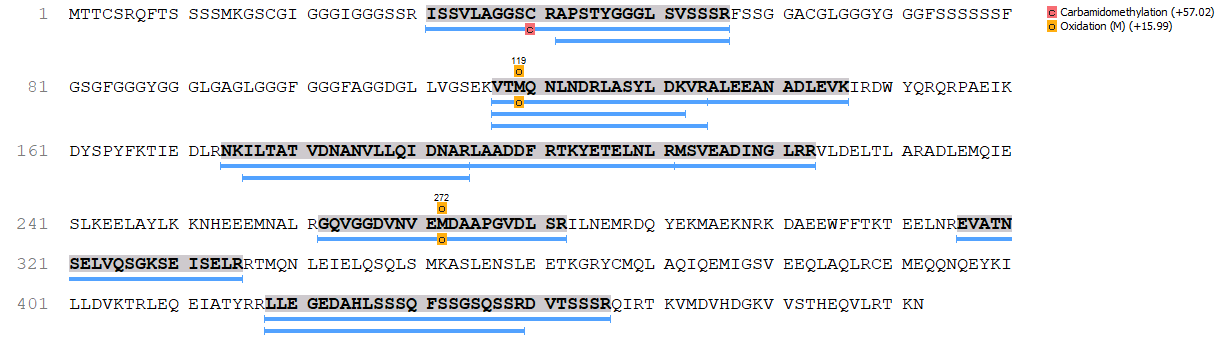

Supplement: Supplementary file 3 [file DataSheet2.ZIP › HTML/img/cov_35.png]
