# Supplementary material for: Stings on wings: Proteotranscriptomic and biochemical profiling of the lesser banded hornet (Vespa affinis) venom
Source: Front Mol Biosci. 2022 Dec 19;9:1066793. doi: 10.3389/fmolb.2022.1066793 (PMC9806352; doi:10.3389/fmolb.2022.1066793)
Supplement: Supplementary file 3 [file DataSheet2.ZIP › HTML/protein.html]

proteins


Summary

  

# 1. Notes

# 2. Result Statistics

**Figure 1.**
False discovery rate (FDR) curve. X axis is the number of peptide-spectrum matches (PSM) being kept. Y axis is the corresponding FDR.

  


  

**Figure 2.**
PSM score distribution.  **(a)**
Distribution of PEAKS peptide score;  **(b)**
Scatterplot of PEAKS peptide score versus precursor mass error.

**(a)**

**(b)**

**Table 1.**
Statistics of data.


|  |  |  |  |  |  |  |  |  |  |  |  |
| --- | --- | --- | --- | --- | --- | --- | --- | --- | --- | --- | --- |
|  | #Scans | | #Features | Identified | | | #Peptides | #Sequences | #Proteins^\*^ | | |
|  | MS1 | MS2 |  | #PSMs | #Scans | #Features |  |  | Groups | All | Top |
| Total | 60095 | 151182 | 402064 | 2796 | 4762 | 42503 | 726 | 697 | 114 | 432 | 266 |
| F1 | 8659 | 20273 | 53028 | 562 | 1117 | 6065 | 345 | 318 | 64 | 234 | 157 |
| F2 | 8294 | 20996 | 63277 | 349 | 584 | 6176 | 246 | 244 | 53 | 168 | 124 |
| F3 | 8263 | 21026 | 63726 | 366 | 618 | 6166 | 250 | 246 | 68 | 261 | 170 |
| F4 | 8227 | 21368 | 63822 | 353 | 594 | 5904 | 234 | 231 | 60 | 166 | 122 |
| F5 | 8275 | 21277 | 60371 | 327 | 546 | 5737 | 222 | 219 | 65 | 175 | 132 |
| F6 | 8186 | 21514 | 68290 | 427 | 655 | 7119 | 306 | 304 | 71 | 276 | 163 |
| F7 | 10191 | 24728 | 29550 | 412 | 648 | 5336 | 256 | 255 | 53 | 149 | 86 |

^\* proteins with significant peptides are used in counts.^

**Figure 3.**
Sample overlap for Proteins and Peptides (up to 8 samples).  **(a)**
All Proteins;  **(b)**
Top Proteins;  **(c)**
Peptides;

**(a)**

|  |  |  |  |  |  |  |  |  |  |  |  |  |  |  |  |  |  |  |  |  |  |  |  |  |  |  |  |  |  |  |  |  |  |  |  |  |  |  |  |  |  |  |  |  |  |  |  |  |  |  |  |  |  |  |  |  |  |  |  |  |  |  |  |  |  |  |  |  |  |  |  |  |
| --- | --- | --- | --- | --- | --- | --- | --- | --- | --- | --- | --- | --- | --- | --- | --- | --- | --- | --- | --- | --- | --- | --- | --- | --- | --- | --- | --- | --- | --- | --- | --- | --- | --- | --- | --- | --- | --- | --- | --- | --- | --- | --- | --- | --- | --- | --- | --- | --- | --- | --- | --- | --- | --- | --- | --- | --- | --- | --- | --- | --- | --- | --- | --- | --- | --- | --- | --- | --- | --- | --- | --- | --- |
|  | 1 | 2 | 3 | 4 | 5 | 6 | 7 | Unique | \|  \|  \|  \|  \|  \|  \|  \|  \|  \| \| --- \| --- \| --- \| --- \| --- \| --- \| --- \| --- \| --- \| \| 1 \| 234 \| 150 \| 168 \| 108 \| 105 \| 146 \| 63 \| 44 \| \| 2 \| 150 \| 168 \| 140 \| 108 \| 104 \| 136 \| 63 \| 8 \| \| 3 \| 168 \| 140 \| 261 \| 141 \| 134 \| 197 \| 120 \| 18 \| \| 4 \| 108 \| 108 \| 141 \| 166 \| 146 \| 137 \| 97 \| 3 \| \| 5 \| 105 \| 104 \| 134 \| 146 \| 175 \| 143 \| 99 \| 13 \| \| 6 \| 146 \| 136 \| 197 \| 137 \| 143 \| 276 \| 133 \| 46 \| \| 7 \| 63 \| 63 \| 120 \| 97 \| 99 \| 133 \| 149 \| 9 \| |

**(b)**

|  |  |  |  |  |  |  |  |  |  |  |  |  |  |  |  |  |  |  |  |  |  |  |  |  |  |  |  |  |  |  |  |  |  |  |  |  |  |  |  |  |  |  |  |  |  |  |  |  |  |  |  |  |  |  |  |  |  |  |  |  |  |  |  |  |  |  |  |  |  |  |  |  |
| --- | --- | --- | --- | --- | --- | --- | --- | --- | --- | --- | --- | --- | --- | --- | --- | --- | --- | --- | --- | --- | --- | --- | --- | --- | --- | --- | --- | --- | --- | --- | --- | --- | --- | --- | --- | --- | --- | --- | --- | --- | --- | --- | --- | --- | --- | --- | --- | --- | --- | --- | --- | --- | --- | --- | --- | --- | --- | --- | --- | --- | --- | --- | --- | --- | --- | --- | --- | --- | --- | --- | --- | --- |
|  | 1 | 2 | 3 | 4 | 5 | 6 | 7 | Unique | \|  \|  \|  \|  \|  \|  \|  \|  \|  \| \| --- \| --- \| --- \| --- \| --- \| --- \| --- \| --- \| --- \| \| 1 \| 157 \| 115 \| 118 \| 86 \| 82 \| 111 \| 48 \| 18 \| \| 2 \| 115 \| 124 \| 100 \| 85 \| 81 \| 102 \| 46 \| 6 \| \| 3 \| 118 \| 100 \| 170 \| 102 \| 93 \| 121 \| 67 \| 18 \| \| 4 \| 86 \| 85 \| 102 \| 122 \| 107 \| 100 \| 66 \| 1 \| \| 5 \| 82 \| 81 \| 93 \| 107 \| 132 \| 107 \| 70 \| 13 \| \| 6 \| 111 \| 102 \| 121 \| 100 \| 107 \| 163 \| 78 \| 13 \| \| 7 \| 48 \| 46 \| 67 \| 66 \| 70 \| 78 \| 86 \| 5 \| |

**(c)**

|  |  |  |  |  |  |  |  |  |  |  |  |  |  |  |  |  |  |  |  |  |  |  |  |  |  |  |  |  |  |  |  |  |  |  |  |  |  |  |  |  |  |  |  |  |  |  |  |  |  |  |  |  |  |  |  |  |  |  |  |  |  |  |  |  |  |  |  |  |  |  |  |  |
| --- | --- | --- | --- | --- | --- | --- | --- | --- | --- | --- | --- | --- | --- | --- | --- | --- | --- | --- | --- | --- | --- | --- | --- | --- | --- | --- | --- | --- | --- | --- | --- | --- | --- | --- | --- | --- | --- | --- | --- | --- | --- | --- | --- | --- | --- | --- | --- | --- | --- | --- | --- | --- | --- | --- | --- | --- | --- | --- | --- | --- | --- | --- | --- | --- | --- | --- | --- | --- | --- | --- | --- | --- |
|  | 1 | 2 | 3 | 4 | 5 | 6 | 7 | Unique | \|  \|  \|  \|  \|  \|  \|  \|  \|  \| \| --- \| --- \| --- \| --- \| --- \| --- \| --- \| --- \| --- \| \| 1 \| 345 \| 177 \| 130 \| 108 \| 104 \| 119 \| 72 \| 146 \| \| 2 \| 177 \| 246 \| 132 \| 116 \| 119 \| 126 \| 79 \| 37 \| \| 3 \| 130 \| 132 \| 250 \| 173 \| 161 \| 179 \| 127 \| 17 \| \| 4 \| 108 \| 116 \| 173 \| 234 \| 174 \| 173 \| 128 \| 13 \| \| 5 \| 104 \| 119 \| 161 \| 174 \| 222 \| 170 \| 123 \| 5 \| \| 6 \| 119 \| 126 \| 179 \| 173 \| 170 \| 306 \| 162 \| 51 \| \| 7 \| 72 \| 79 \| 127 \| 128 \| 123 \| 162 \| 256 \| 68 \| |

**Figure 4.**
Distribution of peptide feature detection.  **(a)**
Feature m/z distribution;  **(b)**
Feature RT distribution.

**(a)**

**(b)**

**Figure 5.**
Distribution of identified peptide features.  **(a)**
Feature abundance distribution;  **(b)**
 *De novo*
sequencing validation.

**(a)**

**(b)**

**Table 2.**
Result filtration parameters.

|  |  |
| --- | --- |
| Peptide -10lgP | ≥47.1 |
| PTM Ascore | ≥0 |
| Protein -10lgP | ≥20 |
| Proteins unique peptides | ≥1 |
| De novo score(%) | ≥50% |


**Table 3.**
Statistics of filtered result.

|  |  |
| --- | --- |
| FDR (Peptide-Spectrum Matches) | 0.1% |
| FDR (Peptide Sequences) | 0.3% |
| FDR (Protein Group) | 1.8% |
| De Novo Only Spectra | 39707 |

**Table 4.**
PTM profile.

| Name | ∆Mass | Position | #PSM | -10lgP | Abundance | AScore || Carbamidomethyl | 57.02 | C | 616 | 132.10 | 3.92E7 | 1000.00 |
| Oxidation | 15.99 | M | 279 | 121.69 | 1.47E6 | 1000.00 |

# 3. Experiment Control

**Figure 6.**
Precursor mass error of peptide-spectrum matches (PSM) in filtered result.  **(a)**
Distribution of precursor mass error in ppm;  **(b)**
Scatterplot of precursor m/z versus precursor mass error in ppm.

**(a)**

**(b)**

**Table 5.**
Number of identified peptides in each sample by the number of missed cleavages.

|  |  |  |  |  |  |
| --- | --- | --- | --- | --- | --- |
| Missed Cleavages | 0 | 1 | 2 | 3 | 4+ |
| F1 | 185 | 116 | 37 | 7 | 0 |
| F2 | 127 | 79 | 35 | 5 | 0 |
| F3 | 153 | 71 | 22 | 4 | 0 |
| F4 | 139 | 64 | 21 | 10 | 0 |
| F5 | 127 | 66 | 21 | 8 | 0 |
| F6 | 163 | 96 | 37 | 10 | 0 |
| F7 | 107 | 87 | 43 | 19 | 0 |

# 4. Other Information

**Table 6.**
Search parameters.

Search Engine Name: PEAKS  
Parent Mass Error Tolerance: 10.0 ppm  
Fragment Mass Error Tolerance: 0.6 Da  
Precursor Mass Search Type: monoisotopic  
Enzyme: Trypsin  
Max Missed Cleavages: 3  
Digest Mode: Specific  
Fixed Modifications:   
  Carbamidomethylation: 57.02  
Variable Modifications:   
  Oxidation (M): 15.99  
Max Variable PTM Per Peptide: 3  
Database: Vespa\_affinis\_transcritpome  
Taxon: All  
Contaminant Database: cRAP\_contaminants  
Searched Entry: 624618  
FDR Estimation: Enabled  
Merge Options: no merge  
Precursor Options: corrected  
Charge Options: no correction  
Filter Charge: 1 - 8  
Process: true  
Associate chimera: yes

**Table 7.**
Instrument parameters.

Fractions: Fraction\_1\_23092022r.raw, Fraction\_2\_23092022.raw, F  
raction\_3\_23092022.raw, Fraction\_4\_23092022.raw, Fraction\_5\_2  
3092022.raw, Fraction\_6\_23092022.raw, Fraction\_7\_23092022.raw  
  
Ion Source: ESI(nano-spray)  
Fragmentation Mode: CID, CAD(y and b ions)  
MS Scan Mode: FT-ICR/Orbitrap  
MS/MS Scan Mode: FT-ICR/Orbitrap

  

Protein List

  

Protein Accession Contains:

Protein Description Contains:

Protein Sample Area >=

Protein PTM Contains:

| Protein Group | Protein ID | Accession | -10lgP | Coverage (%) | Coverage (%) F1 | Coverage (%) F2 | Coverage (%) F3 | Coverage (%) F4 | Coverage (%) F5 | Coverage (%) F6 | Coverage (%) F7 | Area F1 | Area F2 | Area F3 | Area F4 | Area F5 | Area F6 | Area F7 | #Peptides | #Unique | #Spec F1 | #Spec F2 | #Spec F3 | #Spec F4 | #Spec F5 | #Spec F6 | #Spec F7 | PTM | Avg. Mass | Description |
| --- | --- | --- | --- | --- | --- | --- | --- | --- | --- | --- | --- | --- | --- | --- | --- | --- | --- | --- | --- | --- | --- | --- | --- | --- | --- | --- | --- | --- | --- | --- |
| 7 | 14 | TRINITY\_DN4276\_c0\_g1\_i1.p1 | 466.18 | 54 | 54 | 33 | 12 | 12 | 9 | 11 | 0 | 5.7395E9 | 1.1085E8 | 7.1889E6 | 2.8966E6 | 9.8604E5 | 2.9135E6 |  | 46 | 46 | 139 | 26 | 7 | 6 | 4 | 5 | 0 | Y | 68230 | TRINITY\_DN4276\_c0\_g1\_i1.p1 |
| 5 | 3 | P04264\|K2C1\_HUMAN | 432.97 | 68 | 63 | 44 | 35 | 29 | 28 | 36 | 50 | 1.2298E8 | 5.9443E7 | 3.2915E7 | 7.8656E6 | 1.0617E7 | 2.6521E7 | 1.8646E7 | 49 | 36 | 57 | 41 | 29 | 17 | 19 | 31 | 31 | Y | 66039 | Keratin, type II cytoskeletal 1 OS=Homo sapiens OX=9606 GN=KRT1 PE=1 SV=6 |
| 15 | 6 | TRINITY\_DN3450\_c0\_g1\_i51.p1 | 397.97 | 42 | 0 | 5 | 22 | 23 | 22 | 35 | 30 |  | 2.4924E5 | 1.0031E7 | 1.8774E7 | 1.7231E7 | 3.4929E7 | 1.9918E8 | 33 | 33 | 0 | 2 | 16 | 17 | 16 | 29 | 40 | Y | 79620 | TRINITY\_DN3450\_c0\_g1\_i51.p1 |
| 16 | 12 | P35527\|K1C9\_HUMAN | 391.04 | 53 | 38 | 27 | 24 | 17 | 24 | 25 | 32 | 8.9107E7 | 3.9133E7 | 2.1577E7 | 3.6639E6 | 3.5838E6 | 1.9465E7 | 1.3658E7 | 24 | 24 | 28 | 15 | 12 | 7 | 8 | 13 | 12 | Y | 62064 | Keratin, type I cytoskeletal 9 OS=Homo sapiens OX=9606 GN=KRT9 PE=1 SV=3 |
| 12 | 16 | P13645\|K1C10\_HUMAN | 382.92 | 48 | 48 | 42 | 39 | 37 | 34 | 38 | 21 | 9.6056E7 | 2.9579E7 | 3.6067E7 | 7.0729E6 | 9.4737E6 | 2.2275E7 | 1.6945E7 | 27 | 16 | 40 | 21 | 23 | 15 | 15 | 16 | 11 | Y | 58827 | Keratin, type I cytoskeletal 10 OS=Homo sapiens OX=9606 GN=KRT10 PE=1 SV=6 |
| 13 | 2 | P35908\|K22E\_HUMAN | 374.63 | 62 | 56 | 41 | 29 | 22 | 27 | 28 | 27 | 5.284E6 | 2.7455E6 | 5.8629E6 | 1.3454E6 | 1.7026E6 | 5.2095E6 | 3.742E6 | 28 | 1 | 31 | 20 | 18 | 12 | 14 | 15 | 12 | Y | 65433 | Keratin, type II cytoskeletal 2 epidermal OS=Homo sapiens OX=9606 GN=KRT2 PE=1 SV=2 |
| 11 | 21 | TRINITY\_DN2326\_c0\_g1\_i12.p1 | 365.86 | 61 | 4 | 13 | 57 | 52 | 50 | 60 | 44 |  | 2.0625E5 | 9.6952E6 | 1.3312E7 |  | 5.3826E6 | 1.6292E7 | 24 | 5 | 1 | 3 | 30 | 25 | 25 | 21 | 27 | Y | 37536 | TRINITY\_DN2326\_c0\_g1\_i12.p1 |
| 18 | 51 | TRINITY\_DN2326\_c0\_g1\_i13.p1 | 350.79 | 54 | 10 | 9 | 37 | 38 | 37 | 44 | 54 | 3.8953E5 | 3.6794E5 | 3.1909E7 | 9.6099E7 | 6.6022E7 | 2.0947E8 | 2.9856E8 | 16 | 14 | 2 | 2 | 14 | 17 | 15 | 20 | 20 | Y | 32507 | TRINITY\_DN2326\_c0\_g1\_i13.p1 |
| 9 | 39 | A0A0M3KKW3\|PA1\_VESBA | 348.32 | 42 | 20 | 17 | 32 | 26 | 26 | 29 | 37 |  |  |  |  |  |  | 4.2338E5 | 20 | 1 | 5 | 8 | 32 | 30 | 18 | 15 | 28 | Y | 33185 | Phospholipase A1 OS=Vespa basalis OX=7444 PE=1 SV=1 |
| 1 | 45 | P00760\|TRY1\_BOVIN | 341.01 | 64 | 64 | 52 | 56 | 56 | 62 | 57 | 39 | 4.322E10 | 1.7653E10 | 7.9511E9 | 1.731E10 | 1.7866E10 | 1.9134E10 | 1.1791E10 | 14 | 14 | 81 | 34 | 35 | 45 | 46 | 41 | 51 | Y | 25785 | Serine protease 1 OS=Bos taurus OX=9913 GN=PRSS1 PE=1 SV=3 |
| 20 | 61 | TRINITY\_DN6370\_c0\_g1\_i1.p1 | 337.99 | 47 | 0 | 0 | 9 | 31 | 12 | 40 | 43 |  |  | 3.9637E6 | 9.1069E7 | 6.8703E6 | 7.1254E7 | 1.0412E9 | 17 | 15 | 0 | 0 | 3 | 14 | 6 | 19 | 30 | Y | 25005 | TRINITY\_DN6370\_c0\_g1\_i1.p1 |
| 25 | 19 | TRINITY\_DN10047\_c0\_g1\_i1.p1 | 329.91 | 34 | 28 | 32 | 10 | 10 | 6 | 8 | 0 | 4.9368E7 | 3.1714E8 | 6.1376E6 | 2.777E6 | 1.3144E6 | 5.5243E6 |  | 16 | 16 | 17 | 25 | 5 | 3 | 2 | 3 | 0 | Y | 64848 | TRINITY\_DN10047\_c0\_g1\_i1.p1 |
| 19 | 54 | TRINITY\_DN33420\_c0\_g1\_i1.p1 | 326.68 | 57 | 10 | 10 | 44 | 29 | 36 | 53 | 42 | 7.5466E5 | 5.7663E5 | 1.4846E8 | 7.4504E6 | 1.6152E7 | 6.3741E7 | 1.2535E8 | 14 | 14 | 3 | 2 | 21 | 6 | 11 | 16 | 17 | Y | 29157 | TRINITY\_DN33420\_c0\_g1\_i1.p1 |
| 36 | 43 | TRINITY\_DN11986\_c0\_g1\_i3.p1 | 309.84 | 28 | 28 | 19 | 5 | 10 | 0 | 7 | 3 | 1.2819E7 | 2.1133E7 | 1.4783E6 | 1.6436E6 |  | 1.6674E6 | 6.3833E5 | 13 | 13 | 11 | 13 | 2 | 4 | 0 | 3 | 1 | N | 72596 | TRINITY\_DN11986\_c0\_g1\_i3.p1 |
| 17 | 99 | C0HLL3\|PA1\_VESVE | 301.21 | 35 | 4 | 14 | 30 | 30 | 34 | 27 | 35 |  |  |  |  | 7.6272E5 |  | 7.6637E5 | 11 | 1 | 1 | 3 | 22 | 21 | 14 | 12 | 17 | Y | 33957 | Phospholipase A1 OS=Vespa velutina OX=202808 PE=1 SV=1 |
| 23 | 25 | P48668\|K2C6C\_HUMAN | 284.54 | 28 | 22 | 18 | 15 | 8 | 12 | 18 | 8 | 4.9517E4 | 2.2919E5 |  |  | 7.3699E5 | 2.0061E5 |  | 16 | 3 | 10 | 11 | 10 | 5 | 8 | 10 | 3 | Y | 60025 | Keratin, type II cytoskeletal 6C OS=Homo sapiens OX=9606 GN=KRT6C PE=1 SV=3 |
| 34 | 62 | TRINITY\_DN2611\_c0\_g1\_i12.p1 | 273.59 | 25 | 6 | 25 | 3 | 3 | 3 | 11 | 0 | 1.4292E6 | 1.0602E8 | 2.4161E5 | 2.8049E5 | 9.7567E5 | 1.8228E7 |  | 14 | 14 | 3 | 18 | 1 | 1 | 2 | 7 | 0 | Y | 50314 | TRINITY\_DN2611\_c0\_g1\_i12.p1 |
| 34 | 63 | TRINITY\_DN2611\_c0\_g1\_i23.p1 | 273.59 | 25 | 6 | 25 | 3 | 3 | 3 | 11 | 0 | 1.4292E6 | 1.0602E8 | 2.4161E5 | 2.8049E5 | 9.7567E5 | 1.8228E7 |  | 14 | 14 | 3 | 18 | 1 | 1 | 2 | 7 | 0 | Y | 50314 | TRINITY\_DN2611\_c0\_g1\_i23.p1 |
| 34 | 64 | TRINITY\_DN2611\_c0\_g1\_i19.p1 | 273.59 | 25 | 6 | 25 | 3 | 3 | 3 | 11 | 0 | 1.4292E6 | 1.0602E8 | 2.4161E5 | 2.8049E5 | 9.7567E5 | 1.8228E7 |  | 14 | 14 | 3 | 18 | 1 | 1 | 2 | 7 | 0 | Y | 50314 | TRINITY\_DN2611\_c0\_g1\_i19.p1 |
| 34 | 65 | TRINITY\_DN2611\_c0\_g1\_i9.p1 | 273.59 | 25 | 6 | 25 | 3 | 3 | 3 | 11 | 0 | 1.4292E6 | 1.0602E8 | 2.4161E5 | 2.8049E5 | 9.7567E5 | 1.8228E7 |  | 14 | 14 | 3 | 18 | 1 | 1 | 2 | 7 | 0 | Y | 50314 | TRINITY\_DN2611\_c0\_g1\_i9.p1 |
| 42 | 35 | P02533\|K1C14\_HUMAN | 269.91 | 37 | 36 | 12 | 7 | 3 | 3 | 6 | 6 | 2.755E6 | 8.5264E4 | 8.4193E4 |  |  | 1.0711E5 | 3.6841E5 | 13 | 8 | 15 | 3 | 2 | 1 | 1 | 2 | 1 | Y | 51562 | Keratin, type I cytoskeletal 14 OS=Homo sapiens OX=9606 GN=KRT14 PE=1 SV=4 |
| 48 | 57 | TRINITY\_DN13586\_c0\_g2\_i1.p1 | 259.38 | 34 | 0 | 0 | 0 | 5 | 5 | 23 | 21 |  |  |  | 9.9343E4 | 2.2425E5 | 2.5682E7 | 3.1191E8 | 14 | 14 | 0 | 0 | 0 | 1 | 1 | 7 | 16 | N | 30071 | TRINITY\_DN13586\_c0\_g2\_i1.p1 |
| 45 | 68 | TRINITY\_DN10256\_c0\_g10\_i1.p1 | 254.07 | 18 | 4 | 18 | 8 | 8 | 8 | 5 | 0 | 6.9453E5 | 4.3876E7 | 1.6248E6 | 2.1132E6 | 2.7447E6 | 4.5374E5 |  | 11 | 11 | 2 | 12 | 4 | 4 | 5 | 2 | 0 | Y | 67221 | TRINITY\_DN10256\_c0\_g10\_i1.p1 |
| 45 | 69 | TRINITY\_DN10256\_c0\_g5\_i4.p1 | 254.07 | 18 | 4 | 18 | 8 | 8 | 8 | 5 | 0 | 6.9453E5 | 4.3876E7 | 1.6248E6 | 2.1132E6 | 2.7447E6 | 4.5374E5 |  | 11 | 11 | 2 | 12 | 4 | 4 | 5 | 2 | 0 | Y | 67221 | TRINITY\_DN10256\_c0\_g5\_i4.p1 |
| 47 | 26 | TRINITY\_DN1113\_c0\_g1\_i95.p1 | 250.96 | 17 | 0 | 0 | 2 | 14 | 7 | 6 | 1 |  |  | 8.706E5 | 6.6881E6 | 3.9439E6 | 1.79E7 | 2.1683E6 | 11 | 11 | 0 | 0 | 1 | 11 | 6 | 5 | 1 | Y | 107782 | TRINITY\_DN1113\_c0\_g1\_i95.p1 |
| 47 | 27 | TRINITY\_DN1113\_c0\_g1\_i47.p1 | 250.96 | 16 | 0 | 0 | 1 | 13 | 7 | 6 | 1 |  |  | 8.706E5 | 6.6881E6 | 3.9439E6 | 1.79E7 | 2.1683E6 | 11 | 11 | 0 | 0 | 1 | 11 | 6 | 5 | 1 | Y | 113322 | TRINITY\_DN1113\_c0\_g1\_i47.p1 |
| 47 | 28 | TRINITY\_DN1113\_c0\_g1\_i59.p1 | 250.96 | 16 | 0 | 0 | 1 | 13 | 7 | 6 | 1 |  |  | 8.706E5 | 6.6881E6 | 3.9439E6 | 1.79E7 | 2.1683E6 | 11 | 11 | 0 | 0 | 1 | 11 | 6 | 5 | 1 | Y | 113322 | TRINITY\_DN1113\_c0\_g1\_i59.p1 |
| 47 | 29 | TRINITY\_DN1113\_c0\_g1\_i53.p1 | 250.96 | 16 | 0 | 0 | 1 | 13 | 7 | 6 | 1 |  |  | 8.706E5 | 6.6881E6 | 3.9439E6 | 1.79E7 | 2.1683E6 | 11 | 11 | 0 | 0 | 1 | 11 | 6 | 5 | 1 | Y | 113322 | TRINITY\_DN1113\_c0\_g1\_i53.p1 |
| 47 | 30 | TRINITY\_DN1113\_c0\_g1\_i73.p1 | 250.96 | 16 | 0 | 0 | 1 | 13 | 7 | 6 | 1 |  |  | 8.706E5 | 6.6881E6 | 3.9439E6 | 1.79E7 | 2.1683E6 | 11 | 11 | 0 | 0 | 1 | 11 | 6 | 5 | 1 | Y | 113322 | TRINITY\_DN1113\_c0\_g1\_i73.p1 |
| 47 | 31 | TRINITY\_DN1113\_c0\_g1\_i6.p1 | 250.96 | 16 | 0 | 0 | 1 | 13 | 7 | 6 | 1 |  |  | 8.706E5 | 6.6881E6 | 3.9439E6 | 1.79E7 | 2.1683E6 | 11 | 11 | 0 | 0 | 1 | 11 | 6 | 5 | 1 | Y | 113322 | TRINITY\_DN1113\_c0\_g1\_i6.p1 |
| 47 | 32 | TRINITY\_DN1113\_c0\_g1\_i12.p1 | 250.96 | 16 | 0 | 0 | 1 | 13 | 7 | 6 | 1 |  |  | 8.706E5 | 6.6881E6 | 3.9439E6 | 1.79E7 | 2.1683E6 | 11 | 11 | 0 | 0 | 1 | 11 | 6 | 5 | 1 | Y | 113322 | TRINITY\_DN1113\_c0\_g1\_i12.p1 |
| 28 | 50 | TRINITY\_DN1342\_c0\_g1\_i29.p1 | 248.40 | 17 | 11 | 17 | 7 | 6 | 9 | 4 | 2 | 1.0775E6 | 1.6797E7 | 4.1487E5 | 1.2391E5 | 1.2335E5 |  |  | 12 | 3 | 7 | 15 | 3 | 3 | 7 | 4 | 1 | Y | 68886 | TRINITY\_DN1342\_c0\_g1\_i29.p1 |
| 59 | 52 | TRINITY\_DN2373\_c0\_g1\_i19.p1 | 248.03 | 12 | 1 | 6 | 6 | 1 | 5 | 2 | 4 | 1.3169E5 | 4.046E6 | 8.0193E6 | 3.7341E5 | 3.538E5 | 5.2852E5 | 1.6028E7 | 10 | 10 | 1 | 6 | 4 | 1 | 3 | 1 | 4 | Y | 93403 | TRINITY\_DN2373\_c0\_g1\_i19.p1 |
| 21 | 40 | TRINITY\_DN2228\_c0\_g4\_i1.p1 | 246.57 | 30 | 3 | 0 | 12 | 14 | 14 | 23 | 18 | 1.8277E5 |  | 2.0485E7 | 2.0768E7 | 8.7511E7 | 2.5544E8 | 2.7676E7 | 15 | 14 | 1 | 0 | 8 | 8 | 11 | 18 | 13 | Y | 42077 | TRINITY\_DN2228\_c0\_g4\_i1.p1 |
| 22 | 73 | TRINITY\_DN1342\_c0\_g1\_i23.p1 | 246.03 | 15 | 12 | 15 | 5 | 7 | 10 | 4 | 2 | 5.9609E6 | 3.1555E8 |  | 1.1258E6 | 6.8537E6 |  |  | 13 | 5 | 9 | 24 | 2 | 3 | 11 | 4 | 1 | Y | 68697 | TRINITY\_DN1342\_c0\_g1\_i23.p1 |
| 22 | 74 | TRINITY\_DN1342\_c0\_g1\_i54.p1 | 246.03 | 15 | 12 | 15 | 5 | 7 | 10 | 4 | 2 | 5.9609E6 | 3.1555E8 |  | 1.1258E6 | 6.8537E6 |  |  | 13 | 5 | 9 | 24 | 2 | 3 | 11 | 4 | 1 | Y | 68697 | TRINITY\_DN1342\_c0\_g1\_i54.p1 |
| 52 | 134 | TRINITY\_DN2925\_c0\_g1\_i5.p1 | 240.08 | 18 | 0 | 0 | 6 | 15 | 9 | 10 | 4 |  |  | 4.1343E6 | 3.8018E7 | 2.7696E7 | 4.4064E6 | 8.4556E6 | 6 | 6 | 0 | 0 | 3 | 9 | 6 | 4 | 4 | Y | 66119 | TRINITY\_DN2925\_c0\_g1\_i5.p1 |
| 27 | 24 | P13647\|K2C5\_HUMAN | 235.04 | 28 | 26 | 16 | 11 | 10 | 8 | 11 | 4 | 1.6098E6 | 1.3881E6 | 1.1684E6 | 2.0829E5 | 3.3682E4 | 7.8008E4 |  | 15 | 4 | 13 | 11 | 8 | 6 | 5 | 7 | 2 | Y | 62378 | Keratin, type II cytoskeletal 5 OS=Homo sapiens OX=9606 GN=KRT5 PE=1 SV=3 |
| 35 | 60 | TRINITY\_DN1342\_c0\_g1\_i9.p1 | 234.40 | 19 | 14 | 19 | 7 | 6 | 9 | 5 | 2 | 1.2315E6 | 8.1127E5 |  |  |  |  |  | 10 | 1 | 8 | 12 | 2 | 2 | 6 | 4 | 1 | Y | 52685 | TRINITY\_DN1342\_c0\_g1\_i9.p1 |
| 61 | 89 | TRINITY\_DN1709\_c0\_g1\_i4.p1 | 228.01 | 6 | 6 | 3 | 0 | 0 | 0 | 0 | 0 | 1.5335E7 | 1.093E6 |  |  |  |  |  | 9 | 9 | 13 | 4 | 0 | 0 | 0 | 0 | 0 | Y | 107097 | TRINITY\_DN1709\_c0\_g1\_i4.p1 |
| 61 | 90 | TRINITY\_DN1709\_c0\_g1\_i5.p1 | 228.01 | 6 | 6 | 3 | 0 | 0 | 0 | 0 | 0 | 1.5335E7 | 1.093E6 |  |  |  |  |  | 9 | 9 | 13 | 4 | 0 | 0 | 0 | 0 | 0 | Y | 107097 | TRINITY\_DN1709\_c0\_g1\_i5.p1 |
| 61 | 91 | TRINITY\_DN1709\_c0\_g1\_i6.p1 | 228.01 | 6 | 6 | 3 | 0 | 0 | 0 | 0 | 0 | 1.5335E7 | 1.093E6 |  |  |  |  |  | 9 | 9 | 13 | 4 | 0 | 0 | 0 | 0 | 0 | Y | 107097 | TRINITY\_DN1709\_c0\_g1\_i6.p1 |
| 61 | 92 | TRINITY\_DN1709\_c0\_g1\_i8.p1 | 228.01 | 6 | 6 | 3 | 0 | 0 | 0 | 0 | 0 | 1.5335E7 | 1.093E6 |  |  |  |  |  | 9 | 9 | 13 | 4 | 0 | 0 | 0 | 0 | 0 | Y | 107097 | TRINITY\_DN1709\_c0\_g1\_i8.p1 |
| 64 | 41 | P02768\|ALBU\_HUMAN | 221.81 | 19 | 19 | 4 | 2 | 0 | 2 | 0 | 0 | 2.182E6 | 1.6952E5 |  |  |  |  |  | 9 | 6 | 10 | 2 | 2 | 0 | 1 | 0 | 0 | Y | 69367 | Albumin OS=Homo sapiens OX=9606 GN=ALB PE=1 SV=2 |
| 46 | 126 | P0AA29\|THIO\_SALTI | 220.48 | 63 | 63 | 63 | 45 | 42 | 0 | 0 | 0 | 1.4379E7 | 2.1499E7 | 9.7101E5 | 2.5096E6 |  |  |  | 8 | 8 | 9 | 11 | 3 | 4 | 0 | 0 | 0 | Y | 11807 | Thioredoxin 1 OS=Salmonella typhi OX=90370 GN=trxA PE=1 SV=2 |
| 46 | 127 | P0AA26\|THIO\_ECOL6 | 220.48 | 63 | 63 | 63 | 45 | 42 | 0 | 0 | 0 | 1.4379E7 | 2.1499E7 | 9.7101E5 | 2.5096E6 |  |  |  | 8 | 8 | 9 | 11 | 3 | 4 | 0 | 0 | 0 | Y | 11807 | Thioredoxin 1 OS=Escherichia coli O6:H1 (strain CFT073 / ATCC 700928 / UPEC) OX=199310 GN=trxA PE=3 SV=2 |
| 46 | 128 | P0AA27\|THIO\_ECO57 | 220.48 | 63 | 63 | 63 | 45 | 42 | 0 | 0 | 0 | 1.4379E7 | 2.1499E7 | 9.7101E5 | 2.5096E6 |  |  |  | 8 | 8 | 9 | 11 | 3 | 4 | 0 | 0 | 0 | Y | 11807 | Thioredoxin 1 OS=Escherichia coli O157:H7 OX=83334 GN=trxA PE=1 SV=2 |
| 46 | 129 | P0AA30\|THIO\_SHIFL | 220.48 | 63 | 63 | 63 | 45 | 42 | 0 | 0 | 0 | 1.4379E7 | 2.1499E7 | 9.7101E5 | 2.5096E6 |  |  |  | 8 | 8 | 9 | 11 | 3 | 4 | 0 | 0 | 0 | Y | 11807 | Thioredoxin 1 OS=Shigella flexneri OX=623 GN=trxA PE=3 SV=2 |
| 46 | 130 | P0AA25\|THIO\_ECOLI | 220.48 | 63 | 63 | 63 | 45 | 42 | 0 | 0 | 0 | 1.4379E7 | 2.1499E7 | 9.7101E5 | 2.5096E6 |  |  |  | 8 | 8 | 9 | 11 | 3 | 4 | 0 | 0 | 0 | Y | 11807 | Thioredoxin 1 OS=Escherichia coli (strain K12) OX=83333 GN=trxA PE=1 SV=2 |
| 46 | 131 | P0AA28\|THIO\_SALTY | 220.48 | 63 | 63 | 63 | 45 | 42 | 0 | 0 | 0 | 1.4379E7 | 2.1499E7 | 9.7101E5 | 2.5096E6 |  |  |  | 8 | 8 | 9 | 11 | 3 | 4 | 0 | 0 | 0 | Y | 11807 | Thioredoxin 1 OS=Salmonella typhimurium (strain LT2 / SGSC1412 / ATCC 700720) OX=99287 GN=trxA PE=1 SV=2 |
| 75 | 213 | TRINITY\_DN1494\_c0\_g1\_i8.p1 | 220.12 | 15 | 0 | 0 | 11 | 10 | 4 | 0 | 2 |  |  | 1.9487E7 | 7.6098E5 | 1.0534E6 |  | 6.8673E5 | 5 | 5 | 0 | 0 | 6 | 2 | 1 | 0 | 1 | Y | 46901 | TRINITY\_DN1494\_c0\_g1\_i8.p1 |
| 50 | 58 | P08779\|K1C16\_HUMAN | 215.05 | 20 | 15 | 12 | 7 | 3 | 3 | 5 | 5 | 6.5142E5 | 1.265E5 | 2.1668E5 |  |  | 1.0062E5 | 1.6022E5 | 7 | 4 | 9 | 3 | 3 | 1 | 1 | 2 | 1 | Y | 51268 | Keratin, type I cytoskeletal 16 OS=Homo sapiens OX=9606 GN=KRT16 PE=1 SV=4 |
| 76 | 117 | TRINITY\_DN1931\_c0\_g1\_i1.p1 | 208.57 | 53 | 0 | 0 | 0 | 0 | 0 | 53 | 0 |  |  |  |  |  | 5.2059E6 |  | 7 | 7 | 0 | 0 | 0 | 0 | 0 | 11 | 0 | Y | 16125 | TRINITY\_DN1931\_c0\_g1\_i1.p1 |
| 76 | 118 | TRINITY\_DN1931\_c0\_g1\_i2.p1 | 208.57 | 53 | 0 | 0 | 0 | 0 | 0 | 53 | 0 |  |  |  |  |  | 5.2059E6 |  | 7 | 7 | 0 | 0 | 0 | 0 | 0 | 11 | 0 | Y | 16125 | TRINITY\_DN1931\_c0\_g1\_i2.p1 |
| 76 | 119 | TRINITY\_DN1931\_c0\_g1\_i4.p1 | 208.57 | 53 | 0 | 0 | 0 | 0 | 0 | 53 | 0 |  |  |  |  |  | 5.2059E6 |  | 7 | 7 | 0 | 0 | 0 | 0 | 0 | 11 | 0 | Y | 16125 | TRINITY\_DN1931\_c0\_g1\_i4.p1 |
| 40 | 59 | TRINITY\_DN1342\_c0\_g1\_i34.p1 | 200.62 | 15 | 7 | 13 | 5 | 4 | 7 | 6 | 2 |  |  |  |  |  | 7.1972E4 |  | 9 | 1 | 4 | 10 | 2 | 2 | 6 | 5 | 1 | Y | 67118 | TRINITY\_DN1342\_c0\_g1\_i34.p1 |
| 68 | 71 | P02769\|ALBU\_BOVIN | 193.51 | 14 | 11 | 8 | 5 | 0 | 2 | 0 | 0 | 2.0794E6 | 8.381E5 | 9.9163E5 |  |  |  |  | 7 | 4 | 6 | 3 | 3 | 0 | 1 | 0 | 0 | N | 69294 | Albumin OS=Bos taurus OX=9913 GN=ALB PE=1 SV=4 |
| 58 | 137 | Q3TTY5\|K22E\_MOUSE | 187.43 | 9 | 4 | 2 | 2 | 4 | 1 | 2 | 7 |  |  |  | 9.4701E4 |  |  | 5.2227E5 | 6 | 1 | 4 | 4 | 3 | 3 | 2 | 3 | 3 | N | 70923 | Keratin, type II cytoskeletal 2 epidermal OS=Mus musculus OX=10090 GN=Krt2 PE=1 SV=1 |
| 74 | 86 | TRINITY\_DN1455\_c0\_g1\_i1.p1 | 182.77 | 8 | 0 | 8 | 3 | 3 | 3 | 0 | 0 |  | 1.0287E6 | 5.8376E5 | 6.0627E4 | 2.3932E5 |  |  | 4 | 4 | 0 | 4 | 2 | 1 | 2 | 0 | 0 | N | 78639 | TRINITY\_DN1455\_c0\_g1\_i1.p1 |
| 70 | 139 | TRINITY\_DN5849\_c0\_g1\_i1.p1 | 182.48 | 13 | 0 | 0 | 4 | 4 | 4 | 13 | 9 |  |  | 8.7264E5 | 7.3854E5 | 4.5131E5 | 1.2285E7 | 1.3845E6 | 4 | 4 | 0 | 0 | 1 | 1 | 1 | 6 | 2 | Y | 35599 | TRINITY\_DN5849\_c0\_g1\_i1.p1 |
| 70 | 140 | TRINITY\_DN5849\_c0\_g1\_i2.p1 | 182.48 | 13 | 0 | 0 | 4 | 4 | 4 | 13 | 9 |  |  | 8.7264E5 | 7.3854E5 | 4.5131E5 | 1.2285E7 | 1.3845E6 | 4 | 4 | 0 | 0 | 1 | 1 | 1 | 6 | 2 | Y | 35599 | TRINITY\_DN5849\_c0\_g1\_i2.p1 |
| 70 | 141 | TRINITY\_DN5849\_c0\_g1\_i10.p1 | 182.48 | 13 | 0 | 0 | 4 | 4 | 4 | 13 | 9 |  |  | 8.7264E5 | 7.3854E5 | 4.5131E5 | 1.2285E7 | 1.3845E6 | 4 | 4 | 0 | 0 | 1 | 1 | 1 | 6 | 2 | Y | 35599 | TRINITY\_DN5849\_c0\_g1\_i10.p1 |
| 72 | 112 | TRINITY\_DN36831\_c0\_g1\_i1.p1 | 180.75 | 37 | 0 | 0 | 11 | 37 | 16 | 14 | 0 |  |  | 3.993E5 | 1.4579E6 | 1.6147E5 | 2.9245E5 |  | 6 | 6 | 0 | 0 | 2 | 6 | 2 | 2 | 0 | Y | 21424 | TRINITY\_DN36831\_c0\_g1\_i1.p1 |
| 103 | 228 | TRINITY\_DN36822\_c0\_g1\_i1.p1 | 177.27 | 34 | 0 | 0 | 0 | 0 | 0 | 0 | 34 |  |  |  |  |  |  | 2.8606E6 | 4 | 4 | 0 | 0 | 0 | 0 | 0 | 0 | 4 | Y | 16985 | TRINITY\_DN36822\_c0\_g1\_i1.p1 |
| 95 | 184 | TRINITY\_DN5723\_c0\_g1\_i5.p1 | 173.87 | 18 | 0 | 0 | 0 | 0 | 0 | 18 | 0 |  |  |  |  |  | 4.2593E6 |  | 4 | 4 | 0 | 0 | 0 | 0 | 0 | 4 | 0 | Y | 39273 | TRINITY\_DN5723\_c0\_g1\_i5.p1 |
| 95 | 192 | TRINITY\_DN5723\_c0\_g1\_i7.p1 | 173.87 | 20 | 0 | 0 | 0 | 0 | 0 | 20 | 0 |  |  |  |  |  | 4.2593E6 |  | 4 | 4 | 0 | 0 | 0 | 0 | 0 | 4 | 0 | Y | 36626 | TRINITY\_DN5723\_c0\_g1\_i7.p1 |
| 65 | 173 | TRINITY\_DN9761\_c0\_g1\_i1.p1 | 171.34 | 29 | 10 | 29 | 14 | 10 | 14 | 0 | 0 |  | 1.7185E5 |  |  |  |  |  | 4 | 1 | 1 | 6 | 1 | 1 | 4 | 0 | 0 | N | 18606 | TRINITY\_DN9761\_c0\_g1\_i1.p1 |
| 83 | 182 | TRINITY\_DN594\_c1\_g4\_i5.p1 | 170.39 | 20 | 0 | 0 | 0 | 20 | 20 | 0 | 0 |  |  |  | 1.913E6 | 1.3916E6 |  |  | 3 | 3 | 0 | 0 | 0 | 3 | 4 | 0 | 0 | Y | 23117 | TRINITY\_DN594\_c1\_g4\_i5.p1 |
| 83 | 183 | TRINITY\_DN594\_c1\_g4\_i1.p1 | 170.39 | 13 | 0 | 0 | 0 | 13 | 13 | 0 | 0 |  |  |  | 1.913E6 | 1.3916E6 |  |  | 3 | 3 | 0 | 0 | 0 | 3 | 4 | 0 | 0 | Y | 35842 | TRINITY\_DN594\_c1\_g4\_i1.p1 |
| 88 | 132 | Q02413\|DSG1\_HUMAN | 169.01 | 6 | 6 | 0 | 0 | 0 | 0 | 0 | 0 | 1.6772E6 |  |  |  |  |  |  | 4 | 4 | 4 | 0 | 0 | 0 | 0 | 0 | 0 | Y | 113748 | Desmoglein-1 OS=Homo sapiens OX=9606 GN=DSG1 PE=1 SV=2 |
| 53 | 187 | P05787\|K2C8\_HUMAN | 167.78 | 13 | 13 | 13 | 5 | 2 | 4 | 5 | 5 | 3.4882E5 | 1.2177E5 |  |  |  |  |  | 4 | 1 | 4 | 6 | 4 | 2 | 3 | 4 | 2 | N | 53704 | Keratin, type II cytoskeletal 8 OS=Homo sapiens OX=9606 GN=KRT8 PE=1 SV=7 |
| 63 | 359 | P35782\|VA52\_VESCR | 165.93 | 18 | 0 | 0 | 0 | 10 | 0 | 11 | 18 |  |  |  | 4.1891E6 |  | 2.3776E6 | 1.7805E8 | 5 | 3 | 0 | 0 | 0 | 2 | 0 | 3 | 8 | N | 22781 | Venom allergen 5.02 OS=Vespa crabro OX=7445 PE=1 SV=1 |
| 63 | 360 | P35781\|VA51\_VESCR | 165.93 | 18 | 0 | 0 | 0 | 10 | 0 | 11 | 18 |  |  |  | 4.1891E6 |  | 2.3776E6 | 1.7805E8 | 5 | 3 | 0 | 0 | 0 | 2 | 0 | 3 | 8 | N | 22722 | Venom allergen 5.01 OS=Vespa crabro OX=7445 PE=1 SV=1 |
| 62 | 95 | TRINITY\_DN2228\_c0\_g2\_i2.p1 | 165.68 | 14 | 0 | 0 | 4 | 7 | 4 | 11 | 6 |  |  | 1.2245E6 | 2.133E6 | 5.7715E5 | 5.8809E7 | 1.5452E7 | 6 | 6 | 0 | 0 | 1 | 2 | 1 | 5 | 4 | Y | 36957 | TRINITY\_DN2228\_c0\_g2\_i2.p1 |
| 62 | 97 | TRINITY\_DN2228\_c0\_g2\_i1.p1 | 165.68 | 12 | 0 | 0 | 4 | 6 | 4 | 9 | 6 |  |  | 1.2245E6 | 2.133E6 | 5.7715E5 | 5.8809E7 | 1.5452E7 | 6 | 6 | 0 | 0 | 1 | 2 | 1 | 5 | 4 | Y | 41936 | TRINITY\_DN2228\_c0\_g2\_i1.p1 |
| 49 | 229 | P81605\|DCD\_HUMAN | 165.60 | 43 | 32 | 32 | 25 | 36 | 36 | 24 | 36 | 3.6876E6 | 1.92E6 | 1.1022E6 | 2.4439E6 | 1.4849E6 | 9.6352E5 | 3.5122E6 | 4 | 4 | 5 | 4 | 3 | 5 | 4 | 3 | 3 | N | 11284 | Dermcidin OS=Homo sapiens OX=9606 GN=DCD PE=1 SV=2 |
| 80 | 694 | TRINITY\_DN33395\_c0\_g1\_i1.p1 | 162.14 | 21 | 0 | 0 | 0 | 0 | 11 | 21 | 11 |  |  |  |  | 6.1162E4 | 1.2723E7 | 2.5083E6 | 5 | 5 | 0 | 0 | 0 | 0 | 1 | 6 | 3 | N | 14620 | TRINITY\_DN33395\_c0\_g1\_i1.p1 |
| 77 | 175 | TRINITY\_DN2938\_c0\_g1\_i1.p1 | 159.60 | 18 | 0 | 0 | 18 | 5 | 5 | 18 | 5 |  |  | 4.1982E6 | 9.1105E4 | 1.6627E5 | 2.1443E6 | 6.6382E5 | 4 | 4 | 0 | 0 | 4 | 1 | 1 | 4 | 1 | Y | 31741 | TRINITY\_DN2938\_c0\_g1\_i1.p1 |
| 86 | 135 | TRINITY\_DN273\_c0\_g4\_i11.p1 | 158.94 | 18 | 15 | 4 | 7 | 0 | 0 | 4 | 0 | 3.756E5 |  |  |  |  |  |  | 4 | 2 | 3 | 1 | 2 | 0 | 0 | 1 | 0 | N | 41758 | TRINITY\_DN273\_c0\_g4\_i11.p1 |
| 86 | 136 | P83750\|ACTB\_CYPCA | 158.94 | 18 | 15 | 4 | 7 | 0 | 0 | 4 | 0 | 3.756E5 |  |  |  |  |  |  | 4 | 2 | 3 | 1 | 2 | 0 | 0 | 1 | 0 | N | 41753 | Actin, cytoplasmic 1 OS=Cyprinus carpio OX=7962 GN=actb PE=3 SV=1 |
| 86 | 138 | P02572\|ACT2\_DROME | 158.94 | 18 | 15 | 4 | 7 | 0 | 0 | 4 | 0 | 3.756E5 |  |  |  |  |  |  | 4 | 2 | 3 | 1 | 2 | 0 | 0 | 1 | 0 | N | 41824 | Actin-42A OS=Drosophila melanogaster OX=7227 GN=Act42A PE=1 SV=3 |
| 86 | 143 | P60713\|ACTB\_SHEEP | 158.94 | 18 | 15 | 4 | 7 | 0 | 0 | 4 | 0 | 3.756E5 |  |  |  |  |  |  | 4 | 2 | 3 | 1 | 2 | 0 | 0 | 1 | 0 | N | 41737 | Actin, cytoplasmic 1 OS=Ovis aries OX=9940 GN=ACTB PE=2 SV=1 |
| 86 | 144 | Q4R561\|ACTB\_MACFA | 158.94 | 18 | 15 | 4 | 7 | 0 | 0 | 4 | 0 | 3.756E5 |  |  |  |  |  |  | 4 | 2 | 3 | 1 | 2 | 0 | 0 | 1 | 0 | N | 41737 | Actin, cytoplasmic 1 OS=Macaca fascicularis OX=9541 GN=ACTB PE=2 SV=1 |
| 86 | 145 | Q76N69\|ACTB\_CHLAE | 158.94 | 18 | 15 | 4 | 7 | 0 | 0 | 4 | 0 | 3.756E5 |  |  |  |  |  |  | 4 | 2 | 3 | 1 | 2 | 0 | 0 | 1 | 0 | N | 41737 | Actin, cytoplasmic 1 OS=Chlorocebus aethiops OX=9534 GN=ACTB PE=2 SV=1 |
| 86 | 146 | Q4L0Y2\|ACTB\_SPECI | 158.94 | 18 | 15 | 4 | 7 | 0 | 0 | 4 | 0 | 3.756E5 |  |  |  |  |  |  | 4 | 2 | 3 | 1 | 2 | 0 | 0 | 1 | 0 | N | 41737 | Actin, cytoplasmic 1 OS=Spermophilus citellus OX=9997 GN=ACTB PE=2 SV=1 |
| 86 | 147 | TRINITY\_DN273\_c0\_g4\_i2.p1 | 158.94 | 18 | 15 | 4 | 7 | 0 | 0 | 4 | 0 | 3.756E5 |  |  |  |  |  |  | 4 | 2 | 3 | 1 | 2 | 0 | 0 | 1 | 0 | N | 41772 | TRINITY\_DN273\_c0\_g4\_i2.p1 |
| 86 | 148 | TRINITY\_DN273\_c0\_g4\_i4.p1 | 158.94 | 18 | 15 | 4 | 7 | 0 | 0 | 4 | 0 | 3.756E5 |  |  |  |  |  |  | 4 | 2 | 3 | 1 | 2 | 0 | 0 | 1 | 0 | N | 41772 | TRINITY\_DN273\_c0\_g4\_i4.p1 |
| 86 | 149 | TRINITY\_DN273\_c0\_g4\_i9.p1 | 158.94 | 18 | 15 | 4 | 7 | 0 | 0 | 4 | 0 | 3.756E5 |  |  |  |  |  |  | 4 | 2 | 3 | 1 | 2 | 0 | 0 | 1 | 0 | N | 41772 | TRINITY\_DN273\_c0\_g4\_i9.p1 |
| 86 | 151 | P84856\|ACTB\_CHLPG | 158.94 | 18 | 15 | 4 | 7 | 0 | 0 | 4 | 0 | 3.756E5 |  |  |  |  |  |  | 4 | 2 | 3 | 1 | 2 | 0 | 0 | 1 | 0 | N | 40445 | Actin, cytoplasmic 1 OS=Chlorocebus pygerythrus OX=60710 GN=ACTB PE=1 SV=1 |
| 86 | 152 | P63258\|ACTG\_BOVIN | 158.94 | 18 | 15 | 4 | 7 | 0 | 0 | 4 | 0 | 3.756E5 |  |  |  |  |  |  | 4 | 2 | 3 | 1 | 2 | 0 | 0 | 1 | 0 | N | 41793 | Actin, cytoplasmic 2 OS=Bos taurus OX=9913 GN=ACTG1 PE=1 SV=1 |
| 86 | 153 | P63260\|ACTG\_MOUSE | 158.94 | 18 | 15 | 4 | 7 | 0 | 0 | 4 | 0 | 3.756E5 |  |  |  |  |  |  | 4 | 2 | 3 | 1 | 2 | 0 | 0 | 1 | 0 | N | 41793 | Actin, cytoplasmic 2 OS=Mus musculus OX=10090 GN=Actg1 PE=1 SV=1 |
| 86 | 154 | P63259\|ACTG\_RAT | 158.94 | 18 | 15 | 4 | 7 | 0 | 0 | 4 | 0 | 3.756E5 |  |  |  |  |  |  | 4 | 2 | 3 | 1 | 2 | 0 | 0 | 1 | 0 | N | 41793 | Actin, cytoplasmic 2 OS=Rattus norvegicus OX=10116 GN=Actg1 PE=1 SV=1 |
| 86 | 155 | A2BDB0\|ACTG\_XENLA | 158.94 | 18 | 15 | 4 | 7 | 0 | 0 | 4 | 0 | 3.756E5 |  |  |  |  |  |  | 4 | 2 | 3 | 1 | 2 | 0 | 0 | 1 | 0 | N | 41793 | Actin, cytoplasmic 2 OS=Xenopus laevis OX=8355 GN=actg1 PE=2 SV=1 |
| 86 | 156 | Q8JJB8\|ACTG\_TRISC | 158.94 | 18 | 15 | 4 | 7 | 0 | 0 | 4 | 0 | 3.756E5 |  |  |  |  |  |  | 4 | 2 | 3 | 1 | 2 | 0 | 0 | 1 | 0 | N | 41751 | Actin, cytoplasmic 2 OS=Triakis scyllium OX=30494 GN=actg1 PE=2 SV=1 |
| 86 | 157 | Q5JAK2\|ACTG\_PELLE | 158.94 | 18 | 15 | 4 | 7 | 0 | 0 | 4 | 0 | 3.756E5 |  |  |  |  |  |  | 4 | 2 | 3 | 1 | 2 | 0 | 0 | 1 | 0 | N | 41779 | Actin, cytoplasmic 2 OS=Pelophylax lessonae OX=45623 GN=actg1 PE=2 SV=1 |
| 86 | 158 | P15475\|ACTB\_XENBO | 158.94 | 18 | 15 | 4 | 7 | 0 | 0 | 4 | 0 | 3.756E5 |  |  |  |  |  |  | 4 | 2 | 3 | 1 | 2 | 0 | 0 | 1 | 0 | N | 41848 | Actin, cytoplasmic 1 OS=Xenopus borealis OX=8354 GN=actb PE=3 SV=1 |
| 86 | 166 | TRINITY\_DN836\_c0\_g1\_i1.p1 | 158.94 | 18 | 15 | 4 | 7 | 0 | 0 | 4 | 0 | 3.756E5 |  |  |  |  |  |  | 4 | 2 | 3 | 1 | 2 | 0 | 0 | 1 | 0 | N | 41822 | TRINITY\_DN836\_c0\_g1\_i1.p1 |
| 86 | 167 | P10987\|ACT1\_DROME | 158.94 | 18 | 15 | 4 | 7 | 0 | 0 | 4 | 0 | 3.756E5 |  |  |  |  |  |  | 4 | 2 | 3 | 1 | 2 | 0 | 0 | 1 | 0 | N | 41822 | Actin-5C OS=Drosophila melanogaster OX=7227 GN=Act5C PE=1 SV=4 |
| 86 | 168 | TRINITY\_DN836\_c0\_g1\_i3.p1 | 158.94 | 18 | 15 | 4 | 7 | 0 | 0 | 4 | 0 | 3.756E5 |  |  |  |  |  |  | 4 | 2 | 3 | 1 | 2 | 0 | 0 | 1 | 0 | N | 41822 | TRINITY\_DN836\_c0\_g1\_i3.p1 |
| 86 | 169 | P68556\|ACT1\_DIBDE | 158.94 | 18 | 15 | 4 | 7 | 0 | 0 | 4 | 0 | 3.756E5 |  |  |  |  |  |  | 4 | 2 | 3 | 1 | 2 | 0 | 0 | 1 | 0 | N | 41745 | Actin-1/4 OS=Dibothriocephalus dendriticus OX=28845 GN=ACT1 PE=2 SV=1 |
| 86 | 170 | TRINITY\_DN836\_c0\_g1\_i2.p1 | 158.94 | 18 | 15 | 4 | 7 | 0 | 0 | 4 | 0 | 3.756E5 |  |  |  |  |  |  | 4 | 2 | 3 | 1 | 2 | 0 | 0 | 1 | 0 | N | 41822 | TRINITY\_DN836\_c0\_g1\_i2.p1 |
| 86 | 171 | P04829\|ACT3\_BOMMO | 158.94 | 18 | 15 | 4 | 7 | 0 | 0 | 4 | 0 | 3.756E5 |  |  |  |  |  |  | 4 | 2 | 3 | 1 | 2 | 0 | 0 | 1 | 0 | N | 41918 | Actin, cytoplasmic A3 OS=Bombyx mori OX=7091 PE=3 SV=3 |
| 90 | 231 | P86700\|ACT\_CHIOP | 151.36 | 23 | 8 | 8 | 23 | 0 | 0 | 8 | 0 |  |  | 6.6192E5 |  |  |  |  | 3 | 1 | 1 | 1 | 3 | 0 | 0 | 1 | 0 | N | 20803 | Actin, muscle (Fragments) OS=Chionoecetes opilio OX=41210 PE=1 SV=1 |
| 69 | 104 | TRINITY\_DN4312\_c0\_g1\_i15.p1 | 151.32 | 4 | 1 | 0 | 3 | 4 | 2 | 3 | 0 | 1.2152E5 |  | 1.3497E6 | 1.719E6 | 7.491E5 | 1.4169E6 |  | 5 | 5 | 1 | 0 | 3 | 4 | 2 | 4 | 0 | Y | 144784 | TRINITY\_DN4312\_c0\_g1\_i15.p1 |
| 69 | 105 | TRINITY\_DN4312\_c0\_g1\_i7.p1 | 151.32 | 4 | 1 | 0 | 3 | 4 | 2 | 3 | 0 | 1.2152E5 |  | 1.3497E6 | 1.719E6 | 7.491E5 | 1.4169E6 |  | 5 | 5 | 1 | 0 | 3 | 4 | 2 | 4 | 0 | Y | 144784 | TRINITY\_DN4312\_c0\_g1\_i7.p1 |
| 69 | 108 | TRINITY\_DN4312\_c0\_g1\_i9.p1 | 151.32 | 4 | 1 | 0 | 4 | 4 | 2 | 4 | 0 | 1.2152E5 |  | 1.3497E6 | 1.719E6 | 7.491E5 | 1.4169E6 |  | 5 | 5 | 1 | 0 | 3 | 4 | 2 | 4 | 0 | Y | 119721 | TRINITY\_DN4312\_c0\_g1\_i9.p1 |
| 85 | 100 | Q6IFU8\|K1C17\_RAT | 143.88 | 13 | 7 | 0 | 0 | 0 | 0 | 0 | 5 |  |  |  |  |  |  | 1.3888E5 | 3 | 1 | 2 | 0 | 0 | 0 | 0 | 0 | 1 | N | 48123 | Keratin, type I cytoskeletal 17 OS=Rattus norvegicus OX=10116 GN=Krt17 PE=1 SV=1 |
| 85 | 103 | Q9QWL7\|K1C17\_MOUSE | 143.88 | 13 | 7 | 0 | 0 | 0 | 0 | 0 | 5 |  |  |  |  |  |  | 1.3888E5 | 3 | 1 | 2 | 0 | 0 | 0 | 0 | 0 | 1 | N | 48162 | Keratin, type I cytoskeletal 17 OS=Mus musculus OX=10090 GN=Krt17 PE=1 SV=3 |
| 96 | 109 | Q8SPJ1\|PLAK\_BOVIN | 139.73 | 6 | 3 | 0 | 2 | 0 | 0 | 2 | 0 | 2.3793E5 |  | 9.9975E4 |  |  | 4.1769E4 |  | 3 | 3 | 2 | 0 | 1 | 0 | 0 | 1 | 0 | Y | 81821 | Junction plakoglobin OS=Bos taurus OX=9913 GN=JUP PE=2 SV=1 |
| 96 | 123 | P14923\|PLAK\_HUMAN | 139.73 | 6 | 3 | 0 | 2 | 0 | 0 | 2 | 0 | 2.3793E5 |  | 9.9975E4 |  |  | 4.1769E4 |  | 3 | 3 | 2 | 0 | 1 | 0 | 0 | 1 | 0 | Y | 81745 | Junction plakoglobin OS=Homo sapiens OX=9606 GN=JUP PE=1 SV=3 |
| 96 | 124 | Q8WNW3\|PLAK\_PIG | 139.73 | 6 | 3 | 0 | 2 | 0 | 0 | 2 | 0 | 2.3793E5 |  | 9.9975E4 |  |  | 4.1769E4 |  | 3 | 3 | 2 | 0 | 1 | 0 | 0 | 1 | 0 | Y | 81850 | Junction plakoglobin OS=Sus scrofa OX=9823 GN=Jup PE=2 SV=1 |
| 96 | 125 | Q02257\|PLAK\_MOUSE | 139.73 | 6 | 3 | 0 | 2 | 0 | 0 | 2 | 0 | 2.3793E5 |  | 9.9975E4 |  |  | 4.1769E4 |  | 3 | 3 | 2 | 0 | 1 | 0 | 0 | 1 | 0 | Y | 81801 | Junction plakoglobin OS=Mus musculus OX=10090 GN=Jup PE=1 SV=3 |
| 100 | 188 | TRINITY\_DN16204\_c0\_g1\_i2.p1 | 137.89 | 30 | 0 | 0 | 0 | 0 | 0 | 30 | 0 |  |  |  |  |  | 7.6259E5 |  | 4 | 4 | 0 | 0 | 0 | 0 | 0 | 4 | 0 | Y | 23699 | TRINITY\_DN16204\_c0\_g1\_i2.p1 |
| 102 | 691 | TRINITY\_DN838\_c0\_g3\_i1.p1 | 128.43 | 15 | 0 | 0 | 0 | 0 | 15 | 15 | 6 |  |  |  |  | 1.5563E6 | 2.1615E6 | 1.8558E6 | 2 | 2 | 0 | 0 | 0 | 0 | 2 | 2 | 1 | Y | 25927 | TRINITY\_DN838\_c0\_g3\_i1.p1 |
| 102 | 692 | TRINITY\_DN838\_c0\_g3\_i3.p1 | 128.43 | 14 | 0 | 0 | 0 | 0 | 14 | 14 | 5 |  |  |  |  | 1.5563E6 | 2.1615E6 | 1.8558E6 | 2 | 2 | 0 | 0 | 0 | 0 | 2 | 2 | 1 | Y | 29820 | TRINITY\_DN838\_c0\_g3\_i3.p1 |
| 102 | 693 | TRINITY\_DN838\_c0\_g3\_i2.p1 | 128.43 | 14 | 0 | 0 | 0 | 0 | 14 | 14 | 5 |  |  |  |  | 1.5563E6 | 2.1615E6 | 1.8558E6 | 2 | 2 | 0 | 0 | 0 | 0 | 2 | 2 | 1 | Y | 29820 | TRINITY\_DN838\_c0\_g3\_i2.p1 |
| 128 | 696 | TRINITY\_DN2373\_c0\_g1\_i12.p1 | 127.30 | 17 | 0 | 0 | 12 | 0 | 0 | 6 | 0 |  |  | 3.7553E5 |  |  | 1.6124E5 |  | 2 | 2 | 0 | 0 | 1 | 0 | 0 | 1 | 0 | Y | 24574 | TRINITY\_DN2373\_c0\_g1\_i12.p1 |
| 128 | 697 | TRINITY\_DN2373\_c0\_g1\_i1.p1 | 127.30 | 17 | 0 | 0 | 12 | 0 | 0 | 6 | 0 |  |  | 3.7553E5 |  |  | 1.6124E5 |  | 2 | 2 | 0 | 0 | 1 | 0 | 0 | 1 | 0 | Y | 24574 | TRINITY\_DN2373\_c0\_g1\_i1.p1 |
| 128 | 698 | TRINITY\_DN2373\_c0\_g1\_i22.p1 | 127.30 | 17 | 0 | 0 | 12 | 0 | 0 | 6 | 0 |  |  | 3.7553E5 |  |  | 1.6124E5 |  | 2 | 2 | 0 | 0 | 1 | 0 | 0 | 1 | 0 | Y | 24574 | TRINITY\_DN2373\_c0\_g1\_i22.p1 |
| 104 | 2260 | TRINITY\_DN28260\_c0\_g1\_i1.p1 | 126.24 | 11 | 0 | 0 | 9 | 11 | 9 | 0 | 0 |  |  | 2.0272E5 | 1.5783E6 | 3.4602E5 |  |  | 2 | 2 | 0 | 0 | 1 | 2 | 1 | 0 | 0 | N | 21284 | TRINITY\_DN28260\_c0\_g1\_i1.p1 |
| 104 | 2261 | TRINITY\_DN28260\_c0\_g1\_i2.p1 | 126.24 | 11 | 0 | 0 | 9 | 11 | 9 | 0 | 0 |  |  | 2.0272E5 | 1.5783E6 | 3.4602E5 |  |  | 2 | 2 | 0 | 0 | 1 | 2 | 1 | 0 | 0 | N | 21284 | TRINITY\_DN28260\_c0\_g1\_i2.p1 |
| 104 | 2262 | TRINITY\_DN28260\_c0\_g1\_i3.p1 | 126.24 | 9 | 0 | 0 | 8 | 9 | 8 | 0 | 0 |  |  | 2.0272E5 | 1.5783E6 | 3.4602E5 |  |  | 2 | 2 | 0 | 0 | 1 | 2 | 1 | 0 | 0 | N | 25608 | TRINITY\_DN28260\_c0\_g1\_i3.p1 |
| 91 | 368 | TRINITY\_DN7688\_c0\_g2\_i1.p1 | 122.82 | 16 | 0 | 0 | 0 | 0 | 11 | 16 | 5 |  |  |  |  | 8.9582E4 | 1.4217E6 | 1.822E5 | 2 | 2 | 0 | 0 | 0 | 0 | 1 | 3 | 1 | N | 27760 | TRINITY\_DN7688\_c0\_g2\_i1.p1 |
| 91 | 695 | TRINITY\_DN7688\_c0\_g2\_i2.p1 | 122.82 | 23 | 0 | 0 | 0 | 0 | 16 | 23 | 7 |  |  |  |  | 8.9582E4 | 1.4217E6 | 1.822E5 | 2 | 2 | 0 | 0 | 0 | 0 | 1 | 3 | 1 | N | 19394 | TRINITY\_DN7688\_c0\_g2\_i2.p1 |
| 101 | 702 | P0DMB9\|VA5\_VESVE | 120.77 | 14 | 0 | 0 | 0 | 0 | 0 | 0 | 14 |  |  |  |  |  |  | 1.2172E5 | 3 | 1 | 0 | 0 | 0 | 0 | 0 | 0 | 3 | N | 22718 | Venom allergen 5 OS=Vespa velutina OX=202808 PE=1 SV=2 |
| 82 | 313 | P31151\|S10A7\_HUMAN | 117.19 | 25 | 25 | 11 | 11 | 11 | 11 | 11 | 0 | 8.2506E5 | 9.93E4 | 1.1071E5 | 1.1403E5 | 4.9608E4 | 6.224E4 |  | 3 | 3 | 3 | 1 | 1 | 1 | 1 | 1 | 0 | Y | 11471 | Protein S100-A7 OS=Homo sapiens OX=9606 GN=S100A7 PE=1 SV=4 |
| 57 | 249 | Q5D7H4\|HUGAB\_VESVU | 114.81 | 8 | 0 | 0 | 5 | 7 | 7 | 8 | 7 |  |  | 9.5454E4 | 2.26E5 | 1.2458E6 | 1.9886E7 | 3.2093E5 | 3 | 2 | 0 | 0 | 1 | 2 | 3 | 11 | 2 | N | 40072 | Inactive hyaluronidase B OS=Vespula vulgaris OX=7454 PE=1 SV=1 |
| 115 | 247 | TRINITY\_DN1697\_c0\_g1\_i11.p1 | 113.54 | 21 | 0 | 0 | 0 | 0 | 0 | 21 | 0 |  |  |  |  |  | 6.0165E5 |  | 2 | 2 | 0 | 0 | 0 | 0 | 0 | 2 | 0 | Y | 16841 | TRINITY\_DN1697\_c0\_g1\_i11.p1 |
| 115 | 248 | TRINITY\_DN1697\_c0\_g1\_i18.p1 | 113.54 | 21 | 0 | 0 | 0 | 0 | 0 | 21 | 0 |  |  |  |  |  | 6.0165E5 |  | 2 | 2 | 0 | 0 | 0 | 0 | 0 | 2 | 0 | Y | 16841 | TRINITY\_DN1697\_c0\_g1\_i18.p1 |
| 106 | 312 | P00038\|CYC\_APIME | 113.53 | 22 | 0 | 0 | 0 | 0 | 0 | 22 | 22 |  |  |  |  |  | 6.4764E6 | 1.0498E7 | 2 | 2 | 0 | 0 | 0 | 0 | 0 | 2 | 2 | N | 11910 | Cytochrome c OS=Apis mellifera OX=7460 GN=cytC PE=1 SV=2 |
| 98 | 383 | P00768\|CTR2\_VESOR | 112.16 | 9 | 0 | 0 | 0 | 0 | 0 | 4 | 9 |  |  |  |  |  | 1.1555E6 | 3.7355E6 | 3 | 3 | 0 | 0 | 0 | 0 | 0 | 1 | 3 | N | 23471 | Chymotrypsin-2 OS=Vespa orientalis OX=7447 PE=1 SV=1 |
| 98 | 384 | P00769\|CTR2\_VESCR | 112.16 | 9 | 0 | 0 | 0 | 0 | 0 | 4 | 9 |  |  |  |  |  | 1.1555E6 | 3.7355E6 | 3 | 3 | 0 | 0 | 0 | 0 | 0 | 1 | 3 | N | 23677 | Chymotrypsin-2 OS=Vespa crabro OX=7445 PE=1 SV=1 |
| 124 | 390 | TRINITY\_DN3922\_c0\_g1\_i1.p1 | 109.72 | 9 | 0 | 0 | 0 | 0 | 0 | 9 | 0 |  |  |  |  |  | 6.544E5 |  | 2 | 2 | 0 | 0 | 0 | 0 | 0 | 2 | 0 | N | 41545 | TRINITY\_DN3922\_c0\_g1\_i1.p1 |
| 124 | 700 | TRINITY\_DN3922\_c0\_g1\_i2.p1 | 109.72 | 9 | 0 | 0 | 0 | 0 | 0 | 9 | 0 |  |  |  |  |  | 6.544E5 |  | 2 | 2 | 0 | 0 | 0 | 0 | 0 | 2 | 0 | N | 39543 | TRINITY\_DN3922\_c0\_g1\_i2.p1 |
| 124 | 701 | TRINITY\_DN3922\_c0\_g1\_i3.p1 | 109.72 | 9 | 0 | 0 | 0 | 0 | 0 | 9 | 0 |  |  |  |  |  | 6.544E5 |  | 2 | 2 | 0 | 0 | 0 | 0 | 0 | 2 | 0 | N | 39543 | TRINITY\_DN3922\_c0\_g1\_i3.p1 |
| 71 | 287 | Q6IME9\|K2C72\_MOUSE | 106.47 | 4 | 2 | 4 | 4 | 2 | 2 | 2 | 0 |  | 7.6243E4 | 1.7453E5 |  |  |  |  | 2 | 1 | 1 | 3 | 3 | 2 | 2 | 2 | 0 | N | 56750 | Keratin, type II cytoskeletal 72 OS=Mus musculus OX=10090 GN=Krt72 PE=3 SV=1 |
| 71 | 288 | Q86Y46\|K2C73\_HUMAN | 106.47 | 4 | 2 | 4 | 4 | 2 | 2 | 2 | 0 |  | 7.6243E4 | 1.7453E5 |  |  |  |  | 2 | 1 | 1 | 3 | 3 | 2 | 2 | 2 | 0 | N | 58923 | Keratin, type II cytoskeletal 73 OS=Homo sapiens OX=9606 GN=KRT73 PE=1 SV=1 |
| 71 | 298 | Q3SY84\|K2C71\_HUMAN | 106.47 | 4 | 2 | 4 | 4 | 2 | 2 | 2 | 0 |  | 7.6243E4 | 1.7453E5 |  |  |  |  | 2 | 1 | 1 | 3 | 3 | 2 | 2 | 2 | 0 | N | 57292 | Keratin, type II cytoskeletal 71 OS=Homo sapiens OX=9606 GN=KRT71 PE=1 SV=3 |
| 71 | 300 | Q148H5\|K2C71\_BOVIN | 106.47 | 4 | 2 | 4 | 4 | 2 | 2 | 2 | 0 |  | 7.6243E4 | 1.7453E5 |  |  |  |  | 2 | 1 | 1 | 3 | 3 | 2 | 2 | 2 | 0 | N | 57408 | Keratin, type II cytoskeletal 71 OS=Bos taurus OX=9913 GN=KRT71 PE=2 SV=1 |
| 71 | 301 | Q14CN4\|K2C72\_HUMAN | 106.47 | 4 | 2 | 4 | 4 | 2 | 2 | 2 | 0 |  | 7.6243E4 | 1.7453E5 |  |  |  |  | 2 | 1 | 1 | 3 | 3 | 2 | 2 | 2 | 0 | N | 55877 | Keratin, type II cytoskeletal 72 OS=Homo sapiens OX=9606 GN=KRT72 PE=1 SV=2 |
| 71 | 302 | E1AB55\|K2C71\_FELCA | 106.47 | 4 | 2 | 4 | 4 | 2 | 2 | 2 | 0 |  | 7.6243E4 | 1.7453E5 |  |  |  |  | 2 | 1 | 1 | 3 | 3 | 2 | 2 | 2 | 0 | N | 57301 | Keratin, type II cytoskeletal 71 OS=Felis catus OX=9685 GN=KRT71 PE=2 SV=1 |
| 71 | 303 | Q6IG04\|K2C72\_RAT | 106.47 | 4 | 2 | 4 | 4 | 2 | 2 | 2 | 0 |  | 7.6243E4 | 1.7453E5 |  |  |  |  | 2 | 1 | 1 | 3 | 3 | 2 | 2 | 2 | 0 | N | 56851 | Keratin, type II cytoskeletal 72 OS=Rattus norvegicus OX=10116 GN=Krt72 PE=3 SV=2 |
| 71 | 352 | Q7RTS7\|K2C74\_HUMAN | 106.47 | 4 | 2 | 4 | 4 | 2 | 2 | 2 | 0 |  | 7.6243E4 | 1.7453E5 |  |  |  |  | 2 | 1 | 1 | 3 | 3 | 2 | 2 | 2 | 0 | N | 57865 | Keratin, type II cytoskeletal 74 OS=Homo sapiens OX=9606 GN=KRT74 PE=1 SV=2 |
| 78 | 190 | P12273\|PIP\_HUMAN | 105.72 | 28 | 28 | 7 | 7 | 7 | 7 | 7 | 7 | 2.474E6 | 1.2382E6 | 3.5017E5 | 5.2023E5 | 6.6449E5 | 1.8421E6 | 1.2852E6 | 3 | 3 | 3 | 1 | 1 | 1 | 1 | 1 | 1 | Y | 16572 | Prolactin-inducible protein OS=Homo sapiens OX=9606 GN=PIP PE=1 SV=1 |
| 125 | 412 | TRINITY\_DN5303\_c0\_g1\_i4.p1 | 105.14 | 12 | 0 | 0 | 0 | 0 | 0 | 0 | 12 |  |  |  |  |  |  | 1.3949E6 | 2 | 2 | 0 | 0 | 0 | 0 | 0 | 0 | 2 | N | 30767 | TRINITY\_DN5303\_c0\_g1\_i4.p1 |
| 120 | 246 | TRINITY\_DN36500\_c0\_g1\_i2.p1 | 104.98 | 4 | 0 | 4 | 0 | 0 | 0 | 0 | 0 |  | 2.614E5 |  |  |  |  |  | 1 | 1 | 0 | 1 | 0 | 0 | 0 | 0 | 0 | Y | 55122 | TRINITY\_DN36500\_c0\_g1\_i2.p1 |
| 141 | 252 | TRINITY\_DN122\_c0\_g1\_i5.p1 | 104.69 | 4 | 0 | 4 | 0 | 0 | 0 | 0 | 0 |  | 1.0063E7 |  |  |  |  |  | 1 | 1 | 0 | 1 | 0 | 0 | 0 | 0 | 0 | N | 42431 | TRINITY\_DN122\_c0\_g1\_i5.p1 |
| 109 | 337 | B5U6Y8\|OXLA\_ECHOC | 104.20 | 5 | 5 | 0 | 5 | 0 | 0 | 0 | 0 | 5.1122E5 |  | 3.9238E4 |  |  |  |  | 1 | 1 | 2 | 0 | 1 | 0 | 0 | 0 | 0 | Y | 56523 | L-amino-acid oxidase OS=Echis ocellatus OX=99586 PE=1 SV=1 |
| 81 | 344 | P62972\|UBIQP\_XENLA | 103.23 | 17 | 10 | 10 | 17 | 10 | 10 | 7 | 7 | 2.0921E5 | 2.2744E5 | 5.2848E5 | 8.1486E4 | 8.2535E4 | 1.6527E5 | 3.3803E5 | 2 | 2 | 1 | 1 | 2 | 1 | 1 | 1 | 1 | N | 18801 | Polyubiquitin (Fragment) OS=Xenopus laevis OX=8355 PE=1 SV=2 |
| 81 | 345 | P0CG65\|UBB\_PANTR | 103.23 | 12 | 7 | 7 | 12 | 7 | 7 | 5 | 5 | 2.0921E5 | 2.2744E5 | 5.2848E5 | 8.1486E4 | 8.2535E4 | 1.6527E5 | 3.3803E5 | 2 | 2 | 1 | 1 | 2 | 1 | 1 | 1 | 1 | N | 25762 | Polyubiquitin-B OS=Pan troglodytes OX=9598 GN=UBB PE=3 SV=1 |
| 81 | 346 | P0CG62\|UBB\_CHICK | 103.23 | 9 | 5 | 5 | 9 | 5 | 5 | 4 | 4 | 2.0921E5 | 2.2744E5 | 5.2848E5 | 8.1486E4 | 8.2535E4 | 1.6527E5 | 3.3803E5 | 2 | 2 | 1 | 1 | 2 | 1 | 1 | 1 | 1 | N | 34369 | Polyubiquitin-B OS=Gallus gallus OX=9031 GN=UBB PE=2 SV=1 |
| 81 | 347 | P0CG51\|UBB\_RAT | 103.23 | 9 | 5 | 5 | 9 | 5 | 5 | 4 | 4 | 2.0921E5 | 2.2744E5 | 5.2848E5 | 8.1486E4 | 8.2535E4 | 1.6527E5 | 3.3803E5 | 2 | 2 | 1 | 1 | 2 | 1 | 1 | 1 | 1 | N | 34369 | Polyubiquitin-B OS=Rattus norvegicus OX=10116 GN=Ubb PE=1 SV=1 |
| 81 | 348 | P0CG68\|UBC\_PIG | 103.23 | 5 | 3 | 3 | 5 | 3 | 3 | 2 | 2 | 2.0921E5 | 2.2744E5 | 5.2848E5 | 8.1486E4 | 8.2535E4 | 1.6527E5 | 3.3803E5 | 2 | 2 | 1 | 1 | 2 | 1 | 1 | 1 | 1 | N | 59993 | Polyubiquitin-C OS=Sus scrofa OX=9823 GN=UBC PE=2 SV=1 |
| 81 | 349 | P62976\|UBIQP\_CRIGR | 103.23 | 4 | 2 | 2 | 4 | 2 | 2 | 2 | 2 | 2.0921E5 | 2.2744E5 | 5.2848E5 | 8.1486E4 | 8.2535E4 | 1.6527E5 | 3.3803E5 | 2 | 2 | 1 | 1 | 2 | 1 | 1 | 1 | 1 | N | 73950 | Polyubiquitin OS=Cricetulus griseus OX=10029 PE=2 SV=2 |
| 81 | 350 | P0CG48\|UBC\_HUMAN | 103.23 | 4 | 2 | 2 | 4 | 2 | 2 | 2 | 2 | 2.0921E5 | 2.2744E5 | 5.2848E5 | 8.1486E4 | 8.2535E4 | 1.6527E5 | 3.3803E5 | 2 | 2 | 1 | 1 | 2 | 1 | 1 | 1 | 1 | N | 77039 | Polyubiquitin-C OS=Homo sapiens OX=9606 GN=UBC PE=1 SV=3 |
| 81 | 376 | P29504\|RS27A\_MANSE | 103.23 | 18 | 10 | 10 | 18 | 10 | 10 | 8 | 8 | 2.0921E5 | 2.2744E5 | 5.2848E5 | 8.1486E4 | 8.2535E4 | 1.6527E5 | 3.3803E5 | 2 | 2 | 1 | 1 | 2 | 1 | 1 | 1 | 1 | N | 17848 | Ubiquitin-40S ribosomal protein S27a OS=Manduca sexta OX=7130 PE=2 SV=2 |
| 81 | 378 | P15357\|RS27A\_DROME | 103.23 | 18 | 10 | 10 | 18 | 10 | 10 | 8 | 8 | 2.0921E5 | 2.2744E5 | 5.2848E5 | 8.1486E4 | 8.2535E4 | 1.6527E5 | 3.3803E5 | 2 | 2 | 1 | 1 | 2 | 1 | 1 | 1 | 1 | N | 17940 | Ubiquitin-40S ribosomal protein S27a OS=Drosophila melanogaster OX=7227 GN=RpS27A PE=1 SV=2 |
| 81 | 379 | P62979\|RS27A\_HUMAN | 103.23 | 18 | 10 | 10 | 18 | 10 | 10 | 8 | 8 | 2.0921E5 | 2.2744E5 | 5.2848E5 | 8.1486E4 | 8.2535E4 | 1.6527E5 | 3.3803E5 | 2 | 2 | 1 | 1 | 2 | 1 | 1 | 1 | 1 | N | 17965 | Ubiquitin-40S ribosomal protein S27a OS=Homo sapiens OX=9606 GN=RPS27A PE=1 SV=2 |
| 84 | 453 | Q6KB66\|K2C80\_HUMAN | 102.27 | 7 | 2 | 2 | 7 | 0 | 2 | 2 | 2 |  |  | 6.8861E4 |  |  |  |  | 2 | 1 | 1 | 1 | 2 | 0 | 1 | 1 | 1 | N | 50525 | Keratin, type II cytoskeletal 80 OS=Homo sapiens OX=9606 GN=KRT80 PE=1 SV=2 |
| 108 | 328 | P19620\|ANXA2\_PIG | 100.13 | 9 | 9 | 0 | 4 | 0 | 0 | 0 | 0 | 3.4434E5 |  | 5.1373E4 |  |  |  |  | 2 | 2 | 2 | 0 | 1 | 0 | 0 | 0 | 0 | Y | 38542 | Annexin A2 OS=Sus scrofa OX=9823 GN=ANXA2 PE=1 SV=4 |
| 108 | 329 | A2SW69\|ANXA2\_SHEEP | 100.13 | 9 | 9 | 0 | 4 | 0 | 0 | 0 | 0 | 3.4434E5 |  | 5.1373E4 |  |  |  |  | 2 | 2 | 2 | 0 | 1 | 0 | 0 | 0 | 0 | Y | 38612 | Annexin A2 OS=Ovis aries OX=9940 GN=ANXA2 PE=1 SV=1 |
| 108 | 330 | P04272\|ANXA2\_BOVIN | 100.13 | 9 | 9 | 0 | 4 | 0 | 0 | 0 | 0 | 3.4434E5 |  | 5.1373E4 |  |  |  |  | 2 | 2 | 2 | 0 | 1 | 0 | 0 | 0 | 0 | Y | 38612 | Annexin A2 OS=Bos taurus OX=9913 GN=ANXA2 PE=1 SV=2 |
| 108 | 331 | P07356\|ANXA2\_MOUSE | 100.13 | 9 | 9 | 0 | 4 | 0 | 0 | 0 | 0 | 3.4434E5 |  | 5.1373E4 |  |  |  |  | 2 | 2 | 2 | 0 | 1 | 0 | 0 | 0 | 0 | Y | 38676 | Annexin A2 OS=Mus musculus OX=10090 GN=Anxa2 PE=1 SV=2 |
| 108 | 332 | Q6TEQ7\|ANXA2\_CANLF | 100.13 | 9 | 9 | 0 | 4 | 0 | 0 | 0 | 0 | 3.4434E5 |  | 5.1373E4 |  |  |  |  | 2 | 2 | 2 | 0 | 1 | 0 | 0 | 0 | 0 | Y | 38654 | Annexin A2 OS=Canis lupus familiaris OX=9615 GN=ANXA2 PE=1 SV=1 |
| 108 | 333 | Q2Q1M6\|ANXA2\_CEREL | 100.13 | 9 | 9 | 0 | 4 | 0 | 0 | 0 | 0 | 3.4434E5 |  | 5.1373E4 |  |  |  |  | 2 | 2 | 2 | 0 | 1 | 0 | 0 | 0 | 0 | Y | 38637 | Annexin A2 OS=Cervus elaphus OX=9860 GN=ANXA2 PE=2 SV=1 |
| 108 | 355 | C0HJG9\|ANXA2\_MESAU | 100.13 | 15 | 15 | 0 | 7 | 0 | 0 | 0 | 0 | 3.4434E5 |  | 5.1373E4 |  |  |  |  | 2 | 2 | 2 | 0 | 1 | 0 | 0 | 0 | 0 | Y | 22447 | Annexin A2 (Fragments) OS=Mesocricetus auratus OX=10036 PE=1 SV=1 |
| 108 | 356 | Q5R5A0\|ANXA2\_PONAB | 100.13 | 9 | 9 | 0 | 4 | 0 | 0 | 0 | 0 | 3.4434E5 |  | 5.1373E4 |  |  |  |  | 2 | 2 | 2 | 0 | 1 | 0 | 0 | 0 | 0 | Y | 38604 | Annexin A2 OS=Pongo abelii OX=9601 GN=ANXA2 PE=2 SV=1 |
| 108 | 357 | P07355\|ANXA2\_HUMAN | 100.13 | 9 | 9 | 0 | 4 | 0 | 0 | 0 | 0 | 3.4434E5 |  | 5.1373E4 |  |  |  |  | 2 | 2 | 2 | 0 | 1 | 0 | 0 | 0 | 0 | Y | 38604 | Annexin A2 OS=Homo sapiens OX=9606 GN=ANXA2 PE=1 SV=2 |
| 108 | 358 | Q07936\|ANXA2\_RAT | 100.13 | 9 | 9 | 0 | 4 | 0 | 0 | 0 | 0 | 3.4434E5 |  | 5.1373E4 |  |  |  |  | 2 | 2 | 2 | 0 | 1 | 0 | 0 | 0 | 0 | Y | 38678 | Annexin A2 OS=Rattus norvegicus OX=10116 GN=Anxa2 PE=1 SV=2 |
| 99 | 363 | TRINITY\_DN23073\_c0\_g1\_i1.p1 | 98.32 | 8 | 0 | 8 | 0 | 8 | 8 | 0 | 0 |  | 1.9327E6 |  | 1.5115E5 | 2.1031E5 |  |  | 1 | 1 | 0 | 2 | 0 | 1 | 1 | 0 | 0 | N | 24340 | TRINITY\_DN23073\_c0\_g1\_i1.p1 |
| 66 | 413 | G8XQX1\|OXLA\_DABRR | 93.11 | 4 | 4 | 4 | 4 | 4 | 4 | 4 | 4 | 6.8963E6 | 5.4364E6 | 3.7554E6 | 3.4857E6 | 3.1336E6 | 2.7058E6 | 3.37E6 | 1 | 1 | 4 | 3 | 2 | 3 | 1 | 1 | 1 | N | 56888 | L-amino-acid oxidase OS=Daboia russelii OX=8707 PE=1 SV=1 |
| 66 | 452 | Q4F867\|OXLA\_DABSI | 93.11 | 5 | 5 | 5 | 5 | 5 | 5 | 5 | 5 | 6.8963E6 | 5.4364E6 | 3.7554E6 | 3.4857E6 | 3.1336E6 | 2.7058E6 | 3.37E6 | 1 | 1 | 4 | 3 | 2 | 3 | 1 | 1 | 1 | N | 46372 | L-amino-acid oxidase (Fragments) OS=Daboia siamensis OX=343250 PE=1 SV=2 |
| 66 | 2257 | P0C2D7\|OXLA\_VIPBB | 93.11 | 23 | 23 | 23 | 23 | 23 | 23 | 23 | 23 | 6.8963E6 | 5.4364E6 | 3.7554E6 | 3.4857E6 | 3.1336E6 | 2.7058E6 | 3.37E6 | 1 | 1 | 4 | 3 | 2 | 3 | 1 | 1 | 1 | N | 10295 | L-amino-acid oxidase (Fragments) OS=Vipera berus berus OX=31156 PE=1 SV=1 |
| 127 | 1306 | TRINITY\_DN6873\_c0\_g1\_i3.p1 | 92.69 | 25 | 0 | 0 | 0 | 0 | 25 | 25 | 0 |  |  |  |  | 1.1402E6 | 1.4355E6 |  | 1 | 1 | 0 | 0 | 0 | 0 | 1 | 1 | 0 | N | 12918 | TRINITY\_DN6873\_c0\_g1\_i3.p1 |
| 127 | 2258 | TRINITY\_DN6873\_c0\_g1\_i2.p1 | 92.69 | 12 | 0 | 0 | 0 | 0 | 12 | 12 | 0 |  |  |  |  | 1.1402E6 | 1.4355E6 |  | 1 | 1 | 0 | 0 | 0 | 0 | 1 | 1 | 0 | N | 27219 | TRINITY\_DN6873\_c0\_g1\_i2.p1 |
| 113 | 254 | TRINITY\_DN11796\_c0\_g1\_i1.p1 | 92.40 | 1 | 0 | 0 | 0 | 0 | 0 | 1 | 0 |  |  |  |  |  | 2.7447E5 |  | 1 | 1 | 0 | 0 | 0 | 0 | 0 | 1 | 0 | N | 113195 | TRINITY\_DN11796\_c0\_g1\_i1.p1 |
| 89 | 236 | TRINITY\_DN126\_c0\_g1\_i17.p1 | 90.03 | 6 | 6 | 0 | 0 | 0 | 0 | 0 | 0 | 7.0268E5 |  |  |  |  |  |  | 2 | 2 | 2 | 0 | 0 | 0 | 0 | 0 | 0 | N | 85528 | TRINITY\_DN126\_c0\_g1\_i17.p1 |
| 89 | 237 | TRINITY\_DN126\_c0\_g1\_i12.p1 | 90.03 | 6 | 6 | 0 | 0 | 0 | 0 | 0 | 0 | 7.0268E5 |  |  |  |  |  |  | 2 | 2 | 2 | 0 | 0 | 0 | 0 | 0 | 0 | N | 85528 | TRINITY\_DN126\_c0\_g1\_i12.p1 |
| 89 | 238 | TRINITY\_DN126\_c0\_g1\_i25.p1 | 90.03 | 6 | 6 | 0 | 0 | 0 | 0 | 0 | 0 | 7.0268E5 |  |  |  |  |  |  | 2 | 2 | 2 | 0 | 0 | 0 | 0 | 0 | 0 | N | 85528 | TRINITY\_DN126\_c0\_g1\_i25.p1 |
| 118 | 261 | Q8N1N4\|K2C78\_HUMAN | 78.56 | 3 | 3 | 0 | 0 | 0 | 0 | 0 | 0 | 1.7056E5 |  |  |  |  |  |  | 1 | 1 | 1 | 0 | 0 | 0 | 0 | 0 | 0 | N | 56866 | Keratin, type II cytoskeletal 78 OS=Homo sapiens OX=9606 GN=KRT78 PE=1 SV=2 |
| 107 | 281 | TRINITY\_DN7919\_c0\_g1\_i8.p1 | 76.98 | 2 | 0 | 0 | 2 | 0 | 0 | 2 | 2 |  |  | 8.2942E4 |  |  | 3.1285E6 | 2.1533E6 | 1 | 1 | 0 | 0 | 1 | 0 | 0 | 2 | 1 | N | 75133 | TRINITY\_DN7919\_c0\_g1\_i8.p1 |
| 107 | 282 | TRINITY\_DN7919\_c0\_g1\_i2.p1 | 76.98 | 2 | 0 | 0 | 2 | 0 | 0 | 2 | 2 |  |  | 8.2942E4 |  |  | 3.1285E6 | 2.1533E6 | 1 | 1 | 0 | 0 | 1 | 0 | 0 | 2 | 1 | N | 80779 | TRINITY\_DN7919\_c0\_g1\_i2.p1 |
| 107 | 914 | TRINITY\_DN7919\_c0\_g1\_i4.p1 | 76.98 | 3 | 0 | 0 | 3 | 0 | 0 | 3 | 3 |  |  | 8.2942E4 |  |  | 3.1285E6 | 2.1533E6 | 1 | 1 | 0 | 0 | 1 | 0 | 0 | 2 | 1 | N | 49730 | TRINITY\_DN7919\_c0\_g1\_i4.p1 |
| 143 | 430 | Q0TMN0\|EFTU\_CLOP1 | 76.92 | 4 | 0 | 0 | 4 | 0 | 0 | 0 | 0 |  |  | 1.6189E5 |  |  |  |  | 1 | 1 | 0 | 0 | 1 | 0 | 0 | 0 | 0 | N | 43558 | Elongation factor Tu OS=Clostridium perfringens (strain ATCC 13124 / DSM 756 / JCM 1290 / NCIMB 6125 / NCTC 8237 / Type A) OX=195103 GN=tuf1 PE=3 SV=1 |
| 143 | 431 | Q8XFP8\|EFTU\_CLOPE | 76.92 | 4 | 0 | 0 | 4 | 0 | 0 | 0 | 0 |  |  | 1.6189E5 |  |  |  |  | 1 | 1 | 0 | 0 | 1 | 0 | 0 | 0 | 0 | N | 43558 | Elongation factor Tu OS=Clostridium perfringens (strain 13 / Type A) OX=195102 GN=tufA PE=3 SV=1 |
| 143 | 432 | Q0SQC8\|EFTU\_CLOPS | 76.92 | 4 | 0 | 0 | 4 | 0 | 0 | 0 | 0 |  |  | 1.6189E5 |  |  |  |  | 1 | 1 | 0 | 0 | 1 | 0 | 0 | 0 | 0 | N | 43558 | Elongation factor Tu OS=Clostridium perfringens (strain SM101 / Type A) OX=289380 GN=tuf1 PE=3 SV=1 |
| 143 | 439 | Q877L9\|EFTU\_CLOTE | 76.92 | 4 | 0 | 0 | 4 | 0 | 0 | 0 | 0 |  |  | 1.6189E5 |  |  |  |  | 1 | 1 | 0 | 0 | 1 | 0 | 0 | 0 | 0 | N | 43447 | Elongation factor Tu OS=Clostridium tetani (strain Massachusetts / E88) OX=212717 GN=tufA PE=3 SV=1 |
| 143 | 442 | A6LPP6\|EFTU\_CLOB8 | 76.92 | 4 | 0 | 0 | 4 | 0 | 0 | 0 | 0 |  |  | 1.6189E5 |  |  |  |  | 1 | 1 | 0 | 0 | 1 | 0 | 0 | 0 | 0 | N | 43624 | Elongation factor Tu OS=Clostridium beijerinckii (strain ATCC 51743 / NCIMB 8052) OX=290402 GN=tuf1 PE=3 SV=1 |
| 143 | 630 | A9KRZ4\|EFTU\_LACP7 | 76.92 | 4 | 0 | 0 | 4 | 0 | 0 | 0 | 0 |  |  | 1.6189E5 |  |  |  |  | 1 | 1 | 0 | 0 | 1 | 0 | 0 | 0 | 0 | N | 43856 | Elongation factor Tu OS=Lachnoclostridium phytofermentans (strain ATCC 700394 / DSM 18823 / ISDg) OX=357809 GN=tuf PE=3 SV=1 |
| 92 | 2271 | P05109\|S10A8\_HUMAN | 76.32 | 12 | 12 | 12 | 12 | 12 | 12 | 12 | 0 | 5.59E5 | 2.3619E5 | 1.3896E5 | 7.7215E4 | 4.4886E4 | 7.7496E4 |  | 1 | 1 | 1 | 1 | 1 | 1 | 1 | 1 | 0 | Y | 10835 | Protein S100-A8 OS=Homo sapiens OX=9606 GN=S100A8 PE=1 SV=1 |
| 129 | 2266 | TRINITY\_DN1736\_c0\_g1\_i5.p1 | 74.55 | 10 | 0 | 0 | 10 | 10 | 0 | 0 | 0 |  |  | 1.3568E5 | 1.6835E5 |  |  |  | 1 | 1 | 0 | 0 | 1 | 1 | 0 | 0 | 0 | N | 20050 | TRINITY\_DN1736\_c0\_g1\_i5.p1 |
| 129 | 2267 | TRINITY\_DN1736\_c0\_g1\_i25.p1 | 74.55 | 10 | 0 | 0 | 10 | 10 | 0 | 0 | 0 |  |  | 1.3568E5 | 1.6835E5 |  |  |  | 1 | 1 | 0 | 0 | 1 | 1 | 0 | 0 | 0 | N | 20050 | TRINITY\_DN1736\_c0\_g1\_i25.p1 |
| 129 | 2268 | TRINITY\_DN1736\_c0\_g1\_i30.p1 | 74.55 | 6 | 0 | 0 | 6 | 6 | 0 | 0 | 0 |  |  | 1.3568E5 | 1.6835E5 |  |  |  | 1 | 1 | 0 | 0 | 1 | 1 | 0 | 0 | 0 | N | 34706 | TRINITY\_DN1736\_c0\_g1\_i30.p1 |
| 129 | 2269 | TRINITY\_DN1736\_c0\_g1\_i24.p1 | 74.55 | 6 | 0 | 0 | 6 | 6 | 0 | 0 | 0 |  |  | 1.3568E5 | 1.6835E5 |  |  |  | 1 | 1 | 0 | 0 | 1 | 1 | 0 | 0 | 0 | N | 34888 | TRINITY\_DN1736\_c0\_g1\_i24.p1 |
| 129 | 2270 | TRINITY\_DN1736\_c0\_g1\_i4.p1 | 74.55 | 5 | 0 | 0 | 5 | 5 | 0 | 0 | 0 |  |  | 1.3568E5 | 1.6835E5 |  |  |  | 1 | 1 | 0 | 0 | 1 | 1 | 0 | 0 | 0 | N | 37093 | TRINITY\_DN1736\_c0\_g1\_i4.p1 |
| 121 | 326 | TRINITY\_DN1450\_c3\_g2\_i1.p1 | 74.23 | 12 | 6 | 0 | 0 | 0 | 0 | 6 | 0 | 9.9582E4 |  |  |  |  | 7.7847E4 |  | 2 | 2 | 1 | 0 | 0 | 0 | 0 | 1 | 0 | N | 21512 | TRINITY\_DN1450\_c3\_g2\_i1.p1 |
| 121 | 712 | Q9V3P0\|PRDX1\_DROME | 74.23 | 12 | 6 | 0 | 0 | 0 | 0 | 6 | 0 | 9.9582E4 |  |  |  |  | 7.7847E4 |  | 2 | 2 | 1 | 0 | 0 | 0 | 0 | 1 | 0 | N | 21738 | Peroxiredoxin 1 OS=Drosophila melanogaster OX=7227 GN=Jafrac1 PE=1 SV=1 |
| 174 | 2265 | P12763\|FETUA\_BOVIN | 73.75 | 6 | 6 | 0 | 0 | 0 | 0 | 0 | 0 | 4.2769E5 |  |  |  |  |  |  | 1 | 1 | 1 | 0 | 0 | 0 | 0 | 0 | 0 | N | 38419 | Alpha-2-HS-glycoprotein OS=Bos taurus OX=9913 GN=AHSG PE=1 SV=2 |
| 93 | 2273 | P02754\|LACB\_BOVIN | 72.06 | 8 | 8 | 8 | 8 | 8 | 8 | 8 | 0 | 2.8946E5 | 1.1975E5 | 2.0216E5 | 1.4495E5 | 2.1549E5 | 4.217E5 |  | 1 | 1 | 1 | 1 | 1 | 1 | 1 | 1 | 0 | Y | 19883 | Beta-lactoglobulin OS=Bos taurus OX=9913 GN=LGB PE=1 SV=3 |
| 112 | 2280 | P02755\|LACB\_BUBBU | 71.06 | 8 | 0 | 0 | 0 | 0 | 8 | 8 | 0 |  |  |  |  | 5.3867E5 | 3.6218E5 |  | 1 | 1 | 0 | 0 | 0 | 0 | 1 | 1 | 0 | Y | 20023 | Beta-lactoglobulin OS=Bubalus bubalis OX=89462 GN=LGB PE=1 SV=2 |
| 154 | 1385 | TRINITY\_DN254\_c0\_g1\_i1.p1 | 70.42 | 14 | 0 | 0 | 0 | 0 | 14 | 0 | 0 |  |  |  |  | 1.8262E5 |  |  | 1 | 1 | 0 | 0 | 0 | 0 | 1 | 0 | 0 | N | 14441 | TRINITY\_DN254\_c0\_g1\_i1.p1 |
| 172 | 1585 | TRINITY\_DN747\_c0\_g1\_i8.p1 | 68.25 | 2 | 2 | 0 | 0 | 0 | 0 | 0 | 0 | 2.0736E5 |  |  |  |  |  |  | 1 | 1 | 1 | 0 | 0 | 0 | 0 | 0 | 0 | N | 99872 | TRINITY\_DN747\_c0\_g1\_i8.p1 |
| 172 | 1586 | TRINITY\_DN747\_c0\_g1\_i4.p1 | 68.25 | 2 | 2 | 0 | 0 | 0 | 0 | 0 | 0 | 2.0736E5 |  |  |  |  |  |  | 1 | 1 | 1 | 0 | 0 | 0 | 0 | 0 | 0 | N | 99872 | TRINITY\_DN747\_c0\_g1\_i4.p1 |
| 172 | 1587 | TRINITY\_DN747\_c0\_g1\_i7.p1 | 68.25 | 2 | 2 | 0 | 0 | 0 | 0 | 0 | 0 | 2.0736E5 |  |  |  |  |  |  | 1 | 1 | 1 | 0 | 0 | 0 | 0 | 0 | 0 | N | 99872 | TRINITY\_DN747\_c0\_g1\_i7.p1 |
| 172 | 1588 | TRINITY\_DN747\_c0\_g1\_i1.p1 | 68.25 | 2 | 2 | 0 | 0 | 0 | 0 | 0 | 0 | 2.0736E5 |  |  |  |  |  |  | 1 | 1 | 1 | 0 | 0 | 0 | 0 | 0 | 0 | N | 102166 | TRINITY\_DN747\_c0\_g1\_i1.p1 |
| 110 | 484 | TRINITY\_DN27415\_c0\_g1\_i1.p1 | 68.01 | 3 | 0 | 0 | 0 | 3 | 0 | 0 | 0 |  |  |  | 1.1207E5 |  |  |  | 1 | 1 | 0 | 0 | 0 | 2 | 0 | 0 | 0 | N | 48517 | TRINITY\_DN27415\_c0\_g1\_i1.p1 |
| 162 | 814 | P0DO15\|GLCA2\_SOYBN | 66.41 | 2 | 2 | 0 | 0 | 0 | 0 | 0 | 0 | 2.4976E5 |  |  |  |  |  |  | 1 | 1 | 1 | 0 | 0 | 0 | 0 | 0 | 0 | N | 70306 | Beta-conglycinin alpha subunit 2 OS=Glycine max OX=3847 GN=CG-2 PE=1 SV=1 |
| 162 | 815 | P0DO16\|GLCA1\_SOYBN | 66.41 | 2 | 2 | 0 | 0 | 0 | 0 | 0 | 0 | 2.4976E5 |  |  |  |  |  |  | 1 | 1 | 1 | 0 | 0 | 0 | 0 | 0 | 0 | N | 70306 | Beta-conglycinin alpha subunit 1 OS=Glycine max OX=3847 GN=CG-3 PE=1 SV=1 |
| 162 | 2278 | F7J077\|GLCB2\_SOYBN | 66.41 | 3 | 3 | 0 | 0 | 0 | 0 | 0 | 0 | 2.4976E5 |  |  |  |  |  |  | 1 | 1 | 1 | 0 | 0 | 0 | 0 | 0 | 0 | N | 50442 | Beta-conglycinin beta subunit 2 OS=Glycine max OX=3847 GN=CG-4 PE=1 SV=1 |
| 162 | 2279 | P25974\|GLCB1\_SOYBN | 66.41 | 3 | 3 | 0 | 0 | 0 | 0 | 0 | 0 | 2.4976E5 |  |  |  |  |  |  | 1 | 1 | 1 | 0 | 0 | 0 | 0 | 0 | 0 | N | 50476 | Beta-conglycinin beta subunit 1 OS=Glycine max OX=3847 GN=CG-4 PE=1 SV=2 |
| 130 | 2284 | TRINITY\_DN6486\_c0\_g1\_i4.p1 | 65.97 | 7 | 7 | 7 | 0 | 0 | 0 | 0 | 0 | 3.7868E5 | 3.3082E6 |  |  |  |  |  | 1 | 1 | 1 | 1 | 0 | 0 | 0 | 0 | 0 | Y | 23438 | TRINITY\_DN6486\_c0\_g1\_i4.p1 |
| 159 | 546 | TRINITY\_DN3209\_c0\_g2\_i3.p1 | 63.54 | 2 | 0 | 2 | 0 | 0 | 0 | 0 | 0 |  | 3.8513E5 |  |  |  |  |  | 1 | 1 | 0 | 1 | 0 | 0 | 0 | 0 | 0 | N | 53297 | TRINITY\_DN3209\_c0\_g2\_i3.p1 |
| 159 | 547 | TRINITY\_DN3209\_c0\_g2\_i5.p1 | 63.54 | 2 | 0 | 2 | 0 | 0 | 0 | 0 | 0 |  | 3.8513E5 |  |  |  |  |  | 1 | 1 | 0 | 1 | 0 | 0 | 0 | 0 | 0 | N | 53297 | TRINITY\_DN3209\_c0\_g2\_i5.p1 |
| 171 | 1366 | Q29577\|KCRU\_PIG | 63.33 | 4 | 0 | 0 | 4 | 0 | 0 | 0 | 0 |  |  | 7.2941E4 |  |  |  |  | 1 | 1 | 0 | 0 | 1 | 0 | 0 | 0 | 0 | N | 46950 | Creatine kinase U-type, mitochondrial OS=Sus scrofa OX=9823 GN=CKMT1 PE=2 SV=2 |
| 171 | 1367 | Q9TTK8\|KCRU\_BOVIN | 63.33 | 4 | 0 | 0 | 4 | 0 | 0 | 0 | 0 |  |  | 7.2941E4 |  |  |  |  | 1 | 1 | 0 | 0 | 1 | 0 | 0 | 0 | 0 | N | 46897 | Creatine kinase U-type, mitochondrial OS=Bos taurus OX=9913 GN=CKMT1 PE=2 SV=1 |
| 171 | 1368 | P12532\|KCRU\_HUMAN | 63.33 | 4 | 0 | 0 | 4 | 0 | 0 | 0 | 0 |  |  | 7.2941E4 |  |  |  |  | 1 | 1 | 0 | 0 | 1 | 0 | 0 | 0 | 0 | N | 47037 | Creatine kinase U-type, mitochondrial OS=Homo sapiens OX=9606 GN=CKMT1A PE=1 SV=1 |
| 171 | 1369 | P30275\|KCRU\_MOUSE | 63.33 | 4 | 0 | 0 | 4 | 0 | 0 | 0 | 0 |  |  | 7.2941E4 |  |  |  |  | 1 | 1 | 0 | 0 | 1 | 0 | 0 | 0 | 0 | N | 47004 | Creatine kinase U-type, mitochondrial OS=Mus musculus OX=10090 GN=Ckmt1 PE=1 SV=1 |
| 171 | 1370 | P25809\|KCRU\_RAT | 63.33 | 4 | 0 | 0 | 4 | 0 | 0 | 0 | 0 |  |  | 7.2941E4 |  |  |  |  | 1 | 1 | 0 | 0 | 1 | 0 | 0 | 0 | 0 | N | 47029 | Creatine kinase U-type, mitochondrial OS=Rattus norvegicus OX=10116 GN=Ckmt1 PE=1 SV=1 |
| 87 | 736 | O62823\|CASA1\_BUBBU | 62.46 | 5 | 5 | 5 | 5 | 5 | 5 | 5 | 5 | 9.0559E5 | 2.6621E5 | 5.3122E5 | 1.7287E5 | 3.4714E5 | 3.8821E5 | 2.0483E6 | 1 | 1 | 1 | 1 | 1 | 1 | 1 | 1 | 1 | N | 24327 | Alpha-S1-casein OS=Bubalus bubalis OX=89462 GN=CSN1S1 PE=2 SV=2 |
| 87 | 738 | P02662\|CASA1\_BOVIN | 62.46 | 5 | 5 | 5 | 5 | 5 | 5 | 5 | 5 | 9.0559E5 | 2.6621E5 | 5.3122E5 | 1.7287E5 | 3.4714E5 | 3.8821E5 | 2.0483E6 | 1 | 1 | 1 | 1 | 1 | 1 | 1 | 1 | 1 | N | 24529 | Alpha-S1-casein OS=Bos taurus OX=9913 GN=CSN1S1 PE=1 SV=2 |
| 87 | 1545 | P18626\|CASA1\_CAPHI | 62.46 | 5 | 5 | 5 | 5 | 5 | 5 | 5 | 5 | 9.0559E5 | 2.6621E5 | 5.3122E5 | 1.7287E5 | 3.4714E5 | 3.8821E5 | 2.0483E6 | 1 | 1 | 1 | 1 | 1 | 1 | 1 | 1 | 1 | N | 24290 | Alpha-S1-casein OS=Capra hircus OX=9925 GN=CSN1S1 PE=1 SV=2 |
| 87 | 2289 | P04653\|CASA1\_SHEEP | 62.46 | 5 | 5 | 5 | 5 | 5 | 5 | 5 | 5 | 9.0559E5 | 2.6621E5 | 5.3122E5 | 1.7287E5 | 3.4714E5 | 3.8821E5 | 2.0483E6 | 1 | 1 | 1 | 1 | 1 | 1 | 1 | 1 | 1 | N | 24306 | Alpha-S1-casein OS=Ovis aries OX=9940 GN=CSN1S1 PE=1 SV=3 |
| 134 | 257 | E9Q557\|DESP\_MOUSE | 61.14 | 1 | 0 | 0 | 1 | 0 | 0 | 0 | 0 |  |  | 1.0495E5 |  |  |  |  | 1 | 1 | 0 | 0 | 1 | 0 | 0 | 0 | 0 | N | 332913 | Desmoplakin OS=Mus musculus OX=10090 GN=Dsp PE=1 SV=1 |
| 134 | 269 | P15924\|DESP\_HUMAN | 61.14 | 1 | 0 | 0 | 1 | 0 | 0 | 0 | 0 |  |  | 1.0495E5 |  |  |  |  | 1 | 1 | 0 | 0 | 1 | 0 | 0 | 0 | 0 | N | 331774 | Desmoplakin OS=Homo sapiens OX=9606 GN=DSP PE=1 SV=3 |
| 161 | 733 | P06702\|S10A9\_HUMAN | 60.66 | 13 | 13 | 0 | 0 | 0 | 0 | 0 | 0 | 1.7372E5 |  |  |  |  |  |  | 1 | 1 | 1 | 0 | 0 | 0 | 0 | 0 | 0 | N | 13242 | Protein S100-A9 OS=Homo sapiens OX=9606 GN=S100A9 PE=1 SV=1 |
| 175 | 2291 | Q5T749\|KPRP\_HUMAN | 58.89 | 4 | 4 | 0 | 0 | 0 | 0 | 0 | 0 | 2.8275E5 |  |  |  |  |  |  | 1 | 1 | 1 | 0 | 0 | 0 | 0 | 0 | 0 | Y | 64136 | Keratinocyte proline-rich protein OS=Homo sapiens OX=9606 GN=KPRP PE=1 SV=1 |
| 117 | 428 | Q5D862\|FILA2\_HUMAN | 57.75 | 0 | 0 | 0 | 0 | 0 | 0 | 0 | 0 | 3.9196E5 |  | 1.3817E5 |  |  |  |  | 1 | 1 | 1 | 0 | 1 | 0 | 0 | 0 | 0 | N | 248072 | Filaggrin-2 OS=Homo sapiens OX=9606 GN=FLG2 PE=1 SV=1 |
| 67 | 343 | P07477\|TRY1\_HUMAN | 57.43 | 4 | 4 | 4 | 4 | 4 | 4 | 4 | 4 | 2.7418E9 | 1.8884E8 | 5.1849E7 | 7.4928E7 | 7.7897E7 | 6.0169E7 | 4.1853E7 | 1 | 1 | 3 | 1 | 2 | 2 | 2 | 1 | 1 | Y | 26558 | Serine protease 1 OS=Homo sapiens OX=9606 GN=PRSS1 PE=1 SV=1 |
| 67 | 862 | P00763\|TRY2\_RAT | 57.43 | 4 | 4 | 4 | 4 | 4 | 4 | 4 | 4 | 2.7418E9 | 1.8884E8 | 5.1849E7 | 7.4928E7 | 7.7897E7 | 6.0169E7 | 4.1853E7 | 1 | 1 | 3 | 1 | 2 | 2 | 2 | 1 | 1 | Y | 26228 | Anionic trypsin-2 OS=Rattus norvegicus OX=10116 GN=Prss2 PE=1 SV=2 |
| 165 | 2301 | TRINITY\_DN3717\_c3\_g1\_i1.p1 | 57.17 | 6 | 0 | 0 | 0 | 0 | 0 | 6 | 0 |  |  |  |  |  | 1.1528E5 |  | 1 | 1 | 0 | 0 | 0 | 0 | 0 | 1 | 0 | N | 24552 | TRINITY\_DN3717\_c3\_g1\_i1.p1 |
| 131 | 2296 | Q8NHM4\|TRY6\_HUMAN | 56.51 | 13 | 13 | 0 | 0 | 0 | 0 | 0 | 0 | 1.6515E6 |  |  |  |  |  |  | 1 | 1 | 2 | 0 | 0 | 0 | 0 | 0 | 0 | Y | 26537 | Putative trypsin-6 OS=Homo sapiens OX=9606 GN=PRSS3P2 PE=5 SV=2 |
| 94 | 2309 | Q28417\|CASK\_GIRCA | 56.05 | 7 | 7 | 7 | 0 | 7 | 7 | 7 | 0 | 2.3545E5 | 1.2772E5 |  | 7.2711E4 | 2.7997E5 | 2.3126E5 |  | 1 | 1 | 1 | 1 | 0 | 1 | 1 | 1 | 0 | N | 17096 | Kappa-casein (Fragment) OS=Giraffa camelopardalis OX=9894 GN=CSN3 PE=2 SV=1 |
| 94 | 2310 | P11840\|CASK\_BUBBU | 56.05 | 5 | 5 | 5 | 0 | 5 | 5 | 5 | 0 | 2.3545E5 | 1.2772E5 |  | 7.2711E4 | 2.7997E5 | 2.3126E5 |  | 1 | 1 | 1 | 1 | 0 | 1 | 1 | 1 | 0 | N | 21398 | Kappa-casein OS=Bubalus bubalis OX=89462 GN=CSN3 PE=1 SV=2 |
| 94 | 2311 | P02668\|CASK\_BOVIN | 56.05 | 5 | 5 | 5 | 0 | 5 | 5 | 5 | 0 | 2.3545E5 | 1.2772E5 |  | 7.2711E4 | 2.7997E5 | 2.3126E5 |  | 1 | 1 | 1 | 1 | 0 | 1 | 1 | 1 | 0 | N | 21269 | Kappa-casein OS=Bos taurus OX=9913 GN=CSN3 PE=1 SV=1 |
| 94 | 2313 | P50420\|CASK\_CAPSU | 56.05 | 5 | 5 | 5 | 0 | 5 | 5 | 5 | 0 | 2.3545E5 | 1.2772E5 |  | 7.2711E4 | 2.7997E5 | 2.3126E5 |  | 1 | 1 | 1 | 1 | 0 | 1 | 1 | 1 | 0 | N | 21513 | Kappa-casein OS=Capricornis sumatraensis OX=34865 GN=CSN3 PE=2 SV=1 |
| 94 | 2314 | P50423\|CASK\_OREAM | 56.05 | 5 | 5 | 5 | 0 | 5 | 5 | 5 | 0 | 2.3545E5 | 1.2772E5 |  | 7.2711E4 | 2.7997E5 | 2.3126E5 |  | 1 | 1 | 1 | 1 | 0 | 1 | 1 | 1 | 0 | N | 21525 | Kappa-casein OS=Oreamnos americanus OX=34873 GN=CSN3 PE=2 SV=1 |
| 94 | 2315 | P50422\|CASK\_NEMGO | 56.05 | 5 | 5 | 5 | 0 | 5 | 5 | 5 | 0 | 2.3545E5 | 1.2772E5 |  | 7.2711E4 | 2.7997E5 | 2.3126E5 |  | 1 | 1 | 1 | 1 | 0 | 1 | 1 | 1 | 0 | N | 21485 | Kappa-casein OS=Naemorhedus goral OX=34871 GN=CSN3 PE=2 SV=1 |
| 94 | 2316 | P50421\|CASK\_CAPSW | 56.05 | 5 | 5 | 5 | 0 | 5 | 5 | 5 | 0 | 2.3545E5 | 1.2772E5 |  | 7.2711E4 | 2.7997E5 | 2.3126E5 |  | 1 | 1 | 1 | 1 | 0 | 1 | 1 | 1 | 0 | N | 21576 | Kappa-casein OS=Capricornis swinhoei OX=34866 GN=CSN3 PE=2 SV=1 |
| 94 | 2317 | P02670\|CASK\_CAPHI | 56.05 | 5 | 5 | 5 | 0 | 5 | 5 | 5 | 0 | 2.3545E5 | 1.2772E5 |  | 7.2711E4 | 2.7997E5 | 2.3126E5 |  | 1 | 1 | 1 | 1 | 0 | 1 | 1 | 1 | 0 | N | 21441 | Kappa-casein OS=Capra hircus OX=9925 GN=CSN3 PE=1 SV=2 |
| 94 | 2318 | P50424\|CASK\_RUPRU | 56.05 | 5 | 5 | 5 | 0 | 5 | 5 | 5 | 0 | 2.3545E5 | 1.2772E5 |  | 7.2711E4 | 2.7997E5 | 2.3126E5 |  | 1 | 1 | 1 | 1 | 0 | 1 | 1 | 1 | 0 | N | 21442 | Kappa-casein OS=Rupicapra rupicapra OX=34869 GN=CSN3 PE=2 SV=1 |
| 94 | 2319 | P42156\|CASK\_CAPCR | 56.05 | 5 | 5 | 5 | 0 | 5 | 5 | 5 | 0 | 2.3545E5 | 1.2772E5 |  | 7.2711E4 | 2.7997E5 | 2.3126E5 |  | 1 | 1 | 1 | 1 | 0 | 1 | 1 | 1 | 0 | N | 21486 | Kappa-casein OS=Capricornis crispus OX=9966 GN=CSN3 PE=2 SV=1 |
| 94 | 2320 | P02669\|CASK\_SHEEP | 56.05 | 5 | 5 | 5 | 0 | 5 | 5 | 5 | 0 | 2.3545E5 | 1.2772E5 |  | 7.2711E4 | 2.7997E5 | 2.3126E5 |  | 1 | 1 | 1 | 1 | 0 | 1 | 1 | 1 | 0 | N | 21438 | Kappa-casein OS=Ovis aries OX=9940 GN=CSN3 PE=1 SV=2 |
| 94 | 2321 | P50425\|CASK\_SAITA | 56.05 | 5 | 5 | 5 | 0 | 5 | 5 | 5 | 0 | 2.3545E5 | 1.2772E5 |  | 7.2711E4 | 2.7997E5 | 2.3126E5 |  | 1 | 1 | 1 | 1 | 0 | 1 | 1 | 1 | 0 | N | 22656 | Kappa-casein OS=Saiga tatarica OX=34875 GN=CSN3 PE=2 SV=1 |
| 176 | 2324 | TRINITY\_DN1101\_c0\_g1\_i12.p1 | 56.04 | 2 | 0 | 0 | 2 | 0 | 0 | 0 | 0 |  |  | 1.0731E5 |  |  |  |  | 1 | 1 | 0 | 0 | 1 | 0 | 0 | 0 | 0 | N | 58056 | TRINITY\_DN1101\_c0\_g1\_i12.p1 |
| 176 | 2325 | TRINITY\_DN1101\_c0\_g1\_i9.p1 | 56.04 | 2 | 0 | 0 | 2 | 0 | 0 | 0 | 0 |  |  | 1.0731E5 |  |  |  |  | 1 | 1 | 0 | 0 | 1 | 0 | 0 | 0 | 0 | N | 58056 | TRINITY\_DN1101\_c0\_g1\_i9.p1 |
| 176 | 2326 | TRINITY\_DN1101\_c0\_g1\_i15.p1 | 56.04 | 2 | 0 | 0 | 2 | 0 | 0 | 0 | 0 |  |  | 1.0731E5 |  |  |  |  | 1 | 1 | 0 | 0 | 1 | 0 | 0 | 0 | 0 | N | 58056 | TRINITY\_DN1101\_c0\_g1\_i15.p1 |
| 176 | 2327 | TRINITY\_DN1101\_c0\_g1\_i17.p1 | 56.04 | 2 | 0 | 0 | 2 | 0 | 0 | 0 | 0 |  |  | 1.0731E5 |  |  |  |  | 1 | 1 | 0 | 0 | 1 | 0 | 0 | 0 | 0 | N | 58056 | TRINITY\_DN1101\_c0\_g1\_i17.p1 |
| 176 | 2328 | TRINITY\_DN1101\_c0\_g1\_i11.p1 | 56.04 | 2 | 0 | 0 | 2 | 0 | 0 | 0 | 0 |  |  | 1.0731E5 |  |  |  |  | 1 | 1 | 0 | 0 | 1 | 0 | 0 | 0 | 0 | N | 58056 | TRINITY\_DN1101\_c0\_g1\_i11.p1 |
| 97 | 656 | Q06833\|NVJ2\_YEAST | 56.04 | 2 | 0 | 0 | 0 | 2 | 2 | 0 | 0 |  |  |  | 4.1763E5 | 1.3375E6 |  |  | 1 | 1 | 0 | 0 | 0 | 2 | 2 | 0 | 0 | N | 87324 | Nucleus-vacuole junction protein 2 OS=Saccharomyces cerevisiae (strain ATCC 204508 / S288c) OX=559292 GN=NVJ2 PE=1 SV=1 |
| 163 | 859 | TRINITY\_DN13586\_c0\_g1\_i1.p1 | 52.95 | 5 | 0 | 0 | 0 | 0 | 0 | 0 | 5 |  |  |  |  |  |  | 3.3602E5 | 1 | 1 | 0 | 0 | 0 | 0 | 0 | 0 | 1 | N | 30008 | TRINITY\_DN13586\_c0\_g1\_i1.p1 |
| 122 | 759 | P02075\|HBB\_SHEEP | 50.77 | 10 | 0 | 0 | 0 | 0 | 10 | 0 | 0 |  |  |  |  | 1.068E5 |  |  | 1 | 1 | 0 | 0 | 0 | 0 | 1 | 0 | 0 | N | 16073 | Hemoglobin subunit beta OS=Ovis aries OX=9940 GN=HBB PE=1 SV=2 |
| 122 | 760 | P18982\|HBB\_AILFU | 50.77 | 10 | 0 | 0 | 0 | 0 | 10 | 0 | 0 |  |  |  |  | 1.068E5 |  |  | 1 | 1 | 0 | 0 | 0 | 0 | 1 | 0 | 0 | N | 15950 | Hemoglobin subunit beta OS=Ailurus fulgens OX=9649 GN=HBB PE=1 SV=1 |
| 122 | 761 | B3EWD0\|HBB\_OTOBE | 50.77 | 10 | 0 | 0 | 0 | 0 | 10 | 0 | 0 |  |  |  |  | 1.068E5 |  |  | 1 | 1 | 0 | 0 | 0 | 0 | 1 | 0 | 0 | N | 15825 | Hemoglobin subunit beta OS=Otospermophilus beecheyi OX=34862 PE=1 SV=1 |
| 122 | 762 | P68044\|HBB\_MUSPF | 50.77 | 10 | 0 | 0 | 0 | 0 | 10 | 0 | 0 |  |  |  |  | 1.068E5 |  |  | 1 | 1 | 0 | 0 | 0 | 0 | 1 | 0 | 0 | N | 15994 | Hemoglobin subunit beta OS=Mustela putorius furo OX=9669 GN=HBB PE=1 SV=1 |
| 122 | 763 | P20855\|HBB\_CTEGU | 50.77 | 10 | 0 | 0 | 0 | 0 | 10 | 0 | 0 |  |  |  |  | 1.068E5 |  |  | 1 | 1 | 0 | 0 | 0 | 0 | 1 | 0 | 0 | N | 15846 | Hemoglobin subunit beta OS=Ctenodactylus gundi OX=10166 GN=HBB PE=1 SV=1 |
| 122 | 764 | P02087\|HBB\_DASNO | 50.77 | 10 | 0 | 0 | 0 | 0 | 10 | 0 | 0 |  |  |  |  | 1.068E5 |  |  | 1 | 1 | 0 | 0 | 0 | 0 | 1 | 0 | 0 | N | 16351 | Hemoglobin subunit beta OS=Dasypus novemcinctus OX=9361 GN=HBB PE=1 SV=3 |
| 122 | 765 | P19886\|HBD\_COLPO | 50.77 | 10 | 0 | 0 | 0 | 0 | 10 | 0 | 0 |  |  |  |  | 1.068E5 |  |  | 1 | 1 | 0 | 0 | 0 | 0 | 1 | 0 | 0 | N | 15934 | Hemoglobin subunit delta OS=Colobus polykomos OX=9572 GN=HBD PE=2 SV=2 |
| 122 | 766 | P61773\|HBD\_GORGO | 50.77 | 10 | 0 | 0 | 0 | 0 | 10 | 0 | 0 |  |  |  |  | 1.068E5 |  |  | 1 | 1 | 0 | 0 | 0 | 0 | 1 | 0 | 0 | N | 16023 | Hemoglobin subunit delta OS=Gorilla gorilla gorilla OX=9595 GN=HBD PE=1 SV=2 |
| 122 | 767 | P68232\|HBB\_ATEGE | 50.77 | 10 | 0 | 0 | 0 | 0 | 10 | 0 | 0 |  |  |  |  | 1.068E5 |  |  | 1 | 1 | 0 | 0 | 0 | 0 | 1 | 0 | 0 | N | 16044 | Hemoglobin subunit beta OS=Ateles geoffroyi OX=9509 GN=HBB PE=1 SV=2 |
| 122 | 2394 | P60524\|HBB\_CANLF | 50.77 | 10 | 0 | 0 | 0 | 0 | 10 | 0 | 0 |  |  |  |  | 1.068E5 |  |  | 1 | 1 | 0 | 0 | 0 | 0 | 1 | 0 | 0 | N | 15996 | Hemoglobin subunit beta OS=Canis lupus familiaris OX=9615 GN=HBB PE=1 SV=1 |
| 122 | 2395 | P60525\|HBB\_CANLA | 50.77 | 10 | 0 | 0 | 0 | 0 | 10 | 0 | 0 |  |  |  |  | 1.068E5 |  |  | 1 | 1 | 0 | 0 | 0 | 0 | 1 | 0 | 0 | N | 15996 | Hemoglobin subunit beta OS=Canis latrans OX=9614 GN=HBB PE=1 SV=1 |
| 122 | 2396 | P02052\|HBB\_TUPGL | 50.77 | 10 | 0 | 0 | 0 | 0 | 10 | 0 | 0 |  |  |  |  | 1.068E5 |  |  | 1 | 1 | 0 | 0 | 0 | 0 | 1 | 0 | 0 | N | 15855 | Hemoglobin subunit beta OS=Tupaia glis OX=9395 GN=HBB PE=1 SV=1 |
| 111 | 2403 | Q25C93\|FEZF1\_DANRE | 48.20 | 2 | 0 | 0 | 0 | 0 | 0 | 0 | 2 |  |  |  |  |  |  | 5.436E5 | 1 | 1 | 0 | 0 | 0 | 0 | 0 | 0 | 1 | Y | 47395 | Fez family zinc finger protein 1 OS=Danio rerio OX=7955 GN=fezf1 PE=2 SV=1 |
| 170 | 968 | P29508\|SPB3\_HUMAN | 48.14 | 4 | 4 | 0 | 0 | 0 | 0 | 0 | 0 | 2.3427E5 |  |  |  |  |  |  | 1 | 1 | 1 | 0 | 0 | 0 | 0 | 0 | 0 | N | 44565 | Serpin B3 OS=Homo sapiens OX=9606 GN=SERPINB3 PE=1 SV=2 |
| 119 | 364 | TRINITY\_DN4582\_c0\_g1\_i1.p1 | 47.43 | 2 | 0 | 2 | 0 | 0 | 0 | 0 | 0 |  | 4.7244E6 |  |  |  |  |  | 1 | 1 | 0 | 1 | 0 | 0 | 0 | 0 | 0 | N | 68364 | TRINITY\_DN4582\_c0\_g1\_i1.p1 |
| 119 | 365 | TRINITY\_DN4582\_c0\_g1\_i2.p1 | 47.43 | 2 | 0 | 2 | 0 | 0 | 0 | 0 | 0 |  | 4.7244E6 |  |  |  |  |  | 1 | 1 | 0 | 1 | 0 | 0 | 0 | 0 | 0 | N | 69490 | TRINITY\_DN4582\_c0\_g1\_i2.p1 |
| total 266 proteins |  |  |  |  |  |  |  |  |  |  |  |  |  |  |  |  |  |  |  |  |  |  |  |  |  |  |  |  |  |  |
| --- | --- | --- | --- | --- | --- | --- | --- | --- | --- | --- | --- | --- | --- | --- | --- | --- | --- | --- | --- | --- | --- | --- | --- | --- | --- | --- | --- | --- | --- | --- |

  

TRINITY\_DN4276\_c0\_g1\_i1.p1

back to list

  

| Protein Coverage
| Supporting Peptides
|

Protein Coverage:

Supporting Peptides:

| Peptide | Uniq | -10lgP | Mass | Length | ppm | m/z | z | RT | Fraction | Scan | Source File | Area F1 | Area F2 | Area F3 | Area F4 | Area F5 | Area F6 | Area F7 | #Feature | #Feature F1 | #Feature F2 | #Feature F3 | #Feature F4 | #Feature F5 | #Feature F6 | #Feature F7 | Start | End | PTM | AScore | Found By |
| --- | --- | --- | --- | --- | --- | --- | --- | --- | --- | --- | --- | --- | --- | --- | --- | --- | --- | --- | --- | --- | --- | --- | --- | --- | --- | --- | --- | --- | --- | --- | --- |
| R.NVLHTQSSDILRGPTLIDVNC(+57.02)GTDNVC(+57.02)LKNEDVIHTVIR.L | Y | 132.10 | 4415.2324 | 39 | 4.1 | 884.0574 | 5 | 64.10 | 1 | F1:17835 | Fraction\_1\_23092022r.raw | 7.1965E7 | 3.7026E6 |  |  |  |  |  | 5 | 3 | 2 | 0 | 0 | 0 | 0 | 0 | 64 | 102 | Carbamidomethylation | C21:Carbamidomethylation:1000.00;C27:Carbamidomethylation:1000.00 | PEAKS DB |
| R.GPTLIDVNC(+57.02)GTDNVC(+57.02)LKNEDVIHTVIR.L | Y | 123.58 | 3051.5117 | 27 | 0.2 | 1018.1780 | 3 | 62.49 | 1 | F1:17111 | Fraction\_1\_23092022r.raw | 1.6056E8 | 2.1669E6 |  |  |  |  |  | 4 | 2 | 2 | 0 | 0 | 0 | 0 | 0 | 76 | 102 | Carbamidomethylation | C9:Carbamidomethylation:1000.00;C15:Carbamidomethylation:1000.00 | PEAKS DB |
| R.IVKDSDAKLIDC(+57.02)YELGDDTEWFGGPQLR.Y | Y | 112.84 | 3239.5444 | 28 | 0.3 | 1080.8557 | 3 | 70.20 | 1 | F1:20607 | Fraction\_1\_23092022r.raw | 8.1223E7 | 1.4069E6 |  |  |  |  |  | 4 | 3 | 1 | 0 | 0 | 0 | 0 | 0 | 115 | 142 | Carbamidomethylation | C12:Carbamidomethylation:1000.00 | PEAKS DB |
| R.GPTLIDVNC(+57.02)GTDNVC(+57.02)LK.N | Y | 107.75 | 1874.8866 | 17 | 1.0 | 938.4515 | 2 | 57.48 | 1 | F1:14848 | Fraction\_1\_23092022r.raw | 3.3406E7 |  |  |  |  |  |  | 1 | 1 | 0 | 0 | 0 | 0 | 0 | 0 | 76 | 92 | Carbamidomethylation | C9:Carbamidomethylation:1000.00;C15:Carbamidomethylation:1000.00 | PEAKS DB |
| K.YTDLHADITPLILDRMNEC(+57.02)IK.T | Y | 107.49 | 2530.2559 | 21 | 0.1 | 844.4260 | 3 | 68.72 | 1 | F1:19973 | Fraction\_1\_23092022r.raw | 1.5863E8 |  |  |  |  |  |  | 7 | 7 | 0 | 0 | 0 | 0 | 0 | 0 | 549 | 569 | Carbamidomethylation | C19:Carbamidomethylation:1000.00 | PEAKS DB |
| R.GPTLIDVNC(+57.02)GTDNVC(+57.02)LKNEDVIHTVIRLSTNSDVINVSR.I | Y | 105.73 | 4337.1743 | 39 | 1.1 | 868.4431 | 5 | 76.73 | 1 | F1:23503 | Fraction\_1\_23092022r.raw | 3.6443E6 |  |  |  |  |  |  | 2 | 2 | 0 | 0 | 0 | 0 | 0 | 0 | 76 | 114 | Carbamidomethylation | C9:Carbamidomethylation:1000.00;C15:Carbamidomethylation:1000.00 | PEAKS DB |
| K.GTYIYVDMKDPLFLDQNNYM(+15.99)DKYIC(+57.02)LVAK.N | Y | 104.36 | 3545.6921 | 29 | 0.5 | 887.4308 | 4 | 77.18 | 1 | F1:23700 | Fraction\_1\_23092022r.raw | 1.9986E6 |  |  |  |  |  |  | 1 | 1 | 0 | 0 | 0 | 0 | 0 | 0 | 178 | 206 | Oxidation (M); Carbamidomethylation | M20:Oxidation (M):160.66;C25:Carbamidomethylation:1000.00 | PEAKS DB |
| K.YTDLHADITPLILDR.M | Y | 102.15 | 1754.9203 | 15 | 1.6 | 585.9816 | 3 | 65.87 | 1 | F1:18704 | Fraction\_1\_23092022r.raw | 4.1574E8 | 2.3241E6 |  |  |  |  |  | 9 | 7 | 2 | 0 | 0 | 0 | 0 | 0 | 549 | 563 |  |  | PEAKS DB |
| R.NVLHTQSSDILRGPTLIDVNC(+57.02)GTDNVC(+57.02)LK.N | Y | 101.83 | 3238.6074 | 29 | 0.0 | 810.6591 | 4 | 61.01 | 1 | F1:16464 | Fraction\_1\_23092022r.raw | 1.2859E7 |  |  |  |  |  |  | 1 | 1 | 0 | 0 | 0 | 0 | 0 | 0 | 64 | 92 | Carbamidomethylation | C21:Carbamidomethylation:1000.00;C27:Carbamidomethylation:1000.00 | PEAKS DB |
| K.DYVTALANNFDDNIEVR.V | Y | 97.99 | 1967.9225 | 17 | 0.4 | 984.9689 | 2 | 75.48 | 1 | F1:22928 | Fraction\_1\_23092022r.raw | 1.2398E9 | 1.5506E7 | 5.3382E5 | 2.1294E5 | 1.3799E5 |  |  | 13 | 8 | 2 | 1 | 1 | 1 | 0 | 0 | 429 | 445 |  |  | PEAKS DB |
| K.TGAPLNPPIWWVDPTDKTAHK.I | Y | 96.88 | 2343.2012 | 21 | 0.0 | 782.0743 | 3 | 65.11 | 1 | F1:18306 | Fraction\_1\_23092022r.raw | 2.0499E8 | 2.8895E5 |  |  |  |  |  | 5 | 4 | 1 | 0 | 0 | 0 | 0 | 0 | 570 | 590 |  |  | PEAKS DB |
| K.GTYIYVDMKDPLFLDQNNYM(+15.99)DK.Y | Y | 96.44 | 2698.2295 | 22 | 0.9 | 900.4179 | 3 | 72.90 | 1 | F1:21806 | Fraction\_1\_23092022r.raw | 1.7664E6 |  |  |  |  |  |  | 1 | 1 | 0 | 0 | 0 | 0 | 0 | 0 | 178 | 199 | Oxidation (M) | M20:Oxidation (M):181.38 | PEAKS DB |
| K.GTYIYVDM(+15.99)KDPLFLDQNNYM(+15.99)DKYIC(+57.02)LVAK.N | Y | 94.12 | 3561.6870 | 29 | 0.9 | 891.4298 | 4 | 71.47 | 1 | F1:21196 | Fraction\_1\_23092022r.raw | 1.0129E7 |  |  |  |  |  |  | 1 | 1 | 0 | 0 | 0 | 0 | 0 | 0 | 178 | 206 | Oxidation (M); Carbamidomethylation | M8:Oxidation (M):1000.00;M20:Oxidation (M):1000.00;C25:Carbamidomethylation:1000.00 | PEAKS DB |
| R.ESITLNYEIGIFSNPR.V | Y | 93.89 | 1851.9366 | 16 | 0.1 | 926.9757 | 2 | 82.16 | 3 | F3:26521 | Fraction\_3\_23092022.raw | 1.4576E8 | 2.064E7 | 4.7708E6 | 1.2075E6 |  |  |  | 12 | 5 | 2 | 3 | 2 | 0 | 0 | 0 | 215 | 230 |  |  | PEAKS DB |
| K.TGAPLNPPIWWVDPTDK.T | Y | 93.64 | 1905.9624 | 17 | 0.7 | 953.9891 | 2 | 81.96 | 1 | F1:25869 | Fraction\_1\_23092022r.raw | 6.395E7 |  |  |  |  |  |  | 1 | 1 | 0 | 0 | 0 | 0 | 0 | 0 | 570 | 586 |  |  | PEAKS DB |
| K.LIDC(+57.02)YELGDDTEWFGGPQLR.Y | Y | 92.96 | 2383.0791 | 20 | 1.7 | 1192.5488 | 2 | 81.00 | 1 | F1:25406 | Fraction\_1\_23092022r.raw | 4.5875E8 | 7.0609E6 | 8.0799E5 | 5.4813E5 | 3.9879E5 | 3.8792E5 |  | 11 | 5 | 2 | 1 | 1 | 1 | 1 | 0 | 123 | 142 | Carbamidomethylation | C4:Carbamidomethylation:1000.00 | PEAKS DB |
| R.NIKNAVVTIEINQNVATLR.I | Y | 92.57 | 2109.1907 | 19 | 0.4 | 1055.6030 | 2 | 58.38 | 1 | F1:15269 | Fraction\_1\_23092022r.raw | 2.6064E8 | 6.0471E6 | 2.1288E5 | 4.4515E5 | 2.0673E5 | 9.6067E5 |  | 13 | 7 | 2 | 1 | 1 | 1 | 1 | 0 | 32 | 50 |  |  | PEAKS DB |
| K.NAVVTIEINQNVATLR.I | Y | 92.35 | 1753.9686 | 16 | 0.5 | 877.9921 | 2 | 62.19 | 5 | F5:17872 | Fraction\_5\_23092022.raw | 3.2435E8 | 7.5827E6 | 8.6342E5 | 4.8287E5 | 2.4253E5 | 1.0314E6 |  | 12 | 7 | 1 | 1 | 1 | 1 | 1 | 0 | 35 | 50 |  |  | PEAKS DB |
| K.YTDLHADITPLILDRM(+15.99)NEC(+57.02)IK.T | Y | 91.55 | 2546.2510 | 21 | 3.0 | 637.5719 | 4 | 59.53 | 1 | F1:15860 | Fraction\_1\_23092022r.raw | 2.057E8 | 1.3968E6 |  |  |  |  |  | 14 | 12 | 2 | 0 | 0 | 0 | 0 | 0 | 549 | 569 | Oxidation (M); Carbamidomethylation | M16:Oxidation (M):1000.00;C19:Carbamidomethylation:1000.00 | PEAKS DB |
| K.NEDVIHTVIRLSTNSDVINVSR.I | Y | 89.48 | 2480.2983 | 22 | 0.1 | 621.0819 | 4 | 69.63 | 1 | F1:20333 | Fraction\_1\_23092022r.raw | 2.9184E6 |  |  |  |  |  |  | 2 | 2 | 0 | 0 | 0 | 0 | 0 | 0 | 93 | 114 |  |  | PEAKS DB |
| K.KYTDLHADITPLILDR.M | Y | 87.11 | 1883.0153 | 16 | 0.5 | 628.6793 | 3 | 59.02 | 1 | F1:15556 | Fraction\_1\_23092022r.raw | 3.9042E7 |  |  |  |  |  |  | 3 | 3 | 0 | 0 | 0 | 0 | 0 | 0 | 548 | 563 |  |  | PEAKS DB |
| K.KYTDLHADITPLILDRMNEC(+57.02)IK.T | Y | 87.01 | 2658.3508 | 22 | -0.1 | 532.6774 | 5 | 63.40 | 1 | F1:17525 | Fraction\_1\_23092022r.raw | 1.251E7 |  |  |  |  |  |  | 3 | 3 | 0 | 0 | 0 | 0 | 0 | 0 | 548 | 569 | Carbamidomethylation | C20:Carbamidomethylation:1000.00 | PEAKS DB |
| K.DSDAKLIDC(+57.02)YELGDDTEWFGGPQLR.Y | Y | 85.96 | 2899.2969 | 25 | 0.7 | 967.4402 | 3 | 78.81 | 1 | F1:24487 | Fraction\_1\_23092022r.raw | 1.2279E7 | 2.2383E5 |  |  |  |  |  | 2 | 1 | 1 | 0 | 0 | 0 | 0 | 0 | 118 | 142 | Carbamidomethylation | C9:Carbamidomethylation:1000.00 | PEAKS DB |
| K.DPLFLDQNNYM(+15.99)DKYIC(+57.02)LVAK.N | Y | 85.90 | 2475.1814 | 20 | 0.6 | 826.0682 | 3 | 72.05 | 1 | F1:21402 | Fraction\_1\_23092022r.raw | 2.7959E6 |  |  |  |  |  |  | 1 | 1 | 0 | 0 | 0 | 0 | 0 | 0 | 187 | 206 | Oxidation (M); Carbamidomethylation | M11:Oxidation (M):1000.00;C16:Carbamidomethylation:1000.00 | PEAKS DB |
| K.NC(+57.02)EPAYSTALNNSYFVK.S | Y | 83.72 | 1976.8938 | 17 | -0.4 | 989.4537 | 2 | 58.66 | 1 | F1:15393 | Fraction\_1\_23092022r.raw | 8.6203E6 |  |  |  |  |  |  | 2 | 2 | 0 | 0 | 0 | 0 | 0 | 0 | 338 | 354 | Carbamidomethylation | C2:Carbamidomethylation:1000.00 | PEAKS DB |
| K.GTYIYVDM(+15.99)KDPLFLDQNNYMDKYIC(+57.02)LVAK.N | Y | 83.44 | 3545.6921 | 29 | -0.4 | 887.4299 | 4 | 79.81 | 1 | F1:24902 | Fraction\_1\_23092022r.raw | 4.2438E6 |  |  |  |  |  |  | 1 | 1 | 0 | 0 | 0 | 0 | 0 | 0 | 178 | 206 | Oxidation (M); Carbamidomethylation | M8:Oxidation (M):89.82;C25:Carbamidomethylation:1000.00 | PEAKS DB |
| K.GTYIYVDM(+15.99)KDPLFLDQNNYMDK.Y | Y | 81.49 | 2698.2295 | 22 | 1.0 | 900.4180 | 3 | 68.03 | 1 | F1:19611 | Fraction\_1\_23092022r.raw | 1.5295E6 |  |  |  |  |  |  | 1 | 1 | 0 | 0 | 0 | 0 | 0 | 0 | 178 | 199 | Oxidation (M) | M8:Oxidation (M):98.81 | PEAKS DB |
| R.HRESITLNYEIGIFSNPR.V | Y | 77.95 | 2145.0967 | 18 | -0.8 | 716.0389 | 3 | 59.81 | 1 | F1:15959 | Fraction\_1\_23092022r.raw | 4.4706E7 | 2.4355E6 |  |  |  |  |  | 9 | 7 | 2 | 0 | 0 | 0 | 0 | 0 | 213 | 230 |  |  | PEAKS DB |
| R.GFRVTLWIHPFINK.N | Y | 77.54 | 1726.9670 | 14 | 0.3 | 432.7492 | 4 | 67.94 | 1 | F1:19560 | Fraction\_1\_23092022r.raw | 2.2518E6 |  |  |  |  |  |  | 2 | 2 | 0 | 0 | 0 | 0 | 0 | 0 | 324 | 337 |  |  | PEAKS DB |
| R.NVLHTQSSDILR.G | Y | 75.39 | 1381.7313 | 12 | -0.2 | 691.8728 | 2 | 36.08 | 1 | F1:5791 | Fraction\_1\_23092022r.raw | 1.1052E7 |  |  |  |  | 3.198E5 |  | 3 | 2 | 0 | 0 | 0 | 0 | 1 | 0 | 64 | 75 |  |  | PEAKS DB |
| R.YQHWPIQHM(+15.99)YYEEEPYVPTHPVNM(+15.99)AVAER.Y | Y | 73.92 | 3645.6445 | 29 | 0.2 | 730.1364 | 5 | 49.11 | 1 | F1:11234 | Fraction\_1\_23092022r.raw | 7.8258E6 |  |  |  |  |  |  | 2 | 2 | 0 | 0 | 0 | 0 | 0 | 0 | 143 | 171 | Oxidation (M) | M9:Oxidation (M):1000.00;M24:Oxidation (M):1000.00 | PEAKS DB |
| K.GTYIYVDM(+15.99)KDPLFLDQNNYM(+15.99)DK.Y | Y | 72.44 | 2714.2244 | 22 | 1.1 | 905.7498 | 3 | 64.10 | 1 | F1:17868 | Fraction\_1\_23092022r.raw | 4.6244E6 |  |  |  |  |  |  | 1 | 1 | 0 | 0 | 0 | 0 | 0 | 0 | 178 | 199 | Oxidation (M) | M8:Oxidation (M):1000.00;M20:Oxidation (M):1000.00 | PEAKS DB |
| R.VTLWIHPFINK.N | Y | 70.95 | 1366.7761 | 11 | 0.2 | 684.3955 | 2 | 64.95 | 1 | F1:18212 | Fraction\_1\_23092022r.raw | 8.5965E7 |  |  |  |  |  |  | 2 | 2 | 0 | 0 | 0 | 0 | 0 | 0 | 327 | 337 |  |  | PEAKS DB |
| R.LSTNSDVINVSR.I | Y | 70.90 | 1303.6732 | 12 | 1.3 | 652.8447 | 2 | 39.42 | 1 | F1:6965 | Fraction\_1\_23092022r.raw | 7.8193E7 |  |  |  |  | 2.1369E5 |  | 2 | 1 | 0 | 0 | 0 | 0 | 1 | 0 | 103 | 114 |  |  | PEAKS DB |
| R.LKLLEEIGIDSFK.F | Y | 70.20 | 1503.8547 | 13 | 2.7 | 502.2935 | 3 | 71.45 | 1 | F1:21117 | Fraction\_1\_23092022r.raw | 1.1777E7 |  |  |  |  |  |  | 2 | 2 | 0 | 0 | 0 | 0 | 0 | 0 | 388 | 400 |  |  | PEAKS DB |
| R.NSAGEDSC(+57.02)TRNVLHTQSSDILR.G | Y | 70.20 | 2459.1460 | 22 | 0.4 | 820.7229 | 3 | 37.53 | 1 | F1:6187 | Fraction\_1\_23092022r.raw | 1.204E6 |  |  |  |  |  |  | 1 | 1 | 0 | 0 | 0 | 0 | 0 | 0 | 54 | 75 | Carbamidomethylation | C8:Carbamidomethylation:1000.00 | PEAKS DB |
| R.LSTNSDVINVSRIVKDSDAK.L | Y | 70.04 | 2160.1387 | 20 | 4.0 | 541.0441 | 4 | 47.98 | 1 | F1:10647 | Fraction\_1\_23092022r.raw | 2.2844E7 | 6.6757E5 |  |  |  |  |  | 4 | 2 | 2 | 0 | 0 | 0 | 0 | 0 | 103 | 122 |  |  | PEAKS DB |
| R.YQHWPIQHM(+15.99)YYEEEPYVPTHPVNMAVAER.Y | Y | 69.71 | 3629.6497 | 29 | -0.4 | 726.9369 | 5 | 54.00 | 1 | F1:13328 | Fraction\_1\_23092022r.raw | 7.4695E6 |  |  |  |  |  |  | 2 | 2 | 0 | 0 | 0 | 0 | 0 | 0 | 143 | 171 | Oxidation (M) | M9:Oxidation (M):120.73 | PEAKS DB |
| K.IERNIKNAVVTIEINQNVATLR.I | Y | 69.61 | 2507.4182 | 22 | -0.2 | 627.8617 | 4 | 54.56 | 1 | F1:13593 | Fraction\_1\_23092022r.raw | 7.5001E5 |  |  |  |  |  |  | 1 | 1 | 0 | 0 | 0 | 0 | 0 | 0 | 29 | 50 |  |  | PEAKS DB |
| K.LLEEIGIDSFK.F | Y | 68.09 | 1262.6758 | 11 | 1.8 | 632.3463 | 2 | 68.08 | 1 | F1:19620 | Fraction\_1\_23092022r.raw | 3.418E7 | 1.6687E5 |  |  |  |  |  | 2 | 1 | 1 | 0 | 0 | 0 | 0 | 0 | 390 | 400 |  |  | PEAKS DB |
| K.KYTDLHADITPLILDRM(+15.99)NEC(+57.02)IK.T | Y | 66.42 | 2674.3457 | 22 | -0.3 | 892.4556 | 3 | 54.88 | 1 | F1:13724 | Fraction\_1\_23092022r.raw | 2.6479E6 |  |  |  |  |  |  | 2 | 2 | 0 | 0 | 0 | 0 | 0 | 0 | 548 | 569 | Oxidation (M); Carbamidomethylation | M17:Oxidation (M):1000.00;C20:Carbamidomethylation:1000.00 | PEAKS DB |
| R.NIKNAVVTIEINQNVATLRIC(+57.02)RNSAGEDSC(+57.02)TR.N | Y | 65.56 | 3615.8210 | 32 | 0.2 | 724.1716 | 5 | 54.03 | 1 | F1:13452 | Fraction\_1\_23092022r.raw | 3.8551E6 |  |  |  |  |  |  | 2 | 2 | 0 | 0 | 0 | 0 | 0 | 0 | 32 | 63 | Carbamidomethylation | C21:Carbamidomethylation:1000.00;C30:Carbamidomethylation:1000.00 | PEAKS DB |
| R.M(+15.99)IKHPIWSTWAR.Y | Y | 64.64 | 1540.7972 | 12 | -0.2 | 514.6063 | 3 | 43.60 | 1 | F1:8765 | Fraction\_1\_23092022r.raw | 3.1999E7 |  |  |  |  |  |  | 3 | 3 | 0 | 0 | 0 | 0 | 0 | 0 | 252 | 263 | Oxidation (M) | M1:Oxidation (M):1000.00 | PEAKS DB |
| R.MIKHPIWSTWAR.Y | Y | 64.56 | 1524.8024 | 12 | 0.0 | 509.2747 | 3 | 45.95 | 1 | F1:9719 | Fraction\_1\_23092022r.raw | 6.7723E6 |  |  |  |  |  |  | 1 | 1 | 0 | 0 | 0 | 0 | 0 | 0 | 252 | 263 |  |  | PEAKS DB |
| R.MNEC(+57.02)IKTGAPLNPPIWWVDPTDKTAHK.I | Y | 64.06 | 3118.5369 | 27 | -1.9 | 624.7134 | 5 | 62.27 | 1 | F1:17073 | Fraction\_1\_23092022r.raw | 1.5369E6 |  |  |  |  |  |  | 2 | 2 | 0 | 0 | 0 | 0 | 0 | 0 | 564 | 590 | Carbamidomethylation | C4:Carbamidomethylation:1000.00 | PEAKS DB |
| K.DPLFLDQNNYMDK.Y | Y | 63.27 | 1611.7239 | 13 | 0.5 | 806.8696 | 2 | 65.71 | 1 | F1:18588 | Fraction\_1\_23092022r.raw | 3.4432E5 |  |  |  |  |  |  | 1 | 1 | 0 | 0 | 0 | 0 | 0 | 0 | 187 | 199 |  |  | PEAKS DB |
| K.NEDVIHTVIR.L | Y | 58.26 | 1194.6356 | 10 | 0.2 | 598.3252 | 2 | 41.03 | 1 | F1:7672 | Fraction\_1\_23092022r.raw | 8.5911E6 |  |  |  |  |  |  | 2 | 2 | 0 | 0 | 0 | 0 | 0 | 0 | 93 | 102 |  |  | PEAKS DB |
| R.VGWRSQDLPIFVR.M | Y | 57.16 | 1571.8572 | 13 | 0.8 | 524.9601 | 3 | 63.62 | 1 | F1:17600 | Fraction\_1\_23092022r.raw | 9.1183E6 |  |  |  |  |  |  | 1 | 1 | 0 | 0 | 0 | 0 | 0 | 0 | 446 | 458 |  |  | PEAKS DB |
| R.NIKNAVVTIEINQNVATLRIC(+57.02)R.N | Y | 57.03 | 2538.4065 | 22 | 0.4 | 635.6091 | 4 | 60.02 | 1 | F1:16039 | Fraction\_1\_23092022r.raw | 9.9085E5 |  |  |  |  |  |  | 1 | 1 | 0 | 0 | 0 | 0 | 0 | 0 | 32 | 53 | Carbamidomethylation | C21:Carbamidomethylation:1000.00 | PEAKS DB |
| R.SKIERNIKNAVVTIEINQNVATLR.I | Y | 56.14 | 2722.5454 | 24 | 0.4 | 681.6439 | 4 | 53.05 | 1 | F1:12925 | Fraction\_1\_23092022r.raw | 2.3763E5 |  |  |  |  |  |  | 1 | 1 | 0 | 0 | 0 | 0 | 0 | 0 | 27 | 50 |  |  | PEAKS DB |
| K.HPIWSTWAR.Y | Y | 55.31 | 1152.5829 | 9 | 1.2 | 577.2994 | 2 | 47.86 | 1 | F1:10667 | Fraction\_1\_23092022r.raw | 5.1056E7 |  |  |  |  |  |  | 1 | 1 | 0 | 0 | 0 | 0 | 0 | 0 | 255 | 263 |  |  | PEAKS DB |
| R.SQDLPIFVR.M | Y | 54.07 | 1073.5869 | 9 | 1.2 | 537.8014 | 2 | 60.07 | 1 | F1:16014 | Fraction\_1\_23092022r.raw | 1.3682E9 | 3.9237E7 |  |  |  |  |  | 2 | 1 | 1 | 0 | 0 | 0 | 0 | 0 | 450 | 458 |  |  | PEAKS DB |
| K.GTYIYVDMKDPLFLDQNNYMDK.Y | Y | 53.17 | 2682.2346 | 22 | -0.3 | 895.0852 | 3 | 76.13 | 1 | F1:23253 | Fraction\_1\_23092022r.raw | 7.3672E5 |  |  |  |  |  |  | 1 | 1 | 0 | 0 | 0 | 0 | 0 | 0 | 178 | 199 |  |  | PEAKS DB |
| R.LSTNSDVINVSRIVK.D | Y | 48.64 | 1643.9207 | 15 | -0.7 | 548.9805 | 3 | 49.99 | 1 | F1:11545 | Fraction\_1\_23092022r.raw | 6.156E5 |  |  |  |  |  |  | 1 | 1 | 0 | 0 | 0 | 0 | 0 | 0 | 103 | 117 |  |  | PEAKS DB |
| K.NEDVIHTVIRLSTNSDVINVSRIVKDSDAK.L | Y | 48.51 | 3336.7637 | 30 | 0.5 | 668.3604 | 5 | 70.89 | 1 | F1:20918 | Fraction\_1\_23092022r.raw | 1.4819E5 |  |  |  |  |  |  | 1 | 1 | 0 | 0 | 0 | 0 | 0 | 0 | 93 | 122 |  |  | PEAKS DB |
| total 55 peptides |  |  |  |  |  |  |  |  |  |  |  |  |  |  |  |  |  |  |  |  |  |  |  |  |  |  |  |  |  |  |  |
| --- | --- | --- | --- | --- | --- | --- | --- | --- | --- | --- | --- | --- | --- | --- | --- | --- | --- | --- | --- | --- | --- | --- | --- | --- | --- | --- | --- | --- | --- | --- | --- |

P04264|K2C1\_HUMAN

back to list

  

| Protein Coverage
| Supporting Peptides
|

Protein Coverage:

Supporting Peptides:

| Peptide | Uniq | -10lgP | Mass | Length | ppm | m/z | z | RT | Fraction | Scan | Source File | Area F1 | Area F2 | Area F3 | Area F4 | Area F5 | Area F6 | Area F7 | #Feature | #Feature F1 | #Feature F2 | #Feature F3 | #Feature F4 | #Feature F5 | #Feature F6 | #Feature F7 | Start | End | PTM | AScore | Found By |
| --- | --- | --- | --- | --- | --- | --- | --- | --- | --- | --- | --- | --- | --- | --- | --- | --- | --- | --- | --- | --- | --- | --- | --- | --- | --- | --- | --- | --- | --- | --- | --- |
| R.SGGGGGRFSSC(+57.02)GGGGGSFGAGGGFGSR.S | Y | 118.80 | 2292.9678 | 27 | 0.6 | 765.3303 | 3 | 36.42 | 7 | F7:9109 | Fraction\_7\_23092022.raw | 5.0934E5 | 2.8449E6 |  | 1.2764E5 | 1.0065E5 | 1.5029E5 | 5.6802E5 | 6 | 1 | 1 | 0 | 1 | 1 | 1 | 1 | 39 | 65 | Carbamidomethylation | C11:Carbamidomethylation:1000.00 | PEAKS DB |
| K.SKAEAESLYQSKYEELQITAGR.H | Y | 108.66 | 2500.2444 | 22 | 0.2 | 834.4222 | 3 | 47.34 | 1 | F1:10360 | Fraction\_1\_23092022r.raw | 2.1498E7 | 9.2416E6 | 8.8604E6 | 1.3779E6 | 1.8892E6 | 8.9535E6 | 2.5166E6 | 13 | 2 | 2 | 2 | 2 | 2 | 2 | 1 | 365 | 386 |  |  | PEAKS DB |
| R.FSSC(+57.02)GGGGGSFGAGGGFGSR.S | Y | 105.96 | 1764.7274 | 20 | 1.0 | 883.3718 | 2 | 42.93 | 7 | F7:12066 | Fraction\_7\_23092022.raw | 1.8294E7 | 9.2408E6 | 4.9664E6 | 1.3448E6 | 9.6022E5 | 3.0926E6 | 1.4195E6 | 10 | 3 | 1 | 2 | 1 | 1 | 1 | 1 | 46 | 65 | Carbamidomethylation | C4:Carbamidomethylation:1000.00 | PEAKS DB |
| K.AEAESLYQSKYEELQITAGR.H | Y | 102.37 | 2285.1174 | 20 | 0.9 | 762.7138 | 3 | 52.63 | 3 | F3:13276 | Fraction\_3\_23092022.raw | 8.3304E6 | 2.3011E6 | 2.5869E6 |  |  | 1.8828E6 |  | 5 | 2 | 1 | 1 | 0 | 0 | 1 | 0 | 367 | 386 |  |  | PEAKS DB |
| R.FLEQQNQVLQTKWELLQQVDTSTR.T | N | 101.90 | 2931.5090 | 24 | 1.4 | 978.1783 | 3 | 70.89 | 1 | F1:20862 | Fraction\_1\_23092022r.raw | 1.545E7 | 6.6621E5 |  |  | 1.0796E5 | 5.2756E5 |  | 6 | 3 | 1 | 0 | 0 | 1 | 1 | 0 | 200 | 223 |  |  | PEAKS DB |
| R.THNLEPYFESFINNLR.R | Y | 100.49 | 1992.9694 | 16 | 0.2 | 665.3305 | 3 | 78.54 | 4 | F4:25449 | Fraction\_4\_23092022.raw | 7.8036E6 | 3.6542E6 | 9.2992E5 | 4.1137E5 |  | 1.6729E6 | 2.437E6 | 9 | 2 | 2 | 1 | 1 | 0 | 2 | 1 | 224 | 239 |  |  | PEAKS DB |
| R.SGYRSGGGFSSGSAGIINYQR.R | Y | 96.44 | 2120.0034 | 21 | -0.2 | 707.6749 | 3 | 41.81 | 7 | F7:11558 | Fraction\_7\_23092022.raw |  | 2.7805E5 |  |  |  |  | 3.3385E5 | 2 | 0 | 1 | 0 | 0 | 0 | 0 | 1 | 9 | 29 |  |  | PEAKS DB |
| R.TLLEGEESRM(+15.99)SGEC(+57.02)APNVSVSVSTSHTTISGGGSR.G | Y | 94.59 | 3594.6526 | 35 | 0.2 | 899.6706 | 4 | 44.22 | 1 | F1:8997 | Fraction\_1\_23092022r.raw | 1.8861E6 | 4.5726E5 |  |  |  |  |  | 2 | 1 | 1 | 0 | 0 | 0 | 0 | 0 | 484 | 518 | Oxidation (M); Carbamidomethylation | M10:Oxidation (M):1000.00;C14:Carbamidomethylation:1000.00 | PEAKS DB |
| R.MSGEC(+57.02)APNVSVSVSTSHTTISGGGSR.G | Y | 92.25 | 2564.1594 | 26 | 0.2 | 855.7273 | 3 | 40.30 | 1 | F1:7289 | Fraction\_1\_23092022r.raw | 3.5731E6 |  |  |  |  |  |  | 1 | 1 | 0 | 0 | 0 | 0 | 0 | 0 | 493 | 518 | Carbamidomethylation | C5:Carbamidomethylation:1000.00 | PEAKS DB |
| R.SGGGFSSGSAGIINYQRR.T | Y | 86.83 | 1812.8867 | 18 | -0.1 | 605.3028 | 3 | 39.49 | 7 | F7:10504 | Fraction\_7\_23092022.raw | 6.4703E5 | 3.1744E6 | 1.0298E5 | 6.2905E4 | 2.1742E5 | 5.9953E5 | 6.5816E5 | 8 | 1 | 2 | 1 | 1 | 1 | 1 | 1 | 13 | 30 |  |  | PEAKS DB |
| R.SGGGFSSGSAGIINYQR.R | Y | 85.81 | 1656.7855 | 17 | -0.1 | 829.4000 | 2 | 47.84 | 1 | F1:10583 | Fraction\_1\_23092022r.raw | 2.6904E6 | 1.9667E6 | 5.2027E5 |  | 2.7457E5 | 1.0091E6 | 3.7751E5 | 6 | 1 | 1 | 1 | 0 | 1 | 1 | 1 | 13 | 29 |  |  | PEAKS DB |
| R.M(+15.99)SGEC(+57.02)APNVSVSVSTSHTTISGGGSR.G | Y | 85.15 | 2580.1545 | 26 | 0.8 | 861.0594 | 3 | 38.02 | 1 | F1:6371 | Fraction\_1\_23092022r.raw | 1.0032E7 |  | 1.87E5 |  |  | 3.3246E5 | 7.0092E4 | 4 | 1 | 0 | 1 | 0 | 0 | 1 | 1 | 493 | 518 | Oxidation (M); Carbamidomethylation | M1:Oxidation (M):1000.00;C5:Carbamidomethylation:1000.00 | PEAKS DB |
| K.QISNLQQSISDAEQR.G | Y | 82.43 | 1715.8438 | 15 | 0.0 | 858.9291 | 2 | 47.35 | 1 | F1:10357 | Fraction\_1\_23092022r.raw | 1.3527E7 | 1.8835E6 | 1.3085E6 | 5.8787E5 | 6.9414E5 | 4.8023E5 | 2.3967E5 | 14 | 2 | 3 | 3 | 2 | 2 | 1 | 1 | 418 | 432 |  |  | PEAKS DB |
| R.SLVNLGGSKSISISVAR.G | Y | 82.21 | 1686.9628 | 17 | -0.5 | 563.3279 | 3 | 52.86 | 5 | F5:13654 | Fraction\_5\_23092022.raw | 7.302E6 | 9.2273E6 | 1.6252E6 | 1.2995E6 | 4.1436E6 | 3.6138E6 | 1.8729E6 | 12 | 2 | 2 | 2 | 1 | 2 | 2 | 1 | 66 | 82 |  |  | PEAKS DB |
| K.NKLNDLEDALQQAKEDLAR.L | Y | 81.44 | 2183.1182 | 19 | 0.3 | 728.7136 | 3 | 70.64 | 1 | F1:20835 | Fraction\_1\_23092022r.raw | 4.449E6 | 2.8934E5 |  |  |  | 1.7807E5 |  | 3 | 1 | 1 | 0 | 0 | 0 | 1 | 0 | 442 | 460 |  |  | PEAKS DB |
| R.GSYGSGGSSYGSGGGSYGSGGGGGGHGSYGSGSSSGGYR.G | Y | 81.12 | 3311.3008 | 39 | 0.5 | 1104.7748 | 3 | 27.54 | 7 | F7:5208 | Fraction\_7\_23092022.raw | 1.0342E6 |  |  |  |  |  | 8.5781E5 | 2 | 1 | 0 | 0 | 0 | 0 | 0 | 1 | 550 | 588 |  |  | PEAKS DB |
| K.NKLNDLEDALQQAK.E | Y | 80.05 | 1598.8264 | 14 | 0.1 | 800.4205 | 2 | 54.49 | 1 | F1:13565 | Fraction\_1\_23092022r.raw | 9.973E5 | 1.0003E5 | 1.1391E5 |  |  |  |  | 4 | 2 | 1 | 1 | 0 | 0 | 0 | 0 | 442 | 455 |  |  | PEAKS DB |
| K.LNDLEDALQQAK.E | Y | 79.46 | 1356.6885 | 12 | 0.3 | 679.3517 | 2 | 53.62 | 4 | F4:14237 | Fraction\_4\_23092022.raw | 6.0051E6 | 8.0494E6 | 1.159E7 | 2.6536E6 | 2.3375E6 | 3.0877E6 | 3.4363E6 | 7 | 1 | 1 | 1 | 1 | 1 | 1 | 1 | 444 | 455 |  |  | PEAKS DB |
| K.LALDLEIATYR.T | N | 77.60 | 1276.7026 | 11 | 0.4 | 639.3588 | 2 | 69.92 | 5 | F5:21320 | Fraction\_5\_23092022.raw | 1.0555E7 | 3.286E6 | 4.4833E6 |  | 9.0556E5 | 1.6127E6 | 1.5171E6 | 6 | 1 | 1 | 1 | 0 | 1 | 1 | 1 | 473 | 483 |  |  | PEAKS DB |
| R.THNLEPYFESFINNLRR.R | Y | 77.20 | 2149.0703 | 17 | 0.1 | 538.2749 | 4 | 73.02 | 1 | F1:21884 | Fraction\_1\_23092022r.raw | 5.1649E6 | 4.3233E6 |  |  |  | 1.3216E6 | 2.4858E6 | 8 | 2 | 2 | 0 | 0 | 0 | 2 | 2 | 224 | 240 |  |  | PEAKS DB |
| K.WELLQQVDTSTR.T | N | 75.54 | 1474.7416 | 12 | 0.4 | 738.3784 | 2 | 62.27 | 3 | F3:17590 | Fraction\_3\_23092022.raw | 4.3932E6 | 1.0276E6 | 7.5303E5 | 3.9044E5 | 1.2815E5 | 1.1274E6 | 2.2708E5 | 7 | 1 | 1 | 1 | 1 | 1 | 1 | 1 | 212 | 223 |  |  | PEAKS DB |
| K.SLNNQFASFIDKVR.F | N | 75.00 | 1637.8525 | 14 | 0.1 | 819.9336 | 2 | 64.93 | 1 | F1:18201 | Fraction\_1\_23092022r.raw | 6.8927E7 | 1.0717E7 | 1.3632E7 | 2.2062E6 | 1.9222E6 | 1.0131E7 | 7.4412E6 | 15 | 3 | 2 | 2 | 2 | 2 | 2 | 2 | 186 | 199 |  |  | PEAKS DB |
| R.EREQIKSLNNQFASFIDKVR.F | N | 73.50 | 2421.2764 | 20 | -0.5 | 606.3260 | 4 | 58.06 | 2 | F2:15327 | Fraction\_2\_23092022.raw |  | 8.092E5 |  |  |  |  |  | 1 | 0 | 1 | 0 | 0 | 0 | 0 | 0 | 180 | 199 |  |  | PEAKS DB |
| R.SGGGFSSGSAGIINYQRRTTSSSTR.R | Y | 73.33 | 2533.2268 | 25 | 0.6 | 634.3143 | 4 | 34.02 | 7 | F7:8077 | Fraction\_7\_23092022.raw |  |  |  |  |  |  | 7.9048E4 | 1 | 0 | 0 | 0 | 0 | 0 | 0 | 1 | 13 | 37 |  |  | PEAKS DB |
| K.AQYEDIAQKSKAEAESLYQSKYEELQITAGR.H | Y | 72.32 | 3546.7478 | 31 | 0.0 | 710.3568 | 5 | 62.42 | 1 | F1:17105 | Fraction\_1\_23092022r.raw | 6.9791E5 | 7.8389E5 |  |  |  |  |  | 3 | 1 | 2 | 0 | 0 | 0 | 0 | 0 | 356 | 386 |  |  | PEAKS DB |
| K.LALDLEIATYRTLLEGEESR.M | Y | 71.74 | 2291.2009 | 20 | 0.4 | 764.7412 | 3 | 82.43 | 1 | F1:26086 | Fraction\_1\_23092022r.raw | 2.5215E5 |  |  |  |  |  |  | 1 | 1 | 0 | 0 | 0 | 0 | 0 | 0 | 473 | 492 |  |  | PEAKS DB |
| R.TNAENEFVTIK.K | N | 71.61 | 1264.6299 | 11 | 0.8 | 633.3228 | 2 | 44.73 | 3 | F3:9705 | Fraction\_3\_23092022.raw | 4.5718E6 | 3.9495E6 | 5.4204E6 | 1.1702E6 | 8.1968E5 | 1.4631E6 | 6.4716E5 | 7 | 1 | 1 | 1 | 1 | 1 | 1 | 1 | 278 | 288 |  |  | PEAKS DB |
| K.QISNLQQSISDAEQRGENALKDAK.N | Y | 71.59 | 2642.3259 | 24 | 0.3 | 661.5889 | 4 | 61.65 | 1 | F1:16733 | Fraction\_1\_23092022r.raw | 1.5775E6 |  |  |  |  |  |  | 1 | 1 | 0 | 0 | 0 | 0 | 0 | 0 | 418 | 441 |  |  | PEAKS DB |
| K.SLNNQFASFIDK.V | N | 71.31 | 1382.6830 | 12 | -0.3 | 692.3486 | 2 | 63.00 | 1 | F1:17345 | Fraction\_1\_23092022r.raw | 5.9946E6 | 5.1364E5 | 8.2546E5 | 3.0075E5 |  | 3.8456E5 | 2.7708E5 | 6 | 1 | 1 | 1 | 1 | 0 | 1 | 1 | 186 | 197 |  |  | PEAKS DB |
| R.SGYRSGGGFSSGSAGIINYQRR.T | Y | 71.30 | 2276.1045 | 22 | 1.2 | 570.0341 | 4 | 36.14 | 7 | F7:9004 | Fraction\_7\_23092022.raw |  |  |  |  |  |  | 6.0926E5 | 1 | 0 | 0 | 0 | 0 | 0 | 0 | 1 | 9 | 30 |  |  | PEAKS DB |
| K.LNDLEDALQQAKEDLAR.L | Y | 70.67 | 1940.9802 | 17 | 1.9 | 971.4992 | 2 | 72.49 | 1 | F1:21615 | Fraction\_1\_23092022r.raw | 1.7355E6 | 1.5073E5 | 1.2303E5 |  |  | 1.4633E5 |  | 5 | 2 | 1 | 1 | 0 | 0 | 1 | 0 | 444 | 460 |  |  | PEAKS DB |
| K.AEAESLYQSKYEELQITAGRHGDSVR.N | Y | 69.50 | 2936.4263 | 26 | 0.6 | 588.2929 | 5 | 44.92 | 1 | F1:9313 | Fraction\_1\_23092022r.raw | 1.1507E5 | 3.7507E5 |  |  |  |  |  | 3 | 1 | 2 | 0 | 0 | 0 | 0 | 0 | 367 | 392 |  |  | PEAKS DB |
| R.FLEQQNQVLQTK.W | N | 69.06 | 1474.7780 | 12 | 0.5 | 738.3966 | 2 | 42.12 | 3 | F3:8546 | Fraction\_3\_23092022.raw | 8.0973E6 | 7.8535E5 | 1.0847E6 | 3.5575E5 | 2.39E5 | 3.7556E5 | 9.8393E4 | 7 | 1 | 1 | 1 | 1 | 1 | 1 | 1 | 200 | 211 |  |  | PEAKS DB |
| R.TNAENEFVTIKK.D | N | 68.00 | 1392.7249 | 12 | -0.5 | 465.2487 | 3 | 33.01 | 7 | F7:7601 | Fraction\_7\_23092022.raw |  |  |  |  |  | 9.7181E5 | 4.8108E5 | 4 | 0 | 0 | 0 | 0 | 0 | 2 | 2 | 278 | 289 |  |  | PEAKS DB |
| R.SLDLDSIIAEVK.A | N | 67.53 | 1301.7078 | 12 | -0.2 | 651.8610 | 2 | 80.92 | 4 | F4:26543 | Fraction\_4\_23092022.raw | 8.2236E7 | 5.1234E7 | 7.5638E7 | 2.6564E7 | 1.4738E7 | 3.5929E7 | 2.2646E7 | 15 | 2 | 3 | 4 | 1 | 1 | 3 | 1 | 344 | 355 |  |  | PEAKS DB |
| K.KQISNLQQSISDAEQR.G | Y | 66.43 | 1843.9387 | 16 | -0.5 | 615.6532 | 3 | 37.88 | 1 | F1:6339 | Fraction\_1\_23092022r.raw | 5.9913E5 |  |  |  |  |  |  | 1 | 1 | 0 | 0 | 0 | 0 | 0 | 0 | 417 | 432 |  |  | PEAKS DB |
| K.YEELQITAGR.H | N | 66.25 | 1178.5931 | 10 | 0.0 | 590.3038 | 2 | 43.49 | 1 | F1:8687 | Fraction\_1\_23092022r.raw | 4.6349E6 |  | 2.848E6 |  |  |  |  | 3 | 2 | 0 | 1 | 0 | 0 | 0 | 0 | 377 | 386 |  |  | PEAKS DB |
| R.SGGGFSSGSAGIINYQRRTTSSSTRR.S | Y | 64.71 | 2689.3281 | 26 | -0.2 | 673.3392 | 4 | 29.61 | 7 | F7:6110 | Fraction\_7\_23092022.raw |  |  |  |  |  |  | 2.026E5 | 2 | 0 | 0 | 0 | 0 | 0 | 0 | 2 | 13 | 38 |  |  | PEAKS DB |
| R.LDSELKNM(+15.99)QDM(+15.99)VEDYR.N | Y | 60.09 | 2016.8768 | 16 | 0.4 | 673.2998 | 3 | 36.31 | 1 | F1:5806 | Fraction\_1\_23092022r.raw | 6.035E5 |  |  |  |  |  |  | 1 | 1 | 0 | 0 | 0 | 0 | 0 | 0 | 252 | 267 | Oxidation (M) | M8:Oxidation (M):1000.00;M11:Oxidation (M):1000.00 | PEAKS DB |
| R.LLRDYQELMNTK.L | N | 57.86 | 1522.7814 | 12 | -0.8 | 508.6006 | 3 | 47.69 | 1 | F1:10527 | Fraction\_1\_23092022r.raw | 5.6309E5 |  |  |  |  |  |  | 1 | 1 | 0 | 0 | 0 | 0 | 0 | 0 | 461 | 472 |  |  | PEAKS DB |
| R.SLVNLGGSKSISISVARGGGR.G | Y | 56.80 | 2014.1283 | 21 | 0.4 | 504.5396 | 4 | 46.34 | 2 | F2:10048 | Fraction\_2\_23092022.raw | 2.8919E5 | 2.0375E5 |  |  |  |  |  | 2 | 1 | 1 | 0 | 0 | 0 | 0 | 0 | 66 | 86 |  |  | PEAKS DB |
| K.DVDGAYMTKVDLQAK.L | Y | 54.58 | 1652.8080 | 15 | -0.1 | 827.4112 | 2 | 47.08 | 1 | F1:10267 | Fraction\_1\_23092022r.raw | 4.5839E4 |  |  |  |  |  |  | 1 | 1 | 0 | 0 | 0 | 0 | 0 | 0 | 290 | 304 |  |  | PEAKS DB |
| R.DYQELMNTK.L | N | 53.32 | 1140.5121 | 9 | 0.8 | 571.2638 | 2 | 43.26 | 1 | F1:8611 | Fraction\_1\_23092022r.raw | 3.6715E4 |  |  |  |  |  |  | 1 | 1 | 0 | 0 | 0 | 0 | 0 | 0 | 464 | 472 |  |  | PEAKS DB |
| R.GGGGGGYGSGGSSYGSGGGSYGSGGGGGGGR.G | Y | 51.76 | 2382.9446 | 31 | 0.1 | 1192.4797 | 2 | 25.04 | 7 | F7:4209 | Fraction\_7\_23092022.raw |  |  |  |  |  |  | 4.0219E5 | 1 | 0 | 0 | 0 | 0 | 0 | 0 | 1 | 519 | 549 |  |  | PEAKS DB |
| R.NSKIEISELNRVIQR.L | Y | 51.54 | 1798.0061 | 15 | -0.3 | 600.3425 | 3 | 46.84 | 2 | F2:10259 | Fraction\_2\_23092022.raw | 1.7933E5 | 8.9786E5 |  |  |  |  |  | 2 | 1 | 1 | 0 | 0 | 0 | 0 | 0 | 393 | 407 |  |  | PEAKS DB |
| R.M(+15.99)SGEC(+57.02)APNVSVSVSTSHTTISGGGSRGGGGGGYGSGGSSYGSGGGSYGSGGGGGGGR.G | Y | 50.92 | 4945.0884 | 57 | 0.4 | 1237.2799 | 4 | 38.47 | 1 | F1:6582 | Fraction\_1\_23092022r.raw | 6.1732E5 |  |  |  |  |  |  | 1 | 1 | 0 | 0 | 0 | 0 | 0 | 0 | 493 | 549 | Oxidation (M); Carbamidomethylation | M1:Oxidation (M):1000.00;C5:Carbamidomethylation:1000.00 | PEAKS DB |
| R.SGYRSGGGFSSGSAGIINYQRRTTSSSTR.R | Y | 49.67 | 2996.4448 | 29 | 1.7 | 600.2972 | 5 | 32.11 | 7 | F7:7215 | Fraction\_7\_23092022.raw |  |  |  |  |  |  | 2.8886E4 | 1 | 0 | 0 | 0 | 0 | 0 | 0 | 1 | 9 | 37 |  |  | PEAKS DB |
| R.LRSEIDNVKKQISNLQQSISDAEQR.G | Y | 49.60 | 2898.5159 | 25 | 0.1 | 725.6363 | 4 | 62.20 | 1 | F1:17035 | Fraction\_1\_23092022r.raw | 4.1147E5 |  |  |  |  |  |  | 1 | 1 | 0 | 0 | 0 | 0 | 0 | 0 | 408 | 432 |  |  | PEAKS DB |
| K.QISNLQQSISDAEQRGENALK.D | Y | 48.85 | 2328.1670 | 21 | -0.4 | 777.0626 | 3 | 60.56 | 1 | F1:16275 | Fraction\_1\_23092022r.raw | 2.1124E6 |  |  |  |  |  |  | 1 | 1 | 0 | 0 | 0 | 0 | 0 | 0 | 418 | 438 |  |  | PEAKS DB |
| K.FVSTTYSGVTR | Y | 47.71 | 1216.6088 | 11 | 0.3 | 609.3118 | 2 | 35.32 | 7 | F7:8656 | Fraction\_7\_23092022.raw |  |  |  |  |  |  | 5.0361E4 | 1 | 0 | 0 | 0 | 0 | 0 | 0 | 1 | 634 | 644 |  |  | PEAKS DB |
| total 50 peptides |  |  |  |  |  |  |  |  |  |  |  |  |  |  |  |  |  |  |  |  |  |  |  |  |  |  |  |  |  |  |  |
| --- | --- | --- | --- | --- | --- | --- | --- | --- | --- | --- | --- | --- | --- | --- | --- | --- | --- | --- | --- | --- | --- | --- | --- | --- | --- | --- | --- | --- | --- | --- | --- |

TRINITY\_DN3450\_c0\_g1\_i51.p1

back to list

  

| Protein Coverage
| Supporting Peptides
|

Protein Coverage:

Supporting Peptides:

| Peptide | Uniq | -10lgP | Mass | Length | ppm | m/z | z | RT | Fraction | Scan | Source File | Area F1 | Area F2 | Area F3 | Area F4 | Area F5 | Area F6 | Area F7 | #Feature | #Feature F1 | #Feature F2 | #Feature F3 | #Feature F4 | #Feature F5 | #Feature F6 | #Feature F7 | Start | End | PTM | AScore | Found By |
| --- | --- | --- | --- | --- | --- | --- | --- | --- | --- | --- | --- | --- | --- | --- | --- | --- | --- | --- | --- | --- | --- | --- | --- | --- | --- | --- | --- | --- | --- | --- | --- |
| R.AIDHSHGIIYYLATGPGKPSQR.N | Y | 129.48 | 2380.2288 | 22 | 0.6 | 596.0648 | 4 | 45.92 | 7 | F7:13419 | Fraction\_7\_23092022.raw |  | 1.9371E5 | 1.3801E5 | 2.4405E6 | 3.3191E6 | 5.5043E6 | 2.4626E7 | 15 | 0 | 1 | 1 | 3 | 3 | 3 | 4 | 333 | 354 |  |  | PEAKS DB |
| R.NVQEILYLHYGEPGNLVDQYPTEVK.I | Y | 109.89 | 2917.4497 | 25 | 0.3 | 973.4908 | 3 | 70.28 | 4 | F4:21723 | Fraction\_4\_23092022.raw |  |  | 2.3239E5 | 2.3856E6 | 4.719E5 | 2.1779E5 | 1.5527E6 | 5 | 0 | 0 | 1 | 1 | 1 | 1 | 1 | 161 | 185 |  |  | PEAKS DB |
| K.APIELVGTDNVLSNVQWK.D | Y | 106.26 | 1982.0472 | 18 | 0.6 | 992.0315 | 2 | 75.90 | 6 | F6:24672 | Fraction\_6\_23092022.raw |  |  |  |  |  | 2.1479E5 |  | 1 | 0 | 0 | 0 | 0 | 0 | 1 | 0 | 217 | 234 |  |  | PEAKS DB |
| K.AGTSNPVVSLTLVDLHDPTLNKIDLK.A | Y | 105.10 | 2759.5068 | 26 | 1.0 | 690.8846 | 4 | 72.00 | 5 | F5:22224 | Fraction\_5\_23092022.raw |  |  | 2.0917E6 | 7.4661E6 | 2.1356E6 | 9.6734E5 |  | 7 | 0 | 0 | 2 | 2 | 2 | 1 | 0 | 191 | 216 |  |  | PEAKS DB |
| R.SSPIQM(+15.99)DLTPGVTEVQDIR.A | Y | 87.41 | 2101.0361 | 19 | 0.4 | 1051.5258 | 2 | 60.06 | 5 | F5:16927 | Fraction\_5\_23092022.raw |  |  |  |  | 6.4546E5 |  | 8.7982E5 | 2 | 0 | 0 | 0 | 0 | 1 | 0 | 1 | 314 | 332 | Oxidation (M) | M6:Oxidation (M):1000.00 | PEAKS DB |
| K.LGTVEVEDQISVTR.K | Y | 83.26 | 1544.8046 | 14 | 0.8 | 773.4102 | 2 | 50.53 | 7 | F7:15742 | Fraction\_7\_23092022.raw |  |  | 1.7203E6 |  |  |  | 4.6328E6 | 2 | 0 | 0 | 1 | 0 | 0 | 0 | 1 | 525 | 538 |  |  | PEAKS DB |
| K.AGTSNPVVSLTLVDLHDPTLNK.I | Y | 82.85 | 2290.2168 | 22 | 0.4 | 764.4131 | 3 | 69.52 | 3 | F3:20851 | Fraction\_3\_23092022.raw |  |  | 6.1456E5 | 1.8069E6 | 8.228E5 | 3.4027E5 |  | 4 | 0 | 0 | 1 | 1 | 1 | 1 | 0 | 191 | 212 |  |  | PEAKS DB |
| K.FM(+15.99)LIHGSGDDNVHYQQSLALAK.A | Y | 82.40 | 2459.1902 | 22 | 0.0 | 615.8048 | 4 | 49.19 | 6 | F6:12713 | Fraction\_6\_23092022.raw |  |  |  |  |  | 3.4937E6 |  | 1 | 0 | 0 | 0 | 0 | 0 | 1 | 0 | 629 | 650 | Oxidation (M) | M2:Oxidation (M):1000.00 | PEAKS DB |
| K.KFM(+15.99)LIHGSGDDNVHYQQSLALAK.A | Y | 80.97 | 2587.2852 | 23 | 0.1 | 647.8286 | 4 | 43.63 | 6 | F6:10156 | Fraction\_6\_23092022.raw |  |  |  |  |  | 2.4747E6 |  | 1 | 0 | 0 | 0 | 0 | 0 | 1 | 0 | 628 | 650 | Oxidation (M) | M3:Oxidation (M):1000.00 | PEAKS DB |
| K.LARKELPLVKDLYVR.A | Y | 78.96 | 1812.0985 | 15 | -0.8 | 454.0315 | 4 | 44.03 | 7 | F7:12606 | Fraction\_7\_23092022.raw |  |  |  |  | 1.7484E5 |  | 1.2509E7 | 4 | 0 | 0 | 0 | 0 | 1 | 0 | 3 | 431 | 445 |  |  | PEAKS DB |
| R.FHHVIRYEYTFR.S | Y | 76.33 | 1666.8368 | 12 | 0.3 | 417.7166 | 4 | 34.33 | 7 | F7:8215 | Fraction\_7\_23092022.raw |  |  |  |  |  |  | 3.6534E6 | 1 | 0 | 0 | 0 | 0 | 0 | 0 | 1 | 302 | 313 |  |  | PEAKS DB |
| R.SVIYAYIDGRGSSNK.G | Y | 75.39 | 1628.8158 | 15 | -0.1 | 815.4151 | 2 | 42.17 | 6 | F6:9530 | Fraction\_6\_23092022.raw |  |  | 1.6986E5 |  |  | 9.841E5 | 5.7092E5 | 4 | 0 | 0 | 1 | 0 | 0 | 2 | 1 | 499 | 513 |  |  | PEAKS DB |
| R.KLGTVEVEDQISVTR.K | Y | 74.25 | 1672.8995 | 15 | -0.6 | 558.6401 | 3 | 43.29 | 7 | F7:12220 | Fraction\_7\_23092022.raw |  |  | 2.0616E6 | 3.3199E5 | 1.7947E5 | 5.2975E6 | 3.9832E6 | 8 | 0 | 0 | 2 | 1 | 1 | 2 | 2 | 524 | 538 |  |  | PEAKS DB |
| K.VRLFLPHNFDESK.S | Y | 74.09 | 1600.8362 | 13 | -0.1 | 534.6193 | 3 | 46.90 | 7 | F7:13921 | Fraction\_7\_23092022.raw |  |  |  |  |  |  | 1.5592E6 | 2 | 0 | 0 | 0 | 0 | 0 | 0 | 2 | 453 | 465 |  |  | PEAKS DB |
| K.LGTVEVEDQISVTRK.L | Y | 74.00 | 1672.8995 | 15 | -0.1 | 558.6404 | 3 | 42.28 | 7 | F7:11780 | Fraction\_7\_23092022.raw |  |  | 1.9664E5 |  |  | 6.5498E5 | 3.3517E5 | 3 | 0 | 0 | 1 | 0 | 0 | 1 | 1 | 525 | 539 |  |  | PEAKS DB |
| R.YVIYDIELGGYDK.I | Y | 73.54 | 1546.7555 | 13 | 0.6 | 774.3855 | 2 | 69.66 | 6 | F6:21892 | Fraction\_6\_23092022.raw |  |  |  | 1.0394E5 |  | 4.332E5 | 9.399E5 | 3 | 0 | 0 | 0 | 1 | 0 | 1 | 1 | 59 | 71 |  |  | PEAKS DB |
| K.KIYSWENNLSLR.S | Y | 72.97 | 1521.7939 | 12 | 0.9 | 508.2724 | 3 | 50.52 | 7 | F7:15713 | Fraction\_7\_23092022.raw |  |  |  |  |  | 3.2213E5 | 1.097E6 | 2 | 0 | 0 | 0 | 0 | 0 | 1 | 1 | 417 | 428 |  |  | PEAKS DB |
| K.IYSWENNLSLR.S | Y | 72.69 | 1393.6990 | 11 | -0.1 | 697.8567 | 2 | 61.06 | 5 | F5:17355 | Fraction\_5\_23092022.raw |  |  |  | 1.3573E5 | 8.828E5 | 1.4541E6 | 4.0913E6 | 4 | 0 | 0 | 0 | 1 | 1 | 1 | 1 | 418 | 428 |  |  | PEAKS DB |
| M.TGDILQFDVIKQR.L | Y | 72.51 | 1531.8358 | 13 | 0.6 | 511.6195 | 3 | 58.23 | 4 | F4:16325 | Fraction\_4\_23092022.raw |  |  | 2.8091E5 | 1.9997E6 |  | 2.6038E6 | 1.1819E7 | 7 | 0 | 0 | 1 | 2 | 0 | 2 | 2 | 2 | 14 |  |  | PEAKS DB |
| R.KELPLVKDLYVR.A | Y | 71.14 | 1471.8762 | 12 | 0.4 | 368.9765 | 4 | 47.77 | 7 | F7:14316 | Fraction\_7\_23092022.raw |  |  | 9.6471E5 | 5.0971E5 | 3.2805E6 |  | 4.2881E7 | 5 | 0 | 0 | 1 | 1 | 1 | 0 | 2 | 434 | 445 |  |  | PEAKS DB |
| M.TGDILQFDVIK.Q | Y | 69.51 | 1247.6761 | 11 | 0.3 | 624.8455 | 2 | 69.45 | 4 | F4:21363 | Fraction\_4\_23092022.raw |  |  |  | 1.9069E5 |  | 2.2556E5 | 6.1063E5 | 3 | 0 | 0 | 0 | 1 | 0 | 1 | 1 | 2 | 12 |  |  | PEAKS DB |
| R.LFLPHNFDESK.S | Y | 64.70 | 1345.6666 | 11 | 0.1 | 449.5629 | 3 | 48.29 | 7 | F7:14587 | Fraction\_7\_23092022.raw |  |  | 3.5424E5 | 3.9359E5 | 1.7704E6 | 1.6064E6 | 1.1746E7 | 6 | 0 | 0 | 1 | 1 | 1 | 1 | 2 | 455 | 465 |  |  | PEAKS DB |
| K.DLYVRANGYESKVR.L | Y | 64.41 | 1668.8583 | 14 | -0.2 | 557.2933 | 3 | 32.49 | 7 | F7:7364 | Fraction\_7\_23092022.raw |  |  |  |  |  |  | 4.5572E5 | 1 | 0 | 0 | 0 | 0 | 0 | 0 | 1 | 441 | 454 |  |  | PEAKS DB |
| R.FSNNGFNDVQR.V | Y | 64.20 | 1296.5847 | 11 | -0.1 | 649.2996 | 2 | 33.82 | 7 | F7:7958 | Fraction\_7\_23092022.raw |  |  |  |  |  | 4.942E4 | 3.8972E5 | 2 | 0 | 0 | 0 | 0 | 0 | 1 | 1 | 103 | 113 |  |  | PEAKS DB |
| R.SVIYAYIDGRGSSNKGSK.M | Y | 63.42 | 1900.9642 | 18 | 0.5 | 634.6624 | 3 | 34.56 | 7 | F7:8264 | Fraction\_7\_23092022.raw |  |  |  |  |  | 8.7678E5 | 3.8227E6 | 4 | 0 | 0 | 0 | 0 | 0 | 1 | 3 | 499 | 516 |  |  | PEAKS DB |
| K.ELPLVKDLYVR.A | Y | 60.92 | 1343.7812 | 11 | -0.6 | 448.9341 | 3 | 60.72 | 7 | F7:21963 | Fraction\_7\_23092022.raw |  | 5.5529E4 | 7.5096E5 | 3.6436E5 | 3.1945E6 | 4.1851E6 | 5.7128E7 | 9 | 0 | 1 | 1 | 1 | 2 | 2 | 2 | 435 | 445 |  |  | PEAKS DB |
| K.GRFLLIGYDFQK.G | Y | 58.63 | 1455.7874 | 12 | 1.0 | 486.2702 | 3 | 63.64 | 7 | F7:23697 | Fraction\_7\_23092022.raw |  |  |  |  |  | 2.0583E5 | 2.8844E6 | 2 | 0 | 0 | 0 | 0 | 0 | 1 | 1 | 38 | 49 |  |  | PEAKS DB |
| R.KELPLVKDLYVRANGYESK.V | Y | 58.56 | 2221.2107 | 19 | -0.1 | 556.3099 | 4 | 48.72 | 7 | F7:14912 | Fraction\_7\_23092022.raw |  |  |  |  |  |  | 2.6876E6 | 1 | 0 | 0 | 0 | 0 | 0 | 0 | 1 | 434 | 452 |  |  | PEAKS DB |
| R.FLLIGYDFQK.G | Y | 56.95 | 1242.6648 | 10 | 0.5 | 622.3400 | 2 | 74.96 | 6 | F6:24216 | Fraction\_6\_23092022.raw |  |  | 2.8801E5 | 6.4509E5 | 3.5338E5 | 1.7797E6 |  | 4 | 0 | 0 | 1 | 1 | 1 | 1 | 0 | 40 | 49 |  |  | PEAKS DB |
| R.KLGTVEVEDQISVTRK.L | Y | 56.26 | 1800.9945 | 16 | -0.3 | 601.3386 | 3 | 36.67 | 6 | F6:7079 | Fraction\_6\_23092022.raw |  |  | 1.6683E5 |  |  | 5.7131E5 | 2.9486E5 | 3 | 0 | 0 | 1 | 0 | 0 | 1 | 1 | 524 | 539 |  |  | PEAKS DB |
| K.ELPLVKDLYVRANGYESK.V | Y | 53.03 | 2093.1157 | 18 | -0.5 | 698.7122 | 3 | 58.52 | 7 | F7:20710 | Fraction\_7\_23092022.raw |  |  |  |  |  |  | 1.0339E6 | 2 | 0 | 0 | 0 | 0 | 0 | 0 | 2 | 435 | 452 |  |  | PEAKS DB |
| R.FLLIGYDFQKGFR.H | Y | 48.70 | 1602.8558 | 13 | 0.0 | 535.2925 | 3 | 73.15 | 7 | F7:28562 | Fraction\_7\_23092022.raw |  |  |  |  |  |  | 3.0007E6 | 1 | 0 | 0 | 0 | 0 | 0 | 0 | 1 | 40 | 52 |  |  | PEAKS DB |
| R.KLQEM(+15.99)FPWIDSKR.T | Y | 47.47 | 1692.8657 | 13 | 0.4 | 565.2961 | 3 | 47.90 | 6 | F6:12164 | Fraction\_6\_23092022.raw |  |  |  |  |  | 4.657E5 |  | 1 | 0 | 0 | 0 | 0 | 0 | 1 | 0 | 539 | 551 | Oxidation (M) | M5:Oxidation (M):1000.00 | PEAKS DB |
| total 33 peptides |  |  |  |  |  |  |  |  |  |  |  |  |  |  |  |  |  |  |  |  |  |  |  |  |  |  |  |  |  |  |  |
| --- | --- | --- | --- | --- | --- | --- | --- | --- | --- | --- | --- | --- | --- | --- | --- | --- | --- | --- | --- | --- | --- | --- | --- | --- | --- | --- | --- | --- | --- | --- | --- |

P35527|K1C9\_HUMAN

back to list

  

| Protein Coverage
| Supporting Peptides
|

Protein Coverage:

Supporting Peptides:

| Peptide | Uniq | -10lgP | Mass | Length | ppm | m/z | z | RT | Fraction | Scan | Source File | Area F1 | Area F2 | Area F3 | Area F4 | Area F5 | Area F6 | Area F7 | #Feature | #Feature F1 | #Feature F2 | #Feature F3 | #Feature F4 | #Feature F5 | #Feature F6 | #Feature F7 | Start | End | PTM | AScore | Found By |
| --- | --- | --- | --- | --- | --- | --- | --- | --- | --- | --- | --- | --- | --- | --- | --- | --- | --- | --- | --- | --- | --- | --- | --- | --- | --- | --- | --- | --- | --- | --- | --- |
| R.GGGGSFGYSYGGGSGGGFSASSLGGGFGGGSR.G | Y | 127.32 | 2704.1538 | 32 | 0.8 | 1353.0852 | 2 | 64.22 | 2 | F2:18090 | Fraction\_2\_23092022.raw | 1.0449E7 | 1.2773E7 | 4.5417E6 | 1.5477E6 | 1.7544E6 | 6.6E6 | 3.9126E6 | 14 | 2 | 2 | 2 | 2 | 2 | 2 | 2 | 64 | 95 |  |  | PEAKS DB |
| R.GGSGGSHGGGSGFGGESGGSYGGGEEASGSGGGYGGGSGK.S | Y | 116.25 | 3222.2742 | 40 | 0.4 | 1075.0991 | 3 | 26.95 | 7 | F7:4975 | Fraction\_7\_23092022.raw |  |  |  |  | 2.2816E5 |  | 5.2951E5 | 2 | 0 | 0 | 0 | 0 | 1 | 0 | 1 | 580 | 619 |  |  | PEAKS DB |
| R.LASYLDKVQALEEANNDLENK.I | Y | 114.91 | 2376.1809 | 21 | 0.4 | 793.0679 | 3 | 62.34 | 1 | F1:17066 | Fraction\_1\_23092022r.raw | 8.3051E6 | 8.0962E5 | 6.241E5 |  |  | 6.6574E5 | 5.0998E5 | 6 | 2 | 1 | 1 | 0 | 0 | 1 | 1 | 164 | 184 |  |  | PEAKS DB |
| K.DIENQYETQITQIEHEVSSSGQEVQSSAK.E | Y | 105.54 | 3263.5066 | 29 | 0.6 | 1088.8435 | 3 | 74.64 | 3 | F3:23085 | Fraction\_3\_23092022.raw | 1.3801E7 | 5.2053E6 | 3.1999E6 | 5.8106E5 | 6.3169E5 | 2.837E6 | 2.7315E6 | 8 | 2 | 1 | 1 | 1 | 1 | 1 | 1 | 340 | 368 |  |  | PEAKS DB |
| K.DIENQYETQITQIEHEVSSSGQEVQSSAKEVTQLR.H | Y | 103.11 | 3989.9089 | 35 | 0.5 | 998.4850 | 4 | 79.81 | 2 | F2:25063 | Fraction\_2\_23092022.raw | 2.8028E6 | 3.4203E6 | 6.2879E5 | 1.5665E5 | 2.5602E5 | 1.3422E6 |  | 6 | 1 | 1 | 1 | 1 | 1 | 1 | 0 | 340 | 374 |  |  | PEAKS DB |
| K.EIETYHNLLEGGQEDFESSGAGK.I | Y | 98.51 | 2509.1245 | 23 | 0.1 | 837.3822 | 3 | 55.97 | 1 | F1:14223 | Fraction\_1\_23092022r.raw | 6.3311E6 | 8.0004E5 | 5.5376E5 |  |  | 6.5908E5 |  | 5 | 2 | 1 | 1 | 0 | 0 | 1 | 0 | 450 | 472 |  |  | PEAKS DB |
| R.HGVQELEIELQSQLSK.K | Y | 97.55 | 1836.9581 | 16 | 0.8 | 919.4871 | 2 | 63.63 | 2 | F2:17855 | Fraction\_2\_23092022.raw | 1.322E7 | 5.9817E6 | 3.2595E6 | 4.595E5 | 2.431E5 | 1.8834E6 | 2.5033E6 | 10 | 2 | 2 | 2 | 1 | 1 | 1 | 1 | 375 | 390 |  |  | PEAKS DB |
| R.HGVQELEIELQSQLSKK.A | Y | 96.03 | 1965.0531 | 17 | 0.4 | 656.0253 | 3 | 56.76 | 7 | F7:19525 | Fraction\_7\_23092022.raw | 1.2248E7 |  |  |  |  | 2.7415E6 | 2.1209E6 | 3 | 1 | 0 | 0 | 0 | 0 | 1 | 1 | 375 | 391 |  |  | PEAKS DB |
| K.VQALEEANNDLENK.I | Y | 86.18 | 1585.7583 | 14 | 0.4 | 793.8868 | 2 | 38.18 | 7 | F7:9888 | Fraction\_7\_23092022.raw | 1.0998E6 | 1.2058E6 | 1.9072E6 | 2.8175E5 | 2.6402E5 | 3.2816E5 | 2.8373E5 | 10 | 2 | 2 | 2 | 1 | 1 | 1 | 1 | 171 | 184 |  |  | PEAKS DB |
| R.QEIEC(+57.02)QNQEYSLLLSIK.M | Y | 83.87 | 2094.0303 | 17 | 0.5 | 1048.0229 | 2 | 71.72 | 1 | F1:21237 | Fraction\_1\_23092022r.raw | 2.5842E6 |  |  |  |  |  |  | 2 | 2 | 0 | 0 | 0 | 0 | 0 | 0 | 428 | 444 | Carbamidomethylation | C5:Carbamidomethylation:1000.00 | PEAKS DB |
| R.LEKEIETYHNLLEGGQEDFESSGAGK.I | Y | 79.43 | 2879.3459 | 26 | 1.0 | 720.8445 | 4 | 55.38 | 1 | F1:13973 | Fraction\_1\_23092022r.raw | 5.4438E5 |  |  |  |  |  |  | 1 | 1 | 0 | 0 | 0 | 0 | 0 | 0 | 447 | 472 |  |  | PEAKS DB |
| R.KDIENQYETQITQIEHEVSSSGQEVQSSAK.E | Y | 77.13 | 3391.6016 | 30 | -0.7 | 848.9071 | 4 | 64.61 | 1 | F1:18107 | Fraction\_1\_23092022r.raw | 5.1202E5 |  |  |  |  |  |  | 1 | 1 | 0 | 0 | 0 | 0 | 0 | 0 | 339 | 368 |  |  | PEAKS DB |
| R.GGSGGSHGGGSGFGGESGGSYGGGEEASGSGGGYGGGSGKSSHS | Y | 71.53 | 3620.4292 | 44 | 0.3 | 906.1149 | 4 | 23.51 | 7 | F7:3707 | Fraction\_7\_23092022.raw |  |  |  |  |  |  | 2.8278E5 | 1 | 0 | 0 | 0 | 0 | 0 | 0 | 1 | 580 | 623 |  |  | PEAKS DB |
| R.SGGGGGGGLGSGGSIRSSYSR.F | Y | 70.07 | 1811.8510 | 21 | 1.1 | 604.9583 | 3 | 25.90 | 7 | F7:4537 | Fraction\_7\_23092022.raw |  |  |  |  |  |  | 3.6723E4 | 1 | 0 | 0 | 0 | 0 | 0 | 0 | 1 | 14 | 34 |  |  | PEAKS DB |
| K.DQIVDLTVGNNK.T | Y | 68.43 | 1314.6780 | 12 | 0.4 | 658.3466 | 2 | 50.10 | 3 | F3:12123 | Fraction\_3\_23092022.raw | 5.3699E5 | 2.0511E5 | 7.081E5 |  |  |  |  | 3 | 1 | 1 | 1 | 0 | 0 | 0 | 0 | 213 | 224 |  |  | PEAKS DB |
| K.SDLEM(+15.99)QYETLQEELM(+15.99)ALKK.N | Y | 64.71 | 2330.1021 | 19 | 1.1 | 777.7088 | 3 | 76.03 | 1 | F1:23219 | Fraction\_1\_23092022r.raw | 1.1277E6 |  |  |  |  |  |  | 2 | 2 | 0 | 0 | 0 | 0 | 0 | 0 | 272 | 290 | Oxidation (M) | M5:Oxidation (M):1000.00;M15:Oxidation (M):1000.00 | PEAKS DB |
| K.TLNDMRQEYEQLIAK.N | Y | 63.07 | 1850.9197 | 15 | -0.1 | 617.9805 | 3 | 61.23 | 1 | F1:16523 | Fraction\_1\_23092022r.raw | 6.9008E6 |  |  |  |  |  |  | 1 | 1 | 0 | 0 | 0 | 0 | 0 | 0 | 322 | 336 |  |  | PEAKS DB |
| K.TLLDIDNTR.M | Y | 59.12 | 1059.5560 | 9 | 0.6 | 530.7856 | 2 | 48.06 | 7 | F7:14511 | Fraction\_7\_23092022.raw | 6.3217E6 | 4.7772E6 | 6.1536E6 | 6.3721E5 |  | 1.0117E6 | 4.789E5 | 6 | 1 | 1 | 1 | 1 | 0 | 1 | 1 | 225 | 233 |  |  | PEAKS DB |
| K.TLNDM(+15.99)RQEYEQLIAK.N | Y | 57.71 | 1866.9146 | 15 | 0.4 | 623.3124 | 3 | 47.08 | 2 | F2:10362 | Fraction\_2\_23092022.raw |  | 2.9847E6 |  |  | 2.0646E5 | 1.1581E6 |  | 5 | 0 | 2 | 0 | 0 | 1 | 2 | 0 | 322 | 336 | Oxidation (M) | M5:Oxidation (M):1000.00 | PEAKS DB |
| K.NYSPYYNTIDDLK.D | Y | 57.41 | 1604.7358 | 13 | -0.2 | 803.3751 | 2 | 56.42 | 1 | F1:14439 | Fraction\_1\_23092022r.raw | 3.4112E5 |  |  |  |  |  |  | 1 | 1 | 0 | 0 | 0 | 0 | 0 | 0 | 200 | 212 |  |  | PEAKS DB |
| R.MTLDDFR.I | Y | 56.97 | 896.4062 | 7 | 0.4 | 449.2106 | 2 | 51.02 | 1 | F1:12050 | Fraction\_1\_23092022r.raw | 1.0123E6 |  |  |  |  |  |  | 1 | 1 | 0 | 0 | 0 | 0 | 0 | 0 | 234 | 240 |  |  | PEAKS DB |
| R.HGVQELEIELQSQLSKKAALEK.S | Y | 56.31 | 2477.3489 | 22 | 0.4 | 620.3447 | 4 | 57.32 | 6 | F6:16369 | Fraction\_6\_23092022.raw |  | 9.7025E5 |  |  |  | 2.3819E5 |  | 2 | 0 | 1 | 0 | 0 | 0 | 1 | 0 | 375 | 396 |  |  | PEAKS DB |
| R.FSSSGGGGGGGRFSSSSGYGGGSSR.V | Y | 53.05 | 2197.9373 | 25 | 0.1 | 733.6531 | 3 | 24.57 | 7 | F7:4029 | Fraction\_7\_23092022.raw |  |  |  |  |  |  | 2.6807E5 | 1 | 0 | 0 | 0 | 0 | 0 | 0 | 1 | 35 | 59 |  |  | PEAKS DB |
| R.IKFEMEQNLR.Q | Y | 50.84 | 1306.6703 | 10 | -0.2 | 436.5639 | 3 | 43.39 | 1 | F1:8657 | Fraction\_1\_23092022r.raw | 6.8241E5 |  |  |  |  |  |  | 1 | 1 | 0 | 0 | 0 | 0 | 0 | 0 | 241 | 250 |  |  | PEAKS DB |
| K.SDLEM(+15.99)QYETLQEELMALKK.N | Y | 50.62 | 2314.1072 | 19 | 0.8 | 772.3770 | 3 | 78.52 | 1 | F1:24324 | Fraction\_1\_23092022r.raw | 1.156E5 |  |  |  |  |  |  | 1 | 1 | 0 | 0 | 0 | 0 | 0 | 0 | 272 | 290 | Oxidation (M) | M5:Oxidation (M):43.70 | PEAKS DB |
| K.TLLDIDNTRM(+15.99)TLDDFR.I | Y | 49.27 | 1953.9465 | 16 | 0.6 | 652.3232 | 3 | 64.62 | 1 | F1:18095 | Fraction\_1\_23092022r.raw | 1.7001E5 |  |  |  |  |  |  | 1 | 1 | 0 | 0 | 0 | 0 | 0 | 0 | 225 | 240 | Oxidation (M) | M10:Oxidation (M):1000.00 | PEAKS DB |
| total 26 peptides |  |  |  |  |  |  |  |  |  |  |  |  |  |  |  |  |  |  |  |  |  |  |  |  |  |  |  |  |  |  |  |
| --- | --- | --- | --- | --- | --- | --- | --- | --- | --- | --- | --- | --- | --- | --- | --- | --- | --- | --- | --- | --- | --- | --- | --- | --- | --- | --- | --- | --- | --- | --- | --- |

P13645|K1C10\_HUMAN

back to list

  

| Protein Coverage
| Supporting Peptides
|

Protein Coverage:

Supporting Peptides:

| Peptide | Uniq | -10lgP | Mass | Length | ppm | m/z | z | RT | Fraction | Scan | Source File | Area F1 | Area F2 | Area F3 | Area F4 | Area F5 | Area F6 | Area F7 | #Feature | #Feature F1 | #Feature F2 | #Feature F3 | #Feature F4 | #Feature F5 | #Feature F6 | #Feature F7 | Start | End | PTM | AScore | Found By |
| --- | --- | --- | --- | --- | --- | --- | --- | --- | --- | --- | --- | --- | --- | --- | --- | --- | --- | --- | --- | --- | --- | --- | --- | --- | --- | --- | --- | --- | --- | --- | --- |
| R.GSSGGGC(+57.02)FGGSSGGYGGLGGFGGGSFR.G | Y | 126.87 | 2341.9771 | 27 | -1.2 | 1171.9944 | 2 | 61.28 | 5 | F5:17467 | Fraction\_5\_23092022.raw | 1.5792E7 | 3.8379E6 | 5.2059E6 | 1.1871E6 | 1.5145E6 | 4.031E6 | 1.6271E6 | 11 | 2 | 2 | 2 | 1 | 1 | 2 | 1 | 60 | 86 | Carbamidomethylation | C7:Carbamidomethylation:1000.00 | PEAKS DB |
| R.NVSTGDVNVEMNAAPGVDLTQLLNNM(+15.99)R.S | Y | 105.53 | 2887.3804 | 27 | 1.4 | 963.4688 | 3 | 79.48 | 1 | F1:24717 | Fraction\_1\_23092022r.raw | 3.7272E6 |  |  |  |  |  |  | 2 | 2 | 0 | 0 | 0 | 0 | 0 | 0 | 296 | 322 | Oxidation (M) | M26:Oxidation (M):133.36 | PEAKS DB |
| K.NQILNLTTDNANILLQIDNAR.L | Y | 104.06 | 2366.2554 | 21 | 0.1 | 1184.1351 | 2 | 82.41 | 1 | F1:26051 | Fraction\_1\_23092022r.raw | 2.675E6 | 8.2335E5 | 1.1205E6 | 9.2039E4 |  | 1.0172E5 |  | 7 | 2 | 1 | 2 | 1 | 0 | 1 | 0 | 208 | 228 |  |  | PEAKS DB |
| K.GSLGGGFSSGGFSGGSFSR.G | Y | 102.25 | 1706.7648 | 19 | 0.3 | 854.3899 | 2 | 55.16 | 5 | F5:14691 | Fraction\_5\_23092022.raw | 3.7889E7 | 1.3171E7 | 1.8799E7 | 3.0863E6 | 5.0574E6 | 1.378E7 | 1.0368E7 | 10 | 1 | 1 | 4 | 1 | 1 | 1 | 1 | 41 | 59 |  |  | PEAKS DB |
| K.ELTTEIDNNIEQISSYK.S | Y | 98.62 | 1995.9636 | 17 | 0.4 | 998.9895 | 2 | 66.77 | 1 | F1:19027 | Fraction\_1\_23092022r.raw | 3.7204E6 | 1.491E6 | 4.4544E6 | 7.7249E5 | 5.7468E5 | 7.3368E5 |  | 6 | 1 | 1 | 1 | 1 | 1 | 1 | 0 | 346 | 362 |  |  | PEAKS DB |
| R.NVSTGDVNVEM(+15.99)NAAPGVDLTQLLNNM(+15.99)R.S | Y | 97.09 | 2903.3752 | 27 | 1.3 | 968.8003 | 3 | 74.06 | 1 | F1:22288 | Fraction\_1\_23092022r.raw | 1.0332E7 | 1.0315E6 | 7.2533E5 | 3.3252E5 | 2.0239E5 | 3.6883E5 |  | 8 | 2 | 2 | 1 | 1 | 1 | 1 | 0 | 296 | 322 | Oxidation (M) | M11:Oxidation (M):1000.00;M26:Oxidation (M):1000.00 | PEAKS DB |
| R.ISSSKGSLGGGFSSGGFSGGSFSR.G | Y | 94.19 | 2209.0398 | 24 | 0.3 | 737.3541 | 3 | 47.03 | 7 | F7:13977 | Fraction\_7\_23092022.raw | 6.1881E5 | 2.6543E6 |  |  | 5.7646E5 | 6.1611E5 | 3.3283E6 | 5 | 1 | 1 | 0 | 0 | 1 | 1 | 1 | 36 | 59 |  |  | PEAKS DB |
| R.AETEC(+57.02)QNTEYQQLLDIK.I | Y | 92.68 | 2081.9575 | 17 | 1.2 | 1041.9873 | 2 | 60.86 | 5 | F5:17273 | Fraction\_5\_23092022.raw | 6.127E6 | 8.616E5 | 9.6727E5 | 4.4686E5 | 5.0529E5 | 5.831E5 |  | 7 | 2 | 1 | 1 | 1 | 1 | 1 | 0 | 423 | 439 | Carbamidomethylation | C5:Carbamidomethylation:1000.00 | PEAKS DB |
| R.ALEESNYELEGK.I | N | 91.02 | 1380.6409 | 12 | 0.1 | 691.3278 | 2 | 37.46 | 5 | F5:6790 | Fraction\_5\_23092022.raw | 7.8781E5 | 1.0987E5 | 3.4777E6 | 1.0546E6 | 7.6134E5 | 1.1081E6 | 8.9163E5 | 8 | 1 | 1 | 2 | 1 | 1 | 1 | 1 | 166 | 177 |  |  | PEAKS DB |
| R.NVQALEIELQSQLALK.Q | Y | 82.81 | 1796.0043 | 16 | 0.7 | 899.0101 | 2 | 78.36 | 3 | F3:24764 | Fraction\_3\_23092022.raw | 1.5024E6 | 1.2348E6 | 1.5662E6 | 5.1703E5 | 3.6324E5 | 5.6729E5 |  | 14 | 3 | 2 | 3 | 2 | 2 | 2 | 0 | 371 | 386 |  |  | PEAKS DB |
| K.SKELTTEIDNNIEQISSYK.S | Y | 82.04 | 2211.0906 | 19 | 0.5 | 738.0378 | 3 | 58.96 | 1 | F1:15535 | Fraction\_1\_23092022r.raw | 1.0388E6 |  |  |  |  |  |  | 1 | 1 | 0 | 0 | 0 | 0 | 0 | 0 | 344 | 362 |  |  | PEAKS DB |
| K.ELTTEIDNNIEQISSYKSEITELRR.N | Y | 80.74 | 2980.4988 | 25 | 0.9 | 746.1326 | 4 | 68.55 | 1 | F1:19854 | Fraction\_1\_23092022r.raw | 1.5526E6 | 2.0539E5 | 3.0809E5 |  |  |  |  | 3 | 1 | 1 | 1 | 0 | 0 | 0 | 0 | 346 | 370 |  |  | PEAKS DB |
| K.QSLEASLAETEGR.Y | N | 78.74 | 1389.6736 | 13 | -0.5 | 695.8438 | 2 | 44.67 | 7 | F7:12899 | Fraction\_7\_23092022.raw | 4.4287E6 | 2.5816E6 | 5.3296E5 | 8.1428E5 | 7.0666E5 | 5.9797E5 | 3.6144E5 | 8 | 1 | 2 | 1 | 1 | 1 | 1 | 1 | 387 | 399 |  |  | PEAKS DB |
| R.SQYEQLAEQNR.K | Y | 75.63 | 1364.6321 | 11 | 0.1 | 683.3234 | 2 | 29.39 | 7 | F7:6031 | Fraction\_7\_23092022.raw |  |  |  |  |  |  | 6.198E4 | 1 | 0 | 0 | 0 | 0 | 0 | 0 | 1 | 323 | 333 |  |  | PEAKS DB |
| R.LAADDFRLKYENEVALR.Q | Y | 72.85 | 2022.0533 | 17 | 0.1 | 675.0251 | 3 | 53.39 | 2 | F2:13215 | Fraction\_2\_23092022.raw | 8.8907E6 | 4.2685E6 | 2.9208E6 | 6.3854E5 | 6.7978E5 | 1.4936E6 | 1.4353E6 | 13 | 2 | 2 | 2 | 2 | 2 | 1 | 2 | 229 | 245 |  |  | PEAKS DB |
| K.IRLENEIQTYR.S | N | 70.57 | 1433.7627 | 11 | 0.0 | 478.9282 | 3 | 40.46 | 3 | F3:7815 | Fraction\_3\_23092022.raw | 4.5903E6 | 8.1016E5 | 1.002E6 | 2.8274E5 | 2.5433E5 | 3.8169E5 | 1.8818E5 | 7 | 1 | 1 | 1 | 1 | 1 | 1 | 1 | 440 | 450 |  |  | PEAKS DB |
| R.SQYEQLAEQNRKDAEAWFNEK.S | Y | 68.52 | 2583.1990 | 21 | 0.5 | 646.8074 | 4 | 53.68 | 1 | F1:13201 | Fraction\_1\_23092022r.raw | 1.3001E6 |  |  |  |  |  |  | 2 | 2 | 0 | 0 | 0 | 0 | 0 | 0 | 323 | 343 |  |  | PEAKS DB |
| R.ALEESNYELEGKIKEWYEK.H | N | 67.36 | 2357.1426 | 19 | 1.8 | 590.2940 | 4 | 56.44 | 1 | F1:14459 | Fraction\_1\_23092022r.raw | 1.1943E6 |  |  |  |  |  |  | 2 | 2 | 0 | 0 | 0 | 0 | 0 | 0 | 166 | 184 |  |  | PEAKS DB |
| K.DAEAWFNEK.S | N | 64.27 | 1108.4825 | 9 | 0.0 | 555.2485 | 2 | 53.27 | 1 | F1:13013 | Fraction\_1\_23092022r.raw | 3.1891E6 |  |  |  |  |  |  | 1 | 1 | 0 | 0 | 0 | 0 | 0 | 0 | 335 | 343 |  |  | PEAKS DB |
| K.SKELTTEIDNNIEQISSYKSEITELRR.N | Y | 63.98 | 3195.6257 | 27 | 0.9 | 640.1330 | 5 | 63.95 | 1 | F1:17827 | Fraction\_1\_23092022r.raw | 5.5905E5 |  |  |  |  |  |  | 1 | 1 | 0 | 0 | 0 | 0 | 0 | 0 | 344 | 370 |  |  | PEAKS DB |
| K.ELTTEIDNNIEQISSYKSEITELR.R | Y | 63.45 | 2824.3977 | 24 | 1.3 | 942.4744 | 3 | 74.67 | 1 | F1:22588 | Fraction\_1\_23092022r.raw | 3.3114E5 |  |  |  |  |  |  | 1 | 1 | 0 | 0 | 0 | 0 | 0 | 0 | 346 | 369 |  |  | PEAKS DB |
| K.VTM(+15.99)QNLNDRLASYLDKVR.A | N | 58.54 | 2151.1106 | 18 | 0.9 | 538.7854 | 4 | 56.01 | 1 | F1:14301 | Fraction\_1\_23092022r.raw | 7.88E6 | 8.0553E5 |  |  |  |  |  | 3 | 2 | 1 | 0 | 0 | 0 | 0 | 0 | 148 | 165 | Oxidation (M) | M3:Oxidation (M):1000.00 | PEAKS DB |
| K.VTMQNLNDRLASYLDKVR.A | N | 58.08 | 2135.1157 | 18 | 0.3 | 712.7127 | 3 | 63.69 | 1 | F1:17659 | Fraction\_1\_23092022r.raw | 2.701E6 |  |  |  |  |  |  | 2 | 2 | 0 | 0 | 0 | 0 | 0 | 0 | 148 | 165 |  |  | PEAKS DB |
| R.QSVEADINGLR.R | N | 57.26 | 1200.6099 | 11 | 0.7 | 601.3126 | 2 | 44.12 | 4 | F4:9998 | Fraction\_4\_23092022.raw | 2.8401E6 | 5.2058E5 | 4.4994E5 | 1.64E5 | 1.0317E5 | 1.7377E5 |  | 6 | 1 | 1 | 1 | 1 | 1 | 1 | 0 | 246 | 256 |  |  | PEAKS DB |
| K.VTMQNLNDRLASYLDK.V | N | 56.99 | 1879.9462 | 16 | -0.8 | 627.6555 | 3 | 61.55 | 1 | F1:16687 | Fraction\_1\_23092022r.raw | 5.9999E5 |  |  |  |  |  |  | 1 | 1 | 0 | 0 | 0 | 0 | 0 | 0 | 148 | 163 |  |  | PEAKS DB |
| R.ALEESNYELEGKIK.E | N | 56.95 | 1621.8198 | 14 | 0.5 | 811.9176 | 2 | 39.50 | 1 | F1:6978 | Fraction\_1\_23092022r.raw | 4.8202E5 |  |  |  |  |  |  | 2 | 2 | 0 | 0 | 0 | 0 | 0 | 0 | 166 | 179 |  |  | PEAKS DB |
| R.VLDELTLTK.A | N | 55.72 | 1030.5911 | 9 | 0.1 | 516.3029 | 2 | 51.28 | 3 | F3:12634 | Fraction\_3\_23092022.raw | 3.2189E6 | 2.8075E6 | 3.531E6 | 1.112E6 | 1.4529E6 | 8.622E5 | 1.0181E6 | 7 | 1 | 1 | 1 | 1 | 1 | 1 | 1 | 258 | 266 |  |  | PEAKS DB |
| R.QSVEADINGLRR.V | N | 50.96 | 1356.7109 | 12 | -0.1 | 453.2442 | 3 | 37.62 | 1 | F1:6288 | Fraction\_1\_23092022r.raw | 4.0964E6 |  |  |  |  |  |  | 1 | 1 | 0 | 0 | 0 | 0 | 0 | 0 | 246 | 257 |  |  | PEAKS DB |
| R.LKYENEVALR.Q | Y | 47.21 | 1233.6716 | 10 | -1.1 | 412.2307 | 3 | 33.64 | 7 | F7:7885 | Fraction\_7\_23092022.raw |  |  |  |  |  |  | 1.2434E5 | 1 | 0 | 0 | 0 | 0 | 0 | 0 | 1 | 236 | 245 |  |  | PEAKS DB |
| total 29 peptides |  |  |  |  |  |  |  |  |  |  |  |  |  |  |  |  |  |  |  |  |  |  |  |  |  |  |  |  |  |  |  |
| --- | --- | --- | --- | --- | --- | --- | --- | --- | --- | --- | --- | --- | --- | --- | --- | --- | --- | --- | --- | --- | --- | --- | --- | --- | --- | --- | --- | --- | --- | --- | --- |

P35908|K22E\_HUMAN

back to list

  

| Protein Coverage
| Supporting Peptides
|

Protein Coverage:

Supporting Peptides:

| Peptide | Uniq | -10lgP | Mass | Length | ppm | m/z | z | RT | Fraction | Scan | Source File | Area F1 | Area F2 | Area F3 | Area F4 | Area F5 | Area F6 | Area F7 | #Feature | #Feature F1 | #Feature F2 | #Feature F3 | #Feature F4 | #Feature F5 | #Feature F6 | #Feature F7 | Start | End | PTM | AScore | Found By |
| --- | --- | --- | --- | --- | --- | --- | --- | --- | --- | --- | --- | --- | --- | --- | --- | --- | --- | --- | --- | --- | --- | --- | --- | --- | --- | --- | --- | --- | --- | --- | --- |
| R.GGGFGGGSSFGGGSGFSGGGFGGGGFGGGR.F | Y | 118.87 | 2398.0110 | 30 | -0.1 | 1200.0127 | 2 | 63.88 | 7 | F7:23843 | Fraction\_7\_23092022.raw | 5.284E6 | 2.7455E6 | 5.8629E6 | 1.3454E6 | 1.7026E6 | 5.2095E6 | 3.742E6 | 13 | 1 | 2 | 2 | 2 | 2 | 2 | 2 | 93 | 122 |  |  | PEAKS DB |
| R.SLVGLGGTKSISISVAGGGGGFGAAGGFGGR.G | N | 107.15 | 2650.3826 | 31 | 1.2 | 884.4692 | 3 | 66.92 | 5 | F5:19957 | Fraction\_5\_23092022.raw | 2.5347E5 | 3.1515E5 | 6.9208E4 |  | 3.8346E5 | 2.0756E5 |  | 5 | 1 | 1 | 1 | 0 | 1 | 1 | 0 | 62 | 92 |  |  | PEAKS DB |
| R.SKEEAEALYHSKYEELQVTVGR.H | N | 96.23 | 2565.2710 | 22 | 0.8 | 856.0983 | 3 | 44.97 | 1 | F1:9339 | Fraction\_1\_23092022r.raw | 7.0702E6 | 8.8942E5 | 1.2092E6 |  |  |  |  | 4 | 2 | 1 | 1 | 0 | 0 | 0 | 0 | 363 | 384 |  |  | PEAKS DB |
| R.TSQNSELNNMQDLVEDYKK.K | N | 93.44 | 2255.0376 | 19 | 0.9 | 752.6871 | 3 | 56.35 | 1 | F1:14382 | Fraction\_1\_23092022r.raw | 4.5807E6 |  |  |  |  |  |  | 2 | 2 | 0 | 0 | 0 | 0 | 0 | 0 | 248 | 266 |  |  | PEAKS DB |
| K.VLYDAEISQIHQSVTDTNVILSM(+15.99)DNSR.N | N | 91.68 | 3063.4819 | 27 | 1.4 | 1022.1693 | 3 | 68.11 | 1 | F1:19646 | Fraction\_1\_23092022r.raw | 9.5473E5 |  |  |  |  |  |  | 1 | 1 | 0 | 0 | 0 | 0 | 0 | 0 | 315 | 341 | Oxidation (M) | M23:Oxidation (M):1000.00 | PEAKS DB |
| K.AAFGGSGGRGSSSGGGYSSGSSSYGSGGR.Q | N | 88.86 | 2500.0598 | 29 | 0.8 | 834.3612 | 3 | 24.36 | 7 | F7:3967 | Fraction\_7\_23092022.raw |  |  |  |  |  |  | 2.3221E5 | 1 | 0 | 0 | 0 | 0 | 0 | 0 | 1 | 516 | 544 |  |  | PEAKS DB |
| R.TSQNSELNNMQDLVEDYK.K | N | 80.54 | 2126.9426 | 18 | 0.7 | 1064.4794 | 2 | 64.81 | 1 | F1:18162 | Fraction\_1\_23092022r.raw | 5.3704E5 |  |  |  |  |  |  | 1 | 1 | 0 | 0 | 0 | 0 | 0 | 0 | 248 | 265 |  |  | PEAKS DB |
| K.SISISVAGGGGGFGAAGGFGGR.G | N | 79.24 | 1837.9070 | 22 | -0.1 | 919.9607 | 2 | 63.10 | 4 | F4:18566 | Fraction\_4\_23092022.raw | 4.0255E6 | 2.4211E6 | 4.0175E6 | 4.9726E5 | 1.3035E6 | 2.4713E6 | 4.288E5 | 7 | 1 | 1 | 1 | 1 | 1 | 1 | 1 | 71 | 92 |  |  | PEAKS DB |
| K.VDLLNQEIEFLK.V | N | 78.90 | 1459.7922 | 12 | 0.2 | 730.9036 | 2 | 78.62 | 5 | F5:25216 | Fraction\_5\_23092022.raw | 3.9725E6 | 5.0395E6 | 9.7411E6 | 1.2817E6 | 1.2932E6 | 1.4844E6 | 2.0665E6 | 7 | 1 | 1 | 1 | 1 | 1 | 1 | 1 | 303 | 314 |  |  | PEAKS DB |
| R.TSQNSELNNM(+15.99)QDLVEDYKK.K | N | 77.51 | 2271.0325 | 19 | 0.3 | 758.0183 | 3 | 38.16 | 1 | F1:6461 | Fraction\_1\_23092022r.raw | 1.0913E6 |  |  |  |  |  |  | 2 | 2 | 0 | 0 | 0 | 0 | 0 | 0 | 248 | 266 | Oxidation (M) | M10:Oxidation (M):1000.00 | PEAKS DB |
| R.M(+15.99)SGDLSSNVTVSVTSSTISSNVASK.A | N | 77.27 | 2473.1853 | 25 | 0.1 | 1237.6001 | 2 | 54.51 | 1 | F1:13564 | Fraction\_1\_23092022r.raw | 7.315E5 |  |  |  |  |  |  | 2 | 2 | 0 | 0 | 0 | 0 | 0 | 0 | 491 | 515 | Oxidation (M) | M1:Oxidation (M):1000.00 | PEAKS DB |
| K.YEELQVTVGR.H | N | 76.39 | 1192.6088 | 10 | -0.2 | 597.3115 | 2 | 44.41 | 5 | F5:9899 | Fraction\_5\_23092022.raw | 1.4421E6 | 1.1711E6 | 1.7404E6 | 2.5053E5 | 2.3909E5 |  |  | 5 | 1 | 1 | 1 | 1 | 1 | 0 | 0 | 375 | 384 |  |  | PEAKS DB |
| K.LNDLEEALQQAK.E | N | 76.28 | 1370.7041 | 12 | 0.8 | 686.3599 | 2 | 55.92 | 5 | F5:15050 | Fraction\_5\_23092022.raw | 2.4146E6 | 2.8944E6 | 3.8055E6 | 9.5826E5 | 1.1029E6 | 1.3559E6 |  | 6 | 1 | 1 | 1 | 1 | 1 | 1 | 0 | 442 | 453 |  |  | PEAKS DB |
| R.TAAENDFVTLK.K | N | 75.68 | 1207.6084 | 11 | 0.1 | 604.8115 | 2 | 46.18 | 3 | F3:10375 | Fraction\_3\_23092022.raw | 1.0099E6 | 7.7457E5 | 1.1497E6 |  |  |  |  | 3 | 1 | 1 | 1 | 0 | 0 | 0 | 0 | 276 | 286 |  |  | PEAKS DB |
| K.NVQDAIADAEQR.G | N | 74.52 | 1328.6321 | 12 | 0.1 | 665.3234 | 2 | 41.68 | 5 | F5:8658 | Fraction\_5\_23092022.raw | 1.8991E6 | 3.032E6 | 4.2427E6 | 1.1358E6 | 5.9496E5 | 9.5408E5 | 9.4723E5 | 7 | 1 | 1 | 1 | 1 | 1 | 1 | 1 | 419 | 430 |  |  | PEAKS DB |
| K.LNDLEEALQQAKEDLAR.L | N | 74.07 | 1954.9960 | 17 | 0.4 | 652.6729 | 3 | 74.42 | 1 | F1:22474 | Fraction\_1\_23092022r.raw | 3.0756E5 |  |  |  |  |  |  | 1 | 1 | 0 | 0 | 0 | 0 | 0 | 0 | 442 | 458 |  |  | PEAKS DB |
| R.GFSSGSAVVSGGSR.R | N | 72.08 | 1253.6000 | 14 | -0.5 | 627.8069 | 2 | 29.48 | 7 | F7:6074 | Fraction\_7\_23092022.raw |  |  |  |  |  |  | 6.6622E4 | 1 | 0 | 0 | 0 | 0 | 0 | 0 | 1 | 21 | 34 |  |  | PEAKS DB |
| K.TLNNKFASFIDKVR.F | N | 71.43 | 1651.9045 | 14 | -0.2 | 551.6420 | 3 | 51.36 | 2 | F2:12305 | Fraction\_2\_23092022.raw | 2.671E5 | 1.8567E6 | 2.1296E5 |  |  | 8.2338E5 | 3.789E5 | 6 | 1 | 2 | 1 | 0 | 0 | 1 | 1 | 184 | 197 |  |  | PEAKS DB |
| K.NVQDAIADAEQRGEHALKDAR.N | N | 71.22 | 2306.1362 | 21 | 0.4 | 577.5416 | 4 | 53.98 | 6 | F6:14877 | Fraction\_6\_23092022.raw | 1.9881E6 | 1.2033E6 | 5.2445E5 |  |  | 8.7336E5 |  | 4 | 1 | 1 | 1 | 0 | 0 | 1 | 0 | 419 | 439 |  |  | PEAKS DB |
| R.FLEQQNQVLQTK.W | N | 69.06 | 1474.7780 | 12 | 0.5 | 738.3966 | 2 | 42.12 | 3 | F3:8546 | Fraction\_3\_23092022.raw | 8.0973E6 | 7.8535E5 | 1.0847E6 | 3.5575E5 | 2.39E5 | 3.7556E5 | 9.8393E4 | 7 | 1 | 1 | 1 | 1 | 1 | 1 | 1 | 198 | 209 |  |  | PEAKS DB |
| R.NLDLDSIIAEVK.A | N | 67.73 | 1328.7188 | 12 | -0.3 | 665.3665 | 2 | 80.82 | 4 | F4:26420 | Fraction\_4\_23092022.raw | 1.3444E7 | 1.1783E7 | 2.3503E7 | 6.3504E6 | 3.9084E6 | 1.1367E7 | 9.0701E6 | 8 | 1 | 1 | 2 | 1 | 1 | 1 | 1 | 342 | 353 |  |  | PEAKS DB |
| R.GGGGGGFRGFSSGSAVVSGGSR.R | N | 67.66 | 1898.8983 | 22 | -0.6 | 633.9730 | 3 | 38.16 | 6 | F6:7760 | Fraction\_6\_23092022.raw | 7.754E4 | 2.0655E5 |  |  | 8.7993E4 | 1.2583E5 | 1.1908E5 | 5 | 1 | 1 | 0 | 0 | 1 | 1 | 1 | 13 | 34 |  |  | PEAKS DB |
| R.FGGFGGPGGVGGLGGPGGFGPGGYPGGIHEVSVNQSLLQPLNVKVDPEIQNVK.A | N | 67.12 | 5113.6050 | 53 | 0.3 | 1279.4089 | 4 | 80.58 | 1 | F1:25233 | Fraction\_1\_23092022r.raw | 6.5682E5 | 1.1101E6 |  |  |  |  |  | 2 | 1 | 1 | 0 | 0 | 0 | 0 | 0 | 123 | 175 |  |  | PEAKS DB |
| K.FASFIDKVR.F | N | 64.36 | 1081.5920 | 9 | 1.5 | 361.5385 | 3 | 44.57 | 4 | F4:10216 | Fraction\_4\_23092022.raw | 6.0874E6 | 1.3079E6 | 2.6129E6 | 3.499E5 | 3.5976E5 | 1.1286E6 |  | 11 | 1 | 2 | 2 | 2 | 2 | 2 | 0 | 189 | 197 |  |  | PEAKS DB |
| K.LALDVEIATYR.K | N | 62.33 | 1262.6870 | 11 | 0.5 | 632.3511 | 2 | 64.39 | 3 | F3:18523 | Fraction\_3\_23092022.raw | 1.5189E6 | 6.2742E5 | 1.3952E6 | 2.3872E5 | 2.1606E5 | 3.32E5 |  | 6 | 1 | 1 | 1 | 1 | 1 | 1 | 0 | 471 | 481 |  |  | PEAKS DB |
| K.VLYDAEISQIHQSVTDTNVILSMDNSR.N | N | 61.77 | 3047.4871 | 27 | 0.4 | 1016.8367 | 3 | 73.57 | 1 | F1:22123 | Fraction\_1\_23092022r.raw | 1.7326E5 |  |  |  |  |  |  | 1 | 1 | 0 | 0 | 0 | 0 | 0 | 0 | 315 | 341 |  |  | PEAKS DB |
| R.YLDGLTAER.T | N | 57.62 | 1036.5189 | 9 | 0.2 | 519.2668 | 2 | 43.30 | 1 | F1:8634 | Fraction\_1\_23092022r.raw | 2.7187E6 |  |  |  |  |  |  | 1 | 1 | 0 | 0 | 0 | 0 | 0 | 0 | 239 | 247 |  |  | PEAKS DB |
| K.EIKIEISELNR.V | N | 55.12 | 1342.7456 | 11 | -0.3 | 448.5890 | 3 | 50.98 | 1 | F1:11996 | Fraction\_1\_23092022r.raw | 7.4809E5 |  |  |  |  |  |  | 1 | 1 | 0 | 0 | 0 | 0 | 0 | 0 | 391 | 401 |  |  | PEAKS DB |
| K.LALDVEIATYRK.L | N | 51.28 | 1390.7820 | 12 | 0.0 | 464.6013 | 3 | 53.42 | 1 | F1:13083 | Fraction\_1\_23092022r.raw | 4.7232E5 |  |  |  |  |  |  | 1 | 1 | 0 | 0 | 0 | 0 | 0 | 0 | 471 | 482 |  |  | PEAKS DB |
| R.STSSFSC(+57.02)LSR.H | N | 47.66 | 1130.5026 | 10 | 0.0 | 566.2585 | 2 | 33.34 | 7 | F7:7761 | Fraction\_7\_23092022.raw |  |  |  |  |  |  | 1.2519E5 | 1 | 0 | 0 | 0 | 0 | 0 | 0 | 1 | 36 | 45 | Carbamidomethylation | C7:Carbamidomethylation:1000.00 | PEAKS DB |
| total 30 peptides |  |  |  |  |  |  |  |  |  |  |  |  |  |  |  |  |  |  |  |  |  |  |  |  |  |  |  |  |  |  |  |
| --- | --- | --- | --- | --- | --- | --- | --- | --- | --- | --- | --- | --- | --- | --- | --- | --- | --- | --- | --- | --- | --- | --- | --- | --- | --- | --- | --- | --- | --- | --- | --- |

TRINITY\_DN2326\_c0\_g1\_i12.p1

back to list

  

| Protein Coverage
| Supporting Peptides
|

Protein Coverage:

Supporting Peptides:

| Peptide | Uniq | -10lgP | Mass | Length | ppm | m/z | z | RT | Fraction | Scan | Source File | Area F1 | Area F2 | Area F3 | Area F4 | Area F5 | Area F6 | Area F7 | #Feature | #Feature F1 | #Feature F2 | #Feature F3 | #Feature F4 | #Feature F5 | #Feature F6 | #Feature F7 | Start | End | PTM | AScore | Found By |
| --- | --- | --- | --- | --- | --- | --- | --- | --- | --- | --- | --- | --- | --- | --- | --- | --- | --- | --- | --- | --- | --- | --- | --- | --- | --- | --- | --- | --- | --- | --- | --- |
| R.IC(+57.02)ETDAHYVQIIHTSNNLGTER.T | N | 114.42 | 2570.2183 | 22 | 0.2 | 857.7468 | 3 | 44.41 | 5 | F5:9876 | Fraction\_5\_23092022.raw |  | 5.1745E5 | 2.2932E7 | 1.5069E7 | 4.1552E7 | 1.5463E7 | 4.742E6 | 12 | 0 | 1 | 3 | 2 | 2 | 2 | 2 | 214 | 235 | Carbamidomethylation | C2:Carbamidomethylation:1000.00 | PEAKS DB |
| R.LIGHSLGAQIAGFAGKEYQK.F | N | 111.41 | 2087.1162 | 20 | 0.0 | 696.7126 | 3 | 49.84 | 5 | F5:12302 | Fraction\_5\_23092022.raw |  |  | 3.7265E6 | 5.2349E6 | 7.6906E6 | 9.6072E6 | 2.4268E6 | 10 | 0 | 0 | 2 | 2 | 2 | 2 | 2 | 167 | 186 |  |  | PEAKS DB |
| R.LIGHSLGAQIAGFAGK.E | N | 105.57 | 1538.8568 | 16 | -0.6 | 513.9592 | 3 | 52.10 | 5 | F5:13308 | Fraction\_5\_23092022.raw |  |  | 9.856E6 | 7.8605E6 | 1.4847E7 | 1.2515E7 | 2.5951E6 | 9 | 0 | 0 | 2 | 2 | 2 | 2 | 1 | 167 | 182 |  |  | PEAKS DB |
| K.TYPKTGSFYVPVER.K | Y | 97.78 | 1642.8354 | 14 | 0.3 | 548.6193 | 3 | 45.36 | 7 | F7:13154 | Fraction\_7\_23092022.raw |  |  | 1.257E5 | 3.071E6 |  | 1.0585E6 | 4.1939E6 | 6 | 0 | 0 | 1 | 2 | 0 | 1 | 2 | 310 | 323 |  |  | PEAKS DB |
| R.AVEYLTEC(+57.02)IRHEC(+57.02)C(+57.02)LIGVPQSK.N | N | 96.18 | 2661.2712 | 22 | 0.1 | 888.0978 | 3 | 57.79 | 3 | F3:15577 | Fraction\_3\_23092022.raw |  |  | 3.5563E6 | 1.083E6 | 7.3455E5 | 2.3381E6 | 5.9298E6 | 10 | 0 | 0 | 2 | 2 | 2 | 2 | 2 | 267 | 288 | Carbamidomethylation | C8:Carbamidomethylation:1000.00;C13:Carbamidomethylation:1000.00;C14:Carbamidomethylation:1000.00 | PEAKS DB |
| K.LVPEEISFVLSTR.E | N | 93.13 | 1488.8187 | 13 | 0.7 | 497.2805 | 3 | 76.42 | 7 | F7:30218 | Fraction\_7\_23092022.raw |  |  | 2.5384E7 | 5.5521E6 |  | 3.1395E5 | 7.2902E7 | 8 | 0 | 0 | 3 | 2 | 0 | 1 | 2 | 39 | 51 |  |  | PEAKS DB |
| K.DC(+57.02)SQRIC(+57.02)ETDAHYVQIIHTSNNLGTER.T | N | 86.26 | 3216.4675 | 27 | 0.3 | 805.1244 | 4 | 45.52 | 5 | F5:10410 | Fraction\_5\_23092022.raw |  |  | 7.48E5 |  | 9.5662E5 |  |  | 2 | 0 | 0 | 1 | 0 | 1 | 0 | 0 | 209 | 235 | Carbamidomethylation | C2:Carbamidomethylation:1000.00;C7:Carbamidomethylation:1000.00 | PEAKS DB |
| K.NPQPVSTC(+57.02)TRNEC(+57.02)VC(+57.02)VGLNAK.T | Y | 83.32 | 2403.1094 | 21 | -0.5 | 802.0433 | 3 | 36.11 | 7 | F7:8969 | Fraction\_7\_23092022.raw |  |  | 4.6146E5 |  |  | 6.0973E5 | 3.3034E6 | 3 | 0 | 0 | 1 | 0 | 0 | 1 | 1 | 289 | 309 | Carbamidomethylation | C8:Carbamidomethylation:1000.00;C13:Carbamidomethylation:1000.00;C15:Carbamidomethylation:1000.00 | PEAKS DB |
| K.NPQPVSTC(+57.02)TRNEC(+57.02)VC(+57.02)VGLNAKTYPK.T | Y | 82.25 | 2892.3682 | 25 | -0.1 | 724.0992 | 4 | 36.33 | 7 | F7:9075 | Fraction\_7\_23092022.raw |  |  |  |  |  |  | 8.6003E5 | 1 | 0 | 0 | 0 | 0 | 0 | 0 | 1 | 289 | 313 | Carbamidomethylation | C8:Carbamidomethylation:1000.00;C13:Carbamidomethylation:1000.00;C15:Carbamidomethylation:1000.00 | PEAKS DB |
| K.C(+57.02)KLVPEEISFVLSTR.E | N | 82.05 | 1776.9443 | 15 | -0.3 | 593.3219 | 3 | 68.54 | 7 | F7:26245 | Fraction\_7\_23092022.raw |  |  | 8.9535E5 |  |  |  | 5.1253E6 | 2 | 0 | 0 | 1 | 0 | 0 | 0 | 1 | 37 | 51 | Carbamidomethylation | C1:Carbamidomethylation:1000.00 | PEAKS DB |
| R.TLGTVDFYVNNGHNQPGC(+57.02)LPSFLGETC(+57.02)SHTR.A | N | 80.43 | 3477.5830 | 31 | 1.0 | 870.4039 | 4 | 65.29 | 4 | F4:19503 | Fraction\_4\_23092022.raw |  |  | 4.1532E6 | 9.7174E5 | 4.7255E5 | 1.6737E6 |  | 5 | 0 | 0 | 2 | 1 | 1 | 1 | 0 | 236 | 266 | Carbamidomethylation | C18:Carbamidomethylation:1000.00;C27:Carbamidomethylation:1000.00 | PEAKS DB |
| K.FIGYPTAVENTR.T | N | 80.37 | 1366.6881 | 12 | -0.5 | 684.3510 | 2 | 48.74 | 5 | F5:11816 | Fraction\_5\_23092022.raw | 3.3353E5 | 2.1858E5 | 1.0951E7 | 9.4032E6 | 1.3563E7 | 8.6082E6 | 3.584E6 | 8 | 1 | 1 | 2 | 1 | 1 | 1 | 1 | 130 | 141 |  |  | PEAKS DB |
| K.YSEIIGLDPAGPM(+15.99)FK.S | N | 79.02 | 1652.8119 | 15 | 0.6 | 827.4138 | 2 | 66.27 | 5 | F5:19734 | Fraction\_5\_23092022.raw |  |  | 1.2949E7 | 7.2826E6 | 1.3421E7 | 1.3527E7 | 3.307E6 | 10 | 0 | 0 | 2 | 2 | 2 | 2 | 2 | 192 | 206 | Oxidation (M) | M13:Oxidation (M):1000.00 | PEAKS DB |
| R.DGAC(+57.02)TNEFQILKFIGYPTAVENTR.T | N | 75.80 | 2743.3274 | 24 | -4.5 | 915.4456 | 3 | 82.31 | 6 | F6:27538 | Fraction\_6\_23092022.raw |  |  | 3.939E5 |  |  | 2.673E6 |  | 2 | 0 | 0 | 1 | 0 | 0 | 1 | 0 | 118 | 141 | Carbamidomethylation | C4:Carbamidomethylation:1000.00 | PEAKS DB |
| K.TGSFYVPVER.K | Y | 75.62 | 1153.5768 | 10 | -0.2 | 577.7955 | 2 | 48.04 | 7 | F7:14466 | Fraction\_7\_23092022.raw |  | 2.0625E5 | 9.108E6 | 1.0098E7 |  | 3.7144E6 | 7.9345E6 | 5 | 0 | 1 | 1 | 1 | 0 | 1 | 1 | 314 | 323 |  |  | PEAKS DB |
| R.DGAC(+57.02)TNEFQILK.F | N | 74.83 | 1394.6500 | 12 | 0.0 | 698.3323 | 2 | 57.21 | 5 | F5:15614 | Fraction\_5\_23092022.raw |  |  | 1.1719E6 | 1.4003E6 | 2.1747E6 | 1.4246E6 |  | 4 | 0 | 0 | 1 | 1 | 1 | 1 | 0 | 118 | 129 | Carbamidomethylation | C4:Carbamidomethylation:1000.00 | PEAKS DB |
| K.SKDC(+57.02)SQRIC(+57.02)ETDAHYVQIIHTSNNLGTER.T | N | 73.26 | 3431.5947 | 29 | -0.1 | 687.3262 | 5 | 39.88 | 5 | F5:7848 | Fraction\_5\_23092022.raw |  |  |  |  | 2.5666E6 | 7.5961E5 | 1.327E5 | 5 | 0 | 0 | 0 | 0 | 3 | 1 | 1 | 207 | 235 | Carbamidomethylation | C4:Carbamidomethylation:1000.00;C9:Carbamidomethylation:1000.00 | PEAKS DB |
| K.YSEIIGLDPAGPM(+15.99)FKSK.D | N | 72.91 | 1867.9390 | 17 | 0.1 | 623.6537 | 3 | 55.88 | 3 | F3:14752 | Fraction\_3\_23092022.raw |  |  | 1.296E5 |  |  |  |  | 1 | 0 | 0 | 1 | 0 | 0 | 0 | 0 | 192 | 208 | Oxidation (M) | M13:Oxidation (M):1000.00 | PEAKS DB |
| R.TVGKYIAEFSK.L | N | 67.90 | 1241.6655 | 11 | -0.4 | 621.8398 | 2 | 40.96 | 5 | F5:8354 | Fraction\_5\_23092022.raw |  |  |  |  | 9.2439E5 | 7.046E5 |  | 4 | 0 | 0 | 0 | 0 | 3 | 1 | 0 | 142 | 152 |  |  | PEAKS DB |
| R.AVEYLTEC(+57.02)IR.H | N | 66.81 | 1252.6122 | 10 | -0.2 | 627.3132 | 2 | 49.19 | 7 | F7:15094 | Fraction\_7\_23092022.raw |  |  | 1.079E6 | 6.0394E5 |  | 6.2548E5 | 1.1357E6 | 4 | 0 | 0 | 1 | 1 | 0 | 1 | 1 | 267 | 276 | Carbamidomethylation | C8:Carbamidomethylation:1000.00 | PEAKS DB |
| R.HEC(+57.02)C(+57.02)LIGVPQSK.N | N | 66.41 | 1426.6697 | 12 | 0.1 | 714.3422 | 2 | 33.72 | 7 | F7:7919 | Fraction\_7\_23092022.raw |  |  |  |  |  |  | 1.5167E5 | 1 | 0 | 0 | 0 | 0 | 0 | 0 | 1 | 277 | 288 | Carbamidomethylation | C3:Carbamidomethylation:1000.00;C4:Carbamidomethylation:1000.00 | PEAKS DB |
| R.NEC(+57.02)VC(+57.02)VGLNAKTYPK.T | N | 64.53 | 1751.8335 | 15 | 0.2 | 876.9242 | 2 | 35.82 | 7 | F7:8817 | Fraction\_7\_23092022.raw |  |  | 4.6572E5 | 5.8753E5 | 1.8031E6 |  | 5.1284E6 | 9 | 0 | 0 | 1 | 1 | 3 | 0 | 4 | 299 | 313 | Carbamidomethylation | C3:Carbamidomethylation:1000.00;C5:Carbamidomethylation:1000.00 | PEAKS DB |
| R.NEC(+57.02)VC(+57.02)VGLNAK.T | N | 61.26 | 1262.5747 | 11 | 0.2 | 632.2947 | 2 | 34.83 | 7 | F7:8388 | Fraction\_7\_23092022.raw |  |  | 3.1121E6 | 3.1889E6 | 7.1825E5 |  | 3.0319E6 | 10 | 0 | 0 | 2 | 4 | 2 | 0 | 2 | 299 | 309 | Carbamidomethylation | C3:Carbamidomethylation:1000.00;C5:Carbamidomethylation:1000.00 | PEAKS DB |
| K.TGSFYVPVERK.A | Y | 47.95 | 1281.6716 | 11 | 0.3 | 641.8433 | 2 | 37.45 | 4 | F4:7026 | Fraction\_4\_23092022.raw |  |  |  | 1.4327E5 |  |  |  | 1 | 0 | 0 | 0 | 1 | 0 | 0 | 0 | 314 | 324 |  |  | PEAKS DB |
| total 24 peptides |  |  |  |  |  |  |  |  |  |  |  |  |  |  |  |  |  |  |  |  |  |  |  |  |  |  |  |  |  |  |  |
| --- | --- | --- | --- | --- | --- | --- | --- | --- | --- | --- | --- | --- | --- | --- | --- | --- | --- | --- | --- | --- | --- | --- | --- | --- | --- | --- | --- | --- | --- | --- | --- |

TRINITY\_DN2326\_c0\_g1\_i13.p1

back to list

  

| Protein Coverage
| Supporting Peptides
|

Protein Coverage:

Supporting Peptides:

| Peptide | Uniq | -10lgP | Mass | Length | ppm | m/z | z | RT | Fraction | Scan | Source File | Area F1 | Area F2 | Area F3 | Area F4 | Area F5 | Area F6 | Area F7 | #Feature | #Feature F1 | #Feature F2 | #Feature F3 | #Feature F4 | #Feature F5 | #Feature F6 | #Feature F7 | Start | End | PTM | AScore | Found By |
| --- | --- | --- | --- | --- | --- | --- | --- | --- | --- | --- | --- | --- | --- | --- | --- | --- | --- | --- | --- | --- | --- | --- | --- | --- | --- | --- | --- | --- | --- | --- | --- |
| R.TLGTVDFYM(+15.99)NNGYNQPGC(+57.02)SLIDGDSC(+57.02)SHAR.A | Y | 121.69 | 3364.4182 | 30 | 1.9 | 1122.4822 | 3 | 59.54 | 7 | F7:21274 | Fraction\_7\_23092022.raw |  |  |  |  |  |  | 1.4735E6 | 1 | 0 | 0 | 0 | 0 | 0 | 0 | 1 | 235 | 264 | Oxidation (M); Carbamidomethylation | M9:Oxidation (M):1000.00;C18:Carbamidomethylation:1000.00;C26:Carbamidomethylation:1000.00 | PEAKS DB |
| K.YSEIIGLDPAGPLFKSQEC(+57.02)SQR.I | Y | 118.28 | 2494.2161 | 22 | 1.3 | 832.4137 | 3 | 66.74 | 5 | F5:19894 | Fraction\_5\_23092022.raw |  |  | 6.7672E5 | 1.4969E6 | 1.1853E6 | 4.4708E6 | 5.0007E6 | 5 | 0 | 0 | 1 | 1 | 1 | 1 | 1 | 191 | 212 | Carbamidomethylation | C19:Carbamidomethylation:1000.00 | PEAKS DB |
| R.IC(+57.02)ETDANYVQIIHTSSFK.G | Y | 107.70 | 2125.0149 | 18 | -1.5 | 709.3445 | 3 | 55.25 | 5 | F5:14755 | Fraction\_5\_23092022.raw |  |  | 4.4581E5 | 1.9754E6 | 8.6142E5 | 2.2597E6 |  | 4 | 0 | 0 | 1 | 1 | 1 | 1 | 0 | 213 | 230 | Carbamidomethylation | C2:Carbamidomethylation:1000.00 | PEAKS DB |
| R.IC(+57.02)ETDANYVQIIHTSSFKGTLR.T | Y | 105.86 | 2552.2693 | 22 | 0.6 | 639.0750 | 4 | 54.07 | 5 | F5:14209 | Fraction\_5\_23092022.raw |  |  | 1.3949E6 | 4.2902E6 | 2.5963E6 | 6.3456E6 | 1.7424E6 | 9 | 0 | 0 | 2 | 2 | 2 | 2 | 1 | 213 | 234 | Carbamidomethylation | C2:Carbamidomethylation:1000.00 | PEAKS DB |
| R.LIGHSLGAHVSGFAGK.K | Y | 102.96 | 1549.8364 | 16 | 0.5 | 517.6197 | 3 | 34.32 | 7 | F7:8159 | Fraction\_7\_23092022.raw |  |  | 8.726E6 | 2.4662E7 | 1.3571E7 | 6.2907E7 | 6.214E7 | 14 | 0 | 0 | 2 | 3 | 3 | 3 | 3 | 166 | 181 |  |  | PEAKS DB |
| K.YSEIIGLDPAGPLFK.S | Y | 101.75 | 1618.8606 | 15 | -0.2 | 810.4374 | 2 | 78.54 | 7 | F7:31178 | Fraction\_7\_23092022.raw |  | 1.0474E5 | 5.7087E6 | 1.3039E7 | 1.1011E7 | 4.0201E7 | 5.0361E7 | 6 | 0 | 1 | 1 | 1 | 1 | 1 | 1 | 191 | 205 |  |  | PEAKS DB |
| K.YAYYPIAVQNIR.S | Y | 82.84 | 1469.7666 | 12 | 2.4 | 735.8923 | 2 | 59.67 | 7 | F7:21300 | Fraction\_7\_23092022.raw |  |  | 5.2532E6 | 2.4981E7 | 2.2894E7 | 5.2224E7 | 1.0633E8 | 5 | 0 | 0 | 1 | 1 | 1 | 1 | 1 | 129 | 140 |  |  | PEAKS DB |
| R.LIGHSLGAHVSGFAGKK.V | Y | 75.09 | 1677.9314 | 17 | 1.4 | 420.4907 | 4 | 28.17 | 7 | F7:5463 | Fraction\_7\_23092022.raw |  |  |  | 6.2248E5 |  | 2.5269E6 | 6.6358E6 | 4 | 0 | 0 | 0 | 1 | 0 | 1 | 2 | 166 | 182 |  |  | PEAKS DB |
| R.SVGNYIATVTK.T | Y | 70.20 | 1151.6187 | 11 | 0.5 | 576.8169 | 2 | 44.60 | 7 | F7:12832 | Fraction\_7\_23092022.raw | 3.8953E5 | 2.632E5 | 9.612E6 | 2.0408E7 | 1.0122E7 | 3.1536E7 | 4.1914E7 | 7 | 1 | 1 | 1 | 1 | 1 | 1 | 1 | 141 | 151 |  |  | PEAKS DB |
| R.SVGNYIATVTKTLVQK.Y | Y | 68.94 | 1720.9723 | 16 | 0.4 | 574.6649 | 3 | 65.51 | 5 | F5:19322 | Fraction\_5\_23092022.raw |  |  | 9.1546E4 | 1.5229E6 | 1.0203E6 | 1.6382E6 | 8.7521E6 | 8 | 0 | 0 | 1 | 2 | 1 | 2 | 2 | 141 | 156 |  |  | PEAKS DB |
| R.SVGNYIATVTKTLVQKYNVPM(+15.99)ANIR.L | Y | 68.91 | 2795.5002 | 25 | 0.2 | 699.8824 | 4 | 73.56 | 4 | F4:23202 | Fraction\_4\_23092022.raw |  |  |  | 7.4542E5 | 7.1489E5 | 7.7341E5 |  | 3 | 0 | 0 | 0 | 1 | 1 | 1 | 0 | 141 | 165 | Oxidation (M) | M21:Oxidation (M):1000.00 | PEAKS DB |
| R.AVEYFTEC(+57.02)IR.H | Y | 68.79 | 1286.5964 | 10 | 0.5 | 644.3058 | 2 | 52.34 | 4 | F4:13677 | Fraction\_4\_23092022.raw |  |  |  | 7.516E5 | 5.0889E5 | 3.7392E5 | 2.1853E6 | 4 | 0 | 0 | 0 | 1 | 1 | 1 | 1 | 265 | 274 | Carbamidomethylation | C8:Carbamidomethylation:1000.00 | PEAKS DB |
| R.FNPC(+57.02)PYSDDTVKM(+15.99)IILTR.E | N | 67.84 | 2185.0547 | 18 | 0.7 | 729.3594 | 3 | 61.78 | 3 | F3:17358 | Fraction\_3\_23092022.raw |  |  | 2.5328E7 |  |  | 9.1102E5 | 2.8195E5 | 4 | 0 | 0 | 2 | 0 | 0 | 1 | 1 | 34 | 51 | Carbamidomethylation; Oxidation (M) | C4:Carbamidomethylation:1000.00;M13:Oxidation (M):1000.00 | PEAKS DB |
| K.TLVQKYNVPM(+15.99)ANIR.L | Y | 67.18 | 1661.8923 | 14 | 0.1 | 554.9714 | 3 | 40.24 | 7 | F7:10847 | Fraction\_7\_23092022.raw |  |  |  | 1.6047E6 | 1.5365E6 | 3.9489E6 | 1.2025E7 | 11 | 0 | 0 | 0 | 2 | 2 | 3 | 4 | 152 | 165 | Oxidation (M) | M10:Oxidation (M):1000.00 | PEAKS DB |
| R.FNPC(+57.02)PYSDDTVKMIILTR.E | N | 65.72 | 2169.0598 | 18 | 0.6 | 724.0276 | 3 | 67.77 | 3 | F3:20037 | Fraction\_3\_23092022.raw | 4.9936E5 |  | 9.8917E5 |  |  |  |  | 2 | 1 | 0 | 1 | 0 | 0 | 0 | 0 | 34 | 51 | Carbamidomethylation | C4:Carbamidomethylation:1000.00 | PEAKS DB |
| R.FNPC(+57.02)PYSDDTVK.M | N | 58.37 | 1441.6184 | 12 | 0.6 | 721.8169 | 2 | 39.39 | 7 | F7:10521 | Fraction\_7\_23092022.raw |  |  | 2.4537E7 |  |  |  | 5.0642E7 | 2 | 0 | 0 | 1 | 0 | 0 | 0 | 1 | 34 | 45 | Carbamidomethylation | C4:Carbamidomethylation:1000.00 | PEAKS DB |
| K.SQEC(+57.02)SQRIC(+57.02)ETDANYVQIIHTSSFKGTLR.T | Y | 50.71 | 3427.6248 | 29 | -1.2 | 686.5314 | 5 | 50.38 | 6 | F6:13200 | Fraction\_6\_23092022.raw |  |  |  |  |  | 2.6279E5 |  | 1 | 0 | 0 | 0 | 0 | 0 | 1 | 0 | 206 | 234 | Carbamidomethylation | C4:Carbamidomethylation:1000.00;C9:Carbamidomethylation:1000.00 | PEAKS DB |
| total 17 peptides |  |  |  |  |  |  |  |  |  |  |  |  |  |  |  |  |  |  |  |  |  |  |  |  |  |  |  |  |  |  |  |
| --- | --- | --- | --- | --- | --- | --- | --- | --- | --- | --- | --- | --- | --- | --- | --- | --- | --- | --- | --- | --- | --- | --- | --- | --- | --- | --- | --- | --- | --- | --- | --- |

A0A0M3KKW3|PA1\_VESBA

back to list

  

| Protein Coverage
| Supporting Peptides
|

Protein Coverage:

Supporting Peptides:

| Peptide | Uniq | -10lgP | Mass | Length | ppm | m/z | z | RT | Fraction | Scan | Source File | Area F1 | Area F2 | Area F3 | Area F4 | Area F5 | Area F6 | Area F7 | #Feature | #Feature F1 | #Feature F2 | #Feature F3 | #Feature F4 | #Feature F5 | #Feature F6 | #Feature F7 | Start | End | PTM | AScore | Found By |
| --- | --- | --- | --- | --- | --- | --- | --- | --- | --- | --- | --- | --- | --- | --- | --- | --- | --- | --- | --- | --- | --- | --- | --- | --- | --- | --- | --- | --- | --- | --- | --- |
| R.LIGHSLGAHTSGFAGK.K | N | 119.60 | 1551.8157 | 16 | 0.5 | 776.9155 | 2 | 28.03 | 7 | F7:5409 | Fraction\_7\_23092022.raw |  |  | 2.1628E6 | 1.6313E7 | 5.047E6 | 3.3174E7 | 1.2635E8 | 13 | 0 | 0 | 2 | 3 | 3 | 2 | 3 | 133 | 148 |  |  | PEAKS DB |
| K.YSEIIGLDPAGPSFK.S | N | 110.20 | 1592.8086 | 15 | 1.4 | 797.4127 | 2 | 66.23 | 5 | F5:19733 | Fraction\_5\_23092022.raw | 3.4371E6 | 3.3474E6 | 1.0312E8 | 1.5296E8 | 1.0352E8 | 5.9801E7 |  | 12 | 1 | 1 | 3 | 3 | 2 | 2 | 0 | 158 | 172 |  |  | PEAKS DB |
| K.TGSFYVPVESKAPYC(+57.02)NNK.G | N | 97.81 | 2059.9673 | 18 | 0.0 | 1030.9910 | 2 | 45.28 | 3 | F3:9956 | Fraction\_3\_23092022.raw |  |  | 3.5089E8 | 1.0426E8 |  | 1.0646E6 |  | 12 | 0 | 0 | 7 | 4 | 0 | 1 | 0 | 279 | 296 | Carbamidomethylation | C15:Carbamidomethylation:1000.00 | PEAKS DB |
| K.TYPKTGSFYVPVESKAPYC(+57.02)NNK.G | N | 94.16 | 2549.2261 | 22 | 0.5 | 638.3141 | 4 | 43.76 | 3 | F3:9275 | Fraction\_3\_23092022.raw |  |  | 5.2116E6 | 4.0395E6 |  |  |  | 4 | 0 | 0 | 2 | 2 | 0 | 0 | 0 | 275 | 296 | Carbamidomethylation | C19:Carbamidomethylation:1000.00 | PEAKS DB |
| K.TYPKTGSFYVPVESK.A | N | 93.80 | 1701.8613 | 15 | 0.4 | 851.9383 | 2 | 45.22 | 3 | F3:9935 | Fraction\_3\_23092022.raw | 1.9E5 | 2.1319E6 | 2.3998E7 | 7.7395E7 | 2.1649E6 | 1.0072E7 | 4.9841E6 | 14 | 1 | 2 | 3 | 2 | 2 | 2 | 2 | 275 | 289 |  |  | PEAKS DB |
| K.TGSFYVPVESKAPYC(+57.02)NNKGKII | N | 93.49 | 2471.2517 | 22 | 0.6 | 618.8206 | 4 | 48.89 | 4 | F4:12117 | Fraction\_4\_23092022.raw |  | 2.8013E6 | 5.6198E6 | 8.8359E6 | 3.2966E5 | 1.2371E6 | 1.0754E7 | 11 | 0 | 2 | 2 | 2 | 1 | 2 | 2 | 279 | 300 | Carbamidomethylation | C15:Carbamidomethylation:1000.00 | PEAKS DB |
| K.TGSFYVPVESKAPYC(+57.02)NNKGK.I | N | 88.43 | 2245.0837 | 20 | -0.5 | 749.3682 | 3 | 38.45 | 4 | F4:7505 | Fraction\_4\_23092022.raw |  | 2.2402E6 | 9.7062E6 | 4.9178E7 | 2.0417E6 | 5.5689E6 | 1.0137E7 | 15 | 0 | 1 | 3 | 4 | 2 | 2 | 3 | 279 | 298 | Carbamidomethylation | C15:Carbamidomethylation:1000.00 | PEAKS DB |
| K.TGSFYVPVESK.A | N | 86.10 | 1212.6027 | 11 | 1.0 | 607.3092 | 2 | 47.23 | 4 | F4:11441 | Fraction\_4\_23092022.raw | 1.7405E6 | 3.3208E6 | 1.4279E8 | 1.6292E8 | 3.0697E6 | 1.7065E7 | 5.4182E6 | 10 | 1 | 1 | 2 | 2 | 1 | 2 | 1 | 279 | 289 |  |  | PEAKS DB |
| R.LIGHSLGAHTSGFAGKK.V | N | 77.30 | 1679.9106 | 17 | -0.2 | 420.9848 | 4 | 23.12 | 7 | F7:3582 | Fraction\_7\_23092022.raw |  |  |  |  |  |  | 5.9502E6 | 1 | 0 | 0 | 0 | 0 | 0 | 0 | 1 | 133 | 149 |  |  | PEAKS DB |
| R.LVGNYIATVTK.M | N | 75.39 | 1177.6707 | 11 | 0.1 | 589.8427 | 2 | 48.23 | 5 | F5:11564 | Fraction\_5\_23092022.raw | 4.9288E6 | 2.5048E6 | 4.5191E7 | 4.3416E8 | 3.1788E8 | 3.9701E7 | 1.0579E8 | 10 | 1 | 1 | 1 | 2 | 2 | 1 | 2 | 108 | 118 |  |  | PEAKS DB |
| FNPC(+57.02)PYSDDTVKM(+15.99)IILTR.E | N | 67.84 | 2185.0547 | 18 | 0.7 | 729.3594 | 3 | 61.78 | 3 | F3:17358 | Fraction\_3\_23092022.raw |  |  | 2.5328E7 |  |  | 9.1102E5 | 2.8195E5 | 4 | 0 | 0 | 2 | 0 | 0 | 1 | 1 | 1 | 18 | Carbamidomethylation; Oxidation (M) | C4:Carbamidomethylation:1000.00;M13:Oxidation (M):1000.00 | PEAKS DB |
| K.HEC(+57.02)C(+57.02)LIGVPQSK.K | N | 66.41 | 1426.6697 | 12 | 0.1 | 714.3422 | 2 | 33.72 | 7 | F7:7919 | Fraction\_7\_23092022.raw |  |  |  |  |  |  | 1.5167E5 | 1 | 0 | 0 | 0 | 0 | 0 | 0 | 1 | 242 | 253 | Carbamidomethylation | C3:Carbamidomethylation:1000.00;C4:Carbamidomethylation:1000.00 | PEAKS DB |
| FNPC(+57.02)PYSDDTVKMIILTR.E | N | 65.72 | 2169.0598 | 18 | 0.6 | 724.0276 | 3 | 67.77 | 3 | F3:20037 | Fraction\_3\_23092022.raw | 4.9936E5 |  | 9.8917E5 |  |  |  |  | 2 | 1 | 0 | 1 | 0 | 0 | 0 | 0 | 1 | 18 | Carbamidomethylation | C4:Carbamidomethylation:1000.00 | PEAKS DB |
| R.NEC(+57.02)VC(+57.02)VGLNAKTYPK.T | N | 64.53 | 1751.8335 | 15 | 0.2 | 876.9242 | 2 | 35.82 | 7 | F7:8817 | Fraction\_7\_23092022.raw |  |  | 4.6572E5 | 5.8753E5 | 1.8031E6 |  | 5.1284E6 | 9 | 0 | 0 | 1 | 1 | 3 | 0 | 4 | 264 | 278 | Carbamidomethylation | C3:Carbamidomethylation:1000.00;C5:Carbamidomethylation:1000.00 | PEAKS DB |
| FNPC(+57.02)PYSDDTVKM(+15.99)IILTRENK.K | N | 62.94 | 2556.2351 | 21 | -0.4 | 640.0658 | 4 | 54.01 | 7 | F7:17800 | Fraction\_7\_23092022.raw |  |  |  |  |  |  | 1.9691E6 | 1 | 0 | 0 | 0 | 0 | 0 | 0 | 1 | 1 | 21 | Carbamidomethylation; Oxidation (M) | C4:Carbamidomethylation:1000.00;M13:Oxidation (M):1000.00 | PEAKS DB |
| K.C(+57.02)TRNEC(+57.02)VC(+57.02)VGLNAK.T | N | 62.11 | 1679.7542 | 14 | 0.2 | 560.9254 | 3 | 30.48 | 7 | F7:6463 | Fraction\_7\_23092022.raw |  |  |  |  |  |  | 1.1536E6 | 2 | 0 | 0 | 0 | 0 | 0 | 0 | 2 | 261 | 274 | Carbamidomethylation | C1:Carbamidomethylation:1000.00;C6:Carbamidomethylation:1000.00;C8:Carbamidomethylation:1000.00 | PEAKS DB |
| R.ENKKHDFYTLDTIK.K | Y | 61.76 | 1750.8889 | 14 | 0.3 | 584.6371 | 3 | 33.91 | 7 | F7:7990 | Fraction\_7\_23092022.raw |  |  |  |  |  |  | 4.2338E5 | 1 | 0 | 0 | 0 | 0 | 0 | 0 | 1 | 19 | 32 |  |  | PEAKS DB |
| R.NEC(+57.02)VC(+57.02)VGLNAK.T | N | 61.26 | 1262.5747 | 11 | 0.2 | 632.2947 | 2 | 34.83 | 7 | F7:8388 | Fraction\_7\_23092022.raw |  |  | 3.1121E6 | 3.1889E6 | 7.1825E5 |  | 3.0319E6 | 10 | 0 | 0 | 2 | 4 | 2 | 0 | 2 | 264 | 274 | Carbamidomethylation | C3:Carbamidomethylation:1000.00;C5:Carbamidomethylation:1000.00 | PEAKS DB |
| FNPC(+57.02)PYSDDTVK.M | N | 58.37 | 1441.6184 | 12 | 0.6 | 721.8169 | 2 | 39.39 | 7 | F7:10521 | Fraction\_7\_23092022.raw |  |  | 2.4537E7 |  |  |  | 5.0642E7 | 2 | 0 | 0 | 1 | 0 | 0 | 0 | 1 | 1 | 12 | Carbamidomethylation | C4:Carbamidomethylation:1000.00 | PEAKS DB |
| K.KHDFYTLDTIK.K | N | 54.33 | 1379.7085 | 11 | 0.2 | 460.9102 | 3 | 38.68 | 7 | F7:10126 | Fraction\_7\_23092022.raw |  |  |  |  |  |  | 6.3068E5 | 1 | 0 | 0 | 0 | 0 | 0 | 0 | 1 | 22 | 32 |  |  | PEAKS DB |
| K.TYPKTGSFYVPVESKAPYC(+57.02)NNKGK.I | N | 50.63 | 2734.3425 | 24 | 0.1 | 547.8759 | 5 | 38.42 | 4 | F4:7511 | Fraction\_4\_23092022.raw |  |  |  | 5.1464E5 |  |  |  | 1 | 0 | 0 | 0 | 1 | 0 | 0 | 0 | 275 | 298 | Carbamidomethylation | C19:Carbamidomethylation:1000.00 | PEAKS DB |
| total 21 peptides |  |  |  |  |  |  |  |  |  |  |  |  |  |  |  |  |  |  |  |  |  |  |  |  |  |  |  |  |  |  |  |
| --- | --- | --- | --- | --- | --- | --- | --- | --- | --- | --- | --- | --- | --- | --- | --- | --- | --- | --- | --- | --- | --- | --- | --- | --- | --- | --- | --- | --- | --- | --- | --- |

P00760|TRY1\_BOVIN

back to list

  

| Protein Coverage
| Supporting Peptides
|

Protein Coverage:

Supporting Peptides:

| Peptide | Uniq | -10lgP | Mass | Length | ppm | m/z | z | RT | Fraction | Scan | Source File | Area F1 | Area F2 | Area F3 | Area F4 | Area F5 | Area F6 | Area F7 | #Feature | #Feature F1 | #Feature F2 | #Feature F3 | #Feature F4 | #Feature F5 | #Feature F6 | #Feature F7 | Start | End | PTM | AScore | Found By |
| --- | --- | --- | --- | --- | --- | --- | --- | --- | --- | --- | --- | --- | --- | --- | --- | --- | --- | --- | --- | --- | --- | --- | --- | --- | --- | --- | --- | --- | --- | --- | --- |
| R.LGEDNINVVEGNEQFISASK.S | Y | 121.50 | 2162.0491 | 20 | 1.0 | 721.6910 | 3 | 59.80 | 5 | F5:16776 | Fraction\_5\_23092022.raw | 2.2434E10 | 1.6266E10 | 6.5593E9 | 1.4506E10 | 1.5645E10 | 1.6786E10 | 1.0288E10 | 124 | 15 | 13 | 10 | 21 | 21 | 14 | 30 | 73 | 92 |  |  | PEAKS DB |
| K.SIVHPSYNSNTLNNDIMLIK.L | Y | 117.24 | 2272.1521 | 20 | 0.2 | 1137.0835 | 2 | 60.06 | 5 | F5:16905 | Fraction\_5\_23092022.raw | 7.7815E9 | 8.7199E7 | 2.1298E7 | 2.2202E8 | 6.0417E7 | 9.6987E7 | 2.0008E7 | 25 | 12 | 2 | 2 | 4 | 2 | 2 | 1 | 93 | 112 |  |  | PEAKS DB |
| R.VASISLPTSC(+57.02)ASAGTQC(+57.02)LISGWGNTK.S | Y | 114.17 | 2665.2839 | 26 | 0.6 | 1333.6500 | 2 | 71.44 | 5 | F5:22034 | Fraction\_5\_23092022.raw | 8.4621E8 | 9.7042E5 | 7.1976E4 | 1.5115E5 | 3.8809E7 | 2.4884E5 |  | 17 | 9 | 2 | 1 | 1 | 3 | 1 | 0 | 123 | 148 | Carbamidomethylation | C10:Carbamidomethylation:1000.00;C17:Carbamidomethylation:1000.00 | PEAKS DB |
| K.LQGIVSWGSGC(+57.02)AQK.N | Y | 110.64 | 1489.7347 | 14 | 1.0 | 745.8754 | 2 | 50.99 | 5 | F5:12871 | Fraction\_5\_23092022.raw | 2.78E9 | 1.5163E8 | 1.6078E7 | 1.6759E8 | 3.4116E8 | 2.0995E8 | 6.2589E7 | 22 | 7 | 1 | 2 | 4 | 2 | 5 | 1 | 210 | 223 | Carbamidomethylation | C11:Carbamidomethylation:1000.00 | PEAKS DB |
| K.SAYPGQITSNMFC(+57.02)AGYLEGGK.D | Y | 106.76 | 2250.0085 | 21 | 1.0 | 1126.0127 | 2 | 69.01 | 5 | F5:20902 | Fraction\_5\_23092022.raw | 5.3917E8 | 5.8696E5 | 5.5681E5 | 3.2969E5 | 1.0035E6 | 4.6883E5 |  | 9 | 3 | 1 | 1 | 1 | 2 | 1 | 0 | 173 | 193 | Carbamidomethylation | C13:Carbamidomethylation:1000.00 | PEAKS DB |
| K.SIVHPSYNSNTLNNDIM(+15.99)LIK.L | Y | 104.45 | 2288.1470 | 20 | 0.5 | 763.7233 | 3 | 56.09 | 7 | F7:19157 | Fraction\_7\_23092022.raw | 5.7658E9 | 1.038E9 | 2.2611E8 | 8.2567E8 | 4.5647E8 | 3.7932E8 | 2.3519E8 | 51 | 8 | 8 | 10 | 4 | 6 | 8 | 7 | 93 | 112 | Oxidation (M) | M17:Oxidation (M):1000.00 | PEAKS DB |
| K.SAYPGQITSNM(+15.99)FC(+57.02)AGYLEGGK.D | Y | 98.79 | 2266.0034 | 21 | 0.2 | 1134.0092 | 2 | 59.38 | 5 | F5:16637 | Fraction\_5\_23092022.raw | 4.4784E8 | 2.8128E7 | 8.2349E6 | 1.2119E7 | 2.0754E7 | 1.3701E7 | 1.0659E7 | 38 | 14 | 4 | 4 | 4 | 4 | 4 | 4 | 173 | 193 | Oxidation (M); Carbamidomethylation | M11:Oxidation (M):1000.00;C13:Carbamidomethylation:1000.00 | PEAKS DB |
| K.SGIQVRLGEDNINVVEGNEQFISASK.S | Y | 90.47 | 2802.4148 | 26 | 1.5 | 935.1470 | 3 | 63.71 | 5 | F5:18538 | Fraction\_5\_23092022.raw | 1.2244E5 | 2.4081E5 | 4.3185E4 | 1.5418E5 | 2.0903E5 | 1.9088E5 |  | 6 | 1 | 1 | 1 | 1 | 1 | 1 | 0 | 67 | 92 |  |  | PEAKS DB |
| K.SIVHPSYNSNTLNNDIMLIKLK.S | Y | 86.37 | 2513.3311 | 22 | -0.7 | 838.7837 | 3 | 61.82 | 1 | F1:16814 | Fraction\_1\_23092022r.raw | 6.9245E5 |  |  |  |  |  |  | 1 | 1 | 0 | 0 | 0 | 0 | 0 | 0 | 93 | 114 |  |  | PEAKS DB |
| K.SIVHPSYNSNTLNNDIM(+15.99)LIKLK.S | Y | 85.48 | 2529.3262 | 22 | 0.8 | 633.3394 | 4 | 58.89 | 1 | F1:15517 | Fraction\_1\_23092022r.raw | 6.2078E5 |  |  |  |  | 9.6013E4 |  | 2 | 1 | 0 | 0 | 0 | 0 | 1 | 0 | 93 | 114 | Oxidation (M) | M17:Oxidation (M):1000.00 | PEAKS DB |
| K.DSC(+57.02)QGDSGGPVVC(+57.02)SGK.L | Y | 84.90 | 1608.6508 | 16 | 0.5 | 805.3331 | 2 | 22.93 | 5 | F5:3388 | Fraction\_5\_23092022.raw | 1.9478E6 |  |  |  | 2.0258E5 |  |  | 2 | 1 | 0 | 0 | 0 | 1 | 0 | 0 | 194 | 209 | Carbamidomethylation | C3:Carbamidomethylation:1000.00;C13:Carbamidomethylation:1000.00 | PEAKS DB |
| K.APILSDSSC(+57.02)K.S | Y | 76.62 | 1076.5172 | 10 | -0.4 | 539.2657 | 2 | 27.37 | 7 | F7:5117 | Fraction\_7\_23092022.raw | 9.1494E6 | 8.0298E5 | 1.7801E6 | 1.8968E6 | 1.2655E6 | 1.7225E6 | 1.7471E6 | 8 | 1 | 1 | 1 | 1 | 1 | 1 | 2 | 163 | 172 | Carbamidomethylation | C9:Carbamidomethylation:1000.00 | PEAKS DB |
| K.SSGTSYPDVLK.C | Y | 65.96 | 1152.5663 | 11 | 1.5 | 577.2913 | 2 | 40.70 | 7 | F7:11046 | Fraction\_7\_23092022.raw | 7.4472E8 | 7.9108E7 | 1.1101E9 | 1.4888E9 | 1.082E9 | 1.5073E9 | 1.1447E9 | 17 | 3 | 1 | 2 | 3 | 1 | 2 | 5 | 149 | 159 |  |  | PEAKS DB |
| K.SAYPGQITSNMFC(+57.02)AGYLEGGKDSC(+57.02)QGDSGGPVVC(+57.02)SGK.L | Y | 63.79 | 3840.6487 | 37 | 1.2 | 1281.2251 | 3 | 63.33 | 1 | F1:17514 | Fraction\_1\_23092022r.raw | 5.536E5 |  |  |  |  |  |  | 1 | 1 | 0 | 0 | 0 | 0 | 0 | 0 | 173 | 209 | Carbamidomethylation | C13:Carbamidomethylation:1000.00;C24:Carbamidomethylation:1000.00;C34:Carbamidomethylation:1000.00 | PEAKS DB |
| K.VC(+57.02)NYVSWIK.Q | Y | 52.34 | 1167.5747 | 9 | 0.5 | 584.7949 | 2 | 56.71 | 5 | F5:15392 | Fraction\_5\_23092022.raw | 1.8675E9 |  | 7.4896E6 | 8.5439E7 | 2.1758E8 | 1.3813E8 |  | 7 | 3 | 0 | 1 | 1 | 1 | 1 | 0 | 232 | 240 | Carbamidomethylation | C2:Carbamidomethylation:1000.00 | PEAKS DB |
| K.APILSDSSC(+57.02)KSAYPGQITSNM(+15.99)FC(+57.02)AGYLEGGK.D | Y | 51.76 | 3324.5100 | 31 | 0.2 | 832.1349 | 4 | 48.71 | 7 | F7:14875 | Fraction\_7\_23092022.raw |  |  |  |  | 1.5856E6 |  | 2.7881E7 | 2 | 0 | 0 | 0 | 0 | 1 | 0 | 1 | 163 | 193 | Carbamidomethylation; Oxidation (M) | C9:Carbamidomethylation:1000.00;M21:Oxidation (M):1000.00;C23:Carbamidomethylation:1000.00 | PEAKS DB |
| K.C(+57.02)LKAPILSDSSC(+57.02)K.S | Y | 47.72 | 1477.7268 | 13 | 0.6 | 493.5832 | 3 | 36.92 | 1 | F1:5993 | Fraction\_1\_23092022r.raw | 2.7394E4 |  |  |  |  |  |  | 1 | 1 | 0 | 0 | 0 | 0 | 0 | 0 | 160 | 172 | Carbamidomethylation | C1:Carbamidomethylation:1000.00;C12:Carbamidomethylation:1000.00 | PEAKS DB |
| total 17 peptides |  |  |  |  |  |  |  |  |  |  |  |  |  |  |  |  |  |  |  |  |  |  |  |  |  |  |  |  |  |  |  |
| --- | --- | --- | --- | --- | --- | --- | --- | --- | --- | --- | --- | --- | --- | --- | --- | --- | --- | --- | --- | --- | --- | --- | --- | --- | --- | --- | --- | --- | --- | --- | --- |

TRINITY\_DN6370\_c0\_g1\_i1.p1

back to list

  

| Protein Coverage
| Supporting Peptides
|

Protein Coverage:

Supporting Peptides:

| Peptide | Uniq | -10lgP | Mass | Length | ppm | m/z | z | RT | Fraction | Scan | Source File | Area F1 | Area F2 | Area F3 | Area F4 | Area F5 | Area F6 | Area F7 | #Feature | #Feature F1 | #Feature F2 | #Feature F3 | #Feature F4 | #Feature F5 | #Feature F6 | #Feature F7 | Start | End | PTM | AScore | Found By |
| --- | --- | --- | --- | --- | --- | --- | --- | --- | --- | --- | --- | --- | --- | --- | --- | --- | --- | --- | --- | --- | --- | --- | --- | --- | --- | --- | --- | --- | --- | --- | --- |
| R.SVVKASGLTNAEKLEILKQHNEFR.Q | Y | 128.24 | 2710.4766 | 24 | -0.5 | 678.6261 | 4 | 41.71 | 7 | F7:11488 | Fraction\_7\_23092022.raw |  |  |  | 1.8012E6 | 1.4014E5 | 9.5343E5 | 1.2374E8 | 7 | 0 | 0 | 0 | 1 | 1 | 1 | 4 | 52 | 75 |  |  | PEAKS DB |
| K.ASGLTNAEKLEILKQHNEFR.Q | Y | 111.21 | 2297.2126 | 20 | -0.5 | 766.7444 | 3 | 41.28 | 7 | F7:11300 | Fraction\_7\_23092022.raw |  |  | 1.0005E6 | 5.4413E7 | 4.0692E6 | 3.7503E7 | 2.527E8 | 11 | 0 | 0 | 1 | 2 | 2 | 2 | 4 | 56 | 75 |  |  | PEAKS DB |
| K.ASGLTNAEKLEILKQHNEFRQK.V | Y | 99.09 | 2553.3662 | 22 | 3.0 | 511.6820 | 5 | 36.03 | 7 | F7:8978 | Fraction\_7\_23092022.raw |  |  |  | 4.3408E6 | 8.2582E5 | 3.2321E6 | 5.8582E8 | 8 | 0 | 0 | 0 | 1 | 1 | 1 | 5 | 56 | 77 |  |  | PEAKS DB |
| K.AWADEVKDYQYGSPNKGR.K | Y | 88.72 | 2082.9758 | 18 | 0.6 | 695.3329 | 3 | 40.36 | 7 | F7:10894 | Fraction\_7\_23092022.raw |  |  |  | 7.9142E5 |  | 2.9073E6 | 3.9955E6 | 6 | 0 | 0 | 0 | 2 | 0 | 2 | 2 | 155 | 172 |  |  | PEAKS DB |
| K.YGHDDC(+57.02)RNTAKYLVGQNIAK.Q | Y | 86.11 | 2322.1174 | 20 | 0.6 | 581.5370 | 4 | 32.01 | 7 | F7:7134 | Fraction\_7\_23092022.raw |  |  |  |  |  |  | 3.5541E6 | 3 | 0 | 0 | 0 | 0 | 0 | 0 | 3 | 119 | 138 | Carbamidomethylation | C6:Carbamidomethylation:1000.00 | PEAKS DB |
| R.SVVKASGLTNAEKLEILK.Q | Y | 78.58 | 1899.1040 | 18 | -0.1 | 634.0419 | 3 | 45.97 | 7 | F7:13452 | Fraction\_7\_23092022.raw |  |  |  | 5.4849E6 |  | 3.3763E6 | 2.4312E7 | 7 | 0 | 0 | 0 | 2 | 0 | 2 | 3 | 52 | 69 |  |  | PEAKS DB |
| K.YLVGQNIAKQSTTAASFAPVSNM(+15.99)VK.A | Y | 74.46 | 2640.3582 | 25 | 0.3 | 881.1270 | 3 | 52.74 | 4 | F4:13835 | Fraction\_4\_23092022.raw |  |  |  | 3.0247E5 |  | 7.2766E5 |  | 2 | 0 | 0 | 0 | 1 | 0 | 1 | 0 | 130 | 154 | Oxidation (M) | M23:Oxidation (M):1000.00 | PEAKS DB |
| R.NTAKYLVGQNIAK.Q | Y | 74.15 | 1418.7881 | 13 | -0.3 | 710.4011 | 2 | 31.85 | 7 | F7:7110 | Fraction\_7\_23092022.raw |  |  |  |  |  | 3.7878E5 | 1.2002E6 | 4 | 0 | 0 | 0 | 0 | 0 | 2 | 2 | 126 | 138 |  |  | PEAKS DB |
| K.AWADEVKDYQYGSPNK.G | Y | 70.19 | 1869.8533 | 16 | -0.1 | 935.9338 | 2 | 45.49 | 6 | F6:11027 | Fraction\_6\_23092022.raw |  |  |  | 2.1122E5 |  | 7.5074E5 |  | 3 | 0 | 0 | 0 | 1 | 0 | 2 | 0 | 155 | 170 |  |  | PEAKS DB |
| K.ASGLTNAEKLEILK.Q | Y | 69.29 | 1485.8402 | 14 | 0.9 | 496.2878 | 3 | 47.09 | 7 | F7:13989 | Fraction\_7\_23092022.raw |  |  | 2.9631E6 | 2.2338E7 | 1.8351E6 | 9.7441E6 | 3.2738E7 | 10 | 0 | 0 | 2 | 2 | 2 | 2 | 2 | 56 | 69 |  |  | PEAKS DB |
| K.YLVGQNIAK.Q | Y | 60.09 | 1004.5654 | 9 | 0.2 | 503.2901 | 2 | 36.33 | 7 | F7:9050 | Fraction\_7\_23092022.raw |  |  |  | 8.847E5 |  | 1.0698E7 | 1.2472E7 | 3 | 0 | 0 | 0 | 1 | 0 | 1 | 1 | 130 | 138 |  |  | PEAKS DB |
| K.QSTTAASFAPVSNM(+15.99)VK.A | Y | 59.32 | 1653.8032 | 16 | 0.6 | 827.9094 | 2 | 40.92 | 6 | F6:8967 | Fraction\_6\_23092022.raw |  |  |  | 5.0088E5 |  | 7.6404E5 | 1.1568E5 | 3 | 0 | 0 | 0 | 1 | 0 | 1 | 1 | 139 | 154 | Oxidation (M) | M14:Oxidation (M):1000.00 | PEAKS DB |
| K.AWADEVKDYQYGSPNKGRK.I | Y | 58.93 | 2211.0708 | 19 | -0.4 | 553.7748 | 4 | 35.02 | 7 | F7:8490 | Fraction\_7\_23092022.raw |  |  |  |  |  |  | 5.578E5 | 1 | 0 | 0 | 0 | 0 | 0 | 0 | 1 | 155 | 173 |  |  | PEAKS DB |
| R.GLETRGNPGPQPPAK.S | N | 57.93 | 1517.7949 | 15 | -0.4 | 506.9387 | 3 | 22.00 | 7 | F7:3354 | Fraction\_7\_23092022.raw |  |  |  |  |  |  | 7.2952E4 | 1 | 0 | 0 | 0 | 0 | 0 | 0 | 1 | 81 | 95 |  |  | PEAKS DB |
| K.IAQVWASQC(+57.02)K.Y | Y | 57.63 | 1189.5913 | 10 | -0.5 | 595.8026 | 2 | 36.74 | 6 | F6:7122 | Fraction\_6\_23092022.raw |  |  |  |  |  | 5.8961E4 |  | 1 | 0 | 0 | 0 | 0 | 0 | 1 | 0 | 109 | 118 | Carbamidomethylation | C9:Carbamidomethylation:1000.00 | PEAKS DB |
| R.GNPGPQPPAK.S | N | 52.98 | 961.4981 | 10 | 0.3 | 481.7565 | 2 | 17.36 | 7 | F7:2608 | Fraction\_7\_23092022.raw |  |  |  |  |  |  | 3.8663E4 | 1 | 0 | 0 | 0 | 0 | 0 | 0 | 1 | 86 | 95 |  |  | PEAKS DB |
| K.IAQVWASQC(+57.02)KYGHDDC(+57.02)R.N | Y | 48.48 | 2092.9207 | 17 | -0.1 | 524.2374 | 4 | 31.26 | 6 | F6:5037 | Fraction\_6\_23092022.raw |  |  |  |  |  | 1.5963E5 |  | 1 | 0 | 0 | 0 | 0 | 0 | 1 | 0 | 109 | 125 | Carbamidomethylation | C9:Carbamidomethylation:1000.00;C16:Carbamidomethylation:1000.00 | PEAKS DB |
| total 17 peptides |  |  |  |  |  |  |  |  |  |  |  |  |  |  |  |  |  |  |  |  |  |  |  |  |  |  |  |  |  |  |  |
| --- | --- | --- | --- | --- | --- | --- | --- | --- | --- | --- | --- | --- | --- | --- | --- | --- | --- | --- | --- | --- | --- | --- | --- | --- | --- | --- | --- | --- | --- | --- | --- |

TRINITY\_DN10047\_c0\_g1\_i1.p1

back to list

  

| Protein Coverage
| Supporting Peptides
|

Protein Coverage:

Supporting Peptides:

| Peptide | Uniq | -10lgP | Mass | Length | ppm | m/z | z | RT | Fraction | Scan | Source File | Area F1 | Area F2 | Area F3 | Area F4 | Area F5 | Area F6 | Area F7 | #Feature | #Feature F1 | #Feature F2 | #Feature F3 | #Feature F4 | #Feature F5 | #Feature F6 | #Feature F7 | Start | End | PTM | AScore | Found By |
| --- | --- | --- | --- | --- | --- | --- | --- | --- | --- | --- | --- | --- | --- | --- | --- | --- | --- | --- | --- | --- | --- | --- | --- | --- | --- | --- | --- | --- | --- | --- | --- |
| R.GQYYLHQFVAGQPDLNYR.N | Y | 108.15 | 2168.0439 | 18 | 0.0 | 1085.0293 | 2 | 56.80 | 2 | F2:14732 | Fraction\_2\_23092022.raw | 1.552E7 | 1.7261E8 | 3.7628E6 | 2.3123E6 | 1.1948E6 | 1.6543E6 |  | 8 | 1 | 2 | 2 | 1 | 1 | 1 | 0 | 179 | 196 |  |  | PEAKS DB |
| K.TPIIESGSLESILVTEK.V | Y | 102.40 | 1814.9877 | 17 | -0.6 | 908.5006 | 2 | 74.02 | 2 | F2:22451 | Fraction\_2\_23092022.raw | 5.1461E6 | 1.1492E7 | 8.2889E5 | 6.9336E4 | 1.196E5 | 1.6955E5 |  | 8 | 2 | 2 | 1 | 1 | 1 | 1 | 0 | 478 | 494 |  |  | PEAKS DB |
| R.RLPGSTVVLLINFSNSDIQVDAR.T | Y | 101.68 | 2513.3601 | 23 | 0.3 | 838.7943 | 3 | 78.23 | 2 | F2:24381 | Fraction\_2\_23092022.raw |  | 9.4099E5 |  |  |  |  |  | 1 | 0 | 1 | 0 | 0 | 0 | 0 | 0 | 501 | 523 |  |  | PEAKS DB |
| R.DFTYEETVDPAGC(+57.02)QAGPDR.Y | Y | 96.40 | 2126.8850 | 19 | 0.8 | 1064.4507 | 2 | 52.44 | 2 | F2:12778 | Fraction\_2\_23092022.raw | 3.8165E5 | 5.7528E5 |  |  |  |  |  | 2 | 1 | 1 | 0 | 0 | 0 | 0 | 0 | 395 | 413 | Carbamidomethylation | C13:Carbamidomethylation:1000.00 | PEAKS DB |
| R.DFTYEETVDPAGC(+57.02)QAGPDRYYLK.S | Y | 92.19 | 2694.1907 | 23 | 0.6 | 899.0714 | 3 | 58.58 | 3 | F3:15949 | Fraction\_3\_23092022.raw | 7.9891E6 | 4.2569E7 | 1.3343E6 | 3.9536E5 |  |  |  | 6 | 1 | 3 | 1 | 1 | 0 | 0 | 0 | 395 | 417 | Carbamidomethylation | C13:Carbamidomethylation:1000.00 | PEAKS DB |
| K.KTPIIESGSLESILVTEK.V | Y | 91.00 | 1943.0826 | 18 | 0.6 | 648.7019 | 3 | 63.38 | 2 | F2:17714 | Fraction\_2\_23092022.raw | 2.5309E6 | 2.6725E6 | 2.1154E5 |  |  |  |  | 5 | 2 | 2 | 1 | 0 | 0 | 0 | 0 | 477 | 494 |  |  | PEAKS DB |
| R.SFKDSNGDGIGDLNGITSK.L | Y | 89.83 | 1923.9174 | 19 | 1.9 | 962.9678 | 2 | 46.02 | 2 | F2:9900 | Fraction\_2\_23092022.raw | 1.569E6 | 2.7587E6 |  |  |  |  |  | 4 | 2 | 2 | 0 | 0 | 0 | 0 | 0 | 38 | 56 |  |  | PEAKS DB |
| R.GQYYLHQFVAGQPDLNYRNK.D | Y | 87.05 | 2410.1819 | 20 | 0.8 | 603.5532 | 4 | 49.47 | 2 | F2:11448 | Fraction\_2\_23092022.raw |  | 1.488E6 |  |  |  |  |  | 2 | 0 | 2 | 0 | 0 | 0 | 0 | 0 | 179 | 198 |  |  | PEAKS DB |
| K.TLNLDIQKNSLISHYK.V | Y | 79.80 | 1886.0261 | 16 | 0.5 | 629.6830 | 3 | 50.12 | 1 | F1:11607 | Fraction\_1\_23092022r.raw | 9.9281E5 | 2.0578E6 |  |  |  |  |  | 3 | 1 | 2 | 0 | 0 | 0 | 0 | 0 | 452 | 467 |  |  | PEAKS DB |
| K.KTPIIESGSLESILVTEKVLGVVR.R | Y | 79.54 | 2566.4944 | 24 | 0.2 | 642.6310 | 4 | 79.40 | 1 | F1:24709 | Fraction\_1\_23092022r.raw | 4.1597E5 | 1.0039E6 |  |  |  |  |  | 4 | 2 | 2 | 0 | 0 | 0 | 0 | 0 | 477 | 500 |  |  | PEAKS DB |
| K.NSLIYQIYPR.S | Y | 77.99 | 1265.6768 | 10 | 0.3 | 633.8458 | 2 | 60.11 | 2 | F2:16228 | Fraction\_2\_23092022.raw | 1.2519E7 | 4.3107E7 |  |  |  | 3.7005E6 |  | 3 | 1 | 1 | 0 | 0 | 0 | 1 | 0 | 28 | 37 |  |  | PEAKS DB |
| K.IKPYDDYYIWR.N | Y | 75.89 | 1530.7506 | 11 | 0.1 | 511.2575 | 3 | 58.17 | 1 | F1:15221 | Fraction\_1\_23092022r.raw | 1.3678E6 | 1.208E6 |  |  |  |  |  | 2 | 1 | 1 | 0 | 0 | 0 | 0 | 0 | 138 | 148 |  |  | PEAKS DB |
| R.FLDEPSANRPDLPPDDYDTLDHIYTK.N | Y | 67.57 | 3046.4194 | 26 | 1.2 | 1016.4817 | 3 | 61.15 | 1 | F1:16482 | Fraction\_1\_23092022r.raw | 4.5112E5 | 3.2929E7 |  |  |  |  |  | 3 | 1 | 2 | 0 | 0 | 0 | 0 | 0 | 233 | 258 |  |  | PEAKS DB |
| K.DTVANWVVGNHDNHR.A | Y | 66.40 | 1732.8030 | 15 | 0.6 | 578.6086 | 3 | 40.87 | 1 | F1:7531 | Fraction\_1\_23092022r.raw | 3.2277E5 |  |  |  |  |  |  | 1 | 1 | 0 | 0 | 0 | 0 | 0 | 0 | 343 | 357 |  |  | PEAKS DB |
| K.DSNGDGIGDLNGITSK.L | Y | 62.55 | 1561.7219 | 16 | 0.5 | 781.8687 | 2 | 50.72 | 1 | F1:11905 | Fraction\_1\_23092022r.raw | 1.6171E5 |  |  |  |  |  |  | 1 | 1 | 0 | 0 | 0 | 0 | 0 | 0 | 41 | 56 |  |  | PEAKS DB |
| K.NSLISHYKVFKDM(+15.99)IALK.K | Y | 55.83 | 2022.0972 | 17 | -0.7 | 506.5312 | 4 | 47.88 | 2 | F2:10723 | Fraction\_2\_23092022.raw |  | 1.7238E6 |  |  |  |  |  | 2 | 0 | 2 | 0 | 0 | 0 | 0 | 0 | 460 | 476 | Oxidation (M) | M13:Oxidation (M):1000.00 | PEAKS DB |
| total 16 peptides |  |  |  |  |  |  |  |  |  |  |  |  |  |  |  |  |  |  |  |  |  |  |  |  |  |  |  |  |  |  |  |
| --- | --- | --- | --- | --- | --- | --- | --- | --- | --- | --- | --- | --- | --- | --- | --- | --- | --- | --- | --- | --- | --- | --- | --- | --- | --- | --- | --- | --- | --- | --- | --- |

TRINITY\_DN33420\_c0\_g1\_i1.p1

back to list

  

| Protein Coverage
| Supporting Peptides
|

Protein Coverage:

Supporting Peptides:

| Peptide | Uniq | -10lgP | Mass | Length | ppm | m/z | z | RT | Fraction | Scan | Source File | Area F1 | Area F2 | Area F3 | Area F4 | Area F5 | Area F6 | Area F7 | #Feature | #Feature F1 | #Feature F2 | #Feature F3 | #Feature F4 | #Feature F5 | #Feature F6 | #Feature F7 | Start | End | PTM | AScore | Found By |
| --- | --- | --- | --- | --- | --- | --- | --- | --- | --- | --- | --- | --- | --- | --- | --- | --- | --- | --- | --- | --- | --- | --- | --- | --- | --- | --- | --- | --- | --- | --- | --- |
| R.SIVHESYPGGVAPYDIALVELQTPLSLSK.E | Y | 133.98 | 3082.6226 | 29 | 1.2 | 1028.5493 | 3 | 82.39 | 5 | F5:26950 | Fraction\_5\_23092022.raw |  |  | 9.6481E7 | 4.2493E6 | 5.2887E6 | 1.4567E7 |  | 10 | 0 | 0 | 7 | 1 | 1 | 1 | 0 | 111 | 139 |  |  | PEAKS DB |
| R.IVGGENANRGEFPYQVSLQWGLTSSNTK.H | Y | 105.80 | 3051.5049 | 28 | 0.9 | 1018.1765 | 3 | 68.30 | 7 | F7:26144 | Fraction\_7\_23092022.raw |  |  | 7.4009E5 |  | 1.8087E6 | 1.5957E6 | 3.6201E6 | 4 | 0 | 0 | 1 | 0 | 1 | 1 | 1 | 31 | 58 |  |  | PEAKS DB |
| K.YDISYPEKGEQVIKVKR.S | Y | 90.36 | 2051.1050 | 17 | 0.0 | 513.7835 | 4 | 37.04 | 7 | F7:9380 | Fraction\_7\_23092022.raw |  |  | 4.002E5 |  |  | 2.7909E5 | 3.0374E6 | 4 | 0 | 0 | 1 | 0 | 0 | 1 | 2 | 94 | 110 |  |  | PEAKS DB |
| K.AGKYDISYPEKGEQVIK.V | Y | 89.98 | 1923.9941 | 17 | 2.3 | 482.0069 | 4 | 36.48 | 7 | F7:9116 | Fraction\_7\_23092022.raw | 3.5912E5 | 5.0562E5 | 3.0452E7 | 2.5865E6 | 7.4445E6 | 1.5414E7 | 8.8141E7 | 15 | 2 | 1 | 2 | 2 | 2 | 3 | 3 | 91 | 107 |  |  | PEAKS DB |
| K.YDISYPEKGEQVIK.V | Y | 87.06 | 1667.8406 | 14 | 0.4 | 556.9543 | 3 | 42.93 | 7 | F7:12070 | Fraction\_7\_23092022.raw |  |  | 7.2549E6 |  | 5.9338E5 | 1.2977E6 | 3.2744E6 | 8 | 0 | 0 | 2 | 0 | 2 | 2 | 2 | 94 | 107 |  |  | PEAKS DB |
| K.RSIVHESYPGGVAPYDIALVELQTPLSLSK.E | Y | 81.34 | 3238.7236 | 30 | 1.3 | 810.6892 | 4 | 77.53 | 3 | F3:24426 | Fraction\_3\_23092022.raw |  |  | 3.4215E6 |  | 1.069E5 |  |  | 3 | 0 | 0 | 2 | 0 | 1 | 0 | 0 | 110 | 139 |  |  | PEAKS DB |
| K.SNGKTELVGIVSWGIIPC(+57.02)GTR.G | Y | 77.36 | 2243.1731 | 21 | 0.6 | 748.7321 | 3 | 71.80 | 6 | F6:22841 | Fraction\_6\_23092022.raw |  |  | 2.9E5 | 9.9456E4 |  | 2.6381E6 | 3.5337E6 | 4 | 0 | 0 | 1 | 1 | 0 | 1 | 1 | 232 | 252 | Carbamidomethylation | C18:Carbamidomethylation:1000.00 | PEAKS DB |
| R.VSAFNNWILQK.I | Y | 69.07 | 1318.7034 | 11 | 0.8 | 660.3595 | 2 | 64.61 | 6 | F6:19607 | Fraction\_6\_23092022.raw | 3.9554E5 | 7.1007E4 | 1.7413E6 | 3.7565E5 | 2.0316E5 | 2.2757E7 | 5.5819E6 | 7 | 1 | 1 | 1 | 1 | 1 | 1 | 1 | 261 | 271 |  |  | PEAKS DB |
| R.VSAFNNWILQKISK | Y | 68.22 | 1646.9144 | 14 | -0.2 | 549.9786 | 3 | 65.45 | 6 | F6:20009 | Fraction\_6\_23092022.raw |  |  |  |  |  | 1.0245E6 | 2.1406E6 | 3 | 0 | 0 | 0 | 0 | 0 | 2 | 1 | 261 | 274 |  |  | PEAKS DB |
| K.AGKYDISYPEKGEQVIKVK.R | Y | 65.70 | 2151.1575 | 19 | 0.2 | 718.0599 | 3 | 36.97 | 7 | F7:9360 | Fraction\_7\_23092022.raw |  |  | 2.4969E6 | 1.3951E5 | 3.7482E5 | 7.19E5 | 1.0484E7 | 8 | 0 | 0 | 2 | 1 | 1 | 1 | 3 | 91 | 109 |  |  | PEAKS DB |
| K.TSVAEM(+15.99)PDILQK.A | Y | 64.76 | 1346.6752 | 12 | 0.3 | 674.3450 | 2 | 41.19 | 6 | F6:9079 | Fraction\_6\_23092022.raw |  |  | 5.1822E6 |  | 1.7533E5 | 2.5349E6 |  | 5 | 0 | 0 | 2 | 0 | 1 | 2 | 0 | 168 | 179 | Oxidation (M) | M6:Oxidation (M):1000.00 | PEAKS DB |
| R.GEFPYQVSLQWGLTSSNTK.H | Y | 60.42 | 2141.0430 | 19 | 0.5 | 1071.5293 | 2 | 76.68 | 5 | F5:24399 | Fraction\_5\_23092022.raw |  |  |  |  | 1.5659E5 |  |  | 1 | 0 | 0 | 0 | 0 | 1 | 0 | 0 | 40 | 58 |  |  | PEAKS DB |
| K.WIITAGHC(+57.02)INAVPSIGAFLIK.A | Y | 58.10 | 2280.2451 | 21 | 0.5 | 761.0894 | 3 | 81.61 | 6 | F6:27215 | Fraction\_6\_23092022.raw |  |  |  |  |  | 9.1369E5 | 4.6881E6 | 2 | 0 | 0 | 0 | 0 | 0 | 1 | 1 | 70 | 90 | Carbamidomethylation | C8:Carbamidomethylation:1000.00 | PEAKS DB |
| K.HFC(+57.02)GGSILNEK.W | Y | 57.39 | 1260.5920 | 11 | -0.2 | 421.2045 | 3 | 32.09 | 7 | F7:7219 | Fraction\_7\_23092022.raw |  |  |  |  |  |  | 8.5097E5 | 2 | 0 | 0 | 0 | 0 | 0 | 0 | 2 | 59 | 69 | Carbamidomethylation | C3:Carbamidomethylation:1000.00 | PEAKS DB |
| total 14 peptides |  |  |  |  |  |  |  |  |  |  |  |  |  |  |  |  |  |  |  |  |  |  |  |  |  |  |  |  |  |  |  |
| --- | --- | --- | --- | --- | --- | --- | --- | --- | --- | --- | --- | --- | --- | --- | --- | --- | --- | --- | --- | --- | --- | --- | --- | --- | --- | --- | --- | --- | --- | --- | --- |

TRINITY\_DN11986\_c0\_g1\_i3.p1

back to list

  

| Protein Coverage
| Supporting Peptides
|

Protein Coverage:

Supporting Peptides:

| Peptide | Uniq | -10lgP | Mass | Length | ppm | m/z | z | RT | Fraction | Scan | Source File | Area F1 | Area F2 | Area F3 | Area F4 | Area F5 | Area F6 | Area F7 | #Feature | #Feature F1 | #Feature F2 | #Feature F3 | #Feature F4 | #Feature F5 | #Feature F6 | #Feature F7 | Start | End | PTM | AScore | Found By |
| --- | --- | --- | --- | --- | --- | --- | --- | --- | --- | --- | --- | --- | --- | --- | --- | --- | --- | --- | --- | --- | --- | --- | --- | --- | --- | --- | --- | --- | --- | --- | --- |
| K.SYNPQLTSYDYDAPLTEAGDPTEKYFLIRDVISK.Y | Y | 108.25 | 3908.8994 | 34 | 1.2 | 978.2333 | 4 | 76.80 | 2 | F2:23726 | Fraction\_2\_23092022.raw | 1.0871E6 | 5.7549E6 |  |  |  |  |  | 2 | 1 | 1 | 0 | 0 | 0 | 0 | 0 | 328 | 361 |  |  | PEAKS DB |
| R.SYFHITDQPLDTYLDTTGWGK.G | Y | 98.61 | 2457.1489 | 21 | 0.3 | 820.0571 | 3 | 77.78 | 1 | F1:23980 | Fraction\_1\_23092022r.raw | 3.0562E5 |  |  |  |  |  |  | 1 | 1 | 0 | 0 | 0 | 0 | 0 | 0 | 548 | 568 |  |  | PEAKS DB |
| K.SYNPQLTSYDYDAPLTEAGDPTEKYFLIR.D | Y | 97.53 | 3366.5930 | 29 | 1.1 | 1123.2062 | 3 | 75.19 | 2 | F2:23001 | Fraction\_2\_23092022.raw |  | 1.2172E6 |  |  |  |  |  | 1 | 0 | 1 | 0 | 0 | 0 | 0 | 0 | 328 | 356 |  |  | PEAKS DB |
| R.ALIYVDSELVGTLSR.M | Y | 95.49 | 1634.8879 | 15 | 0.9 | 818.4520 | 2 | 74.52 | 4 | F4:23613 | Fraction\_4\_23092022.raw | 1.336E6 | 1.7629E6 | 7.0291E5 | 3.4769E5 |  | 3.8846E5 |  | 6 | 1 | 2 | 1 | 1 | 0 | 1 | 0 | 446 | 460 |  |  | PEAKS DB |
| R.TGQNLLEILELQYVPVTRK.M | Y | 95.15 | 2213.2419 | 19 | 0.3 | 738.7548 | 3 | 82.32 | 7 | F7:32804 | Fraction\_7\_23092022.raw | 3.5673E5 | 1.8611E6 |  | 1.6914E5 |  |  | 6.3833E5 | 4 | 1 | 1 | 0 | 1 | 0 | 0 | 1 | 600 | 618 |  |  | PEAKS DB |
| K.TTETSGILLNGPVFFR.S | Y | 87.55 | 1750.9253 | 16 | -0.1 | 876.4698 | 2 | 78.54 | 2 | F2:24484 | Fraction\_2\_23092022.raw | 6.1885E6 | 5.256E6 | 7.7536E5 | 6.3138E5 |  | 6.6E5 |  | 6 | 1 | 2 | 1 | 1 | 0 | 1 | 0 | 532 | 547 |  |  | PEAKS DB |
| K.IIGNKAVLYTTDGINDR.M | Y | 83.12 | 1861.9897 | 17 | 0.2 | 621.6707 | 3 | 44.86 | 2 | F2:9383 | Fraction\_2\_23092022.raw | 2.7184E5 | 6.3343E5 |  |  |  |  |  | 2 | 1 | 1 | 0 | 0 | 0 | 0 | 0 | 212 | 228 |  |  | PEAKS DB |
| K.SYNPQLTSYDYDAPLTEAGDPTEK.Y | Y | 77.76 | 2674.1921 | 24 | 0.6 | 1338.1041 | 2 | 61.19 | 2 | F2:16747 | Fraction\_2\_23092022.raw |  | 4.1997E5 |  |  |  |  |  | 1 | 0 | 1 | 0 | 0 | 0 | 0 | 0 | 328 | 351 |  |  | PEAKS DB |
| R.YVSGSFHYFR.T | Y | 69.80 | 1261.5880 | 10 | 1.3 | 421.5371 | 3 | 42.55 | 1 | F1:8279 | Fraction\_1\_23092022r.raw | 5.3828E5 | 1.3946E6 |  |  |  |  |  | 3 | 1 | 2 | 0 | 0 | 0 | 0 | 0 | 51 | 60 |  |  | PEAKS DB |
| K.GIAYVNGYNLGR.Y | Y | 67.77 | 1295.6622 | 12 | 0.2 | 648.8385 | 2 | 50.83 | 1 | F1:11932 | Fraction\_1\_23092022r.raw | 2.9059E5 |  |  |  |  |  |  | 1 | 1 | 0 | 0 | 0 | 0 | 0 | 0 | 569 | 580 |  |  | PEAKS DB |
| K.AVLYTTDGINDR.M | Y | 62.30 | 1336.6622 | 12 | -0.3 | 669.3382 | 2 | 43.07 | 1 | F1:8507 | Fraction\_1\_23092022r.raw | 6.9744E5 | 5.3173E5 |  |  |  |  |  | 2 | 1 | 1 | 0 | 0 | 0 | 0 | 0 | 217 | 228 |  |  | PEAKS DB |
| K.ILVENQGHLNYGNEIHDFK.G | Y | 60.38 | 2239.1021 | 19 | 0.3 | 560.7830 | 4 | 46.20 | 1 | F1:9880 | Fraction\_1\_23092022r.raw | 2.4826E5 |  |  |  |  |  |  | 1 | 1 | 0 | 0 | 0 | 0 | 0 | 0 | 479 | 497 |  |  | PEAKS DB |
| K.NIYSLPLLNPYGR.H | Y | 55.69 | 1518.8195 | 13 | 0.7 | 760.4175 | 2 | 77.54 | 6 | F6:25381 | Fraction\_6\_23092022.raw | 1.4989E6 | 2.3009E6 |  | 4.9542E5 |  | 6.1894E5 |  | 4 | 1 | 1 | 0 | 1 | 0 | 1 | 0 | 463 | 475 |  |  | PEAKS DB |
| total 13 peptides |  |  |  |  |  |  |  |  |  |  |  |  |  |  |  |  |  |  |  |  |  |  |  |  |  |  |  |  |  |  |  |
| --- | --- | --- | --- | --- | --- | --- | --- | --- | --- | --- | --- | --- | --- | --- | --- | --- | --- | --- | --- | --- | --- | --- | --- | --- | --- | --- | --- | --- | --- | --- | --- |

C0HLL3|PA1\_VESVE

back to list

  

| Protein Coverage
| Supporting Peptides
|

Protein Coverage:

Supporting Peptides:

| Peptide | Uniq | -10lgP | Mass | Length | ppm | m/z | z | RT | Fraction | Scan | Source File | Area F1 | Area F2 | Area F3 | Area F4 | Area F5 | Area F6 | Area F7 | #Feature | #Feature F1 | #Feature F2 | #Feature F3 | #Feature F4 | #Feature F5 | #Feature F6 | #Feature F7 | Start | End | PTM | AScore | Found By |
| --- | --- | --- | --- | --- | --- | --- | --- | --- | --- | --- | --- | --- | --- | --- | --- | --- | --- | --- | --- | --- | --- | --- | --- | --- | --- | --- | --- | --- | --- | --- | --- |
| R.IC(+57.02)ETDAHYVQIIHTSNNLGTER.T | N | 114.42 | 2570.2183 | 22 | 0.2 | 857.7468 | 3 | 44.41 | 5 | F5:9876 | Fraction\_5\_23092022.raw |  | 5.1745E5 | 2.2932E7 | 1.5069E7 | 4.1552E7 | 1.5463E7 | 4.742E6 | 12 | 0 | 1 | 3 | 2 | 2 | 2 | 2 | 183 | 204 | Carbamidomethylation | C2:Carbamidomethylation:1000.00 | PEAKS DB |
| R.LIGHSLGAQIAGFAGKEYQK.F | N | 111.41 | 2087.1162 | 20 | 0.0 | 696.7126 | 3 | 49.84 | 5 | F5:12302 | Fraction\_5\_23092022.raw |  |  | 3.7265E6 | 5.2349E6 | 7.6906E6 | 9.6072E6 | 2.4268E6 | 10 | 0 | 0 | 2 | 2 | 2 | 2 | 2 | 136 | 155 |  |  | PEAKS DB |
| R.LIGHSLGAQIAGFAGK.E | N | 105.57 | 1538.8568 | 16 | -0.6 | 513.9592 | 3 | 52.10 | 5 | F5:13308 | Fraction\_5\_23092022.raw |  |  | 9.856E6 | 7.8605E6 | 1.4847E7 | 1.2515E7 | 2.5951E6 | 9 | 0 | 0 | 2 | 2 | 2 | 2 | 1 | 136 | 151 |  |  | PEAKS DB |
| K.TGSFYVPVESKAPYC(+57.02)NNK.G | N | 97.81 | 2059.9673 | 18 | 0.0 | 1030.9910 | 2 | 45.28 | 3 | F3:9956 | Fraction\_3\_23092022.raw |  |  | 3.5089E8 | 1.0426E8 |  | 1.0646E6 |  | 12 | 0 | 0 | 7 | 4 | 0 | 1 | 0 | 283 | 300 | Carbamidomethylation | C15:Carbamidomethylation:1000.00 | PEAKS DB |
| K.TGSFYVPVESKAPYC(+57.02)NNKGK.K | N | 88.43 | 2245.0837 | 20 | -0.5 | 749.3682 | 3 | 38.45 | 4 | F4:7505 | Fraction\_4\_23092022.raw |  | 2.2402E6 | 9.7062E6 | 4.9178E7 | 2.0417E6 | 5.5689E6 | 1.0137E7 | 15 | 0 | 1 | 3 | 4 | 2 | 2 | 3 | 283 | 302 | Carbamidomethylation | C15:Carbamidomethylation:1000.00 | PEAKS DB |
| R.HEC(+57.02)C(+57.02)LIGVPQSKNPQPVSK.C | N | 88.10 | 2177.0720 | 19 | -0.3 | 726.6978 | 3 | 31.57 | 7 | F7:6948 | Fraction\_7\_23092022.raw |  |  | 2.8577E5 | 1.4782E6 | 1.272E6 | 5.2592E5 | 6.9825E5 | 7 | 0 | 0 | 1 | 1 | 2 | 1 | 2 | 246 | 264 | Carbamidomethylation | C3:Carbamidomethylation:1000.00;C4:Carbamidomethylation:1000.00 | PEAKS DB |
| K.TGSFYVPVESK.A | N | 86.10 | 1212.6027 | 11 | 1.0 | 607.3092 | 2 | 47.23 | 4 | F4:11441 | Fraction\_4\_23092022.raw | 1.7405E6 | 3.3208E6 | 1.4279E8 | 1.6292E8 | 3.0697E6 | 1.7065E7 | 5.4182E6 | 10 | 1 | 1 | 2 | 2 | 1 | 2 | 1 | 283 | 293 |  |  | PEAKS DB |
| R.HEC(+57.02)C(+57.02)LIGVPQSK.N | N | 66.41 | 1426.6697 | 12 | 0.1 | 714.3422 | 2 | 33.72 | 7 | F7:7919 | Fraction\_7\_23092022.raw |  |  |  |  |  |  | 1.5167E5 | 1 | 0 | 0 | 0 | 0 | 0 | 0 | 1 | 246 | 257 | Carbamidomethylation | C3:Carbamidomethylation:1000.00;C4:Carbamidomethylation:1000.00 | PEAKS DB |
| K.C(+57.02)TRNEC(+57.02)VC(+57.02)VGLNAK.R | N | 62.11 | 1679.7542 | 14 | 0.2 | 560.9254 | 3 | 30.48 | 7 | F7:6463 | Fraction\_7\_23092022.raw |  |  |  |  |  |  | 1.1536E6 | 2 | 0 | 0 | 0 | 0 | 0 | 0 | 2 | 265 | 278 | Carbamidomethylation | C1:Carbamidomethylation:1000.00;C6:Carbamidomethylation:1000.00;C8:Carbamidomethylation:1000.00 | PEAKS DB |
| R.NEC(+57.02)VC(+57.02)VGLNAK.R | N | 61.26 | 1262.5747 | 11 | 0.2 | 632.2947 | 2 | 34.83 | 7 | F7:8388 | Fraction\_7\_23092022.raw |  |  | 3.1121E6 | 3.1889E6 | 7.1825E5 |  | 3.0319E6 | 10 | 0 | 0 | 2 | 4 | 2 | 0 | 2 | 268 | 278 | Carbamidomethylation | C3:Carbamidomethylation:1000.00;C5:Carbamidomethylation:1000.00 | PEAKS DB |
| R.AVQYFTEC(+57.02)IRHEC(+57.02)C(+57.02)LIGVPQSKNPQPVSK.C | Y | 59.81 | 3444.6741 | 29 | 9.4 | 689.9485 | 5 | 44.51 | 5 | F5:9955 | Fraction\_5\_23092022.raw |  |  |  |  | 7.6272E5 |  | 7.6637E5 | 2 | 0 | 0 | 0 | 0 | 1 | 0 | 1 | 236 | 264 | Carbamidomethylation | C8:Carbamidomethylation:1000.00;C13:Carbamidomethylation:1000.00;C14:Carbamidomethylation:1000.00 | PEAKS DB |
| total 11 peptides |  |  |  |  |  |  |  |  |  |  |  |  |  |  |  |  |  |  |  |  |  |  |  |  |  |  |  |  |  |  |  |
| --- | --- | --- | --- | --- | --- | --- | --- | --- | --- | --- | --- | --- | --- | --- | --- | --- | --- | --- | --- | --- | --- | --- | --- | --- | --- | --- | --- | --- | --- | --- | --- |

P48668|K2C6C\_HUMAN

back to list

  

| Protein Coverage
| Supporting Peptides
|

Protein Coverage:

Supporting Peptides:

| Peptide | Uniq | -10lgP | Mass | Length | ppm | m/z | z | RT | Fraction | Scan | Source File | Area F1 | Area F2 | Area F3 | Area F4 | Area F5 | Area F6 | Area F7 | #Feature | #Feature F1 | #Feature F2 | #Feature F3 | #Feature F4 | #Feature F5 | #Feature F6 | #Feature F7 | Start | End | PTM | AScore | Found By |
| --- | --- | --- | --- | --- | --- | --- | --- | --- | --- | --- | --- | --- | --- | --- | --- | --- | --- | --- | --- | --- | --- | --- | --- | --- | --- | --- | --- | --- | --- | --- | --- |
| R.AIGGGLSSVGGGSSTIKYTTTSSSSR.K | Y | 109.90 | 2417.2034 | 26 | 0.7 | 806.7423 | 3 | 41.60 | 5 | F5:8647 | Fraction\_5\_23092022.raw | 4.9517E4 | 1.4777E5 |  |  | 4.6006E5 | 6.9356E4 |  | 4 | 1 | 1 | 0 | 0 | 1 | 1 | 0 | 534 | 559 |  |  | PEAKS DB |
| R.ISIGGGSC(+57.02)AISGGYGSR.A | N | 89.23 | 1597.7518 | 17 | 0.1 | 799.8833 | 2 | 45.05 | 2 | F2:9467 | Fraction\_2\_23092022.raw | 3.4544E5 | 3.97E5 | 2.1039E5 |  |  | 3.439E5 | 1.3764E5 | 5 | 1 | 1 | 1 | 0 | 0 | 1 | 1 | 70 | 86 | Carbamidomethylation | C8:Carbamidomethylation:1000.00 | PEAKS DB |
| R.AIGGGLSSVGGGSSTIK.Y | Y | 74.89 | 1446.7678 | 17 | 0.2 | 724.3914 | 2 | 40.87 | 6 | F6:8999 | Fraction\_6\_23092022.raw |  | 8.1429E4 |  |  | 1.5433E5 | 1.3125E5 |  | 3 | 0 | 1 | 0 | 0 | 1 | 1 | 0 | 534 | 550 |  |  | PEAKS DB |
| K.VLDTKWTLLQEQGTK.T | N | 72.29 | 1758.9515 | 15 | 0.5 | 587.3248 | 3 | 54.32 | 1 | F1:13486 | Fraction\_1\_23092022r.raw | 2.7823E5 |  |  |  |  |  |  | 1 | 1 | 0 | 0 | 0 | 0 | 0 | 0 | 190 | 204 |  |  | PEAKS DB |
| K.TLNNKFASFIDKVR.F | N | 71.43 | 1651.9045 | 14 | -0.2 | 551.6420 | 3 | 51.36 | 2 | F2:12305 | Fraction\_2\_23092022.raw | 2.671E5 | 1.8567E6 | 2.1296E5 |  |  | 8.2338E5 | 3.789E5 | 6 | 1 | 2 | 1 | 0 | 0 | 1 | 1 | 169 | 182 |  |  | PEAKS DB |
| R.NLDLDSIIAEVK.A | N | 67.73 | 1328.7188 | 12 | -0.3 | 665.3665 | 2 | 80.82 | 4 | F4:26420 | Fraction\_4\_23092022.raw | 1.3444E7 | 1.1783E7 | 2.3503E7 | 6.3504E6 | 3.9084E6 | 1.1367E7 | 9.0701E6 | 8 | 1 | 1 | 2 | 1 | 1 | 1 | 1 | 327 | 338 |  |  | PEAKS DB |
| K.ADTLTDEINFLR.A | N | 65.61 | 1406.7041 | 12 | 0.4 | 704.3596 | 2 | 72.52 | 2 | F2:21808 | Fraction\_2\_23092022.raw | 7.689E5 | 6.8039E5 | 1.0794E6 | 1.5412E5 |  | 2.6106E5 |  | 5 | 1 | 1 | 1 | 1 | 0 | 1 | 0 | 288 | 299 |  |  | PEAKS DB |
| R.SGFSSISVSR.S | N | 64.90 | 1025.5142 | 10 | -0.5 | 513.7641 | 2 | 39.10 | 6 | F6:8160 | Fraction\_6\_23092022.raw |  |  |  |  | 4.542E4 | 1.3482E5 |  | 2 | 0 | 0 | 0 | 0 | 1 | 1 | 0 | 31 | 40 |  |  | PEAKS DB |
| K.FASFIDKVR.F | N | 64.36 | 1081.5920 | 9 | 1.5 | 361.5385 | 3 | 44.57 | 4 | F4:10216 | Fraction\_4\_23092022.raw | 6.0874E6 | 1.3079E6 | 2.6129E6 | 3.499E5 | 3.5976E5 | 1.1286E6 |  | 11 | 1 | 2 | 2 | 2 | 2 | 2 | 0 | 174 | 182 |  |  | PEAKS DB |
| K.LALDVEIATYR.K | N | 62.33 | 1262.6870 | 11 | 0.5 | 632.3511 | 2 | 64.39 | 3 | F3:18523 | Fraction\_3\_23092022.raw | 1.5189E6 | 6.2742E5 | 1.3952E6 | 2.3872E5 | 2.1606E5 | 3.32E5 |  | 6 | 1 | 1 | 1 | 1 | 1 | 1 | 0 | 456 | 466 |  |  | PEAKS DB |
| R.AIGGGLSSVGGGSSTIKYTTTSSSSRK.S | Y | 60.69 | 2545.2983 | 27 | 0.1 | 637.3319 | 4 | 35.96 | 5 | F5:6237 | Fraction\_5\_23092022.raw |  |  |  |  | 1.226E5 |  |  | 1 | 0 | 0 | 0 | 0 | 1 | 0 | 0 | 534 | 560 |  |  | PEAKS DB |
| R.TAAENEFVTLK.K | N | 60.47 | 1221.6240 | 11 | 0.3 | 611.8195 | 2 | 46.65 | 3 | F3:10589 | Fraction\_3\_23092022.raw |  |  | 1.8006E5 |  |  |  |  | 1 | 0 | 0 | 1 | 0 | 0 | 0 | 0 | 261 | 271 |  |  | PEAKS DB |
| K.WTLLQEQGTK.T | N | 59.30 | 1202.6295 | 10 | -0.2 | 602.3219 | 2 | 51.37 | 2 | F2:12309 | Fraction\_2\_23092022.raw |  | 1.3976E5 |  |  |  |  |  | 1 | 0 | 1 | 0 | 0 | 0 | 0 | 0 | 195 | 204 |  |  | PEAKS DB |
| K.LEGLEDALQK.A | N | 56.03 | 1114.5869 | 10 | 0.4 | 558.3010 | 2 | 49.41 | 3 | F3:11827 | Fraction\_3\_23092022.raw |  |  | 4.0269E5 |  |  |  |  | 1 | 0 | 0 | 1 | 0 | 0 | 0 | 0 | 427 | 436 |  |  | PEAKS DB |
| K.LALDVEIATYRK.L | N | 51.28 | 1390.7820 | 12 | 0.0 | 464.6013 | 3 | 53.42 | 1 | F1:13083 | Fraction\_1\_23092022r.raw | 4.7232E5 |  |  |  |  |  |  | 1 | 1 | 0 | 0 | 0 | 0 | 0 | 0 | 456 | 467 |  |  | PEAKS DB |
| R.QNLEPLFEQYINNLRR.Q | N | 48.69 | 2046.0646 | 16 | 0.4 | 683.0291 | 3 | 77.65 | 1 | F1:23976 | Fraction\_1\_23092022r.raw | 2.1213E6 |  |  |  |  |  |  | 1 | 1 | 0 | 0 | 0 | 0 | 0 | 0 | 208 | 223 |  |  | PEAKS DB |
| total 16 peptides |  |  |  |  |  |  |  |  |  |  |  |  |  |  |  |  |  |  |  |  |  |  |  |  |  |  |  |  |  |  |  |
| --- | --- | --- | --- | --- | --- | --- | --- | --- | --- | --- | --- | --- | --- | --- | --- | --- | --- | --- | --- | --- | --- | --- | --- | --- | --- | --- | --- | --- | --- | --- | --- |

TRINITY\_DN2611\_c0\_g1\_i12.p1

back to list

  

| Protein Coverage
| Supporting Peptides
|

Protein Coverage:

Supporting Peptides:

| Peptide | Uniq | -10lgP | Mass | Length | ppm | m/z | z | RT | Fraction | Scan | Source File | Area F1 | Area F2 | Area F3 | Area F4 | Area F5 | Area F6 | Area F7 | #Feature | #Feature F1 | #Feature F2 | #Feature F3 | #Feature F4 | #Feature F5 | #Feature F6 | #Feature F7 | Start | End | PTM | AScore | Found By |
| --- | --- | --- | --- | --- | --- | --- | --- | --- | --- | --- | --- | --- | --- | --- | --- | --- | --- | --- | --- | --- | --- | --- | --- | --- | --- | --- | --- | --- | --- | --- | --- |
| K.ELGLQYPGGYKVEDLYEDVNYGVLLPHTK.I | Y | 95.28 | 3308.6604 | 29 | 0.9 | 828.1732 | 4 | 78.63 | 2 | F2:24536 | Fraction\_2\_23092022.raw |  | 5.2189E6 |  |  |  |  |  | 2 | 0 | 2 | 0 | 0 | 0 | 0 | 0 | 365 | 393 |  |  | PEAKS DB |
| K.IIAVDQDPLGIQGR.R | Y | 88.70 | 1493.8202 | 14 | 1.2 | 747.9183 | 2 | 59.22 | 2 | F2:15828 | Fraction\_2\_23092022.raw | 1.0165E6 | 6.4892E7 | 2.4161E5 | 2.8049E5 | 8.3802E5 | 1.3147E7 |  | 6 | 1 | 1 | 1 | 1 | 1 | 1 | 0 | 305 | 318 |  |  | PEAKS DB |
| R.C(+57.02)NTDC(+57.02)KNDPDNC(+57.02)ISDQLFR.T | Y | 85.42 | 2370.9626 | 19 | -1.1 | 791.3273 | 3 | 49.58 | 2 | F2:11492 | Fraction\_2\_23092022.raw |  | 4.6319E6 |  |  |  |  |  | 1 | 0 | 1 | 0 | 0 | 0 | 0 | 0 | 41 | 59 | Carbamidomethylation | C1:Carbamidomethylation:1000.00;C5:Carbamidomethylation:1000.00;C12:Carbamidomethylation:1000.00 | PEAKS DB |
| K.VEDLYEDVNYGVLLPHTK.I | Y | 83.40 | 2103.0525 | 18 | 0.3 | 702.0250 | 3 | 68.15 | 2 | F2:19876 | Fraction\_2\_23092022.raw |  | 3.0315E5 |  |  |  | 1.5647E5 |  | 2 | 0 | 1 | 0 | 0 | 0 | 1 | 0 | 376 | 393 |  |  | PEAKS DB |
| K.KIIAVDQDPLGIQGR.R | Y | 81.39 | 1621.9150 | 15 | 0.5 | 541.6459 | 3 | 49.67 | 5 | F5:12253 | Fraction\_5\_23092022.raw | 2.7777E5 | 1.4111E7 |  |  | 1.3766E5 | 2.7292E6 |  | 6 | 1 | 2 | 0 | 0 | 1 | 2 | 0 | 304 | 318 |  |  | PEAKS DB |
| R.TDGTPSDVSVTLKELGLQYPGGYK.V | Y | 76.14 | 2524.2695 | 24 | 0.6 | 842.4310 | 3 | 73.36 | 2 | F2:22192 | Fraction\_2\_23092022.raw |  | 2.8914E5 |  |  |  |  |  | 1 | 0 | 1 | 0 | 0 | 0 | 0 | 0 | 352 | 375 |  |  | PEAKS DB |
| K.IKVKVNPSGVVILR.C | Y | 63.94 | 1520.9766 | 14 | 0.5 | 507.9997 | 3 | 47.25 | 6 | F6:11791 | Fraction\_6\_23092022.raw |  | 3.9089E6 |  |  |  | 1.0616E6 |  | 2 | 0 | 1 | 0 | 0 | 0 | 1 | 0 | 394 | 407 |  |  | PEAKS DB |
| K.AILQNKKIIAVDQDPLGIQGR.R | Y | 63.34 | 2289.3169 | 21 | -0.3 | 573.3363 | 4 | 51.34 | 2 | F2:12260 | Fraction\_2\_23092022.raw |  | 2.8591E6 |  |  |  |  |  | 1 | 0 | 1 | 0 | 0 | 0 | 0 | 0 | 298 | 318 |  |  | PEAKS DB |
| R.FRC(+57.02)NTDC(+57.02)KNDPDNC(+57.02)ISDQLFR.T | Y | 61.62 | 2674.1323 | 21 | 0.8 | 669.5409 | 4 | 46.02 | 2 | F2:9891 | Fraction\_2\_23092022.raw |  | 1.0062E6 |  |  |  |  |  | 1 | 0 | 1 | 0 | 0 | 0 | 0 | 0 | 39 | 59 | Carbamidomethylation | C3:Carbamidomethylation:1000.00;C7:Carbamidomethylation:1000.00;C14:Carbamidomethylation:1000.00 | PEAKS DB |
| K.KIIAVDQDPLGIQGRR.I | Y | 61.34 | 1778.0162 | 16 | -0.2 | 445.5113 | 4 | 43.25 | 2 | F2:8662 | Fraction\_2\_23092022.raw |  | 2.7076E6 |  |  |  |  |  | 1 | 0 | 1 | 0 | 0 | 0 | 0 | 0 | 304 | 319 |  |  | PEAKS DB |
| K.VNPSGVVILR.C | Y | 57.58 | 1052.6342 | 10 | -0.5 | 527.3241 | 2 | 48.05 | 2 | F2:10800 | Fraction\_2\_23092022.raw | 1.3489E5 | 2.0299E6 |  |  |  | 7.2177E5 |  | 3 | 1 | 1 | 0 | 0 | 0 | 1 | 0 | 398 | 407 |  |  | PEAKS DB |
| K.VKVNPSGVVILR.C | Y | 57.54 | 1279.7976 | 12 | 0.8 | 427.6068 | 3 | 43.60 | 2 | F2:8807 | Fraction\_2\_23092022.raw |  | 1.1973E6 |  |  |  | 4.1132E5 |  | 2 | 0 | 1 | 0 | 0 | 0 | 1 | 0 | 396 | 407 |  |  | PEAKS DB |
| K.NLADYIHSKGLK.F | Y | 56.96 | 1357.7354 | 12 | 0.0 | 453.5857 | 3 | 42.00 | 2 | F2:8063 | Fraction\_2\_23092022.raw |  | 2.7559E6 |  |  |  |  |  | 3 | 0 | 3 | 0 | 0 | 0 | 0 | 0 | 108 | 119 |  |  | PEAKS DB |
| K.AILQNKKIIAVDQDPLGIQGRR.I | Y | 47.47 | 2445.4180 | 22 | 0.4 | 612.3620 | 4 | 46.39 | 2 | F2:10087 | Fraction\_2\_23092022.raw |  | 1.0483E5 |  |  |  |  |  | 1 | 0 | 1 | 0 | 0 | 0 | 0 | 0 | 298 | 319 |  |  | PEAKS DB |
| total 14 peptides |  |  |  |  |  |  |  |  |  |  |  |  |  |  |  |  |  |  |  |  |  |  |  |  |  |  |  |  |  |  |  |
| --- | --- | --- | --- | --- | --- | --- | --- | --- | --- | --- | --- | --- | --- | --- | --- | --- | --- | --- | --- | --- | --- | --- | --- | --- | --- | --- | --- | --- | --- | --- | --- |

TRINITY\_DN2611\_c0\_g1\_i23.p1

back to list

  

| Protein Coverage
| Supporting Peptides
|

Protein Coverage:

Supporting Peptides:

| Peptide | Uniq | -10lgP | Mass | Length | ppm | m/z | z | RT | Fraction | Scan | Source File | Area F1 | Area F2 | Area F3 | Area F4 | Area F5 | Area F6 | Area F7 | #Feature | #Feature F1 | #Feature F2 | #Feature F3 | #Feature F4 | #Feature F5 | #Feature F6 | #Feature F7 | Start | End | PTM | AScore | Found By |
| --- | --- | --- | --- | --- | --- | --- | --- | --- | --- | --- | --- | --- | --- | --- | --- | --- | --- | --- | --- | --- | --- | --- | --- | --- | --- | --- | --- | --- | --- | --- | --- |
| K.ELGLQYPGGYKVEDLYEDVNYGVLLPHTK.I | Y | 95.28 | 3308.6604 | 29 | 0.9 | 828.1732 | 4 | 78.63 | 2 | F2:24536 | Fraction\_2\_23092022.raw |  | 5.2189E6 |  |  |  |  |  | 2 | 0 | 2 | 0 | 0 | 0 | 0 | 0 | 365 | 393 |  |  | PEAKS DB |
| K.IIAVDQDPLGIQGR.R | Y | 88.70 | 1493.8202 | 14 | 1.2 | 747.9183 | 2 | 59.22 | 2 | F2:15828 | Fraction\_2\_23092022.raw | 1.0165E6 | 6.4892E7 | 2.4161E5 | 2.8049E5 | 8.3802E5 | 1.3147E7 |  | 6 | 1 | 1 | 1 | 1 | 1 | 1 | 0 | 305 | 318 |  |  | PEAKS DB |
| R.C(+57.02)NTDC(+57.02)KNDPDNC(+57.02)ISDQLFR.T | Y | 85.42 | 2370.9626 | 19 | -1.1 | 791.3273 | 3 | 49.58 | 2 | F2:11492 | Fraction\_2\_23092022.raw |  | 4.6319E6 |  |  |  |  |  | 1 | 0 | 1 | 0 | 0 | 0 | 0 | 0 | 41 | 59 | Carbamidomethylation | C1:Carbamidomethylation:1000.00;C5:Carbamidomethylation:1000.00;C12:Carbamidomethylation:1000.00 | PEAKS DB |
| K.VEDLYEDVNYGVLLPHTK.I | Y | 83.40 | 2103.0525 | 18 | 0.3 | 702.0250 | 3 | 68.15 | 2 | F2:19876 | Fraction\_2\_23092022.raw |  | 3.0315E5 |  |  |  | 1.5647E5 |  | 2 | 0 | 1 | 0 | 0 | 0 | 1 | 0 | 376 | 393 |  |  | PEAKS DB |
| K.KIIAVDQDPLGIQGR.R | Y | 81.39 | 1621.9150 | 15 | 0.5 | 541.6459 | 3 | 49.67 | 5 | F5:12253 | Fraction\_5\_23092022.raw | 2.7777E5 | 1.4111E7 |  |  | 1.3766E5 | 2.7292E6 |  | 6 | 1 | 2 | 0 | 0 | 1 | 2 | 0 | 304 | 318 |  |  | PEAKS DB |
| R.TDGTPSDVSVTLKELGLQYPGGYK.V | Y | 76.14 | 2524.2695 | 24 | 0.6 | 842.4310 | 3 | 73.36 | 2 | F2:22192 | Fraction\_2\_23092022.raw |  | 2.8914E5 |  |  |  |  |  | 1 | 0 | 1 | 0 | 0 | 0 | 0 | 0 | 352 | 375 |  |  | PEAKS DB |
| K.IKVKVNPSGVVILR.C | Y | 63.94 | 1520.9766 | 14 | 0.5 | 507.9997 | 3 | 47.25 | 6 | F6:11791 | Fraction\_6\_23092022.raw |  | 3.9089E6 |  |  |  | 1.0616E6 |  | 2 | 0 | 1 | 0 | 0 | 0 | 1 | 0 | 394 | 407 |  |  | PEAKS DB |
| K.AILQNKKIIAVDQDPLGIQGR.R | Y | 63.34 | 2289.3169 | 21 | -0.3 | 573.3363 | 4 | 51.34 | 2 | F2:12260 | Fraction\_2\_23092022.raw |  | 2.8591E6 |  |  |  |  |  | 1 | 0 | 1 | 0 | 0 | 0 | 0 | 0 | 298 | 318 |  |  | PEAKS DB |
| R.FRC(+57.02)NTDC(+57.02)KNDPDNC(+57.02)ISDQLFR.T | Y | 61.62 | 2674.1323 | 21 | 0.8 | 669.5409 | 4 | 46.02 | 2 | F2:9891 | Fraction\_2\_23092022.raw |  | 1.0062E6 |  |  |  |  |  | 1 | 0 | 1 | 0 | 0 | 0 | 0 | 0 | 39 | 59 | Carbamidomethylation | C3:Carbamidomethylation:1000.00;C7:Carbamidomethylation:1000.00;C14:Carbamidomethylation:1000.00 | PEAKS DB |
| K.KIIAVDQDPLGIQGRR.I | Y | 61.34 | 1778.0162 | 16 | -0.2 | 445.5113 | 4 | 43.25 | 2 | F2:8662 | Fraction\_2\_23092022.raw |  | 2.7076E6 |  |  |  |  |  | 1 | 0 | 1 | 0 | 0 | 0 | 0 | 0 | 304 | 319 |  |  | PEAKS DB |
| K.VNPSGVVILR.C | Y | 57.58 | 1052.6342 | 10 | -0.5 | 527.3241 | 2 | 48.05 | 2 | F2:10800 | Fraction\_2\_23092022.raw | 1.3489E5 | 2.0299E6 |  |  |  | 7.2177E5 |  | 3 | 1 | 1 | 0 | 0 | 0 | 1 | 0 | 398 | 407 |  |  | PEAKS DB |
| K.VKVNPSGVVILR.C | Y | 57.54 | 1279.7976 | 12 | 0.8 | 427.6068 | 3 | 43.60 | 2 | F2:8807 | Fraction\_2\_23092022.raw |  | 1.1973E6 |  |  |  | 4.1132E5 |  | 2 | 0 | 1 | 0 | 0 | 0 | 1 | 0 | 396 | 407 |  |  | PEAKS DB |
| K.NLADYIHSKGLK.F | Y | 56.96 | 1357.7354 | 12 | 0.0 | 453.5857 | 3 | 42.00 | 2 | F2:8063 | Fraction\_2\_23092022.raw |  | 2.7559E6 |  |  |  |  |  | 3 | 0 | 3 | 0 | 0 | 0 | 0 | 0 | 108 | 119 |  |  | PEAKS DB |
| K.AILQNKKIIAVDQDPLGIQGRR.I | Y | 47.47 | 2445.4180 | 22 | 0.4 | 612.3620 | 4 | 46.39 | 2 | F2:10087 | Fraction\_2\_23092022.raw |  | 1.0483E5 |  |  |  |  |  | 1 | 0 | 1 | 0 | 0 | 0 | 0 | 0 | 298 | 319 |  |  | PEAKS DB |
| total 14 peptides |  |  |  |  |  |  |  |  |  |  |  |  |  |  |  |  |  |  |  |  |  |  |  |  |  |  |  |  |  |  |  |
| --- | --- | --- | --- | --- | --- | --- | --- | --- | --- | --- | --- | --- | --- | --- | --- | --- | --- | --- | --- | --- | --- | --- | --- | --- | --- | --- | --- | --- | --- | --- | --- |

TRINITY\_DN2611\_c0\_g1\_i19.p1

back to list

  

| Protein Coverage
| Supporting Peptides
|

Protein Coverage:

Supporting Peptides:

| Peptide | Uniq | -10lgP | Mass | Length | ppm | m/z | z | RT | Fraction | Scan | Source File | Area F1 | Area F2 | Area F3 | Area F4 | Area F5 | Area F6 | Area F7 | #Feature | #Feature F1 | #Feature F2 | #Feature F3 | #Feature F4 | #Feature F5 | #Feature F6 | #Feature F7 | Start | End | PTM | AScore | Found By |
| --- | --- | --- | --- | --- | --- | --- | --- | --- | --- | --- | --- | --- | --- | --- | --- | --- | --- | --- | --- | --- | --- | --- | --- | --- | --- | --- | --- | --- | --- | --- | --- |
| K.ELGLQYPGGYKVEDLYEDVNYGVLLPHTK.I | Y | 95.28 | 3308.6604 | 29 | 0.9 | 828.1732 | 4 | 78.63 | 2 | F2:24536 | Fraction\_2\_23092022.raw |  | 5.2189E6 |  |  |  |  |  | 2 | 0 | 2 | 0 | 0 | 0 | 0 | 0 | 365 | 393 |  |  | PEAKS DB |
| K.IIAVDQDPLGIQGR.R | Y | 88.70 | 1493.8202 | 14 | 1.2 | 747.9183 | 2 | 59.22 | 2 | F2:15828 | Fraction\_2\_23092022.raw | 1.0165E6 | 6.4892E7 | 2.4161E5 | 2.8049E5 | 8.3802E5 | 1.3147E7 |  | 6 | 1 | 1 | 1 | 1 | 1 | 1 | 0 | 305 | 318 |  |  | PEAKS DB |
| R.C(+57.02)NTDC(+57.02)KNDPDNC(+57.02)ISDQLFR.T | Y | 85.42 | 2370.9626 | 19 | -1.1 | 791.3273 | 3 | 49.58 | 2 | F2:11492 | Fraction\_2\_23092022.raw |  | 4.6319E6 |  |  |  |  |  | 1 | 0 | 1 | 0 | 0 | 0 | 0 | 0 | 41 | 59 | Carbamidomethylation | C1:Carbamidomethylation:1000.00;C5:Carbamidomethylation:1000.00;C12:Carbamidomethylation:1000.00 | PEAKS DB |
| K.VEDLYEDVNYGVLLPHTK.I | Y | 83.40 | 2103.0525 | 18 | 0.3 | 702.0250 | 3 | 68.15 | 2 | F2:19876 | Fraction\_2\_23092022.raw |  | 3.0315E5 |  |  |  | 1.5647E5 |  | 2 | 0 | 1 | 0 | 0 | 0 | 1 | 0 | 376 | 393 |  |  | PEAKS DB |
| K.KIIAVDQDPLGIQGR.R | Y | 81.39 | 1621.9150 | 15 | 0.5 | 541.6459 | 3 | 49.67 | 5 | F5:12253 | Fraction\_5\_23092022.raw | 2.7777E5 | 1.4111E7 |  |  | 1.3766E5 | 2.7292E6 |  | 6 | 1 | 2 | 0 | 0 | 1 | 2 | 0 | 304 | 318 |  |  | PEAKS DB |
| R.TDGTPSDVSVTLKELGLQYPGGYK.V | Y | 76.14 | 2524.2695 | 24 | 0.6 | 842.4310 | 3 | 73.36 | 2 | F2:22192 | Fraction\_2\_23092022.raw |  | 2.8914E5 |  |  |  |  |  | 1 | 0 | 1 | 0 | 0 | 0 | 0 | 0 | 352 | 375 |  |  | PEAKS DB |
| K.IKVKVNPSGVVILR.C | Y | 63.94 | 1520.9766 | 14 | 0.5 | 507.9997 | 3 | 47.25 | 6 | F6:11791 | Fraction\_6\_23092022.raw |  | 3.9089E6 |  |  |  | 1.0616E6 |  | 2 | 0 | 1 | 0 | 0 | 0 | 1 | 0 | 394 | 407 |  |  | PEAKS DB |
| K.AILQNKKIIAVDQDPLGIQGR.R | Y | 63.34 | 2289.3169 | 21 | -0.3 | 573.3363 | 4 | 51.34 | 2 | F2:12260 | Fraction\_2\_23092022.raw |  | 2.8591E6 |  |  |  |  |  | 1 | 0 | 1 | 0 | 0 | 0 | 0 | 0 | 298 | 318 |  |  | PEAKS DB |
| R.FRC(+57.02)NTDC(+57.02)KNDPDNC(+57.02)ISDQLFR.T | Y | 61.62 | 2674.1323 | 21 | 0.8 | 669.5409 | 4 | 46.02 | 2 | F2:9891 | Fraction\_2\_23092022.raw |  | 1.0062E6 |  |  |  |  |  | 1 | 0 | 1 | 0 | 0 | 0 | 0 | 0 | 39 | 59 | Carbamidomethylation | C3:Carbamidomethylation:1000.00;C7:Carbamidomethylation:1000.00;C14:Carbamidomethylation:1000.00 | PEAKS DB |
| K.KIIAVDQDPLGIQGRR.I | Y | 61.34 | 1778.0162 | 16 | -0.2 | 445.5113 | 4 | 43.25 | 2 | F2:8662 | Fraction\_2\_23092022.raw |  | 2.7076E6 |  |  |  |  |  | 1 | 0 | 1 | 0 | 0 | 0 | 0 | 0 | 304 | 319 |  |  | PEAKS DB |
| K.VNPSGVVILR.C | Y | 57.58 | 1052.6342 | 10 | -0.5 | 527.3241 | 2 | 48.05 | 2 | F2:10800 | Fraction\_2\_23092022.raw | 1.3489E5 | 2.0299E6 |  |  |  | 7.2177E5 |  | 3 | 1 | 1 | 0 | 0 | 0 | 1 | 0 | 398 | 407 |  |  | PEAKS DB |
| K.VKVNPSGVVILR.C | Y | 57.54 | 1279.7976 | 12 | 0.8 | 427.6068 | 3 | 43.60 | 2 | F2:8807 | Fraction\_2\_23092022.raw |  | 1.1973E6 |  |  |  | 4.1132E5 |  | 2 | 0 | 1 | 0 | 0 | 0 | 1 | 0 | 396 | 407 |  |  | PEAKS DB |
| K.NLADYIHSKGLK.F | Y | 56.96 | 1357.7354 | 12 | 0.0 | 453.5857 | 3 | 42.00 | 2 | F2:8063 | Fraction\_2\_23092022.raw |  | 2.7559E6 |  |  |  |  |  | 3 | 0 | 3 | 0 | 0 | 0 | 0 | 0 | 108 | 119 |  |  | PEAKS DB |
| K.AILQNKKIIAVDQDPLGIQGRR.I | Y | 47.47 | 2445.4180 | 22 | 0.4 | 612.3620 | 4 | 46.39 | 2 | F2:10087 | Fraction\_2\_23092022.raw |  | 1.0483E5 |  |  |  |  |  | 1 | 0 | 1 | 0 | 0 | 0 | 0 | 0 | 298 | 319 |  |  | PEAKS DB |
| total 14 peptides |  |  |  |  |  |  |  |  |  |  |  |  |  |  |  |  |  |  |  |  |  |  |  |  |  |  |  |  |  |  |  |
| --- | --- | --- | --- | --- | --- | --- | --- | --- | --- | --- | --- | --- | --- | --- | --- | --- | --- | --- | --- | --- | --- | --- | --- | --- | --- | --- | --- | --- | --- | --- | --- |

TRINITY\_DN2611\_c0\_g1\_i9.p1

back to list

  

| Protein Coverage
| Supporting Peptides
|

Protein Coverage:

Supporting Peptides:

| Peptide | Uniq | -10lgP | Mass | Length | ppm | m/z | z | RT | Fraction | Scan | Source File | Area F1 | Area F2 | Area F3 | Area F4 | Area F5 | Area F6 | Area F7 | #Feature | #Feature F1 | #Feature F2 | #Feature F3 | #Feature F4 | #Feature F5 | #Feature F6 | #Feature F7 | Start | End | PTM | AScore | Found By |
| --- | --- | --- | --- | --- | --- | --- | --- | --- | --- | --- | --- | --- | --- | --- | --- | --- | --- | --- | --- | --- | --- | --- | --- | --- | --- | --- | --- | --- | --- | --- | --- |
| K.ELGLQYPGGYKVEDLYEDVNYGVLLPHTK.I | Y | 95.28 | 3308.6604 | 29 | 0.9 | 828.1732 | 4 | 78.63 | 2 | F2:24536 | Fraction\_2\_23092022.raw |  | 5.2189E6 |  |  |  |  |  | 2 | 0 | 2 | 0 | 0 | 0 | 0 | 0 | 365 | 393 |  |  | PEAKS DB |
| K.IIAVDQDPLGIQGR.R | Y | 88.70 | 1493.8202 | 14 | 1.2 | 747.9183 | 2 | 59.22 | 2 | F2:15828 | Fraction\_2\_23092022.raw | 1.0165E6 | 6.4892E7 | 2.4161E5 | 2.8049E5 | 8.3802E5 | 1.3147E7 |  | 6 | 1 | 1 | 1 | 1 | 1 | 1 | 0 | 305 | 318 |  |  | PEAKS DB |
| R.C(+57.02)NTDC(+57.02)KNDPDNC(+57.02)ISDQLFR.T | Y | 85.42 | 2370.9626 | 19 | -1.1 | 791.3273 | 3 | 49.58 | 2 | F2:11492 | Fraction\_2\_23092022.raw |  | 4.6319E6 |  |  |  |  |  | 1 | 0 | 1 | 0 | 0 | 0 | 0 | 0 | 41 | 59 | Carbamidomethylation | C1:Carbamidomethylation:1000.00;C5:Carbamidomethylation:1000.00;C12:Carbamidomethylation:1000.00 | PEAKS DB |
| K.VEDLYEDVNYGVLLPHTK.I | Y | 83.40 | 2103.0525 | 18 | 0.3 | 702.0250 | 3 | 68.15 | 2 | F2:19876 | Fraction\_2\_23092022.raw |  | 3.0315E5 |  |  |  | 1.5647E5 |  | 2 | 0 | 1 | 0 | 0 | 0 | 1 | 0 | 376 | 393 |  |  | PEAKS DB |
| K.KIIAVDQDPLGIQGR.R | Y | 81.39 | 1621.9150 | 15 | 0.5 | 541.6459 | 3 | 49.67 | 5 | F5:12253 | Fraction\_5\_23092022.raw | 2.7777E5 | 1.4111E7 |  |  | 1.3766E5 | 2.7292E6 |  | 6 | 1 | 2 | 0 | 0 | 1 | 2 | 0 | 304 | 318 |  |  | PEAKS DB |
| R.TDGTPSDVSVTLKELGLQYPGGYK.V | Y | 76.14 | 2524.2695 | 24 | 0.6 | 842.4310 | 3 | 73.36 | 2 | F2:22192 | Fraction\_2\_23092022.raw |  | 2.8914E5 |  |  |  |  |  | 1 | 0 | 1 | 0 | 0 | 0 | 0 | 0 | 352 | 375 |  |  | PEAKS DB |
| K.IKVKVNPSGVVILR.C | Y | 63.94 | 1520.9766 | 14 | 0.5 | 507.9997 | 3 | 47.25 | 6 | F6:11791 | Fraction\_6\_23092022.raw |  | 3.9089E6 |  |  |  | 1.0616E6 |  | 2 | 0 | 1 | 0 | 0 | 0 | 1 | 0 | 394 | 407 |  |  | PEAKS DB |
| K.AILQNKKIIAVDQDPLGIQGR.R | Y | 63.34 | 2289.3169 | 21 | -0.3 | 573.3363 | 4 | 51.34 | 2 | F2:12260 | Fraction\_2\_23092022.raw |  | 2.8591E6 |  |  |  |  |  | 1 | 0 | 1 | 0 | 0 | 0 | 0 | 0 | 298 | 318 |  |  | PEAKS DB |
| R.FRC(+57.02)NTDC(+57.02)KNDPDNC(+57.02)ISDQLFR.T | Y | 61.62 | 2674.1323 | 21 | 0.8 | 669.5409 | 4 | 46.02 | 2 | F2:9891 | Fraction\_2\_23092022.raw |  | 1.0062E6 |  |  |  |  |  | 1 | 0 | 1 | 0 | 0 | 0 | 0 | 0 | 39 | 59 | Carbamidomethylation | C3:Carbamidomethylation:1000.00;C7:Carbamidomethylation:1000.00;C14:Carbamidomethylation:1000.00 | PEAKS DB |
| K.KIIAVDQDPLGIQGRR.I | Y | 61.34 | 1778.0162 | 16 | -0.2 | 445.5113 | 4 | 43.25 | 2 | F2:8662 | Fraction\_2\_23092022.raw |  | 2.7076E6 |  |  |  |  |  | 1 | 0 | 1 | 0 | 0 | 0 | 0 | 0 | 304 | 319 |  |  | PEAKS DB |
| K.VNPSGVVILR.C | Y | 57.58 | 1052.6342 | 10 | -0.5 | 527.3241 | 2 | 48.05 | 2 | F2:10800 | Fraction\_2\_23092022.raw | 1.3489E5 | 2.0299E6 |  |  |  | 7.2177E5 |  | 3 | 1 | 1 | 0 | 0 | 0 | 1 | 0 | 398 | 407 |  |  | PEAKS DB |
| K.VKVNPSGVVILR.C | Y | 57.54 | 1279.7976 | 12 | 0.8 | 427.6068 | 3 | 43.60 | 2 | F2:8807 | Fraction\_2\_23092022.raw |  | 1.1973E6 |  |  |  | 4.1132E5 |  | 2 | 0 | 1 | 0 | 0 | 0 | 1 | 0 | 396 | 407 |  |  | PEAKS DB |
| K.NLADYIHSKGLK.F | Y | 56.96 | 1357.7354 | 12 | 0.0 | 453.5857 | 3 | 42.00 | 2 | F2:8063 | Fraction\_2\_23092022.raw |  | 2.7559E6 |  |  |  |  |  | 3 | 0 | 3 | 0 | 0 | 0 | 0 | 0 | 108 | 119 |  |  | PEAKS DB |
| K.AILQNKKIIAVDQDPLGIQGRR.I | Y | 47.47 | 2445.4180 | 22 | 0.4 | 612.3620 | 4 | 46.39 | 2 | F2:10087 | Fraction\_2\_23092022.raw |  | 1.0483E5 |  |  |  |  |  | 1 | 0 | 1 | 0 | 0 | 0 | 0 | 0 | 298 | 319 |  |  | PEAKS DB |
| total 14 peptides |  |  |  |  |  |  |  |  |  |  |  |  |  |  |  |  |  |  |  |  |  |  |  |  |  |  |  |  |  |  |  |
| --- | --- | --- | --- | --- | --- | --- | --- | --- | --- | --- | --- | --- | --- | --- | --- | --- | --- | --- | --- | --- | --- | --- | --- | --- | --- | --- | --- | --- | --- | --- | --- |

P02533|K1C14\_HUMAN

back to list

  

| Protein Coverage
| Supporting Peptides
|

Protein Coverage:

Supporting Peptides:

| Peptide | Uniq | -10lgP | Mass | Length | ppm | m/z | z | RT | Fraction | Scan | Source File | Area F1 | Area F2 | Area F3 | Area F4 | Area F5 | Area F6 | Area F7 | #Feature | #Feature F1 | #Feature F2 | #Feature F3 | #Feature F4 | #Feature F5 | #Feature F6 | #Feature F7 | Start | End | PTM | AScore | Found By |
| --- | --- | --- | --- | --- | --- | --- | --- | --- | --- | --- | --- | --- | --- | --- | --- | --- | --- | --- | --- | --- | --- | --- | --- | --- | --- | --- | --- | --- | --- | --- | --- |
| R.LLEGEDAHLSSSQFSSGSQSSR.D | Y | 95.80 | 2308.0566 | 22 | 0.7 | 770.3600 | 3 | 40.83 | 1 | F1:7538 | Fraction\_1\_23092022r.raw | 6.2238E5 |  |  |  |  |  |  | 1 | 1 | 0 | 0 | 0 | 0 | 0 | 0 | 418 | 439 |  |  | PEAKS DB |
| K.ILTATVDNANVLLQIDNAR.L | Y | 89.65 | 2053.1167 | 19 | 0.5 | 1027.5662 | 2 | 72.98 | 1 | F1:21821 | Fraction\_1\_23092022r.raw | 1.0482E6 |  | 8.4193E4 |  |  |  |  | 3 | 2 | 0 | 1 | 0 | 0 | 0 | 0 | 176 | 194 |  |  | PEAKS DB |
| R.GQVGGDVNVEM(+15.99)DAAPGVDLSR.I | Y | 87.65 | 2100.9746 | 21 | 0.5 | 1051.4951 | 2 | 50.17 | 1 | F1:11644 | Fraction\_1\_23092022r.raw | 4.1512E5 |  |  |  |  |  |  | 1 | 1 | 0 | 0 | 0 | 0 | 0 | 0 | 262 | 282 | Oxidation (M) | M11:Oxidation (M):1000.00 | PEAKS DB |
| R.ALEEANADLEVK.I | N | 74.72 | 1300.6510 | 12 | 0.5 | 651.3331 | 2 | 40.27 | 3 | F3:7722 | Fraction\_3\_23092022.raw | 4.9827E5 | 2.0105E5 | 6.9699E5 | 6.4208E4 | 5.161E4 | 1.2986E5 |  | 6 | 1 | 1 | 1 | 1 | 1 | 1 | 0 | 135 | 146 |  |  | PEAKS DB |
| R.APSTYGGGLSVSSSR.F | Y | 74.23 | 1424.6896 | 15 | 0.7 | 713.3525 | 2 | 34.68 | 6 | F6:6260 | Fraction\_6\_23092022.raw |  |  |  |  |  | 1.0711E5 |  | 1 | 0 | 0 | 0 | 0 | 0 | 1 | 0 | 42 | 56 |  |  | PEAKS DB |
| R.NKILTATVDNANVLLQIDNAR.L | Y | 73.26 | 2295.2546 | 21 | 0.6 | 766.0927 | 3 | 67.17 | 1 | F1:19248 | Fraction\_1\_23092022r.raw | 2.5238E5 |  |  |  |  |  |  | 1 | 1 | 0 | 0 | 0 | 0 | 0 | 0 | 174 | 194 |  |  | PEAKS DB |
| R.ISSVLAGGSC(+57.02)RAPSTYGGGLSVSSSR.F | Y | 68.08 | 2512.2339 | 26 | 0.2 | 838.4187 | 3 | 45.64 | 7 | F7:13350 | Fraction\_7\_23092022.raw | 1.0175E5 |  |  |  |  |  | 3.6841E5 | 2 | 1 | 0 | 0 | 0 | 0 | 0 | 1 | 31 | 56 | Carbamidomethylation | C10:Carbamidomethylation:1000.00 | PEAKS DB |
| R.EVATNSELVQSGKSEISELR.R | N | 63.14 | 2175.1018 | 20 | 0.4 | 726.0415 | 3 | 49.48 | 1 | F1:11348 | Fraction\_1\_23092022r.raw | 1.8745E5 |  |  |  |  |  |  | 1 | 1 | 0 | 0 | 0 | 0 | 0 | 0 | 316 | 335 |  |  | PEAKS DB |
| K.VTM(+15.99)QNLNDRLASYLDKVR.A | N | 58.54 | 2151.1106 | 18 | 0.9 | 538.7854 | 4 | 56.01 | 1 | F1:14301 | Fraction\_1\_23092022r.raw | 7.88E6 | 8.0553E5 |  |  |  |  |  | 3 | 2 | 1 | 0 | 0 | 0 | 0 | 0 | 117 | 134 | Oxidation (M) | M3:Oxidation (M):1000.00 | PEAKS DB |
| K.VTMQNLNDRLASYLDKVR.A | N | 58.08 | 2135.1157 | 18 | 0.3 | 712.7127 | 3 | 63.69 | 1 | F1:17659 | Fraction\_1\_23092022r.raw | 2.701E6 |  |  |  |  |  |  | 2 | 2 | 0 | 0 | 0 | 0 | 0 | 0 | 117 | 134 |  |  | PEAKS DB |
| K.VTMQNLNDRLASYLDK.V | N | 56.99 | 1879.9462 | 16 | -0.8 | 627.6555 | 3 | 61.55 | 1 | F1:16687 | Fraction\_1\_23092022r.raw | 5.9999E5 |  |  |  |  |  |  | 1 | 1 | 0 | 0 | 0 | 0 | 0 | 0 | 117 | 132 |  |  | PEAKS DB |
| R.LLEGEDAHLSSSQFSSGSQSSRDVTSSSR.Q | Y | 53.93 | 3040.3970 | 29 | 0.1 | 761.1066 | 4 | 38.62 | 2 | F2:6607 | Fraction\_2\_23092022.raw |  | 8.5264E4 |  |  |  |  |  | 1 | 0 | 1 | 0 | 0 | 0 | 0 | 0 | 418 | 446 |  |  | PEAKS DB |
| R.MSVEADINGLRR.V | N | 50.41 | 1359.6929 | 12 | -0.4 | 454.2381 | 3 | 43.39 | 1 | F1:8670 | Fraction\_1\_23092022r.raw | 7.1864E4 |  |  |  |  |  |  | 1 | 1 | 0 | 0 | 0 | 0 | 0 | 0 | 212 | 223 |  |  | PEAKS DB |
| R.LAADDFRTKYETELNLR.M | Y | 48.77 | 2054.0432 | 17 | 0.2 | 685.6885 | 3 | 52.79 | 1 | F1:12803 | Fraction\_1\_23092022r.raw | 3.1521E5 |  |  |  |  |  |  | 1 | 1 | 0 | 0 | 0 | 0 | 0 | 0 | 195 | 211 |  |  | PEAKS DB |
| total 14 peptides |  |  |  |  |  |  |  |  |  |  |  |  |  |  |  |  |  |  |  |  |  |  |  |  |  |  |  |  |  |  |  |
| --- | --- | --- | --- | --- | --- | --- | --- | --- | --- | --- | --- | --- | --- | --- | --- | --- | --- | --- | --- | --- | --- | --- | --- | --- | --- | --- | --- | --- | --- | --- | --- |

TRINITY\_DN13586\_c0\_g2\_i1.p1

back to list

  

| Protein Coverage
| Supporting Peptides
|

Protein Coverage:

Supporting Peptides:

| Peptide | Uniq | -10lgP | Mass | Length | ppm | m/z | z | RT | Fraction | Scan | Source File | Area F1 | Area F2 | Area F3 | Area F4 | Area F5 | Area F6 | Area F7 | #Feature | #Feature F1 | #Feature F2 | #Feature F3 | #Feature F4 | #Feature F5 | #Feature F6 | #Feature F7 | Start | End | PTM | AScore | Found By |
| --- | --- | --- | --- | --- | --- | --- | --- | --- | --- | --- | --- | --- | --- | --- | --- | --- | --- | --- | --- | --- | --- | --- | --- | --- | --- | --- | --- | --- | --- | --- | --- |
| K.YVLYLQESGFDVIKR.L | Y | 87.66 | 1828.9723 | 15 | 0.8 | 610.6652 | 3 | 65.52 | 6 | F6:20037 | Fraction\_6\_23092022.raw |  |  |  |  |  | 1.3637E6 |  | 2 | 0 | 0 | 0 | 0 | 0 | 2 | 0 | 126 | 140 |  |  | PEAKS DB |
| K.YVLYLQESGFDVIK.R | Y | 82.33 | 1672.8712 | 14 | -0.2 | 837.4427 | 2 | 75.65 | 6 | F6:24543 | Fraction\_6\_23092022.raw |  |  |  |  |  | 2.0976E6 |  | 1 | 0 | 0 | 0 | 0 | 0 | 1 | 0 | 126 | 139 |  |  | PEAKS DB |
| K.FISYLSDQKFSDIRK.N | Y | 81.88 | 1845.9624 | 15 | 0.2 | 923.9886 | 2 | 47.40 | 7 | F7:14163 | Fraction\_7\_23092022.raw |  |  |  |  |  |  | 2.0263E8 | 4 | 0 | 0 | 0 | 0 | 0 | 0 | 4 | 206 | 220 |  |  | PEAKS DB |
| R.KLKESETFSKFISYLSDQK.F | Y | 81.73 | 2277.1892 | 19 | 0.6 | 570.3049 | 4 | 59.83 | 7 | F7:21409 | Fraction\_7\_23092022.raw |  |  |  |  |  |  | 2.1113E6 | 1 | 0 | 0 | 0 | 0 | 0 | 0 | 1 | 196 | 214 |  |  | PEAKS DB |
| K.ESETFSKFISYLSDQK.F | Y | 78.66 | 1907.9152 | 16 | 0.6 | 636.9794 | 3 | 71.47 | 7 | F7:27704 | Fraction\_7\_23092022.raw |  |  |  |  |  |  | 1.4093E6 | 1 | 0 | 0 | 0 | 0 | 0 | 0 | 1 | 199 | 214 |  |  | PEAKS DB |
| K.FISYLSDQKFSDIR.K | Y | 77.11 | 1717.8674 | 14 | 0.5 | 573.6301 | 3 | 55.96 | 7 | F7:18941 | Fraction\_7\_23092022.raw |  |  |  |  |  | 2.1992E5 | 5.8297E7 | 3 | 0 | 0 | 0 | 0 | 0 | 1 | 2 | 206 | 219 |  |  | PEAKS DB |
| R.YKDDPQIINGYIYLHHK.H | Y | 74.37 | 2116.0742 | 17 | -0.4 | 530.0256 | 4 | 44.92 | 6 | F6:10765 | Fraction\_6\_23092022.raw |  |  |  |  |  | 4.3453E5 |  | 1 | 0 | 0 | 0 | 0 | 0 | 1 | 0 | 92 | 108 |  |  | PEAKS DB |
| K.EIIAILPVEELKELHKR.K | Y | 73.74 | 2029.1935 | 17 | -0.8 | 508.3052 | 4 | 61.19 | 7 | F7:22240 | Fraction\_7\_23092022.raw |  |  |  |  |  |  | 8.7018E6 | 2 | 0 | 0 | 0 | 0 | 0 | 0 | 2 | 179 | 195 |  |  | PEAKS DB |
| K.LKESETFSKFISYLSDQK.F | Y | 63.88 | 2149.0942 | 18 | 0.8 | 717.3726 | 3 | 66.81 | 7 | F7:25373 | Fraction\_7\_23092022.raw |  |  |  |  |  |  | 1.4916E6 | 1 | 0 | 0 | 0 | 0 | 0 | 0 | 1 | 197 | 214 |  |  | PEAKS DB |
| K.EIIAILPVEELKELHK.R | Y | 60.28 | 1873.0924 | 16 | -0.3 | 625.3712 | 3 | 68.82 | 7 | F7:26410 | Fraction\_7\_23092022.raw |  |  |  |  |  |  | 1.9268E6 | 1 | 0 | 0 | 0 | 0 | 0 | 0 | 1 | 179 | 194 |  |  | PEAKS DB |
| K.LIPLDKLGAVLER.Y | Y | 59.03 | 1435.8762 | 13 | 0.0 | 479.6327 | 3 | 68.92 | 5 | F5:20876 | Fraction\_5\_23092022.raw |  |  |  | 9.9343E4 | 2.2425E5 | 2.1566E7 | 4.1166E6 | 5 | 0 | 0 | 0 | 1 | 1 | 2 | 1 | 79 | 91 |  |  | PEAKS DB |
| K.EIIAILPVEELK.E | Y | 58.89 | 1365.8119 | 12 | 0.3 | 683.9135 | 2 | 78.20 | 7 | F7:31056 | Fraction\_7\_23092022.raw |  |  |  |  |  |  | 1.3553E7 | 1 | 0 | 0 | 0 | 0 | 0 | 0 | 1 | 179 | 190 |  |  | PEAKS DB |
| K.FISYLSDQK.F | Y | 58.72 | 1099.5549 | 9 | 0.1 | 550.7848 | 2 | 45.40 | 7 | F7:13206 | Fraction\_7\_23092022.raw |  |  |  |  |  |  | 8.7298E6 | 1 | 0 | 0 | 0 | 0 | 0 | 0 | 1 | 206 | 214 |  |  | PEAKS DB |
| K.ESETFSKFISYLSDQKFSDIRK.N | Y | 51.53 | 2654.3228 | 22 | 0.1 | 664.5880 | 4 | 68.73 | 7 | F7:26341 | Fraction\_7\_23092022.raw |  |  |  |  |  |  | 8.9428E6 | 1 | 0 | 0 | 0 | 0 | 0 | 0 | 1 | 199 | 220 |  |  | PEAKS DB |
| total 14 peptides |  |  |  |  |  |  |  |  |  |  |  |  |  |  |  |  |  |  |  |  |  |  |  |  |  |  |  |  |  |  |  |
| --- | --- | --- | --- | --- | --- | --- | --- | --- | --- | --- | --- | --- | --- | --- | --- | --- | --- | --- | --- | --- | --- | --- | --- | --- | --- | --- | --- | --- | --- | --- | --- |

TRINITY\_DN10256\_c0\_g10\_i1.p1

back to list

  

| Protein Coverage
| Supporting Peptides
|

Protein Coverage:

Supporting Peptides:

| Peptide | Uniq | -10lgP | Mass | Length | ppm | m/z | z | RT | Fraction | Scan | Source File | Area F1 | Area F2 | Area F3 | Area F4 | Area F5 | Area F6 | Area F7 | #Feature | #Feature F1 | #Feature F2 | #Feature F3 | #Feature F4 | #Feature F5 | #Feature F6 | #Feature F7 | Start | End | PTM | AScore | Found By |
| --- | --- | --- | --- | --- | --- | --- | --- | --- | --- | --- | --- | --- | --- | --- | --- | --- | --- | --- | --- | --- | --- | --- | --- | --- | --- | --- | --- | --- | --- | --- | --- |
| R.SLYNNLKAGAESGWDFSGR.W | Y | 95.54 | 2070.9758 | 19 | 0.7 | 691.3330 | 3 | 59.17 | 2 | F2:15841 | Fraction\_2\_23092022.raw |  | 9.8861E5 |  |  |  |  |  | 1 | 0 | 1 | 0 | 0 | 0 | 0 | 0 | 311 | 329 |  |  | PEAKS DB |
| K.TFVDLSQKDDPEITLSK.F | Y | 86.40 | 1934.9836 | 17 | 0.2 | 646.0020 | 3 | 55.15 | 2 | F2:14017 | Fraction\_2\_23092022.raw |  | 7.291E5 |  |  |  |  |  | 1 | 0 | 1 | 0 | 0 | 0 | 0 | 0 | 57 | 73 |  |  | PEAKS DB |
| R.ENNNSPDLC(+57.02)TSQIYC(+57.02)QGNLLK.T | Y | 85.52 | 2467.1108 | 21 | 1.5 | 823.3788 | 3 | 58.53 | 2 | F2:15508 | Fraction\_2\_23092022.raw |  | 3.2098E7 | 4.4193E5 | 7.2858E5 | 5.167E5 |  |  | 7 | 0 | 2 | 1 | 2 | 2 | 0 | 0 | 24 | 44 | Carbamidomethylation | C9:Carbamidomethylation:1000.00;C15:Carbamidomethylation:1000.00 | PEAKS DB |
| R.WFIGPDGKESNNLIDINTK.N | Y | 83.69 | 2160.0850 | 19 | 2.0 | 721.0370 | 3 | 65.13 | 2 | F2:18560 | Fraction\_2\_23092022.raw |  | 4.2328E6 |  |  |  | 3.8644E5 |  | 2 | 0 | 1 | 0 | 0 | 0 | 1 | 0 | 330 | 348 |  |  | PEAKS DB |
| R.HSLIYVDNAFVVPGGR.F | Y | 83.21 | 1742.9104 | 16 | -0.1 | 581.9774 | 3 | 61.13 | 5 | F5:17406 | Fraction\_5\_23092022.raw |  | 1.8244E6 | 1.5223E5 | 4.4864E5 | 3.1981E5 |  |  | 5 | 0 | 2 | 1 | 1 | 1 | 0 | 0 | 156 | 171 |  |  | PEAKS DB |
| R.RENNNSPDLC(+57.02)TSQIYC(+57.02)QGNLLK.T | Y | 78.95 | 2623.2119 | 22 | -0.3 | 875.4110 | 3 | 51.10 | 2 | F2:12167 | Fraction\_2\_23092022.raw |  | 4.6187E5 |  |  |  |  |  | 1 | 0 | 1 | 0 | 0 | 0 | 0 | 0 | 23 | 44 | Carbamidomethylation | C10:Carbamidomethylation:1000.00;C16:Carbamidomethylation:1000.00 | PEAKS DB |
| K.AGAESGWDFSGR.W | Y | 69.21 | 1238.5316 | 12 | 1.1 | 620.2737 | 2 | 47.46 | 1 | F1:10445 | Fraction\_1\_23092022r.raw | 6.326E4 |  |  |  |  |  |  | 1 | 1 | 0 | 0 | 0 | 0 | 0 | 0 | 318 | 329 |  |  | PEAKS DB |
| K.TFVDLSQKDDPEITLSKFK.K | Y | 67.38 | 2210.1470 | 19 | 0.4 | 553.5443 | 4 | 58.45 | 2 | F2:15512 | Fraction\_2\_23092022.raw |  | 2.2053E5 |  |  |  |  |  | 1 | 0 | 1 | 0 | 0 | 0 | 0 | 0 | 57 | 75 |  |  | PEAKS DB |
| R.EIALSAISYLK.K | Y | 66.78 | 1206.6859 | 11 | 0.7 | 604.3506 | 2 | 68.54 | 5 | F5:20709 | Fraction\_5\_23092022.raw | 6.3127E5 | 1.8624E6 | 7.6118E5 | 9.3599E5 | 1.4628E6 | 6.7308E4 |  | 6 | 1 | 1 | 1 | 1 | 1 | 1 | 0 | 439 | 449 |  |  | PEAKS DB |
| R.EIALSAISYLKK.M | Y | 59.67 | 1334.7809 | 12 | -0.2 | 668.3976 | 2 | 57.01 | 2 | F2:14857 | Fraction\_2\_23092022.raw |  | 1.2737E6 | 2.6943E5 |  | 4.4535E5 |  |  | 3 | 0 | 1 | 1 | 0 | 1 | 0 | 0 | 439 | 450 |  |  | PEAKS DB |
| R.WFIGPDGK.E | Y | 48.31 | 918.4599 | 8 | -0.5 | 460.2370 | 2 | 55.08 | 2 | F2:14002 | Fraction\_2\_23092022.raw |  | 1.8407E5 |  |  |  |  |  | 1 | 0 | 1 | 0 | 0 | 0 | 0 | 0 | 330 | 337 |  |  | PEAKS DB |
| total 11 peptides |  |  |  |  |  |  |  |  |  |  |  |  |  |  |  |  |  |  |  |  |  |  |  |  |  |  |  |  |  |  |  |
| --- | --- | --- | --- | --- | --- | --- | --- | --- | --- | --- | --- | --- | --- | --- | --- | --- | --- | --- | --- | --- | --- | --- | --- | --- | --- | --- | --- | --- | --- | --- | --- |

TRINITY\_DN10256\_c0\_g5\_i4.p1

back to list

  

| Protein Coverage
| Supporting Peptides
|

Protein Coverage:

Supporting Peptides:

| Peptide | Uniq | -10lgP | Mass | Length | ppm | m/z | z | RT | Fraction | Scan | Source File | Area F1 | Area F2 | Area F3 | Area F4 | Area F5 | Area F6 | Area F7 | #Feature | #Feature F1 | #Feature F2 | #Feature F3 | #Feature F4 | #Feature F5 | #Feature F6 | #Feature F7 | Start | End | PTM | AScore | Found By |
| --- | --- | --- | --- | --- | --- | --- | --- | --- | --- | --- | --- | --- | --- | --- | --- | --- | --- | --- | --- | --- | --- | --- | --- | --- | --- | --- | --- | --- | --- | --- | --- |
| R.SLYNNLKAGAESGWDFSGR.W | Y | 95.54 | 2070.9758 | 19 | 0.7 | 691.3330 | 3 | 59.17 | 2 | F2:15841 | Fraction\_2\_23092022.raw |  | 9.8861E5 |  |  |  |  |  | 1 | 0 | 1 | 0 | 0 | 0 | 0 | 0 | 311 | 329 |  |  | PEAKS DB |
| K.TFVDLSQKDDPEITLSK.F | Y | 86.40 | 1934.9836 | 17 | 0.2 | 646.0020 | 3 | 55.15 | 2 | F2:14017 | Fraction\_2\_23092022.raw |  | 7.291E5 |  |  |  |  |  | 1 | 0 | 1 | 0 | 0 | 0 | 0 | 0 | 57 | 73 |  |  | PEAKS DB |
| R.ENNNSPDLC(+57.02)TSQIYC(+57.02)QGNLLK.T | Y | 85.52 | 2467.1108 | 21 | 1.5 | 823.3788 | 3 | 58.53 | 2 | F2:15508 | Fraction\_2\_23092022.raw |  | 3.2098E7 | 4.4193E5 | 7.2858E5 | 5.167E5 |  |  | 7 | 0 | 2 | 1 | 2 | 2 | 0 | 0 | 24 | 44 | Carbamidomethylation | C9:Carbamidomethylation:1000.00;C15:Carbamidomethylation:1000.00 | PEAKS DB |
| R.WFIGPDGKESNNLIDINTK.N | Y | 83.69 | 2160.0850 | 19 | 2.0 | 721.0370 | 3 | 65.13 | 2 | F2:18560 | Fraction\_2\_23092022.raw |  | 4.2328E6 |  |  |  | 3.8644E5 |  | 2 | 0 | 1 | 0 | 0 | 0 | 1 | 0 | 330 | 348 |  |  | PEAKS DB |
| R.HSLIYVDNAFVVPGGR.F | Y | 83.21 | 1742.9104 | 16 | -0.1 | 581.9774 | 3 | 61.13 | 5 | F5:17406 | Fraction\_5\_23092022.raw |  | 1.8244E6 | 1.5223E5 | 4.4864E5 | 3.1981E5 |  |  | 5 | 0 | 2 | 1 | 1 | 1 | 0 | 0 | 156 | 171 |  |  | PEAKS DB |
| R.RENNNSPDLC(+57.02)TSQIYC(+57.02)QGNLLK.T | Y | 78.95 | 2623.2119 | 22 | -0.3 | 875.4110 | 3 | 51.10 | 2 | F2:12167 | Fraction\_2\_23092022.raw |  | 4.6187E5 |  |  |  |  |  | 1 | 0 | 1 | 0 | 0 | 0 | 0 | 0 | 23 | 44 | Carbamidomethylation | C10:Carbamidomethylation:1000.00;C16:Carbamidomethylation:1000.00 | PEAKS DB |
| K.AGAESGWDFSGR.W | Y | 69.21 | 1238.5316 | 12 | 1.1 | 620.2737 | 2 | 47.46 | 1 | F1:10445 | Fraction\_1\_23092022r.raw | 6.326E4 |  |  |  |  |  |  | 1 | 1 | 0 | 0 | 0 | 0 | 0 | 0 | 318 | 329 |  |  | PEAKS DB |
| K.TFVDLSQKDDPEITLSKFK.K | Y | 67.38 | 2210.1470 | 19 | 0.4 | 553.5443 | 4 | 58.45 | 2 | F2:15512 | Fraction\_2\_23092022.raw |  | 2.2053E5 |  |  |  |  |  | 1 | 0 | 1 | 0 | 0 | 0 | 0 | 0 | 57 | 75 |  |  | PEAKS DB |
| R.EIALSAISYLK.K | Y | 66.78 | 1206.6859 | 11 | 0.7 | 604.3506 | 2 | 68.54 | 5 | F5:20709 | Fraction\_5\_23092022.raw | 6.3127E5 | 1.8624E6 | 7.6118E5 | 9.3599E5 | 1.4628E6 | 6.7308E4 |  | 6 | 1 | 1 | 1 | 1 | 1 | 1 | 0 | 439 | 449 |  |  | PEAKS DB |
| R.EIALSAISYLKK.M | Y | 59.67 | 1334.7809 | 12 | -0.2 | 668.3976 | 2 | 57.01 | 2 | F2:14857 | Fraction\_2\_23092022.raw |  | 1.2737E6 | 2.6943E5 |  | 4.4535E5 |  |  | 3 | 0 | 1 | 1 | 0 | 1 | 0 | 0 | 439 | 450 |  |  | PEAKS DB |
| R.WFIGPDGK.E | Y | 48.31 | 918.4599 | 8 | -0.5 | 460.2370 | 2 | 55.08 | 2 | F2:14002 | Fraction\_2\_23092022.raw |  | 1.8407E5 |  |  |  |  |  | 1 | 0 | 1 | 0 | 0 | 0 | 0 | 0 | 330 | 337 |  |  | PEAKS DB |
| total 11 peptides |  |  |  |  |  |  |  |  |  |  |  |  |  |  |  |  |  |  |  |  |  |  |  |  |  |  |  |  |  |  |  |
| --- | --- | --- | --- | --- | --- | --- | --- | --- | --- | --- | --- | --- | --- | --- | --- | --- | --- | --- | --- | --- | --- | --- | --- | --- | --- | --- | --- | --- | --- | --- | --- |

TRINITY\_DN1113\_c0\_g1\_i95.p1

back to list

  

| Protein Coverage
| Supporting Peptides
|

Protein Coverage:

Supporting Peptides:

| Peptide | Uniq | -10lgP | Mass | Length | ppm | m/z | z | RT | Fraction | Scan | Source File | Area F1 | Area F2 | Area F3 | Area F4 | Area F5 | Area F6 | Area F7 | #Feature | #Feature F1 | #Feature F2 | #Feature F3 | #Feature F4 | #Feature F5 | #Feature F6 | #Feature F7 | Start | End | PTM | AScore | Found By |
| --- | --- | --- | --- | --- | --- | --- | --- | --- | --- | --- | --- | --- | --- | --- | --- | --- | --- | --- | --- | --- | --- | --- | --- | --- | --- | --- | --- | --- | --- | --- | --- |
| R.DISTINRAQLIDDALNLAR.A | Y | 91.27 | 2111.1335 | 19 | -0.3 | 704.7183 | 3 | 79.44 | 5 | F5:25581 | Fraction\_5\_23092022.raw |  |  |  | 2.0669E5 | 4.0594E5 | 2.772E6 |  | 3 | 0 | 0 | 0 | 1 | 1 | 1 | 0 | 618 | 636 |  |  | PEAKS DB |
| R.TVQQAVEQAEANIK.W | Y | 83.62 | 1527.7893 | 14 | 0.7 | 764.9025 | 2 | 53.92 | 5 | F5:14133 | Fraction\_5\_23092022.raw |  |  |  | 1.2796E6 | 7.1371E5 |  |  | 4 | 0 | 0 | 0 | 2 | 2 | 0 | 0 | 901 | 914 |  |  | PEAKS DB |
| K.FVGQLNDYLQGFYR.S | Y | 81.13 | 1718.8416 | 14 | -0.4 | 860.4277 | 2 | 77.39 | 4 | F4:24889 | Fraction\_4\_23092022.raw |  |  | 8.706E5 | 1.509E6 | 1.2277E5 | 4.5552E5 |  | 4 | 0 | 0 | 1 | 1 | 1 | 1 | 0 | 145 | 158 |  |  | PEAKS DB |
| R.AQLIDDALNLAR.A | Y | 77.33 | 1311.7146 | 12 | 0.3 | 656.8647 | 2 | 68.07 | 5 | F5:20470 | Fraction\_5\_23092022.raw |  |  |  | 9.1366E5 | 2.2643E6 | 1.4183E7 | 2.1683E6 | 4 | 0 | 0 | 0 | 1 | 1 | 1 | 1 | 625 | 636 |  |  | PEAKS DB |
| K.DLIDFITENKDELGSATR.T | Y | 77.32 | 2036.0061 | 18 | -0.5 | 679.6757 | 3 | 77.19 | 5 | F5:24575 | Fraction\_5\_23092022.raw |  |  |  | 1.1819E6 | 4.3715E5 |  |  | 2 | 0 | 0 | 0 | 1 | 1 | 0 | 0 | 883 | 900 |  |  | PEAKS DB |
| R.WIATTQFQATDAR.R | Y | 71.57 | 1507.7419 | 13 | 0.4 | 754.8785 | 2 | 50.93 | 4 | F4:13047 | Fraction\_4\_23092022.raw |  |  |  | 9.3696E5 |  |  |  | 1 | 0 | 0 | 0 | 1 | 0 | 0 | 0 | 169 | 181 |  |  | PEAKS DB |
| R.KM(+15.99)PDPDTNNPISPNLK.S | Y | 66.18 | 1795.8774 | 16 | 1.4 | 599.6339 | 3 | 34.19 | 4 | F4:5844 | Fraction\_4\_23092022.raw |  |  |  | 2.4364E5 |  |  |  | 2 | 0 | 0 | 0 | 2 | 0 | 0 | 0 | 743 | 758 | Oxidation (M) | M2:Oxidation (M):1000.00 | PEAKS DB |
| R.VNYDRTNWQLIIK.Q | Y | 61.26 | 1661.8889 | 13 | -0.4 | 554.9700 | 3 | 56.29 | 6 | F6:15899 | Fraction\_6\_23092022.raw |  |  |  |  |  | 4.1501E5 |  | 1 | 0 | 0 | 0 | 0 | 0 | 1 | 0 | 597 | 609 |  |  | PEAKS DB |
| R.LREYFGTSLVLINNIVK.S | Y | 60.64 | 1978.1251 | 17 | 0.8 | 660.3828 | 3 | 75.86 | 4 | F4:24204 | Fraction\_4\_23092022.raw |  |  |  | 3.3605E5 |  |  |  | 1 | 0 | 0 | 0 | 1 | 0 | 0 | 0 | 853 | 869 |  |  | PEAKS DB |
| R.YLNTNVGSEKDLLLQALGC(+57.02)SR.E | Y | 52.74 | 2350.1951 | 21 | -0.1 | 784.4055 | 3 | 70.62 | 4 | F4:21896 | Fraction\_4\_23092022.raw |  |  |  | 8.0462E4 |  |  |  | 1 | 0 | 0 | 0 | 1 | 0 | 0 | 0 | 781 | 801 | Carbamidomethylation | C19:Carbamidomethylation:1000.00 | PEAKS DB |
| R.NYLNGDATLTQER.F | Y | 52.50 | 1493.7111 | 13 | 0.5 | 747.8632 | 2 | 41.93 | 6 | F6:9406 | Fraction\_6\_23092022.raw |  |  |  |  |  | 7.4213E4 |  | 1 | 0 | 0 | 0 | 0 | 0 | 1 | 0 | 513 | 525 |  |  | PEAKS DB |
| total 11 peptides |  |  |  |  |  |  |  |  |  |  |  |  |  |  |  |  |  |  |  |  |  |  |  |  |  |  |  |  |  |  |  |
| --- | --- | --- | --- | --- | --- | --- | --- | --- | --- | --- | --- | --- | --- | --- | --- | --- | --- | --- | --- | --- | --- | --- | --- | --- | --- | --- | --- | --- | --- | --- | --- |

TRINITY\_DN1113\_c0\_g1\_i47.p1

back to list

  

| Protein Coverage
| Supporting Peptides
|

Protein Coverage:

Supporting Peptides:

| Peptide | Uniq | -10lgP | Mass | Length | ppm | m/z | z | RT | Fraction | Scan | Source File | Area F1 | Area F2 | Area F3 | Area F4 | Area F5 | Area F6 | Area F7 | #Feature | #Feature F1 | #Feature F2 | #Feature F3 | #Feature F4 | #Feature F5 | #Feature F6 | #Feature F7 | Start | End | PTM | AScore | Found By |
| --- | --- | --- | --- | --- | --- | --- | --- | --- | --- | --- | --- | --- | --- | --- | --- | --- | --- | --- | --- | --- | --- | --- | --- | --- | --- | --- | --- | --- | --- | --- | --- |
| R.DISTINRAQLIDDALNLAR.A | Y | 91.27 | 2111.1335 | 19 | -0.3 | 704.7183 | 3 | 79.44 | 5 | F5:25581 | Fraction\_5\_23092022.raw |  |  |  | 2.0669E5 | 4.0594E5 | 2.772E6 |  | 3 | 0 | 0 | 0 | 1 | 1 | 1 | 0 | 669 | 687 |  |  | PEAKS DB |
| R.TVQQAVEQAEANIK.W | Y | 83.62 | 1527.7893 | 14 | 0.7 | 764.9025 | 2 | 53.92 | 5 | F5:14133 | Fraction\_5\_23092022.raw |  |  |  | 1.2796E6 | 7.1371E5 |  |  | 4 | 0 | 0 | 0 | 2 | 2 | 0 | 0 | 952 | 965 |  |  | PEAKS DB |
| K.FVGQLNDYLQGFYR.S | Y | 81.13 | 1718.8416 | 14 | -0.4 | 860.4277 | 2 | 77.39 | 4 | F4:24889 | Fraction\_4\_23092022.raw |  |  | 8.706E5 | 1.509E6 | 1.2277E5 | 4.5552E5 |  | 4 | 0 | 0 | 1 | 1 | 1 | 1 | 0 | 196 | 209 |  |  | PEAKS DB |
| R.AQLIDDALNLAR.A | Y | 77.33 | 1311.7146 | 12 | 0.3 | 656.8647 | 2 | 68.07 | 5 | F5:20470 | Fraction\_5\_23092022.raw |  |  |  | 9.1366E5 | 2.2643E6 | 1.4183E7 | 2.1683E6 | 4 | 0 | 0 | 0 | 1 | 1 | 1 | 1 | 676 | 687 |  |  | PEAKS DB |
| K.DLIDFITENKDELGSATR.T | Y | 77.32 | 2036.0061 | 18 | -0.5 | 679.6757 | 3 | 77.19 | 5 | F5:24575 | Fraction\_5\_23092022.raw |  |  |  | 1.1819E6 | 4.3715E5 |  |  | 2 | 0 | 0 | 0 | 1 | 1 | 0 | 0 | 934 | 951 |  |  | PEAKS DB |
| R.WIATTQFQATDAR.R | Y | 71.57 | 1507.7419 | 13 | 0.4 | 754.8785 | 2 | 50.93 | 4 | F4:13047 | Fraction\_4\_23092022.raw |  |  |  | 9.3696E5 |  |  |  | 1 | 0 | 0 | 0 | 1 | 0 | 0 | 0 | 220 | 232 |  |  | PEAKS DB |
| R.KM(+15.99)PDPDTNNPISPNLK.S | Y | 66.18 | 1795.8774 | 16 | 1.4 | 599.6339 | 3 | 34.19 | 4 | F4:5844 | Fraction\_4\_23092022.raw |  |  |  | 2.4364E5 |  |  |  | 2 | 0 | 0 | 0 | 2 | 0 | 0 | 0 | 794 | 809 | Oxidation (M) | M2:Oxidation (M):1000.00 | PEAKS DB |
| R.VNYDRTNWQLIIK.Q | Y | 61.26 | 1661.8889 | 13 | -0.4 | 554.9700 | 3 | 56.29 | 6 | F6:15899 | Fraction\_6\_23092022.raw |  |  |  |  |  | 4.1501E5 |  | 1 | 0 | 0 | 0 | 0 | 0 | 1 | 0 | 648 | 660 |  |  | PEAKS DB |
| R.LREYFGTSLVLINNIVK.S | Y | 60.64 | 1978.1251 | 17 | 0.8 | 660.3828 | 3 | 75.86 | 4 | F4:24204 | Fraction\_4\_23092022.raw |  |  |  | 3.3605E5 |  |  |  | 1 | 0 | 0 | 0 | 1 | 0 | 0 | 0 | 904 | 920 |  |  | PEAKS DB |
| R.YLNTNVGSEKDLLLQALGC(+57.02)SR.E | Y | 52.74 | 2350.1951 | 21 | -0.1 | 784.4055 | 3 | 70.62 | 4 | F4:21896 | Fraction\_4\_23092022.raw |  |  |  | 8.0462E4 |  |  |  | 1 | 0 | 0 | 0 | 1 | 0 | 0 | 0 | 832 | 852 | Carbamidomethylation | C19:Carbamidomethylation:1000.00 | PEAKS DB |
| R.NYLNGDATLTQER.F | Y | 52.50 | 1493.7111 | 13 | 0.5 | 747.8632 | 2 | 41.93 | 6 | F6:9406 | Fraction\_6\_23092022.raw |  |  |  |  |  | 7.4213E4 |  | 1 | 0 | 0 | 0 | 0 | 0 | 1 | 0 | 564 | 576 |  |  | PEAKS DB |
| total 11 peptides |  |  |  |  |  |  |  |  |  |  |  |  |  |  |  |  |  |  |  |  |  |  |  |  |  |  |  |  |  |  |  |
| --- | --- | --- | --- | --- | --- | --- | --- | --- | --- | --- | --- | --- | --- | --- | --- | --- | --- | --- | --- | --- | --- | --- | --- | --- | --- | --- | --- | --- | --- | --- | --- |

TRINITY\_DN1113\_c0\_g1\_i59.p1

back to list

  

| Protein Coverage
| Supporting Peptides
|

Protein Coverage:

Supporting Peptides:

| Peptide | Uniq | -10lgP | Mass | Length | ppm | m/z | z | RT | Fraction | Scan | Source File | Area F1 | Area F2 | Area F3 | Area F4 | Area F5 | Area F6 | Area F7 | #Feature | #Feature F1 | #Feature F2 | #Feature F3 | #Feature F4 | #Feature F5 | #Feature F6 | #Feature F7 | Start | End | PTM | AScore | Found By |
| --- | --- | --- | --- | --- | --- | --- | --- | --- | --- | --- | --- | --- | --- | --- | --- | --- | --- | --- | --- | --- | --- | --- | --- | --- | --- | --- | --- | --- | --- | --- | --- |
| R.DISTINRAQLIDDALNLAR.A | Y | 91.27 | 2111.1335 | 19 | -0.3 | 704.7183 | 3 | 79.44 | 5 | F5:25581 | Fraction\_5\_23092022.raw |  |  |  | 2.0669E5 | 4.0594E5 | 2.772E6 |  | 3 | 0 | 0 | 0 | 1 | 1 | 1 | 0 | 669 | 687 |  |  | PEAKS DB |
| R.TVQQAVEQAEANIK.W | Y | 83.62 | 1527.7893 | 14 | 0.7 | 764.9025 | 2 | 53.92 | 5 | F5:14133 | Fraction\_5\_23092022.raw |  |  |  | 1.2796E6 | 7.1371E5 |  |  | 4 | 0 | 0 | 0 | 2 | 2 | 0 | 0 | 952 | 965 |  |  | PEAKS DB |
| K.FVGQLNDYLQGFYR.S | Y | 81.13 | 1718.8416 | 14 | -0.4 | 860.4277 | 2 | 77.39 | 4 | F4:24889 | Fraction\_4\_23092022.raw |  |  | 8.706E5 | 1.509E6 | 1.2277E5 | 4.5552E5 |  | 4 | 0 | 0 | 1 | 1 | 1 | 1 | 0 | 196 | 209 |  |  | PEAKS DB |
| R.AQLIDDALNLAR.A | Y | 77.33 | 1311.7146 | 12 | 0.3 | 656.8647 | 2 | 68.07 | 5 | F5:20470 | Fraction\_5\_23092022.raw |  |  |  | 9.1366E5 | 2.2643E6 | 1.4183E7 | 2.1683E6 | 4 | 0 | 0 | 0 | 1 | 1 | 1 | 1 | 676 | 687 |  |  | PEAKS DB |
| K.DLIDFITENKDELGSATR.T | Y | 77.32 | 2036.0061 | 18 | -0.5 | 679.6757 | 3 | 77.19 | 5 | F5:24575 | Fraction\_5\_23092022.raw |  |  |  | 1.1819E6 | 4.3715E5 |  |  | 2 | 0 | 0 | 0 | 1 | 1 | 0 | 0 | 934 | 951 |  |  | PEAKS DB |
| R.WIATTQFQATDAR.R | Y | 71.57 | 1507.7419 | 13 | 0.4 | 754.8785 | 2 | 50.93 | 4 | F4:13047 | Fraction\_4\_23092022.raw |  |  |  | 9.3696E5 |  |  |  | 1 | 0 | 0 | 0 | 1 | 0 | 0 | 0 | 220 | 232 |  |  | PEAKS DB |
| R.KM(+15.99)PDPDTNNPISPNLK.S | Y | 66.18 | 1795.8774 | 16 | 1.4 | 599.6339 | 3 | 34.19 | 4 | F4:5844 | Fraction\_4\_23092022.raw |  |  |  | 2.4364E5 |  |  |  | 2 | 0 | 0 | 0 | 2 | 0 | 0 | 0 | 794 | 809 | Oxidation (M) | M2:Oxidation (M):1000.00 | PEAKS DB |
| R.VNYDRTNWQLIIK.Q | Y | 61.26 | 1661.8889 | 13 | -0.4 | 554.9700 | 3 | 56.29 | 6 | F6:15899 | Fraction\_6\_23092022.raw |  |  |  |  |  | 4.1501E5 |  | 1 | 0 | 0 | 0 | 0 | 0 | 1 | 0 | 648 | 660 |  |  | PEAKS DB |
| R.LREYFGTSLVLINNIVK.S | Y | 60.64 | 1978.1251 | 17 | 0.8 | 660.3828 | 3 | 75.86 | 4 | F4:24204 | Fraction\_4\_23092022.raw |  |  |  | 3.3605E5 |  |  |  | 1 | 0 | 0 | 0 | 1 | 0 | 0 | 0 | 904 | 920 |  |  | PEAKS DB |
| R.YLNTNVGSEKDLLLQALGC(+57.02)SR.E | Y | 52.74 | 2350.1951 | 21 | -0.1 | 784.4055 | 3 | 70.62 | 4 | F4:21896 | Fraction\_4\_23092022.raw |  |  |  | 8.0462E4 |  |  |  | 1 | 0 | 0 | 0 | 1 | 0 | 0 | 0 | 832 | 852 | Carbamidomethylation | C19:Carbamidomethylation:1000.00 | PEAKS DB |
| R.NYLNGDATLTQER.F | Y | 52.50 | 1493.7111 | 13 | 0.5 | 747.8632 | 2 | 41.93 | 6 | F6:9406 | Fraction\_6\_23092022.raw |  |  |  |  |  | 7.4213E4 |  | 1 | 0 | 0 | 0 | 0 | 0 | 1 | 0 | 564 | 576 |  |  | PEAKS DB |
| total 11 peptides |  |  |  |  |  |  |  |  |  |  |  |  |  |  |  |  |  |  |  |  |  |  |  |  |  |  |  |  |  |  |  |
| --- | --- | --- | --- | --- | --- | --- | --- | --- | --- | --- | --- | --- | --- | --- | --- | --- | --- | --- | --- | --- | --- | --- | --- | --- | --- | --- | --- | --- | --- | --- | --- |

TRINITY\_DN1113\_c0\_g1\_i53.p1

back to list

  

| Protein Coverage
| Supporting Peptides
|

Protein Coverage:

Supporting Peptides:

| Peptide | Uniq | -10lgP | Mass | Length | ppm | m/z | z | RT | Fraction | Scan | Source File | Area F1 | Area F2 | Area F3 | Area F4 | Area F5 | Area F6 | Area F7 | #Feature | #Feature F1 | #Feature F2 | #Feature F3 | #Feature F4 | #Feature F5 | #Feature F6 | #Feature F7 | Start | End | PTM | AScore | Found By |
| --- | --- | --- | --- | --- | --- | --- | --- | --- | --- | --- | --- | --- | --- | --- | --- | --- | --- | --- | --- | --- | --- | --- | --- | --- | --- | --- | --- | --- | --- | --- | --- |
| R.DISTINRAQLIDDALNLAR.A | Y | 91.27 | 2111.1335 | 19 | -0.3 | 704.7183 | 3 | 79.44 | 5 | F5:25581 | Fraction\_5\_23092022.raw |  |  |  | 2.0669E5 | 4.0594E5 | 2.772E6 |  | 3 | 0 | 0 | 0 | 1 | 1 | 1 | 0 | 669 | 687 |  |  | PEAKS DB |
| R.TVQQAVEQAEANIK.W | Y | 83.62 | 1527.7893 | 14 | 0.7 | 764.9025 | 2 | 53.92 | 5 | F5:14133 | Fraction\_5\_23092022.raw |  |  |  | 1.2796E6 | 7.1371E5 |  |  | 4 | 0 | 0 | 0 | 2 | 2 | 0 | 0 | 952 | 965 |  |  | PEAKS DB |
| K.FVGQLNDYLQGFYR.S | Y | 81.13 | 1718.8416 | 14 | -0.4 | 860.4277 | 2 | 77.39 | 4 | F4:24889 | Fraction\_4\_23092022.raw |  |  | 8.706E5 | 1.509E6 | 1.2277E5 | 4.5552E5 |  | 4 | 0 | 0 | 1 | 1 | 1 | 1 | 0 | 196 | 209 |  |  | PEAKS DB |
| R.AQLIDDALNLAR.A | Y | 77.33 | 1311.7146 | 12 | 0.3 | 656.8647 | 2 | 68.07 | 5 | F5:20470 | Fraction\_5\_23092022.raw |  |  |  | 9.1366E5 | 2.2643E6 | 1.4183E7 | 2.1683E6 | 4 | 0 | 0 | 0 | 1 | 1 | 1 | 1 | 676 | 687 |  |  | PEAKS DB |
| K.DLIDFITENKDELGSATR.T | Y | 77.32 | 2036.0061 | 18 | -0.5 | 679.6757 | 3 | 77.19 | 5 | F5:24575 | Fraction\_5\_23092022.raw |  |  |  | 1.1819E6 | 4.3715E5 |  |  | 2 | 0 | 0 | 0 | 1 | 1 | 0 | 0 | 934 | 951 |  |  | PEAKS DB |
| R.WIATTQFQATDAR.R | Y | 71.57 | 1507.7419 | 13 | 0.4 | 754.8785 | 2 | 50.93 | 4 | F4:13047 | Fraction\_4\_23092022.raw |  |  |  | 9.3696E5 |  |  |  | 1 | 0 | 0 | 0 | 1 | 0 | 0 | 0 | 220 | 232 |  |  | PEAKS DB |
| R.KM(+15.99)PDPDTNNPISPNLK.S | Y | 66.18 | 1795.8774 | 16 | 1.4 | 599.6339 | 3 | 34.19 | 4 | F4:5844 | Fraction\_4\_23092022.raw |  |  |  | 2.4364E5 |  |  |  | 2 | 0 | 0 | 0 | 2 | 0 | 0 | 0 | 794 | 809 | Oxidation (M) | M2:Oxidation (M):1000.00 | PEAKS DB |
| R.VNYDRTNWQLIIK.Q | Y | 61.26 | 1661.8889 | 13 | -0.4 | 554.9700 | 3 | 56.29 | 6 | F6:15899 | Fraction\_6\_23092022.raw |  |  |  |  |  | 4.1501E5 |  | 1 | 0 | 0 | 0 | 0 | 0 | 1 | 0 | 648 | 660 |  |  | PEAKS DB |
| R.LREYFGTSLVLINNIVK.S | Y | 60.64 | 1978.1251 | 17 | 0.8 | 660.3828 | 3 | 75.86 | 4 | F4:24204 | Fraction\_4\_23092022.raw |  |  |  | 3.3605E5 |  |  |  | 1 | 0 | 0 | 0 | 1 | 0 | 0 | 0 | 904 | 920 |  |  | PEAKS DB |
| R.YLNTNVGSEKDLLLQALGC(+57.02)SR.E | Y | 52.74 | 2350.1951 | 21 | -0.1 | 784.4055 | 3 | 70.62 | 4 | F4:21896 | Fraction\_4\_23092022.raw |  |  |  | 8.0462E4 |  |  |  | 1 | 0 | 0 | 0 | 1 | 0 | 0 | 0 | 832 | 852 | Carbamidomethylation | C19:Carbamidomethylation:1000.00 | PEAKS DB |
| R.NYLNGDATLTQER.F | Y | 52.50 | 1493.7111 | 13 | 0.5 | 747.8632 | 2 | 41.93 | 6 | F6:9406 | Fraction\_6\_23092022.raw |  |  |  |  |  | 7.4213E4 |  | 1 | 0 | 0 | 0 | 0 | 0 | 1 | 0 | 564 | 576 |  |  | PEAKS DB |
| total 11 peptides |  |  |  |  |  |  |  |  |  |  |  |  |  |  |  |  |  |  |  |  |  |  |  |  |  |  |  |  |  |  |  |
| --- | --- | --- | --- | --- | --- | --- | --- | --- | --- | --- | --- | --- | --- | --- | --- | --- | --- | --- | --- | --- | --- | --- | --- | --- | --- | --- | --- | --- | --- | --- | --- |

TRINITY\_DN1113\_c0\_g1\_i73.p1

back to list

  

| Protein Coverage
| Supporting Peptides
|

Protein Coverage:

Supporting Peptides:

| Peptide | Uniq | -10lgP | Mass | Length | ppm | m/z | z | RT | Fraction | Scan | Source File | Area F1 | Area F2 | Area F3 | Area F4 | Area F5 | Area F6 | Area F7 | #Feature | #Feature F1 | #Feature F2 | #Feature F3 | #Feature F4 | #Feature F5 | #Feature F6 | #Feature F7 | Start | End | PTM | AScore | Found By |
| --- | --- | --- | --- | --- | --- | --- | --- | --- | --- | --- | --- | --- | --- | --- | --- | --- | --- | --- | --- | --- | --- | --- | --- | --- | --- | --- | --- | --- | --- | --- | --- |
| R.DISTINRAQLIDDALNLAR.A | Y | 91.27 | 2111.1335 | 19 | -0.3 | 704.7183 | 3 | 79.44 | 5 | F5:25581 | Fraction\_5\_23092022.raw |  |  |  | 2.0669E5 | 4.0594E5 | 2.772E6 |  | 3 | 0 | 0 | 0 | 1 | 1 | 1 | 0 | 669 | 687 |  |  | PEAKS DB |
| R.TVQQAVEQAEANIK.W | Y | 83.62 | 1527.7893 | 14 | 0.7 | 764.9025 | 2 | 53.92 | 5 | F5:14133 | Fraction\_5\_23092022.raw |  |  |  | 1.2796E6 | 7.1371E5 |  |  | 4 | 0 | 0 | 0 | 2 | 2 | 0 | 0 | 952 | 965 |  |  | PEAKS DB |
| K.FVGQLNDYLQGFYR.S | Y | 81.13 | 1718.8416 | 14 | -0.4 | 860.4277 | 2 | 77.39 | 4 | F4:24889 | Fraction\_4\_23092022.raw |  |  | 8.706E5 | 1.509E6 | 1.2277E5 | 4.5552E5 |  | 4 | 0 | 0 | 1 | 1 | 1 | 1 | 0 | 196 | 209 |  |  | PEAKS DB |
| R.AQLIDDALNLAR.A | Y | 77.33 | 1311.7146 | 12 | 0.3 | 656.8647 | 2 | 68.07 | 5 | F5:20470 | Fraction\_5\_23092022.raw |  |  |  | 9.1366E5 | 2.2643E6 | 1.4183E7 | 2.1683E6 | 4 | 0 | 0 | 0 | 1 | 1 | 1 | 1 | 676 | 687 |  |  | PEAKS DB |
| K.DLIDFITENKDELGSATR.T | Y | 77.32 | 2036.0061 | 18 | -0.5 | 679.6757 | 3 | 77.19 | 5 | F5:24575 | Fraction\_5\_23092022.raw |  |  |  | 1.1819E6 | 4.3715E5 |  |  | 2 | 0 | 0 | 0 | 1 | 1 | 0 | 0 | 934 | 951 |  |  | PEAKS DB |
| R.WIATTQFQATDAR.R | Y | 71.57 | 1507.7419 | 13 | 0.4 | 754.8785 | 2 | 50.93 | 4 | F4:13047 | Fraction\_4\_23092022.raw |  |  |  | 9.3696E5 |  |  |  | 1 | 0 | 0 | 0 | 1 | 0 | 0 | 0 | 220 | 232 |  |  | PEAKS DB |
| R.KM(+15.99)PDPDTNNPISPNLK.S | Y | 66.18 | 1795.8774 | 16 | 1.4 | 599.6339 | 3 | 34.19 | 4 | F4:5844 | Fraction\_4\_23092022.raw |  |  |  | 2.4364E5 |  |  |  | 2 | 0 | 0 | 0 | 2 | 0 | 0 | 0 | 794 | 809 | Oxidation (M) | M2:Oxidation (M):1000.00 | PEAKS DB |
| R.VNYDRTNWQLIIK.Q | Y | 61.26 | 1661.8889 | 13 | -0.4 | 554.9700 | 3 | 56.29 | 6 | F6:15899 | Fraction\_6\_23092022.raw |  |  |  |  |  | 4.1501E5 |  | 1 | 0 | 0 | 0 | 0 | 0 | 1 | 0 | 648 | 660 |  |  | PEAKS DB |
| R.LREYFGTSLVLINNIVK.S | Y | 60.64 | 1978.1251 | 17 | 0.8 | 660.3828 | 3 | 75.86 | 4 | F4:24204 | Fraction\_4\_23092022.raw |  |  |  | 3.3605E5 |  |  |  | 1 | 0 | 0 | 0 | 1 | 0 | 0 | 0 | 904 | 920 |  |  | PEAKS DB |
| R.YLNTNVGSEKDLLLQALGC(+57.02)SR.E | Y | 52.74 | 2350.1951 | 21 | -0.1 | 784.4055 | 3 | 70.62 | 4 | F4:21896 | Fraction\_4\_23092022.raw |  |  |  | 8.0462E4 |  |  |  | 1 | 0 | 0 | 0 | 1 | 0 | 0 | 0 | 832 | 852 | Carbamidomethylation | C19:Carbamidomethylation:1000.00 | PEAKS DB |
| R.NYLNGDATLTQER.F | Y | 52.50 | 1493.7111 | 13 | 0.5 | 747.8632 | 2 | 41.93 | 6 | F6:9406 | Fraction\_6\_23092022.raw |  |  |  |  |  | 7.4213E4 |  | 1 | 0 | 0 | 0 | 0 | 0 | 1 | 0 | 564 | 576 |  |  | PEAKS DB |
| total 11 peptides |  |  |  |  |  |  |  |  |  |  |  |  |  |  |  |  |  |  |  |  |  |  |  |  |  |  |  |  |  |  |  |
| --- | --- | --- | --- | --- | --- | --- | --- | --- | --- | --- | --- | --- | --- | --- | --- | --- | --- | --- | --- | --- | --- | --- | --- | --- | --- | --- | --- | --- | --- | --- | --- |

TRINITY\_DN1113\_c0\_g1\_i6.p1

back to list

  

| Protein Coverage
| Supporting Peptides
|

Protein Coverage:

Supporting Peptides:

| Peptide | Uniq | -10lgP | Mass | Length | ppm | m/z | z | RT | Fraction | Scan | Source File | Area F1 | Area F2 | Area F3 | Area F4 | Area F5 | Area F6 | Area F7 | #Feature | #Feature F1 | #Feature F2 | #Feature F3 | #Feature F4 | #Feature F5 | #Feature F6 | #Feature F7 | Start | End | PTM | AScore | Found By |
| --- | --- | --- | --- | --- | --- | --- | --- | --- | --- | --- | --- | --- | --- | --- | --- | --- | --- | --- | --- | --- | --- | --- | --- | --- | --- | --- | --- | --- | --- | --- | --- |
| R.DISTINRAQLIDDALNLAR.A | Y | 91.27 | 2111.1335 | 19 | -0.3 | 704.7183 | 3 | 79.44 | 5 | F5:25581 | Fraction\_5\_23092022.raw |  |  |  | 2.0669E5 | 4.0594E5 | 2.772E6 |  | 3 | 0 | 0 | 0 | 1 | 1 | 1 | 0 | 669 | 687 |  |  | PEAKS DB |
| R.TVQQAVEQAEANIK.W | Y | 83.62 | 1527.7893 | 14 | 0.7 | 764.9025 | 2 | 53.92 | 5 | F5:14133 | Fraction\_5\_23092022.raw |  |  |  | 1.2796E6 | 7.1371E5 |  |  | 4 | 0 | 0 | 0 | 2 | 2 | 0 | 0 | 952 | 965 |  |  | PEAKS DB |
| K.FVGQLNDYLQGFYR.S | Y | 81.13 | 1718.8416 | 14 | -0.4 | 860.4277 | 2 | 77.39 | 4 | F4:24889 | Fraction\_4\_23092022.raw |  |  | 8.706E5 | 1.509E6 | 1.2277E5 | 4.5552E5 |  | 4 | 0 | 0 | 1 | 1 | 1 | 1 | 0 | 196 | 209 |  |  | PEAKS DB |
| R.AQLIDDALNLAR.A | Y | 77.33 | 1311.7146 | 12 | 0.3 | 656.8647 | 2 | 68.07 | 5 | F5:20470 | Fraction\_5\_23092022.raw |  |  |  | 9.1366E5 | 2.2643E6 | 1.4183E7 | 2.1683E6 | 4 | 0 | 0 | 0 | 1 | 1 | 1 | 1 | 676 | 687 |  |  | PEAKS DB |
| K.DLIDFITENKDELGSATR.T | Y | 77.32 | 2036.0061 | 18 | -0.5 | 679.6757 | 3 | 77.19 | 5 | F5:24575 | Fraction\_5\_23092022.raw |  |  |  | 1.1819E6 | 4.3715E5 |  |  | 2 | 0 | 0 | 0 | 1 | 1 | 0 | 0 | 934 | 951 |  |  | PEAKS DB |
| R.WIATTQFQATDAR.R | Y | 71.57 | 1507.7419 | 13 | 0.4 | 754.8785 | 2 | 50.93 | 4 | F4:13047 | Fraction\_4\_23092022.raw |  |  |  | 9.3696E5 |  |  |  | 1 | 0 | 0 | 0 | 1 | 0 | 0 | 0 | 220 | 232 |  |  | PEAKS DB |
| R.KM(+15.99)PDPDTNNPISPNLK.S | Y | 66.18 | 1795.8774 | 16 | 1.4 | 599.6339 | 3 | 34.19 | 4 | F4:5844 | Fraction\_4\_23092022.raw |  |  |  | 2.4364E5 |  |  |  | 2 | 0 | 0 | 0 | 2 | 0 | 0 | 0 | 794 | 809 | Oxidation (M) | M2:Oxidation (M):1000.00 | PEAKS DB |
| R.VNYDRTNWQLIIK.Q | Y | 61.26 | 1661.8889 | 13 | -0.4 | 554.9700 | 3 | 56.29 | 6 | F6:15899 | Fraction\_6\_23092022.raw |  |  |  |  |  | 4.1501E5 |  | 1 | 0 | 0 | 0 | 0 | 0 | 1 | 0 | 648 | 660 |  |  | PEAKS DB |
| R.LREYFGTSLVLINNIVK.S | Y | 60.64 | 1978.1251 | 17 | 0.8 | 660.3828 | 3 | 75.86 | 4 | F4:24204 | Fraction\_4\_23092022.raw |  |  |  | 3.3605E5 |  |  |  | 1 | 0 | 0 | 0 | 1 | 0 | 0 | 0 | 904 | 920 |  |  | PEAKS DB |
| R.YLNTNVGSEKDLLLQALGC(+57.02)SR.E | Y | 52.74 | 2350.1951 | 21 | -0.1 | 784.4055 | 3 | 70.62 | 4 | F4:21896 | Fraction\_4\_23092022.raw |  |  |  | 8.0462E4 |  |  |  | 1 | 0 | 0 | 0 | 1 | 0 | 0 | 0 | 832 | 852 | Carbamidomethylation | C19:Carbamidomethylation:1000.00 | PEAKS DB |
| R.NYLNGDATLTQER.F | Y | 52.50 | 1493.7111 | 13 | 0.5 | 747.8632 | 2 | 41.93 | 6 | F6:9406 | Fraction\_6\_23092022.raw |  |  |  |  |  | 7.4213E4 |  | 1 | 0 | 0 | 0 | 0 | 0 | 1 | 0 | 564 | 576 |  |  | PEAKS DB |
| total 11 peptides |  |  |  |  |  |  |  |  |  |  |  |  |  |  |  |  |  |  |  |  |  |  |  |  |  |  |  |  |  |  |  |
| --- | --- | --- | --- | --- | --- | --- | --- | --- | --- | --- | --- | --- | --- | --- | --- | --- | --- | --- | --- | --- | --- | --- | --- | --- | --- | --- | --- | --- | --- | --- | --- |

TRINITY\_DN1113\_c0\_g1\_i12.p1

back to list

  

| Protein Coverage
| Supporting Peptides
|

Protein Coverage:

Supporting Peptides:

| Peptide | Uniq | -10lgP | Mass | Length | ppm | m/z | z | RT | Fraction | Scan | Source File | Area F1 | Area F2 | Area F3 | Area F4 | Area F5 | Area F6 | Area F7 | #Feature | #Feature F1 | #Feature F2 | #Feature F3 | #Feature F4 | #Feature F5 | #Feature F6 | #Feature F7 | Start | End | PTM | AScore | Found By |
| --- | --- | --- | --- | --- | --- | --- | --- | --- | --- | --- | --- | --- | --- | --- | --- | --- | --- | --- | --- | --- | --- | --- | --- | --- | --- | --- | --- | --- | --- | --- | --- |
| R.DISTINRAQLIDDALNLAR.A | Y | 91.27 | 2111.1335 | 19 | -0.3 | 704.7183 | 3 | 79.44 | 5 | F5:25581 | Fraction\_5\_23092022.raw |  |  |  | 2.0669E5 | 4.0594E5 | 2.772E6 |  | 3 | 0 | 0 | 0 | 1 | 1 | 1 | 0 | 669 | 687 |  |  | PEAKS DB |
| R.TVQQAVEQAEANIK.W | Y | 83.62 | 1527.7893 | 14 | 0.7 | 764.9025 | 2 | 53.92 | 5 | F5:14133 | Fraction\_5\_23092022.raw |  |  |  | 1.2796E6 | 7.1371E5 |  |  | 4 | 0 | 0 | 0 | 2 | 2 | 0 | 0 | 952 | 965 |  |  | PEAKS DB |
| K.FVGQLNDYLQGFYR.S | Y | 81.13 | 1718.8416 | 14 | -0.4 | 860.4277 | 2 | 77.39 | 4 | F4:24889 | Fraction\_4\_23092022.raw |  |  | 8.706E5 | 1.509E6 | 1.2277E5 | 4.5552E5 |  | 4 | 0 | 0 | 1 | 1 | 1 | 1 | 0 | 196 | 209 |  |  | PEAKS DB |
| R.AQLIDDALNLAR.A | Y | 77.33 | 1311.7146 | 12 | 0.3 | 656.8647 | 2 | 68.07 | 5 | F5:20470 | Fraction\_5\_23092022.raw |  |  |  | 9.1366E5 | 2.2643E6 | 1.4183E7 | 2.1683E6 | 4 | 0 | 0 | 0 | 1 | 1 | 1 | 1 | 676 | 687 |  |  | PEAKS DB |
| K.DLIDFITENKDELGSATR.T | Y | 77.32 | 2036.0061 | 18 | -0.5 | 679.6757 | 3 | 77.19 | 5 | F5:24575 | Fraction\_5\_23092022.raw |  |  |  | 1.1819E6 | 4.3715E5 |  |  | 2 | 0 | 0 | 0 | 1 | 1 | 0 | 0 | 934 | 951 |  |  | PEAKS DB |
| R.WIATTQFQATDAR.R | Y | 71.57 | 1507.7419 | 13 | 0.4 | 754.8785 | 2 | 50.93 | 4 | F4:13047 | Fraction\_4\_23092022.raw |  |  |  | 9.3696E5 |  |  |  | 1 | 0 | 0 | 0 | 1 | 0 | 0 | 0 | 220 | 232 |  |  | PEAKS DB |
| R.KM(+15.99)PDPDTNNPISPNLK.S | Y | 66.18 | 1795.8774 | 16 | 1.4 | 599.6339 | 3 | 34.19 | 4 | F4:5844 | Fraction\_4\_23092022.raw |  |  |  | 2.4364E5 |  |  |  | 2 | 0 | 0 | 0 | 2 | 0 | 0 | 0 | 794 | 809 | Oxidation (M) | M2:Oxidation (M):1000.00 | PEAKS DB |
| R.VNYDRTNWQLIIK.Q | Y | 61.26 | 1661.8889 | 13 | -0.4 | 554.9700 | 3 | 56.29 | 6 | F6:15899 | Fraction\_6\_23092022.raw |  |  |  |  |  | 4.1501E5 |  | 1 | 0 | 0 | 0 | 0 | 0 | 1 | 0 | 648 | 660 |  |  | PEAKS DB |
| R.LREYFGTSLVLINNIVK.S | Y | 60.64 | 1978.1251 | 17 | 0.8 | 660.3828 | 3 | 75.86 | 4 | F4:24204 | Fraction\_4\_23092022.raw |  |  |  | 3.3605E5 |  |  |  | 1 | 0 | 0 | 0 | 1 | 0 | 0 | 0 | 904 | 920 |  |  | PEAKS DB |
| R.YLNTNVGSEKDLLLQALGC(+57.02)SR.E | Y | 52.74 | 2350.1951 | 21 | -0.1 | 784.4055 | 3 | 70.62 | 4 | F4:21896 | Fraction\_4\_23092022.raw |  |  |  | 8.0462E4 |  |  |  | 1 | 0 | 0 | 0 | 1 | 0 | 0 | 0 | 832 | 852 | Carbamidomethylation | C19:Carbamidomethylation:1000.00 | PEAKS DB |
| R.NYLNGDATLTQER.F | Y | 52.50 | 1493.7111 | 13 | 0.5 | 747.8632 | 2 | 41.93 | 6 | F6:9406 | Fraction\_6\_23092022.raw |  |  |  |  |  | 7.4213E4 |  | 1 | 0 | 0 | 0 | 0 | 0 | 1 | 0 | 564 | 576 |  |  | PEAKS DB |
| total 11 peptides |  |  |  |  |  |  |  |  |  |  |  |  |  |  |  |  |  |  |  |  |  |  |  |  |  |  |  |  |  |  |  |
| --- | --- | --- | --- | --- | --- | --- | --- | --- | --- | --- | --- | --- | --- | --- | --- | --- | --- | --- | --- | --- | --- | --- | --- | --- | --- | --- | --- | --- | --- | --- | --- |

TRINITY\_DN1342\_c0\_g1\_i29.p1

back to list

  

| Protein Coverage
| Supporting Peptides
|

Protein Coverage:

Supporting Peptides:

| Peptide | Uniq | -10lgP | Mass | Length | ppm | m/z | z | RT | Fraction | Scan | Source File | Area F1 | Area F2 | Area F3 | Area F4 | Area F5 | Area F6 | Area F7 | #Feature | #Feature F1 | #Feature F2 | #Feature F3 | #Feature F4 | #Feature F5 | #Feature F6 | #Feature F7 | Start | End | PTM | AScore | Found By |
| --- | --- | --- | --- | --- | --- | --- | --- | --- | --- | --- | --- | --- | --- | --- | --- | --- | --- | --- | --- | --- | --- | --- | --- | --- | --- | --- | --- | --- | --- | --- | --- |
| R.DPNRTPFQWSYVR.N | N | 92.58 | 1664.8059 | 13 | -0.6 | 555.9423 | 3 | 57.76 | 1 | F1:14997 | Fraction\_1\_23092022r.raw | 2.4455E5 | 9.7444E5 |  |  |  |  |  | 2 | 1 | 1 | 0 | 0 | 0 | 0 | 0 | 432 | 444 |  |  | PEAKS DB |
| K.YYQYGAHIPFNFK.L | Y | 84.11 | 1646.7881 | 13 | 0.1 | 824.4014 | 2 | 58.06 | 2 | F2:15314 | Fraction\_2\_23092022.raw | 1.0775E6 | 9.8453E6 | 4.1487E5 | 1.2391E5 | 1.2335E5 |  |  | 6 | 1 | 2 | 1 | 1 | 1 | 0 | 0 | 315 | 327 |  |  | PEAKS DB |
| R.DYTYLDHIYTKDDPR.T | N | 77.26 | 1913.8795 | 15 | 0.3 | 638.9673 | 3 | 47.98 | 5 | F5:11468 | Fraction\_5\_23092022.raw | 6.7442E5 | 6.2862E6 |  | 2.496E5 | 7.4735E5 |  |  | 5 | 1 | 2 | 0 | 1 | 1 | 0 | 0 | 261 | 275 |  |  | PEAKS DB |
| R.DYTYLDHIYTKDDPRTYELVK.S | N | 76.51 | 2647.2805 | 21 | 1.1 | 662.8281 | 4 | 57.24 | 2 | F2:14940 | Fraction\_2\_23092022.raw |  | 5.1388E6 | 1.891E5 |  | 1.9821E5 |  |  | 4 | 0 | 2 | 1 | 0 | 1 | 0 | 0 | 261 | 281 |  |  | PEAKS DB |
| R.DYTYLDHIYTK.D | N | 74.29 | 1430.6718 | 11 | 0.2 | 716.3433 | 2 | 52.27 | 5 | F5:13434 | Fraction\_5\_23092022.raw |  | 1.8665E5 |  |  | 1.9599E5 |  |  | 3 | 0 | 1 | 0 | 0 | 2 | 0 | 0 | 261 | 271 |  |  | PEAKS DB |
| K.NAVFYQIYPR.S | N | 73.17 | 1269.6505 | 10 | 0.3 | 635.8327 | 2 | 57.87 | 7 | F7:20277 | Fraction\_7\_23092022.raw | 1.1663E7 | 9.8135E7 | 2.3489E6 | 2.1542E5 | 2.417E5 | 9.3508E7 | 6.8859E7 | 8 | 1 | 1 | 1 | 1 | 1 | 2 | 1 | 42 | 51 |  |  | PEAKS DB |
| R.SFMDSNDDGVGDLK.G | N | 66.57 | 1498.6245 | 14 | 0.6 | 750.3200 | 2 | 52.27 | 1 | F1:12582 | Fraction\_1\_23092022r.raw | 1.6384E5 |  |  |  |  |  |  | 1 | 1 | 0 | 0 | 0 | 0 | 0 | 0 | 52 | 65 |  |  | PEAKS DB |
| R.TPFQWSYVR.N | N | 65.12 | 1182.5822 | 9 | 1.5 | 592.2993 | 2 | 59.87 | 1 | F1:15973 | Fraction\_1\_23092022r.raw | 4.492E5 | 6.4111E5 |  |  |  |  |  | 2 | 1 | 1 | 0 | 0 | 0 | 0 | 0 | 436 | 444 |  |  | PEAKS DB |
| R.SFM(+15.99)DSNDDGVGDLK.G | N | 60.49 | 1514.6195 | 14 | -0.3 | 758.3168 | 2 | 39.80 | 6 | F6:8482 | Fraction\_6\_23092022.raw | 4.5737E4 | 1.5252E5 |  |  |  | 3.4969E5 |  | 5 | 1 | 2 | 0 | 0 | 0 | 2 | 0 | 52 | 65 | Oxidation (M) | M3:Oxidation (M):1000.00 | PEAKS DB |
| R.SRDPNRTPFQWSYVR.N | Y | 59.04 | 1907.9391 | 15 | 3.4 | 477.9937 | 4 | 46.49 | 2 | F2:10101 | Fraction\_2\_23092022.raw |  | 6.5811E6 |  |  |  |  |  | 1 | 0 | 1 | 0 | 0 | 0 | 0 | 0 | 430 | 444 |  |  | PEAKS DB |
| R.IDAVPHLFER.Q | N | 55.84 | 1195.6349 | 10 | 1.2 | 399.5527 | 3 | 51.46 | 5 | F5:13030 | Fraction\_5\_23092022.raw |  |  |  |  | 1.2393E5 |  |  | 1 | 0 | 0 | 0 | 0 | 1 | 0 | 0 | 234 | 243 |  |  | PEAKS DB |
| K.GIDGFRIDAVPHLFER.Q | N | 50.68 | 1840.9584 | 16 | 0.0 | 614.6601 | 3 | 68.71 | 2 | F2:20128 | Fraction\_2\_23092022.raw |  | 2.1436E5 |  |  |  |  |  | 1 | 0 | 1 | 0 | 0 | 0 | 0 | 0 | 228 | 243 |  |  | PEAKS DB |
| R.IIEAWIAKTPK.N | Y | 48.69 | 1268.7493 | 11 | 1.0 | 423.9241 | 3 | 46.93 | 2 | F2:10307 | Fraction\_2\_23092022.raw |  | 3.7018E5 |  |  |  |  |  | 1 | 0 | 1 | 0 | 0 | 0 | 0 | 0 | 344 | 354 |  |  | PEAKS DB |
| total 13 peptides |  |  |  |  |  |  |  |  |  |  |  |  |  |  |  |  |  |  |  |  |  |  |  |  |  |  |  |  |  |  |  |
| --- | --- | --- | --- | --- | --- | --- | --- | --- | --- | --- | --- | --- | --- | --- | --- | --- | --- | --- | --- | --- | --- | --- | --- | --- | --- | --- | --- | --- | --- | --- | --- |

TRINITY\_DN2373\_c0\_g1\_i19.p1

back to list

  

| Protein Coverage
| Supporting Peptides
|

Protein Coverage:

Supporting Peptides:

| Peptide | Uniq | -10lgP | Mass | Length | ppm | m/z | z | RT | Fraction | Scan | Source File | Area F1 | Area F2 | Area F3 | Area F4 | Area F5 | Area F6 | Area F7 | #Feature | #Feature F1 | #Feature F2 | #Feature F3 | #Feature F4 | #Feature F5 | #Feature F6 | #Feature F7 | Start | End | PTM | AScore | Found By |
| --- | --- | --- | --- | --- | --- | --- | --- | --- | --- | --- | --- | --- | --- | --- | --- | --- | --- | --- | --- | --- | --- | --- | --- | --- | --- | --- | --- | --- | --- | --- | --- |
| K.ATKELLFHAIDLPGLGYKR.Y | Y | 100.59 | 2141.1997 | 19 | 2.1 | 536.3083 | 4 | 59.56 | 7 | F7:21253 | Fraction\_7\_23092022.raw |  |  |  |  |  |  | 1.1769E6 | 1 | 0 | 0 | 0 | 0 | 0 | 0 | 1 | 558 | 576 |  |  | PEAKS DB |
| K.LYEPISGNYYPVTTK.I | Y | 92.67 | 1743.8719 | 15 | -0.2 | 872.9431 | 2 | 57.03 | 3 | F3:15220 | Fraction\_3\_23092022.raw |  |  | 7.0406E6 |  | 1.724E5 | 5.2852E5 |  | 3 | 0 | 0 | 1 | 0 | 1 | 1 | 0 | 755 | 769 |  |  | PEAKS DB |
| K.YSTYINSEDEFYTDSNGR.E | Y | 86.54 | 2159.8918 | 18 | 0.9 | 1080.9542 | 2 | 53.51 | 3 | F3:13680 | Fraction\_3\_23092022.raw |  |  | 1.1769E5 |  |  |  |  | 1 | 0 | 0 | 1 | 0 | 0 | 0 | 0 | 721 | 738 |  |  | PEAKS DB |
| K.ELLFHAIDLPGLGYKR.Y | Y | 84.99 | 1841.0199 | 16 | 0.6 | 614.6810 | 3 | 69.01 | 2 | F2:20234 | Fraction\_2\_23092022.raw |  | 2.6422E6 |  |  |  |  | 1.3445E7 | 4 | 0 | 2 | 0 | 0 | 0 | 0 | 2 | 561 | 576 |  |  | PEAKS DB |
| R.LLYENIFNNEK.M | Y | 66.36 | 1395.7034 | 11 | 0.5 | 698.8593 | 2 | 63.08 | 5 | F5:18278 | Fraction\_5\_23092022.raw | 1.3169E5 | 7.8026E5 | 2.4406E5 | 3.7341E5 | 1.0101E5 |  |  | 5 | 1 | 1 | 1 | 1 | 1 | 0 | 0 | 449 | 459 |  |  | PEAKS DB |
| K.TVDQYYFGSQSK.I | Y | 56.57 | 1421.6462 | 12 | 0.6 | 711.8308 | 2 | 42.69 | 5 | F5:9150 | Fraction\_5\_23092022.raw |  | 7.6837E4 |  |  | 8.0396E4 |  |  | 2 | 0 | 1 | 0 | 0 | 1 | 0 | 0 | 73 | 84 |  |  | PEAKS DB |
| K.VVSQIIPIHER.V | Y | 53.95 | 1289.7455 | 11 | -0.3 | 430.9223 | 3 | 40.75 | 7 | F7:11098 | Fraction\_7\_23092022.raw |  |  |  |  |  |  | 1.4061E6 | 1 | 0 | 0 | 0 | 0 | 0 | 0 | 1 | 537 | 547 |  |  | PEAKS DB |
| R.LLYENIFNNEKM(+15.99)AAK.S | Y | 51.21 | 1812.9080 | 15 | 0.3 | 605.3101 | 3 | 53.71 | 2 | F2:13370 | Fraction\_2\_23092022.raw |  | 4.5897E5 |  |  |  |  |  | 1 | 0 | 1 | 0 | 0 | 0 | 0 | 0 | 449 | 463 | Oxidation (M) | M12:Oxidation (M):1000.00 | PEAKS DB |
| K.TVDQYYFGSQSKIQK.A | Y | 47.90 | 1790.8839 | 15 | 0.3 | 597.9688 | 3 | 39.55 | 2 | F2:7028 | Fraction\_2\_23092022.raw |  | 8.7729E4 |  |  |  |  |  | 1 | 0 | 1 | 0 | 0 | 0 | 0 | 0 | 73 | 87 |  |  | PEAKS DB |
| K.FSVFTDR.S | Y | 47.84 | 870.4235 | 7 | 0.0 | 436.2191 | 2 | 48.92 | 3 | F3:11591 | Fraction\_3\_23092022.raw |  |  | 6.1699E5 |  |  |  |  | 1 | 0 | 0 | 1 | 0 | 0 | 0 | 0 | 781 | 787 |  |  | PEAKS DB |
| total 10 peptides |  |  |  |  |  |  |  |  |  |  |  |  |  |  |  |  |  |  |  |  |  |  |  |  |  |  |  |  |  |  |  |
| --- | --- | --- | --- | --- | --- | --- | --- | --- | --- | --- | --- | --- | --- | --- | --- | --- | --- | --- | --- | --- | --- | --- | --- | --- | --- | --- | --- | --- | --- | --- | --- |

TRINITY\_DN2228\_c0\_g4\_i1.p1

back to list

  

| Protein Coverage
| Supporting Peptides
|

Protein Coverage:

Supporting Peptides:

| Peptide | Uniq | -10lgP | Mass | Length | ppm | m/z | z | RT | Fraction | Scan | Source File | Area F1 | Area F2 | Area F3 | Area F4 | Area F5 | Area F6 | Area F7 | #Feature | #Feature F1 | #Feature F2 | #Feature F3 | #Feature F4 | #Feature F5 | #Feature F6 | #Feature F7 | Start | End | PTM | AScore | Found By |
| --- | --- | --- | --- | --- | --- | --- | --- | --- | --- | --- | --- | --- | --- | --- | --- | --- | --- | --- | --- | --- | --- | --- | --- | --- | --- | --- | --- | --- | --- | --- | --- |
| R.NVNNLKINKEVSIDLVR.K | Y | 90.06 | 1967.1163 | 17 | 0.6 | 656.7131 | 3 | 48.02 | 5 | F5:11499 | Fraction\_5\_23092022.raw |  |  | 4.9647E5 | 3.6441E6 | 1.812E7 | 1.0791E8 | 8.881E6 | 13 | 0 | 0 | 2 | 2 | 2 | 5 | 2 | 139 | 155 |  |  | PEAKS DB |
| K.ISGGIGVINFNK.W | Y | 82.29 | 1217.6768 | 12 | -0.1 | 609.8456 | 2 | 56.88 | 5 | F5:15472 | Fraction\_5\_23092022.raw |  |  | 8.4174E5 | 2.1313E6 | 9.6524E6 | 7.1056E6 |  | 4 | 0 | 0 | 1 | 1 | 1 | 1 | 0 | 120 | 131 |  |  | PEAKS DB |
| K.KISGGIGVINFNK.W | Y | 79.64 | 1345.7717 | 13 | 0.2 | 449.5980 | 3 | 47.17 | 5 | F5:11168 | Fraction\_5\_23092022.raw |  |  | 3.1909E6 | 6.8845E6 | 2.3022E7 | 1.119E7 | 5.4209E5 | 9 | 0 | 0 | 2 | 2 | 2 | 2 | 1 | 119 | 131 |  |  | PEAKS DB |
| K.FDIKYNSKGNYR.G | Y | 66.49 | 1503.7469 | 12 | 0.7 | 502.2566 | 3 | 29.10 | 7 | F7:5897 | Fraction\_7\_23092022.raw |  |  |  |  |  |  | 8.6251E5 | 2 | 0 | 0 | 0 | 0 | 0 | 0 | 2 | 53 | 64 |  |  | PEAKS DB |
| R.NVNNLKINKEVSIDLVRK.E | Y | 64.50 | 2095.2112 | 18 | 0.7 | 524.8104 | 4 | 41.35 | 6 | F6:9142 | Fraction\_6\_23092022.raw |  |  |  |  | 1.1412E6 | 3.6447E6 | 6.1332E5 | 5 | 0 | 0 | 0 | 0 | 2 | 2 | 1 | 139 | 156 |  |  | PEAKS DB |
| K.YQDVLLPSVYVK.H | Y | 60.27 | 1422.7759 | 12 | 0.5 | 712.3956 | 2 | 66.01 | 5 | F5:19561 | Fraction\_5\_23092022.raw | 1.8277E5 |  | 5.1958E6 | 3.3139E5 | 4.1258E5 | 3.5187E6 |  | 5 | 1 | 0 | 1 | 1 | 1 | 1 | 0 | 235 | 246 |  |  | PEAKS DB |
| K.INKEVSIDLVR.K | Y | 58.65 | 1284.7401 | 11 | -0.6 | 429.2537 | 3 | 41.36 | 7 | F7:11343 | Fraction\_7\_23092022.raw |  |  | 1.0761E7 | 7.7769E6 | 3.5163E7 | 1.1978E8 | 5.5704E6 | 9 | 0 | 0 | 2 | 1 | 2 | 2 | 2 | 145 | 155 |  |  | PEAKS DB |
| K.VLSYWWYAYEDK.M | Y | 56.16 | 1621.7452 | 12 | 0.4 | 811.8802 | 2 | 75.16 | 6 | F6:24324 | Fraction\_6\_23092022.raw |  |  |  |  |  | 2.0996E5 |  | 1 | 0 | 0 | 0 | 0 | 0 | 1 | 0 | 278 | 289 |  |  | PEAKS DB |
| K.WDKSM(+15.99)IETEASNRFEK.S | Y | 55.75 | 1985.9153 | 16 | -0.4 | 497.4859 | 4 | 39.04 | 6 | F6:8127 | Fraction\_6\_23092022.raw |  |  |  |  |  | 1.0503E6 |  | 1 | 0 | 0 | 0 | 0 | 0 | 1 | 0 | 161 | 176 | Oxidation (M) | M5:Oxidation (M):1000.00 | PEAKS DB |
| K.HVLKPEEKIGLVR.G | Y | 52.93 | 1516.9089 | 13 | -0.5 | 380.2343 | 4 | 28.27 | 7 | F7:5567 | Fraction\_7\_23092022.raw |  |  |  |  |  |  | 8.6259E6 | 2 | 0 | 0 | 0 | 0 | 0 | 0 | 2 | 247 | 259 |  |  | PEAKS DB |
| K.WDKSM(+15.99)IETEASNRFEKSAR.I | Y | 52.77 | 2300.0854 | 19 | 0.6 | 576.0290 | 4 | 37.06 | 6 | F6:7252 | Fraction\_6\_23092022.raw |  |  |  |  |  | 6.0704E5 |  | 1 | 0 | 0 | 0 | 0 | 0 | 1 | 0 | 161 | 179 | Oxidation (M) | M5:Oxidation (M):1000.00 | PEAKS DB |
| K.ELDEIYPK.K | N | 50.82 | 1005.5018 | 8 | 0.3 | 503.7584 | 2 | 41.97 | 7 | F7:11626 | Fraction\_7\_23092022.raw |  |  |  | 1.6356E6 | 5.2505E6 | 7.199E5 | 4.2396E5 | 4 | 0 | 0 | 0 | 1 | 1 | 1 | 1 | 111 | 118 |  |  | PEAKS DB |
| K.SM(+15.99)IETEASNRFEKSAR.I | Y | 50.70 | 1870.8843 | 16 | -0.2 | 468.7283 | 4 | 26.49 | 6 | F6:3955 | Fraction\_6\_23092022.raw |  |  |  |  |  | 4.2525E5 |  | 1 | 0 | 0 | 0 | 0 | 0 | 1 | 0 | 164 | 179 | Oxidation (M) | M2:Oxidation (M):1000.00 | PEAKS DB |
| K.INKEVSIDLVRK.E | Y | 47.43 | 1412.8351 | 12 | 0.2 | 471.9524 | 3 | 32.98 | 7 | F7:7590 | Fraction\_7\_23092022.raw |  |  |  |  |  |  | 5.519E5 | 1 | 0 | 0 | 0 | 0 | 0 | 0 | 1 | 145 | 156 |  |  | PEAKS DB |
| K.FDIKYNSK.G | Y | 47.40 | 1013.5182 | 8 | -0.5 | 507.7661 | 2 | 30.18 | 7 | F7:6337 | Fraction\_7\_23092022.raw |  |  |  |  |  |  | 2.0292E6 | 1 | 0 | 0 | 0 | 0 | 0 | 0 | 1 | 53 | 60 |  |  | PEAKS DB |
| total 15 peptides |  |  |  |  |  |  |  |  |  |  |  |  |  |  |  |  |  |  |  |  |  |  |  |  |  |  |  |  |  |  |  |
| --- | --- | --- | --- | --- | --- | --- | --- | --- | --- | --- | --- | --- | --- | --- | --- | --- | --- | --- | --- | --- | --- | --- | --- | --- | --- | --- | --- | --- | --- | --- | --- |

TRINITY\_DN1342\_c0\_g1\_i23.p1

back to list

  

| Protein Coverage
| Supporting Peptides
|

Protein Coverage:

Supporting Peptides:

| Peptide | Uniq | -10lgP | Mass | Length | ppm | m/z | z | RT | Fraction | Scan | Source File | Area F1 | Area F2 | Area F3 | Area F4 | Area F5 | Area F6 | Area F7 | #Feature | #Feature F1 | #Feature F2 | #Feature F3 | #Feature F4 | #Feature F5 | #Feature F6 | #Feature F7 | Start | End | PTM | AScore | Found By |
| --- | --- | --- | --- | --- | --- | --- | --- | --- | --- | --- | --- | --- | --- | --- | --- | --- | --- | --- | --- | --- | --- | --- | --- | --- | --- | --- | --- | --- | --- | --- | --- |
| R.SSDSTATDRDYTYLDHIYTKDDPRTYELVK.S | Y | 88.46 | 3567.6641 | 30 | 0.6 | 892.9238 | 4 | 53.26 | 2 | F2:13133 | Fraction\_2\_23092022.raw | 4.6338E6 | 1.4147E8 |  | 1.1258E6 | 3.1845E6 |  |  | 7 | 1 | 4 | 0 | 1 | 1 | 0 | 0 | 252 | 281 |  |  | PEAKS DB |
| K.GIDGFRIDAVPHLFEREDLHDEPR.S | Y | 80.16 | 2832.3943 | 24 | -0.4 | 473.0728 | 6 | 62.45 | 2 | F2:17317 | Fraction\_2\_23092022.raw |  | 1.0771E7 |  |  |  |  |  | 3 | 0 | 3 | 0 | 0 | 0 | 0 | 0 | 228 | 251 |  |  | PEAKS DB |
| R.DYTYLDHIYTKDDPR.T | N | 77.26 | 1913.8795 | 15 | 0.3 | 638.9673 | 3 | 47.98 | 5 | F5:11468 | Fraction\_5\_23092022.raw | 6.7442E5 | 6.2862E6 |  | 2.496E5 | 7.4735E5 |  |  | 5 | 1 | 2 | 0 | 1 | 1 | 0 | 0 | 261 | 275 |  |  | PEAKS DB |
| R.DYTYLDHIYTKDDPRTYELVK.S | N | 76.51 | 2647.2805 | 21 | 1.1 | 662.8281 | 4 | 57.24 | 2 | F2:14940 | Fraction\_2\_23092022.raw |  | 5.1388E6 | 1.891E5 |  | 1.9821E5 |  |  | 4 | 0 | 2 | 1 | 0 | 1 | 0 | 0 | 261 | 281 |  |  | PEAKS DB |
| R.DYTYLDHIYTK.D | N | 74.29 | 1430.6718 | 11 | 0.2 | 716.3433 | 2 | 52.27 | 5 | F5:13434 | Fraction\_5\_23092022.raw |  | 1.8665E5 |  |  | 1.9599E5 |  |  | 3 | 0 | 1 | 0 | 0 | 2 | 0 | 0 | 261 | 271 |  |  | PEAKS DB |
| K.NAVFYQIYPR.S | N | 73.17 | 1269.6505 | 10 | 0.3 | 635.8327 | 2 | 57.87 | 7 | F7:20277 | Fraction\_7\_23092022.raw | 1.1663E7 | 9.8135E7 | 2.3489E6 | 2.1542E5 | 2.417E5 | 9.3508E7 | 6.8859E7 | 8 | 1 | 1 | 1 | 1 | 1 | 2 | 1 | 42 | 51 |  |  | PEAKS DB |
| R.IDAVPHLFEREDLHDEPR.S | Y | 68.72 | 2187.0708 | 18 | 0.7 | 438.4218 | 5 | 47.06 | 2 | F2:10334 | Fraction\_2\_23092022.raw | 7.6093E5 | 3.8278E6 |  |  | 4.9871E5 |  |  | 6 | 2 | 2 | 0 | 0 | 2 | 0 | 0 | 234 | 251 |  |  | PEAKS DB |
| R.SSDSTATDRDYTYLDHIYTK.D | Y | 67.49 | 2351.0554 | 20 | 0.1 | 588.7712 | 4 | 46.79 | 2 | F2:10235 | Fraction\_2\_23092022.raw | 5.662E5 | 3.4895E6 |  |  | 3.6296E5 |  |  | 4 | 2 | 1 | 0 | 0 | 1 | 0 | 0 | 252 | 271 |  |  | PEAKS DB |
| R.SFMDSNDDGVGDLK.G | N | 66.57 | 1498.6245 | 14 | 0.6 | 750.3200 | 2 | 52.27 | 1 | F1:12582 | Fraction\_1\_23092022r.raw | 1.6384E5 |  |  |  |  |  |  | 1 | 1 | 0 | 0 | 0 | 0 | 0 | 0 | 52 | 65 |  |  | PEAKS DB |
| R.SSDSTATDRDYTYLDHIYTKDDPR.T | Y | 65.52 | 2834.2629 | 24 | -0.5 | 945.7611 | 3 | 44.63 | 2 | F2:9256 | Fraction\_2\_23092022.raw |  | 1.5598E8 |  |  | 2.8075E6 |  |  | 5 | 0 | 4 | 0 | 0 | 1 | 0 | 0 | 252 | 275 |  |  | PEAKS DB |
| R.SFM(+15.99)DSNDDGVGDLK.G | N | 60.49 | 1514.6195 | 14 | -0.3 | 758.3168 | 2 | 39.80 | 6 | F6:8482 | Fraction\_6\_23092022.raw | 4.5737E4 | 1.5252E5 |  |  |  | 3.4969E5 |  | 5 | 1 | 2 | 0 | 0 | 0 | 2 | 0 | 52 | 65 | Oxidation (M) | M3:Oxidation (M):1000.00 | PEAKS DB |
| R.IDAVPHLFER.E | N | 55.84 | 1195.6349 | 10 | 1.2 | 399.5527 | 3 | 51.46 | 5 | F5:13030 | Fraction\_5\_23092022.raw |  |  |  |  | 1.2393E5 |  |  | 1 | 0 | 0 | 0 | 0 | 1 | 0 | 0 | 234 | 243 |  |  | PEAKS DB |
| K.YYQYGSDIPFNFK.L | N | 52.43 | 1640.7511 | 13 | 0.3 | 821.3831 | 2 | 68.00 | 2 | F2:19833 | Fraction\_2\_23092022.raw |  | 1.0668E5 |  |  |  |  |  | 1 | 0 | 1 | 0 | 0 | 0 | 0 | 0 | 315 | 327 |  |  | PEAKS DB |
| K.GIDGFRIDAVPHLFER.E | N | 50.68 | 1840.9584 | 16 | 0.0 | 614.6601 | 3 | 68.71 | 2 | F2:20128 | Fraction\_2\_23092022.raw |  | 2.1436E5 |  |  |  |  |  | 1 | 0 | 1 | 0 | 0 | 0 | 0 | 0 | 228 | 243 |  |  | PEAKS DB |
| total 14 peptides |  |  |  |  |  |  |  |  |  |  |  |  |  |  |  |  |  |  |  |  |  |  |  |  |  |  |  |  |  |  |  |
| --- | --- | --- | --- | --- | --- | --- | --- | --- | --- | --- | --- | --- | --- | --- | --- | --- | --- | --- | --- | --- | --- | --- | --- | --- | --- | --- | --- | --- | --- | --- | --- |

TRINITY\_DN1342\_c0\_g1\_i54.p1

back to list

  

| Protein Coverage
| Supporting Peptides
|

Protein Coverage:

Supporting Peptides:

| Peptide | Uniq | -10lgP | Mass | Length | ppm | m/z | z | RT | Fraction | Scan | Source File | Area F1 | Area F2 | Area F3 | Area F4 | Area F5 | Area F6 | Area F7 | #Feature | #Feature F1 | #Feature F2 | #Feature F3 | #Feature F4 | #Feature F5 | #Feature F6 | #Feature F7 | Start | End | PTM | AScore | Found By |
| --- | --- | --- | --- | --- | --- | --- | --- | --- | --- | --- | --- | --- | --- | --- | --- | --- | --- | --- | --- | --- | --- | --- | --- | --- | --- | --- | --- | --- | --- | --- | --- |
| R.SSDSTATDRDYTYLDHIYTKDDPRTYELVK.S | Y | 88.46 | 3567.6641 | 30 | 0.6 | 892.9238 | 4 | 53.26 | 2 | F2:13133 | Fraction\_2\_23092022.raw | 4.6338E6 | 1.4147E8 |  | 1.1258E6 | 3.1845E6 |  |  | 7 | 1 | 4 | 0 | 1 | 1 | 0 | 0 | 252 | 281 |  |  | PEAKS DB |
| K.GIDGFRIDAVPHLFEREDLHDEPR.S | Y | 80.16 | 2832.3943 | 24 | -0.4 | 473.0728 | 6 | 62.45 | 2 | F2:17317 | Fraction\_2\_23092022.raw |  | 1.0771E7 |  |  |  |  |  | 3 | 0 | 3 | 0 | 0 | 0 | 0 | 0 | 228 | 251 |  |  | PEAKS DB |
| R.DYTYLDHIYTKDDPR.T | N | 77.26 | 1913.8795 | 15 | 0.3 | 638.9673 | 3 | 47.98 | 5 | F5:11468 | Fraction\_5\_23092022.raw | 6.7442E5 | 6.2862E6 |  | 2.496E5 | 7.4735E5 |  |  | 5 | 1 | 2 | 0 | 1 | 1 | 0 | 0 | 261 | 275 |  |  | PEAKS DB |
| R.DYTYLDHIYTKDDPRTYELVK.S | N | 76.51 | 2647.2805 | 21 | 1.1 | 662.8281 | 4 | 57.24 | 2 | F2:14940 | Fraction\_2\_23092022.raw |  | 5.1388E6 | 1.891E5 |  | 1.9821E5 |  |  | 4 | 0 | 2 | 1 | 0 | 1 | 0 | 0 | 261 | 281 |  |  | PEAKS DB |
| R.DYTYLDHIYTK.D | N | 74.29 | 1430.6718 | 11 | 0.2 | 716.3433 | 2 | 52.27 | 5 | F5:13434 | Fraction\_5\_23092022.raw |  | 1.8665E5 |  |  | 1.9599E5 |  |  | 3 | 0 | 1 | 0 | 0 | 2 | 0 | 0 | 261 | 271 |  |  | PEAKS DB |
| K.NAVFYQIYPR.S | N | 73.17 | 1269.6505 | 10 | 0.3 | 635.8327 | 2 | 57.87 | 7 | F7:20277 | Fraction\_7\_23092022.raw | 1.1663E7 | 9.8135E7 | 2.3489E6 | 2.1542E5 | 2.417E5 | 9.3508E7 | 6.8859E7 | 8 | 1 | 1 | 1 | 1 | 1 | 2 | 1 | 42 | 51 |  |  | PEAKS DB |
| R.IDAVPHLFEREDLHDEPR.S | Y | 68.72 | 2187.0708 | 18 | 0.7 | 438.4218 | 5 | 47.06 | 2 | F2:10334 | Fraction\_2\_23092022.raw | 7.6093E5 | 3.8278E6 |  |  | 4.9871E5 |  |  | 6 | 2 | 2 | 0 | 0 | 2 | 0 | 0 | 234 | 251 |  |  | PEAKS DB |
| R.SSDSTATDRDYTYLDHIYTK.D | Y | 67.49 | 2351.0554 | 20 | 0.1 | 588.7712 | 4 | 46.79 | 2 | F2:10235 | Fraction\_2\_23092022.raw | 5.662E5 | 3.4895E6 |  |  | 3.6296E5 |  |  | 4 | 2 | 1 | 0 | 0 | 1 | 0 | 0 | 252 | 271 |  |  | PEAKS DB |
| R.SFMDSNDDGVGDLK.G | N | 66.57 | 1498.6245 | 14 | 0.6 | 750.3200 | 2 | 52.27 | 1 | F1:12582 | Fraction\_1\_23092022r.raw | 1.6384E5 |  |  |  |  |  |  | 1 | 1 | 0 | 0 | 0 | 0 | 0 | 0 | 52 | 65 |  |  | PEAKS DB |
| R.SSDSTATDRDYTYLDHIYTKDDPR.T | Y | 65.52 | 2834.2629 | 24 | -0.5 | 945.7611 | 3 | 44.63 | 2 | F2:9256 | Fraction\_2\_23092022.raw |  | 1.5598E8 |  |  | 2.8075E6 |  |  | 5 | 0 | 4 | 0 | 0 | 1 | 0 | 0 | 252 | 275 |  |  | PEAKS DB |
| R.SFM(+15.99)DSNDDGVGDLK.G | N | 60.49 | 1514.6195 | 14 | -0.3 | 758.3168 | 2 | 39.80 | 6 | F6:8482 | Fraction\_6\_23092022.raw | 4.5737E4 | 1.5252E5 |  |  |  | 3.4969E5 |  | 5 | 1 | 2 | 0 | 0 | 0 | 2 | 0 | 52 | 65 | Oxidation (M) | M3:Oxidation (M):1000.00 | PEAKS DB |
| R.IDAVPHLFER.E | N | 55.84 | 1195.6349 | 10 | 1.2 | 399.5527 | 3 | 51.46 | 5 | F5:13030 | Fraction\_5\_23092022.raw |  |  |  |  | 1.2393E5 |  |  | 1 | 0 | 0 | 0 | 0 | 1 | 0 | 0 | 234 | 243 |  |  | PEAKS DB |
| K.YYQYGSDIPFNFK.L | N | 52.43 | 1640.7511 | 13 | 0.3 | 821.3831 | 2 | 68.00 | 2 | F2:19833 | Fraction\_2\_23092022.raw |  | 1.0668E5 |  |  |  |  |  | 1 | 0 | 1 | 0 | 0 | 0 | 0 | 0 | 315 | 327 |  |  | PEAKS DB |
| K.GIDGFRIDAVPHLFER.E | N | 50.68 | 1840.9584 | 16 | 0.0 | 614.6601 | 3 | 68.71 | 2 | F2:20128 | Fraction\_2\_23092022.raw |  | 2.1436E5 |  |  |  |  |  | 1 | 0 | 1 | 0 | 0 | 0 | 0 | 0 | 228 | 243 |  |  | PEAKS DB |
| total 14 peptides |  |  |  |  |  |  |  |  |  |  |  |  |  |  |  |  |  |  |  |  |  |  |  |  |  |  |  |  |  |  |  |
| --- | --- | --- | --- | --- | --- | --- | --- | --- | --- | --- | --- | --- | --- | --- | --- | --- | --- | --- | --- | --- | --- | --- | --- | --- | --- | --- | --- | --- | --- | --- | --- |

TRINITY\_DN2925\_c0\_g1\_i5.p1

back to list

  

| Protein Coverage
| Supporting Peptides
|

Protein Coverage:

Supporting Peptides:

| Peptide | Uniq | -10lgP | Mass | Length | ppm | m/z | z | RT | Fraction | Scan | Source File | Area F1 | Area F2 | Area F3 | Area F4 | Area F5 | Area F6 | Area F7 | #Feature | #Feature F1 | #Feature F2 | #Feature F3 | #Feature F4 | #Feature F5 | #Feature F6 | #Feature F7 | Start | End | PTM | AScore | Found By |
| --- | --- | --- | --- | --- | --- | --- | --- | --- | --- | --- | --- | --- | --- | --- | --- | --- | --- | --- | --- | --- | --- | --- | --- | --- | --- | --- | --- | --- | --- | --- | --- |
| K.VALQC(+57.02)PPGLHFNPNLQVC(+57.02)DLPEQANC(+57.02)K.S | Y | 116.65 | 3118.4788 | 27 | 1.7 | 1040.5020 | 3 | 65.21 | 4 | F4:19436 | Fraction\_4\_23092022.raw |  |  | 3.4165E6 | 2.4828E7 | 1.9413E6 | 3.772E5 |  | 6 | 0 | 0 | 1 | 3 | 1 | 1 | 0 | 374 | 400 | Carbamidomethylation | C5:Carbamidomethylation:1000.00;C18:Carbamidomethylation:1000.00;C26:Carbamidomethylation:1000.00 | PEAKS DB |
| K.IIHQC(+57.02)PPGLHFNKELQIC(+57.02)DLPEHAK.C | Y | 104.54 | 2993.5002 | 25 | 1.1 | 599.7080 | 5 | 46.12 | 7 | F7:13546 | Fraction\_7\_23092022.raw |  |  |  | 1.4228E5 | 2.7779E6 |  | 6.1179E6 | 6 | 0 | 0 | 0 | 1 | 3 | 0 | 2 | 276 | 300 | Carbamidomethylation | C5:Carbamidomethylation:1000.00;C18:Carbamidomethylation:1000.00 | PEAKS DB |
| K.IIHQC(+57.02)PPGLHFNK.E | Y | 82.92 | 1559.8031 | 13 | 0.1 | 520.9417 | 3 | 32.25 | 4 | F4:5141 | Fraction\_4\_23092022.raw |  |  |  | 2.5474E6 |  |  |  | 2 | 0 | 0 | 0 | 2 | 0 | 0 | 0 | 276 | 288 | Carbamidomethylation | C5:Carbamidomethylation:1000.00 | PEAKS DB |
| K.VALQC(+57.02)PPGLHFNPNLQVC(+57.02)DLPEQANC(+57.02)KSKPGDKEDEK.Q | Y | 77.71 | 4232.0088 | 37 | 1.2 | 706.3429 | 6 | 53.88 | 4 | F4:14405 | Fraction\_4\_23092022.raw |  |  |  | 4.2556E5 |  |  |  | 1 | 0 | 0 | 0 | 1 | 0 | 0 | 0 | 472 | 508 | Carbamidomethylation | C5:Carbamidomethylation:1000.00;C18:Carbamidomethylation:1000.00;C26:Carbamidomethylation:1000.00 | PEAKS DB |
| K.ELQIC(+57.02)DLPEHAK.C | Y | 77.42 | 1451.7079 | 12 | -0.2 | 726.8611 | 2 | 42.40 | 5 | F5:8963 | Fraction\_5\_23092022.raw |  |  | 7.1777E5 | 1.0075E7 | 2.2977E7 | 3.7552E6 | 2.3377E6 | 10 | 0 | 0 | 2 | 2 | 2 | 2 | 2 | 289 | 300 | Carbamidomethylation | C5:Carbamidomethylation:1000.00 | PEAKS DB |
| K.C(+57.02)PPGLHFNPVM(+15.99)QIC(+57.02)DLPENAK.C | Y | 51.62 | 2452.1338 | 21 | 0.4 | 818.3855 | 3 | 60.72 | 6 | F6:17890 | Fraction\_6\_23092022.raw |  |  |  |  |  | 2.7398E5 |  | 1 | 0 | 0 | 0 | 0 | 0 | 1 | 0 | 184 | 204 | Carbamidomethylation; Oxidation (M) | C1:Carbamidomethylation:1000.00;M11:Oxidation (M):1000.00;C14:Carbamidomethylation:1000.00 | PEAKS DB |
| total 6 peptides |  |  |  |  |  |  |  |  |  |  |  |  |  |  |  |  |  |  |  |  |  |  |  |  |  |  |  |  |  |  |  |
| --- | --- | --- | --- | --- | --- | --- | --- | --- | --- | --- | --- | --- | --- | --- | --- | --- | --- | --- | --- | --- | --- | --- | --- | --- | --- | --- | --- | --- | --- | --- | --- |

P13647|K2C5\_HUMAN

back to list

  

| Protein Coverage
| Supporting Peptides
|

Protein Coverage:

Supporting Peptides:

| Peptide | Uniq | -10lgP | Mass | Length | ppm | m/z | z | RT | Fraction | Scan | Source File | Area F1 | Area F2 | Area F3 | Area F4 | Area F5 | Area F6 | Area F7 | #Feature | #Feature F1 | #Feature F2 | #Feature F3 | #Feature F4 | #Feature F5 | #Feature F6 | #Feature F7 | Start | End | PTM | AScore | Found By |
| --- | --- | --- | --- | --- | --- | --- | --- | --- | --- | --- | --- | --- | --- | --- | --- | --- | --- | --- | --- | --- | --- | --- | --- | --- | --- | --- | --- | --- | --- | --- | --- |
| R.VSLAGAC(+57.02)GVGGYGSR.S | Y | 77.82 | 1409.6721 | 15 | 0.0 | 705.8433 | 2 | 41.93 | 2 | F2:8051 | Fraction\_2\_23092022.raw | 4.6468E5 | 3.9894E5 | 2.1731E5 | 2.6554E4 | 3.3682E4 | 7.8008E4 |  | 6 | 1 | 1 | 1 | 1 | 1 | 1 | 0 | 49 | 63 | Carbamidomethylation | C7:Carbamidomethylation:1000.00 | PEAKS DB |
| K.VLDTKWTLLQEQGTK.T | N | 72.29 | 1758.9515 | 15 | 0.5 | 587.3248 | 3 | 54.32 | 1 | F1:13486 | Fraction\_1\_23092022r.raw | 2.7823E5 |  |  |  |  |  |  | 1 | 1 | 0 | 0 | 0 | 0 | 0 | 0 | 195 | 209 |  |  | PEAKS DB |
| R.SGGGGGGGFGRVSLAGAC(+57.02)GVGGYGSR.S | Y | 72.07 | 2256.0454 | 26 | 0.7 | 753.0229 | 3 | 47.29 | 2 | F2:10490 | Fraction\_2\_23092022.raw |  | 1.6936E5 |  |  |  |  |  | 1 | 0 | 1 | 0 | 0 | 0 | 0 | 0 | 38 | 63 | Carbamidomethylation | C18:Carbamidomethylation:1000.00 | PEAKS DB |
| K.TLNNKFASFIDKVR.F | N | 71.43 | 1651.9045 | 14 | -0.2 | 551.6420 | 3 | 51.36 | 2 | F2:12305 | Fraction\_2\_23092022.raw | 2.671E5 | 1.8567E6 | 2.1296E5 |  |  | 8.2338E5 | 3.789E5 | 6 | 1 | 2 | 1 | 0 | 0 | 1 | 1 | 174 | 187 |  |  | PEAKS DB |
| R.NLDLDSIIAEVK.A | N | 67.73 | 1328.7188 | 12 | -0.3 | 665.3665 | 2 | 80.82 | 4 | F4:26420 | Fraction\_4\_23092022.raw | 1.3444E7 | 1.1783E7 | 2.3503E7 | 6.3504E6 | 3.9084E6 | 1.1367E7 | 9.0701E6 | 8 | 1 | 1 | 2 | 1 | 1 | 1 | 1 | 332 | 343 |  |  | PEAKS DB |
| K.FASFIDKVR.F | N | 64.36 | 1081.5920 | 9 | 1.5 | 361.5385 | 3 | 44.57 | 4 | F4:10216 | Fraction\_4\_23092022.raw | 6.0874E6 | 1.3079E6 | 2.6129E6 | 3.499E5 | 3.5976E5 | 1.1286E6 |  | 11 | 1 | 2 | 2 | 2 | 2 | 2 | 0 | 179 | 187 |  |  | PEAKS DB |
| R.SFSTASAITPSVSR.T | N | 63.29 | 1409.7151 | 14 | 0.8 | 705.8654 | 2 | 44.69 | 1 | F1:9208 | Fraction\_1\_23092022r.raw | 2.2271E5 | 3.7261E5 |  |  |  | 1.8547E5 |  | 3 | 1 | 1 | 0 | 0 | 0 | 1 | 0 | 16 | 29 |  |  | PEAKS DB |
| K.LALDVEIATYR.K | N | 62.33 | 1262.6870 | 11 | 0.5 | 632.3511 | 2 | 64.39 | 3 | F3:18523 | Fraction\_3\_23092022.raw | 1.5189E6 | 6.2742E5 | 1.3952E6 | 2.3872E5 | 2.1606E5 | 3.32E5 |  | 6 | 1 | 1 | 1 | 1 | 1 | 1 | 0 | 461 | 471 |  |  | PEAKS DB |
| K.WTLLQEQGTK.T | N | 59.30 | 1202.6295 | 10 | -0.2 | 602.3219 | 2 | 51.37 | 2 | F2:12309 | Fraction\_2\_23092022.raw |  | 1.3976E5 |  |  |  |  |  | 1 | 0 | 1 | 0 | 0 | 0 | 0 | 0 | 200 | 209 |  |  | PEAKS DB |
| R.SRTEAESWYQTKYEELQQTAGR.H | N | 58.16 | 2660.2466 | 22 | 0.0 | 666.0689 | 4 | 50.00 | 1 | F1:11570 | Fraction\_1\_23092022r.raw | 3.189E5 |  |  |  |  |  |  | 1 | 1 | 0 | 0 | 0 | 0 | 0 | 0 | 353 | 374 |  |  | PEAKS DB |
| K.LAELEEALQK.A | Y | 56.77 | 1142.6183 | 10 | 0.5 | 572.3167 | 2 | 48.80 | 3 | F3:11578 | Fraction\_3\_23092022.raw | 3.8165E5 | 8.1985E5 | 9.5108E5 | 1.8174E5 |  |  |  | 4 | 1 | 1 | 1 | 1 | 0 | 0 | 0 | 432 | 441 |  |  | PEAKS DB |
| K.QC(+57.02)ANLQNAIADAEQR.G | N | 52.83 | 1700.7900 | 15 | 0.2 | 851.4025 | 2 | 50.02 | 1 | F1:11583 | Fraction\_1\_23092022r.raw | 1.223E5 |  |  |  |  |  |  | 1 | 1 | 0 | 0 | 0 | 0 | 0 | 0 | 406 | 420 | Carbamidomethylation | C2:Carbamidomethylation:1000.00 | PEAKS DB |
| K.LALDVEIATYRK.L | N | 51.28 | 1390.7820 | 12 | 0.0 | 464.6013 | 3 | 53.42 | 1 | F1:13083 | Fraction\_1\_23092022r.raw | 4.7232E5 |  |  |  |  |  |  | 1 | 1 | 0 | 0 | 0 | 0 | 0 | 0 | 461 | 472 |  |  | PEAKS DB |
| R.ISISTSGGSFR.N | Y | 51.21 | 1110.5669 | 11 | -0.5 | 556.2905 | 2 | 42.03 | 1 | F1:8027 | Fraction\_1\_23092022r.raw | 7.6343E5 |  |  |  |  |  |  | 1 | 1 | 0 | 0 | 0 | 0 | 0 | 0 | 74 | 84 |  |  | PEAKS DB |
| R.QNLEPLFEQYINNLRR.Q | N | 48.69 | 2046.0646 | 16 | 0.4 | 683.0291 | 3 | 77.65 | 1 | F1:23976 | Fraction\_1\_23092022r.raw | 2.1213E6 |  |  |  |  |  |  | 1 | 1 | 0 | 0 | 0 | 0 | 0 | 0 | 213 | 228 |  |  | PEAKS DB |
| total 15 peptides |  |  |  |  |  |  |  |  |  |  |  |  |  |  |  |  |  |  |  |  |  |  |  |  |  |  |  |  |  |  |  |
| --- | --- | --- | --- | --- | --- | --- | --- | --- | --- | --- | --- | --- | --- | --- | --- | --- | --- | --- | --- | --- | --- | --- | --- | --- | --- | --- | --- | --- | --- | --- | --- |

TRINITY\_DN1342\_c0\_g1\_i9.p1

back to list

  

| Protein Coverage
| Supporting Peptides
|

Protein Coverage:

Supporting Peptides:

| Peptide | Uniq | -10lgP | Mass | Length | ppm | m/z | z | RT | Fraction | Scan | Source File | Area F1 | Area F2 | Area F3 | Area F4 | Area F5 | Area F6 | Area F7 | #Feature | #Feature F1 | #Feature F2 | #Feature F3 | #Feature F4 | #Feature F5 | #Feature F6 | #Feature F7 | Start | End | PTM | AScore | Found By |
| --- | --- | --- | --- | --- | --- | --- | --- | --- | --- | --- | --- | --- | --- | --- | --- | --- | --- | --- | --- | --- | --- | --- | --- | --- | --- | --- | --- | --- | --- | --- | --- |
| R.DPNRTPFQWSYVR.N | N | 92.58 | 1664.8059 | 13 | -0.6 | 555.9423 | 3 | 57.76 | 1 | F1:14997 | Fraction\_1\_23092022r.raw | 2.4455E5 | 9.7444E5 |  |  |  |  |  | 2 | 1 | 1 | 0 | 0 | 0 | 0 | 0 | 432 | 444 |  |  | PEAKS DB |
| K.YYQYGSHIPFNFK.M | Y | 81.90 | 1662.7831 | 13 | 0.1 | 832.3989 | 2 | 55.57 | 2 | F2:14195 | Fraction\_2\_23092022.raw | 1.2315E6 | 8.1127E5 |  |  |  |  |  | 3 | 2 | 1 | 0 | 0 | 0 | 0 | 0 | 315 | 327 |  |  | PEAKS DB |
| R.DYTYLDHIYTKDDPR.T | N | 77.26 | 1913.8795 | 15 | 0.3 | 638.9673 | 3 | 47.98 | 5 | F5:11468 | Fraction\_5\_23092022.raw | 6.7442E5 | 6.2862E6 |  | 2.496E5 | 7.4735E5 |  |  | 5 | 1 | 2 | 0 | 1 | 1 | 0 | 0 | 261 | 275 |  |  | PEAKS DB |
| R.DYTYLDHIYTKDDPRTYELVK.S | N | 76.51 | 2647.2805 | 21 | 1.1 | 662.8281 | 4 | 57.24 | 2 | F2:14940 | Fraction\_2\_23092022.raw |  | 5.1388E6 | 1.891E5 |  | 1.9821E5 |  |  | 4 | 0 | 2 | 1 | 0 | 1 | 0 | 0 | 261 | 281 |  |  | PEAKS DB |
| R.DYTYLDHIYTK.D | N | 74.29 | 1430.6718 | 11 | 0.2 | 716.3433 | 2 | 52.27 | 5 | F5:13434 | Fraction\_5\_23092022.raw |  | 1.8665E5 |  |  | 1.9599E5 |  |  | 3 | 0 | 1 | 0 | 0 | 2 | 0 | 0 | 261 | 271 |  |  | PEAKS DB |
| K.NAVFYQIYPR.S | N | 73.17 | 1269.6505 | 10 | 0.3 | 635.8327 | 2 | 57.87 | 7 | F7:20277 | Fraction\_7\_23092022.raw | 1.1663E7 | 9.8135E7 | 2.3489E6 | 2.1542E5 | 2.417E5 | 9.3508E7 | 6.8859E7 | 8 | 1 | 1 | 1 | 1 | 1 | 2 | 1 | 42 | 51 |  |  | PEAKS DB |
| R.SFMDSNDDGVGDLK.G | N | 66.57 | 1498.6245 | 14 | 0.6 | 750.3200 | 2 | 52.27 | 1 | F1:12582 | Fraction\_1\_23092022r.raw | 1.6384E5 |  |  |  |  |  |  | 1 | 1 | 0 | 0 | 0 | 0 | 0 | 0 | 52 | 65 |  |  | PEAKS DB |
| R.TPFQWSYVR.N | N | 65.12 | 1182.5822 | 9 | 1.5 | 592.2993 | 2 | 59.87 | 1 | F1:15973 | Fraction\_1\_23092022r.raw | 4.492E5 | 6.4111E5 |  |  |  |  |  | 2 | 1 | 1 | 0 | 0 | 0 | 0 | 0 | 436 | 444 |  |  | PEAKS DB |
| R.SFM(+15.99)DSNDDGVGDLK.G | N | 60.49 | 1514.6195 | 14 | -0.3 | 758.3168 | 2 | 39.80 | 6 | F6:8482 | Fraction\_6\_23092022.raw | 4.5737E4 | 1.5252E5 |  |  |  | 3.4969E5 |  | 5 | 1 | 2 | 0 | 0 | 0 | 2 | 0 | 52 | 65 | Oxidation (M) | M3:Oxidation (M):1000.00 | PEAKS DB |
| R.IDAVPHLFER.Q | N | 55.84 | 1195.6349 | 10 | 1.2 | 399.5527 | 3 | 51.46 | 5 | F5:13030 | Fraction\_5\_23092022.raw |  |  |  |  | 1.2393E5 |  |  | 1 | 0 | 0 | 0 | 0 | 1 | 0 | 0 | 234 | 243 |  |  | PEAKS DB |
| K.GIDGFRIDAVPHLFER.Q | N | 50.68 | 1840.9584 | 16 | 0.0 | 614.6601 | 3 | 68.71 | 2 | F2:20128 | Fraction\_2\_23092022.raw |  | 2.1436E5 |  |  |  |  |  | 1 | 0 | 1 | 0 | 0 | 0 | 0 | 0 | 228 | 243 |  |  | PEAKS DB |
| total 11 peptides |  |  |  |  |  |  |  |  |  |  |  |  |  |  |  |  |  |  |  |  |  |  |  |  |  |  |  |  |  |  |  |
| --- | --- | --- | --- | --- | --- | --- | --- | --- | --- | --- | --- | --- | --- | --- | --- | --- | --- | --- | --- | --- | --- | --- | --- | --- | --- | --- | --- | --- | --- | --- | --- |

TRINITY\_DN1709\_c0\_g1\_i4.p1

back to list

  

| Protein Coverage
| Supporting Peptides
|

Protein Coverage:

Supporting Peptides:

| Peptide | Uniq | -10lgP | Mass | Length | ppm | m/z | z | RT | Fraction | Scan | Source File | Area F1 | Area F2 | Area F3 | Area F4 | Area F5 | Area F6 | Area F7 | #Feature | #Feature F1 | #Feature F2 | #Feature F3 | #Feature F4 | #Feature F5 | #Feature F6 | #Feature F7 | Start | End | PTM | AScore | Found By |
| --- | --- | --- | --- | --- | --- | --- | --- | --- | --- | --- | --- | --- | --- | --- | --- | --- | --- | --- | --- | --- | --- | --- | --- | --- | --- | --- | --- | --- | --- | --- | --- |
| R.DPNYFIHKDATNLGYYVK.T | Y | 100.58 | 2157.0530 | 18 | -0.2 | 720.0248 | 3 | 54.78 | 1 | F1:13669 | Fraction\_1\_23092022r.raw | 3.1758E6 | 3.3896E5 |  |  |  |  |  | 3 | 2 | 1 | 0 | 0 | 0 | 0 | 0 | 456 | 473 |  |  | PEAKS DB |
| R.QFTVLTGTAPLPQMFTLGYHQSR.W | Y | 91.54 | 2592.3159 | 23 | 1.7 | 865.1141 | 3 | 80.04 | 1 | F1:24992 | Fraction\_1\_23092022r.raw | 4.1699E5 |  |  |  |  |  |  | 1 | 1 | 0 | 0 | 0 | 0 | 0 | 0 | 360 | 382 |  |  | PEAKS DB |
| R.QFTVLTGTAPLPQM(+15.99)FTLGYHQSR.W | Y | 78.28 | 2608.3108 | 23 | 1.1 | 870.4452 | 3 | 71.23 | 1 | F1:21031 | Fraction\_1\_23092022r.raw | 1.0421E6 |  |  |  |  |  |  | 1 | 1 | 0 | 0 | 0 | 0 | 0 | 0 | 360 | 382 | Oxidation (M) | M14:Oxidation (M):1000.00 | PEAKS DB |
| K.RDPNYFIHKDATNLGYYVK.T | Y | 70.24 | 2313.1541 | 19 | 0.5 | 579.2961 | 4 | 46.37 | 1 | F1:9935 | Fraction\_1\_23092022r.raw | 9.6459E5 |  |  |  |  |  |  | 1 | 1 | 0 | 0 | 0 | 0 | 0 | 0 | 455 | 473 |  |  | PEAKS DB |
| K.DATNLGYYVK.T | Y | 66.64 | 1142.5608 | 10 | 0.5 | 572.2880 | 2 | 48.50 | 1 | F1:10872 | Fraction\_1\_23092022r.raw | 3.3709E6 | 5.0668E5 |  |  |  |  |  | 2 | 1 | 1 | 0 | 0 | 0 | 0 | 0 | 464 | 473 |  |  | PEAKS DB |
| R.KLVVIIDPHIK.R | Y | 58.49 | 1273.8121 | 11 | -0.7 | 425.6110 | 3 | 45.20 | 1 | F1:9404 | Fraction\_1\_23092022r.raw | 3.2703E6 | 1.6077E5 |  |  |  |  |  | 2 | 1 | 1 | 0 | 0 | 0 | 0 | 0 | 444 | 454 |  |  | PEAKS DB |
| K.DATNLGYYVKTR.D | Y | 57.56 | 1399.7096 | 12 | -0.8 | 467.5768 | 3 | 42.28 | 1 | F1:8171 | Fraction\_1\_23092022r.raw | 1.1559E5 |  |  |  |  |  |  | 2 | 2 | 0 | 0 | 0 | 0 | 0 | 0 | 464 | 475 |  |  | PEAKS DB |
| K.LVVIIDPHIK.R | Y | 57.23 | 1145.7172 | 10 | -0.9 | 382.9127 | 3 | 53.58 | 1 | F1:13154 | Fraction\_1\_23092022r.raw | 9.6738E5 |  |  |  |  |  |  | 2 | 2 | 0 | 0 | 0 | 0 | 0 | 0 | 445 | 454 |  |  | PEAKS DB |
| K.LVVIIDPHIKR.D | Y | 52.12 | 1301.8184 | 11 | 0.1 | 434.9468 | 3 | 44.44 | 1 | F1:9092 | Fraction\_1\_23092022r.raw | 1.3859E6 | 8.6579E4 |  |  |  |  |  | 2 | 1 | 1 | 0 | 0 | 0 | 0 | 0 | 445 | 455 |  |  | PEAKS DB |
| R.KLVVIIDPHIKR.D | Y | 50.41 | 1429.9132 | 12 | 0.5 | 477.6452 | 3 | 37.88 | 1 | F1:6401 | Fraction\_1\_23092022r.raw | 6.2527E5 |  |  |  |  |  |  | 1 | 1 | 0 | 0 | 0 | 0 | 0 | 0 | 444 | 455 |  |  | PEAKS DB |
| total 10 peptides |  |  |  |  |  |  |  |  |  |  |  |  |  |  |  |  |  |  |  |  |  |  |  |  |  |  |  |  |  |  |  |
| --- | --- | --- | --- | --- | --- | --- | --- | --- | --- | --- | --- | --- | --- | --- | --- | --- | --- | --- | --- | --- | --- | --- | --- | --- | --- | --- | --- | --- | --- | --- | --- |

TRINITY\_DN1709\_c0\_g1\_i5.p1

back to list

  

| Protein Coverage
| Supporting Peptides
|

Protein Coverage:

Supporting Peptides:

| Peptide | Uniq | -10lgP | Mass | Length | ppm | m/z | z | RT | Fraction | Scan | Source File | Area F1 | Area F2 | Area F3 | Area F4 | Area F5 | Area F6 | Area F7 | #Feature | #Feature F1 | #Feature F2 | #Feature F3 | #Feature F4 | #Feature F5 | #Feature F6 | #Feature F7 | Start | End | PTM | AScore | Found By |
| --- | --- | --- | --- | --- | --- | --- | --- | --- | --- | --- | --- | --- | --- | --- | --- | --- | --- | --- | --- | --- | --- | --- | --- | --- | --- | --- | --- | --- | --- | --- | --- |
| R.DPNYFIHKDATNLGYYVK.T | Y | 100.58 | 2157.0530 | 18 | -0.2 | 720.0248 | 3 | 54.78 | 1 | F1:13669 | Fraction\_1\_23092022r.raw | 3.1758E6 | 3.3896E5 |  |  |  |  |  | 3 | 2 | 1 | 0 | 0 | 0 | 0 | 0 | 456 | 473 |  |  | PEAKS DB |
| R.QFTVLTGTAPLPQMFTLGYHQSR.W | Y | 91.54 | 2592.3159 | 23 | 1.7 | 865.1141 | 3 | 80.04 | 1 | F1:24992 | Fraction\_1\_23092022r.raw | 4.1699E5 |  |  |  |  |  |  | 1 | 1 | 0 | 0 | 0 | 0 | 0 | 0 | 360 | 382 |  |  | PEAKS DB |
| R.QFTVLTGTAPLPQM(+15.99)FTLGYHQSR.W | Y | 78.28 | 2608.3108 | 23 | 1.1 | 870.4452 | 3 | 71.23 | 1 | F1:21031 | Fraction\_1\_23092022r.raw | 1.0421E6 |  |  |  |  |  |  | 1 | 1 | 0 | 0 | 0 | 0 | 0 | 0 | 360 | 382 | Oxidation (M) | M14:Oxidation (M):1000.00 | PEAKS DB |
| K.RDPNYFIHKDATNLGYYVK.T | Y | 70.24 | 2313.1541 | 19 | 0.5 | 579.2961 | 4 | 46.37 | 1 | F1:9935 | Fraction\_1\_23092022r.raw | 9.6459E5 |  |  |  |  |  |  | 1 | 1 | 0 | 0 | 0 | 0 | 0 | 0 | 455 | 473 |  |  | PEAKS DB |
| K.DATNLGYYVK.T | Y | 66.64 | 1142.5608 | 10 | 0.5 | 572.2880 | 2 | 48.50 | 1 | F1:10872 | Fraction\_1\_23092022r.raw | 3.3709E6 | 5.0668E5 |  |  |  |  |  | 2 | 1 | 1 | 0 | 0 | 0 | 0 | 0 | 464 | 473 |  |  | PEAKS DB |
| R.KLVVIIDPHIK.R | Y | 58.49 | 1273.8121 | 11 | -0.7 | 425.6110 | 3 | 45.20 | 1 | F1:9404 | Fraction\_1\_23092022r.raw | 3.2703E6 | 1.6077E5 |  |  |  |  |  | 2 | 1 | 1 | 0 | 0 | 0 | 0 | 0 | 444 | 454 |  |  | PEAKS DB |
| K.DATNLGYYVKTR.D | Y | 57.56 | 1399.7096 | 12 | -0.8 | 467.5768 | 3 | 42.28 | 1 | F1:8171 | Fraction\_1\_23092022r.raw | 1.1559E5 |  |  |  |  |  |  | 2 | 2 | 0 | 0 | 0 | 0 | 0 | 0 | 464 | 475 |  |  | PEAKS DB |
| K.LVVIIDPHIK.R | Y | 57.23 | 1145.7172 | 10 | -0.9 | 382.9127 | 3 | 53.58 | 1 | F1:13154 | Fraction\_1\_23092022r.raw | 9.6738E5 |  |  |  |  |  |  | 2 | 2 | 0 | 0 | 0 | 0 | 0 | 0 | 445 | 454 |  |  | PEAKS DB |
| K.LVVIIDPHIKR.D | Y | 52.12 | 1301.8184 | 11 | 0.1 | 434.9468 | 3 | 44.44 | 1 | F1:9092 | Fraction\_1\_23092022r.raw | 1.3859E6 | 8.6579E4 |  |  |  |  |  | 2 | 1 | 1 | 0 | 0 | 0 | 0 | 0 | 445 | 455 |  |  | PEAKS DB |
| R.KLVVIIDPHIKR.D | Y | 50.41 | 1429.9132 | 12 | 0.5 | 477.6452 | 3 | 37.88 | 1 | F1:6401 | Fraction\_1\_23092022r.raw | 6.2527E5 |  |  |  |  |  |  | 1 | 1 | 0 | 0 | 0 | 0 | 0 | 0 | 444 | 455 |  |  | PEAKS DB |
| total 10 peptides |  |  |  |  |  |  |  |  |  |  |  |  |  |  |  |  |  |  |  |  |  |  |  |  |  |  |  |  |  |  |  |
| --- | --- | --- | --- | --- | --- | --- | --- | --- | --- | --- | --- | --- | --- | --- | --- | --- | --- | --- | --- | --- | --- | --- | --- | --- | --- | --- | --- | --- | --- | --- | --- |

TRINITY\_DN1709\_c0\_g1\_i6.p1

back to list

  

| Protein Coverage
| Supporting Peptides
|

Protein Coverage:

Supporting Peptides:

| Peptide | Uniq | -10lgP | Mass | Length | ppm | m/z | z | RT | Fraction | Scan | Source File | Area F1 | Area F2 | Area F3 | Area F4 | Area F5 | Area F6 | Area F7 | #Feature | #Feature F1 | #Feature F2 | #Feature F3 | #Feature F4 | #Feature F5 | #Feature F6 | #Feature F7 | Start | End | PTM | AScore | Found By |
| --- | --- | --- | --- | --- | --- | --- | --- | --- | --- | --- | --- | --- | --- | --- | --- | --- | --- | --- | --- | --- | --- | --- | --- | --- | --- | --- | --- | --- | --- | --- | --- |
| R.DPNYFIHKDATNLGYYVK.T | Y | 100.58 | 2157.0530 | 18 | -0.2 | 720.0248 | 3 | 54.78 | 1 | F1:13669 | Fraction\_1\_23092022r.raw | 3.1758E6 | 3.3896E5 |  |  |  |  |  | 3 | 2 | 1 | 0 | 0 | 0 | 0 | 0 | 456 | 473 |  |  | PEAKS DB |
| R.QFTVLTGTAPLPQMFTLGYHQSR.W | Y | 91.54 | 2592.3159 | 23 | 1.7 | 865.1141 | 3 | 80.04 | 1 | F1:24992 | Fraction\_1\_23092022r.raw | 4.1699E5 |  |  |  |  |  |  | 1 | 1 | 0 | 0 | 0 | 0 | 0 | 0 | 360 | 382 |  |  | PEAKS DB |
| R.QFTVLTGTAPLPQM(+15.99)FTLGYHQSR.W | Y | 78.28 | 2608.3108 | 23 | 1.1 | 870.4452 | 3 | 71.23 | 1 | F1:21031 | Fraction\_1\_23092022r.raw | 1.0421E6 |  |  |  |  |  |  | 1 | 1 | 0 | 0 | 0 | 0 | 0 | 0 | 360 | 382 | Oxidation (M) | M14:Oxidation (M):1000.00 | PEAKS DB |
| K.RDPNYFIHKDATNLGYYVK.T | Y | 70.24 | 2313.1541 | 19 | 0.5 | 579.2961 | 4 | 46.37 | 1 | F1:9935 | Fraction\_1\_23092022r.raw | 9.6459E5 |  |  |  |  |  |  | 1 | 1 | 0 | 0 | 0 | 0 | 0 | 0 | 455 | 473 |  |  | PEAKS DB |
| K.DATNLGYYVK.T | Y | 66.64 | 1142.5608 | 10 | 0.5 | 572.2880 | 2 | 48.50 | 1 | F1:10872 | Fraction\_1\_23092022r.raw | 3.3709E6 | 5.0668E5 |  |  |  |  |  | 2 | 1 | 1 | 0 | 0 | 0 | 0 | 0 | 464 | 473 |  |  | PEAKS DB |
| R.KLVVIIDPHIK.R | Y | 58.49 | 1273.8121 | 11 | -0.7 | 425.6110 | 3 | 45.20 | 1 | F1:9404 | Fraction\_1\_23092022r.raw | 3.2703E6 | 1.6077E5 |  |  |  |  |  | 2 | 1 | 1 | 0 | 0 | 0 | 0 | 0 | 444 | 454 |  |  | PEAKS DB |
| K.DATNLGYYVKTR.D | Y | 57.56 | 1399.7096 | 12 | -0.8 | 467.5768 | 3 | 42.28 | 1 | F1:8171 | Fraction\_1\_23092022r.raw | 1.1559E5 |  |  |  |  |  |  | 2 | 2 | 0 | 0 | 0 | 0 | 0 | 0 | 464 | 475 |  |  | PEAKS DB |
| K.LVVIIDPHIK.R | Y | 57.23 | 1145.7172 | 10 | -0.9 | 382.9127 | 3 | 53.58 | 1 | F1:13154 | Fraction\_1\_23092022r.raw | 9.6738E5 |  |  |  |  |  |  | 2 | 2 | 0 | 0 | 0 | 0 | 0 | 0 | 445 | 454 |  |  | PEAKS DB |
| K.LVVIIDPHIKR.D | Y | 52.12 | 1301.8184 | 11 | 0.1 | 434.9468 | 3 | 44.44 | 1 | F1:9092 | Fraction\_1\_23092022r.raw | 1.3859E6 | 8.6579E4 |  |  |  |  |  | 2 | 1 | 1 | 0 | 0 | 0 | 0 | 0 | 445 | 455 |  |  | PEAKS DB |
| R.KLVVIIDPHIKR.D | Y | 50.41 | 1429.9132 | 12 | 0.5 | 477.6452 | 3 | 37.88 | 1 | F1:6401 | Fraction\_1\_23092022r.raw | 6.2527E5 |  |  |  |  |  |  | 1 | 1 | 0 | 0 | 0 | 0 | 0 | 0 | 444 | 455 |  |  | PEAKS DB |
| total 10 peptides |  |  |  |  |  |  |  |  |  |  |  |  |  |  |  |  |  |  |  |  |  |  |  |  |  |  |  |  |  |  |  |
| --- | --- | --- | --- | --- | --- | --- | --- | --- | --- | --- | --- | --- | --- | --- | --- | --- | --- | --- | --- | --- | --- | --- | --- | --- | --- | --- | --- | --- | --- | --- | --- |

TRINITY\_DN1709\_c0\_g1\_i8.p1

back to list

  

| Protein Coverage
| Supporting Peptides
|

Protein Coverage:

Supporting Peptides:

| Peptide | Uniq | -10lgP | Mass | Length | ppm | m/z | z | RT | Fraction | Scan | Source File | Area F1 | Area F2 | Area F3 | Area F4 | Area F5 | Area F6 | Area F7 | #Feature | #Feature F1 | #Feature F2 | #Feature F3 | #Feature F4 | #Feature F5 | #Feature F6 | #Feature F7 | Start | End | PTM | AScore | Found By |
| --- | --- | --- | --- | --- | --- | --- | --- | --- | --- | --- | --- | --- | --- | --- | --- | --- | --- | --- | --- | --- | --- | --- | --- | --- | --- | --- | --- | --- | --- | --- | --- |
| R.DPNYFIHKDATNLGYYVK.T | Y | 100.58 | 2157.0530 | 18 | -0.2 | 720.0248 | 3 | 54.78 | 1 | F1:13669 | Fraction\_1\_23092022r.raw | 3.1758E6 | 3.3896E5 |  |  |  |  |  | 3 | 2 | 1 | 0 | 0 | 0 | 0 | 0 | 456 | 473 |  |  | PEAKS DB |
| R.QFTVLTGTAPLPQMFTLGYHQSR.W | Y | 91.54 | 2592.3159 | 23 | 1.7 | 865.1141 | 3 | 80.04 | 1 | F1:24992 | Fraction\_1\_23092022r.raw | 4.1699E5 |  |  |  |  |  |  | 1 | 1 | 0 | 0 | 0 | 0 | 0 | 0 | 360 | 382 |  |  | PEAKS DB |
| R.QFTVLTGTAPLPQM(+15.99)FTLGYHQSR.W | Y | 78.28 | 2608.3108 | 23 | 1.1 | 870.4452 | 3 | 71.23 | 1 | F1:21031 | Fraction\_1\_23092022r.raw | 1.0421E6 |  |  |  |  |  |  | 1 | 1 | 0 | 0 | 0 | 0 | 0 | 0 | 360 | 382 | Oxidation (M) | M14:Oxidation (M):1000.00 | PEAKS DB |
| K.RDPNYFIHKDATNLGYYVK.T | Y | 70.24 | 2313.1541 | 19 | 0.5 | 579.2961 | 4 | 46.37 | 1 | F1:9935 | Fraction\_1\_23092022r.raw | 9.6459E5 |  |  |  |  |  |  | 1 | 1 | 0 | 0 | 0 | 0 | 0 | 0 | 455 | 473 |  |  | PEAKS DB |
| K.DATNLGYYVK.T | Y | 66.64 | 1142.5608 | 10 | 0.5 | 572.2880 | 2 | 48.50 | 1 | F1:10872 | Fraction\_1\_23092022r.raw | 3.3709E6 | 5.0668E5 |  |  |  |  |  | 2 | 1 | 1 | 0 | 0 | 0 | 0 | 0 | 464 | 473 |  |  | PEAKS DB |
| R.KLVVIIDPHIK.R | Y | 58.49 | 1273.8121 | 11 | -0.7 | 425.6110 | 3 | 45.20 | 1 | F1:9404 | Fraction\_1\_23092022r.raw | 3.2703E6 | 1.6077E5 |  |  |  |  |  | 2 | 1 | 1 | 0 | 0 | 0 | 0 | 0 | 444 | 454 |  |  | PEAKS DB |
| K.DATNLGYYVKTR.D | Y | 57.56 | 1399.7096 | 12 | -0.8 | 467.5768 | 3 | 42.28 | 1 | F1:8171 | Fraction\_1\_23092022r.raw | 1.1559E5 |  |  |  |  |  |  | 2 | 2 | 0 | 0 | 0 | 0 | 0 | 0 | 464 | 475 |  |  | PEAKS DB |
| K.LVVIIDPHIK.R | Y | 57.23 | 1145.7172 | 10 | -0.9 | 382.9127 | 3 | 53.58 | 1 | F1:13154 | Fraction\_1\_23092022r.raw | 9.6738E5 |  |  |  |  |  |  | 2 | 2 | 0 | 0 | 0 | 0 | 0 | 0 | 445 | 454 |  |  | PEAKS DB |
| K.LVVIIDPHIKR.D | Y | 52.12 | 1301.8184 | 11 | 0.1 | 434.9468 | 3 | 44.44 | 1 | F1:9092 | Fraction\_1\_23092022r.raw | 1.3859E6 | 8.6579E4 |  |  |  |  |  | 2 | 1 | 1 | 0 | 0 | 0 | 0 | 0 | 445 | 455 |  |  | PEAKS DB |
| R.KLVVIIDPHIKR.D | Y | 50.41 | 1429.9132 | 12 | 0.5 | 477.6452 | 3 | 37.88 | 1 | F1:6401 | Fraction\_1\_23092022r.raw | 6.2527E5 |  |  |  |  |  |  | 1 | 1 | 0 | 0 | 0 | 0 | 0 | 0 | 444 | 455 |  |  | PEAKS DB |
| total 10 peptides |  |  |  |  |  |  |  |  |  |  |  |  |  |  |  |  |  |  |  |  |  |  |  |  |  |  |  |  |  |  |  |
| --- | --- | --- | --- | --- | --- | --- | --- | --- | --- | --- | --- | --- | --- | --- | --- | --- | --- | --- | --- | --- | --- | --- | --- | --- | --- | --- | --- | --- | --- | --- | --- |

P02768|ALBU\_HUMAN

back to list

  

| Protein Coverage
| Supporting Peptides
|

Protein Coverage:

Supporting Peptides:

| Peptide | Uniq | -10lgP | Mass | Length | ppm | m/z | z | RT | Fraction | Scan | Source File | Area F1 | Area F2 | Area F3 | Area F4 | Area F5 | Area F6 | Area F7 | #Feature | #Feature F1 | #Feature F2 | #Feature F3 | #Feature F4 | #Feature F5 | #Feature F6 | #Feature F7 | Start | End | PTM | AScore | Found By |
| --- | --- | --- | --- | --- | --- | --- | --- | --- | --- | --- | --- | --- | --- | --- | --- | --- | --- | --- | --- | --- | --- | --- | --- | --- | --- | --- | --- | --- | --- | --- | --- |
| K.VFDEFKPLVEEPQNLIK.Q | Y | 89.69 | 2044.0880 | 17 | -0.1 | 682.3699 | 3 | 71.98 | 1 | F1:21393 | Fraction\_1\_23092022r.raw | 1.006E6 |  |  |  |  |  |  | 1 | 1 | 0 | 0 | 0 | 0 | 0 | 0 | 397 | 413 |  |  | PEAKS DB |
| K.QNC(+57.02)ELFEQLGEYKFQNALLVR.Y | Y | 79.27 | 2598.2900 | 21 | 1.0 | 867.1049 | 3 | 77.52 | 1 | F1:23878 | Fraction\_1\_23092022r.raw | 2.7648E5 |  |  |  |  |  |  | 1 | 1 | 0 | 0 | 0 | 0 | 0 | 0 | 414 | 434 | Carbamidomethylation | C3:Carbamidomethylation:1000.00 | PEAKS DB |
| K.KVPQVSTPTLVEVSR.N | N | 78.42 | 1638.9304 | 15 | 0.1 | 547.3174 | 3 | 46.27 | 1 | F1:9857 | Fraction\_1\_23092022r.raw | 2.9405E6 | 6.5876E5 | 2.0378E5 |  | 1.8424E5 |  |  | 4 | 1 | 1 | 1 | 0 | 1 | 0 | 0 | 438 | 452 |  |  | PEAKS DB |
| K.LVNEVTEFAK.T | N | 67.15 | 1148.6077 | 10 | -0.6 | 575.3108 | 2 | 47.29 | 1 | F1:10351 | Fraction\_1\_23092022r.raw | 6.3091E5 |  |  |  |  |  |  | 1 | 1 | 0 | 0 | 0 | 0 | 0 | 0 | 66 | 75 |  |  | PEAKS DB |
| K.AEFAEVSKLVTDLTK.V | Y | 66.69 | 1649.8876 | 15 | 0.6 | 550.9702 | 3 | 73.71 | 1 | F1:22169 | Fraction\_1\_23092022r.raw | 1.1675E5 |  |  |  |  |  |  | 1 | 1 | 0 | 0 | 0 | 0 | 0 | 0 | 250 | 264 |  |  | PEAKS DB |
| K.AVMDDFAAFVEK.C | Y | 61.31 | 1341.6274 | 12 | 0.4 | 671.8213 | 2 | 74.55 | 1 | F1:22531 | Fraction\_1\_23092022r.raw | 2.3926E5 |  |  |  |  |  |  | 1 | 1 | 0 | 0 | 0 | 0 | 0 | 0 | 570 | 581 |  |  | PEAKS DB |
| K.VPQVSTPTLVEVSR.N | N | 59.16 | 1510.8354 | 14 | 0.5 | 756.4254 | 2 | 53.78 | 3 | F3:13777 | Fraction\_3\_23092022.raw | 1.692E5 |  | 1.8626E5 |  |  |  |  | 2 | 1 | 0 | 1 | 0 | 0 | 0 | 0 | 439 | 452 |  |  | PEAKS DB |
| K.LVAASQAALGL | Y | 57.07 | 1012.5917 | 11 | 0.6 | 507.3034 | 2 | 65.53 | 1 | F1:18514 | Fraction\_1\_23092022r.raw | 2.3598E5 | 1.6952E5 |  |  |  |  |  | 2 | 1 | 1 | 0 | 0 | 0 | 0 | 0 | 599 | 609 |  |  | PEAKS DB |
| R.RHPDYSVVLLLR.L | Y | 52.15 | 1466.8357 | 12 | 0.4 | 489.9527 | 3 | 54.73 | 1 | F1:13663 | Fraction\_1\_23092022r.raw | 2.1143E5 |  |  |  |  |  |  | 1 | 1 | 0 | 0 | 0 | 0 | 0 | 0 | 361 | 372 |  |  | PEAKS DB |
| K.AVM(+15.99)DDFAAFVEK.C | Y | 48.87 | 1357.6223 | 12 | 0.3 | 679.8186 | 2 | 62.27 | 1 | F1:17060 | Fraction\_1\_23092022r.raw | 9.6033E4 |  |  |  |  |  |  | 1 | 1 | 0 | 0 | 0 | 0 | 0 | 0 | 570 | 581 | Oxidation (M) | M3:Oxidation (M):1000.00 | PEAKS DB |
| total 10 peptides |  |  |  |  |  |  |  |  |  |  |  |  |  |  |  |  |  |  |  |  |  |  |  |  |  |  |  |  |  |  |  |
| --- | --- | --- | --- | --- | --- | --- | --- | --- | --- | --- | --- | --- | --- | --- | --- | --- | --- | --- | --- | --- | --- | --- | --- | --- | --- | --- | --- | --- | --- | --- | --- |

P0AA29|THIO\_SALTI

back to list

  

| Protein Coverage
| Supporting Peptides
|

Protein Coverage:

Supporting Peptides:

| Peptide | Uniq | -10lgP | Mass | Length | ppm | m/z | z | RT | Fraction | Scan | Source File | Area F1 | Area F2 | Area F3 | Area F4 | Area F5 | Area F6 | Area F7 | #Feature | #Feature F1 | #Feature F2 | #Feature F3 | #Feature F4 | #Feature F5 | #Feature F6 | #Feature F7 | Start | End | PTM | AScore | Found By |
| --- | --- | --- | --- | --- | --- | --- | --- | --- | --- | --- | --- | --- | --- | --- | --- | --- | --- | --- | --- | --- | --- | --- | --- | --- | --- | --- | --- | --- | --- | --- | --- |
| K.MIAPILDEIADEYQGKLTVAK.L | Y | 94.00 | 2317.2239 | 21 | -0.3 | 773.4150 | 3 | 82.46 | 1 | F1:26078 | Fraction\_1\_23092022r.raw | 7.4787E5 |  |  |  |  |  |  | 1 | 1 | 0 | 0 | 0 | 0 | 0 | 0 | 38 | 58 |  |  | PEAKS DB |
| K.IIHLTDDSFDTDVLK.A | Y | 82.55 | 1730.8727 | 15 | -0.3 | 866.4434 | 2 | 61.84 | 1 | F1:16822 | Fraction\_1\_23092022r.raw | 4.6358E6 | 5.6366E5 |  |  |  |  |  | 3 | 2 | 1 | 0 | 0 | 0 | 0 | 0 | 5 | 19 |  |  | PEAKS DB |
| K.LTVAKLNIDQNPGTAPK.Y | Y | 78.51 | 1778.9890 | 17 | -0.1 | 594.0035 | 3 | 43.40 | 2 | F2:8717 | Fraction\_2\_23092022.raw | 3.6352E5 | 1.7834E6 |  |  |  |  |  | 3 | 1 | 2 | 0 | 0 | 0 | 0 | 0 | 54 | 70 |  |  | PEAKS DB |
| K.M(+15.99)IAPILDEIADEYQGK.L | Y | 73.06 | 1820.8866 | 16 | -0.3 | 911.4503 | 2 | 78.99 | 2 | F2:24679 | Fraction\_2\_23092022.raw | 5.1846E6 | 2.0664E6 | 4.9225E5 | 4.3955E5 |  |  |  | 4 | 1 | 1 | 1 | 1 | 0 | 0 | 0 | 38 | 53 | Oxidation (M) | M1:Oxidation (M):1000.00 | PEAKS DB |
| K.M(+15.99)IAPILDEIADEYQGKLTVAK.L | Y | 71.58 | 2333.2188 | 21 | 0.3 | 778.7471 | 3 | 78.29 | 2 | F2:24355 | Fraction\_2\_23092022.raw | 2.3234E6 | 2.6165E6 |  | 3.8839E5 |  |  |  | 3 | 1 | 1 | 0 | 1 | 0 | 0 | 0 | 38 | 58 | Oxidation (M) | M1:Oxidation (M):1000.00 | PEAKS DB |
| K.LNIDQNPGTAPKYGIR.G | Y | 68.53 | 1755.9268 | 16 | 0.2 | 586.3163 | 3 | 40.14 | 4 | F4:8222 | Fraction\_4\_23092022.raw | 9.9323E5 | 4.0151E6 | 2.1003E5 | 4.5004E5 |  |  |  | 6 | 2 | 2 | 1 | 1 | 0 | 0 | 0 | 59 | 74 |  |  | PEAKS DB |
| R.GIPTLLLFKNGEVAATK.V | Y | 68.38 | 1771.0243 | 17 | -0.1 | 886.5193 | 2 | 66.23 | 1 | F1:18826 | Fraction\_1\_23092022r.raw | 1.3031E5 | 4.627E6 | 2.6873E5 |  |  |  |  | 4 | 1 | 2 | 1 | 0 | 0 | 0 | 0 | 75 | 91 |  |  | PEAKS DB |
| K.M(+15.99)IAPILDEIADEYQGKLTVAKLNIDQNPGTAPK.Y | Y | 66.85 | 3581.8650 | 33 | -0.6 | 896.4730 | 4 | 78.74 | 2 | F2:24625 | Fraction\_2\_23092022.raw |  | 7.7061E5 |  |  |  |  |  | 1 | 0 | 1 | 0 | 0 | 0 | 0 | 0 | 38 | 70 | Oxidation (M) | M1:Oxidation (M):1000.00 | PEAKS DB |
| R.GIPTLLLFK.N | Y | 48.88 | 1000.6321 | 9 | -0.3 | 501.3232 | 2 | 78.13 | 4 | F4:25248 | Fraction\_4\_23092022.raw |  | 5.0567E6 |  | 1.2317E6 |  |  |  | 2 | 0 | 1 | 0 | 1 | 0 | 0 | 0 | 75 | 83 |  |  | PEAKS DB |
| total 9 peptides |  |  |  |  |  |  |  |  |  |  |  |  |  |  |  |  |  |  |  |  |  |  |  |  |  |  |  |  |  |  |  |
| --- | --- | --- | --- | --- | --- | --- | --- | --- | --- | --- | --- | --- | --- | --- | --- | --- | --- | --- | --- | --- | --- | --- | --- | --- | --- | --- | --- | --- | --- | --- | --- |

P0AA26|THIO\_ECOL6

back to list

  

| Protein Coverage
| Supporting Peptides
|

Protein Coverage:

Supporting Peptides:

| Peptide | Uniq | -10lgP | Mass | Length | ppm | m/z | z | RT | Fraction | Scan | Source File | Area F1 | Area F2 | Area F3 | Area F4 | Area F5 | Area F6 | Area F7 | #Feature | #Feature F1 | #Feature F2 | #Feature F3 | #Feature F4 | #Feature F5 | #Feature F6 | #Feature F7 | Start | End | PTM | AScore | Found By |
| --- | --- | --- | --- | --- | --- | --- | --- | --- | --- | --- | --- | --- | --- | --- | --- | --- | --- | --- | --- | --- | --- | --- | --- | --- | --- | --- | --- | --- | --- | --- | --- |
| K.MIAPILDEIADEYQGKLTVAK.L | Y | 94.00 | 2317.2239 | 21 | -0.3 | 773.4150 | 3 | 82.46 | 1 | F1:26078 | Fraction\_1\_23092022r.raw | 7.4787E5 |  |  |  |  |  |  | 1 | 1 | 0 | 0 | 0 | 0 | 0 | 0 | 38 | 58 |  |  | PEAKS DB |
| K.IIHLTDDSFDTDVLK.A | Y | 82.55 | 1730.8727 | 15 | -0.3 | 866.4434 | 2 | 61.84 | 1 | F1:16822 | Fraction\_1\_23092022r.raw | 4.6358E6 | 5.6366E5 |  |  |  |  |  | 3 | 2 | 1 | 0 | 0 | 0 | 0 | 0 | 5 | 19 |  |  | PEAKS DB |
| K.LTVAKLNIDQNPGTAPK.Y | Y | 78.51 | 1778.9890 | 17 | -0.1 | 594.0035 | 3 | 43.40 | 2 | F2:8717 | Fraction\_2\_23092022.raw | 3.6352E5 | 1.7834E6 |  |  |  |  |  | 3 | 1 | 2 | 0 | 0 | 0 | 0 | 0 | 54 | 70 |  |  | PEAKS DB |
| K.M(+15.99)IAPILDEIADEYQGK.L | Y | 73.06 | 1820.8866 | 16 | -0.3 | 911.4503 | 2 | 78.99 | 2 | F2:24679 | Fraction\_2\_23092022.raw | 5.1846E6 | 2.0664E6 | 4.9225E5 | 4.3955E5 |  |  |  | 4 | 1 | 1 | 1 | 1 | 0 | 0 | 0 | 38 | 53 | Oxidation (M) | M1:Oxidation (M):1000.00 | PEAKS DB |
| K.M(+15.99)IAPILDEIADEYQGKLTVAK.L | Y | 71.58 | 2333.2188 | 21 | 0.3 | 778.7471 | 3 | 78.29 | 2 | F2:24355 | Fraction\_2\_23092022.raw | 2.3234E6 | 2.6165E6 |  | 3.8839E5 |  |  |  | 3 | 1 | 1 | 0 | 1 | 0 | 0 | 0 | 38 | 58 | Oxidation (M) | M1:Oxidation (M):1000.00 | PEAKS DB |
| K.LNIDQNPGTAPKYGIR.G | Y | 68.53 | 1755.9268 | 16 | 0.2 | 586.3163 | 3 | 40.14 | 4 | F4:8222 | Fraction\_4\_23092022.raw | 9.9323E5 | 4.0151E6 | 2.1003E5 | 4.5004E5 |  |  |  | 6 | 2 | 2 | 1 | 1 | 0 | 0 | 0 | 59 | 74 |  |  | PEAKS DB |
| R.GIPTLLLFKNGEVAATK.V | Y | 68.38 | 1771.0243 | 17 | -0.1 | 886.5193 | 2 | 66.23 | 1 | F1:18826 | Fraction\_1\_23092022r.raw | 1.3031E5 | 4.627E6 | 2.6873E5 |  |  |  |  | 4 | 1 | 2 | 1 | 0 | 0 | 0 | 0 | 75 | 91 |  |  | PEAKS DB |
| K.M(+15.99)IAPILDEIADEYQGKLTVAKLNIDQNPGTAPK.Y | Y | 66.85 | 3581.8650 | 33 | -0.6 | 896.4730 | 4 | 78.74 | 2 | F2:24625 | Fraction\_2\_23092022.raw |  | 7.7061E5 |  |  |  |  |  | 1 | 0 | 1 | 0 | 0 | 0 | 0 | 0 | 38 | 70 | Oxidation (M) | M1:Oxidation (M):1000.00 | PEAKS DB |
| R.GIPTLLLFK.N | Y | 48.88 | 1000.6321 | 9 | -0.3 | 501.3232 | 2 | 78.13 | 4 | F4:25248 | Fraction\_4\_23092022.raw |  | 5.0567E6 |  | 1.2317E6 |  |  |  | 2 | 0 | 1 | 0 | 1 | 0 | 0 | 0 | 75 | 83 |  |  | PEAKS DB |
| total 9 peptides |  |  |  |  |  |  |  |  |  |  |  |  |  |  |  |  |  |  |  |  |  |  |  |  |  |  |  |  |  |  |  |
| --- | --- | --- | --- | --- | --- | --- | --- | --- | --- | --- | --- | --- | --- | --- | --- | --- | --- | --- | --- | --- | --- | --- | --- | --- | --- | --- | --- | --- | --- | --- | --- |

P0AA27|THIO\_ECO57

back to list

  

| Protein Coverage
| Supporting Peptides
|

Protein Coverage:

Supporting Peptides:

| Peptide | Uniq | -10lgP | Mass | Length | ppm | m/z | z | RT | Fraction | Scan | Source File | Area F1 | Area F2 | Area F3 | Area F4 | Area F5 | Area F6 | Area F7 | #Feature | #Feature F1 | #Feature F2 | #Feature F3 | #Feature F4 | #Feature F5 | #Feature F6 | #Feature F7 | Start | End | PTM | AScore | Found By |
| --- | --- | --- | --- | --- | --- | --- | --- | --- | --- | --- | --- | --- | --- | --- | --- | --- | --- | --- | --- | --- | --- | --- | --- | --- | --- | --- | --- | --- | --- | --- | --- |
| K.MIAPILDEIADEYQGKLTVAK.L | Y | 94.00 | 2317.2239 | 21 | -0.3 | 773.4150 | 3 | 82.46 | 1 | F1:26078 | Fraction\_1\_23092022r.raw | 7.4787E5 |  |  |  |  |  |  | 1 | 1 | 0 | 0 | 0 | 0 | 0 | 0 | 38 | 58 |  |  | PEAKS DB |
| K.IIHLTDDSFDTDVLK.A | Y | 82.55 | 1730.8727 | 15 | -0.3 | 866.4434 | 2 | 61.84 | 1 | F1:16822 | Fraction\_1\_23092022r.raw | 4.6358E6 | 5.6366E5 |  |  |  |  |  | 3 | 2 | 1 | 0 | 0 | 0 | 0 | 0 | 5 | 19 |  |  | PEAKS DB |
| K.LTVAKLNIDQNPGTAPK.Y | Y | 78.51 | 1778.9890 | 17 | -0.1 | 594.0035 | 3 | 43.40 | 2 | F2:8717 | Fraction\_2\_23092022.raw | 3.6352E5 | 1.7834E6 |  |  |  |  |  | 3 | 1 | 2 | 0 | 0 | 0 | 0 | 0 | 54 | 70 |  |  | PEAKS DB |
| K.M(+15.99)IAPILDEIADEYQGK.L | Y | 73.06 | 1820.8866 | 16 | -0.3 | 911.4503 | 2 | 78.99 | 2 | F2:24679 | Fraction\_2\_23092022.raw | 5.1846E6 | 2.0664E6 | 4.9225E5 | 4.3955E5 |  |  |  | 4 | 1 | 1 | 1 | 1 | 0 | 0 | 0 | 38 | 53 | Oxidation (M) | M1:Oxidation (M):1000.00 | PEAKS DB |
| K.M(+15.99)IAPILDEIADEYQGKLTVAK.L | Y | 71.58 | 2333.2188 | 21 | 0.3 | 778.7471 | 3 | 78.29 | 2 | F2:24355 | Fraction\_2\_23092022.raw | 2.3234E6 | 2.6165E6 |  | 3.8839E5 |  |  |  | 3 | 1 | 1 | 0 | 1 | 0 | 0 | 0 | 38 | 58 | Oxidation (M) | M1:Oxidation (M):1000.00 | PEAKS DB |
| K.LNIDQNPGTAPKYGIR.G | Y | 68.53 | 1755.9268 | 16 | 0.2 | 586.3163 | 3 | 40.14 | 4 | F4:8222 | Fraction\_4\_23092022.raw | 9.9323E5 | 4.0151E6 | 2.1003E5 | 4.5004E5 |  |  |  | 6 | 2 | 2 | 1 | 1 | 0 | 0 | 0 | 59 | 74 |  |  | PEAKS DB |
| R.GIPTLLLFKNGEVAATK.V | Y | 68.38 | 1771.0243 | 17 | -0.1 | 886.5193 | 2 | 66.23 | 1 | F1:18826 | Fraction\_1\_23092022r.raw | 1.3031E5 | 4.627E6 | 2.6873E5 |  |  |  |  | 4 | 1 | 2 | 1 | 0 | 0 | 0 | 0 | 75 | 91 |  |  | PEAKS DB |
| K.M(+15.99)IAPILDEIADEYQGKLTVAKLNIDQNPGTAPK.Y | Y | 66.85 | 3581.8650 | 33 | -0.6 | 896.4730 | 4 | 78.74 | 2 | F2:24625 | Fraction\_2\_23092022.raw |  | 7.7061E5 |  |  |  |  |  | 1 | 0 | 1 | 0 | 0 | 0 | 0 | 0 | 38 | 70 | Oxidation (M) | M1:Oxidation (M):1000.00 | PEAKS DB |
| R.GIPTLLLFK.N | Y | 48.88 | 1000.6321 | 9 | -0.3 | 501.3232 | 2 | 78.13 | 4 | F4:25248 | Fraction\_4\_23092022.raw |  | 5.0567E6 |  | 1.2317E6 |  |  |  | 2 | 0 | 1 | 0 | 1 | 0 | 0 | 0 | 75 | 83 |  |  | PEAKS DB |
| total 9 peptides |  |  |  |  |  |  |  |  |  |  |  |  |  |  |  |  |  |  |  |  |  |  |  |  |  |  |  |  |  |  |  |
| --- | --- | --- | --- | --- | --- | --- | --- | --- | --- | --- | --- | --- | --- | --- | --- | --- | --- | --- | --- | --- | --- | --- | --- | --- | --- | --- | --- | --- | --- | --- | --- |

P0AA30|THIO\_SHIFL

back to list

  

| Protein Coverage
| Supporting Peptides
|

Protein Coverage:

Supporting Peptides:

| Peptide | Uniq | -10lgP | Mass | Length | ppm | m/z | z | RT | Fraction | Scan | Source File | Area F1 | Area F2 | Area F3 | Area F4 | Area F5 | Area F6 | Area F7 | #Feature | #Feature F1 | #Feature F2 | #Feature F3 | #Feature F4 | #Feature F5 | #Feature F6 | #Feature F7 | Start | End | PTM | AScore | Found By |
| --- | --- | --- | --- | --- | --- | --- | --- | --- | --- | --- | --- | --- | --- | --- | --- | --- | --- | --- | --- | --- | --- | --- | --- | --- | --- | --- | --- | --- | --- | --- | --- |
| K.MIAPILDEIADEYQGKLTVAK.L | Y | 94.00 | 2317.2239 | 21 | -0.3 | 773.4150 | 3 | 82.46 | 1 | F1:26078 | Fraction\_1\_23092022r.raw | 7.4787E5 |  |  |  |  |  |  | 1 | 1 | 0 | 0 | 0 | 0 | 0 | 0 | 38 | 58 |  |  | PEAKS DB |
| K.IIHLTDDSFDTDVLK.A | Y | 82.55 | 1730.8727 | 15 | -0.3 | 866.4434 | 2 | 61.84 | 1 | F1:16822 | Fraction\_1\_23092022r.raw | 4.6358E6 | 5.6366E5 |  |  |  |  |  | 3 | 2 | 1 | 0 | 0 | 0 | 0 | 0 | 5 | 19 |  |  | PEAKS DB |
| K.LTVAKLNIDQNPGTAPK.Y | Y | 78.51 | 1778.9890 | 17 | -0.1 | 594.0035 | 3 | 43.40 | 2 | F2:8717 | Fraction\_2\_23092022.raw | 3.6352E5 | 1.7834E6 |  |  |  |  |  | 3 | 1 | 2 | 0 | 0 | 0 | 0 | 0 | 54 | 70 |  |  | PEAKS DB |
| K.M(+15.99)IAPILDEIADEYQGK.L | Y | 73.06 | 1820.8866 | 16 | -0.3 | 911.4503 | 2 | 78.99 | 2 | F2:24679 | Fraction\_2\_23092022.raw | 5.1846E6 | 2.0664E6 | 4.9225E5 | 4.3955E5 |  |  |  | 4 | 1 | 1 | 1 | 1 | 0 | 0 | 0 | 38 | 53 | Oxidation (M) | M1:Oxidation (M):1000.00 | PEAKS DB |
| K.M(+15.99)IAPILDEIADEYQGKLTVAK.L | Y | 71.58 | 2333.2188 | 21 | 0.3 | 778.7471 | 3 | 78.29 | 2 | F2:24355 | Fraction\_2\_23092022.raw | 2.3234E6 | 2.6165E6 |  | 3.8839E5 |  |  |  | 3 | 1 | 1 | 0 | 1 | 0 | 0 | 0 | 38 | 58 | Oxidation (M) | M1:Oxidation (M):1000.00 | PEAKS DB |
| K.LNIDQNPGTAPKYGIR.G | Y | 68.53 | 1755.9268 | 16 | 0.2 | 586.3163 | 3 | 40.14 | 4 | F4:8222 | Fraction\_4\_23092022.raw | 9.9323E5 | 4.0151E6 | 2.1003E5 | 4.5004E5 |  |  |  | 6 | 2 | 2 | 1 | 1 | 0 | 0 | 0 | 59 | 74 |  |  | PEAKS DB |
| R.GIPTLLLFKNGEVAATK.V | Y | 68.38 | 1771.0243 | 17 | -0.1 | 886.5193 | 2 | 66.23 | 1 | F1:18826 | Fraction\_1\_23092022r.raw | 1.3031E5 | 4.627E6 | 2.6873E5 |  |  |  |  | 4 | 1 | 2 | 1 | 0 | 0 | 0 | 0 | 75 | 91 |  |  | PEAKS DB |
| K.M(+15.99)IAPILDEIADEYQGKLTVAKLNIDQNPGTAPK.Y | Y | 66.85 | 3581.8650 | 33 | -0.6 | 896.4730 | 4 | 78.74 | 2 | F2:24625 | Fraction\_2\_23092022.raw |  | 7.7061E5 |  |  |  |  |  | 1 | 0 | 1 | 0 | 0 | 0 | 0 | 0 | 38 | 70 | Oxidation (M) | M1:Oxidation (M):1000.00 | PEAKS DB |
| R.GIPTLLLFK.N | Y | 48.88 | 1000.6321 | 9 | -0.3 | 501.3232 | 2 | 78.13 | 4 | F4:25248 | Fraction\_4\_23092022.raw |  | 5.0567E6 |  | 1.2317E6 |  |  |  | 2 | 0 | 1 | 0 | 1 | 0 | 0 | 0 | 75 | 83 |  |  | PEAKS DB |
| total 9 peptides |  |  |  |  |  |  |  |  |  |  |  |  |  |  |  |  |  |  |  |  |  |  |  |  |  |  |  |  |  |  |  |
| --- | --- | --- | --- | --- | --- | --- | --- | --- | --- | --- | --- | --- | --- | --- | --- | --- | --- | --- | --- | --- | --- | --- | --- | --- | --- | --- | --- | --- | --- | --- | --- |

P0AA25|THIO\_ECOLI

back to list

  

| Protein Coverage
| Supporting Peptides
|

Protein Coverage:

Supporting Peptides:

| Peptide | Uniq | -10lgP | Mass | Length | ppm | m/z | z | RT | Fraction | Scan | Source File | Area F1 | Area F2 | Area F3 | Area F4 | Area F5 | Area F6 | Area F7 | #Feature | #Feature F1 | #Feature F2 | #Feature F3 | #Feature F4 | #Feature F5 | #Feature F6 | #Feature F7 | Start | End | PTM | AScore | Found By |
| --- | --- | --- | --- | --- | --- | --- | --- | --- | --- | --- | --- | --- | --- | --- | --- | --- | --- | --- | --- | --- | --- | --- | --- | --- | --- | --- | --- | --- | --- | --- | --- |
| K.MIAPILDEIADEYQGKLTVAK.L | Y | 94.00 | 2317.2239 | 21 | -0.3 | 773.4150 | 3 | 82.46 | 1 | F1:26078 | Fraction\_1\_23092022r.raw | 7.4787E5 |  |  |  |  |  |  | 1 | 1 | 0 | 0 | 0 | 0 | 0 | 0 | 38 | 58 |  |  | PEAKS DB |
| K.IIHLTDDSFDTDVLK.A | Y | 82.55 | 1730.8727 | 15 | -0.3 | 866.4434 | 2 | 61.84 | 1 | F1:16822 | Fraction\_1\_23092022r.raw | 4.6358E6 | 5.6366E5 |  |  |  |  |  | 3 | 2 | 1 | 0 | 0 | 0 | 0 | 0 | 5 | 19 |  |  | PEAKS DB |
| K.LTVAKLNIDQNPGTAPK.Y | Y | 78.51 | 1778.9890 | 17 | -0.1 | 594.0035 | 3 | 43.40 | 2 | F2:8717 | Fraction\_2\_23092022.raw | 3.6352E5 | 1.7834E6 |  |  |  |  |  | 3 | 1 | 2 | 0 | 0 | 0 | 0 | 0 | 54 | 70 |  |  | PEAKS DB |
| K.M(+15.99)IAPILDEIADEYQGK.L | Y | 73.06 | 1820.8866 | 16 | -0.3 | 911.4503 | 2 | 78.99 | 2 | F2:24679 | Fraction\_2\_23092022.raw | 5.1846E6 | 2.0664E6 | 4.9225E5 | 4.3955E5 |  |  |  | 4 | 1 | 1 | 1 | 1 | 0 | 0 | 0 | 38 | 53 | Oxidation (M) | M1:Oxidation (M):1000.00 | PEAKS DB |
| K.M(+15.99)IAPILDEIADEYQGKLTVAK.L | Y | 71.58 | 2333.2188 | 21 | 0.3 | 778.7471 | 3 | 78.29 | 2 | F2:24355 | Fraction\_2\_23092022.raw | 2.3234E6 | 2.6165E6 |  | 3.8839E5 |  |  |  | 3 | 1 | 1 | 0 | 1 | 0 | 0 | 0 | 38 | 58 | Oxidation (M) | M1:Oxidation (M):1000.00 | PEAKS DB |
| K.LNIDQNPGTAPKYGIR.G | Y | 68.53 | 1755.9268 | 16 | 0.2 | 586.3163 | 3 | 40.14 | 4 | F4:8222 | Fraction\_4\_23092022.raw | 9.9323E5 | 4.0151E6 | 2.1003E5 | 4.5004E5 |  |  |  | 6 | 2 | 2 | 1 | 1 | 0 | 0 | 0 | 59 | 74 |  |  | PEAKS DB |
| R.GIPTLLLFKNGEVAATK.V | Y | 68.38 | 1771.0243 | 17 | -0.1 | 886.5193 | 2 | 66.23 | 1 | F1:18826 | Fraction\_1\_23092022r.raw | 1.3031E5 | 4.627E6 | 2.6873E5 |  |  |  |  | 4 | 1 | 2 | 1 | 0 | 0 | 0 | 0 | 75 | 91 |  |  | PEAKS DB |
| K.M(+15.99)IAPILDEIADEYQGKLTVAKLNIDQNPGTAPK.Y | Y | 66.85 | 3581.8650 | 33 | -0.6 | 896.4730 | 4 | 78.74 | 2 | F2:24625 | Fraction\_2\_23092022.raw |  | 7.7061E5 |  |  |  |  |  | 1 | 0 | 1 | 0 | 0 | 0 | 0 | 0 | 38 | 70 | Oxidation (M) | M1:Oxidation (M):1000.00 | PEAKS DB |
| R.GIPTLLLFK.N | Y | 48.88 | 1000.6321 | 9 | -0.3 | 501.3232 | 2 | 78.13 | 4 | F4:25248 | Fraction\_4\_23092022.raw |  | 5.0567E6 |  | 1.2317E6 |  |  |  | 2 | 0 | 1 | 0 | 1 | 0 | 0 | 0 | 75 | 83 |  |  | PEAKS DB |
| total 9 peptides |  |  |  |  |  |  |  |  |  |  |  |  |  |  |  |  |  |  |  |  |  |  |  |  |  |  |  |  |  |  |  |
| --- | --- | --- | --- | --- | --- | --- | --- | --- | --- | --- | --- | --- | --- | --- | --- | --- | --- | --- | --- | --- | --- | --- | --- | --- | --- | --- | --- | --- | --- | --- | --- |

P0AA28|THIO\_SALTY

back to list

  

| Protein Coverage
| Supporting Peptides
|

Protein Coverage:

Supporting Peptides:

| Peptide | Uniq | -10lgP | Mass | Length | ppm | m/z | z | RT | Fraction | Scan | Source File | Area F1 | Area F2 | Area F3 | Area F4 | Area F5 | Area F6 | Area F7 | #Feature | #Feature F1 | #Feature F2 | #Feature F3 | #Feature F4 | #Feature F5 | #Feature F6 | #Feature F7 | Start | End | PTM | AScore | Found By |
| --- | --- | --- | --- | --- | --- | --- | --- | --- | --- | --- | --- | --- | --- | --- | --- | --- | --- | --- | --- | --- | --- | --- | --- | --- | --- | --- | --- | --- | --- | --- | --- |
| K.MIAPILDEIADEYQGKLTVAK.L | Y | 94.00 | 2317.2239 | 21 | -0.3 | 773.4150 | 3 | 82.46 | 1 | F1:26078 | Fraction\_1\_23092022r.raw | 7.4787E5 |  |  |  |  |  |  | 1 | 1 | 0 | 0 | 0 | 0 | 0 | 0 | 38 | 58 |  |  | PEAKS DB |
| K.IIHLTDDSFDTDVLK.A | Y | 82.55 | 1730.8727 | 15 | -0.3 | 866.4434 | 2 | 61.84 | 1 | F1:16822 | Fraction\_1\_23092022r.raw | 4.6358E6 | 5.6366E5 |  |  |  |  |  | 3 | 2 | 1 | 0 | 0 | 0 | 0 | 0 | 5 | 19 |  |  | PEAKS DB |
| K.LTVAKLNIDQNPGTAPK.Y | Y | 78.51 | 1778.9890 | 17 | -0.1 | 594.0035 | 3 | 43.40 | 2 | F2:8717 | Fraction\_2\_23092022.raw | 3.6352E5 | 1.7834E6 |  |  |  |  |  | 3 | 1 | 2 | 0 | 0 | 0 | 0 | 0 | 54 | 70 |  |  | PEAKS DB |
| K.M(+15.99)IAPILDEIADEYQGK.L | Y | 73.06 | 1820.8866 | 16 | -0.3 | 911.4503 | 2 | 78.99 | 2 | F2:24679 | Fraction\_2\_23092022.raw | 5.1846E6 | 2.0664E6 | 4.9225E5 | 4.3955E5 |  |  |  | 4 | 1 | 1 | 1 | 1 | 0 | 0 | 0 | 38 | 53 | Oxidation (M) | M1:Oxidation (M):1000.00 | PEAKS DB |
| K.M(+15.99)IAPILDEIADEYQGKLTVAK.L | Y | 71.58 | 2333.2188 | 21 | 0.3 | 778.7471 | 3 | 78.29 | 2 | F2:24355 | Fraction\_2\_23092022.raw | 2.3234E6 | 2.6165E6 |  | 3.8839E5 |  |  |  | 3 | 1 | 1 | 0 | 1 | 0 | 0 | 0 | 38 | 58 | Oxidation (M) | M1:Oxidation (M):1000.00 | PEAKS DB |
| K.LNIDQNPGTAPKYGIR.G | Y | 68.53 | 1755.9268 | 16 | 0.2 | 586.3163 | 3 | 40.14 | 4 | F4:8222 | Fraction\_4\_23092022.raw | 9.9323E5 | 4.0151E6 | 2.1003E5 | 4.5004E5 |  |  |  | 6 | 2 | 2 | 1 | 1 | 0 | 0 | 0 | 59 | 74 |  |  | PEAKS DB |
| R.GIPTLLLFKNGEVAATK.V | Y | 68.38 | 1771.0243 | 17 | -0.1 | 886.5193 | 2 | 66.23 | 1 | F1:18826 | Fraction\_1\_23092022r.raw | 1.3031E5 | 4.627E6 | 2.6873E5 |  |  |  |  | 4 | 1 | 2 | 1 | 0 | 0 | 0 | 0 | 75 | 91 |  |  | PEAKS DB |
| K.M(+15.99)IAPILDEIADEYQGKLTVAKLNIDQNPGTAPK.Y | Y | 66.85 | 3581.8650 | 33 | -0.6 | 896.4730 | 4 | 78.74 | 2 | F2:24625 | Fraction\_2\_23092022.raw |  | 7.7061E5 |  |  |  |  |  | 1 | 0 | 1 | 0 | 0 | 0 | 0 | 0 | 38 | 70 | Oxidation (M) | M1:Oxidation (M):1000.00 | PEAKS DB |
| R.GIPTLLLFK.N | Y | 48.88 | 1000.6321 | 9 | -0.3 | 501.3232 | 2 | 78.13 | 4 | F4:25248 | Fraction\_4\_23092022.raw |  | 5.0567E6 |  | 1.2317E6 |  |  |  | 2 | 0 | 1 | 0 | 1 | 0 | 0 | 0 | 75 | 83 |  |  | PEAKS DB |
| total 9 peptides |  |  |  |  |  |  |  |  |  |  |  |  |  |  |  |  |  |  |  |  |  |  |  |  |  |  |  |  |  |  |  |
| --- | --- | --- | --- | --- | --- | --- | --- | --- | --- | --- | --- | --- | --- | --- | --- | --- | --- | --- | --- | --- | --- | --- | --- | --- | --- | --- | --- | --- | --- | --- | --- |

TRINITY\_DN1494\_c0\_g1\_i8.p1

back to list

  

| Protein Coverage
| Supporting Peptides
|

Protein Coverage:

Supporting Peptides:

| Peptide | Uniq | -10lgP | Mass | Length | ppm | m/z | z | RT | Fraction | Scan | Source File | Area F1 | Area F2 | Area F3 | Area F4 | Area F5 | Area F6 | Area F7 | #Feature | #Feature F1 | #Feature F2 | #Feature F3 | #Feature F4 | #Feature F5 | #Feature F6 | #Feature F7 | Start | End | PTM | AScore | Found By |
| --- | --- | --- | --- | --- | --- | --- | --- | --- | --- | --- | --- | --- | --- | --- | --- | --- | --- | --- | --- | --- | --- | --- | --- | --- | --- | --- | --- | --- | --- | --- | --- |
| R.SNRVFDLGIISYGSTC(+57.02)ADNSPGVNTK.I | Y | 112.90 | 2771.3184 | 26 | -0.4 | 924.7797 | 3 | 63.64 | 3 | F3:18213 | Fraction\_3\_23092022.raw |  |  | 1.7606E6 | 4.8131E4 |  |  |  | 2 | 0 | 0 | 1 | 1 | 0 | 0 | 0 | 372 | 397 | Carbamidomethylation | C16:Carbamidomethylation:1000.00 | PEAKS DB |
| K.FC(+57.02)GLNSFVVESISSK.M | Y | 102.85 | 1672.8130 | 15 | 1.0 | 837.4146 | 2 | 71.17 | 5 | F5:21870 | Fraction\_5\_23092022.raw |  |  |  | 7.1285E5 | 1.0534E6 |  |  | 2 | 0 | 0 | 0 | 1 | 1 | 0 | 0 | 128 | 142 | Carbamidomethylation | C2:Carbamidomethylation:1000.00 | PEAKS DB |
| K.ISNYLDFIVKSTPGETYC(+57.02)QVY | Y | 77.33 | 2496.1882 | 21 | 0.9 | 1249.1025 | 2 | 82.39 | 3 | F3:26625 | Fraction\_3\_23092022.raw |  |  | 1.6798E6 |  |  |  |  | 1 | 0 | 0 | 1 | 0 | 0 | 0 | 0 | 398 | 418 | Carbamidomethylation | C18:Carbamidomethylation:1000.00 | PEAKS DB |
| R.VFDLGIISYGSTC(+57.02)ADNSPGVNTK.I | Y | 70.84 | 2414.1423 | 23 | -0.9 | 1208.0774 | 2 | 72.86 | 3 | F3:22346 | Fraction\_3\_23092022.raw |  |  | 1.6253E6 |  |  |  |  | 3 | 0 | 0 | 3 | 0 | 0 | 0 | 0 | 375 | 397 | Carbamidomethylation | C13:Carbamidomethylation:1000.00 | PEAKS DB |
| K.ISNYLDFIVK.S | Y | 61.49 | 1210.6598 | 10 | -0.2 | 606.3370 | 2 | 72.84 | 3 | F3:22313 | Fraction\_3\_23092022.raw |  |  | 1.4421E7 |  |  |  | 6.8673E5 | 2 | 0 | 0 | 1 | 0 | 0 | 0 | 1 | 398 | 407 |  |  | PEAKS DB |
| total 5 peptides |  |  |  |  |  |  |  |  |  |  |  |  |  |  |  |  |  |  |  |  |  |  |  |  |  |  |  |  |  |  |  |
| --- | --- | --- | --- | --- | --- | --- | --- | --- | --- | --- | --- | --- | --- | --- | --- | --- | --- | --- | --- | --- | --- | --- | --- | --- | --- | --- | --- | --- | --- | --- | --- |

P08779|K1C16\_HUMAN

back to list

  

| Protein Coverage
| Supporting Peptides
|

Protein Coverage:

Supporting Peptides:

| Peptide | Uniq | -10lgP | Mass | Length | ppm | m/z | z | RT | Fraction | Scan | Source File | Area F1 | Area F2 | Area F3 | Area F4 | Area F5 | Area F6 | Area F7 | #Feature | #Feature F1 | #Feature F2 | #Feature F3 | #Feature F4 | #Feature F5 | #Feature F6 | #Feature F7 | Start | End | PTM | AScore | Found By |
| --- | --- | --- | --- | --- | --- | --- | --- | --- | --- | --- | --- | --- | --- | --- | --- | --- | --- | --- | --- | --- | --- | --- | --- | --- | --- | --- | --- | --- | --- | --- | --- |
| K.IIAATIENAQPILQIDNAR.L | Y | 95.10 | 2063.1375 | 19 | 0.2 | 1032.5762 | 2 | 69.02 | 1 | F1:20068 | Fraction\_1\_23092022r.raw | 5.8134E5 |  | 2.1668E5 |  |  |  |  | 4 | 2 | 0 | 2 | 0 | 0 | 0 | 0 | 178 | 196 |  |  | PEAKS DB |
| R.ALEEANADLEVK.I | N | 74.72 | 1300.6510 | 12 | 0.5 | 651.3331 | 2 | 40.27 | 3 | F3:7722 | Fraction\_3\_23092022.raw | 4.9827E5 | 2.0105E5 | 6.9699E5 | 6.4208E4 | 5.161E4 | 1.2986E5 |  | 6 | 1 | 1 | 1 | 1 | 1 | 1 | 0 | 137 | 148 |  |  | PEAKS DB |
| R.LLEGEDAHLSSQQASGQSYSSR.E | Y | 61.77 | 2349.0833 | 22 | 0.6 | 784.0355 | 3 | 35.11 | 1 | F1:5497 | Fraction\_1\_23092022r.raw | 7.0078E4 |  |  |  |  |  |  | 1 | 1 | 0 | 0 | 0 | 0 | 0 | 0 | 420 | 441 |  |  | PEAKS DB |
| R.ISSVLAGGSC(+57.02)RAPSTYGGGLSVSSR.F | Y | 60.37 | 2425.2019 | 25 | -0.6 | 809.4074 | 3 | 45.90 | 7 | F7:13457 | Fraction\_7\_23092022.raw |  | 1.265E5 |  |  |  |  | 1.6022E5 | 2 | 0 | 1 | 0 | 0 | 0 | 0 | 1 | 31 | 55 | Carbamidomethylation | C10:Carbamidomethylation:1000.00 | PEAKS DB |
| K.VTM(+15.99)QNLNDRLASYLDKVR.A | N | 58.54 | 2151.1106 | 18 | 0.9 | 538.7854 | 4 | 56.01 | 1 | F1:14301 | Fraction\_1\_23092022r.raw | 7.88E6 | 8.0553E5 |  |  |  |  |  | 3 | 2 | 1 | 0 | 0 | 0 | 0 | 0 | 119 | 136 | Oxidation (M) | M3:Oxidation (M):1000.00 | PEAKS DB |
| K.VTMQNLNDRLASYLDKVR.A | N | 58.08 | 2135.1157 | 18 | 0.3 | 712.7127 | 3 | 63.69 | 1 | F1:17659 | Fraction\_1\_23092022r.raw | 2.701E6 |  |  |  |  |  |  | 2 | 2 | 0 | 0 | 0 | 0 | 0 | 0 | 119 | 136 |  |  | PEAKS DB |
| K.VTMQNLNDRLASYLDK.V | N | 56.99 | 1879.9462 | 16 | -0.8 | 627.6555 | 3 | 61.55 | 1 | F1:16687 | Fraction\_1\_23092022r.raw | 5.9999E5 |  |  |  |  |  |  | 1 | 1 | 0 | 0 | 0 | 0 | 0 | 0 | 119 | 134 |  |  | PEAKS DB |
| R.APSTYGGGLSVSSR.F | Y | 53.90 | 1337.6575 | 14 | 0.8 | 669.8365 | 2 | 34.95 | 6 | F6:6355 | Fraction\_6\_23092022.raw |  |  |  |  |  | 1.0062E5 |  | 1 | 0 | 0 | 0 | 0 | 0 | 1 | 0 | 42 | 55 |  |  | PEAKS DB |
| total 8 peptides |  |  |  |  |  |  |  |  |  |  |  |  |  |  |  |  |  |  |  |  |  |  |  |  |  |  |  |  |  |  |  |
| --- | --- | --- | --- | --- | --- | --- | --- | --- | --- | --- | --- | --- | --- | --- | --- | --- | --- | --- | --- | --- | --- | --- | --- | --- | --- | --- | --- | --- | --- | --- | --- |

TRINITY\_DN1931\_c0\_g1\_i1.p1

back to list

  

| Protein Coverage
| Supporting Peptides
|

Protein Coverage:

Supporting Peptides:

| Peptide | Uniq | -10lgP | Mass | Length | ppm | m/z | z | RT | Fraction | Scan | Source File | Area F1 | Area F2 | Area F3 | Area F4 | Area F5 | Area F6 | Area F7 | #Feature | #Feature F1 | #Feature F2 | #Feature F3 | #Feature F4 | #Feature F5 | #Feature F6 | #Feature F7 | Start | End | PTM | AScore | Found By |
| --- | --- | --- | --- | --- | --- | --- | --- | --- | --- | --- | --- | --- | --- | --- | --- | --- | --- | --- | --- | --- | --- | --- | --- | --- | --- | --- | --- | --- | --- | --- | --- |
| K.GTLYFEQTNESQPVQVTGEVSGLKK.G | Y | 89.15 | 2738.3762 | 25 | 0.5 | 913.7998 | 3 | 54.51 | 6 | F6:15080 | Fraction\_6\_23092022.raw |  |  |  |  |  | 6.2755E5 |  | 1 | 0 | 0 | 0 | 0 | 0 | 1 | 0 | 16 | 40 |  |  | PEAKS DB |
| R.TLVVHADPDDLGKGGHELSKTTGNAGGR.L | Y | 86.66 | 2801.4055 | 28 | -0.4 | 561.2881 | 5 | 33.04 | 6 | F6:5615 | Fraction\_6\_23092022.raw |  |  |  |  |  | 1.1091E6 |  | 3 | 0 | 0 | 0 | 0 | 0 | 3 | 0 | 116 | 143 |  |  | PEAKS DB |
| R.TLVVHADPDDLGKGGHELSK.T | Y | 80.08 | 2087.0647 | 20 | -0.4 | 522.7733 | 4 | 34.45 | 6 | F6:6156 | Fraction\_6\_23092022.raw |  |  |  |  |  | 2.104E6 |  | 3 | 0 | 0 | 0 | 0 | 0 | 3 | 0 | 116 | 135 |  |  | PEAKS DB |
| R.HVGDLGNIEAGDNGVAK.I | Y | 71.75 | 1664.8118 | 17 | 0.4 | 555.9448 | 3 | 36.17 | 6 | F6:6849 | Fraction\_6\_23092022.raw |  |  |  |  |  | 2.6132E5 |  | 1 | 0 | 0 | 0 | 0 | 0 | 1 | 0 | 80 | 96 |  |  | PEAKS DB |
| R.LAC(+57.02)GVIGITKSG | Y | 63.71 | 1174.6379 | 12 | 0.6 | 588.3266 | 2 | 43.93 | 6 | F6:10320 | Fraction\_6\_23092022.raw |  |  |  |  |  | 2.6798E5 |  | 1 | 0 | 0 | 0 | 0 | 0 | 1 | 0 | 144 | 155 | Carbamidomethylation | C3:Carbamidomethylation:1000.00 | PEAKS DB |
| R.LAC(+57.02)GVIGITK.S | Y | 62.68 | 1030.5845 | 10 | 0.3 | 516.2997 | 2 | 44.08 | 6 | F6:10377 | Fraction\_6\_23092022.raw |  |  |  |  |  | 7.6327E5 |  | 1 | 0 | 0 | 0 | 0 | 0 | 1 | 0 | 144 | 153 | Carbamidomethylation | C3:Carbamidomethylation:1000.00 | PEAKS DB |
| K.TTGNAGGRLAC(+57.02)GVIGITKSG | Y | 57.81 | 1888.9789 | 20 | 0.1 | 630.6670 | 3 | 43.16 | 6 | F6:9980 | Fraction\_6\_23092022.raw |  |  |  |  |  | 7.2697E4 |  | 1 | 0 | 0 | 0 | 0 | 0 | 1 | 0 | 136 | 155 | Carbamidomethylation | C11:Carbamidomethylation:1000.00 | PEAKS DB |
| total 7 peptides |  |  |  |  |  |  |  |  |  |  |  |  |  |  |  |  |  |  |  |  |  |  |  |  |  |  |  |  |  |  |  |
| --- | --- | --- | --- | --- | --- | --- | --- | --- | --- | --- | --- | --- | --- | --- | --- | --- | --- | --- | --- | --- | --- | --- | --- | --- | --- | --- | --- | --- | --- | --- | --- |

TRINITY\_DN1931\_c0\_g1\_i2.p1

back to list

  

| Protein Coverage
| Supporting Peptides
|

Protein Coverage:

Supporting Peptides:

| Peptide | Uniq | -10lgP | Mass | Length | ppm | m/z | z | RT | Fraction | Scan | Source File | Area F1 | Area F2 | Area F3 | Area F4 | Area F5 | Area F6 | Area F7 | #Feature | #Feature F1 | #Feature F2 | #Feature F3 | #Feature F4 | #Feature F5 | #Feature F6 | #Feature F7 | Start | End | PTM | AScore | Found By |
| --- | --- | --- | --- | --- | --- | --- | --- | --- | --- | --- | --- | --- | --- | --- | --- | --- | --- | --- | --- | --- | --- | --- | --- | --- | --- | --- | --- | --- | --- | --- | --- |
| K.GTLYFEQTNESQPVQVTGEVSGLKK.G | Y | 89.15 | 2738.3762 | 25 | 0.5 | 913.7998 | 3 | 54.51 | 6 | F6:15080 | Fraction\_6\_23092022.raw |  |  |  |  |  | 6.2755E5 |  | 1 | 0 | 0 | 0 | 0 | 0 | 1 | 0 | 16 | 40 |  |  | PEAKS DB |
| R.TLVVHADPDDLGKGGHELSKTTGNAGGR.L | Y | 86.66 | 2801.4055 | 28 | -0.4 | 561.2881 | 5 | 33.04 | 6 | F6:5615 | Fraction\_6\_23092022.raw |  |  |  |  |  | 1.1091E6 |  | 3 | 0 | 0 | 0 | 0 | 0 | 3 | 0 | 116 | 143 |  |  | PEAKS DB |
| R.TLVVHADPDDLGKGGHELSK.T | Y | 80.08 | 2087.0647 | 20 | -0.4 | 522.7733 | 4 | 34.45 | 6 | F6:6156 | Fraction\_6\_23092022.raw |  |  |  |  |  | 2.104E6 |  | 3 | 0 | 0 | 0 | 0 | 0 | 3 | 0 | 116 | 135 |  |  | PEAKS DB |
| R.HVGDLGNIEAGDNGVAK.I | Y | 71.75 | 1664.8118 | 17 | 0.4 | 555.9448 | 3 | 36.17 | 6 | F6:6849 | Fraction\_6\_23092022.raw |  |  |  |  |  | 2.6132E5 |  | 1 | 0 | 0 | 0 | 0 | 0 | 1 | 0 | 80 | 96 |  |  | PEAKS DB |
| R.LAC(+57.02)GVIGITKSG | Y | 63.71 | 1174.6379 | 12 | 0.6 | 588.3266 | 2 | 43.93 | 6 | F6:10320 | Fraction\_6\_23092022.raw |  |  |  |  |  | 2.6798E5 |  | 1 | 0 | 0 | 0 | 0 | 0 | 1 | 0 | 144 | 155 | Carbamidomethylation | C3:Carbamidomethylation:1000.00 | PEAKS DB |
| R.LAC(+57.02)GVIGITK.S | Y | 62.68 | 1030.5845 | 10 | 0.3 | 516.2997 | 2 | 44.08 | 6 | F6:10377 | Fraction\_6\_23092022.raw |  |  |  |  |  | 7.6327E5 |  | 1 | 0 | 0 | 0 | 0 | 0 | 1 | 0 | 144 | 153 | Carbamidomethylation | C3:Carbamidomethylation:1000.00 | PEAKS DB |
| K.TTGNAGGRLAC(+57.02)GVIGITKSG | Y | 57.81 | 1888.9789 | 20 | 0.1 | 630.6670 | 3 | 43.16 | 6 | F6:9980 | Fraction\_6\_23092022.raw |  |  |  |  |  | 7.2697E4 |  | 1 | 0 | 0 | 0 | 0 | 0 | 1 | 0 | 136 | 155 | Carbamidomethylation | C11:Carbamidomethylation:1000.00 | PEAKS DB |
| total 7 peptides |  |  |  |  |  |  |  |  |  |  |  |  |  |  |  |  |  |  |  |  |  |  |  |  |  |  |  |  |  |  |  |
| --- | --- | --- | --- | --- | --- | --- | --- | --- | --- | --- | --- | --- | --- | --- | --- | --- | --- | --- | --- | --- | --- | --- | --- | --- | --- | --- | --- | --- | --- | --- | --- |

TRINITY\_DN1931\_c0\_g1\_i4.p1

back to list

  

| Protein Coverage
| Supporting Peptides
|

Protein Coverage:

Supporting Peptides:

| Peptide | Uniq | -10lgP | Mass | Length | ppm | m/z | z | RT | Fraction | Scan | Source File | Area F1 | Area F2 | Area F3 | Area F4 | Area F5 | Area F6 | Area F7 | #Feature | #Feature F1 | #Feature F2 | #Feature F3 | #Feature F4 | #Feature F5 | #Feature F6 | #Feature F7 | Start | End | PTM | AScore | Found By |
| --- | --- | --- | --- | --- | --- | --- | --- | --- | --- | --- | --- | --- | --- | --- | --- | --- | --- | --- | --- | --- | --- | --- | --- | --- | --- | --- | --- | --- | --- | --- | --- |
| K.GTLYFEQTNESQPVQVTGEVSGLKK.G | Y | 89.15 | 2738.3762 | 25 | 0.5 | 913.7998 | 3 | 54.51 | 6 | F6:15080 | Fraction\_6\_23092022.raw |  |  |  |  |  | 6.2755E5 |  | 1 | 0 | 0 | 0 | 0 | 0 | 1 | 0 | 16 | 40 |  |  | PEAKS DB |
| R.TLVVHADPDDLGKGGHELSKTTGNAGGR.L | Y | 86.66 | 2801.4055 | 28 | -0.4 | 561.2881 | 5 | 33.04 | 6 | F6:5615 | Fraction\_6\_23092022.raw |  |  |  |  |  | 1.1091E6 |  | 3 | 0 | 0 | 0 | 0 | 0 | 3 | 0 | 116 | 143 |  |  | PEAKS DB |
| R.TLVVHADPDDLGKGGHELSK.T | Y | 80.08 | 2087.0647 | 20 | -0.4 | 522.7733 | 4 | 34.45 | 6 | F6:6156 | Fraction\_6\_23092022.raw |  |  |  |  |  | 2.104E6 |  | 3 | 0 | 0 | 0 | 0 | 0 | 3 | 0 | 116 | 135 |  |  | PEAKS DB |
| R.HVGDLGNIEAGDNGVAK.I | Y | 71.75 | 1664.8118 | 17 | 0.4 | 555.9448 | 3 | 36.17 | 6 | F6:6849 | Fraction\_6\_23092022.raw |  |  |  |  |  | 2.6132E5 |  | 1 | 0 | 0 | 0 | 0 | 0 | 1 | 0 | 80 | 96 |  |  | PEAKS DB |
| R.LAC(+57.02)GVIGITKSG | Y | 63.71 | 1174.6379 | 12 | 0.6 | 588.3266 | 2 | 43.93 | 6 | F6:10320 | Fraction\_6\_23092022.raw |  |  |  |  |  | 2.6798E5 |  | 1 | 0 | 0 | 0 | 0 | 0 | 1 | 0 | 144 | 155 | Carbamidomethylation | C3:Carbamidomethylation:1000.00 | PEAKS DB |
| R.LAC(+57.02)GVIGITK.S | Y | 62.68 | 1030.5845 | 10 | 0.3 | 516.2997 | 2 | 44.08 | 6 | F6:10377 | Fraction\_6\_23092022.raw |  |  |  |  |  | 7.6327E5 |  | 1 | 0 | 0 | 0 | 0 | 0 | 1 | 0 | 144 | 153 | Carbamidomethylation | C3:Carbamidomethylation:1000.00 | PEAKS DB |
| K.TTGNAGGRLAC(+57.02)GVIGITKSG | Y | 57.81 | 1888.9789 | 20 | 0.1 | 630.6670 | 3 | 43.16 | 6 | F6:9980 | Fraction\_6\_23092022.raw |  |  |  |  |  | 7.2697E4 |  | 1 | 0 | 0 | 0 | 0 | 0 | 1 | 0 | 136 | 155 | Carbamidomethylation | C11:Carbamidomethylation:1000.00 | PEAKS DB |
| total 7 peptides |  |  |  |  |  |  |  |  |  |  |  |  |  |  |  |  |  |  |  |  |  |  |  |  |  |  |  |  |  |  |  |
| --- | --- | --- | --- | --- | --- | --- | --- | --- | --- | --- | --- | --- | --- | --- | --- | --- | --- | --- | --- | --- | --- | --- | --- | --- | --- | --- | --- | --- | --- | --- | --- |

TRINITY\_DN1342\_c0\_g1\_i34.p1

back to list

  

| Protein Coverage
| Supporting Peptides
|

Protein Coverage:

Supporting Peptides:

| Peptide | Uniq | -10lgP | Mass | Length | ppm | m/z | z | RT | Fraction | Scan | Source File | Area F1 | Area F2 | Area F3 | Area F4 | Area F5 | Area F6 | Area F7 | #Feature | #Feature F1 | #Feature F2 | #Feature F3 | #Feature F4 | #Feature F5 | #Feature F6 | #Feature F7 | Start | End | PTM | AScore | Found By |
| --- | --- | --- | --- | --- | --- | --- | --- | --- | --- | --- | --- | --- | --- | --- | --- | --- | --- | --- | --- | --- | --- | --- | --- | --- | --- | --- | --- | --- | --- | --- | --- |
| R.DYTYLDHIYTKDDPR.T | N | 77.26 | 1913.8795 | 15 | 0.3 | 638.9673 | 3 | 47.98 | 5 | F5:11468 | Fraction\_5\_23092022.raw | 6.7442E5 | 6.2862E6 |  | 2.496E5 | 7.4735E5 |  |  | 5 | 1 | 2 | 0 | 1 | 1 | 0 | 0 | 248 | 262 |  |  | PEAKS DB |
| R.DYTYLDHIYTKDDPRTYELVK.S | N | 76.51 | 2647.2805 | 21 | 1.1 | 662.8281 | 4 | 57.24 | 2 | F2:14940 | Fraction\_2\_23092022.raw |  | 5.1388E6 | 1.891E5 |  | 1.9821E5 |  |  | 4 | 0 | 2 | 1 | 0 | 1 | 0 | 0 | 248 | 268 |  |  | PEAKS DB |
| R.DYTYLDHIYTK.D | N | 74.29 | 1430.6718 | 11 | 0.2 | 716.3433 | 2 | 52.27 | 5 | F5:13434 | Fraction\_5\_23092022.raw |  | 1.8665E5 |  |  | 1.9599E5 |  |  | 3 | 0 | 1 | 0 | 0 | 2 | 0 | 0 | 248 | 258 |  |  | PEAKS DB |
| K.NAVFYQIYPR.S | N | 73.17 | 1269.6505 | 10 | 0.3 | 635.8327 | 2 | 57.87 | 7 | F7:20277 | Fraction\_7\_23092022.raw | 1.1663E7 | 9.8135E7 | 2.3489E6 | 2.1542E5 | 2.417E5 | 9.3508E7 | 6.8859E7 | 8 | 1 | 1 | 1 | 1 | 1 | 2 | 1 | 29 | 38 |  |  | PEAKS DB |
| R.SFMDSNDDGVGDLK.G | N | 66.57 | 1498.6245 | 14 | 0.6 | 750.3200 | 2 | 52.27 | 1 | F1:12582 | Fraction\_1\_23092022r.raw | 1.6384E5 |  |  |  |  |  |  | 1 | 1 | 0 | 0 | 0 | 0 | 0 | 0 | 39 | 52 |  |  | PEAKS DB |
| R.SFM(+15.99)DSNDDGVGDLK.G | N | 60.49 | 1514.6195 | 14 | -0.3 | 758.3168 | 2 | 39.80 | 6 | F6:8482 | Fraction\_6\_23092022.raw | 4.5737E4 | 1.5252E5 |  |  |  | 3.4969E5 |  | 5 | 1 | 2 | 0 | 0 | 0 | 2 | 0 | 39 | 52 | Oxidation (M) | M3:Oxidation (M):1000.00 | PEAKS DB |
| R.IDAVPHLFER.Q | N | 55.84 | 1195.6349 | 10 | 1.2 | 399.5527 | 3 | 51.46 | 5 | F5:13030 | Fraction\_5\_23092022.raw |  |  |  |  | 1.2393E5 |  |  | 1 | 0 | 0 | 0 | 0 | 1 | 0 | 0 | 221 | 230 |  |  | PEAKS DB |
| K.YYQYGSDIPFNFK.L | N | 52.43 | 1640.7511 | 13 | 0.3 | 821.3831 | 2 | 68.00 | 2 | F2:19833 | Fraction\_2\_23092022.raw |  | 1.0668E5 |  |  |  |  |  | 1 | 0 | 1 | 0 | 0 | 0 | 0 | 0 | 302 | 314 |  |  | PEAKS DB |
| K.GIDGFRIDAVPHLFER.Q | N | 50.68 | 1840.9584 | 16 | 0.0 | 614.6601 | 3 | 68.71 | 2 | F2:20128 | Fraction\_2\_23092022.raw |  | 2.1436E5 |  |  |  |  |  | 1 | 0 | 1 | 0 | 0 | 0 | 0 | 0 | 215 | 230 |  |  | PEAKS DB |
| K.VILDLVPNHTSDK.H | Y | 50.32 | 1449.7827 | 13 | -0.1 | 484.2681 | 3 | 48.20 | 6 | F6:12267 | Fraction\_6\_23092022.raw |  |  |  |  |  | 7.1972E4 |  | 1 | 0 | 0 | 0 | 0 | 0 | 1 | 0 | 116 | 128 |  |  | PEAKS DB |
| total 10 peptides |  |  |  |  |  |  |  |  |  |  |  |  |  |  |  |  |  |  |  |  |  |  |  |  |  |  |  |  |  |  |  |
| --- | --- | --- | --- | --- | --- | --- | --- | --- | --- | --- | --- | --- | --- | --- | --- | --- | --- | --- | --- | --- | --- | --- | --- | --- | --- | --- | --- | --- | --- | --- | --- |

P02769|ALBU\_BOVIN

back to list

  

| Protein Coverage
| Supporting Peptides
|

Protein Coverage:

Supporting Peptides:

| Peptide | Uniq | -10lgP | Mass | Length | ppm | m/z | z | RT | Fraction | Scan | Source File | Area F1 | Area F2 | Area F3 | Area F4 | Area F5 | Area F6 | Area F7 | #Feature | #Feature F1 | #Feature F2 | #Feature F3 | #Feature F4 | #Feature F5 | #Feature F6 | #Feature F7 | Start | End | PTM | AScore | Found By |
| --- | --- | --- | --- | --- | --- | --- | --- | --- | --- | --- | --- | --- | --- | --- | --- | --- | --- | --- | --- | --- | --- | --- | --- | --- | --- | --- | --- | --- | --- | --- | --- |
| K.LGEYGFQNALIVR.Y | N | 87.65 | 1478.7881 | 13 | -0.4 | 740.4010 | 2 | 66.86 | 1 | F1:19104 | Fraction\_1\_23092022r.raw | 4.7265E5 | 1.4217E5 |  |  |  |  |  | 2 | 1 | 1 | 0 | 0 | 0 | 0 | 0 | 421 | 433 |  |  | PEAKS DB |
| R.KVPQVSTPTLVEVSR.S | N | 78.42 | 1638.9304 | 15 | 0.1 | 547.3174 | 3 | 46.27 | 1 | F1:9857 | Fraction\_1\_23092022r.raw | 2.9405E6 | 6.5876E5 | 2.0378E5 |  | 1.8424E5 |  |  | 4 | 1 | 1 | 1 | 0 | 1 | 0 | 0 | 437 | 451 |  |  | PEAKS DB |
| K.DAIPENLPPLTADFAEDK.D | Y | 63.32 | 1954.9523 | 18 | 0.9 | 978.4843 | 2 | 75.12 | 3 | F3:23333 | Fraction\_3\_23092022.raw |  | 8.381E5 | 9.9163E5 |  |  |  |  | 2 | 0 | 1 | 1 | 0 | 0 | 0 | 0 | 319 | 336 |  |  | PEAKS DB |
| R.RHPEYAVSVLLR.L | Y | 62.76 | 1438.8044 | 12 | 0.7 | 480.6091 | 3 | 44.13 | 1 | F1:8993 | Fraction\_1\_23092022r.raw | 1.7682E5 |  |  |  |  |  |  | 1 | 1 | 0 | 0 | 0 | 0 | 0 | 0 | 360 | 371 |  |  | PEAKS DB |
| K.TVMENFVAFVDK.C | Y | 62.66 | 1398.6853 | 12 | -0.1 | 700.3499 | 2 | 76.25 | 1 | F1:23312 | Fraction\_1\_23092022r.raw | 3.8211E5 |  |  |  |  |  |  | 1 | 1 | 0 | 0 | 0 | 0 | 0 | 0 | 569 | 580 |  |  | PEAKS DB |
| K.VPQVSTPTLVEVSR.S | N | 59.16 | 1510.8354 | 14 | 0.5 | 756.4254 | 2 | 53.78 | 3 | F3:13777 | Fraction\_3\_23092022.raw | 1.692E5 |  | 1.8626E5 |  |  |  |  | 2 | 1 | 0 | 1 | 0 | 0 | 0 | 0 | 438 | 451 |  |  | PEAKS DB |
| K.DAFLGSFLYEYSR.R | Y | 52.22 | 1566.7354 | 13 | -0.1 | 784.3749 | 2 | 82.62 | 1 | F1:26203 | Fraction\_1\_23092022r.raw | 1.5205E6 |  |  |  |  |  |  | 1 | 1 | 0 | 0 | 0 | 0 | 0 | 0 | 347 | 359 |  |  | PEAKS DB |
| total 7 peptides |  |  |  |  |  |  |  |  |  |  |  |  |  |  |  |  |  |  |  |  |  |  |  |  |  |  |  |  |  |  |  |
| --- | --- | --- | --- | --- | --- | --- | --- | --- | --- | --- | --- | --- | --- | --- | --- | --- | --- | --- | --- | --- | --- | --- | --- | --- | --- | --- | --- | --- | --- | --- | --- |

Q3TTY5|K22E\_MOUSE

back to list

  

| Protein Coverage
| Supporting Peptides
|

Protein Coverage:

Supporting Peptides:

| Peptide | Uniq | -10lgP | Mass | Length | ppm | m/z | z | RT | Fraction | Scan | Source File | Area F1 | Area F2 | Area F3 | Area F4 | Area F5 | Area F6 | Area F7 | #Feature | #Feature F1 | #Feature F2 | #Feature F3 | #Feature F4 | #Feature F5 | #Feature F6 | #Feature F7 | Start | End | PTM | AScore | Found By |
| --- | --- | --- | --- | --- | --- | --- | --- | --- | --- | --- | --- | --- | --- | --- | --- | --- | --- | --- | --- | --- | --- | --- | --- | --- | --- | --- | --- | --- | --- | --- | --- |
| R.FGGGSGFGGGGFGGGSFGGGR.F | Y | 85.13 | 1734.7498 | 21 | -0.6 | 868.3817 | 2 | 56.68 | 7 | F7:19468 | Fraction\_7\_23092022.raw |  |  |  | 9.4701E4 |  |  | 5.2227E5 | 2 | 0 | 0 | 0 | 1 | 0 | 0 | 1 | 132 | 152 |  |  | PEAKS DB |
| R.GFSSGSAVVSGGSR.R | N | 72.08 | 1253.6000 | 14 | -0.5 | 627.8069 | 2 | 29.48 | 7 | F7:6074 | Fraction\_7\_23092022.raw |  |  |  |  |  |  | 6.6622E4 | 1 | 0 | 0 | 0 | 0 | 0 | 0 | 1 | 23 | 36 |  |  | PEAKS DB |
| K.TLNNKFASFIDKVR.F | N | 71.43 | 1651.9045 | 14 | -0.2 | 551.6420 | 3 | 51.36 | 2 | F2:12305 | Fraction\_2\_23092022.raw | 2.671E5 | 1.8567E6 | 2.1296E5 |  |  | 8.2338E5 | 3.789E5 | 6 | 1 | 2 | 1 | 0 | 0 | 1 | 1 | 205 | 218 |  |  | PEAKS DB |
| K.FASFIDKVR.F | N | 64.36 | 1081.5920 | 9 | 1.5 | 361.5385 | 3 | 44.57 | 4 | F4:10216 | Fraction\_4\_23092022.raw | 6.0874E6 | 1.3079E6 | 2.6129E6 | 3.499E5 | 3.5976E5 | 1.1286E6 |  | 11 | 1 | 2 | 2 | 2 | 2 | 2 | 0 | 210 | 218 |  |  | PEAKS DB |
| R.LLRDYQELMNTK.L | N | 57.86 | 1522.7814 | 12 | -0.8 | 508.6006 | 3 | 47.69 | 1 | F1:10527 | Fraction\_1\_23092022r.raw | 5.6309E5 |  |  |  |  |  |  | 1 | 1 | 0 | 0 | 0 | 0 | 0 | 0 | 480 | 491 |  |  | PEAKS DB |
| R.DYQELMNTK.L | N | 53.32 | 1140.5121 | 9 | 0.8 | 571.2638 | 2 | 43.26 | 1 | F1:8611 | Fraction\_1\_23092022r.raw | 3.6715E4 |  |  |  |  |  |  | 1 | 1 | 0 | 0 | 0 | 0 | 0 | 0 | 483 | 491 |  |  | PEAKS DB |
| total 6 peptides |  |  |  |  |  |  |  |  |  |  |  |  |  |  |  |  |  |  |  |  |  |  |  |  |  |  |  |  |  |  |  |
| --- | --- | --- | --- | --- | --- | --- | --- | --- | --- | --- | --- | --- | --- | --- | --- | --- | --- | --- | --- | --- | --- | --- | --- | --- | --- | --- | --- | --- | --- | --- | --- |

TRINITY\_DN1455\_c0\_g1\_i1.p1

back to list

  

| Protein Coverage
| Supporting Peptides
|

Protein Coverage:

Supporting Peptides:

| Peptide | Uniq | -10lgP | Mass | Length | ppm | m/z | z | RT | Fraction | Scan | Source File | Area F1 | Area F2 | Area F3 | Area F4 | Area F5 | Area F6 | Area F7 | #Feature | #Feature F1 | #Feature F2 | #Feature F3 | #Feature F4 | #Feature F5 | #Feature F6 | #Feature F7 | Start | End | PTM | AScore | Found By |
| --- | --- | --- | --- | --- | --- | --- | --- | --- | --- | --- | --- | --- | --- | --- | --- | --- | --- | --- | --- | --- | --- | --- | --- | --- | --- | --- | --- | --- | --- | --- | --- |
| K.LTALGVLGNINDPEYSAR.E | Y | 87.23 | 1901.9846 | 18 | 0.4 | 952.0000 | 2 | 71.30 | 2 | F2:21275 | Fraction\_2\_23092022.raw |  | 1.9919E5 | 2.7094E5 | 6.0627E4 | 1.1387E5 |  |  | 4 | 0 | 1 | 1 | 1 | 1 | 0 | 0 | 161 | 178 |  |  | PEAKS DB |
| R.AITEFNAQPIISEIYGSGSTK.Y | Y | 74.84 | 2225.1216 | 21 | 0.7 | 1113.5688 | 2 | 78.12 | 2 | F2:24320 | Fraction\_2\_23092022.raw |  | 6.2463E5 |  |  |  |  |  | 1 | 0 | 1 | 0 | 0 | 0 | 0 | 0 | 445 | 465 |  |  | PEAKS DB |
| K.LTALGVLGNINDPEYSARENELR.A | Y | 71.83 | 2543.2979 | 23 | -1.0 | 848.7723 | 3 | 66.42 | 3 | F3:19465 | Fraction\_3\_23092022.raw |  | 1.1883E5 | 3.1282E5 |  | 1.2544E5 |  |  | 3 | 0 | 1 | 1 | 0 | 1 | 0 | 0 | 161 | 183 |  |  | PEAKS DB |
| K.TLAVASSPIAPEQHLK.N | Y | 47.35 | 1660.9148 | 16 | -0.3 | 554.6454 | 3 | 42.91 | 2 | F2:8489 | Fraction\_2\_23092022.raw |  | 8.6069E4 |  |  |  |  |  | 1 | 0 | 1 | 0 | 0 | 0 | 0 | 0 | 349 | 364 |  |  | PEAKS DB |
| total 4 peptides |  |  |  |  |  |  |  |  |  |  |  |  |  |  |  |  |  |  |  |  |  |  |  |  |  |  |  |  |  |  |  |
| --- | --- | --- | --- | --- | --- | --- | --- | --- | --- | --- | --- | --- | --- | --- | --- | --- | --- | --- | --- | --- | --- | --- | --- | --- | --- | --- | --- | --- | --- | --- | --- |

TRINITY\_DN5849\_c0\_g1\_i1.p1

back to list

  

| Protein Coverage
| Supporting Peptides
|

Protein Coverage:

Supporting Peptides:

| Peptide | Uniq | -10lgP | Mass | Length | ppm | m/z | z | RT | Fraction | Scan | Source File | Area F1 | Area F2 | Area F3 | Area F4 | Area F5 | Area F6 | Area F7 | #Feature | #Feature F1 | #Feature F2 | #Feature F3 | #Feature F4 | #Feature F5 | #Feature F6 | #Feature F7 | Start | End | PTM | AScore | Found By |
| --- | --- | --- | --- | --- | --- | --- | --- | --- | --- | --- | --- | --- | --- | --- | --- | --- | --- | --- | --- | --- | --- | --- | --- | --- | --- | --- | --- | --- | --- | --- | --- |
| R.VPVHNVSVVDLTVR.L | Y | 101.62 | 1532.8674 | 14 | -0.1 | 511.9630 | 3 | 48.43 | 7 | F7:14689 | Fraction\_7\_23092022.raw |  |  | 8.7264E5 | 7.3854E5 | 4.5131E5 | 8.643E6 | 1.271E6 | 6 | 0 | 0 | 1 | 1 | 1 | 2 | 1 | 232 | 245 |  |  | PEAKS DB |
| K.LTGM(+15.99)AFRVPVHNVSVVDLTVR.L | Y | 72.84 | 2325.2627 | 21 | 0.6 | 582.3233 | 4 | 58.07 | 6 | F6:16689 | Fraction\_6\_23092022.raw |  |  |  |  |  | 2.8368E6 |  | 2 | 0 | 0 | 0 | 0 | 0 | 2 | 0 | 225 | 245 | Oxidation (M) | M4:Oxidation (M):1000.00 | PEAKS DB |
| R.GAAQNIIPAATGAAK.A | Y | 64.85 | 1352.7412 | 15 | 0.3 | 677.3781 | 2 | 40.32 | 6 | F6:8717 | Fraction\_6\_23092022.raw |  |  |  |  |  | 1.3614E5 | 1.1358E5 | 2 | 0 | 0 | 0 | 0 | 0 | 1 | 1 | 198 | 212 |  |  | PEAKS DB |
| K.IGINGFGR.I | Y | 54.17 | 832.4555 | 8 | 0.6 | 417.2353 | 2 | 44.71 | 6 | F6:10653 | Fraction\_6\_23092022.raw |  |  |  |  |  | 6.6921E5 |  | 1 | 0 | 0 | 0 | 0 | 0 | 1 | 0 | 4 | 11 |  |  | PEAKS DB |
| total 4 peptides |  |  |  |  |  |  |  |  |  |  |  |  |  |  |  |  |  |  |  |  |  |  |  |  |  |  |  |  |  |  |  |
| --- | --- | --- | --- | --- | --- | --- | --- | --- | --- | --- | --- | --- | --- | --- | --- | --- | --- | --- | --- | --- | --- | --- | --- | --- | --- | --- | --- | --- | --- | --- | --- |

TRINITY\_DN5849\_c0\_g1\_i2.p1

back to list

  

| Protein Coverage
| Supporting Peptides
|

Protein Coverage:

Supporting Peptides:

| Peptide | Uniq | -10lgP | Mass | Length | ppm | m/z | z | RT | Fraction | Scan | Source File | Area F1 | Area F2 | Area F3 | Area F4 | Area F5 | Area F6 | Area F7 | #Feature | #Feature F1 | #Feature F2 | #Feature F3 | #Feature F4 | #Feature F5 | #Feature F6 | #Feature F7 | Start | End | PTM | AScore | Found By |
| --- | --- | --- | --- | --- | --- | --- | --- | --- | --- | --- | --- | --- | --- | --- | --- | --- | --- | --- | --- | --- | --- | --- | --- | --- | --- | --- | --- | --- | --- | --- | --- |
| R.VPVHNVSVVDLTVR.L | Y | 101.62 | 1532.8674 | 14 | -0.1 | 511.9630 | 3 | 48.43 | 7 | F7:14689 | Fraction\_7\_23092022.raw |  |  | 8.7264E5 | 7.3854E5 | 4.5131E5 | 8.643E6 | 1.271E6 | 6 | 0 | 0 | 1 | 1 | 1 | 2 | 1 | 232 | 245 |  |  | PEAKS DB |
| K.LTGM(+15.99)AFRVPVHNVSVVDLTVR.L | Y | 72.84 | 2325.2627 | 21 | 0.6 | 582.3233 | 4 | 58.07 | 6 | F6:16689 | Fraction\_6\_23092022.raw |  |  |  |  |  | 2.8368E6 |  | 2 | 0 | 0 | 0 | 0 | 0 | 2 | 0 | 225 | 245 | Oxidation (M) | M4:Oxidation (M):1000.00 | PEAKS DB |
| R.GAAQNIIPAATGAAK.A | Y | 64.85 | 1352.7412 | 15 | 0.3 | 677.3781 | 2 | 40.32 | 6 | F6:8717 | Fraction\_6\_23092022.raw |  |  |  |  |  | 1.3614E5 | 1.1358E5 | 2 | 0 | 0 | 0 | 0 | 0 | 1 | 1 | 198 | 212 |  |  | PEAKS DB |
| K.IGINGFGR.I | Y | 54.17 | 832.4555 | 8 | 0.6 | 417.2353 | 2 | 44.71 | 6 | F6:10653 | Fraction\_6\_23092022.raw |  |  |  |  |  | 6.6921E5 |  | 1 | 0 | 0 | 0 | 0 | 0 | 1 | 0 | 4 | 11 |  |  | PEAKS DB |
| total 4 peptides |  |  |  |  |  |  |  |  |  |  |  |  |  |  |  |  |  |  |  |  |  |  |  |  |  |  |  |  |  |  |  |
| --- | --- | --- | --- | --- | --- | --- | --- | --- | --- | --- | --- | --- | --- | --- | --- | --- | --- | --- | --- | --- | --- | --- | --- | --- | --- | --- | --- | --- | --- | --- | --- |

TRINITY\_DN5849\_c0\_g1\_i10.p1

back to list

  

| Protein Coverage
| Supporting Peptides
|

Protein Coverage:

Supporting Peptides:

| Peptide | Uniq | -10lgP | Mass | Length | ppm | m/z | z | RT | Fraction | Scan | Source File | Area F1 | Area F2 | Area F3 | Area F4 | Area F5 | Area F6 | Area F7 | #Feature | #Feature F1 | #Feature F2 | #Feature F3 | #Feature F4 | #Feature F5 | #Feature F6 | #Feature F7 | Start | End | PTM | AScore | Found By |
| --- | --- | --- | --- | --- | --- | --- | --- | --- | --- | --- | --- | --- | --- | --- | --- | --- | --- | --- | --- | --- | --- | --- | --- | --- | --- | --- | --- | --- | --- | --- | --- |
| R.VPVHNVSVVDLTVR.L | Y | 101.62 | 1532.8674 | 14 | -0.1 | 511.9630 | 3 | 48.43 | 7 | F7:14689 | Fraction\_7\_23092022.raw |  |  | 8.7264E5 | 7.3854E5 | 4.5131E5 | 8.643E6 | 1.271E6 | 6 | 0 | 0 | 1 | 1 | 1 | 2 | 1 | 232 | 245 |  |  | PEAKS DB |
| K.LTGM(+15.99)AFRVPVHNVSVVDLTVR.L | Y | 72.84 | 2325.2627 | 21 | 0.6 | 582.3233 | 4 | 58.07 | 6 | F6:16689 | Fraction\_6\_23092022.raw |  |  |  |  |  | 2.8368E6 |  | 2 | 0 | 0 | 0 | 0 | 0 | 2 | 0 | 225 | 245 | Oxidation (M) | M4:Oxidation (M):1000.00 | PEAKS DB |
| R.GAAQNIIPAATGAAK.A | Y | 64.85 | 1352.7412 | 15 | 0.3 | 677.3781 | 2 | 40.32 | 6 | F6:8717 | Fraction\_6\_23092022.raw |  |  |  |  |  | 1.3614E5 | 1.1358E5 | 2 | 0 | 0 | 0 | 0 | 0 | 1 | 1 | 198 | 212 |  |  | PEAKS DB |
| K.IGINGFGR.I | Y | 54.17 | 832.4555 | 8 | 0.6 | 417.2353 | 2 | 44.71 | 6 | F6:10653 | Fraction\_6\_23092022.raw |  |  |  |  |  | 6.6921E5 |  | 1 | 0 | 0 | 0 | 0 | 0 | 1 | 0 | 4 | 11 |  |  | PEAKS DB |
| total 4 peptides |  |  |  |  |  |  |  |  |  |  |  |  |  |  |  |  |  |  |  |  |  |  |  |  |  |  |  |  |  |  |  |
| --- | --- | --- | --- | --- | --- | --- | --- | --- | --- | --- | --- | --- | --- | --- | --- | --- | --- | --- | --- | --- | --- | --- | --- | --- | --- | --- | --- | --- | --- | --- | --- |

TRINITY\_DN36831\_c0\_g1\_i1.p1

back to list

  

| Protein Coverage
| Supporting Peptides
|

Protein Coverage:

Supporting Peptides:

| Peptide | Uniq | -10lgP | Mass | Length | ppm | m/z | z | RT | Fraction | Scan | Source File | Area F1 | Area F2 | Area F3 | Area F4 | Area F5 | Area F6 | Area F7 | #Feature | #Feature F1 | #Feature F2 | #Feature F3 | #Feature F4 | #Feature F5 | #Feature F6 | #Feature F7 | Start | End | PTM | AScore | Found By |
| --- | --- | --- | --- | --- | --- | --- | --- | --- | --- | --- | --- | --- | --- | --- | --- | --- | --- | --- | --- | --- | --- | --- | --- | --- | --- | --- | --- | --- | --- | --- | --- |
| K.YSNVVANPTLR.A | Y | 82.28 | 1232.6514 | 11 | 0.5 | 617.3333 | 2 | 38.72 | 3 | F3:7055 | Fraction\_3\_23092022.raw |  |  | 2.1727E5 | 3.9106E5 | 8.9155E4 | 1.376E5 |  | 4 | 0 | 0 | 1 | 1 | 1 | 1 | 0 | 108 | 118 |  |  | PEAKS DB |
| K.FDQHGYILSAAVSAAATSAR.Q | Y | 79.65 | 2035.0122 | 20 | -0.1 | 679.3446 | 3 | 61.76 | 4 | F4:17937 | Fraction\_4\_23092022.raw |  |  |  | 4.3575E5 | 7.232E4 |  |  | 2 | 0 | 0 | 0 | 1 | 1 | 0 | 0 | 168 | 187 |  |  | PEAKS DB |
| K.IVC(+57.02)YFGSWAVYR.Q | Y | 75.90 | 1519.7281 | 12 | 0.9 | 760.8721 | 2 | 72.48 | 4 | F4:22715 | Fraction\_4\_23092022.raw |  |  |  | 1.477E5 |  |  |  | 1 | 0 | 0 | 0 | 1 | 0 | 0 | 0 | 18 | 29 | Carbamidomethylation | C3:Carbamidomethylation:1000.00 | PEAKS DB |
| K.FVNSVVDFLK.K | Y | 60.59 | 1166.6335 | 10 | 1.1 | 584.3247 | 2 | 71.87 | 3 | F3:21892 | Fraction\_3\_23092022.raw |  |  | 1.8204E5 | 7.3454E4 |  |  |  | 2 | 0 | 0 | 1 | 1 | 0 | 0 | 0 | 121 | 130 |  |  | PEAKS DB |
| R.GGKPADKQNYISLLK.E | Y | 50.60 | 1630.9042 | 15 | 0.6 | 544.6423 | 3 | 38.16 | 6 | F6:7749 | Fraction\_6\_23092022.raw |  |  |  | 2.3055E5 |  | 1.5484E5 |  | 2 | 0 | 0 | 0 | 1 | 0 | 1 | 0 | 148 | 162 |  |  | PEAKS DB |
| R.EKFDQHGYILSAAVSAAATSAR.Q | Y | 48.44 | 2292.1499 | 22 | -0.3 | 574.0446 | 4 | 55.54 | 4 | F4:15128 | Fraction\_4\_23092022.raw |  |  |  | 1.7934E5 |  |  |  | 1 | 0 | 0 | 0 | 1 | 0 | 0 | 0 | 166 | 187 |  |  | PEAKS DB |
| total 6 peptides |  |  |  |  |  |  |  |  |  |  |  |  |  |  |  |  |  |  |  |  |  |  |  |  |  |  |  |  |  |  |  |
| --- | --- | --- | --- | --- | --- | --- | --- | --- | --- | --- | --- | --- | --- | --- | --- | --- | --- | --- | --- | --- | --- | --- | --- | --- | --- | --- | --- | --- | --- | --- | --- |

TRINITY\_DN36822\_c0\_g1\_i1.p1

back to list

  

| Protein Coverage
| Supporting Peptides
|

Protein Coverage:

Supporting Peptides:

| Peptide | Uniq | -10lgP | Mass | Length | ppm | m/z | z | RT | Fraction | Scan | Source File | Area F1 | Area F2 | Area F3 | Area F4 | Area F5 | Area F6 | Area F7 | #Feature | #Feature F1 | #Feature F2 | #Feature F3 | #Feature F4 | #Feature F5 | #Feature F6 | #Feature F7 | Start | End | PTM | AScore | Found By |
| --- | --- | --- | --- | --- | --- | --- | --- | --- | --- | --- | --- | --- | --- | --- | --- | --- | --- | --- | --- | --- | --- | --- | --- | --- | --- | --- | --- | --- | --- | --- | --- |
| K.LGIFNSEGNINEEVLR.S | Y | 87.15 | 1802.9163 | 16 | 0.2 | 902.4656 | 2 | 67.92 | 7 | F7:25933 | Fraction\_7\_23092022.raw |  |  |  |  |  |  | 8.5033E5 | 1 | 0 | 0 | 0 | 0 | 0 | 0 | 1 | 89 | 104 |  |  | PEAKS DB |
| R.LYDSLPEDKVEEIFQK.C | Y | 81.90 | 1951.9778 | 16 | 0.5 | 651.6669 | 3 | 64.66 | 7 | F7:24256 | Fraction\_7\_23092022.raw |  |  |  |  |  |  | 1.0379E6 | 1 | 0 | 0 | 0 | 0 | 0 | 0 | 1 | 107 | 122 |  |  | PEAKS DB |
| K.KLGIFNSEGNINEEVLR.S | Y | 72.93 | 1931.0112 | 17 | -0.1 | 644.6776 | 3 | 56.72 | 7 | F7:19481 | Fraction\_7\_23092022.raw |  |  |  |  |  |  | 8.0931E5 | 1 | 0 | 0 | 0 | 0 | 0 | 0 | 1 | 88 | 104 |  |  | PEAKS DB |
| K.ESC(+57.02)ITESGVDPAIVENAK.N | Y | 64.78 | 1917.8989 | 18 | 3.1 | 959.9597 | 2 | 50.19 | 7 | F7:15562 | Fraction\_7\_23092022.raw |  |  |  |  |  |  | 1.6313E5 | 1 | 0 | 0 | 0 | 0 | 0 | 0 | 1 | 50 | 67 | Carbamidomethylation | C3:Carbamidomethylation:1000.00 | PEAKS DB |
| total 4 peptides |  |  |  |  |  |  |  |  |  |  |  |  |  |  |  |  |  |  |  |  |  |  |  |  |  |  |  |  |  |  |  |
| --- | --- | --- | --- | --- | --- | --- | --- | --- | --- | --- | --- | --- | --- | --- | --- | --- | --- | --- | --- | --- | --- | --- | --- | --- | --- | --- | --- | --- | --- | --- | --- |

TRINITY\_DN5723\_c0\_g1\_i5.p1

back to list

  

| Protein Coverage
| Supporting Peptides
|

Protein Coverage:

Supporting Peptides:

| Peptide | Uniq | -10lgP | Mass | Length | ppm | m/z | z | RT | Fraction | Scan | Source File | Area F1 | Area F2 | Area F3 | Area F4 | Area F5 | Area F6 | Area F7 | #Feature | #Feature F1 | #Feature F2 | #Feature F3 | #Feature F4 | #Feature F5 | #Feature F6 | #Feature F7 | Start | End | PTM | AScore | Found By |
| --- | --- | --- | --- | --- | --- | --- | --- | --- | --- | --- | --- | --- | --- | --- | --- | --- | --- | --- | --- | --- | --- | --- | --- | --- | --- | --- | --- | --- | --- | --- | --- |
| R.IAEHYPYFTGGVIALNK.D | Y | 85.35 | 1891.9832 | 17 | -0.5 | 631.6680 | 3 | 59.95 | 6 | F6:17535 | Fraction\_6\_23092022.raw |  |  |  |  |  | 1.7409E6 |  | 1 | 0 | 0 | 0 | 0 | 0 | 1 | 0 | 299 | 315 |  |  | PEAKS DB |
| R.YKIPGRIGDSPIPGAGAYADQQVGAAAGTGDGDIM(+15.99)M(+15.99)R.F | Y | 83.04 | 3707.7671 | 37 | -1.3 | 927.9479 | 4 | 53.42 | 6 | F6:14583 | Fraction\_6\_23092022.raw |  |  |  |  |  | 3.0039E5 |  | 1 | 0 | 0 | 0 | 0 | 0 | 1 | 0 | 235 | 271 | Oxidation (M) | M35:Oxidation (M):1000.00;M36:Oxidation (M):1000.00 | PEAKS DB |
| R.IGDSPIPGAGAYADQQVGAAAGTGDGDIM(+15.99)M(+15.99)R.F | Y | 74.35 | 2993.3494 | 31 | 0.6 | 998.7910 | 3 | 55.97 | 6 | F6:15730 | Fraction\_6\_23092022.raw |  |  |  |  |  | 1.0585E6 |  | 1 | 0 | 0 | 0 | 0 | 0 | 1 | 0 | 241 | 271 | Oxidation (M) | M29:Oxidation (M):1000.00;M30:Oxidation (M):1000.00 | PEAKS DB |
| R.FLPSFLAVEEM(+15.99)R.R | Y | 56.76 | 1453.7275 | 12 | 0.6 | 727.8715 | 2 | 71.98 | 6 | F6:22974 | Fraction\_6\_23092022.raw |  |  |  |  |  | 1.1595E6 |  | 1 | 0 | 0 | 0 | 0 | 0 | 1 | 0 | 272 | 283 | Oxidation (M) | M11:Oxidation (M):1000.00 | PEAKS DB |
| total 4 peptides |  |  |  |  |  |  |  |  |  |  |  |  |  |  |  |  |  |  |  |  |  |  |  |  |  |  |  |  |  |  |  |
| --- | --- | --- | --- | --- | --- | --- | --- | --- | --- | --- | --- | --- | --- | --- | --- | --- | --- | --- | --- | --- | --- | --- | --- | --- | --- | --- | --- | --- | --- | --- | --- |

TRINITY\_DN5723\_c0\_g1\_i7.p1

back to list

  

| Protein Coverage
| Supporting Peptides
|

Protein Coverage:

Supporting Peptides:

| Peptide | Uniq | -10lgP | Mass | Length | ppm | m/z | z | RT | Fraction | Scan | Source File | Area F1 | Area F2 | Area F3 | Area F4 | Area F5 | Area F6 | Area F7 | #Feature | #Feature F1 | #Feature F2 | #Feature F3 | #Feature F4 | #Feature F5 | #Feature F6 | #Feature F7 | Start | End | PTM | AScore | Found By |
| --- | --- | --- | --- | --- | --- | --- | --- | --- | --- | --- | --- | --- | --- | --- | --- | --- | --- | --- | --- | --- | --- | --- | --- | --- | --- | --- | --- | --- | --- | --- | --- |
| R.IAEHYPYFTGGVIALNK.D | Y | 85.35 | 1891.9832 | 17 | -0.5 | 631.6680 | 3 | 59.95 | 6 | F6:17535 | Fraction\_6\_23092022.raw |  |  |  |  |  | 1.7409E6 |  | 1 | 0 | 0 | 0 | 0 | 0 | 1 | 0 | 299 | 315 |  |  | PEAKS DB |
| R.YKIPGRIGDSPIPGAGAYADQQVGAAAGTGDGDIM(+15.99)M(+15.99)R.F | Y | 83.04 | 3707.7671 | 37 | -1.3 | 927.9479 | 4 | 53.42 | 6 | F6:14583 | Fraction\_6\_23092022.raw |  |  |  |  |  | 3.0039E5 |  | 1 | 0 | 0 | 0 | 0 | 0 | 1 | 0 | 235 | 271 | Oxidation (M) | M35:Oxidation (M):1000.00;M36:Oxidation (M):1000.00 | PEAKS DB |
| R.IGDSPIPGAGAYADQQVGAAAGTGDGDIM(+15.99)M(+15.99)R.F | Y | 74.35 | 2993.3494 | 31 | 0.6 | 998.7910 | 3 | 55.97 | 6 | F6:15730 | Fraction\_6\_23092022.raw |  |  |  |  |  | 1.0585E6 |  | 1 | 0 | 0 | 0 | 0 | 0 | 1 | 0 | 241 | 271 | Oxidation (M) | M29:Oxidation (M):1000.00;M30:Oxidation (M):1000.00 | PEAKS DB |
| R.FLPSFLAVEEM(+15.99)R.R | Y | 56.76 | 1453.7275 | 12 | 0.6 | 727.8715 | 2 | 71.98 | 6 | F6:22974 | Fraction\_6\_23092022.raw |  |  |  |  |  | 1.1595E6 |  | 1 | 0 | 0 | 0 | 0 | 0 | 1 | 0 | 272 | 283 | Oxidation (M) | M11:Oxidation (M):1000.00 | PEAKS DB |
| total 4 peptides |  |  |  |  |  |  |  |  |  |  |  |  |  |  |  |  |  |  |  |  |  |  |  |  |  |  |  |  |  |  |  |
| --- | --- | --- | --- | --- | --- | --- | --- | --- | --- | --- | --- | --- | --- | --- | --- | --- | --- | --- | --- | --- | --- | --- | --- | --- | --- | --- | --- | --- | --- | --- | --- |

TRINITY\_DN9761\_c0\_g1\_i1.p1

back to list

  

| Protein Coverage
| Supporting Peptides
|

Protein Coverage:

Supporting Peptides:

| Peptide | Uniq | -10lgP | Mass | Length | ppm | m/z | z | RT | Fraction | Scan | Source File | Area F1 | Area F2 | Area F3 | Area F4 | Area F5 | Area F6 | Area F7 | #Feature | #Feature F1 | #Feature F2 | #Feature F3 | #Feature F4 | #Feature F5 | #Feature F6 | #Feature F7 | Start | End | PTM | AScore | Found By |
| --- | --- | --- | --- | --- | --- | --- | --- | --- | --- | --- | --- | --- | --- | --- | --- | --- | --- | --- | --- | --- | --- | --- | --- | --- | --- | --- | --- | --- | --- | --- | --- |
| R.DYTYLDHIYTKDDPR.T | N | 77.26 | 1913.8795 | 15 | 0.3 | 638.9673 | 3 | 47.98 | 5 | F5:11468 | Fraction\_5\_23092022.raw | 6.7442E5 | 6.2862E6 |  | 2.496E5 | 7.4735E5 |  |  | 5 | 1 | 2 | 0 | 1 | 1 | 0 | 0 | 127 | 141 |  |  | PEAKS DB |
| R.DYTYLDHIYTKDDPRTYELVK.S | N | 76.51 | 2647.2805 | 21 | 1.1 | 662.8281 | 4 | 57.24 | 2 | F2:14940 | Fraction\_2\_23092022.raw |  | 5.1388E6 | 1.891E5 |  | 1.9821E5 |  |  | 4 | 0 | 2 | 1 | 0 | 1 | 0 | 0 | 127 | 147 |  |  | PEAKS DB |
| R.DYTYLDHIYTK.D | N | 74.29 | 1430.6718 | 11 | 0.2 | 716.3433 | 2 | 52.27 | 5 | F5:13434 | Fraction\_5\_23092022.raw |  | 1.8665E5 |  |  | 1.9599E5 |  |  | 3 | 0 | 1 | 0 | 0 | 2 | 0 | 0 | 127 | 137 |  |  | PEAKS DB |
| K.GVDGFRIDAVPHLFESEDLRDEPR.S | Y | 58.67 | 2768.3518 | 24 | 0.0 | 693.0952 | 4 | 66.25 | 2 | F2:19048 | Fraction\_2\_23092022.raw |  | 1.7185E5 |  |  |  |  |  | 1 | 0 | 1 | 0 | 0 | 0 | 0 | 0 | 94 | 117 |  |  | PEAKS DB |
| total 4 peptides |  |  |  |  |  |  |  |  |  |  |  |  |  |  |  |  |  |  |  |  |  |  |  |  |  |  |  |  |  |  |  |
| --- | --- | --- | --- | --- | --- | --- | --- | --- | --- | --- | --- | --- | --- | --- | --- | --- | --- | --- | --- | --- | --- | --- | --- | --- | --- | --- | --- | --- | --- | --- | --- |

TRINITY\_DN594\_c1\_g4\_i5.p1

back to list

  

| Protein Coverage
| Supporting Peptides
|

Protein Coverage:

Supporting Peptides:

| Peptide | Uniq | -10lgP | Mass | Length | ppm | m/z | z | RT | Fraction | Scan | Source File | Area F1 | Area F2 | Area F3 | Area F4 | Area F5 | Area F6 | Area F7 | #Feature | #Feature F1 | #Feature F2 | #Feature F3 | #Feature F4 | #Feature F5 | #Feature F6 | #Feature F7 | Start | End | PTM | AScore | Found By |
| --- | --- | --- | --- | --- | --- | --- | --- | --- | --- | --- | --- | --- | --- | --- | --- | --- | --- | --- | --- | --- | --- | --- | --- | --- | --- | --- | --- | --- | --- | --- | --- |
| R.SYVYFSESIENPHGFR.V | Y | 103.40 | 1930.8849 | 16 | 0.6 | 644.6360 | 3 | 55.92 | 5 | F5:15053 | Fraction\_5\_23092022.raw |  |  |  | 1.2044E6 | 8.6364E5 |  |  | 3 | 0 | 0 | 0 | 1 | 2 | 0 | 0 | 158 | 173 |  |  | PEAKS DB |
| K.FINYM(+15.99)QAVADLNPK.N | Y | 73.10 | 1638.8075 | 14 | 0.9 | 820.4117 | 2 | 64.73 | 4 | F4:19264 | Fraction\_4\_23092022.raw |  |  |  | 3.6461E5 | 2.691E5 |  |  | 2 | 0 | 0 | 0 | 1 | 1 | 0 | 0 | 44 | 57 | Oxidation (M) | M5:Oxidation (M):1000.00 | PEAKS DB |
| R.AC(+57.02)IPNVALYVAK.F | Y | 60.85 | 1317.7114 | 12 | 0.2 | 659.8631 | 2 | 60.72 | 5 | F5:17213 | Fraction\_5\_23092022.raw |  |  |  | 3.4399E5 | 2.5891E5 |  |  | 2 | 0 | 0 | 0 | 1 | 1 | 0 | 0 | 32 | 43 | Carbamidomethylation | C2:Carbamidomethylation:1000.00 | PEAKS DB |
| total 3 peptides |  |  |  |  |  |  |  |  |  |  |  |  |  |  |  |  |  |  |  |  |  |  |  |  |  |  |  |  |  |  |  |
| --- | --- | --- | --- | --- | --- | --- | --- | --- | --- | --- | --- | --- | --- | --- | --- | --- | --- | --- | --- | --- | --- | --- | --- | --- | --- | --- | --- | --- | --- | --- | --- |

TRINITY\_DN594\_c1\_g4\_i1.p1

back to list

  

| Protein Coverage
| Supporting Peptides
|

Protein Coverage:

Supporting Peptides:

| Peptide | Uniq | -10lgP | Mass | Length | ppm | m/z | z | RT | Fraction | Scan | Source File | Area F1 | Area F2 | Area F3 | Area F4 | Area F5 | Area F6 | Area F7 | #Feature | #Feature F1 | #Feature F2 | #Feature F3 | #Feature F4 | #Feature F5 | #Feature F6 | #Feature F7 | Start | End | PTM | AScore | Found By |
| --- | --- | --- | --- | --- | --- | --- | --- | --- | --- | --- | --- | --- | --- | --- | --- | --- | --- | --- | --- | --- | --- | --- | --- | --- | --- | --- | --- | --- | --- | --- | --- |
| R.SYVYFSESIENPHGFR.V | Y | 103.40 | 1930.8849 | 16 | 0.6 | 644.6360 | 3 | 55.92 | 5 | F5:15053 | Fraction\_5\_23092022.raw |  |  |  | 1.2044E6 | 8.6364E5 |  |  | 3 | 0 | 0 | 0 | 1 | 2 | 0 | 0 | 272 | 287 |  |  | PEAKS DB |
| K.FINYM(+15.99)QAVADLNPK.N | Y | 73.10 | 1638.8075 | 14 | 0.9 | 820.4117 | 2 | 64.73 | 4 | F4:19264 | Fraction\_4\_23092022.raw |  |  |  | 3.6461E5 | 2.691E5 |  |  | 2 | 0 | 0 | 0 | 1 | 1 | 0 | 0 | 158 | 171 | Oxidation (M) | M5:Oxidation (M):1000.00 | PEAKS DB |
| R.AC(+57.02)IPNVALYVAK.F | Y | 60.85 | 1317.7114 | 12 | 0.2 | 659.8631 | 2 | 60.72 | 5 | F5:17213 | Fraction\_5\_23092022.raw |  |  |  | 3.4399E5 | 2.5891E5 |  |  | 2 | 0 | 0 | 0 | 1 | 1 | 0 | 0 | 146 | 157 | Carbamidomethylation | C2:Carbamidomethylation:1000.00 | PEAKS DB |
| total 3 peptides |  |  |  |  |  |  |  |  |  |  |  |  |  |  |  |  |  |  |  |  |  |  |  |  |  |  |  |  |  |  |  |
| --- | --- | --- | --- | --- | --- | --- | --- | --- | --- | --- | --- | --- | --- | --- | --- | --- | --- | --- | --- | --- | --- | --- | --- | --- | --- | --- | --- | --- | --- | --- | --- |

Q02413|DSG1\_HUMAN

back to list

  

| Protein Coverage
| Supporting Peptides
|

Protein Coverage:

Supporting Peptides:

| Peptide | Uniq | -10lgP | Mass | Length | ppm | m/z | z | RT | Fraction | Scan | Source File | Area F1 | Area F2 | Area F3 | Area F4 | Area F5 | Area F6 | Area F7 | #Feature | #Feature F1 | #Feature F2 | #Feature F3 | #Feature F4 | #Feature F5 | #Feature F6 | #Feature F7 | Start | End | PTM | AScore | Found By |
| --- | --- | --- | --- | --- | --- | --- | --- | --- | --- | --- | --- | --- | --- | --- | --- | --- | --- | --- | --- | --- | --- | --- | --- | --- | --- | --- | --- | --- | --- | --- | --- |
| R.YVM(+15.99)GNNPADLLAVDSR.T | Y | 83.76 | 1749.8356 | 16 | -0.2 | 875.9249 | 2 | 59.21 | 1 | F1:15652 | Fraction\_1\_23092022r.raw | 4.7542E5 |  |  |  |  |  |  | 1 | 1 | 0 | 0 | 0 | 0 | 0 | 0 | 423 | 438 | Oxidation (M) | M3:Oxidation (M):1000.00 | PEAKS DB |
| K.TYVVTGNM(+15.99)GSNDKVGDFVATDLDTGRPSTTVR.Y | Y | 79.56 | 3388.6204 | 32 | 0.6 | 848.1628 | 4 | 52.22 | 1 | F1:12546 | Fraction\_1\_23092022r.raw | 4.3315E5 |  |  |  |  |  |  | 1 | 1 | 0 | 0 | 0 | 0 | 0 | 0 | 391 | 422 | Oxidation (M) | M8:Oxidation (M):1000.00 | PEAKS DB |
| K.VGDFVATDLDTGRPSTTVR.Y | Y | 62.78 | 2006.0068 | 19 | -0.1 | 669.6761 | 3 | 48.18 | 1 | F1:10779 | Fraction\_1\_23092022r.raw | 2.3162E5 |  |  |  |  |  |  | 1 | 1 | 0 | 0 | 0 | 0 | 0 | 0 | 404 | 422 |  |  | PEAKS DB |
| R.ISGVGIDQPPYGIFVINQK.T | Y | 61.67 | 2044.0992 | 19 | 0.5 | 1023.0574 | 2 | 77.32 | 1 | F1:23761 | Fraction\_1\_23092022r.raw | 5.3696E5 |  |  |  |  |  |  | 1 | 1 | 0 | 0 | 0 | 0 | 0 | 0 | 87 | 105 |  |  | PEAKS DB |
| total 4 peptides |  |  |  |  |  |  |  |  |  |  |  |  |  |  |  |  |  |  |  |  |  |  |  |  |  |  |  |  |  |  |  |
| --- | --- | --- | --- | --- | --- | --- | --- | --- | --- | --- | --- | --- | --- | --- | --- | --- | --- | --- | --- | --- | --- | --- | --- | --- | --- | --- | --- | --- | --- | --- | --- |

P05787|K2C8\_HUMAN

back to list

  

| Protein Coverage
| Supporting Peptides
|

Protein Coverage:

Supporting Peptides:

| Peptide | Uniq | -10lgP | Mass | Length | ppm | m/z | z | RT | Fraction | Scan | Source File | Area F1 | Area F2 | Area F3 | Area F4 | Area F5 | Area F6 | Area F7 | #Feature | #Feature F1 | #Feature F2 | #Feature F3 | #Feature F4 | #Feature F5 | #Feature F6 | #Feature F7 | Start | End | PTM | AScore | Found By |
| --- | --- | --- | --- | --- | --- | --- | --- | --- | --- | --- | --- | --- | --- | --- | --- | --- | --- | --- | --- | --- | --- | --- | --- | --- | --- | --- | --- | --- | --- | --- | --- |
| K.TTSGYAGGLSSAYGGLTSPGLSYSLGSSFGSGAGSSSFSR.T | Y | 80.81 | 3724.7129 | 40 | 3.3 | 1242.5823 | 3 | 78.22 | 1 | F1:24186 | Fraction\_1\_23092022r.raw | 3.4882E5 | 1.2177E5 |  |  |  |  |  | 2 | 1 | 1 | 0 | 0 | 0 | 0 | 0 | 415 | 454 |  |  | PEAKS DB |
| K.LALDIEIATYR.K | N | 77.60 | 1276.7026 | 11 | 0.4 | 639.3588 | 2 | 69.92 | 5 | F5:21320 | Fraction\_5\_23092022.raw | 1.0555E7 | 3.286E6 | 4.4833E6 |  | 9.0556E5 | 1.6127E6 | 1.5171E6 | 6 | 1 | 1 | 1 | 0 | 1 | 1 | 1 | 382 | 392 |  |  | PEAKS DB |
| K.TLNNKFASFIDKVR.F | N | 71.43 | 1651.9045 | 14 | -0.2 | 551.6420 | 3 | 51.36 | 2 | F2:12305 | Fraction\_2\_23092022.raw | 2.671E5 | 1.8567E6 | 2.1296E5 |  |  | 8.2338E5 | 3.789E5 | 6 | 1 | 2 | 1 | 0 | 0 | 1 | 1 | 97 | 110 |  |  | PEAKS DB |
| K.FASFIDKVR.F | N | 64.36 | 1081.5920 | 9 | 1.5 | 361.5385 | 3 | 44.57 | 4 | F4:10216 | Fraction\_4\_23092022.raw | 6.0874E6 | 1.3079E6 | 2.6129E6 | 3.499E5 | 3.5976E5 | 1.1286E6 |  | 11 | 1 | 2 | 2 | 2 | 2 | 2 | 0 | 102 | 110 |  |  | PEAKS DB |
| total 4 peptides |  |  |  |  |  |  |  |  |  |  |  |  |  |  |  |  |  |  |  |  |  |  |  |  |  |  |  |  |  |  |  |
| --- | --- | --- | --- | --- | --- | --- | --- | --- | --- | --- | --- | --- | --- | --- | --- | --- | --- | --- | --- | --- | --- | --- | --- | --- | --- | --- | --- | --- | --- | --- | --- |

P35782|VA52\_VESCR

back to list

  

| Protein Coverage
| Supporting Peptides
|

Protein Coverage:

Supporting Peptides:

| Peptide | Uniq | -10lgP | Mass | Length | ppm | m/z | z | RT | Fraction | Scan | Source File | Area F1 | Area F2 | Area F3 | Area F4 | Area F5 | Area F6 | Area F7 | #Feature | #Feature F1 | #Feature F2 | #Feature F3 | #Feature F4 | #Feature F5 | #Feature F6 | #Feature F7 | Start | End | PTM | AScore | Found By |
| --- | --- | --- | --- | --- | --- | --- | --- | --- | --- | --- | --- | --- | --- | --- | --- | --- | --- | --- | --- | --- | --- | --- | --- | --- | --- | --- | --- | --- | --- | --- | --- |
| K.ASGLTKQENLEILKQHNEFRQK.V | Y | 84.56 | 2610.3877 | 22 | 3.0 | 523.0864 | 5 | 35.85 | 7 | F7:8886 | Fraction\_7\_23092022.raw |  |  |  |  |  | 4.5984E5 | 1.4646E8 | 3 | 0 | 0 | 0 | 0 | 0 | 1 | 2 | 33 | 54 |  |  | PEAKS DB |
| K.ASGLTKQENLEILKQHNEFR.Q | Y | 75.55 | 2354.2341 | 20 | -0.2 | 589.5657 | 4 | 41.58 | 4 | F4:8850 | Fraction\_4\_23092022.raw |  |  |  | 3.5359E6 |  | 1.9178E6 | 2.9167E7 | 5 | 0 | 0 | 0 | 1 | 0 | 2 | 2 | 33 | 52 |  |  | PEAKS DB |
| R.GLETRGNPGPQPPAK.S | N | 57.93 | 1517.7949 | 15 | -0.4 | 506.9387 | 3 | 22.00 | 7 | F7:3354 | Fraction\_7\_23092022.raw |  |  |  |  |  |  | 7.2952E4 | 1 | 0 | 0 | 0 | 0 | 0 | 0 | 1 | 58 | 72 |  |  | PEAKS DB |
| K.ASGLTKQENLEILK.Q | Y | 54.71 | 1542.8617 | 14 | -0.5 | 515.2943 | 3 | 46.87 | 7 | F7:13890 | Fraction\_7\_23092022.raw |  |  |  | 6.5316E5 |  |  | 2.4172E6 | 3 | 0 | 0 | 0 | 1 | 0 | 0 | 2 | 33 | 46 |  |  | PEAKS DB |
| R.GNPGPQPPAK.S | N | 52.98 | 961.4981 | 10 | 0.3 | 481.7565 | 2 | 17.36 | 7 | F7:2608 | Fraction\_7\_23092022.raw |  |  |  |  |  |  | 3.8663E4 | 1 | 0 | 0 | 0 | 0 | 0 | 0 | 1 | 63 | 72 |  |  | PEAKS DB |
| total 5 peptides |  |  |  |  |  |  |  |  |  |  |  |  |  |  |  |  |  |  |  |  |  |  |  |  |  |  |  |  |  |  |  |
| --- | --- | --- | --- | --- | --- | --- | --- | --- | --- | --- | --- | --- | --- | --- | --- | --- | --- | --- | --- | --- | --- | --- | --- | --- | --- | --- | --- | --- | --- | --- | --- |

P35781|VA51\_VESCR

back to list

  

| Protein Coverage
| Supporting Peptides
|

Protein Coverage:

Supporting Peptides:

| Peptide | Uniq | -10lgP | Mass | Length | ppm | m/z | z | RT | Fraction | Scan | Source File | Area F1 | Area F2 | Area F3 | Area F4 | Area F5 | Area F6 | Area F7 | #Feature | #Feature F1 | #Feature F2 | #Feature F3 | #Feature F4 | #Feature F5 | #Feature F6 | #Feature F7 | Start | End | PTM | AScore | Found By |
| --- | --- | --- | --- | --- | --- | --- | --- | --- | --- | --- | --- | --- | --- | --- | --- | --- | --- | --- | --- | --- | --- | --- | --- | --- | --- | --- | --- | --- | --- | --- | --- |
| K.ASGLTKQENLEILKQHNEFRQK.V | Y | 84.56 | 2610.3877 | 22 | 3.0 | 523.0864 | 5 | 35.85 | 7 | F7:8886 | Fraction\_7\_23092022.raw |  |  |  |  |  | 4.5984E5 | 1.4646E8 | 3 | 0 | 0 | 0 | 0 | 0 | 1 | 2 | 33 | 54 |  |  | PEAKS DB |
| K.ASGLTKQENLEILKQHNEFR.Q | Y | 75.55 | 2354.2341 | 20 | -0.2 | 589.5657 | 4 | 41.58 | 4 | F4:8850 | Fraction\_4\_23092022.raw |  |  |  | 3.5359E6 |  | 1.9178E6 | 2.9167E7 | 5 | 0 | 0 | 0 | 1 | 0 | 2 | 2 | 33 | 52 |  |  | PEAKS DB |
| R.GLETRGNPGPQPPAK.S | N | 57.93 | 1517.7949 | 15 | -0.4 | 506.9387 | 3 | 22.00 | 7 | F7:3354 | Fraction\_7\_23092022.raw |  |  |  |  |  |  | 7.2952E4 | 1 | 0 | 0 | 0 | 0 | 0 | 0 | 1 | 58 | 72 |  |  | PEAKS DB |
| K.ASGLTKQENLEILK.Q | Y | 54.71 | 1542.8617 | 14 | -0.5 | 515.2943 | 3 | 46.87 | 7 | F7:13890 | Fraction\_7\_23092022.raw |  |  |  | 6.5316E5 |  |  | 2.4172E6 | 3 | 0 | 0 | 0 | 1 | 0 | 0 | 2 | 33 | 46 |  |  | PEAKS DB |
| R.GNPGPQPPAK.S | N | 52.98 | 961.4981 | 10 | 0.3 | 481.7565 | 2 | 17.36 | 7 | F7:2608 | Fraction\_7\_23092022.raw |  |  |  |  |  |  | 3.8663E4 | 1 | 0 | 0 | 0 | 0 | 0 | 0 | 1 | 63 | 72 |  |  | PEAKS DB |
| total 5 peptides |  |  |  |  |  |  |  |  |  |  |  |  |  |  |  |  |  |  |  |  |  |  |  |  |  |  |  |  |  |  |  |
| --- | --- | --- | --- | --- | --- | --- | --- | --- | --- | --- | --- | --- | --- | --- | --- | --- | --- | --- | --- | --- | --- | --- | --- | --- | --- | --- | --- | --- | --- | --- | --- |

TRINITY\_DN2228\_c0\_g2\_i2.p1

back to list

  

| Protein Coverage
| Supporting Peptides
|

Protein Coverage:

Supporting Peptides:

| Peptide | Uniq | -10lgP | Mass | Length | ppm | m/z | z | RT | Fraction | Scan | Source File | Area F1 | Area F2 | Area F3 | Area F4 | Area F5 | Area F6 | Area F7 | #Feature | #Feature F1 | #Feature F2 | #Feature F3 | #Feature F4 | #Feature F5 | #Feature F6 | #Feature F7 | Start | End | PTM | AScore | Found By |
| --- | --- | --- | --- | --- | --- | --- | --- | --- | --- | --- | --- | --- | --- | --- | --- | --- | --- | --- | --- | --- | --- | --- | --- | --- | --- | --- | --- | --- | --- | --- | --- |
| R.ELTPDQRIGLVQGRVKEAVR.I | Y | 73.23 | 2263.2759 | 20 | -0.1 | 453.6624 | 5 | 42.68 | 7 | F7:11954 | Fraction\_7\_23092022.raw |  |  |  |  |  | 2.5389E5 | 7.4035E5 | 2 | 0 | 0 | 0 | 0 | 0 | 1 | 1 | 210 | 229 |  |  | PEAKS DB |
| R.ELTPDQRIGLVQGRVK.E | Y | 64.13 | 1808.0267 | 16 | 0.4 | 453.0142 | 4 | 38.13 | 7 | F7:9871 | Fraction\_7\_23092022.raw |  |  |  |  |  | 5.8158E5 | 1.5611E6 | 3 | 0 | 0 | 0 | 0 | 0 | 1 | 2 | 210 | 225 |  |  | PEAKS DB |
| R.ELTPDQRIGLVQGR.V | Y | 62.17 | 1580.8634 | 14 | 1.0 | 527.9623 | 3 | 41.61 | 7 | F7:11520 | Fraction\_7\_23092022.raw |  |  | 1.2245E6 |  | 5.7715E5 | 5.7725E7 | 1.315E7 | 4 | 0 | 0 | 1 | 0 | 1 | 1 | 1 | 210 | 223 |  |  | PEAKS DB |
| R.RELTPDQRIGLVQGR.V | Y | 59.62 | 1736.9645 | 15 | -1.5 | 435.2477 | 4 | 34.51 | 6 | F6:6210 | Fraction\_6\_23092022.raw |  |  |  |  |  | 6.6849E4 |  | 1 | 0 | 0 | 0 | 0 | 0 | 1 | 0 | 209 | 223 |  |  | PEAKS DB |
| R.NFSGIGIIDFER.W | Y | 51.61 | 1366.6881 | 12 | 0.7 | 684.3518 | 2 | 74.24 | 4 | F4:23508 | Fraction\_4\_23092022.raw |  |  |  | 1.4573E5 |  | 1.8187E5 |  | 2 | 0 | 0 | 0 | 1 | 0 | 1 | 0 | 82 | 93 |  |  | PEAKS DB |
| R.LFM(+15.99)EETLKLAK.K | Y | 49.81 | 1337.7264 | 11 | -0.1 | 446.9160 | 3 | 45.99 | 4 | F4:10849 | Fraction\_4\_23092022.raw |  |  |  | 1.9873E6 |  |  |  | 1 | 0 | 0 | 0 | 1 | 0 | 0 | 0 | 142 | 152 | Oxidation (M) | M3:Oxidation (M):1000.00 | PEAKS DB |
| total 6 peptides |  |  |  |  |  |  |  |  |  |  |  |  |  |  |  |  |  |  |  |  |  |  |  |  |  |  |  |  |  |  |  |
| --- | --- | --- | --- | --- | --- | --- | --- | --- | --- | --- | --- | --- | --- | --- | --- | --- | --- | --- | --- | --- | --- | --- | --- | --- | --- | --- | --- | --- | --- | --- | --- |

TRINITY\_DN2228\_c0\_g2\_i1.p1

back to list

  

| Protein Coverage
| Supporting Peptides
|

Protein Coverage:

Supporting Peptides:

| Peptide | Uniq | -10lgP | Mass | Length | ppm | m/z | z | RT | Fraction | Scan | Source File | Area F1 | Area F2 | Area F3 | Area F4 | Area F5 | Area F6 | Area F7 | #Feature | #Feature F1 | #Feature F2 | #Feature F3 | #Feature F4 | #Feature F5 | #Feature F6 | #Feature F7 | Start | End | PTM | AScore | Found By |
| --- | --- | --- | --- | --- | --- | --- | --- | --- | --- | --- | --- | --- | --- | --- | --- | --- | --- | --- | --- | --- | --- | --- | --- | --- | --- | --- | --- | --- | --- | --- | --- |
| R.ELTPDQRIGLVQGRVKEAVR.I | Y | 73.23 | 2263.2759 | 20 | -0.1 | 453.6624 | 5 | 42.68 | 7 | F7:11954 | Fraction\_7\_23092022.raw |  |  |  |  |  | 2.5389E5 | 7.4035E5 | 2 | 0 | 0 | 0 | 0 | 0 | 1 | 1 | 253 | 272 |  |  | PEAKS DB |
| R.ELTPDQRIGLVQGRVK.E | Y | 64.13 | 1808.0267 | 16 | 0.4 | 453.0142 | 4 | 38.13 | 7 | F7:9871 | Fraction\_7\_23092022.raw |  |  |  |  |  | 5.8158E5 | 1.5611E6 | 3 | 0 | 0 | 0 | 0 | 0 | 1 | 2 | 253 | 268 |  |  | PEAKS DB |
| R.ELTPDQRIGLVQGR.V | Y | 62.17 | 1580.8634 | 14 | 1.0 | 527.9623 | 3 | 41.61 | 7 | F7:11520 | Fraction\_7\_23092022.raw |  |  | 1.2245E6 |  | 5.7715E5 | 5.7725E7 | 1.315E7 | 4 | 0 | 0 | 1 | 0 | 1 | 1 | 1 | 253 | 266 |  |  | PEAKS DB |
| R.RELTPDQRIGLVQGR.V | Y | 59.62 | 1736.9645 | 15 | -1.5 | 435.2477 | 4 | 34.51 | 6 | F6:6210 | Fraction\_6\_23092022.raw |  |  |  |  |  | 6.6849E4 |  | 1 | 0 | 0 | 0 | 0 | 0 | 1 | 0 | 252 | 266 |  |  | PEAKS DB |
| R.NFSGIGIIDFER.W | Y | 51.61 | 1366.6881 | 12 | 0.7 | 684.3518 | 2 | 74.24 | 4 | F4:23508 | Fraction\_4\_23092022.raw |  |  |  | 1.4573E5 |  | 1.8187E5 |  | 2 | 0 | 0 | 0 | 1 | 0 | 1 | 0 | 125 | 136 |  |  | PEAKS DB |
| R.LFM(+15.99)EETLKLAK.K | Y | 49.81 | 1337.7264 | 11 | -0.1 | 446.9160 | 3 | 45.99 | 4 | F4:10849 | Fraction\_4\_23092022.raw |  |  |  | 1.9873E6 |  |  |  | 1 | 0 | 0 | 0 | 1 | 0 | 0 | 0 | 185 | 195 | Oxidation (M) | M3:Oxidation (M):1000.00 | PEAKS DB |
| total 6 peptides |  |  |  |  |  |  |  |  |  |  |  |  |  |  |  |  |  |  |  |  |  |  |  |  |  |  |  |  |  |  |  |
| --- | --- | --- | --- | --- | --- | --- | --- | --- | --- | --- | --- | --- | --- | --- | --- | --- | --- | --- | --- | --- | --- | --- | --- | --- | --- | --- | --- | --- | --- | --- | --- |

P81605|DCD\_HUMAN

back to list

  

| Protein Coverage
| Supporting Peptides
|

Protein Coverage:

Supporting Peptides:

| Peptide | Uniq | -10lgP | Mass | Length | ppm | m/z | z | RT | Fraction | Scan | Source File | Area F1 | Area F2 | Area F3 | Area F4 | Area F5 | Area F6 | Area F7 | #Feature | #Feature F1 | #Feature F2 | #Feature F3 | #Feature F4 | #Feature F5 | #Feature F6 | #Feature F7 | Start | End | PTM | AScore | Found By |
| --- | --- | --- | --- | --- | --- | --- | --- | --- | --- | --- | --- | --- | --- | --- | --- | --- | --- | --- | --- | --- | --- | --- | --- | --- | --- | --- | --- | --- | --- | --- | --- |
| K.LGKDAVEDLESVGK.G | Y | 89.03 | 1458.7566 | 14 | 0.6 | 730.3860 | 2 | 46.58 | 4 | F4:11107 | Fraction\_4\_23092022.raw | 2.7311E6 | 9.7168E5 | 8.7419E5 | 1.3943E6 | 1.133E6 | 8.6708E5 | 1.1205E6 | 13 | 2 | 2 | 2 | 2 | 2 | 2 | 1 | 83 | 96 |  |  | PEAKS DB |
| K.GAVHDVKDVLDSVL | Y | 74.52 | 1465.7776 | 14 | 1.1 | 733.8969 | 2 | 71.67 | 1 | F1:21251 | Fraction\_1\_23092022r.raw | 8.8165E5 | 6.9589E5 | 2.28E5 | 9.1474E5 | 3.0072E5 |  | 2.2789E6 | 8 | 2 | 1 | 1 | 2 | 1 | 0 | 1 | 97 | 110 |  |  | PEAKS DB |
| K.AVGGLGKLGKDAVEDLESVGK.G | Y | 68.91 | 2041.1055 | 21 | 0.5 | 681.3761 | 3 | 60.51 | 2 | F2:16466 | Fraction\_2\_23092022.raw | 7.4799E4 | 2.5245E5 |  |  |  |  |  | 2 | 1 | 1 | 0 | 0 | 0 | 0 | 0 | 76 | 96 |  |  | PEAKS DB |
| R.SSLLEKGLDGAK.K | Y | 65.33 | 1216.6663 | 12 | 0.2 | 406.5628 | 3 | 36.69 | 7 | F7:9243 | Fraction\_7\_23092022.raw |  |  |  | 1.3485E5 | 5.1245E4 | 9.6435E4 | 1.128E5 | 4 | 0 | 0 | 0 | 1 | 1 | 1 | 1 | 63 | 74 |  |  | PEAKS DB |
| total 4 peptides |  |  |  |  |  |  |  |  |  |  |  |  |  |  |  |  |  |  |  |  |  |  |  |  |  |  |  |  |  |  |  |
| --- | --- | --- | --- | --- | --- | --- | --- | --- | --- | --- | --- | --- | --- | --- | --- | --- | --- | --- | --- | --- | --- | --- | --- | --- | --- | --- | --- | --- | --- | --- | --- |

TRINITY\_DN33395\_c0\_g1\_i1.p1

back to list

  

| Protein Coverage
| Supporting Peptides
|

Protein Coverage:

Supporting Peptides:

| Peptide | Uniq | -10lgP | Mass | Length | ppm | m/z | z | RT | Fraction | Scan | Source File | Area F1 | Area F2 | Area F3 | Area F4 | Area F5 | Area F6 | Area F7 | #Feature | #Feature F1 | #Feature F2 | #Feature F3 | #Feature F4 | #Feature F5 | #Feature F6 | #Feature F7 | Start | End | PTM | AScore | Found By |
| --- | --- | --- | --- | --- | --- | --- | --- | --- | --- | --- | --- | --- | --- | --- | --- | --- | --- | --- | --- | --- | --- | --- | --- | --- | --- | --- | --- | --- | --- | --- | --- |
| R.SLVFDNIIEGYSK.R | Y | 75.71 | 1483.7559 | 13 | 0.7 | 742.8857 | 2 | 72.99 | 6 | F6:23358 | Fraction\_6\_23092022.raw |  |  |  |  |  | 3.1009E6 | 3.8819E5 | 2 | 0 | 0 | 0 | 0 | 0 | 1 | 1 | 76 | 88 |  |  | PEAKS DB |
| R.YNNFGVYWVNR.A | Y | 74.41 | 1430.6731 | 11 | 0.5 | 716.3442 | 2 | 62.45 | 6 | F6:18677 | Fraction\_6\_23092022.raw |  |  |  |  |  | 7.636E5 |  | 1 | 0 | 0 | 0 | 0 | 0 | 1 | 0 | 90 | 100 |  |  | PEAKS DB |
| R.SLVFDNIIEGYSKR.Y | Y | 70.98 | 1639.8569 | 14 | 0.5 | 820.9362 | 2 | 64.81 | 6 | F6:19710 | Fraction\_6\_23092022.raw |  |  |  |  | 6.1162E4 | 7.9442E6 | 2.1201E6 | 5 | 0 | 0 | 0 | 0 | 1 | 2 | 2 | 76 | 89 |  |  | PEAKS DB |
| R.RSLVFDNIIEGYSKR.Y | Y | 61.61 | 1795.9580 | 15 | -0.4 | 599.6597 | 3 | 54.32 | 6 | F6:15000 | Fraction\_6\_23092022.raw |  |  |  |  |  | 7.7447E5 |  | 1 | 0 | 0 | 0 | 0 | 0 | 1 | 0 | 75 | 89 |  |  | PEAKS DB |
| R.RSLVFDNIIEGYSK.R | Y | 50.79 | 1639.8569 | 14 | 1.1 | 547.6268 | 3 | 60.43 | 6 | F6:17768 | Fraction\_6\_23092022.raw |  |  |  |  |  | 1.4032E5 |  | 1 | 0 | 0 | 0 | 0 | 0 | 1 | 0 | 75 | 88 |  |  | PEAKS DB |
| total 5 peptides |  |  |  |  |  |  |  |  |  |  |  |  |  |  |  |  |  |  |  |  |  |  |  |  |  |  |  |  |  |  |  |
| --- | --- | --- | --- | --- | --- | --- | --- | --- | --- | --- | --- | --- | --- | --- | --- | --- | --- | --- | --- | --- | --- | --- | --- | --- | --- | --- | --- | --- | --- | --- | --- |

TRINITY\_DN2938\_c0\_g1\_i1.p1

back to list

  

| Protein Coverage
| Supporting Peptides
|

Protein Coverage:

Supporting Peptides:

| Peptide | Uniq | -10lgP | Mass | Length | ppm | m/z | z | RT | Fraction | Scan | Source File | Area F1 | Area F2 | Area F3 | Area F4 | Area F5 | Area F6 | Area F7 | #Feature | #Feature F1 | #Feature F2 | #Feature F3 | #Feature F4 | #Feature F5 | #Feature F6 | #Feature F7 | Start | End | PTM | AScore | Found By |
| --- | --- | --- | --- | --- | --- | --- | --- | --- | --- | --- | --- | --- | --- | --- | --- | --- | --- | --- | --- | --- | --- | --- | --- | --- | --- | --- | --- | --- | --- | --- | --- |
| K.SFESIGPILPNLEK.K | Y | 89.39 | 1542.8293 | 14 | 0.7 | 772.4225 | 2 | 72.57 | 3 | F3:22184 | Fraction\_3\_23092022.raw |  |  | 3.1013E6 | 9.1105E4 | 1.6627E5 | 1.134E6 | 6.6382E5 | 5 | 0 | 0 | 1 | 1 | 1 | 1 | 1 | 247 | 260 |  |  | PEAKS DB |
| K.SLDKLETLDLSNNR.I | Y | 66.87 | 1616.8369 | 14 | 0.8 | 539.9534 | 3 | 48.83 | 6 | F6:12520 | Fraction\_6\_23092022.raw |  |  | 8.0244E4 |  |  | 2.153E5 |  | 2 | 0 | 0 | 1 | 0 | 0 | 1 | 0 | 105 | 118 |  |  | PEAKS DB |
| R.ITGC(+57.02)SLEKIEDDAFK.S | Y | 64.62 | 1724.8291 | 15 | 1.5 | 575.9512 | 3 | 52.85 | 6 | F6:14345 | Fraction\_6\_23092022.raw |  |  | 1.9808E5 |  |  | 1.8826E5 |  | 2 | 0 | 0 | 1 | 0 | 0 | 1 | 0 | 90 | 104 | Carbamidomethylation | C4:Carbamidomethylation:1000.00 | PEAKS DB |
| R.FAGSLIELR.I | Y | 60.91 | 1004.5654 | 9 | 0.3 | 503.2901 | 2 | 58.22 | 6 | F6:16757 | Fraction\_6\_23092022.raw |  |  | 8.1851E5 |  |  | 6.0674E5 |  | 2 | 0 | 0 | 1 | 0 | 0 | 1 | 0 | 81 | 89 |  |  | PEAKS DB |
| total 4 peptides |  |  |  |  |  |  |  |  |  |  |  |  |  |  |  |  |  |  |  |  |  |  |  |  |  |  |  |  |  |  |  |
| --- | --- | --- | --- | --- | --- | --- | --- | --- | --- | --- | --- | --- | --- | --- | --- | --- | --- | --- | --- | --- | --- | --- | --- | --- | --- | --- | --- | --- | --- | --- | --- |

TRINITY\_DN273\_c0\_g4\_i11.p1

back to list

  

| Protein Coverage
| Supporting Peptides
|

Protein Coverage:

Supporting Peptides:

| Peptide | Uniq | -10lgP | Mass | Length | ppm | m/z | z | RT | Fraction | Scan | Source File | Area F1 | Area F2 | Area F3 | Area F4 | Area F5 | Area F6 | Area F7 | #Feature | #Feature F1 | #Feature F2 | #Feature F3 | #Feature F4 | #Feature F5 | #Feature F6 | #Feature F7 | Start | End | PTM | AScore | Found By |
| --- | --- | --- | --- | --- | --- | --- | --- | --- | --- | --- | --- | --- | --- | --- | --- | --- | --- | --- | --- | --- | --- | --- | --- | --- | --- | --- | --- | --- | --- | --- | --- |
| K.DLYANTVLSGGTTMYPGIADR.M | Y | 83.63 | 2214.0627 | 21 | 0.8 | 1108.0396 | 2 | 73.94 | 1 | F1:22276 | Fraction\_1\_23092022r.raw | 1.3622E5 |  |  |  |  |  |  | 1 | 1 | 0 | 0 | 0 | 0 | 0 | 0 | 293 | 313 |  |  | PEAKS DB |
| K.SYELPDGQVITIGNER.F | N | 80.24 | 1789.8846 | 16 | 0.5 | 895.9501 | 2 | 65.25 | 3 | F3:18910 | Fraction\_3\_23092022.raw | 3.1717E5 | 6.818E4 | 5.522E5 |  |  | 1.4708E5 |  | 4 | 1 | 1 | 1 | 0 | 0 | 1 | 0 | 240 | 255 |  |  | PEAKS DB |
| R.AVFPSIVGRPR.H | N | 61.14 | 1197.6981 | 11 | -0.2 | 400.2399 | 3 | 45.42 | 3 | F3:10004 | Fraction\_3\_23092022.raw |  |  | 5.4273E5 |  |  |  |  | 1 | 0 | 0 | 1 | 0 | 0 | 0 | 0 | 30 | 40 |  |  | PEAKS DB |
| R.VAPEEHPVLLTEAPLNPK.A | Y | 59.19 | 1953.0570 | 18 | -0.4 | 652.0260 | 3 | 52.34 | 1 | F1:12620 | Fraction\_1\_23092022r.raw | 2.3939E5 |  |  |  |  |  |  | 1 | 1 | 0 | 0 | 0 | 0 | 0 | 0 | 97 | 114 |  |  | PEAKS DB |
| total 4 peptides |  |  |  |  |  |  |  |  |  |  |  |  |  |  |  |  |  |  |  |  |  |  |  |  |  |  |  |  |  |  |  |
| --- | --- | --- | --- | --- | --- | --- | --- | --- | --- | --- | --- | --- | --- | --- | --- | --- | --- | --- | --- | --- | --- | --- | --- | --- | --- | --- | --- | --- | --- | --- | --- |

P83750|ACTB\_CYPCA

back to list

  

| Protein Coverage
| Supporting Peptides
|

Protein Coverage:

Supporting Peptides:

| Peptide | Uniq | -10lgP | Mass | Length | ppm | m/z | z | RT | Fraction | Scan | Source File | Area F1 | Area F2 | Area F3 | Area F4 | Area F5 | Area F6 | Area F7 | #Feature | #Feature F1 | #Feature F2 | #Feature F3 | #Feature F4 | #Feature F5 | #Feature F6 | #Feature F7 | Start | End | PTM | AScore | Found By |
| --- | --- | --- | --- | --- | --- | --- | --- | --- | --- | --- | --- | --- | --- | --- | --- | --- | --- | --- | --- | --- | --- | --- | --- | --- | --- | --- | --- | --- | --- | --- | --- |
| K.DLYANTVLSGGTTMYPGIADR.M | Y | 83.63 | 2214.0627 | 21 | 0.8 | 1108.0396 | 2 | 73.94 | 1 | F1:22276 | Fraction\_1\_23092022r.raw | 1.3622E5 |  |  |  |  |  |  | 1 | 1 | 0 | 0 | 0 | 0 | 0 | 0 | 292 | 312 |  |  | PEAKS DB |
| K.SYELPDGQVITIGNER.F | N | 80.24 | 1789.8846 | 16 | 0.5 | 895.9501 | 2 | 65.25 | 3 | F3:18910 | Fraction\_3\_23092022.raw | 3.1717E5 | 6.818E4 | 5.522E5 |  |  | 1.4708E5 |  | 4 | 1 | 1 | 1 | 0 | 0 | 1 | 0 | 239 | 254 |  |  | PEAKS DB |
| R.AVFPSIVGRPR.H | N | 61.14 | 1197.6981 | 11 | -0.2 | 400.2399 | 3 | 45.42 | 3 | F3:10004 | Fraction\_3\_23092022.raw |  |  | 5.4273E5 |  |  |  |  | 1 | 0 | 0 | 1 | 0 | 0 | 0 | 0 | 29 | 39 |  |  | PEAKS DB |
| R.VAPEEHPVLLTEAPLNPK.A | Y | 59.19 | 1953.0570 | 18 | -0.4 | 652.0260 | 3 | 52.34 | 1 | F1:12620 | Fraction\_1\_23092022r.raw | 2.3939E5 |  |  |  |  |  |  | 1 | 1 | 0 | 0 | 0 | 0 | 0 | 0 | 96 | 113 |  |  | PEAKS DB |
| total 4 peptides |  |  |  |  |  |  |  |  |  |  |  |  |  |  |  |  |  |  |  |  |  |  |  |  |  |  |  |  |  |  |  |
| --- | --- | --- | --- | --- | --- | --- | --- | --- | --- | --- | --- | --- | --- | --- | --- | --- | --- | --- | --- | --- | --- | --- | --- | --- | --- | --- | --- | --- | --- | --- | --- |

P02572|ACT2\_DROME

back to list

  

| Protein Coverage
| Supporting Peptides
|

Protein Coverage:

Supporting Peptides:

| Peptide | Uniq | -10lgP | Mass | Length | ppm | m/z | z | RT | Fraction | Scan | Source File | Area F1 | Area F2 | Area F3 | Area F4 | Area F5 | Area F6 | Area F7 | #Feature | #Feature F1 | #Feature F2 | #Feature F3 | #Feature F4 | #Feature F5 | #Feature F6 | #Feature F7 | Start | End | PTM | AScore | Found By |
| --- | --- | --- | --- | --- | --- | --- | --- | --- | --- | --- | --- | --- | --- | --- | --- | --- | --- | --- | --- | --- | --- | --- | --- | --- | --- | --- | --- | --- | --- | --- | --- |
| K.DLYANTVLSGGTTMYPGIADR.M | Y | 83.63 | 2214.0627 | 21 | 0.8 | 1108.0396 | 2 | 73.94 | 1 | F1:22276 | Fraction\_1\_23092022r.raw | 1.3622E5 |  |  |  |  |  |  | 1 | 1 | 0 | 0 | 0 | 0 | 0 | 0 | 293 | 313 |  |  | PEAKS DB |
| K.SYELPDGQVITIGNER.F | N | 80.24 | 1789.8846 | 16 | 0.5 | 895.9501 | 2 | 65.25 | 3 | F3:18910 | Fraction\_3\_23092022.raw | 3.1717E5 | 6.818E4 | 5.522E5 |  |  | 1.4708E5 |  | 4 | 1 | 1 | 1 | 0 | 0 | 1 | 0 | 240 | 255 |  |  | PEAKS DB |
| R.AVFPSIVGRPR.H | N | 61.14 | 1197.6981 | 11 | -0.2 | 400.2399 | 3 | 45.42 | 3 | F3:10004 | Fraction\_3\_23092022.raw |  |  | 5.4273E5 |  |  |  |  | 1 | 0 | 0 | 1 | 0 | 0 | 0 | 0 | 30 | 40 |  |  | PEAKS DB |
| R.VAPEEHPVLLTEAPLNPK.A | Y | 59.19 | 1953.0570 | 18 | -0.4 | 652.0260 | 3 | 52.34 | 1 | F1:12620 | Fraction\_1\_23092022r.raw | 2.3939E5 |  |  |  |  |  |  | 1 | 1 | 0 | 0 | 0 | 0 | 0 | 0 | 97 | 114 |  |  | PEAKS DB |
| total 4 peptides |  |  |  |  |  |  |  |  |  |  |  |  |  |  |  |  |  |  |  |  |  |  |  |  |  |  |  |  |  |  |  |
| --- | --- | --- | --- | --- | --- | --- | --- | --- | --- | --- | --- | --- | --- | --- | --- | --- | --- | --- | --- | --- | --- | --- | --- | --- | --- | --- | --- | --- | --- | --- | --- |

P60713|ACTB\_SHEEP

back to list

  

| Protein Coverage
| Supporting Peptides
|

Protein Coverage:

Supporting Peptides:

| Peptide | Uniq | -10lgP | Mass | Length | ppm | m/z | z | RT | Fraction | Scan | Source File | Area F1 | Area F2 | Area F3 | Area F4 | Area F5 | Area F6 | Area F7 | #Feature | #Feature F1 | #Feature F2 | #Feature F3 | #Feature F4 | #Feature F5 | #Feature F6 | #Feature F7 | Start | End | PTM | AScore | Found By |
| --- | --- | --- | --- | --- | --- | --- | --- | --- | --- | --- | --- | --- | --- | --- | --- | --- | --- | --- | --- | --- | --- | --- | --- | --- | --- | --- | --- | --- | --- | --- | --- |
| K.DLYANTVLSGGTTMYPGIADR.M | Y | 83.63 | 2214.0627 | 21 | 0.8 | 1108.0396 | 2 | 73.94 | 1 | F1:22276 | Fraction\_1\_23092022r.raw | 1.3622E5 |  |  |  |  |  |  | 1 | 1 | 0 | 0 | 0 | 0 | 0 | 0 | 292 | 312 |  |  | PEAKS DB |
| K.SYELPDGQVITIGNER.F | N | 80.24 | 1789.8846 | 16 | 0.5 | 895.9501 | 2 | 65.25 | 3 | F3:18910 | Fraction\_3\_23092022.raw | 3.1717E5 | 6.818E4 | 5.522E5 |  |  | 1.4708E5 |  | 4 | 1 | 1 | 1 | 0 | 0 | 1 | 0 | 239 | 254 |  |  | PEAKS DB |
| R.AVFPSIVGRPR.H | N | 61.14 | 1197.6981 | 11 | -0.2 | 400.2399 | 3 | 45.42 | 3 | F3:10004 | Fraction\_3\_23092022.raw |  |  | 5.4273E5 |  |  |  |  | 1 | 0 | 0 | 1 | 0 | 0 | 0 | 0 | 29 | 39 |  |  | PEAKS DB |
| R.VAPEEHPVLLTEAPLNPK.A | Y | 59.19 | 1953.0570 | 18 | -0.4 | 652.0260 | 3 | 52.34 | 1 | F1:12620 | Fraction\_1\_23092022r.raw | 2.3939E5 |  |  |  |  |  |  | 1 | 1 | 0 | 0 | 0 | 0 | 0 | 0 | 96 | 113 |  |  | PEAKS DB |
| total 4 peptides |  |  |  |  |  |  |  |  |  |  |  |  |  |  |  |  |  |  |  |  |  |  |  |  |  |  |  |  |  |  |  |
| --- | --- | --- | --- | --- | --- | --- | --- | --- | --- | --- | --- | --- | --- | --- | --- | --- | --- | --- | --- | --- | --- | --- | --- | --- | --- | --- | --- | --- | --- | --- | --- |

Q4R561|ACTB\_MACFA

back to list

  

| Protein Coverage
| Supporting Peptides
|

Protein Coverage:

Supporting Peptides:

| Peptide | Uniq | -10lgP | Mass | Length | ppm | m/z | z | RT | Fraction | Scan | Source File | Area F1 | Area F2 | Area F3 | Area F4 | Area F5 | Area F6 | Area F7 | #Feature | #Feature F1 | #Feature F2 | #Feature F3 | #Feature F4 | #Feature F5 | #Feature F6 | #Feature F7 | Start | End | PTM | AScore | Found By |
| --- | --- | --- | --- | --- | --- | --- | --- | --- | --- | --- | --- | --- | --- | --- | --- | --- | --- | --- | --- | --- | --- | --- | --- | --- | --- | --- | --- | --- | --- | --- | --- |
| K.DLYANTVLSGGTTMYPGIADR.M | Y | 83.63 | 2214.0627 | 21 | 0.8 | 1108.0396 | 2 | 73.94 | 1 | F1:22276 | Fraction\_1\_23092022r.raw | 1.3622E5 |  |  |  |  |  |  | 1 | 1 | 0 | 0 | 0 | 0 | 0 | 0 | 292 | 312 |  |  | PEAKS DB |
| K.SYELPDGQVITIGNER.F | N | 80.24 | 1789.8846 | 16 | 0.5 | 895.9501 | 2 | 65.25 | 3 | F3:18910 | Fraction\_3\_23092022.raw | 3.1717E5 | 6.818E4 | 5.522E5 |  |  | 1.4708E5 |  | 4 | 1 | 1 | 1 | 0 | 0 | 1 | 0 | 239 | 254 |  |  | PEAKS DB |
| R.AVFPSIVGRPR.H | N | 61.14 | 1197.6981 | 11 | -0.2 | 400.2399 | 3 | 45.42 | 3 | F3:10004 | Fraction\_3\_23092022.raw |  |  | 5.4273E5 |  |  |  |  | 1 | 0 | 0 | 1 | 0 | 0 | 0 | 0 | 29 | 39 |  |  | PEAKS DB |
| R.VAPEEHPVLLTEAPLNPK.A | Y | 59.19 | 1953.0570 | 18 | -0.4 | 652.0260 | 3 | 52.34 | 1 | F1:12620 | Fraction\_1\_23092022r.raw | 2.3939E5 |  |  |  |  |  |  | 1 | 1 | 0 | 0 | 0 | 0 | 0 | 0 | 96 | 113 |  |  | PEAKS DB |
| total 4 peptides |  |  |  |  |  |  |  |  |  |  |  |  |  |  |  |  |  |  |  |  |  |  |  |  |  |  |  |  |  |  |  |
| --- | --- | --- | --- | --- | --- | --- | --- | --- | --- | --- | --- | --- | --- | --- | --- | --- | --- | --- | --- | --- | --- | --- | --- | --- | --- | --- | --- | --- | --- | --- | --- |

Q76N69|ACTB\_CHLAE

back to list

  

| Protein Coverage
| Supporting Peptides
|

Protein Coverage:

Supporting Peptides:

| Peptide | Uniq | -10lgP | Mass | Length | ppm | m/z | z | RT | Fraction | Scan | Source File | Area F1 | Area F2 | Area F3 | Area F4 | Area F5 | Area F6 | Area F7 | #Feature | #Feature F1 | #Feature F2 | #Feature F3 | #Feature F4 | #Feature F5 | #Feature F6 | #Feature F7 | Start | End | PTM | AScore | Found By |
| --- | --- | --- | --- | --- | --- | --- | --- | --- | --- | --- | --- | --- | --- | --- | --- | --- | --- | --- | --- | --- | --- | --- | --- | --- | --- | --- | --- | --- | --- | --- | --- |
| K.DLYANTVLSGGTTMYPGIADR.M | Y | 83.63 | 2214.0627 | 21 | 0.8 | 1108.0396 | 2 | 73.94 | 1 | F1:22276 | Fraction\_1\_23092022r.raw | 1.3622E5 |  |  |  |  |  |  | 1 | 1 | 0 | 0 | 0 | 0 | 0 | 0 | 292 | 312 |  |  | PEAKS DB |
| K.SYELPDGQVITIGNER.F | N | 80.24 | 1789.8846 | 16 | 0.5 | 895.9501 | 2 | 65.25 | 3 | F3:18910 | Fraction\_3\_23092022.raw | 3.1717E5 | 6.818E4 | 5.522E5 |  |  | 1.4708E5 |  | 4 | 1 | 1 | 1 | 0 | 0 | 1 | 0 | 239 | 254 |  |  | PEAKS DB |
| R.AVFPSIVGRPR.H | N | 61.14 | 1197.6981 | 11 | -0.2 | 400.2399 | 3 | 45.42 | 3 | F3:10004 | Fraction\_3\_23092022.raw |  |  | 5.4273E5 |  |  |  |  | 1 | 0 | 0 | 1 | 0 | 0 | 0 | 0 | 29 | 39 |  |  | PEAKS DB |
| R.VAPEEHPVLLTEAPLNPK.A | Y | 59.19 | 1953.0570 | 18 | -0.4 | 652.0260 | 3 | 52.34 | 1 | F1:12620 | Fraction\_1\_23092022r.raw | 2.3939E5 |  |  |  |  |  |  | 1 | 1 | 0 | 0 | 0 | 0 | 0 | 0 | 96 | 113 |  |  | PEAKS DB |
| total 4 peptides |  |  |  |  |  |  |  |  |  |  |  |  |  |  |  |  |  |  |  |  |  |  |  |  |  |  |  |  |  |  |  |
| --- | --- | --- | --- | --- | --- | --- | --- | --- | --- | --- | --- | --- | --- | --- | --- | --- | --- | --- | --- | --- | --- | --- | --- | --- | --- | --- | --- | --- | --- | --- | --- |

Q4L0Y2|ACTB\_SPECI

back to list

  

| Protein Coverage
| Supporting Peptides
|

Protein Coverage:

Supporting Peptides:

| Peptide | Uniq | -10lgP | Mass | Length | ppm | m/z | z | RT | Fraction | Scan | Source File | Area F1 | Area F2 | Area F3 | Area F4 | Area F5 | Area F6 | Area F7 | #Feature | #Feature F1 | #Feature F2 | #Feature F3 | #Feature F4 | #Feature F5 | #Feature F6 | #Feature F7 | Start | End | PTM | AScore | Found By |
| --- | --- | --- | --- | --- | --- | --- | --- | --- | --- | --- | --- | --- | --- | --- | --- | --- | --- | --- | --- | --- | --- | --- | --- | --- | --- | --- | --- | --- | --- | --- | --- |
| K.DLYANTVLSGGTTMYPGIADR.M | Y | 83.63 | 2214.0627 | 21 | 0.8 | 1108.0396 | 2 | 73.94 | 1 | F1:22276 | Fraction\_1\_23092022r.raw | 1.3622E5 |  |  |  |  |  |  | 1 | 1 | 0 | 0 | 0 | 0 | 0 | 0 | 292 | 312 |  |  | PEAKS DB |
| K.SYELPDGQVITIGNER.F | N | 80.24 | 1789.8846 | 16 | 0.5 | 895.9501 | 2 | 65.25 | 3 | F3:18910 | Fraction\_3\_23092022.raw | 3.1717E5 | 6.818E4 | 5.522E5 |  |  | 1.4708E5 |  | 4 | 1 | 1 | 1 | 0 | 0 | 1 | 0 | 239 | 254 |  |  | PEAKS DB |
| R.AVFPSIVGRPR.H | N | 61.14 | 1197.6981 | 11 | -0.2 | 400.2399 | 3 | 45.42 | 3 | F3:10004 | Fraction\_3\_23092022.raw |  |  | 5.4273E5 |  |  |  |  | 1 | 0 | 0 | 1 | 0 | 0 | 0 | 0 | 29 | 39 |  |  | PEAKS DB |
| R.VAPEEHPVLLTEAPLNPK.A | Y | 59.19 | 1953.0570 | 18 | -0.4 | 652.0260 | 3 | 52.34 | 1 | F1:12620 | Fraction\_1\_23092022r.raw | 2.3939E5 |  |  |  |  |  |  | 1 | 1 | 0 | 0 | 0 | 0 | 0 | 0 | 96 | 113 |  |  | PEAKS DB |
| total 4 peptides |  |  |  |  |  |  |  |  |  |  |  |  |  |  |  |  |  |  |  |  |  |  |  |  |  |  |  |  |  |  |  |
| --- | --- | --- | --- | --- | --- | --- | --- | --- | --- | --- | --- | --- | --- | --- | --- | --- | --- | --- | --- | --- | --- | --- | --- | --- | --- | --- | --- | --- | --- | --- | --- |

TRINITY\_DN273\_c0\_g4\_i2.p1

back to list

  

| Protein Coverage
| Supporting Peptides
|

Protein Coverage:

Supporting Peptides:

| Peptide | Uniq | -10lgP | Mass | Length | ppm | m/z | z | RT | Fraction | Scan | Source File | Area F1 | Area F2 | Area F3 | Area F4 | Area F5 | Area F6 | Area F7 | #Feature | #Feature F1 | #Feature F2 | #Feature F3 | #Feature F4 | #Feature F5 | #Feature F6 | #Feature F7 | Start | End | PTM | AScore | Found By |
| --- | --- | --- | --- | --- | --- | --- | --- | --- | --- | --- | --- | --- | --- | --- | --- | --- | --- | --- | --- | --- | --- | --- | --- | --- | --- | --- | --- | --- | --- | --- | --- |
| K.DLYANTVLSGGTTMYPGIADR.M | Y | 83.63 | 2214.0627 | 21 | 0.8 | 1108.0396 | 2 | 73.94 | 1 | F1:22276 | Fraction\_1\_23092022r.raw | 1.3622E5 |  |  |  |  |  |  | 1 | 1 | 0 | 0 | 0 | 0 | 0 | 0 | 293 | 313 |  |  | PEAKS DB |
| K.SYELPDGQVITIGNER.F | N | 80.24 | 1789.8846 | 16 | 0.5 | 895.9501 | 2 | 65.25 | 3 | F3:18910 | Fraction\_3\_23092022.raw | 3.1717E5 | 6.818E4 | 5.522E5 |  |  | 1.4708E5 |  | 4 | 1 | 1 | 1 | 0 | 0 | 1 | 0 | 240 | 255 |  |  | PEAKS DB |
| R.AVFPSIVGRPR.H | N | 61.14 | 1197.6981 | 11 | -0.2 | 400.2399 | 3 | 45.42 | 3 | F3:10004 | Fraction\_3\_23092022.raw |  |  | 5.4273E5 |  |  |  |  | 1 | 0 | 0 | 1 | 0 | 0 | 0 | 0 | 30 | 40 |  |  | PEAKS DB |
| R.VAPEEHPVLLTEAPLNPK.A | Y | 59.19 | 1953.0570 | 18 | -0.4 | 652.0260 | 3 | 52.34 | 1 | F1:12620 | Fraction\_1\_23092022r.raw | 2.3939E5 |  |  |  |  |  |  | 1 | 1 | 0 | 0 | 0 | 0 | 0 | 0 | 97 | 114 |  |  | PEAKS DB |
| total 4 peptides |  |  |  |  |  |  |  |  |  |  |  |  |  |  |  |  |  |  |  |  |  |  |  |  |  |  |  |  |  |  |  |
| --- | --- | --- | --- | --- | --- | --- | --- | --- | --- | --- | --- | --- | --- | --- | --- | --- | --- | --- | --- | --- | --- | --- | --- | --- | --- | --- | --- | --- | --- | --- | --- |

TRINITY\_DN273\_c0\_g4\_i4.p1

back to list

  

| Protein Coverage
| Supporting Peptides
|

Protein Coverage:

Supporting Peptides:

| Peptide | Uniq | -10lgP | Mass | Length | ppm | m/z | z | RT | Fraction | Scan | Source File | Area F1 | Area F2 | Area F3 | Area F4 | Area F5 | Area F6 | Area F7 | #Feature | #Feature F1 | #Feature F2 | #Feature F3 | #Feature F4 | #Feature F5 | #Feature F6 | #Feature F7 | Start | End | PTM | AScore | Found By |
| --- | --- | --- | --- | --- | --- | --- | --- | --- | --- | --- | --- | --- | --- | --- | --- | --- | --- | --- | --- | --- | --- | --- | --- | --- | --- | --- | --- | --- | --- | --- | --- |
| K.DLYANTVLSGGTTMYPGIADR.M | Y | 83.63 | 2214.0627 | 21 | 0.8 | 1108.0396 | 2 | 73.94 | 1 | F1:22276 | Fraction\_1\_23092022r.raw | 1.3622E5 |  |  |  |  |  |  | 1 | 1 | 0 | 0 | 0 | 0 | 0 | 0 | 293 | 313 |  |  | PEAKS DB |
| K.SYELPDGQVITIGNER.F | N | 80.24 | 1789.8846 | 16 | 0.5 | 895.9501 | 2 | 65.25 | 3 | F3:18910 | Fraction\_3\_23092022.raw | 3.1717E5 | 6.818E4 | 5.522E5 |  |  | 1.4708E5 |  | 4 | 1 | 1 | 1 | 0 | 0 | 1 | 0 | 240 | 255 |  |  | PEAKS DB |
| R.AVFPSIVGRPR.H | N | 61.14 | 1197.6981 | 11 | -0.2 | 400.2399 | 3 | 45.42 | 3 | F3:10004 | Fraction\_3\_23092022.raw |  |  | 5.4273E5 |  |  |  |  | 1 | 0 | 0 | 1 | 0 | 0 | 0 | 0 | 30 | 40 |  |  | PEAKS DB |
| R.VAPEEHPVLLTEAPLNPK.A | Y | 59.19 | 1953.0570 | 18 | -0.4 | 652.0260 | 3 | 52.34 | 1 | F1:12620 | Fraction\_1\_23092022r.raw | 2.3939E5 |  |  |  |  |  |  | 1 | 1 | 0 | 0 | 0 | 0 | 0 | 0 | 97 | 114 |  |  | PEAKS DB |
| total 4 peptides |  |  |  |  |  |  |  |  |  |  |  |  |  |  |  |  |  |  |  |  |  |  |  |  |  |  |  |  |  |  |  |
| --- | --- | --- | --- | --- | --- | --- | --- | --- | --- | --- | --- | --- | --- | --- | --- | --- | --- | --- | --- | --- | --- | --- | --- | --- | --- | --- | --- | --- | --- | --- | --- |

TRINITY\_DN273\_c0\_g4\_i9.p1

back to list

  

| Protein Coverage
| Supporting Peptides
|

Protein Coverage:

Supporting Peptides:

| Peptide | Uniq | -10lgP | Mass | Length | ppm | m/z | z | RT | Fraction | Scan | Source File | Area F1 | Area F2 | Area F3 | Area F4 | Area F5 | Area F6 | Area F7 | #Feature | #Feature F1 | #Feature F2 | #Feature F3 | #Feature F4 | #Feature F5 | #Feature F6 | #Feature F7 | Start | End | PTM | AScore | Found By |
| --- | --- | --- | --- | --- | --- | --- | --- | --- | --- | --- | --- | --- | --- | --- | --- | --- | --- | --- | --- | --- | --- | --- | --- | --- | --- | --- | --- | --- | --- | --- | --- |
| K.DLYANTVLSGGTTMYPGIADR.M | Y | 83.63 | 2214.0627 | 21 | 0.8 | 1108.0396 | 2 | 73.94 | 1 | F1:22276 | Fraction\_1\_23092022r.raw | 1.3622E5 |  |  |  |  |  |  | 1 | 1 | 0 | 0 | 0 | 0 | 0 | 0 | 293 | 313 |  |  | PEAKS DB |
| K.SYELPDGQVITIGNER.F | N | 80.24 | 1789.8846 | 16 | 0.5 | 895.9501 | 2 | 65.25 | 3 | F3:18910 | Fraction\_3\_23092022.raw | 3.1717E5 | 6.818E4 | 5.522E5 |  |  | 1.4708E5 |  | 4 | 1 | 1 | 1 | 0 | 0 | 1 | 0 | 240 | 255 |  |  | PEAKS DB |
| R.AVFPSIVGRPR.H | N | 61.14 | 1197.6981 | 11 | -0.2 | 400.2399 | 3 | 45.42 | 3 | F3:10004 | Fraction\_3\_23092022.raw |  |  | 5.4273E5 |  |  |  |  | 1 | 0 | 0 | 1 | 0 | 0 | 0 | 0 | 30 | 40 |  |  | PEAKS DB |
| R.VAPEEHPVLLTEAPLNPK.A | Y | 59.19 | 1953.0570 | 18 | -0.4 | 652.0260 | 3 | 52.34 | 1 | F1:12620 | Fraction\_1\_23092022r.raw | 2.3939E5 |  |  |  |  |  |  | 1 | 1 | 0 | 0 | 0 | 0 | 0 | 0 | 97 | 114 |  |  | PEAKS DB |
| total 4 peptides |  |  |  |  |  |  |  |  |  |  |  |  |  |  |  |  |  |  |  |  |  |  |  |  |  |  |  |  |  |  |  |
| --- | --- | --- | --- | --- | --- | --- | --- | --- | --- | --- | --- | --- | --- | --- | --- | --- | --- | --- | --- | --- | --- | --- | --- | --- | --- | --- | --- | --- | --- | --- | --- |

P84856|ACTB\_CHLPG

back to list

  

| Protein Coverage
| Supporting Peptides
|

Protein Coverage:

Supporting Peptides:

| Peptide | Uniq | -10lgP | Mass | Length | ppm | m/z | z | RT | Fraction | Scan | Source File | Area F1 | Area F2 | Area F3 | Area F4 | Area F5 | Area F6 | Area F7 | #Feature | #Feature F1 | #Feature F2 | #Feature F3 | #Feature F4 | #Feature F5 | #Feature F6 | #Feature F7 | Start | End | PTM | AScore | Found By |
| --- | --- | --- | --- | --- | --- | --- | --- | --- | --- | --- | --- | --- | --- | --- | --- | --- | --- | --- | --- | --- | --- | --- | --- | --- | --- | --- | --- | --- | --- | --- | --- |
| K.DLYANTVLSGGTTMYPGIADR.M | Y | 83.63 | 2214.0627 | 21 | 0.8 | 1108.0396 | 2 | 73.94 | 1 | F1:22276 | Fraction\_1\_23092022r.raw | 1.3622E5 |  |  |  |  |  |  | 1 | 1 | 0 | 0 | 0 | 0 | 0 | 0 | 278 | 298 |  |  | PEAKS DB |
| K.SYELPDGQVITIGNER.F | N | 80.24 | 1789.8846 | 16 | 0.5 | 895.9501 | 2 | 65.25 | 3 | F3:18910 | Fraction\_3\_23092022.raw | 3.1717E5 | 6.818E4 | 5.522E5 |  |  | 1.4708E5 |  | 4 | 1 | 1 | 1 | 0 | 0 | 1 | 0 | 225 | 240 |  |  | PEAKS DB |
| R.AVFPSIVGRPR.H | N | 61.14 | 1197.6981 | 11 | -0.2 | 400.2399 | 3 | 45.42 | 3 | F3:10004 | Fraction\_3\_23092022.raw |  |  | 5.4273E5 |  |  |  |  | 1 | 0 | 0 | 1 | 0 | 0 | 0 | 0 | 14 | 24 |  |  | PEAKS DB |
| R.VAPEEHPVLLTEAPLNPK.A | Y | 59.19 | 1953.0570 | 18 | -0.4 | 652.0260 | 3 | 52.34 | 1 | F1:12620 | Fraction\_1\_23092022r.raw | 2.3939E5 |  |  |  |  |  |  | 1 | 1 | 0 | 0 | 0 | 0 | 0 | 0 | 81 | 98 |  |  | PEAKS DB |
| total 4 peptides |  |  |  |  |  |  |  |  |  |  |  |  |  |  |  |  |  |  |  |  |  |  |  |  |  |  |  |  |  |  |  |
| --- | --- | --- | --- | --- | --- | --- | --- | --- | --- | --- | --- | --- | --- | --- | --- | --- | --- | --- | --- | --- | --- | --- | --- | --- | --- | --- | --- | --- | --- | --- | --- |

P63258|ACTG\_BOVIN

back to list

  

| Protein Coverage
| Supporting Peptides
|

Protein Coverage:

Supporting Peptides:

| Peptide | Uniq | -10lgP | Mass | Length | ppm | m/z | z | RT | Fraction | Scan | Source File | Area F1 | Area F2 | Area F3 | Area F4 | Area F5 | Area F6 | Area F7 | #Feature | #Feature F1 | #Feature F2 | #Feature F3 | #Feature F4 | #Feature F5 | #Feature F6 | #Feature F7 | Start | End | PTM | AScore | Found By |
| --- | --- | --- | --- | --- | --- | --- | --- | --- | --- | --- | --- | --- | --- | --- | --- | --- | --- | --- | --- | --- | --- | --- | --- | --- | --- | --- | --- | --- | --- | --- | --- |
| K.DLYANTVLSGGTTMYPGIADR.M | Y | 83.63 | 2214.0627 | 21 | 0.8 | 1108.0396 | 2 | 73.94 | 1 | F1:22276 | Fraction\_1\_23092022r.raw | 1.3622E5 |  |  |  |  |  |  | 1 | 1 | 0 | 0 | 0 | 0 | 0 | 0 | 292 | 312 |  |  | PEAKS DB |
| K.SYELPDGQVITIGNER.F | N | 80.24 | 1789.8846 | 16 | 0.5 | 895.9501 | 2 | 65.25 | 3 | F3:18910 | Fraction\_3\_23092022.raw | 3.1717E5 | 6.818E4 | 5.522E5 |  |  | 1.4708E5 |  | 4 | 1 | 1 | 1 | 0 | 0 | 1 | 0 | 239 | 254 |  |  | PEAKS DB |
| R.AVFPSIVGRPR.H | N | 61.14 | 1197.6981 | 11 | -0.2 | 400.2399 | 3 | 45.42 | 3 | F3:10004 | Fraction\_3\_23092022.raw |  |  | 5.4273E5 |  |  |  |  | 1 | 0 | 0 | 1 | 0 | 0 | 0 | 0 | 29 | 39 |  |  | PEAKS DB |
| R.VAPEEHPVLLTEAPLNPK.A | Y | 59.19 | 1953.0570 | 18 | -0.4 | 652.0260 | 3 | 52.34 | 1 | F1:12620 | Fraction\_1\_23092022r.raw | 2.3939E5 |  |  |  |  |  |  | 1 | 1 | 0 | 0 | 0 | 0 | 0 | 0 | 96 | 113 |  |  | PEAKS DB |
| total 4 peptides |  |  |  |  |  |  |  |  |  |  |  |  |  |  |  |  |  |  |  |  |  |  |  |  |  |  |  |  |  |  |  |
| --- | --- | --- | --- | --- | --- | --- | --- | --- | --- | --- | --- | --- | --- | --- | --- | --- | --- | --- | --- | --- | --- | --- | --- | --- | --- | --- | --- | --- | --- | --- | --- |

P63260|ACTG\_MOUSE

back to list

  

| Protein Coverage
| Supporting Peptides
|

Protein Coverage:

Supporting Peptides:

| Peptide | Uniq | -10lgP | Mass | Length | ppm | m/z | z | RT | Fraction | Scan | Source File | Area F1 | Area F2 | Area F3 | Area F4 | Area F5 | Area F6 | Area F7 | #Feature | #Feature F1 | #Feature F2 | #Feature F3 | #Feature F4 | #Feature F5 | #Feature F6 | #Feature F7 | Start | End | PTM | AScore | Found By |
| --- | --- | --- | --- | --- | --- | --- | --- | --- | --- | --- | --- | --- | --- | --- | --- | --- | --- | --- | --- | --- | --- | --- | --- | --- | --- | --- | --- | --- | --- | --- | --- |
| K.DLYANTVLSGGTTMYPGIADR.M | Y | 83.63 | 2214.0627 | 21 | 0.8 | 1108.0396 | 2 | 73.94 | 1 | F1:22276 | Fraction\_1\_23092022r.raw | 1.3622E5 |  |  |  |  |  |  | 1 | 1 | 0 | 0 | 0 | 0 | 0 | 0 | 292 | 312 |  |  | PEAKS DB |
| K.SYELPDGQVITIGNER.F | N | 80.24 | 1789.8846 | 16 | 0.5 | 895.9501 | 2 | 65.25 | 3 | F3:18910 | Fraction\_3\_23092022.raw | 3.1717E5 | 6.818E4 | 5.522E5 |  |  | 1.4708E5 |  | 4 | 1 | 1 | 1 | 0 | 0 | 1 | 0 | 239 | 254 |  |  | PEAKS DB |
| R.AVFPSIVGRPR.H | N | 61.14 | 1197.6981 | 11 | -0.2 | 400.2399 | 3 | 45.42 | 3 | F3:10004 | Fraction\_3\_23092022.raw |  |  | 5.4273E5 |  |  |  |  | 1 | 0 | 0 | 1 | 0 | 0 | 0 | 0 | 29 | 39 |  |  | PEAKS DB |
| R.VAPEEHPVLLTEAPLNPK.A | Y | 59.19 | 1953.0570 | 18 | -0.4 | 652.0260 | 3 | 52.34 | 1 | F1:12620 | Fraction\_1\_23092022r.raw | 2.3939E5 |  |  |  |  |  |  | 1 | 1 | 0 | 0 | 0 | 0 | 0 | 0 | 96 | 113 |  |  | PEAKS DB |
| total 4 peptides |  |  |  |  |  |  |  |  |  |  |  |  |  |  |  |  |  |  |  |  |  |  |  |  |  |  |  |  |  |  |  |
| --- | --- | --- | --- | --- | --- | --- | --- | --- | --- | --- | --- | --- | --- | --- | --- | --- | --- | --- | --- | --- | --- | --- | --- | --- | --- | --- | --- | --- | --- | --- | --- |

P63259|ACTG\_RAT

back to list

  

| Protein Coverage
| Supporting Peptides
|

Protein Coverage:

Supporting Peptides:

| Peptide | Uniq | -10lgP | Mass | Length | ppm | m/z | z | RT | Fraction | Scan | Source File | Area F1 | Area F2 | Area F3 | Area F4 | Area F5 | Area F6 | Area F7 | #Feature | #Feature F1 | #Feature F2 | #Feature F3 | #Feature F4 | #Feature F5 | #Feature F6 | #Feature F7 | Start | End | PTM | AScore | Found By |
| --- | --- | --- | --- | --- | --- | --- | --- | --- | --- | --- | --- | --- | --- | --- | --- | --- | --- | --- | --- | --- | --- | --- | --- | --- | --- | --- | --- | --- | --- | --- | --- |
| K.DLYANTVLSGGTTMYPGIADR.M | Y | 83.63 | 2214.0627 | 21 | 0.8 | 1108.0396 | 2 | 73.94 | 1 | F1:22276 | Fraction\_1\_23092022r.raw | 1.3622E5 |  |  |  |  |  |  | 1 | 1 | 0 | 0 | 0 | 0 | 0 | 0 | 292 | 312 |  |  | PEAKS DB |
| K.SYELPDGQVITIGNER.F | N | 80.24 | 1789.8846 | 16 | 0.5 | 895.9501 | 2 | 65.25 | 3 | F3:18910 | Fraction\_3\_23092022.raw | 3.1717E5 | 6.818E4 | 5.522E5 |  |  | 1.4708E5 |  | 4 | 1 | 1 | 1 | 0 | 0 | 1 | 0 | 239 | 254 |  |  | PEAKS DB |
| R.AVFPSIVGRPR.H | N | 61.14 | 1197.6981 | 11 | -0.2 | 400.2399 | 3 | 45.42 | 3 | F3:10004 | Fraction\_3\_23092022.raw |  |  | 5.4273E5 |  |  |  |  | 1 | 0 | 0 | 1 | 0 | 0 | 0 | 0 | 29 | 39 |  |  | PEAKS DB |
| R.VAPEEHPVLLTEAPLNPK.A | Y | 59.19 | 1953.0570 | 18 | -0.4 | 652.0260 | 3 | 52.34 | 1 | F1:12620 | Fraction\_1\_23092022r.raw | 2.3939E5 |  |  |  |  |  |  | 1 | 1 | 0 | 0 | 0 | 0 | 0 | 0 | 96 | 113 |  |  | PEAKS DB |
| total 4 peptides |  |  |  |  |  |  |  |  |  |  |  |  |  |  |  |  |  |  |  |  |  |  |  |  |  |  |  |  |  |  |  |
| --- | --- | --- | --- | --- | --- | --- | --- | --- | --- | --- | --- | --- | --- | --- | --- | --- | --- | --- | --- | --- | --- | --- | --- | --- | --- | --- | --- | --- | --- | --- | --- |

A2BDB0|ACTG\_XENLA

back to list

  

| Protein Coverage
| Supporting Peptides
|

Protein Coverage:

Supporting Peptides:

| Peptide | Uniq | -10lgP | Mass | Length | ppm | m/z | z | RT | Fraction | Scan | Source File | Area F1 | Area F2 | Area F3 | Area F4 | Area F5 | Area F6 | Area F7 | #Feature | #Feature F1 | #Feature F2 | #Feature F3 | #Feature F4 | #Feature F5 | #Feature F6 | #Feature F7 | Start | End | PTM | AScore | Found By |
| --- | --- | --- | --- | --- | --- | --- | --- | --- | --- | --- | --- | --- | --- | --- | --- | --- | --- | --- | --- | --- | --- | --- | --- | --- | --- | --- | --- | --- | --- | --- | --- |
| K.DLYANTVLSGGTTMYPGIADR.M | Y | 83.63 | 2214.0627 | 21 | 0.8 | 1108.0396 | 2 | 73.94 | 1 | F1:22276 | Fraction\_1\_23092022r.raw | 1.3622E5 |  |  |  |  |  |  | 1 | 1 | 0 | 0 | 0 | 0 | 0 | 0 | 292 | 312 |  |  | PEAKS DB |
| K.SYELPDGQVITIGNER.F | N | 80.24 | 1789.8846 | 16 | 0.5 | 895.9501 | 2 | 65.25 | 3 | F3:18910 | Fraction\_3\_23092022.raw | 3.1717E5 | 6.818E4 | 5.522E5 |  |  | 1.4708E5 |  | 4 | 1 | 1 | 1 | 0 | 0 | 1 | 0 | 239 | 254 |  |  | PEAKS DB |
| R.AVFPSIVGRPR.H | N | 61.14 | 1197.6981 | 11 | -0.2 | 400.2399 | 3 | 45.42 | 3 | F3:10004 | Fraction\_3\_23092022.raw |  |  | 5.4273E5 |  |  |  |  | 1 | 0 | 0 | 1 | 0 | 0 | 0 | 0 | 29 | 39 |  |  | PEAKS DB |
| R.VAPEEHPVLLTEAPLNPK.A | Y | 59.19 | 1953.0570 | 18 | -0.4 | 652.0260 | 3 | 52.34 | 1 | F1:12620 | Fraction\_1\_23092022r.raw | 2.3939E5 |  |  |  |  |  |  | 1 | 1 | 0 | 0 | 0 | 0 | 0 | 0 | 96 | 113 |  |  | PEAKS DB |
| total 4 peptides |  |  |  |  |  |  |  |  |  |  |  |  |  |  |  |  |  |  |  |  |  |  |  |  |  |  |  |  |  |  |  |
| --- | --- | --- | --- | --- | --- | --- | --- | --- | --- | --- | --- | --- | --- | --- | --- | --- | --- | --- | --- | --- | --- | --- | --- | --- | --- | --- | --- | --- | --- | --- | --- |

Q8JJB8|ACTG\_TRISC

back to list

  

| Protein Coverage
| Supporting Peptides
|

Protein Coverage:

Supporting Peptides:

| Peptide | Uniq | -10lgP | Mass | Length | ppm | m/z | z | RT | Fraction | Scan | Source File | Area F1 | Area F2 | Area F3 | Area F4 | Area F5 | Area F6 | Area F7 | #Feature | #Feature F1 | #Feature F2 | #Feature F3 | #Feature F4 | #Feature F5 | #Feature F6 | #Feature F7 | Start | End | PTM | AScore | Found By |
| --- | --- | --- | --- | --- | --- | --- | --- | --- | --- | --- | --- | --- | --- | --- | --- | --- | --- | --- | --- | --- | --- | --- | --- | --- | --- | --- | --- | --- | --- | --- | --- |
| K.DLYANTVLSGGTTMYPGIADR.M | Y | 83.63 | 2214.0627 | 21 | 0.8 | 1108.0396 | 2 | 73.94 | 1 | F1:22276 | Fraction\_1\_23092022r.raw | 1.3622E5 |  |  |  |  |  |  | 1 | 1 | 0 | 0 | 0 | 0 | 0 | 0 | 292 | 312 |  |  | PEAKS DB |
| K.SYELPDGQVITIGNER.F | N | 80.24 | 1789.8846 | 16 | 0.5 | 895.9501 | 2 | 65.25 | 3 | F3:18910 | Fraction\_3\_23092022.raw | 3.1717E5 | 6.818E4 | 5.522E5 |  |  | 1.4708E5 |  | 4 | 1 | 1 | 1 | 0 | 0 | 1 | 0 | 239 | 254 |  |  | PEAKS DB |
| R.AVFPSIVGRPR.H | N | 61.14 | 1197.6981 | 11 | -0.2 | 400.2399 | 3 | 45.42 | 3 | F3:10004 | Fraction\_3\_23092022.raw |  |  | 5.4273E5 |  |  |  |  | 1 | 0 | 0 | 1 | 0 | 0 | 0 | 0 | 29 | 39 |  |  | PEAKS DB |
| R.VAPEEHPVLLTEAPLNPK.A | Y | 59.19 | 1953.0570 | 18 | -0.4 | 652.0260 | 3 | 52.34 | 1 | F1:12620 | Fraction\_1\_23092022r.raw | 2.3939E5 |  |  |  |  |  |  | 1 | 1 | 0 | 0 | 0 | 0 | 0 | 0 | 96 | 113 |  |  | PEAKS DB |
| total 4 peptides |  |  |  |  |  |  |  |  |  |  |  |  |  |  |  |  |  |  |  |  |  |  |  |  |  |  |  |  |  |  |  |
| --- | --- | --- | --- | --- | --- | --- | --- | --- | --- | --- | --- | --- | --- | --- | --- | --- | --- | --- | --- | --- | --- | --- | --- | --- | --- | --- | --- | --- | --- | --- | --- |

Q5JAK2|ACTG\_PELLE

back to list

  

| Protein Coverage
| Supporting Peptides
|

Protein Coverage:

Supporting Peptides:

| Peptide | Uniq | -10lgP | Mass | Length | ppm | m/z | z | RT | Fraction | Scan | Source File | Area F1 | Area F2 | Area F3 | Area F4 | Area F5 | Area F6 | Area F7 | #Feature | #Feature F1 | #Feature F2 | #Feature F3 | #Feature F4 | #Feature F5 | #Feature F6 | #Feature F7 | Start | End | PTM | AScore | Found By |
| --- | --- | --- | --- | --- | --- | --- | --- | --- | --- | --- | --- | --- | --- | --- | --- | --- | --- | --- | --- | --- | --- | --- | --- | --- | --- | --- | --- | --- | --- | --- | --- |
| K.DLYANTVLSGGTTMYPGIADR.M | Y | 83.63 | 2214.0627 | 21 | 0.8 | 1108.0396 | 2 | 73.94 | 1 | F1:22276 | Fraction\_1\_23092022r.raw | 1.3622E5 |  |  |  |  |  |  | 1 | 1 | 0 | 0 | 0 | 0 | 0 | 0 | 292 | 312 |  |  | PEAKS DB |
| K.SYELPDGQVITIGNER.F | N | 80.24 | 1789.8846 | 16 | 0.5 | 895.9501 | 2 | 65.25 | 3 | F3:18910 | Fraction\_3\_23092022.raw | 3.1717E5 | 6.818E4 | 5.522E5 |  |  | 1.4708E5 |  | 4 | 1 | 1 | 1 | 0 | 0 | 1 | 0 | 239 | 254 |  |  | PEAKS DB |
| R.AVFPSIVGRPR.H | N | 61.14 | 1197.6981 | 11 | -0.2 | 400.2399 | 3 | 45.42 | 3 | F3:10004 | Fraction\_3\_23092022.raw |  |  | 5.4273E5 |  |  |  |  | 1 | 0 | 0 | 1 | 0 | 0 | 0 | 0 | 29 | 39 |  |  | PEAKS DB |
| R.VAPEEHPVLLTEAPLNPK.A | Y | 59.19 | 1953.0570 | 18 | -0.4 | 652.0260 | 3 | 52.34 | 1 | F1:12620 | Fraction\_1\_23092022r.raw | 2.3939E5 |  |  |  |  |  |  | 1 | 1 | 0 | 0 | 0 | 0 | 0 | 0 | 96 | 113 |  |  | PEAKS DB |
| total 4 peptides |  |  |  |  |  |  |  |  |  |  |  |  |  |  |  |  |  |  |  |  |  |  |  |  |  |  |  |  |  |  |  |
| --- | --- | --- | --- | --- | --- | --- | --- | --- | --- | --- | --- | --- | --- | --- | --- | --- | --- | --- | --- | --- | --- | --- | --- | --- | --- | --- | --- | --- | --- | --- | --- |

P15475|ACTB\_XENBO

back to list

  

| Protein Coverage
| Supporting Peptides
|

Protein Coverage:

Supporting Peptides:

| Peptide | Uniq | -10lgP | Mass | Length | ppm | m/z | z | RT | Fraction | Scan | Source File | Area F1 | Area F2 | Area F3 | Area F4 | Area F5 | Area F6 | Area F7 | #Feature | #Feature F1 | #Feature F2 | #Feature F3 | #Feature F4 | #Feature F5 | #Feature F6 | #Feature F7 | Start | End | PTM | AScore | Found By |
| --- | --- | --- | --- | --- | --- | --- | --- | --- | --- | --- | --- | --- | --- | --- | --- | --- | --- | --- | --- | --- | --- | --- | --- | --- | --- | --- | --- | --- | --- | --- | --- |
| K.DLYANTVLSGGTTMYPGIADR.M | Y | 83.63 | 2214.0627 | 21 | 0.8 | 1108.0396 | 2 | 73.94 | 1 | F1:22276 | Fraction\_1\_23092022r.raw | 1.3622E5 |  |  |  |  |  |  | 1 | 1 | 0 | 0 | 0 | 0 | 0 | 0 | 293 | 313 |  |  | PEAKS DB |
| K.SYELPDGQVITIGNER.F | N | 80.24 | 1789.8846 | 16 | 0.5 | 895.9501 | 2 | 65.25 | 3 | F3:18910 | Fraction\_3\_23092022.raw | 3.1717E5 | 6.818E4 | 5.522E5 |  |  | 1.4708E5 |  | 4 | 1 | 1 | 1 | 0 | 0 | 1 | 0 | 240 | 255 |  |  | PEAKS DB |
| R.AVFPSIVGRPR.H | N | 61.14 | 1197.6981 | 11 | -0.2 | 400.2399 | 3 | 45.42 | 3 | F3:10004 | Fraction\_3\_23092022.raw |  |  | 5.4273E5 |  |  |  |  | 1 | 0 | 0 | 1 | 0 | 0 | 0 | 0 | 30 | 40 |  |  | PEAKS DB |
| R.VAPEEHPVLLTEAPLNPK.A | Y | 59.19 | 1953.0570 | 18 | -0.4 | 652.0260 | 3 | 52.34 | 1 | F1:12620 | Fraction\_1\_23092022r.raw | 2.3939E5 |  |  |  |  |  |  | 1 | 1 | 0 | 0 | 0 | 0 | 0 | 0 | 97 | 114 |  |  | PEAKS DB |
| total 4 peptides |  |  |  |  |  |  |  |  |  |  |  |  |  |  |  |  |  |  |  |  |  |  |  |  |  |  |  |  |  |  |  |
| --- | --- | --- | --- | --- | --- | --- | --- | --- | --- | --- | --- | --- | --- | --- | --- | --- | --- | --- | --- | --- | --- | --- | --- | --- | --- | --- | --- | --- | --- | --- | --- |

TRINITY\_DN836\_c0\_g1\_i1.p1

back to list

  

| Protein Coverage
| Supporting Peptides
|

Protein Coverage:

Supporting Peptides:

| Peptide | Uniq | -10lgP | Mass | Length | ppm | m/z | z | RT | Fraction | Scan | Source File | Area F1 | Area F2 | Area F3 | Area F4 | Area F5 | Area F6 | Area F7 | #Feature | #Feature F1 | #Feature F2 | #Feature F3 | #Feature F4 | #Feature F5 | #Feature F6 | #Feature F7 | Start | End | PTM | AScore | Found By |
| --- | --- | --- | --- | --- | --- | --- | --- | --- | --- | --- | --- | --- | --- | --- | --- | --- | --- | --- | --- | --- | --- | --- | --- | --- | --- | --- | --- | --- | --- | --- | --- |
| K.DLYANTVLSGGTTMYPGIADR.M | Y | 83.63 | 2214.0627 | 21 | 0.8 | 1108.0396 | 2 | 73.94 | 1 | F1:22276 | Fraction\_1\_23092022r.raw | 1.3622E5 |  |  |  |  |  |  | 1 | 1 | 0 | 0 | 0 | 0 | 0 | 0 | 293 | 313 |  |  | PEAKS DB |
| K.SYELPDGQVITIGNER.F | N | 80.24 | 1789.8846 | 16 | 0.5 | 895.9501 | 2 | 65.25 | 3 | F3:18910 | Fraction\_3\_23092022.raw | 3.1717E5 | 6.818E4 | 5.522E5 |  |  | 1.4708E5 |  | 4 | 1 | 1 | 1 | 0 | 0 | 1 | 0 | 240 | 255 |  |  | PEAKS DB |
| R.AVFPSIVGRPR.H | N | 61.14 | 1197.6981 | 11 | -0.2 | 400.2399 | 3 | 45.42 | 3 | F3:10004 | Fraction\_3\_23092022.raw |  |  | 5.4273E5 |  |  |  |  | 1 | 0 | 0 | 1 | 0 | 0 | 0 | 0 | 30 | 40 |  |  | PEAKS DB |
| R.VAPEEHPVLLTEAPLNPK.A | Y | 59.19 | 1953.0570 | 18 | -0.4 | 652.0260 | 3 | 52.34 | 1 | F1:12620 | Fraction\_1\_23092022r.raw | 2.3939E5 |  |  |  |  |  |  | 1 | 1 | 0 | 0 | 0 | 0 | 0 | 0 | 97 | 114 |  |  | PEAKS DB |
| total 4 peptides |  |  |  |  |  |  |  |  |  |  |  |  |  |  |  |  |  |  |  |  |  |  |  |  |  |  |  |  |  |  |  |
| --- | --- | --- | --- | --- | --- | --- | --- | --- | --- | --- | --- | --- | --- | --- | --- | --- | --- | --- | --- | --- | --- | --- | --- | --- | --- | --- | --- | --- | --- | --- | --- |

P10987|ACT1\_DROME

back to list

  

| Protein Coverage
| Supporting Peptides
|

Protein Coverage:

Supporting Peptides:

| Peptide | Uniq | -10lgP | Mass | Length | ppm | m/z | z | RT | Fraction | Scan | Source File | Area F1 | Area F2 | Area F3 | Area F4 | Area F5 | Area F6 | Area F7 | #Feature | #Feature F1 | #Feature F2 | #Feature F3 | #Feature F4 | #Feature F5 | #Feature F6 | #Feature F7 | Start | End | PTM | AScore | Found By |
| --- | --- | --- | --- | --- | --- | --- | --- | --- | --- | --- | --- | --- | --- | --- | --- | --- | --- | --- | --- | --- | --- | --- | --- | --- | --- | --- | --- | --- | --- | --- | --- |
| K.DLYANTVLSGGTTMYPGIADR.M | Y | 83.63 | 2214.0627 | 21 | 0.8 | 1108.0396 | 2 | 73.94 | 1 | F1:22276 | Fraction\_1\_23092022r.raw | 1.3622E5 |  |  |  |  |  |  | 1 | 1 | 0 | 0 | 0 | 0 | 0 | 0 | 293 | 313 |  |  | PEAKS DB |
| K.SYELPDGQVITIGNER.F | N | 80.24 | 1789.8846 | 16 | 0.5 | 895.9501 | 2 | 65.25 | 3 | F3:18910 | Fraction\_3\_23092022.raw | 3.1717E5 | 6.818E4 | 5.522E5 |  |  | 1.4708E5 |  | 4 | 1 | 1 | 1 | 0 | 0 | 1 | 0 | 240 | 255 |  |  | PEAKS DB |
| R.AVFPSIVGRPR.H | N | 61.14 | 1197.6981 | 11 | -0.2 | 400.2399 | 3 | 45.42 | 3 | F3:10004 | Fraction\_3\_23092022.raw |  |  | 5.4273E5 |  |  |  |  | 1 | 0 | 0 | 1 | 0 | 0 | 0 | 0 | 30 | 40 |  |  | PEAKS DB |
| R.VAPEEHPVLLTEAPLNPK.A | Y | 59.19 | 1953.0570 | 18 | -0.4 | 652.0260 | 3 | 52.34 | 1 | F1:12620 | Fraction\_1\_23092022r.raw | 2.3939E5 |  |  |  |  |  |  | 1 | 1 | 0 | 0 | 0 | 0 | 0 | 0 | 97 | 114 |  |  | PEAKS DB |
| total 4 peptides |  |  |  |  |  |  |  |  |  |  |  |  |  |  |  |  |  |  |  |  |  |  |  |  |  |  |  |  |  |  |  |
| --- | --- | --- | --- | --- | --- | --- | --- | --- | --- | --- | --- | --- | --- | --- | --- | --- | --- | --- | --- | --- | --- | --- | --- | --- | --- | --- | --- | --- | --- | --- | --- |

TRINITY\_DN836\_c0\_g1\_i3.p1

back to list

  

| Protein Coverage
| Supporting Peptides
|

Protein Coverage:

Supporting Peptides:

| Peptide | Uniq | -10lgP | Mass | Length | ppm | m/z | z | RT | Fraction | Scan | Source File | Area F1 | Area F2 | Area F3 | Area F4 | Area F5 | Area F6 | Area F7 | #Feature | #Feature F1 | #Feature F2 | #Feature F3 | #Feature F4 | #Feature F5 | #Feature F6 | #Feature F7 | Start | End | PTM | AScore | Found By |
| --- | --- | --- | --- | --- | --- | --- | --- | --- | --- | --- | --- | --- | --- | --- | --- | --- | --- | --- | --- | --- | --- | --- | --- | --- | --- | --- | --- | --- | --- | --- | --- |
| K.DLYANTVLSGGTTMYPGIADR.M | Y | 83.63 | 2214.0627 | 21 | 0.8 | 1108.0396 | 2 | 73.94 | 1 | F1:22276 | Fraction\_1\_23092022r.raw | 1.3622E5 |  |  |  |  |  |  | 1 | 1 | 0 | 0 | 0 | 0 | 0 | 0 | 293 | 313 |  |  | PEAKS DB |
| K.SYELPDGQVITIGNER.F | N | 80.24 | 1789.8846 | 16 | 0.5 | 895.9501 | 2 | 65.25 | 3 | F3:18910 | Fraction\_3\_23092022.raw | 3.1717E5 | 6.818E4 | 5.522E5 |  |  | 1.4708E5 |  | 4 | 1 | 1 | 1 | 0 | 0 | 1 | 0 | 240 | 255 |  |  | PEAKS DB |
| R.AVFPSIVGRPR.H | N | 61.14 | 1197.6981 | 11 | -0.2 | 400.2399 | 3 | 45.42 | 3 | F3:10004 | Fraction\_3\_23092022.raw |  |  | 5.4273E5 |  |  |  |  | 1 | 0 | 0 | 1 | 0 | 0 | 0 | 0 | 30 | 40 |  |  | PEAKS DB |
| R.VAPEEHPVLLTEAPLNPK.A | Y | 59.19 | 1953.0570 | 18 | -0.4 | 652.0260 | 3 | 52.34 | 1 | F1:12620 | Fraction\_1\_23092022r.raw | 2.3939E5 |  |  |  |  |  |  | 1 | 1 | 0 | 0 | 0 | 0 | 0 | 0 | 97 | 114 |  |  | PEAKS DB |
| total 4 peptides |  |  |  |  |  |  |  |  |  |  |  |  |  |  |  |  |  |  |  |  |  |  |  |  |  |  |  |  |  |  |  |
| --- | --- | --- | --- | --- | --- | --- | --- | --- | --- | --- | --- | --- | --- | --- | --- | --- | --- | --- | --- | --- | --- | --- | --- | --- | --- | --- | --- | --- | --- | --- | --- |

P68556|ACT1\_DIBDE

back to list

  

| Protein Coverage
| Supporting Peptides
|

Protein Coverage:

Supporting Peptides:

| Peptide | Uniq | -10lgP | Mass | Length | ppm | m/z | z | RT | Fraction | Scan | Source File | Area F1 | Area F2 | Area F3 | Area F4 | Area F5 | Area F6 | Area F7 | #Feature | #Feature F1 | #Feature F2 | #Feature F3 | #Feature F4 | #Feature F5 | #Feature F6 | #Feature F7 | Start | End | PTM | AScore | Found By |
| --- | --- | --- | --- | --- | --- | --- | --- | --- | --- | --- | --- | --- | --- | --- | --- | --- | --- | --- | --- | --- | --- | --- | --- | --- | --- | --- | --- | --- | --- | --- | --- |
| K.DLYANTVLSGGTTMYPGIADR.M | Y | 83.63 | 2214.0627 | 21 | 0.8 | 1108.0396 | 2 | 73.94 | 1 | F1:22276 | Fraction\_1\_23092022r.raw | 1.3622E5 |  |  |  |  |  |  | 1 | 1 | 0 | 0 | 0 | 0 | 0 | 0 | 293 | 313 |  |  | PEAKS DB |
| K.SYELPDGQVITIGNER.F | N | 80.24 | 1789.8846 | 16 | 0.5 | 895.9501 | 2 | 65.25 | 3 | F3:18910 | Fraction\_3\_23092022.raw | 3.1717E5 | 6.818E4 | 5.522E5 |  |  | 1.4708E5 |  | 4 | 1 | 1 | 1 | 0 | 0 | 1 | 0 | 240 | 255 |  |  | PEAKS DB |
| R.AVFPSIVGRPR.H | N | 61.14 | 1197.6981 | 11 | -0.2 | 400.2399 | 3 | 45.42 | 3 | F3:10004 | Fraction\_3\_23092022.raw |  |  | 5.4273E5 |  |  |  |  | 1 | 0 | 0 | 1 | 0 | 0 | 0 | 0 | 30 | 40 |  |  | PEAKS DB |
| R.VAPEEHPVLLTEAPLNPK.A | Y | 59.19 | 1953.0570 | 18 | -0.4 | 652.0260 | 3 | 52.34 | 1 | F1:12620 | Fraction\_1\_23092022r.raw | 2.3939E5 |  |  |  |  |  |  | 1 | 1 | 0 | 0 | 0 | 0 | 0 | 0 | 97 | 114 |  |  | PEAKS DB |
| total 4 peptides |  |  |  |  |  |  |  |  |  |  |  |  |  |  |  |  |  |  |  |  |  |  |  |  |  |  |  |  |  |  |  |
| --- | --- | --- | --- | --- | --- | --- | --- | --- | --- | --- | --- | --- | --- | --- | --- | --- | --- | --- | --- | --- | --- | --- | --- | --- | --- | --- | --- | --- | --- | --- | --- |

TRINITY\_DN836\_c0\_g1\_i2.p1

back to list

  

| Protein Coverage
| Supporting Peptides
|

Protein Coverage:

Supporting Peptides:

| Peptide | Uniq | -10lgP | Mass | Length | ppm | m/z | z | RT | Fraction | Scan | Source File | Area F1 | Area F2 | Area F3 | Area F4 | Area F5 | Area F6 | Area F7 | #Feature | #Feature F1 | #Feature F2 | #Feature F3 | #Feature F4 | #Feature F5 | #Feature F6 | #Feature F7 | Start | End | PTM | AScore | Found By |
| --- | --- | --- | --- | --- | --- | --- | --- | --- | --- | --- | --- | --- | --- | --- | --- | --- | --- | --- | --- | --- | --- | --- | --- | --- | --- | --- | --- | --- | --- | --- | --- |
| K.DLYANTVLSGGTTMYPGIADR.M | Y | 83.63 | 2214.0627 | 21 | 0.8 | 1108.0396 | 2 | 73.94 | 1 | F1:22276 | Fraction\_1\_23092022r.raw | 1.3622E5 |  |  |  |  |  |  | 1 | 1 | 0 | 0 | 0 | 0 | 0 | 0 | 293 | 313 |  |  | PEAKS DB |
| K.SYELPDGQVITIGNER.F | N | 80.24 | 1789.8846 | 16 | 0.5 | 895.9501 | 2 | 65.25 | 3 | F3:18910 | Fraction\_3\_23092022.raw | 3.1717E5 | 6.818E4 | 5.522E5 |  |  | 1.4708E5 |  | 4 | 1 | 1 | 1 | 0 | 0 | 1 | 0 | 240 | 255 |  |  | PEAKS DB |
| R.AVFPSIVGRPR.H | N | 61.14 | 1197.6981 | 11 | -0.2 | 400.2399 | 3 | 45.42 | 3 | F3:10004 | Fraction\_3\_23092022.raw |  |  | 5.4273E5 |  |  |  |  | 1 | 0 | 0 | 1 | 0 | 0 | 0 | 0 | 30 | 40 |  |  | PEAKS DB |
| R.VAPEEHPVLLTEAPLNPK.A | Y | 59.19 | 1953.0570 | 18 | -0.4 | 652.0260 | 3 | 52.34 | 1 | F1:12620 | Fraction\_1\_23092022r.raw | 2.3939E5 |  |  |  |  |  |  | 1 | 1 | 0 | 0 | 0 | 0 | 0 | 0 | 97 | 114 |  |  | PEAKS DB |
| total 4 peptides |  |  |  |  |  |  |  |  |  |  |  |  |  |  |  |  |  |  |  |  |  |  |  |  |  |  |  |  |  |  |  |
| --- | --- | --- | --- | --- | --- | --- | --- | --- | --- | --- | --- | --- | --- | --- | --- | --- | --- | --- | --- | --- | --- | --- | --- | --- | --- | --- | --- | --- | --- | --- | --- |

P04829|ACT3\_BOMMO

back to list

  

| Protein Coverage
| Supporting Peptides
|

Protein Coverage:

Supporting Peptides:

| Peptide | Uniq | -10lgP | Mass | Length | ppm | m/z | z | RT | Fraction | Scan | Source File | Area F1 | Area F2 | Area F3 | Area F4 | Area F5 | Area F6 | Area F7 | #Feature | #Feature F1 | #Feature F2 | #Feature F3 | #Feature F4 | #Feature F5 | #Feature F6 | #Feature F7 | Start | End | PTM | AScore | Found By |
| --- | --- | --- | --- | --- | --- | --- | --- | --- | --- | --- | --- | --- | --- | --- | --- | --- | --- | --- | --- | --- | --- | --- | --- | --- | --- | --- | --- | --- | --- | --- | --- |
| K.DLYANTVLSGGTTMYPGIADR.M | Y | 83.63 | 2214.0627 | 21 | 0.8 | 1108.0396 | 2 | 73.94 | 1 | F1:22276 | Fraction\_1\_23092022r.raw | 1.3622E5 |  |  |  |  |  |  | 1 | 1 | 0 | 0 | 0 | 0 | 0 | 0 | 293 | 313 |  |  | PEAKS DB |
| K.SYELPDGQVITIGNER.F | N | 80.24 | 1789.8846 | 16 | 0.5 | 895.9501 | 2 | 65.25 | 3 | F3:18910 | Fraction\_3\_23092022.raw | 3.1717E5 | 6.818E4 | 5.522E5 |  |  | 1.4708E5 |  | 4 | 1 | 1 | 1 | 0 | 0 | 1 | 0 | 240 | 255 |  |  | PEAKS DB |
| R.AVFPSIVGRPR.H | N | 61.14 | 1197.6981 | 11 | -0.2 | 400.2399 | 3 | 45.42 | 3 | F3:10004 | Fraction\_3\_23092022.raw |  |  | 5.4273E5 |  |  |  |  | 1 | 0 | 0 | 1 | 0 | 0 | 0 | 0 | 30 | 40 |  |  | PEAKS DB |
| R.VAPEEHPVLLTEAPLNPK.A | Y | 59.19 | 1953.0570 | 18 | -0.4 | 652.0260 | 3 | 52.34 | 1 | F1:12620 | Fraction\_1\_23092022r.raw | 2.3939E5 |  |  |  |  |  |  | 1 | 1 | 0 | 0 | 0 | 0 | 0 | 0 | 97 | 114 |  |  | PEAKS DB |
| total 4 peptides |  |  |  |  |  |  |  |  |  |  |  |  |  |  |  |  |  |  |  |  |  |  |  |  |  |  |  |  |  |  |  |
| --- | --- | --- | --- | --- | --- | --- | --- | --- | --- | --- | --- | --- | --- | --- | --- | --- | --- | --- | --- | --- | --- | --- | --- | --- | --- | --- | --- | --- | --- | --- | --- |

P86700|ACT\_CHIOP

back to list

  

| Protein Coverage
| Supporting Peptides
|

Protein Coverage:

Supporting Peptides:

| Peptide | Uniq | -10lgP | Mass | Length | ppm | m/z | z | RT | Fraction | Scan | Source File | Area F1 | Area F2 | Area F3 | Area F4 | Area F5 | Area F6 | Area F7 | #Feature | #Feature F1 | #Feature F2 | #Feature F3 | #Feature F4 | #Feature F5 | #Feature F6 | #Feature F7 | Start | End | PTM | AScore | Found By |
| --- | --- | --- | --- | --- | --- | --- | --- | --- | --- | --- | --- | --- | --- | --- | --- | --- | --- | --- | --- | --- | --- | --- | --- | --- | --- | --- | --- | --- | --- | --- | --- |
| K.IAPEESPVLLTEAPLNPK.T | Y | 90.85 | 1917.0458 | 18 | 1.2 | 959.5313 | 2 | 66.09 | 3 | F3:19284 | Fraction\_3\_23092022.raw |  |  | 6.6192E5 |  |  |  |  | 1 | 0 | 0 | 1 | 0 | 0 | 0 | 0 | 40 | 57 |  |  | PEAKS DB |
| R.SYELPDGQVITIGNER.C | N | 80.24 | 1789.8846 | 16 | 0.5 | 895.9501 | 2 | 65.25 | 3 | F3:18910 | Fraction\_3\_23092022.raw | 3.1717E5 | 6.818E4 | 5.522E5 |  |  | 1.4708E5 |  | 4 | 1 | 1 | 1 | 0 | 0 | 1 | 0 | 115 | 130 |  |  | PEAKS DB |
| R.AVFPSIVGRPR.D | N | 61.14 | 1197.6981 | 11 | -0.2 | 400.2399 | 3 | 45.42 | 3 | F3:10004 | Fraction\_3\_23092022.raw |  |  | 5.4273E5 |  |  |  |  | 1 | 0 | 0 | 1 | 0 | 0 | 0 | 0 | 11 | 21 |  |  | PEAKS DB |
| total 3 peptides |  |  |  |  |  |  |  |  |  |  |  |  |  |  |  |  |  |  |  |  |  |  |  |  |  |  |  |  |  |  |  |
| --- | --- | --- | --- | --- | --- | --- | --- | --- | --- | --- | --- | --- | --- | --- | --- | --- | --- | --- | --- | --- | --- | --- | --- | --- | --- | --- | --- | --- | --- | --- | --- |

TRINITY\_DN4312\_c0\_g1\_i15.p1

back to list

  

| Protein Coverage
| Supporting Peptides
|

Protein Coverage:

Supporting Peptides:

| Peptide | Uniq | -10lgP | Mass | Length | ppm | m/z | z | RT | Fraction | Scan | Source File | Area F1 | Area F2 | Area F3 | Area F4 | Area F5 | Area F6 | Area F7 | #Feature | #Feature F1 | #Feature F2 | #Feature F3 | #Feature F4 | #Feature F5 | #Feature F6 | #Feature F7 | Start | End | PTM | AScore | Found By |
| --- | --- | --- | --- | --- | --- | --- | --- | --- | --- | --- | --- | --- | --- | --- | --- | --- | --- | --- | --- | --- | --- | --- | --- | --- | --- | --- | --- | --- | --- | --- | --- |
| K.SPLVTDISFK.L | Y | 70.92 | 1105.6019 | 10 | 0.6 | 553.8086 | 2 | 57.58 | 4 | F4:16046 | Fraction\_4\_23092022.raw | 1.2152E5 |  | 2.0747E5 | 7.675E5 |  | 5.2151E4 |  | 4 | 1 | 0 | 1 | 1 | 0 | 1 | 0 | 67 | 76 |  |  | PEAKS DB |
| R.VAGILC(+57.02)FSR.L | Y | 67.10 | 1021.5378 | 9 | 0.8 | 511.7766 | 2 | 56.47 | 4 | F4:15555 | Fraction\_4\_23092022.raw |  |  |  | 1.0966E5 | 1.0968E5 |  |  | 2 | 0 | 0 | 0 | 1 | 1 | 0 | 0 | 241 | 249 | Carbamidomethylation | C6:Carbamidomethylation:1000.00 | PEAKS DB |
| K.NYVTITEDEEDKLKK.S | Y | 66.02 | 1823.9153 | 15 | 0.9 | 608.9796 | 3 | 38.45 | 5 | F5:7243 | Fraction\_5\_23092022.raw |  |  | 7.7648E5 | 7.3361E5 | 6.3942E5 | 8.9076E5 |  | 4 | 0 | 0 | 1 | 1 | 1 | 1 | 0 | 700 | 714 |  |  | PEAKS DB |
| K.EFLNLGVLTYAK.N | Y | 58.80 | 1366.7496 | 12 | 0.0 | 684.3821 | 2 | 77.49 | 4 | F4:24958 | Fraction\_4\_23092022.raw |  |  | 3.6571E5 | 1.0821E5 |  | 3.7818E5 |  | 3 | 0 | 0 | 1 | 1 | 0 | 1 | 0 | 688 | 699 |  |  | PEAKS DB |
| K.NYVTITEDEEDKLK.K | Y | 50.70 | 1695.8203 | 14 | 0.8 | 566.2812 | 3 | 44.11 | 6 | F6:10402 | Fraction\_6\_23092022.raw |  |  |  |  |  | 9.581E4 |  | 1 | 0 | 0 | 0 | 0 | 0 | 1 | 0 | 700 | 713 |  |  | PEAKS DB |
| total 5 peptides |  |  |  |  |  |  |  |  |  |  |  |  |  |  |  |  |  |  |  |  |  |  |  |  |  |  |  |  |  |  |  |
| --- | --- | --- | --- | --- | --- | --- | --- | --- | --- | --- | --- | --- | --- | --- | --- | --- | --- | --- | --- | --- | --- | --- | --- | --- | --- | --- | --- | --- | --- | --- | --- |

TRINITY\_DN4312\_c0\_g1\_i7.p1

back to list

  

| Protein Coverage
| Supporting Peptides
|

Protein Coverage:

Supporting Peptides:

| Peptide | Uniq | -10lgP | Mass | Length | ppm | m/z | z | RT | Fraction | Scan | Source File | Area F1 | Area F2 | Area F3 | Area F4 | Area F5 | Area F6 | Area F7 | #Feature | #Feature F1 | #Feature F2 | #Feature F3 | #Feature F4 | #Feature F5 | #Feature F6 | #Feature F7 | Start | End | PTM | AScore | Found By |
| --- | --- | --- | --- | --- | --- | --- | --- | --- | --- | --- | --- | --- | --- | --- | --- | --- | --- | --- | --- | --- | --- | --- | --- | --- | --- | --- | --- | --- | --- | --- | --- |
| K.SPLVTDISFK.L | Y | 70.92 | 1105.6019 | 10 | 0.6 | 553.8086 | 2 | 57.58 | 4 | F4:16046 | Fraction\_4\_23092022.raw | 1.2152E5 |  | 2.0747E5 | 7.675E5 |  | 5.2151E4 |  | 4 | 1 | 0 | 1 | 1 | 0 | 1 | 0 | 67 | 76 |  |  | PEAKS DB |
| R.VAGILC(+57.02)FSR.L | Y | 67.10 | 1021.5378 | 9 | 0.8 | 511.7766 | 2 | 56.47 | 4 | F4:15555 | Fraction\_4\_23092022.raw |  |  |  | 1.0966E5 | 1.0968E5 |  |  | 2 | 0 | 0 | 0 | 1 | 1 | 0 | 0 | 241 | 249 | Carbamidomethylation | C6:Carbamidomethylation:1000.00 | PEAKS DB |
| K.NYVTITEDEEDKLKK.S | Y | 66.02 | 1823.9153 | 15 | 0.9 | 608.9796 | 3 | 38.45 | 5 | F5:7243 | Fraction\_5\_23092022.raw |  |  | 7.7648E5 | 7.3361E5 | 6.3942E5 | 8.9076E5 |  | 4 | 0 | 0 | 1 | 1 | 1 | 1 | 0 | 700 | 714 |  |  | PEAKS DB |
| K.EFLNLGVLTYAK.N | Y | 58.80 | 1366.7496 | 12 | 0.0 | 684.3821 | 2 | 77.49 | 4 | F4:24958 | Fraction\_4\_23092022.raw |  |  | 3.6571E5 | 1.0821E5 |  | 3.7818E5 |  | 3 | 0 | 0 | 1 | 1 | 0 | 1 | 0 | 688 | 699 |  |  | PEAKS DB |
| K.NYVTITEDEEDKLK.K | Y | 50.70 | 1695.8203 | 14 | 0.8 | 566.2812 | 3 | 44.11 | 6 | F6:10402 | Fraction\_6\_23092022.raw |  |  |  |  |  | 9.581E4 |  | 1 | 0 | 0 | 0 | 0 | 0 | 1 | 0 | 700 | 713 |  |  | PEAKS DB |
| total 5 peptides |  |  |  |  |  |  |  |  |  |  |  |  |  |  |  |  |  |  |  |  |  |  |  |  |  |  |  |  |  |  |  |
| --- | --- | --- | --- | --- | --- | --- | --- | --- | --- | --- | --- | --- | --- | --- | --- | --- | --- | --- | --- | --- | --- | --- | --- | --- | --- | --- | --- | --- | --- | --- | --- |

TRINITY\_DN4312\_c0\_g1\_i9.p1

back to list

  

| Protein Coverage
| Supporting Peptides
|

Protein Coverage:

Supporting Peptides:

| Peptide | Uniq | -10lgP | Mass | Length | ppm | m/z | z | RT | Fraction | Scan | Source File | Area F1 | Area F2 | Area F3 | Area F4 | Area F5 | Area F6 | Area F7 | #Feature | #Feature F1 | #Feature F2 | #Feature F3 | #Feature F4 | #Feature F5 | #Feature F6 | #Feature F7 | Start | End | PTM | AScore | Found By |
| --- | --- | --- | --- | --- | --- | --- | --- | --- | --- | --- | --- | --- | --- | --- | --- | --- | --- | --- | --- | --- | --- | --- | --- | --- | --- | --- | --- | --- | --- | --- | --- |
| K.SPLVTDISFK.L | Y | 70.92 | 1105.6019 | 10 | 0.6 | 553.8086 | 2 | 57.58 | 4 | F4:16046 | Fraction\_4\_23092022.raw | 1.2152E5 |  | 2.0747E5 | 7.675E5 |  | 5.2151E4 |  | 4 | 1 | 0 | 1 | 1 | 0 | 1 | 0 | 67 | 76 |  |  | PEAKS DB |
| R.VAGILC(+57.02)FSR.L | Y | 67.10 | 1021.5378 | 9 | 0.8 | 511.7766 | 2 | 56.47 | 4 | F4:15555 | Fraction\_4\_23092022.raw |  |  |  | 1.0966E5 | 1.0968E5 |  |  | 2 | 0 | 0 | 0 | 1 | 1 | 0 | 0 | 241 | 249 | Carbamidomethylation | C6:Carbamidomethylation:1000.00 | PEAKS DB |
| K.NYVTITEDEEDKLKK.S | Y | 66.02 | 1823.9153 | 15 | 0.9 | 608.9796 | 3 | 38.45 | 5 | F5:7243 | Fraction\_5\_23092022.raw |  |  | 7.7648E5 | 7.3361E5 | 6.3942E5 | 8.9076E5 |  | 4 | 0 | 0 | 1 | 1 | 1 | 1 | 0 | 700 | 714 |  |  | PEAKS DB |
| K.EFLNLGVLTYAK.N | Y | 58.80 | 1366.7496 | 12 | 0.0 | 684.3821 | 2 | 77.49 | 4 | F4:24958 | Fraction\_4\_23092022.raw |  |  | 3.6571E5 | 1.0821E5 |  | 3.7818E5 |  | 3 | 0 | 0 | 1 | 1 | 0 | 1 | 0 | 688 | 699 |  |  | PEAKS DB |
| K.NYVTITEDEEDKLK.K | Y | 50.70 | 1695.8203 | 14 | 0.8 | 566.2812 | 3 | 44.11 | 6 | F6:10402 | Fraction\_6\_23092022.raw |  |  |  |  |  | 9.581E4 |  | 1 | 0 | 0 | 0 | 0 | 0 | 1 | 0 | 700 | 713 |  |  | PEAKS DB |
| total 5 peptides |  |  |  |  |  |  |  |  |  |  |  |  |  |  |  |  |  |  |  |  |  |  |  |  |  |  |  |  |  |  |  |
| --- | --- | --- | --- | --- | --- | --- | --- | --- | --- | --- | --- | --- | --- | --- | --- | --- | --- | --- | --- | --- | --- | --- | --- | --- | --- | --- | --- | --- | --- | --- | --- |

Q6IFU8|K1C17\_RAT

back to list

  

| Protein Coverage
| Supporting Peptides
|

Protein Coverage:

Supporting Peptides:

| Peptide | Uniq | -10lgP | Mass | Length | ppm | m/z | z | RT | Fraction | Scan | Source File | Area F1 | Area F2 | Area F3 | Area F4 | Area F5 | Area F6 | Area F7 | #Feature | #Feature F1 | #Feature F2 | #Feature F3 | #Feature F4 | #Feature F5 | #Feature F6 | #Feature F7 | Start | End | PTM | AScore | Found By |
| --- | --- | --- | --- | --- | --- | --- | --- | --- | --- | --- | --- | --- | --- | --- | --- | --- | --- | --- | --- | --- | --- | --- | --- | --- | --- | --- | --- | --- | --- | --- | --- |
| R.TIVEEVQDGKVISSREQVHQTTR | Y | 68.04 | 2638.3674 | 23 | -0.7 | 660.5987 | 4 | 34.97 | 7 | F7:8489 | Fraction\_7\_23092022.raw |  |  |  |  |  |  | 1.3888E5 | 1 | 0 | 0 | 0 | 0 | 0 | 0 | 1 | 411 | 433 |  |  | PEAKS DB |
| R.EVATNSELVQSGKSEISELR.R | N | 63.14 | 2175.1018 | 20 | 0.4 | 726.0415 | 3 | 49.48 | 1 | F1:11348 | Fraction\_1\_23092022r.raw | 1.8745E5 |  |  |  |  |  |  | 1 | 1 | 0 | 0 | 0 | 0 | 0 | 0 | 285 | 304 |  |  | PEAKS DB |
| R.MSVEADINGLRR.V | N | 50.41 | 1359.6929 | 12 | -0.4 | 454.2381 | 3 | 43.39 | 1 | F1:8670 | Fraction\_1\_23092022r.raw | 7.1864E4 |  |  |  |  |  |  | 1 | 1 | 0 | 0 | 0 | 0 | 0 | 0 | 181 | 192 |  |  | PEAKS DB |
| total 3 peptides |  |  |  |  |  |  |  |  |  |  |  |  |  |  |  |  |  |  |  |  |  |  |  |  |  |  |  |  |  |  |  |
| --- | --- | --- | --- | --- | --- | --- | --- | --- | --- | --- | --- | --- | --- | --- | --- | --- | --- | --- | --- | --- | --- | --- | --- | --- | --- | --- | --- | --- | --- | --- | --- |

Q9QWL7|K1C17\_MOUSE

back to list

  

| Protein Coverage
| Supporting Peptides
|

Protein Coverage:

Supporting Peptides:

| Peptide | Uniq | -10lgP | Mass | Length | ppm | m/z | z | RT | Fraction | Scan | Source File | Area F1 | Area F2 | Area F3 | Area F4 | Area F5 | Area F6 | Area F7 | #Feature | #Feature F1 | #Feature F2 | #Feature F3 | #Feature F4 | #Feature F5 | #Feature F6 | #Feature F7 | Start | End | PTM | AScore | Found By |
| --- | --- | --- | --- | --- | --- | --- | --- | --- | --- | --- | --- | --- | --- | --- | --- | --- | --- | --- | --- | --- | --- | --- | --- | --- | --- | --- | --- | --- | --- | --- | --- |
| R.TIVEEVQDGKVISSREQVHQTTR | Y | 68.04 | 2638.3674 | 23 | -0.7 | 660.5987 | 4 | 34.97 | 7 | F7:8489 | Fraction\_7\_23092022.raw |  |  |  |  |  |  | 1.3888E5 | 1 | 0 | 0 | 0 | 0 | 0 | 0 | 1 | 411 | 433 |  |  | PEAKS DB |
| R.EVATNSELVQSGKSEISELR.R | N | 63.14 | 2175.1018 | 20 | 0.4 | 726.0415 | 3 | 49.48 | 1 | F1:11348 | Fraction\_1\_23092022r.raw | 1.8745E5 |  |  |  |  |  |  | 1 | 1 | 0 | 0 | 0 | 0 | 0 | 0 | 285 | 304 |  |  | PEAKS DB |
| R.MSVEADINGLRR.V | N | 50.41 | 1359.6929 | 12 | -0.4 | 454.2381 | 3 | 43.39 | 1 | F1:8670 | Fraction\_1\_23092022r.raw | 7.1864E4 |  |  |  |  |  |  | 1 | 1 | 0 | 0 | 0 | 0 | 0 | 0 | 181 | 192 |  |  | PEAKS DB |
| total 3 peptides |  |  |  |  |  |  |  |  |  |  |  |  |  |  |  |  |  |  |  |  |  |  |  |  |  |  |  |  |  |  |  |
| --- | --- | --- | --- | --- | --- | --- | --- | --- | --- | --- | --- | --- | --- | --- | --- | --- | --- | --- | --- | --- | --- | --- | --- | --- | --- | --- | --- | --- | --- | --- | --- |

Q8SPJ1|PLAK\_BOVIN

back to list

  

| Protein Coverage
| Supporting Peptides
|

Protein Coverage:

Supporting Peptides:

| Peptide | Uniq | -10lgP | Mass | Length | ppm | m/z | z | RT | Fraction | Scan | Source File | Area F1 | Area F2 | Area F3 | Area F4 | Area F5 | Area F6 | Area F7 | #Feature | #Feature F1 | #Feature F2 | #Feature F3 | #Feature F4 | #Feature F5 | #Feature F6 | #Feature F7 | Start | End | PTM | AScore | Found By |
| --- | --- | --- | --- | --- | --- | --- | --- | --- | --- | --- | --- | --- | --- | --- | --- | --- | --- | --- | --- | --- | --- | --- | --- | --- | --- | --- | --- | --- | --- | --- | --- |
| K.SAIVHLINYQDDAELATR.A | Y | 80.50 | 2028.0276 | 18 | 0.0 | 677.0165 | 3 | 56.51 | 3 | F3:15031 | Fraction\_3\_23092022.raw |  |  | 9.9975E4 |  |  | 4.1769E4 |  | 2 | 0 | 0 | 1 | 0 | 0 | 1 | 0 | 125 | 142 |  |  | PEAKS DB |
| R.ALM(+15.99)GSPQLVAAVVR.T | Y | 65.31 | 1426.7966 | 14 | 0.4 | 714.4059 | 2 | 57.87 | 1 | F1:15081 | Fraction\_1\_23092022r.raw | 1.8804E5 |  |  |  |  |  |  | 1 | 1 | 0 | 0 | 0 | 0 | 0 | 0 | 178 | 191 | Oxidation (M) | M3:Oxidation (M):1000.00 | PEAKS DB |
| K.LLNDEDPVVVTK.A | Y | 49.66 | 1340.7188 | 12 | -0.1 | 671.3666 | 2 | 47.10 | 1 | F1:10288 | Fraction\_1\_23092022r.raw | 4.9891E4 |  |  |  |  |  |  | 1 | 1 | 0 | 0 | 0 | 0 | 0 | 0 | 150 | 161 |  |  | PEAKS DB |
| total 3 peptides |  |  |  |  |  |  |  |  |  |  |  |  |  |  |  |  |  |  |  |  |  |  |  |  |  |  |  |  |  |  |  |
| --- | --- | --- | --- | --- | --- | --- | --- | --- | --- | --- | --- | --- | --- | --- | --- | --- | --- | --- | --- | --- | --- | --- | --- | --- | --- | --- | --- | --- | --- | --- | --- |

P14923|PLAK\_HUMAN

back to list

  

| Protein Coverage
| Supporting Peptides
|

Protein Coverage:

Supporting Peptides:

| Peptide | Uniq | -10lgP | Mass | Length | ppm | m/z | z | RT | Fraction | Scan | Source File | Area F1 | Area F2 | Area F3 | Area F4 | Area F5 | Area F6 | Area F7 | #Feature | #Feature F1 | #Feature F2 | #Feature F3 | #Feature F4 | #Feature F5 | #Feature F6 | #Feature F7 | Start | End | PTM | AScore | Found By |
| --- | --- | --- | --- | --- | --- | --- | --- | --- | --- | --- | --- | --- | --- | --- | --- | --- | --- | --- | --- | --- | --- | --- | --- | --- | --- | --- | --- | --- | --- | --- | --- |
| K.SAIVHLINYQDDAELATR.A | Y | 80.50 | 2028.0276 | 18 | 0.0 | 677.0165 | 3 | 56.51 | 3 | F3:15031 | Fraction\_3\_23092022.raw |  |  | 9.9975E4 |  |  | 4.1769E4 |  | 2 | 0 | 0 | 1 | 0 | 0 | 1 | 0 | 125 | 142 |  |  | PEAKS DB |
| R.ALM(+15.99)GSPQLVAAVVR.T | Y | 65.31 | 1426.7966 | 14 | 0.4 | 714.4059 | 2 | 57.87 | 1 | F1:15081 | Fraction\_1\_23092022r.raw | 1.8804E5 |  |  |  |  |  |  | 1 | 1 | 0 | 0 | 0 | 0 | 0 | 0 | 178 | 191 | Oxidation (M) | M3:Oxidation (M):1000.00 | PEAKS DB |
| K.LLNDEDPVVVTK.A | Y | 49.66 | 1340.7188 | 12 | -0.1 | 671.3666 | 2 | 47.10 | 1 | F1:10288 | Fraction\_1\_23092022r.raw | 4.9891E4 |  |  |  |  |  |  | 1 | 1 | 0 | 0 | 0 | 0 | 0 | 0 | 150 | 161 |  |  | PEAKS DB |
| total 3 peptides |  |  |  |  |  |  |  |  |  |  |  |  |  |  |  |  |  |  |  |  |  |  |  |  |  |  |  |  |  |  |  |
| --- | --- | --- | --- | --- | --- | --- | --- | --- | --- | --- | --- | --- | --- | --- | --- | --- | --- | --- | --- | --- | --- | --- | --- | --- | --- | --- | --- | --- | --- | --- | --- |

Q8WNW3|PLAK\_PIG

back to list

  

| Protein Coverage
| Supporting Peptides
|

Protein Coverage:

Supporting Peptides:

| Peptide | Uniq | -10lgP | Mass | Length | ppm | m/z | z | RT | Fraction | Scan | Source File | Area F1 | Area F2 | Area F3 | Area F4 | Area F5 | Area F6 | Area F7 | #Feature | #Feature F1 | #Feature F2 | #Feature F3 | #Feature F4 | #Feature F5 | #Feature F6 | #Feature F7 | Start | End | PTM | AScore | Found By |
| --- | --- | --- | --- | --- | --- | --- | --- | --- | --- | --- | --- | --- | --- | --- | --- | --- | --- | --- | --- | --- | --- | --- | --- | --- | --- | --- | --- | --- | --- | --- | --- |
| K.SAIVHLINYQDDAELATR.A | Y | 80.50 | 2028.0276 | 18 | 0.0 | 677.0165 | 3 | 56.51 | 3 | F3:15031 | Fraction\_3\_23092022.raw |  |  | 9.9975E4 |  |  | 4.1769E4 |  | 2 | 0 | 0 | 1 | 0 | 0 | 1 | 0 | 125 | 142 |  |  | PEAKS DB |
| R.ALM(+15.99)GSPQLVAAVVR.T | Y | 65.31 | 1426.7966 | 14 | 0.4 | 714.4059 | 2 | 57.87 | 1 | F1:15081 | Fraction\_1\_23092022r.raw | 1.8804E5 |  |  |  |  |  |  | 1 | 1 | 0 | 0 | 0 | 0 | 0 | 0 | 178 | 191 | Oxidation (M) | M3:Oxidation (M):1000.00 | PEAKS DB |
| K.LLNDEDPVVVTK.A | Y | 49.66 | 1340.7188 | 12 | -0.1 | 671.3666 | 2 | 47.10 | 1 | F1:10288 | Fraction\_1\_23092022r.raw | 4.9891E4 |  |  |  |  |  |  | 1 | 1 | 0 | 0 | 0 | 0 | 0 | 0 | 150 | 161 |  |  | PEAKS DB |
| total 3 peptides |  |  |  |  |  |  |  |  |  |  |  |  |  |  |  |  |  |  |  |  |  |  |  |  |  |  |  |  |  |  |  |
| --- | --- | --- | --- | --- | --- | --- | --- | --- | --- | --- | --- | --- | --- | --- | --- | --- | --- | --- | --- | --- | --- | --- | --- | --- | --- | --- | --- | --- | --- | --- | --- |

Q02257|PLAK\_MOUSE

back to list

  

| Protein Coverage
| Supporting Peptides
|

Protein Coverage:

Supporting Peptides:

| Peptide | Uniq | -10lgP | Mass | Length | ppm | m/z | z | RT | Fraction | Scan | Source File | Area F1 | Area F2 | Area F3 | Area F4 | Area F5 | Area F6 | Area F7 | #Feature | #Feature F1 | #Feature F2 | #Feature F3 | #Feature F4 | #Feature F5 | #Feature F6 | #Feature F7 | Start | End | PTM | AScore | Found By |
| --- | --- | --- | --- | --- | --- | --- | --- | --- | --- | --- | --- | --- | --- | --- | --- | --- | --- | --- | --- | --- | --- | --- | --- | --- | --- | --- | --- | --- | --- | --- | --- |
| K.SAIVHLINYQDDAELATR.A | Y | 80.50 | 2028.0276 | 18 | 0.0 | 677.0165 | 3 | 56.51 | 3 | F3:15031 | Fraction\_3\_23092022.raw |  |  | 9.9975E4 |  |  | 4.1769E4 |  | 2 | 0 | 0 | 1 | 0 | 0 | 1 | 0 | 125 | 142 |  |  | PEAKS DB |
| R.ALM(+15.99)GSPQLVAAVVR.T | Y | 65.31 | 1426.7966 | 14 | 0.4 | 714.4059 | 2 | 57.87 | 1 | F1:15081 | Fraction\_1\_23092022r.raw | 1.8804E5 |  |  |  |  |  |  | 1 | 1 | 0 | 0 | 0 | 0 | 0 | 0 | 178 | 191 | Oxidation (M) | M3:Oxidation (M):1000.00 | PEAKS DB |
| K.LLNDEDPVVVTK.A | Y | 49.66 | 1340.7188 | 12 | -0.1 | 671.3666 | 2 | 47.10 | 1 | F1:10288 | Fraction\_1\_23092022r.raw | 4.9891E4 |  |  |  |  |  |  | 1 | 1 | 0 | 0 | 0 | 0 | 0 | 0 | 150 | 161 |  |  | PEAKS DB |
| total 3 peptides |  |  |  |  |  |  |  |  |  |  |  |  |  |  |  |  |  |  |  |  |  |  |  |  |  |  |  |  |  |  |  |
| --- | --- | --- | --- | --- | --- | --- | --- | --- | --- | --- | --- | --- | --- | --- | --- | --- | --- | --- | --- | --- | --- | --- | --- | --- | --- | --- | --- | --- | --- | --- | --- |

TRINITY\_DN16204\_c0\_g1\_i2.p1

back to list

  

| Protein Coverage
| Supporting Peptides
|

Protein Coverage:

Supporting Peptides:

| Peptide | Uniq | -10lgP | Mass | Length | ppm | m/z | z | RT | Fraction | Scan | Source File | Area F1 | Area F2 | Area F3 | Area F4 | Area F5 | Area F6 | Area F7 | #Feature | #Feature F1 | #Feature F2 | #Feature F3 | #Feature F4 | #Feature F5 | #Feature F6 | #Feature F7 | Start | End | PTM | AScore | Found By |
| --- | --- | --- | --- | --- | --- | --- | --- | --- | --- | --- | --- | --- | --- | --- | --- | --- | --- | --- | --- | --- | --- | --- | --- | --- | --- | --- | --- | --- | --- | --- | --- |
| R.ALVIHAKPDDFGR.G | Y | 70.56 | 1437.7728 | 13 | -0.4 | 360.4503 | 4 | 34.70 | 6 | F6:6275 | Fraction\_6\_23092022.raw |  |  |  |  |  | 6.6635E4 |  | 1 | 0 | 0 | 0 | 0 | 0 | 1 | 0 | 183 | 195 |  |  | PEAKS DB |
| R.ALVIHAKPDDFGRGGTEESIKTGSSGAR.L | Y | 57.20 | 2855.4524 | 28 | -0.1 | 572.0977 | 5 | 32.95 | 6 | F6:5572 | Fraction\_6\_23092022.raw |  |  |  |  |  | 3.894E5 |  | 1 | 0 | 0 | 0 | 0 | 0 | 1 | 0 | 183 | 210 |  |  | PEAKS DB |
| R.ALGKDGLPIEGPK.G | Y | 53.89 | 1293.7292 | 13 | -0.5 | 432.2501 | 3 | 38.14 | 6 | F6:7740 | Fraction\_6\_23092022.raw |  |  |  |  |  | 1.0075E5 |  | 1 | 0 | 0 | 0 | 0 | 0 | 1 | 0 | 72 | 84 |  |  | PEAKS DB |
| R.EGC(+57.02)DSAGPHFNPYM(+15.99)VNHGAPNDPLR.H | Y | 48.66 | 2767.1868 | 25 | 0.0 | 692.8040 | 4 | 43.53 | 6 | F6:10139 | Fraction\_6\_23092022.raw |  |  |  |  |  | 2.0581E5 |  | 1 | 0 | 0 | 0 | 0 | 0 | 1 | 0 | 122 | 146 | Carbamidomethylation; Oxidation (M) | C3:Carbamidomethylation:1000.00;M14:Oxidation (M):1000.00 | PEAKS DB |
| total 4 peptides |  |  |  |  |  |  |  |  |  |  |  |  |  |  |  |  |  |  |  |  |  |  |  |  |  |  |  |  |  |  |  |
| --- | --- | --- | --- | --- | --- | --- | --- | --- | --- | --- | --- | --- | --- | --- | --- | --- | --- | --- | --- | --- | --- | --- | --- | --- | --- | --- | --- | --- | --- | --- | --- |

TRINITY\_DN838\_c0\_g3\_i1.p1

back to list

  

| Protein Coverage
| Supporting Peptides
|

Protein Coverage:

Supporting Peptides:

| Peptide | Uniq | -10lgP | Mass | Length | ppm | m/z | z | RT | Fraction | Scan | Source File | Area F1 | Area F2 | Area F3 | Area F4 | Area F5 | Area F6 | Area F7 | #Feature | #Feature F1 | #Feature F2 | #Feature F3 | #Feature F4 | #Feature F5 | #Feature F6 | #Feature F7 | Start | End | PTM | AScore | Found By |
| --- | --- | --- | --- | --- | --- | --- | --- | --- | --- | --- | --- | --- | --- | --- | --- | --- | --- | --- | --- | --- | --- | --- | --- | --- | --- | --- | --- | --- | --- | --- | --- |
| K.M(+15.99)VELNAGEPIC(+57.02)SIYNDRGIIQK.M | Y | 86.45 | 2535.2461 | 22 | 0.8 | 846.0900 | 3 | 60.03 | 5 | F5:16923 | Fraction\_5\_23092022.raw |  |  |  |  | 8.2902E5 | 1.4595E6 |  | 2 | 0 | 0 | 0 | 0 | 1 | 1 | 0 | 32 | 53 | Oxidation (M); Carbamidomethylation | M1:Oxidation (M):1000.00;C11:Carbamidomethylation:1000.00 | PEAKS DB |
| K.C(+57.02)IHSIC(+57.02)NYSPIIR.M | Y | 83.94 | 1631.7913 | 13 | 1.0 | 544.9382 | 3 | 45.07 | 5 | F5:10182 | Fraction\_5\_23092022.raw |  |  |  |  | 7.2723E5 | 7.0208E5 | 1.8558E6 | 3 | 0 | 0 | 0 | 0 | 1 | 1 | 1 | 135 | 147 | Carbamidomethylation | C1:Carbamidomethylation:1000.00;C6:Carbamidomethylation:1000.00 | PEAKS DB |
| total 2 peptides |  |  |  |  |  |  |  |  |  |  |  |  |  |  |  |  |  |  |  |  |  |  |  |  |  |  |  |  |  |  |  |
| --- | --- | --- | --- | --- | --- | --- | --- | --- | --- | --- | --- | --- | --- | --- | --- | --- | --- | --- | --- | --- | --- | --- | --- | --- | --- | --- | --- | --- | --- | --- | --- |

TRINITY\_DN838\_c0\_g3\_i3.p1

back to list

  

| Protein Coverage
| Supporting Peptides
|

Protein Coverage:

Supporting Peptides:

| Peptide | Uniq | -10lgP | Mass | Length | ppm | m/z | z | RT | Fraction | Scan | Source File | Area F1 | Area F2 | Area F3 | Area F4 | Area F5 | Area F6 | Area F7 | #Feature | #Feature F1 | #Feature F2 | #Feature F3 | #Feature F4 | #Feature F5 | #Feature F6 | #Feature F7 | Start | End | PTM | AScore | Found By |
| --- | --- | --- | --- | --- | --- | --- | --- | --- | --- | --- | --- | --- | --- | --- | --- | --- | --- | --- | --- | --- | --- | --- | --- | --- | --- | --- | --- | --- | --- | --- | --- |
| K.M(+15.99)VELNAGEPIC(+57.02)SIYNDRGIIQK.M | Y | 86.45 | 2535.2461 | 22 | 0.8 | 846.0900 | 3 | 60.03 | 5 | F5:16923 | Fraction\_5\_23092022.raw |  |  |  |  | 8.2902E5 | 1.4595E6 |  | 2 | 0 | 0 | 0 | 0 | 1 | 1 | 0 | 32 | 53 | Oxidation (M); Carbamidomethylation | M1:Oxidation (M):1000.00;C11:Carbamidomethylation:1000.00 | PEAKS DB |
| K.C(+57.02)IHSIC(+57.02)NYSPIIR.M | Y | 83.94 | 1631.7913 | 13 | 1.0 | 544.9382 | 3 | 45.07 | 5 | F5:10182 | Fraction\_5\_23092022.raw |  |  |  |  | 7.2723E5 | 7.0208E5 | 1.8558E6 | 3 | 0 | 0 | 0 | 0 | 1 | 1 | 1 | 135 | 147 | Carbamidomethylation | C1:Carbamidomethylation:1000.00;C6:Carbamidomethylation:1000.00 | PEAKS DB |
| total 2 peptides |  |  |  |  |  |  |  |  |  |  |  |  |  |  |  |  |  |  |  |  |  |  |  |  |  |  |  |  |  |  |  |
| --- | --- | --- | --- | --- | --- | --- | --- | --- | --- | --- | --- | --- | --- | --- | --- | --- | --- | --- | --- | --- | --- | --- | --- | --- | --- | --- | --- | --- | --- | --- | --- |

TRINITY\_DN838\_c0\_g3\_i2.p1

back to list

  

| Protein Coverage
| Supporting Peptides
|

Protein Coverage:

Supporting Peptides:

| Peptide | Uniq | -10lgP | Mass | Length | ppm | m/z | z | RT | Fraction | Scan | Source File | Area F1 | Area F2 | Area F3 | Area F4 | Area F5 | Area F6 | Area F7 | #Feature | #Feature F1 | #Feature F2 | #Feature F3 | #Feature F4 | #Feature F5 | #Feature F6 | #Feature F7 | Start | End | PTM | AScore | Found By |
| --- | --- | --- | --- | --- | --- | --- | --- | --- | --- | --- | --- | --- | --- | --- | --- | --- | --- | --- | --- | --- | --- | --- | --- | --- | --- | --- | --- | --- | --- | --- | --- |
| K.M(+15.99)VELNAGEPIC(+57.02)SIYNDRGIIQK.M | Y | 86.45 | 2535.2461 | 22 | 0.8 | 846.0900 | 3 | 60.03 | 5 | F5:16923 | Fraction\_5\_23092022.raw |  |  |  |  | 8.2902E5 | 1.4595E6 |  | 2 | 0 | 0 | 0 | 0 | 1 | 1 | 0 | 32 | 53 | Oxidation (M); Carbamidomethylation | M1:Oxidation (M):1000.00;C11:Carbamidomethylation:1000.00 | PEAKS DB |
| K.C(+57.02)IHSIC(+57.02)NYSPIIR.M | Y | 83.94 | 1631.7913 | 13 | 1.0 | 544.9382 | 3 | 45.07 | 5 | F5:10182 | Fraction\_5\_23092022.raw |  |  |  |  | 7.2723E5 | 7.0208E5 | 1.8558E6 | 3 | 0 | 0 | 0 | 0 | 1 | 1 | 1 | 135 | 147 | Carbamidomethylation | C1:Carbamidomethylation:1000.00;C6:Carbamidomethylation:1000.00 | PEAKS DB |
| total 2 peptides |  |  |  |  |  |  |  |  |  |  |  |  |  |  |  |  |  |  |  |  |  |  |  |  |  |  |  |  |  |  |  |
| --- | --- | --- | --- | --- | --- | --- | --- | --- | --- | --- | --- | --- | --- | --- | --- | --- | --- | --- | --- | --- | --- | --- | --- | --- | --- | --- | --- | --- | --- | --- | --- |

TRINITY\_DN2373\_c0\_g1\_i12.p1

back to list

  

| Protein Coverage
| Supporting Peptides
|

Protein Coverage:

Supporting Peptides:

| Peptide | Uniq | -10lgP | Mass | Length | ppm | m/z | z | RT | Fraction | Scan | Source File | Area F1 | Area F2 | Area F3 | Area F4 | Area F5 | Area F6 | Area F7 | #Feature | #Feature F1 | #Feature F2 | #Feature F3 | #Feature F4 | #Feature F5 | #Feature F6 | #Feature F7 | Start | End | PTM | AScore | Found By |
| --- | --- | --- | --- | --- | --- | --- | --- | --- | --- | --- | --- | --- | --- | --- | --- | --- | --- | --- | --- | --- | --- | --- | --- | --- | --- | --- | --- | --- | --- | --- | --- |
| R.LLKDDAFGVDEALNEM(+15.99)AYDEGLVVR.G | Y | 94.27 | 2797.3479 | 25 | 1.5 | 933.4580 | 3 | 73.18 | 3 | F3:22468 | Fraction\_3\_23092022.raw |  |  | 3.7553E5 |  |  |  |  | 1 | 0 | 0 | 1 | 0 | 0 | 0 | 0 | 12 | 36 | Oxidation (M) | M16:Oxidation (M):1000.00 | PEAKS DB |
| K.ALFTSFTITSIK.E | Y | 66.05 | 1327.7388 | 12 | 0.4 | 664.8770 | 2 | 74.03 | 6 | F6:23845 | Fraction\_6\_23092022.raw |  |  |  |  |  | 1.6124E5 |  | 1 | 0 | 0 | 0 | 0 | 0 | 1 | 0 | 141 | 152 |  |  | PEAKS DB |
| total 2 peptides |  |  |  |  |  |  |  |  |  |  |  |  |  |  |  |  |  |  |  |  |  |  |  |  |  |  |  |  |  |  |  |
| --- | --- | --- | --- | --- | --- | --- | --- | --- | --- | --- | --- | --- | --- | --- | --- | --- | --- | --- | --- | --- | --- | --- | --- | --- | --- | --- | --- | --- | --- | --- | --- |

TRINITY\_DN2373\_c0\_g1\_i1.p1

back to list

  

| Protein Coverage
| Supporting Peptides
|

Protein Coverage:

Supporting Peptides:

| Peptide | Uniq | -10lgP | Mass | Length | ppm | m/z | z | RT | Fraction | Scan | Source File | Area F1 | Area F2 | Area F3 | Area F4 | Area F5 | Area F6 | Area F7 | #Feature | #Feature F1 | #Feature F2 | #Feature F3 | #Feature F4 | #Feature F5 | #Feature F6 | #Feature F7 | Start | End | PTM | AScore | Found By |
| --- | --- | --- | --- | --- | --- | --- | --- | --- | --- | --- | --- | --- | --- | --- | --- | --- | --- | --- | --- | --- | --- | --- | --- | --- | --- | --- | --- | --- | --- | --- | --- |
| R.LLKDDAFGVDEALNEM(+15.99)AYDEGLVVR.G | Y | 94.27 | 2797.3479 | 25 | 1.5 | 933.4580 | 3 | 73.18 | 3 | F3:22468 | Fraction\_3\_23092022.raw |  |  | 3.7553E5 |  |  |  |  | 1 | 0 | 0 | 1 | 0 | 0 | 0 | 0 | 12 | 36 | Oxidation (M) | M16:Oxidation (M):1000.00 | PEAKS DB |
| K.ALFTSFTITSIK.E | Y | 66.05 | 1327.7388 | 12 | 0.4 | 664.8770 | 2 | 74.03 | 6 | F6:23845 | Fraction\_6\_23092022.raw |  |  |  |  |  | 1.6124E5 |  | 1 | 0 | 0 | 0 | 0 | 0 | 1 | 0 | 141 | 152 |  |  | PEAKS DB |
| total 2 peptides |  |  |  |  |  |  |  |  |  |  |  |  |  |  |  |  |  |  |  |  |  |  |  |  |  |  |  |  |  |  |  |
| --- | --- | --- | --- | --- | --- | --- | --- | --- | --- | --- | --- | --- | --- | --- | --- | --- | --- | --- | --- | --- | --- | --- | --- | --- | --- | --- | --- | --- | --- | --- | --- |

TRINITY\_DN2373\_c0\_g1\_i22.p1

back to list

  

| Protein Coverage
| Supporting Peptides
|

Protein Coverage:

Supporting Peptides:

| Peptide | Uniq | -10lgP | Mass | Length | ppm | m/z | z | RT | Fraction | Scan | Source File | Area F1 | Area F2 | Area F3 | Area F4 | Area F5 | Area F6 | Area F7 | #Feature | #Feature F1 | #Feature F2 | #Feature F3 | #Feature F4 | #Feature F5 | #Feature F6 | #Feature F7 | Start | End | PTM | AScore | Found By |
| --- | --- | --- | --- | --- | --- | --- | --- | --- | --- | --- | --- | --- | --- | --- | --- | --- | --- | --- | --- | --- | --- | --- | --- | --- | --- | --- | --- | --- | --- | --- | --- |
| R.LLKDDAFGVDEALNEM(+15.99)AYDEGLVVR.G | Y | 94.27 | 2797.3479 | 25 | 1.5 | 933.4580 | 3 | 73.18 | 3 | F3:22468 | Fraction\_3\_23092022.raw |  |  | 3.7553E5 |  |  |  |  | 1 | 0 | 0 | 1 | 0 | 0 | 0 | 0 | 12 | 36 | Oxidation (M) | M16:Oxidation (M):1000.00 | PEAKS DB |
| K.ALFTSFTITSIK.E | Y | 66.05 | 1327.7388 | 12 | 0.4 | 664.8770 | 2 | 74.03 | 6 | F6:23845 | Fraction\_6\_23092022.raw |  |  |  |  |  | 1.6124E5 |  | 1 | 0 | 0 | 0 | 0 | 0 | 1 | 0 | 141 | 152 |  |  | PEAKS DB |
| total 2 peptides |  |  |  |  |  |  |  |  |  |  |  |  |  |  |  |  |  |  |  |  |  |  |  |  |  |  |  |  |  |  |  |
| --- | --- | --- | --- | --- | --- | --- | --- | --- | --- | --- | --- | --- | --- | --- | --- | --- | --- | --- | --- | --- | --- | --- | --- | --- | --- | --- | --- | --- | --- | --- | --- |

TRINITY\_DN28260\_c0\_g1\_i1.p1

back to list

  

| Protein Coverage
| Supporting Peptides
|

Protein Coverage:

Supporting Peptides:

| Peptide | Uniq | -10lgP | Mass | Length | ppm | m/z | z | RT | Fraction | Scan | Source File | Area F1 | Area F2 | Area F3 | Area F4 | Area F5 | Area F6 | Area F7 | #Feature | #Feature F1 | #Feature F2 | #Feature F3 | #Feature F4 | #Feature F5 | #Feature F6 | #Feature F7 | Start | End | PTM | AScore | Found By |
| --- | --- | --- | --- | --- | --- | --- | --- | --- | --- | --- | --- | --- | --- | --- | --- | --- | --- | --- | --- | --- | --- | --- | --- | --- | --- | --- | --- | --- | --- | --- | --- |
| R.QISYITGVATSIEGIAR.S | Y | 86.40 | 1777.9574 | 17 | 0.6 | 889.9865 | 2 | 75.00 | 4 | F4:23825 | Fraction\_4\_23092022.raw |  |  | 2.0272E5 | 1.5358E6 | 3.4602E5 |  |  | 3 | 0 | 0 | 1 | 1 | 1 | 0 | 0 | 77 | 93 |  |  | PEAKS DB |
| K.VVNRQISYITGVATSIEGIAR.S | Y | 79.68 | 2246.2383 | 21 | 0.8 | 749.7540 | 3 | 71.90 | 4 | F4:22477 | Fraction\_4\_23092022.raw |  |  |  | 4.2439E4 |  |  |  | 1 | 0 | 0 | 0 | 1 | 0 | 0 | 0 | 73 | 93 |  |  | PEAKS DB |
| total 2 peptides |  |  |  |  |  |  |  |  |  |  |  |  |  |  |  |  |  |  |  |  |  |  |  |  |  |  |  |  |  |  |  |
| --- | --- | --- | --- | --- | --- | --- | --- | --- | --- | --- | --- | --- | --- | --- | --- | --- | --- | --- | --- | --- | --- | --- | --- | --- | --- | --- | --- | --- | --- | --- | --- |

TRINITY\_DN28260\_c0\_g1\_i2.p1

back to list

  

| Protein Coverage
| Supporting Peptides
|

Protein Coverage:

Supporting Peptides:

| Peptide | Uniq | -10lgP | Mass | Length | ppm | m/z | z | RT | Fraction | Scan | Source File | Area F1 | Area F2 | Area F3 | Area F4 | Area F5 | Area F6 | Area F7 | #Feature | #Feature F1 | #Feature F2 | #Feature F3 | #Feature F4 | #Feature F5 | #Feature F6 | #Feature F7 | Start | End | PTM | AScore | Found By |
| --- | --- | --- | --- | --- | --- | --- | --- | --- | --- | --- | --- | --- | --- | --- | --- | --- | --- | --- | --- | --- | --- | --- | --- | --- | --- | --- | --- | --- | --- | --- | --- |
| R.QISYITGVATSIEGIAR.S | Y | 86.40 | 1777.9574 | 17 | 0.6 | 889.9865 | 2 | 75.00 | 4 | F4:23825 | Fraction\_4\_23092022.raw |  |  | 2.0272E5 | 1.5358E6 | 3.4602E5 |  |  | 3 | 0 | 0 | 1 | 1 | 1 | 0 | 0 | 77 | 93 |  |  | PEAKS DB |
| K.VVNRQISYITGVATSIEGIAR.S | Y | 79.68 | 2246.2383 | 21 | 0.8 | 749.7540 | 3 | 71.90 | 4 | F4:22477 | Fraction\_4\_23092022.raw |  |  |  | 4.2439E4 |  |  |  | 1 | 0 | 0 | 0 | 1 | 0 | 0 | 0 | 73 | 93 |  |  | PEAKS DB |
| total 2 peptides |  |  |  |  |  |  |  |  |  |  |  |  |  |  |  |  |  |  |  |  |  |  |  |  |  |  |  |  |  |  |  |
| --- | --- | --- | --- | --- | --- | --- | --- | --- | --- | --- | --- | --- | --- | --- | --- | --- | --- | --- | --- | --- | --- | --- | --- | --- | --- | --- | --- | --- | --- | --- | --- |

TRINITY\_DN28260\_c0\_g1\_i3.p1

back to list

  

| Protein Coverage
| Supporting Peptides
|

Protein Coverage:

Supporting Peptides:

| Peptide | Uniq | -10lgP | Mass | Length | ppm | m/z | z | RT | Fraction | Scan | Source File | Area F1 | Area F2 | Area F3 | Area F4 | Area F5 | Area F6 | Area F7 | #Feature | #Feature F1 | #Feature F2 | #Feature F3 | #Feature F4 | #Feature F5 | #Feature F6 | #Feature F7 | Start | End | PTM | AScore | Found By |
| --- | --- | --- | --- | --- | --- | --- | --- | --- | --- | --- | --- | --- | --- | --- | --- | --- | --- | --- | --- | --- | --- | --- | --- | --- | --- | --- | --- | --- | --- | --- | --- |
| R.QISYITGVATSIEGIAR.S | Y | 86.40 | 1777.9574 | 17 | 0.6 | 889.9865 | 2 | 75.00 | 4 | F4:23825 | Fraction\_4\_23092022.raw |  |  | 2.0272E5 | 1.5358E6 | 3.4602E5 |  |  | 3 | 0 | 0 | 1 | 1 | 1 | 0 | 0 | 112 | 128 |  |  | PEAKS DB |
| K.VVNRQISYITGVATSIEGIAR.S | Y | 79.68 | 2246.2383 | 21 | 0.8 | 749.7540 | 3 | 71.90 | 4 | F4:22477 | Fraction\_4\_23092022.raw |  |  |  | 4.2439E4 |  |  |  | 1 | 0 | 0 | 0 | 1 | 0 | 0 | 0 | 108 | 128 |  |  | PEAKS DB |
| total 2 peptides |  |  |  |  |  |  |  |  |  |  |  |  |  |  |  |  |  |  |  |  |  |  |  |  |  |  |  |  |  |  |  |
| --- | --- | --- | --- | --- | --- | --- | --- | --- | --- | --- | --- | --- | --- | --- | --- | --- | --- | --- | --- | --- | --- | --- | --- | --- | --- | --- | --- | --- | --- | --- | --- |

TRINITY\_DN7688\_c0\_g2\_i1.p1

back to list

  

| Protein Coverage
| Supporting Peptides
|

Protein Coverage:

Supporting Peptides:

| Peptide | Uniq | -10lgP | Mass | Length | ppm | m/z | z | RT | Fraction | Scan | Source File | Area F1 | Area F2 | Area F3 | Area F4 | Area F5 | Area F6 | Area F7 | #Feature | #Feature F1 | #Feature F2 | #Feature F3 | #Feature F4 | #Feature F5 | #Feature F6 | #Feature F7 | Start | End | PTM | AScore | Found By |
| --- | --- | --- | --- | --- | --- | --- | --- | --- | --- | --- | --- | --- | --- | --- | --- | --- | --- | --- | --- | --- | --- | --- | --- | --- | --- | --- | --- | --- | --- | --- | --- |
| K.NVAAGSPAVITGWGYVSNSSPAFSQTLR.K | Y | 88.99 | 2836.4143 | 28 | 0.7 | 946.4794 | 3 | 76.41 | 5 | F5:24238 | Fraction\_5\_23092022.raw |  |  |  |  | 8.9582E4 | 1.5861E5 |  | 2 | 0 | 0 | 0 | 0 | 1 | 1 | 0 | 139 | 166 |  |  | PEAKS DB |
| K.TVRPISIIQANK.N | Y | 67.65 | 1338.7983 | 12 | -0.8 | 447.2730 | 3 | 38.73 | 6 | F6:8023 | Fraction\_6\_23092022.raw |  |  |  |  |  | 1.2631E6 | 1.822E5 | 3 | 0 | 0 | 0 | 0 | 0 | 2 | 1 | 127 | 138 |  |  | PEAKS DB |
| total 2 peptides |  |  |  |  |  |  |  |  |  |  |  |  |  |  |  |  |  |  |  |  |  |  |  |  |  |  |  |  |  |  |  |
| --- | --- | --- | --- | --- | --- | --- | --- | --- | --- | --- | --- | --- | --- | --- | --- | --- | --- | --- | --- | --- | --- | --- | --- | --- | --- | --- | --- | --- | --- | --- | --- |

TRINITY\_DN7688\_c0\_g2\_i2.p1

back to list

  

| Protein Coverage
| Supporting Peptides
|

Protein Coverage:

Supporting Peptides:

| Peptide | Uniq | -10lgP | Mass | Length | ppm | m/z | z | RT | Fraction | Scan | Source File | Area F1 | Area F2 | Area F3 | Area F4 | Area F5 | Area F6 | Area F7 | #Feature | #Feature F1 | #Feature F2 | #Feature F3 | #Feature F4 | #Feature F5 | #Feature F6 | #Feature F7 | Start | End | PTM | AScore | Found By |
| --- | --- | --- | --- | --- | --- | --- | --- | --- | --- | --- | --- | --- | --- | --- | --- | --- | --- | --- | --- | --- | --- | --- | --- | --- | --- | --- | --- | --- | --- | --- | --- |
| K.NVAAGSPAVITGWGYVSNSSPAFSQTLR.K | Y | 88.99 | 2836.4143 | 28 | 0.7 | 946.4794 | 3 | 76.41 | 5 | F5:24238 | Fraction\_5\_23092022.raw |  |  |  |  | 8.9582E4 | 1.5861E5 |  | 2 | 0 | 0 | 0 | 0 | 1 | 1 | 0 | 64 | 91 |  |  | PEAKS DB |
| K.TVRPISIIQANK.N | Y | 67.65 | 1338.7983 | 12 | -0.8 | 447.2730 | 3 | 38.73 | 6 | F6:8023 | Fraction\_6\_23092022.raw |  |  |  |  |  | 1.2631E6 | 1.822E5 | 3 | 0 | 0 | 0 | 0 | 0 | 2 | 1 | 52 | 63 |  |  | PEAKS DB |
| total 2 peptides |  |  |  |  |  |  |  |  |  |  |  |  |  |  |  |  |  |  |  |  |  |  |  |  |  |  |  |  |  |  |  |
| --- | --- | --- | --- | --- | --- | --- | --- | --- | --- | --- | --- | --- | --- | --- | --- | --- | --- | --- | --- | --- | --- | --- | --- | --- | --- | --- | --- | --- | --- | --- | --- |

P0DMB9|VA5\_VESVE

back to list

  

| Protein Coverage
| Supporting Peptides
|

Protein Coverage:

Supporting Peptides:

| Peptide | Uniq | -10lgP | Mass | Length | ppm | m/z | z | RT | Fraction | Scan | Source File | Area F1 | Area F2 | Area F3 | Area F4 | Area F5 | Area F6 | Area F7 | #Feature | #Feature F1 | #Feature F2 | #Feature F3 | #Feature F4 | #Feature F5 | #Feature F6 | #Feature F7 | Start | End | PTM | AScore | Found By |
| --- | --- | --- | --- | --- | --- | --- | --- | --- | --- | --- | --- | --- | --- | --- | --- | --- | --- | --- | --- | --- | --- | --- | --- | --- | --- | --- | --- | --- | --- | --- | --- |
| K.AEKLEILKQHNEFR.Q | Y | 62.43 | 1753.9475 | 14 | -0.3 | 439.4940 | 4 | 32.15 | 7 | F7:7226 | Fraction\_7\_23092022.raw |  |  |  |  |  |  | 1.2172E5 | 1 | 0 | 0 | 0 | 0 | 0 | 0 | 1 | 39 | 52 |  |  | PEAKS DB |
| R.GLETRGNPGPQPPAK.S | N | 57.93 | 1517.7949 | 15 | -0.4 | 506.9387 | 3 | 22.00 | 7 | F7:3354 | Fraction\_7\_23092022.raw |  |  |  |  |  |  | 7.2952E4 | 1 | 0 | 0 | 0 | 0 | 0 | 0 | 1 | 58 | 72 |  |  | PEAKS DB |
| R.GNPGPQPPAK.S | N | 52.98 | 961.4981 | 10 | 0.3 | 481.7565 | 2 | 17.36 | 7 | F7:2608 | Fraction\_7\_23092022.raw |  |  |  |  |  |  | 3.8663E4 | 1 | 0 | 0 | 0 | 0 | 0 | 0 | 1 | 63 | 72 |  |  | PEAKS DB |
| total 3 peptides |  |  |  |  |  |  |  |  |  |  |  |  |  |  |  |  |  |  |  |  |  |  |  |  |  |  |  |  |  |  |  |
| --- | --- | --- | --- | --- | --- | --- | --- | --- | --- | --- | --- | --- | --- | --- | --- | --- | --- | --- | --- | --- | --- | --- | --- | --- | --- | --- | --- | --- | --- | --- | --- |

P31151|S10A7\_HUMAN

back to list

  

| Protein Coverage
| Supporting Peptides
|

Protein Coverage:

Supporting Peptides:

| Peptide | Uniq | -10lgP | Mass | Length | ppm | m/z | z | RT | Fraction | Scan | Source File | Area F1 | Area F2 | Area F3 | Area F4 | Area F5 | Area F6 | Area F7 | #Feature | #Feature F1 | #Feature F2 | #Feature F3 | #Feature F4 | #Feature F5 | #Feature F6 | #Feature F7 | Start | End | PTM | AScore | Found By |
| --- | --- | --- | --- | --- | --- | --- | --- | --- | --- | --- | --- | --- | --- | --- | --- | --- | --- | --- | --- | --- | --- | --- | --- | --- | --- | --- | --- | --- | --- | --- | --- |
| K.GTNYLADVFEK.K | Y | 68.92 | 1255.6084 | 11 | 1.0 | 628.8121 | 2 | 64.32 | 4 | F4:19080 | Fraction\_4\_23092022.raw | 2.8908E5 | 9.93E4 | 1.1071E5 | 1.1403E5 | 4.9608E4 | 6.224E4 |  | 6 | 1 | 1 | 1 | 1 | 1 | 1 | 0 | 51 | 61 |  |  | PEAKS DB |
| K.ENFPNFLSAC(+57.02)DKK.G | Y | 60.71 | 1568.7292 | 13 | -0.6 | 523.9167 | 3 | 53.25 | 1 | F1:13031 | Fraction\_1\_23092022r.raw | 3.9291E5 |  |  |  |  |  |  | 1 | 1 | 0 | 0 | 0 | 0 | 0 | 0 | 38 | 50 | Carbamidomethylation | C10:Carbamidomethylation:1000.00 | PEAKS DB |
| K.GTNYLADVFEKK.D | Y | 53.73 | 1383.7034 | 12 | 0.2 | 692.8591 | 2 | 55.55 | 1 | F1:14033 | Fraction\_1\_23092022r.raw | 1.4307E5 |  |  |  |  |  |  | 1 | 1 | 0 | 0 | 0 | 0 | 0 | 0 | 51 | 62 |  |  | PEAKS DB |
| total 3 peptides |  |  |  |  |  |  |  |  |  |  |  |  |  |  |  |  |  |  |  |  |  |  |  |  |  |  |  |  |  |  |  |
| --- | --- | --- | --- | --- | --- | --- | --- | --- | --- | --- | --- | --- | --- | --- | --- | --- | --- | --- | --- | --- | --- | --- | --- | --- | --- | --- | --- | --- | --- | --- | --- |

Q5D7H4|HUGAB\_VESVU

back to list

  

| Protein Coverage
| Supporting Peptides
|

Protein Coverage:

Supporting Peptides:

| Peptide | Uniq | -10lgP | Mass | Length | ppm | m/z | z | RT | Fraction | Scan | Source File | Area F1 | Area F2 | Area F3 | Area F4 | Area F5 | Area F6 | Area F7 | #Feature | #Feature F1 | #Feature F2 | #Feature F3 | #Feature F4 | #Feature F5 | #Feature F6 | #Feature F7 | Start | End | PTM | AScore | Found By |
| --- | --- | --- | --- | --- | --- | --- | --- | --- | --- | --- | --- | --- | --- | --- | --- | --- | --- | --- | --- | --- | --- | --- | --- | --- | --- | --- | --- | --- | --- | --- | --- |
| R.NVDNLKINKDISIDLVR.K | Y | 70.50 | 1968.1003 | 17 | 0.3 | 657.0409 | 3 | 50.83 | 6 | F6:13384 | Fraction\_6\_23092022.raw |  |  | 9.5454E4 | 2.26E5 | 1.2458E6 | 1.9023E7 | 3.2093E5 | 12 | 0 | 0 | 1 | 1 | 2 | 7 | 1 | 120 | 136 |  |  | PEAKS DB |
| R.NVDNLKINKDISIDLVRK.E | Y | 54.72 | 2096.1953 | 18 | 0.3 | 525.0563 | 4 | 44.25 | 6 | F6:10451 | Fraction\_6\_23092022.raw |  |  |  |  |  | 8.6278E5 |  | 3 | 0 | 0 | 0 | 0 | 0 | 3 | 0 | 120 | 137 |  |  | PEAKS DB |
| K.ELDEIYPK.K | N | 50.82 | 1005.5018 | 8 | 0.3 | 503.7584 | 2 | 41.97 | 7 | F7:11626 | Fraction\_7\_23092022.raw |  |  |  | 1.6356E6 | 5.2505E6 | 7.199E5 | 4.2396E5 | 4 | 0 | 0 | 0 | 1 | 1 | 1 | 1 | 92 | 99 |  |  | PEAKS DB |
| total 3 peptides |  |  |  |  |  |  |  |  |  |  |  |  |  |  |  |  |  |  |  |  |  |  |  |  |  |  |  |  |  |  |  |
| --- | --- | --- | --- | --- | --- | --- | --- | --- | --- | --- | --- | --- | --- | --- | --- | --- | --- | --- | --- | --- | --- | --- | --- | --- | --- | --- | --- | --- | --- | --- | --- |

TRINITY\_DN1697\_c0\_g1\_i11.p1

back to list

  

| Protein Coverage
| Supporting Peptides
|

Protein Coverage:

Supporting Peptides:

| Peptide | Uniq | -10lgP | Mass | Length | ppm | m/z | z | RT | Fraction | Scan | Source File | Area F1 | Area F2 | Area F3 | Area F4 | Area F5 | Area F6 | Area F7 | #Feature | #Feature F1 | #Feature F2 | #Feature F3 | #Feature F4 | #Feature F5 | #Feature F6 | #Feature F7 | Start | End | PTM | AScore | Found By |
| --- | --- | --- | --- | --- | --- | --- | --- | --- | --- | --- | --- | --- | --- | --- | --- | --- | --- | --- | --- | --- | --- | --- | --- | --- | --- | --- | --- | --- | --- | --- | --- |
| K.AQWDFTVTADTAILKPQVK.V | Y | 88.03 | 2131.1313 | 19 | 0.7 | 711.3849 | 3 | 67.55 | 6 | F6:20959 | Fraction\_6\_23092022.raw |  |  |  |  |  | 2.5047E5 |  | 1 | 0 | 0 | 0 | 0 | 0 | 1 | 0 | 56 | 74 |  |  | PEAKS DB |
| R.KC(+57.02)EGGLPEPNELR.I | Y | 51.02 | 1497.7245 | 13 | 0.1 | 500.2488 | 3 | 34.64 | 6 | F6:6211 | Fraction\_6\_23092022.raw |  |  |  |  |  | 3.5119E5 |  | 1 | 0 | 0 | 0 | 0 | 0 | 1 | 0 | 25 | 37 | Carbamidomethylation | C2:Carbamidomethylation:1000.00 | PEAKS DB |
| total 2 peptides |  |  |  |  |  |  |  |  |  |  |  |  |  |  |  |  |  |  |  |  |  |  |  |  |  |  |  |  |  |  |  |
| --- | --- | --- | --- | --- | --- | --- | --- | --- | --- | --- | --- | --- | --- | --- | --- | --- | --- | --- | --- | --- | --- | --- | --- | --- | --- | --- | --- | --- | --- | --- | --- |

TRINITY\_DN1697\_c0\_g1\_i18.p1

back to list

  

| Protein Coverage
| Supporting Peptides
|

Protein Coverage:

Supporting Peptides:

| Peptide | Uniq | -10lgP | Mass | Length | ppm | m/z | z | RT | Fraction | Scan | Source File | Area F1 | Area F2 | Area F3 | Area F4 | Area F5 | Area F6 | Area F7 | #Feature | #Feature F1 | #Feature F2 | #Feature F3 | #Feature F4 | #Feature F5 | #Feature F6 | #Feature F7 | Start | End | PTM | AScore | Found By |
| --- | --- | --- | --- | --- | --- | --- | --- | --- | --- | --- | --- | --- | --- | --- | --- | --- | --- | --- | --- | --- | --- | --- | --- | --- | --- | --- | --- | --- | --- | --- | --- |
| K.AQWDFTVTADTAILKPQVK.V | Y | 88.03 | 2131.1313 | 19 | 0.7 | 711.3849 | 3 | 67.55 | 6 | F6:20959 | Fraction\_6\_23092022.raw |  |  |  |  |  | 2.5047E5 |  | 1 | 0 | 0 | 0 | 0 | 0 | 1 | 0 | 56 | 74 |  |  | PEAKS DB |
| R.KC(+57.02)EGGLPEPNELR.I | Y | 51.02 | 1497.7245 | 13 | 0.1 | 500.2488 | 3 | 34.64 | 6 | F6:6211 | Fraction\_6\_23092022.raw |  |  |  |  |  | 3.5119E5 |  | 1 | 0 | 0 | 0 | 0 | 0 | 1 | 0 | 25 | 37 | Carbamidomethylation | C2:Carbamidomethylation:1000.00 | PEAKS DB |
| total 2 peptides |  |  |  |  |  |  |  |  |  |  |  |  |  |  |  |  |  |  |  |  |  |  |  |  |  |  |  |  |  |  |  |
| --- | --- | --- | --- | --- | --- | --- | --- | --- | --- | --- | --- | --- | --- | --- | --- | --- | --- | --- | --- | --- | --- | --- | --- | --- | --- | --- | --- | --- | --- | --- | --- |

P00038|CYC\_APIME

back to list

  

| Protein Coverage
| Supporting Peptides
|

Protein Coverage:

Supporting Peptides:

| Peptide | Uniq | -10lgP | Mass | Length | ppm | m/z | z | RT | Fraction | Scan | Source File | Area F1 | Area F2 | Area F3 | Area F4 | Area F5 | Area F6 | Area F7 | #Feature | #Feature F1 | #Feature F2 | #Feature F3 | #Feature F4 | #Feature F5 | #Feature F6 | #Feature F7 | Start | End | PTM | AScore | Found By |
| --- | --- | --- | --- | --- | --- | --- | --- | --- | --- | --- | --- | --- | --- | --- | --- | --- | --- | --- | --- | --- | --- | --- | --- | --- | --- | --- | --- | --- | --- | --- | --- |
| R.ADLIAYIEQASK | Y | 77.90 | 1320.6925 | 12 | 0.1 | 661.3536 | 2 | 74.89 | 7 | F7:29501 | Fraction\_7\_23092022.raw |  |  |  |  |  | 6.3116E6 | 1.0339E7 | 2 | 0 | 0 | 0 | 0 | 0 | 1 | 1 | 97 | 108 |  |  | PEAKS DB |
| K.VGPNLYGVYGRK.T | Y | 71.26 | 1321.7142 | 12 | -0.2 | 441.5786 | 3 | 39.26 | 7 | F7:10402 | Fraction\_7\_23092022.raw |  |  |  |  |  | 1.6484E5 | 1.5958E5 | 2 | 0 | 0 | 0 | 0 | 0 | 1 | 1 | 33 | 44 |  |  | PEAKS DB |
| total 2 peptides |  |  |  |  |  |  |  |  |  |  |  |  |  |  |  |  |  |  |  |  |  |  |  |  |  |  |  |  |  |  |  |
| --- | --- | --- | --- | --- | --- | --- | --- | --- | --- | --- | --- | --- | --- | --- | --- | --- | --- | --- | --- | --- | --- | --- | --- | --- | --- | --- | --- | --- | --- | --- | --- |

P00768|CTR2\_VESOR

back to list

  

| Protein Coverage
| Supporting Peptides
|

Protein Coverage:

Supporting Peptides:

| Peptide | Uniq | -10lgP | Mass | Length | ppm | m/z | z | RT | Fraction | Scan | Source File | Area F1 | Area F2 | Area F3 | Area F4 | Area F5 | Area F6 | Area F7 | #Feature | #Feature F1 | #Feature F2 | #Feature F3 | #Feature F4 | #Feature F5 | #Feature F6 | #Feature F7 | Start | End | PTM | AScore | Found By |
| --- | --- | --- | --- | --- | --- | --- | --- | --- | --- | --- | --- | --- | --- | --- | --- | --- | --- | --- | --- | --- | --- | --- | --- | --- | --- | --- | --- | --- | --- | --- | --- |
| K.YPYQVSLRAPK.H | Y | 64.31 | 1320.7190 | 11 | -1.1 | 441.2465 | 3 | 40.17 | 7 | F7:10797 | Fraction\_7\_23092022.raw |  |  |  |  |  |  | 8.3929E5 | 1 | 0 | 0 | 0 | 0 | 0 | 0 | 1 | 12 | 22 |  |  | PEAKS DB |
| K.YPYQVSLR.A | Y | 62.54 | 1024.5342 | 8 | -0.1 | 513.2743 | 2 | 45.13 | 7 | F7:13104 | Fraction\_7\_23092022.raw |  |  |  |  |  |  | 3.4821E5 | 1 | 0 | 0 | 0 | 0 | 0 | 0 | 1 | 12 | 19 |  |  | PEAKS DB |
| R.LINDIGLIR.V | Y | 49.71 | 1025.6233 | 9 | -0.5 | 513.8187 | 2 | 58.17 | 6 | F6:16716 | Fraction\_6\_23092022.raw |  |  |  |  |  | 1.1555E6 | 2.548E6 | 2 | 0 | 0 | 0 | 0 | 0 | 1 | 1 | 79 | 87 |  |  | PEAKS DB |
| total 3 peptides |  |  |  |  |  |  |  |  |  |  |  |  |  |  |  |  |  |  |  |  |  |  |  |  |  |  |  |  |  |  |  |
| --- | --- | --- | --- | --- | --- | --- | --- | --- | --- | --- | --- | --- | --- | --- | --- | --- | --- | --- | --- | --- | --- | --- | --- | --- | --- | --- | --- | --- | --- | --- | --- |

P00769|CTR2\_VESCR

back to list

  

| Protein Coverage
| Supporting Peptides
|

Protein Coverage:

Supporting Peptides:

| Peptide | Uniq | -10lgP | Mass | Length | ppm | m/z | z | RT | Fraction | Scan | Source File | Area F1 | Area F2 | Area F3 | Area F4 | Area F5 | Area F6 | Area F7 | #Feature | #Feature F1 | #Feature F2 | #Feature F3 | #Feature F4 | #Feature F5 | #Feature F6 | #Feature F7 | Start | End | PTM | AScore | Found By |
| --- | --- | --- | --- | --- | --- | --- | --- | --- | --- | --- | --- | --- | --- | --- | --- | --- | --- | --- | --- | --- | --- | --- | --- | --- | --- | --- | --- | --- | --- | --- | --- |
| K.YPYQVSLRAPK.H | Y | 64.31 | 1320.7190 | 11 | -1.1 | 441.2465 | 3 | 40.17 | 7 | F7:10797 | Fraction\_7\_23092022.raw |  |  |  |  |  |  | 8.3929E5 | 1 | 0 | 0 | 0 | 0 | 0 | 0 | 1 | 12 | 22 |  |  | PEAKS DB |
| K.YPYQVSLR.A | Y | 62.54 | 1024.5342 | 8 | -0.1 | 513.2743 | 2 | 45.13 | 7 | F7:13104 | Fraction\_7\_23092022.raw |  |  |  |  |  |  | 3.4821E5 | 1 | 0 | 0 | 0 | 0 | 0 | 0 | 1 | 12 | 19 |  |  | PEAKS DB |
| R.LINDIGLIR.V | Y | 49.71 | 1025.6233 | 9 | -0.5 | 513.8187 | 2 | 58.17 | 6 | F6:16716 | Fraction\_6\_23092022.raw |  |  |  |  |  | 1.1555E6 | 2.548E6 | 2 | 0 | 0 | 0 | 0 | 0 | 1 | 1 | 81 | 89 |  |  | PEAKS DB |
| total 3 peptides |  |  |  |  |  |  |  |  |  |  |  |  |  |  |  |  |  |  |  |  |  |  |  |  |  |  |  |  |  |  |  |
| --- | --- | --- | --- | --- | --- | --- | --- | --- | --- | --- | --- | --- | --- | --- | --- | --- | --- | --- | --- | --- | --- | --- | --- | --- | --- | --- | --- | --- | --- | --- | --- |

TRINITY\_DN3922\_c0\_g1\_i1.p1

back to list

  

| Protein Coverage
| Supporting Peptides
|

Protein Coverage:

Supporting Peptides:

| Peptide | Uniq | -10lgP | Mass | Length | ppm | m/z | z | RT | Fraction | Scan | Source File | Area F1 | Area F2 | Area F3 | Area F4 | Area F5 | Area F6 | Area F7 | #Feature | #Feature F1 | #Feature F2 | #Feature F3 | #Feature F4 | #Feature F5 | #Feature F6 | #Feature F7 | Start | End | PTM | AScore | Found By |
| --- | --- | --- | --- | --- | --- | --- | --- | --- | --- | --- | --- | --- | --- | --- | --- | --- | --- | --- | --- | --- | --- | --- | --- | --- | --- | --- | --- | --- | --- | --- | --- |
| R.IVLNADRWTVTDSDSIPTGEIR.S | Y | 82.13 | 2457.2500 | 22 | 0.8 | 820.0912 | 3 | 62.08 | 6 | F6:18516 | Fraction\_6\_23092022.raw |  |  |  |  |  | 3.2699E5 |  | 1 | 0 | 0 | 0 | 0 | 0 | 1 | 0 | 219 | 240 |  |  | PEAKS DB |
| R.FVLDGKVYQVSR.N | Y | 55.17 | 1409.7666 | 12 | 0.6 | 470.9297 | 3 | 47.30 | 6 | F6:11874 | Fraction\_6\_23092022.raw |  |  |  |  |  | 3.274E5 |  | 1 | 0 | 0 | 0 | 0 | 0 | 1 | 0 | 110 | 121 |  |  | PEAKS DB |
| total 2 peptides |  |  |  |  |  |  |  |  |  |  |  |  |  |  |  |  |  |  |  |  |  |  |  |  |  |  |  |  |  |  |  |
| --- | --- | --- | --- | --- | --- | --- | --- | --- | --- | --- | --- | --- | --- | --- | --- | --- | --- | --- | --- | --- | --- | --- | --- | --- | --- | --- | --- | --- | --- | --- | --- |

TRINITY\_DN3922\_c0\_g1\_i2.p1

back to list

  

| Protein Coverage
| Supporting Peptides
|

Protein Coverage:

Supporting Peptides:

| Peptide | Uniq | -10lgP | Mass | Length | ppm | m/z | z | RT | Fraction | Scan | Source File | Area F1 | Area F2 | Area F3 | Area F4 | Area F5 | Area F6 | Area F7 | #Feature | #Feature F1 | #Feature F2 | #Feature F3 | #Feature F4 | #Feature F5 | #Feature F6 | #Feature F7 | Start | End | PTM | AScore | Found By |
| --- | --- | --- | --- | --- | --- | --- | --- | --- | --- | --- | --- | --- | --- | --- | --- | --- | --- | --- | --- | --- | --- | --- | --- | --- | --- | --- | --- | --- | --- | --- | --- |
| R.IVLNADRWTVTDSDSIPTGEIR.S | Y | 82.13 | 2457.2500 | 22 | 0.8 | 820.0912 | 3 | 62.08 | 6 | F6:18516 | Fraction\_6\_23092022.raw |  |  |  |  |  | 3.2699E5 |  | 1 | 0 | 0 | 0 | 0 | 0 | 1 | 0 | 202 | 223 |  |  | PEAKS DB |
| R.FVLDGKVYQVSR.N | Y | 55.17 | 1409.7666 | 12 | 0.6 | 470.9297 | 3 | 47.30 | 6 | F6:11874 | Fraction\_6\_23092022.raw |  |  |  |  |  | 3.274E5 |  | 1 | 0 | 0 | 0 | 0 | 0 | 1 | 0 | 93 | 104 |  |  | PEAKS DB |
| total 2 peptides |  |  |  |  |  |  |  |  |  |  |  |  |  |  |  |  |  |  |  |  |  |  |  |  |  |  |  |  |  |  |  |
| --- | --- | --- | --- | --- | --- | --- | --- | --- | --- | --- | --- | --- | --- | --- | --- | --- | --- | --- | --- | --- | --- | --- | --- | --- | --- | --- | --- | --- | --- | --- | --- |

TRINITY\_DN3922\_c0\_g1\_i3.p1

back to list

  

| Protein Coverage
| Supporting Peptides
|

Protein Coverage:

Supporting Peptides:

| Peptide | Uniq | -10lgP | Mass | Length | ppm | m/z | z | RT | Fraction | Scan | Source File | Area F1 | Area F2 | Area F3 | Area F4 | Area F5 | Area F6 | Area F7 | #Feature | #Feature F1 | #Feature F2 | #Feature F3 | #Feature F4 | #Feature F5 | #Feature F6 | #Feature F7 | Start | End | PTM | AScore | Found By |
| --- | --- | --- | --- | --- | --- | --- | --- | --- | --- | --- | --- | --- | --- | --- | --- | --- | --- | --- | --- | --- | --- | --- | --- | --- | --- | --- | --- | --- | --- | --- | --- |
| R.IVLNADRWTVTDSDSIPTGEIR.S | Y | 82.13 | 2457.2500 | 22 | 0.8 | 820.0912 | 3 | 62.08 | 6 | F6:18516 | Fraction\_6\_23092022.raw |  |  |  |  |  | 3.2699E5 |  | 1 | 0 | 0 | 0 | 0 | 0 | 1 | 0 | 202 | 223 |  |  | PEAKS DB |
| R.FVLDGKVYQVSR.N | Y | 55.17 | 1409.7666 | 12 | 0.6 | 470.9297 | 3 | 47.30 | 6 | F6:11874 | Fraction\_6\_23092022.raw |  |  |  |  |  | 3.274E5 |  | 1 | 0 | 0 | 0 | 0 | 0 | 1 | 0 | 93 | 104 |  |  | PEAKS DB |
| total 2 peptides |  |  |  |  |  |  |  |  |  |  |  |  |  |  |  |  |  |  |  |  |  |  |  |  |  |  |  |  |  |  |  |
| --- | --- | --- | --- | --- | --- | --- | --- | --- | --- | --- | --- | --- | --- | --- | --- | --- | --- | --- | --- | --- | --- | --- | --- | --- | --- | --- | --- | --- | --- | --- | --- |

Q6IME9|K2C72\_MOUSE

back to list

  

| Protein Coverage
| Supporting Peptides
|

Protein Coverage:

Supporting Peptides:

| Peptide | Uniq | -10lgP | Mass | Length | ppm | m/z | z | RT | Fraction | Scan | Source File | Area F1 | Area F2 | Area F3 | Area F4 | Area F5 | Area F6 | Area F7 | #Feature | #Feature F1 | #Feature F2 | #Feature F3 | #Feature F4 | #Feature F5 | #Feature F6 | #Feature F7 | Start | End | PTM | AScore | Found By |
| --- | --- | --- | --- | --- | --- | --- | --- | --- | --- | --- | --- | --- | --- | --- | --- | --- | --- | --- | --- | --- | --- | --- | --- | --- | --- | --- | --- | --- | --- | --- | --- |
| K.FASFIDKVR.F | N | 64.36 | 1081.5920 | 9 | 1.5 | 361.5385 | 3 | 44.57 | 4 | F4:10216 | Fraction\_4\_23092022.raw | 6.0874E6 | 1.3079E6 | 2.6129E6 | 3.499E5 | 3.5976E5 | 1.1286E6 |  | 11 | 1 | 2 | 2 | 2 | 2 | 2 | 0 | 145 | 153 |  |  | PEAKS DB |
| R.FLEQQNQVLETK.W | Y | 56.69 | 1475.7620 | 12 | 0.1 | 738.8883 | 2 | 43.35 | 3 | F3:9095 | Fraction\_3\_23092022.raw |  | 7.6243E4 | 1.7453E5 |  |  |  |  | 2 | 0 | 1 | 1 | 0 | 0 | 0 | 0 | 154 | 165 |  |  | PEAKS DB |
| total 2 peptides |  |  |  |  |  |  |  |  |  |  |  |  |  |  |  |  |  |  |  |  |  |  |  |  |  |  |  |  |  |  |  |
| --- | --- | --- | --- | --- | --- | --- | --- | --- | --- | --- | --- | --- | --- | --- | --- | --- | --- | --- | --- | --- | --- | --- | --- | --- | --- | --- | --- | --- | --- | --- | --- |

Q86Y46|K2C73\_HUMAN

back to list

  

| Protein Coverage
| Supporting Peptides
|

Protein Coverage:

Supporting Peptides:

| Peptide | Uniq | -10lgP | Mass | Length | ppm | m/z | z | RT | Fraction | Scan | Source File | Area F1 | Area F2 | Area F3 | Area F4 | Area F5 | Area F6 | Area F7 | #Feature | #Feature F1 | #Feature F2 | #Feature F3 | #Feature F4 | #Feature F5 | #Feature F6 | #Feature F7 | Start | End | PTM | AScore | Found By |
| --- | --- | --- | --- | --- | --- | --- | --- | --- | --- | --- | --- | --- | --- | --- | --- | --- | --- | --- | --- | --- | --- | --- | --- | --- | --- | --- | --- | --- | --- | --- | --- |
| K.FASFIDKVR.F | N | 64.36 | 1081.5920 | 9 | 1.5 | 361.5385 | 3 | 44.57 | 4 | F4:10216 | Fraction\_4\_23092022.raw | 6.0874E6 | 1.3079E6 | 2.6129E6 | 3.499E5 | 3.5976E5 | 1.1286E6 |  | 11 | 1 | 2 | 2 | 2 | 2 | 2 | 0 | 143 | 151 |  |  | PEAKS DB |
| R.FLEQQNQVLETK.W | Y | 56.69 | 1475.7620 | 12 | 0.1 | 738.8883 | 2 | 43.35 | 3 | F3:9095 | Fraction\_3\_23092022.raw |  | 7.6243E4 | 1.7453E5 |  |  |  |  | 2 | 0 | 1 | 1 | 0 | 0 | 0 | 0 | 152 | 163 |  |  | PEAKS DB |
| total 2 peptides |  |  |  |  |  |  |  |  |  |  |  |  |  |  |  |  |  |  |  |  |  |  |  |  |  |  |  |  |  |  |  |
| --- | --- | --- | --- | --- | --- | --- | --- | --- | --- | --- | --- | --- | --- | --- | --- | --- | --- | --- | --- | --- | --- | --- | --- | --- | --- | --- | --- | --- | --- | --- | --- |

Q3SY84|K2C71\_HUMAN

back to list

  

| Protein Coverage
| Supporting Peptides
|

Protein Coverage:

Supporting Peptides:

| Peptide | Uniq | -10lgP | Mass | Length | ppm | m/z | z | RT | Fraction | Scan | Source File | Area F1 | Area F2 | Area F3 | Area F4 | Area F5 | Area F6 | Area F7 | #Feature | #Feature F1 | #Feature F2 | #Feature F3 | #Feature F4 | #Feature F5 | #Feature F6 | #Feature F7 | Start | End | PTM | AScore | Found By |
| --- | --- | --- | --- | --- | --- | --- | --- | --- | --- | --- | --- | --- | --- | --- | --- | --- | --- | --- | --- | --- | --- | --- | --- | --- | --- | --- | --- | --- | --- | --- | --- |
| K.FASFIDKVR.F | N | 64.36 | 1081.5920 | 9 | 1.5 | 361.5385 | 3 | 44.57 | 4 | F4:10216 | Fraction\_4\_23092022.raw | 6.0874E6 | 1.3079E6 | 2.6129E6 | 3.499E5 | 3.5976E5 | 1.1286E6 |  | 11 | 1 | 2 | 2 | 2 | 2 | 2 | 0 | 141 | 149 |  |  | PEAKS DB |
| R.FLEQQNQVLETK.W | Y | 56.69 | 1475.7620 | 12 | 0.1 | 738.8883 | 2 | 43.35 | 3 | F3:9095 | Fraction\_3\_23092022.raw |  | 7.6243E4 | 1.7453E5 |  |  |  |  | 2 | 0 | 1 | 1 | 0 | 0 | 0 | 0 | 150 | 161 |  |  | PEAKS DB |
| total 2 peptides |  |  |  |  |  |  |  |  |  |  |  |  |  |  |  |  |  |  |  |  |  |  |  |  |  |  |  |  |  |  |  |
| --- | --- | --- | --- | --- | --- | --- | --- | --- | --- | --- | --- | --- | --- | --- | --- | --- | --- | --- | --- | --- | --- | --- | --- | --- | --- | --- | --- | --- | --- | --- | --- |

Q148H5|K2C71\_BOVIN

back to list

  

| Protein Coverage
| Supporting Peptides
|

Protein Coverage:

Supporting Peptides:

| Peptide | Uniq | -10lgP | Mass | Length | ppm | m/z | z | RT | Fraction | Scan | Source File | Area F1 | Area F2 | Area F3 | Area F4 | Area F5 | Area F6 | Area F7 | #Feature | #Feature F1 | #Feature F2 | #Feature F3 | #Feature F4 | #Feature F5 | #Feature F6 | #Feature F7 | Start | End | PTM | AScore | Found By |
| --- | --- | --- | --- | --- | --- | --- | --- | --- | --- | --- | --- | --- | --- | --- | --- | --- | --- | --- | --- | --- | --- | --- | --- | --- | --- | --- | --- | --- | --- | --- | --- |
| K.FASFIDKVR.F | N | 64.36 | 1081.5920 | 9 | 1.5 | 361.5385 | 3 | 44.57 | 4 | F4:10216 | Fraction\_4\_23092022.raw | 6.0874E6 | 1.3079E6 | 2.6129E6 | 3.499E5 | 3.5976E5 | 1.1286E6 |  | 11 | 1 | 2 | 2 | 2 | 2 | 2 | 0 | 143 | 151 |  |  | PEAKS DB |
| R.FLEQQNQVLETK.W | Y | 56.69 | 1475.7620 | 12 | 0.1 | 738.8883 | 2 | 43.35 | 3 | F3:9095 | Fraction\_3\_23092022.raw |  | 7.6243E4 | 1.7453E5 |  |  |  |  | 2 | 0 | 1 | 1 | 0 | 0 | 0 | 0 | 152 | 163 |  |  | PEAKS DB |
| total 2 peptides |  |  |  |  |  |  |  |  |  |  |  |  |  |  |  |  |  |  |  |  |  |  |  |  |  |  |  |  |  |  |  |
| --- | --- | --- | --- | --- | --- | --- | --- | --- | --- | --- | --- | --- | --- | --- | --- | --- | --- | --- | --- | --- | --- | --- | --- | --- | --- | --- | --- | --- | --- | --- | --- |

Q14CN4|K2C72\_HUMAN

back to list

  

| Protein Coverage
| Supporting Peptides
|

Protein Coverage:

Supporting Peptides:

| Peptide | Uniq | -10lgP | Mass | Length | ppm | m/z | z | RT | Fraction | Scan | Source File | Area F1 | Area F2 | Area F3 | Area F4 | Area F5 | Area F6 | Area F7 | #Feature | #Feature F1 | #Feature F2 | #Feature F3 | #Feature F4 | #Feature F5 | #Feature F6 | #Feature F7 | Start | End | PTM | AScore | Found By |
| --- | --- | --- | --- | --- | --- | --- | --- | --- | --- | --- | --- | --- | --- | --- | --- | --- | --- | --- | --- | --- | --- | --- | --- | --- | --- | --- | --- | --- | --- | --- | --- |
| K.FASFIDKVR.F | N | 64.36 | 1081.5920 | 9 | 1.5 | 361.5385 | 3 | 44.57 | 4 | F4:10216 | Fraction\_4\_23092022.raw | 6.0874E6 | 1.3079E6 | 2.6129E6 | 3.499E5 | 3.5976E5 | 1.1286E6 |  | 11 | 1 | 2 | 2 | 2 | 2 | 2 | 0 | 136 | 144 |  |  | PEAKS DB |
| R.FLEQQNQVLETK.W | Y | 56.69 | 1475.7620 | 12 | 0.1 | 738.8883 | 2 | 43.35 | 3 | F3:9095 | Fraction\_3\_23092022.raw |  | 7.6243E4 | 1.7453E5 |  |  |  |  | 2 | 0 | 1 | 1 | 0 | 0 | 0 | 0 | 145 | 156 |  |  | PEAKS DB |
| total 2 peptides |  |  |  |  |  |  |  |  |  |  |  |  |  |  |  |  |  |  |  |  |  |  |  |  |  |  |  |  |  |  |  |
| --- | --- | --- | --- | --- | --- | --- | --- | --- | --- | --- | --- | --- | --- | --- | --- | --- | --- | --- | --- | --- | --- | --- | --- | --- | --- | --- | --- | --- | --- | --- | --- |

E1AB55|K2C71\_FELCA

back to list

  

| Protein Coverage
| Supporting Peptides
|

Protein Coverage:

Supporting Peptides:

| Peptide | Uniq | -10lgP | Mass | Length | ppm | m/z | z | RT | Fraction | Scan | Source File | Area F1 | Area F2 | Area F3 | Area F4 | Area F5 | Area F6 | Area F7 | #Feature | #Feature F1 | #Feature F2 | #Feature F3 | #Feature F4 | #Feature F5 | #Feature F6 | #Feature F7 | Start | End | PTM | AScore | Found By |
| --- | --- | --- | --- | --- | --- | --- | --- | --- | --- | --- | --- | --- | --- | --- | --- | --- | --- | --- | --- | --- | --- | --- | --- | --- | --- | --- | --- | --- | --- | --- | --- |
| K.FASFIDKVR.F | N | 64.36 | 1081.5920 | 9 | 1.5 | 361.5385 | 3 | 44.57 | 4 | F4:10216 | Fraction\_4\_23092022.raw | 6.0874E6 | 1.3079E6 | 2.6129E6 | 3.499E5 | 3.5976E5 | 1.1286E6 |  | 11 | 1 | 2 | 2 | 2 | 2 | 2 | 0 | 142 | 150 |  |  | PEAKS DB |
| R.FLEQQNQVLETK.W | Y | 56.69 | 1475.7620 | 12 | 0.1 | 738.8883 | 2 | 43.35 | 3 | F3:9095 | Fraction\_3\_23092022.raw |  | 7.6243E4 | 1.7453E5 |  |  |  |  | 2 | 0 | 1 | 1 | 0 | 0 | 0 | 0 | 151 | 162 |  |  | PEAKS DB |
| total 2 peptides |  |  |  |  |  |  |  |  |  |  |  |  |  |  |  |  |  |  |  |  |  |  |  |  |  |  |  |  |  |  |  |
| --- | --- | --- | --- | --- | --- | --- | --- | --- | --- | --- | --- | --- | --- | --- | --- | --- | --- | --- | --- | --- | --- | --- | --- | --- | --- | --- | --- | --- | --- | --- | --- |

Q6IG04|K2C72\_RAT

back to list

  

| Protein Coverage
| Supporting Peptides
|

Protein Coverage:

Supporting Peptides:

| Peptide | Uniq | -10lgP | Mass | Length | ppm | m/z | z | RT | Fraction | Scan | Source File | Area F1 | Area F2 | Area F3 | Area F4 | Area F5 | Area F6 | Area F7 | #Feature | #Feature F1 | #Feature F2 | #Feature F3 | #Feature F4 | #Feature F5 | #Feature F6 | #Feature F7 | Start | End | PTM | AScore | Found By |
| --- | --- | --- | --- | --- | --- | --- | --- | --- | --- | --- | --- | --- | --- | --- | --- | --- | --- | --- | --- | --- | --- | --- | --- | --- | --- | --- | --- | --- | --- | --- | --- |
| K.FASFIDKVR.F | N | 64.36 | 1081.5920 | 9 | 1.5 | 361.5385 | 3 | 44.57 | 4 | F4:10216 | Fraction\_4\_23092022.raw | 6.0874E6 | 1.3079E6 | 2.6129E6 | 3.499E5 | 3.5976E5 | 1.1286E6 |  | 11 | 1 | 2 | 2 | 2 | 2 | 2 | 0 | 145 | 153 |  |  | PEAKS DB |
| R.FLEQQNQVLETK.W | Y | 56.69 | 1475.7620 | 12 | 0.1 | 738.8883 | 2 | 43.35 | 3 | F3:9095 | Fraction\_3\_23092022.raw |  | 7.6243E4 | 1.7453E5 |  |  |  |  | 2 | 0 | 1 | 1 | 0 | 0 | 0 | 0 | 154 | 165 |  |  | PEAKS DB |
| total 2 peptides |  |  |  |  |  |  |  |  |  |  |  |  |  |  |  |  |  |  |  |  |  |  |  |  |  |  |  |  |  |  |  |
| --- | --- | --- | --- | --- | --- | --- | --- | --- | --- | --- | --- | --- | --- | --- | --- | --- | --- | --- | --- | --- | --- | --- | --- | --- | --- | --- | --- | --- | --- | --- | --- |

Q7RTS7|K2C74\_HUMAN

back to list

  

| Protein Coverage
| Supporting Peptides
|

Protein Coverage:

Supporting Peptides:

| Peptide | Uniq | -10lgP | Mass | Length | ppm | m/z | z | RT | Fraction | Scan | Source File | Area F1 | Area F2 | Area F3 | Area F4 | Area F5 | Area F6 | Area F7 | #Feature | #Feature F1 | #Feature F2 | #Feature F3 | #Feature F4 | #Feature F5 | #Feature F6 | #Feature F7 | Start | End | PTM | AScore | Found By |
| --- | --- | --- | --- | --- | --- | --- | --- | --- | --- | --- | --- | --- | --- | --- | --- | --- | --- | --- | --- | --- | --- | --- | --- | --- | --- | --- | --- | --- | --- | --- | --- |
| K.FASFIDKVR.F | N | 64.36 | 1081.5920 | 9 | 1.5 | 361.5385 | 3 | 44.57 | 4 | F4:10216 | Fraction\_4\_23092022.raw | 6.0874E6 | 1.3079E6 | 2.6129E6 | 3.499E5 | 3.5976E5 | 1.1286E6 |  | 11 | 1 | 2 | 2 | 2 | 2 | 2 | 0 | 151 | 159 |  |  | PEAKS DB |
| R.FLEQQNQVLETK.W | Y | 56.69 | 1475.7620 | 12 | 0.1 | 738.8883 | 2 | 43.35 | 3 | F3:9095 | Fraction\_3\_23092022.raw |  | 7.6243E4 | 1.7453E5 |  |  |  |  | 2 | 0 | 1 | 1 | 0 | 0 | 0 | 0 | 160 | 171 |  |  | PEAKS DB |
| total 2 peptides |  |  |  |  |  |  |  |  |  |  |  |  |  |  |  |  |  |  |  |  |  |  |  |  |  |  |  |  |  |  |  |
| --- | --- | --- | --- | --- | --- | --- | --- | --- | --- | --- | --- | --- | --- | --- | --- | --- | --- | --- | --- | --- | --- | --- | --- | --- | --- | --- | --- | --- | --- | --- | --- |

P12273|PIP\_HUMAN

back to list

  

| Protein Coverage
| Supporting Peptides
|

Protein Coverage:

Supporting Peptides:

| Peptide | Uniq | -10lgP | Mass | Length | ppm | m/z | z | RT | Fraction | Scan | Source File | Area F1 | Area F2 | Area F3 | Area F4 | Area F5 | Area F6 | Area F7 | #Feature | #Feature F1 | #Feature F2 | #Feature F3 | #Feature F4 | #Feature F5 | #Feature F6 | #Feature F7 | Start | End | PTM | AScore | Found By |
| --- | --- | --- | --- | --- | --- | --- | --- | --- | --- | --- | --- | --- | --- | --- | --- | --- | --- | --- | --- | --- | --- | --- | --- | --- | --- | --- | --- | --- | --- | --- | --- |
| R.ELGIC(+57.02)PDDAAVIPIK.N | Y | 59.23 | 1609.8385 | 15 | -0.3 | 805.9263 | 2 | 68.14 | 1 | F1:19670 | Fraction\_1\_23092022r.raw | 2.3594E5 |  |  |  |  |  |  | 1 | 1 | 0 | 0 | 0 | 0 | 0 | 0 | 119 | 133 | Carbamidomethylation | C5:Carbamidomethylation:1000.00 | PEAKS DB |
| R.FYTIEILKVE | Y | 58.67 | 1253.6907 | 10 | 0.0 | 627.8526 | 2 | 74.00 | 6 | F6:23803 | Fraction\_6\_23092022.raw | 2.1299E6 | 1.2382E6 | 3.5017E5 | 5.2023E5 | 6.6449E5 | 1.8421E6 | 1.2852E6 | 7 | 1 | 1 | 1 | 1 | 1 | 1 | 1 | 137 | 146 |  |  | PEAKS DB |
| K.TYLISSIPLQGAFNYK.Y | Y | 51.46 | 1813.9614 | 16 | 0.5 | 907.9885 | 2 | 78.54 | 1 | F1:24331 | Fraction\_1\_23092022r.raw | 1.0821E5 |  |  |  |  |  |  | 1 | 1 | 0 | 0 | 0 | 0 | 0 | 0 | 70 | 85 |  |  | PEAKS DB |
| total 3 peptides |  |  |  |  |  |  |  |  |  |  |  |  |  |  |  |  |  |  |  |  |  |  |  |  |  |  |  |  |  |  |  |
| --- | --- | --- | --- | --- | --- | --- | --- | --- | --- | --- | --- | --- | --- | --- | --- | --- | --- | --- | --- | --- | --- | --- | --- | --- | --- | --- | --- | --- | --- | --- | --- |

TRINITY\_DN5303\_c0\_g1\_i4.p1

back to list

  

| Protein Coverage
| Supporting Peptides
|

Protein Coverage:

Supporting Peptides:

| Peptide | Uniq | -10lgP | Mass | Length | ppm | m/z | z | RT | Fraction | Scan | Source File | Area F1 | Area F2 | Area F3 | Area F4 | Area F5 | Area F6 | Area F7 | #Feature | #Feature F1 | #Feature F2 | #Feature F3 | #Feature F4 | #Feature F5 | #Feature F6 | #Feature F7 | Start | End | PTM | AScore | Found By |
| --- | --- | --- | --- | --- | --- | --- | --- | --- | --- | --- | --- | --- | --- | --- | --- | --- | --- | --- | --- | --- | --- | --- | --- | --- | --- | --- | --- | --- | --- | --- | --- |
| K.IILDVHQFKPEEISVR.V | Y | 80.51 | 1922.0625 | 16 | 0.0 | 481.5229 | 4 | 57.05 | 7 | F7:19706 | Fraction\_7\_23092022.raw |  |  |  |  |  |  | 1.3336E6 | 1 | 0 | 0 | 0 | 0 | 0 | 0 | 1 | 128 | 143 |  |  | PEAKS DB |
| R.VVNRYLVVEGKHEER.R | Y | 49.25 | 1825.9799 | 15 | -0.4 | 457.5021 | 4 | 23.62 | 7 | F7:3749 | Fraction\_7\_23092022.raw |  |  |  |  |  |  | 6.1283E4 | 1 | 0 | 0 | 0 | 0 | 0 | 0 | 1 | 144 | 158 |  |  | PEAKS DB |
| total 2 peptides |  |  |  |  |  |  |  |  |  |  |  |  |  |  |  |  |  |  |  |  |  |  |  |  |  |  |  |  |  |  |  |
| --- | --- | --- | --- | --- | --- | --- | --- | --- | --- | --- | --- | --- | --- | --- | --- | --- | --- | --- | --- | --- | --- | --- | --- | --- | --- | --- | --- | --- | --- | --- | --- |

TRINITY\_DN36500\_c0\_g1\_i2.p1

back to list

  

| Protein Coverage
| Supporting Peptides
|

Protein Coverage:

Supporting Peptides:

| Peptide | Uniq | -10lgP | Mass | Length | ppm | m/z | z | RT | Fraction | Scan | Source File | Area F1 | Area F2 | Area F3 | Area F4 | Area F5 | Area F6 | Area F7 | #Feature | #Feature F1 | #Feature F2 | #Feature F3 | #Feature F4 | #Feature F5 | #Feature F6 | #Feature F7 | Start | End | PTM | AScore | Found By |
| --- | --- | --- | --- | --- | --- | --- | --- | --- | --- | --- | --- | --- | --- | --- | --- | --- | --- | --- | --- | --- | --- | --- | --- | --- | --- | --- | --- | --- | --- | --- | --- |
| K.VFYTVNEPAILC(+57.02)GEGYGSTTK.A | Y | 83.07 | 2305.0935 | 21 | 0.5 | 1153.5546 | 2 | 66.75 | 2 | F2:19248 | Fraction\_2\_23092022.raw |  | 2.614E5 |  |  |  |  |  | 1 | 0 | 1 | 0 | 0 | 0 | 0 | 0 | 159 | 179 | Carbamidomethylation | C12:Carbamidomethylation:1000.00 | PEAKS DB |
| total 1 peptides |  |  |  |  |  |  |  |  |  |  |  |  |  |  |  |  |  |  |  |  |  |  |  |  |  |  |  |  |  |  |  |
| --- | --- | --- | --- | --- | --- | --- | --- | --- | --- | --- | --- | --- | --- | --- | --- | --- | --- | --- | --- | --- | --- | --- | --- | --- | --- | --- | --- | --- | --- | --- | --- |

TRINITY\_DN122\_c0\_g1\_i5.p1

back to list

  

| Protein Coverage
| Supporting Peptides
|

Protein Coverage:

Supporting Peptides:

| Peptide | Uniq | -10lgP | Mass | Length | ppm | m/z | z | RT | Fraction | Scan | Source File | Area F1 | Area F2 | Area F3 | Area F4 | Area F5 | Area F6 | Area F7 | #Feature | #Feature F1 | #Feature F2 | #Feature F3 | #Feature F4 | #Feature F5 | #Feature F6 | #Feature F7 | Start | End | PTM | AScore | Found By |
| --- | --- | --- | --- | --- | --- | --- | --- | --- | --- | --- | --- | --- | --- | --- | --- | --- | --- | --- | --- | --- | --- | --- | --- | --- | --- | --- | --- | --- | --- | --- | --- |
| R.YSLGRVPIGGSDFSTR.A | Y | 104.69 | 1710.8689 | 16 | 1.9 | 571.2980 | 3 | 51.63 | 2 | F2:12390 | Fraction\_2\_23092022.raw |  | 1.0063E7 |  |  |  |  |  | 1 | 0 | 1 | 0 | 0 | 0 | 0 | 0 | 144 | 159 |  |  | PEAKS DB |
| total 1 peptides |  |  |  |  |  |  |  |  |  |  |  |  |  |  |  |  |  |  |  |  |  |  |  |  |  |  |  |  |  |  |  |
| --- | --- | --- | --- | --- | --- | --- | --- | --- | --- | --- | --- | --- | --- | --- | --- | --- | --- | --- | --- | --- | --- | --- | --- | --- | --- | --- | --- | --- | --- | --- | --- |

B5U6Y8|OXLA\_ECHOC

back to list

  

| Protein Coverage
| Supporting Peptides
|

Protein Coverage:

Supporting Peptides:

| Peptide | Uniq | -10lgP | Mass | Length | ppm | m/z | z | RT | Fraction | Scan | Source File | Area F1 | Area F2 | Area F3 | Area F4 | Area F5 | Area F6 | Area F7 | #Feature | #Feature F1 | #Feature F2 | #Feature F3 | #Feature F4 | #Feature F5 | #Feature F6 | #Feature F7 | Start | End | PTM | AScore | Found By |
| --- | --- | --- | --- | --- | --- | --- | --- | --- | --- | --- | --- | --- | --- | --- | --- | --- | --- | --- | --- | --- | --- | --- | --- | --- | --- | --- | --- | --- | --- | --- | --- |
| K.DIVVVGAGMSGLSAAYVLAGAGHK.V | Y | 104.20 | 2242.1780 | 24 | 0.0 | 748.3999 | 3 | 79.31 | 1 | F1:24687 | Fraction\_1\_23092022r.raw | 2.516E5 |  |  |  |  |  |  | 1 | 1 | 0 | 0 | 0 | 0 | 0 | 0 | 53 | 76 |  |  | PEAKS DB |
| K.DIVVVGAGM(+15.99)SGLSAAYVLAGAGHK.V | Y | 92.41 | 2258.1729 | 24 | 1.3 | 753.7325 | 3 | 70.57 | 1 | F1:20752 | Fraction\_1\_23092022r.raw | 2.5963E5 |  | 3.9238E4 |  |  |  |  | 2 | 1 | 0 | 1 | 0 | 0 | 0 | 0 | 53 | 76 | Oxidation (M) | M9:Oxidation (M):1000.00 | PEAKS DB |
| total 2 peptides |  |  |  |  |  |  |  |  |  |  |  |  |  |  |  |  |  |  |  |  |  |  |  |  |  |  |  |  |  |  |  |
| --- | --- | --- | --- | --- | --- | --- | --- | --- | --- | --- | --- | --- | --- | --- | --- | --- | --- | --- | --- | --- | --- | --- | --- | --- | --- | --- | --- | --- | --- | --- | --- |

P62972|UBIQP\_XENLA

back to list

  

| Protein Coverage
| Supporting Peptides
|

Protein Coverage:

Supporting Peptides:

| Peptide | Uniq | -10lgP | Mass | Length | ppm | m/z | z | RT | Fraction | Scan | Source File | Area F1 | Area F2 | Area F3 | Area F4 | Area F5 | Area F6 | Area F7 | #Feature | #Feature F1 | #Feature F2 | #Feature F3 | #Feature F4 | #Feature F5 | #Feature F6 | #Feature F7 | Start | End | PTM | AScore | Found By |
| --- | --- | --- | --- | --- | --- | --- | --- | --- | --- | --- | --- | --- | --- | --- | --- | --- | --- | --- | --- | --- | --- | --- | --- | --- | --- | --- | --- | --- | --- | --- | --- |
| K.TITLEVEPSDTIENVK.A | Y | 74.53 | 1786.9200 | 16 | 0.3 | 894.4675 | 2 | 59.25 | 5 | F5:16571 | Fraction\_5\_23092022.raw | 2.0921E5 | 2.2744E5 | 4.5763E5 | 8.1486E4 | 8.2535E4 |  |  | 5 | 1 | 1 | 1 | 1 | 1 | 0 | 0 | 27 | 42 |  |  | PEAKS DB |
| R.LIFAGKQLEDGR.T | Y | 57.40 | 1345.7354 | 12 | 0.8 | 449.5861 | 3 | 41.76 | 7 | F7:11524 | Fraction\_7\_23092022.raw |  |  | 7.085E4 |  |  | 1.6527E5 | 3.3803E5 | 3 | 0 | 0 | 1 | 0 | 0 | 1 | 1 | 58 | 69 |  |  | PEAKS DB |
| total 2 peptides |  |  |  |  |  |  |  |  |  |  |  |  |  |  |  |  |  |  |  |  |  |  |  |  |  |  |  |  |  |  |  |
| --- | --- | --- | --- | --- | --- | --- | --- | --- | --- | --- | --- | --- | --- | --- | --- | --- | --- | --- | --- | --- | --- | --- | --- | --- | --- | --- | --- | --- | --- | --- | --- |

P0CG65|UBB\_PANTR

back to list

  

| Protein Coverage
| Supporting Peptides
|

Protein Coverage:

Supporting Peptides:

| Peptide | Uniq | -10lgP | Mass | Length | ppm | m/z | z | RT | Fraction | Scan | Source File | Area F1 | Area F2 | Area F3 | Area F4 | Area F5 | Area F6 | Area F7 | #Feature | #Feature F1 | #Feature F2 | #Feature F3 | #Feature F4 | #Feature F5 | #Feature F6 | #Feature F7 | Start | End | PTM | AScore | Found By |
| --- | --- | --- | --- | --- | --- | --- | --- | --- | --- | --- | --- | --- | --- | --- | --- | --- | --- | --- | --- | --- | --- | --- | --- | --- | --- | --- | --- | --- | --- | --- | --- |
| K.TITLEVEPSDTIENVK.A | Y | 74.53 | 1786.9200 | 16 | 0.3 | 894.4675 | 2 | 59.25 | 5 | F5:16571 | Fraction\_5\_23092022.raw | 2.0921E5 | 2.2744E5 | 4.5763E5 | 8.1486E4 | 8.2535E4 |  |  | 5 | 1 | 1 | 1 | 1 | 1 | 0 | 0 | 12 | 27 |  |  | PEAKS DB |
| R.LIFAGKQLEDGR.T | Y | 57.40 | 1345.7354 | 12 | 0.8 | 449.5861 | 3 | 41.76 | 7 | F7:11524 | Fraction\_7\_23092022.raw |  |  | 7.085E4 |  |  | 1.6527E5 | 3.3803E5 | 3 | 0 | 0 | 1 | 0 | 0 | 1 | 1 | 43 | 54 |  |  | PEAKS DB |
| total 2 peptides |  |  |  |  |  |  |  |  |  |  |  |  |  |  |  |  |  |  |  |  |  |  |  |  |  |  |  |  |  |  |  |
| --- | --- | --- | --- | --- | --- | --- | --- | --- | --- | --- | --- | --- | --- | --- | --- | --- | --- | --- | --- | --- | --- | --- | --- | --- | --- | --- | --- | --- | --- | --- | --- |

P0CG62|UBB\_CHICK

back to list

  

| Protein Coverage
| Supporting Peptides
|

Protein Coverage:

Supporting Peptides:

| Peptide | Uniq | -10lgP | Mass | Length | ppm | m/z | z | RT | Fraction | Scan | Source File | Area F1 | Area F2 | Area F3 | Area F4 | Area F5 | Area F6 | Area F7 | #Feature | #Feature F1 | #Feature F2 | #Feature F3 | #Feature F4 | #Feature F5 | #Feature F6 | #Feature F7 | Start | End | PTM | AScore | Found By |
| --- | --- | --- | --- | --- | --- | --- | --- | --- | --- | --- | --- | --- | --- | --- | --- | --- | --- | --- | --- | --- | --- | --- | --- | --- | --- | --- | --- | --- | --- | --- | --- |
| K.TITLEVEPSDTIENVK.A | Y | 74.53 | 1786.9200 | 16 | 0.3 | 894.4675 | 2 | 59.25 | 5 | F5:16571 | Fraction\_5\_23092022.raw | 2.0921E5 | 2.2744E5 | 4.5763E5 | 8.1486E4 | 8.2535E4 |  |  | 5 | 1 | 1 | 1 | 1 | 1 | 0 | 0 | 12 | 27 |  |  | PEAKS DB |
| R.LIFAGKQLEDGR.T | Y | 57.40 | 1345.7354 | 12 | 0.8 | 449.5861 | 3 | 41.76 | 7 | F7:11524 | Fraction\_7\_23092022.raw |  |  | 7.085E4 |  |  | 1.6527E5 | 3.3803E5 | 3 | 0 | 0 | 1 | 0 | 0 | 1 | 1 | 43 | 54 |  |  | PEAKS DB |
| total 2 peptides |  |  |  |  |  |  |  |  |  |  |  |  |  |  |  |  |  |  |  |  |  |  |  |  |  |  |  |  |  |  |  |
| --- | --- | --- | --- | --- | --- | --- | --- | --- | --- | --- | --- | --- | --- | --- | --- | --- | --- | --- | --- | --- | --- | --- | --- | --- | --- | --- | --- | --- | --- | --- | --- |

P0CG51|UBB\_RAT

back to list

  

| Protein Coverage
| Supporting Peptides
|

Protein Coverage:

Supporting Peptides:

| Peptide | Uniq | -10lgP | Mass | Length | ppm | m/z | z | RT | Fraction | Scan | Source File | Area F1 | Area F2 | Area F3 | Area F4 | Area F5 | Area F6 | Area F7 | #Feature | #Feature F1 | #Feature F2 | #Feature F3 | #Feature F4 | #Feature F5 | #Feature F6 | #Feature F7 | Start | End | PTM | AScore | Found By |
| --- | --- | --- | --- | --- | --- | --- | --- | --- | --- | --- | --- | --- | --- | --- | --- | --- | --- | --- | --- | --- | --- | --- | --- | --- | --- | --- | --- | --- | --- | --- | --- |
| K.TITLEVEPSDTIENVK.A | Y | 74.53 | 1786.9200 | 16 | 0.3 | 894.4675 | 2 | 59.25 | 5 | F5:16571 | Fraction\_5\_23092022.raw | 2.0921E5 | 2.2744E5 | 4.5763E5 | 8.1486E4 | 8.2535E4 |  |  | 5 | 1 | 1 | 1 | 1 | 1 | 0 | 0 | 12 | 27 |  |  | PEAKS DB |
| R.LIFAGKQLEDGR.T | Y | 57.40 | 1345.7354 | 12 | 0.8 | 449.5861 | 3 | 41.76 | 7 | F7:11524 | Fraction\_7\_23092022.raw |  |  | 7.085E4 |  |  | 1.6527E5 | 3.3803E5 | 3 | 0 | 0 | 1 | 0 | 0 | 1 | 1 | 43 | 54 |  |  | PEAKS DB |
| total 2 peptides |  |  |  |  |  |  |  |  |  |  |  |  |  |  |  |  |  |  |  |  |  |  |  |  |  |  |  |  |  |  |  |
| --- | --- | --- | --- | --- | --- | --- | --- | --- | --- | --- | --- | --- | --- | --- | --- | --- | --- | --- | --- | --- | --- | --- | --- | --- | --- | --- | --- | --- | --- | --- | --- |

P0CG68|UBC\_PIG

back to list

  

| Protein Coverage
| Supporting Peptides
|

Protein Coverage:

Supporting Peptides:

| Peptide | Uniq | -10lgP | Mass | Length | ppm | m/z | z | RT | Fraction | Scan | Source File | Area F1 | Area F2 | Area F3 | Area F4 | Area F5 | Area F6 | Area F7 | #Feature | #Feature F1 | #Feature F2 | #Feature F3 | #Feature F4 | #Feature F5 | #Feature F6 | #Feature F7 | Start | End | PTM | AScore | Found By |
| --- | --- | --- | --- | --- | --- | --- | --- | --- | --- | --- | --- | --- | --- | --- | --- | --- | --- | --- | --- | --- | --- | --- | --- | --- | --- | --- | --- | --- | --- | --- | --- |
| K.TITLEVEPSDTIENVK.G | Y | 74.53 | 1786.9200 | 16 | 0.3 | 894.4675 | 2 | 59.25 | 5 | F5:16571 | Fraction\_5\_23092022.raw | 2.0921E5 | 2.2744E5 | 4.5763E5 | 8.1486E4 | 8.2535E4 |  |  | 5 | 1 | 1 | 1 | 1 | 1 | 0 | 0 | 12 | 27 |  |  | PEAKS DB |
| R.LIFAGKQLEDGR.T | Y | 57.40 | 1345.7354 | 12 | 0.8 | 449.5861 | 3 | 41.76 | 7 | F7:11524 | Fraction\_7\_23092022.raw |  |  | 7.085E4 |  |  | 1.6527E5 | 3.3803E5 | 3 | 0 | 0 | 1 | 0 | 0 | 1 | 1 | 43 | 54 |  |  | PEAKS DB |
| total 2 peptides |  |  |  |  |  |  |  |  |  |  |  |  |  |  |  |  |  |  |  |  |  |  |  |  |  |  |  |  |  |  |  |
| --- | --- | --- | --- | --- | --- | --- | --- | --- | --- | --- | --- | --- | --- | --- | --- | --- | --- | --- | --- | --- | --- | --- | --- | --- | --- | --- | --- | --- | --- | --- | --- |

P62976|UBIQP\_CRIGR

back to list

  

| Protein Coverage
| Supporting Peptides
|

Protein Coverage:

Supporting Peptides:

| Peptide | Uniq | -10lgP | Mass | Length | ppm | m/z | z | RT | Fraction | Scan | Source File | Area F1 | Area F2 | Area F3 | Area F4 | Area F5 | Area F6 | Area F7 | #Feature | #Feature F1 | #Feature F2 | #Feature F3 | #Feature F4 | #Feature F5 | #Feature F6 | #Feature F7 | Start | End | PTM | AScore | Found By |
| --- | --- | --- | --- | --- | --- | --- | --- | --- | --- | --- | --- | --- | --- | --- | --- | --- | --- | --- | --- | --- | --- | --- | --- | --- | --- | --- | --- | --- | --- | --- | --- |
| K.TITLEVEPSDTIENVK.A | Y | 74.53 | 1786.9200 | 16 | 0.3 | 894.4675 | 2 | 59.25 | 5 | F5:16571 | Fraction\_5\_23092022.raw | 2.0921E5 | 2.2744E5 | 4.5763E5 | 8.1486E4 | 8.2535E4 |  |  | 5 | 1 | 1 | 1 | 1 | 1 | 0 | 0 | 12 | 27 |  |  | PEAKS DB |
| R.LIFAGKQLEDGR.T | Y | 57.40 | 1345.7354 | 12 | 0.8 | 449.5861 | 3 | 41.76 | 7 | F7:11524 | Fraction\_7\_23092022.raw |  |  | 7.085E4 |  |  | 1.6527E5 | 3.3803E5 | 3 | 0 | 0 | 1 | 0 | 0 | 1 | 1 | 43 | 54 |  |  | PEAKS DB |
| total 2 peptides |  |  |  |  |  |  |  |  |  |  |  |  |  |  |  |  |  |  |  |  |  |  |  |  |  |  |  |  |  |  |  |
| --- | --- | --- | --- | --- | --- | --- | --- | --- | --- | --- | --- | --- | --- | --- | --- | --- | --- | --- | --- | --- | --- | --- | --- | --- | --- | --- | --- | --- | --- | --- | --- |

P0CG48|UBC\_HUMAN

back to list

  

| Protein Coverage
| Supporting Peptides
|

Protein Coverage:

Supporting Peptides:

| Peptide | Uniq | -10lgP | Mass | Length | ppm | m/z | z | RT | Fraction | Scan | Source File | Area F1 | Area F2 | Area F3 | Area F4 | Area F5 | Area F6 | Area F7 | #Feature | #Feature F1 | #Feature F2 | #Feature F3 | #Feature F4 | #Feature F5 | #Feature F6 | #Feature F7 | Start | End | PTM | AScore | Found By |
| --- | --- | --- | --- | --- | --- | --- | --- | --- | --- | --- | --- | --- | --- | --- | --- | --- | --- | --- | --- | --- | --- | --- | --- | --- | --- | --- | --- | --- | --- | --- | --- |
| K.TITLEVEPSDTIENVK.A | Y | 74.53 | 1786.9200 | 16 | 0.3 | 894.4675 | 2 | 59.25 | 5 | F5:16571 | Fraction\_5\_23092022.raw | 2.0921E5 | 2.2744E5 | 4.5763E5 | 8.1486E4 | 8.2535E4 |  |  | 5 | 1 | 1 | 1 | 1 | 1 | 0 | 0 | 12 | 27 |  |  | PEAKS DB |
| R.LIFAGKQLEDGR.T | Y | 57.40 | 1345.7354 | 12 | 0.8 | 449.5861 | 3 | 41.76 | 7 | F7:11524 | Fraction\_7\_23092022.raw |  |  | 7.085E4 |  |  | 1.6527E5 | 3.3803E5 | 3 | 0 | 0 | 1 | 0 | 0 | 1 | 1 | 43 | 54 |  |  | PEAKS DB |
| total 2 peptides |  |  |  |  |  |  |  |  |  |  |  |  |  |  |  |  |  |  |  |  |  |  |  |  |  |  |  |  |  |  |  |
| --- | --- | --- | --- | --- | --- | --- | --- | --- | --- | --- | --- | --- | --- | --- | --- | --- | --- | --- | --- | --- | --- | --- | --- | --- | --- | --- | --- | --- | --- | --- | --- |

P29504|RS27A\_MANSE

back to list

  

| Protein Coverage
| Supporting Peptides
|

Protein Coverage:

Supporting Peptides:

| Peptide | Uniq | -10lgP | Mass | Length | ppm | m/z | z | RT | Fraction | Scan | Source File | Area F1 | Area F2 | Area F3 | Area F4 | Area F5 | Area F6 | Area F7 | #Feature | #Feature F1 | #Feature F2 | #Feature F3 | #Feature F4 | #Feature F5 | #Feature F6 | #Feature F7 | Start | End | PTM | AScore | Found By |
| --- | --- | --- | --- | --- | --- | --- | --- | --- | --- | --- | --- | --- | --- | --- | --- | --- | --- | --- | --- | --- | --- | --- | --- | --- | --- | --- | --- | --- | --- | --- | --- |
| K.TITLEVEPSDTIENVK.A | Y | 74.53 | 1786.9200 | 16 | 0.3 | 894.4675 | 2 | 59.25 | 5 | F5:16571 | Fraction\_5\_23092022.raw | 2.0921E5 | 2.2744E5 | 4.5763E5 | 8.1486E4 | 8.2535E4 |  |  | 5 | 1 | 1 | 1 | 1 | 1 | 0 | 0 | 12 | 27 |  |  | PEAKS DB |
| R.LIFAGKQLEDGR.T | Y | 57.40 | 1345.7354 | 12 | 0.8 | 449.5861 | 3 | 41.76 | 7 | F7:11524 | Fraction\_7\_23092022.raw |  |  | 7.085E4 |  |  | 1.6527E5 | 3.3803E5 | 3 | 0 | 0 | 1 | 0 | 0 | 1 | 1 | 43 | 54 |  |  | PEAKS DB |
| total 2 peptides |  |  |  |  |  |  |  |  |  |  |  |  |  |  |  |  |  |  |  |  |  |  |  |  |  |  |  |  |  |  |  |
| --- | --- | --- | --- | --- | --- | --- | --- | --- | --- | --- | --- | --- | --- | --- | --- | --- | --- | --- | --- | --- | --- | --- | --- | --- | --- | --- | --- | --- | --- | --- | --- |

P15357|RS27A\_DROME

back to list

  

| Protein Coverage
| Supporting Peptides
|

Protein Coverage:

Supporting Peptides:

| Peptide | Uniq | -10lgP | Mass | Length | ppm | m/z | z | RT | Fraction | Scan | Source File | Area F1 | Area F2 | Area F3 | Area F4 | Area F5 | Area F6 | Area F7 | #Feature | #Feature F1 | #Feature F2 | #Feature F3 | #Feature F4 | #Feature F5 | #Feature F6 | #Feature F7 | Start | End | PTM | AScore | Found By |
| --- | --- | --- | --- | --- | --- | --- | --- | --- | --- | --- | --- | --- | --- | --- | --- | --- | --- | --- | --- | --- | --- | --- | --- | --- | --- | --- | --- | --- | --- | --- | --- |
| K.TITLEVEPSDTIENVK.A | Y | 74.53 | 1786.9200 | 16 | 0.3 | 894.4675 | 2 | 59.25 | 5 | F5:16571 | Fraction\_5\_23092022.raw | 2.0921E5 | 2.2744E5 | 4.5763E5 | 8.1486E4 | 8.2535E4 |  |  | 5 | 1 | 1 | 1 | 1 | 1 | 0 | 0 | 12 | 27 |  |  | PEAKS DB |
| R.LIFAGKQLEDGR.T | Y | 57.40 | 1345.7354 | 12 | 0.8 | 449.5861 | 3 | 41.76 | 7 | F7:11524 | Fraction\_7\_23092022.raw |  |  | 7.085E4 |  |  | 1.6527E5 | 3.3803E5 | 3 | 0 | 0 | 1 | 0 | 0 | 1 | 1 | 43 | 54 |  |  | PEAKS DB |
| total 2 peptides |  |  |  |  |  |  |  |  |  |  |  |  |  |  |  |  |  |  |  |  |  |  |  |  |  |  |  |  |  |  |  |
| --- | --- | --- | --- | --- | --- | --- | --- | --- | --- | --- | --- | --- | --- | --- | --- | --- | --- | --- | --- | --- | --- | --- | --- | --- | --- | --- | --- | --- | --- | --- | --- |

P62979|RS27A\_HUMAN

back to list

  

| Protein Coverage
| Supporting Peptides
|

Protein Coverage:

Supporting Peptides:

| Peptide | Uniq | -10lgP | Mass | Length | ppm | m/z | z | RT | Fraction | Scan | Source File | Area F1 | Area F2 | Area F3 | Area F4 | Area F5 | Area F6 | Area F7 | #Feature | #Feature F1 | #Feature F2 | #Feature F3 | #Feature F4 | #Feature F5 | #Feature F6 | #Feature F7 | Start | End | PTM | AScore | Found By |
| --- | --- | --- | --- | --- | --- | --- | --- | --- | --- | --- | --- | --- | --- | --- | --- | --- | --- | --- | --- | --- | --- | --- | --- | --- | --- | --- | --- | --- | --- | --- | --- |
| K.TITLEVEPSDTIENVK.A | Y | 74.53 | 1786.9200 | 16 | 0.3 | 894.4675 | 2 | 59.25 | 5 | F5:16571 | Fraction\_5\_23092022.raw | 2.0921E5 | 2.2744E5 | 4.5763E5 | 8.1486E4 | 8.2535E4 |  |  | 5 | 1 | 1 | 1 | 1 | 1 | 0 | 0 | 12 | 27 |  |  | PEAKS DB |
| R.LIFAGKQLEDGR.T | Y | 57.40 | 1345.7354 | 12 | 0.8 | 449.5861 | 3 | 41.76 | 7 | F7:11524 | Fraction\_7\_23092022.raw |  |  | 7.085E4 |  |  | 1.6527E5 | 3.3803E5 | 3 | 0 | 0 | 1 | 0 | 0 | 1 | 1 | 43 | 54 |  |  | PEAKS DB |
| total 2 peptides |  |  |  |  |  |  |  |  |  |  |  |  |  |  |  |  |  |  |  |  |  |  |  |  |  |  |  |  |  |  |  |
| --- | --- | --- | --- | --- | --- | --- | --- | --- | --- | --- | --- | --- | --- | --- | --- | --- | --- | --- | --- | --- | --- | --- | --- | --- | --- | --- | --- | --- | --- | --- | --- |

Q6KB66|K2C80\_HUMAN

back to list

  

| Protein Coverage
| Supporting Peptides
|

Protein Coverage:

Supporting Peptides:

| Peptide | Uniq | -10lgP | Mass | Length | ppm | m/z | z | RT | Fraction | Scan | Source File | Area F1 | Area F2 | Area F3 | Area F4 | Area F5 | Area F6 | Area F7 | #Feature | #Feature F1 | #Feature F2 | #Feature F3 | #Feature F4 | #Feature F5 | #Feature F6 | #Feature F7 | Start | End | PTM | AScore | Found By |
| --- | --- | --- | --- | --- | --- | --- | --- | --- | --- | --- | --- | --- | --- | --- | --- | --- | --- | --- | --- | --- | --- | --- | --- | --- | --- | --- | --- | --- | --- | --- | --- |
| K.LALDIEIATYR.K | N | 77.60 | 1276.7026 | 11 | 0.4 | 639.3588 | 2 | 69.92 | 5 | F5:21320 | Fraction\_5\_23092022.raw | 1.0555E7 | 3.286E6 | 4.4833E6 |  | 9.0556E5 | 1.6127E6 | 1.5171E6 | 6 | 1 | 1 | 1 | 0 | 1 | 1 | 1 | 374 | 384 |  |  | PEAKS DB |
| R.SLEEAEAYSRSQLEEQAAR.S | Y | 49.35 | 2166.0188 | 19 | 0.1 | 723.0136 | 3 | 46.13 | 3 | F3:10368 | Fraction\_3\_23092022.raw |  |  | 6.8861E4 |  |  |  |  | 1 | 0 | 0 | 1 | 0 | 0 | 0 | 0 | 266 | 284 |  |  | PEAKS DB |
| total 2 peptides |  |  |  |  |  |  |  |  |  |  |  |  |  |  |  |  |  |  |  |  |  |  |  |  |  |  |  |  |  |  |  |
| --- | --- | --- | --- | --- | --- | --- | --- | --- | --- | --- | --- | --- | --- | --- | --- | --- | --- | --- | --- | --- | --- | --- | --- | --- | --- | --- | --- | --- | --- | --- | --- |

P19620|ANXA2\_PIG

back to list

  

| Protein Coverage
| Supporting Peptides
|

Protein Coverage:

Supporting Peptides:

| Peptide | Uniq | -10lgP | Mass | Length | ppm | m/z | z | RT | Fraction | Scan | Source File | Area F1 | Area F2 | Area F3 | Area F4 | Area F5 | Area F6 | Area F7 | #Feature | #Feature F1 | #Feature F2 | #Feature F3 | #Feature F4 | #Feature F5 | #Feature F6 | #Feature F7 | Start | End | PTM | AScore | Found By |
| --- | --- | --- | --- | --- | --- | --- | --- | --- | --- | --- | --- | --- | --- | --- | --- | --- | --- | --- | --- | --- | --- | --- | --- | --- | --- | --- | --- | --- | --- | --- | --- |
| K.GLGTDEDSLIEIIC(+57.02)SR.T | Y | 67.14 | 1776.8563 | 16 | -0.6 | 889.4349 | 2 | 80.94 | 1 | F1:25390 | Fraction\_1\_23092022r.raw | 1.4108E5 |  |  |  |  |  |  | 1 | 1 | 0 | 0 | 0 | 0 | 0 | 0 | 120 | 135 | Carbamidomethylation | C14:Carbamidomethylation:1000.00 | PEAKS DB |
| K.GVDEVTIVNILTNR.S | Y | 65.97 | 1541.8413 | 14 | 1.5 | 771.9291 | 2 | 80.14 | 1 | F1:25039 | Fraction\_1\_23092022r.raw | 2.0326E5 |  | 5.1373E4 |  |  |  |  | 2 | 1 | 0 | 1 | 0 | 0 | 0 | 0 | 50 | 63 |  |  | PEAKS DB |
| total 2 peptides |  |  |  |  |  |  |  |  |  |  |  |  |  |  |  |  |  |  |  |  |  |  |  |  |  |  |  |  |  |  |  |
| --- | --- | --- | --- | --- | --- | --- | --- | --- | --- | --- | --- | --- | --- | --- | --- | --- | --- | --- | --- | --- | --- | --- | --- | --- | --- | --- | --- | --- | --- | --- | --- |

A2SW69|ANXA2\_SHEEP

back to list

  

| Protein Coverage
| Supporting Peptides
|

Protein Coverage:

Supporting Peptides:

| Peptide | Uniq | -10lgP | Mass | Length | ppm | m/z | z | RT | Fraction | Scan | Source File | Area F1 | Area F2 | Area F3 | Area F4 | Area F5 | Area F6 | Area F7 | #Feature | #Feature F1 | #Feature F2 | #Feature F3 | #Feature F4 | #Feature F5 | #Feature F6 | #Feature F7 | Start | End | PTM | AScore | Found By |
| --- | --- | --- | --- | --- | --- | --- | --- | --- | --- | --- | --- | --- | --- | --- | --- | --- | --- | --- | --- | --- | --- | --- | --- | --- | --- | --- | --- | --- | --- | --- | --- |
| K.GLGTDEDSLIEIIC(+57.02)SR.T | Y | 67.14 | 1776.8563 | 16 | -0.6 | 889.4349 | 2 | 80.94 | 1 | F1:25390 | Fraction\_1\_23092022r.raw | 1.4108E5 |  |  |  |  |  |  | 1 | 1 | 0 | 0 | 0 | 0 | 0 | 0 | 120 | 135 | Carbamidomethylation | C14:Carbamidomethylation:1000.00 | PEAKS DB |
| K.GVDEVTIVNILTNR.S | Y | 65.97 | 1541.8413 | 14 | 1.5 | 771.9291 | 2 | 80.14 | 1 | F1:25039 | Fraction\_1\_23092022r.raw | 2.0326E5 |  | 5.1373E4 |  |  |  |  | 2 | 1 | 0 | 1 | 0 | 0 | 0 | 0 | 50 | 63 |  |  | PEAKS DB |
| total 2 peptides |  |  |  |  |  |  |  |  |  |  |  |  |  |  |  |  |  |  |  |  |  |  |  |  |  |  |  |  |  |  |  |
| --- | --- | --- | --- | --- | --- | --- | --- | --- | --- | --- | --- | --- | --- | --- | --- | --- | --- | --- | --- | --- | --- | --- | --- | --- | --- | --- | --- | --- | --- | --- | --- |

P04272|ANXA2\_BOVIN

back to list

  

| Protein Coverage
| Supporting Peptides
|

Protein Coverage:

Supporting Peptides:

| Peptide | Uniq | -10lgP | Mass | Length | ppm | m/z | z | RT | Fraction | Scan | Source File | Area F1 | Area F2 | Area F3 | Area F4 | Area F5 | Area F6 | Area F7 | #Feature | #Feature F1 | #Feature F2 | #Feature F3 | #Feature F4 | #Feature F5 | #Feature F6 | #Feature F7 | Start | End | PTM | AScore | Found By |
| --- | --- | --- | --- | --- | --- | --- | --- | --- | --- | --- | --- | --- | --- | --- | --- | --- | --- | --- | --- | --- | --- | --- | --- | --- | --- | --- | --- | --- | --- | --- | --- |
| K.GLGTDEDSLIEIIC(+57.02)SR.T | Y | 67.14 | 1776.8563 | 16 | -0.6 | 889.4349 | 2 | 80.94 | 1 | F1:25390 | Fraction\_1\_23092022r.raw | 1.4108E5 |  |  |  |  |  |  | 1 | 1 | 0 | 0 | 0 | 0 | 0 | 0 | 120 | 135 | Carbamidomethylation | C14:Carbamidomethylation:1000.00 | PEAKS DB |
| K.GVDEVTIVNILTNR.S | Y | 65.97 | 1541.8413 | 14 | 1.5 | 771.9291 | 2 | 80.14 | 1 | F1:25039 | Fraction\_1\_23092022r.raw | 2.0326E5 |  | 5.1373E4 |  |  |  |  | 2 | 1 | 0 | 1 | 0 | 0 | 0 | 0 | 50 | 63 |  |  | PEAKS DB |
| total 2 peptides |  |  |  |  |  |  |  |  |  |  |  |  |  |  |  |  |  |  |  |  |  |  |  |  |  |  |  |  |  |  |  |
| --- | --- | --- | --- | --- | --- | --- | --- | --- | --- | --- | --- | --- | --- | --- | --- | --- | --- | --- | --- | --- | --- | --- | --- | --- | --- | --- | --- | --- | --- | --- | --- |

P07356|ANXA2\_MOUSE

back to list

  

| Protein Coverage
| Supporting Peptides
|

Protein Coverage:

Supporting Peptides:

| Peptide | Uniq | -10lgP | Mass | Length | ppm | m/z | z | RT | Fraction | Scan | Source File | Area F1 | Area F2 | Area F3 | Area F4 | Area F5 | Area F6 | Area F7 | #Feature | #Feature F1 | #Feature F2 | #Feature F3 | #Feature F4 | #Feature F5 | #Feature F6 | #Feature F7 | Start | End | PTM | AScore | Found By |
| --- | --- | --- | --- | --- | --- | --- | --- | --- | --- | --- | --- | --- | --- | --- | --- | --- | --- | --- | --- | --- | --- | --- | --- | --- | --- | --- | --- | --- | --- | --- | --- |
| K.GLGTDEDSLIEIIC(+57.02)SR.T | Y | 67.14 | 1776.8563 | 16 | -0.6 | 889.4349 | 2 | 80.94 | 1 | F1:25390 | Fraction\_1\_23092022r.raw | 1.4108E5 |  |  |  |  |  |  | 1 | 1 | 0 | 0 | 0 | 0 | 0 | 0 | 120 | 135 | Carbamidomethylation | C14:Carbamidomethylation:1000.00 | PEAKS DB |
| K.GVDEVTIVNILTNR.S | Y | 65.97 | 1541.8413 | 14 | 1.5 | 771.9291 | 2 | 80.14 | 1 | F1:25039 | Fraction\_1\_23092022r.raw | 2.0326E5 |  | 5.1373E4 |  |  |  |  | 2 | 1 | 0 | 1 | 0 | 0 | 0 | 0 | 50 | 63 |  |  | PEAKS DB |
| total 2 peptides |  |  |  |  |  |  |  |  |  |  |  |  |  |  |  |  |  |  |  |  |  |  |  |  |  |  |  |  |  |  |  |
| --- | --- | --- | --- | --- | --- | --- | --- | --- | --- | --- | --- | --- | --- | --- | --- | --- | --- | --- | --- | --- | --- | --- | --- | --- | --- | --- | --- | --- | --- | --- | --- |

Q6TEQ7|ANXA2\_CANLF

back to list

  

| Protein Coverage
| Supporting Peptides
|

Protein Coverage:

Supporting Peptides:

| Peptide | Uniq | -10lgP | Mass | Length | ppm | m/z | z | RT | Fraction | Scan | Source File | Area F1 | Area F2 | Area F3 | Area F4 | Area F5 | Area F6 | Area F7 | #Feature | #Feature F1 | #Feature F2 | #Feature F3 | #Feature F4 | #Feature F5 | #Feature F6 | #Feature F7 | Start | End | PTM | AScore | Found By |
| --- | --- | --- | --- | --- | --- | --- | --- | --- | --- | --- | --- | --- | --- | --- | --- | --- | --- | --- | --- | --- | --- | --- | --- | --- | --- | --- | --- | --- | --- | --- | --- |
| K.GLGTDEDSLIEIIC(+57.02)SR.T | Y | 67.14 | 1776.8563 | 16 | -0.6 | 889.4349 | 2 | 80.94 | 1 | F1:25390 | Fraction\_1\_23092022r.raw | 1.4108E5 |  |  |  |  |  |  | 1 | 1 | 0 | 0 | 0 | 0 | 0 | 0 | 120 | 135 | Carbamidomethylation | C14:Carbamidomethylation:1000.00 | PEAKS DB |
| K.GVDEVTIVNILTNR.S | Y | 65.97 | 1541.8413 | 14 | 1.5 | 771.9291 | 2 | 80.14 | 1 | F1:25039 | Fraction\_1\_23092022r.raw | 2.0326E5 |  | 5.1373E4 |  |  |  |  | 2 | 1 | 0 | 1 | 0 | 0 | 0 | 0 | 50 | 63 |  |  | PEAKS DB |
| total 2 peptides |  |  |  |  |  |  |  |  |  |  |  |  |  |  |  |  |  |  |  |  |  |  |  |  |  |  |  |  |  |  |  |
| --- | --- | --- | --- | --- | --- | --- | --- | --- | --- | --- | --- | --- | --- | --- | --- | --- | --- | --- | --- | --- | --- | --- | --- | --- | --- | --- | --- | --- | --- | --- | --- |

Q2Q1M6|ANXA2\_CEREL

back to list

  

| Protein Coverage
| Supporting Peptides
|

Protein Coverage:

Supporting Peptides:

| Peptide | Uniq | -10lgP | Mass | Length | ppm | m/z | z | RT | Fraction | Scan | Source File | Area F1 | Area F2 | Area F3 | Area F4 | Area F5 | Area F6 | Area F7 | #Feature | #Feature F1 | #Feature F2 | #Feature F3 | #Feature F4 | #Feature F5 | #Feature F6 | #Feature F7 | Start | End | PTM | AScore | Found By |
| --- | --- | --- | --- | --- | --- | --- | --- | --- | --- | --- | --- | --- | --- | --- | --- | --- | --- | --- | --- | --- | --- | --- | --- | --- | --- | --- | --- | --- | --- | --- | --- |
| K.GLGTDEDSLIEIIC(+57.02)SR.T | Y | 67.14 | 1776.8563 | 16 | -0.6 | 889.4349 | 2 | 80.94 | 1 | F1:25390 | Fraction\_1\_23092022r.raw | 1.4108E5 |  |  |  |  |  |  | 1 | 1 | 0 | 0 | 0 | 0 | 0 | 0 | 120 | 135 | Carbamidomethylation | C14:Carbamidomethylation:1000.00 | PEAKS DB |
| K.GVDEVTIVNILTNR.S | Y | 65.97 | 1541.8413 | 14 | 1.5 | 771.9291 | 2 | 80.14 | 1 | F1:25039 | Fraction\_1\_23092022r.raw | 2.0326E5 |  | 5.1373E4 |  |  |  |  | 2 | 1 | 0 | 1 | 0 | 0 | 0 | 0 | 50 | 63 |  |  | PEAKS DB |
| total 2 peptides |  |  |  |  |  |  |  |  |  |  |  |  |  |  |  |  |  |  |  |  |  |  |  |  |  |  |  |  |  |  |  |
| --- | --- | --- | --- | --- | --- | --- | --- | --- | --- | --- | --- | --- | --- | --- | --- | --- | --- | --- | --- | --- | --- | --- | --- | --- | --- | --- | --- | --- | --- | --- | --- |

C0HJG9|ANXA2\_MESAU

back to list

  

| Protein Coverage
| Supporting Peptides
|

Protein Coverage:

Supporting Peptides:

| Peptide | Uniq | -10lgP | Mass | Length | ppm | m/z | z | RT | Fraction | Scan | Source File | Area F1 | Area F2 | Area F3 | Area F4 | Area F5 | Area F6 | Area F7 | #Feature | #Feature F1 | #Feature F2 | #Feature F3 | #Feature F4 | #Feature F5 | #Feature F6 | #Feature F7 | Start | End | PTM | AScore | Found By |
| --- | --- | --- | --- | --- | --- | --- | --- | --- | --- | --- | --- | --- | --- | --- | --- | --- | --- | --- | --- | --- | --- | --- | --- | --- | --- | --- | --- | --- | --- | --- | --- |
| K.GLGTDEDSLIEIIC(+57.02)SR.T | Y | 67.14 | 1776.8563 | 16 | -0.6 | 889.4349 | 2 | 80.94 | 1 | F1:25390 | Fraction\_1\_23092022r.raw | 1.4108E5 |  |  |  |  |  |  | 1 | 1 | 0 | 0 | 0 | 0 | 0 | 0 | 80 | 95 | Carbamidomethylation | C14:Carbamidomethylation:1000.00 | PEAKS DB |
| K.GVDEVTIVNILTNR.Q | Y | 65.97 | 1541.8413 | 14 | 1.5 | 771.9291 | 2 | 80.14 | 1 | F1:25039 | Fraction\_1\_23092022r.raw | 2.0326E5 |  | 5.1373E4 |  |  |  |  | 2 | 1 | 0 | 1 | 0 | 0 | 0 | 0 | 38 | 51 |  |  | PEAKS DB |
| total 2 peptides |  |  |  |  |  |  |  |  |  |  |  |  |  |  |  |  |  |  |  |  |  |  |  |  |  |  |  |  |  |  |  |
| --- | --- | --- | --- | --- | --- | --- | --- | --- | --- | --- | --- | --- | --- | --- | --- | --- | --- | --- | --- | --- | --- | --- | --- | --- | --- | --- | --- | --- | --- | --- | --- |

Q5R5A0|ANXA2\_PONAB

back to list

  

| Protein Coverage
| Supporting Peptides
|

Protein Coverage:

Supporting Peptides:

| Peptide | Uniq | -10lgP | Mass | Length | ppm | m/z | z | RT | Fraction | Scan | Source File | Area F1 | Area F2 | Area F3 | Area F4 | Area F5 | Area F6 | Area F7 | #Feature | #Feature F1 | #Feature F2 | #Feature F3 | #Feature F4 | #Feature F5 | #Feature F6 | #Feature F7 | Start | End | PTM | AScore | Found By |
| --- | --- | --- | --- | --- | --- | --- | --- | --- | --- | --- | --- | --- | --- | --- | --- | --- | --- | --- | --- | --- | --- | --- | --- | --- | --- | --- | --- | --- | --- | --- | --- |
| K.GLGTDEDSLIEIIC(+57.02)SR.T | Y | 67.14 | 1776.8563 | 16 | -0.6 | 889.4349 | 2 | 80.94 | 1 | F1:25390 | Fraction\_1\_23092022r.raw | 1.4108E5 |  |  |  |  |  |  | 1 | 1 | 0 | 0 | 0 | 0 | 0 | 0 | 120 | 135 | Carbamidomethylation | C14:Carbamidomethylation:1000.00 | PEAKS DB |
| K.GVDEVTIVNILTNR.S | Y | 65.97 | 1541.8413 | 14 | 1.5 | 771.9291 | 2 | 80.14 | 1 | F1:25039 | Fraction\_1\_23092022r.raw | 2.0326E5 |  | 5.1373E4 |  |  |  |  | 2 | 1 | 0 | 1 | 0 | 0 | 0 | 0 | 50 | 63 |  |  | PEAKS DB |
| total 2 peptides |  |  |  |  |  |  |  |  |  |  |  |  |  |  |  |  |  |  |  |  |  |  |  |  |  |  |  |  |  |  |  |
| --- | --- | --- | --- | --- | --- | --- | --- | --- | --- | --- | --- | --- | --- | --- | --- | --- | --- | --- | --- | --- | --- | --- | --- | --- | --- | --- | --- | --- | --- | --- | --- |

P07355|ANXA2\_HUMAN

back to list

  

| Protein Coverage
| Supporting Peptides
|

Protein Coverage:

Supporting Peptides:

| Peptide | Uniq | -10lgP | Mass | Length | ppm | m/z | z | RT | Fraction | Scan | Source File | Area F1 | Area F2 | Area F3 | Area F4 | Area F5 | Area F6 | Area F7 | #Feature | #Feature F1 | #Feature F2 | #Feature F3 | #Feature F4 | #Feature F5 | #Feature F6 | #Feature F7 | Start | End | PTM | AScore | Found By |
| --- | --- | --- | --- | --- | --- | --- | --- | --- | --- | --- | --- | --- | --- | --- | --- | --- | --- | --- | --- | --- | --- | --- | --- | --- | --- | --- | --- | --- | --- | --- | --- |
| K.GLGTDEDSLIEIIC(+57.02)SR.T | Y | 67.14 | 1776.8563 | 16 | -0.6 | 889.4349 | 2 | 80.94 | 1 | F1:25390 | Fraction\_1\_23092022r.raw | 1.4108E5 |  |  |  |  |  |  | 1 | 1 | 0 | 0 | 0 | 0 | 0 | 0 | 120 | 135 | Carbamidomethylation | C14:Carbamidomethylation:1000.00 | PEAKS DB |
| K.GVDEVTIVNILTNR.S | Y | 65.97 | 1541.8413 | 14 | 1.5 | 771.9291 | 2 | 80.14 | 1 | F1:25039 | Fraction\_1\_23092022r.raw | 2.0326E5 |  | 5.1373E4 |  |  |  |  | 2 | 1 | 0 | 1 | 0 | 0 | 0 | 0 | 50 | 63 |  |  | PEAKS DB |
| total 2 peptides |  |  |  |  |  |  |  |  |  |  |  |  |  |  |  |  |  |  |  |  |  |  |  |  |  |  |  |  |  |  |  |
| --- | --- | --- | --- | --- | --- | --- | --- | --- | --- | --- | --- | --- | --- | --- | --- | --- | --- | --- | --- | --- | --- | --- | --- | --- | --- | --- | --- | --- | --- | --- | --- |

Q07936|ANXA2\_RAT

back to list

  

| Protein Coverage
| Supporting Peptides
|

Protein Coverage:

Supporting Peptides:

| Peptide | Uniq | -10lgP | Mass | Length | ppm | m/z | z | RT | Fraction | Scan | Source File | Area F1 | Area F2 | Area F3 | Area F4 | Area F5 | Area F6 | Area F7 | #Feature | #Feature F1 | #Feature F2 | #Feature F3 | #Feature F4 | #Feature F5 | #Feature F6 | #Feature F7 | Start | End | PTM | AScore | Found By |
| --- | --- | --- | --- | --- | --- | --- | --- | --- | --- | --- | --- | --- | --- | --- | --- | --- | --- | --- | --- | --- | --- | --- | --- | --- | --- | --- | --- | --- | --- | --- | --- |
| K.GLGTDEDSLIEIIC(+57.02)SR.T | Y | 67.14 | 1776.8563 | 16 | -0.6 | 889.4349 | 2 | 80.94 | 1 | F1:25390 | Fraction\_1\_23092022r.raw | 1.4108E5 |  |  |  |  |  |  | 1 | 1 | 0 | 0 | 0 | 0 | 0 | 0 | 120 | 135 | Carbamidomethylation | C14:Carbamidomethylation:1000.00 | PEAKS DB |
| K.GVDEVTIVNILTNR.S | Y | 65.97 | 1541.8413 | 14 | 1.5 | 771.9291 | 2 | 80.14 | 1 | F1:25039 | Fraction\_1\_23092022r.raw | 2.0326E5 |  | 5.1373E4 |  |  |  |  | 2 | 1 | 0 | 1 | 0 | 0 | 0 | 0 | 50 | 63 |  |  | PEAKS DB |
| total 2 peptides |  |  |  |  |  |  |  |  |  |  |  |  |  |  |  |  |  |  |  |  |  |  |  |  |  |  |  |  |  |  |  |
| --- | --- | --- | --- | --- | --- | --- | --- | --- | --- | --- | --- | --- | --- | --- | --- | --- | --- | --- | --- | --- | --- | --- | --- | --- | --- | --- | --- | --- | --- | --- | --- |

TRINITY\_DN23073\_c0\_g1\_i1.p1

back to list

  

| Protein Coverage
| Supporting Peptides
|

Protein Coverage:

Supporting Peptides:

| Peptide | Uniq | -10lgP | Mass | Length | ppm | m/z | z | RT | Fraction | Scan | Source File | Area F1 | Area F2 | Area F3 | Area F4 | Area F5 | Area F6 | Area F7 | #Feature | #Feature F1 | #Feature F2 | #Feature F3 | #Feature F4 | #Feature F5 | #Feature F6 | #Feature F7 | Start | End | PTM | AScore | Found By |
| --- | --- | --- | --- | --- | --- | --- | --- | --- | --- | --- | --- | --- | --- | --- | --- | --- | --- | --- | --- | --- | --- | --- | --- | --- | --- | --- | --- | --- | --- | --- | --- |
| K.LLNLESQKNIAGASVYK.V | Y | 76.98 | 1847.0153 | 17 | 0.7 | 924.5156 | 2 | 48.00 | 2 | F2:10802 | Fraction\_2\_23092022.raw |  | 1.9327E6 |  | 1.5115E5 | 2.1031E5 |  |  | 4 | 0 | 2 | 0 | 1 | 1 | 0 | 0 | 86 | 102 |  |  | PEAKS DB |
| total 1 peptides |  |  |  |  |  |  |  |  |  |  |  |  |  |  |  |  |  |  |  |  |  |  |  |  |  |  |  |  |  |  |  |
| --- | --- | --- | --- | --- | --- | --- | --- | --- | --- | --- | --- | --- | --- | --- | --- | --- | --- | --- | --- | --- | --- | --- | --- | --- | --- | --- | --- | --- | --- | --- | --- |

G8XQX1|OXLA\_DABRR

back to list

  

| Protein Coverage
| Supporting Peptides
|

Protein Coverage:

Supporting Peptides:

| Peptide | Uniq | -10lgP | Mass | Length | ppm | m/z | z | RT | Fraction | Scan | Source File | Area F1 | Area F2 | Area F3 | Area F4 | Area F5 | Area F6 | Area F7 | #Feature | #Feature F1 | #Feature F2 | #Feature F3 | #Feature F4 | #Feature F5 | #Feature F6 | #Feature F7 | Start | End | PTM | AScore | Found By |
| --- | --- | --- | --- | --- | --- | --- | --- | --- | --- | --- | --- | --- | --- | --- | --- | --- | --- | --- | --- | --- | --- | --- | --- | --- | --- | --- | --- | --- | --- | --- | --- |
| R.IFFAGEYTANAHGWIDSTIK.S | Y | 93.11 | 2240.0901 | 20 | 1.1 | 747.7048 | 3 | 69.01 | 7 | F7:26501 | Fraction\_7\_23092022.raw | 6.8963E6 | 5.4364E6 | 3.7554E6 | 3.4857E6 | 3.1336E6 | 2.7058E6 | 3.37E6 | 15 | 4 | 3 | 2 | 3 | 1 | 1 | 1 | 470 | 489 |  |  | PEAKS DB |
| total 1 peptides |  |  |  |  |  |  |  |  |  |  |  |  |  |  |  |  |  |  |  |  |  |  |  |  |  |  |  |  |  |  |  |
| --- | --- | --- | --- | --- | --- | --- | --- | --- | --- | --- | --- | --- | --- | --- | --- | --- | --- | --- | --- | --- | --- | --- | --- | --- | --- | --- | --- | --- | --- | --- | --- |

Q4F867|OXLA\_DABSI

back to list

  

| Protein Coverage
| Supporting Peptides
|

Protein Coverage:

Supporting Peptides:

| Peptide | Uniq | -10lgP | Mass | Length | ppm | m/z | z | RT | Fraction | Scan | Source File | Area F1 | Area F2 | Area F3 | Area F4 | Area F5 | Area F6 | Area F7 | #Feature | #Feature F1 | #Feature F2 | #Feature F3 | #Feature F4 | #Feature F5 | #Feature F6 | #Feature F7 | Start | End | PTM | AScore | Found By |
| --- | --- | --- | --- | --- | --- | --- | --- | --- | --- | --- | --- | --- | --- | --- | --- | --- | --- | --- | --- | --- | --- | --- | --- | --- | --- | --- | --- | --- | --- | --- | --- |
| R.IFFAGEYTANAHGWIDSTIK.S | Y | 93.11 | 2240.0901 | 20 | 1.1 | 747.7048 | 3 | 69.01 | 7 | F7:26501 | Fraction\_7\_23092022.raw | 6.8963E6 | 5.4364E6 | 3.7554E6 | 3.4857E6 | 3.1336E6 | 2.7058E6 | 3.37E6 | 15 | 4 | 3 | 2 | 3 | 1 | 1 | 1 | 373 | 392 |  |  | PEAKS DB |
| total 1 peptides |  |  |  |  |  |  |  |  |  |  |  |  |  |  |  |  |  |  |  |  |  |  |  |  |  |  |  |  |  |  |  |
| --- | --- | --- | --- | --- | --- | --- | --- | --- | --- | --- | --- | --- | --- | --- | --- | --- | --- | --- | --- | --- | --- | --- | --- | --- | --- | --- | --- | --- | --- | --- | --- |

P0C2D7|OXLA\_VIPBB

back to list

  

| Protein Coverage
| Supporting Peptides
|

Protein Coverage:

Supporting Peptides:

| Peptide | Uniq | -10lgP | Mass | Length | ppm | m/z | z | RT | Fraction | Scan | Source File | Area F1 | Area F2 | Area F3 | Area F4 | Area F5 | Area F6 | Area F7 | #Feature | #Feature F1 | #Feature F2 | #Feature F3 | #Feature F4 | #Feature F5 | #Feature F6 | #Feature F7 | Start | End | PTM | AScore | Found By |
| --- | --- | --- | --- | --- | --- | --- | --- | --- | --- | --- | --- | --- | --- | --- | --- | --- | --- | --- | --- | --- | --- | --- | --- | --- | --- | --- | --- | --- | --- | --- | --- |
| K.IFFAGEYTANAHGWIDSTIK | Y | 93.11 | 2240.0901 | 20 | 1.1 | 747.7048 | 3 | 69.01 | 7 | F7:26501 | Fraction\_7\_23092022.raw | 6.8963E6 | 5.4364E6 | 3.7554E6 | 3.4857E6 | 3.1336E6 | 2.7058E6 | 3.37E6 | 15 | 4 | 3 | 2 | 3 | 1 | 1 | 1 | 69 | 88 |  |  | PEAKS DB |
| total 1 peptides |  |  |  |  |  |  |  |  |  |  |  |  |  |  |  |  |  |  |  |  |  |  |  |  |  |  |  |  |  |  |  |
| --- | --- | --- | --- | --- | --- | --- | --- | --- | --- | --- | --- | --- | --- | --- | --- | --- | --- | --- | --- | --- | --- | --- | --- | --- | --- | --- | --- | --- | --- | --- | --- |

TRINITY\_DN6873\_c0\_g1\_i3.p1

back to list

  

| Protein Coverage
| Supporting Peptides
|

Protein Coverage:

Supporting Peptides:

| Peptide | Uniq | -10lgP | Mass | Length | ppm | m/z | z | RT | Fraction | Scan | Source File | Area F1 | Area F2 | Area F3 | Area F4 | Area F5 | Area F6 | Area F7 | #Feature | #Feature F1 | #Feature F2 | #Feature F3 | #Feature F4 | #Feature F5 | #Feature F6 | #Feature F7 | Start | End | PTM | AScore | Found By |
| --- | --- | --- | --- | --- | --- | --- | --- | --- | --- | --- | --- | --- | --- | --- | --- | --- | --- | --- | --- | --- | --- | --- | --- | --- | --- | --- | --- | --- | --- | --- | --- |
| R.DKPTEDVVISNSGSETVTEPFGVSRDDATE | Y | 92.69 | 3180.4583 | 30 | 0.6 | 1061.1606 | 3 | 56.92 | 5 | F5:15526 | Fraction\_5\_23092022.raw |  |  |  |  | 1.1402E6 | 1.4355E6 |  | 2 | 0 | 0 | 0 | 0 | 1 | 1 | 0 | 91 | 120 |  |  | PEAKS DB |
| total 1 peptides |  |  |  |  |  |  |  |  |  |  |  |  |  |  |  |  |  |  |  |  |  |  |  |  |  |  |  |  |  |  |  |
| --- | --- | --- | --- | --- | --- | --- | --- | --- | --- | --- | --- | --- | --- | --- | --- | --- | --- | --- | --- | --- | --- | --- | --- | --- | --- | --- | --- | --- | --- | --- | --- |

TRINITY\_DN6873\_c0\_g1\_i2.p1

back to list

  

| Protein Coverage
| Supporting Peptides
|

Protein Coverage:

Supporting Peptides:

| Peptide | Uniq | -10lgP | Mass | Length | ppm | m/z | z | RT | Fraction | Scan | Source File | Area F1 | Area F2 | Area F3 | Area F4 | Area F5 | Area F6 | Area F7 | #Feature | #Feature F1 | #Feature F2 | #Feature F3 | #Feature F4 | #Feature F5 | #Feature F6 | #Feature F7 | Start | End | PTM | AScore | Found By |
| --- | --- | --- | --- | --- | --- | --- | --- | --- | --- | --- | --- | --- | --- | --- | --- | --- | --- | --- | --- | --- | --- | --- | --- | --- | --- | --- | --- | --- | --- | --- | --- |
| R.DKPTEDVVISNSGSETVTEPFGVSRDDATE | Y | 92.69 | 3180.4583 | 30 | 0.6 | 1061.1606 | 3 | 56.92 | 5 | F5:15526 | Fraction\_5\_23092022.raw |  |  |  |  | 1.1402E6 | 1.4355E6 |  | 2 | 0 | 0 | 0 | 0 | 1 | 1 | 0 | 219 | 248 |  |  | PEAKS DB |
| total 1 peptides |  |  |  |  |  |  |  |  |  |  |  |  |  |  |  |  |  |  |  |  |  |  |  |  |  |  |  |  |  |  |  |
| --- | --- | --- | --- | --- | --- | --- | --- | --- | --- | --- | --- | --- | --- | --- | --- | --- | --- | --- | --- | --- | --- | --- | --- | --- | --- | --- | --- | --- | --- | --- | --- |

TRINITY\_DN11796\_c0\_g1\_i1.p1

back to list

  

| Protein Coverage
| Supporting Peptides
|

Protein Coverage:

Supporting Peptides:

| Peptide | Uniq | -10lgP | Mass | Length | ppm | m/z | z | RT | Fraction | Scan | Source File | Area F1 | Area F2 | Area F3 | Area F4 | Area F5 | Area F6 | Area F7 | #Feature | #Feature F1 | #Feature F2 | #Feature F3 | #Feature F4 | #Feature F5 | #Feature F6 | #Feature F7 | Start | End | PTM | AScore | Found By |
| --- | --- | --- | --- | --- | --- | --- | --- | --- | --- | --- | --- | --- | --- | --- | --- | --- | --- | --- | --- | --- | --- | --- | --- | --- | --- | --- | --- | --- | --- | --- | --- |
| R.YIATSKFEPTYAR.R | Y | 69.62 | 1545.7827 | 13 | 0.1 | 516.2682 | 3 | 38.21 | 6 | F6:7752 | Fraction\_6\_23092022.raw |  |  |  |  |  | 2.7447E5 |  | 1 | 0 | 0 | 0 | 0 | 0 | 1 | 0 | 224 | 236 |  |  | PEAKS DB |
| total 1 peptides |  |  |  |  |  |  |  |  |  |  |  |  |  |  |  |  |  |  |  |  |  |  |  |  |  |  |  |  |  |  |  |
| --- | --- | --- | --- | --- | --- | --- | --- | --- | --- | --- | --- | --- | --- | --- | --- | --- | --- | --- | --- | --- | --- | --- | --- | --- | --- | --- | --- | --- | --- | --- | --- |

TRINITY\_DN126\_c0\_g1\_i17.p1

back to list

  

| Protein Coverage
| Supporting Peptides
|

Protein Coverage:

Supporting Peptides:

| Peptide | Uniq | -10lgP | Mass | Length | ppm | m/z | z | RT | Fraction | Scan | Source File | Area F1 | Area F2 | Area F3 | Area F4 | Area F5 | Area F6 | Area F7 | #Feature | #Feature F1 | #Feature F2 | #Feature F3 | #Feature F4 | #Feature F5 | #Feature F6 | #Feature F7 | Start | End | PTM | AScore | Found By |
| --- | --- | --- | --- | --- | --- | --- | --- | --- | --- | --- | --- | --- | --- | --- | --- | --- | --- | --- | --- | --- | --- | --- | --- | --- | --- | --- | --- | --- | --- | --- | --- |
| R.YENAEEIDPEGEISYERPPSYLPGSQVNALNK.G | Y | 50.20 | 3607.6953 | 32 | -0.8 | 1203.5714 | 3 | 63.16 | 1 | F1:17499 | Fraction\_1\_23092022r.raw | 5.21E5 |  |  |  |  |  |  | 1 | 1 | 0 | 0 | 0 | 0 | 0 | 0 | 375 | 406 |  |  | PEAKS DB |
| R.DGLYDFYQVR.D | Y | 48.72 | 1274.5931 | 10 | 0.8 | 638.3044 | 2 | 67.94 | 1 | F1:19571 | Fraction\_1\_23092022r.raw | 1.8168E5 |  |  |  |  |  |  | 1 | 1 | 0 | 0 | 0 | 0 | 0 | 0 | 454 | 463 |  |  | PEAKS DB |
| total 2 peptides |  |  |  |  |  |  |  |  |  |  |  |  |  |  |  |  |  |  |  |  |  |  |  |  |  |  |  |  |  |  |  |
| --- | --- | --- | --- | --- | --- | --- | --- | --- | --- | --- | --- | --- | --- | --- | --- | --- | --- | --- | --- | --- | --- | --- | --- | --- | --- | --- | --- | --- | --- | --- | --- |

TRINITY\_DN126\_c0\_g1\_i12.p1

back to list

  

| Protein Coverage
| Supporting Peptides
|

Protein Coverage:

Supporting Peptides:

| Peptide | Uniq | -10lgP | Mass | Length | ppm | m/z | z | RT | Fraction | Scan | Source File | Area F1 | Area F2 | Area F3 | Area F4 | Area F5 | Area F6 | Area F7 | #Feature | #Feature F1 | #Feature F2 | #Feature F3 | #Feature F4 | #Feature F5 | #Feature F6 | #Feature F7 | Start | End | PTM | AScore | Found By |
| --- | --- | --- | --- | --- | --- | --- | --- | --- | --- | --- | --- | --- | --- | --- | --- | --- | --- | --- | --- | --- | --- | --- | --- | --- | --- | --- | --- | --- | --- | --- | --- |
| R.YENAEEIDPEGEISYERPPSYLPGSQVNALNK.G | Y | 50.20 | 3607.6953 | 32 | -0.8 | 1203.5714 | 3 | 63.16 | 1 | F1:17499 | Fraction\_1\_23092022r.raw | 5.21E5 |  |  |  |  |  |  | 1 | 1 | 0 | 0 | 0 | 0 | 0 | 0 | 375 | 406 |  |  | PEAKS DB |
| R.DGLYDFYQVR.D | Y | 48.72 | 1274.5931 | 10 | 0.8 | 638.3044 | 2 | 67.94 | 1 | F1:19571 | Fraction\_1\_23092022r.raw | 1.8168E5 |  |  |  |  |  |  | 1 | 1 | 0 | 0 | 0 | 0 | 0 | 0 | 454 | 463 |  |  | PEAKS DB |
| total 2 peptides |  |  |  |  |  |  |  |  |  |  |  |  |  |  |  |  |  |  |  |  |  |  |  |  |  |  |  |  |  |  |  |
| --- | --- | --- | --- | --- | --- | --- | --- | --- | --- | --- | --- | --- | --- | --- | --- | --- | --- | --- | --- | --- | --- | --- | --- | --- | --- | --- | --- | --- | --- | --- | --- |

TRINITY\_DN126\_c0\_g1\_i25.p1

back to list

  

| Protein Coverage
| Supporting Peptides
|

Protein Coverage:

Supporting Peptides:

| Peptide | Uniq | -10lgP | Mass | Length | ppm | m/z | z | RT | Fraction | Scan | Source File | Area F1 | Area F2 | Area F3 | Area F4 | Area F5 | Area F6 | Area F7 | #Feature | #Feature F1 | #Feature F2 | #Feature F3 | #Feature F4 | #Feature F5 | #Feature F6 | #Feature F7 | Start | End | PTM | AScore | Found By |
| --- | --- | --- | --- | --- | --- | --- | --- | --- | --- | --- | --- | --- | --- | --- | --- | --- | --- | --- | --- | --- | --- | --- | --- | --- | --- | --- | --- | --- | --- | --- | --- |
| R.YENAEEIDPEGEISYERPPSYLPGSQVNALNK.G | Y | 50.20 | 3607.6953 | 32 | -0.8 | 1203.5714 | 3 | 63.16 | 1 | F1:17499 | Fraction\_1\_23092022r.raw | 5.21E5 |  |  |  |  |  |  | 1 | 1 | 0 | 0 | 0 | 0 | 0 | 0 | 375 | 406 |  |  | PEAKS DB |
| R.DGLYDFYQVR.D | Y | 48.72 | 1274.5931 | 10 | 0.8 | 638.3044 | 2 | 67.94 | 1 | F1:19571 | Fraction\_1\_23092022r.raw | 1.8168E5 |  |  |  |  |  |  | 1 | 1 | 0 | 0 | 0 | 0 | 0 | 0 | 454 | 463 |  |  | PEAKS DB |
| total 2 peptides |  |  |  |  |  |  |  |  |  |  |  |  |  |  |  |  |  |  |  |  |  |  |  |  |  |  |  |  |  |  |  |
| --- | --- | --- | --- | --- | --- | --- | --- | --- | --- | --- | --- | --- | --- | --- | --- | --- | --- | --- | --- | --- | --- | --- | --- | --- | --- | --- | --- | --- | --- | --- | --- |

Q8N1N4|K2C78\_HUMAN

back to list

  

| Protein Coverage
| Supporting Peptides
|

Protein Coverage:

Supporting Peptides:

| Peptide | Uniq | -10lgP | Mass | Length | ppm | m/z | z | RT | Fraction | Scan | Source File | Area F1 | Area F2 | Area F3 | Area F4 | Area F5 | Area F6 | Area F7 | #Feature | #Feature F1 | #Feature F2 | #Feature F3 | #Feature F4 | #Feature F5 | #Feature F6 | #Feature F7 | Start | End | PTM | AScore | Found By |
| --- | --- | --- | --- | --- | --- | --- | --- | --- | --- | --- | --- | --- | --- | --- | --- | --- | --- | --- | --- | --- | --- | --- | --- | --- | --- | --- | --- | --- | --- | --- | --- |
| R.TLNNQFASFIDKVR.F | Y | 55.33 | 1651.8682 | 14 | -0.7 | 551.6296 | 3 | 64.32 | 1 | F1:17972 | Fraction\_1\_23092022r.raw | 1.7056E5 |  |  |  |  |  |  | 1 | 1 | 0 | 0 | 0 | 0 | 0 | 0 | 117 | 130 |  |  | PEAKS DB |
| total 1 peptides |  |  |  |  |  |  |  |  |  |  |  |  |  |  |  |  |  |  |  |  |  |  |  |  |  |  |  |  |  |  |  |
| --- | --- | --- | --- | --- | --- | --- | --- | --- | --- | --- | --- | --- | --- | --- | --- | --- | --- | --- | --- | --- | --- | --- | --- | --- | --- | --- | --- | --- | --- | --- | --- |

TRINITY\_DN7919\_c0\_g1\_i8.p1

back to list

  

| Protein Coverage
| Supporting Peptides
|

Protein Coverage:

Supporting Peptides:

| Peptide | Uniq | -10lgP | Mass | Length | ppm | m/z | z | RT | Fraction | Scan | Source File | Area F1 | Area F2 | Area F3 | Area F4 | Area F5 | Area F6 | Area F7 | #Feature | #Feature F1 | #Feature F2 | #Feature F3 | #Feature F4 | #Feature F5 | #Feature F6 | #Feature F7 | Start | End | PTM | AScore | Found By |
| --- | --- | --- | --- | --- | --- | --- | --- | --- | --- | --- | --- | --- | --- | --- | --- | --- | --- | --- | --- | --- | --- | --- | --- | --- | --- | --- | --- | --- | --- | --- | --- |
| R.LLLPNYADGIQAPTR.S | Y | 76.98 | 1640.8885 | 15 | 0.6 | 821.4520 | 2 | 63.74 | 6 | F6:19218 | Fraction\_6\_23092022.raw |  |  | 8.2942E4 |  |  | 3.1285E6 | 2.1533E6 | 4 | 0 | 0 | 1 | 0 | 0 | 2 | 1 | 133 | 147 |  |  | PEAKS DB |
| total 1 peptides |  |  |  |  |  |  |  |  |  |  |  |  |  |  |  |  |  |  |  |  |  |  |  |  |  |  |  |  |  |  |  |
| --- | --- | --- | --- | --- | --- | --- | --- | --- | --- | --- | --- | --- | --- | --- | --- | --- | --- | --- | --- | --- | --- | --- | --- | --- | --- | --- | --- | --- | --- | --- | --- |

TRINITY\_DN7919\_c0\_g1\_i2.p1

back to list

  

| Protein Coverage
| Supporting Peptides
|

Protein Coverage:

Supporting Peptides:

| Peptide | Uniq | -10lgP | Mass | Length | ppm | m/z | z | RT | Fraction | Scan | Source File | Area F1 | Area F2 | Area F3 | Area F4 | Area F5 | Area F6 | Area F7 | #Feature | #Feature F1 | #Feature F2 | #Feature F3 | #Feature F4 | #Feature F5 | #Feature F6 | #Feature F7 | Start | End | PTM | AScore | Found By |
| --- | --- | --- | --- | --- | --- | --- | --- | --- | --- | --- | --- | --- | --- | --- | --- | --- | --- | --- | --- | --- | --- | --- | --- | --- | --- | --- | --- | --- | --- | --- | --- |
| R.LLLPNYADGIQAPTR.S | Y | 76.98 | 1640.8885 | 15 | 0.6 | 821.4520 | 2 | 63.74 | 6 | F6:19218 | Fraction\_6\_23092022.raw |  |  | 8.2942E4 |  |  | 3.1285E6 | 2.1533E6 | 4 | 0 | 0 | 1 | 0 | 0 | 2 | 1 | 133 | 147 |  |  | PEAKS DB |
| total 1 peptides |  |  |  |  |  |  |  |  |  |  |  |  |  |  |  |  |  |  |  |  |  |  |  |  |  |  |  |  |  |  |  |
| --- | --- | --- | --- | --- | --- | --- | --- | --- | --- | --- | --- | --- | --- | --- | --- | --- | --- | --- | --- | --- | --- | --- | --- | --- | --- | --- | --- | --- | --- | --- | --- |

TRINITY\_DN7919\_c0\_g1\_i4.p1

back to list

  

| Protein Coverage
| Supporting Peptides
|

Protein Coverage:

Supporting Peptides:

| Peptide | Uniq | -10lgP | Mass | Length | ppm | m/z | z | RT | Fraction | Scan | Source File | Area F1 | Area F2 | Area F3 | Area F4 | Area F5 | Area F6 | Area F7 | #Feature | #Feature F1 | #Feature F2 | #Feature F3 | #Feature F4 | #Feature F5 | #Feature F6 | #Feature F7 | Start | End | PTM | AScore | Found By |
| --- | --- | --- | --- | --- | --- | --- | --- | --- | --- | --- | --- | --- | --- | --- | --- | --- | --- | --- | --- | --- | --- | --- | --- | --- | --- | --- | --- | --- | --- | --- | --- |
| R.LLLPNYADGIQAPTR.S | Y | 76.98 | 1640.8885 | 15 | 0.6 | 821.4520 | 2 | 63.74 | 6 | F6:19218 | Fraction\_6\_23092022.raw |  |  | 8.2942E4 |  |  | 3.1285E6 | 2.1533E6 | 4 | 0 | 0 | 1 | 0 | 0 | 2 | 1 | 133 | 147 |  |  | PEAKS DB |
| total 1 peptides |  |  |  |  |  |  |  |  |  |  |  |  |  |  |  |  |  |  |  |  |  |  |  |  |  |  |  |  |  |  |  |
| --- | --- | --- | --- | --- | --- | --- | --- | --- | --- | --- | --- | --- | --- | --- | --- | --- | --- | --- | --- | --- | --- | --- | --- | --- | --- | --- | --- | --- | --- | --- | --- |

Q0TMN0|EFTU\_CLOP1

back to list

  

| Protein Coverage
| Supporting Peptides
|

Protein Coverage:

Supporting Peptides:

| Peptide | Uniq | -10lgP | Mass | Length | ppm | m/z | z | RT | Fraction | Scan | Source File | Area F1 | Area F2 | Area F3 | Area F4 | Area F5 | Area F6 | Area F7 | #Feature | #Feature F1 | #Feature F2 | #Feature F3 | #Feature F4 | #Feature F5 | #Feature F6 | #Feature F7 | Start | End | PTM | AScore | Found By |
| --- | --- | --- | --- | --- | --- | --- | --- | --- | --- | --- | --- | --- | --- | --- | --- | --- | --- | --- | --- | --- | --- | --- | --- | --- | --- | --- | --- | --- | --- | --- | --- |
| K.LLDEAQAGDNIGALLR.G | Y | 76.92 | 1667.8842 | 16 | 0.6 | 834.9498 | 2 | 69.72 | 3 | F3:20923 | Fraction\_3\_23092022.raw |  |  | 1.6189E5 |  |  |  |  | 1 | 0 | 0 | 1 | 0 | 0 | 0 | 0 | 268 | 283 |  |  | PEAKS DB |
| total 1 peptides |  |  |  |  |  |  |  |  |  |  |  |  |  |  |  |  |  |  |  |  |  |  |  |  |  |  |  |  |  |  |  |
| --- | --- | --- | --- | --- | --- | --- | --- | --- | --- | --- | --- | --- | --- | --- | --- | --- | --- | --- | --- | --- | --- | --- | --- | --- | --- | --- | --- | --- | --- | --- | --- |

Q8XFP8|EFTU\_CLOPE

back to list

  

| Protein Coverage
| Supporting Peptides
|

Protein Coverage:

Supporting Peptides:

| Peptide | Uniq | -10lgP | Mass | Length | ppm | m/z | z | RT | Fraction | Scan | Source File | Area F1 | Area F2 | Area F3 | Area F4 | Area F5 | Area F6 | Area F7 | #Feature | #Feature F1 | #Feature F2 | #Feature F3 | #Feature F4 | #Feature F5 | #Feature F6 | #Feature F7 | Start | End | PTM | AScore | Found By |
| --- | --- | --- | --- | --- | --- | --- | --- | --- | --- | --- | --- | --- | --- | --- | --- | --- | --- | --- | --- | --- | --- | --- | --- | --- | --- | --- | --- | --- | --- | --- | --- |
| K.LLDEAQAGDNIGALLR.G | Y | 76.92 | 1667.8842 | 16 | 0.6 | 834.9498 | 2 | 69.72 | 3 | F3:20923 | Fraction\_3\_23092022.raw |  |  | 1.6189E5 |  |  |  |  | 1 | 0 | 0 | 1 | 0 | 0 | 0 | 0 | 268 | 283 |  |  | PEAKS DB |
| total 1 peptides |  |  |  |  |  |  |  |  |  |  |  |  |  |  |  |  |  |  |  |  |  |  |  |  |  |  |  |  |  |  |  |
| --- | --- | --- | --- | --- | --- | --- | --- | --- | --- | --- | --- | --- | --- | --- | --- | --- | --- | --- | --- | --- | --- | --- | --- | --- | --- | --- | --- | --- | --- | --- | --- |

Q0SQC8|EFTU\_CLOPS

back to list

  

| Protein Coverage
| Supporting Peptides
|

Protein Coverage:

Supporting Peptides:

| Peptide | Uniq | -10lgP | Mass | Length | ppm | m/z | z | RT | Fraction | Scan | Source File | Area F1 | Area F2 | Area F3 | Area F4 | Area F5 | Area F6 | Area F7 | #Feature | #Feature F1 | #Feature F2 | #Feature F3 | #Feature F4 | #Feature F5 | #Feature F6 | #Feature F7 | Start | End | PTM | AScore | Found By |
| --- | --- | --- | --- | --- | --- | --- | --- | --- | --- | --- | --- | --- | --- | --- | --- | --- | --- | --- | --- | --- | --- | --- | --- | --- | --- | --- | --- | --- | --- | --- | --- |
| K.LLDEAQAGDNIGALLR.G | Y | 76.92 | 1667.8842 | 16 | 0.6 | 834.9498 | 2 | 69.72 | 3 | F3:20923 | Fraction\_3\_23092022.raw |  |  | 1.6189E5 |  |  |  |  | 1 | 0 | 0 | 1 | 0 | 0 | 0 | 0 | 268 | 283 |  |  | PEAKS DB |
| total 1 peptides |  |  |  |  |  |  |  |  |  |  |  |  |  |  |  |  |  |  |  |  |  |  |  |  |  |  |  |  |  |  |  |
| --- | --- | --- | --- | --- | --- | --- | --- | --- | --- | --- | --- | --- | --- | --- | --- | --- | --- | --- | --- | --- | --- | --- | --- | --- | --- | --- | --- | --- | --- | --- | --- |

Q877L9|EFTU\_CLOTE

back to list

  

| Protein Coverage
| Supporting Peptides
|

Protein Coverage:

Supporting Peptides:

| Peptide | Uniq | -10lgP | Mass | Length | ppm | m/z | z | RT | Fraction | Scan | Source File | Area F1 | Area F2 | Area F3 | Area F4 | Area F5 | Area F6 | Area F7 | #Feature | #Feature F1 | #Feature F2 | #Feature F3 | #Feature F4 | #Feature F5 | #Feature F6 | #Feature F7 | Start | End | PTM | AScore | Found By |
| --- | --- | --- | --- | --- | --- | --- | --- | --- | --- | --- | --- | --- | --- | --- | --- | --- | --- | --- | --- | --- | --- | --- | --- | --- | --- | --- | --- | --- | --- | --- | --- |
| K.LLDEAQAGDNIGALLR.G | Y | 76.92 | 1667.8842 | 16 | 0.6 | 834.9498 | 2 | 69.72 | 3 | F3:20923 | Fraction\_3\_23092022.raw |  |  | 1.6189E5 |  |  |  |  | 1 | 0 | 0 | 1 | 0 | 0 | 0 | 0 | 268 | 283 |  |  | PEAKS DB |
| total 1 peptides |  |  |  |  |  |  |  |  |  |  |  |  |  |  |  |  |  |  |  |  |  |  |  |  |  |  |  |  |  |  |  |
| --- | --- | --- | --- | --- | --- | --- | --- | --- | --- | --- | --- | --- | --- | --- | --- | --- | --- | --- | --- | --- | --- | --- | --- | --- | --- | --- | --- | --- | --- | --- | --- |

A6LPP6|EFTU\_CLOB8

back to list

  

| Protein Coverage
| Supporting Peptides
|

Protein Coverage:

Supporting Peptides:

| Peptide | Uniq | -10lgP | Mass | Length | ppm | m/z | z | RT | Fraction | Scan | Source File | Area F1 | Area F2 | Area F3 | Area F4 | Area F5 | Area F6 | Area F7 | #Feature | #Feature F1 | #Feature F2 | #Feature F3 | #Feature F4 | #Feature F5 | #Feature F6 | #Feature F7 | Start | End | PTM | AScore | Found By |
| --- | --- | --- | --- | --- | --- | --- | --- | --- | --- | --- | --- | --- | --- | --- | --- | --- | --- | --- | --- | --- | --- | --- | --- | --- | --- | --- | --- | --- | --- | --- | --- |
| K.LLDEAQAGDNIGALLR.G | Y | 76.92 | 1667.8842 | 16 | 0.6 | 834.9498 | 2 | 69.72 | 3 | F3:20923 | Fraction\_3\_23092022.raw |  |  | 1.6189E5 |  |  |  |  | 1 | 0 | 0 | 1 | 0 | 0 | 0 | 0 | 268 | 283 |  |  | PEAKS DB |
| total 1 peptides |  |  |  |  |  |  |  |  |  |  |  |  |  |  |  |  |  |  |  |  |  |  |  |  |  |  |  |  |  |  |  |
| --- | --- | --- | --- | --- | --- | --- | --- | --- | --- | --- | --- | --- | --- | --- | --- | --- | --- | --- | --- | --- | --- | --- | --- | --- | --- | --- | --- | --- | --- | --- | --- |

A9KRZ4|EFTU\_LACP7

back to list

  

| Protein Coverage
| Supporting Peptides
|

Protein Coverage:

Supporting Peptides:

| Peptide | Uniq | -10lgP | Mass | Length | ppm | m/z | z | RT | Fraction | Scan | Source File | Area F1 | Area F2 | Area F3 | Area F4 | Area F5 | Area F6 | Area F7 | #Feature | #Feature F1 | #Feature F2 | #Feature F3 | #Feature F4 | #Feature F5 | #Feature F6 | #Feature F7 | Start | End | PTM | AScore | Found By |
| --- | --- | --- | --- | --- | --- | --- | --- | --- | --- | --- | --- | --- | --- | --- | --- | --- | --- | --- | --- | --- | --- | --- | --- | --- | --- | --- | --- | --- | --- | --- | --- |
| K.LLDEAQAGDNIGALLR.G | Y | 76.92 | 1667.8842 | 16 | 0.6 | 834.9498 | 2 | 69.72 | 3 | F3:20923 | Fraction\_3\_23092022.raw |  |  | 1.6189E5 |  |  |  |  | 1 | 0 | 0 | 1 | 0 | 0 | 0 | 0 | 268 | 283 |  |  | PEAKS DB |
| total 1 peptides |  |  |  |  |  |  |  |  |  |  |  |  |  |  |  |  |  |  |  |  |  |  |  |  |  |  |  |  |  |  |  |
| --- | --- | --- | --- | --- | --- | --- | --- | --- | --- | --- | --- | --- | --- | --- | --- | --- | --- | --- | --- | --- | --- | --- | --- | --- | --- | --- | --- | --- | --- | --- | --- |

P05109|S10A8\_HUMAN

back to list

  

| Protein Coverage
| Supporting Peptides
|

Protein Coverage:

Supporting Peptides:

| Peptide | Uniq | -10lgP | Mass | Length | ppm | m/z | z | RT | Fraction | Scan | Source File | Area F1 | Area F2 | Area F3 | Area F4 | Area F5 | Area F6 | Area F7 | #Feature | #Feature F1 | #Feature F2 | #Feature F3 | #Feature F4 | #Feature F5 | #Feature F6 | #Feature F7 | Start | End | PTM | AScore | Found By |
| --- | --- | --- | --- | --- | --- | --- | --- | --- | --- | --- | --- | --- | --- | --- | --- | --- | --- | --- | --- | --- | --- | --- | --- | --- | --- | --- | --- | --- | --- | --- | --- |
| K.LLETEC(+57.02)PQYIR.K | Y | 76.32 | 1420.7020 | 11 | -0.4 | 711.3580 | 2 | 46.20 | 5 | F5:10710 | Fraction\_5\_23092022.raw | 5.59E5 | 2.3619E5 | 1.3896E5 | 7.7215E4 | 4.4886E4 | 7.7496E4 |  | 6 | 1 | 1 | 1 | 1 | 1 | 1 | 0 | 37 | 47 | Carbamidomethylation | C6:Carbamidomethylation:1000.00 | PEAKS DB |
| total 1 peptides |  |  |  |  |  |  |  |  |  |  |  |  |  |  |  |  |  |  |  |  |  |  |  |  |  |  |  |  |  |  |  |
| --- | --- | --- | --- | --- | --- | --- | --- | --- | --- | --- | --- | --- | --- | --- | --- | --- | --- | --- | --- | --- | --- | --- | --- | --- | --- | --- | --- | --- | --- | --- | --- |

TRINITY\_DN1736\_c0\_g1\_i5.p1

back to list

  

| Protein Coverage
| Supporting Peptides
|

Protein Coverage:

Supporting Peptides:

| Peptide | Uniq | -10lgP | Mass | Length | ppm | m/z | z | RT | Fraction | Scan | Source File | Area F1 | Area F2 | Area F3 | Area F4 | Area F5 | Area F6 | Area F7 | #Feature | #Feature F1 | #Feature F2 | #Feature F3 | #Feature F4 | #Feature F5 | #Feature F6 | #Feature F7 | Start | End | PTM | AScore | Found By |
| --- | --- | --- | --- | --- | --- | --- | --- | --- | --- | --- | --- | --- | --- | --- | --- | --- | --- | --- | --- | --- | --- | --- | --- | --- | --- | --- | --- | --- | --- | --- | --- |
| R.QIAAYDYNPLSGTISNKR.I | Y | 74.55 | 2010.0170 | 18 | -0.7 | 671.0125 | 3 | 52.72 | 4 | F4:13863 | Fraction\_4\_23092022.raw |  |  | 1.3568E5 | 1.6835E5 |  |  |  | 2 | 0 | 0 | 1 | 1 | 0 | 0 | 0 | 56 | 73 |  |  | PEAKS DB |
| total 1 peptides |  |  |  |  |  |  |  |  |  |  |  |  |  |  |  |  |  |  |  |  |  |  |  |  |  |  |  |  |  |  |  |
| --- | --- | --- | --- | --- | --- | --- | --- | --- | --- | --- | --- | --- | --- | --- | --- | --- | --- | --- | --- | --- | --- | --- | --- | --- | --- | --- | --- | --- | --- | --- | --- |

TRINITY\_DN1736\_c0\_g1\_i25.p1

back to list

  

| Protein Coverage
| Supporting Peptides
|

Protein Coverage:

Supporting Peptides:

| Peptide | Uniq | -10lgP | Mass | Length | ppm | m/z | z | RT | Fraction | Scan | Source File | Area F1 | Area F2 | Area F3 | Area F4 | Area F5 | Area F6 | Area F7 | #Feature | #Feature F1 | #Feature F2 | #Feature F3 | #Feature F4 | #Feature F5 | #Feature F6 | #Feature F7 | Start | End | PTM | AScore | Found By |
| --- | --- | --- | --- | --- | --- | --- | --- | --- | --- | --- | --- | --- | --- | --- | --- | --- | --- | --- | --- | --- | --- | --- | --- | --- | --- | --- | --- | --- | --- | --- | --- |
| R.QIAAYDYNPLSGTISNKR.I | Y | 74.55 | 2010.0170 | 18 | -0.7 | 671.0125 | 3 | 52.72 | 4 | F4:13863 | Fraction\_4\_23092022.raw |  |  | 1.3568E5 | 1.6835E5 |  |  |  | 2 | 0 | 0 | 1 | 1 | 0 | 0 | 0 | 56 | 73 |  |  | PEAKS DB |
| total 1 peptides |  |  |  |  |  |  |  |  |  |  |  |  |  |  |  |  |  |  |  |  |  |  |  |  |  |  |  |  |  |  |  |
| --- | --- | --- | --- | --- | --- | --- | --- | --- | --- | --- | --- | --- | --- | --- | --- | --- | --- | --- | --- | --- | --- | --- | --- | --- | --- | --- | --- | --- | --- | --- | --- |

TRINITY\_DN1736\_c0\_g1\_i30.p1

back to list

  

| Protein Coverage
| Supporting Peptides
|

Protein Coverage:

Supporting Peptides:

| Peptide | Uniq | -10lgP | Mass | Length | ppm | m/z | z | RT | Fraction | Scan | Source File | Area F1 | Area F2 | Area F3 | Area F4 | Area F5 | Area F6 | Area F7 | #Feature | #Feature F1 | #Feature F2 | #Feature F3 | #Feature F4 | #Feature F5 | #Feature F6 | #Feature F7 | Start | End | PTM | AScore | Found By |
| --- | --- | --- | --- | --- | --- | --- | --- | --- | --- | --- | --- | --- | --- | --- | --- | --- | --- | --- | --- | --- | --- | --- | --- | --- | --- | --- | --- | --- | --- | --- | --- |
| R.QIAAYDYNPLSGTISNKR.I | Y | 74.55 | 2010.0170 | 18 | -0.7 | 671.0125 | 3 | 52.72 | 4 | F4:13863 | Fraction\_4\_23092022.raw |  |  | 1.3568E5 | 1.6835E5 |  |  |  | 2 | 0 | 0 | 1 | 1 | 0 | 0 | 0 | 193 | 210 |  |  | PEAKS DB |
| total 1 peptides |  |  |  |  |  |  |  |  |  |  |  |  |  |  |  |  |  |  |  |  |  |  |  |  |  |  |  |  |  |  |  |
| --- | --- | --- | --- | --- | --- | --- | --- | --- | --- | --- | --- | --- | --- | --- | --- | --- | --- | --- | --- | --- | --- | --- | --- | --- | --- | --- | --- | --- | --- | --- | --- |

TRINITY\_DN1736\_c0\_g1\_i24.p1

back to list

  

| Protein Coverage
| Supporting Peptides
|

Protein Coverage:

Supporting Peptides:

| Peptide | Uniq | -10lgP | Mass | Length | ppm | m/z | z | RT | Fraction | Scan | Source File | Area F1 | Area F2 | Area F3 | Area F4 | Area F5 | Area F6 | Area F7 | #Feature | #Feature F1 | #Feature F2 | #Feature F3 | #Feature F4 | #Feature F5 | #Feature F6 | #Feature F7 | Start | End | PTM | AScore | Found By |
| --- | --- | --- | --- | --- | --- | --- | --- | --- | --- | --- | --- | --- | --- | --- | --- | --- | --- | --- | --- | --- | --- | --- | --- | --- | --- | --- | --- | --- | --- | --- | --- |
| R.QIAAYDYNPLSGTISNKR.I | Y | 74.55 | 2010.0170 | 18 | -0.7 | 671.0125 | 3 | 52.72 | 4 | F4:13863 | Fraction\_4\_23092022.raw |  |  | 1.3568E5 | 1.6835E5 |  |  |  | 2 | 0 | 0 | 1 | 1 | 0 | 0 | 0 | 194 | 211 |  |  | PEAKS DB |
| total 1 peptides |  |  |  |  |  |  |  |  |  |  |  |  |  |  |  |  |  |  |  |  |  |  |  |  |  |  |  |  |  |  |  |
| --- | --- | --- | --- | --- | --- | --- | --- | --- | --- | --- | --- | --- | --- | --- | --- | --- | --- | --- | --- | --- | --- | --- | --- | --- | --- | --- | --- | --- | --- | --- | --- |

TRINITY\_DN1736\_c0\_g1\_i4.p1

back to list

  

| Protein Coverage
| Supporting Peptides
|

Protein Coverage:

Supporting Peptides:

| Peptide | Uniq | -10lgP | Mass | Length | ppm | m/z | z | RT | Fraction | Scan | Source File | Area F1 | Area F2 | Area F3 | Area F4 | Area F5 | Area F6 | Area F7 | #Feature | #Feature F1 | #Feature F2 | #Feature F3 | #Feature F4 | #Feature F5 | #Feature F6 | #Feature F7 | Start | End | PTM | AScore | Found By |
| --- | --- | --- | --- | --- | --- | --- | --- | --- | --- | --- | --- | --- | --- | --- | --- | --- | --- | --- | --- | --- | --- | --- | --- | --- | --- | --- | --- | --- | --- | --- | --- |
| R.QIAAYDYNPLSGTISNKR.I | Y | 74.55 | 2010.0170 | 18 | -0.7 | 671.0125 | 3 | 52.72 | 4 | F4:13863 | Fraction\_4\_23092022.raw |  |  | 1.3568E5 | 1.6835E5 |  |  |  | 2 | 0 | 0 | 1 | 1 | 0 | 0 | 0 | 212 | 229 |  |  | PEAKS DB |
| total 1 peptides |  |  |  |  |  |  |  |  |  |  |  |  |  |  |  |  |  |  |  |  |  |  |  |  |  |  |  |  |  |  |  |
| --- | --- | --- | --- | --- | --- | --- | --- | --- | --- | --- | --- | --- | --- | --- | --- | --- | --- | --- | --- | --- | --- | --- | --- | --- | --- | --- | --- | --- | --- | --- | --- |

TRINITY\_DN1450\_c3\_g2\_i1.p1

back to list

  

| Protein Coverage
| Supporting Peptides
|

Protein Coverage:

Supporting Peptides:

| Peptide | Uniq | -10lgP | Mass | Length | ppm | m/z | z | RT | Fraction | Scan | Source File | Area F1 | Area F2 | Area F3 | Area F4 | Area F5 | Area F6 | Area F7 | #Feature | #Feature F1 | #Feature F2 | #Feature F3 | #Feature F4 | #Feature F5 | #Feature F6 | #Feature F7 | Start | End | PTM | AScore | Found By |
| --- | --- | --- | --- | --- | --- | --- | --- | --- | --- | --- | --- | --- | --- | --- | --- | --- | --- | --- | --- | --- | --- | --- | --- | --- | --- | --- | --- | --- | --- | --- | --- |
| R.GLFIIDDKQNLR.Q | Y | 50.20 | 1430.7881 | 12 | 0.3 | 477.9368 | 3 | 53.71 | 6 | F6:14729 | Fraction\_6\_23092022.raw |  |  |  |  |  | 7.7847E4 |  | 1 | 0 | 0 | 0 | 0 | 0 | 1 | 0 | 124 | 135 |  |  | PEAKS DB |
| R.QITVNDLPVGR.S | Y | 48.07 | 1210.6670 | 11 | -0.1 | 606.3407 | 2 | 50.63 | 1 | F1:11862 | Fraction\_1\_23092022r.raw | 9.9582E4 |  |  |  |  |  |  | 1 | 1 | 0 | 0 | 0 | 0 | 0 | 0 | 136 | 146 |  |  | PEAKS DB |
| total 2 peptides |  |  |  |  |  |  |  |  |  |  |  |  |  |  |  |  |  |  |  |  |  |  |  |  |  |  |  |  |  |  |  |
| --- | --- | --- | --- | --- | --- | --- | --- | --- | --- | --- | --- | --- | --- | --- | --- | --- | --- | --- | --- | --- | --- | --- | --- | --- | --- | --- | --- | --- | --- | --- | --- |

Q9V3P0|PRDX1\_DROME

back to list

  

| Protein Coverage
| Supporting Peptides
|

Protein Coverage:

Supporting Peptides:

| Peptide | Uniq | -10lgP | Mass | Length | ppm | m/z | z | RT | Fraction | Scan | Source File | Area F1 | Area F2 | Area F3 | Area F4 | Area F5 | Area F6 | Area F7 | #Feature | #Feature F1 | #Feature F2 | #Feature F3 | #Feature F4 | #Feature F5 | #Feature F6 | #Feature F7 | Start | End | PTM | AScore | Found By |
| --- | --- | --- | --- | --- | --- | --- | --- | --- | --- | --- | --- | --- | --- | --- | --- | --- | --- | --- | --- | --- | --- | --- | --- | --- | --- | --- | --- | --- | --- | --- | --- |
| R.GLFIIDDKQNLR.Q | Y | 50.20 | 1430.7881 | 12 | 0.3 | 477.9368 | 3 | 53.71 | 6 | F6:14729 | Fraction\_6\_23092022.raw |  |  |  |  |  | 7.7847E4 |  | 1 | 0 | 0 | 0 | 0 | 0 | 1 | 0 | 124 | 135 |  |  | PEAKS DB |
| R.QITVNDLPVGR.S | Y | 48.07 | 1210.6670 | 11 | -0.1 | 606.3407 | 2 | 50.63 | 1 | F1:11862 | Fraction\_1\_23092022r.raw | 9.9582E4 |  |  |  |  |  |  | 1 | 1 | 0 | 0 | 0 | 0 | 0 | 0 | 136 | 146 |  |  | PEAKS DB |
| total 2 peptides |  |  |  |  |  |  |  |  |  |  |  |  |  |  |  |  |  |  |  |  |  |  |  |  |  |  |  |  |  |  |  |
| --- | --- | --- | --- | --- | --- | --- | --- | --- | --- | --- | --- | --- | --- | --- | --- | --- | --- | --- | --- | --- | --- | --- | --- | --- | --- | --- | --- | --- | --- | --- | --- |

P12763|FETUA\_BOVIN

back to list

  

| Protein Coverage
| Supporting Peptides
|

Protein Coverage:

Supporting Peptides:

| Peptide | Uniq | -10lgP | Mass | Length | ppm | m/z | z | RT | Fraction | Scan | Source File | Area F1 | Area F2 | Area F3 | Area F4 | Area F5 | Area F6 | Area F7 | #Feature | #Feature F1 | #Feature F2 | #Feature F3 | #Feature F4 | #Feature F5 | #Feature F6 | #Feature F7 | Start | End | PTM | AScore | Found By |
| --- | --- | --- | --- | --- | --- | --- | --- | --- | --- | --- | --- | --- | --- | --- | --- | --- | --- | --- | --- | --- | --- | --- | --- | --- | --- | --- | --- | --- | --- | --- | --- |
| R.HTFSGVASVESSSGEAFHVGK.T | Y | 73.75 | 2118.9971 | 21 | -1.1 | 707.3389 | 3 | 43.07 | 1 | F1:8489 | Fraction\_1\_23092022r.raw | 4.2769E5 |  |  |  |  |  |  | 1 | 1 | 0 | 0 | 0 | 0 | 0 | 0 | 313 | 333 |  |  | PEAKS DB |
| total 1 peptides |  |  |  |  |  |  |  |  |  |  |  |  |  |  |  |  |  |  |  |  |  |  |  |  |  |  |  |  |  |  |  |
| --- | --- | --- | --- | --- | --- | --- | --- | --- | --- | --- | --- | --- | --- | --- | --- | --- | --- | --- | --- | --- | --- | --- | --- | --- | --- | --- | --- | --- | --- | --- | --- |

P02754|LACB\_BOVIN

back to list

  

| Protein Coverage
| Supporting Peptides
|

Protein Coverage:

Supporting Peptides:

| Peptide | Uniq | -10lgP | Mass | Length | ppm | m/z | z | RT | Fraction | Scan | Source File | Area F1 | Area F2 | Area F3 | Area F4 | Area F5 | Area F6 | Area F7 | #Feature | #Feature F1 | #Feature F2 | #Feature F3 | #Feature F4 | #Feature F5 | #Feature F6 | #Feature F7 | Start | End | PTM | AScore | Found By |
| --- | --- | --- | --- | --- | --- | --- | --- | --- | --- | --- | --- | --- | --- | --- | --- | --- | --- | --- | --- | --- | --- | --- | --- | --- | --- | --- | --- | --- | --- | --- | --- |
| R.LSFNPTQLEEQC(+57.02)HI | Y | 72.06 | 1714.7985 | 14 | 0.0 | 858.4065 | 2 | 65.07 | 5 | F5:19163 | Fraction\_5\_23092022.raw | 2.8946E5 | 1.1975E5 | 2.0216E5 | 1.4495E5 | 2.1549E5 | 4.217E5 |  | 6 | 1 | 1 | 1 | 1 | 1 | 1 | 0 | 165 | 178 | Carbamidomethylation | C12:Carbamidomethylation:1000.00 | PEAKS DB |
| total 1 peptides |  |  |  |  |  |  |  |  |  |  |  |  |  |  |  |  |  |  |  |  |  |  |  |  |  |  |  |  |  |  |  |
| --- | --- | --- | --- | --- | --- | --- | --- | --- | --- | --- | --- | --- | --- | --- | --- | --- | --- | --- | --- | --- | --- | --- | --- | --- | --- | --- | --- | --- | --- | --- | --- |

P02755|LACB\_BUBBU

back to list

  

| Protein Coverage
| Supporting Peptides
|

Protein Coverage:

Supporting Peptides:

| Peptide | Uniq | -10lgP | Mass | Length | ppm | m/z | z | RT | Fraction | Scan | Source File | Area F1 | Area F2 | Area F3 | Area F4 | Area F5 | Area F6 | Area F7 | #Feature | #Feature F1 | #Feature F2 | #Feature F3 | #Feature F4 | #Feature F5 | #Feature F6 | #Feature F7 | Start | End | PTM | AScore | Found By |
| --- | --- | --- | --- | --- | --- | --- | --- | --- | --- | --- | --- | --- | --- | --- | --- | --- | --- | --- | --- | --- | --- | --- | --- | --- | --- | --- | --- | --- | --- | --- | --- |
| R.LSFNPTQLEEQC(+57.02)HV | Y | 71.06 | 1700.7828 | 14 | 0.1 | 851.3987 | 2 | 61.62 | 5 | F5:17618 | Fraction\_5\_23092022.raw |  |  |  |  | 5.3867E5 | 3.6218E5 |  | 2 | 0 | 0 | 0 | 0 | 1 | 1 | 0 | 167 | 180 | Carbamidomethylation | C12:Carbamidomethylation:1000.00 | PEAKS DB |
| total 1 peptides |  |  |  |  |  |  |  |  |  |  |  |  |  |  |  |  |  |  |  |  |  |  |  |  |  |  |  |  |  |  |  |
| --- | --- | --- | --- | --- | --- | --- | --- | --- | --- | --- | --- | --- | --- | --- | --- | --- | --- | --- | --- | --- | --- | --- | --- | --- | --- | --- | --- | --- | --- | --- | --- |

TRINITY\_DN254\_c0\_g1\_i1.p1

back to list

  

| Protein Coverage
| Supporting Peptides
|

Protein Coverage:

Supporting Peptides:

| Peptide | Uniq | -10lgP | Mass | Length | ppm | m/z | z | RT | Fraction | Scan | Source File | Area F1 | Area F2 | Area F3 | Area F4 | Area F5 | Area F6 | Area F7 | #Feature | #Feature F1 | #Feature F2 | #Feature F3 | #Feature F4 | #Feature F5 | #Feature F6 | #Feature F7 | Start | End | PTM | AScore | Found By |
| --- | --- | --- | --- | --- | --- | --- | --- | --- | --- | --- | --- | --- | --- | --- | --- | --- | --- | --- | --- | --- | --- | --- | --- | --- | --- | --- | --- | --- | --- | --- | --- |
| K.TDGTGAFPSLLKGGPGSR.N | Y | 70.42 | 1716.8794 | 18 | -1.3 | 573.2997 | 3 | 51.53 | 5 | F5:13065 | Fraction\_5\_23092022.raw |  |  |  |  | 1.8262E5 |  |  | 1 | 0 | 0 | 0 | 0 | 1 | 0 | 0 | 91 | 108 |  |  | PEAKS DB |
| total 1 peptides |  |  |  |  |  |  |  |  |  |  |  |  |  |  |  |  |  |  |  |  |  |  |  |  |  |  |  |  |  |  |  |
| --- | --- | --- | --- | --- | --- | --- | --- | --- | --- | --- | --- | --- | --- | --- | --- | --- | --- | --- | --- | --- | --- | --- | --- | --- | --- | --- | --- | --- | --- | --- | --- |

TRINITY\_DN747\_c0\_g1\_i8.p1

back to list

  

| Protein Coverage
| Supporting Peptides
|

Protein Coverage:

Supporting Peptides:

| Peptide | Uniq | -10lgP | Mass | Length | ppm | m/z | z | RT | Fraction | Scan | Source File | Area F1 | Area F2 | Area F3 | Area F4 | Area F5 | Area F6 | Area F7 | #Feature | #Feature F1 | #Feature F2 | #Feature F3 | #Feature F4 | #Feature F5 | #Feature F6 | #Feature F7 | Start | End | PTM | AScore | Found By |
| --- | --- | --- | --- | --- | --- | --- | --- | --- | --- | --- | --- | --- | --- | --- | --- | --- | --- | --- | --- | --- | --- | --- | --- | --- | --- | --- | --- | --- | --- | --- | --- |
| R.VLLNLGDSVTTDHISPAGSIAR.N | Y | 68.25 | 2235.1858 | 22 | 1.6 | 746.0704 | 3 | 61.18 | 1 | F1:16561 | Fraction\_1\_23092022r.raw | 2.0736E5 |  |  |  |  |  |  | 1 | 1 | 0 | 0 | 0 | 0 | 0 | 0 | 679 | 700 |  |  | PEAKS DB |
| total 1 peptides |  |  |  |  |  |  |  |  |  |  |  |  |  |  |  |  |  |  |  |  |  |  |  |  |  |  |  |  |  |  |  |
| --- | --- | --- | --- | --- | --- | --- | --- | --- | --- | --- | --- | --- | --- | --- | --- | --- | --- | --- | --- | --- | --- | --- | --- | --- | --- | --- | --- | --- | --- | --- | --- |

TRINITY\_DN747\_c0\_g1\_i4.p1

back to list

  

| Protein Coverage
| Supporting Peptides
|

Protein Coverage:

Supporting Peptides:

| Peptide | Uniq | -10lgP | Mass | Length | ppm | m/z | z | RT | Fraction | Scan | Source File | Area F1 | Area F2 | Area F3 | Area F4 | Area F5 | Area F6 | Area F7 | #Feature | #Feature F1 | #Feature F2 | #Feature F3 | #Feature F4 | #Feature F5 | #Feature F6 | #Feature F7 | Start | End | PTM | AScore | Found By |
| --- | --- | --- | --- | --- | --- | --- | --- | --- | --- | --- | --- | --- | --- | --- | --- | --- | --- | --- | --- | --- | --- | --- | --- | --- | --- | --- | --- | --- | --- | --- | --- |
| R.VLLNLGDSVTTDHISPAGSIAR.N | Y | 68.25 | 2235.1858 | 22 | 1.6 | 746.0704 | 3 | 61.18 | 1 | F1:16561 | Fraction\_1\_23092022r.raw | 2.0736E5 |  |  |  |  |  |  | 1 | 1 | 0 | 0 | 0 | 0 | 0 | 0 | 679 | 700 |  |  | PEAKS DB |
| total 1 peptides |  |  |  |  |  |  |  |  |  |  |  |  |  |  |  |  |  |  |  |  |  |  |  |  |  |  |  |  |  |  |  |
| --- | --- | --- | --- | --- | --- | --- | --- | --- | --- | --- | --- | --- | --- | --- | --- | --- | --- | --- | --- | --- | --- | --- | --- | --- | --- | --- | --- | --- | --- | --- | --- |

TRINITY\_DN747\_c0\_g1\_i7.p1

back to list

  

| Protein Coverage
| Supporting Peptides
|

Protein Coverage:

Supporting Peptides:

| Peptide | Uniq | -10lgP | Mass | Length | ppm | m/z | z | RT | Fraction | Scan | Source File | Area F1 | Area F2 | Area F3 | Area F4 | Area F5 | Area F6 | Area F7 | #Feature | #Feature F1 | #Feature F2 | #Feature F3 | #Feature F4 | #Feature F5 | #Feature F6 | #Feature F7 | Start | End | PTM | AScore | Found By |
| --- | --- | --- | --- | --- | --- | --- | --- | --- | --- | --- | --- | --- | --- | --- | --- | --- | --- | --- | --- | --- | --- | --- | --- | --- | --- | --- | --- | --- | --- | --- | --- |
| R.VLLNLGDSVTTDHISPAGSIAR.N | Y | 68.25 | 2235.1858 | 22 | 1.6 | 746.0704 | 3 | 61.18 | 1 | F1:16561 | Fraction\_1\_23092022r.raw | 2.0736E5 |  |  |  |  |  |  | 1 | 1 | 0 | 0 | 0 | 0 | 0 | 0 | 679 | 700 |  |  | PEAKS DB |
| total 1 peptides |  |  |  |  |  |  |  |  |  |  |  |  |  |  |  |  |  |  |  |  |  |  |  |  |  |  |  |  |  |  |  |
| --- | --- | --- | --- | --- | --- | --- | --- | --- | --- | --- | --- | --- | --- | --- | --- | --- | --- | --- | --- | --- | --- | --- | --- | --- | --- | --- | --- | --- | --- | --- | --- |

TRINITY\_DN747\_c0\_g1\_i1.p1

back to list

  

| Protein Coverage
| Supporting Peptides
|

Protein Coverage:

Supporting Peptides:

| Peptide | Uniq | -10lgP | Mass | Length | ppm | m/z | z | RT | Fraction | Scan | Source File | Area F1 | Area F2 | Area F3 | Area F4 | Area F5 | Area F6 | Area F7 | #Feature | #Feature F1 | #Feature F2 | #Feature F3 | #Feature F4 | #Feature F5 | #Feature F6 | #Feature F7 | Start | End | PTM | AScore | Found By |
| --- | --- | --- | --- | --- | --- | --- | --- | --- | --- | --- | --- | --- | --- | --- | --- | --- | --- | --- | --- | --- | --- | --- | --- | --- | --- | --- | --- | --- | --- | --- | --- |
| R.VLLNLGDSVTTDHISPAGSIAR.N | Y | 68.25 | 2235.1858 | 22 | 1.6 | 746.0704 | 3 | 61.18 | 1 | F1:16561 | Fraction\_1\_23092022r.raw | 2.0736E5 |  |  |  |  |  |  | 1 | 1 | 0 | 0 | 0 | 0 | 0 | 0 | 694 | 715 |  |  | PEAKS DB |
| total 1 peptides |  |  |  |  |  |  |  |  |  |  |  |  |  |  |  |  |  |  |  |  |  |  |  |  |  |  |  |  |  |  |  |
| --- | --- | --- | --- | --- | --- | --- | --- | --- | --- | --- | --- | --- | --- | --- | --- | --- | --- | --- | --- | --- | --- | --- | --- | --- | --- | --- | --- | --- | --- | --- | --- |

TRINITY\_DN27415\_c0\_g1\_i1.p1

back to list

  

| Protein Coverage
| Supporting Peptides
|

Protein Coverage:

Supporting Peptides:

| Peptide | Uniq | -10lgP | Mass | Length | ppm | m/z | z | RT | Fraction | Scan | Source File | Area F1 | Area F2 | Area F3 | Area F4 | Area F5 | Area F6 | Area F7 | #Feature | #Feature F1 | #Feature F2 | #Feature F3 | #Feature F4 | #Feature F5 | #Feature F6 | #Feature F7 | Start | End | PTM | AScore | Found By |
| --- | --- | --- | --- | --- | --- | --- | --- | --- | --- | --- | --- | --- | --- | --- | --- | --- | --- | --- | --- | --- | --- | --- | --- | --- | --- | --- | --- | --- | --- | --- | --- |
| R.FTSVNLHSLSR.G | Y | 68.01 | 1259.6622 | 11 | -0.3 | 420.8946 | 3 | 40.69 | 4 | F4:8484 | Fraction\_4\_23092022.raw |  |  |  | 1.1207E5 |  |  |  | 2 | 0 | 0 | 0 | 2 | 0 | 0 | 0 | 276 | 286 |  |  | PEAKS DB |
| total 1 peptides |  |  |  |  |  |  |  |  |  |  |  |  |  |  |  |  |  |  |  |  |  |  |  |  |  |  |  |  |  |  |  |
| --- | --- | --- | --- | --- | --- | --- | --- | --- | --- | --- | --- | --- | --- | --- | --- | --- | --- | --- | --- | --- | --- | --- | --- | --- | --- | --- | --- | --- | --- | --- | --- |

P0DO15|GLCA2\_SOYBN

back to list

  

| Protein Coverage
| Supporting Peptides
|

Protein Coverage:

Supporting Peptides:

| Peptide | Uniq | -10lgP | Mass | Length | ppm | m/z | z | RT | Fraction | Scan | Source File | Area F1 | Area F2 | Area F3 | Area F4 | Area F5 | Area F6 | Area F7 | #Feature | #Feature F1 | #Feature F2 | #Feature F3 | #Feature F4 | #Feature F5 | #Feature F6 | #Feature F7 | Start | End | PTM | AScore | Found By |
| --- | --- | --- | --- | --- | --- | --- | --- | --- | --- | --- | --- | --- | --- | --- | --- | --- | --- | --- | --- | --- | --- | --- | --- | --- | --- | --- | --- | --- | --- | --- | --- |
| K.FFEITPEKNPQLR.D | Y | 66.41 | 1617.8514 | 13 | 0.8 | 540.2915 | 3 | 50.17 | 1 | F1:11651 | Fraction\_1\_23092022r.raw | 2.4976E5 |  |  |  |  |  |  | 1 | 1 | 0 | 0 | 0 | 0 | 0 | 0 | 422 | 434 |  |  | PEAKS DB |
| total 1 peptides |  |  |  |  |  |  |  |  |  |  |  |  |  |  |  |  |  |  |  |  |  |  |  |  |  |  |  |  |  |  |  |
| --- | --- | --- | --- | --- | --- | --- | --- | --- | --- | --- | --- | --- | --- | --- | --- | --- | --- | --- | --- | --- | --- | --- | --- | --- | --- | --- | --- | --- | --- | --- | --- |

P0DO16|GLCA1\_SOYBN

back to list

  

| Protein Coverage
| Supporting Peptides
|

Protein Coverage:

Supporting Peptides:

| Peptide | Uniq | -10lgP | Mass | Length | ppm | m/z | z | RT | Fraction | Scan | Source File | Area F1 | Area F2 | Area F3 | Area F4 | Area F5 | Area F6 | Area F7 | #Feature | #Feature F1 | #Feature F2 | #Feature F3 | #Feature F4 | #Feature F5 | #Feature F6 | #Feature F7 | Start | End | PTM | AScore | Found By |
| --- | --- | --- | --- | --- | --- | --- | --- | --- | --- | --- | --- | --- | --- | --- | --- | --- | --- | --- | --- | --- | --- | --- | --- | --- | --- | --- | --- | --- | --- | --- | --- |
| K.FFEITPEKNPQLR.D | Y | 66.41 | 1617.8514 | 13 | 0.8 | 540.2915 | 3 | 50.17 | 1 | F1:11651 | Fraction\_1\_23092022r.raw | 2.4976E5 |  |  |  |  |  |  | 1 | 1 | 0 | 0 | 0 | 0 | 0 | 0 | 422 | 434 |  |  | PEAKS DB |
| total 1 peptides |  |  |  |  |  |  |  |  |  |  |  |  |  |  |  |  |  |  |  |  |  |  |  |  |  |  |  |  |  |  |  |
| --- | --- | --- | --- | --- | --- | --- | --- | --- | --- | --- | --- | --- | --- | --- | --- | --- | --- | --- | --- | --- | --- | --- | --- | --- | --- | --- | --- | --- | --- | --- | --- |

F7J077|GLCB2\_SOYBN

back to list

  

| Protein Coverage
| Supporting Peptides
|

Protein Coverage:

Supporting Peptides:

| Peptide | Uniq | -10lgP | Mass | Length | ppm | m/z | z | RT | Fraction | Scan | Source File | Area F1 | Area F2 | Area F3 | Area F4 | Area F5 | Area F6 | Area F7 | #Feature | #Feature F1 | #Feature F2 | #Feature F3 | #Feature F4 | #Feature F5 | #Feature F6 | #Feature F7 | Start | End | PTM | AScore | Found By |
| --- | --- | --- | --- | --- | --- | --- | --- | --- | --- | --- | --- | --- | --- | --- | --- | --- | --- | --- | --- | --- | --- | --- | --- | --- | --- | --- | --- | --- | --- | --- | --- |
| K.FFEITPEKNPQLR.D | Y | 66.41 | 1617.8514 | 13 | 0.8 | 540.2915 | 3 | 50.17 | 1 | F1:11651 | Fraction\_1\_23092022r.raw | 2.4976E5 |  |  |  |  |  |  | 1 | 1 | 0 | 0 | 0 | 0 | 0 | 0 | 256 | 268 |  |  | PEAKS DB |
| total 1 peptides |  |  |  |  |  |  |  |  |  |  |  |  |  |  |  |  |  |  |  |  |  |  |  |  |  |  |  |  |  |  |  |
| --- | --- | --- | --- | --- | --- | --- | --- | --- | --- | --- | --- | --- | --- | --- | --- | --- | --- | --- | --- | --- | --- | --- | --- | --- | --- | --- | --- | --- | --- | --- | --- |

P25974|GLCB1\_SOYBN

back to list

  

| Protein Coverage
| Supporting Peptides
|

Protein Coverage:

Supporting Peptides:

| Peptide | Uniq | -10lgP | Mass | Length | ppm | m/z | z | RT | Fraction | Scan | Source File | Area F1 | Area F2 | Area F3 | Area F4 | Area F5 | Area F6 | Area F7 | #Feature | #Feature F1 | #Feature F2 | #Feature F3 | #Feature F4 | #Feature F5 | #Feature F6 | #Feature F7 | Start | End | PTM | AScore | Found By |
| --- | --- | --- | --- | --- | --- | --- | --- | --- | --- | --- | --- | --- | --- | --- | --- | --- | --- | --- | --- | --- | --- | --- | --- | --- | --- | --- | --- | --- | --- | --- | --- |
| K.FFEITPEKNPQLR.D | Y | 66.41 | 1617.8514 | 13 | 0.8 | 540.2915 | 3 | 50.17 | 1 | F1:11651 | Fraction\_1\_23092022r.raw | 2.4976E5 |  |  |  |  |  |  | 1 | 1 | 0 | 0 | 0 | 0 | 0 | 0 | 256 | 268 |  |  | PEAKS DB |
| total 1 peptides |  |  |  |  |  |  |  |  |  |  |  |  |  |  |  |  |  |  |  |  |  |  |  |  |  |  |  |  |  |  |  |
| --- | --- | --- | --- | --- | --- | --- | --- | --- | --- | --- | --- | --- | --- | --- | --- | --- | --- | --- | --- | --- | --- | --- | --- | --- | --- | --- | --- | --- | --- | --- | --- |

TRINITY\_DN6486\_c0\_g1\_i4.p1

back to list

  

| Protein Coverage
| Supporting Peptides
|

Protein Coverage:

Supporting Peptides:

| Peptide | Uniq | -10lgP | Mass | Length | ppm | m/z | z | RT | Fraction | Scan | Source File | Area F1 | Area F2 | Area F3 | Area F4 | Area F5 | Area F6 | Area F7 | #Feature | #Feature F1 | #Feature F2 | #Feature F3 | #Feature F4 | #Feature F5 | #Feature F6 | #Feature F7 | Start | End | PTM | AScore | Found By |
| --- | --- | --- | --- | --- | --- | --- | --- | --- | --- | --- | --- | --- | --- | --- | --- | --- | --- | --- | --- | --- | --- | --- | --- | --- | --- | --- | --- | --- | --- | --- | --- |
| R.GFPVC(+57.02)GNFKPSVYI | Y | 65.97 | 1583.7806 | 14 | 0.4 | 792.8979 | 2 | 71.37 | 2 | F2:21356 | Fraction\_2\_23092022.raw | 3.7868E5 | 3.3082E6 |  |  |  |  |  | 2 | 1 | 1 | 0 | 0 | 0 | 0 | 0 | 191 | 204 | Carbamidomethylation | C5:Carbamidomethylation:1000.00 | PEAKS DB |
| total 1 peptides |  |  |  |  |  |  |  |  |  |  |  |  |  |  |  |  |  |  |  |  |  |  |  |  |  |  |  |  |  |  |  |
| --- | --- | --- | --- | --- | --- | --- | --- | --- | --- | --- | --- | --- | --- | --- | --- | --- | --- | --- | --- | --- | --- | --- | --- | --- | --- | --- | --- | --- | --- | --- | --- |

TRINITY\_DN3209\_c0\_g2\_i3.p1

back to list

  

| Protein Coverage
| Supporting Peptides
|

Protein Coverage:

Supporting Peptides:

| Peptide | Uniq | -10lgP | Mass | Length | ppm | m/z | z | RT | Fraction | Scan | Source File | Area F1 | Area F2 | Area F3 | Area F4 | Area F5 | Area F6 | Area F7 | #Feature | #Feature F1 | #Feature F2 | #Feature F3 | #Feature F4 | #Feature F5 | #Feature F6 | #Feature F7 | Start | End | PTM | AScore | Found By |
| --- | --- | --- | --- | --- | --- | --- | --- | --- | --- | --- | --- | --- | --- | --- | --- | --- | --- | --- | --- | --- | --- | --- | --- | --- | --- | --- | --- | --- | --- | --- | --- |
| K.AAEFGAVAALIR.S | Y | 63.54 | 1187.6663 | 12 | 1.2 | 594.8411 | 2 | 69.63 | 2 | F2:20503 | Fraction\_2\_23092022.raw |  | 3.8513E5 |  |  |  |  |  | 1 | 0 | 1 | 0 | 0 | 0 | 0 | 0 | 198 | 209 |  |  | PEAKS DB |
| total 1 peptides |  |  |  |  |  |  |  |  |  |  |  |  |  |  |  |  |  |  |  |  |  |  |  |  |  |  |  |  |  |  |  |
| --- | --- | --- | --- | --- | --- | --- | --- | --- | --- | --- | --- | --- | --- | --- | --- | --- | --- | --- | --- | --- | --- | --- | --- | --- | --- | --- | --- | --- | --- | --- | --- |

TRINITY\_DN3209\_c0\_g2\_i5.p1

back to list

  

| Protein Coverage
| Supporting Peptides
|

Protein Coverage:

Supporting Peptides:

| Peptide | Uniq | -10lgP | Mass | Length | ppm | m/z | z | RT | Fraction | Scan | Source File | Area F1 | Area F2 | Area F3 | Area F4 | Area F5 | Area F6 | Area F7 | #Feature | #Feature F1 | #Feature F2 | #Feature F3 | #Feature F4 | #Feature F5 | #Feature F6 | #Feature F7 | Start | End | PTM | AScore | Found By |
| --- | --- | --- | --- | --- | --- | --- | --- | --- | --- | --- | --- | --- | --- | --- | --- | --- | --- | --- | --- | --- | --- | --- | --- | --- | --- | --- | --- | --- | --- | --- | --- |
| K.AAEFGAVAALIR.S | Y | 63.54 | 1187.6663 | 12 | 1.2 | 594.8411 | 2 | 69.63 | 2 | F2:20503 | Fraction\_2\_23092022.raw |  | 3.8513E5 |  |  |  |  |  | 1 | 0 | 1 | 0 | 0 | 0 | 0 | 0 | 198 | 209 |  |  | PEAKS DB |
| total 1 peptides |  |  |  |  |  |  |  |  |  |  |  |  |  |  |  |  |  |  |  |  |  |  |  |  |  |  |  |  |  |  |  |
| --- | --- | --- | --- | --- | --- | --- | --- | --- | --- | --- | --- | --- | --- | --- | --- | --- | --- | --- | --- | --- | --- | --- | --- | --- | --- | --- | --- | --- | --- | --- | --- |

Q29577|KCRU\_PIG

back to list

  

| Protein Coverage
| Supporting Peptides
|

Protein Coverage:

Supporting Peptides:

| Peptide | Uniq | -10lgP | Mass | Length | ppm | m/z | z | RT | Fraction | Scan | Source File | Area F1 | Area F2 | Area F3 | Area F4 | Area F5 | Area F6 | Area F7 | #Feature | #Feature F1 | #Feature F2 | #Feature F3 | #Feature F4 | #Feature F5 | #Feature F6 | #Feature F7 | Start | End | PTM | AScore | Found By |
| --- | --- | --- | --- | --- | --- | --- | --- | --- | --- | --- | --- | --- | --- | --- | --- | --- | --- | --- | --- | --- | --- | --- | --- | --- | --- | --- | --- | --- | --- | --- | --- |
| R.VVVDALSGLKGDLAGR.Y | Y | 63.33 | 1568.8885 | 16 | 0.5 | 523.9704 | 3 | 57.58 | 3 | F3:15518 | Fraction\_3\_23092022.raw |  |  | 7.2941E4 |  |  |  |  | 1 | 0 | 0 | 1 | 0 | 0 | 0 | 0 | 190 | 205 |  |  | PEAKS DB |
| total 1 peptides |  |  |  |  |  |  |  |  |  |  |  |  |  |  |  |  |  |  |  |  |  |  |  |  |  |  |  |  |  |  |  |
| --- | --- | --- | --- | --- | --- | --- | --- | --- | --- | --- | --- | --- | --- | --- | --- | --- | --- | --- | --- | --- | --- | --- | --- | --- | --- | --- | --- | --- | --- | --- | --- |

Q9TTK8|KCRU\_BOVIN

back to list

  

| Protein Coverage
| Supporting Peptides
|

Protein Coverage:

Supporting Peptides:

| Peptide | Uniq | -10lgP | Mass | Length | ppm | m/z | z | RT | Fraction | Scan | Source File | Area F1 | Area F2 | Area F3 | Area F4 | Area F5 | Area F6 | Area F7 | #Feature | #Feature F1 | #Feature F2 | #Feature F3 | #Feature F4 | #Feature F5 | #Feature F6 | #Feature F7 | Start | End | PTM | AScore | Found By |
| --- | --- | --- | --- | --- | --- | --- | --- | --- | --- | --- | --- | --- | --- | --- | --- | --- | --- | --- | --- | --- | --- | --- | --- | --- | --- | --- | --- | --- | --- | --- | --- |
| R.VVVDALSGLKGDLAGR.Y | Y | 63.33 | 1568.8885 | 16 | 0.5 | 523.9704 | 3 | 57.58 | 3 | F3:15518 | Fraction\_3\_23092022.raw |  |  | 7.2941E4 |  |  |  |  | 1 | 0 | 0 | 1 | 0 | 0 | 0 | 0 | 190 | 205 |  |  | PEAKS DB |
| total 1 peptides |  |  |  |  |  |  |  |  |  |  |  |  |  |  |  |  |  |  |  |  |  |  |  |  |  |  |  |  |  |  |  |
| --- | --- | --- | --- | --- | --- | --- | --- | --- | --- | --- | --- | --- | --- | --- | --- | --- | --- | --- | --- | --- | --- | --- | --- | --- | --- | --- | --- | --- | --- | --- | --- |

P12532|KCRU\_HUMAN

back to list

  

| Protein Coverage
| Supporting Peptides
|

Protein Coverage:

Supporting Peptides:

| Peptide | Uniq | -10lgP | Mass | Length | ppm | m/z | z | RT | Fraction | Scan | Source File | Area F1 | Area F2 | Area F3 | Area F4 | Area F5 | Area F6 | Area F7 | #Feature | #Feature F1 | #Feature F2 | #Feature F3 | #Feature F4 | #Feature F5 | #Feature F6 | #Feature F7 | Start | End | PTM | AScore | Found By |
| --- | --- | --- | --- | --- | --- | --- | --- | --- | --- | --- | --- | --- | --- | --- | --- | --- | --- | --- | --- | --- | --- | --- | --- | --- | --- | --- | --- | --- | --- | --- | --- |
| R.VVVDALSGLKGDLAGR.Y | Y | 63.33 | 1568.8885 | 16 | 0.5 | 523.9704 | 3 | 57.58 | 3 | F3:15518 | Fraction\_3\_23092022.raw |  |  | 7.2941E4 |  |  |  |  | 1 | 0 | 0 | 1 | 0 | 0 | 0 | 0 | 190 | 205 |  |  | PEAKS DB |
| total 1 peptides |  |  |  |  |  |  |  |  |  |  |  |  |  |  |  |  |  |  |  |  |  |  |  |  |  |  |  |  |  |  |  |
| --- | --- | --- | --- | --- | --- | --- | --- | --- | --- | --- | --- | --- | --- | --- | --- | --- | --- | --- | --- | --- | --- | --- | --- | --- | --- | --- | --- | --- | --- | --- | --- |

P30275|KCRU\_MOUSE

back to list

  

| Protein Coverage
| Supporting Peptides
|

Protein Coverage:

Supporting Peptides:

| Peptide | Uniq | -10lgP | Mass | Length | ppm | m/z | z | RT | Fraction | Scan | Source File | Area F1 | Area F2 | Area F3 | Area F4 | Area F5 | Area F6 | Area F7 | #Feature | #Feature F1 | #Feature F2 | #Feature F3 | #Feature F4 | #Feature F5 | #Feature F6 | #Feature F7 | Start | End | PTM | AScore | Found By |
| --- | --- | --- | --- | --- | --- | --- | --- | --- | --- | --- | --- | --- | --- | --- | --- | --- | --- | --- | --- | --- | --- | --- | --- | --- | --- | --- | --- | --- | --- | --- | --- |
| R.VVVDALSGLKGDLAGR.Y | Y | 63.33 | 1568.8885 | 16 | 0.5 | 523.9704 | 3 | 57.58 | 3 | F3:15518 | Fraction\_3\_23092022.raw |  |  | 7.2941E4 |  |  |  |  | 1 | 0 | 0 | 1 | 0 | 0 | 0 | 0 | 191 | 206 |  |  | PEAKS DB |
| total 1 peptides |  |  |  |  |  |  |  |  |  |  |  |  |  |  |  |  |  |  |  |  |  |  |  |  |  |  |  |  |  |  |  |
| --- | --- | --- | --- | --- | --- | --- | --- | --- | --- | --- | --- | --- | --- | --- | --- | --- | --- | --- | --- | --- | --- | --- | --- | --- | --- | --- | --- | --- | --- | --- | --- |

P25809|KCRU\_RAT

back to list

  

| Protein Coverage
| Supporting Peptides
|

Protein Coverage:

Supporting Peptides:

| Peptide | Uniq | -10lgP | Mass | Length | ppm | m/z | z | RT | Fraction | Scan | Source File | Area F1 | Area F2 | Area F3 | Area F4 | Area F5 | Area F6 | Area F7 | #Feature | #Feature F1 | #Feature F2 | #Feature F3 | #Feature F4 | #Feature F5 | #Feature F6 | #Feature F7 | Start | End | PTM | AScore | Found By |
| --- | --- | --- | --- | --- | --- | --- | --- | --- | --- | --- | --- | --- | --- | --- | --- | --- | --- | --- | --- | --- | --- | --- | --- | --- | --- | --- | --- | --- | --- | --- | --- |
| R.VVVDALSGLKGDLAGR.Y | Y | 63.33 | 1568.8885 | 16 | 0.5 | 523.9704 | 3 | 57.58 | 3 | F3:15518 | Fraction\_3\_23092022.raw |  |  | 7.2941E4 |  |  |  |  | 1 | 0 | 0 | 1 | 0 | 0 | 0 | 0 | 191 | 206 |  |  | PEAKS DB |
| total 1 peptides |  |  |  |  |  |  |  |  |  |  |  |  |  |  |  |  |  |  |  |  |  |  |  |  |  |  |  |  |  |  |  |
| --- | --- | --- | --- | --- | --- | --- | --- | --- | --- | --- | --- | --- | --- | --- | --- | --- | --- | --- | --- | --- | --- | --- | --- | --- | --- | --- | --- | --- | --- | --- | --- |

O62823|CASA1\_BUBBU

back to list

  

| Protein Coverage
| Supporting Peptides
|

Protein Coverage:

Supporting Peptides:

| Peptide | Uniq | -10lgP | Mass | Length | ppm | m/z | z | RT | Fraction | Scan | Source File | Area F1 | Area F2 | Area F3 | Area F4 | Area F5 | Area F6 | Area F7 | #Feature | #Feature F1 | #Feature F2 | #Feature F3 | #Feature F4 | #Feature F5 | #Feature F6 | #Feature F7 | Start | End | PTM | AScore | Found By |
| --- | --- | --- | --- | --- | --- | --- | --- | --- | --- | --- | --- | --- | --- | --- | --- | --- | --- | --- | --- | --- | --- | --- | --- | --- | --- | --- | --- | --- | --- | --- | --- |
| R.YLGYLEQLLR.L | Y | 62.46 | 1266.6971 | 10 | 0.9 | 634.3564 | 2 | 79.83 | 3 | F3:25448 | Fraction\_3\_23092022.raw | 9.0559E5 | 2.6621E5 | 5.3122E5 | 1.7287E5 | 3.4714E5 | 3.8821E5 | 2.0483E6 | 7 | 1 | 1 | 1 | 1 | 1 | 1 | 1 | 106 | 115 |  |  | PEAKS DB |
| total 1 peptides |  |  |  |  |  |  |  |  |  |  |  |  |  |  |  |  |  |  |  |  |  |  |  |  |  |  |  |  |  |  |  |
| --- | --- | --- | --- | --- | --- | --- | --- | --- | --- | --- | --- | --- | --- | --- | --- | --- | --- | --- | --- | --- | --- | --- | --- | --- | --- | --- | --- | --- | --- | --- | --- |

P02662|CASA1\_BOVIN

back to list

  

| Protein Coverage
| Supporting Peptides
|

Protein Coverage:

Supporting Peptides:

| Peptide | Uniq | -10lgP | Mass | Length | ppm | m/z | z | RT | Fraction | Scan | Source File | Area F1 | Area F2 | Area F3 | Area F4 | Area F5 | Area F6 | Area F7 | #Feature | #Feature F1 | #Feature F2 | #Feature F3 | #Feature F4 | #Feature F5 | #Feature F6 | #Feature F7 | Start | End | PTM | AScore | Found By |
| --- | --- | --- | --- | --- | --- | --- | --- | --- | --- | --- | --- | --- | --- | --- | --- | --- | --- | --- | --- | --- | --- | --- | --- | --- | --- | --- | --- | --- | --- | --- | --- |
| R.YLGYLEQLLR.L | Y | 62.46 | 1266.6971 | 10 | 0.9 | 634.3564 | 2 | 79.83 | 3 | F3:25448 | Fraction\_3\_23092022.raw | 9.0559E5 | 2.6621E5 | 5.3122E5 | 1.7287E5 | 3.4714E5 | 3.8821E5 | 2.0483E6 | 7 | 1 | 1 | 1 | 1 | 1 | 1 | 1 | 106 | 115 |  |  | PEAKS DB |
| total 1 peptides |  |  |  |  |  |  |  |  |  |  |  |  |  |  |  |  |  |  |  |  |  |  |  |  |  |  |  |  |  |  |  |
| --- | --- | --- | --- | --- | --- | --- | --- | --- | --- | --- | --- | --- | --- | --- | --- | --- | --- | --- | --- | --- | --- | --- | --- | --- | --- | --- | --- | --- | --- | --- | --- |

P18626|CASA1\_CAPHI

back to list

  

| Protein Coverage
| Supporting Peptides
|

Protein Coverage:

Supporting Peptides:

| Peptide | Uniq | -10lgP | Mass | Length | ppm | m/z | z | RT | Fraction | Scan | Source File | Area F1 | Area F2 | Area F3 | Area F4 | Area F5 | Area F6 | Area F7 | #Feature | #Feature F1 | #Feature F2 | #Feature F3 | #Feature F4 | #Feature F5 | #Feature F6 | #Feature F7 | Start | End | PTM | AScore | Found By |
| --- | --- | --- | --- | --- | --- | --- | --- | --- | --- | --- | --- | --- | --- | --- | --- | --- | --- | --- | --- | --- | --- | --- | --- | --- | --- | --- | --- | --- | --- | --- | --- |
| R.YLGYLEQLLR.L | Y | 62.46 | 1266.6971 | 10 | 0.9 | 634.3564 | 2 | 79.83 | 3 | F3:25448 | Fraction\_3\_23092022.raw | 9.0559E5 | 2.6621E5 | 5.3122E5 | 1.7287E5 | 3.4714E5 | 3.8821E5 | 2.0483E6 | 7 | 1 | 1 | 1 | 1 | 1 | 1 | 1 | 106 | 115 |  |  | PEAKS DB |
| total 1 peptides |  |  |  |  |  |  |  |  |  |  |  |  |  |  |  |  |  |  |  |  |  |  |  |  |  |  |  |  |  |  |  |
| --- | --- | --- | --- | --- | --- | --- | --- | --- | --- | --- | --- | --- | --- | --- | --- | --- | --- | --- | --- | --- | --- | --- | --- | --- | --- | --- | --- | --- | --- | --- | --- |

P04653|CASA1\_SHEEP

back to list

  

| Protein Coverage
| Supporting Peptides
|

Protein Coverage:

Supporting Peptides:

| Peptide | Uniq | -10lgP | Mass | Length | ppm | m/z | z | RT | Fraction | Scan | Source File | Area F1 | Area F2 | Area F3 | Area F4 | Area F5 | Area F6 | Area F7 | #Feature | #Feature F1 | #Feature F2 | #Feature F3 | #Feature F4 | #Feature F5 | #Feature F6 | #Feature F7 | Start | End | PTM | AScore | Found By |
| --- | --- | --- | --- | --- | --- | --- | --- | --- | --- | --- | --- | --- | --- | --- | --- | --- | --- | --- | --- | --- | --- | --- | --- | --- | --- | --- | --- | --- | --- | --- | --- |
| R.YLGYLEQLLR.L | Y | 62.46 | 1266.6971 | 10 | 0.9 | 634.3564 | 2 | 79.83 | 3 | F3:25448 | Fraction\_3\_23092022.raw | 9.0559E5 | 2.6621E5 | 5.3122E5 | 1.7287E5 | 3.4714E5 | 3.8821E5 | 2.0483E6 | 7 | 1 | 1 | 1 | 1 | 1 | 1 | 1 | 106 | 115 |  |  | PEAKS DB |
| total 1 peptides |  |  |  |  |  |  |  |  |  |  |  |  |  |  |  |  |  |  |  |  |  |  |  |  |  |  |  |  |  |  |  |
| --- | --- | --- | --- | --- | --- | --- | --- | --- | --- | --- | --- | --- | --- | --- | --- | --- | --- | --- | --- | --- | --- | --- | --- | --- | --- | --- | --- | --- | --- | --- | --- |

E9Q557|DESP\_MOUSE

back to list

  

| Protein Coverage
| Supporting Peptides
|

Protein Coverage:

Supporting Peptides:

| Peptide | Uniq | -10lgP | Mass | Length | ppm | m/z | z | RT | Fraction | Scan | Source File | Area F1 | Area F2 | Area F3 | Area F4 | Area F5 | Area F6 | Area F7 | #Feature | #Feature F1 | #Feature F2 | #Feature F3 | #Feature F4 | #Feature F5 | #Feature F6 | #Feature F7 | Start | End | PTM | AScore | Found By |
| --- | --- | --- | --- | --- | --- | --- | --- | --- | --- | --- | --- | --- | --- | --- | --- | --- | --- | --- | --- | --- | --- | --- | --- | --- | --- | --- | --- | --- | --- | --- | --- |
| R.LLEAQIATGGIIDPK.E | Y | 61.14 | 1537.8715 | 15 | 0.5 | 769.9434 | 2 | 62.22 | 3 | F3:17586 | Fraction\_3\_23092022.raw |  |  | 1.0495E5 |  |  |  |  | 1 | 0 | 0 | 1 | 0 | 0 | 0 | 0 | 2379 | 2393 |  |  | PEAKS DB |
| total 1 peptides |  |  |  |  |  |  |  |  |  |  |  |  |  |  |  |  |  |  |  |  |  |  |  |  |  |  |  |  |  |  |  |
| --- | --- | --- | --- | --- | --- | --- | --- | --- | --- | --- | --- | --- | --- | --- | --- | --- | --- | --- | --- | --- | --- | --- | --- | --- | --- | --- | --- | --- | --- | --- | --- |

P15924|DESP\_HUMAN

back to list

  

| Protein Coverage
| Supporting Peptides
|

Protein Coverage:

Supporting Peptides:

| Peptide | Uniq | -10lgP | Mass | Length | ppm | m/z | z | RT | Fraction | Scan | Source File | Area F1 | Area F2 | Area F3 | Area F4 | Area F5 | Area F6 | Area F7 | #Feature | #Feature F1 | #Feature F2 | #Feature F3 | #Feature F4 | #Feature F5 | #Feature F6 | #Feature F7 | Start | End | PTM | AScore | Found By |
| --- | --- | --- | --- | --- | --- | --- | --- | --- | --- | --- | --- | --- | --- | --- | --- | --- | --- | --- | --- | --- | --- | --- | --- | --- | --- | --- | --- | --- | --- | --- | --- |
| R.LLEAQIATGGIIDPK.E | Y | 61.14 | 1537.8715 | 15 | 0.5 | 769.9434 | 2 | 62.22 | 3 | F3:17586 | Fraction\_3\_23092022.raw |  |  | 1.0495E5 |  |  |  |  | 1 | 0 | 0 | 1 | 0 | 0 | 0 | 0 | 2367 | 2381 |  |  | PEAKS DB |
| total 1 peptides |  |  |  |  |  |  |  |  |  |  |  |  |  |  |  |  |  |  |  |  |  |  |  |  |  |  |  |  |  |  |  |
| --- | --- | --- | --- | --- | --- | --- | --- | --- | --- | --- | --- | --- | --- | --- | --- | --- | --- | --- | --- | --- | --- | --- | --- | --- | --- | --- | --- | --- | --- | --- | --- |

P06702|S10A9\_HUMAN

back to list

  

| Protein Coverage
| Supporting Peptides
|

Protein Coverage:

Supporting Peptides:

| Peptide | Uniq | -10lgP | Mass | Length | ppm | m/z | z | RT | Fraction | Scan | Source File | Area F1 | Area F2 | Area F3 | Area F4 | Area F5 | Area F6 | Area F7 | #Feature | #Feature F1 | #Feature F2 | #Feature F3 | #Feature F4 | #Feature F5 | #Feature F6 | #Feature F7 | Start | End | PTM | AScore | Found By |
| --- | --- | --- | --- | --- | --- | --- | --- | --- | --- | --- | --- | --- | --- | --- | --- | --- | --- | --- | --- | --- | --- | --- | --- | --- | --- | --- | --- | --- | --- | --- | --- |
| R.NIETIINTFHQYSVK.L | Y | 60.66 | 1805.9312 | 15 | 0.9 | 602.9849 | 3 | 70.04 | 1 | F1:20537 | Fraction\_1\_23092022r.raw | 1.7372E5 |  |  |  |  |  |  | 1 | 1 | 0 | 0 | 0 | 0 | 0 | 0 | 11 | 25 |  |  | PEAKS DB |
| total 1 peptides |  |  |  |  |  |  |  |  |  |  |  |  |  |  |  |  |  |  |  |  |  |  |  |  |  |  |  |  |  |  |  |
| --- | --- | --- | --- | --- | --- | --- | --- | --- | --- | --- | --- | --- | --- | --- | --- | --- | --- | --- | --- | --- | --- | --- | --- | --- | --- | --- | --- | --- | --- | --- | --- |

Q5T749|KPRP\_HUMAN

back to list

  

| Protein Coverage
| Supporting Peptides
|

Protein Coverage:

Supporting Peptides:

| Peptide | Uniq | -10lgP | Mass | Length | ppm | m/z | z | RT | Fraction | Scan | Source File | Area F1 | Area F2 | Area F3 | Area F4 | Area F5 | Area F6 | Area F7 | #Feature | #Feature F1 | #Feature F2 | #Feature F3 | #Feature F4 | #Feature F5 | #Feature F6 | #Feature F7 | Start | End | PTM | AScore | Found By |
| --- | --- | --- | --- | --- | --- | --- | --- | --- | --- | --- | --- | --- | --- | --- | --- | --- | --- | --- | --- | --- | --- | --- | --- | --- | --- | --- | --- | --- | --- | --- | --- |
| R.TSFSPC(+57.02)VPQC(+57.02)QTQGSYGSFTEQHR.S | Y | 58.89 | 2788.1970 | 24 | 0.9 | 930.4071 | 3 | 47.53 | 1 | F1:10450 | Fraction\_1\_23092022r.raw | 2.8275E5 |  |  |  |  |  |  | 1 | 1 | 0 | 0 | 0 | 0 | 0 | 0 | 225 | 248 | Carbamidomethylation | C6:Carbamidomethylation:1000.00;C10:Carbamidomethylation:1000.00 | PEAKS DB |
| total 1 peptides |  |  |  |  |  |  |  |  |  |  |  |  |  |  |  |  |  |  |  |  |  |  |  |  |  |  |  |  |  |  |  |
| --- | --- | --- | --- | --- | --- | --- | --- | --- | --- | --- | --- | --- | --- | --- | --- | --- | --- | --- | --- | --- | --- | --- | --- | --- | --- | --- | --- | --- | --- | --- | --- |

Q5D862|FILA2\_HUMAN

back to list

  

| Protein Coverage
| Supporting Peptides
|

Protein Coverage:

Supporting Peptides:

| Peptide | Uniq | -10lgP | Mass | Length | ppm | m/z | z | RT | Fraction | Scan | Source File | Area F1 | Area F2 | Area F3 | Area F4 | Area F5 | Area F6 | Area F7 | #Feature | #Feature F1 | #Feature F2 | #Feature F3 | #Feature F4 | #Feature F5 | #Feature F6 | #Feature F7 | Start | End | PTM | AScore | Found By |
| --- | --- | --- | --- | --- | --- | --- | --- | --- | --- | --- | --- | --- | --- | --- | --- | --- | --- | --- | --- | --- | --- | --- | --- | --- | --- | --- | --- | --- | --- | --- | --- |
| R.SVVTVIDVFYK.Y | Y | 57.75 | 1268.7017 | 11 | -0.2 | 635.3580 | 2 | 76.64 | 3 | F3:24038 | Fraction\_3\_23092022.raw | 3.9196E5 |  | 1.3817E5 |  |  |  |  | 2 | 1 | 0 | 1 | 0 | 0 | 0 | 0 | 7 | 17 |  |  | PEAKS DB |
| total 1 peptides |  |  |  |  |  |  |  |  |  |  |  |  |  |  |  |  |  |  |  |  |  |  |  |  |  |  |  |  |  |  |  |
| --- | --- | --- | --- | --- | --- | --- | --- | --- | --- | --- | --- | --- | --- | --- | --- | --- | --- | --- | --- | --- | --- | --- | --- | --- | --- | --- | --- | --- | --- | --- | --- |

P07477|TRY1\_HUMAN

back to list

  

| Protein Coverage
| Supporting Peptides
|

Protein Coverage:

Supporting Peptides:

| Peptide | Uniq | -10lgP | Mass | Length | ppm | m/z | z | RT | Fraction | Scan | Source File | Area F1 | Area F2 | Area F3 | Area F4 | Area F5 | Area F6 | Area F7 | #Feature | #Feature F1 | #Feature F2 | #Feature F3 | #Feature F4 | #Feature F5 | #Feature F6 | #Feature F7 | Start | End | PTM | AScore | Found By |
| --- | --- | --- | --- | --- | --- | --- | --- | --- | --- | --- | --- | --- | --- | --- | --- | --- | --- | --- | --- | --- | --- | --- | --- | --- | --- | --- | --- | --- | --- | --- | --- |
| K.TLNNDIMLIK.L | Y | 57.43 | 1173.6427 | 10 | 0.4 | 587.8289 | 2 | 57.88 | 4 | F4:16144 | Fraction\_4\_23092022.raw | 1.0805E9 |  | 8.4451E5 | 4.535E6 | 1.3832E6 |  |  | 4 | 1 | 0 | 1 | 1 | 1 | 0 | 0 | 103 | 112 |  |  | PEAKS DB |
| K.TLNNDIM(+15.99)LIK.L | Y | 56.92 | 1189.6377 | 10 | -0.5 | 595.8258 | 2 | 50.96 | 5 | F5:12811 | Fraction\_5\_23092022.raw | 1.6613E9 | 1.8884E8 | 5.1004E7 | 7.0393E7 | 7.6514E7 | 6.0169E7 | 4.1853E7 | 8 | 2 | 1 | 1 | 1 | 1 | 1 | 1 | 103 | 112 | Oxidation (M) | M7:Oxidation (M):1000.00 | PEAKS DB |
| total 2 peptides |  |  |  |  |  |  |  |  |  |  |  |  |  |  |  |  |  |  |  |  |  |  |  |  |  |  |  |  |  |  |  |
| --- | --- | --- | --- | --- | --- | --- | --- | --- | --- | --- | --- | --- | --- | --- | --- | --- | --- | --- | --- | --- | --- | --- | --- | --- | --- | --- | --- | --- | --- | --- | --- |

P00763|TRY2\_RAT

back to list

  

| Protein Coverage
| Supporting Peptides
|

Protein Coverage:

Supporting Peptides:

| Peptide | Uniq | -10lgP | Mass | Length | ppm | m/z | z | RT | Fraction | Scan | Source File | Area F1 | Area F2 | Area F3 | Area F4 | Area F5 | Area F6 | Area F7 | #Feature | #Feature F1 | #Feature F2 | #Feature F3 | #Feature F4 | #Feature F5 | #Feature F6 | #Feature F7 | Start | End | PTM | AScore | Found By |
| --- | --- | --- | --- | --- | --- | --- | --- | --- | --- | --- | --- | --- | --- | --- | --- | --- | --- | --- | --- | --- | --- | --- | --- | --- | --- | --- | --- | --- | --- | --- | --- |
| K.TLNNDIMLIK.L | Y | 57.43 | 1173.6427 | 10 | 0.4 | 587.8289 | 2 | 57.88 | 4 | F4:16144 | Fraction\_4\_23092022.raw | 1.0805E9 |  | 8.4451E5 | 4.535E6 | 1.3832E6 |  |  | 4 | 1 | 0 | 1 | 1 | 1 | 0 | 0 | 103 | 112 |  |  | PEAKS DB |
| K.TLNNDIM(+15.99)LIK.L | Y | 56.92 | 1189.6377 | 10 | -0.5 | 595.8258 | 2 | 50.96 | 5 | F5:12811 | Fraction\_5\_23092022.raw | 1.6613E9 | 1.8884E8 | 5.1004E7 | 7.0393E7 | 7.6514E7 | 6.0169E7 | 4.1853E7 | 8 | 2 | 1 | 1 | 1 | 1 | 1 | 1 | 103 | 112 | Oxidation (M) | M7:Oxidation (M):1000.00 | PEAKS DB |
| total 2 peptides |  |  |  |  |  |  |  |  |  |  |  |  |  |  |  |  |  |  |  |  |  |  |  |  |  |  |  |  |  |  |  |
| --- | --- | --- | --- | --- | --- | --- | --- | --- | --- | --- | --- | --- | --- | --- | --- | --- | --- | --- | --- | --- | --- | --- | --- | --- | --- | --- | --- | --- | --- | --- | --- |

TRINITY\_DN3717\_c3\_g1\_i1.p1

back to list

  

| Protein Coverage
| Supporting Peptides
|

Protein Coverage:

Supporting Peptides:

| Peptide | Uniq | -10lgP | Mass | Length | ppm | m/z | z | RT | Fraction | Scan | Source File | Area F1 | Area F2 | Area F3 | Area F4 | Area F5 | Area F6 | Area F7 | #Feature | #Feature F1 | #Feature F2 | #Feature F3 | #Feature F4 | #Feature F5 | #Feature F6 | #Feature F7 | Start | End | PTM | AScore | Found By |
| --- | --- | --- | --- | --- | --- | --- | --- | --- | --- | --- | --- | --- | --- | --- | --- | --- | --- | --- | --- | --- | --- | --- | --- | --- | --- | --- | --- | --- | --- | --- | --- |
| K.GDVNTQIALGPALK.F | Y | 57.17 | 1395.7721 | 14 | -0.1 | 698.8932 | 2 | 53.06 | 6 | F6:14440 | Fraction\_6\_23092022.raw |  |  |  |  |  | 1.1528E5 |  | 1 | 0 | 0 | 0 | 0 | 0 | 1 | 0 | 72 | 85 |  |  | PEAKS DB |
| total 1 peptides |  |  |  |  |  |  |  |  |  |  |  |  |  |  |  |  |  |  |  |  |  |  |  |  |  |  |  |  |  |  |  |
| --- | --- | --- | --- | --- | --- | --- | --- | --- | --- | --- | --- | --- | --- | --- | --- | --- | --- | --- | --- | --- | --- | --- | --- | --- | --- | --- | --- | --- | --- | --- | --- |

Q8NHM4|TRY6\_HUMAN

back to list

  

| Protein Coverage
| Supporting Peptides
|

Protein Coverage:

Supporting Peptides:

| Peptide | Uniq | -10lgP | Mass | Length | ppm | m/z | z | RT | Fraction | Scan | Source File | Area F1 | Area F2 | Area F3 | Area F4 | Area F5 | Area F6 | Area F7 | #Feature | #Feature F1 | #Feature F2 | #Feature F3 | #Feature F4 | #Feature F5 | #Feature F6 | #Feature F7 | Start | End | PTM | AScore | Found By |
| --- | --- | --- | --- | --- | --- | --- | --- | --- | --- | --- | --- | --- | --- | --- | --- | --- | --- | --- | --- | --- | --- | --- | --- | --- | --- | --- | --- | --- | --- | --- | --- |
| K.DSC(+57.02)QGDSGGPVVC(+57.02)NGQLQGIVSWGYGC(+57.02)AQKR.R | Y | 56.51 | 3339.4819 | 31 | 5.7 | 1114.1743 | 3 | 55.52 | 1 | F1:14028 | Fraction\_1\_23092022r.raw | 1.6515E6 |  |  |  |  |  |  | 2 | 2 | 0 | 0 | 0 | 0 | 0 | 0 | 194 | 224 | Carbamidomethylation | C3:Carbamidomethylation:1000.00;C13:Carbamidomethylation:1000.00;C27:Carbamidomethylation:1000.00 | PEAKS DB |
| total 1 peptides |  |  |  |  |  |  |  |  |  |  |  |  |  |  |  |  |  |  |  |  |  |  |  |  |  |  |  |  |  |  |  |
| --- | --- | --- | --- | --- | --- | --- | --- | --- | --- | --- | --- | --- | --- | --- | --- | --- | --- | --- | --- | --- | --- | --- | --- | --- | --- | --- | --- | --- | --- | --- | --- |

Q28417|CASK\_GIRCA

back to list

  

| Protein Coverage
| Supporting Peptides
|

Protein Coverage:

Supporting Peptides:

| Peptide | Uniq | -10lgP | Mass | Length | ppm | m/z | z | RT | Fraction | Scan | Source File | Area F1 | Area F2 | Area F3 | Area F4 | Area F5 | Area F6 | Area F7 | #Feature | #Feature F1 | #Feature F2 | #Feature F3 | #Feature F4 | #Feature F5 | #Feature F6 | #Feature F7 | Start | End | PTM | AScore | Found By |
| --- | --- | --- | --- | --- | --- | --- | --- | --- | --- | --- | --- | --- | --- | --- | --- | --- | --- | --- | --- | --- | --- | --- | --- | --- | --- | --- | --- | --- | --- | --- | --- |
| K.YIPIQYVLSR.Y | Y | 56.05 | 1250.7023 | 10 | 0.2 | 626.3585 | 2 | 68.89 | 5 | F5:20866 | Fraction\_5\_23092022.raw | 2.3545E5 | 1.2772E5 |  | 7.2711E4 | 2.7997E5 | 2.3126E5 |  | 5 | 1 | 1 | 0 | 1 | 1 | 1 | 0 | 9 | 18 |  |  | PEAKS DB |
| total 1 peptides |  |  |  |  |  |  |  |  |  |  |  |  |  |  |  |  |  |  |  |  |  |  |  |  |  |  |  |  |  |  |  |
| --- | --- | --- | --- | --- | --- | --- | --- | --- | --- | --- | --- | --- | --- | --- | --- | --- | --- | --- | --- | --- | --- | --- | --- | --- | --- | --- | --- | --- | --- | --- | --- |

P11840|CASK\_BUBBU

back to list

  

| Protein Coverage
| Supporting Peptides
|

Protein Coverage:

Supporting Peptides:

| Peptide | Uniq | -10lgP | Mass | Length | ppm | m/z | z | RT | Fraction | Scan | Source File | Area F1 | Area F2 | Area F3 | Area F4 | Area F5 | Area F6 | Area F7 | #Feature | #Feature F1 | #Feature F2 | #Feature F3 | #Feature F4 | #Feature F5 | #Feature F6 | #Feature F7 | Start | End | PTM | AScore | Found By |
| --- | --- | --- | --- | --- | --- | --- | --- | --- | --- | --- | --- | --- | --- | --- | --- | --- | --- | --- | --- | --- | --- | --- | --- | --- | --- | --- | --- | --- | --- | --- | --- |
| K.YIPIQYVLSR.Y | Y | 56.05 | 1250.7023 | 10 | 0.2 | 626.3585 | 2 | 68.89 | 5 | F5:20866 | Fraction\_5\_23092022.raw | 2.3545E5 | 1.2772E5 |  | 7.2711E4 | 2.7997E5 | 2.3126E5 |  | 5 | 1 | 1 | 0 | 1 | 1 | 1 | 0 | 46 | 55 |  |  | PEAKS DB |
| total 1 peptides |  |  |  |  |  |  |  |  |  |  |  |  |  |  |  |  |  |  |  |  |  |  |  |  |  |  |  |  |  |  |  |
| --- | --- | --- | --- | --- | --- | --- | --- | --- | --- | --- | --- | --- | --- | --- | --- | --- | --- | --- | --- | --- | --- | --- | --- | --- | --- | --- | --- | --- | --- | --- | --- |

P02668|CASK\_BOVIN

back to list

  

| Protein Coverage
| Supporting Peptides
|

Protein Coverage:

Supporting Peptides:

| Peptide | Uniq | -10lgP | Mass | Length | ppm | m/z | z | RT | Fraction | Scan | Source File | Area F1 | Area F2 | Area F3 | Area F4 | Area F5 | Area F6 | Area F7 | #Feature | #Feature F1 | #Feature F2 | #Feature F3 | #Feature F4 | #Feature F5 | #Feature F6 | #Feature F7 | Start | End | PTM | AScore | Found By |
| --- | --- | --- | --- | --- | --- | --- | --- | --- | --- | --- | --- | --- | --- | --- | --- | --- | --- | --- | --- | --- | --- | --- | --- | --- | --- | --- | --- | --- | --- | --- | --- |
| K.YIPIQYVLSR.Y | Y | 56.05 | 1250.7023 | 10 | 0.2 | 626.3585 | 2 | 68.89 | 5 | F5:20866 | Fraction\_5\_23092022.raw | 2.3545E5 | 1.2772E5 |  | 7.2711E4 | 2.7997E5 | 2.3126E5 |  | 5 | 1 | 1 | 0 | 1 | 1 | 1 | 0 | 46 | 55 |  |  | PEAKS DB |
| total 1 peptides |  |  |  |  |  |  |  |  |  |  |  |  |  |  |  |  |  |  |  |  |  |  |  |  |  |  |  |  |  |  |  |
| --- | --- | --- | --- | --- | --- | --- | --- | --- | --- | --- | --- | --- | --- | --- | --- | --- | --- | --- | --- | --- | --- | --- | --- | --- | --- | --- | --- | --- | --- | --- | --- |

P50420|CASK\_CAPSU

back to list

  

| Protein Coverage
| Supporting Peptides
|

Protein Coverage:

Supporting Peptides:

| Peptide | Uniq | -10lgP | Mass | Length | ppm | m/z | z | RT | Fraction | Scan | Source File | Area F1 | Area F2 | Area F3 | Area F4 | Area F5 | Area F6 | Area F7 | #Feature | #Feature F1 | #Feature F2 | #Feature F3 | #Feature F4 | #Feature F5 | #Feature F6 | #Feature F7 | Start | End | PTM | AScore | Found By |
| --- | --- | --- | --- | --- | --- | --- | --- | --- | --- | --- | --- | --- | --- | --- | --- | --- | --- | --- | --- | --- | --- | --- | --- | --- | --- | --- | --- | --- | --- | --- | --- |
| K.YIPIQYVLSR.Y | Y | 56.05 | 1250.7023 | 10 | 0.2 | 626.3585 | 2 | 68.89 | 5 | F5:20866 | Fraction\_5\_23092022.raw | 2.3545E5 | 1.2772E5 |  | 7.2711E4 | 2.7997E5 | 2.3126E5 |  | 5 | 1 | 1 | 0 | 1 | 1 | 1 | 0 | 46 | 55 |  |  | PEAKS DB |
| total 1 peptides |  |  |  |  |  |  |  |  |  |  |  |  |  |  |  |  |  |  |  |  |  |  |  |  |  |  |  |  |  |  |  |
| --- | --- | --- | --- | --- | --- | --- | --- | --- | --- | --- | --- | --- | --- | --- | --- | --- | --- | --- | --- | --- | --- | --- | --- | --- | --- | --- | --- | --- | --- | --- | --- |

P50423|CASK\_OREAM

back to list

  

| Protein Coverage
| Supporting Peptides
|

Protein Coverage:

Supporting Peptides:

| Peptide | Uniq | -10lgP | Mass | Length | ppm | m/z | z | RT | Fraction | Scan | Source File | Area F1 | Area F2 | Area F3 | Area F4 | Area F5 | Area F6 | Area F7 | #Feature | #Feature F1 | #Feature F2 | #Feature F3 | #Feature F4 | #Feature F5 | #Feature F6 | #Feature F7 | Start | End | PTM | AScore | Found By |
| --- | --- | --- | --- | --- | --- | --- | --- | --- | --- | --- | --- | --- | --- | --- | --- | --- | --- | --- | --- | --- | --- | --- | --- | --- | --- | --- | --- | --- | --- | --- | --- |
| K.YIPIQYVLSR.Y | Y | 56.05 | 1250.7023 | 10 | 0.2 | 626.3585 | 2 | 68.89 | 5 | F5:20866 | Fraction\_5\_23092022.raw | 2.3545E5 | 1.2772E5 |  | 7.2711E4 | 2.7997E5 | 2.3126E5 |  | 5 | 1 | 1 | 0 | 1 | 1 | 1 | 0 | 46 | 55 |  |  | PEAKS DB |
| total 1 peptides |  |  |  |  |  |  |  |  |  |  |  |  |  |  |  |  |  |  |  |  |  |  |  |  |  |  |  |  |  |  |  |
| --- | --- | --- | --- | --- | --- | --- | --- | --- | --- | --- | --- | --- | --- | --- | --- | --- | --- | --- | --- | --- | --- | --- | --- | --- | --- | --- | --- | --- | --- | --- | --- |

P50422|CASK\_NEMGO

back to list

  

| Protein Coverage
| Supporting Peptides
|

Protein Coverage:

Supporting Peptides:

| Peptide | Uniq | -10lgP | Mass | Length | ppm | m/z | z | RT | Fraction | Scan | Source File | Area F1 | Area F2 | Area F3 | Area F4 | Area F5 | Area F6 | Area F7 | #Feature | #Feature F1 | #Feature F2 | #Feature F3 | #Feature F4 | #Feature F5 | #Feature F6 | #Feature F7 | Start | End | PTM | AScore | Found By |
| --- | --- | --- | --- | --- | --- | --- | --- | --- | --- | --- | --- | --- | --- | --- | --- | --- | --- | --- | --- | --- | --- | --- | --- | --- | --- | --- | --- | --- | --- | --- | --- |
| K.YIPIQYVLSR.Y | Y | 56.05 | 1250.7023 | 10 | 0.2 | 626.3585 | 2 | 68.89 | 5 | F5:20866 | Fraction\_5\_23092022.raw | 2.3545E5 | 1.2772E5 |  | 7.2711E4 | 2.7997E5 | 2.3126E5 |  | 5 | 1 | 1 | 0 | 1 | 1 | 1 | 0 | 46 | 55 |  |  | PEAKS DB |
| total 1 peptides |  |  |  |  |  |  |  |  |  |  |  |  |  |  |  |  |  |  |  |  |  |  |  |  |  |  |  |  |  |  |  |
| --- | --- | --- | --- | --- | --- | --- | --- | --- | --- | --- | --- | --- | --- | --- | --- | --- | --- | --- | --- | --- | --- | --- | --- | --- | --- | --- | --- | --- | --- | --- | --- |

P50421|CASK\_CAPSW

back to list

  

| Protein Coverage
| Supporting Peptides
|

Protein Coverage:

Supporting Peptides:

| Peptide | Uniq | -10lgP | Mass | Length | ppm | m/z | z | RT | Fraction | Scan | Source File | Area F1 | Area F2 | Area F3 | Area F4 | Area F5 | Area F6 | Area F7 | #Feature | #Feature F1 | #Feature F2 | #Feature F3 | #Feature F4 | #Feature F5 | #Feature F6 | #Feature F7 | Start | End | PTM | AScore | Found By |
| --- | --- | --- | --- | --- | --- | --- | --- | --- | --- | --- | --- | --- | --- | --- | --- | --- | --- | --- | --- | --- | --- | --- | --- | --- | --- | --- | --- | --- | --- | --- | --- |
| K.YIPIQYVLSR.Y | Y | 56.05 | 1250.7023 | 10 | 0.2 | 626.3585 | 2 | 68.89 | 5 | F5:20866 | Fraction\_5\_23092022.raw | 2.3545E5 | 1.2772E5 |  | 7.2711E4 | 2.7997E5 | 2.3126E5 |  | 5 | 1 | 1 | 0 | 1 | 1 | 1 | 0 | 46 | 55 |  |  | PEAKS DB |
| total 1 peptides |  |  |  |  |  |  |  |  |  |  |  |  |  |  |  |  |  |  |  |  |  |  |  |  |  |  |  |  |  |  |  |
| --- | --- | --- | --- | --- | --- | --- | --- | --- | --- | --- | --- | --- | --- | --- | --- | --- | --- | --- | --- | --- | --- | --- | --- | --- | --- | --- | --- | --- | --- | --- | --- |

P02670|CASK\_CAPHI

back to list

  

| Protein Coverage
| Supporting Peptides
|

Protein Coverage:

Supporting Peptides:

| Peptide | Uniq | -10lgP | Mass | Length | ppm | m/z | z | RT | Fraction | Scan | Source File | Area F1 | Area F2 | Area F3 | Area F4 | Area F5 | Area F6 | Area F7 | #Feature | #Feature F1 | #Feature F2 | #Feature F3 | #Feature F4 | #Feature F5 | #Feature F6 | #Feature F7 | Start | End | PTM | AScore | Found By |
| --- | --- | --- | --- | --- | --- | --- | --- | --- | --- | --- | --- | --- | --- | --- | --- | --- | --- | --- | --- | --- | --- | --- | --- | --- | --- | --- | --- | --- | --- | --- | --- |
| K.YIPIQYVLSR.Y | Y | 56.05 | 1250.7023 | 10 | 0.2 | 626.3585 | 2 | 68.89 | 5 | F5:20866 | Fraction\_5\_23092022.raw | 2.3545E5 | 1.2772E5 |  | 7.2711E4 | 2.7997E5 | 2.3126E5 |  | 5 | 1 | 1 | 0 | 1 | 1 | 1 | 0 | 46 | 55 |  |  | PEAKS DB |
| total 1 peptides |  |  |  |  |  |  |  |  |  |  |  |  |  |  |  |  |  |  |  |  |  |  |  |  |  |  |  |  |  |  |  |
| --- | --- | --- | --- | --- | --- | --- | --- | --- | --- | --- | --- | --- | --- | --- | --- | --- | --- | --- | --- | --- | --- | --- | --- | --- | --- | --- | --- | --- | --- | --- | --- |

P50424|CASK\_RUPRU

back to list

  

| Protein Coverage
| Supporting Peptides
|

Protein Coverage:

Supporting Peptides:

| Peptide | Uniq | -10lgP | Mass | Length | ppm | m/z | z | RT | Fraction | Scan | Source File | Area F1 | Area F2 | Area F3 | Area F4 | Area F5 | Area F6 | Area F7 | #Feature | #Feature F1 | #Feature F2 | #Feature F3 | #Feature F4 | #Feature F5 | #Feature F6 | #Feature F7 | Start | End | PTM | AScore | Found By |
| --- | --- | --- | --- | --- | --- | --- | --- | --- | --- | --- | --- | --- | --- | --- | --- | --- | --- | --- | --- | --- | --- | --- | --- | --- | --- | --- | --- | --- | --- | --- | --- |
| K.YIPIQYVLSR.Y | Y | 56.05 | 1250.7023 | 10 | 0.2 | 626.3585 | 2 | 68.89 | 5 | F5:20866 | Fraction\_5\_23092022.raw | 2.3545E5 | 1.2772E5 |  | 7.2711E4 | 2.7997E5 | 2.3126E5 |  | 5 | 1 | 1 | 0 | 1 | 1 | 1 | 0 | 46 | 55 |  |  | PEAKS DB |
| total 1 peptides |  |  |  |  |  |  |  |  |  |  |  |  |  |  |  |  |  |  |  |  |  |  |  |  |  |  |  |  |  |  |  |
| --- | --- | --- | --- | --- | --- | --- | --- | --- | --- | --- | --- | --- | --- | --- | --- | --- | --- | --- | --- | --- | --- | --- | --- | --- | --- | --- | --- | --- | --- | --- | --- |

P42156|CASK\_CAPCR

back to list

  

| Protein Coverage
| Supporting Peptides
|

Protein Coverage:

Supporting Peptides:

| Peptide | Uniq | -10lgP | Mass | Length | ppm | m/z | z | RT | Fraction | Scan | Source File | Area F1 | Area F2 | Area F3 | Area F4 | Area F5 | Area F6 | Area F7 | #Feature | #Feature F1 | #Feature F2 | #Feature F3 | #Feature F4 | #Feature F5 | #Feature F6 | #Feature F7 | Start | End | PTM | AScore | Found By |
| --- | --- | --- | --- | --- | --- | --- | --- | --- | --- | --- | --- | --- | --- | --- | --- | --- | --- | --- | --- | --- | --- | --- | --- | --- | --- | --- | --- | --- | --- | --- | --- |
| K.YIPIQYVLSR.Y | Y | 56.05 | 1250.7023 | 10 | 0.2 | 626.3585 | 2 | 68.89 | 5 | F5:20866 | Fraction\_5\_23092022.raw | 2.3545E5 | 1.2772E5 |  | 7.2711E4 | 2.7997E5 | 2.3126E5 |  | 5 | 1 | 1 | 0 | 1 | 1 | 1 | 0 | 46 | 55 |  |  | PEAKS DB |
| total 1 peptides |  |  |  |  |  |  |  |  |  |  |  |  |  |  |  |  |  |  |  |  |  |  |  |  |  |  |  |  |  |  |  |
| --- | --- | --- | --- | --- | --- | --- | --- | --- | --- | --- | --- | --- | --- | --- | --- | --- | --- | --- | --- | --- | --- | --- | --- | --- | --- | --- | --- | --- | --- | --- | --- |

P02669|CASK\_SHEEP

back to list

  

| Protein Coverage
| Supporting Peptides
|

Protein Coverage:

Supporting Peptides:

| Peptide | Uniq | -10lgP | Mass | Length | ppm | m/z | z | RT | Fraction | Scan | Source File | Area F1 | Area F2 | Area F3 | Area F4 | Area F5 | Area F6 | Area F7 | #Feature | #Feature F1 | #Feature F2 | #Feature F3 | #Feature F4 | #Feature F5 | #Feature F6 | #Feature F7 | Start | End | PTM | AScore | Found By |
| --- | --- | --- | --- | --- | --- | --- | --- | --- | --- | --- | --- | --- | --- | --- | --- | --- | --- | --- | --- | --- | --- | --- | --- | --- | --- | --- | --- | --- | --- | --- | --- |
| K.YIPIQYVLSR.Y | Y | 56.05 | 1250.7023 | 10 | 0.2 | 626.3585 | 2 | 68.89 | 5 | F5:20866 | Fraction\_5\_23092022.raw | 2.3545E5 | 1.2772E5 |  | 7.2711E4 | 2.7997E5 | 2.3126E5 |  | 5 | 1 | 1 | 0 | 1 | 1 | 1 | 0 | 46 | 55 |  |  | PEAKS DB |
| total 1 peptides |  |  |  |  |  |  |  |  |  |  |  |  |  |  |  |  |  |  |  |  |  |  |  |  |  |  |  |  |  |  |  |
| --- | --- | --- | --- | --- | --- | --- | --- | --- | --- | --- | --- | --- | --- | --- | --- | --- | --- | --- | --- | --- | --- | --- | --- | --- | --- | --- | --- | --- | --- | --- | --- |

P50425|CASK\_SAITA

back to list

  

| Protein Coverage
| Supporting Peptides
|

Protein Coverage:

Supporting Peptides:

| Peptide | Uniq | -10lgP | Mass | Length | ppm | m/z | z | RT | Fraction | Scan | Source File | Area F1 | Area F2 | Area F3 | Area F4 | Area F5 | Area F6 | Area F7 | #Feature | #Feature F1 | #Feature F2 | #Feature F3 | #Feature F4 | #Feature F5 | #Feature F6 | #Feature F7 | Start | End | PTM | AScore | Found By |
| --- | --- | --- | --- | --- | --- | --- | --- | --- | --- | --- | --- | --- | --- | --- | --- | --- | --- | --- | --- | --- | --- | --- | --- | --- | --- | --- | --- | --- | --- | --- | --- |
| K.YIPIQYVLSR.Y | Y | 56.05 | 1250.7023 | 10 | 0.2 | 626.3585 | 2 | 68.89 | 5 | F5:20866 | Fraction\_5\_23092022.raw | 2.3545E5 | 1.2772E5 |  | 7.2711E4 | 2.7997E5 | 2.3126E5 |  | 5 | 1 | 1 | 0 | 1 | 1 | 1 | 0 | 46 | 55 |  |  | PEAKS DB |
| total 1 peptides |  |  |  |  |  |  |  |  |  |  |  |  |  |  |  |  |  |  |  |  |  |  |  |  |  |  |  |  |  |  |  |
| --- | --- | --- | --- | --- | --- | --- | --- | --- | --- | --- | --- | --- | --- | --- | --- | --- | --- | --- | --- | --- | --- | --- | --- | --- | --- | --- | --- | --- | --- | --- | --- |

TRINITY\_DN1101\_c0\_g1\_i12.p1

back to list

  

| Protein Coverage
| Supporting Peptides
|

Protein Coverage:

Supporting Peptides:

| Peptide | Uniq | -10lgP | Mass | Length | ppm | m/z | z | RT | Fraction | Scan | Source File | Area F1 | Area F2 | Area F3 | Area F4 | Area F5 | Area F6 | Area F7 | #Feature | #Feature F1 | #Feature F2 | #Feature F3 | #Feature F4 | #Feature F5 | #Feature F6 | #Feature F7 | Start | End | PTM | AScore | Found By |
| --- | --- | --- | --- | --- | --- | --- | --- | --- | --- | --- | --- | --- | --- | --- | --- | --- | --- | --- | --- | --- | --- | --- | --- | --- | --- | --- | --- | --- | --- | --- | --- |
| K.VAVLDFVTPSPR.G | Y | 56.04 | 1299.7186 | 12 | 0.5 | 650.8669 | 2 | 66.89 | 3 | F3:19685 | Fraction\_3\_23092022.raw |  |  | 1.0731E5 |  |  |  |  | 1 | 0 | 0 | 1 | 0 | 0 | 0 | 0 | 73 | 84 |  |  | PEAKS DB |
| total 1 peptides |  |  |  |  |  |  |  |  |  |  |  |  |  |  |  |  |  |  |  |  |  |  |  |  |  |  |  |  |  |  |  |
| --- | --- | --- | --- | --- | --- | --- | --- | --- | --- | --- | --- | --- | --- | --- | --- | --- | --- | --- | --- | --- | --- | --- | --- | --- | --- | --- | --- | --- | --- | --- | --- |

TRINITY\_DN1101\_c0\_g1\_i9.p1

back to list

  

| Protein Coverage
| Supporting Peptides
|

Protein Coverage:

Supporting Peptides:

| Peptide | Uniq | -10lgP | Mass | Length | ppm | m/z | z | RT | Fraction | Scan | Source File | Area F1 | Area F2 | Area F3 | Area F4 | Area F5 | Area F6 | Area F7 | #Feature | #Feature F1 | #Feature F2 | #Feature F3 | #Feature F4 | #Feature F5 | #Feature F6 | #Feature F7 | Start | End | PTM | AScore | Found By |
| --- | --- | --- | --- | --- | --- | --- | --- | --- | --- | --- | --- | --- | --- | --- | --- | --- | --- | --- | --- | --- | --- | --- | --- | --- | --- | --- | --- | --- | --- | --- | --- |
| K.VAVLDFVTPSPR.G | Y | 56.04 | 1299.7186 | 12 | 0.5 | 650.8669 | 2 | 66.89 | 3 | F3:19685 | Fraction\_3\_23092022.raw |  |  | 1.0731E5 |  |  |  |  | 1 | 0 | 0 | 1 | 0 | 0 | 0 | 0 | 73 | 84 |  |  | PEAKS DB |
| total 1 peptides |  |  |  |  |  |  |  |  |  |  |  |  |  |  |  |  |  |  |  |  |  |  |  |  |  |  |  |  |  |  |  |
| --- | --- | --- | --- | --- | --- | --- | --- | --- | --- | --- | --- | --- | --- | --- | --- | --- | --- | --- | --- | --- | --- | --- | --- | --- | --- | --- | --- | --- | --- | --- | --- |

TRINITY\_DN1101\_c0\_g1\_i15.p1

back to list

  

| Protein Coverage
| Supporting Peptides
|

Protein Coverage:

Supporting Peptides:

| Peptide | Uniq | -10lgP | Mass | Length | ppm | m/z | z | RT | Fraction | Scan | Source File | Area F1 | Area F2 | Area F3 | Area F4 | Area F5 | Area F6 | Area F7 | #Feature | #Feature F1 | #Feature F2 | #Feature F3 | #Feature F4 | #Feature F5 | #Feature F6 | #Feature F7 | Start | End | PTM | AScore | Found By |
| --- | --- | --- | --- | --- | --- | --- | --- | --- | --- | --- | --- | --- | --- | --- | --- | --- | --- | --- | --- | --- | --- | --- | --- | --- | --- | --- | --- | --- | --- | --- | --- |
| K.VAVLDFVTPSPR.G | Y | 56.04 | 1299.7186 | 12 | 0.5 | 650.8669 | 2 | 66.89 | 3 | F3:19685 | Fraction\_3\_23092022.raw |  |  | 1.0731E5 |  |  |  |  | 1 | 0 | 0 | 1 | 0 | 0 | 0 | 0 | 73 | 84 |  |  | PEAKS DB |
| total 1 peptides |  |  |  |  |  |  |  |  |  |  |  |  |  |  |  |  |  |  |  |  |  |  |  |  |  |  |  |  |  |  |  |
| --- | --- | --- | --- | --- | --- | --- | --- | --- | --- | --- | --- | --- | --- | --- | --- | --- | --- | --- | --- | --- | --- | --- | --- | --- | --- | --- | --- | --- | --- | --- | --- |

TRINITY\_DN1101\_c0\_g1\_i17.p1

back to list

  

| Protein Coverage
| Supporting Peptides
|

Protein Coverage:

Supporting Peptides:

| Peptide | Uniq | -10lgP | Mass | Length | ppm | m/z | z | RT | Fraction | Scan | Source File | Area F1 | Area F2 | Area F3 | Area F4 | Area F5 | Area F6 | Area F7 | #Feature | #Feature F1 | #Feature F2 | #Feature F3 | #Feature F4 | #Feature F5 | #Feature F6 | #Feature F7 | Start | End | PTM | AScore | Found By |
| --- | --- | --- | --- | --- | --- | --- | --- | --- | --- | --- | --- | --- | --- | --- | --- | --- | --- | --- | --- | --- | --- | --- | --- | --- | --- | --- | --- | --- | --- | --- | --- |
| K.VAVLDFVTPSPR.G | Y | 56.04 | 1299.7186 | 12 | 0.5 | 650.8669 | 2 | 66.89 | 3 | F3:19685 | Fraction\_3\_23092022.raw |  |  | 1.0731E5 |  |  |  |  | 1 | 0 | 0 | 1 | 0 | 0 | 0 | 0 | 73 | 84 |  |  | PEAKS DB |
| total 1 peptides |  |  |  |  |  |  |  |  |  |  |  |  |  |  |  |  |  |  |  |  |  |  |  |  |  |  |  |  |  |  |  |
| --- | --- | --- | --- | --- | --- | --- | --- | --- | --- | --- | --- | --- | --- | --- | --- | --- | --- | --- | --- | --- | --- | --- | --- | --- | --- | --- | --- | --- | --- | --- | --- |

TRINITY\_DN1101\_c0\_g1\_i11.p1

back to list

  

| Protein Coverage
| Supporting Peptides
|

Protein Coverage:

Supporting Peptides:

| Peptide | Uniq | -10lgP | Mass | Length | ppm | m/z | z | RT | Fraction | Scan | Source File | Area F1 | Area F2 | Area F3 | Area F4 | Area F5 | Area F6 | Area F7 | #Feature | #Feature F1 | #Feature F2 | #Feature F3 | #Feature F4 | #Feature F5 | #Feature F6 | #Feature F7 | Start | End | PTM | AScore | Found By |
| --- | --- | --- | --- | --- | --- | --- | --- | --- | --- | --- | --- | --- | --- | --- | --- | --- | --- | --- | --- | --- | --- | --- | --- | --- | --- | --- | --- | --- | --- | --- | --- |
| K.VAVLDFVTPSPR.G | Y | 56.04 | 1299.7186 | 12 | 0.5 | 650.8669 | 2 | 66.89 | 3 | F3:19685 | Fraction\_3\_23092022.raw |  |  | 1.0731E5 |  |  |  |  | 1 | 0 | 0 | 1 | 0 | 0 | 0 | 0 | 73 | 84 |  |  | PEAKS DB |
| total 1 peptides |  |  |  |  |  |  |  |  |  |  |  |  |  |  |  |  |  |  |  |  |  |  |  |  |  |  |  |  |  |  |  |
| --- | --- | --- | --- | --- | --- | --- | --- | --- | --- | --- | --- | --- | --- | --- | --- | --- | --- | --- | --- | --- | --- | --- | --- | --- | --- | --- | --- | --- | --- | --- | --- |

Q06833|NVJ2\_YEAST

back to list

  

| Protein Coverage
| Supporting Peptides
|

Protein Coverage:

Supporting Peptides:

| Peptide | Uniq | -10lgP | Mass | Length | ppm | m/z | z | RT | Fraction | Scan | Source File | Area F1 | Area F2 | Area F3 | Area F4 | Area F5 | Area F6 | Area F7 | #Feature | #Feature F1 | #Feature F2 | #Feature F3 | #Feature F4 | #Feature F5 | #Feature F6 | #Feature F7 | Start | End | PTM | AScore | Found By |
| --- | --- | --- | --- | --- | --- | --- | --- | --- | --- | --- | --- | --- | --- | --- | --- | --- | --- | --- | --- | --- | --- | --- | --- | --- | --- | --- | --- | --- | --- | --- | --- |
| K.LEEQLGVKVFNK.G | Y | 56.04 | 1402.7820 | 12 | 7.9 | 468.6050 | 3 | 47.93 | 5 | F5:11466 | Fraction\_5\_23092022.raw |  |  |  | 4.1763E5 | 1.3375E6 |  |  | 4 | 0 | 0 | 0 | 2 | 2 | 0 | 0 | 72 | 83 |  |  | PEAKS DB |
| total 1 peptides |  |  |  |  |  |  |  |  |  |  |  |  |  |  |  |  |  |  |  |  |  |  |  |  |  |  |  |  |  |  |  |
| --- | --- | --- | --- | --- | --- | --- | --- | --- | --- | --- | --- | --- | --- | --- | --- | --- | --- | --- | --- | --- | --- | --- | --- | --- | --- | --- | --- | --- | --- | --- | --- |

TRINITY\_DN13586\_c0\_g1\_i1.p1

back to list

  

| Protein Coverage
| Supporting Peptides
|

Protein Coverage:

Supporting Peptides:

| Peptide | Uniq | -10lgP | Mass | Length | ppm | m/z | z | RT | Fraction | Scan | Source File | Area F1 | Area F2 | Area F3 | Area F4 | Area F5 | Area F6 | Area F7 | #Feature | #Feature F1 | #Feature F2 | #Feature F3 | #Feature F4 | #Feature F5 | #Feature F6 | #Feature F7 | Start | End | PTM | AScore | Found By |
| --- | --- | --- | --- | --- | --- | --- | --- | --- | --- | --- | --- | --- | --- | --- | --- | --- | --- | --- | --- | --- | --- | --- | --- | --- | --- | --- | --- | --- | --- | --- | --- |
| K.EYVAILPVKEIK.E | Y | 52.95 | 1400.8279 | 12 | 0.6 | 467.9502 | 3 | 53.64 | 7 | F7:17578 | Fraction\_7\_23092022.raw |  |  |  |  |  |  | 3.3602E5 | 1 | 0 | 0 | 0 | 0 | 0 | 0 | 1 | 183 | 194 |  |  | PEAKS DB |
| total 1 peptides |  |  |  |  |  |  |  |  |  |  |  |  |  |  |  |  |  |  |  |  |  |  |  |  |  |  |  |  |  |  |  |
| --- | --- | --- | --- | --- | --- | --- | --- | --- | --- | --- | --- | --- | --- | --- | --- | --- | --- | --- | --- | --- | --- | --- | --- | --- | --- | --- | --- | --- | --- | --- | --- |

P02075|HBB\_SHEEP

back to list

  

| Protein Coverage
| Supporting Peptides
|

Protein Coverage:

Supporting Peptides:

| Peptide | Uniq | -10lgP | Mass | Length | ppm | m/z | z | RT | Fraction | Scan | Source File | Area F1 | Area F2 | Area F3 | Area F4 | Area F5 | Area F6 | Area F7 | #Feature | #Feature F1 | #Feature F2 | #Feature F3 | #Feature F4 | #Feature F5 | #Feature F6 | #Feature F7 | Start | End | PTM | AScore | Found By |
| --- | --- | --- | --- | --- | --- | --- | --- | --- | --- | --- | --- | --- | --- | --- | --- | --- | --- | --- | --- | --- | --- | --- | --- | --- | --- | --- | --- | --- | --- | --- | --- |
| K.VVAGVANALAHKYH | Y | 50.77 | 1448.7888 | 14 | -0.9 | 483.9365 | 3 | 34.60 | 5 | F5:5741 | Fraction\_5\_23092022.raw |  |  |  |  | 1.068E5 |  |  | 1 | 0 | 0 | 0 | 0 | 1 | 0 | 0 | 132 | 145 |  |  | PEAKS DB |
| total 1 peptides |  |  |  |  |  |  |  |  |  |  |  |  |  |  |  |  |  |  |  |  |  |  |  |  |  |  |  |  |  |  |  |
| --- | --- | --- | --- | --- | --- | --- | --- | --- | --- | --- | --- | --- | --- | --- | --- | --- | --- | --- | --- | --- | --- | --- | --- | --- | --- | --- | --- | --- | --- | --- | --- |

P18982|HBB\_AILFU

back to list

  

| Protein Coverage
| Supporting Peptides
|

Protein Coverage:

Supporting Peptides:

| Peptide | Uniq | -10lgP | Mass | Length | ppm | m/z | z | RT | Fraction | Scan | Source File | Area F1 | Area F2 | Area F3 | Area F4 | Area F5 | Area F6 | Area F7 | #Feature | #Feature F1 | #Feature F2 | #Feature F3 | #Feature F4 | #Feature F5 | #Feature F6 | #Feature F7 | Start | End | PTM | AScore | Found By |
| --- | --- | --- | --- | --- | --- | --- | --- | --- | --- | --- | --- | --- | --- | --- | --- | --- | --- | --- | --- | --- | --- | --- | --- | --- | --- | --- | --- | --- | --- | --- | --- |
| K.VVAGVANALAHKYH | Y | 50.77 | 1448.7888 | 14 | -0.9 | 483.9365 | 3 | 34.60 | 5 | F5:5741 | Fraction\_5\_23092022.raw |  |  |  |  | 1.068E5 |  |  | 1 | 0 | 0 | 0 | 0 | 1 | 0 | 0 | 133 | 146 |  |  | PEAKS DB |
| total 1 peptides |  |  |  |  |  |  |  |  |  |  |  |  |  |  |  |  |  |  |  |  |  |  |  |  |  |  |  |  |  |  |  |
| --- | --- | --- | --- | --- | --- | --- | --- | --- | --- | --- | --- | --- | --- | --- | --- | --- | --- | --- | --- | --- | --- | --- | --- | --- | --- | --- | --- | --- | --- | --- | --- |

B3EWD0|HBB\_OTOBE

back to list

  

| Protein Coverage
| Supporting Peptides
|

Protein Coverage:

Supporting Peptides:

| Peptide | Uniq | -10lgP | Mass | Length | ppm | m/z | z | RT | Fraction | Scan | Source File | Area F1 | Area F2 | Area F3 | Area F4 | Area F5 | Area F6 | Area F7 | #Feature | #Feature F1 | #Feature F2 | #Feature F3 | #Feature F4 | #Feature F5 | #Feature F6 | #Feature F7 | Start | End | PTM | AScore | Found By |
| --- | --- | --- | --- | --- | --- | --- | --- | --- | --- | --- | --- | --- | --- | --- | --- | --- | --- | --- | --- | --- | --- | --- | --- | --- | --- | --- | --- | --- | --- | --- | --- |
| K.VVAGVANALAHKYH | Y | 50.77 | 1448.7888 | 14 | -0.9 | 483.9365 | 3 | 34.60 | 5 | F5:5741 | Fraction\_5\_23092022.raw |  |  |  |  | 1.068E5 |  |  | 1 | 0 | 0 | 0 | 0 | 1 | 0 | 0 | 133 | 146 |  |  | PEAKS DB |
| total 1 peptides |  |  |  |  |  |  |  |  |  |  |  |  |  |  |  |  |  |  |  |  |  |  |  |  |  |  |  |  |  |  |  |
| --- | --- | --- | --- | --- | --- | --- | --- | --- | --- | --- | --- | --- | --- | --- | --- | --- | --- | --- | --- | --- | --- | --- | --- | --- | --- | --- | --- | --- | --- | --- | --- |

P68044|HBB\_MUSPF

back to list

  

| Protein Coverage
| Supporting Peptides
|

Protein Coverage:

Supporting Peptides:

| Peptide | Uniq | -10lgP | Mass | Length | ppm | m/z | z | RT | Fraction | Scan | Source File | Area F1 | Area F2 | Area F3 | Area F4 | Area F5 | Area F6 | Area F7 | #Feature | #Feature F1 | #Feature F2 | #Feature F3 | #Feature F4 | #Feature F5 | #Feature F6 | #Feature F7 | Start | End | PTM | AScore | Found By |
| --- | --- | --- | --- | --- | --- | --- | --- | --- | --- | --- | --- | --- | --- | --- | --- | --- | --- | --- | --- | --- | --- | --- | --- | --- | --- | --- | --- | --- | --- | --- | --- |
| K.VVAGVANALAHKYH | Y | 50.77 | 1448.7888 | 14 | -0.9 | 483.9365 | 3 | 34.60 | 5 | F5:5741 | Fraction\_5\_23092022.raw |  |  |  |  | 1.068E5 |  |  | 1 | 0 | 0 | 0 | 0 | 1 | 0 | 0 | 133 | 146 |  |  | PEAKS DB |
| total 1 peptides |  |  |  |  |  |  |  |  |  |  |  |  |  |  |  |  |  |  |  |  |  |  |  |  |  |  |  |  |  |  |  |
| --- | --- | --- | --- | --- | --- | --- | --- | --- | --- | --- | --- | --- | --- | --- | --- | --- | --- | --- | --- | --- | --- | --- | --- | --- | --- | --- | --- | --- | --- | --- | --- |

P20855|HBB\_CTEGU

back to list

  

| Protein Coverage
| Supporting Peptides
|

Protein Coverage:

Supporting Peptides:

| Peptide | Uniq | -10lgP | Mass | Length | ppm | m/z | z | RT | Fraction | Scan | Source File | Area F1 | Area F2 | Area F3 | Area F4 | Area F5 | Area F6 | Area F7 | #Feature | #Feature F1 | #Feature F2 | #Feature F3 | #Feature F4 | #Feature F5 | #Feature F6 | #Feature F7 | Start | End | PTM | AScore | Found By |
| --- | --- | --- | --- | --- | --- | --- | --- | --- | --- | --- | --- | --- | --- | --- | --- | --- | --- | --- | --- | --- | --- | --- | --- | --- | --- | --- | --- | --- | --- | --- | --- |
| K.VVAGVANALAHKYH | Y | 50.77 | 1448.7888 | 14 | -0.9 | 483.9365 | 3 | 34.60 | 5 | F5:5741 | Fraction\_5\_23092022.raw |  |  |  |  | 1.068E5 |  |  | 1 | 0 | 0 | 0 | 0 | 1 | 0 | 0 | 133 | 146 |  |  | PEAKS DB |
| total 1 peptides |  |  |  |  |  |  |  |  |  |  |  |  |  |  |  |  |  |  |  |  |  |  |  |  |  |  |  |  |  |  |  |
| --- | --- | --- | --- | --- | --- | --- | --- | --- | --- | --- | --- | --- | --- | --- | --- | --- | --- | --- | --- | --- | --- | --- | --- | --- | --- | --- | --- | --- | --- | --- | --- |

P02087|HBB\_DASNO

back to list

  

| Protein Coverage
| Supporting Peptides
|

Protein Coverage:

Supporting Peptides:

| Peptide | Uniq | -10lgP | Mass | Length | ppm | m/z | z | RT | Fraction | Scan | Source File | Area F1 | Area F2 | Area F3 | Area F4 | Area F5 | Area F6 | Area F7 | #Feature | #Feature F1 | #Feature F2 | #Feature F3 | #Feature F4 | #Feature F5 | #Feature F6 | #Feature F7 | Start | End | PTM | AScore | Found By |
| --- | --- | --- | --- | --- | --- | --- | --- | --- | --- | --- | --- | --- | --- | --- | --- | --- | --- | --- | --- | --- | --- | --- | --- | --- | --- | --- | --- | --- | --- | --- | --- |
| K.VVAGVANALAHKYH | Y | 50.77 | 1448.7888 | 14 | -0.9 | 483.9365 | 3 | 34.60 | 5 | F5:5741 | Fraction\_5\_23092022.raw |  |  |  |  | 1.068E5 |  |  | 1 | 0 | 0 | 0 | 0 | 1 | 0 | 0 | 134 | 147 |  |  | PEAKS DB |
| total 1 peptides |  |  |  |  |  |  |  |  |  |  |  |  |  |  |  |  |  |  |  |  |  |  |  |  |  |  |  |  |  |  |  |
| --- | --- | --- | --- | --- | --- | --- | --- | --- | --- | --- | --- | --- | --- | --- | --- | --- | --- | --- | --- | --- | --- | --- | --- | --- | --- | --- | --- | --- | --- | --- | --- |

P19886|HBD\_COLPO

back to list

  

| Protein Coverage
| Supporting Peptides
|

Protein Coverage:

Supporting Peptides:

| Peptide | Uniq | -10lgP | Mass | Length | ppm | m/z | z | RT | Fraction | Scan | Source File | Area F1 | Area F2 | Area F3 | Area F4 | Area F5 | Area F6 | Area F7 | #Feature | #Feature F1 | #Feature F2 | #Feature F3 | #Feature F4 | #Feature F5 | #Feature F6 | #Feature F7 | Start | End | PTM | AScore | Found By |
| --- | --- | --- | --- | --- | --- | --- | --- | --- | --- | --- | --- | --- | --- | --- | --- | --- | --- | --- | --- | --- | --- | --- | --- | --- | --- | --- | --- | --- | --- | --- | --- |
| K.VVAGVANALAHKYH | Y | 50.77 | 1448.7888 | 14 | -0.9 | 483.9365 | 3 | 34.60 | 5 | F5:5741 | Fraction\_5\_23092022.raw |  |  |  |  | 1.068E5 |  |  | 1 | 0 | 0 | 0 | 0 | 1 | 0 | 0 | 134 | 147 |  |  | PEAKS DB |
| total 1 peptides |  |  |  |  |  |  |  |  |  |  |  |  |  |  |  |  |  |  |  |  |  |  |  |  |  |  |  |  |  |  |  |
| --- | --- | --- | --- | --- | --- | --- | --- | --- | --- | --- | --- | --- | --- | --- | --- | --- | --- | --- | --- | --- | --- | --- | --- | --- | --- | --- | --- | --- | --- | --- | --- |

P61773|HBD\_GORGO

back to list

  

| Protein Coverage
| Supporting Peptides
|

Protein Coverage:

Supporting Peptides:

| Peptide | Uniq | -10lgP | Mass | Length | ppm | m/z | z | RT | Fraction | Scan | Source File | Area F1 | Area F2 | Area F3 | Area F4 | Area F5 | Area F6 | Area F7 | #Feature | #Feature F1 | #Feature F2 | #Feature F3 | #Feature F4 | #Feature F5 | #Feature F6 | #Feature F7 | Start | End | PTM | AScore | Found By |
| --- | --- | --- | --- | --- | --- | --- | --- | --- | --- | --- | --- | --- | --- | --- | --- | --- | --- | --- | --- | --- | --- | --- | --- | --- | --- | --- | --- | --- | --- | --- | --- |
| K.VVAGVANALAHKYH | Y | 50.77 | 1448.7888 | 14 | -0.9 | 483.9365 | 3 | 34.60 | 5 | F5:5741 | Fraction\_5\_23092022.raw |  |  |  |  | 1.068E5 |  |  | 1 | 0 | 0 | 0 | 0 | 1 | 0 | 0 | 134 | 147 |  |  | PEAKS DB |
| total 1 peptides |  |  |  |  |  |  |  |  |  |  |  |  |  |  |  |  |  |  |  |  |  |  |  |  |  |  |  |  |  |  |  |
| --- | --- | --- | --- | --- | --- | --- | --- | --- | --- | --- | --- | --- | --- | --- | --- | --- | --- | --- | --- | --- | --- | --- | --- | --- | --- | --- | --- | --- | --- | --- | --- |

P68232|HBB\_ATEGE

back to list

  

| Protein Coverage
| Supporting Peptides
|

Protein Coverage:

Supporting Peptides:

| Peptide | Uniq | -10lgP | Mass | Length | ppm | m/z | z | RT | Fraction | Scan | Source File | Area F1 | Area F2 | Area F3 | Area F4 | Area F5 | Area F6 | Area F7 | #Feature | #Feature F1 | #Feature F2 | #Feature F3 | #Feature F4 | #Feature F5 | #Feature F6 | #Feature F7 | Start | End | PTM | AScore | Found By |
| --- | --- | --- | --- | --- | --- | --- | --- | --- | --- | --- | --- | --- | --- | --- | --- | --- | --- | --- | --- | --- | --- | --- | --- | --- | --- | --- | --- | --- | --- | --- | --- |
| K.VVAGVANALAHKYH | Y | 50.77 | 1448.7888 | 14 | -0.9 | 483.9365 | 3 | 34.60 | 5 | F5:5741 | Fraction\_5\_23092022.raw |  |  |  |  | 1.068E5 |  |  | 1 | 0 | 0 | 0 | 0 | 1 | 0 | 0 | 134 | 147 |  |  | PEAKS DB |
| total 1 peptides |  |  |  |  |  |  |  |  |  |  |  |  |  |  |  |  |  |  |  |  |  |  |  |  |  |  |  |  |  |  |  |
| --- | --- | --- | --- | --- | --- | --- | --- | --- | --- | --- | --- | --- | --- | --- | --- | --- | --- | --- | --- | --- | --- | --- | --- | --- | --- | --- | --- | --- | --- | --- | --- |

P60524|HBB\_CANLF

back to list

  

| Protein Coverage
| Supporting Peptides
|

Protein Coverage:

Supporting Peptides:

| Peptide | Uniq | -10lgP | Mass | Length | ppm | m/z | z | RT | Fraction | Scan | Source File | Area F1 | Area F2 | Area F3 | Area F4 | Area F5 | Area F6 | Area F7 | #Feature | #Feature F1 | #Feature F2 | #Feature F3 | #Feature F4 | #Feature F5 | #Feature F6 | #Feature F7 | Start | End | PTM | AScore | Found By |
| --- | --- | --- | --- | --- | --- | --- | --- | --- | --- | --- | --- | --- | --- | --- | --- | --- | --- | --- | --- | --- | --- | --- | --- | --- | --- | --- | --- | --- | --- | --- | --- |
| K.VVAGVANALAHKYH | Y | 50.77 | 1448.7888 | 14 | -0.9 | 483.9365 | 3 | 34.60 | 5 | F5:5741 | Fraction\_5\_23092022.raw |  |  |  |  | 1.068E5 |  |  | 1 | 0 | 0 | 0 | 0 | 1 | 0 | 0 | 133 | 146 |  |  | PEAKS DB |
| total 1 peptides |  |  |  |  |  |  |  |  |  |  |  |  |  |  |  |  |  |  |  |  |  |  |  |  |  |  |  |  |  |  |  |
| --- | --- | --- | --- | --- | --- | --- | --- | --- | --- | --- | --- | --- | --- | --- | --- | --- | --- | --- | --- | --- | --- | --- | --- | --- | --- | --- | --- | --- | --- | --- | --- |

P60525|HBB\_CANLA

back to list

  

| Protein Coverage
| Supporting Peptides
|

Protein Coverage:

Supporting Peptides:

| Peptide | Uniq | -10lgP | Mass | Length | ppm | m/z | z | RT | Fraction | Scan | Source File | Area F1 | Area F2 | Area F3 | Area F4 | Area F5 | Area F6 | Area F7 | #Feature | #Feature F1 | #Feature F2 | #Feature F3 | #Feature F4 | #Feature F5 | #Feature F6 | #Feature F7 | Start | End | PTM | AScore | Found By |
| --- | --- | --- | --- | --- | --- | --- | --- | --- | --- | --- | --- | --- | --- | --- | --- | --- | --- | --- | --- | --- | --- | --- | --- | --- | --- | --- | --- | --- | --- | --- | --- |
| K.VVAGVANALAHKYH | Y | 50.77 | 1448.7888 | 14 | -0.9 | 483.9365 | 3 | 34.60 | 5 | F5:5741 | Fraction\_5\_23092022.raw |  |  |  |  | 1.068E5 |  |  | 1 | 0 | 0 | 0 | 0 | 1 | 0 | 0 | 133 | 146 |  |  | PEAKS DB |
| total 1 peptides |  |  |  |  |  |  |  |  |  |  |  |  |  |  |  |  |  |  |  |  |  |  |  |  |  |  |  |  |  |  |  |
| --- | --- | --- | --- | --- | --- | --- | --- | --- | --- | --- | --- | --- | --- | --- | --- | --- | --- | --- | --- | --- | --- | --- | --- | --- | --- | --- | --- | --- | --- | --- | --- |

P02052|HBB\_TUPGL

back to list

  

| Protein Coverage
| Supporting Peptides
|

Protein Coverage:

Supporting Peptides:

| Peptide | Uniq | -10lgP | Mass | Length | ppm | m/z | z | RT | Fraction | Scan | Source File | Area F1 | Area F2 | Area F3 | Area F4 | Area F5 | Area F6 | Area F7 | #Feature | #Feature F1 | #Feature F2 | #Feature F3 | #Feature F4 | #Feature F5 | #Feature F6 | #Feature F7 | Start | End | PTM | AScore | Found By |
| --- | --- | --- | --- | --- | --- | --- | --- | --- | --- | --- | --- | --- | --- | --- | --- | --- | --- | --- | --- | --- | --- | --- | --- | --- | --- | --- | --- | --- | --- | --- | --- |
| K.VVAGVANALAHKYH | Y | 50.77 | 1448.7888 | 14 | -0.9 | 483.9365 | 3 | 34.60 | 5 | F5:5741 | Fraction\_5\_23092022.raw |  |  |  |  | 1.068E5 |  |  | 1 | 0 | 0 | 0 | 0 | 1 | 0 | 0 | 133 | 146 |  |  | PEAKS DB |
| total 1 peptides |  |  |  |  |  |  |  |  |  |  |  |  |  |  |  |  |  |  |  |  |  |  |  |  |  |  |  |  |  |  |  |
| --- | --- | --- | --- | --- | --- | --- | --- | --- | --- | --- | --- | --- | --- | --- | --- | --- | --- | --- | --- | --- | --- | --- | --- | --- | --- | --- | --- | --- | --- | --- | --- |

Q25C93|FEZF1\_DANRE

back to list

  

| Protein Coverage
| Supporting Peptides
|

Protein Coverage:

Supporting Peptides:

| Peptide | Uniq | -10lgP | Mass | Length | ppm | m/z | z | RT | Fraction | Scan | Source File | Area F1 | Area F2 | Area F3 | Area F4 | Area F5 | Area F6 | Area F7 | #Feature | #Feature F1 | #Feature F2 | #Feature F3 | #Feature F4 | #Feature F5 | #Feature F6 | #Feature F7 | Start | End | PTM | AScore | Found By |
| --- | --- | --- | --- | --- | --- | --- | --- | --- | --- | --- | --- | --- | --- | --- | --- | --- | --- | --- | --- | --- | --- | --- | --- | --- | --- | --- | --- | --- | --- | --- | --- |
| R.C(+57.02)PPGVSFK.E | Y | 48.20 | 890.4320 | 8 | -1.6 | 891.4379 | 1 | 66.11 | 7 | F7:25071 | Fraction\_7\_23092022.raw |  |  |  |  |  |  | 5.436E5 | 1 | 0 | 0 | 0 | 0 | 0 | 0 | 1 | 198 | 205 | Carbamidomethylation | C1:Carbamidomethylation:1000.00 | PEAKS DB |
| total 1 peptides |  |  |  |  |  |  |  |  |  |  |  |  |  |  |  |  |  |  |  |  |  |  |  |  |  |  |  |  |  |  |  |
| --- | --- | --- | --- | --- | --- | --- | --- | --- | --- | --- | --- | --- | --- | --- | --- | --- | --- | --- | --- | --- | --- | --- | --- | --- | --- | --- | --- | --- | --- | --- | --- |

P29508|SPB3\_HUMAN

back to list

  

| Protein Coverage
| Supporting Peptides
|

Protein Coverage:

Supporting Peptides:

| Peptide | Uniq | -10lgP | Mass | Length | ppm | m/z | z | RT | Fraction | Scan | Source File | Area F1 | Area F2 | Area F3 | Area F4 | Area F5 | Area F6 | Area F7 | #Feature | #Feature F1 | #Feature F2 | #Feature F3 | #Feature F4 | #Feature F5 | #Feature F6 | #Feature F7 | Start | End | PTM | AScore | Found By |
| --- | --- | --- | --- | --- | --- | --- | --- | --- | --- | --- | --- | --- | --- | --- | --- | --- | --- | --- | --- | --- | --- | --- | --- | --- | --- | --- | --- | --- | --- | --- | --- |
| R.FKVEESYDLKDTLR.T | Y | 48.14 | 1741.8885 | 14 | 0.1 | 581.6368 | 3 | 46.83 | 1 | F1:10159 | Fraction\_1\_23092022r.raw | 2.3427E5 |  |  |  |  |  |  | 1 | 1 | 0 | 0 | 0 | 0 | 0 | 0 | 287 | 300 |  |  | PEAKS DB |
| total 1 peptides |  |  |  |  |  |  |  |  |  |  |  |  |  |  |  |  |  |  |  |  |  |  |  |  |  |  |  |  |  |  |  |
| --- | --- | --- | --- | --- | --- | --- | --- | --- | --- | --- | --- | --- | --- | --- | --- | --- | --- | --- | --- | --- | --- | --- | --- | --- | --- | --- | --- | --- | --- | --- | --- |

TRINITY\_DN4582\_c0\_g1\_i1.p1

back to list

  

| Protein Coverage
| Supporting Peptides
|

Protein Coverage:

Supporting Peptides:

| Peptide | Uniq | -10lgP | Mass | Length | ppm | m/z | z | RT | Fraction | Scan | Source File | Area F1 | Area F2 | Area F3 | Area F4 | Area F5 | Area F6 | Area F7 | #Feature | #Feature F1 | #Feature F2 | #Feature F3 | #Feature F4 | #Feature F5 | #Feature F6 | #Feature F7 | Start | End | PTM | AScore | Found By |
| --- | --- | --- | --- | --- | --- | --- | --- | --- | --- | --- | --- | --- | --- | --- | --- | --- | --- | --- | --- | --- | --- | --- | --- | --- | --- | --- | --- | --- | --- | --- | --- |
| R.SISISPTNVQPR.S | Y | 47.43 | 1297.6990 | 12 | 0.5 | 649.8571 | 2 | 42.23 | 2 | F2:8246 | Fraction\_2\_23092022.raw |  | 4.7244E6 |  |  |  |  |  | 1 | 0 | 1 | 0 | 0 | 0 | 0 | 0 | 440 | 451 |  |  | PEAKS DB |
| total 1 peptides |  |  |  |  |  |  |  |  |  |  |  |  |  |  |  |  |  |  |  |  |  |  |  |  |  |  |  |  |  |  |  |
| --- | --- | --- | --- | --- | --- | --- | --- | --- | --- | --- | --- | --- | --- | --- | --- | --- | --- | --- | --- | --- | --- | --- | --- | --- | --- | --- | --- | --- | --- | --- | --- |

TRINITY\_DN4582\_c0\_g1\_i2.p1

back to list

  

| Protein Coverage
| Supporting Peptides
|

Protein Coverage:

Supporting Peptides:

| Peptide | Uniq | -10lgP | Mass | Length | ppm | m/z | z | RT | Fraction | Scan | Source File | Area F1 | Area F2 | Area F3 | Area F4 | Area F5 | Area F6 | Area F7 | #Feature | #Feature F1 | #Feature F2 | #Feature F3 | #Feature F4 | #Feature F5 | #Feature F6 | #Feature F7 | Start | End | PTM | AScore | Found By |
| --- | --- | --- | --- | --- | --- | --- | --- | --- | --- | --- | --- | --- | --- | --- | --- | --- | --- | --- | --- | --- | --- | --- | --- | --- | --- | --- | --- | --- | --- | --- | --- |
| R.SISISPTNVQPR.S | Y | 47.43 | 1297.6990 | 12 | 0.5 | 649.8571 | 2 | 42.23 | 2 | F2:8246 | Fraction\_2\_23092022.raw |  | 4.7244E6 |  |  |  |  |  | 1 | 0 | 1 | 0 | 0 | 0 | 0 | 0 | 440 | 451 |  |  | PEAKS DB |
| total 1 peptides |  |  |  |  |  |  |  |  |  |  |  |  |  |  |  |  |  |  |  |  |  |  |  |  |  |  |  |  |  |  |  |
| --- | --- | --- | --- | --- | --- | --- | --- | --- | --- | --- | --- | --- | --- | --- | --- | --- | --- | --- | --- | --- | --- | --- | --- | --- | --- | --- | --- | --- | --- | --- | --- |

Peptide List

  
  

---

Prepared with PEAKS ™ (bioinfor.com)
